# Supplementary material for: Carbapenem-resistant Enterobacterales among patients with bloodstream infections in South Africa: Consolidated surveillance data, 2015–2021
Source: PLoS One. 2025 Jul 2;20(7):e0324262. doi: 10.1371/journal.pone.0324262 (PMC12221022; doi:10.1371/journal.pone.0324262)
Supplement: S1 Table — (PDF) [file pone.0324262.s001.pdf]

Building of statistical model and adjusting for other covariates

We tested for multicollinearity among the exposure variables before adding them on the final model. We used the correlation coefficients and variance inflation factor (VIF) to report on variables that were highly correlated. Variables with coefficients above 0.5 in the correlation matrix were further explored with VIF. Variables that had VIF values above 10 were not added in the final model.

Model building was done using purposive selection of variables with p-values <0.25 in univariate analysis apart from age and sex which were considered important therefore added irrespective of their p-values. The final model was also checked for the goodness of fit (GOF) using Chi-Square GOF test. Due to differences in the pre-existing conditions in children and adults, we performed separate subgroup analysis to check possible effect modification or interaction. We used bivariable logistic regression and variables used in this subgroup analysis were ward type categorised as paediatric and adult wards and age with nine categories. We then assessed the effect of pre-existing conditions on mortality based on age or ward type.

Description of fitted models

The Chi-Square GOF test assessed the null hypothesis that the observed values are the same as the model-predicted values with a p-value of <0.05 indicating a model is not a good fit for the data. Medical device inserted had a high variance inflation factor (VIF=16), additionally p-value on univariate analysis was 0.442 which is above the cut off for inclusion in the final model (0.25). Mechanical ventilation was highly correlated with mental status and therefore we only included mechanical ventilation in the final model.

The model with all the variables included showed a somewhat acceptable fit (p=0.077) (**S1 Table**). After

removing medical device and mental status the model GOF improved a little (p=0.063). Additionally, removing pre-existing conditions made the model a best fit (p=0.220) (Table 3). Lastly, we added an interaction term for age and pre-existing conditions in the final model, however it did not improve the model (p=0.057) therefore it was removed.

Final model (description)

We assessed and adjusted for the effect of the following exposures simultaneously on in hospital mortality among CRE BSI cases; sex, age, current and previous exposure to antibiotics, past hospitalisation, being oxygenated and mechanically ventilated on current admission. We did this after checking if all the exposure variables are not correlated. Simultaneous adjusting of the above factors showed that being male (aOR=0.8; 95%CI: 0.65-0.99; p=0.030) and being on antibiotics on current admission (aOR=0.59; 95%CI: 0.42-0.82; p=0.002) was associated with lower odds of death compared to being female and not receiving antibiotics respectively. Children 1-11 months (aOR=0.54; 95%CI: 0.36-0.82; p=0.004) and 1-9 years (aOR=0.38; 95%CI: 0.22-0.66; p=0.001) had lower odds of death compared to 30-39 years age group.

The other factors that were associated with high odds of death were age >60 years (aOR=2.21; 95%CI: 1.54-3.16; p<001), past hospitalisation (aOR=1.54; 95%CI: 1.18-2.03; p=0.002), past exposure to antibiotics (aOR=1.45; 95%CI: 1.18-1.79; p<0.001), being oxygenated (aOR=1.68; 95%CI: 1.35-2.07; p<0.001) and being mechanically ventilated (aOR=2.76; 95%CI:2.23-3.42; p<0.001) Pre-existing condition was not adjusted in the final model, it was presented separately for ward type and age below.

S1 Table: Initial model with all variables.

Characteristics	Alive<br>n (row %)	Dead<br>n (row %)	OR (95% CI)	p-value	aOR (95% CI)	p-value
**Sex**						
Female	739 (**58.4**)	526 (**41.6**)	Ref		Ref	
Male	965 (**64.7**)	525 (**35.2**)	0.76 (0.65-0.89)	0.001	0.82 (0.66-1.03)	0.08
**Age categories**						
<30 days	281 (**69.9**)	121 (**30.1**)	0.69 (0.52-0.93)	0.015	0.90 (0.60-1.37)	0.647
1-11 months	201 (**72.8**)	75 (**27.2**)	0.60 (0.43-0.83)	0.003	0.78 (0.49-1.24)	0.298
1-9 years	163 (**78.7**)	44 (**21.3**)	0.43 (0.30-0.64)	0.000	0.50 (0.28-0.91)	0.024
10-19 years	94 (**70.1**)	40 (**29.8**)	0.68 (0.44-1.04)	0.079	1.04 (0.59-1.84)	0.871
20-29	166 (**66.4**)	84 (**33.6**)	0.81 (0.58-1.13)	0.225	0.88 (0.55-1.41)	0.601
30-39	243 (**61.7**)	151 (**38.3**)	Ref		Ref	
40-49	182 (**56.0**)	143 (**44.0**)	1.26 (0.94-1.70)	0.124	1.39 (0.91-2.10)	0.123
50-59	189 (**54.8**)	156 (**45.2**)	1.32 (0.99-1.78)	0.058	1.62 (1.07-2.44)	0.021
>=60 years	196 (**45.2**)	238 (**54.8**)	1.95 (1.48-2.57)	<0.001	2.02 (1.36-2.99)	<0.001
**Pre-existing conditions**						
No	807 (**71.0**)	329 (**28.9**)	Ref		Ref	
Yes	759 (**58.6**)	536 (**41.4**)	1.73 (1.46-2.05)	<0.001	1.42 (1.13-1.79)	0.003
**Medical device inserted**						
No	48 (**68.6**)	22 (**31.4**)	Ref		Ref	
Yes	1,587 (**64.1**)	889 (**35.9**)	1.22 (0.73-2.03)	0.442	0.84 (0.43-1.64)	0.608
**Antibiotics on current admission**						
No	146 (**53.5**)	127 (**46.5**)	Ref		Ref	

Yes	1,500 (**65.7**)	783 (**34.3**)	0.60 (0.46-0.77)	<0.001	0.57 (0.40-0.82)	0.002
**Previous exposure to antibiotics**						
No	854 (**71.9**)	333 (**28.0**)	Ref		Ref	
Yes	655 (**59.8**)	440 (**40.2**)	1.72 (1.44-2.05)	<0.001	1.42 (1.13-1.79)	0.002
**Oxygenated**						
No	839 (**69.3**)	372 (**30.7**)	Ref		Ref	
Yes	835 (**57.8**)	609 (**42.2**)	1.28 (1.18-1.39)	<0.001	1.47 (1.15-1.87)	0.002
**Mechanical ventilation**						
No	1,216 (**71.3**)	489 (**28.7**)	Ref		Ref	
Yes	428 (**50.4**)	421 (**49.6**)	1.56 (1.43-1.70)	<0.001	1.69 (1.12-2.53)	0.012
**Previous hospital admission**						
No	1,295 (**67.8**)	614 (**32.2**)	Ref		Ref	
Yes	266 (**59.8**)	179 (**40.2**)	1.19 (1.07-1.32)	0.001	1.28 (0.94-1.73)	0.104
**Mental status**						
Alert	958 (**80.0**)	239 (**20.0**)	Ref		Ref	
Disorientated	112 (**47.5**)	124 (**52.5**)	4.43 (3.31-5.94)	<0.001	2.86 (2.00-4.10)	<0.001
Sedated	390 (**51.4**)	369 (**48.6**)	3.79 (3.10-4.63)	<0.001	2.13 (1.39-3.28)	0.001
Unconscious	26 (**65.1**)	66 (**34.9**)	10.17 (6.32-16.36)	<0.001	7.32 (3.94-13.60)	<0.001
