# Supplementary material for: Carbapenem-resistant Enterobacterales among patients with bloodstream infections in South Africa: Consolidated surveillance data, 2015–2021
Source: PLoS One. 2025 Jul 2;20(7):e0324262. doi: 10.1371/journal.pone.0324262 (PMC12221022; doi:10.1371/journal.pone.0324262)
Supplement: S2 Table — (PDF) [file pone.0324262.s002.pdf]

Subgroup analysis for pre-existing conditions by ward type and age category

We performed bivariate analysis using ward type and pre-existing conditions, we noted that those in adult wards had higher odds of death () in the presence of pre-existing conditions compared to those in paediatric wards (**S2 Table**). When age categories were used instead of ward type, we noted that the elderly (>60 years) with pre-existing conditions had odds of death compared to the 30-39 age group.

S2 Table: Bivariate analysis of ward type and pre-existing conditions.

Characteristics	Alive<br>n (row %)	Dead<br>n (row %)	OR (95% CI)	p-value	aOR (95% CI)	p-value
**Ward type**						
Paediatric	677 (**72.6**)	256 (**27.4**)	Ref		Ref	
Adult	967 (**56.3**)	751 (**43.7**)	2.05 (1.73-2.44)	<0.001	1.96 (1.62-2.37)	<0.001
**Pre-existing conditions**						
No	807 (**71.0**)	329 (**28.9**)	Ref		Ref	
Yes	759 (**58.6**)	536 (**41.4**)	1.73 (1.46-2.05)	<0.001	1.53 (1.29-1.82)	<0.001
