# Supplementary material for: Carbapenem-resistant Enterobacterales among patients with bloodstream infections in South Africa: Consolidated surveillance data, 2015–2021
Source: PLoS One. 2025 Jul 2;20(7):e0324262. doi: 10.1371/journal.pone.0324262 (PMC12221022; doi:10.1371/journal.pone.0324262)
Supplement: S3 Table — (PDF) [file pone.0324262.s003.pdf]

Children below the age of 20 years with pre-existing conditions had lower odds of death overall compared to the 30-39 age group (**S3 Table**). While these results are the same, using the age categories assists to identify the group at higher risk in the adult wards. Overall, we see that the odds of death in the presence of pre-existing conditions differs in children compared to adults. Therefore, since confounding could not be demonstrated we did not adjust for pre-existing conditions in the final model.

S3 Table: Bivariate analysis of age categories with pre-existing conditions.

**Characteristics**	**Alive**<br>**n (row %)**	**Dead**<br>**n (row %)**	**OR (95% CI)**	**p-value**	**aOR (95% CI)**	**p-value**
**Age categories**						
<30 days	281 (**69.9**)	121 (**30.1**)	0.69 (0.52-0.93)	0.015	0.64 (0.47-0.90)	0.008
1-11 months	201 (**72.8**)	75 (**27.2**)	0.60 (0.43-0.83)	0.003	0.62 (0.43-0.89)	0.010
1-9 years	163 (**78.7**)	44 (**21.3**)	0.43 (0.30-0.64)	0.000	0.38 (0.24-0.58)	<0.001
10-19 years	94 (**70.1**)	40 (**29.8**)	0.68 (0.44-1.04)	0.079	0.57 (0.36-0.91)	0.018
20-29	166 (**66.4**)	84 (**33.6**)	0.81 (0.58-1.13)	0.225	0.77 (0.54-1.10)	0.156
30-39	243 (**61.7**)	151 (**38.3**)	Ref		Ref	
40-49	182 (**56.0**)	143 (**44.0**)	1.26 (0.94-1.70)	0.124	1.09 (0.79-1.51)	0.596
50-59	189 (**54.8**)	156 (**45.2**)	1.32 (0.99-1.78)	0.058	1.07 (0.78-1.48)	0.656
>=60 years	196 (**45.2**)	238 (**54.8**)	1.95 (1.48-2.57)	<0.001	1.74 (1.29-2.35)	<0.001
**Pre-existing conditions**						
No	807 (**71.0**)	329 (**28.9**)	Ref		Ref	
Yes	759 (**58.6**)	536 (**41.4**)	1.73 (1.46-2.05)	<0.001	1.50 (1.26-1.80)	<0.001
