# Supplementary material for: Carbapenem-resistant Enterobacterales among patients with bloodstream infections in South Africa: Consolidated surveillance data, 2015–2021
Source: PLoS One. 2025 Jul 2;20(7):e0324262. doi: 10.1371/journal.pone.0324262 (PMC12221022; doi:10.1371/journal.pone.0324262)
Supplement: S4 Data — (PDF) [file pone.0324262.s004.pdf]

unique-id	year	month	province	ward_type	sex	age	ageunits
1	2015	Jul	GA	Adults	Male	39	Y
2	2015	Jul	GA	Adults	Female	63	Y
3	2015	Jul	KZ	Adults	Male	28	Y
4	2015	Jul	GA	Paeds	Female	2	M
5	2015	Jul	GA	Adults	Male	60	Y
6	2015	Aug	GA	Paeds	Male	26	D
7	2015	Aug	GA	Adults	Female	83	Y
8	2015	Jul	GA	Paeds	Male	6	Y
9	2015	Aug	GA	Adults	Female	38	Y
10	2015	Aug	GA	Adults	Female	24	Y
12	2015	Aug	GA	Paeds	Male	10	D
13	2015	Aug	GA	Paeds	Unknown		
15	2015	Aug	KZ	Adults	Male	28	Y
16	2015	Aug	KZ	Adults	Male	29	Y
18	2015	Aug	GA	Adults	Female	28	Y
19	2015	Aug	GA	Paeds	Female	7	M
22	2015	Sep	WC	Adults	Female	33	Y
23	2015	Sep	KZ	Paeds	Male	4	M
24	2015	Sep	KZ	Paeds	Male	16	D
25	2015	Sep	KZ	Adults	Male	46	Y
26	2015	Sep	KZ	Paeds	Female	3	D
27	2015	Sep	KZ	Paeds	Female	26	D
28	2015	Sep	KZ	Paeds	Male	1	M
29	2015	Sep	KZ	Paeds	Female	9	M
30	2015	Sep	KZ	Adults	Male	36	Y
31	2015	Sep	GA	Adults	Male	37	Y
32	2015	Sep	GA	Paeds	Female	6	M
33	2015	Oct	KZ	Paeds	Female	27	D
34	2015	Oct	KZ	Paeds	Male	22	D
35	2015	Sep	GA	Paeds	Male	44	M
36	2015	Oct	KZ	Paeds	Female	2	M
37	2015	Aug	GA	Adults	Male	55	Y
38	2015	Sep	GA	Adults	Female	25	Y
39	2015	Oct	GA	Adults	Female	43	Y
40	2015	Sep	GA	Adults	Female	53	Y
41	2015	Jul	GA	Paeds	Male	23	M
42	2015	Jul	GA	Paeds	Male	9	D
43	2015	Aug	GA	Paeds	Female	6	Y
44	2015	Sep	WC	Adults	Male	16	Y
46	2015	Oct	KZ	Paeds	Male	2	M
48	2015	Sep	KZ	Adults	Female	80	Y
50	2015	Oct	KZ	Adults	Male	42	Y
51	2015	Oct	KZ	Adults	Male	35	Y
52	2015	Oct	KZ	Paeds	Male	6	M
53	2015	Oct	GA	Paeds	Female	28	D
54	2015	Oct	GA	Paeds	Male	11	Y
55	2015	Oct	GA	Paeds	Male	50	M
56	2015	Oct	GA	Paeds	Female	12	Y
58	2015	Oct	GA	Paeds	Male	23	M
59	2015	Oct	GA	Paeds	Female	8	Y
64	2015	Nov	GA	Paeds	Female	12	Y
65	2015	Nov	KZ	Paeds	Female	3	M
67	2015	Nov	WC	Adults	Female	53	Y
68	2015	Nov	GA	Paeds	Female	5	Y
69	2015	Nov	GA	Adults	Female	49	Y

70	2015	Nov	GA	Adults	Male	36	Y
72	2015	Nov	GA	Adults	Female	51	Y
73	2015	Nov	GA	Adults	Female	63	Y
74	2015	Nov	GA	Paeds	Male	13	Y
75	2015	Nov	GA	Adults	Male	61	Y
76	2015	Nov	GA	Adults	Male	52	Y
77	2015	Nov	GA	Adults	Female	60	Y
78	2015	Nov	KZ	Paeds	Male	2	M
79	2015	Nov	KZ	Adults	Male	32	Y
80	2015	Nov	KZ	Paeds	Male	9	D
81	2015	Nov	GA	Paeds	Female	32	M
82	2015	Nov	GA	Adults	Female	52	Y
83	2015	Nov	GA	Paeds	Female	12	Y
85	2015	Nov	GA	Adults	Male	69	Y
86	2015	Nov	KZ	Paeds	Male	1	M
87	2015	Nov	GA	Adults	Female	79	Y
89	2015	Nov	GA	Adults	Male	70	Y
90	2015	Dec	GA	Adults	Female	77	Y
93	2015	Dec	GA	Adults	Female	54	Y
94	2015	Dec	GA	Adults	Female	32	Y
95	2015	Dec	GA	Adults	Female	66	Y
96	2015	Dec	GA	Adults	Male	58	Y
99	2015	Dec	KZ	Adults	Male	32	Y
100	2015	Dec	GA	Adults	Male	31	Y
101	2015	Dec	GA	Paeds	Female	7	Y
102	2015	Dec	GA	Adults	Female	19	Y
103	2015	Dec	KZ	Adults	Female	69	Y
106	2015	Dec	KZ	Adults	Male	47	Y
107	2015	Dec	GA	Paeds	Male	22	M
108	2015	Dec	FS	Adults	Female	35	Y
116	2015	Nov	WC	Paeds	Male	55	M
131	2015	Dec	GA	Adults	Female	44	Y
132	2015	Dec	GA	Adults	Female	44	Y
135	2015	Sep	GA	Paeds	Male	6	M
136	2015	Sep	GA	Adults	Male	41	Y
137	2015	Sep	GA	Adults	Male	16	Y
138	2015	Nov	GA	Adults	Male	20	Y
139	2015	Oct	GA	Adults	Female	37	Y
140	2015	Dec	GA	Paeds	Female	10	Y
141	2015	Nov	GA	Adults	Male	58	Y
142	2015	Dec	GA	Adults	Male	40	Y
143	2015	Dec	GA	Paeds	Female	6	Y
144	2015	Dec	GA	Adults	Female	34	Y
145	2015	Dec	GA	Adults	Male	23	Y
146	2015	Dec	KZ	Adults	Female	28	Y
147	2015	Dec	GA	Paeds	Male	6	D
148	2015	Oct	GA	Paeds	Male	6	M
149	2015	Oct	GA	Adults	Male	51	Y
155	2015	Sep	GA	Paeds	Male	15	D
156	2015	Nov	GA	Paeds	Female	8	Y
159	2015	Dec	GA	Adults	Female	54	Y
162	2015	Dec	GA	Adults	Female	57	Y
174	2015	Jul	KZ	Adults	Female	73	Y
202	2015	Aug	KZ	Adults	Female	23	Y
203	2015	Aug	KZ	Paeds	Male	14	D
230	2015	Nov	KZ	Paeds	Female	7	M

236	2015	Nov	GA	Adults	Female	30	Y
238	2015	Dec	GA	Adults	Male	43	Y
239	2015	Dec	GA	Adults	Female	31	Y
240	2015	Dec	GA	Adults	Female	38	Y
308	2015	Dec	GA	Adults	Female	75	Y
383	2015	Oct	GA	Paeds	Female	15	Y
104	2016	Jan	GA	Adults	Male	47	Y
105	2016	Jan	GA	Adults	Male	30	Y
109	2016	Jan	KZ	Adults	Female	35	Y
112	2016	Jan	KZ	Adults	Male	25	Y
113	2016	Jan	GA	Adults	Female	58	Y
114	2016	Jan	KZ	Paeds	Male	7	Y
115	2016	Jan	GA	Paeds	Female	25	D
117	2016	Jan	GA	Adults	Male	16	Y
118	2016	Jan	GA	Adults	Male	47	Y
119	2016	Jan	GA	Adults	Male	47	Y
120	2016	Jan	GA	Unkown	Female		
121	2016	Jan	GA	Adults	Male	75	Y
122	2016	Jan	GA	Paeds	Female	15	Y
123	2016	Jan	GA	Adults	Female	33	Y
124	2016	Jan	GA	Paeds	Male	13	Y
125	2016	Jan	GA	Adults	Female	49	Y
126	2016	Feb	GA	Adults	Female	41	Y
127	2016	Feb	GA	Adults	Male	31	Y
128	2016	Feb	GA	Paeds	Male	10	D
129	2016	Feb	GA	Adults	Male	36	Y
130	2016	Jan	GA	Adults	Female	37	Y
133	2016	Jan	KZ	Adults	Male	33	Y
134	2016	Feb	GA	Adults	Male	60	Y
150	2016	Feb	KZ	Paeds	Male	6	D
151	2016	Feb	GA	Paeds	Male	53	M
152	2016	Jan	WC	Adults	Male	46	Y
153	2016	Jan	WC	Paeds	Female	1	M
154	2016	Jan	WC	Paeds	Female	8	Y
157	2016	Feb	GA	Adults	Male	51	Y
158	2016	Feb	GA	Adults	Female	38	Y
166	2016	Feb	GA	Paeds	Male	6	Y
167	2016	Feb	GA	Paeds	Female	13	Y
168	2016	Feb	GA	Adults	Male	53	Y
169	2016	Feb	GA	Adults	Male	43	Y
170	2016	Mar	GA	Adults	Female	21	Y
171	2016	Mar	GA	Paeds	Female	14	Y
172	2016	Mar	GA	Adults	Female	22	Y
173	2016	Mar	GA	Paeds	Male	15	Y
175	2016	Mar	KZ	Paeds	Male	1	M
176	2016	Mar	KZ	Paeds	Male	28	D
177	2016	Mar	KZ	Paeds	Female	10	D
178	2016	Mar	KZ	Paeds	Male	23	D
179	2016	Mar	GA	Adults	Female	36	Y
180	2016	Mar	GA	Adults	Female	50	Y
181	2016	Mar	GA	Paeds	Male	5	M
182	2016	Mar	GA	Adults	Male	50	Y
188	2016	Mar	GA	Adults	Male	24	Y
189	2016	Mar	GA	Adults	Male	46	Y
190	2016	Mar	GA	Adults	Male	29	Y
191	2016	Mar	GA	Paeds	Male	39	M

192	2016	Mar	GA	Adults	Male	23	Y
193	2016	Mar	GA	Paeds	Male	8	M
194	2016	Mar	KZ	Paeds	Female	8	Y
195	2016	Mar	KZ	Paeds	Male	1	M
196	2016	Apr	KZ	Adults	Female	32	Y
197	2016	Feb	GA	Paeds	Female	36	M
198	2016	Mar	GA	Adults	Male	55	Y
199	2016	Mar	KZ	Paeds	Female	1	D
200	2016	Apr	GA	Adults	Female	65	Y
201	2016	Apr	KZ	Adults	Female	30	Y
204	2016	Apr	WC	Adults	Female	36	Y
205	2016	Apr	KZ	Adults	Male	24	Y
206	2016	Mar	KZ	Paeds	Male	14	D
207	2016	Apr	GA	Paeds	Female	24	D
208	2016	Mar	GA	Adults	Male	45	Y
209	2016	Apr	GA	Adults	Female	59	Y
210	2016	Apr	GA	Paeds	Male	46	M
211	2016	Apr	KZ	Adults	Male	59	Y
212	2016	Apr	WC	Adults	Female	53	Y
213	2016	Apr	KZ	Adults	Female	25	Y
214	2016	Apr	KZ	Adults	Female	35	Y
215	2016	Apr	GA	Adults	Female	50	Y
216	2016	Apr	KZ	Paeds	Female	13	Y
217	2016	Apr	GA	Adults	Male	57	Y
218	2016	Apr	GA	Adults	Female	24	Y
219	2016	Apr	GA	Adults	Male	64	Y
221	2016	Apr	KZ	Paeds	Female	4	D
222	2016	Apr	KZ	Adults	Female	23	Y
223	2016	Apr	KZ	Paeds	Male	9	M
224	2016	Jan	KZ	Adults	Male	25	Y
225	2016	Apr	KZ	Adults	Male	41	Y
226	2016	May	WC	Paeds	Male	15	Y
227	2016	Mar	FS	Paeds	Male	5	M
228	2016	Apr	GA	Paeds	Female	2	D
229	2016	May	GA	Adults	Male	79	Y
231	2016	Mar	KZ	Adults	Female	63	Y
232	2016	Apr	KZ	Paeds	Male	4	D
233	2016	Jan	GA	Adults	Male	52	Y
234	2016	Mar	GA	Adults	Female	76	Y
235	2016	Mar	GA	Adults	Female	38	Y
237	2016	Feb	GA	Adults	Female	69	Y
241	2016	Mar	GA	Adults	Male	59	Y
242	2016	May	KZ	Paeds	Female	10	D
245	2016	May	GA	Paeds	Male	1	M
246	2016	Apr	GA	Adults	Female	45	Y
247	2016	Apr	GA	Adults	Female	59	Y
249	2016	May	GA	Adults	Male	37	Y
250	2016	May	GA	Adults	Female	53	Y
251	2016	May	GA	Adults	Female	56	Y
252	2016	May	GA	Paeds	Male	12	Y
253	2016	Apr	GA	Adults	Female	50	Y
256	2016	Sep	GA	Adults	Female	26	Y
257	2016	May	GA	Adults	Female	42	Y
258	2016	May	GA	Adults	Female	47	Y
259	2016	May	GA	Adults	Female	44	Y
260	2016	May	KZ	Paeds	Male	25	D

261	2016	Apr	KZ	Paeds	Male	9	D
262	2016	May	KZ	Paeds	Male	3	M
264	2016	May	KZ	Paeds	Female	5	D
265	2016	May	KZ	Paeds	Female	11	D
267	2016	May	GA	Adults	Male	42	Y
268	2016	Jun	GA	Adults	Male	52	Y
269	2016	Apr	KZ	Paeds	Female	13	D
271	2016	May	GA	Adults	Female	39	Y
272	2016	Jun	GA	Paeds	Male	24	D
273	2016	Jun	GA	Adults	Male	59	Y
275	2016	Jun	KZ	Paeds	Male	1	M
276	2016	Jun	KZ	Adults	Male	24	Y
277	2016	Jun	KZ	Paeds	Female	2	M
278	2016	Jun	KZ	Paeds	Male	46	M
279	2016	Jun	GA	Adults	Male	50	Y
280	2016	Jun	GA	Adults	Female	58	Y
281	2016	Jun	GA	Adults	Male	50	Y
282	2016	Jun	GA	Adults	Male	34	Y
283	2016	Jun	GA	Adults	Female	70	Y
284	2016	Jun	WC	Paeds	Female	11	Y
285	2016	Jun	GA	Paeds	Male	14	Y
287	2016	Jun	WC	Adults	Male	64	Y
288	2016	Jun	GA	Adults	Female	39	Y
289	2016	Jun	GA	Adults	Female	53	Y
291	2016	Jun	KZ	Paeds	Female	18	D
292	2016	Jun	GA	Adults	Female	37	Y
293	2016	Jul	GA	Adults	Male	60	Y
294	2016	Jul	GA	Adults	Female	17	Y
295	2016	Jul	GA	Adults	Female	76	Y
296	2016	Jul	KZ	Paeds	Female	2	M
297	2016	Jul	KZ	Adults	Female	23	Y
298	2016	Jul	GA	Adults	Female	27	Y
299	2016	Jul	GA	Adults	Female	39	Y
300	2016	Aug	KZ	Paeds	Female	7	D
301	2016	Aug	GA	Paeds	Female	35	M
302	2016	Aug	KZ	Adults	Male	42	Y
303	2016	Aug	GA	Paeds	Female	27	M
304	2016	Jul	GA	Adults	Female	65	Y
305	2016	Aug	KZ	Adults	Female	27	Y
306	2016	Aug	KZ	Paeds	Male	5	D
309	2016	May	GA	Adults	Female	37	Y
310	2016	Aug	GA	Paeds	Male	3	M
311	2016	Jul	GA	Adults	Male	43	Y
313	2016	Aug	GA	Adults	Male	31	Y
314	2016	Aug	GA	Adults	Male	80	Y
315	2016	Aug	KZ	Paeds	Male	2	D
316	2016	Sep	GA	Adults	Male	31	Y
317	2016	Mar	GA	Adults	Female	34	Y
318	2016	Apr	GA	Paeds	Male	46	M
319	2016	May	GA	Adults	Male	37	Y
320	2016	Jun	GA	Adults	Male	54	Y
321	2016	Aug	WC	Adults	Male	38	Y
322	2016	Aug	GA	Adults	Female	61	Y
323	2016	Sep	GA	Adults	Female	58	Y
324	2016	Aug	GA	Adults	Female	59	Y
326	2016	Aug	GA	Adults	Male	30	Y

327	2016	Aug	WC	Adults	Female	35	Y
328	2016	Aug	GA	Adults	Female	68	Y
329	2016	Aug	GA	Adults	Female	43	Y
330	2016	Sep	GA	Adults	Male	22	Y
332	2016	Sep	KZ	Adults	Female	43	Y
333	2016	Sep	GA	Adults	Male	71	Y
334	2016	Sep	KZ	Adults	Female	39	Y
335	2016	Sep	GA	Adults	Male	76	Y
336	2016	Sep	KZ	Paeds	Male	0	D
337	2016	Oct	GA	Paeds	Male	24	M
338	2016	Sep	GA	Paeds	Male	14	Y
340	2016	Oct	GA	Adults	Male	50	Y
341	2016	Oct	GA	Paeds	Female	22	M
342	2016	Oct	GA	Paeds	Male	11	D
343	2016	Sep	GA	Adults	Female	29	Y
344	2016	Sep	GA	Adults	Female	64	Y
345	2016	Sep	GA	Adults	Female	59	Y
346	2016	Sep	GA	Adults	Male	53	Y
347	2016	Sep	GA	Adults	Female	34	Y
349	2016	Sep	GA	Paeds	Male	13	M
350	2016	Oct	GA	Paeds	Male	2	M
351	2016	Oct	GA	Unkown	Female		
352	2016	Oct	GA	Adults	Female	36	Y
353	2016	Oct	GA	Adults	Male	59	Y
354	2016	Oct	GA	Adults	Male	67	Y
355	2016	Oct	GA	Paeds	Male	23	M
356	2016	Oct	GA	Paeds	Male	12	M
357	2016	Oct	GA	Paeds	Female	55	M
358	2016	Oct	KZ	Adults	Female	35	Y
359	2016	Oct	KZ	Paeds	Female	1	M
360	2016	Oct	GA	Adults	Male	71	Y
361	2016	Oct	GA	Adults	Female	48	Y
362	2016	Oct	WC	Paeds	Female	7	Y
363	2016	Oct	KZ	Adults	Male	53	Y
364	2016	Oct	GA	Adults	Female	63	Y
365	2016	Nov	GA	Adults	Female	59	Y
366	2016	Nov	GA	Adults	Female	54	Y
367	2016	Nov	WC	Adults	Female	48	Y
368	2016	Nov	KZ	Adults	Male	31	Y
369	2016	Nov	GA	Adults	Male	36	Y
370	2016	Nov	KZ	Paeds	Female	4	D
375	2016	Oct	KZ	Adults	Female	37	Y
376	2016	Nov	WC	Adults	Male	77	Y
377	2016	Oct	GA	Adults	Male	64	Y
378	2016	Aug	GA	Adults	Male	61	Y
380	2016	Nov	GA	Adults	Female	31	Y
381	2016	May	GA	Paeds	Female	3	D
382	2016	Oct	GA	Adults	Female	19	Y
384	2016	Sep	GA	Adults	Female	35	Y
385	2016	Sep	GA	Adults	Female	64	Y
386	2016	Sep	GA	Adults	Male	55	Y
387	2016	Oct	GA	Paeds	Male	36	M
388	2016	Sep	GA	Paeds	Female	20	M
389	2016	Oct	GA	Adults	Male	22	Y
390	2016	Dec	GA	Adults	Male	51	Y
391	2016	Nov	GA	Paeds	Male	37	M

392	2016	Dec	GA	Adults	Female	19	Y
393	2016	Oct	GA	Paeds	Male	16	Y
394	2016	Nov	GA	Adults	Female	49	Y
395	2016	Nov	GA	Adults	Female	69	Y
396	2016	Nov	GA	Paeds	Male	5	M
397	2016	Nov	GA	Paeds	Male	40	M
399	2016	Nov	GA	Adults	Male	53	Y
402	2016	Nov	GA	Paeds	Male	38	M
403	2016	Nov	GA	Adults	Male	46	Y
404	2016	Nov	GA	Paeds	Male	20	M
405	2016	Nov	GA	Paeds	Male	12	Y
406	2016	Nov	GA	Paeds	Male	8	D
408	2016	Dec	GA	Paeds	Female	18	D
409	2016	Dec	GA	Adults	Female	57	Y
410	2016	Dec	GA	Adults	Male	44	Y
411	2016	Dec	KZ	Paeds	Female	15	M
412	2016	Nov	KZ	Adults	Female	39	Y
413	2016	Dec	GA	Adults	Male	88	Y
414	2016	Dec	WC	Adults	Male	60	Y
417	2016	Dec	GA	Adults	Male	60	Y
423	2016	Jul	GA	Adults	Female	60	Y
440	2016	Dec	GA	Adults	Female	40	Y
450	2016	Jan	KZ	Paeds	Female	13	M
451	2016	Jan	GA	Adults	Female	54	Y
455	2016	Jan	GA	Unkown	Male		
456	2016	Jan	KZ	Adults	Male	32	Y
458	2016	Jan	GA	Adults	Male	41	Y
459	2016	Feb	GA	Adults	Female	63	Y
461	2016	Mar	GA	Adults	Female	24	Y
462	2016	Mar	KZ	Paeds	Female	6	D
463	2016	Mar	KZ	Adults	Female	36	Y
464	2016	Apr	GA	Adults	Male	46	Y
465	2016	Apr	GA	Adults	Female	56	Y
466	2016	Apr	GA	Adults	Female	53	Y
467	2016	Apr	KZ	Adults	Female	53	Y
468	2016	Apr	KZ	Paeds	Male	1	M
469	2016	May	GA	Adults	Female	43	Y
470	2016	May	GA	Adults	Female	66	Y
472	2016	May	KZ	Adults	Male	21	Y
473	2016	May	KZ	Adults	Male	25	Y
474	2016	May	FS	Adults	Female	55	Y
475	2016	Jun	GA	Adults	Male	37	Y
476	2016	Jun	GA	Adults	Male	50	Y
477	2016	Jun	GA	Paeds	Male	1	M
478	2016	Jun	GA	Paeds	Male	45	M
480	2016	Jun	GA	Adults	Female	38	Y
481	2016	Jun	GA	Paeds	Male	11	Y
484	2016	Jul	GA	Adults	Male	29	Y
485	2016	Jul	KZ	Paeds	Male	12	D
486	2016	Jul	KZ	Paeds	Female	3	M
487	2016	Jul	KZ	Paeds	Female	6	D
488	2016	Aug	KZ	Adults	Female	32	Y
489	2016	Aug	GA	Adults	Male	49	Y
490	2016	Aug	GA	Adults	Male	73	Y
492	2016	Jan	WC	Adults	Female	29	Y
496	2016	Mar	WC	Adults	Female	76	Y

497	2016	Mar	WC	Adults	Male	57	Y
504	2016	May	FS	Paeds	Female	14	D
509	2016	Jun	WC	Paeds	Male	3	M
510	2016	Jul	WC	Adults	Male	34	Y
512	2016	Jul	WC	Adults	Male	57	Y
513	2016	Aug	WC	Adults	Female	59	Y
514	2016	Aug	GA	Adults	Male	48	Y
515	2016	Aug	WC	Adults	Female	83	Y
516	2016	Aug	WC	Adults	Male	41	Y
524	2016	May	KZ	Paeds	Female	15	D
530	2016	Dec	WC	Adults	Male	61	Y
532	2016	Oct	GA	Adults	Female	67	Y
539	2016	Dec	GA	Adults	Male	51	Y
540	2016	Dec	GA	Adults	Male	41	Y
542	2016	Jun	GA	Adults	Female	32	Y
543	2016	Aug	GA	Adults	Male	34	Y
544	2016	Aug	GA	Paeds	Male	6	Y
545	2016	Aug	GA	Adults	Male	60	Y
546	2016	Aug	GA	Adults	Female	59	Y
548	2016	Aug	WC	Adults	Female	32	Y
549	2016	Sep	GA	Adults	Female	58	Y
550	2016	Sep	GA	Adults	Male	62	Y
551	2016	Sep	GA	Paeds	Male	1	M
553	2016	Sep	GA	Adults	Male	65	Y
554	2016	Sep	GA	Adults	Female	60	Y
555	2016	Sep	WC	Adults	Female	28	Y
556	2016	Oct	GA	Adults	Female	41	Y
557	2016	Oct	GA	Adults	Female	46	Y
559	2016	Oct	GA	Adults	Female	52	Y
560	2016	Nov	KZ	Adults	Female	34	Y
562	2016	Nov	GA	Adults	Male	54	Y
563	2016	Nov	KZ	Adults	Female	57	Y
564	2016	Nov	GA	Adults	Male	76	Y
565	2016	Nov	GA	Adults	Male	34	Y
566	2016	Nov	GA	Adults	Female	46	Y
569	2016	Dec	GA	Unkown	Female		
570	2016	Dec	GA	Adults	Female	50	Y
571	2016	Dec	WC	Adults	Female	81	Y
572	2016	Dec	GA	Adults	Male	36	Y
573	2016	Dec	GA	Paeds	Male	9	M
574	2016	Dec	WC	Adults	Male	57	Y
575	2016	Dec	GA	Adults	Female	37	Y
576	2016	Dec	GA	Paeds	Male	4	D
577	2016	Dec	GA	Adults	Male	24	Y
579	2016	Dec	GA	Unkown	Male		
580	2016	Dec	KZ	Adults	Female	21	Y
1022	2016	Apr	WC	Adults	Female	30	Y
415	2017	Jan	GA	Paeds	Male	10	D
418	2017	Jan	GA	Paeds	Male	12	D
419	2017	Jan	GA	Paeds	Male	6	M
420	2017	Jan	KZ	Paeds	Female	1	M
421	2017	Jan	KZ	Paeds	Female	14	Y
422	2017	Jan	KZ	Paeds	Male	1	M
424	2017	Jan	KZ	Adults	Male	68	Y
425	2017	Jan	GA	Adults	Male	61	Y
426	2017	Jan	WC	Adults	Female	46	Y

427	2017	Jan	GA	Adults	Female	39	Y
430	2017	Jan	GA	Paeds	Male	12	M
431	2017	Jan	GA	Adults	Female	37	Y
432	2017	Jan	GA	Adults	Male	46	Y
434	2017	Jan	GA	Paeds	Female	14	Y
436	2017	Jan	GA	Adults	Female	61	Y
437	2017	Jan	GA	Adults	Male	78	Y
438	2017	Jan	GA	Adults	Male	54	Y
441	2017	Feb	GA	Adults	Male	71	Y
442	2017	Jan	GA	Adults	Female	67	Y
443	2017	Jan	GA	Adults	Male	61	Y
444	2017	Jan	GA	Adults	Female	32	Y
445	2017	Jan	GA	Paeds	Male	1	D
446	2017	Jan	GA	Adults	Male	52	Y
447	2017	Jan	GA	Paeds	Female	16	Y
448	2017	Jan	GA	Adults	Male	52	Y
452	2017	Feb	GA	Adults	Male	68	Y
453	2017	Jan	WC	Paeds	Female	28	M
457	2017	Jan	GA	Adults	Male	27	Y
517	2017	Jan	GA	Adults	Male	57	Y
518	2017	Feb	GA	Adults	Male	47	Y
519	2017	Feb	GA	Adults	Male	46	Y
520	2017	Feb	GA	Paeds	Male	4	M
521	2017	Feb	GA	Paeds	Male	1	M
522	2017	Feb	GA	Adults	Female	25	Y
523	2017	Feb	GA	Adults	Female	43	Y
525	2017	Feb	GA	Paeds	Male	1	M
526	2017	Feb	GA	Paeds	Female	1	M
527	2017	Feb	KZ	Adults	Female	58	Y
528	2017	Feb	KZ	Adults	Male	59	Y
529	2017	Jan	GA	Paeds	Male	6	D
531	2017	Jan	KZ	Paeds	Male	9	D
533	2017	Feb	GA	Adults	Female	24	Y
534	2017	Jan	GA	Adults	Female	73	Y
535	2017	Jan	GA	Adults	Female	44	Y
536	2017	Jan	GA	Paeds	Male	1	M
537	2017	Feb	GA	Adults	Female	48	Y
538	2017	Feb	GA	Adults	Female	34	Y
541	2017	Jan	GA	Adults	Male	47	Y
582	2017	Feb	KZ	Adults	Male	38	Y
583	2017	Mar	GA	Adults	Male	37	Y
585	2017	Mar	KZ	Adults	Female	66	Y
586	2017	Mar	GA	Adults	Male	38	Y
588	2017	Feb	KZ	Paeds	Male	5	Y
589	2017	Mar	GA	Adults	Male	32	Y
590	2017	Mar	GA	Adults	Male	68	Y
591	2017	Mar	GA	Adults	Female	18	Y
592	2017	Mar	WC	Adults	Female	50	Y
593	2017	Mar	GA	Adults	Male	56	Y
594	2017	Mar	GA	Paeds	Male	9	D
596	2017	Feb	FS	Adults	Male	35	Y
597	2017	Mar	GA	Adults	Female	64	Y
598	2017	Mar	GA	Adults	Male	27	Y
599	2017	Mar	GA	Adults	Female	31	Y
600	2017	Mar	KZ	Paeds	Male	15	D
601	2017	Mar	GA	Adults	Male	59	Y

602	2017	Mar	GA	Adults	Female	60	Y
603	2017	Feb	GA	Paeds	Female	2	D
604	2017	Mar	GA	Paeds	Female	8	D
605	2017	Mar	GA	Paeds	Female	0	D
606	2017	Feb	GA	Adults	Male	27	Y
607	2017	Mar	GA	Adults	Male	41	Y
608	2017	Mar	GA	Adults	Male	37	Y
609	2017	Mar	GA	Adults	Male	23	Y
610	2017	Mar	GA	Paeds	Male	5	Y
611	2017	Feb	GA	Paeds	Male	2	M
612	2017	Mar	GA	Paeds	Male	2	M
614	2017	Apr	GA	Unkown	Male	51	Y
615	2017	Apr	GA	Unkown	Male	28	Y
616	2017	Apr	GA	Adults	Female	39	Y
617	2017	Mar	KZ	Paeds	Male	11	Y
618	2017	Mar	KZ	Paeds	Male	12	Y
619	2017	Mar	GA	Adults	Female	60	Y
620	2017	Apr	WC	Paeds	Male	24	D
622	2017	Apr	GA	Adults	Female	82	Y
623	2017	Apr	GA	Adults	Male	64	Y
624	2017	Apr	WC	Paeds	Male	30	D
625	2017	Apr	GA	Adults	Male	70	Y
626	2017	Apr	GA	Paeds	Male	13	D
627	2017	Apr	GA	Paeds	Female	10	Y
628	2017	Apr	GA	Paeds	Male	5	D
629	2017	Mar	GA	Adults	Female	62	Y
630	2017	Apr	GA	Paeds	Male	10	Y
631	2017	Apr	KZ	Paeds	Male	10	Y
633	2017	Jan	GA	Adults	Male	29	Y
634	2017	Jan	KZ	Adults	Female	82	Y
635	2017	Jan	WC	Adults	Male	48	Y
636	2017	Feb	GA	Paeds	Female	6	M
637	2017	Feb	GA	Adults	Male	29	Y
638	2017	Feb	GA	Adults	Female	34	Y
639	2017	Apr	GA	Adults	Male	66	Y
640	2017	Feb	GA	Adults	Male	64	Y
641	2017	Feb	GA	Adults	Female	36	Y
642	2017	Feb	GA	Adults	Male	45	Y
643	2017	Feb	GA	Adults	Female	67	Y
644	2017	Mar	GA	Paeds	Male	6	Y
645	2017	Mar	GA	Adults	Female	24	Y
646	2017	Mar	GA	Adults	Female	42	Y
647	2017	Mar	KZ	Adults	Male	27	Y
648	2017	Mar	GA	Unkown	Female	16	Y
649	2017	Mar	GA	Adults	Female	74	Y
650	2017	Mar	GA	Adults	Female	56	Y
651	2017	Mar	GA	Adults	Female	62	Y
652	2017	Mar	GA	Adults	Female	54	Y
653	2017	Mar	KZ	Adults	Male	55	Y
654	2017	Mar	GA	Adults	Male	32	Y
655	2017	Mar	GA	Paeds	Male	2	M
656	2017	Mar	WC	Unkown	Male	57	Y
657	2017	Mar	GA	Adults	Female	23	Y
658	2017	Mar	GA	Adults	Male	32	Y
659	2017	Mar	GA	Adults	Female	28	Y
660	2017	Apr	GA	Adults	Male	37	Y

661	2017	Apr	GA	Paeds	Female	6	D
662	2017	Apr	GA	Adults	Male	35	Y
663	2017	May	GA	Adults	Female	30	Y
664	2017	Apr	GA	Adults	Male	69	Y
665	2017	Apr	GA	Paeds	Male	32	M
667	2017	May	GA	Paeds	Male	15	D
668	2017	May	GA	Paeds	Male	26	D
669	2017	Apr	GA	Adults	Male	65	Y
670	2017	Apr	GA	Adults	Female	68	Y
673	2017	Apr	GA	Adults	Female	49	Y
674	2017	Mar	KZ	Adults	Male	43	Y
675	2017	Feb	GA	Adults	Male	61	Y
676	2017	Feb	GA	Paeds	Female	57	M
678	2017	Apr	GA	Adults	Female	34	Y
679	2017	Mar	GA	Adults	Female	56	Y
680	2017	Mar	GA	Adults	Female	57	Y
681	2017	Apr	GA	Paeds	Female	3	M
682	2017	May	GA	Adults	Male	34	Y
683	2017	May	KZ	Adults	Male	68	Y
684	2017	May	WC	Paeds	Male	17	M
685	2017	May	GA	Adults	Male	49	Y
686	2017	May	GA	Adults	Male	31	Y
687	2017	May	WC	Adults	Female	68	Y
688	2017	May	GA	Paeds	Female	4	M
689	2017	Apr	GA	Adults	Female	26	Y
690	2017	May	GA	Paeds	Male	8	Y
691	2017	May	GA	Unkown	Male	62	Y
692	2017	May	WC	Adults	Male	77	Y
693	2017	May	GA	Paeds	Female	6	D
694	2017	May	WC	Paeds	Female	1	M
695	2017	May	GA	Paeds	Male	28	D
696	2017	May	GA	Adults	Female	26	Y
697	2017	May	GA	Paeds	Male	1	M
698	2017	May	FS	Adults	Male	59	Y
699	2017	May	GA	Adults	Male	50	Y
700	2017	May	GA	Adults	Female	51	Y
701	2017	May	GA	Paeds	Female	11	D
702	2017	May	GA	Paeds	Male	1	M
703	2017	May	GA	Adults	Male	87	Y
704	2017	May	WC	Adults	Female	48	Y
705	2017	May	GA	Adults	Male	27	Y
706	2017	Apr	GA	Unkown	Male	37	Y
707	2017	Jun	KZ	Paeds	Female	13	M
708	2017	May	GA	Adults	Male	64	Y
709	2017	May	GA	Paeds	Female	1	M
710	2017	Jun	KZ	Adults	Female	38	Y
711	2017	May	GA	Paeds	Male	11	D
712	2017	May	GA	Unkown	Male	57	Y
713	2017	May	GA	Unkown	Female	36	Y
714	2017	Apr	GA	Unkown	Male	77	Y
715	2017	Apr	GA	Unkown	Male		
716	2017	Jun	FS	Adults	Male	58	Y
717	2017	Apr	GA	Paeds	Female	0	D
718	2017	Jun	GA	Adults	Male	27	Y
719	2017	Jun	FS	Adults	Male	67	Y
721	2017	May	GA	Adults	Male	75	Y

722	2017	May	GA	Unkown	Female	44	Y
723	2017	Jun	GA	Adults	Female	59	Y
724	2017	May	GA	Adults	Female	58	Y
725	2017	May	GA	Adults	Female	66	Y
726	2017	May	GA	Adults	Female	25	Y
727	2017	May	WC	Adults	Male	45	Y
728	2017	Jun	GA	Paeds	Male	2	M
729	2017	Jun	GA	Paeds	Female	10	D
802	2017	Jun	GA	Adults	Male	54	Y
803	2017	Jun	KZ	Paeds	Female	1	M
913	2017	Jun	KZ	Adults	Female	58	Y
914	2017	Jun	KZ	Adults	Female	57	Y
915	2017	Jun	KZ	Unkown	Female	71	Y
916	2017	Jun	GA	Unkown	Male	36	Y
917	2017	Jun	GA	Paeds	Male	4	M
919	2017	Jun	GA	Unkown	Female	28	Y
920	2017	Jun	GA	Unkown	Male	72	Y
921	2017	Jun	GA	Unkown	Unknown		Y
922	2017	Jun	GA	Unkown	Female	44	Y
923	2017	Jun	GA	Adults	Male	23	Y
924	2017	May	GA	Adults	Male	69	Y
925	2017	Jun	GA	Adults	Female	31	Y
926	2017	Jul	KZ	Paeds	Male	24	D
927	2017	Jul	KZ	Paeds	Male	24	D
928	2017	May	KZ	Adults	Male	37	Y
929	2017	Apr	GA	Paeds	Male	13	M
930	2017	May	GA	Adults	Male	45	Y
931	2017	May	GA	Adults	Female	33	Y
932	2017	Jun	GA	Adults	Male	29	Y
933	2017	Jun	GA	Paeds	Male	8	D
934	2017	Jun	GA	Adults	Female	56	Y
935	2017	Jul	GA	Adults	Female	43	Y
936	2017	Jul	GA	Adults	Male	30	Y
937	2017	Jun	GA	Adults	Female	61	Y
939	2017	Jun	WC	Adults	Female	30	Y
940	2017	Jun	WC	Adults	Female	30	Y
941	2017	Jul	GA	Adults	Female	53	Y
942	2017	Jul	GA	Adults	Male	31	Y
943	2017	Jul	WC	Paeds	Male	14	Y
944	2017	Jul	FS	Adults	Female	42	Y
945	2017	Jul	GA	Paeds	Female	2	M
946	2017	Jul	WC	Adults	Male	47	Y
947	2017	Jul	GA	Adults	Male	43	Y
948	2017	Jul	GA	Paeds	Male	5	M
950	2017	Jul	GA	Adults	Male	25	Y
951	2017	Jul	FS	Adults	Male	26	Y
952	2017	Jul	KZ	Paeds	Male	8	M
955	2017	Aug	GA	Paeds	Male	24	M
956	2017	Jul	GA	Paeds	Female	1	M
957	2017	Jul	GA	Adults	Female	68	Y
959	2017	Jul	GA	Adults	Female	42	Y
960	2017	Jul	GA	Paeds	Female	5	D
961	2017	Jul	GA	Unkown	Female	67	Y
962	2017	Jul	GA	Unkown	Male		
963	2017	Jul	GA	Adults	Male	42	Y
964	2017	Jul	GA	Adults	Male	57	Y

965	2017	Jul	GA	Unkown	Male	22	Y
966	2017	Jul	GA	Paeds	Female	56	M
967	2017	Jul	GA	Unkown	Male	40	Y
968	2017	Jul	GA	Paeds	Male	16	M
969	2017	Jul	GA	Paeds	Male	13	M
970	2017	Jul	GA	Unkown	Male	44	Y
971	2017	Jul	GA	Adults	Male	16	Y
973	2017	Jul	GA	Paeds	Male	1	M
974	2017	Jul	GA	Adults	Female	2	M
975	2017	Jul	GA	Adults	Male	55	Y
976	2017	Jul	GA	Paeds	Male	2	D
977	2017	Jul	KZ	Paeds	Female	25	M
979	2017	Jul	KZ	Adults	Male	66	Y
980	2017	Aug	GA	Adults	Male	19	Y
981	2017	Aug	GA	Adults	Male	65	Y
983	2017	Jul	GA	Paeds	Male	23	M
984	2017	Jul	GA	Paeds	Female	9	D
985	2017	Jul	GA	Paeds	Female	1	M
986	2017	Jul	GA	Paeds	Male	18	D
987	2017	Jul	GA	Adults	Female	17	Y
988	2017	Aug	GA	Adults	Male	34	Y
989	2017	Jul	GA	Adults	Female	38	Y
990	2017	Jul	GA	Adults	Male	34	Y
991	2017	Jul	GA	Adults	Male	29	Y
992	2017	Aug	KZ	Adults	Male	39	Y
994	2017	Aug	KZ	Paeds	Female	1	M
995	2017	Aug	GA	Adults	Male	55	Y
996	2017	Aug	GA	Adults	Female	49	Y
997	2017	Jun	GA	Paeds	Female	2	M
999	2017	Aug	GA	Adults	Male	57	Y
1000	2017	Aug	GA	Adults	Female	26	Y
1001	2017	Aug	GA	Unkown	Male	10	D
1002	2017	Aug	GA	Adults	Male	16	Y
1003	2017	Aug	GA	Unkown	Female	75	Y
1004	2017	Aug	GA	Adults	Female	68	Y
1006	2017	Aug	GA	Paeds	Male	17	D
1007	2017	Aug	KZ	Paeds	Male	1	D
1008	2017	Aug	GA	Adults	Male	22	Y
1009	2017	Aug	GA	Adults	Male	22	Y
1010	2017	Aug	GA	Adults	Male	60	Y
1011	2017	Aug	GA	Paeds	Male	11	Y
1012	2017	Aug	WC	Adults	Female	68	Y
1013	2017	Jun	FS	Adults	Male	64	Y
1014	2017	Aug	GA	Adults	Female	68	Y
1015	2017	Feb	FS	Adults	Male	42	Y
1016	2017	Sep	KZ	Paeds	Female	8	D
1017	2017	Sep	KZ	Paeds	Male	13	D
1018	2017	Sep	GA	Adults	Male	25	Y
1019	2017	Sep	GA	Adults	Male	33	Y
1020	2017	Sep	GA	Adults	Male	67	Y
1021	2017	Sep	FS	Adults	Male	37	Y
1023	2017	Jul	GA	Adults	Male	55	Y
1024	2017	Jun	WC	Adults	Female	65	Y
1025	2017	May	GA	Adults	Female	30	Y
1026	2017	Aug	GA	Paeds	Male	36	M
1027	2017	Sep	GA	Adults	Male	36	Y

1028	2017	Aug	KZ	Paeds	Female	11	D
1029	2017	Sep	GA	Paeds	Male	46	Y
1030	2017	Sep	KZ	Paeds	Female	18	D
1031	2017	Sep	GA	Adults	Female	76	Y
1032	2017	Sep	GA	Adults	Male	71	Y
1033	2017	Sep	GA	Paeds	Male	12	Y
1034	2017	Sep	GA	Adults	Male	75	Y
1036	2017	Oct	KZ	Adults	Male	19	Y
1037	2017	Sep	GA	Paeds	Male	1	M
1038	2017	Oct	KZ	Adults	Female	46	Y
1039	2017	Oct	KZ	Paeds	Male	28	D
1040	2017	Oct	KZ	Adults	Male	25	Y
1041	2017	Oct	KZ	Paeds	Male	9	D
1042	2017	Aug	GA	Adults	Male	24	Y
1043	2017	Aug	GA	Adults	Female	74	Y
1044	2017	Oct	GA	Adults	Female	36	Y
1046	2017	Oct	GA	Adults	Male	29	Y
1049	2017	Oct	KZ	Adults	Male	40	Y
1050	2017	Oct	GA	Adults	Female	30	Y
1051	2017	Sep	GA	Paeds	Male	14	Y
1052	2017	Oct	KZ	Paeds	Female	6	D
1054	2017	Oct	GA	Adults	Male	47	Y
1055	2017	Oct	GA	Paeds	Male	4	D
1056	2017	Oct	KZ	Adults	Female	23	Y
1057	2017	Oct	KZ	Paeds	Male	13	D
1058	2017	Oct	KZ	Paeds	Female	25	D
1059	2017	Oct	KZ	Paeds	Male	8	D
1061	2017	Oct	GA	Paeds	Male	39	Y
1062	2017	Oct	GA	Paeds	Male	1	M
1063	2017	Nov	WC	Adults	Male	56	Y
1064	2017	Oct	FS	Paeds	Female	12	Y
1065	2017	Oct	GA	Paeds	Female	0	D
1066	2017	Nov	GA	Adults	Female	52	Y
1067	2017	Oct	GA	Adults	Male	36	Y
1068	2017	Nov	KZ	Paeds	Female	1	M
1069	2017	Nov	KZ	Adults	Male	40	Y
1070	2017	Oct	GA	Adults	Male	47	Y
1071	2017	Nov	GA	Adults	Male	35	Y
1072	2017	Oct	GA	Adults	Male	51	Y
1073	2017	Oct	FS	Adults	Female	50	Y
1074	2017	Nov	GA	Adults	Male	28	Y
1075	2017	Sep	KZ	Paeds	Male	6	D
1076	2017	Nov	GA	Adults	Male	37	Y
1077	2017	Nov	WC	Paeds	Male	24	M
1078	2017	Nov	GA	Adults	Female	19	Y
1079	2017	Nov	GA	Adults	Male	42	Y
1080	2017	Dec	GA	Adults	Male	48	Y
1081	2017	Nov	KZ	Paeds	Female	0	D
1082	2017	Nov	KZ	Paeds	Male	13	D
1083	2017	Nov	KZ	Paeds	Female	50	M
1084	2017	Nov	KZ	Paeds	Male	11	D
1085	2017	Dec	GA	Adults	Female	32	Y
1088	2017	Dec	GA	Adults	Male	36	Y
1089	2017	Nov	KZ	Paeds	Male	22	M
1091	2017	Dec	GA	Adults	Female	75	Y
1092	2017	Dec	KZ	Unkown	Male	1	M

1093	2017	Dec	KZ	Paeds	Male	1	M
1095	2017	Dec	GA	Adults	Male	57	Y
1097	2017	Dec	GA	Adults	Male	36	Y
1098	2017	Dec	GA	Adults	Female	31	Y
1099	2017	Dec	GA	Adults	Male	36	Y
1100	2017	Dec	GA	Adults	Female	27	Y
1105	2017	Dec	GA	Adults	Female	92	Y
1106	2017	Dec	GA	Adults	Female	49	Y
1107	2017	Dec	GA	Adults	Female	55	Y
1108	2017	Dec	GA	Adults	Male	70	Y
1111	2017	Dec	GA	Adults	Female	23	Y
1112	2017	Dec	GA	Adults	Female	65	Y
1114	2017	Nov	GA	Adults	Female	44	Y
1121	2017	Dec	GA	Adults	Female	85	Y
1168	2017	Jan	KZ	Adults	Male	43	Y
1169	2017	Apr	GA	Adults	Male	59	Y
1170	2017	Apr	GA	Adults	Male	28	Y
1171	2017	Apr	KZ	Adults	Female	29	Y
1172	2017	Apr	KZ	Paeds	Male	6	D
1173	2017	Apr	GA	Adults	Male	51	Y
1174	2017	Apr	GA	Adults	Male	65	Y
1175	2017	Apr	GA	Paeds	Male	10	Y
1176	2017	Apr	GA	Adults	Male	78	Y
1177	2017	Apr	GA	Adults	Male	17	Y
1178	2017	Apr	GA	Unkown	Male	40	Y
1179	2017	Apr	KZ	Adults	Male	17	Y
1180	2017	May	GA	Paeds	Male	24	D
1181	2017	May	GA	Adults	Female	71	Y
1182	2017	May	KZ	Adults	Female	34	Y
1183	2017	Jun	GA	Adults	Male	27	Y
1184	2017	Jun	GA	Adults	Female	60	Y
1185	2017	Jul	GA	Adults	Male	62	Y
1186	2017	Jul	GA	Adults	Female	87	Y
1187	2017	Jul	GA	Adults	Male	44	Y
1188	2017	Jul	GA	Unkown	Female	19	M
1189	2017	Aug	GA	Paeds	Male	5	M
1190	2017	Aug	GA	Adults	Male	33	Y
1191	2017	Aug	GA	Adults	Female	55	Y
1192	2017	Aug	GA	Unkown	Male	37	Y
1193	2017	Aug	GA	Adults	Female	62	Y
1194	2017	Aug	GA	Paeds	Female	14	Y
1195	2017	Aug	GA	Paeds	Male	17	M
1196	2017	Aug	GA	Adults	Male	64	Y
1197	2017	Aug	GA	Adults	Male	34	Y
1198	2017	Aug	GA	Paeds	Male	1	D
1199	2017	Aug	GA	Paeds	Male	1	D
1200	2017	Aug	GA	Paeds	Male	2	M
1201	2017	Aug	GA	Paeds	Female	1	D
1202	2017	Aug	GA	Paeds	Female	1	D
1203	2017	Aug	GA	Paeds	Female	25	D
1204	2017	Aug	GA	Paeds	Male	1	M
1205	2017	Aug	GA	Paeds	Male	1	M
1206	2017	Aug	GA	Paeds	Female	7	D
1207	2017	Aug	GA	Paeds	Female	7	D
1208	2017	Sep	GA	Unkown	Male	36	Y
1209	2017	Sep	GA	Paeds	Female	0	D

1210	2017	Sep	KZ	Adults	Female	31	Y
1211	2017	Sep	GA	Adults	Female	45	Y
1212	2017	Sep	GA	Unkown	Male	69	Y
1213	2017	Sep	GA	Paeds	Female	14	Y
1214	2017	Sep	GA	Unkown	Male	4	M
1215	2017	Sep	GA	Adults	Male	45	Y
1216	2017	Sep	GA	Adults	Male	23	Y
1217	2017	Sep	GA	Adults	Female	66	Y
1218	2017	Sep	GA	Adults	Male	44	Y
1219	2017	Sep	KZ	Adults	Male	60	Y
1220	2017	Sep	GA	Unkown	Male	46	Y
1221	2017	Oct	GA	Paeds	Male	6	M
1222	2017	Oct	GA	Unkown	Male	3	M
1223	2017	Oct	GA	Paeds	Female	2	M
1224	2017	Oct	KZ	Adults	Female	37	Y
1225	2017	Oct	KZ	Paeds	Male	1	M
1226	2017	Oct	GA	Paeds	Male	2	M
1227	2017	Oct	GA	Adults	Male	49	Y
1229	2017	Oct	GA	Adults	Female	50	Y
1230	2017	Oct	GA	Paeds	Male	15	M
1231	2017	Oct	GA	Adults	Female	43	Y
1232	2017	Oct	GA	Adults	Female	37	Y
1233	2017	Oct	KZ	Unkown	Female		
1234	2017	Oct	GA	Paeds	Female	53	M
1235	2017	Oct	GA	Paeds	Male	5	Y
1236	2017	Oct	GA	Unkown	Female		
1237	2017	Oct	GA	Adults	Male	17	Y
1238	2017	Oct	KZ	Paeds	Female	33	M
1239	2017	Oct	GA	Paeds	Female	14	Y
1240	2017	Oct	GA	Unkown	Female	69	Y
1241	2017	Oct	KZ	Paeds	Female	12	D
1242	2017	Oct	GA	Adults	Female	27	Y
1243	2017	Oct	GA	Adults	Male	31	Y
1244	2017	Oct	KZ	Paeds	Male	0	D
1245	2017	Oct	KZ	Paeds	Male	6	D
1246	2017	Oct	GA	Adults	Male	40	Y
1247	2017	Oct	GA	Adults	Male	42	Y
1248	2017	Nov	GA	Adults	Male	17	Y
1249	2017	Nov	GA	Adults	Male	25	Y
1250	2017	Nov	GA	Adults	Male	49	Y
1251	2017	Nov	GA	Unkown	Female		
1252	2017	Nov	GA	Adults	Male	39	Y
1253	2017	Nov	GA	Paeds	Female	36	M
1254	2017	Nov	GA	Adults	Male	31	Y
1255	2017	Nov	GA	Adults	Male	66	Y
1256	2017	Nov	GA	Adults	Female	72	Y
1257	2017	Nov	GA	Paeds	Female	7	Y
1258	2017	Nov	GA	Paeds	Male	2	M
1259	2017	Nov	GA	Unkown	Female		
1260	2017	Nov	GA	Unkown	Female	61	Y
1261	2017	Nov	GA	Paeds	Male	57	M
1262	2017	Nov	GA	Adults	Male	63	Y
1263	2017	Nov	GA	Adults	Male	66	Y
1264	2017	Nov	GA	Unkown	Male		
1265	2017	Nov	GA	Paeds	Female	8	M
1266	2017	Nov	KZ	Adults	Female	54	Y

1267	2017	Nov	GA	Adults	Female	61	Y
1268	2017	Dec	GA	Unkown	Female	82	Y
1269	2017	Dec	GA	Adults	Female	32	Y
1270	2017	Dec	GA	Paeds	Male	14	Y
1271	2017	Dec	GA	Unkown	Male	63	Y
1272	2017	Dec	GA	Paeds	Female	14	Y
1273	2017	Dec	GA	Paeds	Female	59	M
1274	2017	Dec	GA	Adults	Male	24	Y
1275	2017	Dec	GA	Adults	Male	45	Y
1276	2017	Dec	GA	Adults	Female	30	Y
1277	2017	Dec	GA	Adults	Female	70	Y
1278	2017	Dec	GA	Adults	Male	68	Y
1279	2017	Dec	GA	Adults	Female	30	Y
1280	2017	Dec	GA	Paeds	Female	2	M
1281	2017	Dec	GA	Paeds	Male	6	Y
1282	2017	Dec	GA	Adults	Male	60	Y
1283	2017	Dec	GA	Paeds	Male	4	M
1284	2017	Dec	GA	Paeds	Male	25	M
1293	2017	Oct	GA	Adults	Male	52	Y
1294	2017	Nov	GA	Paeds	Male	5	Y
1295	2017	Dec	GA	Paeds	Male	14	Y
1296	2017	Dec	GA	Paeds	Male	14	Y
1297	2017	Dec	GA	Adults	Female	26	Y
1298	2017	Dec	GA	Adults	Female	62	Y
1094	2018	Jan	KZ	Adults	Male	15	D
1096	2018	Jan	GA	Adults	Male	32	Y
1101	2018	Jan	GA	Paeds	Female	22	D
1102	2018	Jan	GA	Adults	Male	62	Y
1103	2018	Jan	GA	Paeds	Female	1	M
1104	2018	Jan	KZ	Adults	Female	29	Y
1109	2018	Jan	GA	Adults	Male	47	Y
1110	2018	Jan	KZ	Paeds	Male	3	M
1113	2018	Jan	GA	Adults	Female	21	Y
1115	2018	Jan	GA	Paeds	Male	4	M
1116	2018	Jan	GA	Adults	Female	64	Y
1117	2018	Jan	GA	Paeds	Female	3	M
1118	2018	Jan	GA	Adults	Male	23	Y
1119	2018	Jan	GA	Paeds	Female	57	M
1120	2018	Jan	GA	Paeds	Female	24	M
1122	2018	Jan	GA	Paeds	Female	37	M
1123	2018	Jan	GA	Paeds	Female	42	Y
1124	2018	Jan	GA	Unkown	Male	52	Y
1125	2018	Sep	KZ	Paeds	Female	1	M
1126	2018	Jan	GA	Adults	Female	61	Y
1127	2018	Jan	KZ	Paeds	Male	17	D
1128	2018	Jan	GA	Adults	Female	67	Y
1129	2018	Jan	KZ	Adults	Female	27	Y
1130	2018	Jan	FS	Adults	Male	19	Y
1131	2018	Jan	GA	Adults	Female	26	Y
1132	2018	Jan	GA	Adults	Female	62	Y
1133	2018	Jan	GA	Unkown	Male	42	Y
1134	2018	Jan	GA	Paeds	Female	10	Y
1135	2018	Jan	GA	Adults	Male	40	Y
1136	2018	Jan	GA	Adults	Male	52	Y
1137	2018	Feb	GA	Paeds	Male	6	D
1138	2018	Feb	GA	Paeds	Female	5	D

1139	2018	Feb	GA	Adults	Male	55	Y
1140	2018	Feb	GA	Adults	Male	37	Y
1141	2018	Feb	GA	Paeds	Female		
1142	2018	Feb	GA	Paeds	Female	35	M
1143	2018	Feb	GA	Paeds	Female	7	D
1144	2018	Feb	GA	Paeds	Male	43	M
1145	2018	Feb	GA	Paeds	Male	12	M
1146	2018	Feb	GA	Adults	Female	40	Y
1147	2018	Feb	KZ	Paeds	Male	2	M
1148	2018	Feb	GA	Adults	Female	76	Y
1150	2018	Feb	GA	Adults	Female	59	Y
1151	2018	Feb	GA	Adults	Female	65	Y
1152	2018	Feb	GA	Paeds	Female	2	M
1153	2018	Jan	GA	Adults	Male	22	Y
1154	2018	Feb	GA	Paeds	Female	9	D
1155	2018	Jan	GA	Adults	Female	15	Y
1156	2018	Feb	GA	Unkown	Male	60	Y
1157	2018	Feb	GA	Unkown	Female	47	Y
1158	2018	Feb	GA	Adults	Female	24	Y
1159	2018	Feb	GA	Paeds	Male	11	M
1160	2018	Feb	GA	Paeds	Male	9	Y
1161	2018	Feb	GA	Adults	Female	37	Y
1162	2018	Feb	GA	Adults	Male	38	Y
1163	2018	Feb	GA	Paeds	Male	13	D
1164	2018	Feb	GA	Paeds	Male	11	D
1165	2018	Feb	GA	Paeds	Male	1	M
1166	2018	Mar	GA	Adults	Female	52	Y
1167	2018	Feb	WC	Adults	Female	21	Y
1228	2018	Mar	GA	Paeds	Female	12	M
1285	2018	Mar	GA	Adults	Male	40	Y
1286	2018	Feb	GA	Adults	Female	56	Y
1287	2018	Feb	GA	Adults	Female	63	Y
1288	2018	Mar	GA	Adults	Female	73	Y
1289	2018	Mar	GA	Adults	Female	20	Y
1290	2018	Mar	GA	Adults	Male	49	Y
1291	2018	Feb	GA	Adults	Male	40	Y
1292	2018	Mar	GA	Adults	Female	26	Y
1299	2018	Jan	GA	Adults	Male	26	Y
1300	2018	Jan	GA	Paeds	Male	11	M
1301	2018	Jan	GA	Adults	Female	61	Y
1302	2018	Feb	GA	Adults	Male	33	Y
1303	2018	Feb	GA	Adults	Female	39	Y
1304	2018	Feb	GA	Adults	Female	78	Y
1305	2018	Feb	KZ	Adults	Female	18	Y
1306	2018	Feb	GA	Adults	Male	57	Y
1307	2018	Mar	KZ	Adults	Female	47	Y
1308	2018	Mar	GA	Adults	Female	57	Y
1309	2018	Mar	GA	Unkown	Female	9	D
1310	2018	Feb	GA	Paeds	Female	0	D
1311	2018	Feb	GA	Paeds	Female	1	M
1312	2018	Mar	GA	Paeds	Male	6	Y
1313	2018	Feb	GA	Unkown	Male		
1314	2018	Feb	GA	Adults	Male	72	Y
1315	2018	Feb	GA	Paeds	Unknown	0	D
1316	2018	Mar	GA	Adults	Male	68	Y
1317	2018	Feb	GA	Adults	Male	36	Y

1318	2018	Feb	GA	Paeds	Unknown	0	D
1319	2018	Mar	GA	Adults	Female	27	Y
1320	2018	Mar	GA	Adults	Male	53	Y
1321	2018	Mar	GA	Paeds	Male	2	M
1322	2018	Mar	GA	Paeds	Female	2	D
1323	2018	Mar	GA	Adults	Male	24	Y
1324	2018	Mar	GA	Paeds	Female	11	Y
1325	2018	Mar	GA	Adults	Male	38	Y
1326	2018	Mar	GA	Adults	Male	39	Y
1327	2018	Mar	GA	Adults	Female	42	Y
1328	2018	Mar	GA	Paeds	Female	11	Y
1329	2018	Mar	GA	Adults	Female	50	Y
1330	2018	Mar	GA	Paeds	Female	21	M
1331	2018	Mar	GA	Adults	Female	63	Y
1332	2018	Mar	GA	Adults	Male	28	Y
1333	2018	Mar	KZ	Adults	Male	44	Y
1335	2018	Apr	GA	Adults	Male	31	Y
1336	2018	Apr	GA	Adults	Male	70	Y
1337	2018	Apr	GA	Paeds	Male	13	M
1338	2018	Apr	GA	Adults	Female	52	Y
1340	2018	Mar	GA	Adults	Male	49	Y
1341	2018	Apr	GA	Adults	Male	59	Y
1342	2018	Apr	GA	Adults	Female	22	Y
1343	2018	Apr	GA	Adults	Male	64	Y
1344	2018	Apr	GA	Adults	Female	49	Y
1345	2018	Apr	GA	Adults	Female	24	Y
1346	2018	Apr	GA	Adults	Female	68	Y
1347	2018	Jan	GA	Adults	Female	62	Y
1348	2018	Jan	GA	Paeds	Female	15	D
1351	2018	Feb	GA	Adults	Male	47	Y
1352	2018	Feb	FS	Adults	Female	71	Y
1353	2018	Feb	GA	Adults	Male	62	Y
1354	2018	Mar	KZ	Paeds	Male	15	M
1355	2018	Mar	GA	Adults	Female	17	Y
1357	2018	Mar	GA	Adults	Male	47	Y
1358	2018	Mar	GA	Adults	Male	58	Y
1359	2018	Mar	GA	Adults	Female	47	Y
1360	2018	Mar	GA	Adults	Female	1	D
1361	2018	Mar	GA	Paeds	Male	1	D
1362	2018	Mar	GA	Paeds	Male	1	D
1363	2018	Mar	GA	Adults	Female	22	Y
1364	2018	Mar	KZ	Adults	Female	54	Y
1365	2018	Mar	GA	Adults	Male	69	Y
1366	2018	Apr	GA	Paeds	Male	0	D
1367	2018	Apr	GA	Paeds	Female	21	D
1368	2018	Apr	GA	Adults	Male	53	Y
1369	2018	Apr	GA	Adults	Male	38	Y
1370	2018	May	KZ	Paeds	Female	29	M
1371	2018	Apr	GA	Adults	Male	63	Y
1372	2018	Apr	GA	Paeds	Female	3	D
1373	2018	Nov	KZ	Adults	Female	53	Y
1374	2018	May	WC	Adults	Male	65	Y
1375	2018	May	GA	Adults	Male	37	Y
1377	2018	May	GA	Paeds	Male	7	D
1378	2018	May	GA	Adults	Female	49	Y
1379	2018	May	FS	Adults	Female	71	Y

1381	2018	May	GA	Adults	Female	62	Y
1382	2018	May	GA	Paeds	Male	7	D
1383	2018	May	FS	Adults	Male	68	Y
1384	2018	May	FS	Adults	Female	35	Y
1385	2018	May	GA	Paeds	Female	26	D
1386	2018	May	WC	Paeds	Female	6	M
1387	2018	May	KZ	Adults	Female	44	Y
1388	2018	Jan	GA	Paeds	Female	9	Y
1390	2018	Mar	GA	Adults	Male	60	Y
1391	2018	Mar	GA	Adults	Female	31	Y
1392	2018	Apr	GA	Paeds	Male	8	D
1393	2018	Apr	GA	Paeds	Male	1	M
1394	2018	Apr	GA	Paeds	Male	2	M
1395	2018	Apr	GA	Adults	Male	37	Y
1396	2018	Apr	GA	Paeds	Female	13	D
1397	2018	Apr	GA	Paeds	Female	6	D
1398	2018	Apr	GA	Adults	Female	73	Y
1399	2018	May	GA	Adults	Female	75	Y
1400	2018	May	GA	Paeds	Female	8	M
1401	2018	May	GA	Adults	Male	68	Y
1402	2018	Apr	GA	Adults	Female	55	Y
1404	2018	May	GA	Adults	Male	51	Y
1405	2018	May	GA	Adults	Female	49	Y
1406	2018	May	GA	Adults	Female	38	Y
1407	2018	Jun	GA	Adults	Female	43	Y
1408	2018	Jun	GA	Adults	Female	32	Y
1409	2018	Apr	GA	Paeds	Female	19	D
1410	2018	Apr	GA	Adults	Male	24	Y
1411	2018	Apr	GA	Paeds	Male	1	M
1412	2018	Apr	GA	Paeds	Male	46	M
1413	2018	Jun	GA	Paeds	Female	12	D
1414	2018	Jun	GA	Adults	Female	37	Y
1415	2018	Jun	WC	Adults	Female	59	Y
1416	2018	Jun	GA	Adults	Male	65	Y
1417	2018	Jun	KZ	Adults	Female	42	Y
1418	2018	Jun	GA	Adults	Female	22	Y
1419	2018	Jun	GA	Adults	Female	57	Y
1420	2018	May	GA	Adults	Male	82	Y
1421	2018	May	GA	Adults	Female	33	Y
1425	2018	Jun	GA	Paeds	Male	1	M
1426	2018	Jun	GA	Adults	Male	31	Y
1427	2018	Jun	GA	Adults	Female	86	Y
1430	2018	May	KZ	Paeds	Female	4	D
1432	2018	May	GA	Unkown	Male	4	M
1433	2018	May	GA	Adults	Male	66	Y
1434	2018	Jun	GA	Adults	Male	59	Y
1435	2018	Jun	GA	Adults	Female	49	Y
1436	2018	Jun	GA	Adults	Female	44	Y
1437	2018	Jun	GA	Adults	Male	50	Y
1438	2018	Jun	GA	Adults	Female	32	Y
1440	2018	Jun	GA	Paeds	Male	18	D
1441	2018	May	GA	Adults	Male	57	Y
1442	2018	Jun	WC	Adults	Female	79	Y
1443	2018	Jun	GA	Adults	Male	31	Y
1444	2018	Jul	GA	Adults	Male	43	Y
1445	2018	Jul	GA	Adults	Male	33	Y

1446	2018	Jul	KZ	Adults	Female	30	Y
1447	2018	Jun	WC	Paeds	Male	14	Y
1448	2018	Jul	GA	Adults	Male	70	Y
1449	2018	Jul	GA	Adults	Male	68	Y
1450	2018	Jul	GA	Adults	Male	23	Y
1451	2018	Jul	KZ	Paeds	Female	9	M
1453	2018	Jul	GA	Adults	Male	26	Y
1454	2018	Jul	GA	Adults	Female	28	Y
1455	2018	Jul	GA	Paeds	Male	9	D
1456	2018	Aug	WC	Adults	Female	56	Y
1457	2018	Aug	FS	Adults	Female	62	Y
1458	2018	Aug	WC	Adults	Male	55	Y
1459	2018	Aug	WC	Adults	Female	66	Y
1461	2018	Jul	GA	Paeds	Female	14	Y
1462	2018	Aug	GA	Adults	Male	54	Y
1464	2018	Aug	GA	Adults	Female	44	Y
1465	2018	Aug	GA	Adults	Female	74	Y
1466	2018	Jul	GA	Adults	Female	72	Y
1467	2018	Jul	WC	Paeds	Female	13	M
1469	2018	Apr	GA	Adults	Female	53	Y
1470	2018	Apr	GA	Paeds	Female	12	D
1471	2018	Apr	GA	Adults	Male	70	Y
1472	2018	May	KZ	Adults	Male	41	Y
1473	2018	May	GA	Paeds	Male	9	M
1474	2018	May	GA	Adults	Male	63	Y
1476	2018	May	GA	Adults	Female	67	Y
1484	2018	Jun	GA	Paeds	Male	1	M
1485	2018	Jun	GA	Adults	Male	63	Y
1486	2018	Jun	GA	Adults	Female	35	Y
1487	2018	Jun	GA	Paeds	Female	21	D
1488	2018	Jun	GA	Paeds	Female	7	D
1490	2018	Jun	GA	Paeds	Female	24	M
1493	2018	Aug	WC	Paeds	Female	25	M
1494	2018	Aug	GA	Adults	Male	57	Y
1496	2018	Aug	KZ	Paeds	Female	17	D
1497	2018	Aug	KZ	Paeds	Male	24	M
1498	2018	Aug	KZ	Paeds	Male	30	D
1499	2018	Aug	GA	Adults	Female	64	Y
1500	2018	Aug	GA	Adults	Male	35	Y
1501	2018	Jun	GA	Adults	Male	58	Y
1502	2018	May	GA	Unkown	Female	43	Y
1503	2018	Jul	GA	Paeds	Male	6	D
1504	2018	Jul	GA	Paeds	Female	4	D
1505	2018	May	GA	Adults	Male	34	Y
1506	2018	Jun	GA	Adults	Female	56	Y
1507	2018	Aug	GA	Adults	Male	80	Y
1508	2018	Aug	KZ	Paeds	Female	0	D
1509	2018	Aug	GA	Adults	Female	64	Y
1510	2018	Aug	GA	Adults	Female	59	Y
1511	2018	Aug	KZ	Paeds	Male	5	D
1512	2018	Aug	GA	Paeds	Female	40	Y
1513	2018	Aug	GA	Adults	Female	35	Y
1514	2018	Oct	GA	Paeds	Female	13	D
1515	2018	Jan	GA	Paeds	Female	12	M
1516	2018	Jan	KZ	Paeds	Unknown	4	M
1518	2018	Jan	GA	Paeds	Female	9	Y

1519	2018	Feb	GA	Paeds	Female	9	Y
1520	2018	Feb	KZ	Adults	Female	22	Y
1522	2018	Mar	GA	Unkown	Male	5	Y
1523	2018	Mar	GA	Paeds	Female	21	M
1524	2018	Apr	GA	Paeds	Female	4	M
1525	2018	Apr	GA	Adults	Female	64	Y
1526	2018	Apr	GA	Adults	Male	70	Y
1527	2018	Apr	GA	Adults	Male	30	Y
1528	2018	Apr	GA	Adults	Female	71	Y
1529	2018	Apr	GA	Adults	Male	58	Y
1530	2018	Apr	GA	Adults	Male	53	Y
1531	2018	Apr	GA	Adults	Female	26	Y
1532	2018	Apr	GA	Unkown	Male	48	Y
1533	2018	Apr	KZ	Paeds	Male	4	D
1534	2018	Apr	GA	Paeds	Female	5	M
1535	2018	Apr	KZ	Adults	Female	17	Y
1536	2018	Apr	GA	Paeds	Female	7	D
1537	2018	Apr	GA	Adults	Female	52	Y
1538	2018	Apr	GA	Paeds	Male	27	D
1539	2018	Apr	GA	Paeds	Male	14	Y
1540	2018	Apr	GA	Paeds	Male	6	M
1541	2018	Apr	KZ	Paeds	Male	5	Y
1542	2018	Apr	GA	Unkown	Female		
1543	2018	Apr	GA	Adults	Female	37	Y
1544	2018	Apr	GA	Unkown	Male	20	Y
1545	2018	Apr	GA	Adults	Female	43	Y
1546	2018	May	GA	Unkown	Female		
1547	2018	May	GA	Paeds	Female	2	M
1549	2018	May	GA	Unkown	Male	70	Y
1550	2018	May	GA	Adults	Male	29	Y
1551	2018	May	GA	Unkown	Female	43	Y
1552	2018	May	GA	Adults	Male	74	Y
1553	2018	May	GA	Paeds	Male	17	D
1554	2018	May	GA	Paeds	Female	1	M
1555	2018	May	GA	Adults	Male	60	Y
1556	2018	May	GA	Adults	Male	46	Y
1557	2018	Nov	GA	Paeds	Male	53	Y
1558	2018	May	KZ	Paeds	Female	15	M
1559	2018	May	GA	Adults	Male	63	Y
1560	2018	May	KZ	Adults	Male	63	Y
1561	2018	Jun	GA	Adults	Female	18	Y
1562	2018	Jun	GA	Paeds	Male	8	D
1563	2018	Jun	GA	Adults	Female	15	Y
1564	2018	Jun	GA	Paeds	Male	16	D
1565	2018	Jun	GA	Adults	Male	52	Y
1566	2018	Jun	GA	Paeds	Male	10	D
1567	2018	Jun	WC	Adults	Male	26	Y
1568	2018	Jun	GA	Adults	Female	42	Y
1569	2018	Jun	GA	Adults	Male	46	Y
1570	2018	Jun	GA	Unkown	Female		
1571	2018	Jun	GA	Unkown	Female	26	Y
1572	2018	Jun	GA	Unkown	Male		
1573	2018	Jun	GA	Unkown	Female	24	Y
1574	2018	Jun	GA	Adults	Male	27	Y
1575	2018	Jun	KZ	Adults	Female	24	Y
1576	2018	Jun	GA	Unkown	Male	14	D

1577	2018	Nov	GA	Paeds	Female	24	D
1578	2018	Jun	KZ	Paeds	Female	1	M
1579	2018	Jun	KZ	Paeds	Male	1	M
1580	2018	Jun	KZ	Paeds	Female	5	Y
1581	2018	Jun	GA	Adults	Female	52	Y
1582	2018	Jun	GA	Adults	Female	62	Y
1583	2018	Jun	KZ	Paeds	Male	17	D
1584	2018	Jun	KZ	Paeds	Female	18	D
1585	2018	Jun	KZ	Paeds	Female	24	D
1586	2018	Jun	GA	Unkown	Female		
1587	2018	Aug	GA	Adults	Male	26	Y
1590	2018	Sep	GA	Adults	Male	33	Y
1592	2018	Sep	GA	Adults	Male	41	Y
1593	2018	Sep	KZ	Paeds	Male	5	D
1595	2018	Sep	GA	Adults	Male	47	Y
1596	2018	Sep	WC	Adults	Male	71	Y
1597	2018	Sep	WC	Adults	Male	43	Y
1598	2018	Sep	WC	Adults	Female	51	Y
1599	2018	Oct	WC	Paeds	Male	30	M
1600	2018	Sep	GA	Adults	Female	36	Y
1601	2018	Sep	GA	Adults	Female	57	Y
1602	2018	Sep	GA	Adults	Male	69	Y
1603	2018	Sep	KZ	Paeds	Unknown	4	D
1604	2018	Oct	WC	Adults	Female	47	Y
1605	2018	Oct	FS	Adults	Male	38	Y
1606	2018	Oct	GA	Adults	Male	30	Y
1607	2018	Oct	GA	Paeds	Female	26	D
1608	2018	Oct	GA	Paeds	Male	4	D
1610	2018	Oct	GA	Adults	Female	57	Y
1611	2018	Oct	KZ	Adults	Female	47	Y
1612	2018	Oct	GA	Adults	Female	67	Y
1613	2018	Oct	KZ	Adults	Female	59	Y
1614	2018	Oct	KZ	Paeds	Male	22	D
1615	2018	Oct	GA	Adults	Female	64	Y
1616	2018	Oct	WC	Adults	Male	26	Y
1617	2018	Oct	GA	Adults	Male	23	Y
1618	2018	Oct	GA	Adults	Female	83	Y
1619	2018	Oct	GA	Adults	Female	56	Y
1620	2018	Oct	WC	Adults	Male	44	Y
1621	2018	Oct	GA	Adults	Male	43	Y
1622	2018	Oct	GA	Adults	Male	45	Y
1624	2018	Oct	GA	Adults	Male	35	M
1625	2018	Nov	GA	Adults	Male	63	Y
1626	2018	Nov	GA	Paeds	Female	8	M
1627	2018	Nov	GA	Adults	Female	63	Y
1628	2018	Sep	GA	Adults	Female	38	Y
1629	2018	Oct	GA	Paeds	Male	46	Y
1630	2018	Nov	GA	Adults	Male	66	Y
1631	2018	Nov	GA	Adults	Male	67	Y
1632	2018	Nov	GA	Adults	Male	60	Y
1633	2018	Nov	GA	Adults	Female	38	Y
1634	2018	Nov	GA	Adults	Male	35	Y
1635	2018	Nov	GA	Paeds	Male	9	D
1636	2018	Nov	GA	Paeds	Female	8	D
1637	2018	Nov	GA	Adults	Male	27	Y
1638	2018	Nov	WC	Adults	Male	22	Y

1639	2018	Nov	KZ	Paeds	Male	8	D
1640	2018	Nov	GA	Paeds	Male	2	M
1641	2018	Nov	WC	Adults	Male	64	Y
1642	2018	Nov	GA	Adults	Male	35	Y
1643	2018	Nov	GA	Adults	Female	55	Y
1644	2018	Nov	WC	Adults	Male	55	Y
1645	2018	Nov	GA	Paeds	Female	15	D
1646	2018	Nov	GA	Adults	Male	41	Y
1647	2018	Nov	GA	Adults	Male	37	Y
1649	2018	Nov	WC	Adults	Male	30	Y
1650	2018	Nov	WC	Paeds	Female	14	Y
1651	2018	Nov	GA	Adults	Male	32	Y
1652	2018	Nov	GA	Adults	Female	27	Y
1653	2018	Dec	KZ	Paeds	Female	4	D
1654	2018	Dec	WC	Paeds	Male	7	D
1656	2018	Dec	WC	Adults	Male	47	Y
1657	2018	Dec	GA	Adults	Male	81	Y
1658	2018	Dec	WC	Adults	Male	67	Y
1659	2018	Dec	GA	Adults	Male	62	Y
1660	2018	Dec	KZ	Paeds	Female	1	M
1664	2018	Jan	FS	Paeds	Male	46	M
1667	2018	Jan	KZ	Paeds	Male	10	Y
1670	2018	Jan	GA	Adults	Male	49	Y
1679	2018	Feb	GA	Paeds	Female	10	Y
1685	2018	Feb	GA	Adults	Male	49	Y
1705	2018	Apr	GA	Adults	Male	71	Y
1707	2018	Apr	GA	Adults	Female	30	Y
1712	2018	Apr	GA	Adults	Female	22	Y
1715	2018	Apr	GA	Paeds	Male	4	D
1718	2018	Apr	GA	Adults	Female	56	Y
1723	2018	Apr	GA	Adults	Female	43	Y
1730	2018	May	GA	Unkown	Male	58	Y
1734	2018	May	WC	Adults	Female	54	Y
1737	2018	May	GA	Unkown	Male		
1742	2018	May	FS	Paeds	Female	8	Y
1744	2018	May	GA	Paeds	Male	5	D
1748	2018	Jun	GA	Paeds	Male	20	D
1751	2018	Jun	KZ	Paeds	Female	1	M
1752	2018	Jun	GA	Paeds	Female	5	D
1757	2018	Jun	GA	Paeds	Male	23	D
1759	2018	Jun	GA	Adults	Male	80	Y
1762	2018	Jun	GA	Paeds	Male	15	D
1767	2018	Jun	GA	Paeds	Male	12	D
1769	2018	Jun	GA	Adults	Male	47	Y
1773	2018	Jul	GA	Adults	Male	40	Y
1777	2018	Jul	KZ	Adults	Female	24	Y
1781	2018	Jul	GA	Paeds	Male	7	M
1783	2018	Jul	GA	Adults	Male	62	Y
1787	2018	Jul	GA	Paeds	Female	21	D
1789	2018	Jul	GA	Adults	Female	26	Y
1791	2018	Jul	GA	Paeds	Male	6	D
1792	2018	Jul	GA	Adults	Male	20	Y
1793	2018	Jul	GA	Paeds	Female	1	M
1796	2018	Jul	GA	Paeds	Male	9	D
1797	2018	Jul	GA	Paeds	Female	12	D
1798	2018	Jul	GA	Adults	Male	40	Y

1799	2018	Jul	KZ	Paeds	Male	4	M
1800	2018	Jul	GA	Paeds	Male	15	Y
1801	2018	Jul	GA	Unkown	Male		
1802	2018	Jul	GA	Adults	Female	55	Y
1806	2018	Jul	GA	Unkown	Female	64	Y
1807	2018	Jul	KZ	Adults	Male	35	Y
1809	2018	Jul	KZ	Paeds	Female	27	M
1810	2018	Jul	GA	Adults	Male	42	Y
1812	2018	Aug	GA	Paeds	Female	4	D
1814	2018	Aug	KZ	Paeds	Male	1	M
1815	2018	Aug	GA	Paeds	Male	11	D
1817	2018	Aug	GA	Adults	Male	51	Y
1818	2018	Aug	GA	Paeds	Female	25	D
1819	2018	Aug	GA	Unkown	Male		
1821	2018	Aug	GA	Paeds	Female	28	D
1822	2018	Aug	KZ	Paeds	Female	16	D
1823	2018	Aug	GA	Paeds	Female	26	M
1824	2018	Aug	GA	Adults	Female	61	Y
1826	2018	Aug	GA	Adults	Male	37	Y
1829	2018	Aug	GA	Adults	Male	40	Y
1830	2018	Aug	GA	Adults	Male	54	Y
1831	2018	Aug	GA	Adults	Male	55	Y
1832	2018	Aug	KZ	Paeds	Male	2	M
1835	2018	Aug	GA	Adults	Female	84	Y
1836	2018	Aug	GA	Paeds	Male	12	Y
1837	2018	Aug	WC	Adults	Male	41	Y
1841	2018	Aug	GA	Paeds	Male	6	D
1843	2018	Aug	KZ	Paeds	Male	8	M
1844	2018	Aug	WC	Adults	Male	25	Y
1845	2018	Aug	GA	Adults	Male	46	Y
1846	2018	Aug	GA	Paeds	Male	5	M
1847	2018	Aug	KZ	Paeds	Male	54	M
1848	2018	Aug	GA	Paeds	Female	1	M
1850	2018	Aug	KZ	Paeds	Male	19	M
1851	2018	Aug	GA	Paeds	Male	4	M
1852	2018	Aug	GA	Adults	Female	21	Y
1856	2018	Aug	GA	Paeds	Female	4	M
1857	2018	Aug	GA	Paeds	Male	7	M
1858	2018	Aug	GA	Paeds	Male	4	M
1859	2018	Sep	GA	Paeds	Male	3	M
1860	2018	Sep	GA	Paeds	Male	27	M
1861	2018	Sep	GA	Unkown	Male	48	Y
1865	2018	Sep	GA	Adults	Female	22	Y
1867	2018	Sep	GA	Adults	Female	60	Y
1868	2018	Sep	GA	Paeds	Female	15	D
1871	2018	Sep	WC	Adults	Male	55	Y
1873	2018	Sep	GA	Adults	Female	67	Y
1874	2018	Sep	KZ	Adults	Male	36	Y
1875	2018	Sep	GA	Paeds	Male	26	D
1876	2018	Sep	GA	Paeds	Female	14	M
1877	2018	Sep	GA	Adults	Male	35	Y
1882	2018	Sep	GA	Adults	Male	37	Y
1884	2018	Sep	GA	Paeds	Male	1	M
1886	2018	Sep	GA	Paeds	Female	3	M
1888	2018	Sep	GA	Adults	Male	28	Y
1892	2018	Sep	GA	Unkown	Female	34	Y

1893	2018	Sep	KZ	Adults	Male	43	Y
1894	2018	Sep	GA	Paeds	Female	15	D
1895	2018	Sep	GA	Paeds	Female	46	M
1898	2018	Sep	KZ	Adults	Female	20	Y
1903	2018	Dec	WC	Adults	Male	25	Y
1904	2018	Dec	WC	Adults	Male	57	Y
1908	2018	Dec	KZ	Adults	Male	63	Y
1909	2018	Dec	GA	Adults	Male	61	Y
1911	2018	Dec	KZ	Paeds	Male	6	D
1912	2018	Dec	GA	Adults	Male	32	Y
1913	2018	Dec	WC	Paeds	Male	1	M
1914	2018	Dec	GA	Adults	Male	41	Y
1915	2018	Dec	GA	Adults	Male	24	Y
1916	2018	Dec	WC	Paeds	Male	10	D
1917	2018	Dec	WC	Paeds	Male	6	D
1918	2018	Dec	WC	Adults	Female	20	Y
1919	2018	Dec	WC	Adults	Female	88	Y
1920	2018	Nov	KZ	Unkown	Male	2	M
1921	2018	Dec	GA	Adults	Male	47	Y
1923	2018	Sep	GA	Paeds	Male	1	M
1924	2018	Jul	GA	Adults	Male	56	Y
1925	2018	Jul	WC	Adults	Male	34	Y
1926	2018	Jul	GA	Adults	Female	78	Y
1927	2018	Aug	GA	Adults	Male	26	Y
1928	2018	Aug	GA	Paeds	Male	6	Y
1929	2018	Aug	GA	Unkown	Female	19	D
1930	2018	Aug	GA	Adults	Female	28	Y
1931	2018	Sep	GA	Adults	Male	36	Y
1932	2018	Sep	GA	Unkown	Male	35	Y
1934	2018	Aug	GA	Unkown	Male	41	Y
1935	2018	Aug	GA	Adults	Male	57	Y
1936	2018	Sep	GA	Adults	Male	39	Y
1937	2018	Aug	GA	Adults	Female	32	Y
1938	2018	Aug	GA	Paeds	Male	3	M
1939	2018	Sep	GA	Paeds	Male	29	D
1946	2018	Nov	KZ	Unkown	Male	3	M
1948	2018	Dec	GA	Adults	Male	47	Y
1949	2018	Dec	GA	Adults	Female	66	Y
1950	2018	Dec	KZ	Adults	Female	34	Y
1951	2018	Dec	GA	Adults	Male	33	Y
1952	2018	Dec	GA	Adults	Female	66	Y
1953	2018	Dec	GA	Adults	Male	69	Y
1955	2018	Oct	GA	Paeds	Male	14	D
1960	2018	Dec	FS	Paeds	Male	2	M
1972	2018	Dec	KZ	Adults	Female	47	Y
1973	2018	Nov	KZ	Paeds	Female	5	D
2010	2018	Oct	GA	Paeds	Male	6	Y
2018	2018	Apr	GA	Unkown	Female		
2020	2018	May	FS	Adults	Female	34	Y
2021	2018	Aug	KZ	Paeds	Male	27	D
2026	2018	Oct	WC	Adults	Female	75	Y
2027	2018	Oct	GA	Unkown	Female	28	Y
2028	2018	Oct	GA	Paeds	Female	12	Y
2029	2018	Oct	GA	Adults	Male	67	Y
2030	2018	Nov	GA	Adults	Male	19	Y
2031	2018	Nov	KZ	Adults	Male	20	Y

2032	2018	Nov	KZ	Unkown	Female		
2033	2018	Nov	KZ	Adults	Male	55	Y
2034	2018	Nov	GA	Adults	Male	41	Y
2036	2018	Dec	GA	Unkown	Female	23	Y
2037	2018	Dec	GA	Adults	Male	21	Y
2038	2018	Dec	GA	Adults	Male	21	Y
2039	2018	Dec	GA	Adults	Male	61	Y
2041	2018	Dec	GA	Paeds	Male	46	M
2057	2018	Apr	GA	Adults	Male	37	Y
2059	2018	Oct	GA	Paeds	Female	21	D
2060	2018	Oct	GA	Adults	Female	42	Y
2061	2018	Oct	GA	Adults	Male	59	Y
2062	2018	Oct	GA	Adults	Female	46	Y
2063	2018	Oct	GA	Adults	Male	58	Y
2064	2018	Oct	GA	Adults	Male	41	Y
2065	2018	Oct	GA	Adults	Female	45	Y
2066	2018	Oct	GA	Adults	Male	64	Y
2067	2018	Oct	WC	Adults	Male	44	Y
2068	2018	Oct	GA	Paeds	Female	23	M
2069	2018	Oct	GA	Adults	Female	77	Y
2070	2018	Oct	GA	Paeds	Male	1	M
2071	2018	Oct	GA	Adults	Male	43	Y
2072	2018	Oct	GA	Adults	Male	61	Y
2073	2018	Oct	GA	Paeds	Male	5	Y
2074	2018	Oct	GA	Adults	Female	49	Y
2075	2018	Nov	GA	Paeds	Female	21	D
2076	2018	Nov	GA	Paeds	Male	9	Y
2077	2018	Nov	GA	Paeds	Female	7	D
2078	2018	Nov	GA	Paeds	Male	20	D
2079	2018	Nov	GA	Paeds	Female	8	M
2080	2018	Nov	GA	Paeds	Female	4	D
2081	2018	Nov	GA	Paeds	Male	13	D
2082	2018	Nov	GA	Paeds	Female	1	M
2083	2018	Nov	GA	Paeds	Male	25	D
2084	2018	Nov	GA	Adults	Female	52	Y
2085	2018	Nov	GA	Adults	Male	36	Y
2086	2018	Nov	GA	Paeds	Female	36	M
2087	2018	Nov	GA	Paeds	Male	16	M
2088	2018	Nov	GA	Paeds	Female	1	M
2089	2018	Nov	GA	Paeds	Male	5	D
2090	2018	Nov	GA	Paeds	Male	2	M
2091	2018	Nov	GA	Adults	Male	50	Y
2092	2018	Dec	GA	Paeds	Male	10	M
2093	2018	Dec	GA	Paeds	Male	7	D
2094	2018	Dec	GA	Paeds	Female	9	M
2095	2018	Dec	GA	Unkown	Male		
2096	2018	Dec	GA	Paeds	Female	10	D
2097	2018	Dec	GA	Adults	Female	68	Y
2098	2018	Dec	GA	Paeds	Male	5	M
2099	2018	Dec	GA	Paeds	Female	8	Y
2100	2018	Dec	GA	Adults	Male	22	Y
2101	2018	Dec	GA	Adults	Male	22	Y
2102	2018	Dec	GA	Paeds	Male	6	D
2103	2018	Dec	GA	Adults	Female	39	Y
2104	2018	Dec	GA	Adults	Male	63	Y
2105	2018	Dec	GA	Adults	Female	72	Y

2106	2018	Dec	WC	Adults	Female	39	Y
2107	2018	Dec	GA	Adults	Male	57	Y
2108	2018	Dec	GA	Adults	Male	19	Y
2291	2018	Apr	GA	Adults	Female	79	Y
2292	2018	May	GA	Paeds	Male	3	D
2293	2018	May	WC	Adults	Male	65	Y
2294	2018	Jul	KZ	Paeds	Unknown	9	M
2295	2018	Jul	WC	Adults	Male	19	Y
2296	2018	Sep	GA	Adults	Male	34	Y
2297	2018	Sep	GA	Adults	Male	47	Y
1339	2019	Jan	WC	Adults	Male	66	Y
1349	2019	Feb	GA	Paeds	Female	27	M
1356	2019	Feb	GA	Adults	Male	44	Y
1376	2019	Feb	GA	Paeds	Female	2	M
1424	2019	Feb	GA	Adults	Female	37	Y
1431	2019	Feb	GA	Paeds	Male	9	D
1460	2019	Jul	GA	Adults	Female	30	Y
1477	2019	Jul	KZ	Paeds	Female	11	D
1479	2019	Jul	WC	Adults	Male	30	Y
1480	2019	Jul	WC	Paeds	Male	2	M
1481	2019	Jul	WC	Adults	Male	31	Y
1482	2019	Jul	GA	Adults	Male	67	Y
1495	2019	Feb	GA	Paeds	Female	21	D
1521	2019	Feb	GA	Paeds	Male	1	M
1548	2019	Feb	WC	Paeds	Female	19	D
1589	2019	Jun	GA	Adults	Female	61	Y
1665	2019	Jul	GA	Unkown	Female		
1666	2019	Aug	WC	Adults	Male	56	Y
1668	2019	Aug	KZ	Adults	Female	16	Y
1671	2019	Aug	GA	Unkown	Male	52	Y
1673	2019	Aug	WC	Paeds	Male	6	D
1674	2019	Jul	GA	Unkown	Male		
1675	2019	Aug	WC	Paeds	Male	7	D
1676	2019	Aug	WC	Adults	Male	26	Y
1678	2019	Aug	WC	Paeds	Female	8	D
1686	2019	Sep	WC	Paeds	Female	12	M
1688	2019	Sep	KZ	Adults	Male	31	Y
1689	2019	Sep	WC	Adults	Male	27	Y
1692	2019	Sep	KZ	Adults	Female	0	D
1695	2019	Aug	KZ	Paeds	Female	14	D
1696	2019	Sep	KZ	Adults	Female	89	Y
1697	2019	Sep	GA	Adults	Male	71	Y
1698	2019	Sep	KZ	Paeds	Female	0	D
1700	2019	Sep	KZ	Adults	Female	56	Y
1701	2019	Sep	KZ	Adults	Male	42	Y
1706	2019	Sep	KZ	Paeds	Male	17	D
1709	2019	Sep	KZ	Paeds	Male	1	M
1710	2019	Sep	GA	Adults	Male	34	Y
1713	2019	Aug	GA	Paeds	Male	23	M
1714	2019	Sep	GA	Unkown	Male		
1716	2019	Sep	GA	Adults	Male	36	Y
1717	2019	Sep	GA	Unkown	Male	34	Y
1719	2019	Sep	GA	Adults	Female	49	Y
1720	2019	Sep	GA	Adults	Male	62	Y
1721	2019	Sep	GA	Adults	Female	57	Y
1722	2019	Sep	KZ	Paeds	Male	13	D

1724	2019	Sep	WC	Paeds	Male	46	M
1725	2019	Sep	WC	Adults	Female	57	Y
1728	2019	Sep	KZ	Adults	Female	28	Y
1731	2019	Sep	WC	Adults	Female	56	Y
1733	2019	Sep	WC	Adults	Female	43	Y
1738	2019	Sep	GA	Adults	Male	24	Y
1740	2019	Sep	WC	Adults	Female	43	Y
1745	2019	Sep	WC	Adults	Female	38	Y
1746	2019	Sep	GA	Adults	Female	39	Y
1758	2019	Feb	KZ	Adults	Female	58	Y
1761	2019	Sep	GA	Adults	Male	30	Y
1766	2019	Sep	GA	Adults	Female	55	Y
1770	2019	Sep	GA	Adults	Male	25	Y
1771	2019	Sep	KZ	Paeds	Male	9	D
1774	2019	Sep	GA	Paeds	Female	2	D
1775	2019	Sep	GA	Adults	Female	56	Y
1776	2019	Sep	GA	Unkown	Female	34	Y
1778	2019	Sep	GA	Adults	Male	37	Y
1780	2019	Sep	GA	Paeds	Male	3	M
1784	2019	Sep	GA	Adults	Male	26	Y
1786	2019	May	GA	Unkown	Male	72	Y
1790	2019	Sep	GA	Paeds	Female	4	M
1803	2019	Sep	GA	Adults	Female	51	Y
1808	2019	Sep	GA	Adults	Male	52	Y
1813	2019	Mar	GA	Adults	Female	43	Y
1816	2019	Sep	GA	Adults	Male	24	Y
1825	2019	Sep	GA	Paeds	Male	10	Y
1828	2019	Sep	KZ	Paeds	Male	7	D
1833	2019	Sep	KZ	Paeds	Female	14	Y
1834	2019	Oct	GA	Adults	Male	45	Y
1838	2019	Oct	GA	Adults	Female	35	Y
1840	2019	Oct	WC	Adults	Male	57	Y
1842	2019	Oct	WC	Adults	Female	39	Y
1849	2019	Oct	WC	Adults	Female	39	Y
1853	2019	Jan	WC	Adults	Female	64	Y
1862	2019	Oct	KZ	Paeds	Male	7	D
1864	2019	Oct	GA	Adults	Male	47	Y
1866	2019	Oct	GA	Adults	Female	43	Y
1870	2019	Oct	GA	Paeds	Male	1	M
1872	2019	Feb	WC	Adults	Male	35	Y
1878	2019	Oct	GA	Paeds	Female	13	D
1879	2019	Oct	GA	Adults	Female	32	Y
1881	2019	Oct	WC	Paeds	Female	19	M
1885	2019	Oct	GA	Adults	Female	65	Y
1887	2019	Oct	KZ	Adults	Female	51	Y
1890	2019	Oct	KZ	Paeds	Male	3	D
1900	2019	Oct	GA	Adults	Female	61	Y
1906	2019	Jan	KZ	Paeds	Male	9	D
1907	2019	Jan	GA	Paeds	Female	11	Y
1922	2019	Feb	WC	Adults	Female	58	Y
1933	2019	Feb	WC	Adults	Male	33	Y
1940	2019	Feb	GA	Paeds	Male	2	M
1941	2019	Feb	GA	Adults	Male	22	Y
1942	2019	Feb	GA	Paeds	Male	31	M
1943	2019	Feb	GA	Adults	Male	42	Y
1944	2019	Feb	GA	Adults	Female	21	Y

1945	2019	Apr	KZ	Paeds	Male	3	M
1947	2019	Jan	GA	Adults	Female	41	Y
1954	2019	Jan	GA	Adults	Female	36	Y
1956	2019	Jan	GA	Adults	Female	18	Y
1957	2019	Jan	GA	Adults	Male	28	Y
1961	2019	Jan	KZ	Paeds	Female	2	M
1962	2019	Apr	KZ	Paeds	Male	7	D
1963	2019	May	WC	Adults	Female	28	Y
1964	2019	Feb	KZ	Paeds	Female	3	M
1965	2019	Feb	KZ	Adults	Female	25	Y
1966	2019	Feb	KZ	Adults	Male	79	Y
1967	2019	Feb	WC	Adults	Male	46	Y
1968	2019	Feb	FS	Adults	Male	62	Y
1969	2019	Feb	KZ	Paeds	Male		
1970	2019	Jan	KZ	Paeds	Female	8	M
1971	2019	Jan	KZ	Adults	Female	60	Y
1974	2019	Jan	WC	Adults	Female	51	Y
1975	2019	Jan	KZ	Adults	Female	44	Y
1976	2019	Feb	WC	Adults	Male	49	Y
1977	2019	Feb	WC	Adults	Male	70	Y
1978	2019	Feb	KZ	Paeds	Female	31	M
1979	2019	Jan	KZ	Adults	Male	35	Y
1980	2019	Feb	WC	Adults	Male	67	Y
1981	2019	Jan	WC	Adults	Male	30	Y
1982	2019	Feb	WC	Adults	Male	41	Y
1983	2019	Jan	WC	Paeds	Female	10	Y
1984	2019	Feb	GA	Unkown	Male	11	M
1985	2019	Jan	KZ	Paeds	Female	11	D
1987	2019	Feb	WC	Adults	Female	50	Y
1988	2019	Jan	WC	Adults	Male	30	Y
1989	2019	Feb	WC	Adults	Male	30	Y
1990	2019	Feb	WC	Adults	Male	29	Y
1991	2019	Feb	GA	Paeds	Female	8	M
1992	2019	Feb	WC	Adults	Male	21	Y
1993	2019	Feb	GA	Adults	Male	61	Y
1994	2019	Feb	KZ	Adults	Male	47	Y
1995	2019	Feb	GA	Adults	Male	66	Y
1996	2019	Feb	WC	Adults	Female	23	Y
1997	2019	Feb	WC	Adults	Male	50	Y
1998	2019	Feb	KZ	Adults	Female	54	Y
1999	2019	Feb	WC	Adults	Female	52	Y
2000	2019	Feb	WC	Adults	Male	57	Y
2001	2019	Feb	WC	Adults	Female	60	Y
2002	2019	Feb	WC	Adults	Male	63	Y
2003	2019	Feb	KZ	Paeds	Male	10	D
2005	2019	Feb	WC	Adults	Male	49	Y
2006	2019	Feb	WC	Adults	Male	58	Y
2007	2019	Jan	GA	Adults	Female	64	Y
2008	2019	Feb	KZ	Paeds	Male	2	M
2009	2019	Mar	KZ	Paeds	Male	1	M
2011	2019	Mar	WC	Adults	Male	22	Y
2012	2019	Mar	WC	Adults	Male	34	Y
2013	2019	Mar	WC	Adults	Male	51	Y
2044	2019	Mar	WC	Adults	Male	30	Y
2045	2019	Mar	GA	Adults	Female	29	Y
2046	2019	Mar	KZ	Paeds	Male	21	D

2047	2019	Mar	KZ	Adults	Female	48	Y
2048	2019	Apr	FS	Adults	Male	69	Y
2050	2019	Mar	WC	Adults	Male	23	Y
2051	2019	Mar	WC	Adults	Male	34	Y
2052	2019	Mar	WC	Adults	Male	30	Y
2053	2019	Mar	GA	Adults	Female	21	Y
2054	2019	Mar	WC	Adults	Female	60	Y
2055	2019	Mar	WC	Adults	Male	58	Y
2056	2019	Mar	WC	Adults	Male	32	Y
2109	2019	Jun	GA	Adults	Female	52	Y
2110	2019	Jun	GA	Adults	Male	52	Y
2111	2019	Mar	GA	Adults	Female	29	Y
2112	2019	Mar	KZ	Adults	Female	28	Y
2114	2019	Mar	KZ	Adults	Female	67	Y
2115	2019	Apr	KZ	Adults	Male	75	Y
2116	2019	Apr	GA	Paeds	Female	17	M
2117	2019	Apr	WC	Adults	Female	23	Y
2118	2019	Apr	GA	Adults	Male	71	Y
2119	2019	Apr	WC	Adults	Female	66	Y
2120	2019	Mar	GA	Paeds	Male	6	Y
2122	2019	Apr	WC	Adults	Female	19	Y
2123	2019	Apr	WC	Adults	Male	47	Y
2124	2019	Apr	GA	Adults	Male	60	Y
2125	2019	Apr	GA	Adults	Female	29	Y
2126	2019	Apr	GA	Adults	Female	50	Y
2127	2019	Apr	WC	Paeds	Male	9	M
2128	2019	Apr	WC	Adults	Male	68	Y
2129	2019	Apr	KZ	Adults	Female	59	Y
2131	2019	Apr	WC	Adults	Male	58	Y
2132	2019	Apr	GA	Adults	Female	55	Y
2133	2019	Apr	GA	Adults	Female	29	Y
2134	2019	Apr	KZ	Paeds	Female	1	D
2135	2019	Jul	GA	Paeds	Male	26	M
2136	2019	May	KZ	Paeds	Male	1	M
2137	2019	Apr	WC	Adults	Male	57	Y
2138	2019	Apr	GA	Adults	Female	39	Y
2139	2019	May	WC	Adults	Female	21	Y
2140	2019	May	WC	Paeds	Female	19	M
2141	2019	May	GA	Paeds	Male	12	D
2142	2019	May	WC	Paeds	Male	7	D
2143	2019	May	GA	Adults	Male	29	Y
2144	2019	Apr	KZ	Paeds	Male	13	M
2145	2019	May	WC	Paeds	Male	37	M
2147	2019	May	GA	Paeds	Male	1	M
2148	2019	May	KZ	Paeds	Female	3	M
2149	2019	May	WC	Adults	Female	67	Y
2150	2019	May	WC	Paeds	Female	25	D
2151	2019	Apr	KZ	Paeds	Male	29	M
2152	2019	Mar	WC	Paeds	Female	19	D
2153	2019	May	KZ	Paeds	Male	12	D
2154	2019	Apr	KZ	Paeds	Female	27	D
2155	2019	Mar	KZ	Adults	Female	30	Y
2156	2019	May	KZ	Adults	Male	55	Y
2157	2019	May	KZ	Paeds	Male	21	D
2158	2019	May	KZ	Paeds	Female	2	M
2159	2019	Jan	GA	Paeds	Female	30	D

2160	2019	Jan	GA	Paeds	Female	1	M
2161	2019	Jan	KZ	Paeds	Male	3	M
2162	2019	Jan	GA	Paeds	Female	6	D
2163	2019	Jan	WC	Adults	Female	54	Y
2164	2019	Jan	GA	Adults	Female	25	Y
2165	2019	Jan	GA	Adults	Female	57	Y
2166	2019	Jan	GA	Adults	Male	15	Y
2167	2019	Jan	GA	Paeds	Male	14	Y
2168	2019	Jan	GA	Adults	Female	0	D
2169	2019	Jan	GA	Adults	Female	82	Y
2170	2019	Jan	KZ	Paeds	Male	2	D
2171	2019	Jan	GA	Adults	Male	81	Y
2172	2019	Jan	GA	Unkown	Female	0	D
2173	2019	Jan	GA	Paeds	Male	1	M
2174	2019	Jan	KZ	Paeds	Female	3	D
2175	2019	Jan	GA	Adults	Male	76	Y
2176	2019	Jan	GA	Unkown	Female		
2177	2019	Jan	GA	Adults	Male	68	Y
2178	2019	Jan	GA	Adults	Female	59	Y
2179	2019	Jan	GA	Adults	Female	30	Y
2180	2019	Jan	GA	Paeds	Female	9	D
2182	2019	Jan	GA	Adults	Male	47	Y
2183	2019	Jan	GA	Adults	Female	51	Y
2184	2019	Jan	WC	Paeds	Male	1	M
2185	2019	Jan	GA	Adults	Female	79	Y
2186	2019	Jan	GA	Adults	Female	39	Y
2187	2019	Jan	GA	Adults	Male	39	Y
2188	2019	Jan	GA	Paeds	Female	2	M
2189	2019	Jan	GA	Adults	Female	63	Y
2190	2019	Jan	GA	Adults	Female	48	Y
2191	2019	Jan	KZ	Paeds	Female	44	Y
2192	2019	Jan	WC	Adults	Female	36	Y
2193	2019	Jan	GA	Adults	Male	29	Y
2194	2019	Feb	GA	Adults	Male	61	Y
2195	2019	Feb	GA	Adults	Female	31	Y
2196	2019	Feb	GA	Paeds	Female	6	D
2197	2019	Feb	KZ	Adults	Male	32	Y
2198	2019	Feb	KZ	Paeds	Female	2	M
2199	2019	Feb	GA	Adults	Male	61	Y
2201	2019	Feb	GA	Adults	Male	49	Y
2202	2019	Feb	GA	Adults	Female	34	M
2203	2019	Feb	KZ	Adults	Female	24	Y
2204	2019	Feb	GA	Adults	Female	33	Y
2205	2019	Feb	GA	Paeds	Female	25	M
2206	2019	Feb	GA	Adults	Male	41	Y
2207	2019	Feb	GA	Paeds	Female	2	M
2208	2019	Feb	GA	Adults	Female	61	Y
2209	2019	Feb	GA	Adults	Male	59	Y
2210	2019	Feb	GA	Adults	Male	65	Y
2211	2019	Feb	GA	Adults	Male	90	Y
2212	2019	Feb	WC	Adults	Male	29	Y
2213	2019	Feb	GA	Adults	Female	63	Y
2214	2019	Feb	GA	Paeds	Male	9	Y
2215	2019	Feb	GA	Adults	Male	59	Y
2216	2019	Feb	FS	Adults	Male	57	Y
2217	2019	Feb	GA	Adults	Female	54	Y

2218	2019	Feb	GA	Unkown	Male	21	Y
2219	2019	Feb	GA	Adults	Male	39	Y
2220	2019	Feb	GA	Unkown	Female	36	Y
2221	2019	Feb	GA	Adults	Male	43	Y
2222	2019	Feb	GA	Adults	Female	40	Y
2223	2019	Feb	GA	Adults	Female	25	Y
2224	2019	Feb	GA	Unkown	Female	53	Y
2225	2019	Feb	GA	Adults	Female	61	Y
2226	2019	Feb	GA	Unkown	Female	59	Y
2227	2019	Feb	KZ	Paeds	Male	20	D
2228	2019	Feb	GA	Adults	Female	53	Y
2229	2019	Feb	GA	Adults	Male	32	Y
2230	2019	Feb	GA	Paeds	Male	3	D
2231	2019	Feb	KZ	Paeds	Female	3	D
2232	2019	Feb	GA	Adults	Female	57	Y
2233	2019	Feb	GA	Adults	Male	61	Y
2234	2019	Feb	KZ	Paeds	Male	2	M
2235	2019	Feb	GA	Paeds	Female	10	Y
2236	2019	Feb	KZ	Paeds	Female	18	D
2239	2019	Mar	GA	Paeds	Male	6	M
2240	2019	Mar	GA	Adults	Female	66	Y
2241	2019	Mar	GA	Adults	Male	64	Y
2242	2019	Mar	GA	Unkown	Female		
2243	2019	Mar	GA	Adults	Male	55	Y
2244	2019	Mar	GA	Adults	Male	29	Y
2245	2019	Mar	GA	Adults	Female	29	Y
2246	2019	Mar	GA	Adults	Male	19	Y
2247	2019	Mar	GA	Adults	Male	37	Y
2248	2019	Mar	KZ	Paeds	Male	3	D
2249	2019	Mar	GA	Adults	Male	27	Y
2250	2019	Mar	GA	Adults	Female	82	Y
2251	2019	Mar	GA	Paeds	Male	10	D
2252	2019	Mar	GA	Adults	Male	36	Y
2253	2019	Mar	GA	Paeds	Male	1	M
2254	2019	Mar	GA	Paeds	Male	6	D
2255	2019	Mar	GA	Unkown	Female	13	M
2256	2019	Mar	GA	Unkown	Male		
2257	2019	Mar	GA	Adults	Male	72	Y
2258	2019	Mar	GA	Adults	Female	39	Y
2259	2019	Mar	GA	Adults	Male	41	Y
2260	2019	Mar	GA	Paeds	Male	4	M
2261	2019	Mar	GA	Unkown	Male		
2262	2019	Mar	GA	Paeds	Female	4	M
2263	2019	Mar	KZ	Paeds	Female	10	D
2264	2019	Mar	WC	Adults	Male	65	Y
2265	2019	Mar	GA	Adults	Male	40	Y
2266	2019	Mar	GA	Adults	Male	55	Y
2267	2019	Mar	GA	Paeds	Male	7	D
2268	2019	Mar	GA	Adults	Female	51	Y
2269	2019	Jul	GA	Adults	Female	24	Y
2270	2019	Mar	WC	Adults	Female	36	Y
2271	2019	Mar	KZ	Adults	Male	34	Y
2273	2019	Mar	GA	Unkown	Female	40	Y
2274	2019	Mar	GA	Adults	Male	45	Y
2275	2019	Mar	GA	Adults	Female	40	Y
2276	2019	Mar	GA	Adults	Male	15	Y

2277	2019	Mar	GA	Adults	Male	35	Y
2278	2019	Mar	GA	Adults	Male	41	Y
2279	2019	Mar	GA	Unkown	Female		
2280	2019	Mar	GA	Adults	Female	45	Y
2281	2019	Mar	FS	Adults	Female	61	Y
2282	2019	Mar	GA	Adults	Male	23	Y
2283	2019	Mar	GA	Adults	Female	51	Y
2284	2019	Mar	GA	Paeds	Female	6	D
2285	2019	Mar	WC	Paeds	Male	9	D
2286	2019	Mar	GA	Adults	Male	56	Y
2287	2019	Mar	GA	Unkown	Female	40	Y
2288	2019	Mar	GA	Paeds	Female	9	Y
2289	2019	May	GA	Paeds	Male	52	M
2290	2019	Mar	GA	Paeds	Female	12	D
2298	2019	May	GA	Unkown	Male		
2299	2019	Apr	WC	Adults	Female	60	Y
2300	2019	Jun	WC	Paeds	Female	2	D
2301	2019	Jun	WC	Adults	Male	58	Y
2302	2019	Jun	GA	Adults	Female	46	Y
2304	2019	May	KZ	Paeds	Female	11	D
2305	2019	May	KZ	Adults	Male	70	Y
2306	2019	Jun	KZ	Paeds	Female	1	M
2307	2019	Jun	KZ	Adults	Male	63	Y
2308	2019	Jun	GA	Adults	Female	17	Y
2309	2019	Jun	GA	Adults	Male	56	Y
2310	2019	Jun	GA	Adults	Male	48	Y
2311	2019	Jun	FS	Adults	Male	20	Y
2313	2019	Jan	KZ	Adults	Female	42	Y
2314	2019	Jan	KZ	Paeds	Male	1	M
2316	2019	Jan	GA	Paeds	Male	1	M
2318	2019	Jan	GA	Adults	Female	80	Y
2319	2019	Jan	GA	Paeds	Female	1	D
2320	2019	Jan	WC	Adults	Female	31	Y
2321	2019	Jan	GA	Adults	Male	34	Y
2323	2019	Jan	KZ	Paeds	Female	2	M
2324	2019	Jan	GA	Adults	Female	41	Y
2325	2019	Jan	GA	Paeds	Male	4	D
2326	2019	Jan	KZ	Adults	Female	22	Y
2328	2019	Feb	GA	Paeds	Female	48	M
2329	2019	Feb	KZ	Adults	Female	18	Y
2330	2019	Feb	GA	Paeds	Female	1	D
2331	2019	Feb	GA	Paeds	Male	28	M
2332	2019	Feb	GA	Adults	Male	60	Y
2333	2019	Feb	GA	Adults	Male	60	Y
2334	2019	Feb	GA	Adults	Female	40	Y
2335	2019	Feb	KZ	Paeds	Female	18	M
2336	2019	Feb	GA	Paeds	Male	18	M
2337	2019	Feb	GA	Paeds	Female	3	M
2338	2019	Feb	GA	Adults	Male	18	Y
2340	2019	Feb	KZ	Paeds	Male	5	D
2341	2019	Feb	GA	Adults	Male	63	Y
2342	2019	Feb	WC	Adults	Male	57	Y
2343	2019	Mar	GA	Adults	Male	30	Y
2345	2019	Mar	GA	Adults	Male	50	Y
2346	2019	Mar	WC	Adults	Female	60	Y
2347	2019	Mar	GA	Adults	Male	22	Y

2348	2019	Mar	KZ	Adults	Female	35	Y
2349	2019	Mar	GA	Paeds	Female	1	M
2350	2019	Mar	GA	Adults	Female	68	Y
2351	2019	Mar	GA	Adults	Male	27	Y
2352	2019	Mar	GA	Adults	Female	33	Y
2355	2019	Mar	WC	Paeds	Male	3	D
2356	2019	Mar	KZ	Adults	Male	35	Y
2358	2019	Apr	GA	Paeds	Male	5	Y
2359	2019	Apr	GA	Adults	Female	26	Y
2360	2019	Apr	GA	Adults	Male	56	Y
2361	2019	Apr	GA	Paeds	Male	6	D
2362	2019	Apr	GA	Adults	Female	42	Y
2363	2019	Apr	GA	Adults	Male	24	Y
2364	2019	Apr	GA	Adults	Male	35	Y
2365	2019	Jul	FS	Adults	Female	25	Y
2366	2019	Apr	GA	Adults	Female	44	Y
2367	2019	Apr	GA	Adults	Male	69	Y
2368	2019	Apr	GA	Adults	Male	42	Y
2369	2019	Apr	GA	Adults	Female	63	Y
2370	2019	Apr	WC	Adults	Female	23	Y
2371	2019	Apr	GA	Adults	Female	45	Y
2372	2019	Apr	GA	Adults	Female	64	Y
2374	2019	Jul	KZ	Adults	Male	58	Y
2375	2019	Apr	GA	Adults	Male	33	Y
2376	2019	Apr	KZ	Paeds	Male	7	D
2377	2019	Apr	GA	Paeds	Male	3	D
2378	2019	May	GA	Adults	Male	40	Y
2379	2019	May	GA	Adults	Female	38	Y
2380	2019	May	GA	Adults	Female	36	Y
2381	2019	May	GA	Adults	Female	57	Y
2382	2019	May	GA	Paeds	Female	1	M
2383	2019	May	GA	Paeds	Female	7	D
2384	2019	May	GA	Adults	Female	55	Y
2385	2019	May	GA	Paeds	Female	9	Y
2386	2019	Jun	KZ	Paeds	Male	7	D
2387	2019	Jun	GA	Adults	Female	78	Y
2388	2019	Jun	GA	Adults	Female	53	Y
2389	2019	Jun	WC	Adults	Female	50	Y
2390	2019	Jul	WC	Adults	Male	53	Y
2391	2019	Jul	WC	Adults	Male	80	Y
2392	2019	Jul	WC	Paeds	Female	2	M
2393	2019	Jul	WC	Adults	Male	52	Y
2394	2019	Jul	KZ	Adults	Male	44	Y
2395	2019	Jul	KZ	Paeds	Female	1	M
2396	2019	Jun	GA	Adults	Female	56	Y
2397	2019	Jun	WC	Adults	Male	33	Y
2398	2019	Jun	GA	Adults	Female	36	Y
2400	2019	Jun	GA	Adults	Male	28	Y
2401	2019	Jul	KZ	Paeds	Female	16	D
2402	2019	Jun	KZ	Paeds	Female	1	M
2403	2019	Jul	KZ	Adults	Female	32	Y
2404	2019	Jun	GA	Adults	Male	35	Y
2405	2019	Jul	KZ	Paeds	Female	49	M
2406	2019	Jul	GA	Adults	Female	50	Y
2407	2019	Jun	GA	Adults	Male	35	Y
2408	2019	Jul	GA	Paeds	Female	19	D

2409	2019	Aug	KZ	Paeds	Female	1	M
2410	2019	Jul	GA	Adults	Male	46	Y
2412	2019	Jul	KZ	Paeds	Male	2	M
2414	2019	Jul	KZ	Paeds	Female	17	D
2415	2019	Jul	KZ	Paeds	Male	7	M
2416	2019	Jul	KZ	Paeds	Female	2	M
2417	2019	Jul	WC	Adults	Female	42	Y
2418	2019	Jul	GA	Paeds	Female	11	D
2419	2019	Jul	WC	Adults	Male	52	Y
2420	2019	Aug	KZ	Adults	Female	29	Y
2421	2019	Aug	WC	Adults	Male	44	Y
2422	2019	Jul	KZ	Adults	Female	23	Y
2423	2019	Jun	GA	Adults	Female	29	Y
2424	2019	Aug	GA	Adults	Male	41	Y
2425	2019	Jun	GA	Adults	Male	61	Y
2426	2019	Jun	KZ	Paeds	Female	2	M
2427	2019	Jul	WC	Adults	Female	38	Y
2428	2019	Aug	KZ	Paeds	Female	2	M
2429	2019	Jul	WC	Paeds	Male	10	D
2430	2019	Jul	KZ	Paeds	Male	8	D
2431	2019	Jul	WC	Adults	Male	59	Y
2432	2019	Jul	GA	Paeds	Female	35	M
2433	2019	Jul	KZ	Adults	Female	64	Y
2434	2019	Jul	WC	Adults	Female	50	Y
2436	2019	Aug	GA	Adults	Male	33	Y
2437	2019	Jul	KZ	Adults	Male	40	Y
2438	2019	Aug	GA	Adults	Female	19	Y
2439	2019	Jun	GA	Paeds	Male	32	M
2440	2019	Jul	FS	Adults	Male	39	Y
2441	2019	Aug	GA	Paeds	Male	14	Y
2442	2019	Aug	WC	Paeds	Male	7	M
2443	2019	Aug	KZ	Paeds	Female	3	M
2444	2019	Aug	WC	Paeds	Female	14	D
2445	2019	Aug	KZ	Paeds	Female	22	D
2446	2019	Aug	GA	Adults	Female	30	Y
2447	2019	Aug	WC	Adults	Male	24	Y
2448	2019	Aug	GA	Adults	Female	33	Y
2449	2019	Aug	GA	Adults	Male	32	Y
2450	2019	Aug	GA	Adults	Male	52	Y
2451	2019	Aug	GA	Adults	Female	50	Y
2452	2019	Aug	WC	Paeds	Male	22	M
2453	2019	Aug	KZ	Adults	Male	52	Y
2454	2019	Aug	KZ	Adults	Male	19	Y
2455	2019	Aug	WC	Adults	Female	48	Y
2456	2019	Aug	GA	Adults	Female	16	Y
2457	2019	Aug	GA	Adults	Female	86	Y
2458	2019	Aug	GA	Adults	Male	43	Y
2459	2019	Aug	WC	Paeds	Male	8	D
2460	2019	Aug	GA	Adults	Female	36	Y
2461	2019	Jul	GA	Adults	Male	62	Y
2462	2019	Jul	GA	Unkown	Female	47	Y
2463	2019	Jul	GA	Adults	Male	51	Y
2464	2019	Jul	GA	Adults	Male	22	Y
2466	2019	Aug	KZ	Unkown	Male	37	Y
2467	2019	Oct	GA	Unkown	Female	26	Y
2468	2019	Jan	GA	Paeds	Male	8	D

2469	2019	Jan	WC	Paeds	Female	9	Y
2471	2019	Oct	GA	Adults	Male	58	Y
2472	2019	Feb	GA	Paeds	Female	3	M
2473	2019	Feb	GA	Unkown	Male	53	Y
2474	2019	Feb	GA	Adults	Male	39	Y
2475	2019	Mar	GA	Unkown	Male	74	Y
2476	2019	Mar	WC	Adults	Female	31	Y
2477	2019	Mar	GA	Adults	Female	73	Y
2478	2019	Mar	GA	Adults	Male	59	Y
2479	2019	Mar	WC	Adults	Male	60	Y
2480	2019	Apr	GA	Adults	Female	31	Y
2481	2019	Apr	GA	Adults	Male	44	Y
2482	2019	Apr	KZ	Paeds	Female	7	M
2483	2019	Apr	GA	Paeds	Male	20	D
2484	2019	Apr	KZ	Adults	Male	46	Y
2487	2019	Apr	GA	Adults	Male	31	Y
2488	2019	Apr	GA	Paeds	Male	11	D
2489	2019	Apr	KZ	Adults	Male	41	Y
2490	2019	Apr	GA	Paeds	Female	6	D
2491	2019	Apr	GA	Adults	Female	48	Y
2492	2019	Apr	GA	Adults	Male	53	Y
2493	2019	Apr	GA	Adults	Male	32	Y
2494	2019	Apr	GA	Paeds	Female	1	M
2495	2019	Apr	KZ	Paeds	Male	1	M
2496	2019	Apr	GA	Unkown	Male		
2497	2019	Apr	GA	Adults	Female	46	Y
2498	2019	Apr	GA	Adults	Female	52	Y
2499	2019	Apr	GA	Unkown	Female	57	Y
2500	2019	Apr	WC	Paeds	Male	6	D
2501	2019	Apr	WC	Adults	Male	78	Y
2502	2019	Apr	GA	Adults	Female	39	Y
2503	2019	Apr	GA	Adults	Female	71	Y
2504	2019	Apr	GA	Paeds	Male	27	D
2505	2019	Apr	WC	Paeds	Male	1	M
2506	2019	Apr	GA	Unkown	Male	33	Y
2507	2019	Apr	GA	Paeds	Female	9	M
2508	2019	Oct	GA	Adults	Male	78	Y
2509	2019	Apr	GA	Paeds	Female	15	Y
2510	2019	Apr	WC	Adults	Male	20	Y
2512	2019	Apr	KZ	Paeds	Male	5	M
2513	2019	Apr	KZ	Paeds	Female	20	D
2514	2019	Apr	GA	Adults	Female	57	Y
2515	2019	Apr	GA	Adults	Male	83	Y
2516	2019	Apr	FS	Adults	Female	21	Y
2517	2019	Apr	GA	Adults	Female	71	Y
2518	2019	Apr	GA	Paeds	Male	9	Y
2519	2019	Apr	GA	Adults	Female	60	Y
2520	2019	Apr	GA	Unkown	Female		
2521	2019	Apr	GA	Adults	Male	30	Y
2522	2019	Apr	KZ	Paeds	Female	32	M
2523	2019	Apr	GA	Adults	Female	42	Y
2524	2019	Apr	GA	Adults	Male	46	Y
2525	2019	May	GA	Paeds	Female	13	Y
2526	2019	May	GA	Paeds	Female	3	M
2527	2019	May	GA	Paeds	Male	23	D
2528	2019	May	KZ	Paeds	Female	28	Y

2529	2019	May	GA	Adults	Male	36	Y
2530	2019	May	GA	Unkown	Male	27	Y
2531	2019	May	GA	Adults	Female	42	Y
2532	2019	May	GA	Unkown	Female	1	M
2533	2019	May	WC	Paeds	Female	7	D
2534	2019	May	GA	Adults	Female	31	Y
2535	2019	May	GA	Paeds	Female	13	D
2536	2019	Sep	GA	Adults	Male	65	Y
2537	2019	May	GA	Adults	Female	53	Y
2538	2019	Oct	GA	Adults	Male	45	Y
2539	2019	May	GA	Adults	Male	36	Y
2540	2019	May	GA	Paeds	Female	26	D
2541	2019	May	GA	Adults	Male	61	Y
2542	2019	May	GA	Paeds	Male	2	M
2543	2019	May	GA	Unkown	Female	71	Y
2544	2019	May	GA	Adults	Female	58	Y
2545	2019	May	GA	Paeds	Male	14	D
2546	2019	May	KZ	Adults	Male	30	Y
2547	2019	May	GA	Adults	Male	56	Y
2548	2019	Jun	GA	Adults	Female	42	Y
2549	2019	Jun	GA	Paeds	Male	14	M
2550	2019	Jun	GA	Adults	Female	51	Y
2551	2019	Jun	GA	Adults	Female	32	Y
2552	2019	Jun	FS	Adults	Male	44	Y
2553	2019	Jun	GA	Adults	Male	48	Y
2554	2019	Jun	KZ	Adults	Female	34	Y
2555	2019	Jun	GA	Adults	Male	34	Y
2556	2019	Jun	GA	Adults	Male	56	Y
2557	2019	Jun	GA	Paeds	Male	19	D
2558	2019	Jun	GA	Paeds	Male	3	M
2559	2019	Jun	GA	Paeds	Male	4	D
2560	2019	Jun	KZ	Adults	Male	37	Y
2561	2019	Jun	GA	Adults	Male	83	Y
2562	2019	Jun	GA	Adults	Male	20	Y
2563	2019	Jun	GA	Adults	Female	64	Y
2564	2019	Jun	GA	Adults	Male	40	Y
2565	2019	Jun	KZ	Paeds	Male	1	M
2566	2019	Jun	WC	Adults	Male	55	Y
2567	2019	Jun	GA	Adults	Male	64	Y
2568	2019	Jun	GA	Adults	Female	26	Y
2569	2019	Jun	GA	Adults	Male	45	Y
2570	2019	Oct	GA	Adults	Female	38	Y
2571	2019	Oct	GA	Adults	Female	31	Y
2572	2019	Oct	GA	Paeds	Female	6	Y
2573	2019	Sep	GA	Adults	Female	43	Y
2574	2019	Sep	GA	Adults	Male	55	Y
2575	2019	Oct	GA	Adults	Male	25	Y
2576	2019	Jan	WC	Adults	Female	52	Y
2577	2019	Feb	GA	Adults	Female	62	Y
2579	2019	Mar	KZ	Paeds	Female	4	M
2580	2019	Mar	KZ	Adults	Female	41	Y
2581	2019	Mar	WC	Adults	Female	38	Y
2582	2019	Apr	GA	Adults	Female	35	Y
2583	2019	Apr	GA	Adults	Male	42	Y
2584	2019	May	KZ	Adults	Male	79	Y
2585	2019	May	GA	Adults	Male	36	Y

2586	2019	May	GA	Adults	Female	58	Y
2587	2019	Jun	GA	Adults	Male	37	Y
2588	2019	Jun	WC	Adults	Male	51	Y
2589	2019	Nov	KZ	Adults	Female	41	Y
2590	2019	Apr	GA	Adults	Female	34	Y
2591	2019	Oct	KZ	Paeds	Female	1	M
2592	2019	Oct	KZ	Adults	Female	39	Y
2593	2019	Oct	GA	Adults	Female	59	Y
2594	2019	Oct	GA	Paeds	Female	12	Y
2595	2019	Sep	GA	Unkown	Male	74	Y
2596	2019	Oct	GA	Adults	Female	23	Y
2597	2019	Aug	KZ	Adults	Female	32	Y
2598	2019	Aug	GA	Adults	Female	30	Y
2599	2019	Oct	GA	Adults	Male	31	Y
2600	2019	Nov	GA	Paeds	Male	1	M
2601	2019	Nov	KZ	Adults	Male	49	Y
2602	2019	Nov	KZ	Paeds	Male	6	D
2603	2019	Nov	KZ	Adults	Female	39	Y
2604	2019	Nov	GA	Adults	Female	54	Y
2605	2019	Nov	GA	Adults	Male	31	Y
2606	2019	Nov	GA	Adults	Male	43	Y
2607	2019	Nov	WC	Adults	Male	53	Y
2608	2019	Nov	WC	Adults	Male	32	Y
2609	2019	Nov	GA	Adults	Female	35	Y
2610	2019	Nov	WC	Adults	Female	40	Y
2612	2019	Dec	GA	Adults	Male	45	Y
2613	2019	Nov	GA	Paeds	Female	4	M
2615	2019	Nov	GA	Adults	Male	47	Y
2616	2019	Nov	GA	Paeds	Male	6	Y
2617	2019	Nov	WC	Adults	Female	59	Y
2618	2019	Oct	WC	Adults	Female	22	Y
2619	2019	Oct	WC	Adults	Male	32	Y
2620	2019	Nov	WC	Paeds	Female	47	M
2621	2019	Nov	KZ	Paeds	Female	21	D
2622	2019	Nov	KZ	Adults	Male	38	Y
2623	2019	Nov	GA	Unkown	Female	58	Y
2627	2019	Nov	KZ	Paeds	Female	24	D
2628	2019	Nov	KZ	Paeds	Male	5	D
2629	2019	Nov	GA	Paeds	Male	2	M
2630	2019	Nov	GA	Adults	Female	41	Y
2631	2019	Nov	GA	Adults	Male	63	Y
2632	2019	Nov	KZ	Adults	Female	27	Y
2633	2019	Nov	KZ	Adults	Female	28	Y
2634	2019	Nov	WC	Adults	Female	50	Y
2635	2019	Nov	KZ	Adults	Female	46	Y
2636	2019	Nov	GA	Adults	Male	38	Y
2637	2019	Nov	GA	Adults	Male	76	Y
2638	2019	Nov	WC	Adults	Female	34	Y
2639	2019	Nov	GA	Adults	Female	42	Y
2640	2019	Nov	WC	Adults	Female	63	Y
2641	2019	Nov	GA	Paeds	Male	36	M
2642	2019	Nov	GA	Adults	Male	38	Y
2643	2019	Nov	GA	Adults	Female	24	Y
2644	2019	Nov	GA	Adults	Male	32	Y
2645	2019	Nov	KZ	Paeds	Male	5	D
2646	2019	Nov	KZ	Paeds	Male	19	D

2647	2019	Nov	GA	Unkown	Female	47	Y
2648	2019	Nov	KZ	Paeds	Female	2	M
2649	2019	Jul	KZ	Adults	Male	55	Y
2650	2019	Nov	WC	Adults	Female	63	Y
2651	2019	Nov	WC	Adults	Female	40	Y
2652	2019	Nov	GA	Adults	Male	48	Y
2653	2019	Jan	WC	Adults	Female	60	Y
2654	2019	Jan	GA	Unkown	Female		
2655	2019	Jan	KZ	Unkown	Male		
2656	2019	Jan	WC	Paeds	Male	3	D
2657	2019	Jan	GA	Adults	Female	54	Y
2658	2019	Jan	KZ	Adults	Female	43	Y
2660	2019	Jan	KZ	Adults	Male	44	Y
2661	2019	Feb	GA	Paeds	Female	34	M
2662	2019	Feb	KZ	Paeds	Female	14	M
2663	2019	Feb	GA	Adults	Male	46	Y
2664	2019	Feb	GA	Adults	Male	28	Y
2665	2019	Mar	GA	Adults	Male	46	Y
2667	2019	Mar	GA	Paeds	Male	1	M
2668	2019	Mar	GA	Unkown	Male		
2669	2019	Mar	GA	Adults	Female	29	Y
2670	2019	Mar	GA	Paeds	Male	8	D
2672	2019	Apr	GA	Adults	Male	24	Y
2673	2019	Apr	KZ	Adults	Male	24	Y
2674	2019	Apr	GA	Adults	Female	70	Y
2675	2019	Apr	WC	Adults	Male	38	Y
2676	2019	Apr	GA	Paeds	Male	13	Y
2677	2019	May	GA	Unkown	Male		
2678	2019	May	GA	Adults	Female	72	Y
2679	2019	May	GA	Adults	Female	56	Y
2680	2019	May	GA	Adults	Female	41	Y
2681	2019	May	GA	Adults	Female	39	Y
2682	2019	May	GA	Adults	Female	45	Y
2683	2019	Jul	GA	Paeds	Female	0	D
2684	2019	Jul	GA	Paeds	Male	4	D
2685	2019	Jul	GA	Paeds	Male	1	M
2686	2019	Jul	GA	Paeds	Female	13	D
2687	2019	Jul	KZ	Adults	Male	33	Y
2688	2019	Jul	GA	Paeds	Female	0	D
2689	2019	Jul	GA	Paeds	Male	0	D
2690	2019	Jul	GA	Paeds	Male	1	D
2691	2019	Jul	GA	Adults	Male	61	Y
2692	2019	Jul	GA	Paeds	Male	2	M
2693	2019	Jul	GA	Adults	Male	42	Y
2694	2019	Jul	KZ	Paeds	Female	1	M
2695	2019	Jul	GA	Adults	Female	78	Y
2696	2019	Jul	GA	Paeds	Female	21	D
2697	2019	Jul	GA	Unkown	Female	8	M
2698	2019	Jul	GA	Paeds	Male	10	M
2699	2019	Jul	GA	Adults	Female	68	Y
2700	2019	Jul	GA	Paeds	Male	13	Y
2701	2019	Jul	GA	Adults	Male	36	Y
2702	2019	Jul	KZ	Paeds	Male	8	D
2703	2019	Jul	GA	Paeds	Male	4	D
2704	2019	Jul	GA	Adults	Female	78	Y
2705	2019	Jul	GA	Adults	Male	38	Y

2706	2019	Jul	KZ	Adults	Male	36	Y
2707	2019	Jul	GA	Adults	Female	20	Y
2708	2019	Jul	GA	Adults	Female	64	Y
2709	2019	Jul	GA	Adults	Male	28	Y
2710	2019	Jul	KZ	Adults	Male	54	Y
2711	2019	Jul	GA	Adults	Male	59	Y
2712	2019	Aug	GA	Unkown	Female	23	Y
2713	2019	Aug	GA	Adults	Female	19	Y
2714	2019	Aug	GA	Paeds	Male	13	Y
2715	2019	Aug	GA	Adults	Male	50	Y
2716	2019	Aug	KZ	Paeds	Male	5	D
2717	2019	Aug	GA	Paeds	Male	36	M
2718	2019	Aug	GA	Adults	Male	51	Y
2719	2019	Aug	GA	Adults	Male	74	Y
2720	2019	Aug	GA	Adults	Female	50	Y
2721	2019	Aug	GA	Adults	Female	30	Y
2722	2019	Aug	GA	Paeds	Male	16	M
2723	2019	Aug	GA	Unkown	Male		
2724	2019	Aug	GA	Adults	Female	57	Y
2725	2019	Aug	GA	Adults	Male	67	Y
2726	2019	Aug	GA	Paeds	Male	1	D
2727	2019	Aug	WC	Adults	Female	37	Y
2728	2019	Aug	WC	Paeds	Male	5	D
2729	2019	Aug	GA	Adults	Male	21	Y
2730	2019	Aug	GA	Adults	Male	42	Y
2731	2019	Aug	GA	Unkown	Female	50	Y
2732	2019	Aug	GA	Adults	Male	35	Y
2733	2019	Aug	GA	Unkown	Male	11	Y
2734	2019	Aug	KZ	Paeds	Male	13	D
2735	2019	Aug	GA	Adults	Male	63	Y
2736	2019	Aug	GA	Unkown	Male		
2737	2019	Aug	GA	Paeds	Unknown	2	M
2738	2019	Aug	GA	Paeds	Female	23	D
2739	2019	Aug	GA	Adults	Female	38	Y
2740	2019	Aug	GA	Adults	Male	17	Y
2741	2019	Aug	GA	Paeds	Female	36	M
2742	2019	Aug	GA	Adults	Female	66	Y
2743	2019	Aug	GA	Adults	Male	48	Y
2744	2019	Aug	WC	Paeds	Female	18	M
2745	2019	Aug	GA	Adults	Female	73	Y
2746	2019	Aug	GA	Paeds	Male	1	M
2747	2019	Aug	GA	Adults	Female	24	Y
2748	2019	Aug	GA	Paeds	Female	22	Y
2749	2019	Aug	WC	Adults	Female	38	Y
2750	2019	Aug	GA	Adults	Female	61	Y
2751	2019	Aug	GA	Adults	Male	51	Y
2752	2019	Aug	GA	Paeds	Female	12	D
2753	2019	Aug	GA	Adults	Female	80	Y
2754	2019	Aug	GA	Adults	Male	44	Y
2755	2019	Sep	GA	Adults	Female	60	Y
2756	2019	Sep	GA	Adults	Male	49	Y
2757	2019	Sep	GA	Adults	Male	51	Y
2758	2019	Sep	GA	Adults	Female	40	Y
2759	2019	Sep	GA	Adults	Male	28	Y
2760	2019	Sep	WC	Adults	Female	31	Y
2761	2019	Sep	GA	Adults	Female	73	Y

2762	2019	Sep	GA	Paeds	Male	2	M
2763	2019	Sep	GA	Adults	Male	29	Y
2764	2019	Sep	KZ	Paeds	Male	1	M
2765	2019	Sep	GA	Adults	Male	32	Y
2766	2019	Sep	GA	Adults	Male	18	Y
2767	2019	Sep	GA	Adults	Male	53	Y
2768	2019	Sep	KZ	Adults	Male	60	Y
2769	2019	Sep	GA	Adults	Unknown	61	Y
2770	2019	Sep	GA	Adults	Female	77	Y
2771	2019	Sep	GA	Adults	Male	32	Y
2772	2019	Sep	GA	Adults	Male	38	Y
2773	2019	Sep	GA	Adults	Male	57	Y
2774	2019	Sep	WC	Adults	Male	57	Y
2775	2019	Sep	GA	Adults	Female	47	Y
2776	2019	Sep	GA	Adults	Female	55	Y
2777	2019	Sep	GA	Unkown	Female		
2778	2019	Sep	WC	Paeds	Male	8	D
2779	2019	Sep	GA	Paeds	Female	1	M
2780	2019	Sep	GA	Adults	Female	43	Y
2781	2019	Sep	GA	Adults	Male	43	Y
2782	2019	Sep	GA	Paeds	Female	7	Y
2783	2019	Sep	GA	Adults	Female	66	Y
2784	2019	Sep	GA	Adults	Male	35	Y
2785	2019	Sep	GA	Adults	Male	62	Y
2786	2019	Sep	GA	Paeds	Female	0	D
2787	2019	Sep	GA	Adults	Male	24	Y
2788	2019	Sep	GA	Adults	Female	33	Y
2789	2019	Sep	GA	Adults	Male	67	Y
2790	2019	Sep	GA	Paeds	Male	1	M
2791	2019	Sep	GA	Paeds	Female	14	Y
2792	2019	Nov	WC	Adults	Male	36	Y
2793	2019	Dec	WC	Adults	Male	28	Y
2794	2019	Nov	GA	Adults	Female	23	Y
2795	2019	Nov	WC	Adults	Male	36	Y
2796	2019	Nov	GA	Adults	Female	54	Y
2797	2019	Nov	GA	Unkown	Male		
2798	2019	Nov	KZ	Adults	Female	18	Y
2799	2019	Nov	KZ	Paeds	Male	9	Y
2801	2019	Aug	WC	Adults	Male	59	Y
2802	2019	Sep	KZ	Paeds	Female	2	M
2803	2019	Aug	GA	Adults	Male	52	Y
2804	2019	Jul	GA	Adults	Male	52	Y
2805	2019	Sep	GA	Adults	Male	57	Y
2806	2019	Jul	GA	Adults	Male	59	Y
2807	2019	Sep	GA	Adults	Female	45	Y
2808	2019	Jul	GA	Adults	Male	77	Y
2809	2019	Sep	GA	Adults	Female	53	Y
2810	2019	Dec	FS	Adults	Female	24	Y
2811	2019	Nov	KZ	Paeds	Male	2	D
2812	2019	Dec	KZ	Adults	Male	17	Y
2813	2019	Oct	KZ	Paeds	Female	7	D
2814	2019	Dec	KZ	Adults	Female	43	Y
2815	2019	Nov	GA	Adults	Female	52	Y
2816	2019	Nov	KZ	Paeds	Male	16	D
2817	2019	Nov	KZ	Adults	Female	33	Y
2818	2019	Nov	KZ	Adults	Male	31	Y

2819	2019	Nov	KZ	Adults	Male	21	Y
2820	2019	Nov	GA	Paeds	Male	29	M
2821	2019	Nov	GA	Unkown	Male		
2822	2019	Nov	GA	Unkown	Female	32	Y
2823	2019	Dec	GA	Adults	Female	30	Y
2824	2019	Dec	GA	Adults	Female	59	Y
2825	2019	Dec	GA	Adults	Male	53	Y
2826	2019	Dec	KZ	Adults	Male	32	Y
2827	2019	Dec	KZ	Paeds	Male	12	M
2828	2019	Dec	KZ	Paeds	Male	10	Y
2830	2019	Dec	WC	Adults	Female	66	Y
2831	2019	Dec	KZ	Adults	Male	23	Y
2832	2019	Dec	WC	Paeds	Female	4	M
2833	2019	Dec	WC	Adults	Male	38	Y
2834	2019	Dec	WC	Adults	Male	37	Y
2835	2019	Dec	WC	Paeds	Male	4	M
2836	2019	Dec	KZ	Adults	Female	89	Y
2837	2019	Dec	WC	Adults	Male	28	Y
2838	2019	Dec	KZ	Paeds	Male	14	D
2840	2019	Dec	KZ	Paeds	Male	3	D
2844	2019	Dec	WC	Adults	Male	34	Y
2849	2019	Dec	KZ	Paeds	Female	21	D
2850	2019	Nov	WC	Adults	Male	67	Y
2851	2019	Dec	GA	Adults	Male	71	Y
2853	2019	Dec	GA	Adults	Female	66	Y
2854	2019	Dec	GA	Paeds	Female	1	M
2855	2019	Dec	GA	Paeds	Female	3	M
2858	2019	Dec	GA	Adults	Male	22	Y
2859	2019	Dec	GA	Paeds	Male	2	M
2866	2019	Dec	GA	Adults	Male	18	Y
2867	2019	Dec	GA	Adults	Male	60	Y
2870	2019	Jun	KZ	Adults	Male	46	Y
2871	2019	Nov	GA	Adults	Male	57	Y
2872	2019	Dec	GA	Adults	Male	17	Y
2873	2019	Nov	FS	Adults	Female	64	Y
2874	2019	Nov	GA	Adults	Female	16	Y
2875	2019	Dec	GA	Adults	Male	16	Y
2876	2019	Nov	GA	Adults	Male	15	Y
2886	2019	Dec	KZ	Paeds	Female	20	D
2894	2019	Oct	KZ	Adults	Female	38	Y
2896	2019	Dec	KZ	Adults	Female	21	Y
2949	2019	Nov	GA	Unkown	Male	15	Y
2950	2019	Nov	GA	Adults	Male	56	Y
2951	2019	Nov	GA	Unkown	Male	47	Y
2953	2019	Nov	GA	Adults	Female	40	Y
2954	2019	Nov	GA	Unkown	Male		
2955	2019	Dec	GA	Paeds	Male	12	Y
2956	2019	Dec	GA	Unkown	Male	45	Y
2957	2019	Dec	GA	Paeds	Male	47	Y
2958	2019	Dec	GA	Paeds	Male	2	M
2979	2019	Nov	GA	Adults	Female	83	Y
2980	2019	Nov	GA	Adults	Male	56	Y
2981	2019	Nov	GA	Paeds	Female	6	D
2982	2019	Nov	GA	Adults	Male	48	Y
2987	2019	Nov	GA	Adults	Male	39	Y
3006	2019	Nov	GA	Paeds	Female	2	M

3007	2019	Nov	GA	Paeds	Male	1	M
3008	2019	Dec	GA	Paeds	Male	53	M
3009	2019	Dec	GA	Paeds	Male	7	Y
3094	2019	Nov	GA	Adults	Male	61	Y
3119	2019	Jan	WC	Adults	Unknown	55	Y
3120	2019	Apr	KZ	Paeds	Unknown	8	D
3121	2019	May	GA	Adults	Female	42	Y
3123	2019	Sep	GA	Adults	Male	84	Y
3124	2019	Oct	GA	Adults	Female	28	Y
3125	2019	Oct	GA	Adults	Male	34	Y
3127	2019	Oct	GA	Adults	Male	50	Y
3128	2019	Nov	GA	Unkown	Male	59	M
3129	2019	Nov	GA	Adults	Male	36	Y
3130	2019	Nov	GA	Paeds	Male	2	M
3131	2019	Nov	GA	Unkown	Female	47	Y
3132	2019	Nov	GA	Adults	Unknown	68	Y
3133	2019	Nov	GA	Adults	Unknown	37	Y
3134	2019	Dec	FS	Adults	Unknown	30	Y
3135	2019	Dec	GA	Paeds	Unknown	8	Y
3136	2019	Dec	GA	Adults	Unknown	67	Y
3137	2019	Dec	GA	Adults	Male	54	Y
3138	2019	Dec	GA	Adults	Male	35	Y
3139	2019	Dec	GA	Paeds	Male	2	M
3140	2019	Dec	GA	Paeds	Male	5	M
3217	2019	Aug	GA	Adults	Male	39	Y
3218	2019	Oct	WC	Adults	Male	54	Y
3219	2019	Oct	GA	Adults	Male	63	Y
3220	2019	Oct	GA	Adults	Male	21	Y
3221	2019	Oct	GA	Paeds	Male	2	M
3222	2019	Oct	GA	Paeds	Male	1	M
3223	2019	Oct	GA	Adults	Female	45	Y
3224	2019	Oct	KZ	Adults	Female	38	Y
3225	2019	Oct	GA	Adults	Male	59	Y
3226	2019	Oct	GA	Adults	Female	77	Y
3227	2019	Oct	GA	Adults	Male	23	Y
3228	2019	Oct	GA	Adults	Female	75	Y
3229	2019	Oct	KZ	Adults	Male	53	Y
3230	2019	Oct	KZ	Adults	Female	61	Y
3231	2019	Oct	GA	Adults	Male	37	Y
3232	2019	Oct	GA	Paeds	Male	15	M
3233	2019	Oct	GA	Adults	Male	35	Y
3234	2019	Oct	GA	Paeds	Female	29	D
3235	2019	Oct	GA	Adults	Male	29	Y
3236	2019	Oct	GA	Adults	Male	61	Y
3237	2019	Oct	GA	Paeds	Male	3	M
3238	2019	Oct	WC	Adults	Male	59	Y
3239	2019	Nov	GA	Adults	Female	17	Y
3240	2019	Nov	GA	Adults	Female	36	Y
3241	2019	Nov	GA	Adults	Female	60	Y
3242	2019	Nov	GA	Adults	Female	26	Y
3243	2019	Nov	KZ	Adults	Female	63	Y
3244	2019	Nov	GA	Adults	Male	36	Y
3245	2019	Nov	WC	Adults	Female	73	Y
3246	2019	Nov	GA	Adults	Female	34	Y
3247	2019	Nov	WC	Adults	Female	39	Y
3248	2019	Nov	GA	Adults	Male	59	Y

3249	2019	Dec	WC	Paeds	Female	5	Y
3250	2019	Dec	GA	Paeds	Female	12	Y
3251	2019	Dec	GA	Unkown	Male		
3252	2019	Dec	GA	Adults	Female	54	Y
3253	2019	Dec	KZ	Paeds	Female	10	Y
3254	2019	Dec	GA	Adults	Male	27	Y
3256	2019	Dec	GA	Adults	Male	70	Y
3257	2019	Dec	GA	Paeds	Male	2	M
3258	2019	Dec	GA	Adults	Female	39	Y
3259	2019	Dec	GA	Paeds	Female	9	D
3260	2019	Dec	GA	Adults	Female	64	Y
3261	2019	Dec	KZ	Adults	Male	24	Y
3366	2019	Dec	KZ	Adults	Male	30	Y
3377	2019	Nov	KZ	Paeds	Male	21	D
3379	2019	Oct	GA	Adults	Female	79	Y
3381	2019	Nov	GA	Adults	Female	46	Y
3386	2019	Nov	GA	Adults	Male	57	Y
3387	2019	Dec	GA	Adults	Female	29	Y
3397	2019	Feb	KZ	Adults	Male	23	Y
3399	2019	Apr	KZ	Adults	Male	62	Y
3400	2019	Jan	KZ	Paeds	Unknown	5	D
3402	2019	Apr	FS	Adults	Female	73	Y
3403	2019	Jul	KZ	Paeds	Female	2	M
3406	2019	Nov	WC	Adults	Female	32	Y
3407	2019	May	GA	Adults	Female	30	Y
3409	2019	Oct	KZ	Paeds	Unknown	10	D
3410	2019	Dec	GA	Adults	Male	47	Y
3431	2019	Jan	KZ	Adults	Male	28	Y
3432	2019	Feb	GA	Adults	Male	29	Y
3434	2019	Feb	GA	Adults	Male	61	Y
3435	2019	Mar	KZ	Paeds	Male	6	Y
3436	2019	Mar	WC	Paeds	Female	48	M
3438	2019	Mar	GA	Adults	Female	25	Y
3439	2019	Mar	GA	Adults	Female	30	Y
3441	2019	Apr	KZ	Adults	Female	32	Y
3442	2019	Apr	GA	Adults	Female	27	Y
3443	2019	Apr	GA	Unkown	Male		
3444	2019	Apr	KZ	Paeds	Female	18	D
3446	2019	May	GA	Paeds	Female	11	Y
3448	2019	Jun	GA	Paeds	Male	3	M
3449	2019	Jun	FS	Adults	Female	56	Y
3453	2019	Sep	FS	Adults	Female	69	Y
3454	2019	Oct	GA	Adults	Female	43	Y
3455	2019	Oct	KZ	Paeds	Male	15	Y
3456	2019	Oct	KZ	Adults	Female	68	Y
3457	2019	Oct	GA	Unkown	Male		
3458	2019	Oct	GA	Adults	Male	62	Y
3459	2019	Oct	GA	Paeds	Male	3	M
3460	2019	Oct	GA	Paeds	Male	17	D
3461	2019	Oct	GA	Adults	Male	50	Y
3462	2019	Oct	GA	Unkown	Male		
3464	2019	Oct	GA	Adults	Male	35	Y
3465	2019	Oct	GA	Adults	Male	50	Y
3466	2019	Oct	KZ	Adults	Male	78	Y
3467	2019	Oct	GA	Adults	Female	73	Y
3468	2019	Oct	WC	Adults	Male	41	Y

3469	2019	Oct	WC	Adults	Male	91	Y
3470	2019	Oct	KZ	Adults	Male	16	Y
3471	2019	Oct	GA	Adults	Female	58	Y
3472	2019	Oct	KZ	Paeds	Male	31	M
3473	2019	Oct	KZ	Paeds	Unknown	25	D
3474	2019	Nov	GA	Unkown	Female		
3475	2019	Nov	GA	Paeds	Male	1	M
3476	2019	Nov	GA	Adults	Female	66	Y
3477	2019	Nov	GA	Paeds	Unknown	2	M
3478	2019	Nov	GA	Adults	Female	17	Y
3479	2019	Nov	GA	Paeds	Male	36	M
3480	2019	Nov	GA	Unkown	Male		
3481	2019	Nov	KZ	Adults	Female	48	Y
3482	2019	Nov	GA	Adults	Female	20	Y
3483	2019	Nov	GA	Adults	Female	30	Y
3484	2019	Nov	GA	Adults	Female	31	Y
3485	2019	Nov	GA	Paeds	Male	22	M
3486	2019	Nov	KZ	Adults	Female	65	Y
3487	2019	Nov	GA	Adults	Female	53	Y
3488	2019	Nov	GA	Adults	Male	45	Y
3489	2019	Nov	WC	Paeds	Male	3	M
3490	2019	Nov	FS	Adults	Female	35	Y
3491	2019	Nov	WC	Adults	Female	38	Y
3492	2019	Nov	GA	Unkown	Male		
3493	2019	Nov	GA	Adults	Male	68	Y
3494	2019	Dec	GA	Paeds	Female	19	M
3495	2019	Dec	GA	Adults	Male	44	Y
3496	2019	Dec	GA	Adults	Male	73	Y
3497	2019	Dec	GA	Adults	Female	35	Y
3498	2019	Dec	GA	Paeds	Male	58	M
3499	2019	Dec	KZ	Adults	Female	72	Y
3500	2019	Dec	GA	Paeds	Female	31	M
3501	2019	Dec	GA	Adults	Male	47	Y
3502	2019	Dec	GA	Adults	Female	65	Y
3503	2019	Dec	GA	Adults	Male	67	Y
3504	2019	Dec	GA	Adults	Male	33	Y
3505	2019	Dec	GA	Adults	Male	49	Y
3506	2019	Dec	GA	Unkown	Male		
3507	2019	Dec	GA	Paeds	Male	3	M
3508	2019	Dec	GA	Adults	Female	17	Y
3509	2019	Dec	WC	Adults	Male	28	Y
3510	2019	Dec	KZ	Paeds	Male	3	M
3511	2019	Dec	KZ	Adults	Female	59	Y
3512	2019	Dec	GA	Adults	Female	28	Y
3513	2019	Dec	GA	Adults	Male	66	Y
3514	2019	Dec	GA	Adults	Female	35	Y
3515	2019	Dec	GA	Adults	Male	40	Y
3516	2019	Dec	GA	Unkown	Male		
3517	2019	Dec	GA	Paeds	Unknown	23	D
1711	2020	Oct	WC	Paeds	Female	5	Y
2200	2020	Oct	GA	Adults	Male	52	Y
2435	2020	Jul	WC	Adults	Male	41	Y
2465	2020	Jul	GA	Adults	Male	31	Y
2845	2020	Jan	KZ	Paeds	Male	8	M
2846	2020	Jan	WC	Adults	Female	18	Y
2847	2020	Jan	WC	Adults	Female	1	M

2848	2020	Jan	WC	Adults	Male	71	Y
2852	2020	Jan	GA	Adults	Female	26	Y
2856	2020	Jan	KZ	Adults	Male	34	Y
2857	2020	Jan	GA	Adults	Female	33	Y
2860	2020	Jan	KZ	Paeds	Male	1	Y
2861	2020	Jan	GA	Adults	Female	52	Y
2862	2020	Jan	GA	Paeds	Male	1	M
2864	2020	Jan	GA	Adults	Male	40	Y
2865	2020	Jan	WC	Adults	Female	39	Y
2868	2020	Jan	GA	Adults	Female	30	Y
2869	2020	Jan	GA	Adults	Female	38	Y
2877	2020	Jan	WC	Adults	Male	63	Y
2878	2020	Jan	KZ	Adults	Male	22	Y
2879	2020	Jan	KZ	Paeds	Male	13	D
2880	2020	Jan	KZ	Paeds	Female	14	D
2881	2020	Jan	KZ	Adults	Male	35	Y
2882	2020	Jan	KZ	Adults	Male	55	Y
2883	2020	Jan	WC	Adults	Female	39	Y
2884	2020	Jan	GA	Adults	Female	34	Y
2885	2020	Jan	GA	Adults	Female	64	Y
2887	2020	Jan	GA	Paeds	Female	6	Y
2888	2020	Jan	GA	Adults	Female	36	Y
2889	2020	Jan	GA	Adults	Female	61	Y
2890	2020	Jan	WC	Adults	Male	75	Y
2891	2020	Jan	GA	Paeds	Female	59	M
2892	2020	Jan	GA	Adults	Male	58	Y
2893	2020	Jan	GA	Paeds	Female	1	M
2897	2020	Jan	GA	Adults	Male	86	Y
2899	2020	Jan	GA	Adults	Male	58	Y
2900	2020	Feb	GA	Adults	Male	27	Y
2901	2020	Feb	GA	Adults	Male	16	Y
2902	2020	Feb	GA	Adults	Female	46	Y
2903	2020	Jan	WC	Adults	Female	30	Y
2904	2020	Jan	WC	Adults	Male	75	Y
2905	2020	Jan	KZ	Paeds	Female	17	D
2906	2020	Jan	KZ	Paeds	Male	5	D
2907	2020	Feb	KZ	Adults	Male	53	Y
2908	2020	Feb	KZ	Paeds	Male	10	D
2910	2020	Feb	GA	Adults	Female	34	Y
2911	2020	Feb	GA	Adults	Female	22	Y
2912	2020	Jan	KZ	Paeds	Male	9	D
2913	2020	Feb	KZ	Adults	Male	57	Y
2914	2020	Feb	WC	Adults	Female	35	Y
2915	2020	Feb	WC	Adults	Male	62	Y
2916	2020	Feb	WC	Adults	Female	32	Y
2918	2020	Jan	WC	Paeds	Male	4	D
2919	2020	Feb	WC	Adults	Male	68	Y
2920	2020	Feb	FS	Adults	Male	31	Y
2921	2020	Feb	GA	Adults	Female	35	Y
2922	2020	Jan	WC	Adults	Female	35	Y
2923	2020	Feb	WC	Adults	Male	51	Y
2924	2020	Feb	GA	Paeds	Female	5	M
2925	2020	Feb	GA	Adults	Male	60	Y
2927	2020	Jan	KZ	Adults	Female	27	Y
2928	2020	Jan	KZ	Paeds	Female	9	D
2930	2020	Feb	GA	Paeds	Male	37	M

2932	2020	Feb	GA	Paeds	Male	4	D
2933	2020	Feb	GA	Adults	Female	38	Y
2934	2020	Feb	GA	Adults	Male	35	Y
2935	2020	Feb	GA	Adults	Female	71	Y
2936	2020	Feb	KZ	Paeds	Male	1	M
2937	2020	Feb	GA	Adults	Female	39	Y
2938	2020	Feb	KZ	Paeds	Male	8	D
2939	2020	Feb	GA	Adults	Male	25	Y
2940	2020	Feb	GA	Adults	Male	30	Y
2941	2020	Feb	GA	Paeds	Male	29	D
2942	2020	Feb	GA	Unkown	Male		
2943	2020	Feb	WC	Adults	Male	32	Y
2944	2020	Feb	GA	Paeds	Male	58	M
2945	2020	Feb	GA	Adults	Male	35	Y
2946	2020	Sep	GA	Unkown	Female	2	M
2947	2020	Feb	WC	Adults	Male	69	Y
2948	2020	Sep	WC	Adults	Male	42	Y
2959	2020	Jan	GA	Adults	Female	40	Y
2960	2020	Jan	GA	Adults	Male	48	Y
2961	2020	Jan	GA	Paeds	Female	13	D
2962	2020	Jan	GA	Adults	Male	15	Y
2963	2020	Jan	GA	Adults	Female	45	Y
2965	2020	Jan	GA	Adults	Male	43	Y
2966	2020	Jan	GA	Paeds	Male	17	M
2967	2020	Jan	GA	Adults	Male	58	Y
2969	2020	Jan	GA	Adults	Male	58	Y
2970	2020	Feb	GA	Paeds	Female	14	Y
2971	2020	Feb	GA	Adults	Male	33	Y
2972	2020	Feb	GA	Adults	Male	28	Y
2973	2020	Feb	GA	Paeds	Male	14	M
2974	2020	Feb	KZ	Paeds	Female	15	M
2975	2020	Feb	GA	Adults	Female	35	Y
2977	2020	Jan	GA	Adults	Female	37	Y
2978	2020	Feb	GA	Adults	Male	63	Y
2983	2020	Jan	GA	Adults	Male	58	Y
2984	2020	Feb	KZ	Paeds	Male	1	M
2985	2020	Feb	GA	Paeds	Female	10	D
2988	2020	Jan	GA	Paeds	Female	20	D
2989	2020	Feb	GA	Paeds	Male	13	Y
2990	2020	Feb	GA	Adults	Female	52	Y
2991	2020	Feb	GA	Adults	Male	68	Y
2992	2020	Feb	GA	Adults	Female	31	Y
2993	2020	Feb	GA	Adults	Male	38	Y
2996	2020	Feb	KZ	Paeds	Male	10	Y
2997	2020	Jan	GA	Adults	Male	25	Y
2998	2020	Feb	GA	Paeds	Female	25	D
2999	2020	Jan	GA	Paeds	Male	6	Y
3000	2020	Mar	GA	Adults	Male	25	Y
3001	2020	Feb	GA	Paeds	Female	5	M
3002	2020	Feb	GA	Paeds	Female	16	D
3003	2020	Feb	GA	Unkown	Male		
3004	2020	Feb	GA	Adults	Female	86	Y
3010	2020	Jan	GA	Adults	Female	31	Y
3012	2020	Feb	GA	Adults	Female	67	Y
3014	2020	Mar	WC	Adults	Male	42	Y
3015	2020	Mar	GA	Adults	Female	55	Y

3016	2020	Mar	WC	Adults	Male	47	Y
3017	2020	Jan	GA	Adults	Male	28	Y
3019	2020	Mar	GA	Adults	Female	20	Y
3020	2020	Mar	GA	Adults	Male	28	Y
3021	2020	Mar	GA	Adults	Male	46	Y
3022	2020	Mar	GA	Adults	Female	49	Y
3023	2020	Mar	KZ	Paeds	Male	22	D
3024	2020	Mar	GA	Adults	Male	47	Y
3025	2020	Mar	GA	Adults	Male	47	Y
3026	2020	Mar	GA	Adults	Female	30	Y
3027	2020	Mar	GA	Adults	Male	47	Y
3028	2020	Mar	KZ	Paeds	Female	42	M
3029	2020	Mar	KZ	Paeds	Female	9	D
3030	2020	Mar	KZ	Paeds	Male	9	Y
3031	2020	Mar	GA	Unkown	Female	54	Y
3032	2020	Mar	WC	Adults	Male	58	Y
3033	2020	Mar	GA	Adults	Female	18	Y
3034	2020	Mar	GA	Adults	Male	31	Y
3035	2020	Mar	WC	Adults	Female	39	Y
3036	2020	Mar	GA	Adults	Male	22	Y
3037	2020	Feb	WC	Paeds	Male	36	M
3038	2020	Feb	WC	Adults	Male	27	Y
3039	2020	Mar	GA	Unkown	Male		
3040	2020	Mar	GA	Adults	Female	22	Y
3041	2020	Feb	WC	Paeds	Male	0	D
3042	2020	Feb	WC	Adults	Male	22	Y
3043	2020	Feb	GA	Adults	Female	65	Y
3044	2020	Apr	GA	Adults	Male	61	Y
3045	2020	Apr	GA	Adults	Female	58	Y
3046	2020	Apr	GA	Paeds	Female	11	D
3047	2020	Apr	KZ	Paeds	Female	7	D
3048	2020	Apr	GA	Adults	Male	59	Y
3049	2020	Mar	WC	Adults	Female	23	Y
3050	2020	Mar	GA	Paeds	Female	25	D
3051	2020	Mar	WC	Adults	Male	28	Y
3052	2020	Mar	GA	Adults	Male	39	Y
3053	2020	Apr	GA	Adults	Female	59	Y
3054	2020	Apr	GA	Adults	Female	38	Y
3055	2020	Apr	KZ	Paeds	Female	5	D
3056	2020	Mar	GA	Adults	Female	62	Y
3057	2020	Mar	GA	Paeds	Male	28	M
3058	2020	Apr	GA	Adults	Female	68	Y
3059	2020	Mar	GA	Adults	Male	27	Y
3060	2020	Feb	GA	Paeds	Male	1	M
3061	2020	Mar	WC	Adults	Male	33	Y
3062	2020	Apr	GA	Adults	Male	48	Y
3063	2020	Mar	GA	Adults	Female	65	Y
3065	2020	Apr	GA	Adults	Male	30	Y
3066	2020	Mar	WC	Paeds	Female	24	M
3067	2020	Apr	GA	Adults	Female	58	Y
3069	2020	Apr	GA	Adults	Female	40	Y
3070	2020	Mar	KZ	Paeds	Female	16	D
3071	2020	Mar	KZ	Adults	Male	32	Y
3072	2020	Mar	KZ	Paeds	Female	3	D
3073	2020	Apr	GA	Paeds	Female	21	D
3074	2020	Apr	FS	Adults	Male	12	Y

3075	2020	Mar	KZ	Paeds	Male	18	D
3076	2020	Mar	GA	Adults	Female	68	Y
3077	2020	Apr	GA	Paeds	Male	22	D
3078	2020	Apr	GA	Adults	Male	51	Y
3080	2020	Apr	GA	Paeds	Male	10	D
3081	2020	Apr	GA	Paeds	Male	2	M
3082	2020	Apr	GA	Unkown	Male	24	Y
3083	2020	Apr	WC	Adults	Male	57	Y
3084	2020	Apr	WC	Adults	Male	59	Y
3085	2020	Apr	GA	Paeds	Female	6	D
3086	2020	Apr	WC	Paeds	Female	4	D
3087	2020	Apr	GA	Adults	Female	46	Y
3088	2020	Apr	WC	Adults	Female	40	Y
3089	2020	Mar	FS	Adults	Female	25	Y
3090	2020	Apr	KZ	Paeds	Male	11	D
3091	2020	Mar	KZ	Paeds	Female	4	M
3092	2020	Mar	KZ	Paeds	Female	18	D
3093	2020	Apr	KZ	Paeds	Female	23	D
3095	2020	Mar	FS	Adults	Female	56	Y
3096	2020	Apr	GA	Adults	Male	53	Y
3097	2020	Apr	KZ	Paeds	Female	8	Y
3098	2020	Apr	KZ	Adults	Male	48	Y
3099	2020	Apr	KZ	Adults	Male	36	Y
3100	2020	Apr	GA	Adults	Female	41	Y
3101	2020	May	GA	Paeds	Female	23	D
3102	2020	Apr	GA	Adults	Female	43	Y
3103	2020	Apr	KZ	Paeds	Male	26	M
3105	2020	Apr	GA	Paeds	Male	1	M
3106	2020	Apr	GA	Paeds	Male	0	D
3107	2020	Apr	GA	Adults	Male	59	Y
3108	2020	May	KZ	Paeds	Male	1	M
3109	2020	May	KZ	Paeds	Male	9	Y
3110	2020	May	GA	Paeds	Female	3	M
3111	2020	May	GA	Adults	Male	62	Y
3112	2020	May	GA	Unkown	Male	29	Y
3113	2020	May	GA	Paeds	Male	35	M
3114	2020	May	FS	Adults	Male	57	Y
3115	2020	May	GA	Paeds	Male	3	D
3116	2020	May	GA	Paeds	Male	28	M
3117	2020	May	WC	Adults	Male	76	Y
3118	2020	May	WC	Adults	Female	76	Y
3126	2020	Oct	FS	Adults	Male	40	Y
3141	2020	Apr	GA	Adults	Female	55	Y
3142	2020	May	FS	Adults	Male	76	Y
3143	2020	May	GA	Adults	Male	66	Y
3144	2020	May	GA	Adults	Male	22	Y
3145	2020	May	GA	Paeds	Male	27	D
3146	2020	May	GA	Paeds	Female	17	D
3147	2020	Apr	GA	Paeds	Male	2	D
3148	2020	May	GA	Unkown	Male		
3149	2020	May	GA	Adults	Male	76	Y
3150	2020	May	GA	Paeds	Female	10	D
3151	2020	May	GA	Adults	Male	34	Y
3153	2020	May	KZ	Paeds	Female	11	D
3154	2020	May	KZ	Paeds	Female	6	D
3155	2020	May	KZ	Paeds	Female	12	D

3156	2020	May	GA	Adults	Female	83	Y
3158	2020	May	KZ	Paeds	Female	9	D
3159	2020	May	GA	Adults	Male	38	Y
3160	2020	May	GA	Unkown	Male	67	Y
3161	2020	May	FS	Adults	Male	61	Y
3162	2020	May	WC	Paeds	Female	26	M
3163	2020	May	WC	Adults	Male	43	Y
3165	2020	May	KZ	Paeds	Male	18	M
3166	2020	May	WC	Adults	Male	45	Y
3167	2020	May	FS	Adults	Male	69	Y
3168	2020	May	GA	Adults	Female	49	Y
3169	2020	Apr	GA	Adults	Female	78	Y
3170	2020	May	GA	Adults	Female	47	Y
3171	2020	Apr	GA	Paeds	Male	3	M
3172	2020	May	GA	Adults	Male	54	Y
3173	2020	May	GA	Paeds	Female	1	M
3174	2020	May	GA	Adults	Female	55	Y
3175	2020	May	WC	Adults	Female	39	Y
3176	2020	May	WC	Adults	Male	66	Y
3177	2020	May	GA	Adults	Female	16	Y
3178	2020	May	WC	Adults	Male	37	Y
3179	2020	May	GA	Adults	Female	66	Y
3180	2020	May	GA	Adults	Male	51	Y
3181	2020	May	KZ	Adults	Male	46	Y
3182	2020	May	GA	Adults	Male	39	Y
3183	2020	May	KZ	Adults	Male	56	Y
3184	2020	Jan	WC	Adults	Male	22	Y
3185	2020	May	KZ	Adults	Female	27	Y
3188	2020	May	KZ	Adults	Male	28	Y
3189	2020	May	KZ	Adults	Male	33	Y
3190	2020	May	KZ	Paeds	Female	21	D
3191	2020	May	KZ	Paeds	Female	2	M
3192	2020	May	KZ	Paeds	Male	5	D
3193	2020	May	KZ	Paeds	Female	18	D
3194	2020	Jun	GA	Paeds	Female	1	M
3195	2020	Jun	GA	Paeds	Male	11	D
3196	2020	Jun	GA	Paeds	Female	23	D
3197	2020	Jun	GA	Adults	Female	48	Y
3198	2020	Jun	WC	Adults	Female	58	Y
3199	2020	May	GA	Adults	Female	74	Y
3200	2020	May	GA	Adults	Female	43	Y
3201	2020	May	GA	Adults	Male	48	Y
3202	2020	Jun	GA	Adults	Male	44	Y
3203	2020	Jun	WC	Paeds	Male	7	D
3204	2020	Jun	WC	Adults	Female	27	Y
3205	2020	Jun	GA	Adults	Female	59	Y
3206	2020	Jun	GA	Paeds	Male	36	M
3207	2020	Jun	GA	Paeds	Male	2	M
3208	2020	Jun	GA	Adults	Female	48	Y
3209	2020	Jun	KZ	Adults	Female	29	Y
3212	2020	Jun	WC	Adults	Female	34	Y
3214	2020	Jun	GA	Paeds	Male	30	M
3215	2020	Jun	GA	Adults	Male	34	Y
3216	2020	Jun	GA	Paeds	Male	1	M
3262	2020	Jun	GA	Adults	Male	57	Y
3263	2020	Jun	GA	Adults	Male	30	Y

3264	2020	Jun	GA	Adults	Male	44	Y
3265	2020	Jun	GA	Adults	Female	33	Y
3266	2020	Jun	GA	Adults	Male	60	Y
3267	2020	Jun	GA	Paeds	Male	1	M
3268	2020	Jun	GA	Adults	Female	38	Y
3269	2020	Jun	GA	Adults	Female	28	Y
3270	2020	Jun	WC	Adults	Male	49	Y
3271	2020	Jun	FS	Adults	Male	26	Y
3272	2020	Jun	KZ	Paeds	Female	12	Y
3273	2020	Jun	KZ	Adults	Male	33	Y
3274	2020	May	KZ	Adults	Female	52	Y
3275	2020	May	KZ	Paeds	Male	16	M
3276	2020	Jun	KZ	Paeds	Male	9	Y
3277	2020	May	KZ	Paeds	Male	20	M
3278	2020	Jun	WC	Adults	Male	41	Y
3280	2020	Jun	WC	Adults	Male	57	Y
3281	2020	Jun	WC	Adults	Female	57	Y
3282	2020	Jun	WC	Adults	Female	38	Y
3283	2020	Jun	GA	Adults	Female	50	Y
3284	2020	Jun	GA	Adults	Male	43	Y
3286	2020	Jun	GA	Adults	Male	52	Y
3287	2020	Jun	WC	Adults	Male	46	Y
3288	2020	Jun	WC	Adults	Male	25	Y
3289	2020	Jun	GA	Adults	Female	39	Y
3290	2020	Jun	GA	Adults	Male	46	Y
3291	2020	Jun	GA	Adults	Male	28	Y
3292	2020	Jul	GA	Paeds	Male	4	D
3293	2020	Jun	GA	Paeds	Female	24	D
3294	2020	Jun	GA	Adults	Female	85	Y
3295	2020	Jul	GA	Adults	Female	54	Y
3296	2020	Jul	GA	Adults	Female	53	Y
3297	2020	Jul	GA	Adults	Female	42	Y
3298	2020	Jul	GA	Adults	Female	68	Y
3299	2020	Jul	GA	Adults	Female	74	Y
3301	2020	Jul	GA	Adults	Male		Y
3302	2020	Jul	GA	Unkown	Female		
3303	2020	Jul	GA	Paeds	Female	5	D
3304	2020	Jul	FS	Adults	Male	50	Y
3305	2020	Jul	GA	Adults	Female	31	Y
3306	2020	Jul	GA	Adults	Female	34	Y
3307	2020	Jul	GA	Adults	Male	39	Y
3308	2020	Jul	GA	Adults	Female	52	Y
3309	2020	Jul	GA	Adults	Female	16	Y
3310	2020	Jul	GA	Paeds	Male	42	M
3311	2020	Jul	GA	Adults	Male	54	Y
3312	2020	Jul	GA	Paeds	Male	2	M
3313	2020	Jul	GA	Paeds	Female	2	M
3314	2020	Jul	GA	Adults	Female	20	Y
3315	2020	Jul	GA	Adults	Male	46	Y
3316	2020	Jul	GA	Paeds	Male	42	M
3317	2020	Jul	GA	Paeds	Female	6	Y
3318	2020	Jul	GA	Adults	Female	39	Y
3319	2020	Jul	WC	Paeds	Male	4	D
3320	2020	Jul	GA	Adults	Female	21	Y
3321	2020	Jul	GA	Paeds	Female	47	M
3322	2020	Jul	GA	Paeds	Female	46	M

3323	2020	Jul	GA	Adults	Female	62	Y
3324	2020	Jul	GA	Adults	Female	36	Y
3325	2020	Jul	GA	Adults	Male	37	Y
3326	2020	Jul	GA	Adults	Male	82	Y
3327	2020	Jul	WC	Adults	Male	27	Y
3328	2020	Jul	GA	Adults	Male	40	Y
3329	2020	Aug	GA	Paeds	Male	8	M
3330	2020	Jul	GA	Paeds	Female	11	D
3331	2020	Jul	GA	Adults	Female	39	Y
3332	2020	Jul	WC	Adults	Male	37	Y
3333	2020	Jul	GA	Paeds	Female	8	D
3334	2020	Jul	GA	Paeds	Male	9	D
3335	2020	Jul	WC	Adults	Female	39	Y
3336	2020	Jul	KZ	Adults	Female	83	Y
3337	2020	Jul	WC	Paeds	Female	6	D
3338	2020	Jul	GA	Paeds	Female	9	Y
3339	2020	Jul	GA	Adults	Male	29	Y
3340	2020	Aug	GA	Adults	Male	75	Y
3341	2020	Jul	GA	Paeds	Female	15	D
3342	2020	Jul	GA	Adults	Male	44	Y
3343	2020	Aug	WC	Adults	Male	69	Y
3344	2020	Aug	GA	Adults	Female	61	Y
3345	2020	Aug	GA	Paeds	Female	8	Y
3346	2020	Aug	WC	Adults	Male	74	Y
3347	2020	Aug	GA	Adults	Female	64	Y
3348	2020	Aug	GA	Paeds	Male	27	D
3349	2020	Aug	GA	Adults	Male	80	Y
3350	2020	Aug	GA	Paeds	Male	5	Y
3351	2020	Aug	GA	Adults	Male	23	Y
3352	2020	Aug	GA	Adults	Male	72	Y
3353	2020	Aug	GA	Paeds	Male	10	D
3354	2020	Aug	GA	Adults	Female	36	Y
3355	2020	Aug	GA	Paeds	Female	8	D
3356	2020	Jul	KZ	Adults	Male	60	Y
3357	2020	Jul	KZ	Paeds	Male	1	M
3358	2020	Aug	GA	Adults	Male	33	Y
3359	2020	Aug	KZ	Adults	Female	52	Y
3360	2020	Aug	GA	Adults	Female	52	Y
3361	2020	Aug	GA	Adults	Male	53	Y
3362	2020	Aug	GA	Paeds	Female	7	D
3363	2020	Aug	GA	Adults	Male	54	Y
3364	2020	Aug	GA	Adults	Male	28	Y
3367	2020	Aug	GA	Unkown	Female	22	Y
3369	2020	Aug	KZ	Adults	Male	33	Y
3370	2020	Aug	KZ	Adults	Female	55	Y
3371	2020	Aug	GA	Paeds	Male	1	M
3372	2020	Aug	GA	Paeds	Male	7	M
3373	2020	Aug	GA	Adults	Female	56	Y
3375	2020	Aug	GA	Adults	Male	40	Y
3376	2020	Aug	GA	Paeds	Male	8	D
3378	2020	Aug	GA	Paeds	Male	24	D
3380	2020	Aug	GA	Adults	Male	62	Y
3382	2020	Aug	GA	Adults	Male	59	Y
3383	2020	Aug	GA	Adults	Female	50	Y
3384	2020	Aug	KZ	Paeds	Female	17	D
3385	2020	Aug	GA	Paeds	Male	1	M

3389	2020	Aug	GA	Adults	Male	48	Y
3390	2020	Aug	KZ	Paeds	Female	1	M
3391	2020	Aug	KZ	Paeds	Female	12	M
3392	2020	Aug	GA	Paeds	Female	58	M
3393	2020	Aug	GA	Adults	Male	26	Y
3394	2020	Aug	GA	Adults	Male	23	Y
3395	2020	Aug	GA	Paeds	Male	1	M
3398	2020	Aug	KZ	Paeds	Male	27	D
3404	2020	Apr	KZ	Paeds	Female	13	Y
3405	2020	May	GA	Adults	Female	39	Y
3408	2020	Oct	KZ	Paeds	Male	23	D
3411	2020	Feb	WC	Adults	Male	34	Y
3412	2020	Mar	GA	Paeds	Male	17	M
3413	2020	Oct	KZ	Adults	Male	42	Y
3414	2020	Jun	KZ	Adults	Male	38	Y
3416	2020	Aug	GA	Adults	Male	45	Y
3417	2020	Aug	GA	Adults	Male	18	Y
3419	2020	Aug	GA	Paeds	Male	5	D
3420	2020	Aug	GA	Paeds	Female	12	D
3421	2020	Sep	GA	Unkown	Female		
3423	2020	Sep	GA	Adults	Female	33	Y
3425	2020	Sep	GA	Paeds	Female	1	M
3426	2020	Aug	KZ	Paeds	Female	4	D
3427	2020	Aug	KZ	Adults	Female	39	Y
3428	2020	Sep	GA	Paeds	Female	11	M
3429	2020	Sep	KZ	Paeds	Male	3	D
3430	2020	Sep	GA	Adults	Female	69	Y
3437	2020	Oct	KZ	Paeds	Female	6	M
3518	2020	Jan	GA	Paeds	Female	6	M
3519	2020	Jan	GA	Adults	Female	35	Y
3520	2020	Jan	GA	Adults	Male	36	Y
3521	2020	Jan	GA	Paeds	Male	6	M
3522	2020	Jan	GA	Paeds	Male	6	M
3523	2020	Jan	GA	Unkown	Male	30	Y
3524	2020	Jan	GA	Unkown	Male		
3525	2020	Jan	KZ	Adults	Female	51	Y
3526	2020	Jan	GA	Adults	Female	50	Y
3527	2020	Jan	GA	Adults	Male	16	Y
3528	2020	Jan	GA	Adults	Male	16	Y
3529	2020	Jan	GA	Adults	Male	66	Y
3530	2020	Jan	GA	Paeds	Female	1	M
3531	2020	Jan	GA	Unkown	Female		
3532	2020	Jan	GA	Adults	Male	52	Y
3533	2020	Jan	GA	Adults	Male	34	Y
3534	2020	Jan	FS	Paeds	Female	1	M
3535	2020	Jan	WC	Adults	Female	60	Y
3536	2020	Jan	GA	Unkown	Unknown		
3537	2020	Jan	GA	Adults	Female	26	Y
3538	2020	Jan	WC	Adults	Male	47	Y
3539	2020	Jan	GA	Paeds	Female	31	M
3540	2020	Jan	KZ	Paeds	Male	14	M
3541	2020	Jan	GA	Adults	Male	71	Y
3542	2020	Jan	KZ	Unkown	Male	31	Y
3543	2020	Jan	GA	Adults	Female	31	Y
3544	2020	Jan	GA	Paeds	Female	24	M
3545	2020	Jan	GA	Adults	Male	48	Y

3546	2020	Jan	GA	Adults	Male	38	Y
3547	2020	Jan	GA	Adults	Female	25	Y
3548	2020	Jan	GA	Adults	Female	47	Y
3549	2020	Jan	KZ	Adults	Female	55	Y
3550	2020	Jan	GA	Paeds	Female	2	M
3551	2020	Jan	KZ	Adults	Male	38	Y
3552	2020	Jan	WC	Paeds	Male	12	D
3553	2020	Jan	GA	Adults	Male	56	Y
3554	2020	Jan	GA	Unkown	Female	17	Y
3555	2020	Jan	GA	Paeds	Female	13	Y
3556	2020	Jan	GA	Adults	Female	60	Y
3557	2020	Jan	GA	Paeds	Female	13	Y
3558	2020	Jan	GA	Adults	Male	74	Y
3559	2020	Jan	KZ	Adults	Male	69	Y
3560	2020	Jan	GA	Paeds	Female	1	D
3561	2020	Jan	GA	Paeds	Female	18	M
3562	2020	Feb	GA	Adults	Female	35	Y
3563	2020	Feb	GA	Paeds	Female	29	D
3564	2020	Feb	FS	Adults	Male	29	Y
3565	2020	Feb	FS	Adults	Female	49	Y
3566	2020	Feb	KZ	Adults	Female	51	Y
3567	2020	Feb	KZ	Adults	Male	41	Y
3568	2020	Feb	GA	Adults	Female	47	Y
3569	2020	Feb	GA	Adults	Female	81	Y
3570	2020	Feb	KZ	Adults	Male	26	Y
3571	2020	Feb	GA	Adults	Male	47	Y
3572	2020	Feb	GA	Paeds	Female	1	M
3573	2020	Feb	GA	Unkown	Male		
3574	2020	Feb	KZ	Adults	Male	32	Y
3575	2020	Feb	GA	Adults	Female	49	Y
3576	2020	Feb	KZ	Paeds	Female	6	D
3577	2020	Feb	GA	Unkown	Female		
3578	2020	Feb	KZ	Adults	Male	23	Y
3579	2020	Feb	GA	Paeds	Female	9	Y
3580	2020	Feb	KZ	Paeds	Female	16	D
3581	2020	Feb	GA	Adults	Male	69	Y
3582	2020	Feb	GA	Adults	Male	59	Y
3583	2020	Feb	GA	Adults	Male	42	Y
3585	2020	Feb	GA	Adults	Female	44	Y
3586	2020	Feb	GA	Adults	Female	46	Y
3587	2020	Feb	KZ	Adults	Female	39	Y
3588	2020	Feb	KZ	Paeds	Female		Y
3589	2020	Feb	GA	Adults	Male	31	Y
3590	2020	Mar	GA	Paeds	Male	28	D
3591	2020	Mar	KZ	Adults	Male	22	Y
3592	2020	Mar	GA	Adults	Female	30	Y
3593	2020	Mar	FS	Adults	Female	35	Y
3594	2020	Mar	GA	Adults	Female	27	Y
3595	2020	Mar	GA	Adults	Male	50	Y
3598	2020	Mar	GA	Paeds	Female	2	D
3599	2020	Mar	GA	Adults	Male	37	Y
3600	2020	Mar	GA	Adults	Female	66	Y
3601	2020	Mar	GA	Unkown	Male	49	Y
3602	2020	Mar	GA	Adults	Male	50	Y
3603	2020	Mar	GA	Paeds	Female	6	D
3604	2020	Mar	KZ	Adults	Male	41	Y

3605	2020	Mar	GA	Adults	Female	69	Y
3606	2020	Oct	GA	Adults	Male	72	Y
3607	2020	Mar	GA	Adults	Female	56	Y
3608	2020	Mar	GA	Unkown	Male	14	M
3609	2020	Mar	KZ	Paeds	Female	2	M
3610	2020	Mar	GA	Unkown	Female	22	M
3611	2020	Mar	GA	Paeds	Female	1	M
3612	2020	Mar	GA	Paeds	Female	2	M
3613	2020	Mar	GA	Adults	Male	29	Y
3614	2020	Mar	FS	Adults	Female	51	Y
3615	2020	Mar	KZ	Adults	Female	49	Y
3616	2020	Mar	WC	Adults	Female	59	Y
3617	2020	Mar	KZ	Adults	Female	17	Y
3618	2020	Mar	KZ	Paeds	Male	24	D
3619	2020	Mar	WC	Adults	Male	59	Y
3620	2020	Mar	GA	Unkown	Female		
3621	2020	Mar	GA	Paeds	Male	3	M
3622	2020	Mar	WC	Adults	Male	35	Y
3623	2020	Mar	KZ	Paeds	Male	21	D
3624	2020	Mar	GA	Paeds	Female	6	Y
3625	2020	Mar	GA	Adults	Male	72	Y
3627	2020	Mar	KZ	Adults	Male	27	Y
3628	2020	Mar	GA	Unkown	Female	31	Y
3629	2020	Mar	GA	Adults	Male	34	Y
3630	2020	Mar	GA	Adults	Male	50	Y
3631	2020	Mar	KZ	Adults	Female	41	Y
3632	2020	Mar	GA	Paeds	Male	26	D
3633	2020	Mar	GA	Paeds	Male	14	M
3634	2020	Apr	KZ	Adults	Female	20	Y
3636	2020	Apr	WC	Adults	Female	57	Y
3637	2020	Apr	KZ	Paeds	Male	5	Y
3638	2020	Apr	GA	Unkown	Female		
3639	2020	Apr	GA	Adults	Male	28	Y
3640	2020	Apr	GA	Adults	Female	67	Y
3641	2020	Apr	GA	Paeds	Female	7	D
3642	2020	Apr	GA	Adults	Female	70	Y
3643	2020	Apr	GA	Paeds	Male	14	D
3644	2020	Apr	KZ	Paeds	Female	3	D
3645	2020	Apr	GA	Paeds	Female	2	M
3646	2020	Apr	GA	Adults	Female	52	Y
3647	2020	Apr	KZ	Paeds	Female	7	D
3648	2020	Apr	GA	Adults	Female	22	Y
3649	2020	Apr	GA	Unkown	Male		
3650	2020	Apr	GA	Adults	Male	49	Y
3651	2020	Apr	GA	Paeds	Male	2	M
3652	2020	Apr	KZ	Adults	Female	29	Y
3653	2020	Apr	GA	Adults	Male	46	Y
3654	2020	Apr	GA	Paeds	Male	20	D
3655	2020	Apr	KZ	Paeds	Female	8	M
3656	2020	Apr	GA	Adults	Female	16	Y
3657	2020	Sep	GA	Adults	Male	25	Y
3658	2020	Apr	KZ	Adults	Female	22	Y
3659	2020	Apr	GA	Adults	Male	58	Y
3660	2020	Apr	GA	Adults	Female	59	Y
3661	2020	Apr	FS	Paeds	Male	15	D
3662	2020	Apr	GA	Adults	Male	40	Y

3663	2020	Apr	KZ	Adults	Female	69	Y
3664	2020	Apr	GA	Unkown	Male		
3665	2020	Apr	GA	Paeds	Male	23	D
3666	2020	Apr	GA	Paeds	Male	1	M
3667	2020	Apr	GA	Adults	Female	60	Y
3668	2020	Apr	WC	Adults	Male	69	Y
3669	2020	May	GA	Adults	Male	38	Y
3670	2020	May	KZ	Paeds	Female	2	M
3671	2020	May	KZ	Paeds	Female	4	M
3672	2020	May	GA	Paeds	Female	7	D
3673	2020	May	GA	Adults	Male	71	Y
3675	2020	May	KZ	Paeds	Male	1	M
3676	2020	May	KZ	Paeds	Female	8	M
3677	2020	May	KZ	Adults	Female	38	Y
3678	2020	May	GA	Adults	Male	55	Y
3679	2020	May	GA	Paeds	Male	2	M
3680	2020	May	FS	Paeds	Female	1	M
3681	2020	May	GA	Adults	Female	43	Y
3682	2020	May	KZ	Adults	Male	23	Y
3683	2020	May	KZ	Paeds	Male	1	D
3684	2020	May	GA	Unkown	Male		
3685	2020	May	KZ	Adults	Male	22	Y
3686	2020	May	GA	Paeds	Male	30	D
3687	2020	May	GA	Adults	Female	63	Y
3688	2020	May	KZ	Adults	Male	39	Y
3689	2020	May	WC	Adults	Female	73	Y
3690	2020	May	GA	Adults	Male	71	Y
3692	2020	May	KZ	Paeds	Male	13	D
3693	2020	May	GA	Adults	Male	49	Y
3694	2020	May	KZ	Unkown	Female	66	Y
3695	2020	May	GA	Adults	Female	24	Y
3696	2020	May	GA	Adults	Female	56	Y
3698	2020	May	GA	Adults	Female	73	Y
3699	2020	May	GA	Adults	Female	43	Y
3700	2020	May	WC	Adults	Male	44	Y
3701	2020	Jun	GA	Adults	Male	34	Y
3702	2020	Jun	GA	Paeds	Male	11	D
3703	2020	Jun	KZ	Adults	Female	40	Y
3704	2020	Jun	GA	Unkown	Male	45	Y
3705	2020	Jun	GA	Adults	Male	56	Y
3706	2020	Jun	WC	Adults	Male	77	Y
3707	2020	Jun	GA	Paeds	Male	6	M
3708	2020	Jun	KZ	Paeds	Male	6	D
3709	2020	Jun	GA	Unkown	Female	53	Y
3710	2020	Jun	GA	Paeds	Male	16	D
3711	2020	Jun	GA	Paeds	Male	9	D
3712	2020	Jun	WC	Adults	Male	51	Y
3713	2020	Jun	GA	Paeds	Female	1	D
3714	2020	Jun	GA	Adults	Female	60	Y
3715	2020	Jun	GA	Paeds	Male	14	D
3716	2020	Jun	KZ	Adults	Female		Y
3717	2020	Jun	GA	Adults	Male	52	Y
3718	2020	Aug	KZ	Paeds	Male	10	Y
3719	2020	Sep	GA	Paeds	Female	5	D
3720	2020	Sep	GA	Adults	Male	62	Y
3721	2020	Sep	GA	Adults	Male	55	Y

3722	2020	Sep	GA	Adults	Female	61	Y
3723	2020	Sep	GA	Adults	Male	64	Y
3724	2020	Sep	GA	Adults	Female	43	Y
3725	2020	Sep	GA	Paeds	Male	1	M
3726	2020	Sep	GA	Adults	Male	55	Y
3727	2020	Sep	GA	Unkown	Unknown		
3728	2020	Sep	WC	Adults	Female	56	Y
3729	2020	Sep	WC	Paeds	Male	3	D
3730	2020	Sep	GA	Adults	Male	44	Y
3731	2020	Sep	GA	Adults	Male	70	Y
3732	2020	Sep	GA	Adults	Female	75	Y
3733	2020	Sep	GA	Adults	Male	28	Y
3735	2020	Sep	GA	Paeds	Male	4	D
3736	2020	Sep	GA	Paeds	Male	2	D
3737	2020	Sep	GA	Paeds	Male	13	M
3738	2020	Aug	GA	Adults	Female	56	Y
3739	2020	Sep	GA	Adults	Male	43	Y
3740	2020	Sep	GA	Adults	Female	20	Y
3741	2020	Feb	KZ	Paeds	Male	17	M
3742	2020	May	KZ	Adults	Female	76	Y
3743	2020	May	KZ	Adults	Male	34	Y
3744	2020	Jun	KZ	Adults	Female	24	Y
3745	2020	May	GA	Adults	Male	69	Y
3746	2020	Mar	GA	Unkown	Female	55	Y
3747	2020	Apr	GA	Adults	Male	41	Y
3748	2020	Jun	GA	Adults	Female	36	Y
3749	2020	Jun	GA	Unkown	Female	33	Y
3750	2020	Apr	GA	Paeds	Female	7	D
3751	2020	May	GA	Paeds	Female	8	D
3753	2020	Jan	WC	Adults	Female	63	Y
3754	2020	Apr	GA	Paeds	Female	1	M
3755	2020	Sep	GA	Unkown	Female	49	Y
3756	2020	Jan	GA	Adults	Female	55	Y
3757	2020	Mar	GA	Adults	Male	33	Y
3758	2020	Jan	GA	Paeds	Male	21	M
3759	2020	Sep	WC	Paeds	Male	1	D
3760	2020	Sep	WC	Adults	Male	42	Y
3761	2020	Sep	GA	Unkown	Female	12	Y
3762	2020	Sep	GA	Unkown	Unknown		
3763	2020	Sep	GA	Paeds	Male	1	M
3764	2020	Oct	GA	Paeds	Male	4	M
3765	2020	Oct	GA	Unkown	Male	8	M
3766	2020	Oct	GA	Unkown	Male	70	Y
3767	2020	Oct	GA	Paeds	Female	0	D
3768	2020	Sep	GA	Adults	Male	38	Y
3769	2020	Sep	WC	Adults	Female	44	Y
3770	2020	Sep	GA	Paeds	Female	2	M
3771	2020	Sep	GA	Adults	Male	32	Y
3772	2020	Sep	GA	Paeds	Male	5	M
3773	2020	Sep	GA	Adults	Male	64	Y
3774	2020	Sep	GA	Paeds	Male	11	D
3775	2020	Sep	GA	Paeds	Female	0	D
3776	2020	Sep	GA	Paeds	Female	17	M
3777	2020	Oct	GA	Paeds	Female	9	D
3778	2020	Oct	GA	Adults	Female	28	Y
3779	2020	Oct	GA	Paeds	Female	28	M

3780	2020	Sep	GA	Paeds	Male	1	M
3781	2020	Sep	GA	Adults	Male	50	Y
3782	2020	Oct	GA	Paeds	Male	3	M
3783	2020	Sep	KZ	Adults	Male	58	Y
3784	2020	Sep	KZ	Paeds	Male	11	D
3785	2020	Sep	KZ	Paeds	Male	1	M
3786	2020	Oct	GA	Paeds	Female	23	D
3787	2020	Sep	GA	Unkown	Male	3	M
3788	2020	Oct	GA	Paeds	Male	1	D
3789	2020	Sep	GA	Paeds	Female	11	D
3790	2020	Oct	GA	Adults	Female	33	Y
3791	2020	Oct	GA	Adults	Male	65	Y
3792	2020	Oct	GA	Paeds	Male	9	Y
3793	2020	Oct	GA	Adults	Female	50	Y
3794	2020	Oct	GA	Paeds	Female	0	D
3795	2020	Oct	GA	Paeds	Female	8	D
3796	2020	Oct	GA	Adults	Male	76	Y
3797	2020	Oct	GA	Adults	Female	33	Y
3798	2020	Oct	GA	Paeds	Male	17	M
3799	2020	Oct	GA	Adults	Male	40	Y
3800	2020	Oct	WC	Adults	Female	44	Y
3801	2020	Oct	KZ	Adults	Female	66	Y
3802	2020	Oct	KZ	Adults	Female	67	Y
3803	2020	Oct	KZ	Paeds	Male	1	M
3804	2020	Oct	GA	Adults	Female	36	Y
3805	2020	Oct	GA	Adults	Male	73	Y
3806	2020	Oct	GA	Adults	Male	76	Y
3807	2020	Oct	GA	Adults	Female	35	Y
3808	2020	Oct	WC	Adults	Female	67	Y
3809	2020	Oct	GA	Adults	Male	49	Y
3810	2020	Oct	GA	Paeds	Male	7	D
3811	2020	Oct	GA	Paeds	Male	13	M
3812	2020	Oct	GA	Paeds	Male	9	D
3813	2020	Oct	GA	Paeds	Female	15	D
3815	2020	Oct	GA	Adults	Male	60	Y
3816	2020	Oct	GA	Paeds	Female	12	Y
3817	2020	Nov	KZ	Paeds	Male	4	D
3818	2020	Oct	GA	Adults	Male	63	Y
3819	2020	Oct	GA	Paeds	Female	6	D
3820	2020	Oct	GA	Paeds	Male	8	D
3821	2020	Oct	GA	Paeds	Male	8	D
3822	2020	Oct	WC	Adults	Male	27	Y
3823	2020	Oct	WC	Adults	Male	51	Y
3824	2020	Oct	WC	Adults	Female	58	Y
3825	2020	Oct	WC	Adults	Male	32	Y
3826	2020	Oct	WC	Adults	Male	43	Y
3827	2020	Oct	GA	Paeds	Male	14	D
3828	2020	Oct	GA	Adults	Female	32	Y
3829	2020	Oct	GA	Unkown	Male	14	D
3830	2020	Oct	WC	Adults	Female	55	Y
3831	2020	Oct	GA	Paeds	Male	3	D
3832	2020	Oct	GA	Adults	Female	34	Y
3833	2020	Oct	GA	Adults	Male	40	Y
3834	2020	Oct	GA	Adults	Male	45	Y
3835	2020	Oct	GA	Adults	Female	53	Y
3836	2020	Oct	GA	Adults	Female	22	Y

3837	2020	Oct	GA	Adults	Male	62	Y
3838	2020	Oct	GA	Adults	Male	37	Y
3840	2020	Nov	KZ	Paeds	Male	48	M
3841	2020	Oct	KZ	Adults	Male	23	Y
3842	2020	Nov	WC	Adults	Male	27	Y
3843	2020	Oct	KZ	Paeds	Female	1	M
3844	2020	Oct	KZ	Paeds	Male	1	M
3845	2020	Oct	WC	Adults	Female	17	Y
3846	2020	Oct	GA	Adults	Male	50	Y
3847	2020	Oct	GA	Adults	Female	24	Y
3848	2020	Nov	GA	Adults	Male	41	Y
3849	2020	Oct	GA	Paeds	Male	4	M
3850	2020	Oct	GA	Paeds	Female	2	M
3851	2020	Oct	GA	Adults	Female	42	Y
3852	2020	Oct	KZ	Adults	Male	31	Y
3853	2020	Oct	KZ	Adults	Female	24	Y
3854	2020	Oct	KZ	Adults	Female	47	Y
3855	2020	Oct	KZ	Paeds	Male	1	M
3856	2020	Nov	GA	Adults	Male	55	Y
3857	2020	Nov	GA	Paeds	Male	1	Y
3858	2020	Nov	GA	Unkown	Male	26	Y
3859	2020	Nov	GA	Adults	Male	17	Y
3860	2020	Nov	KZ	Paeds	Female	28	D
3861	2020	Oct	KZ	Paeds	Female	2	M
3862	2020	Nov	GA	Adults	Male	59	Y
3863	2020	Nov	GA	Paeds	Female	12	D
3864	2020	Nov	GA	Paeds	Female	4	D
3865	2020	Nov	WC	Adults	Male	40	Y
3866	2020	Nov	GA	Adults	Female	31	Y
3867	2020	Nov	GA	Paeds	Female	29	M
3868	2020	Nov	GA	Adults	Female	41	Y
3869	2020	Nov	KZ	Paeds	Male	5	M
3872	2020	Nov	KZ	Paeds	Male	3	M
3873	2020	Nov	WC	Adults	Male	51	Y
3874	2020	Nov	WC	Paeds	Female	19	D
3875	2020	Nov	GA	Unkown	Male	9	Y
3876	2020	Nov	GA	Unkown	Male	60	
3877	2020	Nov	GA	Paeds	Male	9	M
3878	2020	Nov	GA	Adults	Male	55	Y
3879	2020	Nov	GA	Adults	Male	59	Y
3880	2020	Nov	GA	Unkown	Female	32	
3881	2020	Nov	GA	Unkown	Male	43	
3882	2020	Nov	GA	Paeds	Female	10	D
3883	2020	Nov	WC	Adults	Female	51	Y
3884	2020	Nov	GA	Adults	Male	78	Y
3885	2020	Nov	GA	Adults	Female	61	Y
3886	2020	Nov	KZ	Unkown	Female	1	M
3887	2020	Nov	GA	Paeds	Male	6	D
3889	2020	Dec	GA	Adults	Female	41	Y
3890	2020	Nov	GA	Adults	Female	27	Y
3891	2020	Nov	KZ	Paeds	Male	29	M
3892	2020	Nov	KZ	Paeds	Female	2	M
3893	2020	Nov	GA	Adults	Female	32	Y
3894	2020	Nov	GA	Adults	Female	41	Y
3895	2020	Nov	GA	Paeds	Female	17	D
3896	2020	Nov	GA	Adults	Female	27	Y

3897	2020	Nov	GA	Adults	Female	94	Y
3898	2020	Nov	WC	Adults	Male	26	Y
3899	2020	Nov	KZ	Paeds	Female	10	D
3900	2020	Dec	GA	Paeds	Male	2	Y
3901	2020	Dec	GA	Unkown	Female		
3902	2020	Nov	KZ	Paeds	Female	25	D
3903	2020	Dec	GA	Adults	Male	49	Y
3904	2020	Dec	GA	Adults	Female	32	Y
3905	2020	Dec	GA	Adults	Male	20	Y
3906	2020	Dec	WC	Paeds	Male	4	D
3907	2020	Dec	WC	Paeds	Male	28	D
3908	2020	Dec	WC	Paeds	Female	2	M
3909	2020	Dec	KZ	Unkown	Female	4	D
3910	2020	Nov	WC	Adults	Female	26	Y
3911	2020	Dec	WC	Paeds	Male	4	M
3912	2020	Dec	GA	Adults	Female	59	Y
3913	2020	Dec	GA	Adults	Male	55	Y
3914	2020	Dec	GA	Adults	Female	16	Y
3915	2020	Dec	GA	Paeds	Male	14	D
3916	2020	Dec	WC	Adults	Male	62	Y
3917	2020	Dec	GA	Adults	Male	47	Y
3918	2020	Dec	GA	Paeds	Female	21	D
3919	2020	Dec	GA	Adults	Female	43	Y
3920	2020	Dec	GA	Adults	Male	52	Y
3921	2020	Dec	KZ	Adults	Male	41	Y
3922	2020	Dec	KZ	Paeds	Female	3	M
3923	2020	Dec	WC	Adults	Male	25	Y
3924	2020	Dec	GA	Adults	Female	26	Y
3925	2020	Dec	GA	Adults	Female	35	Y
3926	2020	Dec	GA	Paeds	Male	22	M
3927	2020	Dec	GA	Paeds	Female	1	M
3928	2020	Dec	GA	Adults	Male	29	Y
3929	2020	Dec	GA	Adults	Male	43	Y
3930	2020	Feb	GA	Adults	Male	33	Y
3931	2020	Dec	GA	Paeds	Female	7	D
3932	2020	Dec	GA	Paeds	Female	3	D
3933	2020	Dec	GA	Unkown	Female	41	Y
3934	2020	Dec	GA	Adults	Female	75	Y
3935	2020	Dec	FS	Adults	Female	57	Y
3936	2020	Dec	GA	Adults	Male	34	Y
3937	2020	Dec	GA	Adults	Male	31	Y
3938	2020	Dec	GA	Paeds	Male	21	M
3940	2020	Dec	GA	Paeds	Male	7	Y
3941	2020	Dec	GA	Adults	Female	35	Y
3942	2020	Dec	GA	Adults	Female	52	Y
3943	2020	Dec	GA	Adults	Female	56	Y
3944	2020	Dec	GA	Paeds	Male	11	Y
3945	2020	Dec	GA	Adults	Female	50	Y
3946	2020	Dec	GA	Adults	Female	35	Y
3947	2020	Dec	GA	Adults	Male	79	Y
3948	2020	Dec	GA	Adults	Female	73	Y
3949	2020	Dec	KZ	Paeds	Male	1	M
3950	2020	Dec	GA	Paeds	Female	2	M
3952	2020	Dec	GA	Adults	Female	75	Y
3953	2020	Dec	GA	Adults	Male	67	Y
3954	2020	Dec	GA	Adults	Male	50	Y

3955	2020	Dec	GA	Adults	Male	40	Y
3964	2020	Dec	WC	Adults	Male	45	Y
3993	2020	Nov	KZ	Paeds	Male	7	M
3994	2020	Dec	KZ	Paeds	Female	4	M
4174	2020	Jan	GA	Adults	Male	36	Y
4175	2020	Feb	GA	Adults	Male	52	Y
4176	2020	Feb	KZ	Paeds	Male	12	Y
4177	2020	Feb	GA	Adults	Male	36	Y
4178	2020	Feb	GA	Adults	Male	28	Y
4179	2020	Feb	KZ	Adults	Male	30	Y
4180	2020	Feb	GA	Adults	Male	55	Y
4181	2020	Mar	GA	Adults	Female	69	Y
4182	2020	Mar	KZ	Adults	Male	39	Y
4183	2020	Mar	GA	Adults	Female	37	Y
4184	2020	Jun	WC	Adults	Male	60	Y
4185	2020	Jul	KZ	Paeds	Female	2	M
4186	2020	Jul	KZ	Adults	Male	24	Y
4187	2020	Jul	KZ	Paeds	Male	4	M
4188	2020	Jul	KZ	Adults	Male	24	Y
4189	2020	Jul	GA	Adults	Male	62	Y
4190	2020	Jul	KZ	Adults	Male	28	Y
4191	2020	Jul	GA	Adults	Female	16	Y
4192	2020	Jul	WC	Adults	Male	34	Y
4193	2020	Jul	WC	Adults	Male	41	Y
4194	2020	Jul	GA	Paeds	Male	37	M
4195	2020	Jul	KZ	Paeds	Male	8	D
4196	2020	Jul	KZ	Paeds	Female	19	D
4197	2020	Jul	WC	Adults	Male	20	Y
4198	2020	Jul	GA	Adults	Male	45	Y
4199	2020	Jul	KZ	Paeds	Male	19	M
4200	2020	Jul	WC	Adults	Male	48	Y
4201	2020	Jul	GA	Adults	Female	17	Y
4202	2020	Jul	GA	Adults	Male	40	Y
4203	2020	Jul	KZ	Adults	Male	38	Y
4204	2020	Jul	GA	Paeds	Female	17	D
4205	2020	Jul	GA	Adults	Female	36	Y
4206	2020	Jul	GA	Adults	Male	49	Y
4207	2020	Jul	KZ	Adults	Female	80	Y
4208	2020	Jul	WC	Adults	Male	68	Y
4209	2020	Jul	GA	Unkown	Male		
4210	2020	Jul	KZ	Paeds	Male	50	M
4211	2020	Jul	GA	Paeds	Male	2	M
4212	2020	Jul	GA	Unkown	Female		
4213	2020	Jul	WC	Adults	Male	48	Y
4214	2020	Jul	GA	Adults	Female	28	Y
4215	2020	Jul	FS	Adults	Female	67	Y
4216	2020	Jul	GA	Adults	Male	40	Y
4217	2020	Jul	KZ	Paeds	Male	30	D
4218	2020	Jul	GA	Adults	Male	48	Y
4219	2020	Jul	GA	Adults	Female	31	Y
4220	2020	Jul	GA	Adults	Male	41	Y
4221	2020	Jul	KZ	Paeds	Male	1	M
4222	2020	Jul	GA	Adults	Female	65	Y
4223	2020	Jul	GA	Paeds	Male	14	D
4224	2020	Aug	GA	Adults	Female	27	Y
4225	2020	Aug	GA	Paeds	Female	13	D

4226	2020	Aug	GA	Adults	Male	34	Y
4227	2020	Aug	KZ	Adults	Male	36	Y
4228	2020	Aug	GA	Adults	Male	48	Y
4229	2020	Aug	GA	Adults	Male	33	Y
4230	2020	Aug	GA	Adults	Female	40	Y
4231	2020	Aug	GA	Adults	Female	55	Y
4232	2020	Aug	GA	Paeds	Male	19	D
4234	2020	Aug	GA	Adults	Male	18	Y
4235	2020	Aug	GA	Adults	Male	46	Y
4236	2020	Aug	GA	Unkown	Male		
4237	2020	Aug	WC	Adults	Male	58	Y
4238	2020	Aug	WC	Adults	Female	46	Y
4239	2020	Aug	GA	Adults	Male	35	Y
4240	2020	Aug	KZ	Adults	Female		Y
4241	2020	Aug	KZ	Paeds	Male	28	D
4242	2020	Aug	WC	Adults	Male	50	Y
4243	2020	Aug	GA	Paeds	Female	47	M
4244	2020	Aug	GA	Paeds	Male	9	D
4245	2020	Aug	KZ	Adults	Male	39	Y
4246	2020	Aug	WC	Paeds	Male	2	D
4247	2020	Aug	GA	Paeds	Female	9	D
4248	2020	Aug	KZ	Paeds	Female	5	M
4249	2020	Sep	GA	Adults	Female	35	Y
4250	2020	Sep	WC	Adults	Male	50	Y
4251	2020	Sep	KZ	Adults	Female	74	Y
4252	2020	Sep	KZ	Paeds	Male	2	M
4253	2020	Sep	GA	Unkown	Male	42	Y
4254	2020	Sep	KZ	Paeds	Female	5	M
4255	2020	Sep	GA	Paeds	Male	2	M
4256	2020	Sep	GA	Adults	Female	35	Y
4257	2020	Sep	KZ	Paeds	Male	6	Y
4258	2020	Sep	GA	Adults	Male	54	Y
4259	2020	Sep	GA	Paeds	Female	23	D
4260	2020	Sep	GA	Adults	Male	16	Y
4261	2020	Sep	GA	Adults	Male	58	Y
4262	2020	Sep	GA	Adults	Female	16	Y
4263	2020	Sep	WC	Adults	Female	50	Y
4264	2020	Sep	GA	Unkown	Female		
4265	2020	Sep	GA	Adults	Female	47	Y
4266	2020	Sep	GA	Adults	Female	36	Y
4267	2020	Sep	KZ	Adults	Male	29	Y
4268	2020	Sep	KZ	Paeds	Female		Y
4269	2020	Sep	FS	Adults	Female	43	Y
4270	2020	Sep	GA	Adults	Female	61	Y
4271	2020	Sep	KZ	Paeds	Female	9	D
4272	2020	Sep	WC	Adults	Male	51	Y
4273	2020	Sep	GA	Paeds	Female	1	M
4274	2020	Sep	GA	Adults	Female	56	Y
4275	2020	Sep	WC	Adults	Male	25	Y
4276	2020	Sep	WC	Paeds	Female	3	D
4277	2020	Sep	GA	Adults	Male	51	Y
4278	2020	Sep	KZ	Adults	Male	30	Y
4279	2020	Sep	GA	Adults	Female	30	Y
4280	2020	Sep	GA	Paeds	Male	49	M
4281	2020	Sep	GA	Adults	Male	74	Y
4282	2020	Sep	GA	Adults	Male	30	Y

4283	2020	Sep	WC	Adults	Female	77	Y
4284	2020	Sep	WC	Adults	Female	22	Y
4285	2020	Sep	GA	Adults	Female	69	Y
4286	2020	Sep	GA	Paeds	Female	0	D
4287	2020	Sep	GA	Paeds	Female	6	D
4288	2020	Oct	KZ	Adults	Female	31	Y
4289	2020	Oct	WC	Adults	Male	80	Y
4290	2020	Oct	GA	Unkown	Female	64	Y
4291	2020	Oct	GA	Adults	Male	31	Y
4292	2020	Oct	GA	Adults	Male	44	Y
4293	2020	Oct	FS	Paeds	Female	10	D
4294	2020	Oct	GA	Adults	Female	56	Y
4295	2020	Oct	GA	Adults	Male	64	Y
4296	2020	Oct	KZ	Adults	Female	42	Y
4297	2020	Oct	FS	Adults	Male	50	Y
4298	2020	Oct	GA	Paeds	Male	5	M
4299	2020	Oct	GA	Adults	Male	35	Y
4300	2020	Oct	GA	Adults	Female	19	Y
4301	2020	Oct	KZ	Paeds	Male	48	M
4302	2020	Oct	GA	Adults	Male	34	Y
4303	2020	Oct	FS	Adults	Male	37	Y
4304	2020	Oct	KZ	Adults	Male	54	Y
4305	2020	Oct	GA	Adults	Female	48	Y
4306	2020	Oct	GA	Adults	Male	40	Y
4307	2020	Oct	GA	Paeds	Female	1	M
4308	2020	Oct	GA	Adults	Female	35	Y
4309	2020	Oct	GA	Adults	Female	43	Y
4310	2020	Oct	KZ	Paeds	Male	12	D
4311	2020	Oct	KZ	Paeds	Male	12	Y
4312	2020	Oct	GA	Adults	Male	58	Y
4313	2020	Oct	GA	Adults	Male	66	Y
4314	2020	Oct	GA	Adults	Female	30	Y
4315	2020	Oct	GA	Adults	Male	40	Y
4316	2020	Oct	GA	Adults	Male	78	Y
4317	2020	Oct	GA	Adults	Female	72	Y
4318	2020	Oct	GA	Adults	Female	30	Y
4319	2020	Oct	GA	Adults	Female	45	Y
4320	2020	Oct	GA	Adults	Female	40	Y
4321	2020	Oct	GA	Adults	Male	28	Y
4322	2020	Oct	WC	Adults	Male	30	Y
4323	2020	Nov	GA	Adults	Female	41	Y
4324	2020	Nov	GA	Adults	Male	31	Y
4325	2020	Nov	GA	Adults	Female	40	Y
4326	2020	Nov	GA	Paeds	Male	2	M
4327	2020	Nov	GA	Unkown	Male	34	Y
4328	2020	Nov	KZ	Paeds	Male	20	M
4329	2020	Nov	GA	Adults	Female	45	Y
4330	2020	Nov	GA	Paeds	Male	57	M
4331	2020	Nov	GA	Adults	Male	24	Y
4332	2020	Nov	GA	Adults	Male	75	Y
4333	2020	Nov	GA	Adults	Male	42	Y
4334	2020	Nov	GA	Adults	Male	32	Y
4335	2020	Nov	KZ	Adults	Male	56	Y
4336	2020	Nov	GA	Adults	Male	65	Y
4337	2020	Nov	GA	Paeds	Male	2	D
4338	2020	Nov	KZ	Adults	Female	91	Y

4339	2020	Nov	GA	Unkown	Female		
4341	2020	Nov	GA	Paeds	Male	26	M
4342	2020	Nov	WC	Adults	Male	21	Y
4343	2020	Nov	GA	Adults	Female	46	Y
4345	2020	Nov	GA	Paeds	Male	21	D
4346	2020	Nov	GA	Adults	Male	36	Y
4347	2020	Nov	GA	Unkown	Male		
4348	2020	Nov	GA	Adults	Female	53	Y
4349	2020	Nov	GA	Paeds	Female	59	M
4350	2020	Nov	GA	Adults	Female	51	Y
4351	2020	Nov	KZ	Paeds	Male	14	D
4352	2020	Nov	GA	Adults	Male	38	Y
4353	2020	Nov	GA	Adults	Male	72	Y
4354	2020	Nov	GA	Paeds	Male	18	D
4355	2020	Nov	GA	Paeds	Male	15	Y
4356	2020	Nov	GA	Adults	Male	30	Y
4357	2020	Nov	GA	Adults	Male	46	Y
4358	2020	Nov	GA	Paeds	Female	3	D
4359	2020	Nov	GA	Adults	Female	30	Y
4360	2020	Nov	KZ	Paeds	Male	3	D
4361	2020	Dec	GA	Paeds	Male	1	M
4362	2020	Dec	KZ	Adults	Male	46	Y
4363	2020	Dec	GA	Adults	Female	47	Y
4364	2020	Dec	GA	Adults	Female	52	Y
4365	2020	Dec	GA	Adults	Female	60	Y
4366	2020	Dec	KZ	Paeds	Male	4	M
4367	2020	Dec	FS	Unkown	Male	31	Y
4368	2020	Dec	KZ	Paeds	Female	12	D
4369	2020	Dec	GA	Adults	Female	72	Y
4371	2020	Dec	GA	Adults	Female	29	Y
4372	2020	Dec	GA	Paeds	Male	21	D
4373	2020	Dec	WC	Adults	Male	21	Y
4374	2020	Dec	GA	Adults	Male	55	Y
4375	2020	Dec	GA	Adults	Male	60	Y
4376	2020	Dec	GA	Adults	Male	62	Y
4377	2020	Dec	GA	Paeds	Female	23	M
4378	2020	Dec	GA	Adults	Female	57	Y
4379	2020	Dec	GA	Paeds	Male	1	M
4380	2020	Dec	KZ	Paeds	Male	18	D
4381	2020	Dec	KZ	Paeds	Male	2	M
4382	2020	Dec	GA	Paeds	Male	18	M
4383	2020	Dec	GA	Paeds	Male	20	D
4384	2020	Dec	GA	Adults	Female	58	Y
4385	2020	Dec	GA	Adults	Female	49	Y
4386	2020	Dec	WC	Adults	Male	54	Y
4387	2020	Dec	GA	Adults	Female	47	Y
4388	2020	Dec	GA	Adults	Male	63	Y
4389	2020	Dec	GA	Unkown	Female	51	Y
4390	2020	Dec	GA	Unkown	Male		
4391	2020	Dec	WC	Adults	Female	53	Y
4392	2020	Dec	KZ	Adults	Male	50	Y
4393	2020	Dec	GA	Adults	Male	51	Y
4394	2020	Dec	GA	Adults	Male	25	Y
4395	2020	Dec	GA	Adults	Male	55	Y
4396	2020	Dec	GA	Adults	Male	40	Y
4397	2020	Dec	GA	Adults	Male	72	Y

4398	2020	Dec	KZ	Adults	Female	47	Y
4399	2020	Dec	KZ	Paeds	Male	11	M
4400	2020	Dec	GA	Adults	Male	65	Y
4401	2020	Dec	WC	Adults	Female	20	Y
4402	2020	Dec	KZ	Adults	Female	24	Y
4403	2020	Dec	GA	Adults	Female	62	Y
4404	2020	Dec	GA	Adults	Male	50	Y
4405	2020	Dec	GA	Adults	Female	58	Y
4406	2020	Dec	GA	Paeds	Unknown	7	D
4407	2020	Dec	GA	Paeds	Male	46	M
4408	2020	Dec	GA	Adults	Male	51	Y
4447	2020	Aug	WC	Adults	Female	42	Y
4449	2020	Jul	WC	Adults	Female	65	Y
4450	2020	May	GA	Adults	Male	45	Y
4451	2020	May	GA	Paeds	Male	27	M
4452	2020	Aug	GA	Adults	Female	13	D
4453	2020	Apr	GA	Adults	Male	21	Y
4454	2020	Apr	GA	Paeds	Male	33	M
4464	2020	Dec	GA	Adults	Male	51	Y
4490	2020	Sep	GA	Adults	Male	87	Y
4494	2020	Mar	GA	Paeds	Male	18	M
4495	2020	Oct	GA	Paeds	Male	11	M
4496	2020	Nov	KZ	Adults	Female	61	Y
4497	2020	Sep	GA	Paeds	Female	4	M
3939	2021	Jan	WC	Adults	Male	16	Y
3951	2021	Jan	KZ	Paeds	Female	21	M
3956	2021	Jan	WC	Adults	Female	56	Y
3957	2021	Jan	WC	Adults	Male	29	Y
3958	2021	Jan	WC	Adults	Female	63	Y
3959	2021	Jan	WC	Adults	Female	21	Y
3960	2021	Jan	WC	Paeds	Male	1	Y
3961	2021	Jan	GA	Paeds	Female	10	Y
3963	2021	Jan	GA	Adults	Male	51	Y
3965	2021	Jan	GA	Adults	Female	60	Y
3966	2021	Feb	GA	Paeds	Female	2	M
3969	2021	Jan	GA	Paeds	Female	15	M
3970	2021	Jan	GA	Paeds	Female	15	M
3971	2021	Jan	GA	Adults	Female	27	Y
3972	2021	Jan	GA	Adults	Male	56	Y
3973	2021	Jan	GA	Adults	Male	69	Y
3974	2021	Jan	KZ	Paeds	Unknown	6	D
3975	2021	Jan	KZ	Adults	Female	58	Y
3976	2021	Jan	KZ	Paeds	Female	2	M
3977	2021	Jan	KZ	Adults	Female	32	Y
3978	2021	Jan	KZ	Adults	Male	68	Y
3980	2021	Jan	WC	Adults	Male	54	Y
3981	2021	Jan	GA	Adults	Female	28	Y
3982	2021	Jan	GA	Adults	Female	67	Y
3983	2021	Jan	GA	Adults	Male	60	Y
3984	2021	Jan	GA	Adults	Female	48	Y
3985	2021	Jan	GA	Adults	Female	43	Y
3986	2021	Jan	GA	Paeds	Male	12	M
3987	2021	Jan	GA	Unkown	Male		
3988	2021	Jan	GA	Adults	Female	62	Y
3989	2021	Jan	GA	Paeds	Female	28	M
3990	2021	Jan	GA	Adults	Female	47	Y

3991	2021	Jan	WC	Adults	Male	56	Y
3992	2021	Jan	KZ	Adults	Female	30	Y
3996	2021	Jan	KZ	Paeds	Male	1	M
3997	2021	Jan	KZ	Paeds	Male	28	M
3998	2021	Jan	KZ	Paeds	Male	53	M
3999	2021	Jan	KZ	Adults	Female	60	Y
4000	2021	Jan	WC	Adults	Male	63	Y
4001	2021	Jan	WC	Adults	Male	63	Y
4002	2021	Jan	GA	Paeds	Male	18	D
4003	2021	Jan	GA	Adults	Male	23	Y
4004	2021	Jan	GA	Adults	Male	54	Y
4005	2021	Jan	GA	Adults	Female	59	Y
4006	2021	Jan	GA	Paeds	Male	1	M
4007	2021	Jan	GA	Adults	Male	41	Y
4008	2021	Jan	GA	Paeds	Male	1	Y
4009	2021	Jan	GA	Adults	Male	49	Y
4010	2021	Jan	GA	Paeds	Female	12	M
4011	2021	Feb	GA	Paeds	Male	8	D
4012	2021	Feb	GA	Adults	Male	37	Y
4013	2021	Jan	WC	Adults	Male	47	Y
4014	2021	Jan	WC	Adults	Male	44	Y
4015	2021	Feb	WC	Adults	Female	27	Y
4016	2021	Feb	KZ	Adults	Male	58	Y
4017	2021	Jan	WC	Adults	Female	24	Y
4018	2021	Jan	GA	Paeds	Male	0	D
4019	2021	Jan	GA	Adults	Male	57	Y
4020	2021	Feb	WC	Paeds	Male	9	Y
4021	2021	Feb	WC	Adults	Female	47	Y
4022	2021	Feb	KZ	Adults	Female	29	Y
4023	2021	Feb	KZ	Adults	Female	31	Y
4024	2021	Feb	WC	Paeds	Male	5	D
4025	2021	Feb	WC	Adults	Male	54	Y
4026	2021	Feb	GA	Paeds	Female	1	M
4027	2021	Feb	GA	Adults	Male	42	Y
4028	2021	Feb	GA	Adults	Male	42	Y
4029	2021	Feb	GA	Adults	Male	34	Y
4030	2021	Feb	GA	Paeds	Male	7	M
4031	2021	Feb	WC	Adults	Female	46	Y
4032	2021	Feb	KZ	Adults	Male	68	Y
4033	2021	Feb	KZ	Paeds	Male	2	D
4034	2021	Feb	KZ	Adults	Male	60	Y
4035	2021	Feb	GA	Adults	Male	38	Y
4036	2021	Feb	GA	Adults	Male	59	Y
4037	2021	Feb	GA	Paeds	Female	4	Y
4038	2021	Jan	WC	Paeds	Male	0	D
4039	2021	Jan	GA	Adults	Female	63	Y
4040	2021	Feb	GA	Adults	Female	64	Y
4041	2021	Feb	GA	Adults	Female	51	Y
4042	2021	Feb	GA	Unkown	Female		
4044	2021	Feb	GA	Paeds	Male	17	M
4045	2021	Feb	GA	Adults	Male	32	Y
4046	2021	Feb	GA	Adults	Male	35	Y
4047	2021	Feb	GA	Unkown	Female		
4049	2021	Feb	GA	Adults	Female	58	Y
4050	2021	Feb	GA	Paeds	Male	3	M
4051	2021	Feb	GA	Unkown	Female		

4052	2021	Feb	GA	Unkown	Male		
4053	2021	Feb	GA	Unkown	Female		
4054	2021	Feb	GA	Unkown	Female		
4056	2021	Feb	KZ	Paeds	Male	10	Y
4057	2021	Feb	KZ	Adults	Female	29	Y
4058	2021	Feb	KZ	Paeds	Female	2	M
4059	2021	Feb	WC	Adults	Male	38	Y
4060	2021	Feb	WC	Adults	Female	48	Y
4061	2021	Feb	GA	Paeds	Female	3	M
4063	2021	Feb	WC	Paeds	Male	2	M
4064	2021	Feb	WC	Paeds	Male	2	M
4065	2021	Feb	GA	Adults	Male	53	Y
4066	2021	Feb	GA	Adults	Female	61	Y
4067	2021	Feb	GA	Adults	Female	57	Y
4068	2021	Feb	GA	Paeds	Male	13	Y
4069	2021	Mar	WC	Adults	Female	24	Y
4070	2021	Feb	GA	Adults	Female	26	Y
4071	2021	Feb	GA	Adults	Male	50	Y
4072	2021	Mar	GA	Adults	Male	54	Y
4073	2021	Mar	GA	Adults	Female	56	Y
4074	2021	Feb	GA	Paeds	Male	8	M
4076	2021	Feb	GA	Paeds	Male	1	M
4077	2021	Mar	GA	Paeds	Female	2	M
4078	2021	Feb	GA	Paeds	Female	18	M
4079	2021	Feb	GA	Adults	Male	53	Y
4080	2021	Feb	GA	Paeds	Male	42	M
4081	2021	Feb	GA	Adults	Female	54	Y
4082	2021	Feb	GA	Adults	Female	58	Y
4083	2021	Feb	KZ	Adults	Male	39	Y
4084	2021	Mar	KZ	Adults	Male	28	Y
4085	2021	Mar	WC	Adults	Female	33	Y
4087	2021	Mar	GA	Adults	Male	66	Y
4088	2021	Mar	GA	Adults	Male	33	Y
4089	2021	Mar	GA	Adults	Male	39	Y
4090	2021	Mar	GA	Adults	Male	68	Y
4091	2021	Mar	GA	Paeds	Male	7	Y
4092	2021	Mar	GA	Paeds	Male	11	D
4093	2021	Feb	KZ	Adults	Female	75	Y
4094	2021	Feb	KZ	Adults	Female	69	Y
4095	2021	Feb	GA	Paeds	Male	34	M
4096	2021	Mar	GA	Adults	Female	49	Y
4097	2021	Feb	GA	Unkown	Female		
4098	2021	Mar	GA	Paeds	Female	4	D
4099	2021	Mar	GA	Adults	Female	45	Y
4100	2021	Mar	KZ	Paeds	Male	9	Y
4101	2021	Jan	KZ	Adults	Male	63	Y
4102	2021	Feb	KZ	Paeds	Female	1	M
4103	2021	Feb	KZ	Paeds	Male	2	M
4104	2021	Jan	KZ	Paeds	Male	10	Y
4105	2021	Jan	KZ	Adults	Male	31	Y
4106	2021	Mar	GA	Adults	Female	37	Y
4107	2021	Mar	GA	Adults	Female	45	Y
4108	2021	Mar	GA	Paeds	Male	10	D
4109	2021	Mar	KZ	Unkown	Female		
4110	2021	Mar	GA	Adults	Female	27	Y
4111	2021	Mar	GA	Paeds	Male	4	D

4112	2021	Mar	GA	Adults	Female	31	Y
4113	2021	Mar	GA	Paeds	Male	7	D
4114	2021	Mar	GA	Paeds	Male	12	D
4115	2021	Mar	GA	Adults	Male	69	Y
4116	2021	Mar	GA	Adults	Female	54	Y
4117	2021	Mar	GA	Adults	Male	44	Y
4118	2021	Mar	GA	Paeds	Male	8	D
4119	2021	Mar	GA	Adults	Female	68	Y
4120	2021	Mar	GA	Unkown	Male		
4121	2021	Mar	WC	Adults	Female	37	Y
4122	2021	Mar	WC	Adults	Male	61	Y
4123	2021	Mar	WC	Adults	Female	32	Y
4124	2021	Mar	KZ	Adults	Male	61	Y
4125	2021	Mar	KZ	Paeds	Female	10	M
4126	2021	Mar	KZ	Adults	Male	56	Y
4127	2021	Mar	KZ	Adults	Male	44	Y
4128	2021	Mar	KZ	Paeds	Female	3	M
4129	2021	Mar	KZ	Adults	Male	71	Y
4130	2021	Mar	GA	Adults	Female	69	Y
4131	2021	Mar	GA	Adults	Male	20	Y
4133	2021	Mar	GA	Adults	Female	81	Y
4134	2021	May	GA	Unkown	Female		
4135	2021	Mar	GA	Paeds	Female	19	M
4136	2021	Mar	GA	Paeds	Male	2	M
4137	2021	Mar	GA	Adults	Female	44	Y
4138	2021	Mar	GA	Paeds	Male	9	D
4139	2021	Mar	GA	Adults	Female	81	Y
4140	2021	Mar	GA	Paeds	Male	24	D
4141	2021	Mar	GA	Adults	Male	39	Y
4142	2021	Mar	GA	Adults	Female	38	Y
4143	2021	Mar	GA	Unkown	Male		
4144	2021	Apr	WC	Adults	Female	43	Y
4145	2021	Apr	WC	Adults	Male	55	Y
4146	2021	Mar	GA	Unkown	Female		
4147	2021	Mar	KZ	Adults	Male	25	Y
4148	2021	Mar	KZ	Adults	Male	39	Y
4149	2021	Mar	KZ	Paeds	Male	6	D
4150	2021	Mar	WC	Adults	Male	23	Y
4151	2021	Mar	GA	Paeds	Female	6	Y
4152	2021	Mar	GA	Adults	Female	63	Y
4153	2021	Apr	GA	Paeds	Male	3	D
4154	2021	Mar	GA	Paeds	Male	17	D
4156	2021	Mar	GA	Adults	Male	73	Y
4157	2021	Mar	GA	Adults	Male	60	Y
4158	2021	Apr	WC	Paeds	Female	4	M
4159	2021	Apr	FS	Adults	Female	45	Y
4160	2021	Apr	WC	Paeds	Male	8	D
4161	2021	Apr	GA	Adults	Female	35	Y
4162	2021	Apr	WC	Adults	Male	73	Y
4173	2021	Apr	GA	Unkown	Female		
4344	2021	May	GA	Unkown	Male		
4409	2021	Apr	WC	Paeds	Female	6	D
4410	2021	Apr	GA	Adults	Female	49	Y
4411	2021	Apr	GA	Paeds	Female	12	D
4412	2021	Apr	GA	Unkown	Male		
4413	2021	Apr	KZ	Adults	Female	56	Y

4414	2021	Apr	WC	Adults	Female	31	Y
4415	2021	Apr	GA	Adults	Female	49	Y
4416	2021	Apr	KZ	Adults	Male	56	Y
4417	2021	Apr	WC	Adults	Female	63	Y
4418	2021	Apr	GA	Adults	Male	67	Y
4419	2021	Apr	GA	Adults	Male	36	Y
4420	2021	Apr	GA	Adults	Male	67	Y
4421	2021	Apr	GA	Adults	Female	21	Y
4422	2021	Mar	GA	Unkown	Female		
4423	2021	Apr	WC	Adults	Female	60	Y
4424	2021	Apr	WC	Paeds	Female	3	M
4425	2021	Apr	WC	Adults	Male	83	Y
4426	2021	Apr	WC	Adults	Female	63	Y
4427	2021	Apr	KZ	Unkown	Female		
4428	2021	Apr	KZ	Paeds	Male	10	Y
4430	2021	Apr	WC	Adults	Female	50	Y
4431	2021	Apr	GA	Paeds	Female	9	D
4432	2021	Apr	KZ	Paeds	Female	1	M
4433	2021	Apr	KZ	Paeds	Female	13	M
4434	2021	Apr	KZ	Paeds	Male	16	D
4435	2021	May	GA	Paeds	Female	3	D
4436	2021	Apr	GA	Adults	Female	42	Y
4437	2021	Apr	GA	Adults	Female	56	Y
4438	2021	Apr	GA	Paeds	Female	21	D
4439	2021	Apr	GA	Adults	Female	61	Y
4440	2021	Apr	GA	Paeds	Female	9	Y
4441	2021	Apr	WC	Paeds	Female	11	D
4442	2021	Mar	KZ	Adults	Female	33	Y
4443	2021	Apr	KZ	Adults	Male	56	Y
4444	2021	Apr	GA	Paeds	Male	1	D
4445	2021	May	GA	Adults	Male	23	Y
4446	2021	May	GA	Adults	Male	43	Y
4448	2021	May	GA	Paeds	Male	5	D
4455	2021	May	GA	Adults	Female	38	Y
4456	2021	May	GA	Adults	Female	40	Y
4457	2021	May	WC	Adults	Male	38	Y
4458	2021	May	KZ	Paeds	Male	1	D
4459	2021	May	WC	Adults	Female	29	Y
4460	2021	May	GA	Adults	Male	33	Y
4461	2021	May	WC	Paeds	Female	19	D
4462	2021	May	GA	Paeds	Female	16	M
4463	2021	May	GA	Adults	Male	47	Y
4465	2021	May	GA	Adults	Male	33	Y
4466	2021	May	GA	Paeds	Male	13	Y
4467	2021	May	GA	Paeds	Male	9	D
4468	2021	May	KZ	Paeds	Male	1	M
4469	2021	May	WC	Adults	Male	25	Y
4470	2021	May	WC	Adults	Male	35	Y
4471	2021	May	GA	Adults	Male	57	Y
4473	2021	May	WC	Adults	Male	40	Y
4474	2021	May	GA	Paeds	Male	13	Y
4475	2021	May	WC	Adults	Female	55	Y
4476	2021	May	KZ	Adults	Male	52	Y
4477	2021	May	GA	Paeds	Female	18	D
4478	2021	May	GA	Paeds	Male	11	Y
4479	2021	May	WC	Adults	Female	44	Y

4480	2021	May	GA	Adults	Male	22	Y
4481	2021	May	GA	Paeds	Female	6	Y
4482	2021	May	GA	Adults	Female	47	Y
4483	2021	May	GA	Adults	Female	44	Y
4484	2021	May	GA	Unkown	Male		
4485	2021	May	KZ	Adults	Male	46	Y
4486	2021	May	GA	Paeds	Male	7	D
4487	2021	May	GA	Adults	Female	49	Y
4488	2021	May	GA	Unkown	Female		
4489	2021	May	WC	Adults	Male	57	Y
4491	2021	Jun	WC	Adults	Female	45	Y
4492	2021	Jun	WC	Adults	Female	44	Y
4498	2021	May	GA	Paeds	Male	20	D
4499	2021	May	GA	Unkown	Female		
4500	2021	Jun	GA	Adults	Male	58	Y
4501	2021	May	GA	Adults	Female	56	Y
4502	2021	May	GA	Paeds	Female	9	Y
4503	2021	Jun	GA	Adults	Female	57	Y
4504	2021	Jun	GA	Paeds	Female	2	M
4505	2021	May	GA	Paeds	Female	27	M
4506	2021	Jun	WC	Adults	Female	49	Y
4507	2021	Jun	WC	Adults	Male	40	Y
4508	2021	Jun	GA	Adults	Male	29	Y
4509	2021	Jun	GA	Adults	Female	52	Y
4510	2021	May	GA	Adults	Female	81	Y
4511	2021	Jun	GA	Adults	Female	70	Y
4512	2021	Jun	GA	Paeds	Female	4	D
4513	2021	Jan	FS	Adults	Female	51	Y
4514	2021	Jan	FS	Adults	Female	51	Y
4515	2021	Jan	GA	Unkown	Male		
4516	2021	Jan	GA	Unkown	Male		
4517	2021	Jan	GA	Adults	Female	1	M
4518	2021	Jan	GA	Adults	Female	71	Y
4519	2021	Jan	GA	Unkown	Female		
4520	2021	Jan	GA	Paeds	Male	10	D
4521	2021	Jan	GA	Adults	Male	56	Y
4522	2021	Jan	GA	Unkown	Male		
4523	2021	Jan	WC	Adults	Male	63	Y
4524	2021	Jan	GA	Adults	Male	70	Y
4525	2021	Jan	GA	Unkown	Female		
4526	2021	Jan	WC	Adults	Male	38	Y
4527	2021	Jan	GA	Adults	Female	60	Y
4528	2021	Jan	WC	Paeds	Male	2	D
4529	2021	Jan	GA	Adults	Male	32	Y
4530	2021	Jan	GA	Adults	Female	22	D
4531	2021	Jan	WC	Adults	Male	6	Y
4532	2021	Jan	KZ	Adults	Female	16	D
4533	2021	Jan	GA	Adults	Female	9	Y
4534	2021	Jan	GA	Adults	Female	9	Y
4535	2021	Jan	GA	Adults	Male	4	M
4536	2021	Jan	KZ	Adults	Female	16	D
4537	2021	Jan	GA	Adults	Unknown	72	Y
4538	2021	Jan	WC	Adults	Male	47	Y
4539	2021	Jan	GA	Adults	Male	51	Y
4540	2021	Jan	GA	Unkown	Unknown		
4541	2021	Jan	WC	Adults	Female	62	Y

4542	2021	Jan	GA	Adults	Female	56	Y
4543	2021	Jan	GA	Unkown	Female		
4544	2021	Jan	FS	Adults	Male	41	Y
4545	2021	Jan	GA	Adults	Female	55	Y
4546	2021	Jan	GA	Unkown	Male		
4547	2021	Jan	GA	Unkown	Female		
4548	2021	Jan	GA	Unkown	Male		
4549	2021	Jan	GA	Unkown	Male		
4550	2021	Jan	FS	Adults	Female	24	Y
4551	2021	Jan	GA	Adults	Female	28	Y
4552	2021	Jan	GA	Adults	Female	41	Y
4553	2021	Jan	WC	Adults	Male	3	M
4554	2021	Jan	GA	Adults	Female	8	M
4555	2021	Jan	KZ	Adults	Female	31	Y
4556	2021	Jan	GA	Unkown	Female		
4557	2021	Jan	GA	Adults	Female	62	Y
4558	2021	Jan	GA	Adults	Male	11	M
4559	2021	Jan	WC	Adults	Male	29	Y
4560	2021	Jan	WC	Adults	Female	48	Y
4561	2021	Jan	GA	Adults	Female	57	Y
4562	2021	Jan	GA	Unkown	Female		
4563	2021	Jan	GA	Adults	Male	1	M
4564	2021	Jan	GA	Adults	Male	2	M
4565	2021	Jan	GA	Adults	Male	37	Y
4566	2021	Jan	GA	Adults	Female	35	Y
4567	2021	Jan	GA	Adults	Female	55	Y
4568	2021	Jan	WC	Adults	Female	48	Y
4569	2021	Jan	GA	Adults	Female	36	Y
4570	2021	Jul	WC	Adults	Male	64	Y
4571	2021	Feb	GA	Unkown	Male		
4572	2021	Feb	KZ	Adults	Male	69	Y
4573	2021	Feb	GA	Adults	Male	51	Y
4574	2021	Feb	GA	Adults	Male	48	Y
4575	2021	Feb	GA	Unkown	Female		
4576	2021	Feb	GA	Unkown	Male		
4577	2021	Feb	GA	Unkown	Male		
4578	2021	Feb	GA	Adults	Female	42	Y
4579	2021	Feb	FS	Adults	Female	56	Y
4580	2021	Feb	GA	Adults	Female	58	Y
4581	2021	Feb	GA	Adults	Female	58	Y
4582	2021	Feb	GA	Adults	Female	52	Y
4583	2021	Feb	WC	Adults	Female	80	Y
4584	2021	Feb	GA	Adults	Female	61	Y
4585	2021	Feb	KZ	Adults	Female	54	Y
4586	2021	Feb	GA	Adults	Female	57	Y
4587	2021	Feb	KZ	Adults	Male	18	D
4588	2021	Feb	GA	Unkown	Female		
4589	2021	Feb	KZ	Paeds	Male	14	D
4590	2021	Feb	GA	Adults	Female	15	Y
4591	2021	Feb	GA	Adults	Female	30	Y
4592	2021	Feb	GA	Adults	Female	27	Y
4593	2021	Feb	GA	Adults	Female	15	Y
4594	2021	Feb	GA	Adults	Male	60	Y
4595	2021	Feb	GA	Adults	Male	61	Y
4596	2021	Feb	KZ	Adults	Male	41	Y
4597	2021	Feb	GA	Adults	Male	53	Y

4598	2021	Feb	GA	Paeds	Female	8	D
4599	2021	Feb	GA	Adults	Male	53	Y
4600	2021	Feb	GA	Adults	Female	22	Y
4601	2021	Feb	KZ	Adults	Male	21	Y
4602	2021	Feb	WC	Adults	Male	71	Y
4603	2021	Feb	GA	Unkown	Female		
4604	2021	Feb	GA	Adults	Female	16	M
4605	2021	Feb	GA	Adults	Male	30	Y
4606	2021	Feb	GA	Adults	Male	20	Y
4607	2021	Feb	GA	Unkown	Male		
4608	2021	Feb	FS	Adults	Female	39	Y
4609	2021	Feb	GA	Adults	Male	20	Y
4610	2021	Feb	GA	Unkown	Male		
4611	2021	Feb	GA	Adults	Female	42	Y
4612	2021	Feb	GA	Unkown	Male		
4613	2021	Feb	GA	Unkown	Female		
4614	2021	Feb	GA	Adults	Male	27	Y
4615	2021	Jul	GA	Adults	Male	43	Y
4616	2021	Feb	GA	Unkown	Female		
4617	2021	Feb	GA	Adults	Male	17	M
4618	2021	Feb	GA	Adults	Female	9	M
4619	2021	Feb	GA	Paeds	Male	10	D
4620	2021	Feb	KZ	Unkown	Male		
4621	2021	Feb	GA	Adults	Female	54	Y
4622	2021	Feb	KZ	Adults	Male	57	Y
4623	2021	Feb	KZ	Adults	Female	74	Y
4624	2021	Feb	FS	Paeds	Male	2	D
4625	2021	Feb	KZ	Adults	Male	37	Y
4626	2021	Mar	GA	Adults	Female	19	D
4627	2021	Mar	GA	Adults	Female	19	D
4628	2021	Mar	KZ	Adults	Male	37	Y
4629	2021	Mar	GA	Adults	Male	34	Y
4630	2021	Mar	GA	Adults	Female	34	Y
4631	2021	Mar	GA	Adults	Female	61	Y
4632	2021	Mar	GA	Adults	Female	81	Y
4633	2021	Mar	GA	Adults	Female	43	Y
4634	2021	Mar	GA	Unkown	Female		
4635	2021	Mar	GA	Unkown	Female		
4636	2021	Mar	KZ	Adults	Male	22	D
4637	2021	Mar	GA	Unkown	Female		
4638	2021	Jul	GA	Paeds	Male	23	M
4639	2021	Mar	WC	Adults	Male	48	Y
4640	2021	Mar	GA	Unkown	Female		
4641	2021	Mar	GA	Adults	Female	1	M
4642	2021	Mar	GA	Adults	Female	12	Y
4643	2021	Mar	GA	Adults	Female	36	Y
4644	2021	Mar	GA	Adults	Female	3	M
4645	2021	Mar	FS	Adults	Female	66	Y
4646	2021	Mar	GA	Adults	Female	54	Y
4647	2021	Mar	KZ	Adults	Female	1	M
4648	2021	Mar	GA	Adults	Female	65	Y
4649	2021	Mar	GA	Adults	Male	66	Y
4650	2021	Mar	WC	Adults	Male	44	Y
4651	2021	Mar	GA	Unkown	Female		
4652	2021	Mar	GA	Adults	Male	66	Y
4653	2021	Mar	GA	Adults	Male	1	M

4654	2021	Mar	GA	Adults	Male	35	Y
4655	2021	Mar	GA	Adults	Female	72	Y
4656	2021	Mar	WC	Adults	Male	25	Y
4657	2021	Mar	WC	Adults	Male	25	Y
4658	2021	Mar	KZ	Adults	Male	1	M
4659	2021	Mar	KZ	Adults	Female	24	D
4660	2021	Mar	GA	Adults	Male	30	Y
4661	2021	Mar	GA	Adults	Male	31	Y
4662	2021	Mar	KZ	Adults	Female	24	D
4663	2021	Mar	FS	Adults	Female	58	Y
4664	2021	Mar	KZ	Adults	Male	33	Y
4665	2021	Mar	GA	Adults	Male	46	Y
4666	2021	Mar	FS	Adults	Male	43	Y
4667	2021	Mar	GA	Unkown	Male		
4668	2021	Mar	GA	Unkown	Male		
4669	2021	Mar	GA	Unkown	Male		
4670	2021	Mar	GA	Adults	Male	81	Y
4671	2021	Mar	GA	Adults	Male	39	Y
4672	2021	Mar	FS	Adults	Male	5	M
4673	2021	Mar	GA	Adults	Female	57	Y
4674	2021	Jul	GA	Adults	Male	41	Y
4675	2021	Mar	KZ	Paeds	Female	6	D
4676	2021	Mar	FS	Adults	Male	37	Y
4677	2021	Mar	GA	Adults	Female	2	M
4678	2021	Mar	GA	Adults	Female	52	Y
4679	2021	Mar	GA	Adults	Male	56	Y
4680	2021	Mar	GA	Unkown	Male		
4681	2021	Mar	FS	Adults	Male	19	Y
4682	2021	Mar	WC	Adults	Male	62	Y
4683	2021	Mar	GA	Adults	Male	50	Y
4684	2021	Mar	GA	Adults	Male	56	Y
4685	2021	Mar	GA	Adults	Female	58	Y
4686	2021	Mar	GA	Adults	Female	58	Y
4687	2021	Mar	GA	Adults	Male	41	Y
4688	2021	Mar	GA	Adults	Female	70	Y
4689	2021	Mar	WC	Adults	Female	67	Y
4690	2021	Mar	GA	Adults	Unknown	72	Y
4691	2021	Mar	GA	Adults	Female	1	M
4692	2021	Jun	GA	Adults	Male	31	Y
4693	2021	Jun	GA	Adults	Male	55	Y
4694	2021	Jun	GA	Adults	Male	48	Y
4695	2021	Jun	GA	Adults	Male	42	Y
4696	2021	Jun	GA	Adults	Male	31	Y
4697	2021	May	GA	Paeds	Female	4	M
4698	2021	May	GA	Paeds	Female	17	D
4699	2021	May	GA	Adults	Female	32	Y
4700	2021	Jun	KZ	Adults	Female	49	Y
4702	2021	Jun	WC	Adults	Female	41	Y
4703	2021	Jun	GA	Adults	Male	42	Y
4704	2021	Jun	GA	Paeds	Female	9	Y
4705	2021	Jun	GA	Adults	Male	46	Y
4706	2021	Jun	WC	Adults	Male	44	Y
4707	2021	Jun	GA	Adults	Female	73	Y
4708	2021	Jun	GA	Adults	Male	67	Y
4709	2021	Jun	GA	Adults	Male	44	Y
4711	2021	Jun	GA	Adults	Female	65	Y

4712	2021	Jun	GA	Adults	Male	65	Y
4713	2021	Jun	WC	Adults	Female	41	Y
4714	2021	Jun	KZ	Adults	Male	26	Y
4715	2021	Jun	KZ	Adults	Female	46	Y
4717	2021	Jun	GA	Paeds	Female	4	D
4718	2021	Jun	GA	Paeds	Female	9	D
4719	2021	Jun	GA	Unkown	Female		
4720	2021	Jun	GA	Adults	Male	71	Y
4721	2021	Jun	WC	Adults	Male	74	Y
4722	2021	Jun	WC	Adults	Female	47	Y
4723	2021	Jun	KZ	Adults	Female	28	Y
4724	2021	Jun	KZ	Adults	Female	55	Y
4725	2021	Jun	WC	Adults	Female	52	Y
4726	2021	Jun	KZ	Paeds	Male	1	M
4727	2021	Jun	GA	Adults	Female	22	Y
4728	2021	Jun	GA	Adults	Female	22	Y
4729	2021	Jun	GA	Adults	Female	30	Y
4730	2021	Jun	GA	Adults	Male	69	Y
4731	2021	Jun	GA	Adults	Female	65	Y
4732	2021	Jun	GA	Adults	Female	54	Y
4733	2021	Jun	GA	Adults	Male	63	Y
4734	2021	Jun	GA	Adults	Female	33	Y
4736	2021	Jun	GA	Adults	Female	68	Y
4737	2021	Jun	GA	Adults	Female	68	Y
4738	2021	Jun	GA	Adults	Male	53	Y
4740	2021	Jul	GA	Paeds	Female	2	M
4742	2021	Jun	GA	Adults	Female	69	Y
4743	2021	Jun	GA	Paeds	Female	11	Y
4744	2021	Jul	WC	Adults	Female	64	Y
4745	2021	Jul	WC	Adults	Male	71	Y
4747	2021	Jul	GA	Paeds	Male	25	D
4748	2021	Jun	GA	Paeds	Male	7	M
4749	2021	Jun	GA	Adults	Male	27	Y
4750	2021	Jul	GA	Paeds	Female	35	M
4751	2021	Jul	GA	Adults	Male	53	Y
4752	2021	Jul	GA	Paeds	Male	11	D
4753	2021	Jun	GA	Adults	Male	26	Y
4754	2021	Jun	GA	Adults	Female	47	Y
4755	2021	Jun	GA	Unkown	Unknown		
4757	2021	Jun	GA	Adults	Male	36	Y
4758	2021	Jul	GA	Adults	Male	43	Y
4759	2021	Jul	WC	Adults	Female	60	Y
4760	2021	Jul	GA	Unkown	Female		
4761	2021	Jul	GA	Adults	Female	62	Y
4762	2021	Jul	GA	Adults	Male	53	Y
4763	2021	Jul	GA	Paeds	Male	1	M
4764	2021	Jul	GA	Paeds	Male	2	M
4765	2021	Jul	WC	Paeds	Male	5	M
4767	2021	Jul	GA	Adults	Male	49	Y
4768	2021	Jul	WC	Adults	Female	60	Y
4769	2021	Jul	GA	Adults	Male	75	Y
4770	2021	Jul	KZ	Adults	Female	28	Y
4771	2021	Jul	KZ	Paeds	Female	1	M
4772	2021	Jul	WC	Paeds	Male	5	D
4773	2021	Jul	WC	Adults	Female	62	Y
4774	2021	Jul	WC	Adults	Female	62	Y

4775	2021	Jul	GA	Adults	Female	47	Y
4776	2021	Jul	GA	Unkown	Female		
4777	2021	Jul	GA	Adults	Male	44	Y
4779	2021	Jul	KZ	Paeds	Male	9	Y
4780	2021	Jul	KZ	Paeds	Female	14	D
4781	2021	Jul	KZ	Paeds	Male	1	M
4782	2021	Jul	WC	Adults	Male	76	Y
4783	2021	Jul	WC	Adults	Male	35	Y
4784	2021	Jul	WC	Adults	Female	41	Y
4785	2021	Jul	WC	Adults	Female	72	Y
4786	2021	Jul	WC	Paeds	Female	1	M
4787	2021	Jul	GA	Adults	Female	59	Y
4788	2021	Jul	GA	Adults	Male	33	Y
4789	2021	Jul	GA	Adults	Male	73	Y
4790	2021	Jul	GA	Paeds	Male	4	Y
4791	2021	Jul	GA	Adults	Female	40	Y
4792	2021	Jul	GA	Adults	Male	56	Y
4793	2021	Jul	GA	Adults	Female	43	Y
4794	2021	Jul	WC	Adults	Male	40	Y
4795	2021	Jul	GA	Paeds	Male	3	M
4796	2021	Jul	WC	Adults	Male	41	Y
4797	2021	Jul	GA	Adults	Female	59	Y
4798	2021	Jul	GA	Paeds	Male	1	M
4799	2021	Jul	GA	Adults	Male	39	Y
4800	2021	Jul	GA	Adults	Female	52	Y
4801	2021	Jul	GA	Adults	Female	56	Y
4802	2021	Jul	GA	Unkown	Male		
4803	2021	Jul	GA	Adults	Male	61	Y
4804	2021	Jul	GA	Adults	Female	49	Y
4805	2021	Jul	GA	Adults	Female	49	Y
4806	2021	Jul	GA	Adults	Male	56	Y
4807	2021	Aug	GA	Adults	Female	56	Y
4808	2021	Aug	GA	Adults	Male	45	Y
4809	2021	Aug	GA	Adults	Female	44	Y
4810	2021	Aug	GA	Adults	Female	57	Y
4812	2021	Aug	GA	Paeds	Female	3	M
4813	2021	Aug	GA	Adults	Male	27	Y
4814	2021	Jul	GA	Paeds	Female	13	Y
4815	2021	Jul	GA	Adults	Female	37	Y
4816	2021	Aug	GA	Adults	Male	56	Y
4817	2021	Aug	WC	Paeds	Male	6	D
4818	2021	Aug	WC	Adults	Female	59	Y
4819	2021	Aug	WC	Paeds	Female	9	D
4820	2021	Aug	KZ	Unkown	Male		
4821	2021	Aug	GA	Adults	Male	35	Y
4822	2021	Aug	GA	Adults	Female	52	Y
4823	2021	Aug	GA	Adults	Male	61	Y
4825	2021	Aug	GA	Adults	Female	71	Y
4826	2021	Aug	WC	Adults	Female	76	Y
4827	2021	Aug	GA	Adults	Male	35	Y
4828	2021	Aug	GA	Paeds	Female	5	D
4829	2021	Aug	GA	Adults	Male	30	Y
4830	2021	Aug	WC	Adults	Male	53	Y
4832	2021	Aug	GA	Adults	Male	56	Y
4833	2021	Aug	GA	Paeds	Female	5	D
4834	2021	Aug	GA	Adults	Female	56	Y

4835	2021	Aug	GA	Paeds	Female	11	D
4836	2021	Aug	GA	Adults	Male	32	Y
4837	2021	Aug	GA	Adults	Male	39	Y
4838	2021	Aug	GA	Adults	Male	30	Y
4839	2021	Aug	GA	Adults	Female	63	Y
4840	2021	Aug	KZ	Paeds	Female	12	Y
4841	2021	Aug	KZ	Adults	Male	35	Y
4842	2021	Aug	KZ	Adults	Female	22	Y
4843	2021	Aug	KZ	Paeds	Male	8	M
4844	2021	Aug	GA	Adults	Female	33	Y
4845	2021	Aug	WC	Adults	Male	68	Y
4846	2021	Aug	WC	Paeds	Male	9	D
4847	2021	Aug	WC	Adults	Male	40	Y
4848	2021	Aug	KZ	Adults	Female	38	Y
4849	2021	Aug	WC	Adults	Male	43	Y
4850	2021	Aug	WC	Adults	Female	41	Y
4851	2021	Aug	WC	Adults	Female	39	Y
4852	2021	Aug	WC	Adults	Male	31	Y
4853	2021	Aug	WC	Adults	Female	54	Y
4854	2021	Sep	WC	Paeds	Male	7	D
4855	2021	Aug	GA	Adults	Female	56	Y
4856	2021	Aug	GA	Paeds	Unknown	11	D
4857	2021	Aug	GA	Adults	Female	47	Y
4858	2021	Aug	GA	Adults	Male	36	Y
4860	2021	Aug	GA	Adults	Female	52	Y
4861	2021	Aug	GA	Adults	Male	32	Y
4862	2021	Aug	GA	Paeds	Female	8	Y
4863	2021	Aug	GA	Adults	Female	18	Y
4864	2021	Aug	GA	Adults	Male	58	Y
4865	2021	Aug	GA	Adults	Female	49	Y
4866	2021	Aug	GA	Adults	Female	74	Y
4867	2021	Aug	GA	Adults	Female	54	Y
4868	2021	Aug	GA	Paeds	Male	17	D
4869	2021	Sep	WC	Paeds	Male	5	D
4870	2021	Aug	GA	Unkown	Male		
4871	2021	Aug	GA	Unkown	Male		
4872	2021	Aug	GA	Adults	Female	49	Y
4873	2021	Aug	GA	Adults	Female	33	Y
4874	2021	Aug	GA	Adults	Male	81	Y
4875	2021	Aug	GA	Unkown	Male		
4877	2021	Aug	GA	Adults	Male	66	Y
4878	2021	Aug	WC	Paeds	Female	28	M
4879	2021	Aug	WC	Adults	Male	58	Y
4881	2021	Aug	GA	Adults	Male	24	Y
4883	2021	Jul	GA	Adults	Female	55	Y
4884	2021	Aug	GA	Paeds	Female	2	M
4885	2021	Aug	GA	Paeds	Male	2	M
4889	2021	Sep	WC	Paeds	Female	3	D
4891	2021	Aug	GA	Unkown	Female	65	Y
4892	2021	Sep	GA	Adults	Male	50	Y
4893	2021	Sep	GA	Adults	Female	41	Y
4894	2021	Sep	GA	Paeds	Male	3	M
4895	2021	Sep	FS	Paeds	Female	16	D
4896	2021	Sep	GA	Adults	Female	59	Y
4897	2021	Sep	WC	Adults	Female	37	Y
4898	2021	Sep	GA	Adults	Female	69	Y

4899	2021	Sep	GA	Paeds	Male	0	D
4900	2021	Aug	GA	Adults	Male	17	Y
4901	2021	Sep	GA	Paeds	Male	28	M
4902	2021	Sep	GA	Adults	Male	44	Y
4903	2021	Sep	GA	Adults	Male	44	Y
4904	2021	Sep	GA	Paeds	Male	6	D
4905	2021	Jul	WC	Adults	Male	46	Y
4906	2021	Sep	GA	Paeds	Female	2	M
4907	2021	Sep	GA	Adults	Female	16	Y
4909	2021	Sep	KZ	Adults	Female	22	Y
4910	2021	Sep	GA	Adults	Male	41	Y
4911	2021	Sep	WC	Paeds	Female	3	M
4912	2021	Sep	WC	Adults	Male	46	Y
4913	2021	Sep	GA	Adults	Male	60	Y
4914	2021	Sep	GA	Adults	Male	108	Y
4915	2021	Sep	GA	Paeds	Male	2	M
4916	2021	Sep	GA	Adults	Female	40	Y
4917	2021	Sep	GA	Adults	Male	39	Y
4918	2021	Sep	GA	Adults	Male	62	Y
4920	2021	Sep	WC	Paeds	Male	7	D
4921	2021	Sep	GA	Unkown	Female		
4922	2021	Sep	GA	Unkown	Female		
4923	2021	Sep	GA	Adults	Male	62	Y
4924	2021	Sep	GA	Adults	Male	40	Y
4925	2021	Sep	GA	Paeds	Male	4	M
4926	2021	Sep	GA	Adults	Male	46	Y
4927	2021	Sep	GA	Paeds	Male	3	M
4928	2021	Sep	GA	Paeds	Female	14	D
4929	2021	Sep	GA	Paeds	Female	3	D
4930	2021	Sep	GA	Adults	Male	71	Y
4931	2021	Sep	GA	Paeds	Female	10	D
4932	2021	Sep	GA	Adults	Male	35	Y
4934	2021	Sep	GA	Adults	Female	62	Y
4935	2021	Sep	GA	Adults	Male	26	Y
4936	2021	Sep	WC	Paeds	Female	6	D
4937	2021	Sep	WC	Adults	Female	64	Y
4938	2021	Sep	GA	Adults	Female	17	Y
4939	2021	Sep	GA	Adults	Male	30	Y
4940	2021	Sep	GA	Adults	Female	50	Y
4941	2021	Sep	GA	Adults	Male	32	Y
4942	2021	Sep	GA	Paeds	Female	44	M
4943	2021	Sep	GA	Adults	Male	53	Y
4944	2021	Sep	GA	Adults	Female	66	Y
4945	2021	Sep	WC	Adults	Female	50	Y
4947	2021	Oct	GA	Paeds	Male	9	D
4949	2021	Oct	GA	Paeds	Male	3	M
4950	2021	Sep	WC	Paeds	Female	3	M
4951	2021	Sep	GA	Adults	Male	32	Y
4952	2021	Sep	GA	Adults	Male	55	Y
4953	2021	Sep	GA	Adults	Female	22	Y
4954	2021	Sep	GA	Paeds	Male	20	D
4955	2021	Sep	GA	Paeds	Male	31	M
4956	2021	Sep	GA	Adults	Male	31	Y
4957	2021	Sep	GA	Unkown	Female		
4958	2021	Sep	GA	Paeds	Male	22	D
4959	2021	Sep	GA	Adults	Male	62	Y

4960	2021	Sep	GA	Adults	Female	53	Y
4961	2021	Oct	WC	Paeds	Male	4	D
4962	2021	Oct	GA	Unkown	Male		
4963	2021	Oct	WC	Paeds	Male	2	D
4964	2021	Sep	GA	Adults	Female	21	Y
4965	2021	Sep	GA	Adults	Male	22	Y
4966	2021	Sep	GA	Adults	Male	39	Y
4967	2021	Sep	GA	Unkown	Female		
4968	2021	Oct	GA	Paeds	Male	13	D
4969	2021	Oct	WC	Adults	Male	52	Y
4970	2021	Oct	GA	Adults	Female	51	Y
4971	2021	Oct	GA	Adults	Male	72	Y
4972	2021	Oct	GA	Paeds	Male	18	M
4973	2021	Oct	GA	Adults	Male	62	Y
4974	2021	Oct	GA	Adults	Male	29	Y
4975	2021	Oct	KZ	Paeds	Female	10	M
4976	2021	Oct	WC	Paeds	Male	2	M
4977	2021	Oct	WC	Paeds	Male	1	M
4978	2021	Oct	WC	Paeds	Male	1	M
4979	2021	Oct	GA	Adults	Male	61	Y
4980	2021	Oct	GA	Paeds	Female	8	Y
4981	2021	Oct	KZ	Paeds	Female	5	Y
4982	2021	Oct	GA	Unkown	Male		
4983	2021	Oct	GA	Adults	Male	57	Y
4984	2021	Oct	GA	Adults	Female	18	Y
4985	2021	Oct	GA	Paeds	Female	27	D
4986	2021	Oct	KZ	Paeds	Female	2	M
4987	2021	Oct	KZ	Adults	Female	53	Y
4988	2021	Oct	KZ	Adults	Male	63	Y
4989	2021	Oct	GA	Paeds	Male	18	M
4990	2021	Oct	WC	Paeds	Male	10	D
4991	2021	Oct	KZ	Unkown	Male		
4992	2021	Oct	GA	Unkown	Male		
4993	2021	Oct	GA	Adults	Male	36	Y
4994	2021	Oct	WC	Paeds	Female	5	M
4995	2021	Oct	WC	Adults	Female	80	Y
4996	2021	Oct	GA	Paeds	Female	17	D
4997	2021	Oct	GA	Adults	Male	32	Y
4998	2021	Oct	GA	Paeds	Female	18	M
4999	2021	Oct	GA	Unkown	Male		
5000	2021	Oct	WC	Adults	Female	81	Y
5001	2021	Oct	WC	Adults	Female	69	Y
5002	2021	Nov	GA	Adults	Female	36	Y
5003	2021	Jan	GA	Unkown	Male		
5004	2021	Jan	GA	Unkown	Male		
5005	2021	Jan	GA	Adults	Male	42	Y
5006	2021	Jan	KZ	Adults	Female	88	Y
5008	2021	Jan	GA	Adults	Male	63	Y
5009	2021	Jan	KZ	Adults	Female	66	Y
5010	2021	Jan	FS	Adults	Female	19	Y
5011	2021	Jan	FS	Adults	Female	17	Y
5012	2021	Jan	FS	Adults	Male	18	Y
5013	2021	Jan	GA	Adults	Female	56	Y
5014	2021	Jan	GA	Adults	Male	20	Y
5015	2021	Jan	GA	Adults	Male	22	Y
5016	2021	Jan	GA	Adults	Female	61	Y

5017	2021	Jan	FS	Paeds	Female	11	Y
5018	2021	Jan	GA	Paeds	Male	33	M
5019	2021	Jan	KZ	Adults	Female	36	Y
5020	2021	Jan	GA	Unkown	Female		
5021	2021	Jan	GA	Adults	Male	57	Y
5022	2021	Jan	KZ	Adults	Female	17	Y
5023	2021	Jan	GA	Adults	Male	51	Y
5024	2021	Jan	GA	Adults	Male	51	Y
5025	2021	Jan	GA	Paeds	Male	11	Y
5026	2021	Jan	GA	Unkown	Female		
5027	2021	Jan	FS	Adults	Female	69	Y
5028	2021	Jan	FS	Adults	Female	69	Y
5029	2021	Jan	FS	Adults	Female	69	Y
5030	2021	Feb	GA	Unkown	Female		
5031	2021	Feb	FS	Adults	Male	23	Y
5032	2021	Feb	GA	Adults	Male	57	Y
5033	2021	Feb	GA	Adults	Male	60	Y
5034	2021	Feb	KZ	Adults	Male	36	Y
5035	2021	Feb	KZ	Adults	Female	66	Y
5036	2021	Feb	GA	Unkown	Male		
5037	2021	Feb	GA	Adults	Female	33	Y
5038	2021	Feb	GA	Adults	Male	50	Y
5039	2021	Feb	KZ	Paeds	Male	5	D
5040	2021	Feb	FS	Adults	Female	21	Y
5041	2021	Feb	GA	Unkown	Unknown		
5042	2021	Feb	KZ	Adults	Female	35	Y
5043	2021	Feb	KZ	Paeds	Female	4	D
5044	2021	Feb	KZ	Paeds	Male	1	M
5045	2021	Feb	FS	Adults	Male	20	Y
5046	2021	Mar	GA	Adults	Male	40	Y
5047	2021	Mar	GA	Paeds	Female	12	D
5048	2021	Mar	KZ	Paeds	Male	8	Y
5049	2021	Mar	WC	Adults	Female	56	Y
5050	2021	Mar	GA	Adults	Male	24	Y
5051	2021	Mar	FS	Unkown	Female		
5052	2021	Mar	KZ	Paeds	Female	24	D
5053	2021	Mar	KZ	Paeds	Male	25	D
5054	2021	Mar	GA	Adults	Female	56	Y
5055	2021	Nov	GA	Paeds	Male	2	M
5056	2021	Mar	GA	Adults	Female	67	Y
5057	2021	Mar	FS	Adults	Female	46	Y
5058	2021	Mar	GA	Adults	Male	59	Y
5059	2021	Mar	FS	Adults	Female	21	Y
5060	2021	Mar	FS	Adults	Female	24	Y
5061	2021	Mar	KZ	Adults	Male	42	Y
5062	2021	Mar	WC	Adults	Male	31	Y
5063	2021	Mar	GA	Adults	Female	18	Y
5064	2021	Mar	GA	Adults	Male	39	Y
5065	2021	Mar	GA	Adults	Male	39	Y
5066	2021	Mar	GA	Adults	Female	47	Y
5067	2021	Mar	GA	Unkown	Female		
5068	2021	Mar	KZ	Unkown	Male		
5069	2021	Apr	WC	Adults	Male	51	Y
5070	2021	Apr	KZ	Paeds	Female	10	M
5071	2021	Apr	GA	Adults	Male	39	Y
5072	2021	Apr	GA	Adults	Male	39	Y

5073	2021	Apr	GA	Adults	Male	39	Y
5074	2021	Apr	FS	Adults	Female	44	Y
5075	2021	Apr	KZ	Adults	Female	75	Y
5076	2021	Apr	GA	Unkown	Female		
5077	2021	Apr	WC	Paeds	Female	2	M
5078	2021	Apr	GA	Adults	Male	72	Y
5079	2021	Apr	GA	Paeds	Male	1	M
5080	2021	Apr	KZ	Adults	Female	44	Y
5081	2021	Apr	GA	Adults	Male	52	Y
5082	2021	Apr	GA	Adults	Male	52	Y
5083	2021	Apr	GA	Adults	Female	68	Y
5084	2021	Apr	GA	Adults	Female	68	Y
5085	2021	Apr	WC	Paeds	Female	3	M
5086	2021	Apr	GA	Adults	Male	73	Y
5087	2021	Apr	GA	Adults	Female	63	Y
5088	2021	Apr	GA	Adults	Male	72	Y
5089	2021	Apr	GA	Unkown	Male		
5090	2021	Apr	KZ	Paeds	Female	2	M
5091	2021	Apr	GA	Paeds	Female	15	Y
5092	2021	Apr	GA	Paeds	Female	15	Y
5093	2021	Apr	GA	Adults	Male	56	Y
5094	2021	Apr	FS	Adults	Male	61	Y
5095	2021	Apr	GA	Unkown	Male		
5096	2021	Apr	GA	Unkown	Female		
5097	2021	Apr	GA	Unkown	Male		
5098	2021	Apr	GA	Paeds	Female	1	D
5100	2021	Apr	GA	Adults	Male	59	Y
5101	2021	Apr	GA	Adults	Female	54	Y
5102	2021	Apr	GA	Adults	Male	66	Y
5103	2021	Apr	FS	Adults	Male	70	Y
5104	2021	Apr	GA	Unkown	Female		
5105	2021	Apr	FS	Adults	Male	16	Y
5106	2021	Apr	KZ	Adults	Male	37	Y
5107	2021	Apr	KZ	Adults	Male	44	Y
5108	2021	Apr	GA	Unkown	Female		
5109	2021	Apr	GA	Unkown	Female		
5110	2021	Apr	GA	Unkown	Female		
5111	2021	Apr	GA	Unkown	Female		
5112	2021	Apr	KZ	Adults	Female	28	Y
5113	2021	Apr	FS	Adults	Female	55	Y
5114	2021	Apr	GA	Adults	Male	38	Y
5115	2021	Apr	GA	Paeds	Female	0	D
5116	2021	Apr	GA	Paeds	Female	0	D
5117	2021	Apr	FS	Paeds	Male	3	M
5118	2021	Apr	FS	Paeds	Male	3	M
5119	2021	Apr	GA	Paeds	Male	9	Y
5120	2021	Apr	GA	Paeds	Male	9	Y
5121	2021	Apr	GA	Adults	Male	86	Y
5122	2021	Apr	FS	Adults	Female	17	Y
5123	2021	Apr	FS	Adults	Female	38	Y
5124	2021	Apr	KZ	Adults	Male	43	Y
5125	2021	Apr	GA	Adults	Female	52	Y
5126	2021	Apr	GA	Paeds	Female	6	D
5127	2021	Apr	GA	Paeds	Male	10	Y
5128	2021	Apr	WC	Adults	Male	21	Y
5129	2021	Apr	KZ	Adults	Female	30	Y

5130	2021	Apr	GA	Adults	Female	63	Y
5131	2021	Apr	GA	Adults	Female	63	Y
5132	2021	Apr	GA	Paeds	Male	1	M
5133	2021	Apr	FS	Adults	Female	49	Y
5134	2021	Apr	FS	Adults	Female	49	Y
5135	2021	Apr	GA	Adults	Female	61	Y
5136	2021	Apr	GA	Paeds	Male	15	Y
5137	2021	Apr	WC	Adults	Male	23	Y
5138	2021	Apr	KZ	Paeds	Female	7	D
5139	2021	Apr	GA	Adults	Male	67	Y
5140	2021	Apr	GA	Unkown	Female		
5141	2021	Apr	GA	Unkown	Female		
5142	2021	Apr	GA	Paeds	Male	20	M
5143	2021	Apr	WC	Adults	Male	69	Y
5144	2021	Apr	GA	Unkown	Female		
5145	2021	Apr	GA	Unkown	Male		
5146	2021	Apr	GA	Paeds	Female	14	D
5147	2021	Apr	FS	Adults	Male	29	Y
5148	2021	Apr	GA	Adults	Female	65	Y
5149	2021	Apr	GA	Adults	Female	66	Y
5150	2021	Apr	FS	Adults	Female	25	Y
5151	2021	Apr	WC	Adults	Female	42	Y
5152	2021	Apr	WC	Adults	Female	42	Y
5153	2021	Apr	GA	Adults	Female	51	Y
5154	2021	Apr	GA	Adults	Female	51	Y
5155	2021	Apr	GA	Adults	Male	60	Y
5156	2021	Apr	GA	Unkown	Female		
5157	2021	Apr	KZ	Paeds	Male	9	D
5158	2021	Apr	GA	Paeds	Male	4	M
5159	2021	Apr	KZ	Adults	Male	20	Y
5160	2021	Apr	WC	Adults	Male	55	Y
5161	2021	Apr	GA	Unkown	Female		
5162	2021	May	GA	Paeds	Female	6	D
5163	2021	May	KZ	Adults	Male	48	Y
5164	2021	May	GA	Unkown	Male		
5165	2021	May	GA	Adults	Male	76	Y
5166	2021	May	WC	Adults	Female	38	Y
5167	2021	May	GA	Unkown	Male		
5168	2021	May	WC	Adults	Male	26	Y
5169	2021	May	KZ	Adults	Female	31	Y
5170	2021	May	WC	Adults	Female	59	Y
5171	2021	May	GA	Unkown	Female		
5172	2021	May	GA	Paeds	Female	30	D
5173	2021	May	GA	Paeds	Female	30	D
5174	2021	May	GA	Paeds	Female	28	M
5175	2021	May	GA	Paeds	Male	51	M
5176	2021	May	GA	Adults	Male	32	Y
5177	2021	May	GA	Adults	Female	62	Y
5178	2021	May	KZ	Adults	Male	42	Y
5179	2021	May	GA	Adults	Female	69	Y
5180	2021	May	KZ	Paeds	Male	14	Y
5181	2021	May	KZ	Adults	Male	23	Y
5182	2021	May	GA	Unkown	Female		
5183	2021	May	KZ	Adults	Female	23	Y
5184	2021	May	GA	Adults	Female	43	Y
5185	2021	May	GA	Adults	Female	43	Y

5186	2021	May	GA	Adults	Male	50	Y
5187	2021	May	GA	Adults	Male	50	Y
5188	2021	May	GA	Unkown	Male		
5189	2021	May	GA	Unkown	Female		
5190	2021	May	FS	Adults	Male	59	Y
5191	2021	May	KZ	Paeds	Male	0	D
5192	2021	May	KZ	Paeds	Male	0	D
5193	2021	May	KZ	Adults	Male	41	Y
5194	2021	May	KZ	Adults	Male	41	Y
5195	2021	May	FS	Adults	Female	49	Y
5196	2021	May	GA	Adults	Female	26	Y
5197	2021	May	KZ	Unkown	Female		
5198	2021	May	GA	Paeds	Female	3	D
5199	2021	May	GA	Unkown	Male		
5200	2021	May	FS	Paeds	Female	13	Y
5201	2021	May	KZ	Paeds	Male	10	Y
5202	2021	May	KZ	Adults	Male	43	Y
5203	2021	May	GA	Adults	Male	30	Y
5204	2021	May	GA	Adults	Female	35	Y
5205	2021	May	KZ	Paeds	Female	6	M
5206	2021	May	KZ	Adults	Female	27	Y
5207	2021	May	GA	Adults	Male	36	Y
5208	2021	May	GA	Unkown	Female		
5209	2021	May	GA	Unkown	Female		
5210	2021	May	FS	Adults	Female	64	Y
5211	2021	May	GA	Unkown	Male		
5212	2021	Jun	KZ	Paeds	Male	13	Y
5213	2021	Jun	KZ	Paeds	Male	13	Y
5214	2021	Jun	WC	Adults	Male	35	Y
5215	2021	Jun	GA	Adults	Female	35	Y
5216	2021	Jun	GA	Adults	Female	57	Y
5217	2021	Jun	FS	Adults	Female	30	Y
5218	2021	Jun	GA	Adults	Male	36	Y
5219	2021	Jun	FS	Adults	Female	41	Y
5220	2021	Jun	WC	Adults	Male	57	Y
5221	2021	Jun	GA	Adults	Female	71	Y
5222	2021	Jun	GA	Adults	Female	71	Y
5223	2021	Jun	KZ	Adults	Male	24	Y
5224	2021	Jun	KZ	Paeds	Female	1	M
5225	2021	Jun	GA	Adults	Female	26	Y
5226	2021	Jun	WC	Adults	Male	63	Y
5227	2021	Jun	WC	Adults	Male	63	Y
5228	2021	Jun	KZ	Paeds	Female	1	M
5229	2021	Jun	GA	Adults	Male	43	Y
5230	2021	Jun	FS	Paeds	Male	6	D
5231	2021	Jun	GA	Adults	Female	70	Y
5232	2021	Jun	GA	Adults	Female	70	Y
5233	2021	Jun	GA	Paeds	Female	2	M
5234	2021	Jun	KZ	Paeds	Female	2	M
5235	2021	Jun	KZ	Adults	Female	58	Y
5236	2021	Jun	GA	Adults	Male	38	Y
5237	2021	Jun	GA	Adults	Male	38	Y
5238	2021	Jun	GA	Adults	Female	69	Y
5239	2021	Jun	GA	Adults	Female	69	Y
5240	2021	Jun	GA	Adults	Female	69	Y
5241	2021	Jun	GA	Adults	Female	74	Y

5242	2021	Jun	GA	Adults	Female	35	Y
5243	2021	Jun	GA	Unkown	Female		
5244	2021	Jun	KZ	Adults	Male	59	Y
5245	2021	Jun	GA	Adults	Female	71	Y
5246	2021	Jun	GA	Adults	Female	71	Y
5247	2021	Jun	GA	Adults	Male	68	Y
5248	2021	Jun	GA	Paeds	Male	12	M
5249	2021	Jun	GA	Paeds	Male	12	M
5250	2021	Jun	KZ	Paeds	Male	42	M
5251	2021	Jun	KZ	Adults	Male	31	Y
5252	2021	Jun	GA	Adults	Female	49	Y
5253	2021	Jun	GA	Paeds	Female	15	D
5254	2021	Jun	GA	Paeds	Female	15	D
5255	2021	Jun	KZ	Adults	Female	29	Y
5256	2021	Jun	GA	Adults	Male	78	Y
5257	2021	Jun	GA	Adults	Male	78	Y
5258	2021	Jun	WC	Adults	Male	66	Y
5259	2021	Jun	KZ	Paeds	Female	1	M
5260	2021	Jun	GA	Paeds	Male	3	M
5261	2021	Jun	GA	Adults	Male	64	Y
5262	2021	Jun	FS	Adults	Female	40	Y
5263	2021	Jun	GA	Adults	Female	53	Y
5264	2021	Jun	GA	Unkown	Male		
5265	2021	Jun	GA	Adults	Female	35	Y
5266	2021	Jun	GA	Adults	Female	35	Y
5267	2021	Jun	GA	Adults	Female	50	Y
5268	2021	Jun	GA	Adults	Female	69	Y
5269	2021	Jun	GA	Adults	Female	69	Y
5270	2021	Nov	WC	Paeds	Male	16	M
5271	2021	Jun	GA	Unkown	Male		
5272	2021	Jun	KZ	Paeds	Male	2	M
5273	2021	Nov	WC	Adults	Male	37	Y
5274	2021	Jun	GA	Adults	Female	47	Y
5275	2021	Nov	WC	Paeds	Female	2	M
5276	2021	Jun	GA	Adults	Female	64	Y
5277	2021	Oct	GA	Adults	Female	62	Y
5278	2021	Oct	WC	Adults	Female	62	Y
5279	2021	Oct	GA	Paeds	Female	13	Y
5280	2021	Oct	GA	Adults	Male	69	Y
5281	2021	Oct	GA	Adults	Male	64	Y
5282	2021	Oct	WC	Paeds	Male	2	M
5283	2021	Oct	GA	Paeds	Male	5	M
5285	2021	Oct	GA	Adults	Male	73	Y
5286	2021	Oct	GA	Adults	Female	50	Y
5287	2021	Oct	GA	Paeds	Male	11	M
5288	2021	Oct	GA	Adults	Female	69	Y
5289	2021	Oct	GA	Paeds	Male	13	D
5290	2021	Oct	GA	Adults	Male	43	Y
5291	2021	Oct	GA	Paeds	Male	1	M
5292	2021	Oct	GA	Adults	Female	30	Y
5293	2021	Nov	WC	Paeds	Male	3	D
5294	2021	Oct	WC	Paeds	Male	6	D
5295	2021	Oct	GA	Paeds	Male	28	D
5296	2021	Nov	GA	Adults	Male	41	Y
5297	2021	Oct	GA	Adults	Male	57	Y
5298	2021	Oct	GA	Adults	Male	16	Y

5299	2021	Oct	GA	Adults	Male	46	Y
5300	2021	Oct	GA	Adults	Female	67	Y
5301	2021	Oct	GA	Paeds	Male	55	M
5302	2021	Nov	GA	Adults	Male	43	Y
5303	2021	Oct	GA	Adults	Male	46	Y
5304	2021	Nov	GA	Adults	Female	31	Y
5305	2021	Oct	GA	Paeds	Female	28	M
5306	2021	Oct	GA	Adults	Female	67	Y
5307	2021	Oct	GA	Adults	Male	37	Y
5308	2021	Nov	GA	Paeds	Female	11	Y
5309	2021	Oct	GA	Paeds	Female	20	M
5310	2021	Oct	GA	Unkown	Female		
5311	2021	Oct	GA	Adults	Male	61	Y
5312	2021	Nov	GA	Unkown	Male		
5313	2021	Oct	GA	Paeds	Male	2	Y
5314	2021	Nov	GA	Paeds	Male	2	Y
5315	2021	Oct	KZ	Adults	Male	46	Y
5316	2021	Nov	GA	Unkown	Female	2	Y
5318	2021	Nov	GA	Adults	Male	34	Y
5319	2021	Oct	GA	Adults	Male	60	Y
5320	2021	Nov	GA	Adults	Male	48	Y
5322	2021	Nov	KZ	Paeds	Female	4	M
5324	2021	Nov	GA	Adults	Male	50	Y
5325	2021	Nov	GA	Adults	Male	17	Y
5326	2021	Nov	GA	Adults	Female	51	Y
5327	2021	Nov	WC	Paeds	Female	4	M
5328	2021	Nov	WC	Adults	Male	34	Y
5329	2021	Nov	GA	Adults	Female	52	Y
5330	2021	Nov	GA	Adults	Male	42	Y
5331	2021	Nov	GA	Adults	Female	35	Y
5332	2021	Nov	GA	Adults	Male	50	Y
5333	2021	Nov	WC	Paeds	Male	14	M
5334	2021	Nov	WC	Paeds	Male	7	D
5335	2021	Nov	GA	Unkown	Male		
5336	2021	Nov	GA	Adults	Female	21	Y
5337	2021	Nov	GA	Paeds	Male	7	Y
5338	2021	Nov	GA	Adults	Male	32	Y
5339	2021	Nov	GA	Adults	Male	39	Y
5340	2021	Nov	GA	Adults	Female	34	Y
5341	2021	Nov	GA	Adults	Female	36	Y
5342	2021	Nov	KZ	Adults	Female	47	Y
5344	2021	Nov	GA	Adults	Female	48	Y
5345	2021	Nov	GA	Adults	Female	57	Y
5346	2021	Nov	GA	Adults	Female	43	Y
5348	2021	Nov	GA	Paeds	Female	11	D
5350	2021	Nov	GA	Adults	Male	42	Y
5351	2021	Nov	GA	Adults	Female	37	Y
5352	2021	Nov	GA	Adults	Male	58	Y
5353	2021	Nov	GA	Adults	Female	48	Y
5354	2021	Nov	GA	Paeds	Male	7	M
5355	2021	Nov	GA	Adults	Female	23	Y
5356	2021	Nov	GA	Adults	Male	51	Y
5357	2021	Nov	GA	Paeds	Female	2	M
5358	2021	Nov	GA	Adults	Male	74	Y
5359	2021	Nov	GA	Adults	Female	28	Y
5360	2021	Oct	GA	Adults	Female	38	Y

5361	2021	Nov	GA	Adults	Male	28	Y
5362	2021	Nov	GA	Adults	Male	75	Y
5363	2021	Nov	GA	Adults	Male	32	Y
5364	2021	Nov	GA	Adults	Female	16	Y
5365	2021	Nov	GA	Adults	Male	33	Y
5366	2021	Dec	KZ	Paeds	Male	5	M
5367	2021	Nov	GA	Adults	Female	32	Y
5368	2021	Nov	GA	Unkown	Female		
5369	2021	Nov	GA	Paeds	Unknown	10	Y
5370	2021	Nov	GA	Adults	Male	48	Y
5371	2021	Nov	GA	Unkown	Female		
5373	2021	Nov	GA	Adults	Female	49	Y
5374	2021	Nov	GA	Adults	Male	23	Y
5375	2021	Nov	GA	Paeds	Female	28	M
5376	2021	Nov	GA	Paeds	Female	1	M
5377	2021	Nov	GA	Unkown	Female		
5378	2021	Nov	GA	Adults	Female	78	Y
5379	2021	Nov	GA	Paeds	Female	11	D
5380	2021	Dec	GA	Paeds	Female	16	D
5381	2021	Dec	GA	Paeds	Male	11	M
5382	2021	Dec	GA	Adults	Female	51	Y
5383	2021	Dec	GA	Adults	Female	19	Y
5384	2021	Dec	GA	Adults	Female	37	Y
5385	2021	Nov	GA	Paeds	Male	15	M
5386	2021	Nov	GA	Adults	Male	40	Y
5387	2021	Nov	GA	Paeds	Male	3	D
5388	2021	Dec	WC	Adults	Female	71	Y
5389	2021	Dec	GA	Adults	Female	18	Y
5390	2021	Dec	WC	Paeds	Female	6	D
5391	2021	Dec	WC	Adults	Male	18	Y
5392	2021	Dec	WC	Paeds	Male	21	D
5393	2021	Dec	WC	Paeds	Male	21	D
5394	2021	Dec	WC	Paeds	Female	11	D
5395	2021	Dec	WC	Paeds	Female	9	M
5396	2021	Dec	GA	Adults	Male	25	Y
5397	2021	Dec	GA	Paeds	Female	7	M
5398	2021	Dec	GA	Adults	Female	30	Y
5399	2021	Dec	GA	Adults	Male	72	Y
5400	2021	Dec	GA	Adults	Female	45	Y
5402	2021	Dec	GA	Adults	Male	33	Y
5403	2021	Dec	GA	Adults	Male	29	Y
5404	2021	Dec	GA	Paeds	Female	4	D
5405	2021	Dec	GA	Paeds	Male	2	M
5406	2021	Dec	GA	Paeds	Male	2	M
5407	2021	Dec	GA	Paeds	Male	2	M
5408	2021	Dec	GA	Paeds	Female	15	Y
5409	2021	Dec	GA	Adults	Female	34	Y
5410	2021	Dec	GA	Unkown	Male		
5411	2021	Dec	GA	Paeds	Male	4	D
5412	2021	Dec	GA	Adults	Male	47	Y
5413	2021	Dec	GA	Unkown	Male		
5415	2021	Dec	GA	Adults	Male	50	Y
5416	2021	Dec	GA	Adults	Male	57	Y
5417	2021	Dec	GA	Paeds	Male	2	Y
5418	2021	Dec	GA	Adults	Female	70	Y
5419	2021	Dec	GA	Paeds	Female	5	D

5420	2021	Dec	GA	Paeds	Female	1	Y
5421	2021	Dec	GA	Adults	Female	32	Y
5422	2021	Dec	GA	Unkown	Male		
5423	2021	Dec	WC	Paeds	Female	20	M
5424	2021	Dec	GA	Paeds	Female	14	D
5425	2021	Dec	GA	Paeds	Female	4	D
5426	2021	Dec	GA	Paeds	Male	6	D
5427	2021	Dec	GA	Paeds	Male	26	D
5428	2021	Dec	GA	Adults	Male	35	Y
5429	2021	Dec	GA	Adults	Female	71	Y
5430	2021	Dec	GA	Paeds	Male	14	D
5431	2021	Dec	GA	Paeds	Female	22	D
5432	2021	Dec	GA	Adults	Male	29	Y
5433	2021	Dec	GA	Adults	Male	61	Y
5434	2021	Dec	WC	Adults	Male	52	Y
5435	2021	Dec	WC	Adults	Male	52	Y
5436	2021	Dec	KZ	Adults	Female	43	Y
5437	2021	Dec	GA	Adults	Male	16	Y
5438	2021	Dec	GA	Paeds	Male	20	D
5439	2021	Dec	GA	Adults	Female	68	Y
5440	2021	Dec	GA	Adults	Male	62	Y
5441	2021	Dec	GA	Paeds	Male	22	D
5442	2021	Dec	GA	Paeds	Male	8	D
5443	2021	Dec	GA	Adults	Female	79	Y
5444	2021	Dec	GA	Adults	Female	71	Y
5445	2021	Dec	GA	Paeds	Male	10	D
5446	2021	Dec	GA	Adults	Female	68	Y
5447	2021	Dec	GA	Adults	Male	61	Y
5453	2021	Jun	GA	Paeds	Male	0	D
5454	2021	Aug	GA	Paeds	Female	0	D
5455	2021	Aug	KZ	Paeds	Female	0	D
5456	2021	Aug	GA	Paeds	Unknown	0	D
5457	2021	Sep	GA	Paeds	Male	0	D
5458	2021	Sep	GA	Paeds	Male	0	D
5459	2021	Dec	GA	Paeds	Male	0	D
5460	2021	Oct	GA	Paeds	Male	2	D
5461	2021	Jul	KZ	Paeds	Female	3	D
5462	2021	Jan	KZ	Paeds	Male	4	D
5463	2021	Oct	KZ	Paeds	Male	4	D
5464	2021	Oct	KZ	Paeds	Male	4	D
5465	2021	Oct	GA	Paeds	Male	5	D
5466	2021	Jul	GA	Paeds	Female	6	D
5467	2021	Aug	KZ	Paeds	Female	6	D
5468	2021	Aug	KZ	Paeds	Male	6	D
5469	2021	Sep	GA	Paeds	Female	6	D
5470	2021	Dec	FS	Paeds	Male	6	D
5471	2021	Sep	GA	Paeds	Male	7	D
5472	2021	Sep	GA	Paeds	Female	7	D
5473	2021	Nov	GA	Paeds	Male	7	D
5474	2021	Aug	GA	Paeds	Male	8	D
5475	2021	Sep	GA	Paeds	Female	8	D
5477	2021	Oct	GA	Paeds	Female	9	D
5478	2021	Nov	FS	Paeds	Male	9	D
5479	2021	Nov	GA	Paeds	Male	10	D
5480	2021	Oct	GA	Paeds	Female	11	D
5481	2021	Nov	KZ	Paeds	Unknown	11	D

5482	2021	Aug	GA	Paeds	Male	12	D
5483	2021	May	KZ	Paeds	Male	14	D
5484	2021	Nov	FS	Paeds	Male	15	D
5485	2021	Jul	KZ	Paeds	Female	16	D
5486	2021	Jul	GA	Paeds	Male	17	D
5487	2021	Dec	GA	Paeds	Female	18	D
5488	2021	Jul	KZ	Paeds	Female	19	D
5489	2021	Nov	GA	Paeds	Male	20	D
5491	2021	Dec	KZ	Paeds	Unknown	20	D
5493	2021	Oct	FS	Paeds	Female	24	D
5494	2021	Dec	GA	Paeds	Male	24	D
5495	2021	Aug	GA	Paeds	Female	25	D
5496	2021	Jan	KZ	Paeds	Female	27	D
5497	2021	Oct	GA	Paeds	Male	27	D
5498	2021	Oct	GA	Paeds	Unknown	29	D
5499	2021	Oct	GA	Paeds	Male	1	M
5500	2021	Jul	KZ	Paeds	Female	1	M
5501	2021	Aug	GA	Paeds	Female	1	M
5502	2021	Aug	GA	Paeds	Male	1	M
5503	2021	Nov	GA	Paeds	Male	1	M
5504	2021	Sep	KZ	Paeds	Unknown	1	M
5505	2021	Aug	KZ	Paeds	Female	1	M
5506	2021	Dec	GA	Paeds	Female	1	M
5507	2021	Nov	GA	Paeds	Male	1	M
5508	2021	Sep	KZ	Paeds	Male	1	M
5509	2021	Oct	GA	Paeds	Male	1	M
5510	2021	Oct	GA	Paeds	Male	1	M
5511	2021	Dec	FS	Paeds	Male	1	M
5512	2021	Dec	GA	Paeds	Male	1	M
5513	2021	Sep	GA	Paeds	Male	1	M
5514	2021	Aug	GA	Paeds	Male	1	M
5515	2021	Dec	GA	Paeds	Female	1	M
5516	2021	Dec	GA	Paeds	Female	1	M
5517	2021	Dec	GA	Paeds	Female	1	M
5518	2021	Nov	GA	Paeds	Male	1	M
5519	2021	Sep	FS	Paeds	Female	1	M
5520	2021	Jul	GA	Paeds	Male	2	M
5521	2021	Nov	GA	Paeds	Male	2	M
5522	2021	Dec	GA	Paeds	Male	2	M
5523	2021	Nov	GA	Paeds	Female	2	M
5524	2021	Dec	GA	Paeds	Male	2	M
5525	2021	Oct	WC	Paeds	Female	3	M
5526	2021	Jul	GA	Paeds	Female	3	M
5527	2021	Oct	GA	Paeds	Male	3	M
5528	2021	Dec	GA	Paeds	Male	3	M
5529	2021	Sep	GA	Paeds	Unknown	3	M
5530	2021	Jun	GA	Paeds	Male	3	M
5531	2021	Jul	KZ	Paeds	Male	3	M
5532	2021	Nov	FS	Paeds	Male	3	M
5533	2021	Sep	FS	Paeds	Male	3	M
5534	2021	Jul	GA	Paeds	Female	3	M
5535	2021	Jul	GA	Paeds	Female	3	M
5536	2021	Aug	KZ	Paeds	Female	4	M
5537	2021	Jun	WC	Paeds	Female	4	M
5538	2021	Jan	GA	Paeds	Male	7	M
5539	2021	Jul	GA	Paeds	Male	7	M

5540	2021	Feb	KZ	Paeds	Female	8	M
5541	2021	Feb	GA	Paeds	Female	9	M
5542	2021	Jul	GA	Paeds	Male	10	M
5543	2021	Nov	KZ	Paeds	Male	11	M
5544	2021	Aug	KZ	Paeds	Female	14	M
5545	2021	Dec	GA	Paeds	Male	16	M
5546	2021	Nov	KZ	Paeds	Female	17	M
5547	2021	Feb	GA	Paeds	Female	18	M
5548	2021	Nov	GA	Paeds	Female	21	M
5549	2021	Nov	GA	Paeds	Male	23	M
5550	2021	Dec	GA	Paeds	Male	24	M
5553	2021	Feb	GA	Paeds	Female	28	M
5554	2021	Dec	GA	Paeds	Male	29	M
5555	2021	Dec	GA	Paeds	Female	32	M
5556	2021	Nov	GA	Paeds	Male	36	M
5557	2021	Dec	KZ	Paeds	Female	38	M
5558	2021	Oct	KZ	Paeds	Male	41	M
5560	2021	Jul	GA	Paeds	Male	46	M
5561	2021	Dec	KZ	Paeds	Female	58	M
5562	2021	Jul	KZ	Paeds	Male	5	Y
5563	2021	Dec	GA	Paeds	Female	6	Y
5564	2021	Sep	KZ	Paeds	Male	6	Y
5566	2021	Mar	WC	Paeds	Male	9	Y
5567	2021	Oct	KZ	Paeds	Male	9	Y
5569	2021	Nov	GA	Paeds	Male	10	Y
5570	2021	Oct	GA	Paeds	Male	10	Y
5572	2021	May	KZ	Paeds	Female	11	Y
5573	2021	Aug	KZ	Paeds	Female	12	Y
5574	2021	Jun	KZ	Paeds	Female	12	Y
5575	2021	Jul	GA	Paeds	Male	15	Y
5576	2021	Oct	GA	Adults	Female	17	Y
5577	2021	Aug	GA	Adults	Female	18	Y
5578	2021	Dec	WC	Adults	Male	18	Y
5579	2021	Dec	KZ	Adults	Male	19	Y
5580	2021	Dec	KZ	Adults	Male	19	Y
5581	2021	Dec	GA	Adults	Female	20	Y
5582	2021	Aug	KZ	Adults	Female	20	Y
5583	2021	Mar	FS	Adults	Male	20	Y
5584	2021	Dec	KZ	Adults	Male	21	Y
5585	2021	Dec	KZ	Adults	Male	21	Y
5586	2021	Dec	FS	Adults	Male	21	Y
5587	2021	Dec	GA	Adults	Male	21	Y
5588	2021	Jun	GA	Adults	Female	22	Y
5589	2021	Feb	GA	Adults	Female	22	Y
5590	2021	May	KZ	Adults	Female	23	Y
5591	2021	Dec	KZ	Adults	Female	23	Y
5592	2021	Jan	KZ	Adults	Female	23	Y
5593	2021	Jul	GA	Adults	Unknown	24	Y
5594	2021	Sep	GA	Adults	Male	24	Y
5595	2021	Feb	GA	Adults	Female	24	Y
5596	2021	Sep	GA	Adults	Male	25	Y
5598	2021	Aug	KZ	Adults	Male	25	Y
5599	2021	Aug	KZ	Adults	Male	25	Y
5600	2021	Jul	KZ	Adults	Female	25	Y
5601	2021	Sep	GA	Adults	Male	25	Y
5602	2021	Aug	GA	Adults	Female	25	Y

5603	2021	Oct	KZ	Adults	Female	26	Y
5604	2021	Feb	GA	Adults	Female	26	Y
5605	2021	Sep	KZ	Adults	Male	26	Y
5606	2021	Dec	GA	Adults	Male	26	Y
5607	2021	Sep	KZ	Adults	Male	28	Y
5608	2021	Nov	GA	Adults	Female	28	Y
5609	2021	Jul	WC	Adults	Female	28	Y
5610	2021	Nov	KZ	Adults	Male	29	Y
5611	2021	Jul	GA	Adults	Male	29	Y
5612	2021	Mar	KZ	Adults	Male	30	Y
5613	2021	Aug	GA	Adults	Male	30	Y
5614	2021	Oct	KZ	Adults	Female	30	Y
5615	2021	Feb	WC	Adults	Male	31	Y
5616	2021	Jul	KZ	Adults	Female	31	Y
5617	2021	Nov	GA	Adults	Female	31	Y
5618	2021	Sep	KZ	Adults	Male	31	Y
5619	2021	Nov	GA	Adults	Female	32	Y
5620	2021	Feb	GA	Adults	Male	32	Y
5621	2021	Aug	GA	Adults	Male	32	Y
5622	2021	Nov	WC	Adults	Male	33	Y
5623	2021	Mar	FS	Adults	Male	33	Y
5624	2021	Aug	GA	Adults	Male	33	Y
5625	2021	Dec	GA	Adults	Male	33	Y
5626	2021	Aug	GA	Adults	Female	34	Y
5627	2021	Oct	KZ	Adults	Male	34	Y
5628	2021	Sep	GA	Adults	Male	34	Y
5629	2021	Aug	KZ	Adults	Male	34	Y
5630	2021	Feb	GA	Adults	Female	34	Y
5631	2021	Nov	KZ	Adults	Female	34	Y
5632	2021	Sep	KZ	Adults	Male	34	Y
5633	2021	Aug	KZ	Adults	Male	34	Y
5634	2021	Nov	GA	Adults	Male	34	Y
5635	2021	Aug	GA	Adults	Male	35	Y
5636	2021	Oct	GA	Adults	Male	35	Y
5637	2021	Jul	KZ	Adults	Female	35	Y
5638	2021	Dec	KZ	Adults	Male	35	Y
5639	2021	Jul	GA	Adults	Male	36	Y
5640	2021	Dec	GA	Adults	Male	36	Y
5641	2021	Dec	WC	Adults	Female	36	Y
5642	2021	Dec	KZ	Adults	Female	37	Y
5643	2021	Oct	WC	Adults	Female	37	Y
5644	2021	Jun	KZ	Adults	Female	38	Y
5645	2021	Dec	GA	Adults	Male	38	Y
5646	2021	Sep	GA	Adults	Male	38	Y
5647	2021	Nov	WC	Adults	Male	38	Y
5648	2021	Oct	KZ	Adults	Male	39	Y
5649	2021	Jan	WC	Adults	Female	39	Y
5650	2021	Nov	WC	Adults	Male	39	Y
5651	2021	Oct	KZ	Adults	Male	39	Y
5652	2021	Jul	GA	Adults	Female	40	Y
5653	2021	Aug	GA	Adults	Male	40	Y
5654	2021	Sep	WC	Adults	Male	40	Y
5655	2021	Aug	GA	Adults	Male	40	Y
5656	2021	Nov	GA	Adults	Male	40	Y
5657	2021	Nov	GA	Adults	Male	40	Y
5658	2021	Oct	WC	Adults	Male	40	Y

5659	2021	Jul	GA	Adults	Female	41	Y
5660	2021	Jun	GA	Adults	Female	41	Y
5661	2021	Aug	GA	Adults	Male	41	Y
5662	2021	Nov	GA	Adults	Male	41	Y
5663	2021	Nov	FS	Adults	Female	41	Y
5664	2021	Nov	GA	Adults	Female	41	Y
5665	2021	Nov	FS	Adults	Male	42	Y
5666	2021	Jul	KZ	Adults	Unknown	42	Y
5667	2021	Dec	GA	Adults	Male	42	Y
5668	2021	Aug	WC	Adults	Female	42	Y
5669	2021	Dec	GA	Adults	Male	42	Y
5670	2021	Aug	GA	Adults	Female	42	Y
5671	2021	Aug	GA	Adults	Male	42	Y
5672	2021	Sep	GA	Adults	Male	42	Y
5673	2021	Dec	GA	Adults	Male	42	Y
5674	2021	Jul	GA	Adults	Male	43	Y
5675	2021	Nov	GA	Adults	Female	43	Y
5676	2021	Aug	WC	Adults	Male	43	Y
5677	2021	Nov	GA	Adults	Male	44	Y
5678	2021	Aug	GA	Adults	Male	44	Y
5679	2021	Jan	WC	Adults	Male	44	Y
5680	2021	Oct	KZ	Adults	Female	45	Y
5681	2021	Aug	GA	Adults	Male	45	Y
5682	2021	Sep	GA	Adults	Female	45	Y
5683	2021	Nov	GA	Adults	Male	45	Y
5684	2021	Aug	GA	Adults	Female	45	Y
5685	2021	Sep	GA	Adults	Male	45	Y
5686	2021	Aug	GA	Adults	Female	45	Y
5687	2021	Aug	GA	Adults	Male	45	Y
5688	2021	Nov	GA	Adults	Female	46	Y
5689	2021	Jan	GA	Adults	Male	46	Y
5690	2021	Aug	KZ	Adults	Male	46	Y
5691	2021	Dec	KZ	Adults	Male	46	Y
5692	2021	Oct	GA	Adults	Female	46	Y
5693	2021	Sep	GA	Adults	Female	47	Y
5694	2021	Jun	GA	Adults	Female	47	Y
5695	2021	Oct	KZ	Adults	Female	47	Y
5696	2021	Jun	GA	Adults	Male	47	Y
5697	2021	Oct	KZ	Adults	Male	47	Y
5698	2021	Oct	KZ	Adults	Male	47	Y
5699	2021	Aug	GA	Adults	Male	47	Y
5700	2021	Jul	GA	Adults	Female	48	Y
5701	2021	Oct	KZ	Adults	Male	48	Y
5702	2021	Aug	WC	Adults	Male	48	Y
5703	2021	Aug	WC	Adults	Male	48	Y
5704	2021	Aug	GA	Adults	Male	49	Y
5705	2021	Dec	KZ	Adults	Male	49	Y
5706	2021	Dec	GA	Adults	Male	50	Y
5707	2021	Dec	GA	Adults	Male	50	Y
5708	2021	Jul	GA	Adults	Male	50	Y
5709	2021	Jul	GA	Adults	Female	50	Y
5710	2021	Jul	WC	Adults	Male	50	Y
5711	2021	Jul	GA	Adults	Male	51	Y
5712	2021	Oct	GA	Adults	Female	51	Y
5713	2021	Oct	GA	Adults	Female	51	Y
5714	2021	Nov	GA	Adults	Female	51	Y

5715	2021	Jul	WC	Adults	Female	51	Y
5716	2021	Nov	GA	Adults	Female	51	Y
5717	2021	Nov	GA	Adults	Female	52	Y
5718	2021	Jan	GA	Adults	Female	52	Y
5719	2021	Dec	KZ	Adults	Female	52	Y
5720	2021	Feb	GA	Adults	Male	52	Y
5721	2021	Jan	WC	Adults	Male	52	Y
5722	2021	Nov	GA	Adults	Female	52	Y
5723	2021	Dec	WC	Adults	Female	52	Y
5724	2021	Feb	KZ	Adults	Female	53	Y
5725	2021	Sep	GA	Adults	Male	53	Y
5726	2021	Sep	GA	Adults	Female	53	Y
5727	2021	Nov	GA	Adults	Female	54	Y
5728	2021	Nov	GA	Adults	Male	54	Y
5729	2021	Nov	GA	Adults	Male	54	Y
5730	2021	Nov	GA	Adults	Male	54	Y
5731	2021	Jul	GA	Adults	Female	55	Y
5732	2021	Dec	FS	Adults	Male	55	Y
5733	2021	Dec	GA	Adults	Female	55	Y
5734	2021	Dec	GA	Adults	Female	55	Y
5735	2021	Sep	GA	Adults	Male	55	Y
5736	2021	Jun	KZ	Adults	Female	55	Y
5737	2021	Jul	KZ	Adults	Female	55	Y
5738	2021	Sep	GA	Adults	Female	56	Y
5739	2021	Jan	GA	Adults	Male	56	Y
5740	2021	Oct	KZ	Adults	Female	56	Y
5741	2021	Dec	KZ	Adults	Male	56	Y
5742	2021	Jan	KZ	Adults	Female	56	Y
5743	2021	Oct	WC	Adults	Male	56	Y
5744	2021	Oct	GA	Adults	Female	56	Y
5745	2021	Jul	GA	Adults	Male	56	Y
5746	2021	Dec	KZ	Adults	Male	56	Y
5747	2021	Dec	GA	Adults	Male	56	Y
5748	2021	Oct	KZ	Adults	Male	56	Y
5749	2021	Jan	GA	Adults	Male	57	Y
5750	2021	Sep	KZ	Adults	Female	57	Y
5751	2021	Dec	GA	Adults	Male	57	Y
5752	2021	Jun	GA	Adults	Male	57	Y
5753	2021	Aug	GA	Adults	Female	57	Y
5754	2021	Sep	GA	Adults	Female	57	Y
5755	2021	Jul	WC	Adults	Male	58	Y
5756	2021	Aug	GA	Adults	Male	58	Y
5757	2021	Feb	WC	Adults	Male	58	Y
5758	2021	Oct	KZ	Adults	Male	58	Y
5759	2021	Aug	GA	Adults	Male	59	Y
5762	2021	Jul	GA	Adults	Female	59	Y
5763	2021	Aug	WC	Adults	Female	59	Y
5764	2021	Nov	FS	Adults	Female	59	Y
5765	2021	Jul	GA	Adults	Female	59	Y
5766	2021	Oct	KZ	Adults	Male	59	Y
5767	2021	Nov	GA	Adults	Female	60	Y
5768	2021	Nov	WC	Adults	Female	60	Y
5769	2021	Feb	GA	Adults	Male	60	Y
5770	2021	Dec	KZ	Adults	Male	60	Y
5771	2021	Jan	KZ	Adults	Female	61	Y
5773	2021	Oct	WC	Adults	Male	61	Y

5774	2021	Sep	GA	Adults	Male	61	Y
5775	2021	Feb	WC	Adults	Male	62	Y
5776	2021	Mar	GA	Adults	Female	62	Y
5777	2021	Feb	GA	Adults	Female	62	Y
5778	2021	May	GA	Adults	Female	62	Y
5779	2021	Mar	GA	Adults	Female	62	Y
5780	2021	Mar	GA	Adults	Male	63	Y
5781	2021	Jul	GA	Adults	Male	63	Y
5782	2021	Aug	GA	Adults	Male	64	Y
5783	2021	Dec	GA	Adults	Female	64	Y
5784	2021	Dec	GA	Adults	Female	64	Y
5785	2021	Dec	GA	Adults	Male	64	Y
5786	2021	Sep	GA	Adults	Female	64	Y
5787	2021	Dec	GA	Adults	Male	64	Y
5788	2021	Dec	GA	Adults	Female	64	Y
5789	2021	Aug	GA	Adults	Male	64	Y
5790	2021	Oct	GA	Adults	Male	64	Y
5791	2021	Sep	GA	Adults	Male	64	Y
5792	2021	Nov	GA	Adults	Female	64	Y
5793	2021	Oct	KZ	Adults	Female	64	Y
5794	2021	Aug	GA	Adults	Female	64	Y
5795	2021	Jul	GA	Adults	Male	64	Y
5796	2021	Jun	GA	Adults	Male	65	Y
5797	2021	Aug	GA	Adults	Female	65	Y
5798	2021	Oct	KZ	Adults	Female	65	Y
5799	2021	Aug	GA	Adults	Female	66	Y
5800	2021	Aug	GA	Adults	Male	66	Y
5801	2021	Sep	KZ	Adults	Male	66	Y
5802	2021	Dec	GA	Adults	Female	66	Y
5803	2021	Sep	GA	Adults	Female	66	Y
5804	2021	Nov	FS	Adults	Female	67	Y
5805	2021	Sep	GA	Adults	Male	67	Y
5806	2021	Nov	GA	Adults	Male	67	Y
5808	2021	Feb	GA	Adults	Male	68	Y
5809	2021	Jul	GA	Adults	Female	68	Y
5810	2021	Jul	GA	Adults	Female	68	Y
5811	2021	Sep	GA	Adults	Female	68	Y
5812	2021	Apr	GA	Adults	Female	68	Y
5813	2021	Sep	GA	Adults	Female	68	Y
5814	2021	Dec	WC	Adults	Female	69	Y
5815	2021	Sep	WC	Adults	Male	70	Y
5816	2021	Sep	GA	Adults	Female	70	Y
5817	2021	Nov	GA	Adults	Female	70	Y
5818	2021	Mar	WC	Adults	Male	71	Y
5819	2021	Jul	GA	Adults	Male	71	Y
5820	2021	Sep	GA	Adults	Male	71	Y
5821	2021	Oct	GA	Adults	Male	73	Y
5822	2021	Aug	WC	Adults	Male	73	Y
5823	2021	Oct	GA	Adults	Male	74	Y
5824	2021	Feb	WC	Adults	Male	76	Y
5825	2021	Aug	GA	Adults	Female	83	Y
5826	2021	Sep	WC	Adults	Female	84	Y
5827	2021	Mar	KZ	Adults	Male	86	Y
5828	2021	Dec	GA	Adults	Male	88	Y
5829	2021	Sep	WC	Adults	Male	89	Y
5830	2021	Mar	GA	Adults	Female	90	Y

5831	2021	Sep	GA	Unkown	Male
5832	2021	Jul	GA	Unkown	Male
5833	2021	Aug	GA	Unkown	Male
5834	2021	Oct	GA	Unkown	Male
5835	2021	Nov	GA	Unkown	Male
5836	2021	Dec	GA	Unkown	Male
5837	2021	Jul	GA	Unkown	Male
5838	2021	Feb	GA	Unkown	Male
5839	2021	Mar	GA	Unkown	Male
5840	2021	Mar	GA	Unkown	Male
5842	2021	Mar	GA	Unkown	Female
5843	2021	Jun	GA	Unkown	Male
5844	2021	Jul	GA	Unkown	Female
5845	2021	Jul	GA	Unkown	Female
5847	2021	Aug	GA	Unkown	Female
5848	2021	Aug	GA	Unkown	Female
5849	2021	Sep	GA	Unkown	Male
5850	2021	Sep	GA	Unkown	Female
5851	2021	Oct	GA	Unkown	Female
5852	2021	Oct	GA	Unkown	Female
5853	2021	Oct	GA	Unkown	Male
5854	2021	Nov	GA	Unkown	Male
5855	2021	Dec	GA	Unkown	Male
5857	2021	Dec	GA	Unkown	Female
5858	2021	Dec	GA	Unkown	Female
5860	2021	Jan	GA	Unkown	Male
5861	2021	Feb	GA	Unkown	Male
5862	2021	Mar	GA	Unkown	Male
5863	2021	Apr	GA	Unkown	Male
5864	2021	May	GA	Unkown	Male
5865	2021	Jul	GA	Unkown	Female
5866	2021	Jul	GA	Unkown	Male
5867	2021	Sep	GA	Unkown	Male
5868	2021	Sep	GA	Unkown	Female
5869	2021	Oct	GA	Unkown	Female
5870	2021	Oct	GA	Unkown	Male
5871	2021	Oct	GA	Unkown	Male
5872	2021	Oct	GA	Unkown	Female
5873	2021	Nov	GA	Unkown	Male
5874	2021	Dec	GA	Unkown	Female
5875	2021	Dec	GA	Unkown	Male
5876	2021	Dec	GA	Unkown	Male
5877	2021	Jul	KZ	Unkown	Female
5878	2021	Jul	KZ	Unkown	Male
5879	2021	Aug	KZ	Unkown	Female
5880	2021	Sep	KZ	Unkown	Male
5881	2021	Sep	KZ	Unkown	Male
5882	2021	Sep	KZ	Unkown	Male
5883	2021	Aug	FS	Unkown	Male
5884	2021	Oct	KZ	Unkown	Male
5885	2021	Sep	KZ	Unkown	Male

organism	specimen collected	viability	antibiotic
Serratia marcescens	Blood culture	V	
Escherichia coli	Blood culture	V	Y
Klebsiella pneumoniae	Blood culture	V	Y
Klebsiella pneumoniae	Blood culture	V	Y
Klebsiella pneumoniae	Blood culture	V	Y
Klebsiella pneumoniae	Blood culture	V	Y
Klebsiella pneumoniae	Blood culture	V	U
Klebsiella oxytoca	Blood culture	V	Y
Klebsiella pneumoniae	Blood culture	V	Y
Klebsiella pneumoniae	Blood culture	V	Y
Klebsiella pneumoniae	Blood culture	V	
Klebsiella pneumoniae	Blood culture	V	
Klebsiella pneumoniae	Blood culture	V	
Klebsiella pneumoniae	Blood culture	V	Y
Escherichia coli	Blood culture	V	Y
Klebsiella pneumoniae	Blood culture	V	Y
Enterobacter cloacae	Blood culture	V	Y
Klebsiella pneumoniae	Blood culture	V	Y
Klebsiella pneumoniae	Blood culture	V	Y
Klebsiella pneumoniae	Blood culture	V	Y
Klebsiella pneumoniae	Blood culture	V	Y
Klebsiella pneumoniae	Blood culture	V	Y
Klebsiella pneumoniae	Blood culture	V	Y
Klebsiella pneumoniae	Blood culture	V	Y
Klebsiella pneumoniae	Blood culture	V	Y
Enterobacter cloacae	Blood culture	V	Y
Klebsiella pneumoniae	Blood culture	V	Y
Klebsiella pneumoniae	Blood culture	V	Y
Klebsiella pneumoniae	Blood culture	V	Y
Klebsiella pneumoniae	Blood culture	V	Y
Enterobacter aerogenes	Blood culture	V	Y
Klebsiella pneumoniae	Blood culture	V	Y
Klebsiella pneumoniae	Blood culture	V	Y
Klebsiella pneumoniae	Blood culture	V	
Escherichia coli	Blood culture	M	Y
Enterobacter cloacae	Blood culture	M	Y
Klebsiella pneumoniae	Blood culture	M	Y
Klebsiella pneumoniae	Blood culture	M	Y
Klebsiella pneumoniae	Blood culture	V	Y
Klebsiella pneumoniae	Blood culture	V	Y
Klebsiella pneumoniae	Blood culture	V	Y
Klebsiella oxytoca	Blood culture	V	Y
Klebsiella pneumoniae	Blood culture	V	Y
Klebsiella pneumoniae	Blood culture	V	Y
Klebsiella pneumoniae	Blood culture	V	Y
Klebsiella pneumoniae	Blood culture	V	Y
Klebsiella oxytoca	Blood culture	V	Y
Klebsiella pneumoniae	Blood culture	V	Y
Klebsiella pneumoniae	Blood culture	V	Y
Escherichia coli	Blood culture	V	Y
Klebsiella pneumoniae	Blood culture	V	Y
Klebsiella pneumoniae	Blood culture	V	Y
Enterobacter cloacae	Blood culture	V	Y
Klebsiella pneumoniae	Blood culture	V	Y
Klebsiella pneumoniae	Blood culture	V	Y

Klebsiella pneumoniae	Blood culture	V	Y
Serratia marcescens	Blood culture	V	Y
Klebsiella pneumoniae	Blood culture	V	Y
Klebsiella pneumoniae	Blood culture	V	Y
Klebsiella pneumoniae	Blood culture	V	Y
Klebsiella pneumoniae	Blood culture	V	U
Klebsiella pneumoniae	Blood culture	V	Y
Klebsiella pneumoniae	Blood culture	V	Y
Serratia marcescens	Blood culture	V	Y
Klebsiella pneumoniae	Blood culture	V	Y
Klebsiella pneumoniae	Blood culture	V	Y
Escherichia coli	Blood culture	V	Y
Klebsiella pneumoniae	Blood culture	V	Y
Providencia rettgeri	Blood culture	N	Y
Klebsiella pneumoniae	Blood culture	V	Y
Enterobacter cloacae complex	Blood culture	V	Y
Klebsiella pneumoniae	Blood culture	V	Y
Klebsiella pneumoniae	Blood culture	V	Y
Klebsiella pneumoniae	Blood culture	V	Y
Klebsiella pneumoniae	Blood culture	V	Y
Klebsiella pneumoniae	Blood culture	V	Y
Klebsiella pneumoniae	Blood culture	V	Y
Klebsiella pneumoniae	Blood culture	V	Y
Klebsiella pneumoniae	Blood culture	V	U
Klebsiella pneumoniae	Blood culture	V	Y
Klebsiella pneumoniae	Blood culture	V	Y
Klebsiella pneumoniae	Blood culture	V	Y
Klebsiella pneumoniae	Blood culture	V	Y
Klebsiella pneumoniae	Blood culture	V	
Enterobacter cloacae	Blood culture	V	Y
Klebsiella pneumoniae	Blood culture	V	Y
Escherichia coli	Blood culture	V	
Klebsiella pneumoniae	Blood culture	V	Y
Morganella morganii	Blood culture	M	Y
Morganella morganii	Blood culture	M	Y
Enterobacter aerogenes	Blood culture	M	Y
Klebsiella pneumoniae	Blood culture	M	Y
Escherichia coli	Blood culture	M	Y
Escherichia coli	Blood culture	M	Y
Klebsiella pneumoniae	Blood culture	M	Y
Enterobacter cloacae	Blood culture	M	Y
Klebsiella pneumoniae	Blood culture	M	Y
Klebsiella pneumoniae	Blood culture	M	Y
Enterobacter cloacae	Blood culture	M	Y
Klebsiella pneumoniae	Blood culture	M	Y
Enterobacter cloacae	Blood culture	M	Y
Klebsiella pneumoniae	Blood culture	M	Y
Enterobacter cloacae	Blood culture	M	Y
Klebsiella oxytoca	Blood culture	M	Y
Klebsiella pneumoniae	Blood culture	M	Y
Klebsiella pneumoniae	Blood culture	V	Y
Klebsiella oxytoca	Blood culture	V	Y
Klebsiella pneumoniae	Blood culture	M	Y
Klebsiella pneumoniae	Blood culture	M	
Klebsiella pneumoniae	Blood culture	M	
Klebsiella pneumoniae	Blood culture	M	Y

Enterobacter cloacae	Blood culture	M	Y
Escherichia coli	Blood culture	M	Y
Klebsiella pneumoniae	Blood culture	M	U
Klebsiella pneumoniae	Blood culture	M	Y
Klebsiella pneumoniae	Blood culture	M	Y
Klebsiella pneumoniae	Blood culture	M	Y
Klebsiella pneumoniae	Blood culture	V	Y
Providencia rettgeri	Blood culture	V	Y
Klebsiella pneumoniae	Blood culture	V	Y
Klebsiella pneumoniae	Blood culture	V	U
Enterobacter cloacae	Blood culture	V	Y
Klebsiella pneumoniae	Blood culture	V	Y
Klebsiella pneumoniae	Blood culture	V	Y
Klebsiella oxytoca	Blood culture	V	Y
Enterobacter aerogenes	Blood culture	V	Y
Enterobacter cloacae	Blood culture	V	U
Klebsiella pneumoniae	Blood culture	V	
Klebsiella pneumoniae	Blood culture	V	Y
Klebsiella oxytoca	Blood culture	V	Y
Escherichia coli	Blood culture	V	Y
Klebsiella pneumoniae	Blood culture	V	U
Klebsiella pneumoniae	Blood culture	V	U
Enterobacter cloacae	Blood culture	V	Y
Enterobacter cloacae	Blood culture	V	Y
Klebsiella pneumoniae	Blood culture	V	Y
Klebsiella pneumoniae	Blood culture	V	Y
Klebsiella pneumoniae	Blood culture	V	U
Serratia marcescens	Blood culture	V	Y
Klebsiella pneumoniae	Blood culture	V	Y
Serratia marcescens	Blood culture	V	Y
Klebsiella pneumoniae	Blood culture	V	Y
Klebsiella pneumoniae	Blood culture	V	Y
Klebsiella pneumoniae	Blood culture	V	Y
Klebsiella pneumoniae	Blood culture	V	Y
Klebsiella pneumoniae	Blood culture	V	Y
Enterobacter cloacae	Blood culture	V	Y
Klebsiella pneumoniae	Blood culture	V	Y
Klebsiella pneumoniae	Blood culture	V	Y
Klebsiella pneumoniae	Blood culture	V	Y
Klebsiella pneumoniae	Blood culture	V	Y
Klebsiella pneumoniae	Blood culture	V	Y
Klebsiella pneumoniae	Blood culture	V	Y
Enterobacter cloacae	Blood culture	V	Y
Enterobacter cloacae	Blood culture	V	Y
Serratia marcescens	Blood culture	V	Y
Serratia marcescens	Blood culture	V	Y
Klebsiella pneumoniae	Blood culture	V	Y
Klebsiella pneumoniae	Blood culture	V	Y
Enterobacter aerogenes	Blood culture	V	Y
Klebsiella pneumoniae	Blood culture	V	Y
Klebsiella pneumoniae	Blood culture	V	Y
Klebsiella pneumoniae	Blood culture	V	Y
Klebsiella pneumoniae	Blood culture	V	Y
Enterobacter cloacae	Blood culture	V	Y
Klebsiella pneumoniae	Blood culture	V	N
Klebsiella pneumoniae	Blood culture	V	Y

Escherichia coli	Blood culture	V	Y
Escherichia coli	Blood culture	V	Y
Klebsiella oxytoca	Blood culture	V	Y
Enterobacter cloacae	Blood culture	V	Y
Serratia marcescens	Blood culture	V	Y
Klebsiella pneumoniae	Blood culture	V	Y
Enterobacter cloacae	Blood culture	V	U
Klebsiella pneumoniae	Blood culture	V	Y
Klebsiella pneumoniae	Blood culture	V	Y
Klebsiella pneumoniae	Blood culture	V	U
Klebsiella pneumoniae	Blood culture	V	Y
Klebsiella pneumoniae	Blood culture	V	Y
Klebsiella ozaenae	Blood culture	V	Y
Klebsiella pneumoniae	Blood culture	V	Y
Klebsiella pneumoniae	Blood culture	V	U
Klebsiella pneumoniae	Blood culture	V	U
Klebsiella pneumoniae	Blood culture	V	U
Serratia marcescens	Blood culture	V	Y
Serratia marcescens	Blood culture	V	Y
Enterobacter cloacae	Blood culture	V	U
Klebsiella pneumoniae	Blood culture	V	Y
Enterobacter cloacae	Blood culture	V	Y
Klebsiella pneumoniae	Blood culture	V	Y
Klebsiella pneumoniae	Blood culture	V	Y
Klebsiella pneumoniae	Blood culture	V	Y
Klebsiella pneumoniae	Blood culture	V	Y
Klebsiella pneumoniae	Blood culture	V	Y
Klebsiella pneumoniae	Blood culture	V	Y
Klebsiella pneumoniae	Blood culture	V	
Klebsiella pneumoniae	Blood culture	M	Y
Klebsiella pneumoniae	Blood culture	V	Y
Klebsiella pneumoniae	Blood culture	V	Y
Klebsiella pneumoniae	Blood culture	V	Y
Enterobacter cloacae	Blood culture	V	Y
Klebsiella pneumoniae	Blood culture	V	Y
Klebsiella pneumoniae	Blood culture	M	Y
Klebsiella pneumoniae	Blood culture	M	Y
Escherichia coli	Blood culture	M	Y
Klebsiella pneumoniae	Blood culture	M	Y
Escherichia coli	Blood culture	M	Y
Klebsiella pneumoniae	Blood culture	M	Y
Escherichia coli	Blood culture	M	Y
Serratia marcescens	Blood culture	V	Y
Klebsiella pneumoniae	Blood culture	V	Y
Klebsiella pneumoniae	Blood culture	V	U
Klebsiella pneumoniae	Blood culture	V	Y
Klebsiella pneumoniae	Blood culture	V	Y
Escherichia coli	Blood culture	V	N
Klebsiella pneumoniae	Blood culture	V	Y
Klebsiella pneumoniae	Blood culture	V	Y
Klebsiella pneumoniae	Blood culture	V	U
Enterobacter cloacae	Blood culture	V	Y
Klebsiella pneumoniae	Blood culture	V	Y
Klebsiella pneumoniae	Blood culture	V	Y
Klebsiella pneumoniae	Blood culture	V	Y
Klebsiella pneumoniae	Blood culture	V	Y

Klebsiella pneumoniae	Blood culture	V	Y
Serratia marcescens	Blood culture	V	Y
Serratia marcescens	Blood culture	V	Y
Klebsiella pneumoniae	Blood culture	V	N
Klebsiella pneumoniae	Blood culture	V	Y
Klebsiella pneumoniae	Blood culture	V	Y
Klebsiella pneumoniae	Blood culture	M	
Klebsiella pneumoniae	Blood culture	V	Y
Klebsiella pneumoniae	Blood culture	V	U
Klebsiella pneumoniae	Blood culture	V	Y
Serratia marcescens	Blood culture	V	Y
Serratia marcescens	Blood culture	V	Y
Serratia marcescens	Blood culture	V	Y
Serratia marcescens	Blood culture	V	Y
Enterobacter cloacae	Blood culture	V	U
Klebsiella pneumoniae	Blood culture	V	Y
Klebsiella pneumoniae	Blood culture	V	U
Klebsiella pneumoniae	Blood culture	V	Y
Escherichia coli	Blood culture	V	Y
Klebsiella pneumoniae	Blood culture	V	Y
Klebsiella pneumoniae	Blood culture	V	Y
Enterobacter cloacae	Blood culture	V	Y
Serratia marcescens	Blood culture	V	Y
Klebsiella pneumoniae	Blood culture	V	Y
Klebsiella pneumoniae	Blood culture	V	Y
Klebsiella pneumoniae	Blood culture	V	U
Enterobacter cloacae complex	Blood culture	V	Y
Klebsiella pneumoniae	Blood culture	V	Y
Klebsiella pneumoniae	Blood culture	V	Y
Klebsiella pneumoniae	Blood culture	V	
Klebsiella pneumoniae	Blood culture	V	Y
Klebsiella pneumoniae	Blood culture	V	Y
Klebsiella pneumoniae	Blood culture	V	Y
Klebsiella pneumoniae	Blood culture	V	Y
Klebsiella pneumoniae	Blood culture	V	Y
Klebsiella pneumoniae	Blood culture	V	Y
Klebsiella pneumoniae	Blood culture	V	Y
Klebsiella pneumoniae	Blood culture	M	N
Klebsiella pneumoniae	Blood culture	V	Y
Klebsiella pneumoniae	Blood culture	V	Y
Enterobacter cloacae	Blood culture	M	U
Klebsiella pneumoniae	Blood culture	V	Y
Escherichia coli	Blood culture	M	N
Klebsiella pneumoniae	Blood culture	V	Y
Escherichia coli	Blood culture	M	Y
Klebsiella pneumoniae	Blood culture	V	Y
Escherichia coli	Blood culture	V	Y
Klebsiella pneumoniae	Blood culture	M	U
Serratia marcescens	Blood culture	M	U
Escherichia coli	Blood culture	M	Y
Klebsiella pneumoniae	Blood culture	M	Y
Proteus mirabilis	Blood culture	M	Y
Klebsiella pneumoniae	Blood culture	V	Y
Klebsiella pneumoniae	Blood culture	V	U
Enterobacter cloacae	Blood culture	V	Y
Klebsiella pneumoniae	Blood culture	V	Y

Proteus mirabilis	Blood culture	M	Y
Klebsiella pneumoniae	Blood culture	V	U
Klebsiella pneumoniae	Blood culture	V	N
Klebsiella pneumoniae	Blood culture	V	U
Klebsiella pneumoniae	Blood culture	V	Y
Klebsiella pneumoniae	Blood culture	V	Y
Enterobacter cloacae	Blood culture	V	Y
Klebsiella pneumoniae	Blood culture	V	Y
Klebsiella pneumoniae	Blood culture	V	Y
Klebsiella pneumoniae	Blood culture	V	Y
Klebsiella pneumoniae	Blood culture	V	Y
Klebsiella pneumoniae	Blood culture	V	Y
Klebsiella pneumoniae	Blood culture	V	Y
Klebsiella pneumoniae	Blood culture	V	Y
Klebsiella ozaenae	Blood culture	V	Y
Klebsiella pneumoniae	Blood culture	V	U
Klebsiella pneumoniae	Blood culture	V	U
Enterobacter cloacae	Blood culture	V	
Escherichia coli	Blood culture	V	U
Klebsiella pneumoniae	Blood culture	V	Y
Klebsiella pneumoniae	Blood culture	V	U
Klebsiella pneumoniae	Blood culture	V	U
Escherichia coli	Blood culture	V	U
Klebsiella pneumoniae	Blood culture	V	U
Klebsiella pneumoniae	Blood culture	V	U
Klebsiella pneumoniae	Blood culture	V	Y
Klebsiella oxytoca	Blood culture	V	N
Klebsiella pneumoniae	Blood culture	V	U
Enterobacter cloacae	Blood culture	V	Y
Klebsiella pneumoniae	Blood culture	V	Y
Klebsiella pneumoniae	Blood culture	V	Y
Enterobacter cloacae	Blood culture	V	Y
Klebsiella pneumoniae	Blood culture	V	Y
Klebsiella pneumoniae	Blood culture	V	Y
Klebsiella pneumoniae	Blood culture	V	Y
Escherichia coli	Blood culture	V	Y
Klebsiella pneumoniae	Blood culture	V	Y
Klebsiella pneumoniae	Blood culture	V	Y
Enterobacter cloacae	Blood culture	V	Y
Citrobacter sedlakii	Blood culture	V	Y
Klebsiella pneumoniae	Blood culture	V	Y
Escherichia coli	Blood culture	M	Y
Serratia marcescens	Blood culture	M	Y
Klebsiella pneumoniae	Blood culture	M	N
Enterobacter cloacae	Blood culture	M	Y
Klebsiella pneumoniae	Blood culture	V	Y
Klebsiella pneumoniae	Blood culture	M	U
Escherichia coli	Blood culture	M	Y
Escherichia coli	Blood culture	M	Y
Escherichia coli	Blood culture	M	Y
Klebsiella pneumoniae	Blood culture	M	Y
Klebsiella pneumoniae	Blood culture	V	Y
Klebsiella pneumoniae	Blood culture	M	Y
Klebsiella pneumoniae subsp pneumoniae	Blood culture	M	Y
Klebsiella pneumoniae	Blood culture	V	Y
Klebsiella pneumoniae	Blood culture	V	Y

Klebsiella pneumoniae	Blood culture	V	Y
Klebsiella pneumoniae	Blood culture	V	Y
Klebsiella pneumoniae	Blood culture	V	U
Klebsiella pneumoniae	Blood culture	V	U
Klebsiella pneumoniae	Blood culture	V	U
Klebsiella pneumoniae	Blood culture	V	Y
Enterobacter cloacae	Blood culture	V	Y
Klebsiella pneumoniae	Blood culture	V	
Klebsiella pneumoniae	Blood culture	V	U
Klebsiella pneumoniae	Blood culture	V	Y
Klebsiella oxytoca	Blood culture	V	U
Klebsiella pneumoniae	Blood culture	V	U
Klebsiella pneumoniae	Blood culture	V	Y
Klebsiella pneumoniae	Blood culture	V	Y
Escherichia coli	Blood culture	V	Y
Klebsiella pneumoniae	Blood culture	V	Y
Klebsiella pneumoniae	Blood culture	V	Y
Escherichia coli	Blood culture	V	U
Serratia marcescens	Blood culture	V	Y
Klebsiella pneumoniae	Blood culture	V	Y
Klebsiella pneumoniae subsp pneumoniae	Blood culture	M	N
Klebsiella pneumoniae	Blood culture	V	Y
Klebsiella pneumoniae	Blood culture	A	
Klebsiella pneumoniae	Blood culture	A	Y
Enterobacter cloacae	Blood culture	A	U
Klebsiella pneumoniae	Blood culture	A	
Klebsiella pneumoniae	Blood culture	A	N
Klebsiella pneumoniae	Blood culture	A	Y
Klebsiella pneumoniae	Blood culture	A	N
Klebsiella pneumoniae	Blood culture	A	
Klebsiella pneumoniae	Blood culture	A	Y
Enterobacter cloacae	Blood culture	A	U
Klebsiella pneumoniae	Blood culture	A	Y
Enterobacter cloacae	Blood culture	A	Y
Enterobacter cloacae	Blood culture	A	
Klebsiella pneumoniae	Blood culture	A	Y
Citrobacter koseri	Blood culture	A	Y
Klebsiella pneumoniae	Blood culture	A	U
Providencia rettgeri	Blood culture	A	
Klebsiella pneumoniae	Blood culture	A	
Klebsiella pneumoniae	Blood culture	A	U
Escherichia coli	Blood culture	A	N
Escherichia coli	Blood culture	A	U
Klebsiella pneumoniae	Blood culture	A	N
Klebsiella oxytoca	Blood culture	A	Y
Enterobacter cloacae	Blood culture	A	Y
Klebsiella pneumoniae	Blood culture	A	U
Morganella morganii	Blood culture	A	U
Klebsiella pneumoniae	Blood culture	A	Y
Klebsiella pneumoniae	Blood culture	A	Y
Klebsiella pneumoniae	Blood culture	A	Y
Klebsiella pneumoniae	Blood culture	A	Y
Klebsiella pneumoniae	Blood culture	A	U
Klebsiella pneumoniae	Blood culture	A	Y
Proteus mirabilis	Blood culture	A	Y
Morganella morganii	Blood culture	A	Y

Proteus mirabilis	Blood culture	A	Y
Serratia marcescens	Blood culture	A	Y
Morganella morganii	Blood culture	A	Y
Morganella morganii	Blood culture	A	Y
Morganella morganii	Blood culture	A	Y
Enterobacter cloacae	Blood culture	A	Y
Klebsiella pneumoniae	Blood culture	A	Y
Proteus mirabilis	Blood culture	A	Y
Morganella morganii	Blood culture	A	Y
Klebsiella pneumoniae subsp ozaenae	Blood culture	A	Y
Proteus mirabilis	Blood culture	M	Y
Klebsiella pneumoniae	Blood culture	M	U
Klebsiella pneumoniae	Blood culture	M	Y
Klebsiella pneumoniae	Blood culture	M	Y
Citrobacter freundii	Blood culture	A	Y
Morganella morganii	Blood culture	A	N
Citrobacter freundii	Blood culture	A	N
Klebsiella pneumoniae	Blood culture	A	U
Klebsiella pneumoniae subsp pneumoniae	Blood culture	A	U
Proteus mirabilis	Blood culture	A	Y
Klebsiella pneumoniae	Blood culture	A	Y
Escherichia coli	Blood culture	A	U
Klebsiella pneumoniae	Blood culture	A	U
Klebsiella pneumoniae	Blood culture	A	U
Klebsiella pneumoniae	Blood culture	A	N
Proteus mirabilis	Blood culture	A	Y
Klebsiella pneumoniae subsp pneumoniae	Blood culture	A	Y
Klebsiella pneumoniae subsp pneumoniae	Blood culture	A	Y
Klebsiella pneumoniae	Blood culture	A	Y
Enterobacter cloacae subsp dissolvens	Blood culture	A	U
Klebsiella pneumoniae	Blood culture	A	Y
Enterobacter cloacae	Blood culture	A	Y
Klebsiella pneumoniae	Blood culture	A	U
Klebsiella pneumoniae	Blood culture	A	U
Klebsiella pneumoniae subsp pneumoniae	Blood culture	A	Y
Enterobacter cloacae	Blood culture	A	U
Morganella morganii	Blood culture	A	U
Proteus mirabilis	Blood culture	A	Y
Serratia marcescens	Blood culture	A	Y
Klebsiella pneumoniae	Blood culture	A	N
Proteus mirabilis	Blood culture	A	Y
Enterobacter cloacae	Blood culture	A	U
Klebsiella pneumoniae subsp pneumoniae	Blood culture	A	Y
Klebsiella pneumoniae subsp pneumoniae	Blood culture	A	U
Enterobacter cloacae	Blood culture	A	U
Morganella morganii subsp morganii	Blood culture	A	
Escherichia coli	Blood culture	M	
Klebsiella pneumoniae	Blood culture	V	Y
Klebsiella pneumoniae	Blood culture	V	Y
Klebsiella pneumoniae	Blood culture	V	U
Serratia marcescens	Blood culture	V	Y
Klebsiella pneumoniae subsp pneumoniae	Blood culture	V	Y
Klebsiella pneumoniae	Blood culture	M	Y
Klebsiella pneumoniae subsp pneumoniae	Blood culture	V	Y
Proteus mirabilis	Blood culture	V	U
Enterobacter cloacae	Blood culture	V	Y

Klebsiella pneumoniae	Blood culture	V	Y
Klebsiella pneumoniae	Blood culture	V	Y
Klebsiella pneumoniae	Blood culture	V	Y
Klebsiella pneumoniae	Blood culture	V	Y
Klebsiella pneumoniae subsp pneumoniae	Blood culture	V	Y
Klebsiella pneumoniae	Blood culture	V	Y
Klebsiella pneumoniae	Blood culture	V	Y
Klebsiella pneumoniae	Blood culture	V	Y
Klebsiella pneumoniae subsp pneumoniae	Blood culture	V	Y
Klebsiella pneumoniae	Blood culture	V	Y
Enterobacter cloacae	Blood culture	V	U
Klebsiella pneumoniae	Blood culture	V	Y
Klebsiella pneumoniae	Blood culture	V	Y
Klebsiella pneumoniae	Blood culture	V	Y
Klebsiella pneumoniae	Blood culture	V	
Enterobacter cloacae	Blood culture	V	Y
Klebsiella pneumoniae subsp pneumoniae	Blood culture	V	Y
Salmonella non-typhi	Blood culture	V	Y
Klebsiella pneumoniae	Blood culture	M	Y
Proteus mirabilis	Blood culture	V	U
Klebsiella pneumoniae subsp pneumoniae	Blood culture	V	Y
Klebsiella pneumoniae	Blood culture	V	U
Klebsiella pneumoniae	Blood culture	V	Y
Klebsiella pneumoniae	Blood culture	V	Y
Klebsiella pneumoniae	Blood culture	V	Y
Klebsiella pneumoniae	Blood culture	V	U
Klebsiella pneumoniae	Blood culture	V	Y
Klebsiella pneumoniae	Blood culture	V	Y
Klebsiella pneumoniae subsp pneumoniae	Blood culture	V	Y
Klebsiella pneumoniae	Blood culture	V	N
Klebsiella pneumoniae	Blood culture	V	Y
Klebsiella pneumoniae subsp pneumoniae	Blood culture	M	Y
Klebsiella pneumoniae	Blood culture	M	Y
Klebsiella pneumoniae	Blood culture	M	Y
Citrobacter freundii	Blood culture	M	N
Klebsiella pneumoniae	Blood culture	M	Y
Klebsiella pneumoniae	Blood culture	M	Y
Klebsiella pneumoniae	Blood culture	V	Y
Klebsiella pneumoniae	Blood culture	M	Y
Enterobacter cloacae	Blood culture	V	Y
Klebsiella pneumoniae subsp pneumoniae	Blood culture	V	U
Klebsiella pneumoniae subsp pneumoniae	Blood culture	V	Y
Klebsiella pneumoniae	Blood culture	V	Y
Klebsiella pneumoniae subsp pneumoniae	Blood culture	V	U
Klebsiella pneumoniae subsp pneumoniae	Blood culture	V	Y
Klebsiella pneumoniae	Blood culture	V	Y
Klebsiella pneumoniae subsp pneumoniae	Blood culture	V	Y
Klebsiella pneumoniae subsp pneumoniae	Blood culture	V	Y
Klebsiella pneumoniae	Blood culture	V	Y
Klebsiella pneumoniae subsp pneumoniae	Blood culture	V	Y
Klebsiella pneumoniae subsp pneumoniae	Blood culture	M	Y
Klebsiella pneumoniae	Blood culture	V	Y
Klebsiella pneumoniae subsp pneumoniae	Blood culture	V	Y
Klebsiella pneumoniae	Blood culture	V	Y
Klebsiella pneumoniae subsp pneumoniae	Blood culture	V	Y
Klebsiella pneumoniae subsp ozaenae	Blood culture	V	Y

Klebsiella pneumoniae subsp pneumoniae	Blood culture	V	Y
Klebsiella pneumoniae	Blood culture	N	Y
Klebsiella pneumoniae	Blood culture	V	Y
Klebsiella pneumoniae	Blood culture	V	U
Klebsiella pneumoniae	Blood culture	V	U
Klebsiella pneumoniae	Blood culture	V	U
Klebsiella pneumoniae	Blood culture	V	U
Klebsiella pneumoniae subsp pneumoniae	Blood culture	V	U
Enterobacter cloacae	Blood culture	V	U
Klebsiella pneumoniae	Blood culture	V	U
Raoutella ornithinolytica	Blood culture	V	U
Enterobacter cloacae	Blood culture	V	Y
Enterobacter cloacae	Blood culture	V	N
Klebsiella pneumoniae subsp pneumoniae	Blood culture	V	Y
Serratia marcescens	Blood culture	V	Y
Klebsiella pneumoniae subsp pneumoniae	Blood culture	V	Y
Klebsiella pneumoniae subsp pneumoniae	Blood culture	V	Y
Klebsiella pneumoniae subsp pneumoniae	Blood culture	V	Y
Klebsiella pneumoniae subsp pneumoniae	Blood culture	V	Y
Klebsiella pneumoniae	Blood culture	V	Y
Klebsiella pneumoniae subsp pneumoniae	Blood culture	V	Y
Klebsiella pneumoniae subsp pneumoniae	Blood culture	V	Y
Klebsiella pneumoniae	Blood culture	V	Y
Klebsiella pneumoniae subsp pneumoniae	Blood culture	V	Y
Klebsiella pneumoniae subsp pneumoniae	Blood culture	V	Y
Klebsiella pneumoniae	Blood culture	V	Y
Klebsiella pneumoniae subsp pneumoniae	Blood culture	V	Y
Serratia marcescens	Blood culture	V	Y
Klebsiella pneumoniae subsp pneumoniae	Blood culture	A	Y
Klebsiella pneumoniae subsp pneumoniae	Blood culture	A	
Klebsiella pneumoniae subsp pneumoniae	Blood culture	A	Y
Escherichia coli	Blood culture	A	U
Klebsiella pneumoniae	Blood culture	A	
Klebsiella pneumoniae	Blood culture	A	Y
Klebsiella pneumoniae subsp pneumoniae	Blood culture	V	Y
Klebsiella pneumoniae	Blood culture	A	Y
Klebsiella pneumoniae subsp pneumoniae	Blood culture	A	Y
Enterobacter aerogenes	Blood culture	A	Y
Proteus vulgaris	Blood culture	A	Y
Enterobacter cloacae complex	Blood culture	A	Y
Klebsiella pneumoniae subsp pneumoniae	Blood culture	A	Y
Klebsiella pneumoniae subsp pneumoniae	Blood culture	A	Y
Klebsiella pneumoniae subsp pneumoniae	Blood culture	A	Y
Klebsiella pneumoniae	Blood culture	A	U
Escherichia coli	Blood culture	A	U
Klebsiella pneumoniae subsp pneumoniae	Blood culture	A	Y
Klebsiella pneumoniae subsp pneumoniae	Blood culture	A	U
Enterobacter cloacae	Blood culture	A	U
Enterobacter cloacae complex	Blood culture	A	Y
Klebsiella pneumoniae	Blood culture	A	Y
Enterobacter aerogenes	Blood culture	A	U
Morganella morganii subsp morganii	Blood culture	A	Y
Klebsiella pneumoniae	Blood culture	A	U
Enterobacter cloacae	Blood culture	A	U
Serratia marcescens	Blood culture	A	Y
Klebsiella pneumoniae subsp pneumoniae	Blood culture	V	Y

Klebsiella pneumoniae	Blood culture	V	Y
Klebsiella pneumoniae	Blood culture	V	Y
Klebsiella pneumoniae subsp pneumoniae	Blood culture	V	Y
Klebsiella pneumoniae subsp pneumoniae	Blood culture	M	Y
Klebsiella pneumoniae	Blood culture	V	Y
Klebsiella planticola	Blood culture	V	Y
Klebsiella pneumoniae	Blood culture	V	Y
Enterobacter cloacae	Blood culture	M	Y
Klebsiella pneumoniae	Blood culture	M	Y
Klebsiella pneumoniae subsp pneumoniae	Blood culture	M	Y
Enterobacter cloacae	Blood culture	M	Y
Klebsiella pneumoniae	Blood culture	M	U
Citrobacter koseri	Blood culture	M	Y
Klebsiella pneumoniae	Blood culture	M	Y
Klebsiella pneumoniae	Blood culture	M	N
Klebsiella pneumoniae	Blood culture	M	U
Klebsiella pneumoniae	Blood culture	V	Y
Klebsiella pneumoniae subsp pneumoniae	Blood culture	V	Y
Serratia marcescens	Blood culture	V	Y
Klebsiella pneumoniae subsp pneumoniae	Blood culture	V	Y
Klebsiella pneumoniae	Blood culture	V	Y
Klebsiella pneumoniae subsp pneumoniae	Blood culture	V	Y
Klebsiella pneumoniae subsp pneumoniae	Blood culture	V	Y
Klebsiella pneumoniae	Blood culture	V	Y
Klebsiella pneumoniae subsp pneumoniae	Blood culture	M	Y
Escherichia coli	Blood culture	N	N
Klebsiella pneumoniae	Blood culture	M	N
Klebsiella pneumoniae subsp pneumoniae	Blood culture	V	Y
Klebsiella pneumoniae subsp pneumoniae	Blood culture	V	Y
Klebsiella pneumoniae subsp pneumoniae	Blood culture	V	Y
Klebsiella pneumoniae	Blood culture	V	Y
Klebsiella pneumoniae	Blood culture	M	N
Klebsiella pneumoniae subsp pneumoniae	Blood culture	M	Y
Klebsiella pneumoniae	Blood culture	V	Y
Klebsiella pneumoniae	Blood culture	V	Y
Klebsiella pneumoniae	Blood culture	V	Y
Klebsiella pneumoniae	Blood culture	V	Y
Klebsiella pneumoniae subsp pneumoniae	Blood culture	V	Y
Klebsiella pneumoniae subsp pneumoniae	Blood culture	V	Y
Klebsiella pneumoniae subsp pneumoniae	Blood culture	V	Y
Klebsiella pneumoniae subsp pneumoniae	Blood culture	V	Y
Klebsiella pneumoniae	Blood culture	V	U
Klebsiella pneumoniae subsp pneumoniae	Blood culture	V	Y
Klebsiella pneumoniae subsp pneumoniae	Blood culture	V	N
Klebsiella pneumoniae	Blood culture	V	Y
Proteus mirabilis	Blood culture	V	
Klebsiella pneumoniae	Blood culture	V	Y
Klebsiella pneumoniae	Blood culture	N	Y
Klebsiella pneumoniae	Blood culture	V	U
Klebsiella pneumoniae	Blood culture	V	Y
Klebsiella pneumoniae	Blood culture	V	
Klebsiella pneumoniae subsp pneumoniae	Blood culture	V	Y
Klebsiella pneumoniae	Blood culture	V	U
Klebsiella pneumoniae subsp pneumoniae	Blood culture	V	Y
Klebsiella pneumoniae subsp pneumoniae	Blood culture	V	U
Citrobacter freundii	Blood culture	V	U

Klebsiella pneumoniae	Blood culture	V	Y
Klebsiella pneumoniae	Blood culture	V	Y
Klebsiella pneumoniae	Blood culture	V	U
Klebsiella pneumoniae	Blood culture	V	U
Klebsiella pneumoniae	Blood culture	V	U
Klebsiella pneumoniae subsp pneumoniae	Blood culture	V	Y
Klebsiella pneumoniae	Blood culture	V	Y
Klebsiella pneumoniae	Blood culture	V	Y
Klebsiella pneumoniae	Blood culture	V	Y
Serratia marcescens	Blood culture	V	Y
Klebsiella pneumoniae subsp pneumoniae	Blood culture	V	Y
Klebsiella pneumoniae subsp pneumoniae	Blood culture	V	Y
Klebsiella pneumoniae subsp pneumoniae	Blood culture	V	Y
Klebsiella pneumoniae	Blood culture	V	Y
Klebsiella pneumoniae	Blood culture	V	U
Klebsiella pneumoniae	Blood culture	V	U
Klebsiella pneumoniae	Blood culture	V	Y
Klebsiella pneumoniae	Blood culture	V	U
Klebsiella pneumoniae	Blood culture	V	U
Klebsiella pneumoniae subsp ozaenae	Blood culture	V	Y
Enterobacter cloacae	Blood culture	V	Y
Klebsiella pneumoniae subsp pneumoniae	Blood culture	V	Y
Klebsiella pneumoniae subsp pneumoniae	Blood culture	V	Y
Klebsiella pneumoniae subsp pneumoniae	Blood culture	V	Y
Enterobacter cloacae	Blood culture	M	Y
Klebsiella pneumoniae	Blood culture	M	U
Enterobacter cloacae	Blood culture	M	Y
Klebsiella pneumoniae	Blood culture	M	N
Klebsiella pneumoniae	Blood culture	V	Y
Klebsiella pneumoniae	Blood culture	V	U
Klebsiella pneumoniae subsp pneumoniae	Blood culture	V	Y
Klebsiella pneumoniae subsp pneumoniae	Blood culture	V	Y
Klebsiella pneumoniae subsp pneumoniae	Blood culture	V	Y
Klebsiella pneumoniae	Blood culture	V	Y
Morganella morganii	Blood culture	M	Y
Citrobacter freundii	Blood culture	M	
Escherichia coli	Blood culture	V	Y
Klebsiella pneumoniae	Blood culture	V	Y
Klebsiella pneumoniae subsp pneumoniae	Blood culture	V	Y
Klebsiella pneumoniae	Blood culture	V	U
Klebsiella pneumoniae	Blood culture	M	Y
Citrobacter freundii	Blood culture	V	Y
Klebsiella pneumoniae subsp pneumoniae	Blood culture	V	Y
Klebsiella pneumoniae subsp pneumoniae	Blood culture	V	Y
Klebsiella pneumoniae subsp pneumoniae	Blood culture	M	
Klebsiella pneumoniae subsp pneumoniae	Blood culture	V	Y
Klebsiella pneumoniae subsp pneumoniae	Blood culture	V	Y
Klebsiella pneumoniae	Blood culture	V	Y
Klebsiella pneumoniae	Blood culture	V	Y
Klebsiella pneumoniae	Blood culture	V	Y
Klebsiella pneumoniae	Blood culture	V	U
Klebsiella pneumoniae	Blood culture	V	U
Klebsiella pneumoniae	Blood culture	V	Y
Klebsiella pneumoniae	Blood culture	V	U
Klebsiella pneumoniae	Blood culture	V	U
Klebsiella pneumoniae	Blood culture	V	U

Klebsiella pneumoniae	Blood culture	V	U
Enterobacter cloacae	Blood culture	V	Y
Escherichia coli	Blood culture	V	U
Klebsiella pneumoniae subsp pneumoniae	Blood culture	V	U
Klebsiella pneumoniae	Blood culture	V	
Klebsiella pneumoniae	Blood culture	V	U
Klebsiella pneumoniae	Blood culture	V	U
Klebsiella pneumoniae subsp pneumoniae	Blood culture	V	Y
Klebsiella pneumoniae subsp ozaenae	Blood culture	V	Y
Enterobacter cloacae	Blood culture	V	Y
Klebsiella pneumoniae	Blood culture	V	Y
Klebsiella pneumoniae subsp pneumoniae	Blood culture	V	Y
Enterobacter cloacae	Blood culture	V	Y
Klebsiella pneumoniae subsp pneumoniae	Blood culture	V	Y
Klebsiella pneumoniae	Blood culture	M	Y
Klebsiella pneumoniae	Blood culture	M	Y
Klebsiella pneumoniae	Blood culture	V	Y
Klebsiella pneumoniae	Blood culture	V	Y
Klebsiella pneumoniae	Blood culture	V	Y
Enterobacter cloacae	Blood culture	V	Y
Klebsiella pneumoniae subsp pneumoniae	Blood culture	M	Y
Klebsiella pneumoniae subsp pneumoniae	Blood culture	V	Y
Klebsiella pneumoniae	Blood culture	M	N
Klebsiella pneumoniae	Blood culture	V	Y
Klebsiella pneumoniae subsp pneumoniae	Blood culture	V	Y
Klebsiella pneumoniae subsp pneumoniae	Blood culture	V	Y
Klebsiella pneumoniae	Blood culture	V	Y
Proteus mirabilis	Blood culture	V	Y
Klebsiella pneumoniae	Blood culture	M	U
Klebsiella pneumoniae subsp pneumoniae	Blood culture	V	Y
Klebsiella pneumoniae subsp pneumoniae	Blood culture	V	Y
Klebsiella pneumoniae	Blood culture	M	Y
Klebsiella pneumoniae	Blood culture	M	Y
Klebsiella pneumoniae	Blood culture	M	Y
Klebsiella pneumoniae subsp pneumoniae	Blood culture	V	Y
Klebsiella pneumoniae subsp pneumoniae	Blood culture	V	Y
Klebsiella pneumoniae subsp pneumoniae	Blood culture	V	Y
Enterobacter cloacae	Blood culture	V	Y
Klebsiella pneumoniae subsp pneumoniae	Blood culture	V	Y
Klebsiella pneumoniae subsp pneumoniae	Blood culture	V	Y
Serratia marcescens	Blood culture	V	Y
Serratia marcescens	Blood culture	V	U
Enterobacter cloacae	Blood culture	M	Y
Klebsiella pneumoniae subsp pneumoniae	Blood culture	V	U
Enterobacter cloacae	Blood culture	M	Y
Klebsiella pneumoniae subsp pneumoniae	Blood culture	V	Y
Klebsiella pneumoniae subsp pneumoniae	Blood culture	V	Y
Klebsiella pneumoniae subsp pneumoniae	Blood culture	V	Y
Klebsiella pneumoniae	Blood culture	V	Y
Klebsiella pneumoniae	Blood culture	V	Y
Enterobacter cloacae	Blood culture	V	Y
Enterobacter cloacae	Blood culture	V	Y
Enterobacter cloacae	Blood culture	M	Y
Klebsiella pneumoniae subsp pneumoniae	Blood culture	M	Y
Klebsiella pneumoniae	Blood culture	V	Y
Klebsiella pneumoniae subsp pneumoniae	Blood culture	V	Y

Klebsiella pneumoniae subsp pneumoniae	Blood culture	V	Y
Serratia marcescens	Blood culture	V	Y
Klebsiella pneumoniae subsp pneumoniae	Blood culture	V	Y
Klebsiella pneumoniae subsp pneumoniae	Blood culture	V	Y
Klebsiella pneumoniae subsp pneumoniae	Blood culture	V	Y
Klebsiella pneumoniae subsp pneumoniae	Blood culture	V	Y
Enterobacter cloacae	Blood culture	V	Y
Serratia marcescens	Blood culture	V	Y
Enterobacter cloacae	Blood culture	V	Y
Enterobacter cloacae	Blood culture	V	Y
Klebsiella pneumoniae subsp pneumoniae	Blood culture	V	Y
Klebsiella pneumoniae subsp pneumoniae	Blood culture	V	Y
Klebsiella pneumoniae subsp pneumoniae	Blood culture	V	Y
Klebsiella pneumoniae subsp pneumoniae	Blood culture	V	Y
Klebsiella pneumoniae subsp pneumoniae	Blood culture	V	Y
Klebsiella pneumoniae	Blood culture	V	Y
Klebsiella pneumoniae subsp pneumoniae	Blood culture	V	Y
Klebsiella pneumoniae subsp pneumoniae	Blood culture	V	Y
Klebsiella pneumoniae subsp pneumoniae	Blood culture	V	Y
Klebsiella pneumoniae subsp pneumoniae	Blood culture	V	N
Klebsiella pneumoniae subsp pneumoniae	Blood culture	V	Y
Klebsiella pneumoniae	Blood culture	V	Y
Klebsiella pneumoniae	Blood culture	V	Y
Klebsiella pneumoniae subsp pneumoniae	Blood culture	V	Y
Klebsiella pneumoniae subsp pneumoniae	Blood culture	V	Y
Klebsiella pneumoniae subsp pneumoniae	Blood culture	V	Y
Klebsiella pneumoniae subsp pneumoniae	Blood culture	V	Y
Klebsiella pneumoniae	Blood culture	V	Y
Klebsiella pneumoniae	Blood culture	V	Y
Klebsiella pneumoniae subsp pneumoniae	Blood culture	V	Y
Klebsiella pneumoniae subsp pneumoniae	Blood culture	V	Y
Escherichia hermannii	Blood culture	V	Y
Serratia marcescens	Blood culture	V	Y
Klebsiella pneumoniae subsp pneumoniae	Blood culture	V	Y
Klebsiella pneumoniae subsp pneumoniae	Blood culture	V	Y
Providencia rettgeri	Blood culture	V	Y
Klebsiella pneumoniae subsp pneumoniae	Blood culture	V	Y
Enterobacter cloacae	Blood culture	V	Y
Klebsiella pneumoniae	Blood culture	V	Y
Klebsiella pneumoniae subsp pneumoniae	Blood culture	V	Y
Klebsiella pneumoniae subsp pneumoniae	Blood culture	V	Y
Klebsiella pneumoniae subsp pneumoniae	Blood culture	V	Y
Klebsiella pneumoniae	Blood culture	V	Y
Klebsiella pneumoniae subsp pneumoniae	Blood culture	V	Y
Klebsiella pneumoniae subsp pneumoniae	Blood culture	V	Y
Klebsiella pneumoniae subsp pneumoniae	Blood culture	V	Y
Enterobacter cloacae	Blood culture	V	Y
Klebsiella pneumoniae subsp pneumoniae	Blood culture	V	N
Klebsiella pneumoniae subsp pneumoniae	Blood culture	V	Y
Klebsiella pneumoniae subsp pneumoniae	Blood culture	V	Y
Klebsiella pneumoniae subsp pneumoniae	Blood culture	V	Y
Klebsiella pneumoniae subsp pneumoniae	Blood culture	V	Y
Klebsiella pneumoniae	Blood culture	V	Y
Klebsiella pneumoniae subsp pneumoniae	Blood culture	V	Y
Klebsiella pneumoniae subsp pneumoniae	Blood culture	V	Y
Klebsiella pneumoniae subsp pneumoniae	Blood culture	V	Y

Klebsiella pneumoniae subsp pneumoniae	Blood culture	V	U
Klebsiella pneumoniae subsp pneumoniae	Blood culture	V	Y
Klebsiella pneumoniae subsp pneumoniae	Blood culture	V	Y
Klebsiella pneumoniae subsp pneumoniae	Blood culture	V	N
Klebsiella pneumoniae subsp pneumoniae	Blood culture	V	Y
Klebsiella pneumoniae subsp ozaenae	Blood culture	V	Y
Klebsiella pneumoniae subsp pneumoniae	Blood culture	V	Y
Klebsiella pneumoniae subsp pneumoniae	Blood culture	V	Y
Klebsiella pneumoniae	Blood culture	V	Y
Klebsiella pneumoniae subsp pneumoniae	Blood culture	V	Y
Enterobacter cloacae	Blood culture	V	U
Klebsiella pneumoniae	Blood culture	V	Y
Klebsiella pneumoniae	Blood culture	V	U
Klebsiella pneumoniae	Blood culture	V	U
Enterobacter cloacae subsp cloacae	Blood culture	A	
Klebsiella pneumoniae subsp pneumoniae	Blood culture	A	Y
Klebsiella pneumoniae subsp pneumoniae	Blood culture	A	N
Klebsiella oxytoca	Blood culture	A	
Klebsiella pneumoniae subsp pneumoniae	Blood culture	A	
Klebsiella pneumoniae	Blood culture	A	U
Klebsiella pneumoniae subsp pneumoniae	Blood culture	A	Y
Klebsiella pneumoniae subsp pneumoniae	Blood culture	A	N
Proteus mirabilis	Blood culture	A	Y
Klebsiella pneumoniae	Blood culture	A	U
Enterobacter cloacae	Blood culture	A	U
Klebsiella pneumoniae subsp pneumoniae	Blood culture	A	Y
Klebsiella pneumoniae	Blood culture	A	U
Citrobacter freundii	Blood culture	A	U
Escherichia coli	Blood culture	A	Y
Klebsiella pneumoniae	Blood culture	A	
Klebsiella pneumoniae	Blood culture	A	U
Enterobacter cloacae	Blood culture	A	
Klebsiella pneumoniae	Blood culture	A	U
Providencia stuartii	Blood culture	A	Y
Klebsiella pneumoniae	Blood culture	A	Y
Klebsiella pneumoniae	Blood culture	A	U
Klebsiella pneumoniae	Blood culture	A	Y
Klebsiella pneumoniae	Blood culture	A	U
Klebsiella pneumoniae	Blood culture	A	Y
Klebsiella pneumoniae	Blood culture	A	N
Klebsiella pneumoniae	Blood culture	A	Y
Klebsiella pneumoniae	Blood culture	A	Y
Enterobacter aerogenes	Blood culture	A	U
Klebsiella pneumoniae	Blood culture	A	Y
Klebsiella oxytoca	Blood culture	A	U
Providencia stuartii	Blood culture	A	U
Klebsiella pneumoniae	Blood culture	A	Y
Klebsiella oxytoca	Blood culture	A	
Providencia stuartii	Blood culture	A	
Klebsiella oxytoca	Blood culture	A	U
Klebsiella oxytoca	Blood culture	A	
Providencia rettgeri	Blood culture	A	U
Klebsiella oxytoca	Blood culture	A	Y
Providencia rettgeri	Blood culture	A	
Klebsiella pneumoniae	Blood culture	A	U
Klebsiella pneumoniae	Blood culture	A	U

Enterobacter cloacae complex	Blood culture	A	Y
Klebsiella pneumoniae	Blood culture	A	
Klebsiella pneumoniae	Blood culture	A	Y
Klebsiella pneumoniae	Blood culture	A	Y
Klebsiella pneumoniae	Blood culture	A	Y
Klebsiella pneumoniae	Blood culture	A	Y
Klebsiella pneumoniae subsp pneumoniae	Blood culture	A	Y
Enterobacter cloacae	Blood culture	A	Y
Klebsiella pneumoniae	Blood culture	A	Y
Klebsiella pneumoniae subsp pneumoniae	Blood culture	A	Y
Klebsiella pneumoniae	Blood culture	A	Y
Klebsiella pneumoniae	Blood culture	A	Y
Klebsiella oxytoca	Blood culture	A	Y
Klebsiella pneumoniae	Blood culture	A	Y
Klebsiella pneumoniae	Blood culture	A	Y
Klebsiella pneumoniae subsp pneumoniae	Blood culture	A	Y
Providencia rettgeri	Blood culture	A	
Klebsiella pneumoniae	Blood culture	A	Y
Klebsiella pneumoniae subsp pneumoniae	Blood culture	A	Y
Klebsiella pneumoniae	Blood culture	A	
Klebsiella pneumoniae	Blood culture	A	Y
Klebsiella pneumoniae	Blood culture	A	Y
Enterobacter cloacae subsp cloacae	Blood culture	A	
Klebsiella pneumoniae	Blood culture	A	N
Klebsiella oxytoca	Blood culture	A	U
Klebsiella pneumoniae	Blood culture	A	
Enterobacter cloacae	Blood culture	A	U
Enterobacter cloacae complex	Blood culture	A	Y
Klebsiella pneumoniae	Blood culture	A	Y
Klebsiella pneumoniae	Blood culture	A	Y
Klebsiella pneumoniae subsp pneumoniae	Blood culture	A	Y
Klebsiella pneumoniae subsp pneumoniae	Blood culture	A	Y
Klebsiella pneumoniae	Blood culture	A	Y
Klebsiella pneumoniae subsp pneumoniae	Blood culture	A	Y
Klebsiella pneumoniae subsp pneumoniae	Blood culture	A	Y
Klebsiella pneumoniae	Blood culture	A	Y
Klebsiella oxytoca	Blood culture	A	Y
Enterobacter cloacae	Blood culture	A	U
Klebsiella pneumoniae	Blood culture	A	U
Klebsiella pneumoniae	Blood culture	A	U
Klebsiella pneumoniae subsp pneumoniae	Blood culture	A	
Klebsiella pneumoniae subsp pneumoniae	Blood culture	A	Y
Klebsiella pneumoniae	Blood culture	A	Y
Klebsiella pneumoniae	Blood culture	A	Y
Enterobacter aerogenes	Blood culture	A	
Klebsiella oxytoca	Blood culture	A	U
Klebsiella pneumoniae	Blood culture	A	
Klebsiella pneumoniae	Blood culture	A	
Klebsiella pneumoniae	Blood culture	A	U
Enterobacter cloacae	Blood culture	A	U
Klebsiella pneumoniae	Blood culture	A	Y
Klebsiella pneumoniae	Blood culture	A	Y
Klebsiella pneumoniae	Blood culture	A	Y
Klebsiella oxytoca	Blood culture	A	U
Klebsiella pneumoniae	Blood culture	A	Y
Klebsiella pneumoniae subsp pneumoniae	Blood culture	A	Y

Klebsiella pneumoniae	Blood culture	A	N
Klebsiella pneumoniae	Blood culture	A	Y
Serratia marcescens	Blood culture	A	N
Klebsiella oxytoca	Blood culture	A	U
Klebsiella oxytoca	Blood culture	A	U
Klebsiella pneumoniae	Blood culture	A	Y
Klebsiella pneumoniae	Blood culture	A	Y
Klebsiella pneumoniae	Blood culture	A	
Klebsiella pneumoniae	Blood culture	A	U
Klebsiella pneumoniae	Blood culture	A	Y
Klebsiella pneumoniae	Blood culture	A	Y
Klebsiella pneumoniae subsp pneumoniae	Blood culture	A	Y
Klebsiella pneumoniae	Blood culture	A	U
Klebsiella pneumoniae subsp pneumoniae	Blood culture	A	Y
Klebsiella pneumoniae	Blood culture	A	U
Klebsiella pneumoniae	Blood culture	A	U
Klebsiella pneumoniae subsp pneumoniae	Blood culture	A	Y
Klebsiella pneumoniae	Blood culture	A	Y
Enterobacter cloacae	Blood culture	M	U
Klebsiella pneumoniae	Blood culture	M	Y
Enterobacter cloacae	Blood culture	M	U
Proteus mirabilis	Blood culture	M	U
Klebsiella pneumoniae	Blood culture	M	Y
Enterobacter cloacae	Blood culture	M	Y
Enterobacter cloacae	Blood culture	V	Y
Klebsiella pneumoniae subsp pneumoniae	Blood culture	V	Y
Serratia marcescens	Blood culture	V	Y
Klebsiella pneumoniae subsp pneumoniae	Blood culture	V	Y
Serratia marcescens	Blood culture	V	Y
Klebsiella pneumoniae	Blood culture	V	Y
Klebsiella pneumoniae subsp pneumoniae	Blood culture	V	Y
Klebsiella pneumoniae subsp pneumoniae	Blood culture	V	Y
Klebsiella pneumoniae	Blood culture	V	Y
Klebsiella pneumoniae	Blood culture	V	Y
Klebsiella pneumoniae subsp pneumoniae	Blood culture	V	Y
Serratia marcescens	Blood culture	V	Y
Enterobacter aerogenes	Blood culture	V	Y
Klebsiella pneumoniae	Blood culture	V	Y
Klebsiella pneumoniae	Blood culture	V	U
Klebsiella pneumoniae	Blood culture	V	Y
Klebsiella pneumoniae	Blood culture	V	Y
Klebsiella pneumoniae	Blood culture	V	Y
Klebsiella pneumoniae subsp pneumoniae	Blood culture	V	Y
Klebsiella pneumoniae	Blood culture	V	Y
Klebsiella pneumoniae subsp pneumoniae	Blood culture	V	Y
Klebsiella pneumoniae subsp pneumoniae	Blood culture	V	Y
Klebsiella pneumoniae subsp pneumoniae	Blood culture	V	Y
Providencia rettgeri	Blood culture	V	Y
Klebsiella pneumoniae subsp pneumoniae	Blood culture	V	Y
Klebsiella pneumoniae subsp pneumoniae	Blood culture	V	Y
Enterobacter cloacae	Blood culture	V	U
Klebsiella pneumoniae	Blood culture	V	
Klebsiella pneumoniae	Blood culture	V	Y
Klebsiella pneumoniae	Blood culture	V	U
Serratia marcescens	Blood culture	V	Y
Serratia marcescens	Blood culture	V	Y

Leclercia adecarboxylata	Blood culture	V	Y
Klebsiella pneumoniae	Blood culture	V	U
Klebsiella pneumoniae	Blood culture	V	U
Escherichia coli	Blood culture	V	Y
Klebsiella pneumoniae subsp pneumoniae	Blood culture	V	Y
Klebsiella pneumoniae	Blood culture	V	Y
Klebsiella pneumoniae	Blood culture	V	Y
Klebsiella pneumoniae	Blood culture	V	Y
Klebsiella pneumoniae subsp pneumoniae	Blood culture	V	Y
Klebsiella pneumoniae	Blood culture	V	U
Klebsiella pneumoniae	Blood culture	V	U
Klebsiella pneumoniae	Blood culture	V	Y
Klebsiella pneumoniae	Blood culture	V	U
Klebsiella pneumoniae	Blood culture	V	U
Klebsiella pneumoniae	Blood culture	V	Y
Klebsiella pneumoniae	Blood culture	V	Y
Klebsiella pneumoniae	Blood culture	V	Y
Klebsiella oxytoca	Blood culture	V	U
Klebsiella pneumoniae subsp pneumoniae	Blood culture	V	Y
Escherichia coli	Blood culture	V	Y
Klebsiella pneumoniae	Blood culture	V	U
Klebsiella pneumoniae subsp pneumoniae	Blood culture	V	Y
Klebsiella pneumoniae subsp pneumoniae	Blood culture	V	Y
Klebsiella pneumoniae subsp pneumoniae	Blood culture	V	Y
Serratia marcescens	Blood culture	V	Y
Serratia marcescens	Blood culture	V	Y
Klebsiella pneumoniae subsp pneumoniae	Blood culture	V	Y
Klebsiella oxytoca	Blood culture	V	Y
Serratia marcescens	Blood culture	V	U
Klebsiella pneumoniae	Blood culture	V	Y
Klebsiella pneumoniae	Blood culture	V	Y
Klebsiella pneumoniae	Blood culture	V	U
Klebsiella pneumoniae	Blood culture	V	Y
Klebsiella pneumoniae	Blood culture	V	N
Klebsiella pneumoniae subsp ozaenae	Blood culture	V	Y
Escherichia coli	Blood culture	V	Y
Klebsiella pneumoniae	Blood culture	V	Y
Klebsiella pneumoniae	Blood culture	M	Y
Escherichia coli	Blood culture	M	Y
Klebsiella pneumoniae	Blood culture	M	Y
Klebsiella pneumoniae	Blood culture	M	Y
Klebsiella pneumoniae	Blood culture	V	Y
Klebsiella pneumoniae	Blood culture	M	Y
Klebsiella pneumoniae subsp pneumoniae	Blood culture	V	Y
Klebsiella pneumoniae	Blood culture	M	Y
Klebsiella pneumoniae subsp pneumoniae	Blood culture	V	Y
Klebsiella pneumoniae	Blood culture	V	Y
Klebsiella pneumoniae	Blood culture	V	Y
Klebsiella pneumoniae	Blood culture	V	U
Klebsiella pneumoniae	Blood culture	V	Y
Escherichia coli	Blood culture	V	U
Klebsiella pneumoniae	Blood culture	V	U
Klebsiella pneumoniae	Blood culture	V	U
Klebsiella pneumoniae	Blood culture	N	U
Klebsiella pneumoniae subsp pneumoniae	Blood culture	V	Y
Klebsiella pneumoniae	Blood culture	V	Y

Citrobacter freundii	Blood culture	V	U
Klebsiella pneumoniae	Blood culture	V	Y
Klebsiella pneumoniae	Blood culture	V	Y
Klebsiella pneumoniae subsp pneumoniae	Blood culture	V	Y
Serratia marcescens	Blood culture	V	N
Klebsiella pneumoniae	Blood culture	V	Y
Klebsiella pneumoniae subsp pneumoniae	Blood culture	V	N
Klebsiella pneumoniae subsp pneumoniae	Blood culture	V	Y
Klebsiella pneumoniae subsp pneumoniae	Blood culture	V	Y
Klebsiella pneumoniae subsp ozaenae	Blood culture	V	Y
Klebsiella pneumoniae subsp pneumoniae	Blood culture	V	Y
Klebsiella pneumoniae	Blood culture	V	Y
Klebsiella pneumoniae	Blood culture	V	Y
Klebsiella pneumoniae	Blood culture	V	Y
Klebsiella pneumoniae subsp pneumoniae	Blood culture	V	Y
Klebsiella pneumoniae subsp pneumoniae	Blood culture	V	Y
Klebsiella pneumoniae	Blood culture	V	Y
Klebsiella pneumoniae subsp pneumoniae	Blood culture	V	N
Klebsiella pneumoniae subsp pneumoniae	Blood culture	V	Y
Klebsiella pneumoniae subsp pneumoniae	Blood culture	V	Y
Enterobacter cloacae	Blood culture	V	Y
Klebsiella pneumoniae	Blood culture	V	Y
Enterobacter cloacae	Blood culture	V	Y
Escherichia coli	Blood culture	V	Y
Klebsiella pneumoniae subsp pneumoniae	Blood culture	V	Y
Klebsiella pneumoniae subsp ozaenae	Blood culture	V	Y
Klebsiella pneumoniae	Blood culture	V	Y
Klebsiella pneumoniae subsp ozaenae	Blood culture	A	Y
Serratia marcescens	Blood culture	A	Y
Enterobacter cloacae	Blood culture	A	U
Klebsiella pneumoniae subsp pneumoniae	Blood culture	A	Y
Klebsiella pneumoniae	Blood culture	A	Y
Enterobacter cloacae subsp cloacae	Blood culture	A	Y
Klebsiella pneumoniae subsp pneumoniae	Blood culture	A	Y
Klebsiella pneumoniae subsp pneumoniae	Blood culture	A	Y
Klebsiella pneumoniae	Blood culture	A	Y
Klebsiella pneumoniae	Blood culture	A	U
Klebsiella pneumoniae	Blood culture	A	Y
Citrobacter freundii	Blood culture	A	U
Klebsiella pneumoniae	Blood culture	A	U
Klebsiella pneumoniae	Blood culture	A	Y
Klebsiella pneumoniae subsp pneumoniae	Blood culture	A	Y
Escherichia coli	Blood culture	V	Y
Serratia marcescens	Blood culture	V	Y
Klebsiella pneumoniae	Blood culture	V	Y
Klebsiella pneumoniae subsp pneumoniae	Blood culture	V	Y
Klebsiella pneumoniae subsp pneumoniae	Blood culture	V	U
Citrobacter freundii	Blood culture	V	Y
Klebsiella pneumoniae	Blood culture	V	Y
Klebsiella pneumoniae subsp pneumoniae	Blood culture	V	Y
Klebsiella pneumoniae subsp pneumoniae	Blood culture	V	Y
Klebsiella pneumoniae subsp pneumoniae	Blood culture	V	Y
Klebsiella pneumoniae	Blood culture	V	Y
Enterobacter cloacae	Blood culture	V	Y
Klebsiella pneumoniae subsp ozaenae	Blood culture	V	Y
Enterobacter cloacae	Blood culture	V	Y

Klebsiella pneumoniae	Blood culture	V	Y
Serratia marcescens	Blood culture	V	Y
Klebsiella pneumoniae	Blood culture	V	Y
Serratia marcescens	Blood culture	V	
Klebsiella pneumoniae	Blood culture	V	Y
Klebsiella pneumoniae subsp pneumoniae	Blood culture	V	Y
Serratia marcescens	Blood culture	V	Y
Klebsiella pneumoniae subsp pneumoniae	Blood culture	A	Y
Klebsiella pneumoniae	Blood culture	V	U
Klebsiella pneumoniae	Blood culture	V	
Klebsiella pneumoniae	Blood culture	V	U
Klebsiella pneumoniae	Blood culture	V	U
Klebsiella pneumoniae	Blood culture	V	
Klebsiella pneumoniae	Blood culture	V	U
Klebsiella pneumoniae	Blood culture	V	U
Klebsiella pneumoniae	Blood culture	V	U
Klebsiella pneumoniae	Blood culture	V	U
Klebsiella pneumoniae	Blood culture	V	U
Klebsiella pneumoniae	Blood culture	V	U
Klebsiella pneumoniae	Blood culture	V	Y
Enterobacter cloacae	Blood culture	V	U
Klebsiella pneumoniae subsp pneumoniae	Blood culture	V	Y
Klebsiella pneumoniae subsp ozaenae	Blood culture	V	N
Klebsiella pneumoniae	Blood culture	V	U
Klebsiella pneumoniae	Blood culture	V	U
Klebsiella pneumoniae	Blood culture	V	Y
Klebsiella pneumoniae	Blood culture	V	U
Klebsiella pneumoniae	Blood culture	V	U
Klebsiella pneumoniae	Blood culture	N	U
Klebsiella pneumoniae	Blood culture	V	Y
Klebsiella pneumoniae	Blood culture	V	N
Klebsiella pneumoniae	Blood culture	V	
Klebsiella pneumoniae subsp pneumoniae	Blood culture	V	Y
Proteus penneri	Blood culture	V	U
Enterobacter cloacae	Blood culture	V	Y
Klebsiella pneumoniae	Blood culture	V	Y
Klebsiella pneumoniae	Blood culture	V	Y
Klebsiella pneumoniae	Blood culture	V	Y
Klebsiella pneumoniae	Blood culture	V	Y
Klebsiella pneumoniae subsp pneumoniae	Blood culture	V	N
Enterobacter aerogenes	Blood culture	V	Y
Klebsiella pneumoniae	Blood culture	V	U
Enterobacter cloacae	Blood culture	V	Y
Klebsiella pneumoniae	Blood culture	V	Y
Klebsiella pneumoniae	Blood culture	V	Y
Klebsiella pneumoniae subsp pneumoniae	Blood culture	V	Y
Klebsiella pneumoniae	Blood culture	V	Y
Serratia marcescens	Blood culture	V	N
Klebsiella oxytoca	Blood culture	V	N
Klebsiella pneumoniae	Blood culture	V	Y
Klebsiella pneumoniae	Blood culture	V	Y
Klebsiella pneumoniae	Blood culture	V	Y
Klebsiella pneumoniae subsp pneumoniae	Blood culture	V	Y
Enterobacter cloacae	Blood culture	V	Y
Providencia rettgeri	Blood culture	V	N
Klebsiella pneumoniae subsp pneumoniae	Blood culture	V	Y

Klebsiella pneumoniae subsp pneumoniae	Blood culture	V	U
Klebsiella pneumoniae subsp pneumoniae	Blood culture	V	Y
Klebsiella pneumoniae subsp pneumoniae	Blood culture	V	Y
Serratia marcescens	Blood culture	V	Y
Enterobacter cloacae	Blood culture	V	Y
Klebsiella pneumoniae subsp pneumoniae	Blood culture	V	U
Klebsiella pneumoniae subsp pneumoniae	Blood culture	V	Y
Klebsiella pneumoniae subsp pneumoniae	Blood culture	V	Y
Klebsiella pneumoniae subsp pneumoniae	Blood culture	V	Y
Klebsiella pneumoniae subsp pneumoniae	Blood culture	V	Y
Klebsiella pneumoniae subsp pneumoniae	Blood culture	V	Y
Providencia stuartii	Blood culture	V	Y
Escherichia coli	Blood culture	V	Y
Enterobacter cloacae	Blood culture	V	Y
Klebsiella pneumoniae subsp pneumoniae	Blood culture	V	U
Enterobacter cloacae	Blood culture	V	Y
Klebsiella pneumoniae subsp pneumoniae	Blood culture	V	Y
Klebsiella pneumoniae	Blood culture	V	Y
Klebsiella pneumoniae subsp pneumoniae	Blood culture	V	Y
Proteus mirabilis	Blood culture	A	Y
Klebsiella pneumoniae	Blood culture	A	N
Klebsiella pneumoniae	Blood culture	A	N
Proteus mirabilis	Blood culture	A	Y
Klebsiella pneumoniae	Blood culture	A	Y
Klebsiella pneumoniae	Blood culture	A	Y
Klebsiella pneumoniae	Blood culture	V	Y
Klebsiella pneumoniae	Blood culture	A	Y
Klebsiella pneumoniae	Blood culture	A	Y
Klebsiella pneumoniae	Blood culture	A	N
Klebsiella pneumoniae	Blood culture	A	U
Klebsiella pneumoniae	Blood culture	A	Y
Escherichia coli	Blood culture	A	Y
Klebsiella pneumoniae subsp pneumoniae	Blood culture	V	Y
Klebsiella pneumoniae subsp pneumoniae	Blood culture	V	
Klebsiella pneumoniae subsp ozaenae	Blood culture	V	N
Klebsiella pneumoniae subsp pneumoniae	Blood culture	V	Y
Klebsiella pneumoniae subsp pneumoniae	Blood culture	V	Y
Klebsiella pneumoniae	Blood culture	V	Y
Klebsiella pneumoniae subsp pneumoniae	Blood culture	V	N
Klebsiella pneumoniae	Blood culture	V	U
Enterobacter cloacae	Blood culture	V	N
Klebsiella pneumoniae	Blood culture	V	Y
Klebsiella pneumoniae	Blood culture	V	Y
Klebsiella pneumoniae	Blood culture	V	U
Klebsiella oxytoca	Blood culture	V	U
Klebsiella pneumoniae	Blood culture	V	Y
Klebsiella pneumoniae subsp pneumoniae	Blood culture	V	
Klebsiella pneumoniae subsp pneumoniae	Blood culture	V	Y
Klebsiella pneumoniae subsp pneumoniae	Blood culture	V	Y
Klebsiella pneumoniae subsp pneumoniae	Blood culture	V	Y
Enterobacter cloacae	Blood culture	V	Y
Serratia marcescens	Blood culture	V	Y
Klebsiella pneumoniae subsp pneumoniae	Blood culture	V	Y
Escherichia coli	Blood culture	A	
Proteus mirabilis	Blood culture	A	
Klebsiella pneumoniae	Blood culture	A	Y

Klebsiella pneumoniae	Blood culture	A	Y
Proteus mirabilis	Blood culture	A	
Klebsiella pneumoniae subsp pneumoniae	Blood culture	A	U
Proteus mirabilis	Blood culture	A	U
Klebsiella pneumoniae	Blood culture	A	Y
Serratia marcescens	Blood culture	A	Y
Proteus mirabilis	Blood culture	A	N
Enterobacter cloacae subsp cloacae	Blood culture	A	U
Klebsiella pneumoniae subsp pneumoniae	Blood culture	A	Y
Klebsiella pneumoniae subsp pneumoniae	Blood culture	A	Y
Klebsiella pneumoniae subsp pneumoniae	Blood culture	A	Y
Klebsiella pneumoniae	Blood culture	A	Y
Proteus mirabilis	Blood culture	A	U
Klebsiella pneumoniae	Blood culture	A	Y
Proteus mirabilis	Blood culture	A	U
Klebsiella pneumoniae subsp pneumoniae	Blood culture	A	Y
Klebsiella pneumoniae subsp pneumoniae	Blood culture	A	U
Klebsiella pneumoniae subsp pneumoniae	Blood culture	A	Y
Klebsiella pneumoniae subsp pneumoniae	Blood culture	A	Y
Morganella morganii subsp morganii	Blood culture	A	U
Klebsiella pneumoniae subsp pneumoniae	Blood culture	A	Y
Klebsiella pneumoniae	Blood culture	A	Y
Klebsiella pneumoniae subsp pneumoniae	Blood culture	A	U
Klebsiella pneumoniae	Blood culture	A	Y
Klebsiella pneumoniae	Blood culture	A	U
Proteus mirabilis	Blood culture	A	Y
Proteus mirabilis	Blood culture	A	
Klebsiella pneumoniae subsp ozaenae	Blood culture	A	U
Proteus mirabilis	Blood culture	A	U
Proteus mirabilis	Blood culture	A	Y
Klebsiella pneumoniae subsp pneumoniae	Blood culture	A	N
Proteus mirabilis	Blood culture	A	Y
Klebsiella pneumoniae subsp pneumoniae	Blood culture	A	Y
Proteus penneri	Blood culture	A	Y
Klebsiella pneumoniae subsp pneumoniae	Blood culture	A	Y
Klebsiella pneumoniae subsp pneumoniae	Blood culture	A	Y
Klebsiella pneumoniae subsp pneumoniae	Blood culture	V	N
Klebsiella pneumoniae	Blood culture	A	
Morganella morganii	Blood culture	A	Y
Klebsiella pneumoniae subsp pneumoniae	Blood culture	A	Y
Klebsiella pneumoniae	Blood culture	A	Y
Klebsiella oxytoca	Blood culture	A	U
Klebsiella pneumoniae	Blood culture	A	Y
Proteus mirabilis	Blood culture	A	N
Klebsiella pneumoniae subsp ozaenae	Blood culture	A	
Klebsiella pneumoniae subsp pneumoniae	Blood culture	A	U
Klebsiella pneumoniae	Blood culture	A	Y
Klebsiella pneumoniae	Blood culture	A	U
Klebsiella pneumoniae subsp pneumoniae	Blood culture	V	Y
Klebsiella pneumoniae	Blood culture	A	
Klebsiella pneumoniae subsp pneumoniae	Blood culture	A	Y
Enterobacter cloacae	Blood culture	A	
Proteus mirabilis	Blood culture	A	Y
Klebsiella pneumoniae subsp pneumoniae	Blood culture	V	Y
Klebsiella pneumoniae	Blood culture	A	Y
Klebsiella pneumoniae	Blood culture	A	Y

Serratia marcescens	Blood culture	V	Y
Klebsiella pneumoniae	Blood culture	A	Y
Klebsiella pneumoniae	Blood culture	A	Y
Klebsiella pneumoniae	Blood culture	A	Y
Klebsiella pneumoniae	Blood culture	V	U
Klebsiella pneumoniae	Blood culture	A	Y
Klebsiella pneumoniae	Blood culture	A	Y
Klebsiella pneumoniae subsp pneumoniae	Blood culture	A	Y
Klebsiella pneumoniae	Blood culture	A	Y
Klebsiella pneumoniae	Blood culture	A	
Klebsiella pneumoniae subsp pneumoniae	Blood culture	V	Y
Klebsiella pneumoniae subsp pneumoniae	Blood culture	V	Y
Klebsiella pneumoniae subsp pneumoniae	Blood culture	V	Y
Klebsiella pneumoniae subsp pneumoniae	Blood culture	V	Y
Klebsiella pneumoniae	Blood culture	V	Y
Klebsiella pneumoniae subsp pneumoniae	Blood culture	V	Y
Klebsiella pneumoniae subsp pneumoniae	Blood culture	V	Y
Klebsiella pneumoniae subsp pneumoniae	Blood culture	V	Y
Klebsiella pneumoniae subsp pneumoniae	Blood culture	V	Y
Klebsiella pneumoniae subsp pneumoniae	Blood culture	V	Y
Klebsiella pneumoniae	Blood culture	V	Y
Enterobacter cloacae	Blood culture	V	Y
Klebsiella pneumoniae subsp pneumoniae	Blood culture	V	
Klebsiella pneumoniae subsp pneumoniae	Blood culture	V	Y
Klebsiella pneumoniae subsp pneumoniae	Blood culture	V	Y
Klebsiella pneumoniae subsp pneumoniae	Blood culture	V	Y
Klebsiella pneumoniae subsp pneumoniae	Blood culture	V	U
Klebsiella pneumoniae subsp pneumoniae	Blood culture	V	Y
Citrobacter freundii	Blood culture	V	Y
Klebsiella pneumoniae subsp pneumoniae	Blood culture	V	Y
Providencia rettgeri	Blood culture	V	Y
Klebsiella pneumoniae subsp pneumoniae	Blood culture	V	Y
Klebsiella pneumoniae subsp pneumoniae	Blood culture	V	Y
Klebsiella pneumoniae subsp pneumoniae	Blood culture	V	N
Klebsiella pneumoniae subsp ozaenae	Blood culture	V	
Klebsiella pneumoniae	Blood culture	V	Y
Klebsiella pneumoniae	Blood culture	V	Y
Klebsiella pneumoniae	Blood culture	V	Y
Klebsiella pneumoniae subsp pneumoniae	Blood culture	V	Y
Escherichia coli	Blood culture	V	U
Klebsiella pneumoniae subsp pneumoniae	Blood culture	V	Y
Klebsiella pneumoniae subsp pneumoniae	Blood culture	V	
Serratia marcescens	Blood culture	V	Y
Serratia marcescens	Blood culture	V	N
Klebsiella pneumoniae	Blood culture	V	Y
Klebsiella pneumoniae	Blood culture	V	Y
Klebsiella pneumoniae	Blood culture	V	Y
Klebsiella pneumoniae	Blood culture	V	Y
Klebsiella pneumoniae	Blood culture	V	Y
Klebsiella pneumoniae	Blood culture	V	N
Klebsiella pneumoniae	Blood culture	V	Y
Serratia marcescens	Blood culture	V	Y
Serratia marcescens	Blood culture	V	Y
Klebsiella pneumoniae subsp pneumoniae	Blood culture	V	U
Klebsiella pneumoniae	Blood culture	V	Y
Enterobacter aerogenes	Blood culture	V	Y

Klebsiella pneumoniae subsp pneumoniae	Blood culture	V	Y
Klebsiella pneumoniae	Blood culture	V	N
Klebsiella pneumoniae subsp pneumoniae	Blood culture	V	Y
Klebsiella pneumoniae subsp pneumoniae	Blood culture	V	Y
Klebsiella pneumoniae	Blood culture	V	Y
Escherichia coli	Blood culture	V	
Serratia marcescens	Blood culture	V	Y
Klebsiella pneumoniae subsp pneumoniae	Blood culture	V	Y
Klebsiella pneumoniae subsp pneumoniae	Blood culture	V	Y
Klebsiella pneumoniae subsp pneumoniae	Blood culture	V	
Serratia marcescens	Blood culture	V	Y
Klebsiella pneumoniae subsp pneumoniae	Blood culture	V	Y
Klebsiella pneumoniae subsp pneumoniae	Blood culture	V	Y
Klebsiella pneumoniae subsp pneumoniae	Blood culture	N	Y
Klebsiella pneumoniae subsp pneumoniae	Blood culture	V	Y
Klebsiella pneumoniae subsp pneumoniae	Blood culture	V	Y
Klebsiella pneumoniae subsp pneumoniae	Blood culture	V	Y
Klebsiella pneumoniae subsp pneumoniae	Blood culture	V	Y
Klebsiella pneumoniae subsp pneumoniae	Blood culture	V	N
Klebsiella pneumoniae subsp pneumoniae	Blood culture	V	Y
Enterobacter cloacae subsp cloacae	Blood culture	A	
Klebsiella pneumoniae subsp pneumoniae	Blood culture	A	
Enterobacter aerogenes	Blood culture	A	
Klebsiella pneumoniae subsp pneumoniae	Blood culture	A	
Klebsiella pneumoniae	Blood culture	A	U
Morganella morganii	Blood culture	A	
Klebsiella pneumoniae subsp pneumoniae	Blood culture	A	
Klebsiella pneumoniae subsp pneumoniae	Blood culture	A	
Klebsiella pneumoniae	Blood culture	A	
Enterobacter cloacae	Blood culture	A	
Klebsiella pneumoniae subsp pneumoniae	Blood culture	A	
Citrobacter freundii	Blood culture	A	U
Citrobacter freundii	Blood culture	A	U
Enterobacter species	Blood culture	A	
Serratia marcescens	Blood culture	A	
Klebsiella pneumoniae subsp pneumoniae	Blood culture	A	
Klebsiella pneumoniae	Blood culture	A	
Klebsiella pneumoniae subsp pneumoniae	Blood culture	A	
Klebsiella pneumoniae	Blood culture	A	
Klebsiella pneumoniae	Blood culture	A	
Escherichia coli	Blood culture	A	
Klebsiella pneumoniae	Blood culture	A	
Klebsiella pneumoniae	Blood culture	A	
Klebsiella pneumoniae	Blood culture	A	
Serratia marcescens	Blood culture	A	
Klebsiella pneumoniae subsp pneumoniae	Blood culture	A	
Klebsiella pneumoniae	Blood culture	A	
Klebsiella pneumoniae	Blood culture	A	
Klebsiella pneumoniae	Blood culture	A	Y
Enterobacter cloacae	Blood culture	A	
Klebsiella pneumoniae	Blood culture	A	Y
Serratia marcescens	Blood culture	A	
Klebsiella pneumoniae	Blood culture	A	Y
Klebsiella pneumoniae	Blood culture	A	Y
Klebsiella pneumoniae	Blood culture	A	Y
Klebsiella pneumoniae	Blood culture	A	U

Klebsiella pneumoniae	Blood culture	A	
Klebsiella pneumoniae	Blood culture	A	Y
Serratia marcescens	Blood culture	A	
Klebsiella pneumoniae	Blood culture	A	N
Enterobacter cloacae	Blood culture	A	U
Klebsiella pneumoniae subsp pneumoniae	Blood culture	A	
Klebsiella pneumoniae subsp pneumoniae	Blood culture	A	Y
Klebsiella pneumoniae	Blood culture	A	
Klebsiella pneumoniae	Blood culture	A	Y
Klebsiella pneumoniae subsp pneumoniae	Blood culture	A	
Klebsiella pneumoniae	Blood culture	A	
Klebsiella pneumoniae subsp pneumoniae	Blood culture	A	Y
Klebsiella pneumoniae	Blood culture	A	Y
Escherichia coli	Blood culture	A	
Klebsiella pneumoniae	Blood culture	A	U
Klebsiella pneumoniae subsp pneumoniae	Blood culture	A	
Klebsiella pneumoniae	Blood culture	A	Y
Klebsiella pneumoniae	Blood culture	A	Y
Klebsiella pneumoniae	Blood culture	A	Y
Enterobacter cloacae	Blood culture	A	
Klebsiella pneumoniae	Blood culture	A	U
Klebsiella pneumoniae subsp ozaenae	Blood culture	A	N
Klebsiella pneumoniae subsp pneumoniae	Blood culture	A	
Klebsiella pneumoniae subsp pneumoniae	Blood culture	A	Y
Klebsiella pneumoniae	Blood culture	A	
Klebsiella pneumoniae subsp pneumoniae	Blood culture	A	Y
Klebsiella pneumoniae	Blood culture	A	Y
Klebsiella pneumoniae subsp pneumoniae	Blood culture	A	
Klebsiella pneumoniae subsp pneumoniae	Blood culture	A	Y
Serratia marcescens	Blood culture	A	
Klebsiella pneumoniae	Blood culture	A	
Klebsiella pneumoniae subsp pneumoniae	Blood culture	A	
Klebsiella pneumoniae	Blood culture	A	
Klebsiella pneumoniae subsp pneumoniae	Blood culture	A	
Klebsiella pneumoniae	Blood culture	A	
Klebsiella pneumoniae subsp pneumoniae	Blood culture	A	
Klebsiella pneumoniae	Blood culture	A	Y
Klebsiella pneumoniae	Blood culture	A	Y
Klebsiella pneumoniae	Blood culture	A	N
Klebsiella pneumoniae	Blood culture	A	Y
Klebsiella pneumoniae	Blood culture	A	Y
Klebsiella pneumoniae	Blood culture	A	Y
Klebsiella pneumoniae subsp pneumoniae	Blood culture	A	U
Klebsiella pneumoniae	Blood culture	A	Y
Klebsiella pneumoniae	Blood culture	A	
Providencia rettgeri	Blood culture	A	N
Klebsiella pneumoniae	Blood culture	A	N
Escherichia coli	Blood culture	A	
Klebsiella pneumoniae	Blood culture	A	
Klebsiella pneumoniae	Blood culture	A	Y
Proteus mirabilis	Blood culture	A	Y
Klebsiella pneumoniae subsp pneumoniae	Blood culture	A	Y
Klebsiella pneumoniae	Blood culture	A	N
Klebsiella pneumoniae	Blood culture	A	
Klebsiella pneumoniae subsp pneumoniae	Blood culture	A	U
Enterobacter cloacae	Blood culture	A	Y

Escherichia coli	Blood culture	A	
Klebsiella pneumoniae subsp pneumoniae	Blood culture	A	N
Klebsiella pneumoniae subsp pneumoniae	Blood culture	A	Y
Klebsiella pneumoniae subsp pneumoniae	Blood culture	A	
Klebsiella oxytoca	Blood culture	V	
Klebsiella pneumoniae subsp pneumoniae	Blood culture	V	U
Enterobacter cloacae	Blood culture	V	Y
Klebsiella pneumoniae	Blood culture	V	Y
Klebsiella pneumoniae subsp pneumoniae	Blood culture	V	Y
Klebsiella oxytoca	Blood culture	V	U
Klebsiella pneumoniae subsp pneumoniae	Blood culture	V	Y
Klebsiella pneumoniae subsp pneumoniae	Blood culture	V	N
Klebsiella pneumoniae subsp pneumoniae	Blood culture	V	Y
Klebsiella pneumoniae subsp pneumoniae	Blood culture	V	Y
Klebsiella pneumoniae subsp pneumoniae	Blood culture	V	Y
Klebsiella pneumoniae subsp pneumoniae	Blood culture	V	Y
Klebsiella pneumoniae subsp pneumoniae	Blood culture	V	Y
Klebsiella pneumoniae subsp pneumoniae	Blood culture	V	Y
Proteus mirabilis	Blood culture	V	Y
Klebsiella pneumoniae	Blood culture	A	Y
Klebsiella pneumoniae	Blood culture	A	Y
Klebsiella pneumoniae subsp pneumoniae	Blood culture	A	Y
Klebsiella pneumoniae	Blood culture	A	U
Klebsiella pneumoniae	Blood culture	A	Y
Klebsiella pneumoniae	Blood culture	A	Y
Klebsiella pneumoniae	Blood culture	A	U
Klebsiella pneumoniae	Blood culture	A	Y
Klebsiella pneumoniae	Blood culture	A	Y
Klebsiella pneumoniae	Blood culture	A	N
Enterobacter cloacae	Blood culture	A	Y
Klebsiella pneumoniae	Blood culture	A	U
Klebsiella pneumoniae	Blood culture	A	Y
Klebsiella pneumoniae subsp pneumoniae	Blood culture	A	Y
Klebsiella pneumoniae	Blood culture	A	Y
Klebsiella pneumoniae	Blood culture	A	Y
Klebsiella pneumoniae subsp pneumoniae	Blood culture	V	Y
Klebsiella pneumoniae subsp pneumoniae	Blood culture	V	Y
Klebsiella pneumoniae subsp pneumoniae	Blood culture	M	Y
Klebsiella pneumoniae subsp pneumoniae	Blood culture	V	N
Klebsiella pneumoniae subsp pneumoniae	Blood culture	V	N
Proteus mirabilis	Blood culture	V	
Klebsiella pneumoniae subsp pneumoniae	Blood culture	V	Y
Klebsiella pneumoniae	Blood culture	V	Y
Klebsiella pneumoniae subsp pneumoniae	Blood culture	V	Y
Klebsiella pneumoniae subsp pneumoniae	Blood culture	V	Y
Klebsiella pneumoniae subsp pneumoniae	Blood culture	M	Y
Klebsiella pneumoniae	Blood culture	V	
Serratia marcescens	Blood culture	A	
Serratia marcescens	Blood culture	A	
Enterobacter cloacae complex	Blood culture	A	
Klebsiella oxytoca	Blood culture	A	N
Citrobacter freundii	Blood culture	A	Y
Serratia marcescens	Blood culture	A	Y
Enterobacter cloacae	Blood culture	A	N
Serratia marcescens	Blood culture	A	N
Enterobacter cloacae	Blood culture	A	

Enterobacter cloacae subsp dissolvens	Blood culture	A	
Serratia marcescens	Blood culture	A	Y
Enterobacter cloacae complex	Blood culture	A	Y
Enterobacter cloacae	Blood culture	A	Y
Enterobacter cloacae	Blood culture	A	Y
Providencia rettgeri	Blood culture	A	
Serratia marcescens	Blood culture	A	Y
Serratia marcescens	Blood culture	A	Y
Klebsiella pneumoniae	Blood culture	V	Y
Klebsiella pneumoniae	Blood culture	A	Y
Klebsiella pneumoniae	Blood culture	A	Y
Klebsiella pneumoniae	Blood culture	A	Y
Klebsiella pneumoniae	Blood culture	A	Y
Proteus mirabilis	Blood culture	A	Y
Klebsiella pneumoniae	Blood culture	A	Y
Klebsiella pneumoniae	Blood culture	A	Y
Klebsiella pneumoniae	Blood culture	A	Y
Klebsiella pneumoniae	Blood culture	A	Y
Klebsiella pneumoniae	Blood culture	A	Y
Klebsiella pneumoniae	Blood culture	A	Y
Klebsiella pneumoniae	Blood culture	A	Y
Enterobacter cloacae	Blood culture	A	N
Klebsiella pneumoniae	Blood culture	A	Y
Klebsiella pneumoniae	Blood culture	A	Y
Klebsiella pneumoniae	Blood culture	A	Y
Klebsiella pneumoniae	Blood culture	A	U
Enterobacter cloacae	Blood culture	A	Y
Klebsiella pneumoniae	Blood culture	A	Y
Klebsiella pneumoniae	Blood culture	A	Y
Klebsiella pneumoniae	Blood culture	A	Y
Klebsiella pneumoniae	Blood culture	A	Y
Klebsiella pneumoniae	Blood culture	A	Y
Klebsiella pneumoniae	Blood culture	A	Y
Klebsiella pneumoniae	Blood culture	A	N
Klebsiella pneumoniae	Blood culture	A	Y
Klebsiella pneumoniae	Blood culture	A	Y
Klebsiella pneumoniae	Blood culture	A	Y
Klebsiella pneumoniae	Blood culture	A	U
Klebsiella pneumoniae	Blood culture	A	N
Klebsiella pneumoniae	Blood culture	A	Y
Klebsiella pneumoniae	Blood culture	A	N
Klebsiella oxytoca	Blood culture	A	Y
Klebsiella pneumoniae	Blood culture	A	Y
Klebsiella pneumoniae	Blood culture	A	Y
Klebsiella pneumoniae	Blood culture	A	Y
Klebsiella pneumoniae	Blood culture	A	
Klebsiella pneumoniae	Blood culture	A	Y
Klebsiella pneumoniae	Blood culture	A	N
Klebsiella pneumoniae	Blood culture	A	Y
Klebsiella pneumoniae	Blood culture	A	Y
Klebsiella pneumoniae	Blood culture	A	U
Enterobacter cloacae	Blood culture	A	U
Klebsiella pneumoniae	Blood culture	A	Y
Klebsiella pneumoniae	Blood culture	A	Y
Klebsiella pneumoniae	Blood culture	A	U
Klebsiella pneumoniae	Blood culture	A	Y

Klebsiella pneumoniae	Blood culture	A	Y
Klebsiella pneumoniae	Blood culture	A	U
Enterobacter cloacae	Blood culture	A	N
Klebsiella pneumoniae subsp pneumoniae	Blood culture	A	
Enterobacter cloacae	Blood culture	A	
Enterobacter cloacae	Blood culture	A	Y
Enterobacter cloacae	Blood culture	A	
Enterobacter aerogenes	Blood culture	A	Y
Enterobacter cloacae	Blood culture	A	
Klebsiella pneumoniae subsp pneumoniae	Blood culture	A	Y
Klebsiella pneumoniae subsp pneumoniae	Blood culture	V	Y
Klebsiella pneumoniae	Blood culture	V	
Enterobacter cloacae	Blood culture	V	
Klebsiella pneumoniae	Blood culture	V	Y
Klebsiella pneumoniae subsp pneumoniae	Blood culture	V	Y
Klebsiella pneumoniae	Blood culture	V	
Enterobacter aerogenes	Blood culture	V	
Klebsiella pneumoniae subsp pneumoniae	Blood culture	V	Y
Klebsiella pneumoniae subsp pneumoniae	Blood culture	V	Y
Klebsiella pneumoniae subsp pneumoniae	Blood culture	V	Y
Klebsiella pneumoniae subsp pneumoniae	Blood culture	V	Y
Enterobacter cloacae	Blood culture	V	U
Klebsiella pneumoniae	Blood culture	V	
Klebsiella pneumoniae	Blood culture	V	Y
Klebsiella pneumoniae subsp pneumoniae	Blood culture	V	Y
Klebsiella pneumoniae subsp pneumoniae	Blood culture	V	Y
Klebsiella pneumoniae	Blood culture	V	
Klebsiella pneumoniae subsp pneumoniae	Blood culture	V	Y
Klebsiella pneumoniae subsp pneumoniae	Blood culture	V	Y
Klebsiella pneumoniae	Blood culture	V	Y
Klebsiella pneumoniae subsp pneumoniae	Blood culture	V	Y
Klebsiella pneumoniae	Blood culture	V	U
Klebsiella pneumoniae subsp pneumoniae	Blood culture	N	Y
Providencia stuartii	Blood culture	V	Y
Klebsiella pneumoniae subsp pneumoniae	Blood culture	V	Y
Klebsiella pneumoniae subsp pneumoniae	Blood culture	V	Y
Enterobacter aerogenes	Blood culture	V	Y
Providencia stuartii	Blood culture	V	
Klebsiella pneumoniae subsp pneumoniae	Blood culture	V	N
Klebsiella pneumoniae subsp pneumoniae	Blood culture	V	N
Klebsiella pneumoniae subsp pneumoniae	Blood culture	V	Y
Klebsiella pneumoniae subsp pneumoniae	Blood culture	V	Y
Klebsiella pneumoniae subsp pneumoniae	Blood culture	V	Y
Klebsiella pneumoniae subsp pneumoniae	Blood culture	V	Y
Klebsiella pneumoniae subsp pneumoniae	Blood culture	N	Y
Klebsiella pneumoniae subsp pneumoniae	Blood culture	V	N
Klebsiella pneumoniae subsp pneumoniae	Blood culture	V	Y
Klebsiella pneumoniae	Blood culture	V	Y
Klebsiella pneumoniae	Blood culture	V	Y
Klebsiella pneumoniae	Blood culture	V	
Klebsiella pneumoniae subsp pneumoniae	Blood culture	V	Y
Klebsiella pneumoniae subsp pneumoniae	Blood culture	V	Y
Klebsiella pneumoniae subsp pneumoniae	Blood culture	V	Y
Klebsiella pneumoniae subsp pneumoniae	Blood culture	V	Y
Klebsiella pneumoniae subsp pneumoniae	Blood culture	V	Y
Klebsiella pneumoniae subsp pneumoniae	Blood culture	V	Y

Klebsiella pneumoniae subsp pneumoniae	Blood culture	V	Y
Escherichia coli	Blood culture	V	Y
Proteus mirabilis	Blood culture	V	Y
Klebsiella pneumoniae subsp pneumoniae	Blood culture	V	Y
Klebsiella pneumoniae	Blood culture	V	Y
Klebsiella pneumoniae subsp pneumoniae	Blood culture	V	Y
Klebsiella pneumoniae subsp pneumoniae	Blood culture	V	Y
Escherichia coli	Blood culture	V	N
Klebsiella pneumoniae subsp pneumoniae	Blood culture	V	Y
Serratia marcescens	Blood culture	V	
Klebsiella pneumoniae subsp pneumoniae	Blood culture	V	Y
Klebsiella pneumoniae subsp pneumoniae	Blood culture	V	Y
Klebsiella pneumoniae subsp pneumoniae	Blood culture	V	N
Klebsiella pneumoniae subsp pneumoniae	Blood culture	V	Y
Enterobacter cloacae	Blood culture	V	U
Klebsiella pneumoniae	Blood culture	V	U
Klebsiella pneumoniae	Blood culture	V	Y
Klebsiella pneumoniae	Blood culture	V	
Klebsiella pneumoniae	Blood culture	V	
Enterobacter cloacae	Blood culture	V	Y
Klebsiella pneumoniae	Blood culture	V	U
Klebsiella pneumoniae	Blood culture	V	Y
Klebsiella oxytoca	Blood culture	A	
Klebsiella pneumoniae	Blood culture	V	
Serratia marcescens	Blood culture	V	Y
Klebsiella pneumoniae	Blood culture	V	
Klebsiella pneumoniae	Blood culture	V	
Klebsiella pneumoniae subsp pneumoniae	Blood culture	V	Y
Klebsiella pneumoniae subsp pneumoniae	Blood culture	V	Y
Klebsiella pneumoniae subsp pneumoniae	Blood culture	V	N
Klebsiella pneumoniae subsp pneumoniae	Blood culture	V	Y
Klebsiella pneumoniae subsp pneumoniae	Blood culture	V	N
Providencia stuartii	Blood culture	V	
Klebsiella pneumoniae subsp pneumoniae	Blood culture	V	Y
Serratia marcescens	Blood culture	V	Y
Klebsiella pneumoniae subsp pneumoniae	Blood culture	V	Y
Klebsiella pneumoniae	Blood culture	V	Y
Klebsiella pneumoniae	Blood culture	V	
Klebsiella pneumoniae subsp pneumoniae	Blood culture	V	Y
Klebsiella pneumoniae subsp pneumoniae	Blood culture	V	Y
Klebsiella pneumoniae subsp pneumoniae	Blood culture	V	N
Klebsiella pneumoniae subsp pneumoniae	Blood culture	V	Y
Serratia marcescens	Blood culture	V	
Klebsiella pneumoniae subsp pneumoniae	Blood culture	V	Y
Klebsiella pneumoniae subsp pneumoniae	Blood culture	V	Y
Klebsiella pneumoniae subsp pneumoniae	Blood culture	V	Y
Klebsiella pneumoniae subsp pneumoniae	Blood culture	V	Y
Klebsiella pneumoniae subsp pneumoniae	Blood culture	V	Y
Klebsiella pneumoniae subsp pneumoniae	Blood culture	V	Y
Enterobacter cloacae	Blood culture	V	Y
Serratia marcescens	Blood culture	V	Y
Klebsiella pneumoniae	Blood culture	V	
Klebsiella pneumoniae subsp pneumoniae	Blood culture	V	Y
Klebsiella pneumoniae subsp pneumoniae	Blood culture	V	Y
Klebsiella pneumoniae subsp pneumoniae	Blood culture	V	Y
Providencia rettgeri	Blood culture	V	Y

Klebsiella pneumoniae subsp pneumoniae	Blood culture	V	Y
Klebsiella pneumoniae subsp pneumoniae	Blood culture	V	Y
Klebsiella pneumoniae subsp pneumoniae	Blood culture	V	Y
Serratia marcescens	Blood culture	A	Y
Klebsiella pneumoniae subsp pneumoniae	Blood culture	N	N
Klebsiella pneumoniae subsp pneumoniae	Blood culture	V	Y
Klebsiella pneumoniae subsp pneumoniae	Blood culture	V	Y
Klebsiella pneumoniae subsp pneumoniae	Blood culture	V	Y
Klebsiella pneumoniae subsp pneumoniae	Blood culture	V	Y
Klebsiella pneumoniae subsp pneumoniae	Blood culture	V	
Klebsiella pneumoniae subsp pneumoniae	Blood culture	V	Y
Klebsiella pneumoniae subsp pneumoniae	Blood culture	V	N
Enterobacter cloacae	Blood culture	V	Y
Klebsiella pneumoniae subsp pneumoniae	Blood culture	V	
Klebsiella pneumoniae subsp pneumoniae	Blood culture	V	
Klebsiella pneumoniae subsp pneumoniae	Blood culture	V	Y
Klebsiella pneumoniae subsp ozaenae	Blood culture	V	Y
Klebsiella pneumoniae subsp pneumoniae	Blood culture	V	U
Klebsiella pneumoniae subsp pneumoniae	Blood culture	V	Y
Klebsiella pneumoniae subsp pneumoniae	Blood culture	V	
Klebsiella species	Blood culture	V	Y
Klebsiella pneumoniae subsp pneumoniae	Blood culture	V	N
Klebsiella pneumoniae subsp pneumoniae	Blood culture	V	Y
Serratia marcescens	Blood culture	V	Y
Serratia marcescens	Blood culture	V	Y
Klebsiella pneumoniae subsp pneumoniae	Blood culture	V	Y
Klebsiella pneumoniae subsp pneumoniae	Blood culture	V	Y
Enterobacter cloacae	Blood culture	V	N
Klebsiella pneumoniae subsp pneumoniae	Blood culture	V	Y
Klebsiella pneumoniae subsp pneumoniae	Blood culture	V	Y
Klebsiella pneumoniae subsp pneumoniae	Blood culture	V	
Escherichia coli	Blood culture	V	Y
Klebsiella pneumoniae subsp pneumoniae	Blood culture	V	N
Klebsiella pneumoniae subsp pneumoniae	Blood culture	V	Y
Klebsiella pneumoniae subsp pneumoniae	Blood culture	V	Y
Klebsiella pneumoniae subsp pneumoniae	Blood culture	V	Y
Klebsiella pneumoniae	Blood culture	V	Y
Serratia marcescens	Blood culture	V	Y
Klebsiella pneumoniae subsp pneumoniae	Blood culture	V	Y
Providencia rettgeri	Blood culture	V	Y
Escherichia coli	Blood culture	V	Y
Escherichia coli	Blood culture	V	Y
Klebsiella pneumoniae subsp pneumoniae	Blood culture	V	N
Klebsiella pneumoniae subsp pneumoniae	Blood culture	V	U
Klebsiella pneumoniae subsp pneumoniae	Blood culture	V	Y
Klebsiella pneumoniae subsp pneumoniae	Blood culture	V	Y
Klebsiella pneumoniae subsp pneumoniae	Blood culture	V	
Klebsiella pneumoniae subsp pneumoniae	Blood culture	V	Y
Klebsiella pneumoniae subsp pneumoniae	Blood culture	A	Y
Klebsiella pneumoniae subsp pneumoniae	Blood culture	V	Y
Serratia marcescens	Blood culture	V	Y
Klebsiella pneumoniae subsp pneumoniae	Blood culture	V	N
Klebsiella pneumoniae subsp pneumoniae	Blood culture	V	
Klebsiella pneumoniae subsp pneumoniae	Blood culture	V	Y
Klebsiella pneumoniae	Blood culture	V	Y
Klebsiella pneumoniae subsp pneumoniae	Blood culture	V	Y

Klebsiella pneumoniae subsp pneumoniae	Blood culture	V	Y
Klebsiella pneumoniae subsp pneumoniae	Blood culture	V	Y
Serratia marcescens	Blood culture	V	N
Klebsiella pneumoniae subsp pneumoniae	Blood culture	V	Y
Serratia marcescens	Blood culture	V	Y
Klebsiella pneumoniae subsp pneumoniae	Blood culture	V	Y
Klebsiella pneumoniae subsp pneumoniae	Blood culture	V	N
Escherichia coli	Blood culture	V	N
Enterobacter cloacae	Blood culture	N	N
Serratia marcescens	Blood culture	V	Y
Klebsiella pneumoniae subsp pneumoniae	Blood culture	V	Y
Enterobacter aerogenes	Blood culture	V	N
Klebsiella pneumoniae subsp pneumoniae	Blood culture	V	N
Enterobacter cloacae	Blood culture	V	Y
Klebsiella pneumoniae subsp pneumoniae	Blood culture	V	N
Klebsiella pneumoniae subsp pneumoniae	Blood culture	V	Y
Serratia marcescens	Blood culture	V	N
Klebsiella pneumoniae subsp pneumoniae	Blood culture	V	Y
Morganella morganii	Blood culture	V	Y
Klebsiella pneumoniae	Blood culture	V	N
Klebsiella pneumoniae subsp pneumoniae	Blood culture	V	Y
Serratia liquefaciens	Blood culture	V	Y
Klebsiella pneumoniae subsp pneumoniae	Blood culture	V	U
Klebsiella pneumoniae subsp pneumoniae	Blood culture	V	Y
Klebsiella pneumoniae subsp pneumoniae	Blood culture	V	Y
Klebsiella pneumoniae subsp pneumoniae	Blood culture	V	Y
Citrobacter freundii	Blood culture	V	Y
Enterobacter cloacae	Blood culture	V	N
Klebsiella pneumoniae subsp pneumoniae	Blood culture	V	Y
Klebsiella pneumoniae subsp pneumoniae	Blood culture	V	Y
Serratia marcescens	Blood culture	V	Y
Klebsiella pneumoniae subsp pneumoniae	Blood culture	V	
Klebsiella pneumoniae subsp pneumoniae	Blood culture	V	
Klebsiella pneumoniae subsp pneumoniae	Blood culture	V	Y
Serratia marcescens	Blood culture	V	Y
Klebsiella pneumoniae subsp pneumoniae	Blood culture	V	Y
Serratia marcescens	Blood culture	V	Y
Serratia marcescens	Blood culture	V	
Serratia marcescens	Blood culture	V	Y
Klebsiella pneumoniae subsp pneumoniae	Blood culture	V	Y
Klebsiella pneumoniae subsp pneumoniae	Blood culture	V	N
Klebsiella pneumoniae subsp pneumoniae	Blood culture	V	Y
Klebsiella pneumoniae subsp pneumoniae	Blood culture	V	Y
Klebsiella pneumoniae subsp pneumoniae	Blood culture	V	Y
Klebsiella pneumoniae subsp pneumoniae	Blood culture	V	Y
Klebsiella pneumoniae subsp pneumoniae	Blood culture	V	Y
Serratia marcescens	Blood culture	V	
Klebsiella pneumoniae subsp pneumoniae	Blood culture	B/L	Y
Klebsiella pneumoniae subsp pneumoniae	Blood culture	M	Y
Klebsiella pneumoniae subsp pneumoniae	Blood culture	V	Y
Klebsiella pneumoniae subsp pneumoniae	Blood culture	V	Y
Klebsiella pneumoniae subsp ozaenae	Blood culture	V	Y
Klebsiella pneumoniae subsp pneumoniae	Blood culture	V	Y
Klebsiella pneumoniae subsp pneumoniae	Blood culture	V	Y
Klebsiella pneumoniae subsp pneumoniae	Blood culture	V	Y
Klebsiella pneumoniae	Blood culture	A	

Klebsiella pneumoniae	Blood culture	A	
Klebsiella pneumoniae subsp pneumoniae	Blood culture	A	
Klebsiella pneumoniae	Blood culture	A	Y
Klebsiella pneumoniae subsp pneumoniae	Blood culture	A	Y
Klebsiella pneumoniae	Blood culture	A	Y
Serratia marcescens	Blood culture	A	Y
Klebsiella pneumoniae	Blood culture	A	Y
Klebsiella pneumoniae	Blood culture	A	Y
Klebsiella pneumoniae	Blood culture	A	Y
Klebsiella pneumoniae subsp pneumoniae	Blood culture	A	Y
Klebsiella pneumoniae subsp pneumoniae	Blood culture	A	Y
Enterobacter cloacae	Blood culture	A	Y
Klebsiella pneumoniae	Blood culture	A	U
Klebsiella pneumoniae	Blood culture	A	
Klebsiella pneumoniae subsp pneumoniae	Blood culture	A	Y
Klebsiella pneumoniae	Blood culture	A	Y
Klebsiella pneumoniae	Blood culture	A	
Serratia marcescens	Blood culture	A	Y
Klebsiella pneumoniae	Blood culture	A	
Serratia marcescens	Blood culture	A	Y
Klebsiella pneumoniae	Blood culture	A	N
Klebsiella pneumoniae	Blood culture	A	Y
Klebsiella pneumoniae	Blood culture	A	
Klebsiella pneumoniae	Blood culture	A	Y
Klebsiella pneumoniae	Blood culture	A	Y
Klebsiella oxytoca	Blood culture	A	Y
Escherichia coli	Blood culture	A	Y
Klebsiella pneumoniae subsp pneumoniae	Blood culture	A	Y
Klebsiella pneumoniae subsp pneumoniae	Blood culture	A	
Klebsiella pneumoniae subsp pneumoniae	Blood culture	A	Y
Klebsiella pneumoniae subsp pneumoniae	Blood culture	A	Y
Klebsiella pneumoniae subsp pneumoniae	Blood culture	A	Y
Klebsiella pneumoniae	Blood culture	A	Y
Klebsiella pneumoniae	Blood culture	A	Y
Klebsiella pneumoniae	Blood culture	A	Y
Klebsiella pneumoniae	Blood culture	A	N
Klebsiella pneumoniae subsp pneumoniae	Blood culture	A	
Klebsiella pneumoniae subsp pneumoniae	Blood culture	A	Y
Klebsiella pneumoniae	Blood culture	A	Y
Klebsiella pneumoniae	Blood culture	A	
Klebsiella pneumoniae	Blood culture	A	Y
Escherichia coli	Blood culture	A	N
Klebsiella pneumoniae subsp pneumoniae	Blood culture	A	Y
Klebsiella pneumoniae	Blood culture	A	Y
Serratia marcescens	Blood culture	A	N
Klebsiella pneumoniae	Blood culture	A	Y
Klebsiella pneumoniae	Blood culture	A	Y
Klebsiella pneumoniae	Blood culture	A	U
Klebsiella pneumoniae	Blood culture	A	Y
Klebsiella pneumoniae	Blood culture	A	Y
Proteus mirabilis	Blood culture	A	Y
Klebsiella pneumoniae	Blood culture	A	Y
Klebsiella pneumoniae	Blood culture	A	N
Klebsiella pneumoniae subsp pneumoniae	Blood culture	A	Y
Klebsiella pneumoniae subsp pneumoniae	Blood culture	A	
Klebsiella pneumoniae	Blood culture	A	Y

Klebsiella pneumoniae	Blood culture	A	U
Enterobacter cloacae complex	Blood culture	A	Y
Enterobacter cloacae	Blood culture	A	N
Serratia marcescens	Blood culture	A	Y
Klebsiella pneumoniae subsp pneumoniae	Blood culture	A	Y
Klebsiella pneumoniae	Blood culture	A	Y
Klebsiella pneumoniae	Blood culture	A	N
Klebsiella pneumoniae	Blood culture	A	U
Klebsiella pneumoniae	Blood culture	A	U
Enterobacter cloacae complex	Blood culture	A	
Klebsiella pneumoniae	Blood culture	A	Y
Klebsiella pneumoniae	Blood culture	A	Y
Klebsiella pneumoniae subsp pneumoniae	Blood culture	A	Y
Klebsiella pneumoniae subsp pneumoniae	Blood culture	A	Y
Klebsiella pneumoniae	Blood culture	A	Y
Klebsiella pneumoniae	Blood culture	A	Y
Klebsiella pneumoniae subsp pneumoniae	Blood culture	A	
Klebsiella pneumoniae subsp pneumoniae	Blood culture	A	Y
Klebsiella pneumoniae subsp pneumoniae	Blood culture	A	
Enterobacter cloacae	Blood culture	A	Y
Klebsiella pneumoniae	Blood culture	A	Y
Klebsiella pneumoniae	Blood culture	A	Y
Klebsiella pneumoniae	Blood culture	A	
Klebsiella pneumoniae	Blood culture	A	
Klebsiella pneumoniae	Blood culture	A	Y
Klebsiella pneumoniae subsp pneumoniae	Blood culture	A	Y
Klebsiella pneumoniae	Blood culture	A	
Klebsiella pneumoniae	Blood culture	A	N
Serratia marcescens	Blood culture	A	
Klebsiella pneumoniae	Blood culture	A	Y
Klebsiella pneumoniae subsp pneumoniae	Blood culture	A	Y
Klebsiella pneumoniae	Blood culture	A	
Klebsiella pneumoniae	Blood culture	A	Y
Klebsiella pneumoniae	Blood culture	A	U
Klebsiella pneumoniae	Blood culture	A	
Klebsiella pneumoniae subsp pneumoniae	Blood culture	A	Y
Enterobacter cloacae	Blood culture	A	U
Klebsiella pneumoniae	Blood culture	A	Y
Klebsiella pneumoniae	Blood culture	A	U
Morganella morganii subsp morganii	Blood culture	A	
Klebsiella pneumoniae	Blood culture	A	
Klebsiella oxytoca	Blood culture	A	U
Serratia marcescens	Blood culture	A	Y
Klebsiella pneumoniae subsp pneumoniae	Blood culture	A	
Escherichia coli	Blood culture	A	Y
Klebsiella pneumoniae	Blood culture	A	Y
Klebsiella pneumoniae	Blood culture	A	Y
Klebsiella pneumoniae	Blood culture	A	Y
Klebsiella oxytoca	Blood culture	A	N
Klebsiella pneumoniae subsp pneumoniae	Blood culture	V	Y
Providencia rettgeri	Blood culture	A	Y
Klebsiella pneumoniae subsp pneumoniae	Blood culture	B/L	Y
Klebsiella pneumoniae	Blood culture	A	Y
Klebsiella pneumoniae	Blood culture	A	U
Klebsiella pneumoniae	Blood culture	A	Y
Klebsiella pneumoniae subsp pneumoniae	Blood culture	A	Y

Klebsiella pneumoniae	Blood culture	A	Y
Providencia rettgeri	Blood culture	A	U
Klebsiella pneumoniae	Blood culture	A	U
Klebsiella pneumoniae	Blood culture	A	N
Klebsiella pneumoniae subsp pneumoniae	Blood culture	A	Y
Serratia marcescens	Blood culture	A	
Klebsiella pneumoniae	Blood culture	A	
Klebsiella pneumoniae	Blood culture	A	Y
Klebsiella pneumoniae subsp pneumoniae	Blood culture	A	Y
Klebsiella pneumoniae	Blood culture	A	Y
Klebsiella pneumoniae	Blood culture	A	Y
Klebsiella pneumoniae	Blood culture	A	Y
Serratia marcescens	Blood culture	N	Y
Klebsiella pneumoniae subsp pneumoniae	Blood culture	V	
Klebsiella pneumoniae	Blood culture	V	U
Klebsiella pneumoniae subsp pneumoniae	Blood culture	V	Y
Klebsiella pneumoniae subsp pneumoniae	Blood culture	V	Y
Klebsiella pneumoniae subsp pneumoniae	Blood culture	V	Y
Klebsiella pneumoniae subsp pneumoniae	Blood culture	V	Y
Klebsiella pneumoniae subsp pneumoniae	Blood culture	V	Y
Klebsiella pneumoniae subsp pneumoniae	Blood culture	V	Y
Klebsiella pneumoniae subsp pneumoniae	Blood culture	V	Y
Serratia marcescens	Blood culture	V	N
Klebsiella pneumoniae subsp pneumoniae	Blood culture	V	Y
Klebsiella pneumoniae subsp pneumoniae	Blood culture	V	U
Klebsiella pneumoniae subsp pneumoniae	Blood culture	V	Y
Enterobacter cloacae	Blood culture	V	
Escherichia coli	Blood culture	M	Y
Klebsiella pneumoniae	Blood culture	M	Y
Klebsiella pneumoniae	Blood culture	M	Y
Klebsiella pneumoniae	Blood culture	M	
Klebsiella pneumoniae	Blood culture	M	N
Salmonella non-typhi	Blood culture	M	Y
Morganella morganii	Blood culture	M	Y
Klebsiella pneumoniae	Blood culture	M	Y
Klebsiella pneumoniae	Blood culture	M	Y
Enterobacter cloacae	Blood culture	M	Y
Klebsiella pneumoniae	Blood culture	M	Y
Klebsiella pneumoniae	Blood culture	M	Y
Klebsiella pneumoniae	Blood culture	M	Y
Klebsiella pneumoniae	Blood culture	M	U
Klebsiella pneumoniae	Blood culture	M	Y
Klebsiella pneumoniae	Blood culture	M	Y
Escherichia coli	Blood culture	M	Y
Klebsiella pneumoniae	Blood culture	M	N
Klebsiella pneumoniae	Blood culture	M	Y
Enterobacter cloacae	Blood culture	M	Y
Klebsiella pneumoniae	Blood culture	M	N
Klebsiella pneumoniae	Blood culture	M	Y
Klebsiella pneumoniae	Blood culture	M	Y
Klebsiella pneumoniae	Blood culture	M	Y
Serratia marcescens	Blood culture	M	N
Klebsiella pneumoniae	Blood culture	M	Y
Klebsiella pneumoniae	Blood culture	M	Y
Klebsiella pneumoniae	Blood culture	M	Y
Klebsiella pneumoniae	Blood culture	M	Y

Enterobacter cloacae complex	Blood culture	M	N
Enterobacter cloacae	Blood culture	M	Y
Klebsiella pneumoniae	Blood culture	M	Y
Klebsiella pneumoniae	Blood culture	M	Y
Klebsiella pneumoniae	Blood culture	M	Y
Serratia marcescens	Blood culture	M	Y
Klebsiella pneumoniae	Blood culture	M	N
Klebsiella pneumoniae	Blood culture	A	Y
Klebsiella pneumoniae	Blood culture	M	Y
Klebsiella pneumoniae	Blood culture	M	Y
Klebsiella pneumoniae	Blood culture	M	
Klebsiella pneumoniae	Blood culture	M	Y
Klebsiella pneumoniae	Blood culture	M	N
Klebsiella pneumoniae	Blood culture	M	Y
Klebsiella pneumoniae subsp pneumoniae	Blood culture	V	Y
Klebsiella pneumoniae	Blood culture	M	Y
Klebsiella pneumoniae	Blood culture	M	Y
Klebsiella pneumoniae	Blood culture	M	Y
Serratia marcescens	Blood culture	M	N
Klebsiella pneumoniae	Blood culture	M	Y
Escherichia coli	Blood culture	M	Y
Klebsiella pneumoniae	Blood culture	M	Y
Klebsiella pneumoniae subsp pneumoniae	Blood culture	V	Y
Klebsiella oxytoca	Blood culture	M	Y
Klebsiella pneumoniae	Blood culture	M	Y
Klebsiella pneumoniae	Blood culture	M	N
Klebsiella pneumoniae	Blood culture	M	Y
Klebsiella pneumoniae	Blood culture	M	Y
Klebsiella pneumoniae	Blood culture	M	Y
Klebsiella pneumoniae	Blood culture	M	
Klebsiella pneumoniae	Blood culture	M	N
Klebsiella pneumoniae	Blood culture	M	Y
Klebsiella pneumoniae	Blood culture	M	Y
Klebsiella pneumoniae	Blood culture	M	Y
Klebsiella pneumoniae	Blood culture	V	Y
Klebsiella pneumoniae	Blood culture	M	
Enterobacter cloacae	Blood culture	M	Y
Escherichia coli	Blood culture	M	Y
Klebsiella pneumoniae subsp pneumoniae	Blood culture	V	
Citrobacter freundii	Blood culture	V	N
Klebsiella pneumoniae subsp pneumoniae	Blood culture	V	Y
Morganella morganii	Blood culture	V	Y
Klebsiella pneumoniae subsp pneumoniae	Blood culture	V	Y
Klebsiella pneumoniae	Blood culture	V	Y
Enterobacter cloacae	Blood culture	V	
Providencia stuartii	Blood culture	V	Y
Klebsiella pneumoniae	Blood culture	V	U
Enterobacter cloacae	Blood culture	V	Y
Klebsiella pneumoniae subsp pneumoniae	Blood culture	V	Y
Klebsiella pneumoniae subsp pneumoniae	Blood culture	V	Y
Enterobacter cloacae	Blood culture	V	Y
Klebsiella pneumoniae	Blood culture	V	N
Klebsiella pneumoniae subsp pneumoniae	Blood culture	V	Y
Klebsiella pneumoniae	Blood culture	V	U
Klebsiella pneumoniae	Blood culture	V	Y
Serratia marcescens	Blood culture	V	Y

Klebsiella pneumoniae subsp pneumoniae	Blood culture	V	Y
Klebsiella pneumoniae subsp pneumoniae	Blood culture	V	Y
Klebsiella pneumoniae subsp pneumoniae	Blood culture	V	Y
Klebsiella pneumoniae subsp pneumoniae	Blood culture	V	Y
Klebsiella pneumoniae subsp pneumoniae	Blood culture	V	N
Klebsiella pneumoniae subsp pneumoniae	Blood culture	V	
Escherichia coli	Blood culture	V	Y
Serratia marcescens	Blood culture	V	Y
Klebsiella pneumoniae subsp pneumoniae	Blood culture	V	Y
Klebsiella pneumoniae subsp pneumoniae	Blood culture	V	Y
Klebsiella pneumoniae subsp pneumoniae	Blood culture	V	Y
Klebsiella pneumoniae subsp pneumoniae	Blood culture	V	Y
Serratia marcescens	Blood culture	V	Y
Klebsiella pneumoniae subsp pneumoniae	Blood culture	V	Y
Klebsiella pneumoniae	Blood culture	V	Y
Klebsiella pneumoniae subsp pneumoniae	Blood culture	V	Y
Escherichia coli	Blood culture	V	Y
Klebsiella pneumoniae subsp pneumoniae	Blood culture	V	Y
Klebsiella pneumoniae	Blood culture	V	N
Klebsiella pneumoniae subsp pneumoniae	Blood culture	V	N
Klebsiella pneumoniae subsp pneumoniae	Blood culture	V	Y
Klebsiella pneumoniae subsp pneumoniae	Blood culture	V	Y
Klebsiella pneumoniae subsp pneumoniae	Blood culture	V	N
Klebsiella pneumoniae subsp pneumoniae	Blood culture	V	Y
Klebsiella pneumoniae subsp pneumoniae	Blood culture	V	Y
Klebsiella pneumoniae	Blood culture	V	Y
Klebsiella pneumoniae	Blood culture	V	Y
Klebsiella pneumoniae	Blood culture	V	Y
Enterobacter cloacae	Blood culture	V	
Klebsiella pneumoniae subsp pneumoniae	Blood culture	V	Y
Klebsiella pneumoniae subsp pneumoniae	Blood culture	V	
Klebsiella pneumoniae subsp pneumoniae	Blood culture	V	Y
Klebsiella pneumoniae subsp pneumoniae	Blood culture	V	Y
Klebsiella pneumoniae subsp pneumoniae	Blood culture	V	Y
Klebsiella pneumoniae subsp pneumoniae	Blood culture	V	N
Klebsiella pneumoniae subsp pneumoniae	Blood culture	V	N
Klebsiella pneumoniae subsp pneumoniae	Blood culture	V	Y
Klebsiella pneumoniae	Blood culture	V	Y
Klebsiella pneumoniae subsp pneumoniae	Blood culture	V	Y
Klebsiella pneumoniae subsp pneumoniae	Blood culture	V	Y
Klebsiella pneumoniae subsp pneumoniae	Blood culture	V	Y
Klebsiella pneumoniae subsp pneumoniae	Blood culture	V	Y
Klebsiella pneumoniae subsp pneumoniae	Blood culture	V	Y
Serratia marcescens	Blood culture	V	Y
Klebsiella pneumoniae subsp pneumoniae	Blood culture	V	Y
Klebsiella pneumoniae subsp pneumoniae	Blood culture	V	Y
Klebsiella pneumoniae subsp pneumoniae	Blood culture	V	Y
Klebsiella pneumoniae subsp pneumoniae	Blood culture	V	Y
Klebsiella pneumoniae subsp pneumoniae	Blood culture	V	Y
Klebsiella pneumoniae	Blood culture	V	U
Klebsiella pneumoniae	Blood culture	V	Y
Klebsiella pneumoniae	Blood culture	V	
Klebsiella pneumoniae subsp pneumoniae	Blood culture	V	Y
Klebsiella pneumoniae subsp pneumoniae	Blood culture	V	Y
Klebsiella pneumoniae	Blood culture	V	Y
Klebsiella pneumoniae	Blood culture	A	Y

Proteus mirabilis	Blood culture	A	
Klebsiella pneumoniae	Blood culture	V	U
Klebsiella pneumoniae	Blood culture	A	U
Klebsiella pneumoniae	Blood culture	A	U
Klebsiella pneumoniae	Blood culture	A	U
Klebsiella pneumoniae	Blood culture	A	U
Proteus mirabilis	Blood culture	A	Y
Klebsiella pneumoniae	Blood culture	A	Y
Klebsiella pneumoniae subsp pneumoniae	Blood culture	A	N
Enterobacter aerogenes	Blood culture	A	Y
Klebsiella pneumoniae	Blood culture	A	U
Klebsiella pneumoniae	Blood culture	A	U
Klebsiella pneumoniae subsp pneumoniae	Blood culture	A	Y
Klebsiella pneumoniae	Blood culture	A	Y
Klebsiella pneumoniae subsp pneumoniae	Blood culture	A	Y
Klebsiella pneumoniae	Blood culture	A	Y
Klebsiella pneumoniae	Blood culture	A	Y
Citrobacter freundii	Blood culture	A	Y
Klebsiella pneumoniae	Blood culture	A	U
Serratia marcescens	Blood culture	A	Y
Klebsiella pneumoniae subsp pneumoniae	Blood culture	A	Y
Klebsiella pneumoniae	Blood culture	A	U
Klebsiella pneumoniae	Blood culture	A	U
Klebsiella pneumoniae subsp pneumoniae	Blood culture	A	Y
Klebsiella pneumoniae	Blood culture	A	U
Enterobacter cloacae complex	Blood culture	A	Y
Klebsiella pneumoniae subsp pneumoniae	Blood culture	A	Y
Klebsiella pneumoniae	Blood culture	A	U
Klebsiella pneumoniae subsp pneumoniae	Blood culture	A	Y
Klebsiella pneumoniae subsp pneumoniae	Blood culture	A	Y
Klebsiella pneumoniae	Blood culture	A	
Klebsiella pneumoniae	Blood culture	A	Y
Enterobacter cloacae complex	Blood culture	A	Y
Klebsiella pneumoniae subsp pneumoniae	Blood culture	A	Y
Klebsiella pneumoniae	Blood culture	A	U
Klebsiella pneumoniae	Blood culture	A	N
Klebsiella pneumoniae	Blood culture	V	U
Klebsiella pneumoniae	Blood culture	A	U
Klebsiella pneumoniae subsp pneumoniae	Blood culture	A	Y
Klebsiella pneumoniae subsp pneumoniae	Blood culture	A	Y
Klebsiella pneumoniae subsp pneumoniae	Blood culture	A	
Klebsiella pneumoniae	Blood culture	A	U
Klebsiella pneumoniae subsp pneumoniae	Blood culture	A	Y
Escherichia coli	Blood culture	A	Y
Klebsiella pneumoniae	Blood culture	A	U
Klebsiella pneumoniae	Blood culture	A	U
Klebsiella pneumoniae	Blood culture	A	U
Klebsiella pneumoniae	Blood culture	A	U
Klebsiella pneumoniae subsp pneumoniae	Blood culture	A	Y
Klebsiella pneumoniae subsp pneumoniae	Blood culture	A	Y
Klebsiella pneumoniae subsp pneumoniae	Blood culture	A	Y
Klebsiella pneumoniae	Blood culture	A	U
Klebsiella pneumoniae	Blood culture	A	Y
Klebsiella pneumoniae	Blood culture	A	
Klebsiella pneumoniae	Blood culture	A	Y
Klebsiella pneumoniae subsp pneumoniae	Blood culture	A	Y

Enterobacter cloacae complex	Blood culture	A	Y
Klebsiella pneumoniae	Blood culture	A	U
Klebsiella pneumoniae subsp pneumoniae	Blood culture	A	Y
Klebsiella pneumoniae	Blood culture	A	Y
Klebsiella pneumoniae subsp pneumoniae	Blood culture	A	Y
Klebsiella pneumoniae subsp pneumoniae	Blood culture	A	Y
Serratia marcescens	Blood culture	A	Y
Klebsiella pneumoniae	Blood culture	V	Y
Klebsiella pneumoniae subsp pneumoniae	Blood culture	A	Y
Klebsiella pneumoniae	Blood culture	V	U
Klebsiella pneumoniae subsp pneumoniae	Blood culture	A	Y
Serratia marcescens	Blood culture	A	Y
Klebsiella pneumoniae	Blood culture	A	Y
Klebsiella pneumoniae	Blood culture	A	Y
Klebsiella pneumoniae	Blood culture	A	U
Klebsiella pneumoniae subsp pneumoniae	Blood culture	A	Y
Klebsiella pneumoniae	Blood culture	A	Y
Enterobacter cloacae subsp cloacae	Blood culture	A	N
Klebsiella pneumoniae	Blood culture	A	Y
Klebsiella pneumoniae subsp pneumoniae	Blood culture	A	Y
Klebsiella pneumoniae subsp pneumoniae	Blood culture	A	
Klebsiella pneumoniae	Blood culture	A	U
Klebsiella pneumoniae subsp pneumoniae	Blood culture	A	Y
Enterobacter aerogenes	Blood culture	A	Y
Enterobacter cloacae complex	Blood culture	A	
Klebsiella pneumoniae subsp pneumoniae	Blood culture	A	Y
Klebsiella pneumoniae	Blood culture	A	
Klebsiella pneumoniae subsp pneumoniae	Blood culture	A	Y
Klebsiella pneumoniae	Blood culture	A	U
Klebsiella pneumoniae	Blood culture	A	Y
Citrobacter freundii	Blood culture	A	Y
Escherichia coli	Blood culture	A	Y
Klebsiella pneumoniae	Blood culture	A	Y
Klebsiella pneumoniae	Blood culture	A	Y
Klebsiella pneumoniae subsp pneumoniae	Blood culture	A	N
Klebsiella pneumoniae subsp pneumoniae	Blood culture	A	Y
Klebsiella pneumoniae subsp pneumoniae	Blood culture	A	N
Klebsiella pneumoniae subsp pneumoniae	Blood culture	A	Y
Escherichia coli	Blood culture	A	Y
Klebsiella pneumoniae subsp pneumoniae	Blood culture	A	Y
Klebsiella pneumoniae subsp pneumoniae	Blood culture	A	Y
Klebsiella pneumoniae	Blood culture	V	N
Klebsiella pneumoniae	Blood culture	V	U
Klebsiella pneumoniae	Blood culture	N	
Klebsiella pneumoniae subsp pneumoniae	Blood culture	V	Y
Klebsiella pneumoniae	Blood culture	V	
Enterobacter cloacae	Blood culture	N	
Klebsiella pneumoniae	Blood culture	M	Y
Klebsiella pneumoniae	Blood culture	M	N
Klebsiella pneumoniae	Blood culture	M	
Enterobacter cloacae	Blood culture	M	
Escherichia coli	Blood culture	M	Y
Klebsiella pneumoniae	Blood culture	M	N
Klebsiella pneumoniae	Blood culture	M	Y
Escherichia coli	Blood culture	M	
Proteus vulgaris	Blood culture	M	

Morganella morganii	Blood culture	M	
Klebsiella pneumoniae	Blood culture	M	Y
Enterobacter cloacae	Blood culture	M	Y
Klebsiella pneumoniae subsp pneumoniae	Blood culture	V	Y
Escherichia coli	Blood culture	M	Y
Klebsiella pneumoniae subsp pneumoniae	Blood culture	V	Y
Klebsiella pneumoniae subsp pneumoniae	Blood culture	V	Y
Klebsiella pneumoniae	Blood culture	V	Y
Klebsiella oxytoca	Blood culture	N	Y
Klebsiella pneumoniae	Blood culture	V	Y
Klebsiella pneumoniae	Blood culture	V	Y
Klebsiella pneumoniae	Blood culture	V	Y
Klebsiella pneumoniae subsp pneumoniae	Blood culture	V	N
Klebsiella pneumoniae	Blood culture	V	Y
Klebsiella pneumoniae	Blood culture	V	Y
Klebsiella pneumoniae subsp pneumoniae	Blood culture	V	Y
Klebsiella pneumoniae subsp pneumoniae	Blood culture	V	Y
Klebsiella pneumoniae subsp pneumoniae	Blood culture	V	Y
Klebsiella pneumoniae	Blood culture	V	
Klebsiella pneumoniae subsp pneumoniae	Blood culture	V	N
Klebsiella pneumoniae	Blood culture	V	
Klebsiella pneumoniae subsp pneumoniae	Blood culture	V	N
Serratia marcescens	Blood culture	V	N
Klebsiella pneumoniae subsp pneumoniae	Blood culture	V	Y
Escherichia coli	Blood culture	V	N
Klebsiella pneumoniae	Blood culture	V	N
Klebsiella pneumoniae	Blood culture	V	
Enterobacter cloacae	Blood culture	V	Y
Enterobacter cloacae	Blood culture	V	
Klebsiella pneumoniae subsp pneumoniae	Blood culture	V	Y
Escherichia coli	Blood culture	V	Y
Enterobacter cloacae	Blood culture	V	
Klebsiella pneumoniae subsp pneumoniae	Blood culture	V	Y
Klebsiella pneumoniae subsp pneumoniae	Blood culture	V	Y
Klebsiella pneumoniae subsp pneumoniae	Blood culture	V	N
Enterobacter cloacae	Blood culture	V	Y
Klebsiella pneumoniae subsp pneumoniae	Blood culture	V	Y
Klebsiella pneumoniae	Blood culture	V	Y
Klebsiella pneumoniae	Blood culture	V	
Klebsiella pneumoniae	Blood culture	V	
Klebsiella pneumoniae	Blood culture	V	Y
Klebsiella pneumoniae subsp pneumoniae	Blood culture	V	Y
Klebsiella pneumoniae subsp pneumoniae	Blood culture	V	Y
Klebsiella pneumoniae	Blood culture	V	
Klebsiella pneumoniae subsp pneumoniae	Blood culture	V	Y
Klebsiella pneumoniae	Blood culture	V	
Klebsiella pneumoniae	Blood culture	V	Y
Klebsiella pneumoniae subsp pneumoniae	Blood culture	V	Y
Klebsiella pneumoniae subsp pneumoniae	Blood culture	V	Y
Enterobacter cloacae	Blood culture	V	Y
Klebsiella pneumoniae	Blood culture	V	Y
Klebsiella pneumoniae subsp pneumoniae	Blood culture	V	Y
Klebsiella pneumoniae	Blood culture	V	Y
Klebsiella pneumoniae	Blood culture	V	Y
Enterobacter cloacae	Blood culture	V	Y
Klebsiella pneumoniae subsp pneumoniae	Blood culture	V	Y

Enterobacter cloacae	Blood culture	V	Y
Klebsiella pneumoniae subsp pneumoniae	Blood culture	V	N
Klebsiella pneumoniae	Blood culture	M	N
Klebsiella pneumoniae subsp pneumoniae	Blood culture	V	Y
Klebsiella pneumoniae subsp pneumoniae	Blood culture	V	Y
Klebsiella pneumoniae subsp pneumoniae	Blood culture	V	N
Enterobacter cloacae subsp cloacae	Blood culture	A	Y
Proteus mirabilis	Blood culture	A	
Enterobacter cloacae subsp cloacae	Blood culture	A	
Enterobacter aerogenes	Blood culture	A	Y
Serratia fonticola	Blood culture	A	Y
Klebsiella pneumoniae subsp pneumoniae	Blood culture	A	
Enterobacter cloacae complex	Blood culture	A	
Klebsiella pneumoniae	Blood culture	A	
Enterobacter aerogenes	Blood culture	A	
Enterobacter aerogenes	Blood culture	A	
Enterobacter cloacae	Blood culture	A	
Escherichia coli	Blood culture	A	
Enterobacter aerogenes	Blood culture	A	N
Escherichia coli	Blood culture	A	
Enterobacter aerogenes	Blood culture	A	
Klebsiella pneumoniae subsp pneumoniae	Blood culture	A	N
Klebsiella pneumoniae subsp pneumoniae	Blood culture	A	
Klebsiella pneumoniae subsp pneumoniae	Blood culture	A	Y
Klebsiella pneumoniae	Blood culture	A	
Klebsiella pneumoniae subsp pneumoniae	Blood culture	A	
Klebsiella pneumoniae	Blood culture	A	
Enterobacter cloacae	Blood culture	A	
Klebsiella pneumoniae subsp pneumoniae	Blood culture	A	Y
Klebsiella pneumoniae	Blood culture	A	
Klebsiella pneumoniae	Blood culture	A	
Enterobacter cloacae	Blood culture	A	Y
Klebsiella pneumoniae	Blood culture	A	Y
Klebsiella pneumoniae subsp pneumoniae	Blood culture	A	Y
Klebsiella pneumoniae subsp pneumoniae	Blood culture	A	U
Klebsiella pneumoniae subsp pneumoniae	Blood culture	A	
Klebsiella pneumoniae subsp pneumoniae	Blood culture	A	Y
Klebsiella pneumoniae subsp pneumoniae	Blood culture	A	Y
Klebsiella pneumoniae subsp pneumoniae	Blood culture	A	
Klebsiella pneumoniae subsp pneumoniae	Blood culture	A	Y
Klebsiella pneumoniae subsp pneumoniae	Blood culture	A	Y
Klebsiella pneumoniae subsp pneumoniae	Blood culture	A	Y
Klebsiella pneumoniae subsp pneumoniae	Blood culture	A	Y
Escherichia coli	Blood culture	A	Y
Serratia fonticola	Blood culture	A	Y
Proteus mirabilis	Blood culture	A	
Klebsiella pneumoniae subsp pneumoniae	Blood culture	A	Y
Klebsiella pneumoniae	Blood culture	A	Y
Klebsiella pneumoniae subsp pneumoniae	Blood culture	A	Y
Klebsiella pneumoniae	Blood culture	A	Y
Klebsiella pneumoniae	Blood culture	A	Y
Klebsiella pneumoniae subsp pneumoniae	Blood culture	A	
Klebsiella pneumoniae subsp pneumoniae	Blood culture	A	Y
Klebsiella pneumoniae subsp pneumoniae	Blood culture	A	
Klebsiella pneumoniae subsp pneumoniae	Blood culture	A	N
Klebsiella pneumoniae subsp pneumoniae	Blood culture	A	Y

Enterobacter aerogenes	Blood culture	A	
Klebsiella pneumoniae	Blood culture	A	
Klebsiella pneumoniae subsp pneumoniae	Blood culture	A	Y
Klebsiella pneumoniae	Blood culture	A	
Enterobacter cloacae subsp dissolvens	Blood culture	A	Y
Klebsiella pneumoniae	Blood culture	A	Y
Klebsiella pneumoniae	Blood culture	A	Y
Klebsiella pneumoniae	Blood culture	A	
Klebsiella pneumoniae	Blood culture	A	N
Enterobacter cloacae	Blood culture	A	Y
Klebsiella pneumoniae subsp pneumoniae	Blood culture	A	Y
Klebsiella pneumoniae subsp pneumoniae	Blood culture	A	Y
Klebsiella pneumoniae	Blood culture	A	Y
Serratia marcescens	Blood culture	A	
Klebsiella pneumoniae	Blood culture	A	
Klebsiella pneumoniae subsp pneumoniae	Blood culture	A	Y
Klebsiella pneumoniae	Blood culture	A	
Klebsiella pneumoniae subsp ozaenae	Blood culture	A	
Klebsiella pneumoniae subsp pneumoniae	Blood culture	A	Y
Klebsiella oxytoca	Blood culture	A	N
Klebsiella pneumoniae subsp pneumoniae	Blood culture	A	Y
Klebsiella pneumoniae subsp pneumoniae	Blood culture	A	Y
Klebsiella pneumoniae subsp pneumoniae	Blood culture	A	Y
Klebsiella pneumoniae	Blood culture	A	Y
Klebsiella oxytoca	Blood culture	A	Y
Klebsiella pneumoniae	Blood culture	A	Y
Klebsiella pneumoniae	Blood culture	A	
Klebsiella pneumoniae	Blood culture	A	Y
Klebsiella pneumoniae subsp pneumoniae	Blood culture	A	N
Klebsiella pneumoniae subsp pneumoniae	Blood culture	A	
Klebsiella pneumoniae	Blood culture	A	
Klebsiella pneumoniae	Blood culture	A	
Klebsiella pneumoniae subsp pneumoniae	Blood culture	A	Y
Klebsiella pneumoniae subsp pneumoniae	Blood culture	A	Y
Klebsiella pneumoniae	Blood culture	A	
Klebsiella pneumoniae subsp pneumoniae	Blood culture	A	Y
Klebsiella pneumoniae subsp pneumoniae	Blood culture	A	Y
Klebsiella pneumoniae subsp pneumoniae	Blood culture	A	Y
Proteus mirabilis	Blood culture	A	
Klebsiella pneumoniae	Blood culture	A	Y
Klebsiella pneumoniae	Blood culture	A	Y
Klebsiella pneumoniae subsp pneumoniae	Blood culture	A	Y
Klebsiella pneumoniae	Blood culture	A	Y
Escherichia coli	Blood culture	A	N
Escherichia coli	Blood culture	A	Y
Klebsiella pneumoniae	Blood culture	A	
Klebsiella pneumoniae subsp pneumoniae	Blood culture	A	Y
Klebsiella pneumoniae	Blood culture	A	Y
Klebsiella pneumoniae	Blood culture	A	
Klebsiella pneumoniae	Blood culture	A	Y
Enterobacter cloacae	Blood culture	A	
Klebsiella pneumoniae	Blood culture	A	Y
Klebsiella pneumoniae	Blood culture	A	Y
Klebsiella pneumoniae	Blood culture	A	N
Klebsiella pneumoniae subsp pneumoniae	Blood culture	A	Y
Escherichia coli	Blood culture	A	Y

Klebsiella pneumoniae	Blood culture	A	
Klebsiella pneumoniae	Blood culture	A	Y
Serratia marcescens	Blood culture	A	Y
Klebsiella pneumoniae	Blood culture	A	Y
Klebsiella pneumoniae subsp pneumoniae	Blood culture	A	Y
Klebsiella pneumoniae subsp pneumoniae	Blood culture	A	Y
Escherichia coli	Blood culture	A	
Serratia marcescens	Blood culture	A	
Klebsiella pneumoniae	Blood culture	A	
Enterobacter cloacae subsp cloacae	Blood culture	A	Y
Enterobacter cloacae	Blood culture	A	
Klebsiella pneumoniae	Blood culture	A	Y
Enterobacter aerogenes	Blood culture	A	
Klebsiella pneumoniae	Blood culture	A	Y
Klebsiella pneumoniae	Blood culture	A	
Enterobacter cloacae	Blood culture	A	
Serratia marcescens	Blood culture	A	Y
Serratia marcescens	Blood culture	A	Y
Klebsiella pneumoniae subsp pneumoniae	Blood culture	A	Y
Klebsiella pneumoniae	Blood culture	A	Y
Klebsiella oxytoca	Blood culture	A	Y
Klebsiella pneumoniae	Blood culture	A	
Klebsiella pneumoniae	Blood culture	A	U
Klebsiella oxytoca	Blood culture	A	
Klebsiella pneumoniae	Blood culture	A	Y
Klebsiella pneumoniae	Blood culture	A	N
Klebsiella pneumoniae subsp pneumoniae	Blood culture	A	Y
Klebsiella pneumoniae	Blood culture	A	U
Klebsiella pneumoniae	Blood culture	A	N
Klebsiella pneumoniae subsp pneumoniae	Blood culture	A	Y
Klebsiella pneumoniae subsp pneumoniae	Blood culture	V	Y
Citrobacter freundii	Blood culture	V	Y
Klebsiella pneumoniae	Blood culture	V	Y
Klebsiella pneumoniae subsp pneumoniae	Blood culture	V	Y
Escherichia coli	Blood culture	V	
Escherichia coli	Blood culture	V	
Klebsiella pneumoniae subsp pneumoniae	Blood culture	V	Y
Klebsiella pneumoniae subsp pneumoniae	Blood culture	V	Y
Klebsiella pneumoniae	Blood culture	M	Y
Klebsiella pneumoniae subsp pneumoniae	Blood culture	M	N
Escherichia coli	Blood culture	M	Y
Klebsiella pneumoniae	Blood culture	M	Y
Klebsiella pneumoniae subsp pneumoniae	Blood culture	V	Y
Klebsiella pneumoniae	Blood culture	M	Y
Klebsiella pneumoniae	Blood culture	M	Y
Klebsiella pneumoniae	Blood culture	M	U
Klebsiella pneumoniae	Blood culture	M	Y
Klebsiella pneumoniae subsp pneumoniae	Blood culture	V	
Klebsiella pneumoniae	Blood culture	V	
Klebsiella pneumoniae	Blood culture	V	Y
Klebsiella pneumoniae subsp pneumoniae	Blood culture	V	Y
Klebsiella pneumoniae	Blood culture	V	Y
Klebsiella pneumoniae subsp pneumoniae	Blood culture	V	
Klebsiella pneumoniae subsp pneumoniae	Blood culture	V	Y
Klebsiella pneumoniae subsp pneumoniae	Blood culture	V	Y
Klebsiella pneumoniae subsp pneumoniae	Blood culture	V	Y

Klebsiella pneumoniae subsp pneumoniae	Blood culture	V	Y
Enterobacter cloacae	Blood culture	V	U
Klebsiella pneumoniae	Blood culture	V	
Klebsiella pneumoniae	Blood culture	V	U
Klebsiella pneumoniae subsp pneumoniae	Blood culture	V	Y
Klebsiella pneumoniae	Blood culture	V	U
Klebsiella pneumoniae subsp pneumoniae	Blood culture	V	Y
Enterobacter aerogenes	Blood culture	V	Y
Klebsiella pneumoniae subsp pneumoniae	Blood culture	V	Y
Klebsiella pneumoniae subsp pneumoniae	Blood culture	V	Y
Klebsiella pneumoniae subsp pneumoniae	Blood culture	V	N
Klebsiella oxytoca	Blood culture	V	Y
Klebsiella pneumoniae subsp pneumoniae	Blood culture	V	Y
Klebsiella pneumoniae subsp pneumoniae	Blood culture	V	Y
Klebsiella pneumoniae subsp pneumoniae	Blood culture	V	Y
Klebsiella pneumoniae subsp pneumoniae	Blood culture	V	Y
Enterobacter cloacae	Blood culture	V	Y
Klebsiella pneumoniae subsp pneumoniae	Blood culture	V	Y
Klebsiella pneumoniae subsp pneumoniae	Blood culture	V	Y
Klebsiella pneumoniae subsp pneumoniae	Blood culture	V	Y
Klebsiella pneumoniae subsp pneumoniae	Blood culture	V	Y
Klebsiella pneumoniae subsp pneumoniae	Blood culture	V	Y
Klebsiella pneumoniae subsp pneumoniae	Blood culture	N	Y
Klebsiella pneumoniae subsp pneumoniae	Blood culture	V	Y
Klebsiella pneumoniae subsp pneumoniae	Blood culture	V	Y
Serratia marcescens	Blood culture	V	N
Klebsiella pneumoniae subsp pneumoniae	Blood culture	V	N
Klebsiella pneumoniae	Blood culture	V	
Klebsiella pneumoniae subsp pneumoniae	Blood culture	V	Y
Klebsiella pneumoniae	Blood culture	V	
Klebsiella pneumoniae	Blood culture	V	
Klebsiella pneumoniae subsp pneumoniae	Blood culture	V	Y
Klebsiella pneumoniae subsp pneumoniae	Blood culture	V	Y
Klebsiella pneumoniae subsp pneumoniae	Blood culture	V	Y
Klebsiella pneumoniae subsp pneumoniae	Blood culture	V	Y
Klebsiella pneumoniae subsp pneumoniae	Blood culture	V	Y
Klebsiella pneumoniae subsp pneumoniae	Blood culture	V	Y
Klebsiella pneumoniae subsp pneumoniae	Blood culture	V	Y
Klebsiella pneumoniae subsp pneumoniae	Blood culture	V	
Klebsiella pneumoniae	Blood culture	V	
Klebsiella pneumoniae subsp pneumoniae	Blood culture	V	
Klebsiella oxytoca	Blood culture	V	Y
Enterobacter aerogenes	Blood culture	V	U
Klebsiella pneumoniae	Blood culture	V	U
Klebsiella pneumoniae	Blood culture	V	
Klebsiella pneumoniae	Blood culture	V	U
Klebsiella pneumoniae	Blood culture	V	
Klebsiella pneumoniae	Blood culture	V	U
Klebsiella pneumoniae	Blood culture	V	N
Klebsiella pneumoniae	Blood culture	V	N
Klebsiella pneumoniae	Blood culture	V	Y
Klebsiella pneumoniae	Blood culture	V	Y
Klebsiella pneumoniae	Blood culture	V	Y
Klebsiella pneumoniae	Blood culture	V	U
Klebsiella pneumoniae	Blood culture	V	Y
Klebsiella pneumoniae	Blood culture	V	Y

Enterobacter cloacae	Blood culture	V	
Klebsiella pneumoniae	Blood culture	V	Y
Enterobacter hormaechei	Blood culture	V	Y
Enterobacter cloacae	Blood culture	V	Y
Serratia marcescens	Blood culture	A	
Enterobacter cloacae subsp cloacae	Blood culture	A	
Enterobacter cloacae complex	Blood culture	A	Y
Enterobacter cloacae	Blood culture	A	U
Enterobacter cloacae	Blood culture	A	Y
Enterobacter cloacae	Blood culture	A	Y
Serratia marcescens	Blood culture	A	U
Enterobacter cloacae	Blood culture	A	Y
Enterobacter cloacae	Blood culture	A	N
Serratia marcescens	Blood culture	A	N
Enterobacter cloacae	Blood culture	A	U
Citrobacter freundii	Blood culture	A	
Enterobacter cloacae	Blood culture	A	
Enterobacter cloacae subsp cloacae	Blood culture	A	
Klebsiella oxytoca	Blood culture	A	
Enterobacter cloacae	Blood culture	A	
Enterobacter cloacae	Blood culture	A	U
Providencia stuartii	Blood culture	A	Y
Serratia marcescens	Blood culture	A	Y
Serratia marcescens	Blood culture	A	Y
Klebsiella pneumoniae	Blood culture	M	Y
Klebsiella pneumoniae	Blood culture	M	Y
Klebsiella pneumoniae	Blood culture	M	N
Klebsiella pneumoniae	Blood culture	M	Y
Klebsiella pneumoniae	Blood culture	M	Y
Klebsiella pneumoniae	Blood culture	M	Y
Klebsiella pneumoniae	Blood culture	M	Y
Klebsiella pneumoniae	Blood culture	M	Y
Klebsiella pneumoniae	Blood culture	M	Y
Klebsiella pneumoniae	Blood culture	M	Y
Klebsiella pneumoniae	Blood culture	M	N
Klebsiella pneumoniae	Blood culture	M	Y
Klebsiella pneumoniae	Blood culture	M	Y
Klebsiella pneumoniae	Blood culture	M	Y
Klebsiella pneumoniae	Blood culture	M	N
Klebsiella pneumoniae	Blood culture	M	Y
Klebsiella pneumoniae	Blood culture	M	Y
Serratia marcescens	Blood culture	M	N
Klebsiella pneumoniae	Blood culture	M	U
Klebsiella pneumoniae	Blood culture	M	Y
Klebsiella pneumoniae	Blood culture	M	Y
Proteus mirabilis	Blood culture	M	Y
Klebsiella pneumoniae	Blood culture	M	Y
Klebsiella pneumoniae	Blood culture	M	Y
Klebsiella pneumoniae	Blood culture	M	Y
Klebsiella pneumoniae	Blood culture	M	Y
Klebsiella pneumoniae	Blood culture	M	Y
Klebsiella pneumoniae	Blood culture	M	N
Proteus mirabilis	Blood culture	M	Y
Klebsiella pneumoniae	Blood culture	M	Y
Klebsiella pneumoniae	Blood culture	M	N
Klebsiella pneumoniae	Blood culture	M	Y

Klebsiella pneumoniae	Blood culture	M	Y
Klebsiella pneumoniae	Blood culture	M	Y
Enterobacter cloacae	Blood culture	M	Y
Klebsiella pneumoniae	Blood culture	M	Y
Klebsiella pneumoniae	Blood culture	M	Y
Enterobacter aerogenes	Blood culture	M	Y
Klebsiella pneumoniae	Blood culture	M	Y
Klebsiella pneumoniae	Blood culture	M	N
Klebsiella pneumoniae	Blood culture	M	Y
Klebsiella pneumoniae	Blood culture	M	N
Klebsiella pneumoniae	Blood culture	M	Y
Klebsiella pneumoniae	Blood culture	M	Y
Enterobacter aerogenes	Blood culture	A	Y
Klebsiella pneumoniae subsp pneumoniae	Blood culture	A	Y
Proteus mirabilis	Blood culture	A	Y
Klebsiella pneumoniae	Blood culture	A	Y
Klebsiella pneumoniae subsp pneumoniae	Blood culture	A	N
Klebsiella pneumoniae subsp pneumoniae	Blood culture	A	N
Escherichia coli	Blood culture	A	
Klebsiella pneumoniae subsp pneumoniae	Blood culture	A	
Klebsiella pneumoniae subsp pneumoniae	Blood culture	A	
Klebsiella pneumoniae subsp pneumoniae	Blood culture	A	
Klebsiella pneumoniae subsp pneumoniae	Blood culture	A	
Klebsiella pneumoniae subsp pneumoniae	Blood culture	A	
Klebsiella pneumoniae subsp pneumoniae	Blood culture	B/L	
Klebsiella pneumoniae subsp pneumoniae	Blood culture	B/L	
Escherichia coli	Blood culture	B/L	
Enterobacter aerogenes	Blood culture	A	
Klebsiella pneumoniae	Blood culture	A	
Klebsiella pneumoniae	Blood culture	A	
Citrobacter freundii	Blood culture	A	
Enterobacter cloacae subsp cloacae	Blood culture	A	
Klebsiella pneumoniae subsp pneumoniae	Blood culture	A	
Enterobacter cloacae	Blood culture	A	
Klebsiella pneumoniae subsp pneumoniae	Blood culture	A	
Escherichia coli	Blood culture	A	
Klebsiella pneumoniae	Blood culture	A	
Klebsiella pneumoniae subsp pneumoniae	Blood culture	A	
Klebsiella pneumoniae	Blood culture	A	
Klebsiella pneumoniae subsp pneumoniae	Blood culture	A	
Klebsiella pneumoniae subsp pneumoniae	Blood culture	A	
Klebsiella pneumoniae subsp pneumoniae	Blood culture	A	
Klebsiella pneumoniae subsp pneumoniae	Blood culture	A	
Enterobacter cloacae subsp cloacae	Blood culture	A	
Enterobacter cloacae complex	Blood culture	A	
Klebsiella pneumoniae	Blood culture	A	
Klebsiella pneumoniae	Blood culture	A	
Klebsiella pneumoniae	Blood culture	A	
Klebsiella pneumoniae	Blood culture	A	
Klebsiella pneumoniae	Blood culture	A	
Klebsiella pneumoniae	Blood culture	A	
Klebsiella pneumoniae	Blood culture	A	
Klebsiella pneumoniae	Blood culture	A	
Enterobacter cloacae complex	Blood culture	A	
Klebsiella pneumoniae subsp pneumoniae	Blood culture	A	
Klebsiella pneumoniae subsp pneumoniae	Blood culture	A	

Klebsiella pneumoniae subsp pneumoniae	Blood culture	A	
Klebsiella pneumoniae subsp pneumoniae	Blood culture	A	
Klebsiella pneumoniae subsp pneumoniae	Blood culture	A	
Enterobacter cloacae subsp dissolvens	Blood culture	A	
Klebsiella pneumoniae subsp pneumoniae	Blood culture	A	
Klebsiella pneumoniae subsp pneumoniae	Blood culture	A	
Enterobacter aerogenes	Blood culture	A	
Escherichia coli	Blood culture	A	
Escherichia coli	Blood culture	A	
Klebsiella pneumoniae	Blood culture	A	
Klebsiella pneumoniae	Blood culture	A	
Klebsiella pneumoniae	Blood culture	A	
Klebsiella pneumoniae subsp pneumoniae	Blood culture	A	
Klebsiella pneumoniae	Blood culture	A	
Klebsiella pneumoniae	Blood culture	A	
Klebsiella pneumoniae subsp pneumoniae	Blood culture	A	
Klebsiella pneumoniae subsp pneumoniae	Blood culture	A	
Enterobacter cloacae	Blood culture	A	
Klebsiella pneumoniae	Blood culture	A	
Klebsiella pneumoniae	Blood culture	A	
Klebsiella pneumoniae subsp pneumoniae	Blood culture	A	
Enterobacter cloacae complex	Blood culture	A	
Klebsiella pneumoniae subsp pneumoniae	Blood culture	A	
Klebsiella pneumoniae	Blood culture	A	
Enterobacter cloacae complex	Blood culture	A	
Klebsiella pneumoniae subsp pneumoniae	Blood culture	A	
Klebsiella pneumoniae	Blood culture	A	
Klebsiella pneumoniae	Blood culture	A	
Klebsiella pneumoniae subsp pneumoniae	Blood culture	A	
Klebsiella pneumoniae subsp pneumoniae	Blood culture	A	
Proteus mirabilis	Blood culture	A	
Klebsiella pneumoniae subsp pneumoniae	Blood culture	A	
Enterobacter aerogenes	Blood culture	A	
Klebsiella pneumoniae	Blood culture	A	
Klebsiella pneumoniae	Blood culture	A	
Klebsiella pneumoniae	Blood culture	A	
Klebsiella pneumoniae	Blood culture	A	
Klebsiella pneumoniae	Blood culture	A	
Klebsiella pneumoniae	Blood culture	A	
Klebsiella pneumoniae subsp pneumoniae	Blood culture	A	
Enterobacter cloacae subsp cloacae	Blood culture	A	
Serratia plymuthica	Blood culture	A	
Enterobacter cloacae complex	Blood culture	A	
Klebsiella pneumoniae	Blood culture	A	
Klebsiella pneumoniae	Blood culture	A	
Klebsiella pneumoniae	Blood culture	A	
Klebsiella pneumoniae	Blood culture	A	
Klebsiella pneumoniae	Blood culture	A	
Klebsiella pneumoniae	Blood culture	A	
Klebsiella pneumoniae subsp pneumoniae	Blood culture	V	
Klebsiella pneumoniae subsp pneumoniae	Blood culture	V	N
Klebsiella pneumoniae subsp pneumoniae	Blood culture	V	N
Klebsiella pneumoniae subsp pneumoniae	Blood culture	V	
Klebsiella pneumoniae subsp pneumoniae	Blood culture	V	Y
Escherichia coli	Blood culture	V	
Klebsiella pneumoniae subsp pneumoniae	Blood culture	V	Y

Klebsiella pneumoniae subsp pneumoniae	Blood culture	V	
Klebsiella pneumoniae	Blood culture	V	Y
Klebsiella pneumoniae subsp ozaenae	Blood culture	V	Y
Klebsiella pneumoniae	Blood culture	V	Y
Klebsiella pneumoniae	Blood culture	V	
Klebsiella pneumoniae subsp pneumoniae	Blood culture	V	N
Klebsiella pneumoniae subsp pneumoniae	Blood culture	V	Y
Klebsiella pneumoniae	Blood culture	V	
Klebsiella pneumoniae subsp pneumoniae	Blood culture	V	Y
Klebsiella pneumoniae subsp pneumoniae	Blood culture	V	N
Klebsiella pneumoniae subsp pneumoniae	Blood culture	V	Y
Serratia marcescens	Blood culture	V	Y
Klebsiella pneumoniae subsp pneumoniae	Blood culture	V	Y
Klebsiella pneumoniae subsp pneumoniae	Blood culture	V	Y
Klebsiella pneumoniae subsp pneumoniae	Blood culture	V	N
Klebsiella pneumoniae subsp pneumoniae	Blood culture	V	Y
Escherichia coli	Blood culture	V	Y
Klebsiella pneumoniae subsp pneumoniae	Blood culture	V	Y
Serratia marcescens	Blood culture	V	Y
Klebsiella pneumoniae	Blood culture	V	
Klebsiella pneumoniae subsp pneumoniae	Blood culture	V	N
Klebsiella pneumoniae subsp pneumoniae	Blood culture	V	Y
Klebsiella pneumoniae subsp pneumoniae	Blood culture	V	Y
Klebsiella pneumoniae subsp pneumoniae	Blood culture	V	Y
Klebsiella pneumoniae	Blood culture	V	Y
Klebsiella pneumoniae	Blood culture	V	Y
Klebsiella pneumoniae	Blood culture	V	
Enterobacter cloacae	Blood culture	V	Y
Klebsiella pneumoniae subsp pneumoniae	Blood culture	V	Y
Klebsiella pneumoniae	Blood culture	V	
Klebsiella pneumoniae	Blood culture	V	N
Klebsiella pneumoniae	Blood culture	V	Y
Serratia marcescens	Blood culture	V	Y
Klebsiella pneumoniae subsp pneumoniae	Blood culture	V	Y
Klebsiella pneumoniae subsp pneumoniae	Blood culture	V	Y
Klebsiella pneumoniae subsp pneumoniae	Blood culture	V	Y
Klebsiella pneumoniae subsp pneumoniae	Blood culture	V	Y
Klebsiella pneumoniae subsp pneumoniae	Blood culture	V	Y
Serratia marcescens	Blood culture	V	Y
Klebsiella pneumoniae subsp pneumoniae	Blood culture	V	Y
Klebsiella pneumoniae subsp pneumoniae	Blood culture	V	Y
Klebsiella pneumoniae subsp pneumoniae	Blood culture	V	Y
Klebsiella pneumoniae subsp pneumoniae	Blood culture	V	Y
Escherichia coli	Blood culture	V	Y
Providencia stuartii	Blood culture	V	Y
Klebsiella pneumoniae subsp pneumoniae	Blood culture	V	
Escherichia coli	Blood culture	V	Y
Enterobacter cloacae	Blood culture	V	Y
Klebsiella pneumoniae	Blood culture	V	Y
Klebsiella pneumoniae subsp pneumoniae	Blood culture	V	Y
Klebsiella pneumoniae subsp pneumoniae	Blood culture	V	Y
Klebsiella pneumoniae subsp pneumoniae	Blood culture	V	Y
Klebsiella pneumoniae subsp pneumoniae	Blood culture	V	Y
Klebsiella pneumoniae subsp pneumoniae	Blood culture	V	Y
Klebsiella pneumoniae subsp pneumoniae	Blood culture	V	Y
Klebsiella pneumoniae subsp pneumoniae	Blood culture	V	Y

Serratia marcescens	Blood culture	V	Y
Klebsiella pneumoniae	Blood culture	V	Y
Enterobacter cloacae	Blood culture	V	N
Klebsiella pneumoniae	Blood culture	V	
Klebsiella pneumoniae subsp pneumoniae	Blood culture	V	Y
Escherichia coli	Blood culture	V	
Klebsiella pneumoniae subsp pneumoniae	Blood culture	V	Y
Klebsiella pneumoniae	Blood culture	V	Y
Klebsiella pneumoniae	Blood culture	V	
Klebsiella pneumoniae	Blood culture	V	Y
Klebsiella pneumoniae	Blood culture	V	
Klebsiella pneumoniae subsp pneumoniae	Blood culture	V	Y
Escherichia coli	Blood culture	V	
Klebsiella pneumoniae	Blood culture	V	Y
Klebsiella pneumoniae	Blood culture	V	N
Klebsiella pneumoniae subsp pneumoniae	Blood culture	V	Y
Klebsiella pneumoniae subsp pneumoniae	Blood culture	V	Y
Klebsiella pneumoniae	Blood culture	V	N
Klebsiella pneumoniae	Blood culture	V	Y
Klebsiella pneumoniae	Blood culture	V	U
Klebsiella pneumoniae	Blood culture	V	Y
Enterobacter cloacae	Blood culture	V	
Serratia marcescens	Blood culture	V	
Klebsiella pneumoniae	Blood culture	V	N
Enterobacter cloacae	Blood culture	V	U
Klebsiella oxytoca	Blood culture	V	U
Klebsiella pneumoniae	Blood culture	V	Y
Klebsiella pneumoniae	Blood culture	V	Y
Klebsiella pneumoniae	Blood culture	V	
Klebsiella pneumoniae	Blood culture	V	U
Klebsiella pneumoniae subsp pneumoniae	Blood culture	V	Y
Klebsiella pneumoniae	Blood culture	V	Y
Klebsiella pneumoniae	Blood culture	V	
Klebsiella pneumoniae	Blood culture	V	Y
Enterobacter cloacae	Blood culture	V	
Klebsiella pneumoniae subsp pneumoniae	Blood culture	V	
Klebsiella pneumoniae	Blood culture	V	Y
Klebsiella pneumoniae	Blood culture	V	U
Enterobacter cloacae	Blood culture	V	N
Klebsiella pneumoniae subsp pneumoniae	Blood culture	V	Y
Klebsiella pneumoniae subsp pneumoniae	Blood culture	V	Y
Klebsiella pneumoniae subsp pneumoniae	Blood culture	V	N
Klebsiella pneumoniae subsp pneumoniae	Blood culture	V	Y
Klebsiella pneumoniae subsp pneumoniae	Blood culture	V	N
Klebsiella pneumoniae	Blood culture	V	U
Klebsiella pneumoniae	Blood culture	V	Y
Citrobacter freundii	Blood culture	V	Y
Klebsiella pneumoniae subsp pneumoniae	Blood culture	V	N
Klebsiella pneumoniae subsp pneumoniae	Blood culture	V	Y
Klebsiella pneumoniae	Blood culture	N	U
Klebsiella oxytoca	Blood culture	V	
Klebsiella pneumoniae	Blood culture	V	
Klebsiella pneumoniae	Blood culture	V	
Klebsiella pneumoniae	Blood culture	V	
Klebsiella pneumoniae subsp pneumoniae	Blood culture	V	Y
Klebsiella pneumoniae	Blood culture	V	

Klebsiella pneumoniae subsp pneumoniae	Blood culture	V	Y
Klebsiella pneumoniae	Blood culture	V	
Klebsiella pneumoniae	Blood culture	V	
Klebsiella pneumoniae	Blood culture	V	
Klebsiella pneumoniae	Blood culture	V	Y
Enterobacter cloacae	Blood culture	V	
Klebsiella pneumoniae subsp pneumoniae	Blood culture	V	Y
Klebsiella pneumoniae subsp pneumoniae	Blood culture	V	Y
Klebsiella pneumoniae subsp pneumoniae	Blood culture	V	Y
Klebsiella oxytoca	Blood culture	V	Y
Klebsiella pneumoniae subsp pneumoniae	Blood culture	V	Y
Klebsiella pneumoniae subsp pneumoniae	Blood culture	V	Y
Klebsiella pneumoniae subsp pneumoniae	Blood culture	V	Y
Klebsiella pneumoniae subsp pneumoniae	Blood culture	V	N
Klebsiella pneumoniae	Blood culture	V	Y
Klebsiella pneumoniae subsp pneumoniae	Blood culture	V	
Klebsiella pneumoniae subsp pneumoniae	Blood culture	V	Y
Klebsiella pneumoniae	Blood culture	V	Y
Klebsiella pneumoniae subsp pneumoniae	Blood culture	V	Y
Klebsiella pneumoniae	Blood culture	V	Y
Serratia marcescens	Blood culture	V	Y
Providencia stuartii	Blood culture	V	Y
Escherichia coli	Blood culture	V	
Klebsiella pneumoniae	Blood culture	V	Y
Klebsiella pneumoniae subsp pneumoniae	Blood culture	V	
Klebsiella pneumoniae subsp pneumoniae	Blood culture	V	Y
Escherichia coli	Blood culture	V	
Klebsiella pneumoniae	Blood culture	V	Y
Klebsiella pneumoniae	Blood culture	V	N
Klebsiella pneumoniae	Blood culture	V	Y
Klebsiella pneumoniae	Blood culture	V	N
Klebsiella pneumoniae	Blood culture	V	Y
Klebsiella pneumoniae subsp pneumoniae	Blood culture	V	Y
Klebsiella pneumoniae subsp pneumoniae	Blood culture	V	Y
Klebsiella pneumoniae subsp pneumoniae	Blood culture	V	Y
Klebsiella pneumoniae	Blood culture	V	N
Klebsiella pneumoniae	Blood culture	V	U
Klebsiella pneumoniae subsp pneumoniae	Blood culture	V	Y
Klebsiella pneumoniae subsp pneumoniae	Blood culture	V	
Klebsiella pneumoniae	Blood culture	V	Y
Klebsiella pneumoniae	Blood culture	V	Y
Klebsiella pneumoniae	Blood culture	V	Y
Serratia marcescens	Blood culture	V	Y
Klebsiella pneumoniae subsp pneumoniae	Blood culture	V	
Klebsiella pneumoniae subsp pneumoniae	Blood culture	V	Y
Enterobacter aerogenes	Blood culture	V	Y
Klebsiella pneumoniae	Blood culture	V	Y
Klebsiella pneumoniae subsp pneumoniae	Blood culture	V	Y
Klebsiella pneumoniae subsp pneumoniae	Blood culture	V	Y
Klebsiella pneumoniae subsp pneumoniae	Blood culture	V	Y
Klebsiella pneumoniae	Blood culture	V	
Klebsiella oxytoca	Blood culture	V	Y
Klebsiella pneumoniae subsp pneumoniae	Blood culture	V	Y
Klebsiella pneumoniae subsp pneumoniae	Blood culture	V	Y
Klebsiella pneumoniae	Blood culture	V	Y
Klebsiella pneumoniae subsp pneumoniae	Blood culture	V	Y

Klebsiella pneumoniae	Blood culture	V	Y
Klebsiella pneumoniae	Blood culture	V	U
Serratia marcescens	Blood culture	V	N
Klebsiella pneumoniae subsp pneumoniae	Blood culture	V	Y
Klebsiella pneumoniae	Blood culture	V	Y
Klebsiella pneumoniae	Blood culture	V	Y
Klebsiella pneumoniae	Blood culture	V	Y
Klebsiella pneumoniae subsp pneumoniae	Blood culture	V	N
Providencia stuartii	Blood culture	V	Y
Klebsiella pneumoniae subsp pneumoniae	Blood culture	V	N
Klebsiella pneumoniae subsp pneumoniae	Blood culture	V	Y
Klebsiella pneumoniae	Blood culture	V	Y
Klebsiella pneumoniae subsp pneumoniae	Blood culture	V	Y
Klebsiella pneumoniae subsp pneumoniae	Blood culture	V	Y
Klebsiella pneumoniae subsp pneumoniae	Blood culture	V	
Klebsiella pneumoniae	Blood culture	V	Y
Enterobacter cloacae	Blood culture	V	Y
Klebsiella pneumoniae subsp pneumoniae	Blood culture	V	Y
Klebsiella pneumoniae subsp pneumoniae	Blood culture	V	Y
Klebsiella pneumoniae subsp pneumoniae	Blood culture	V	Y
Klebsiella pneumoniae subsp pneumoniae	Blood culture	V	Y
Enterobacter cloacae	Blood culture	V	Y
Enterobacter dissolvens	Blood culture	V	Y
Klebsiella pneumoniae	Blood culture	V	U
Klebsiella pneumoniae subsp pneumoniae	Blood culture	V	Y
Klebsiella pneumoniae subsp pneumoniae	Blood culture	V	
Klebsiella pneumoniae subsp pneumoniae	Blood culture	V	Y
Klebsiella pneumoniae	Blood culture	V	Y
Klebsiella pneumoniae	Blood culture	V	Y
Klebsiella pneumoniae subsp pneumoniae	Blood culture	V	Y
Klebsiella pneumoniae subsp pneumoniae	Blood culture	V	Y
Klebsiella pneumoniae subsp pneumoniae	Blood culture	V	Y
Klebsiella pneumoniae	Blood culture	V	Y
Klebsiella pneumoniae	Blood culture	V	Y
Enterobacter aerogenes	Blood culture	V	U
Klebsiella pneumoniae	Blood culture	V	Y
Klebsiella pneumoniae subsp pneumoniae	Blood culture	V	U
Klebsiella pneumoniae	Blood culture	V	Y
Klebsiella pneumoniae	Blood culture	V	
Klebsiella pneumoniae subsp pneumoniae	Blood culture	V	
Klebsiella pneumoniae subsp pneumoniae	Blood culture	V	Y
Klebsiella pneumoniae subsp pneumoniae	Blood culture	V	Y
Klebsiella pneumoniae subsp pneumoniae	Blood culture	V	Y
Enterobacter cloacae	Blood culture	V	Y
Klebsiella pneumoniae	Blood culture	V	Y
Klebsiella pneumoniae	Blood culture	V	N
Klebsiella pneumoniae	Blood culture	V	N
Klebsiella pneumoniae	Blood culture	V	
Klebsiella pneumoniae	Blood culture	V	Y
Klebsiella pneumoniae	Blood culture	V	
Klebsiella pneumoniae	Blood culture	V	N
Klebsiella pneumoniae	Blood culture	V	
Klebsiella pneumoniae	Blood culture	V	N
Klebsiella pneumoniae subsp pneumoniae	Blood culture	V	Y
Klebsiella pneumoniae subsp pneumoniae	Blood culture	V	Y
Klebsiella pneumoniae subsp pneumoniae	Blood culture	V	Y

Klebsiella pneumoniae	Blood culture	V	N
Klebsiella pneumoniae	Blood culture	V	Y
Klebsiella pneumoniae	Blood culture	V	
Klebsiella pneumoniae	Blood culture	V	Y
Citrobacter braakii	Blood culture	V	Y
Klebsiella pneumoniae subsp pneumoniae	Blood culture	V	Y
Enterobacter cloacae	Blood culture	V	N
Klebsiella pneumoniae subsp pneumoniae	Blood culture	V	Y
Klebsiella pneumoniae subsp pneumoniae	Blood culture	N	Y
Klebsiella pneumoniae subsp pneumoniae	Blood culture	V	Y
Klebsiella pneumoniae subsp pneumoniae	Blood culture	V	Y
Klebsiella pneumoniae subsp pneumoniae	Blood culture	V	Y
Klebsiella pneumoniae subsp pneumoniae	Blood culture	V	
Klebsiella pneumoniae	Blood culture	V	U
Klebsiella pneumoniae subsp pneumoniae	Blood culture	V	Y
Serratia marcescens	Blood culture	V	Y
Enterobacter cloacae	Blood culture	V	Y
Serratia marcescens	Blood culture	V	Y
Klebsiella pneumoniae subsp pneumoniae	Blood culture	V	
Escherichia coli	Blood culture	V	Y
Klebsiella pneumoniae subsp pneumoniae	Blood culture	V	
Klebsiella pneumoniae subsp pneumoniae	Blood culture	V	Y
Klebsiella pneumoniae	Blood culture	V	N
Klebsiella pneumoniae	Blood culture	V	N
Klebsiella pneumoniae	Blood culture	V	Y
Klebsiella pneumoniae subsp pneumoniae	Blood culture	V	U
Klebsiella pneumoniae subsp pneumoniae	Blood culture	V	Y
Klebsiella pneumoniae subsp pneumoniae	Blood culture	V	Y
Klebsiella pneumoniae subsp pneumoniae	Blood culture	V	Y
Enterobacter cloacae	Blood culture	V	Y
Klebsiella pneumoniae subsp pneumoniae	Blood culture	V	Y
Klebsiella pneumoniae subsp pneumoniae	Blood culture	V	Y
Klebsiella pneumoniae subsp pneumoniae	Blood culture	V	Y
Klebsiella pneumoniae subsp pneumoniae	Blood culture	V	Y
Klebsiella pneumoniae	Blood culture	V	Y
Klebsiella pneumoniae	Blood culture	V	Y
Klebsiella pneumoniae subsp pneumoniae	Blood culture	V	Y
Escherichia coli	Blood culture	V	
Klebsiella pneumoniae subsp pneumoniae	Blood culture	V	Y
Enterobacter cloacae	Blood culture	V	
Klebsiella pneumoniae subsp pneumoniae	Blood culture	V	Y
Klebsiella pneumoniae subsp pneumoniae	Blood culture	V	
Klebsiella pneumoniae subsp pneumoniae	Blood culture	V	Y
Klebsiella pneumoniae subsp pneumoniae	Blood culture	V	Y
Escherichia coli	Blood culture	V	N
Klebsiella pneumoniae	Blood culture	V	N
Klebsiella pneumoniae	Blood culture	V	N
Klebsiella pneumoniae	Blood culture	V	Y
Klebsiella pneumoniae subsp pneumoniae	Blood culture	V	Y
Klebsiella pneumoniae subsp pneumoniae	Blood culture	V	Y
Serratia marcescens	Blood culture	V	Y
Enterobacter cloacae	Blood culture	V	Y
Klebsiella pneumoniae subsp pneumoniae	Blood culture	V	Y
Serratia liquefaciens	Blood culture	V	Y
Escherichia coli	Blood culture	V	
Escherichia coli	Blood culture	V	

Escherichia coli	Blood culture	V	Y
Klebsiella pneumoniae subsp pneumoniae	Blood culture	V	Y
Klebsiella pneumoniae subsp pneumoniae	Blood culture	V	
Klebsiella pneumoniae subsp pneumoniae	Blood culture	V	Y
Klebsiella pneumoniae subsp pneumoniae	Blood culture	V	Y
Klebsiella pneumoniae	Blood culture	V	U
Klebsiella pneumoniae subsp pneumoniae	Blood culture	V	Y
Klebsiella pneumoniae subsp pneumoniae	Blood culture	V	Y
Klebsiella pneumoniae subsp pneumoniae	Blood culture	V	Y
Klebsiella pneumoniae subsp pneumoniae	Blood culture	V	Y
Klebsiella pneumoniae subsp ozaenae	Blood culture	V	N
Klebsiella pneumoniae subsp pneumoniae	Blood culture	V	Y
Klebsiella pneumoniae subsp pneumoniae	Blood culture	V	Y
Klebsiella pneumoniae subsp pneumoniae	Blood culture	V	Y
Klebsiella pneumoniae subsp pneumoniae	Blood culture	V	Y
Klebsiella pneumoniae subsp pneumoniae	Blood culture	V	Y
Serratia marcescens	Blood culture	V	
Klebsiella pneumoniae subsp pneumoniae	Blood culture	V	Y
Klebsiella pneumoniae subsp pneumoniae	Blood culture	V	
Enterobacter cloacae	Blood culture	V	
Enterobacter cloacae	Blood culture	V	Y
Klebsiella pneumoniae subsp pneumoniae	Blood culture	V	Y
Enterobacter cloacae	Blood culture	V	Y
Klebsiella pneumoniae	Blood culture	V	Y
Klebsiella pneumoniae subsp pneumoniae	Blood culture	V	Y
Klebsiella pneumoniae	Blood culture	V	Y
Klebsiella pneumoniae	Blood culture	V	N
Klebsiella oxytoca	Blood culture	V	Y
Klebsiella pneumoniae subsp pneumoniae	Blood culture	V	Y
Klebsiella pneumoniae subsp pneumoniae	Blood culture	V	
Klebsiella pneumoniae subsp pneumoniae	Blood culture	V	
Escherichia coli	Blood culture	V	
Enterobacter cloacae	Blood culture	V	N
Klebsiella pneumoniae	Blood culture	V	Y
Klebsiella pneumoniae subsp pneumoniae	Blood culture	V	Y
Klebsiella pneumoniae subsp pneumoniae	Blood culture	V	
Klebsiella pneumoniae subsp pneumoniae	Blood culture	V	
Klebsiella pneumoniae subsp pneumoniae	Blood culture	V	Y
Escherichia coli	Blood culture	V	
Escherichia coli	Blood culture	V	
Klebsiella pneumoniae subsp pneumoniae	Blood culture	V	Y
Klebsiella pneumoniae subsp pneumoniae	Blood culture	V	Y
Klebsiella pneumoniae subsp pneumoniae	Blood culture	V	Y
Klebsiella pneumoniae subsp pneumoniae	Blood culture	V	N
Enterobacter cloacae	Blood culture	V	Y
Escherichia coli	Blood culture	V	
Klebsiella pneumoniae subsp pneumoniae	Blood culture	V	
Escherichia coli	Blood culture	V	
Escherichia coli	Blood culture	V	
Klebsiella oxytoca	Blood culture	V	
Klebsiella pneumoniae	Blood culture	V	Y
Klebsiella pneumoniae	Blood culture	V	Y
Klebsiella pneumoniae subsp pneumoniae	Blood culture	V	Y
Klebsiella pneumoniae	Blood culture	V	U
Klebsiella oxytoca	Blood culture	V	
Klebsiella pneumoniae subsp pneumoniae	Blood culture	V	Y

Klebsiella pneumoniae	Blood culture	V	
Klebsiella pneumoniae	Blood culture	V	N
Klebsiella pneumoniae	Blood culture	V	
Klebsiella pneumoniae	Blood culture	V	N
Klebsiella pneumoniae subsp pneumoniae	Blood culture	V	Y
Enterobacter cloacae	Blood culture	V	
Klebsiella pneumoniae	Blood culture	V	
Klebsiella pneumoniae	Blood culture	V	Y
Klebsiella pneumoniae subsp pneumoniae	Blood culture	V	Y
Klebsiella pneumoniae subsp pneumoniae	Blood culture	V	Y
Klebsiella pneumoniae subsp pneumoniae	Blood culture	V	Y
Klebsiella pneumoniae subsp pneumoniae	Blood culture	V	Y
Klebsiella pneumoniae subsp pneumoniae	Blood culture	V	Y
Klebsiella pneumoniae subsp pneumoniae	Blood culture	V	Y
Klebsiella pneumoniae subsp pneumoniae	Blood culture	V	Y
Enterobacter cloacae	Blood culture	V	
Klebsiella pneumoniae	Blood culture	V	Y
Klebsiella pneumoniae	Blood culture	V	
Klebsiella pneumoniae	Blood culture	V	Y
Klebsiella pneumoniae subsp pneumoniae	Blood culture	V	N
Klebsiella pneumoniae subsp pneumoniae	Blood culture	V	Y
Klebsiella pneumoniae subsp pneumoniae	Blood culture	V	Y
Klebsiella pneumoniae subsp pneumoniae	Blood culture	V	Y
Klebsiella pneumoniae subsp pneumoniae	Blood culture	V	
Klebsiella pneumoniae	Blood culture	V	Y
Klebsiella pneumoniae	Blood culture	V	Y
Klebsiella pneumoniae	Blood culture	V	Y
Klebsiella pneumoniae	Blood culture	V	Y
Klebsiella pneumoniae	Blood culture	V	
Klebsiella pneumoniae	Blood culture	V	
Klebsiella pneumoniae	Blood culture	V	
Klebsiella pneumoniae	Blood culture	V	
Klebsiella pneumoniae	Blood culture	V	
Klebsiella pneumoniae subsp pneumoniae	Blood culture	V	Y
Klebsiella pneumoniae subsp pneumoniae	Blood culture	V	Y
Klebsiella pneumoniae subsp pneumoniae	Blood culture	V	
Klebsiella pneumoniae subsp pneumoniae	Blood culture	V	Y
Klebsiella pneumoniae subsp pneumoniae	Blood culture	V	Y
Klebsiella pneumoniae	Blood culture	V	
Klebsiella pneumoniae	Blood culture	V	Y
Klebsiella pneumoniae	Blood culture	V	Y
Klebsiella pneumoniae	Blood culture	V	Y
Klebsiella pneumoniae	Blood culture	V	Y
Enterobacter cloacae	Blood culture	V	Y
Klebsiella pneumoniae subsp pneumoniae	Blood culture	V	Y
Klebsiella pneumoniae	Blood culture	V	Y
Enterobacter cloacae	Blood culture	V	
Klebsiella pneumoniae	Blood culture	V	
Enterobacter cloacae	Blood culture	V	
Klebsiella pneumoniae	Blood culture	V	Y
Klebsiella pneumoniae	Blood culture	V	
Klebsiella pneumoniae	Blood culture	V	
Klebsiella pneumoniae	Blood culture	V	
Klebsiella pneumoniae subsp pneumoniae	Blood culture	V	Y
Klebsiella pneumoniae subsp pneumoniae	Blood culture	V	Y
Klebsiella pneumoniae	Blood culture	V	

Klebsiella pneumoniae subsp pneumoniae	Blood culture	V	N
Klebsiella pneumoniae subsp pneumoniae	Blood culture	V	Y
Klebsiella pneumoniae subsp pneumoniae	Blood culture	V	Y
Klebsiella pneumoniae	Blood culture	V	Y
Klebsiella pneumoniae	Blood culture	V	N
Klebsiella pneumoniae	Blood culture	V	
Klebsiella pneumoniae	Blood culture	V	Y
Enterobacter cloacae	Blood culture	V	Y
Klebsiella pneumoniae subsp pneumoniae	Blood culture	A	
Klebsiella pneumoniae	Blood culture	A	Y
Klebsiella pneumoniae subsp pneumoniae	Blood culture	V	Y
Klebsiella pneumoniae subsp pneumoniae	Blood culture	B/L	Y
Klebsiella pneumoniae	Blood culture	B/L	Y
Klebsiella pneumoniae subsp pneumoniae	Blood culture	V	Y
Klebsiella pneumoniae	Blood culture	A	U
Klebsiella pneumoniae	Blood culture	V	
Klebsiella pneumoniae subsp pneumoniae	Blood culture	V	Y
Enterobacter cloacae	Blood culture	V	
Klebsiella pneumoniae	Blood culture	V	Y
Klebsiella pneumoniae	Blood culture	V	
Klebsiella pneumoniae	Blood culture	V	Y
Klebsiella pneumoniae	Blood culture	V	Y
Klebsiella pneumoniae subsp pneumoniae	Blood culture	V	Y
Enterobacter cloacae	Blood culture	V	U
Klebsiella pneumoniae	Blood culture	V	
Klebsiella pneumoniae subsp pneumoniae	Blood culture	V	
Klebsiella pneumoniae subsp pneumoniae	Blood culture	V	Y
Klebsiella pneumoniae subsp pneumoniae	Blood culture	V	Y
Klebsiella pneumoniae	Blood culture	A	
Klebsiella pneumoniae subsp pneumoniae	Blood culture	A	Y
Enterobacter aerogenes	Blood culture	A	
Klebsiella oxytoca	Blood culture	A	
Klebsiella pneumoniae	Blood culture	A	Y
Klebsiella pneumoniae	Blood culture	A	N
Klebsiella pneumoniae	Blood culture	A	
Enterobacter cloacae subsp cloacae	Blood culture	A	N
Klebsiella pneumoniae	Blood culture	A	Y
Klebsiella pneumoniae	Blood culture	A	Y
Klebsiella pneumoniae	Blood culture	A	
Klebsiella pneumoniae subsp pneumoniae	Blood culture	A	Y
Klebsiella pneumoniae	Blood culture	A	
Klebsiella pneumoniae	Blood culture	A	
Enterobacter cloacae	Blood culture	A	N
Klebsiella pneumoniae	Blood culture	A	Y
Klebsiella pneumoniae subsp pneumoniae	Blood culture	A	
Escherichia coli	Blood culture	A	Y
Klebsiella pneumoniae	Blood culture	A	
Klebsiella pneumoniae subsp pneumoniae	Blood culture	A	Y
Klebsiella pneumoniae subsp pneumoniae	Blood culture	A	Y
Klebsiella pneumoniae	Blood culture	A	Y
Klebsiella pneumoniae subsp pneumoniae	Blood culture	A	Y
Klebsiella pneumoniae subsp pneumoniae	Blood culture	A	Y
Enterobacter cloacae complex	Blood culture	A	U
Escherichia coli	Blood culture	A	N
Klebsiella pneumoniae	Blood culture	A	
Klebsiella pneumoniae subsp pneumoniae	Blood culture	A	Y

Enterobacter cloacae	Blood culture	A	Y
Klebsiella pneumoniae	Blood culture	A	N
Klebsiella pneumoniae subsp pneumoniae	Blood culture	A	Y
Klebsiella pneumoniae	Blood culture	A	Y
Citrobacter koseri	Blood culture	A	
Klebsiella pneumoniae subsp ozaenae	Blood culture	A	N
Klebsiella pneumoniae subsp pneumoniae	Blood culture	A	N
Enterobacter cloacae	Blood culture	A	Y
Klebsiella pneumoniae	Blood culture	A	Y
Klebsiella pneumoniae	Blood culture	A	
Enterobacter cloacae complex	Blood culture	A	Y
Klebsiella pneumoniae	Blood culture	A	
Klebsiella pneumoniae	Blood culture	A	Y
Klebsiella pneumoniae subsp pneumoniae	Blood culture	A	
Klebsiella pneumoniae	Blood culture	A	Y
Klebsiella pneumoniae subsp pneumoniae	Blood culture	A	Y
Enterobacter cloacae	Blood culture	A	
Klebsiella pneumoniae	Blood culture	A	
Klebsiella pneumoniae	Blood culture	A	Y
Klebsiella pneumoniae subsp pneumoniae	Blood culture	A	Y
Klebsiella pneumoniae subsp pneumoniae	Blood culture	A	N
Klebsiella pneumoniae subsp pneumoniae	Blood culture	A	Y
Klebsiella pneumoniae	Blood culture	A	Y
Klebsiella pneumoniae	Blood culture	A	Y
Klebsiella pneumoniae	Blood culture	A	N
Enterobacter cloacae complex	Blood culture	A	Y
Serratia marcescens	Blood culture	A	Y
Klebsiella pneumoniae	Blood culture	A	
Serratia marcescens	Blood culture	A	N
Klebsiella pneumoniae subsp pneumoniae	Blood culture	A	
Klebsiella pneumoniae subsp pneumoniae	Blood culture	A	Y
Klebsiella pneumoniae	Blood culture	A	
Serratia marcescens	Blood culture	A	Y
Klebsiella pneumoniae	Blood culture	A	Y
Enterobacter cloacae complex	Blood culture	A	
Enterobacter cloacae complex	Blood culture	A	Y
Klebsiella pneumoniae	Blood culture	A	Y
Klebsiella pneumoniae	Blood culture	A	N
Klebsiella pneumoniae	Blood culture	A	Y
Klebsiella pneumoniae	Blood culture	A	
Klebsiella pneumoniae subsp pneumoniae	Blood culture	A	
Klebsiella pneumoniae subsp pneumoniae	Blood culture	A	U
Klebsiella pneumoniae	Blood culture	A	
Klebsiella pneumoniae	Blood culture	A	Y
Klebsiella pneumoniae subsp pneumoniae	Blood culture	A	Y
Enterobacter aerogenes	Blood culture	A	
Klebsiella pneumoniae subsp pneumoniae	Blood culture	A	Y
Klebsiella pneumoniae subsp pneumoniae	Blood culture	A	Y
Klebsiella pneumoniae subsp pneumoniae	Blood culture	A	Y
Klebsiella pneumoniae subsp pneumoniae	Blood culture	A	Y
Klebsiella pneumoniae subsp pneumoniae	Blood culture	A	N
Enterobacter cloacae	Blood culture	A	Y
Klebsiella pneumoniae	Blood culture	A	Y
Klebsiella pneumoniae	Blood culture	A	
Klebsiella pneumoniae	Blood culture	A	U
Klebsiella pneumoniae subsp pneumoniae	Blood culture	A	Y

Klebsiella pneumoniae	Blood culture	A	N
Enterobacter cloacae	Blood culture	V	Y
Klebsiella pneumoniae	Blood culture	A	Y
Klebsiella pneumoniae	Blood culture	A	Y
Klebsiella pneumoniae subsp pneumoniae	Blood culture	A	
Klebsiella pneumoniae	Blood culture	A	Y
Klebsiella pneumoniae	Blood culture	A	Y
Serratia marcescens	Blood culture	A	Y
Enterobacter aerogenes	Blood culture	A	
Klebsiella pneumoniae subsp pneumoniae	Blood culture	A	
Klebsiella pneumoniae subsp pneumoniae	Blood culture	A	Y
Klebsiella pneumoniae subsp pneumoniae	Blood culture	A	Y
Klebsiella pneumoniae subsp pneumoniae	Blood culture	A	N
Klebsiella pneumoniae subsp pneumoniae	Blood culture	A	Y
Providencia stuartii	Blood culture	A	Y
Enterobacter cloacae	Blood culture	A	
Klebsiella pneumoniae	Blood culture	A	
Klebsiella pneumoniae subsp pneumoniae	Blood culture	A	Y
Klebsiella pneumoniae subsp pneumoniae	Blood culture	A	Y
Klebsiella pneumoniae	Blood culture	A	Y
Klebsiella pneumoniae	Blood culture	A	Y
Klebsiella pneumoniae	Blood culture	A	U
Klebsiella pneumoniae	Blood culture	A	Y
Klebsiella pneumoniae subsp pneumoniae	Blood culture	A	N
Serratia marcescens	Blood culture	A	N
Klebsiella pneumoniae subsp pneumoniae	Blood culture	A	Y
Klebsiella pneumoniae subsp pneumoniae	Blood culture	A	Y
Serratia marcescens	Blood culture	A	Y
Klebsiella pneumoniae subsp pneumoniae	Blood culture	A	Y
Klebsiella pneumoniae subsp pneumoniae	Blood culture	A	Y
Klebsiella pneumoniae subsp pneumoniae	Blood culture	A	Y
Klebsiella pneumoniae	Blood culture	A	
Serratia marcescens	Blood culture	A	
Enterobacter cloacae complex	Blood culture	A	Y
Klebsiella pneumoniae	Blood culture	A	Y
Klebsiella pneumoniae subsp pneumoniae	Blood culture	A	Y
Serratia marcescens	Blood culture	A	Y
Klebsiella pneumoniae subsp pneumoniae	Blood culture	A	
Klebsiella pneumoniae subsp pneumoniae	Blood culture	A	N
Citrobacter koseri	Blood culture	A	
Klebsiella pneumoniae subsp pneumoniae	Blood culture	A	
Enterobacter cloacae	Blood culture	A	Y
Klebsiella pneumoniae	Blood culture	A	
Klebsiella pneumoniae subsp pneumoniae	Blood culture	A	N
Klebsiella pneumoniae	Blood culture	A	N
Klebsiella pneumoniae subsp ozaenae	Blood culture	A	Y
Klebsiella pneumoniae	Blood culture	A	U
Klebsiella pneumoniae subsp pneumoniae	Blood culture	A	Y
Klebsiella pneumoniae subsp pneumoniae	Blood culture	A	Y
Klebsiella pneumoniae subsp pneumoniae	Blood culture	A	
Klebsiella pneumoniae subsp pneumoniae	Blood culture	V	Y
Klebsiella pneumoniae subsp pneumoniae	Blood culture	A	
Enterobacter cloacae	Blood culture	A	
Klebsiella pneumoniae subsp pneumoniae	Blood culture	A	Y
Klebsiella pneumoniae subsp pneumoniae	Blood culture	A	Y
Escherichia coli	Blood culture	A	

Klebsiella pneumoniae subsp pneumoniae	Blood culture	A	Y
Enterobacter cloacae	Blood culture	A	
Klebsiella pneumoniae	Blood culture	A	
Serratia marcescens	Blood culture	A	Y
Klebsiella pneumoniae	Blood culture	A	Y
Escherichia coli	Blood culture	A	
Enterobacter cloacae	Blood culture	A	
Klebsiella pneumoniae subsp pneumoniae	Blood culture	A	Y
Klebsiella pneumoniae subsp pneumoniae	Blood culture	A	
Klebsiella pneumoniae	Blood culture	A	Y
Klebsiella pneumoniae subsp pneumoniae	Blood culture	A	Y
Klebsiella pneumoniae subsp pneumoniae	Blood culture	A	
Enterobacter cloacae subsp dissolvens	Blood culture	A	
Enterobacter cloacae subsp dissolvens	Blood culture	A	
Klebsiella pneumoniae subsp pneumoniae	Blood culture	A	Y
Klebsiella pneumoniae	Blood culture	A	Y
Klebsiella pneumoniae subsp pneumoniae	Blood culture	A	Y
Klebsiella pneumoniae	Blood culture	A	Y
Klebsiella pneumoniae subsp pneumoniae	Blood culture	A	
Klebsiella pneumoniae subsp pneumoniae	Blood culture	A	Y
Serratia marcescens	Blood culture	A	
Klebsiella pneumoniae subsp pneumoniae	Blood culture	A	Y
Klebsiella pneumoniae	Blood culture	A	N
Klebsiella pneumoniae	Blood culture	A	
Klebsiella pneumoniae subsp pneumoniae	Blood culture	A	
Proteus mirabilis	Blood culture	A	
Klebsiella pneumoniae subsp pneumoniae	Blood culture	A	
Klebsiella pneumoniae subsp pneumoniae	Blood culture	A	
Klebsiella pneumoniae	Blood culture	A	
Klebsiella pneumoniae subsp pneumoniae	Blood culture	A	N
Klebsiella pneumoniae	Blood culture	A	Y
Klebsiella pneumoniae	Blood culture	A	U
Enterobacter aerogenes	Blood culture	A	Y
Klebsiella pneumoniae	Blood culture	A	
Klebsiella pneumoniae subsp pneumoniae	Blood culture	A	Y
Citrobacter freundii	Blood culture	A	Y
Klebsiella pneumoniae	Blood culture	A	U
Enterobacter cloacae subsp dissolvens	Blood culture	A	Y
Klebsiella pneumoniae	Blood culture	A	Y
Enterobacter cloacae	Blood culture	A	
Escherichia coli	Blood culture	A	Y
Klebsiella pneumoniae subsp pneumoniae	Blood culture	A	
Klebsiella pneumoniae subsp pneumoniae	Blood culture	A	
Klebsiella pneumoniae subsp pneumoniae	Blood culture	A	Y
Klebsiella pneumoniae	Blood culture	A	Y
Klebsiella pneumoniae subsp pneumoniae	Blood culture	A	N
Enterobacter aerogenes	Blood culture	A	
Klebsiella pneumoniae	Blood culture	A	N
Klebsiella pneumoniae	Blood culture	A	
Klebsiella pneumoniae	Blood culture	A	
Klebsiella pneumoniae subsp pneumoniae	Blood culture	A	U
Serratia species	Blood culture	A	
Enterobacter cloacae	Blood culture	V	Y
Klebsiella pneumoniae	Blood culture	V	Y
Klebsiella pneumoniae subsp pneumoniae	Blood culture	V	Y
Klebsiella pneumoniae subsp pneumoniae	Blood culture	V	Y

Klebsiella pneumoniae subsp ozaenae	Blood culture	V	
Klebsiella pneumoniae subsp pneumoniae	Blood culture	V	Y
Klebsiella pneumoniae subsp pneumoniae	Blood culture	V	Y
Klebsiella pneumoniae subsp pneumoniae	Blood culture	V	Y
Klebsiella pneumoniae subsp pneumoniae	Blood culture	V	
Klebsiella pneumoniae	Blood culture	V	
Escherichia coli	Blood culture	V	
Klebsiella pneumoniae subsp pneumoniae	Blood culture	V	
Klebsiella pneumoniae subsp pneumoniae	Blood culture	V	Y
Klebsiella pneumoniae subsp pneumoniae	Blood culture	V	Y
Klebsiella pneumoniae subsp pneumoniae	Blood culture	V	Y
Klebsiella pneumoniae	Blood culture	V	Y
Klebsiella pneumoniae	Blood culture	V	Y
Klebsiella pneumoniae subsp pneumoniae	Blood culture	V	Y
Klebsiella pneumoniae	Blood culture	V	Y
Klebsiella pneumoniae subsp pneumoniae	Blood culture	V	Y
Klebsiella pneumoniae	Blood culture	V	Y
Klebsiella pneumoniae	Blood culture	V	
Klebsiella pneumoniae subsp pneumoniae	Blood culture	A	Y
Enterobacter aerogenes	Blood culture	A	N
Klebsiella pneumoniae subsp pneumoniae	Blood culture	A	Y
Enterobacter aerogenes	Blood culture	A	Y
Klebsiella pneumoniae subsp pneumoniae	Blood culture	A	Y
Klebsiella pneumoniae	Blood culture	A	Y
Klebsiella pneumoniae	Blood culture	A	Y
Klebsiella pneumoniae	Blood culture	A	N
Klebsiella pneumoniae	Blood culture	A	U
Klebsiella pneumoniae	Blood culture	A	Y
Klebsiella pneumoniae	Blood culture	A	Y
Escherichia coli	Blood culture	A	Y
Klebsiella pneumoniae subsp pneumoniae	Blood culture	A	Y
Klebsiella pneumoniae	Blood culture	V	Y
Klebsiella pneumoniae subsp pneumoniae	Blood culture	A	Y
Klebsiella pneumoniae subsp pneumoniae	Blood culture	A	Y
Klebsiella oxytoca	Blood culture	A	U
Klebsiella pneumoniae subsp pneumoniae	Blood culture	V	
Escherichia coli	Blood culture	V	
Klebsiella pneumoniae	Blood culture	V	Y
Klebsiella pneumoniae subsp pneumoniae	Blood culture	V	
Enterobacter cloacae	Blood culture	V	Y
Klebsiella pneumoniae	Blood culture	V	Y
Klebsiella pneumoniae	Blood culture	V	Y
Klebsiella pneumoniae	Blood culture	V	Y
Klebsiella pneumoniae subsp pneumoniae	Blood culture	V	Y
Klebsiella pneumoniae subsp pneumoniae	Blood culture	V	Y
Klebsiella pneumoniae subsp pneumoniae	Blood culture	V	Y
Serratia marcescens	Blood culture	V	Y
Klebsiella pneumoniae	Blood culture	V	Y
Klebsiella pneumoniae	Blood culture	V	Y
Klebsiella oxytoca	Blood culture	V	
Klebsiella pneumoniae	Blood culture	V	Y
Klebsiella pneumoniae	Blood culture	V	
Klebsiella pneumoniae subsp pneumoniae	Blood culture	V	N
Escherichia coli	Blood culture	V	
Escherichia coli	Blood culture	V	
Klebsiella pneumoniae	Blood culture	V	

Klebsiella pneumoniae subsp pneumoniae	Blood culture	V	Y
Klebsiella pneumoniae subsp pneumoniae	Blood culture	V	
Klebsiella pneumoniae subsp pneumoniae	Blood culture	V	Y
Klebsiella pneumoniae subsp pneumoniae	Blood culture	V	Y
Klebsiella pneumoniae subsp pneumoniae	Blood culture	V	Y
Klebsiella pneumoniae subsp pneumoniae	Blood culture	V	Y
Klebsiella pneumoniae subsp pneumoniae	Blood culture	V	Y
Klebsiella pneumoniae	Blood culture	V	Y
Klebsiella pneumoniae subsp pneumoniae	Blood culture	V	Y
Klebsiella pneumoniae	Blood culture	V	
Escherichia coli	Blood culture	V	
Escherichia coli	Blood culture	V	
Enterobacter cloacae	Blood culture	V	
Serratia marcescens	Blood culture	V	
Klebsiella pneumoniae	Blood culture	V	Y
Klebsiella pneumoniae subsp pneumoniae	Blood culture	V	
Klebsiella pneumoniae	Blood culture	V	
Enterobacter cloacae	Blood culture	V	
Klebsiella pneumoniae subsp ozaenae	Blood culture	V	
Klebsiella pneumoniae subsp pneumoniae	Blood culture	V	
Klebsiella pneumoniae subsp pneumoniae	Blood culture	V	Y
Klebsiella pneumoniae subsp pneumoniae	Blood culture	V	Y
Escherichia coli	Blood culture	V	Y
Klebsiella pneumoniae subsp pneumoniae	Blood culture	V	Y
Klebsiella pneumoniae subsp pneumoniae	Blood culture	V	
Klebsiella pneumoniae subsp pneumoniae	Blood culture	V	Y
Klebsiella pneumoniae subsp pneumoniae	Blood culture	V	Y
Enterobacter cloacae	Blood culture	V	Y
Klebsiella pneumoniae subsp pneumoniae	Blood culture	V	
Klebsiella pneumoniae subsp pneumoniae	Blood culture	V	Y
Klebsiella pneumoniae subsp pneumoniae	Blood culture	V	Y
Klebsiella pneumoniae subsp pneumoniae	Blood culture	V	Y
Klebsiella pneumoniae subsp pneumoniae	Blood culture	V	Y
Klebsiella pneumoniae subsp pneumoniae	Blood culture	V	Y
Klebsiella oxytoca	Blood culture	V	Y
Escherichia coli	Blood culture	V	Y
Klebsiella pneumoniae subsp pneumoniae	Blood culture	V	
Klebsiella pneumoniae subsp pneumoniae	Blood culture	V	Y
Serratia marcescens	Blood culture	V	Y
Serratia marcescens	Blood culture	V	Y
Klebsiella pneumoniae subsp pneumoniae	Blood culture	V	Y
Klebsiella pneumoniae subsp pneumoniae	Blood culture	V	Y
Escherichia coli	Blood culture	V	Y
Escherichia coli	Blood culture	V	Y
Klebsiella pneumoniae subsp pneumoniae	Blood culture	V	Y
Klebsiella pneumoniae subsp pneumoniae	Blood culture	V	
Klebsiella pneumoniae	Blood culture	V	
Klebsiella pneumoniae	Blood culture	V	Y
Klebsiella pneumoniae	Blood culture	V	Y
Klebsiella pneumoniae subsp pneumoniae	Blood culture	V	Y
Klebsiella pneumoniae	Blood culture	V	Y
Enterobacter cloacae	Blood culture	V	
Klebsiella pneumoniae subsp pneumoniae	Blood culture	V	N
Klebsiella pneumoniae	Blood culture	V	U
Klebsiella pneumoniae	Blood culture	V	
Escherichia coli	Blood culture	V	Y

Klebsiella pneumoniae subsp pneumoniae	Blood culture	V	Y
Klebsiella pneumoniae subsp pneumoniae	Blood culture	V	Y
Klebsiella pneumoniae subsp pneumoniae	Blood culture	V	
Escherichia coli	Blood culture	V	Y
Klebsiella pneumoniae subsp pneumoniae	Blood culture	V	
Klebsiella pneumoniae subsp pneumoniae	Blood culture	V	Y
Klebsiella pneumoniae subsp pneumoniae	Blood culture	V	U
Providencia stuartii	Blood culture	V	Y
Enterobacter cloacae	Blood culture	V	
Klebsiella pneumoniae	Blood culture	V	Y
Klebsiella pneumoniae	Blood culture	V	N
Klebsiella pneumoniae subsp pneumoniae	Blood culture	V	Y
Klebsiella pneumoniae	Blood culture	V	
Klebsiella aerogenes	Blood culture	V	
Klebsiella pneumoniae subsp pneumoniae	Blood culture	V	Y
Klebsiella pneumoniae subsp pneumoniae	Blood culture	V	Y
Klebsiella pneumoniae subsp pneumoniae	Blood culture	V	Y
Klebsiella pneumoniae subsp pneumoniae	Blood culture	V	Y
Klebsiella pneumoniae	Blood culture	V	Y
Klebsiella pneumoniae	Blood culture	V	
Enterobacter cloacae	Blood culture	V	Y
Klebsiella pneumoniae	Blood culture	V	Y
Klebsiella pneumoniae subsp pneumoniae	Blood culture	V	Y
Klebsiella pneumoniae subsp pneumoniae	Blood culture	V	Y
Klebsiella pneumoniae subsp pneumoniae	Blood culture	V	Y
Klebsiella pneumoniae subsp pneumoniae	Blood culture	V	
Klebsiella pneumoniae	Blood culture	V	Y
Klebsiella pneumoniae subsp pneumoniae	Blood culture	V	Y
Klebsiella pneumoniae	Blood culture	V	Y
Klebsiella pneumoniae	Blood culture	V	Y
Klebsiella pneumoniae	Blood culture	V	
Klebsiella pneumoniae subsp pneumoniae	Blood culture	V	Y
Klebsiella pneumoniae subsp pneumoniae	Blood culture	V	Y
Klebsiella pneumoniae subsp pneumoniae	Blood culture	V	Y
Serratia marcescens	Blood culture	V	
Klebsiella pneumoniae subsp pneumoniae	Blood culture	V	Y
Klebsiella pneumoniae	Blood culture	V	
Klebsiella pneumoniae subsp pneumoniae	Blood culture	V	Y
Klebsiella pneumoniae subsp pneumoniae	Blood culture	V	Y
Klebsiella pneumoniae subsp pneumoniae	Blood culture	V	
Klebsiella pneumoniae	Blood culture	V	
Klebsiella pneumoniae	Blood culture	V	
Klebsiella oxytoca	Blood culture	V	
Serratia marcescens	Blood culture	V	Y
Klebsiella pneumoniae subsp pneumoniae	Blood culture	V	N
Klebsiella pneumoniae subsp pneumoniae	Blood culture	V	
Klebsiella pneumoniae subsp pneumoniae	Blood culture	V	Y
Serratia marcescens	Blood culture	V	Y
Enterobacter cloacae	Blood culture	V	Y
Serratia marcescens	Blood culture	V	
Klebsiella pneumoniae subsp pneumoniae	Blood culture	V	Y
Klebsiella pneumoniae subsp pneumoniae	Blood culture	V	N
Enterobacter cloacae	Blood culture	V	
Klebsiella pneumoniae	Blood culture	V	Y
Klebsiella pneumoniae subsp pneumoniae	Blood culture	N	
Klebsiella pneumoniae subsp pneumoniae	Blood culture	V	N

Klebsiella pneumoniae	Blood culture	V	
Klebsiella pneumoniae subsp pneumoniae	Blood culture	V	
Klebsiella pneumoniae subsp pneumoniae	Blood culture	V	Y
Klebsiella pneumoniae	Blood culture	V	
Klebsiella pneumoniae subsp ozaenae	Blood culture	V	
Klebsiella pneumoniae subsp pneumoniae	Blood culture	V	Y
Klebsiella pneumoniae	Blood culture	V	Y
Serratia marcescens	Blood culture	V	
Klebsiella pneumoniae	Blood culture	V	Y
Klebsiella pneumoniae subsp pneumoniae	Blood culture	V	Y
Serratia marcescens	Blood culture	V	
Klebsiella pneumoniae subsp pneumoniae	Blood culture	V	Y
Klebsiella pneumoniae subsp pneumoniae	Blood culture	V	Y
Providencia stuartii	Blood culture	V	Y
Klebsiella pneumoniae subsp pneumoniae	Blood culture	V	Y
Enterobacter cloacae	Blood culture	V	N
Klebsiella pneumoniae subsp pneumoniae	Blood culture	V	Y
Klebsiella pneumoniae subsp pneumoniae	Blood culture	V	Y
Serratia marcescens	Blood culture	V	
Klebsiella pneumoniae subsp pneumoniae	Blood culture	V	Y
Klebsiella pneumoniae subsp pneumoniae	Blood culture	V	
Serratia marcescens	Blood culture	V	N
Klebsiella pneumoniae subsp pneumoniae	Blood culture	V	
Klebsiella pneumoniae	Blood culture	V	
Klebsiella pneumoniae subsp pneumoniae	Blood culture	V	Y
Klebsiella pneumoniae subsp pneumoniae	Blood culture	V	Y
Klebsiella pneumoniae subsp pneumoniae	Blood culture	V	
Klebsiella pneumoniae	Blood culture	V	
Klebsiella pneumoniae	Blood culture	V	
Klebsiella pneumoniae	Blood culture	V	Y
Klebsiella pneumoniae	Blood culture	V	
Klebsiella pneumoniae	Blood culture	V	Y
Klebsiella pneumoniae	Blood culture	V	
Klebsiella oxytoca	Blood culture	V	
Klebsiella pneumoniae	Blood culture	V	
Klebsiella pneumoniae	Blood culture	V	Y
Klebsiella pneumoniae	Blood culture	V	
Klebsiella pneumoniae	Blood culture	V	
Klebsiella pneumoniae subsp pneumoniae	Blood culture	V	U
Klebsiella pneumoniae subsp pneumoniae	Blood culture	V	Y
Klebsiella pneumoniae subsp pneumoniae	Blood culture	V	Y
Klebsiella pneumoniae subsp pneumoniae	Blood culture	V	Y
Klebsiella pneumoniae subsp pneumoniae	Blood culture	V	Y
Enterobacter cloacae	Blood culture	V	Y
Klebsiella pneumoniae subsp pneumoniae	Blood culture	V	Y
Klebsiella pneumoniae subsp pneumoniae	Blood culture	V	Y
Enterobacter cloacae	Blood culture	V	Y
Klebsiella pneumoniae	Blood culture	V	Y
Klebsiella oxytoca	Blood culture	V	Y
Klebsiella pneumoniae	Blood culture	V	Y
Klebsiella pneumoniae	Blood culture	V	
Klebsiella pneumoniae subsp pneumoniae	Blood culture	V	Y
Klebsiella pneumoniae	Blood culture	V	Y
Klebsiella pneumoniae subsp pneumoniae	Blood culture	V	
Klebsiella pneumoniae	Blood culture	V	
Enterobacter cloacae	Blood culture	V	

Klebsiella pneumoniae	Blood culture	V	Y
Serratia marcescens	Blood culture	V	Y
Klebsiella pneumoniae subsp pneumoniae	Blood culture	V	
Klebsiella pneumoniae subsp pneumoniae	Blood culture	V	Y
Klebsiella pneumoniae	Blood culture	A	
Klebsiella pneumoniae	Blood culture	A	
Klebsiella pneumoniae subsp pneumoniae	Blood culture	A	N
Enterobacter aerogenes	Blood culture	A	
Klebsiella pneumoniae subsp pneumoniae	Blood culture	A	Y
Enterobacter cloacae complex	Blood culture	A	
Providencia rettgeri	Blood culture	A	Y
Escherichia coli	Blood culture	A	
Serratia marcescens	Blood culture	A	
Escherichia coli	Blood culture	A	
Klebsiella pneumoniae subsp pneumoniae	Blood culture	A	
Klebsiella pneumoniae subsp pneumoniae	Blood culture	A	Y
Klebsiella oxytoca	Blood culture	A	Y
Klebsiella pneumoniae subsp pneumoniae	Blood culture	A	Y
Klebsiella pneumoniae subsp pneumoniae	Blood culture	A	
Klebsiella pneumoniae subsp pneumoniae	Blood culture	A	
Klebsiella pneumoniae subsp pneumoniae	Blood culture	A	Y
Klebsiella pneumoniae subsp pneumoniae	Blood culture	A	
Klebsiella pneumoniae subsp pneumoniae	Blood culture	A	
Klebsiella pneumoniae subsp pneumoniae	Blood culture	A	Y
Klebsiella pneumoniae	Blood culture	A	N
Klebsiella pneumoniae subsp pneumoniae	Blood culture	A	Y
Klebsiella pneumoniae subsp pneumoniae	Blood culture	A	Y
Klebsiella pneumoniae subsp pneumoniae	Blood culture	A	U
Klebsiella pneumoniae	Blood culture	A	
Klebsiella pneumoniae subsp pneumoniae	Blood culture	A	Y
Serratia marcescens	Blood culture	A	U
Serratia marcescens	Blood culture	A	
Klebsiella pneumoniae subsp pneumoniae	Blood culture	A	N
Klebsiella pneumoniae subsp pneumoniae	Blood culture	A	Y
Klebsiella pneumoniae	Blood culture	A	
Klebsiella pneumoniae subsp pneumoniae	Blood culture	A	Y
Klebsiella pneumoniae subsp pneumoniae	Blood culture	A	Y
Klebsiella pneumoniae subsp pneumoniae	Blood culture	A	Y
Klebsiella pneumoniae subsp pneumoniae	Blood culture	A	N
Citrobacter freundii	Blood culture	A	
Klebsiella pneumoniae subsp pneumoniae	Blood culture	A	Y
Klebsiella pneumoniae subsp pneumoniae	Blood culture	A	N
Klebsiella pneumoniae	Blood culture	A	
Providencia stuartii	Blood culture	A	
Klebsiella pneumoniae subsp pneumoniae	Blood culture	A	
Klebsiella pneumoniae subsp pneumoniae	Blood culture	A	Y
Klebsiella pneumoniae	Blood culture	A	Y
Klebsiella pneumoniae subsp pneumoniae	Blood culture	A	Y
Enterobacter cloacae	Blood culture	A	U
Citrobacter koseri	Blood culture	A	
Serratia marcescens	Blood culture	A	
Klebsiella pneumoniae subsp pneumoniae	Blood culture	A	Y
Enterobacter aerogenes	Blood culture	A	N
Klebsiella pneumoniae	Blood culture	A	Y
Klebsiella pneumoniae	Blood culture	A	
Klebsiella pneumoniae	Blood culture	A	Y

Klebsiella pneumoniae	Blood culture	A	
Proteus mirabilis	Blood culture	A	Y
Klebsiella pneumoniae	Blood culture	A	Y
Klebsiella pneumoniae	Blood culture	A	N
Klebsiella aerogenes	Blood culture	A	
Klebsiella pneumoniae	Blood culture	A	Y
Klebsiella pneumoniae	Blood culture	A	N
Klebsiella pneumoniae	Blood culture	A	Y
Klebsiella pneumoniae subsp pneumoniae	Blood culture	A	Y
Klebsiella pneumoniae	Blood culture	A	
Klebsiella pneumoniae subsp pneumoniae	Blood culture	A	
Klebsiella pneumoniae subsp pneumoniae	Blood culture	A	Y
Klebsiella pneumoniae	Blood culture	A	
Enterobacter aerogenes	Blood culture	A	U
Serratia plymuthica	Blood culture	A	
Serratia fonticola	Blood culture	A	
Klebsiella pneumoniae	Blood culture	A	Y
Klebsiella pneumoniae	Blood culture	A	
Klebsiella pneumoniae subsp pneumoniae	Blood culture	A	
Klebsiella pneumoniae subsp pneumoniae	Blood culture	A	Y
Klebsiella pneumoniae	Blood culture	A	Y
Klebsiella pneumoniae subsp pneumoniae	Blood culture	A	
Enterobacter cloacae	Blood culture	A	
Serratia marcescens	Blood culture	A	Y
Escherichia coli	Blood culture	A	
Klebsiella pneumoniae subsp pneumoniae	Blood culture	A	U
Klebsiella pneumoniae	Blood culture	A	Y
Klebsiella pneumoniae subsp pneumoniae	Blood culture	A	Y
Serratia marcescens	Blood culture	A	
Enterobacter aerogenes	Blood culture	A	
Enterobacter cloacae complex	Blood culture	A	N
Klebsiella pneumoniae	Blood culture	A	
Klebsiella pneumoniae	Blood culture	A	
Klebsiella pneumoniae subsp pneumoniae	Blood culture	A	
Klebsiella pneumoniae	Blood culture	A	
Klebsiella pneumoniae subsp pneumoniae	Blood culture	A	
Klebsiella pneumoniae subsp pneumoniae	Blood culture	A	Y
Klebsiella pneumoniae	Blood culture	A	
Klebsiella pneumoniae	Blood culture	A	
Klebsiella pneumoniae	Blood culture	A	U
Proteus mirabilis	Blood culture	A	
Klebsiella pneumoniae	Blood culture	A	U
Klebsiella pneumoniae subsp pneumoniae	Blood culture	A	Y
Klebsiella pneumoniae	Blood culture	A	
Klebsiella pneumoniae subsp pneumoniae	Blood culture	A	Y
Klebsiella pneumoniae subsp pneumoniae	Blood culture	A	N
Klebsiella pneumoniae	Blood culture	A	Y
Klebsiella pneumoniae subsp pneumoniae	Blood culture	A	
Escherichia coli	Blood culture	A	U
Klebsiella pneumoniae subsp pneumoniae	Blood culture	A	
Enterobacter cloacae	Blood culture	A	
Enterobacter cloacae complex	Blood culture	A	
Enterobacter cloacae	Blood culture	A	
Klebsiella pneumoniae	Blood culture	A	Y
Klebsiella pneumoniae	Blood culture	A	Y
Klebsiella pneumoniae	Blood culture	A	Y

Klebsiella pneumoniae subsp pneumoniae	Blood culture	A	Y
Klebsiella pneumoniae subsp pneumoniae	Blood culture	A	Y
Klebsiella pneumoniae subsp pneumoniae	Blood culture	A	Y
Klebsiella pneumoniae	Blood culture	A	
Klebsiella pneumoniae	Blood culture	A	
Klebsiella pneumoniae	Blood culture	A	N
Klebsiella pneumoniae subsp pneumoniae	Blood culture	A	Y
Klebsiella pneumoniae subsp pneumoniae	Blood culture	A	Y
Klebsiella pneumoniae subsp pneumoniae	Blood culture	A	Y
Klebsiella pneumoniae subsp ozaenae	Blood culture	A	
Serratia marcescens	Blood culture	A	
Klebsiella pneumoniae	Blood culture	A	Y
Serratia fonticola	Blood culture	A	
Klebsiella pneumoniae subsp pneumoniae	Blood culture	A	Y
Klebsiella pneumoniae subsp pneumoniae	Blood culture	A	Y
Klebsiella pneumoniae	Blood culture	A	
Enterobacter cloacae	Blood culture	A	
Enterobacter aerogenes	Blood culture	A	
Klebsiella pneumoniae subsp pneumoniae	Blood culture	A	Y
Klebsiella pneumoniae	Blood culture	A	Y
Escherichia coli	Blood culture	A	
Klebsiella pneumoniae subsp pneumoniae	Blood culture	A	
Klebsiella pneumoniae	Blood culture	A	
Klebsiella pneumoniae	Blood culture	A	
Klebsiella pneumoniae	Blood culture	A	
Enterobacter cloacae subsp cloacae	Blood culture	A	Y
Klebsiella pneumoniae	Blood culture	A	N
Klebsiella pneumoniae subsp pneumoniae	Blood culture	A	Y
Klebsiella pneumoniae subsp pneumoniae	Blood culture	A	Y
Enterobacter cloacae subsp cloacae	Blood culture	A	
Enterobacter cloacae complex	Blood culture	A	Y
Klebsiella pneumoniae	Blood culture	A	
Enterobacter cloacae	Blood culture	A	
Klebsiella pneumoniae	Blood culture	A	Y
Enterobacter cloacae subsp cloacae	Blood culture	A	Y
Enterobacter cloacae	Blood culture	A	
Enterobacter cloacae	Blood culture	A	
Enterobacter cloacae	Blood culture	A	Y
Klebsiella pneumoniae	Blood culture	A	
Klebsiella pneumoniae subsp pneumoniae	Blood culture	A	Y
Enterobacter cloacae	Blood culture	A	Y
Klebsiella pneumoniae	Blood culture	A	
Klebsiella pneumoniae	Blood culture	A	Y
Klebsiella pneumoniae subsp pneumoniae	Blood culture	A	
Klebsiella pneumoniae	Blood culture	A	Y
Klebsiella pneumoniae subsp pneumoniae	Blood culture	A	
Klebsiella pneumoniae subsp pneumoniae	Blood culture	A	Y
Klebsiella pneumoniae subsp pneumoniae	Blood culture	A	Y
Klebsiella pneumoniae subsp pneumoniae	Blood culture	A	Y
Klebsiella pneumoniae subsp pneumoniae	Blood culture	A	
Klebsiella pneumoniae subsp pneumoniae	Blood culture	A	Y
Enterobacter cloacae	Blood culture	A	
Klebsiella pneumoniae subsp pneumoniae	Blood culture	A	
Enterobacter cloacae subsp cloacae	Blood culture	A	
Klebsiella pneumoniae subsp pneumoniae	Blood culture	A	
Klebsiella pneumoniae subsp pneumoniae	Blood culture	A	N

Klebsiella pneumoniae	Blood culture	A	
Klebsiella pneumoniae	Blood culture	A	Y
Escherichia coli	Blood culture	A	
Klebsiella pneumoniae subsp ozaenae	Blood culture	A	
Enterobacter cloacae	Blood culture	A	
Klebsiella pneumoniae subsp pneumoniae	Blood culture	A	
Citrobacter freundii	Blood culture	A	
Klebsiella pneumoniae	Blood culture	A	
Klebsiella pneumoniae subsp pneumoniae	Blood culture	A	
Serratia fonticola	Blood culture	A	
Klebsiella pneumoniae subsp pneumoniae	Blood culture	A	
Klebsiella pneumoniae subsp pneumoniae	Blood culture	A	
Klebsiella pneumoniae	Blood culture	A	N
Klebsiella pneumoniae subsp pneumoniae	Blood culture	A	N
Klebsiella pneumoniae subsp pneumoniae	Blood culture	A	Y
Enterobacter aerogenes	Blood culture	A	
Klebsiella pneumoniae	Blood culture	A	
Klebsiella pneumoniae	Blood culture	A	Y
Proteus mirabilis	Blood culture	A	
Klebsiella pneumoniae subsp pneumoniae	Blood culture	A	
Klebsiella pneumoniae subsp pneumoniae	Blood culture	A	Y
Klebsiella pneumoniae	Blood culture	A	
Enterobacter cloacae	Blood culture	A	
Klebsiella pneumoniae	Blood culture	A	
Klebsiella pneumoniae	Blood culture	A	
Klebsiella pneumoniae subsp pneumoniae	Blood culture	A	
Klebsiella pneumoniae subsp pneumoniae	Blood culture	A	N
Klebsiella pneumoniae subsp pneumoniae	Blood culture	A	Y
Klebsiella pneumoniae	Blood culture	A	Y
Enterobacter cloacae	Blood culture	A	
Klebsiella pneumoniae subsp pneumoniae	Blood culture	A	
Klebsiella pneumoniae subsp pneumoniae	Blood culture	A	
Klebsiella pneumoniae	Blood culture	A	
Klebsiella pneumoniae	Blood culture	A	N
Klebsiella pneumoniae subsp pneumoniae	Blood culture	A	Y
Klebsiella pneumoniae	Blood culture	A	
Enterobacter aerogenes	Blood culture	A	
Klebsiella pneumoniae subsp pneumoniae	Blood culture	A	Y
Klebsiella oxytoca	Blood culture	A	U
Klebsiella pneumoniae subsp pneumoniae	Blood culture	A	U
Enterobacter cloacae subsp cloacae	Blood culture	A	
Klebsiella pneumoniae subsp pneumoniae	Blood culture	A	Y
Klebsiella pneumoniae subsp pneumoniae	Blood culture	A	
Enterobacter aerogenes	Blood culture	A	
Serratia marcescens	Blood culture	A	Y
Klebsiella pneumoniae subsp pneumoniae	Blood culture	A	
Klebsiella pneumoniae	Blood culture	A	Y
Klebsiella pneumoniae	Blood culture	A	Y
Klebsiella pneumoniae	Blood culture	A	
Enterobacter cloacae subsp cloacae	Blood culture	A	Y
Klebsiella pneumoniae subsp pneumoniae	Blood culture	A	Y
Klebsiella pneumoniae	Blood culture	A	
Enterobacter aerogenes	Blood culture	A	Y
Klebsiella pneumoniae	Blood culture	A	
Proteus mirabilis	Blood culture	A	
Klebsiella pneumoniae	Blood culture	A	Y

Enterobacter cloacae complex	Blood culture	A	Y
Klebsiella pneumoniae subsp pneumoniae	Blood culture	A	Y
Klebsiella pneumoniae	Blood culture	A	
Klebsiella pneumoniae subsp pneumoniae	Blood culture	A	Y
Klebsiella pneumoniae subsp pneumoniae	Blood culture	A	U
Enterobacter aerogenes	Blood culture	A	
Klebsiella pneumoniae subsp pneumoniae	Blood culture	A	
Klebsiella pneumoniae subsp pneumoniae	Blood culture	A	
Klebsiella pneumoniae	Blood culture	A	
Klebsiella pneumoniae subsp pneumoniae	Blood culture	A	
Klebsiella pneumoniae subsp pneumoniae	Blood culture	A	Y
Klebsiella pneumoniae subsp pneumoniae	Blood culture	M	Y
Klebsiella pneumoniae subsp pneumoniae	Blood culture	M	Y
Klebsiella pneumoniae subsp pneumoniae	Blood culture	M	Y
Klebsiella pneumoniae	Blood culture	M	N
Klebsiella pneumoniae	Blood culture	M	Y
Klebsiella pneumoniae	Blood culture	M	N
Serratia marcescens	Blood culture	M	Y
Klebsiella pneumoniae	Blood culture	M	Y
Escherichia coli	Blood culture		
Serratia marcescens	Blood culture		
Serratia marcescens	Blood culture	V	
Enterobacter aerogenes	Blood culture	M	Y
Klebsiella pneumoniae	Blood culture	M	Y
Klebsiella pneumoniae subsp pneumoniae	Blood culture	V	
Klebsiella pneumoniae subsp pneumoniae	Blood culture	V	
Klebsiella pneumoniae	Blood culture	V	
Klebsiella pneumoniae subsp pneumoniae	Blood culture	V	
Klebsiella pneumoniae subsp pneumoniae	Blood culture	V	
Klebsiella pneumoniae subsp pneumoniae	Blood culture	V	
Klebsiella pneumoniae subsp pneumoniae	Blood culture	V	
Klebsiella pneumoniae	Blood culture	V	
Klebsiella pneumoniae	Blood culture	V	
Klebsiella pneumoniae subsp pneumoniae	Blood culture	V	
Klebsiella pneumoniae	Blood culture	V	
Klebsiella pneumoniae subsp pneumoniae	Blood culture	V	
Serratia marcescens	Blood culture	V	
Klebsiella pneumoniae	Blood culture	V	
Klebsiella pneumoniae subsp pneumoniae	Blood culture	V	
Providencia rettgeri	Blood culture	V	
Klebsiella pneumoniae	Blood culture	V	
Klebsiella pneumoniae subsp pneumoniae	Blood culture	V	
Klebsiella pneumoniae subsp pneumoniae	Blood culture	V	
Klebsiella pneumoniae subsp pneumoniae	Blood culture	V	
Providencia rettgeri	Blood culture	V	
Klebsiella pneumoniae subsp pneumoniae	Blood culture	V	
Klebsiella pneumoniae	Blood culture	V	
Klebsiella pneumoniae subsp pneumoniae	Blood culture	V	
Klebsiella pneumoniae subsp pneumoniae	Blood culture	V	
Providencia rettgeri	Blood culture	V	
Klebsiella pneumoniae subsp pneumoniae	Blood culture	V	
Klebsiella pneumoniae subsp pneumoniae	Blood culture	V	
Klebsiella pneumoniae	Blood culture	V	
Klebsiella pneumoniae	Blood culture	V	
Klebsiella pneumoniae	Blood culture	V	
Klebsiella pneumoniae	Blood culture	V	

Klebsiella pneumoniae subsp pneumoniae	Blood culture	V
Klebsiella pneumoniae subsp ozaenae	Blood culture	V
Klebsiella pneumoniae subsp pneumoniae	Blood culture	V
Klebsiella pneumoniae subsp pneumoniae	Blood culture	V
Enterobacter cloacae	Blood culture	V
Klebsiella pneumoniae subsp pneumoniae	Blood culture	V
Klebsiella pneumoniae subsp pneumoniae	Blood culture	V
Klebsiella pneumoniae subsp pneumoniae	Blood culture	V
Klebsiella pneumoniae subsp pneumoniae	Blood culture	V
Klebsiella pneumoniae subsp pneumoniae	Blood culture	V
Klebsiella pneumoniae subsp pneumoniae	Blood culture	V
Klebsiella pneumoniae subsp pneumoniae	Blood culture	V
Klebsiella pneumoniae subsp pneumoniae	Blood culture	V
Klebsiella pneumoniae subsp pneumoniae	Blood culture	V
Klebsiella pneumoniae subsp pneumoniae	Blood culture	V
Enterobacter cloacae	Blood culture	V
Klebsiella pneumoniae	Blood culture	V
Klebsiella pneumoniae subsp pneumoniae	Blood culture	V
Klebsiella pneumoniae subsp pneumoniae	Blood culture	V
Klebsiella pneumoniae subsp pneumoniae	Blood culture	V
Klebsiella pneumoniae subsp pneumoniae	Blood culture	V
Klebsiella pneumoniae subsp pneumoniae	Blood culture	V
Klebsiella pneumoniae subsp pneumoniae	Blood culture	V
Klebsiella pneumoniae subsp pneumoniae	Blood culture	V
Klebsiella pneumoniae subsp pneumoniae	Blood culture	V
Klebsiella pneumoniae subsp pneumoniae	Blood culture	V
Klebsiella pneumoniae subsp pneumoniae	Blood culture	V
Providencia rettgeri	Blood culture	V
Klebsiella pneumoniae subsp pneumoniae	Blood culture	V
Klebsiella pneumoniae subsp pneumoniae	Blood culture	V
Klebsiella pneumoniae subsp pneumoniae	Blood culture	V
Klebsiella pneumoniae subsp pneumoniae	Blood culture	V
Serratia marcescens	Blood culture	V
Klebsiella pneumoniae subsp pneumoniae	Blood culture	V
Klebsiella pneumoniae subsp pneumoniae	Blood culture	V
Providencia rettgeri	Blood culture	V
Klebsiella pneumoniae subsp pneumoniae	Blood culture	V
Klebsiella pneumoniae subsp pneumoniae	Blood culture	V
Klebsiella pneumoniae subsp pneumoniae	Blood culture	V
Klebsiella pneumoniae subsp pneumoniae	Blood culture	V
Klebsiella pneumoniae subsp pneumoniae	Blood culture	V
Klebsiella pneumoniae subsp pneumoniae	Blood culture	V
Klebsiella pneumoniae subsp pneumoniae	Blood culture	V
Klebsiella pneumoniae subsp pneumoniae	Blood culture	V
Klebsiella pneumoniae subsp pneumoniae	Blood culture	V
Klebsiella pneumoniae	Blood culture	V
Klebsiella pneumoniae	Blood culture	V
Klebsiella pneumoniae subsp pneumoniae	Blood culture	V
Klebsiella pneumoniae	Blood culture	V
Klebsiella pneumoniae	Blood culture	V
Klebsiella pneumoniae	Blood culture	V
Klebsiella pneumoniae	Blood culture	V
Klebsiella pneumoniae	Blood culture	V
Enterobacter cloacae	Blood culture	V
Klebsiella pneumoniae	Blood culture	V
Klebsiella pneumoniae	Blood culture	V

Klebsiella pneumoniae	Blood culture	V
Klebsiella pneumoniae	Blood culture	V
Klebsiella pneumoniae	Blood culture	V
Klebsiella pneumoniae subsp pneumoniae	Blood culture	V
Klebsiella pneumoniae subsp pneumoniae	Blood culture	V
Klebsiella pneumoniae subsp pneumoniae	Blood culture	V
Klebsiella pneumoniae subsp pneumoniae	Blood culture	V
Klebsiella pneumoniae subsp pneumoniae	Blood culture	V
Serratia marcescens	Blood culture	V
Klebsiella pneumoniae subsp pneumoniae	Blood culture	V
Enterobacter cloacae	Blood culture	V
Proteus mirabilis	Blood culture	V
Klebsiella pneumoniae subsp pneumoniae	Blood culture	V
Klebsiella pneumoniae subsp pneumoniae	Blood culture	V
Enterobacter cloacae	Blood culture	V
Klebsiella pneumoniae subsp pneumoniae	Blood culture	V
Klebsiella pneumoniae subsp pneumoniae	Blood culture	V
Klebsiella pneumoniae subsp pneumoniae	Blood culture	V
Klebsiella pneumoniae subsp pneumoniae	Blood culture	V
Klebsiella pneumoniae	Blood culture	V
Klebsiella pneumoniae	Blood culture	V
Klebsiella pneumoniae subsp pneumoniae	Blood culture	V
Klebsiella pneumoniae subsp pneumoniae	Blood culture	V
Klebsiella pneumoniae subsp pneumoniae	Blood culture	V
Klebsiella pneumoniae subsp pneumoniae	Blood culture	V
Klebsiella pneumoniae subsp pneumoniae	Blood culture	V
Klebsiella pneumoniae	Blood culture	V
Klebsiella pneumoniae	Blood culture	V
Klebsiella pneumoniae subsp pneumoniae	Blood culture	V
Klebsiella pneumoniae subsp pneumoniae	Blood culture	V
Klebsiella pneumoniae subsp pneumoniae	Blood culture	V
Klebsiella pneumoniae subsp pneumoniae	Blood culture	V
Klebsiella pneumoniae subsp pneumoniae	Blood culture	V
Klebsiella pneumoniae subsp pneumoniae	Blood culture	V
Klebsiella pneumoniae subsp pneumoniae	Blood culture	V
Klebsiella pneumoniae subsp pneumoniae	Blood culture	V
Serratia marcescens	Blood culture	V
Klebsiella pneumoniae subsp pneumoniae	Blood culture	V
Klebsiella pneumoniae subsp pneumoniae	Blood culture	V
Klebsiella pneumoniae	Blood culture	V
Klebsiella pneumoniae	Blood culture	V
Serratia marcescens	Blood culture	V
Klebsiella pneumoniae	Blood culture	V
Klebsiella pneumoniae	Blood culture	V
Klebsiella pneumoniae	Blood culture	V
Klebsiella pneumoniae subsp pneumoniae	Blood culture	V
Klebsiella pneumoniae	Blood culture	V
Klebsiella pneumoniae subsp pneumoniae	Blood culture	V
Klebsiella pneumoniae subsp pneumoniae	Blood culture	V
Klebsiella pneumoniae subsp pneumoniae	Blood culture	V
Klebsiella pneumoniae subsp pneumoniae	Blood culture	V
Klebsiella pneumoniae subsp pneumoniae	Blood culture	V
Enterobacter asburiae	Blood culture	V
Klebsiella pneumoniae subsp pneumoniae	Blood culture	V
Klebsiella pneumoniae subsp pneumoniae	Blood culture	V
Serratia marcescens	Blood culture	V

Enterobacter cloacae	Blood culture	V
Klebsiella pneumoniae subsp pneumoniae	Blood culture	V
Serratia marcescens	Blood culture	V
Klebsiella pneumoniae subsp pneumoniae	Blood culture	V
Klebsiella pneumoniae subsp pneumoniae	Blood culture	V
Serratia marcescens	Blood culture	V
Serratia marcescens	Blood culture	V
Providencia stuartii	Blood culture	V
Klebsiella pneumoniae subsp pneumoniae	Blood culture	V
Klebsiella pneumoniae subsp pneumoniae	Blood culture	V
Klebsiella pneumoniae subsp pneumoniae	Blood culture	V
Klebsiella pneumoniae subsp pneumoniae	Blood culture	V
Klebsiella pneumoniae subsp pneumoniae	Blood culture	V
Klebsiella pneumoniae	Blood culture	V
Enterobacter cloacae	Blood culture	V
Enterobacter cloacae	Blood culture	V
Klebsiella pneumoniae	Blood culture	V
Enterobacter cloacae	Blood culture	V
Klebsiella pneumoniae	Blood culture	V
Klebsiella pneumoniae	Blood culture	V
Klebsiella pneumoniae	Blood culture	V
Klebsiella pneumoniae subsp pneumoniae	Blood culture	V
Klebsiella pneumoniae subsp pneumoniae	Blood culture	V
Klebsiella pneumoniae subsp pneumoniae	Blood culture	V
Klebsiella pneumoniae subsp pneumoniae	Blood culture	V
Serratia marcescens	Blood culture	V
Klebsiella pneumoniae	Blood culture	V
Klebsiella pneumoniae	Blood culture	V
Klebsiella pneumoniae	Blood culture	V
Klebsiella pneumoniae	Blood culture	V
Klebsiella pneumoniae	Blood culture	V
Klebsiella pneumoniae	Blood culture	V
Klebsiella pneumoniae subsp pneumoniae	Blood culture	V
Klebsiella pneumoniae subsp pneumoniae	Blood culture	V
Klebsiella pneumoniae subsp pneumoniae	Blood culture	V
Klebsiella pneumoniae subsp pneumoniae	Blood culture	V
Klebsiella pneumoniae subsp pneumoniae	Blood culture	V
Providencia stuartii	Blood culture	V
Klebsiella pneumoniae subsp pneumoniae	Blood culture	V
Enterobacter cloacae	Blood culture	V
Klebsiella pneumoniae subsp pneumoniae	Blood culture	V
Klebsiella pneumoniae subsp pneumoniae	Blood culture	V
Klebsiella pneumoniae subsp pneumoniae	Blood culture	V
Klebsiella pneumoniae subsp pneumoniae	Blood culture	V
Serratia marcescens	Blood culture	V
Klebsiella pneumoniae subsp pneumoniae	Blood culture	V
Klebsiella pneumoniae subsp pneumoniae	Blood culture	V
Klebsiella pneumoniae	Blood culture	V
Klebsiella pneumoniae subsp pneumoniae	Blood culture	V
Klebsiella pneumoniae	Blood culture	V
Klebsiella pneumoniae subsp pneumoniae	Blood culture	V
Klebsiella pneumoniae subsp pneumoniae	Blood culture	V
Klebsiella pneumoniae	Blood culture	V
Serratia marcescens	Blood culture	V
Klebsiella pneumoniae	Blood culture	V
Klebsiella pneumoniae subsp pneumoniae	Blood culture	V

Klebsiella pneumoniae subsp pneumoniae	Blood culture	V
Klebsiella pneumoniae subsp pneumoniae	Blood culture	V
Serratia marcescens	Blood culture	V
Klebsiella pneumoniae subsp pneumoniae	Blood culture	V
Klebsiella pneumoniae subsp pneumoniae	Blood culture	V
Klebsiella pneumoniae subsp pneumoniae	Blood culture	V
Klebsiella pneumoniae subsp pneumoniae	Blood culture	V
Citrobacter braakii	Blood culture	V
Enterobacter cloacae	Blood culture	V
Klebsiella pneumoniae subsp pneumoniae	Blood culture	V
Klebsiella pneumoniae subsp pneumoniae	Blood culture	V
Escherichia coli	Blood culture	V
Klebsiella pneumoniae subsp pneumoniae	Blood culture	V
Klebsiella pneumoniae subsp pneumoniae	Blood culture	V
Klebsiella pneumoniae subsp pneumoniae	Blood culture	V
Klebsiella pneumoniae subsp pneumoniae	Blood culture	V
Klebsiella pneumoniae	Blood culture	V
Klebsiella pneumoniae subsp pneumoniae	Blood culture	V
Klebsiella pneumoniae subsp pneumoniae	Blood culture	V
Klebsiella pneumoniae subsp pneumoniae	Blood culture	V
Klebsiella pneumoniae subsp pneumoniae	Blood culture	V
Proteus mirabilis	Blood culture	V
Klebsiella pneumoniae subsp pneumoniae	Blood culture	V
Klebsiella pneumoniae subsp pneumoniae	Blood culture	V
Klebsiella pneumoniae subsp pneumoniae	Blood culture	V
Klebsiella pneumoniae subsp pneumoniae	Blood culture	V
Serratia marcescens	Blood culture	V
Klebsiella pneumoniae subsp pneumoniae	Blood culture	V
Klebsiella pneumoniae subsp pneumoniae	Blood culture	V
Klebsiella pneumoniae	Blood culture	V
Klebsiella pneumoniae	Blood culture	V
Klebsiella pneumoniae subsp pneumoniae	Blood culture	V
Klebsiella pneumoniae subsp pneumoniae	Blood culture	V
Klebsiella pneumoniae subsp pneumoniae	Blood culture	V
Enterobacter cloacae	Blood culture	V
Klebsiella pneumoniae subsp pneumoniae	Blood culture	V
Serratia marcescens	Blood culture	V
Serratia marcescens	Blood culture	N
Klebsiella pneumoniae subsp pneumoniae	Blood culture	V
Klebsiella pneumoniae subsp pneumoniae	Blood culture	V
Klebsiella pneumoniae subsp pneumoniae	Blood culture	V
Klebsiella pneumoniae subsp pneumoniae	Blood culture	V
Proteus mirabilis	Blood culture	V
Klebsiella pneumoniae subsp pneumoniae	Blood culture	V
Serratia marcescens	Blood culture	V
Klebsiella pneumoniae subsp pneumoniae	Blood culture	V
Klebsiella pneumoniae subsp pneumoniae	Blood culture	V
Klebsiella pneumoniae subsp pneumoniae	Blood culture	V
Klebsiella pneumoniae	Blood culture	V
Klebsiella pneumoniae	Blood culture	V
Klebsiella pneumoniae subsp pneumoniae	Blood culture	V
Klebsiella pneumoniae subsp pneumoniae	Blood culture	V
Klebsiella pneumoniae subsp pneumoniae	Blood culture	V
Klebsiella pneumoniae subsp pneumoniae	Blood culture	V
Klebsiella pneumoniae	Blood culture	V
Klebsiella pneumoniae subsp pneumoniae	Blood culture	V

Klebsiella pneumoniae	Blood culture	V
Klebsiella pneumoniae	Blood culture	V
Klebsiella pneumoniae	Blood culture	V
Klebsiella pneumoniae	Blood culture	V
Klebsiella pneumoniae	Blood culture	V
Klebsiella pneumoniae subsp pneumoniae	Blood culture	V
Klebsiella pneumoniae	Blood culture	V
Klebsiella pneumoniae	Blood culture	V
Klebsiella pneumoniae	Blood culture	V
Klebsiella pneumoniae subsp pneumoniae	Blood culture	V
Klebsiella pneumoniae subsp pneumoniae	Blood culture	V
Klebsiella pneumoniae subsp pneumoniae	Blood culture	V
Klebsiella pneumoniae subsp pneumoniae	Blood culture	V
Klebsiella pneumoniae subsp pneumoniae	Blood culture	V
Enterobacter aerogenes	Blood culture	V
Proteus mirabilis	Blood culture	V
Klebsiella pneumoniae subsp pneumoniae	Blood culture	V
Klebsiella pneumoniae subsp pneumoniae	Blood culture	V
Serratia marcescens	Blood culture	V
Klebsiella pneumoniae subsp pneumoniae	Blood culture	V
Klebsiella pneumoniae	Blood culture	V
Klebsiella pneumoniae subsp pneumoniae	Blood culture	V
Klebsiella pneumoniae subsp pneumoniae	Blood culture	V
Escherichia coli	Blood culture	V
Klebsiella pneumoniae	Blood culture	V
Klebsiella pneumoniae	Blood culture	V
Enterobacter cloacae	Blood culture	V
Enterobacter cloacae subsp cloacae	Blood culture	A
Klebsiella pneumoniae subsp pneumoniae	Blood culture	A
Escherichia coli	Blood culture	A
Klebsiella pneumoniae	Blood culture	A
Klebsiella pneumoniae subsp pneumoniae	Blood culture	A
Enterobacter cloacae	Blood culture	A
Klebsiella pneumoniae	Blood culture	A
Klebsiella pneumoniae	Blood culture	A
Enterobacter cloacae	Blood culture	A
Klebsiella pneumoniae subsp pneumoniae	Blood culture	A
Klebsiella pneumoniae subsp pneumoniae	Blood culture	A
Serratia fonticola	Blood culture	A
Proteus mirabilis	Blood culture	A
Providencia rettgeri	Blood culture	A
Enterobacter cloacae subsp cloacae	Blood culture	A
Klebsiella pneumoniae subsp pneumoniae	Blood culture	A
Klebsiella pneumoniae subsp pneumoniae	Blood culture	A
Enterobacter cloacae subsp cloacae	Blood culture	A
Klebsiella pneumoniae subsp pneumoniae	Blood culture	A
Enterobacter cloacae subsp cloacae	Blood culture	A
Escherichia coli	Blood culture	A
Klebsiella pneumoniae	Blood culture	A
Klebsiella pneumoniae	Blood culture	A
Klebsiella pneumoniae subsp pneumoniae	Blood culture	A
Enterobacter cloacae subsp cloacae	Blood culture	A
Serratia marcescens	Blood culture	A
Klebsiella pneumoniae subsp pneumoniae	Blood culture	A
Klebsiella pneumoniae	Blood culture	A
Enterobacter aerogenes	Blood culture	A

Enterobacter cloacae	Blood culture	A
Klebsiella pneumoniae	Blood culture	A
Klebsiella pneumoniae	Blood culture	A
Klebsiella pneumoniae subsp pneumoniae	Blood culture	A
Enterobacter cloacae	Blood culture	A
Klebsiella pneumoniae	Blood culture	A
Klebsiella pneumoniae	Blood culture	A
Enterobacter cloacae	Blood culture	A
Klebsiella pneumoniae subsp pneumoniae	Blood culture	A
Klebsiella pneumoniae subsp pneumoniae	Blood culture	A
Klebsiella pneumoniae subsp pneumoniae	Blood culture	A
Klebsiella pneumoniae subsp pneumoniae	Blood culture	A
Klebsiella pneumoniae	Blood culture	A
Enterobacter cloacae complex	Blood culture	A
Enterobacter cloacae complex	Blood culture	A
Klebsiella pneumoniae subsp pneumoniae	Blood culture	A
Klebsiella pneumoniae subsp pneumoniae	Blood culture	A
Klebsiella pneumoniae subsp pneumoniae	Blood culture	A
Serratia marcescens	Blood culture	A
Enterobacter cloacae complex	Blood culture	A
Klebsiella pneumoniae	Blood culture	A
Klebsiella pneumoniae	Blood culture	A
Klebsiella pneumoniae	Blood culture	A
Serratia fonticola	Blood culture	A
Klebsiella pneumoniae	Blood culture	A
Klebsiella pneumoniae subsp pneumoniae	Blood culture	A
Providencia rettgeri	Blood culture	A
Klebsiella pneumoniae subsp pneumoniae	Blood culture	A
Serratia marcescens	Blood culture	V
Morganella morganii	Blood culture	A
Klebsiella pneumoniae	Blood culture	A
Klebsiella pneumoniae subsp pneumoniae	Blood culture	A
Klebsiella pneumoniae subsp pneumoniae	Blood culture	A
Serratia fonticola	Blood culture	A
Klebsiella pneumoniae	Blood culture	A
Morganella morganii	Blood culture	A
Klebsiella pneumoniae	Blood culture	A
Klebsiella pneumoniae subsp pneumoniae	Blood culture	A
Escherichia coli	Blood culture	A
Klebsiella pneumoniae	Blood culture	A
Providencia rettgeri	Blood culture	A
Enterobacter aerogenes	Blood culture	A
Klebsiella pneumoniae	Blood culture	A
Providencia rettgeri	Blood culture	A
Klebsiella pneumoniae subsp pneumoniae	Blood culture	A
Klebsiella pneumoniae subsp pneumoniae	Blood culture	A
Klebsiella pneumoniae	Blood culture	A
Klebsiella pneumoniae subsp pneumoniae	Blood culture	A
Enterobacter cloacae	Blood culture	A
Escherichia coli	Blood culture	A
Klebsiella pneumoniae subsp pneumoniae	Blood culture	A
Proteus mirabilis	Blood culture	A
Enterobacter cloacae complex	Blood culture	A
Klebsiella pneumoniae subsp pneumoniae	Blood culture	A
Enterobacter aerogenes	Blood culture	A
Enterobacter cloacae subsp cloacae	Blood culture	A

Klebsiella pneumoniae	Blood culture	A
Providencia stuartii	Blood culture	A
Klebsiella pneumoniae subsp pneumoniae	Blood culture	A
Klebsiella pneumoniae subsp pneumoniae	Blood culture	A
Klebsiella pneumoniae subsp pneumoniae	Blood culture	A
Enterobacter aerogenes	Blood culture	A
Enterobacter cloacae	Blood culture	A
Klebsiella pneumoniae	Blood culture	A
Enterobacter cloacae subsp cloacae	Blood culture	A
Escherichia coli	Blood culture	A
Klebsiella pneumoniae subsp pneumoniae	Blood culture	A
Proteus hauseri	Blood culture	A
Klebsiella pneumoniae	Blood culture	A
Klebsiella pneumoniae subsp pneumoniae	Blood culture	A
Enterobacter cloacae	Blood culture	A
Klebsiella pneumoniae	Blood culture	A
Enterobacter cloacae	Blood culture	A
Klebsiella pneumoniae subsp pneumoniae	Blood culture	V
Klebsiella pneumoniae	Blood culture	A
Klebsiella pneumoniae	Blood culture	A
Klebsiella pneumoniae subsp pneumoniae	Blood culture	A
Enterobacter cloacae	Blood culture	A
Enterobacter cloacae complex	Blood culture	A
Klebsiella pneumoniae	Blood culture	A
Klebsiella pneumoniae subsp pneumoniae	Blood culture	A
Enterobacter cloacae complex	Blood culture	A
Klebsiella pneumoniae subsp pneumoniae	Blood culture	A
Klebsiella pneumoniae subsp pneumoniae	Blood culture	A
Escherichia coli	Blood culture	A
Klebsiella pneumoniae	Blood culture	A
Klebsiella pneumoniae subsp pneumoniae	Blood culture	A
Enterobacter cloacae subsp cloacae	Blood culture	A
Klebsiella pneumoniae	Blood culture	A
Escherichia coli	Blood culture	A
Escherichia coli	Blood culture	A
Klebsiella pneumoniae	Blood culture	A
Klebsiella pneumoniae	Blood culture	A
Enterobacter cloacae	Blood culture	A
Enterobacter cloacae complex	Blood culture	A
Klebsiella pneumoniae	Blood culture	A
Klebsiella pneumoniae subsp pneumoniae	Blood culture	V
Klebsiella pneumoniae subsp pneumoniae	Blood culture	A
Proteus mirabilis	Blood culture	A
Klebsiella pneumoniae	Blood culture	A
Klebsiella pneumoniae subsp pneumoniae	Blood culture	A
Klebsiella pneumoniae subsp pneumoniae	Blood culture	A
Klebsiella pneumoniae subsp pneumoniae	Blood culture	A
Klebsiella pneumoniae subsp pneumoniae	Blood culture	A
Klebsiella pneumoniae	Blood culture	A
Klebsiella pneumoniae subsp ozaenae	Blood culture	A
Klebsiella pneumoniae subsp pneumoniae	Blood culture	A
Enterobacter cloacae subsp cloacae	Blood culture	A
Escherichia coli	Blood culture	A
Klebsiella pneumoniae subsp pneumoniae	Blood culture	A
Proteus vulgaris	Blood culture	A
Klebsiella pneumoniae	Blood culture	A

Klebsiella pneumoniae	Blood culture	A
Klebsiella pneumoniae subsp pneumoniae	Blood culture	A
Proteus mirabilis	Blood culture	A
Providencia stuartii	Blood culture	A
Escherichia coli	Blood culture	A
Klebsiella pneumoniae subsp pneumoniae	Blood culture	A
Klebsiella pneumoniae subsp pneumoniae	Blood culture	A
Klebsiella pneumoniae subsp pneumoniae	Blood culture	A
Serratia liquefaciens group	Blood culture	A
Klebsiella pneumoniae	Blood culture	A
Klebsiella pneumoniae subsp pneumoniae	Blood culture	A
Klebsiella pneumoniae subsp pneumoniae	Blood culture	A
Klebsiella pneumoniae subsp pneumoniae	Blood culture	A
Proteus mirabilis	Blood culture	A
Serratia fonticola	Blood culture	A
Enterobacter cloacae complex	Blood culture	A
Klebsiella pneumoniae	Blood culture	A
Klebsiella pneumoniae subsp pneumoniae	Blood culture	A
Klebsiella pneumoniae subsp pneumoniae	Blood culture	A
Klebsiella pneumoniae	Blood culture	A
Klebsiella pneumoniae	Blood culture	V
Klebsiella pneumoniae subsp pneumoniae	Blood culture	A
Klebsiella pneumoniae subsp pneumoniae	Blood culture	A
Klebsiella pneumoniae	Blood culture	A
Klebsiella pneumoniae	Blood culture	A
Klebsiella pneumoniae	Blood culture	A
Klebsiella pneumoniae	Blood culture	A
Klebsiella pneumoniae subsp pneumoniae	Blood culture	A
Enterobacter aerogenes	Blood culture	A
Klebsiella pneumoniae	Blood culture	A
Klebsiella pneumoniae	Blood culture	A
Citrobacter freundii	Blood culture	A
Klebsiella pneumoniae subsp pneumoniae	Blood culture	A
Klebsiella pneumoniae subsp pneumoniae	Blood culture	A
Enterobacter cloacae complex	Blood culture	A
Klebsiella pneumoniae subsp pneumoniae	Blood culture	A
Klebsiella pneumoniae subsp pneumoniae	Blood culture	A
Klebsiella pneumoniae	Blood culture	A
Citrobacter freundii	Blood culture	V
Klebsiella pneumoniae	Blood culture	V
Enterobacter cloacae	Blood culture	V
Providencia stuartii	Blood culture	V
Klebsiella pneumoniae	Blood culture	V
Klebsiella pneumoniae subsp pneumoniae	Blood culture	V
Klebsiella pneumoniae subsp pneumoniae	Blood culture	V
Klebsiella pneumoniae	Blood culture	V
Klebsiella pneumoniae	Blood culture	V
Klebsiella pneumoniae subsp pneumoniae	Blood culture	V
Klebsiella pneumoniae	Blood culture	V
Klebsiella pneumoniae subsp pneumoniae	Blood culture	V
Klebsiella pneumoniae subsp pneumoniae	Blood culture	V
Klebsiella pneumoniae subsp pneumoniae	Blood culture	V
Klebsiella pneumoniae subsp pneumoniae	Blood culture	V
Klebsiella pneumoniae subsp pneumoniae	Blood culture	V
Enterobacter cloacae	Blood culture	V
Klebsiella pneumoniae subsp pneumoniae	Blood culture	V

Klebsiella pneumoniae subsp pneumoniae	Blood culture	V
Serratia marcescens	Blood culture	V
Klebsiella pneumoniae subsp pneumoniae	Blood culture	V
Klebsiella pneumoniae	Blood culture	V
Klebsiella pneumoniae	Blood culture	V
Klebsiella pneumoniae	Blood culture	V
Klebsiella pneumoniae	Blood culture	V
Klebsiella pneumoniae	Blood culture	V
Klebsiella pneumoniae subsp pneumoniae	Blood culture	V
Klebsiella pneumoniae subsp pneumoniae	Blood culture	V
Klebsiella pneumoniae subsp ozaenae	Blood culture	V
Klebsiella pneumoniae subsp pneumoniae	Blood culture	V
Klebsiella pneumoniae subsp pneumoniae	Blood culture	V
Klebsiella pneumoniae subsp pneumoniae	Blood culture	V
Klebsiella pneumoniae subsp pneumoniae	Blood culture	V
Enterobacter cloacae	Blood culture	V
Klebsiella pneumoniae subsp pneumoniae	Blood culture	V
Enterobacter aerogenes	Blood culture	V
Klebsiella pneumoniae subsp pneumoniae	Blood culture	V
Proteus mirabilis	Blood culture	V
Proteus mirabilis	Blood culture	V
Enterobacter aerogenes	Blood culture	V
Proteus mirabilis	Blood culture	V
Enterobacter aerogenes	Blood culture	V
Klebsiella pneumoniae	Blood culture	V
Klebsiella pneumoniae subsp pneumoniae	Blood culture	V
Klebsiella pneumoniae subsp pneumoniae	Blood culture	V
Klebsiella pneumoniae	Blood culture	V
Enterobacter cloacae	Blood culture	V
Klebsiella pneumoniae subsp pneumoniae	Blood culture	V
Klebsiella pneumoniae subsp pneumoniae	Blood culture	V
Klebsiella pneumoniae subsp pneumoniae	Blood culture	V
Klebsiella pneumoniae subsp pneumoniae	Blood culture	V
Klebsiella pneumoniae subsp pneumoniae	Blood culture	V
Enterobacter aerogenes	Blood culture	V
Klebsiella pneumoniae subsp pneumoniae	Blood culture	V
Klebsiella pneumoniae subsp pneumoniae	Blood culture	V
Enterobacter aerogenes	Blood culture	V
Klebsiella pneumoniae	Blood culture	V
Klebsiella pneumoniae	Blood culture	V
Escherichia coli	Blood culture	V
Enterobacter cloacae	Blood culture	V
Klebsiella pneumoniae	Blood culture	V
Klebsiella pneumoniae	Blood culture	V
Klebsiella pneumoniae	Blood culture	V
Klebsiella pneumoniae subsp pneumoniae	Blood culture	V
Klebsiella pneumoniae subsp pneumoniae	Blood culture	V
Proteus mirabilis	Blood culture	V
Klebsiella pneumoniae subsp pneumoniae	Blood culture	V
Klebsiella pneumoniae	Blood culture	V
Klebsiella pneumoniae	Blood culture	V
Klebsiella pneumoniae subsp pneumoniae	Blood culture	V
Klebsiella pneumoniae subsp pneumoniae	Blood culture	V
Klebsiella pneumoniae subsp pneumoniae	Blood culture	V
Klebsiella pneumoniae subsp pneumoniae	Blood culture	V
Serratia marcescens	Blood culture	V

Klebsiella pneumoniae	Blood culture	V
Klebsiella pneumoniae	Blood culture	V
Enterobacter cloacae	Blood culture	V
Klebsiella pneumoniae subsp pneumoniae	Blood culture	V
Enterobacter cloacae	Blood culture	M
Klebsiella pneumoniae subsp pneumoniae	Blood culture	V
Serratia marcescens	Blood culture	V
Klebsiella pneumoniae subsp pneumoniae	Blood culture	V
Klebsiella pneumoniae subsp pneumoniae	Blood culture	V
Klebsiella pneumoniae subsp pneumoniae	Blood culture	V
Klebsiella pneumoniae subsp pneumoniae	Blood culture	V
Klebsiella pneumoniae subsp pneumoniae	Blood culture	V
Proteus mirabilis	Blood culture	V
Klebsiella pneumoniae subsp pneumoniae	Blood culture	V
Klebsiella pneumoniae subsp pneumoniae	Blood culture	V
Proteus mirabilis	Blood culture	V
Escherichia coli	Blood culture	V
Klebsiella pneumoniae subsp pneumoniae	Blood culture	V
Klebsiella pneumoniae	Blood culture	V
Klebsiella pneumoniae subsp pneumoniae	Blood culture	V
Klebsiella pneumoniae subsp pneumoniae	Blood culture	V
Enterobacter cloacae	Blood culture	V
Klebsiella pneumoniae subsp pneumoniae	Blood culture	V
Klebsiella pneumoniae subsp pneumoniae	Blood culture	V
Klebsiella pneumoniae subsp pneumoniae	Blood culture	V
Klebsiella pneumoniae subsp pneumoniae	Blood culture	V
Serratia marcescens	Blood culture	V
Proteus mirabilis	Blood culture	V
Proteus mirabilis	Blood culture	V
Klebsiella pneumoniae subsp pneumoniae	Blood culture	V
Klebsiella pneumoniae subsp pneumoniae	Blood culture	V
Klebsiella pneumoniae subsp pneumoniae	Blood culture	V
Proteus mirabilis	Blood culture	V
Klebsiella pneumoniae subsp pneumoniae	Blood culture	V
Klebsiella pneumoniae subsp pneumoniae	Blood culture	V
Klebsiella pneumoniae	Blood culture	V
Klebsiella pneumoniae	Blood culture	V
Klebsiella pneumoniae	Blood culture	V
Klebsiella pneumoniae	Blood culture	V
Klebsiella pneumoniae	Blood culture	V
Klebsiella pneumoniae subsp pneumoniae	Blood culture	V
Serratia marcescens	Blood culture	V
Klebsiella pneumoniae subsp pneumoniae	Blood culture	V
Klebsiella pneumoniae subsp pneumoniae	Blood culture	V
Klebsiella pneumoniae subsp pneumoniae	Blood culture	V
Klebsiella pneumoniae subsp pneumoniae	Blood culture	V
Klebsiella pneumoniae subsp pneumoniae	Blood culture	V
Klebsiella pneumoniae subsp pneumoniae	Blood culture	V
Escherichia coli	Blood culture	V
Enterobacter aerogenes	Blood culture	V
Escherichia coli	Blood culture	V
Klebsiella pneumoniae subsp pneumoniae	Blood culture	V
Klebsiella pneumoniae subsp pneumoniae	Blood culture	V
Proteus mirabilis	Blood culture	V
Escherichia coli	Blood culture	V
Klebsiella pneumoniae subsp pneumoniae	Blood culture	V

Klebsiella pneumoniae subsp pneumoniae	Blood culture	V
Klebsiella pneumoniae subsp pneumoniae	Blood culture	V
Klebsiella pneumoniae subsp pneumoniae	Blood culture	V
Klebsiella pneumoniae subsp pneumoniae	Blood culture	V
Enterobacter aerogenes	Blood culture	V
Klebsiella pneumoniae subsp pneumoniae	Blood culture	V
Enterobacter cloacae	Blood culture	V
Klebsiella pneumoniae subsp pneumoniae	Blood culture	V
Klebsiella pneumoniae subsp pneumoniae	Blood culture	V
Klebsiella pneumoniae subsp pneumoniae	Blood culture	V
Klebsiella pneumoniae subsp pneumoniae	Blood culture	V
Klebsiella pneumoniae subsp pneumoniae	Blood culture	V
Klebsiella pneumoniae subsp pneumoniae	Blood culture	V
Klebsiella pneumoniae subsp pneumoniae	Blood culture	V
Klebsiella pneumoniae subsp pneumoniae	Blood culture	V
Klebsiella pneumoniae subsp pneumoniae	Blood culture	V
Klebsiella pneumoniae subsp pneumoniae	Blood culture	V
Klebsiella pneumoniae subsp pneumoniae	Blood culture	V
Escherichia coli	Blood culture	V
Klebsiella pneumoniae subsp pneumoniae	Blood culture	V
Morganella morganii	Blood culture	V
Klebsiella pneumoniae subsp pneumoniae	Blood culture	V
Klebsiella pneumoniae subsp pneumoniae	Blood culture	V
Klebsiella pneumoniae subsp pneumoniae	Blood culture	V
Klebsiella pneumoniae subsp pneumoniae	Blood culture	V
Klebsiella pneumoniae subsp pneumoniae	Blood culture	V
Klebsiella pneumoniae subsp pneumoniae	Blood culture	V
Enterobacter cloacae	Blood culture	V
Klebsiella pneumoniae subsp pneumoniae	Blood culture	V
Klebsiella pneumoniae subsp pneumoniae	Blood culture	V
Klebsiella pneumoniae subsp pneumoniae	Blood culture	V
Enterobacter cloacae	Blood culture	V
Enterobacter cloacae	Blood culture	V
Klebsiella pneumoniae subsp pneumoniae	Blood culture	V
Klebsiella pneumoniae	Blood culture	V
Klebsiella pneumoniae	Blood culture	V
Klebsiella pneumoniae subsp pneumoniae	Blood culture	V
Klebsiella pneumoniae	Blood culture	V
Klebsiella pneumoniae	Blood culture	V
Klebsiella pneumoniae subsp pneumoniae	Blood culture	V
Proteus mirabilis	Blood culture	V
Serratia marcescens	Blood culture	V
Serratia marcescens	Blood culture	V
Klebsiella pneumoniae subsp pneumoniae	Blood culture	V
Klebsiella pneumoniae subsp pneumoniae	Blood culture	V
Klebsiella pneumoniae	Blood culture	V
Klebsiella pneumoniae subsp pneumoniae	Blood culture	V
Klebsiella pneumoniae subsp pneumoniae	Blood culture	V
Escherichia coli	Blood culture	V
Klebsiella pneumoniae subsp pneumoniae	Blood culture	V
Klebsiella pneumoniae	Blood culture	V
Klebsiella pneumoniae	Blood culture	V
Klebsiella pneumoniae subsp pneumoniae	Blood culture	M
Klebsiella pneumoniae	Blood culture	V
Serratia marcescens	Blood culture	V
Klebsiella pneumoniae subsp pneumoniae	Blood culture	V

Klebsiella pneumoniae	Blood culture	V
Enterobacter cloacae	Blood culture	V
Klebsiella pneumoniae	Blood culture	V
Klebsiella pneumoniae	Blood culture	V
Klebsiella pneumoniae	Blood culture	V
Klebsiella oxytoca	Blood culture	V
Klebsiella pneumoniae subsp pneumoniae	Blood culture	V
Klebsiella pneumoniae	Blood culture	V
Klebsiella pneumoniae	Blood culture	V
Klebsiella pneumoniae subsp pneumoniae	Blood culture	V
Klebsiella pneumoniae subsp pneumoniae	Blood culture	V
Klebsiella pneumoniae subsp pneumoniae	Blood culture	V
Klebsiella pneumoniae subsp pneumoniae	Blood culture	V
Enterobacter cloacae	Blood culture	V
Klebsiella pneumoniae	Blood culture	V
Klebsiella pneumoniae	Blood culture	V
Enterobacter cloacae	Blood culture	V
Klebsiella pneumoniae	Blood culture	V
Klebsiella oxytoca	Blood culture	V
Klebsiella pneumoniae subsp pneumoniae	Blood culture	V
Serratia marcescens	Blood culture	V
Klebsiella oxytoca	Blood culture	V
Proteus mirabilis	Blood culture	V
Klebsiella pneumoniae subsp pneumoniae	Blood culture	V
Klebsiella pneumoniae subsp pneumoniae	Blood culture	V
Klebsiella pneumoniae subsp pneumoniae	Blood culture	V
Klebsiella pneumoniae	Blood culture	V
Klebsiella pneumoniae	Blood culture	V
Klebsiella pneumoniae	Blood culture	V
Klebsiella pneumoniae	Blood culture	V
Klebsiella pneumoniae subsp pneumoniae	Blood culture	V
Klebsiella pneumoniae	Blood culture	V
Klebsiella pneumoniae subsp pneumoniae	Blood culture	V
Enterobacter cloacae	Blood culture	V
Klebsiella pneumoniae subsp pneumoniae	Blood culture	V
Serratia marcescens	Blood culture	V
Klebsiella pneumoniae	Blood culture	V
Klebsiella pneumoniae	Blood culture	V
Klebsiella pneumoniae	Blood culture	V
Escherichia coli	Blood culture	V
Klebsiella pneumoniae	Blood culture	V
Klebsiella pneumoniae	Blood culture	V
Klebsiella pneumoniae	Blood culture	V
Klebsiella pneumoniae subsp pneumoniae	Blood culture	V
Klebsiella pneumoniae	Blood culture	V
Klebsiella pneumoniae subsp pneumoniae	Blood culture	V
Klebsiella pneumoniae subsp pneumoniae	Blood culture	V
Proteus mirabilis	Blood culture	V
Enterobacter cloacae	Blood culture	V
Klebsiella pneumoniae subsp pneumoniae	Blood culture	V
Klebsiella pneumoniae subsp pneumoniae	Blood culture	V
Escherichia coli	Blood culture	V
Klebsiella pneumoniae subsp pneumoniae	Blood culture	V
Klebsiella pneumoniae	Blood culture	V
Serratia marcescens	Blood culture	V
Proteus mirabilis	Blood culture	V

Enterobacter cloacae	Blood culture	V
Klebsiella pneumoniae subsp pneumoniae	Blood culture	V
Klebsiella pneumoniae	Blood culture	V
Klebsiella pneumoniae subsp pneumoniae	Blood culture	V
Enterobacter aerogenes	Blood culture	V
Klebsiella pneumoniae	Blood culture	V
Klebsiella pneumoniae	Blood culture	V
Klebsiella pneumoniae	Blood culture	V
Klebsiella pneumoniae	Blood culture	V
Klebsiella pneumoniae subsp pneumoniae	Blood culture	V
Klebsiella pneumoniae subsp pneumoniae	Blood culture	V
Klebsiella pneumoniae subsp pneumoniae	Blood culture	V
Klebsiella pneumoniae	Blood culture	V
Proteus mirabilis	Blood culture	V
Klebsiella pneumoniae	Blood culture	V
Klebsiella pneumoniae subsp pneumoniae	Blood culture	V
Klebsiella pneumoniae subsp pneumoniae	Blood culture	V
Klebsiella pneumoniae subsp pneumoniae	Blood culture	V
Klebsiella pneumoniae subsp pneumoniae	Blood culture	V
Enterobacter cloacae	Blood culture	V
Klebsiella pneumoniae	Blood culture	V
Klebsiella pneumoniae	Blood culture	V
Klebsiella pneumoniae	Blood culture	V
Klebsiella pneumoniae	Blood culture	V
Klebsiella pneumoniae	Blood culture	V
Klebsiella pneumoniae	Blood culture	V
Escherichia coli	Blood culture	V
Klebsiella pneumoniae subsp pneumoniae	Blood culture	V
Enterobacter cloacae	Blood culture	V
Klebsiella pneumoniae	Blood culture	V
Klebsiella pneumoniae subsp pneumoniae	Blood culture	V
Morganella morganii	Blood culture	V
Klebsiella pneumoniae	Blood culture	V
Klebsiella pneumoniae subsp pneumoniae	Blood culture	V
Klebsiella pneumoniae subsp pneumoniae	Blood culture	V
Klebsiella pneumoniae subsp pneumoniae	Blood culture	V
Klebsiella pneumoniae subsp pneumoniae	Blood culture	V
Klebsiella pneumoniae subsp pneumoniae	Blood culture	V
Klebsiella pneumoniae	Blood culture	V
Klebsiella pneumoniae subsp pneumoniae	Blood culture	V
Escherichia coli	Blood culture	N
Klebsiella pneumoniae	Blood culture	V
Klebsiella pneumoniae subsp pneumoniae	Blood culture	V
Klebsiella pneumoniae	Blood culture	A
Proteus mirabilis	Blood culture	A
Klebsiella pneumoniae	Blood culture	A
Aeromonas hydrophila	Blood culture	A
Klebsiella pneumoniae subsp pneumoniae	Blood culture	A
Klebsiella pneumoniae subsp pneumoniae	Blood culture	A
Klebsiella pneumoniae subsp pneumoniae	Blood culture	A
Klebsiella pneumoniae subsp pneumoniae	Blood culture	A
Klebsiella pneumoniae subsp pneumoniae	Blood culture	A
Klebsiella pneumoniae	Blood culture	A
Klebsiella pneumoniae subsp pneumoniae	Blood culture	A
Escherichia coli	Blood culture	A
Klebsiella pneumoniae	Blood culture	A

Klebsiella pneumoniae subsp pneumoniae	Blood culture	A
Klebsiella pneumoniae subsp pneumoniae	Blood culture	A
Proteus mirabilis	Blood culture	A
Klebsiella pneumoniae	Blood culture	A
Escherichia coli	Blood culture	A
Klebsiella pneumoniae subsp pneumoniae	Blood culture	A
Klebsiella pneumoniae subsp pneumoniae	Blood culture	A
Serratia marcescens	Blood culture	A
Enterobacter cloacae	Blood culture	A
Klebsiella pneumoniae	Blood culture	A
Citrobacter freundii	Blood culture	A
Klebsiella pneumoniae subsp pneumoniae	Blood culture	A
Serratia marcescens	Blood culture	A
Klebsiella pneumoniae	Blood culture	A
Klebsiella pneumoniae subsp pneumoniae	Blood culture	A
Enterobacter cloacae	Blood culture	A
Providencia rettgeri	Blood culture	A
Morganella morganii subsp morganii	Blood culture	A
Klebsiella pneumoniae subsp pneumoniae	Blood culture	A
Proteus mirabilis	Blood culture	A
Escherichia coli	Blood culture	A
Aeromonas hydrophila	Blood culture	A
Klebsiella pneumoniae subsp pneumoniae	Blood culture	A
Klebsiella pneumoniae subsp pneumoniae	Blood culture	A
Klebsiella pneumoniae subsp pneumoniae	Blood culture	A
Enterobacter cloacae complex	Blood culture	A
Klebsiella pneumoniae subsp pneumoniae	Blood culture	A
Klebsiella pneumoniae subsp pneumoniae	Blood culture	A
Klebsiella pneumoniae subsp pneumoniae	Blood culture	A
Aeromonas hydrophila	Blood culture	A
Klebsiella pneumoniae	Blood culture	A
Enterobacter cloacae complex	Blood culture	A
Klebsiella pneumoniae subsp pneumoniae	Blood culture	A
Klebsiella pneumoniae subsp pneumoniae	Blood culture	A
Klebsiella pneumoniae subsp pneumoniae	Blood culture	A
Klebsiella pneumoniae subsp pneumoniae	Blood culture	A
Klebsiella pneumoniae subsp pneumoniae	Blood culture	A
Klebsiella pneumoniae subsp pneumoniae	Blood culture	A
Klebsiella pneumoniae subsp pneumoniae	Blood culture	V
Serratia fonticola	Blood culture	A
Klebsiella pneumoniae subsp pneumoniae	Blood culture	A
Klebsiella pneumoniae subsp pneumoniae	Blood culture	A
Klebsiella pneumoniae subsp pneumoniae	Blood culture	A
Klebsiella pneumoniae subsp pneumoniae	Blood culture	A
Proteus mirabilis	Blood culture	A
Aeromonas hydrophila	Blood culture	A
Enterobacter cloacae complex	Blood culture	A
Citrobacter freundii	Blood culture	A
Klebsiella pneumoniae subsp pneumoniae	Blood culture	A
Proteus mirabilis	Blood culture	A
Klebsiella pneumoniae	Blood culture	A
Escherichia coli	Blood culture	A
Enterobacter cloacae subsp cloacae	Blood culture	A
Klebsiella pneumoniae subsp pneumoniae	Blood culture	A
Enterobacter cloacae subsp cloacae	Blood culture	A
Proteus vulgaris	Blood culture	A

Providencia stuartii	Blood culture	A
Klebsiella pneumoniae subsp pneumoniae	Blood culture	A
Aeromonas hydrophila/caviae	Blood culture	A
Proteus mirabilis	Blood culture	A
Escherichia coli	Blood culture	A
Enterobacter cloacae	Blood culture	A
Serratia marcescens	Blood culture	A
Klebsiella pneumoniae subsp pneumoniae	Blood culture	A
Klebsiella pneumoniae subsp pneumoniae	Blood culture	A
Proteus mirabilis	Blood culture	A
Klebsiella pneumoniae	Blood culture	A
Proteus mirabilis	Blood culture	A
Escherichia coli	Blood culture	A
Klebsiella pneumoniae subsp pneumoniae	Blood culture	A
Klebsiella pneumoniae subsp pneumoniae	Blood culture	A
Klebsiella pneumoniae subsp pneumoniae	Blood culture	A
Klebsiella pneumoniae subsp pneumoniae	Blood culture	A
Serratia marcescens	Blood culture	A
Enterobacter cloacae subsp cloacae	Blood culture	A
Proteus hauseri	Blood culture	A
Klebsiella pneumoniae	Blood culture	A
Aeromonas sobria	Blood culture	A
Enterobacter cloacae	Blood culture	A
Morganella morganii	Blood culture	A
Enterobacter cloacae complex	Blood culture	A
Aeromonas hydrophila	Blood culture	A
Proteus hauseri	Blood culture	A
Klebsiella pneumoniae	Blood culture	A
Klebsiella pneumoniae subsp pneumoniae	Blood culture	A
Klebsiella pneumoniae subsp pneumoniae	Blood culture	A
Escherichia coli	Blood culture	A
Klebsiella pneumoniae subsp pneumoniae	Blood culture	A
Klebsiella pneumoniae subsp pneumoniae	Blood culture	A
Enterobacter cloacae subsp cloacae	Blood culture	A
Klebsiella pneumoniae	Blood culture	A
Klebsiella pneumoniae	Blood culture	A
Enterobacter cloacae complex	Blood culture	A
Klebsiella pneumoniae	Blood culture	A
Enterobacter cloacae complex	Blood culture	A
Klebsiella pneumoniae subsp pneumoniae	Blood culture	A
Citrobacter freundii	Blood culture	A
Klebsiella pneumoniae	Blood culture	A
Proteus vulgaris	Blood culture	A
Klebsiella pneumoniae subsp pneumoniae	Blood culture	A
Serratia plymuthica	Blood culture	A
Enterobacter cloacae complex	Blood culture	A
Klebsiella pneumoniae subsp pneumoniae	Blood culture	A
Klebsiella pneumoniae subsp pneumoniae	Blood culture	A
Klebsiella pneumoniae subsp pneumoniae	Blood culture	A
Klebsiella pneumoniae subsp pneumoniae	Blood culture	A
Klebsiella pneumoniae subsp pneumoniae	Blood culture	A
Klebsiella pneumoniae	Blood culture	A
Klebsiella pneumoniae	Blood culture	A
Klebsiella pneumoniae subsp pneumoniae	Blood culture	A
Enterobacter cloacae complex	Blood culture	A
Klebsiella pneumoniae subsp pneumoniae	Blood culture	A

Enterobacter cloacae subsp cloacae	Blood culture	A
Proteus hauseri	Blood culture	A
Klebsiella pneumoniae	Blood culture	A
Klebsiella pneumoniae subsp pneumoniae	Blood culture	A
Proteus mirabilis	Blood culture	A
Serratia marcescens	Blood culture	A
Klebsiella pneumoniae	Blood culture	A
Escherichia coli	Blood culture	A
Klebsiella pneumoniae subsp pneumoniae	Blood culture	A
Klebsiella pneumoniae	Blood culture	A
Klebsiella pneumoniae	Blood culture	A
Citrobacter freundii	Blood culture	A
Klebsiella pneumoniae	Blood culture	A
Proteus mirabilis	Blood culture	A
Klebsiella pneumoniae	Blood culture	A
Proteus mirabilis	Blood culture	A
Klebsiella pneumoniae	Blood culture	A
Klebsiella pneumoniae subsp pneumoniae	Blood culture	A
Morganella morganii subsp morganii	Blood culture	A
Klebsiella pneumoniae	Blood culture	A
Klebsiella pneumoniae subsp pneumoniae	Blood culture	A
Escherichia coli	Blood culture	A
Klebsiella pneumoniae subsp pneumoniae	Blood culture	A
Enterobacter cloacae subsp cloacae	Blood culture	A
Proteus vulgaris	Blood culture	A
Klebsiella pneumoniae subsp pneumoniae	Blood culture	A
Klebsiella pneumoniae	Blood culture	A
Klebsiella pneumoniae subsp pneumoniae	Blood culture	A
Serratia marcescens	Blood culture	A
Morganella morganii subsp morganii	Blood culture	A
Klebsiella pneumoniae subsp pneumoniae	Blood culture	A
Proteus mirabilis	Blood culture	A
Klebsiella pneumoniae subsp pneumoniae	Blood culture	A
Klebsiella pneumoniae subsp pneumoniae	Blood culture	A
Klebsiella pneumoniae	Blood culture	A
Klebsiella pneumoniae subsp pneumoniae	Blood culture	A
Enterobacter cloacae	Blood culture	A
Klebsiella pneumoniae subsp pneumoniae	Blood culture	A
Enterobacter cloacae subsp cloacae	Blood culture	A
Aeromonas hydrophila/caviae	Blood culture	A
Proteus mirabilis	Blood culture	A
Klebsiella pneumoniae	Blood culture	A
Escherichia coli	Blood culture	A
Klebsiella species	Blood culture	A
Klebsiella pneumoniae	Blood culture	A
Proteus vulgaris	Blood culture	A
Klebsiella pneumoniae	Blood culture	A
Proteus mirabilis	Blood culture	A
Klebsiella pneumoniae subsp pneumoniae	Blood culture	A
Proteus mirabilis	Blood culture	A
Klebsiella pneumoniae subsp pneumoniae	Blood culture	A
Klebsiella pneumoniae subsp pneumoniae	Blood culture	A
Proteus mirabilis	Blood culture	A
Klebsiella pneumoniae	Blood culture	A
Enterobacter cloacae subsp cloacae	Blood culture	A
Proteus vulgaris	Blood culture	A

Morganella morganii subsp morganii	Blood culture	A
Proteus vulgaris	Blood culture	A
Klebsiella pneumoniae	Blood culture	A
Proteus mirabilis	Blood culture	A
Klebsiella pneumoniae subsp pneumoniae	Blood culture	A
Klebsiella pneumoniae subsp pneumoniae	Blood culture	A
Proteus mirabilis	Blood culture	A
Klebsiella pneumoniae subsp pneumoniae	Blood culture	A
Proteus mirabilis	Blood culture	A
Klebsiella pneumoniae subsp pneumoniae	Blood culture	A
Klebsiella pneumoniae	Blood culture	A
Klebsiella pneumoniae subsp pneumoniae	Blood culture	A
Serratia marcescens	Blood culture	A
Proteus mirabilis	Blood culture	A
Klebsiella pneumoniae	Blood culture	A
Klebsiella pneumoniae subsp pneumoniae	Blood culture	A
Klebsiella pneumoniae subsp pneumoniae	Blood culture	A
Klebsiella pneumoniae	Blood culture	A
Klebsiella pneumoniae subsp pneumoniae	Blood culture	A
Klebsiella pneumoniae subsp pneumoniae	Blood culture	A
Klebsiella pneumoniae subsp pneumoniae	Blood culture	A
Morganella morganii subsp morganii	Blood culture	A
Klebsiella pneumoniae subsp pneumoniae	Blood culture	A
Klebsiella pneumoniae	Blood culture	A
Klebsiella pneumoniae subsp pneumoniae	Blood culture	A
Klebsiella pneumoniae subsp pneumoniae	Blood culture	A
Enterobacter cloacae subsp cloacae	Blood culture	A
Klebsiella pneumoniae subsp pneumoniae	Blood culture	A
Enterobacter cloacae subsp cloacae	Blood culture	A
Klebsiella pneumoniae subsp pneumoniae	Blood culture	A
Klebsiella pneumoniae subsp pneumoniae	Blood culture	A
Klebsiella pneumoniae subsp pneumoniae	Blood culture	A
Klebsiella pneumoniae	Blood culture	A
Klebsiella pneumoniae subsp pneumoniae	Blood culture	A
Escherichia coli	Blood culture	A
Klebsiella pneumoniae	Blood culture	A
Proteus vulgaris	Blood culture	A
Serratia marcescens	Blood culture	A
Klebsiella pneumoniae subsp pneumoniae	Blood culture	A
Enterobacter cloacae	Blood culture	A
Escherichia coli	Blood culture	A
Klebsiella oxytoca	Blood culture	A
Klebsiella pneumoniae subsp pneumoniae	Blood culture	A
Enterobacter cloacae subsp cloacae	Blood culture	A
Klebsiella pneumoniae subsp pneumoniae	Blood culture	A
Enterobacter cloacae subsp cloacae	Blood culture	A
Proteus hauseri	Blood culture	A
Klebsiella pneumoniae subsp pneumoniae	Blood culture	A
Klebsiella pneumoniae subsp pneumoniae	Blood culture	A
Proteus mirabilis	Blood culture	A
Enterobacter cloacae subsp cloacae	Blood culture	A
Proteus penneri	Blood culture	A
Enterobacter cloacae subsp cloacae	Blood culture	A
Klebsiella pneumoniae subsp pneumoniae	Blood culture	A
Proteus penneri	Blood culture	A
Proteus mirabilis	Blood culture	A

Proteus penneri	Blood culture	A
Klebsiella pneumoniae	Blood culture	A
Morganella morganii subsp morganii	Blood culture	A
Klebsiella pneumoniae	Blood culture	A
Proteus vulgaris	Blood culture	A
Enterobacter aerogenes	Blood culture	A
Enterobacter cloacae complex	Blood culture	A
Klebsiella pneumoniae subsp pneumoniae	Blood culture	A
Enterobacter cloacae subsp cloacae	Blood culture	A
Proteus mirabilis	Blood culture	A
Proteus vulgaris	Blood culture	A
Klebsiella oxytoca	Blood culture	A
Serratia marcescens	Blood culture	A
Klebsiella pneumoniae subsp pneumoniae	Blood culture	A
Enterobacter cloacae subsp cloacae	Blood culture	A
Proteus penneri	Blood culture	A
Proteus mirabilis	Blood culture	A
Klebsiella pneumoniae subsp pneumoniae	Blood culture	A
Klebsiella pneumoniae subsp pneumoniae	Blood culture	A
Enterobacter aerogenes	Blood culture	A
Klebsiella pneumoniae subsp pneumoniae	Blood culture	A
Enterobacter cloacae subsp cloacae	Blood culture	A
Citrobacter freundii	Blood culture	A
Klebsiella oxytoca	Blood culture	A
Klebsiella pneumoniae	Blood culture	A
Proteus vulgaris	Blood culture	A
Enterobacter aerogenes	Blood culture	A
Proteus mirabilis	Blood culture	A
Klebsiella oxytoca	Blood culture	V
Proteus mirabilis	Blood culture	A
Klebsiella pneumoniae subsp pneumoniae	Blood culture	A
Enterobacter cloacae	Blood culture	V
Klebsiella pneumoniae subsp pneumoniae	Blood culture	A
Klebsiella pneumoniae subsp pneumoniae	Blood culture	V
Enterobacter cloacae subsp cloacae	Blood culture	A
Klebsiella pneumoniae	Blood culture	V
Klebsiella pneumoniae subsp pneumoniae	Blood culture	V
Klebsiella pneumoniae	Blood culture	V
Klebsiella pneumoniae	Blood culture	V
Klebsiella pneumoniae	Blood culture	V
Enterobacter asburiae	Blood culture	V
Klebsiella pneumoniae subsp pneumoniae	Blood culture	M
Klebsiella pneumoniae subsp pneumoniae	Blood culture	V
Enterobacter cloacae	Blood culture	V
Klebsiella pneumoniae subsp pneumoniae	Blood culture	V
Klebsiella pneumoniae subsp pneumoniae	Blood culture	V
Klebsiella pneumoniae subsp pneumoniae	Blood culture	V
Klebsiella pneumoniae subsp pneumoniae	Blood culture	V
Klebsiella pneumoniae subsp pneumoniae	Blood culture	V
Klebsiella pneumoniae subsp pneumoniae	Blood culture	V
Klebsiella pneumoniae subsp pneumoniae	Blood culture	V
Klebsiella pneumoniae subsp pneumoniae	Blood culture	V
Serratia marcescens	Blood culture	V
Klebsiella pneumoniae subsp pneumoniae	Blood culture	V
Klebsiella pneumoniae subsp pneumoniae	Blood culture	V
Klebsiella pneumoniae subsp pneumoniae	Blood culture	V

Klebsiella pneumoniae	Blood culture	V
Klebsiella pneumoniae	Blood culture	V
Klebsiella pneumoniae	Blood culture	V
Klebsiella pneumoniae	Blood culture	V
Klebsiella pneumoniae	Blood culture	V
Klebsiella pneumoniae	Blood culture	V
Klebsiella pneumoniae	Blood culture	V
Klebsiella oxytoca	Blood culture	V
Klebsiella pneumoniae	Blood culture	V
Klebsiella pneumoniae	Blood culture	V
Klebsiella pneumoniae	Blood culture	V
Klebsiella pneumoniae	Blood culture	V
Klebsiella oxytoca	Blood culture	V
Klebsiella pneumoniae	Blood culture	V
Klebsiella oxytoca	Blood culture	V
Klebsiella pneumoniae	Blood culture	V
Klebsiella pneumoniae subsp pneumoniae	Blood culture	V
Klebsiella pneumoniae	Blood culture	V
Klebsiella pneumoniae subsp pneumoniae	Blood culture	V
Klebsiella pneumoniae subsp pneumoniae	Blood culture	V
Klebsiella pneumoniae subsp pneumoniae	Blood culture	V
Klebsiella pneumoniae subsp pneumoniae	Blood culture	V
Klebsiella pneumoniae	Blood culture	V
Klebsiella pneumoniae subsp pneumoniae	Blood culture	V
Klebsiella pneumoniae	Blood culture	V
Klebsiella pneumoniae subsp pneumoniae	Blood culture	V
Klebsiella pneumoniae subsp pneumoniae	Blood culture	V
Klebsiella pneumoniae subsp pneumoniae	Blood culture	V
Klebsiella pneumoniae subsp pneumoniae	Blood culture	V
Klebsiella pneumoniae subsp pneumoniae	Blood culture	V
Klebsiella pneumoniae subsp pneumoniae	Blood culture	V
Klebsiella pneumoniae subsp pneumoniae	Blood culture	V
Serratia marcescens	Blood culture	V
Klebsiella pneumoniae	Blood culture	V
Klebsiella pneumoniae	Blood culture	V
Klebsiella pneumoniae	Blood culture	V
Klebsiella pneumoniae	Blood culture	V
Klebsiella pneumoniae	Blood culture	V
Klebsiella pneumoniae	Blood culture	V
Klebsiella pneumoniae	Blood culture	V
Klebsiella pneumoniae subsp pneumoniae	Blood culture	V
Klebsiella pneumoniae	Blood culture	V
Klebsiella pneumoniae	Blood culture	V
Klebsiella pneumoniae	Blood culture	V
Klebsiella pneumoniae	Blood culture	V
Klebsiella pneumoniae	Blood culture	V
Klebsiella oxytoca	Blood culture	V
Enterobacter cloacae	Blood culture	V
Enterobacter cloacae	Blood culture	V
Klebsiella pneumoniae subsp pneumoniae	Blood culture	V
Klebsiella pneumoniae subsp pneumoniae	Blood culture	V
Klebsiella pneumoniae subsp pneumoniae	Blood culture	V
Klebsiella pneumoniae subsp pneumoniae	Blood culture	V
Proteus mirabilis	Blood culture	V
Klebsiella pneumoniae subsp pneumoniae	Blood culture	V
Escherichia coli	Blood culture	V

Klebsiella pneumoniae	Blood culture	V
Klebsiella pneumoniae subsp pneumoniae	Blood culture	V
Proteus mirabilis	Blood culture	V
Klebsiella pneumoniae subsp pneumoniae	Blood culture	V
Klebsiella pneumoniae subsp pneumoniae	Blood culture	V
Klebsiella pneumoniae subsp pneumoniae	Blood culture	V
Klebsiella pneumoniae	Blood culture	V
Klebsiella pneumoniae	Blood culture	V
Klebsiella pneumoniae	Blood culture	V
Klebsiella pneumoniae	Blood culture	V
Klebsiella pneumoniae	Blood culture	V
Klebsiella pneumoniae	Blood culture	V
Klebsiella pneumoniae	Blood culture	V
Klebsiella pneumoniae subsp pneumoniae	Blood culture	V
Serratia marcescens	Blood culture	V
Serratia marcescens	Blood culture	V
Klebsiella pneumoniae	Blood culture	V
Klebsiella pneumoniae	Blood culture	V
Escherichia coli	Blood culture	V
Klebsiella pneumoniae subsp pneumoniae	Blood culture	V
Enterobacter cloacae	Blood culture	V
Klebsiella pneumoniae	Blood culture	V
Klebsiella pneumoniae	Blood culture	V
Klebsiella pneumoniae	Blood culture	V
Klebsiella pneumoniae	Blood culture	V
Klebsiella pneumoniae	Blood culture	V
Enterobacter aerogenes	Blood culture	V
Klebsiella pneumoniae subsp pneumoniae	Blood culture	V
Klebsiella pneumoniae subsp pneumoniae	Blood culture	V
Klebsiella pneumoniae subsp pneumoniae	Blood culture	V
Serratia marcescens	Blood culture	V
Klebsiella pneumoniae subsp pneumoniae	Blood culture	V
Klebsiella pneumoniae subsp pneumoniae	Blood culture	V
Klebsiella pneumoniae subsp pneumoniae	Blood culture	V
Klebsiella pneumoniae subsp pneumoniae	Blood culture	V
Klebsiella pneumoniae subsp pneumoniae	Blood culture	V
Klebsiella pneumoniae subsp pneumoniae	Blood culture	V
Klebsiella pneumoniae	Blood culture	V
Klebsiella pneumoniae subsp pneumoniae	Blood culture	V
Klebsiella pneumoniae subsp pneumoniae	Blood culture	V
Klebsiella pneumoniae subsp pneumoniae	Blood culture	V
Klebsiella pneumoniae subsp pneumoniae	Blood culture	V
Citrobacter freundii	Blood culture	V
Escherichia coli	Blood culture	V
Klebsiella pneumoniae subsp pneumoniae	Blood culture	V
Klebsiella pneumoniae subsp pneumoniae	Blood culture	V
Enterobacter cloacae	Blood culture	V
Klebsiella pneumoniae subsp pneumoniae	Blood culture	V
Klebsiella pneumoniae	Blood culture	V
Klebsiella pneumoniae	Blood culture	V
Klebsiella pneumoniae	Blood culture	V
Klebsiella pneumoniae	Blood culture	V
Klebsiella pneumoniae	Blood culture	V
Klebsiella pneumoniae	Blood culture	V
Enterobacter cloacae	Blood culture	V
Klebsiella pneumoniae	Blood culture	V

Klebsiella pneumoniae	Blood culture	V
Klebsiella pneumoniae	Blood culture	V
Enterobacter cloacae	Blood culture	V
Serratia marcescens	Blood culture	V
Klebsiella pneumoniae	Blood culture	V
Klebsiella pneumoniae	Blood culture	V
Klebsiella pneumoniae	Blood culture	V
Klebsiella pneumoniae	Blood culture	V
Klebsiella pneumoniae	Blood culture	V
Klebsiella pneumoniae subsp pneumoniae	Blood culture	V
Klebsiella pneumoniae	Blood culture	V
Klebsiella pneumoniae	Blood culture	V
Klebsiella pneumoniae subsp pneumoniae	Blood culture	V
Enterobacter cloacae	Blood culture	V
Escherichia coli	Blood culture	V
Klebsiella pneumoniae subsp pneumoniae	Blood culture	V
Klebsiella pneumoniae subsp pneumoniae	Blood culture	V
Citrobacter freundii	Blood culture	V
Klebsiella pneumoniae subsp pneumoniae	Blood culture	V
Klebsiella pneumoniae subsp pneumoniae	Blood culture	V
Escherichia coli	Blood culture	V
Klebsiella pneumoniae	Blood culture	V
Klebsiella pneumoniae	Blood culture	V
Klebsiella pneumoniae	Blood culture	V
Klebsiella pneumoniae	Blood culture	V
Klebsiella pneumoniae	Blood culture	V
Klebsiella pneumoniae	Blood culture	V
Klebsiella pneumoniae	Blood culture	V
Morganella morganii	Blood culture	A
Proteus mirabilis	Blood culture	A
Klebsiella pneumoniae subsp pneumoniae	Blood culture	A
Klebsiella pneumoniae	Blood culture	A
Klebsiella pneumoniae	Blood culture	A
Klebsiella pneumoniae	Blood culture	A
Klebsiella pneumoniae	Blood culture	A
Enterobacter cloacae complex	Blood culture	A
Klebsiella pneumoniae	Blood culture	A
Klebsiella pneumoniae subsp pneumoniae	Blood culture	A
Proteus mirabilis	Blood culture	A
Enterobacter aerogenes	Blood culture	A
Enterobacter cloacae	Blood culture	A
Proteus mirabilis	Blood culture	A
Klebsiella pneumoniae subsp pneumoniae	Blood culture	A
Enterobacter cloacae complex	Blood culture	A
Klebsiella pneumoniae	Blood culture	A
Klebsiella pneumoniae subsp pneumoniae	Blood culture	A
Escherichia coli	Blood culture	A
Klebsiella pneumoniae subsp pneumoniae	Blood culture	A
Klebsiella pneumoniae	Blood culture	A
Klebsiella pneumoniae	Blood culture	A
Klebsiella pneumoniae	Blood culture	A
Enterobacter cloacae	Blood culture	A
Klebsiella pneumoniae subsp pneumoniae	Blood culture	A
Klebsiella pneumoniae	Blood culture	A
Klebsiella pneumoniae	Blood culture	A
Klebsiella pneumoniae subsp pneumoniae	Blood culture	A

Klebsiella pneumoniae	Blood culture	A
Proteus mirabilis	Blood culture	A
Serratia marcescens	Blood culture	A
Klebsiella pneumoniae subsp pneumoniae	Blood culture	A
Klebsiella pneumoniae subsp pneumoniae	Blood culture	A
Klebsiella pneumoniae	Blood culture	A
Klebsiella pneumoniae subsp pneumoniae	Blood culture	A
Klebsiella pneumoniae	Blood culture	A
Klebsiella pneumoniae subsp pneumoniae	Blood culture	A
Klebsiella pneumoniae subsp pneumoniae	Blood culture	A
Klebsiella pneumoniae	Blood culture	A
Klebsiella pneumoniae subsp pneumoniae	Blood culture	A
Proteus mirabilis	Blood culture	A
Klebsiella pneumoniae	Blood culture	A
Enterobacter cloacae	Blood culture	A
Escherichia coli	Blood culture	A
Klebsiella pneumoniae subsp pneumoniae	Blood culture	A
Klebsiella pneumoniae subsp pneumoniae	Blood culture	A
Klebsiella pneumoniae subsp pneumoniae	Blood culture	A
Serratia marcescens	Blood culture	A
Klebsiella pneumoniae subsp pneumoniae	Blood culture	A
Serratia marcescens	Blood culture	A
Klebsiella pneumoniae subsp pneumoniae	Blood culture	A
Klebsiella pneumoniae subsp pneumoniae	Blood culture	A
Klebsiella pneumoniae subsp pneumoniae	Blood culture	A
Klebsiella pneumoniae subsp pneumoniae	Blood culture	A
Klebsiella pneumoniae subsp pneumoniae	Blood culture	A
Klebsiella pneumoniae subsp pneumoniae	Blood culture	A
Klebsiella pneumoniae subsp pneumoniae	Blood culture	A
Escherichia coli	Blood culture	A
Klebsiella pneumoniae subsp pneumoniae	Blood culture	A
Klebsiella pneumoniae	Blood culture	A
Klebsiella pneumoniae	Blood culture	A
Proteus mirabilis	Blood culture	A
Klebsiella pneumoniae subsp pneumoniae	Blood culture	A
Klebsiella pneumoniae subsp pneumoniae	Blood culture	A
Klebsiella pneumoniae subsp pneumoniae	Blood culture	A
Klebsiella pneumoniae	Blood culture	A
Klebsiella pneumoniae subsp pneumoniae	Blood culture	A
Klebsiella pneumoniae	Blood culture	A
Klebsiella pneumoniae	Blood culture	A
Klebsiella pneumoniae subsp pneumoniae	Blood culture	A
Klebsiella pneumoniae subsp pneumoniae	Blood culture	A
Klebsiella pneumoniae	Blood culture	A
Klebsiella pneumoniae	Blood culture	A
Klebsiella pneumoniae	Blood culture	A
Enterobacter cloacae	Blood culture	A
Klebsiella pneumoniae subsp pneumoniae	Blood culture	A
Klebsiella pneumoniae subsp pneumoniae	Blood culture	A
Enterobacter cloacae subsp cloacae	Blood culture	A
Escherichia coli	Blood culture	A
Klebsiella pneumoniae	Blood culture	A
Klebsiella pneumoniae subsp pneumoniae	Blood culture	A
Morganella morganii subsp morganii	Blood culture	A
Klebsiella pneumoniae	Blood culture	A
Klebsiella pneumoniae	Blood culture	A

Klebsiella pneumoniae subsp pneumoniae	Blood culture	A
Klebsiella pneumoniae subsp pneumoniae	Blood culture	A
Klebsiella pneumoniae	Blood culture	A
Klebsiella pneumoniae subsp pneumoniae	Blood culture	A
Klebsiella pneumoniae subsp pneumoniae	Blood culture	A
Klebsiella pneumoniae	Blood culture	A
Escherichia coli	Blood culture	A
Klebsiella pneumoniae	Blood culture	A
Klebsiella pneumoniae	Blood culture	A
Cronobacter sakazakii group	Blood culture	A
Klebsiella pneumoniae	Blood culture	A
Klebsiella pneumoniae	Blood culture	A
Klebsiella oxytoca	Blood culture	A
Klebsiella pneumoniae	Blood culture	A
Klebsiella pneumoniae	Blood culture	A
Klebsiella pneumoniae subsp pneumoniae	Blood culture	A
Klebsiella pneumoniae subsp pneumoniae	Blood culture	A
Klebsiella pneumoniae subsp pneumoniae	Blood culture	A
Klebsiella pneumoniae subsp pneumoniae	Blood culture	A
Enterobacter aerogenes	Blood culture	A
Serratia marcescens	Blood culture	A
Klebsiella pneumoniae subsp pneumoniae	Blood culture	A
Klebsiella pneumoniae subsp pneumoniae	Blood culture	A
Klebsiella pneumoniae subsp pneumoniae	Blood culture	A
Klebsiella pneumoniae	Blood culture	A
Klebsiella pneumoniae	Blood culture	A
Serratia marcescens	Blood culture	A
Klebsiella pneumoniae subsp pneumoniae	Blood culture	A
Klebsiella pneumoniae subsp pneumoniae	Blood culture	A
Klebsiella pneumoniae	Blood culture	A
Klebsiella pneumoniae	Blood culture	A
Enterobacter aerogenes	Blood culture	A
Proteus mirabilis	Blood culture	A
Pantoea species	Blood culture	A
Pantoea species	Blood culture	A
Klebsiella pneumoniae	Blood culture	A
Klebsiella pneumoniae	Blood culture	A
Klebsiella pneumoniae subsp pneumoniae	Blood culture	A
Klebsiella pneumoniae subsp pneumoniae	Blood culture	A
Serratia marcescens	Blood culture	A
Enterobacter cloacae subsp cloacae	Blood culture	A
Klebsiella pneumoniae	Blood culture	A
Enterobacter aerogenes	Blood culture	A
Proteus mirabilis	Blood culture	A
Enterobacter cloacae subsp dissolvens	Blood culture	A
Klebsiella pneumoniae subsp pneumoniae	Blood culture	A
Proteus mirabilis	Blood culture	A
Klebsiella pneumoniae subsp pneumoniae	Blood culture	A
Proteus mirabilis	Blood culture	A
Klebsiella pneumoniae subsp pneumoniae	Blood culture	A
Morganella morganii	Blood culture	A
Enterobacter cloacae complex	Blood culture	A
Enterobacter cloacae complex	Blood culture	A
Providencia stuartii	Blood culture	A
Klebsiella pneumoniae	Blood culture	A
Providencia species	Blood culture	A

Enterobacter cloacae complex	Blood culture	A
Proteus mirabilis	Blood culture	A
Klebsiella pneumoniae	Blood culture	A
Klebsiella pneumoniae	Blood culture	A
Klebsiella pneumoniae subsp pneumoniae	Blood culture	A
Klebsiella pneumoniae	Blood culture	A
Escherichia coli	Blood culture	A
Proteus mirabilis	Blood culture	A
Citrobacter koseri	Blood culture	A
Morganella morganii subsp morganii	Blood culture	A
Proteus mirabilis	Blood culture	A
Klebsiella pneumoniae subsp pneumoniae	Blood culture	A
Proteus mirabilis	Blood culture	A
Klebsiella pneumoniae subsp pneumoniae	Blood culture	A
Klebsiella pneumoniae	Blood culture	A
Klebsiella pneumoniae subsp pneumoniae	Blood culture	A
Enterobacter cloacae	Blood culture	A
Proteus mirabilis	Blood culture	A
Klebsiella pneumoniae	Blood culture	A
Proteus mirabilis	Blood culture	A
Enterobacter cloacae	Blood culture	A
Klebsiella pneumoniae	Blood culture	A
Proteus mirabilis	Blood culture	A
Klebsiella pneumoniae subsp pneumoniae	Blood culture	A
Proteus mirabilis	Blood culture	A
Klebsiella pneumoniae subsp pneumoniae	Blood culture	A
Klebsiella pneumoniae	Blood culture	A
Proteus mirabilis	Blood culture	A
Klebsiella pneumoniae subsp pneumoniae	Blood culture	A
Proteus mirabilis	Blood culture	A
Enterobacter species	Blood culture	A
Enterobacter cloacae	Blood culture	A
Enterobacter aerogenes	Blood culture	A
Klebsiella pneumoniae subsp pneumoniae	Blood culture	A
Klebsiella pneumoniae subsp pneumoniae	Blood culture	A
Klebsiella pneumoniae	Blood culture	A
Klebsiella pneumoniae subsp pneumoniae	Blood culture	A
Klebsiella pneumoniae	Blood culture	A
Klebsiella pneumoniae subsp pneumoniae	Blood culture	A
Klebsiella pneumoniae subsp pneumoniae	Blood culture	A
Serratia marcescens	Blood culture	A
Proteus mirabilis	Blood culture	A
Klebsiella pneumoniae	Blood culture	A
Klebsiella pneumoniae	Blood culture	A
Enterobacter cloacae subsp cloacae	Blood culture	A
Enterobacter cloacae complex	Blood culture	A
Enterobacter cloacae complex	Blood culture	A
Providencia stuartii	Blood culture	A
Enterobacter cloacae complex	Blood culture	A
Enterobacter cloacae complex	Blood culture	A
Klebsiella pneumoniae	Blood culture	A
Proteus mirabilis	Blood culture	A
Klebsiella pneumoniae	Blood culture	A
Proteus vulgaris	Blood culture	A
Proteus mirabilis	Blood culture	A
Klebsiella pneumoniae subsp pneumoniae	Blood culture	A

Klebsiella pneumoniae subsp pneumoniae	Blood culture	A
Enterobacter aerogenes	Blood culture	A
Morganella morganii	Blood culture	A
Klebsiella pneumoniae	Blood culture	A
Klebsiella pneumoniae subsp pneumoniae	Blood culture	A
Enterobacter aerogenes	Blood culture	A
Klebsiella pneumoniae subsp pneumoniae	Blood culture	A
Klebsiella oxytoca	Blood culture	A
Klebsiella pneumoniae	Blood culture	A
Proteus mirabilis	Blood culture	A
Klebsiella pneumoniae subsp pneumoniae	Blood culture	A
Klebsiella pneumoniae subsp pneumoniae	Blood culture	A
Klebsiella pneumoniae	Blood culture	A
Klebsiella oxytoca	Blood culture	A
Klebsiella pneumoniae subsp pneumoniae	Blood culture	A
Proteus mirabilis	Blood culture	A
Klebsiella pneumoniae	Blood culture	A
Klebsiella pneumoniae subsp pneumoniae	Blood culture	A
Klebsiella pneumoniae	Blood culture	A
Klebsiella pneumoniae	Blood culture	A
Proteus mirabilis	Blood culture	A
Klebsiella pneumoniae subsp pneumoniae	Blood culture	A
Proteus mirabilis	Blood culture	A
Morganella morganii subsp morganii	Blood culture	A
Klebsiella pneumoniae	Blood culture	A
Proteus mirabilis	Blood culture	A
Proteus vulgaris	Blood culture	A
Proteus mirabilis	Blood culture	A
Klebsiella pneumoniae	Blood culture	A
Klebsiella pneumoniae	Blood culture	A
Klebsiella pneumoniae	Blood culture	A
Proteus mirabilis	Blood culture	A
Klebsiella pneumoniae subsp pneumoniae	Blood culture	A
Klebsiella pneumoniae	Blood culture	A
Klebsiella pneumoniae	Blood culture	A
Proteus hauseri	Blood culture	A
Enterobacter cloacae complex	Blood culture	A
Enterobacter aerogenes	Blood culture	A
Klebsiella pneumoniae subsp pneumoniae	Blood culture	A
Serratia marcescens	Blood culture	A
Citrobacter freundii	Blood culture	A
Klebsiella pneumoniae subsp pneumoniae	Blood culture	A
Enterobacter cloacae subsp cloacae	Blood culture	A
Klebsiella pneumoniae subsp pneumoniae	Blood culture	A
Serratia marcescens	Blood culture	A
Klebsiella pneumoniae subsp pneumoniae	Blood culture	A
Proteus mirabilis	Blood culture	A
Morganella morganii	Blood culture	A
Klebsiella pneumoniae	Blood culture	A
Klebsiella pneumoniae subsp pneumoniae	Blood culture	A
Enterobacter aerogenes	Blood culture	A
Proteus mirabilis	Blood culture	A
Enterobacter cloacae	Blood culture	A
Klebsiella pneumoniae subsp pneumoniae	Blood culture	A
Klebsiella pneumoniae subsp pneumoniae	Blood culture	A
Klebsiella pneumoniae	Blood culture	A

Klebsiella pneumoniae subsp pneumoniae	Blood culture	A
Enterobacter cloacae	Blood culture	A
Klebsiella pneumoniae	Blood culture	A
Proteus mirabilis	Blood culture	A
Klebsiella pneumoniae subsp pneumoniae	Blood culture	A
Klebsiella pneumoniae subsp pneumoniae	Blood culture	A
Proteus mirabilis	Blood culture	A
Klebsiella pneumoniae subsp pneumoniae	Blood culture	A
Klebsiella pneumoniae subsp pneumoniae	Blood culture	A
Proteus mirabilis	Blood culture	A
Klebsiella pneumoniae subsp pneumoniae	Blood culture	A
Proteus mirabilis	Blood culture	A
Escherichia coli	Blood culture	A
Klebsiella pneumoniae	Blood culture	A
Proteus mirabilis	Blood culture	A
Klebsiella pneumoniae subsp pneumoniae	Blood culture	A
Klebsiella pneumoniae subsp pneumoniae	Blood culture	A
Klebsiella pneumoniae subsp pneumoniae	Blood culture	A
Proteus mirabilis	Blood culture	A
Proteus vulgaris	Blood culture	A
Enterobacter cloacae	Blood culture	A
Klebsiella pneumoniae subsp pneumoniae	Blood culture	A
Klebsiella pneumoniae subsp pneumoniae	Blood culture	A
Klebsiella pneumoniae subsp pneumoniae	Blood culture	A
Proteus mirabilis	Blood culture	A
Morganella morganii subsp morganii	Blood culture	A
Enterobacter cloacae subsp dissolvens	Blood culture	A
Klebsiella pneumoniae subsp pneumoniae	Blood culture	A
Klebsiella pneumoniae subsp pneumoniae	Blood culture	A
Proteus mirabilis	Blood culture	A
Klebsiella pneumoniae subsp pneumoniae	Blood culture	A
Klebsiella pneumoniae subsp pneumoniae	Blood culture	A
Enterobacter cloacae complex	Blood culture	A
Klebsiella pneumoniae subsp pneumoniae	Blood culture	A
Morganella morganii	Blood culture	A
Enterobacter cloacae complex	Blood culture	A
Enterobacter aerogenes	Blood culture	A
Enterobacter cloacae complex	Blood culture	A
Klebsiella pneumoniae	Blood culture	A
Aeromonas hydrophila/caviae	Blood culture	A
Proteus mirabilis	Blood culture	A
Klebsiella pneumoniae subsp pneumoniae	Blood culture	A
Enterobacter cloacae complex	Blood culture	A
Klebsiella pneumoniae subsp pneumoniae	Blood culture	A
Enterobacter aerogenes	Blood culture	A
Enterobacter cloacae	Blood culture	A
Morganella morganii subsp morganii	Blood culture	A
Klebsiella pneumoniae subsp pneumoniae	Blood culture	A
Klebsiella pneumoniae subsp pneumoniae	Blood culture	A
Proteus mirabilis	Blood culture	A
Proteus mirabilis	Blood culture	A
Enterobacter cloacae complex	Blood culture	A
Proteus mirabilis	Blood culture	A
Klebsiella pneumoniae subsp pneumoniae	Blood culture	A
Klebsiella pneumoniae subsp pneumoniae	Blood culture	A
Proteus mirabilis	Blood culture	A

Aeromonas hydrophila/caviae	Blood culture	A
Klebsiella pneumoniae subsp pneumoniae	Blood culture	A
Enterobacter cloacae complex	Blood culture	A
Proteus mirabilis	Blood culture	A
Enterobacter aerogenes	Blood culture	A
Klebsiella pneumoniae	Blood culture	A
Proteus mirabilis	Blood culture	A
Enterobacter cloacae	Blood culture	A
Klebsiella pneumoniae subsp pneumoniae	Blood culture	A
Proteus mirabilis	Blood culture	A
Klebsiella pneumoniae	Blood culture	A
Klebsiella pneumoniae subsp pneumoniae	Blood culture	A
Enterobacter cloacae complex	Blood culture	A
Klebsiella pneumoniae subsp pneumoniae	Blood culture	A
Enterobacter cloacae complex	Blood culture	A
Klebsiella pneumoniae subsp pneumoniae	Blood culture	A
Klebsiella pneumoniae subsp pneumoniae	Blood culture	A
Klebsiella pneumoniae subsp pneumoniae	Blood culture	A
Klebsiella aerogenes	Blood culture	A
Proteus penneri	Blood culture	A
Klebsiella pneumoniae	Blood culture	A
Klebsiella pneumoniae	Blood culture	A
Klebsiella pneumoniae	Blood culture	A
Klebsiella pneumoniae subsp pneumoniae	Blood culture	A
Serratia fonticola	Blood culture	A
Escherichia coli	Blood culture	A
Klebsiella pneumoniae subsp pneumoniae	Blood culture	A
Enterobacter cloacae subsp dissolvens	Blood culture	A
Providencia stuartii	Blood culture	A
Klebsiella pneumoniae subsp pneumoniae	Blood culture	A
Klebsiella pneumoniae subsp pneumoniae	Blood culture	A
Klebsiella pneumoniae	Blood culture	A
Klebsiella pneumoniae	Blood culture	A
Proteus mirabilis	Blood culture	A
Enterobacter aerogenes	Blood culture	A
Raoultella ornithinolytica	Blood culture	A
Enterobacter cloacae subsp cloacae	Blood culture	A
Morganella morganii subsp morganii	Blood culture	A
Klebsiella pneumoniae	Blood culture	A
Proteus mirabilis	Blood culture	A
Proteus penneri	Blood culture	A
Providencia rettgeri	Blood culture	A
Klebsiella pneumoniae	Blood culture	A
Proteus mirabilis	Blood culture	A
Enterobacter cloacae	Blood culture	A
Enterobacter cloacae complex	Blood culture	A
Klebsiella pneumoniae	Blood culture	A
Proteus vulgaris	Blood culture	A
Klebsiella pneumoniae	Blood culture	A
Proteus vulgaris	Blood culture	A
Klebsiella pneumoniae subsp pneumoniae	Blood culture	A
Proteus mirabilis	Blood culture	A
Proteus penneri	Blood culture	A
Klebsiella pneumoniae	Blood culture	A
Morganella morganii subsp morganii	Blood culture	A
Proteus mirabilis	Blood culture	A

Citrobacter freundii	Blood culture	A
Klebsiella oxytoca	Blood culture	A
Proteus mirabilis	Blood culture	A
Enterobacter cloacae complex	Blood culture	A
Proteus mirabilis	Blood culture	A
Klebsiella pneumoniae	Blood culture	A
Klebsiella pneumoniae	Blood culture	A
Morganella morganii	Blood culture	A
Morganella morganii	Blood culture	A
Morganella morganii	Blood culture	A
Klebsiella oxytoca	Blood culture	A
Klebsiella pneumoniae	Blood culture	A
Klebsiella pneumoniae	Blood culture	A
Klebsiella pneumoniae	Blood culture	A
Klebsiella pneumoniae	Blood culture	A
Klebsiella pneumoniae	Blood culture	A
Escherichia coli	Blood culture	A
Klebsiella pneumoniae	Blood culture	A
Proteus mirabilis	Blood culture	A
Klebsiella pneumoniae	Blood culture	A
Klebsiella pneumoniae	Blood culture	A
Klebsiella pneumoniae	Blood culture	A
Klebsiella pneumoniae	Blood culture	A
Enterobacter cloacae	Blood culture	A
Klebsiella pneumoniae	Blood culture	A
Morganella morganii subsp morganii	Blood culture	A
Enterobacter cloacae complex	Blood culture	A
Proteus mirabilis	Blood culture	A
Proteus mirabilis	Blood culture	A
Enterobacter cloacae complex	Blood culture	A
Klebsiella pneumoniae	Blood culture	A
Enterobacter cloacae complex	Blood culture	A
Enterobacter aerogenes	Blood culture	A
Proteus hauseri	Blood culture	A
Proteus mirabilis	Blood culture	A
Enterobacter cloacae	Blood culture	A
Enterobacter cloacae	Blood culture	A
Enterobacter asburiae	Blood culture	A
Enterobacter cloacae complex	Blood culture	A
Enterobacter cloacae	Blood culture	A
Morganella morganii subsp morganii	Blood culture	A
Proteus mirabilis	Blood culture	A
Klebsiella pneumoniae subsp pneumoniae	Blood culture	A
Klebsiella pneumoniae	Blood culture	A
Klebsiella pneumoniae subsp pneumoniae	Blood culture	A
Klebsiella pneumoniae subsp ozaenae	Blood culture	A
Proteus mirabilis	Blood culture	A
Klebsiella pneumoniae subsp pneumoniae	Blood culture	A
Klebsiella pneumoniae	Blood culture	A
Enterobacter cloacae subsp cloacae	Blood culture	A
Enterobacter cloacae complex	Blood culture	A

medical_device	iv_line	cvc	urinary_catheter	drainage_port	intraarterial_line
Y		Y	Y		
Y	Y				
Y		Y	Y	Y	
Y	Y				
U					
Y	Y				
Y	Y		Y		
Y	Y				
Y	Y		Y		
Y	Y		Y		
Y	Y	Y			
Y	Y		Y		
Y	Y		Y		
Y	Y		Y		
Y	Y				
Y	Y	Y	Y		
Y	Y				
Y	Y	Y	Y		
Y	Y			Y	
Y	Y				
Y			Y		
Y	Y		Y	Y	
Y	Y				
Y	Y				
Y		Y	Y		
Y	Y				
Y	Y				
Y	Y				
Y	Y	Y			
Y	Y	Y	Y		
Y	Y	Y	Y		
N					
Y	Y		Y		
Y				Y	
Y	Y		Y		
U					
Y	Y				
Y	Y				
Y	Y				
N					
Y	Y				
Y	Y		Y		

Y		Y	Y		
Y	Y		Y	Y	
N					
N					
Y		Y	Y		
U					
Y	Y		Y		
Y	Y				
Y	Y				
Y	Y	Y			
Y	Y		Y		
Y	Y		Y		
Y	Y	Y			
N					
Y	Y		Y		
Y		Y	Y		
N					
Y		Y	Y		
Y		Y			
Y		Y	Y		
Y	Y				
Y		Y	Y		Y
Y	Y	Y	Y		
Y	Y		Y		
Y	Y	Y			
Y	Y		Y		
Y	Y				
Y	Y				
Y		Y			
Y	Y				
Y	Y				
Y	Y				
Y	Y		Y		
Y	Y				
Y		Y		Y	
U					
Y	Y		Y	Y	
Y		Y	Y		
N					
Y	Y				
Y	Y				
Y	Y	Y	Y		
Y	Y	Y	Y		Y
Y	Y				
Y	Y				
Y	Y	Y	Y		
Y	Y				
Y	Y				
Y		Y	Y	Y	
Y	Y	Y			
Y	Y		Y		
Y	Y	Y			

Y	Y		Y		
U					
U					
Y	Y				
Y	Y	Y	Y		
Y	Y			Y	
Y	Y		Y		
Y		Y	Y	Y	
Y	Y		Y		
Y	Y				
Y	Y			Y	
Y	Y		Y		
N					
Y	Y	Y	Y	Y	
Y	Y		Y		
U					
Y	Y		Y		
Y	Y		Y		
Y	Y				
U					
Y	Y	Y	Y		
Y	Y				
Y	Y	Y	Y		
N					
Y	Y		Y	Y	
U					
Y	Y		Y	Y	
N					
Y	Y				
Y	Y				Y
Y	Y		Y		
Y	Y				
Y	Y			Y	
Y		Y	Y		
Y	Y				
Y	Y	Y			
Y	Y		Y		Y
Y	Y				
U					
Y	Y	Y	Y		
Y	Y	Y	Y	Y	
Y	Y				
Y	Y	Y			
Y	Y				
Y	Y				
Y	Y	Y			
Y	Y	Y			
Y	Y		Y	Y	
N					
N					
N					
Y	Y	Y	Y	Y	
Y	Y				
Y	Y		Y		
Y	Y				

Y	Y				
Y	Y	Y			
Y	Y				
Y	Y	Y		Y	
Y	Y	Y	Y		
Y	Y		Y		Y
U					
Y	Y	Y			
Y	Y	Y	Y		
U					
Y	Y				
Y	Y				
Y	Y				
Y	Y				
U					
U					
U					
Y	Y	Y	Y	Y	
Y	Y				
U					
Y	Y				
Y	Y		Y	Y	Y
Y	Y				
Y	Y		Y		
Y	Y	Y	Y		
Y	Y				
Y	Y				
Y	Y	Y	Y		
Y	Y	Y	Y		
Y	Y	Y	Y		
Y	Y	Y	Y	Y	
Y					
Y	Y	Y			
Y	Y	Y	Y	Y	
Y	Y	Y	Y		Y
Y	Y	Y	Y		Y
Y	Y				
Y	Y		Y		
Y	Y				
Y	Y		Y		
Y	Y				
Y	Y				
Y		Y		Y	
U					
Y	Y		Y		
Y	Y	Y	Y		
Y	Y				
Y	Y		Y		
Y	Y				
U					
Y	Y	Y		Y	
Y	Y		Y		
N					
Y					
Y	Y				

Y	Y				
Y	Y		Y		
Y	Y				
N					
Y	Y	Y	Y		
Y	Y		Y		
Y	Y	Y	Y		
U					
Y		Y	Y		
Y	Y		Y		
Y	Y	Y	Y		
Y	Y		Y		Y
Y	Y				
U					
Y	Y		Y		
U					
Y		Y	Y		Y
Y	Y		Y		
Y	Y				
Y	Y				
Y	Y	Y	Y	Y	
Y	Y	Y	Y		
Y	Y				
Y	Y				Y
U					
Y		Y	Y	Y	
Y	Y				
Y	Y		Y		
Y	Y	Y	Y		
Y	Y	Y	Y		
Y		Y	Y		
Y	Y				
Y	Y				
Y	Y	Y	Y	Y	
Y		Y			
U					
Y	Y				
Y	Y				
U					
Y		Y			
Y	Y		Y		
Y	Y		Y	Y	
Y	Y				
Y	Y				
Y	Y		Y		
Y	Y		Y		
Y	Y		Y		
Y	Y				
Y	Y				
Y					
Y	Y	Y			
U					
N					
Y	Y			Y	

Y	Y		Y		
U					
Y	Y				
U					
Y	Y				
Y	Y				
Y	Y				
Y		Y			
Y	Y				
Y		Y		Y	
Y					Y
Y	Y	Y	Y	Y	
Y	Y	Y			
Y	Y			Y	
Y	Y		Y	Y	
U					
U					
U					
Y	Y	Y	Y		Y
U					
U					
U					
Y	Y		Y		
U					
U					
Y	Y	Y			
U					
Y	Y		Y		
Y	Y				
Y	Y	Y	Y		
Y	Y	Y			
Y	Y				
Y	Y		Y		
Y	Y				
Y	Y		Y		
Y	Y				
Y	Y				
Y	Y	Y	Y		
Y	Y	Y	Y	Y	Y
Y	Y				
Y	Y				
Y	Y	Y	Y		
Y	Y		Y	Y	
Y		Y	Y		
Y	Y				
U					
Y	Y				
Y	Y	Y	Y		
Y	Y		Y		
Y	Y	Y	Y		
Y	Y				Y
Y	Y				
N					
Y	Y	Y	Y	Y	Y
N					

Y	Y	Y	Y	
Y	Y	Y	Y	
U				
U				
U				
Y	Y			
Y	Y			
U				
Y	Y			
U				
Y		Y		
Y	Y	Y		
Y		Y	Y	
Y		Y	Y	
N				
Y	Y			
U				
Y	Y			Y
N				
N				
Y	Y		Y	
Y		Y	Y	
U				
N				
Y	Y			
U				
Y	Y	Y	Y	
U				
Y			Y	
Y	Y	Y	Y	
Y	Y	Y		
Y	Y		Y	
U				
U				
Y	Y			
U				
U				
Y	Y			Y
N				
U				
U				
N				
Y	Y	Y		
Y	Y			
Y	Y		Y	
U				
Y		Y		
N				
Y	Y			

Y	Y		Y		
Y	Y		Y		
Y		Y			Y
Y	Y	Y			
Y	Y		Y		
Y	Y				
Y	Y	Y	Y	Y	
Y	Y				
Y	Y	Y	Y	Y	
Y	Y				
Y	Y				
U					
U					
Y	Y	Y	Y		Y
Y	Y	Y	Y		
Y	Y			Y	
Y	Y	Y			
U					
U					
Y	Y				
Y	Y	Y	Y		
U					
U					
U					
Y	Y		Y		
Y	Y		Y		
Y	Y				
Y		Y	Y		
Y	Y	Y	Y		
U					
Y			Y	Y	
Y	Y	Y	Y		
U					
Y	Y				
Y		Y	Y		
U					
U					
Y	Y		Y		
Y	Y	Y	Y		
N					
Y	Y			Y	
U					
Y	Y				
U					
U					
Y	Y				
Y	Y	Y			
U					
Y	Y				
Y	Y				
Y	Y				
Y	Y	Y	Y	Y	
U					
Y	Y				

Y	Y			Y	
Y	Y	Y			
Y		Y		Y	
Y	Y			Y	
N					
Y	Y		Y		Y
Y		Y			
Y	Y	Y	Y		
Y	Y	Y	Y		
Y	Y	Y	Y		
U					
Y	Y		Y		
Y	Y				
Y	Y				Y
Y	Y	Y			
Y	Y				
Y	Y	Y			
Y	Y	Y			
U					
Y	Y	Y	Y		
U					
Y	Y		Y		
Y	Y		Y		
Y	Y				
U					
Y	Y				
Y	Y				
Y	Y		Y		
Y	Y		Y		
Y		Y			
Y	Y				
Y	Y		Y		
Y	Y	Y	Y		
Y	Y	Y			
Y	Y			Y	
Y	Y	Y		Y	
Y	Y		Y		
Y	Y				
Y	Y	Y	Y		
U					
Y	Y				
Y	Y		Y		
U					
Y	Y		Y		
Y	Y	Y	Y		
Y	Y	Y			
Y	Y		Y		
Y	Y	Y			
Y	Y	Y			
Y	Y		Y	Y	
Y	Y	Y	Y		
Y	Y	Y	Y		
Y	Y			Y	
Y	Y				
Y	Y				

Y	Y				
Y	Y	Y	Y		
Y	Y	Y			
U					
U					
Y	Y				
U					
U					
U					
Y	Y				
U					
Y	Y	Y	Y		
U					
Y	Y				
Y	Y		Y	Y	
Y	Y	Y	Y		
Y		Y	Y		
Y	Y				
Y	Y		Y	Y	
N					
Y	Y				
Y	Y			Y	
Y	Y	Y			
Y	Y	Y	Y		Y
Y	Y				
Y	Y		Y		
Y	Y				
Y	Y	Y	Y		
Y		Y	Y		
Y	Y	Y	Y		
Y	Y				
Y	Y				
Y	Y				
Y	Y	Y	Y		
Y	Y				
Y	Y				
Y	Y				
Y		Y			
Y		Y			
Y	Y	Y	Y	Y	
Y	Y	Y	Y	Y	
U					
U					
Y	Y	Y	Y		
U					
U					
Y	Y		Y		
Y		Y	Y		Y
U					
Y	Y	Y			Y
U					
U					
Y	Y	Y	Y	Y	
Y	Y	Y	Y	Y	

Y		Y		Y	
Y	Y		Y		
Y	Y				
Y	Y	Y	Y		
Y	Y			Y	
Y	Y	Y			
Y	Y	Y			
Y	Y				
Y		Y	Y		
Y	Y	Y	Y		
Y	Y	Y	Y		
U					
Y	Y				
Y	Y				
Y	Y				
U					
Y	Y			Y	
Y		Y	Y		
Y	Y	Y	Y	Y	
Y	Y				
Y	Y		Y		
Y	Y	Y	Y		
Y	Y		Y		
Y		Y			
Y	Y	Y			
Y				Y	
Y	Y				
Y	Y				
Y	Y				
Y	Y	Y			
Y		Y			
Y	Y				
Y	Y				
Y	Y	Y	Y		
Y	Y	Y	Y		
Y	Y		Y		
Y		Y			
Y		Y		Y	
Y	Y				
Y	Y	Y	Y	Y	
Y	Y		Y	Y	
U					
Y	Y	Y	Y	Y	
Y	Y		Y		
Y	Y	Y			
Y	Y				
Y	Y				
U					
Y	Y		Y		
Y	Y	Y			
U					
Y	Y	Y	Y	Y	Y
U					
U					

Y	Y				
Y	Y		Y		
U					
U					
U					
Y	Y	Y	Y	Y	Y
Y		Y			Y
Y	Y				
Y	Y	Y			
Y	Y				
Y	Y				
Y	Y				
Y	Y	Y			
Y	Y				
U					
U					
Y	Y				
U					
U					
Y	Y	Y			
Y	Y				
Y	Y	Y			
Y	Y				
Y	Y				
Y	Y		Y	Y	
U					
Y		Y	Y		Y
Y	Y				
Y	Y				
U					
Y	Y	Y		Y	
Y	Y	Y			
Y	Y	Y			
Y	Y				
Y	Y			Y	
Y	Y				
Y		Y			
Y	Y	Y			
U					
Y		Y			
Y		Y		Y	Y
Y	Y	Y		Y	
Y	Y	Y	Y		
Y	Y	Y			
Y	Y				
Y	Y	Y			
Y	Y	Y			
Y	Y	Y		Y	
U					
U					
Y	Y				
U					
U					
U					

U					
Y	Y				
U					
Y		Y	Y		
U					
U					
Y	Y	Y			
Y	Y	Y			
Y		Y		Y	
Y	Y	Y		Y	
Y		Y		Y	
Y	Y				
Y	Y	Y			
Y	Y				
Y	Y				
Y	Y				
Y	Y	Y			
Y	Y				
Y	Y	Y			
Y	Y	Y			
Y	Y				
Y	Y	Y			
Y	Y	Y		Y	
Y	Y	Y		Y	
Y	Y			Y	
Y	Y			Y	
Y	Y		Y		
U					
Y	Y	Y			
Y	Y	Y			
Y	Y				
Y	Y				
Y		Y			
Y	Y				
Y	Y				
Y	Y	Y		Y	
Y		Y		Y	
Y	Y	Y		Y	
Y	Y				Y
Y	Y			Y	
U					
Y	Y	Y			
U					
Y	Y				
Y	Y			Y	
Y	Y			Y	
Y	Y	Y		Y	
Y		Y		Y	
Y		Y			
Y	Y	Y			
Y	Y				
Y	Y			Y	
Y	Y	Y	Y		Y
Y	Y				
Y		Y			Y

Y	Y				
Y	Y	Y			
Y	Y			Y	
Y	Y	Y			
Y	Y	Y		Y	
Y	Y	Y			
Y		Y			Y
Y	Y				
Y		Y		Y	
Y	Y				
Y	Y			Y	
Y	Y	Y		Y	
U					
Y	Y	Y			
Y	Y	Y			
Y	Y	Y			
Y	Y				
Y	Y		Y		
Y	Y	Y			
Y				Y	
Y					
Y	Y	Y			
Y	Y	Y			
Y		Y		Y	
Y	Y			Y	
Y	Y			Y	
Y	Y			Y	
Y	Y	Y			
Y	Y				
Y	Y				Y
Y	Y	Y	Y		
Y	Y				
Y	Y	Y			
Y	Y	Y	Y	Y	Y
Y	Y			Y	
Y	Y				
Y	Y				
Y	Y	Y			
Y	Y			Y	
Y	Y				
Y	Y			Y	
Y	Y			Y	
Y	Y	Y			
Y	Y				
Y	Y				
Y	Y				
Y	Y		Y		
Y	Y				
Y	Y			Y	
Y	Y				
Y	Y			Y	
Y	Y	Y	Y		
Y	Y		Y		
Y	Y	Y		Y	
Y	Y		Y	Y	
Y	Y				

U					
Y	Y		Y		
Y	Y		Y	Y	
Y	Y				
Y	Y		Y		
Y	Y		Y		
Y	Y		Y		
Y	Y		Y		
Y	Y	Y	Y		
Y	Y				
U					
Y	Y	Y	Y		
U					
U					
Y		Y	Y		
N					
U					
Y		Y	Y		
Y	Y				
Y	Y		Y		
U					
Y					
Y	Y				
U					
U					
U					
U					
U					
Y		Y	Y		
Y	Y	Y			
U					
N					
U					
Y	Y				
N					
Y	Y	Y			
Y	Y	Y			
U					
Y		Y			Y
U					
U					
Y	Y				
Y	Y				
U					
Y	Y				
U					
U					

Y		Y	Y		Y
Y	Y				
Y		Y			
Y	Y				
Y	Y				
Y	Y	Y			
Y	Y		Y		
Y	Y				
Y	Y				
Y	Y				Y
Y	Y				
Y	Y	Y			
Y		Y			
Y	Y				
Y	Y	Y	Y	Y	Y
Y	Y		Y		
Y	Y		Y		
Y	Y				
Y	Y				
Y	Y				
U					
Y	Y				
Y	Y				
Y	Y				
Y	Y				
Y	Y	Y		Y	
Y		Y	Y		
Y	Y				
Y	Y				
Y		Y			
Y	Y	Y		Y	Y
U					
U					
Y	Y				
U					
Y		Y	Y		
Y	Y		Y	Y	
Y	Y				
U					
U					
U					
Y	Y			Y	
N					
Y	Y				
U					
Y					Y
Y	Y				

N					
Y		Y	Y		
Y	Y	Y	Y		
U					
U					
Y	Y	Y	Y	Y	Y
Y	Y			Y	
U					
N					
Y	Y	Y	Y		
Y		Y			
U					
Y		Y			
U					
U					
Y	Y	Y			
Y	Y	Y	Y		
U					
Y		Y			
U					
U					
Y	Y				
Y	Y				
Y	Y	Y	Y	Y	
Y	Y		Y		Y
Y	Y	Y			
Y	Y				
Y		Y			
Y	Y		Y	Y	
Y	Y	Y	Y		Y
Y	Y	Y		Y	
N					
Y	Y	Y		Y	
Y	Y				Y
Y	Y	Y	Y		
Y	Y		Y		
Y	Y				
U					
Y		Y			
Y		Y	Y		Y
Y	Y	Y	Y		Y
Y	Y				Y
Y	Y				
Y	Y			Y	
Y	Y	Y	Y		
Y		Y	Y		
Y	Y	Y	Y		
Y	Y				
Y	Y	Y	Y		Y
U					
Y	Y		Y		
U					
Y	Y				
Y	Y				

Y	Y		Y		
U					
U					
Y	Y			Y	
Y	Y				
Y	Y				
Y	Y	Y			
Y	Y		Y		
Y	Y	Y	Y	Y	
U					
U					
Y	Y		Y		
U					
U					
Y	Y				
Y	Y		Y		
Y	Y		Y		
U					
Y	Y				
Y		Y	Y		
U					
Y	Y	Y			
Y	Y				
Y	Y				
Y	Y				
Y	Y	Y			
Y	Y				
Y	Y				
U					
Y				Y	
Y	Y				
U					
Y	Y	Y	Y	Y	
Y	Y	Y	Y		Y
Y	Y				
Y	Y				
N					
Y	Y	Y	Y		
Y		Y	Y		
Y	Y	Y			
Y	Y	Y	Y		
Y		Y	Y		
Y	Y		Y		
Y	Y	Y	Y	Y	
Y	Y		Y	Y	
Y	Y		Y	Y	
Y	Y				
N					
U					
Y	Y				
U					
U					
U					
U					
Y	Y		Y	Y	
Y	Y				

U					
Y	Y	Y			
Y	Y		Y	Y	
Y	Y	Y			
Y					Y
Y	Y	Y	Y		
Y	Y				
Y	Y	Y	Y		
Y	Y				
Y	Y		Y		
Y		Y			
Y	Y				
Y	Y				
Y	Y		Y		
Y	Y				
Y	Y	Y	Y	Y	
Y	Y		Y		
Y	Y	Y	Y		
Y		Y			
Y	Y	Y	Y		
Y	Y		Y		Y
Y			Y		Y
Y	Y	Y		Y	
Y	Y				
Y	Y	Y			
Y	Y				
Y	Y		Y		
Y	Y	Y	Y		
Y	Y	Y			
Y	Y				
Y	Y		Y		
Y			Y		Y
Y	Y				
Y	Y	Y	Y		
Y		Y	Y	Y	
Y	Y				
U					
Y	Y				
Y	Y				
Y	Y				
Y	Y		Y		
Y	Y		Y		
Y	Y				
Y	Y				
Y		Y			
Y	Y	Y	Y		
U					
Y	Y				
Y	Y		Y		Y
Y	Y				
Y		Y	Y	Y	
Y	Y				
Y	Y		Y		
Y	Y				
Y	Y		Y		
Y	Y	Y	Y		

Y	Y		Y		
Y	Y				
Y	Y		Y		
Y	Y				
Y	Y				
Y	Y	Y			
Y	Y	Y	Y		
U					
U					
U					
U					
U					
U					
U					
U					
U					
Y	Y		Y		
U					
Y	Y				
Y	Y	Y	Y		Y
U					
U					
Y	Y		Y		
U					
U					
U					
Y	Y			Y	
Y	Y				
Y	Y	Y	Y		Y
U					
Y	Y	Y	Y	Y	
Y	Y				
Y	Y				
Y	Y		Y		
Y		Y	Y		
N					
Y	Y		Y		
U					
Y	Y		Y		
Y		Y			
Y		Y			
Y	Y		Y		
Y	Y		Y		
Y				Y	
Y		Y	Y		
Y		Y	Y		Y
Y	Y				
Y	Y				
Y	Y		Y		
Y	Y		Y		
Y	Y				
Y	Y	Y			

Y	Y				
Y	Y				
Y	Y		Y		
Y	Y		Y		
Y	Y				
U					
Y	Y		Y		
Y	Y		Y		
Y	Y				
Y	Y	Y	Y		
Y	Y	Y	Y		
Y	Y		Y		Y
Y	Y		Y	Y	
Y	Y				
N					
Y	Y	Y	Y		
Y	Y	Y	Y		Y
Y	Y	Y	Y		
Y	Y				Y
Y	Y		Y		
Y	Y				
Y	Y				
Y	Y	Y	Y		Y
Y	Y				
Y	Y		Y		
Y		Y	Y		Y
Y	Y				
Y	Y		Y		
Y	Y				
U					
Y	Y				
Y	Y				
Y	Y				
Y	Y				
Y		Y	Y	Y	Y
Y	Y		Y	Y	
Y	Y		Y		
Y	Y	Y	Y		Y
U					
Y	Y				
Y	Y				
Y	Y				
U					
U					
Y		Y	Y		
Y	Y				
Y	Y		Y		
Y	Y				
Y	Y	Y	Y		
Y			Y		
Y	Y				
Y	Y				

Y	Y	Y			
U					
U					
Y	Y				
Y	Y		Y		
Y	Y				
U					
Y		Y	Y		Y
Y	Y		Y		
Y	Y	Y			
Y	Y	Y			
U					
Y	Y				
U					
Y		Y	Y	Y	Y
U					
Y	Y	Y	Y	Y	Y
Y	Y				
U					
Y	Y				
Y		Y			
U					
Y	Y		Y	Y	
U					
Y	Y	Y	Y		
U					
U					
Y	Y	Y	Y		Y
Y	Y				
Y	Y		Y		
Y		Y			
Y	Y				
Y		Y	Y		
Y	Y		Y		
N					
Y	Y				
Y	Y		Y	Y	
Y					Y
U					
Y		Y			
U					
U					
Y	Y		Y		
U					
Y	Y				
Y	Y				
Y	Y				
Y	Y				
Y	Y	Y		Y	
Y	Y				

Y	Y				
Y	Y	Y			
Y	Y		Y		
Y		Y	Y	Y	
U					
Y	Y				
Y		Y			
Y	Y				
Y	Y				
Y	Y				
Y	Y				
Y	Y		Y		
Y	Y				
Y		Y	Y	Y	
Y	Y	Y	Y		
Y	Y	Y	Y		Y
Y	Y				
Y	Y				
Y	Y				
Y	Y				
Y	Y				
Y	Y				
Y	Y				
Y	Y	Y	Y		
U					
Y	Y	Y			
Y	Y		Y		Y
Y	Y	Y	Y	Y	
Y	Y	Y	Y		
Y		Y	Y		
Y		Y	Y	Y	
N					
Y	Y				
Y	Y	Y	Y		Y
Y		Y	Y		Y
Y	Y		Y		
U					
Y	Y	Y	Y		
Y	Y				
U					
N					
Y		Y	Y		
Y					Y
Y	Y				
Y	Y				Y
Y	Y				
Y	Y	Y	Y		Y
Y		Y	Y		Y
Y	Y				Y
U					
Y	Y		Y		
Y	Y	Y	Y	Y	Y

Y	Y		Y	Y
Y		Y		
Y	Y			
Y		Y	Y	Y
Y	Y	Y		
Y	Y			
Y		Y		Y
Y	Y			
Y	Y	Y	Y	
Y		Y	Y	
Y			Y	Y
Y	Y			
Y	Y			
Y	Y			
Y	Y			
Y	Y			
Y	Y		Y	
Y	Y			

U

U
U

Y	Y	
Y		Y
Y	Y	
Y		Y
Y	Y	

U

Y	Y				
Y	Y		Y	Y	
U					
Y	Y		Y		
Y		Y			
Y	Y	Y	Y		Y
Y	Y				
U					
Y		Y			
Y	Y				
Y	Y				
U					
Y	Y				
Y	Y				
Y	Y	Y		Y	Y
Y	Y				
Y	Y	Y	Y		
Y		Y	Y		
Y		Y			
Y		Y			
Y		Y			
Y		Y			
U					
U					
Y	Y	Y	Y		
Y		Y	Y		Y
Y		Y	Y		
N					
Y		Y			Y
Y	Y		Y		
Y		Y			
U					
Y	Y				

Y		Y			
Y		Y			
U					
Y	Y				
Y	Y				
Y	Y				
U					
Y	Y	Y			
Y	Y				
Y	Y				Y
Y	Y				
Y	Y				
Y	Y	Y			
Y	Y			Y	
Y	Y	Y			
Y	Y	Y			
Y	Y				
Y	Y				
Y	Y	Y	Y		
U					
Y					
Y	Y		Y		
U					
Y	Y	Y	Y		
Y	Y	Y	Y		
Y	Y	Y			
Y	Y				
U					
Y	Y				
Y	Y				
Y		Y			
Y	Y				
Y	Y				
Y					Y
Y		Y	Y		
Y	Y	Y	Y		
Y	Y				
Y	Y	Y			
Y		Y			
Y	Y	Y	Y		Y
Y	Y				
Y	Y		Y		
Y		Y	Y		
Y	Y				
Y	Y	Y			Y
Y	Y		Y		
Y		Y			

Y	Y	Y			
Y	Y		Y		
Y	Y		Y		
Y		Y			Y
Y		Y	Y		
Y		Y			
Y			Y		Y
Y	Y				
Y	Y				
Y	Y				
Y	Y				
Y	Y				
Y		Y	Y	Y	
Y	Y				
Y	Y				
Y	Y		Y		
Y	Y				
Y			Y		
Y	Y				
Y	Y				
Y	Y				
Y		Y	Y		
Y		Y	Y		Y
U					
Y	Y				
Y		Y			
Y	Y				
Y		Y			Y
Y		Y			
Y	Y				
Y	Y				
U					
Y	Y				
Y					
Y		Y			
U					
Y	Y				
Y		Y			
Y					
Y	Y				
Y		Y			Y
Y		Y			
Y		Y			
Y		Y			
Y	Y				
Y	Y				
Y		Y			
U					
U					
Y		Y			
Y	Y				
U					
Y			Y		

Y	Y				
U					
Y		Y			
Y	Y	Y			Y
Y	Y		Y		Y
Y		Y	Y		
Y	Y		Y		
Y		Y			
Y	Y	Y			
Y		Y	Y		
Y	Y	Y	Y		Y
Y	Y	Y			Y
Y	Y		Y	Y	
U					
Y		Y			
Y	Y				
Y	Y				
Y	Y	Y	Y		Y
Y	Y	Y	Y	Y	Y
Y	Y				
Y	Y				
U					
Y	Y	Y			
Y	Y	Y	Y		Y
Y	Y				
Y	Y	Y			
Y	Y		Y	Y	Y
Y	Y	Y		Y	
N					
Y	Y	Y		Y	
Y	Y	Y	Y		
N					
Y	Y		Y	Y	
Y	Y		Y		
Y	Y			Y	
Y	Y	Y			
Y		Y			
Y		Y			
Y	Y				
Y	Y	Y	Y		
Y	Y	Y	Y		
Y			Y		Y
Y	Y		Y		Y
Y	Y		Y		

Y	Y	Y			
N					
Y	Y	Y	Y		
N					
Y	Y	Y	Y		
Y	Y	Y	Y		
Y	Y	Y			
Y	Y	Y	Y	Y	
Y	Y	Y	Y		
Y		Y			Y
Y	Y				
N					
Y	Y		Y	Y	
U					
Y	Y				
Y	Y	Y			
N					
U					
Y		Y			Y
Y	Y	Y			
N					
Y	Y		Y		
Y	Y				
Y	Y	Y	Y	Y	Y
Y	Y		Y		
Y	Y		Y		
N					
Y	Y	Y		Y	
Y	Y				
Y	Y	Y			
Y	Y				
Y	Y				
Y	Y		Y		
Y	Y				
Y	Y	Y	Y		
Y	Y				
Y		Y			
Y	Y				
Y		Y			
Y		Y		Y	
Y	Y		Y		Y
Y		Y	Y		Y
Y	Y				
Y		Y	Y	Y	Y
N					

Y	Y				
Y	Y	Y	Y		
Y	Y				
Y		Y	Y		
Y	Y		Y		
Y	Y				
Y	Y	Y			
Y	Y				
Y		Y	Y		
Y	Y		Y		
Y	Y	Y	Y	Y	Y
Y	Y				
Y		Y			
Y	Y				
Y			Y		
Y	Y			Y	
Y	Y		Y		
Y	Y		Y	Y	
Y	Y				
Y	Y		Y		
Y	Y	Y	Y	Y	Y
Y	Y				
Y	Y				
Y					Y
Y	Y	Y	Y		
Y	Y	Y	Y		Y
Y	Y			Y	
Y	Y				
Y	Y		Y		Y
Y	Y		Y		
Y		Y	Y		
Y	Y		Y		
Y	Y	Y	Y	Y	
Y	Y		Y		
Y	Y		Y		
Y	Y				
Y	Y				
Y	Y	Y	Y	Y	
U					
Y	Y				
Y	Y	Y			
Y		Y	Y		
Y	Y	Y			
Y	Y	Y	Y		
Y	Y	Y	Y		Y
Y	Y				
Y	Y	Y	Y		Y
Y	Y				
Y		Y	Y	Y	

Y	Y				
Y	Y	Y	Y		Y
Y	Y	Y	Y	Y	Y
Y	Y		Y		
Y	Y		Y		Y
Y	Y				
Y	Y		Y		
Y	Y				
Y	Y				
Y	Y	Y	Y		
Y	Y	Y			
Y	Y	Y	Y		
Y	Y		Y		
N					
Y	Y		Y		
Y	Y	Y			
Y	Y	Y	Y		Y
Y	Y				
Y	Y	Y	Y		
Y	Y				
Y	Y		Y		
Y	Y	Y	Y	Y	Y
U					
Y	Y	Y	Y		
Y		Y	Y		
Y	Y	Y	Y		
Y	Y		Y	Y	
Y	Y		Y		
Y	Y	Y	Y	Y	
Y	Y		Y		
N					
Y	Y			Y	
Y	Y	Y	Y	Y	Y
Y	Y	Y			
Y	Y				
Y	Y				
Y	Y				
Y	Y	Y			
Y	Y				
Y	Y				
Y	Y		Y		
Y			Y		Y
Y	Y		Y		
Y		Y	Y		
Y	Y		Y		Y
Y	Y	Y			Y
Y	Y				
Y		Y	Y		Y
Y		Y	Y		Y
Y	Y	Y			
Y		Y	Y		Y

Y	Y				
Y	Y	Y	Y		Y
Y		Y			Y
Y	Y				
Y		Y	Y	Y	
Y	Y	Y			
Y	Y				
Y	Y				
Y	Y				
N					
U					
Y	Y	Y			Y
Y	Y	Y	Y		
Y	Y			Y	
Y			Y		
Y	Y	Y			
Y	Y				
Y		Y			
Y	Y				
Y	Y				
Y	Y	Y	Y	Y	Y
Y		Y	Y	Y	Y
Y		Y	Y		
Y	Y	Y	Y		
Y	Y				
Y	Y		Y		
Y		Y	Y		Y
Y	Y		Y		
Y	Y				
Y	Y	Y	Y	Y	Y
Y	Y				
Y	Y				
Y	Y		Y		
Y	Y				
Y		Y			Y
Y	Y				
Y		Y			Y
Y	Y		Y		
U					
U					
Y	Y				
Y	Y			Y	
Y		Y	Y	Y	
Y		Y			
Y		Y	Y		
Y	Y				

U				
Y	Y		Y	
Y	Y			
Y	Y			
Y	Y		Y	
Y	Y			
Y	Y		Y	
U				
U				
Y		Y		
Y	Y		Y	
Y	Y	Y		
Y	Y			
Y	Y	Y		Y
Y		Y		
Y	Y			
Y		Y		
N				
Y	Y			
Y	Y			
Y	Y	Y	Y	
Y		Y		
Y		Y	Y	
Y	Y		Y	
Y	Y			
U				
Y	Y	Y		
U				
Y	Y			
U				
U				
Y	Y			
Y	Y			Y
Y	Y		Y	
Y	Y			
Y	Y			
Y	Y	Y	Y	
Y	Y	Y	Y	
Y			Y	
Y	Y	Y		
Y	Y			
U				
Y	Y			
Y	Y		Y	

Y	Y	Y			
U					
U					
Y	Y	Y			
Y		Y	Y		Y
Y		Y			
Y	Y				Y
Y		Y		Y	
Y	Y				
Y	Y				
Y	Y	Y			
U					
Y	Y	Y			
Y	Y				
Y	Y		Y		
Y	Y	Y			
Y	Y			Y	
Y	Y		Y	Y	
Y	Y			Y	
Y	Y				
Y	Y	Y	Y		
U					
Y		Y	Y		Y
Y	Y		Y	Y	
Y	Y		Y		
Y	Y				
Y	Y				
Y	Y		Y		
Y	Y				
Y	Y		Y		
Y		Y			
N					
Y	Y				
Y	Y	Y			
Y	Y			Y	
U					
Y	Y				
Y	Y				
Y	Y				
Y	Y				
Y		Y	Y		Y
Y		Y			
Y	Y				
Y	Y				
Y	Y				
Y	Y				
Y	Y		Y		
Y		Y			Y
Y	Y				
Y	Y	Y	Y		Y
Y		Y			

Y	Y				
Y	Y				
Y	Y		Y		
Y	Y				
Y	Y				
Y					
Y					
Y		Y			
Y		Y	Y		Y
Y	Y		Y		
Y	Y	Y	Y		Y
Y		Y	Y		
Y	Y		Y	Y	
Y	Y		Y		
Y	Y				
Y		Y			
Y		Y			Y
Y		Y	Y		
Y	Y	Y	Y		
Y			Y		
Y		Y			Y
Y		Y	Y		
Y	Y				
Y	Y		Y		
Y	Y				
Y	Y	Y	Y		Y
Y	Y				
Y	Y	Y			
Y	Y				
Y	Y				
Y	Y	Y	Y		
Y	Y				
Y			Y		
N					
Y	Y			Y	
Y	Y		Y	Y	
Y	Y	Y	Y		
Y	Y		Y	Y	
Y	Y		Y		
Y		Y			
Y	Y	Y	Y		
U					
Y		Y	Y		
Y		Y	Y		
Y	Y			Y	
Y	Y	Y			
Y	Y	Y	Y		
Y	Y	Y	Y		
U					
Y	Y	Y			
Y	Y				

Y	Y				
Y	Y	Y	Y		
Y	Y				
Y	Y	Y	Y		
Y	Y				
Y	Y	Y		Y	
Y	Y				
Y	Y	Y			
Y	Y	Y	Y		
Y	Y				
Y	Y				
Y	Y	Y	Y		Y
Y	Y				
Y	Y				
Y	Y	Y	Y		
Y	Y				
Y	Y	Y		Y	
Y	Y				
Y				Y	
Y	Y		Y		
Y	Y	Y		Y	
Y	Y				
Y	Y		Y		
Y	Y		Y		
Y	Y	Y	Y	Y	
Y	Y				
Y	Y				
Y		Y	Y		
Y	Y	Y	Y		
Y	Y				
Y		Y	Y		
Y	Y				
Y	Y		Y		
Y		Y	Y		
Y	Y		Y	Y	
Y		Y	Y		
Y	Y		Y		
Y	Y				
Y	Y				
Y	Y				
Y	Y	Y	Y	Y	Y
Y					Y
Y		Y	Y		
Y	Y	Y	Y		
Y	Y	Y			
Y		Y	Y		Y
Y		Y			Y
Y	Y				
Y	Y				
Y	Y		Y		
Y	Y				
Y	Y				

Y	Y			
U				
U				
U				
U				
Y	Y			
Y	Y			
Y		Y	Y	
Y	Y		Y	
U				
U				
Y	Y			
Y		Y		
Y	Y		Y	
Y	Y			
Y	Y			
Y	Y		Y	
Y	Y			
Y		Y	Y	
Y	Y	Y		
U				
U				
Y	Y			
U				
Y	Y	Y	Y	
U				
U				
Y	Y			
Y	Y	Y	Y	Y
Y	Y		Y	
Y	Y	Y		
Y	Y	Y		Y
U				
Y		Y		
U				
U				
Y	Y	Y	Y	Y
Y	Y		Y	
U				
Y	Y	Y	Y	Y
Y	Y	Y	Y	
U				
U				
U				
U				
Y	Y	Y	Y	Y
Y	Y	Y	Y	
Y	Y			
U				
Y		Y	Y	
Y	Y			
Y	Y			

Y	Y				
U					
Y	Y		Y		
Y					
Y	Y			Y	
Y		Y	Y		Y
Y	Y				
Y	Y		Y		
Y	Y				
U					
Y		Y	Y		Y
Y	Y	Y			
Y	Y		Y		
Y		Y			
U					
Y		Y	Y		Y
Y		Y			
Y	Y				
Y	Y				
Y	Y	Y	Y		
U					
Y	Y				
Y	Y	Y	Y		
Y	Y	Y	Y		Y
Y	Y	Y			
U					
Y	Y				
Y	Y				
Y	Y		Y		
Y	Y		Y		
N					
Y	Y				
Y		Y	Y	Y	Y
Y	Y				
Y	Y		Y		
Y	Y				
Y	Y	Y	Y		
Y	Y		Y		
Y	Y				
U					
Y	Y	Y			
Y		Y	Y	Y	
Y		Y			
Y				Y	
Y	Y		Y		
Y		Y	Y		Y

Y	Y		Y		
Y	Y				
Y	Y	Y	Y		
Y			Y		Y
Y	Y				
Y	Y	Y	Y		
Y		Y	Y		
Y	Y				
Y	Y				
Y	Y				
Y	Y	Y	Y	Y	
Y	Y				
Y	Y	Y	Y		
Y		Y			
Y	Y				
Y		Y	Y	Y	
Y	Y		Y		
Y		Y	Y		
Y	Y	Y			
Y	Y	Y	Y	Y	Y
Y	Y		Y		
Y	Y	Y	Y	Y	
Y	Y	Y	Y		
Y	Y				
Y	Y	Y	Y	Y	
Y	Y	Y	Y		
Y	Y				
Y	Y		Y		
Y	Y	Y	Y	Y	
Y	Y				
Y		Y	Y	Y	Y
Y	Y		Y		
Y	Y				
Y	Y		Y		Y
Y	Y				
Y	Y				
Y			Y		
Y	Y	Y	Y	Y	Y
Y	Y				
Y	Y				
Y	Y				
Y	Y	Y	Y		
Y	Y				
Y	Y				
Y	Y		Y		Y
Y	Y				

Y	Y				
Y	Y		Y		Y
Y	Y		Y		
Y	Y	Y	Y	Y	Y
Y	Y		Y	Y	
Y	Y				
Y	Y		Y	Y	Y
Y	Y				
Y	Y	Y			
Y	Y	Y			
Y	Y				
Y	Y		Y		
Y	Y	Y	Y		
Y	Y		Y		
Y	Y				
Y	Y				
Y		Y			
Y		Y			
Y	Y				
Y	Y				
Y	Y				
Y	Y				
Y	Y				
Y	Y		Y	Y	
Y	Y		Y		
Y		Y			
Y	Y				
N					
U					
N					
Y	Y		Y	Y	
Y	Y				
Y	Y	Y			

Y	Y		Y		
Y	Y		Y		
Y	Y				
Y		Y			
Y	Y				
Y	Y		Y		
Y	Y				
Y	Y				
Y	Y				
Y		Y	Y		
Y	Y				
Y	Y				
Y	Y				
Y	Y	Y	Y		
Y	Y		Y		Y
Y		Y			
Y	Y				
Y	Y				
Y	Y				
Y	Y	Y	Y		
Y		Y			
Y		Y	Y		Y
Y		Y			
Y	Y		Y		
Y	Y		Y	Y	
Y	Y				
Y		Y			
Y		Y	Y		
Y	Y				
Y	Y		Y		
Y		Y	Y		
Y	Y				
Y	Y		Y		
Y		Y	Y		
Y		Y	Y		
Y	Y				
Y	Y				
Y	Y	Y	Y	Y	Y
Y	Y		Y		

Y	Y				
Y	Y	Y	Y		
Y		Y	Y		
Y	Y		Y		
Y	Y				
Y	Y				
Y		Y	Y		
Y		Y			
Y	Y				
Y	Y				
Y	Y		Y		
Y	Y		Y		
Y	Y				
U					
Y	Y				
U					
Y	Y		Y	Y	
U					
Y	Y			Y	
Y	Y				
Y	Y	Y	Y		
Y	Y	Y	Y		
Y	Y				
Y	Y	Y	Y	Y	Y
Y	Y	Y	Y		Y
Y	Y	Y	Y	Y	Y
Y	Y	Y	Y		
Y	Y				
Y		Y	Y		
Y	Y				
Y	Y				
Y	Y		Y		
Y		Y	Y		
U					
Y	Y		Y		
Y	Y		Y		
Y	Y		Y		
Y	Y				
Y	Y				
Y	Y		Y		
Y	Y	Y	Y		

Y	Y				
U					
U					
Y		Y	Y		
U					
Y	Y	Y	Y		
Y	Y	Y	Y	Y	Y
Y	Y	Y	Y	Y	
Y	Y	Y	Y	Y	
Y	Y	Y	Y		
Y	Y	Y	Y	Y	Y
Y	Y				
Y	Y	Y	Y	Y	Y
Y	Y				
Y	Y				
Y	Y		Y		
Y	Y				
Y	Y		Y		
Y	Y		Y		
Y	Y	Y	Y	Y	
Y	Y			Y	
Y	Y	Y	Y	Y	Y
Y	Y				
Y	Y	Y	Y	Y	Y
Y	Y	Y			
Y	Y		Y		
Y	Y	Y			
Y	Y		Y		
Y		Y	Y		
Y	Y				
Y		Y	Y	Y	Y
Y	Y				
Y	Y				
Y	Y				Y
Y		Y			
U					
U					
U					
U					
Y	Y		Y		
Y	Y	Y			
Y	Y				
Y	Y				
Y	Y				
U					
Y	Y		Y		
Y		Y			

Y	Y	Y			
Y	Y				
Y	Y	Y	Y		
Y	Y	Y	Y		
U					
Y		Y			
Y	Y				
U					
Y	Y				
N					
Y	Y				
U					
U					
Y	Y				
Y	Y	Y		Y	
Y	Y				
Y	Y	Y	Y		Y
Y	Y		Y		
Y		Y			
Y	Y				
Y	Y				
Y		Y			
Y	Y	Y	Y		
Y	Y		Y	Y	
Y	Y				
Y	Y				
Y	Y				
Y	Y				
Y	Y		Y		
Y	Y				
Y	Y	Y			Y
Y		Y			
Y	Y				
Y	Y				
Y	Y				
Y	Y				
Y	Y				
Y	Y				
Y	Y		Y		
Y	Y				
Y		Y			
Y	Y	Y	Y		
Y	Y				
Y	Y	Y	Y		
Y	Y		Y		
Y	Y				
Y	Y			Y	
Y	Y	Y	Y	Y	

Y	Y	Y			
Y	Y				
Y	Y				
Y	Y				
Y	Y		Y		
Y	Y	Y	Y	Y	
Y	Y		Y		
Y	Y				
Y	Y		Y		
Y	Y	Y			
Y	Y		Y		
Y	Y	Y	Y	Y	Y
Y	Y	Y	Y	Y	Y
Y	Y	Y	Y		
Y	Y		Y		
Y	Y	Y			
Y	Y	Y		Y	
Y	Y	Y	Y		

Y	Y	Y		
Y	Y			
Y	Y	Y	Y	Y
Y	Y	Y	Y	

Y	Y				
Y	Y		Y	Y	
Y	Y				
N					
Y	Y			Y	
Y	Y	Y		Y	
Y	Y				
Y	Y	Y	Y		
Y		Y			
Y	Y		Y		
Y	Y				
Y	Y	Y			
Y	Y	Y	Y	Y	Y
Y	Y	Y	Y	Y	Y
Y	Y	Y	Y		
Y		Y		Y	
Y	Y			Y	
Y	Y				
Y	Y		Y		
Y	Y	Y	Y	Y	Y
Y					
Y		Y	Y	Y	Y
Y	Y				
Y	Y	Y			
U					
Y		Y	Y	Y	
Y	Y	Y	Y		
Y	Y	Y	Y		
Y	Y		Y		
Y	Y		Y		
Y					
Y	Y		Y		
Y		Y		Y	
Y	Y	Y			
Y	Y	Y	Y	Y	
Y	Y				
Y	Y		Y		
Y	Y		Y		
Y	Y	Y	Y		
Y	Y				
Y		Y			
Y		Y	Y		Y
Y	Y				
Y	Y	Y			
Y	Y				
Y	Y				
Y		Y		Y	
Y	Y				
Y	Y				

Y	Y	Y			
Y	Y	Y			
Y	Y				
Y	Y		Y		
Y	Y		Y		
Y	Y		Y	Y	
Y		Y			
Y	Y	Y	Y		
Y	Y		Y		
Y	Y				
Y	Y		Y		
Y	Y	Y	Y	Y	Y
Y	Y	Y			
Y	Y				
U					
Y	Y				
Y	Y				
U					
U					
Y	Y				
Y	Y	Y	Y		Y
U					
Y		Y	Y	Y	
Y		Y	Y		
Y	Y				
Y		Y			
U					
Y		Y			
Y	Y				
Y	Y	Y			
Y	Y				
Y	Y	Y			
Y	Y	Y	Y	Y	Y
U					
Y	Y				
Y	Y				
N					
Y	Y				
U					
Y	Y	Y	Y		

Y	Y	Y	Y		
N					
Y	Y				
Y		Y	Y		Y
Y	Y	Y	Y		
Y	Y				
Y	Y				
Y	Y	Y	Y		
Y	Y				
Y	Y			Y	
Y	Y		Y		
Y	Y				
Y	Y		Y		
Y	Y	Y	Y	Y	
Y	Y				
Y	Y				
Y	Y	Y	Y		
Y	Y	Y	Y		Y
Y		Y			Y
Y	Y			Y	
Y	Y	Y	Y		
Y	Y				
Y	Y				
Y	Y	Y	Y		
Y	Y	Y	Y		Y
Y		Y			
Y	Y	Y			
Y	Y	Y	Y		Y
U					
Y	Y	Y			
Y	Y		Y		
Y	Y				
Y		Y	Y		
Y	Y				
Y	Y	Y	Y		
Y	Y			Y	
Y	Y		Y		
Y	Y		Y	Y	
Y	Y				
Y	Y	Y			
Y	Y	Y	Y		
Y	Y	Y	Y	Y	Y
Y	Y				
Y		Y			
Y		Y	Y		Y

Y	Y	Y			
Y	Y				
Y	Y				
Y	Y				
Y	Y				
Y	Y				
Y	Y				
Y			Y		
Y		Y		Y	Y
Y	Y				Y
Y	Y				
Y	Y		Y		
Y	Y	Y	Y		Y
Y	Y	Y			
Y	Y		Y	Y	
Y	Y		Y		
Y	Y		Y	Y	
Y		Y	Y		Y
Y	Y	Y	Y		
Y	Y				
Y	Y				
Y	Y		Y		
U					
Y	Y				
Y		Y			
Y		Y			
Y	Y				
Y	Y				
Y	Y				
Y	Y		Y		
Y	Y				
Y	Y		Y		
Y	Y				
Y	Y				
U					
Y		Y			
Y	Y	Y	Y	Y	Y
Y		Y	Y		
Y	Y				Y
Y		Y	Y		Y
Y	Y				
Y			Y		
Y		Y			
Y	Y				
Y		Y			
Y		Y			
Y	Y	Y	Y		
Y	Y				
Y		Y			

Y	Y				
Y		Y			
Y	Y				
Y	Y		Y		
Y	Y				
Y				Y	
Y	Y				
Y	Y		Y	Y	
Y	Y	Y	Y		Y
Y	Y	Y	Y		
Y	Y	Y	Y		
U					
Y	Y	Y	Y	Y	Y
Y	Y	Y	Y	Y	Y
Y	Y				
Y	Y	Y			Y
Y	Y				
Y	Y	Y	Y		
Y	Y				
Y	Y	Y	Y		
Y	Y	Y			
U					
Y			Y		
Y	Y				
Y	Y	Y	Y	Y	Y
Y	Y				
Y	Y	Y			
Y		Y			
Y	Y	Y	Y		Y
Y		Y	Y		Y
Y	Y				
Y	Y				
Y		Y			
Y		Y		Y	Y
Y	Y	Y	Y		
Y	Y		Y	Y	Y
Y	Y				
Y	Y	Y		Y	Y
Y	Y				
Y	Y	Y	Y		
Y	Y				
Y	Y		Y		
Y	Y		Y		
Y	Y				Y
Y	Y				
Y	Y	Y	Y		
Y	Y				

Y	Y				
Y	Y	Y			Y
Y		Y	Y	Y	
Y		Y			
U					
Y	Y	Y	Y	Y	
Y	Y		Y		
Y	Y		Y		
Y	Y		Y		
Y		Y	Y		
Y	Y				
Y	Y				
Y	Y	Y			
Y	Y	Y			
Y	Y	Y		Y	
Y	Y				
Y	Y	Y			
Y		Y			Y
Y	Y		Y	Y	
Y		Y			
Y	Y				
Y	Y	Y	Y		
U					
Y	Y				
Y	Y	Y	Y	Y	Y
Y	Y	Y			
Y		Y	Y		Y
Y		Y			
Y		Y	Y		Y
Y	Y		Y		
Y	Y				
Y	Y	Y			
Y	Y				
Y	Y	Y	Y		
Y	Y			Y	
Y		Y			
Y	Y				Y
U					
Y		Y			

Y			Y		
Y		Y	Y		
Y	Y	Y	Y		
Y	Y	Y	Y		
Y	Y		Y	Y	
Y	Y	Y			Y
Y	Y	Y			
Y	Y		Y		
Y	Y				
Y	Y		Y		
U					
Y		Y			
Y	Y				
Y	Y	Y	Y	Y	Y
Y	Y	Y	Y	Y	Y
Y	Y				
Y		Y			
Y	Y				
Y		Y			
Y	Y				
Y		Y			Y
Y	Y				
Y	Y	Y	Y		
Y		Y			
Y	Y	Y	Y		
Y	Y				
Y	Y				
Y	Y				
Y	Y				
Y	Y	Y	Y	Y	Y
N					
Y	Y				
Y	Y				
Y	Y				
Y	Y	Y	Y		

Y	Y	Y	Y		Y
Y	Y	Y			
Y	Y				
Y	Y				
Y		Y			
Y	Y				
Y	Y		Y		
Y	Y	Y	Y		
Y	Y	Y	Y		
Y	Y	Y	Y		
Y	Y				
Y	Y		Y		
U					
Y	Y	Y		Y	
Y	Y				
Y	Y	Y			
Y		Y			
Y	Y				
U					
Y	Y	Y	Y		
Y	Y	Y	Y		
Y	Y	Y	Y		
Y	Y		Y		
Y	Y		Y		
N					
Y	Y				
Y	Y				
Y	Y	Y	Y	Y	
Y	Y				
Y	Y				
Y	Y		Y		
Y	Y		Y		
Y	Y			Y	
Y		Y			
Y	Y	Y	Y		
Y	Y	Y	Y		Y
U					
Y	Y				
Y	Y		Y		

Y	Y				
Y	Y		Y		
Y		Y	Y		Y
Y	Y				
Y	Y				
Y	Y				
Y	Y	Y			
Y	Y				
Y		Y	Y		Y
Y	Y		Y		
Y	Y				
Y	Y				
Y	Y				
Y		Y	Y		Y
U					
Y	Y		Y	Y	
Y	Y	Y			
Y	Y		Y		
Y	Y	Y			
Y	Y				
Y		Y			
U					
Y	Y	Y			
Y	Y	Y	Y	Y	Y
Y	Y		Y		
Y	Y				
Y	Y		Y		
Y	Y				
Y	Y	Y			
U					
Y		Y			
Y	Y				
Y					
Y	Y	Y	Y		
Y	Y		Y		
Y	Y				
Y	Y	Y		Y	
Y	Y				
Y	Y		Y		
U					
Y	Y		Y	Y	

Y	Y		Y		
Y		Y	Y		
Y		Y	Y		
Y	Y	Y			
Y	Y				
Y	Y				
Y	Y				
Y	Y	Y	Y		Y
Y	Y	Y	Y		Y
Y	Y				
Y	Y		Y		
Y	Y	Y	Y		Y
Y	Y		Y		
Y	Y		Y		
Y	Y		Y		
Y		Y			Y
U					
Y	Y				
Y	Y				
Y	Y				
Y	Y	Y			
Y	Y				
Y	Y				
Y	Y				
Y	Y			Y	
Y		Y	Y	Y	Y
Y	Y				
Y	Y				
Y	Y	Y	Y	Y	Y
Y					Y
Y	Y				
Y		Y	Y		
Y	Y				
Y					
Y	Y				
U					
Y	Y				
Y	Y		Y		
Y	Y				
Y	Y		Y	Y	Y
Y	Y				

Y	Y				
Y	Y				
Y	Y		Y		
Y	Y		Y		
Y	Y				
Y	Y				
Y	Y				
Y	Y				
Y		Y			
Y	Y				
Y	Y				
Y	Y	Y	Y	Y	Y
Y		Y			
N					
Y	Y				
U					
Y	Y				
Y	Y				
Y	Y				
U					
Y	Y				
Y	Y				
Y	Y		Y		
Y	Y				
Y	Y		Y		
Y	Y	Y		Y	
Y		Y			
U					
Y	Y	Y			
Y	Y				
Y	Y				Y
Y	Y				

Y	Y		Y	Y	Y
Y	Y	Y	Y		
Y	Y				
Y	Y	Y	Y		
Y	Y				Y
Y	Y				
Y	Y	Y		Y	
Y	Y				
Y	Y		Y		
Y	Y				
Y	Y		Y		
Y	Y	Y	Y		
Y	Y	Y	Y		Y
Y	Y		Y		
Y					
Y	Y		Y		
Y	Y				
Y		Y			Y
Y	Y				
Y	Y		Y		
Y	Y		Y		
Y	Y				
Y	Y				
Y	Y				
Y		Y			
Y	Y		Y		
Y	Y	Y	Y		
Y	Y				
U					
Y	Y		Y		
Y	Y				
Y	Y		Y		
Y		Y			Y
Y	Y		Y		
Y	Y				
Y	Y				
Y	Y	Y			Y
Y	Y				
Y	Y				
Y	Y	Y			
Y	Y				
Y	Y				

Y	Y			
Y	Y	Y		
Y		Y	Y	Y
Y	Y			
Y	Y	Y		
Y	Y			
Y	Y			
Y	Y			
Y	Y			
Y	Y	Y		
Y	Y	Y	Y	Y
Y	Y	Y		
Y		Y		Y
Y	Y			
Y	Y	Y	Y	
Y	Y			
Y		Y	Y	
Y	Y	Y		
Y	Y			
Y	Y	Y		
Y		Y		
Y	Y			
Y	Y	Y		
Y	Y			
Y	Y			
Y	Y			
Y	Y			
Y	Y	Y	Y	Y
Y	Y			
Y	Y	Y		Y
Y	Y		Y	
Y	Y			
Y	Y			
Y	Y	Y		Y
Y	Y			
Y	Y	Y	Y	
U				
Y	Y			

Y	Y		Y		
Y	Y	Y	Y		
Y	Y	Y	Y		
Y	Y	Y		Y	
Y	Y				
U					
Y	Y	Y			
Y	Y				
Y		Y	Y		
Y	Y	Y	Y	Y	Y
Y		Y	Y		
Y	Y				
Y	Y	Y	Y		
N					
Y	Y				
Y	Y	Y	Y		
Y	Y	Y	Y		
Y	Y				
Y	Y	Y			
Y	Y				
Y	Y			Y	
Y	Y		Y	Y	
Y	Y	Y	Y		
Y	Y	Y	Y	Y	Y
Y	Y	Y	Y	Y	
Y	Y	Y	Y	Y	Y
Y	Y	Y			
Y	Y	Y			
Y	Y				
Y	Y		Y		Y
Y	Y	Y	Y		Y
Y	Y				
Y	Y				
Y	Y	Y			
Y	Y	Y	Y		
Y	Y	Y	Y		
Y	Y	Y	Y		
Y	Y				

Y	Y		Y	Y	
Y	Y	Y			
Y	Y				
Y	Y	Y	Y	Y	Y
Y	Y	Y			
Y	Y	Y	Y		Y
Y	Y				
Y	Y		Y	Y	
Y	Y				
Y	Y				
Y	Y				
Y	Y				
Y	Y				
Y	Y				
Y	Y	Y	Y	Y	
Y	Y	Y			
Y				Y	
Y	Y	Y	Y		Y
Y	Y				
U					
Y	Y				
Y	Y		Y		
Y	Y				
Y	Y	Y			
Y	Y				
Y	Y				
Y	Y				
Y	Y				
Y	Y	Y	Y		
Y	Y	Y	Y		
Y	Y	Y	Y		Y
Y	Y	Y			
Y	Y				

Y		Y			Y
Y	Y	Y	Y		
Y	Y	Y	Y		
Y		Y			Y
Y		Y	Y	Y	Y
Y	Y		Y		
Y		Y			
Y	Y				
Y	Y		Y		Y
Y	Y	Y	Y		Y
Y	Y	Y	Y		Y
Y		Y			
Y	Y				
Y	Y				
U					
Y	Y		Y		
U					
Y	Y				
Y	Y	Y	Y		
Y		Y			
Y	Y				
Y	Y				
U					
Y	Y	Y			
Y	Y				
Y		Y	Y		Y
Y	Y				
Y	Y				
U					
Y	Y		Y		
Y		Y			
Y		Y			
N					

Y	Y	Y		Y	
Y	Y				
Y	Y		Y		
Y	Y	Y	Y		
Y	Y				
Y		Y			
Y	Y		Y		
Y	Y		Y		Y
U					
Y	Y				
U					
Y	Y				
Y	Y	Y	Y		
U					
Y	Y		Y		
U					
Y	Y				
Y	Y	Y	Y		
Y	Y				
U					
Y	Y	Y	Y		Y
Y	Y				
Y	Y	Y	Y		Y
Y	Y				
Y	Y		Y		
Y					
Y	Y				
Y		Y	Y	Y	

Y	Y			Y	
Y	Y	Y	Y		Y
Y	Y	Y			
U					
Y	Y		Y		
Y	Y				
Y		Y	Y		Y
Y	Y	Y			
Y		Y	Y	Y	Y
Y		Y	Y	Y	Y
Y		Y			
Y	Y				
Y	Y	Y			
Y	Y		Y		
Y	Y		Y		
Y	Y				
Y	Y				
Y	Y				
Y	Y	Y	Y		
Y	Y				
Y	Y		Y	Y	Y
Y	Y		Y		
Y	Y		Y		
Y	Y	Y		Y	
Y		Y	Y		Y
Y	Y				
Y	Y				
Y	Y				
Y	Y				

Y	Y				
Y	Y				
Y	Y				
Y	Y	Y			
Y	Y				
Y	Y				
Y	Y	Y	Y	Y	Y
Y	Y				
Y	Y				
Y	Y				
Y	Y		Y		
Y	Y				
U					
U					
Y	Y				
Y	Y	Y	Y		
Y	Y		Y		
Y	Y		Y		
Y	Y	Y	Y		
U					
Y	Y				
Y	Y		Y		

Y	Y		Y		Y
Y	Y		Y		
Y	Y		Y		
U					

Y		Y	Y		Y
Y	Y	Y	Y		
Y	Y	Y	Y	Y	Y
Y		Y			
Y	Y	Y	Y		
Y	Y				
Y	Y				
Y	Y				
Y	Y				

Y	Y	Y	Y		
Y	Y				

other_meddevice	other_meddevice_spec	risk	underlying_con	diabetes
		Y	Yes	
		Y	Yes	
		Y	Yes	
		N	No	
		N	No	
		U	Unknown	
		U	Unknown	
		N	No	
		Y	Yes	
		N	No	
		Y	Yes	
		Y	Yes	
		N	No	
		N	No	
		N	No	
		N	No	
		N	No	
		N	No	
		N	No	
		Y	Yes	Y
		Y	Yes	
		Y	Yes	
		N	No	
		N	No	
		Y	Yes	
		Y	Yes	
		U	Unknown	
		N	No	
Y	NG TUBE	Y	Yes	Y
		N	No	
		N	No	
Y	PORT POMTONGAL DIALYSIS	Y	Yes	
		Y	Yes	
Y	NASO-GASTRIC TUBE	Y	Yes	
		Y	Yes	Y
		Y	Yes	Y
		U	Unknown	
		N	No	
Y	PORTOVAC	Y	Yes	
		Y	Yes	
		Y	Yes	
		Y	Yes	
Y	CHEMOTHERAPY PORT	Y	Yes	
		Y	Yes	
		N	No	
		Y	Yes	Y
		Y	Yes	
		N	No	

		Y	Yes	
		Y	Yes	
		Y	Yes	
		Y	Yes	
		Y	Yes	
		U	Unknown	
		N	No	
		N	No	
		N	No	
Y	CVP	N	No	
		Y	Yes	
		Y	Yes	
		N	No	
Y	pre mature	Y	Yes	
		N	No	
		N	No	
		Y	Yes	Y
		Y	Yes	
		Y	Yes	
		Y	Yes	
Y	ENDOTRACHEAL TUBE, NASOGASTRIC	Y	Yes	
Y	INTERCOSTAL DRAIN	N	No	
		U	Unknown	
		Y	Yes	
		Y	Yes	
		Y	Yes	
		Y	Yes	Y
Y	HICKMAN LINE	Y	Yes	
		Y	Yes	
Y	COLOSTOMY BAG	N	No	
		Y	Yes	
		N	No	
		Y	Yes	
Y	STOMA BAG	N	No	
		U	Unknown	
		Y	Yes	
		Y	Yes	
		N	No	
		N	No	
		U	Unknown	
Y	NASOGASTRIC TUBE	N	No	
		N	No	
Y	STOMA - PEFORATED BOWEL	Y	Yes	
Y	CHEMOTHERAPY PORT	Y	Yes	
		Y	Yes	
		N	No	
Y	CHEMOTHERAPY PORT	Y	Yes	
		N	No	
		Y	Yes	
		Y	Yes	Y
		N	No	

		N	No	
		U	Unknown	
		U	Unknown	
		N	No	
		Y	Yes	
		U	Unknown	
		N	No	
		N	No	
		N	No	
		N	No	
		Y	Yes	
		Y	Yes	
		N	No	
Y	NASOGASTRIC TUBE	Y	Yes	
		N	No	
		U	Unknown	
		Y	Yes	Y
		N	No	
		Y	Yes	
		U	Unknown	
		U	Unknown	
		Y	Yes	
Y	GASTRIC TUBE JEJUNOSTOMY	Y	Yes	
		Y	Yes	
		N	No	
		U	Unknown	
		N	No	
		N	No	
		Y	Yes	
Y	NASOGASTRIC TUBE	N	No	
		N	No	
		Y	Yes	
		Y	Yes	Y
		Y	Yes	
		Y	Yes	
Y	NASOGASTRIC TUBE	Y	Yes	
Y	TRACHEOSTOMY	Y	Yes	Y
		Y	Yes	
		Y	Yes	
		Y	Yes	
		Y	Yes	
		Y	Yes	Y
Y	NASOGASTRIC TUBE	N	No	
		N	No	
Y	NASO GASTRIC TUBE	Y	Yes	
Y	NASO GASTRIC TUBE	N	No	
Y	LEFT SIDED INTERCOSTAL DRAIN	Y	Yes	
		N	No	
		N	No	
		Y	Yes	
		N	No	
		Y	Yes	Y
		N	No	
		N	No	

		Y	Yes	
		Y	Yes	
		N	No	
		N	No	
Y	TRACHEOTOMY	Y	Yes	
		N	No	
		U	Unknown	
		N	No	
		Y	Yes	Y
		U	Unknown	
		Y	Yes	
		N	No	
		N	No	
		N	No	
		U	Unknown	
		U	Unknown	
		Y	Yes	
Y	TRACHEOTOMY TUBE	Y	Yes	
		Y	Yes	
		U	Unknown	
		Y	Yes	
		Y	Yes	
		Y	Yes	
		Y	Yes	
		Y	Yes	
		Y	Yes	Y
		N	No	
Y	NASO GASTRIC TUBE	N	No	
		N	No	
Y	NASO GASTRIC TUBE	N	No	
		Y	Yes	
Y	HICKMAN LINE	Y	Yes	
Y	INTERCOASTAL DRAIN	Y	Yes	
		Y	Yes	
		U	Unknown	
Y	NASO GASTRIC TUBE	Y	Yes	
		N	No	
		N	No	
		N	No	
		N	No	
		N	No	
		N	No	
		Y	Yes	
		U	Unknown	
		Y	Yes	
		Y	Yes	
Y	Q-LINE	Y	Yes	
		U	Unknown	
		Y	Yes	
		U	Unknown	
		Y	Yes	
		N	No	
		N	No	
Y	COLOSTOMY BAG	Y	Yes	
		N	No	

		N	No	
		N	No	
		N	No	
		N	No	
		Y	Yes	
		Y	Yes	
		Y	Yes	
		U	Unknown	
Y	TRACHEOSTOMY	Y	Yes	
		N	No	
		Y	Yes	
		N	No	
		N	No	
		U	Unknown	
		Y	Yes	Y
U		U	Unknown	
Y	ENDOTRACHEAL TUBE	N	No	
		Y	Yes	Y
		Y	Yes	
		Y	Yes	
Y	BILATERAL STOMAS	Y	Yes	
Y	THRACHEOSTOMY	N	No	
		Y	Yes	
		Y	Yes	
		U	Unknown	
		Y	Yes	Y
		Y	Yes	
		N	No	
		N	No	
		N	No	
		N	No	
		Y	Yes	
		Y	Yes	
Y	STOMA BAG	N	No	
		Y	Yes	
		Y	Yes	
		Y	Yes	
		Y	Yes	
		U	Unknown	
		U	Unknown	
Y	NG TUBE	N	No	
		N	No	
		N	No	
		N	No	
		N	No	
Y	COLOSTOMY BAG	U	Unknown	
		Y	Yes	
		Y	Yes	
		N	No	
Y	VASCULAR CATHETER	Y	Yes	
Y	PTC I, STENT	Y	Yes	
		U	Unknown	
		Y	Yes	Y
		Y	Yes	Y

		Y	Yes	
		U	Unknown	
		Y	Yes	Y
		U	Unknown	
		N	No	
		N	No	
Y	VASCULAR CATHETER	Y	Yes	
		Y	Yes	
		N	No	
		Y	Yes	
		Y	Yes	
		N	No	
		Y	Yes	
		Y	Yes	
		Y	Yes	
		U	Unknown	
		U	Unknown	
		U	Unknown	
Y	NASAL PRONGS, NG TUBE,	N	No	
		U	Unknown	
		U	Unknown	
		U	Unknown	
Y	VENTILATOR	U	Unknown	
		U	Unknown	
		U	Unknown	
		N	No	
		U	Unknown	
		N	No	
		N	No	
		Y	Yes	Y
		Y	Yes	Y
		Y	Yes	
		N	No	
		Y	Yes	Y
		Y	Yes	
		N	No	
		Y	Yes	
		N	No	
		Y	Yes	
		N	No	
		N	No	
		Y	Yes	
		N	No	
		Y	Yes	
		Y	Yes	
		U	Unknown	
		U	Unknown	
		Y	Yes	
		Y	Yes	Y
Y	PORTOVAC	N	No	
		Y	Yes	
		U	Unknown	
		N	No	
		Y	Yes	
		N	No	

		Y	Yes	Y
Y	CANNULAS DISTAL LUMEN (FOR FEE	N	No	
		U	Unknown	
		U	Unknown	
		U	Unknown	
		N	No	
		Y	Yes	Y
		U	Unknown	
		N	No	
		U	Unknown	
		U	Unknown	
		N	No	
Y	VP SHUNT	Y	Yes	
Y	GASTROSTOMY TUBE	N	No	
		Y	Yes	
		Y	Yes	Y
		U	Unknown	
		Y	Yes	
		Y	Yes	
		Y	Yes	
		Y	Yes	
Y	ICD	N	No	
		U	Unknown	
		Y	Yes	
		N	No	
		N	No	
Y	FEEDING ON TPN	Y	Yes	Y
		U	Unknown	
		N	No	
		Y	Yes	Y
		N	No	
		Y	Yes	
		U	Unknown	
		U	Unknown	
		U	Unknown	
		U	Unknown	
		Y	Yes	
		Y	Yes	
		Y	Yes	
		U	Unknown	
		U	Unknown	
		Y	Yes	
Y	MEDICAL REPORT IS INCOMPLETE	N	No	
		Y	Yes	
		N	No	
		U	Unknown	
Y	SUPRAPUBIC CATHETER	Y	Yes	Y
		N	No	
		Y	Yes	Y

Y	TEMPORARY PACEMAKER	Y	Yes	
		N	No	
		Y	Yes	
		Y	Yes	
		Y	Yes	
Y	TENKHOFF	Y	Yes	
		Y	Yes	
Y	NASOGASTRIC TUBE	Y	Yes	
Y	NASOGASTRIC TUBE, OPEN ABDOMEN	Y	Yes	
		Y	Yes	
		Y	Yes	
		U	Unknown	
		U	Unknown	
Y	VENTILATOR	Y	Yes	
Y	A-V SHUNT	Y	Yes	
Y	3 PIGTAILS AND FISTULA	Y	Yes	
		N	No	
		U	Unknown	
		Y	Yes	
Y	A DOUBLE-J STENT AND A UROSTOM	Y	Yes	
		N	No	
		U	Unknown	
		U	Unknown	
		U	Unknown	
		Y	Yes	
		Y	Yes	
		Y	Yes	Y
Y	TRACHEOSTOMY	Y	Yes	
Y	TRACHEOSTOMY	Y	Yes	Y
		U	Unknown	
		Y	Yes	
		U	Unknown	
		U	Unknown	
		Y	Yes	
Y	TRACHEOSTOMY	Y	Yes	
		U	Unknown	
		U	Unknown	
Y	NASOGASTRIC TUBE	Y	Yes	Y
Y	VASCATH	Y	Yes	
		N	No	
		Y	Yes	
		U	Unknown	
		Y	Yes	
		U	Unknown	
		U	Unknown	
		N	No	
		U	Unknown	
		U	Unknown	
		N	No	
		Y	Yes	
		N	No	
		N	No	
		U	Unknown	
		N	No	

		Y	Yes	
		Y	Yes	
		Y	Yes	
		N	No	
		N	No	
		N	No	
		N	No	
Y	GASTRIC TUBE	Y	Yes	Y
Y	NG TUBE	U	Unknown	
		U	Unknown	
		U	Unknown	
		N	No	
		Y	Yes	
		N	No	
		N	No	
		Y	Yes	Y
Y	BROVIAC LINE	Y	Yes	
		Y	Yes	Y
		U	Unknown	
		Y	Yes	Y
		U	Unknown	
Y	VENTILATOR, NASOGASTRIC TUBE	U	Unknown	
Y	VENTILATOR	Y	Yes	
		Y	Yes	
		U	Unknown	
Y	VENTILATOR	N	No	
Y	CPAP VENTILATOR, OROGASTRIC TU	N	No	
		Y	Yes	Y
		Y	Yes	
		Y	Yes	
		N	No	
		N	No	
		N	No	
Y	TRACHEOSTOMY	Y	Yes	Y
		Y	Yes	
		Y	Yes	
		N	No	
		U	Unknown	
		Y	Yes	
		U	Unknown	
		N	No	
		Y	Yes	Y
		U	Unknown	
Y	VENTRICULAR PERITORAL SHUNT	N	No	
Y	COLOSTOMY BAG	Y	Yes	
		N	No	
		N	No	
		Y	Yes	
		Y	Yes	
		N	No	
		Y	Yes	
Y	VENTRICULAR SHUNT	N	No	
		Y	Yes	
		N	No	
		N	No	

		Y	Yes	Y
Y	VENTILATOR, I C DRAIN	Y	Yes	
		N	No	
		U	Unknown	
		U	Unknown	
Y	ILLEAL DRAIN	U	Unknown	
		U	Unknown	
		U	Unknown	
		U	Unknown	
		U	Unknown	
		U	Unknown	
Y	NASOGASTRIC TUBE, VENTILATOR	N	No	
		U	Unknown	
		Y	Yes	
		N	No	
		Y	Yes	
Y	NG TUBE/TRACHEOSTOMY	Y	Yes	Y
		N	No	
		Y	Yes	
		Y	Yes	Y
Y	NASO-GASTRIC TUBE	Y	Yes	
		Y	Yes	
		Y	Yes	
		Y	Yes	
		Y	Yes	
		Y	Yes	Y
Y	NASOGASTRIC TUBE	Y	Yes	
		N	No	
Y	PEG TUBE	N	No	
		N	No	
		N	No	
		N	No	
		Y	Yes	
		Y	Yes	
		N	No	
		N	No	
		N	No	
		Y	Yes	
		Y	Yes	
		N	No	
		N	No	
		U	Unknown	
		U	Unknown	
		Y	Yes	Y
		U	Unknown	
		U	Unknown	
		Y	Yes	
		N	No	
		U	Unknown	
Y	NASOGASTRIC TUBE, ENDOTRACHEAL	Y	Yes	
		U	Unknown	
		U	Unknown	
		Y	Yes	
		Y	Yes	

		Y	Yes	
		Y	Yes	
		N	No	
		Y	Yes	
		Y	Yes	
		Y	Yes	
		Y	Yes	
		N	No	
		Y	Yes	
		Y	Yes	
Y	NO FULL INFORMATION CAN BE FOU	N	No	
		U	Unknown	
		U	Unknown	
Y	COLOSTOMY BAG	Y	Yes	
		U	Unknown	
		Y	Yes	
		Y	Yes	
Y	TRACHEOSTOMY	N	No	
Y	COLOSTOMY BAG	N	No	
		Y	Yes	
Y	COLOSTOMY BAG	N	No	
		N	No	
		Y	Yes	
Y	NASO GASTRIC TUBE	N	No	
		Y	Yes	
		Y	Yes	
Y	IRRIGATION	Y	Yes	Y
		Y	Yes	Y
		Y	Yes	
		N	No	
Y	NG TUBE	U	Unknown	
		Y	Yes	
		Y	Yes	
		Y	Yes	Y
		Y	Yes	
		Y	Yes	Y
		Y	Yes	
		Y	Yes	
		Y	Yes	
		Y	Yes	
		N	No	
		U	Unknown	
		N	No	
		N	No	
		Y	Yes	
		U	Unknown	
Y	OXYGEN MASK	Y	Yes	Y
		U	Unknown	
		N	No	
		Y	Yes	Y
		U	Unknown	
Y	TRACHEOSTOMY	N	No	
		U	Unknown	
		U	Unknown	

Y	PEG	Y	Yes	
		Y	Yes	Y
		U	Unknown	
		U	Unknown	
		U	Unknown	
Y	COLOSTOMY BAG,NASOGASTRIC TUBE	N	No	
Y	NASOGASTRIC TUBE	Y	Yes	
		Y	Yes	
		Y	Yes	Y
		Y	Yes	
		Y	Yes	Y
		Y	Yes	Y
Y	NASOGASTRIC TUBE	Y	Yes	Y
		Y	Yes	
		U	Unknown	
		U	Unknown	
		Y	Yes	Y
		U	Unknown	
		U	Unknown	
		Y	Yes	
		Y	Yes	Y
		Y	Yes	
		N	No	
		N	No	
		N	No	
		U	Unknown	
Y	NG TUBE	Y	Yes	
		Y	Yes	
		N	No	
		U	Unknown	
		Y	Yes	Y
		Y	Yes	
Y	TRACHEOSTOMY	N	No	
		Y	Yes	
		Y	Yes	
		U	Unknown	
		N	No	
		N	No	
		U	Unknown	
		Y	Yes	
Y	STOMA	N	No	
		N	No	
Y	BROVIAN LINE	N	No	
		N	No	
		N	No	
		N	No	
		Y	Yes	
		Y	Yes	
		U	Unknown	
		U	Unknown	
		N	No	
		U	Unknown	
		U	Unknown	
		U	Unknown	

		U	Unknown	
		Y	Yes	
		U	Unknown	
		N	No	
		U	Unknown	
		Y	Yes	
		N	No	
		N	No	
Y	CENTRAL VENOUS CATHETER FOR HA	Y	Yes	Y
		Y	Yes	
		N	No	
		Y	Yes	Y
		N	No	
		N	No	
		U	Unknown	
Y	NASOGASTRIC TUBE	Y	Yes	
		Y	Yes	
Y	NASOGASTRIC TUBE	Y	Yes	
		Y	Yes	
		N	No	
		Y	Yes	
		N	No	
		Y	Yes	
		N	No	
		N	No	
		Y	Yes	Y
		Y	Yes	
		U	Unknown	
		N	No	
		N	No	
Y	OROGASTRIC TUBE, UMBILICAL VEI	N	No	
		Y	Yes	
Y	OXYGEN MASK	Y	Yes	Y
		N	No	
		N	No	
		N	No	
		Y	Yes	
Y	TRACHEOSTOMY	N	No	
		Y	Yes	
		Y	Yes	
		U	Unknown	
		N	No	
		U	Unknown	
		Y	Yes	
Y	PIC LINE	N	No	
		N	No	
		N	No	
		N	No	
		Y	Yes	
		N	No	
		N	No	
		Y	Yes	
Y	TRACHEOSTOMY	Y	Yes	
		Y	Yes	
Y	TRACHEOSTOMY	N	No	

		N	No	
		Y	Yes	
		N	No	
Y	NASOGASTRIC TUBE	N	No	
		N	No	
		Y	Yes	
		N	No	
		N	No	
		N	No	
		N	No	
		N	No	
Y	RENAL LINE	N	No	
		N	No	
Y	TRACHEOSTOMY	N	No	
		Y	Yes	Y
		Y	Yes	Y
		Y	Yes	
		U	Unknown	
		Y	Yes	
		Y	Yes	
Y	UMBILICAL LINE	N	No	
Y	ENDOTRACHEAL TUBE	U	Unknown	
		N	No	
		N	No	
		N	No	
		N	No	
		N	No	
		Y	Yes	
Y	NASOGASTRIC TUBE	Y	Yes	
		Y	Yes	Y
		Y	Yes	Y
		Y	Yes	
		N	No	
		N	No	
		N	No	
		N	No	
		Y	Yes	
		Y	Yes	
		Y	Yes	
		Y	Yes	Y
		Y	Yes	
		N	No	
		Y	Yes	
		Y	Yes	
		Y	Yes	
Y	NASO GASTRIC TUBE	N	No	
		N	No	
		N	No	
Y	UMBILICAL LINE	N	No	
		N	No	
Y	UMBILICAL LINE	N	No	
		Y	Yes	
		N	No	
		N	No	
		Y	Yes	
		N	No	

		U	Unknown	
		N	No	
		N	No	
		N	No	
		N	No	
		Y	Yes	
		N	No	
		N	No	
		Y	Yes	Y
		Y	Yes	
		U	Unknown	
		N	No	
		U	Unknown	
		U	Unknown	
		Y	Yes	
		N	No	
		U	Unknown	
		Y	Yes	Y
		N	No	
		Y	Yes	
		U	Unknown	
Y	DJ STEMI	U	Unknown	
		U	Unknown	
		U	Unknown	
		U	Unknown	
		U	Unknown	
		U	Unknown	
		U	Unknown	
		N	No	
Y	VENTILATOR	Y	Yes	
		U	Unknown	
		N	No	
		U	Unknown	
		Y	Yes	
		N	No	
		Y	Yes	
Y	NASOGASTRIC TUBE	N	No	
		U	Unknown	
Y	VENTILATOR, NASOGASTRIC TUBE,	U	Unknown	
		U	Unknown	
		U	Unknown	
		U	Unknown	
		Y	Yes	
		U	Unknown	
Y	VENTILATOR	N	No	
		U	Unknown	
		U	Unknown	

		N	No	
		Y	Yes	Y
		Y	Yes	
		N	No	
		Y	Yes	
		U	Unknown	
		Y	Yes	
		U	Unknown	
		Y	Yes	Y
Y	NGT	N	No	
		N	No	
Y	VENTILATOR	Y	Yes	
		Y	Yes	
		U	Unknown	
		N	No	
		Y	Yes	
		N	No	
		Y	Yes	Y
		Y	Yes	
		N	No	
		U	Unknown	
		U	Unknown	
		N	No	
		Y	Yes	
		Y	Yes	Y
		N	No	
		N	No	
		Y	Yes	
		N	No	
		N	No	
		N	No	
		Y	Yes	Y
		U	Unknown	
		U	Unknown	
		U	Unknown	
		N	No	
		Y	Yes	
		N	No	
		U	Unknown	
		U	Unknown	
		U	Unknown	
		U	Unknown	
		Y	Yes	
		Y	Yes	
		U	Unknown	
Y	CVP	N	No	
		U	Unknown	

		Y	Yes	
		Y	Yes	
		N	No	
		U	Unknown	
		U	Unknown	
		N	No	
		Y	Yes	
		U	Unknown	
		Y	Yes	Y
Y	DJ_STENT	Y	Yes	Y
Y	CHEMOTHERAPY PORT	Y	Yes	
		U	Unknown	
		Y	Yes	
		U	Unknown	
		U	Unknown	
		Y	Yes	
		N	No	
		U	Unknown	
		N	No	
		U	Unknown	
		U	Unknown	
		Y	Yes	
		Y	Yes	
		N	No	
		N	No	
		Y	Yes	
		N	No	
Y	ICD	N	No	
Y	HD CATHETER	Y	Yes	
		N	No	
		N	No	
		N	No	
		Y	Yes	
		N	No	
		N	No	
		N	No	
		U	Unknown	
		U	Unknown	
		N	No	
		Y	Yes	
		Y	Yes	
		N	No	
		Y	Yes	Y
Y	UMBILICAL LINE	N	No	
		N	No	
		N	No	
		N	No	
		Y	Yes	
		N	No	
		U	Unknown	
		N	No	
		U	Unknown	
		Y	Yes	
		Y	Yes	

		N	No	
		U	Unknown	
		U	Unknown	
		Y	Yes	
		N	No	
		Y	Yes	
		Y	Yes	
		Y	Yes	
		N	No	
		U	Unknown	
		U	Unknown	
		U	Unknown	
		U	Unknown	
		U	Unknown	
		N	No	
		N	No	
Y	COLOSTOMY BAG	N	No	
		U	Unknown	
		Y	Yes	
		Y	Yes	
		U	Unknown	
		Y	Yes	
		N	No	
		N	No	
		N	No	
		N	No	
		N	No	
		N	No	
		U	Unknown	
		Y	Yes	
		Y	Yes	Y
		U	Unknown	
		Y	Yes	
		N	No	
		N	No	
		Y	Yes	Y
		N	No	
		N	No	
		Y	Yes	
		Y	Yes	
		U	Unknown	
		N	No	
		Y	Yes	Y
		N	No	
		N	No	
		N	No	
		Y	Yes	Y
		N	No	
		U	Unknown	
		U	Unknown	
		Y	Yes	
		U	Unknown	
		U	Unknown	
		U	Unknown	
		Y	Yes	Y
		N	No	

		U	Unknown	
		Y	Yes	
		Y	Yes	
		N	No	
		N	No	
		N	No	
		Y	Yes	
		N	No	
		N	No	
		N	No	
		Y	Yes	
		Y	Yes	Y
		Y	Yes	
		N	No	
		N	No	
		N	No	
		Y	Yes	Y
		Y	Yes	
		N	No	
		Y	Yes	
		Y	Yes	
		Y	Yes	
		Y	Yes	
		Y	Yes	
		Y	Yes	
		N	No	
		Y	Yes	Y
		N	No	
		Y	Yes	
		N	No	
		Y	Yes	
		U	Unknown	
		N	No	
		Y	Yes	
		Y	Yes	
		U	Unknown	
		U	Unknown	
		N	No	
		U	Unknown	
		U	Unknown	
Y	TRACHEOSTOMY	N	No	
		Y	Yes	
		Y	Yes	
		N	No	
		Y	Yes	
		N	No	
		U	Unknown	
		N	No	
		Y	Yes	
		Y	Yes	
		N	No	
		Y	Yes	
		N	No	
		N	No	
		N	No	
		N	No	

		Y	Yes	Y
		N	No	
		Y	Yes	Y
		Y	Yes	
		Y	Yes	
		N	No	
		N	No	
		U	Unknown	
		U	Unknown	
		U	Unknown	
		U	Unknown	
		U	Unknown	
		U	Unknown	
		U	Unknown	
		U	Unknown	
		U	Unknown	
		N	No	
		U	Unknown	
		N	No	
		Y	Yes	
		U	Unknown	
		Y	Yes	Y
		N	No	
		U	Unknown	
		U	Unknown	
		U	Unknown	
		U	Unknown	
		Y	Yes	
Y	DIALYSIS LINE	Y	Yes	
		U	Unknown	
		N	No	
		Y	Yes	
		Y	Yes	
		U	Unknown	
		N	No	
		N	No	
		Y	Yes	
		Y	Yes	
		N	No	
		N	No	
		Y	Yes	
		Y	Yes	Y
		Y	Yes	Y
		Y	Yes	
		U	Unknown	
		Y	Yes	
		N	No	
		Y	Yes	
		Y	Yes	
		Y	Yes	
		N	No	
		Y	Yes	

		N	No	
		Y	Yes	
		Y	Yes	Y
		Y	Yes	
		N	No	
		U	Unknown	
		N	No	
		N	No	
		N	No	
Y	STOMA BAG	Y	Yes	
		Y	Yes	Y
Y	DIALYSIS LINE	N	No	
		Y	Yes	
Y	CHEMO PORT	Y	Yes	
		Y	Yes	
		Y	Yes	
Y	NG TUBE	Y	Yes	
		Y	Yes	Y
		N	No	
		Y	Yes	
		Y	Yes	
		Y	Yes	
		N	No	
		N	No	
		U	Unknown	
		Y	Yes	Y
		U	Unknown	
		Y	Yes	Y
		U	Unknown	
		U	Unknown	
		U	Unknown	
		Y	Yes	
		Y	Yes	
		N	No	
		N	No	
		N	No	
		Y	Yes	
Y	NG TUBE	N	No	
		U	Unknown	
		U	Unknown	
		Y	Yes	
		Y	Yes	
		U	Unknown	
		U	Unknown	
Y	TRACHEOSTOMY	N	No	
		Y	Yes	
		Y	Yes	Y
		N	No	
		N	No	
Y	TRACHEAL TUBE	Y	Yes	
		N	No	
		Y	Yes	

		Y	Yes	
		U	Unknown	
		U	Unknown	
		N	No	
		Y	Yes	
		Y	Yes	
		U	Unknown	
		N	No	
		Y	Yes	
		Y	Yes	
		N	No	
		U	Unknown	
		N	No	
		U	Unknown	
Y	NGT TUBE	N	No	
		U	Unknown	
		Y	Yes	Y
		N	No	
		U	Unknown	
		N	No	
		N	No	
		U	Unknown	
		Y	Yes	
		U	Unknown	
		N	No	
		U	Unknown	
		U	Unknown	
		N	No	
		U	Unknown	
		N	No	
		N	No	
		Y	Yes	
		N	No	
		U	Unknown	
		N	No	
		Y	Yes	
		N	No	
		Y	Yes	
		U	Unknown	
		N	No	
		U	Unknown	
		U	Unknown	
		Y	Yes	
		U	Unknown	
		Y	Yes	
		N	No	
		Y	Yes	
		Y	Yes	
Y	NASOGASTRIC TUBE	U	Unknown	
		Y	Yes	

		N	No	
		N	No	
		Y	Yes	
		N	No	
		U	Unknown	
		U	Unknown	
		U	Unknown	
		Y	Yes	
		U	Unknown	
		Y	Yes	
		Y	Yes	
		N	No	
Y	NASO GASTRIC TUBE	N	No	
		N	No	
Y	NASO-GASTRIC TUBE	Y	Yes	
Y	VASCULAR CATHETER FEMORAL PORT	Y	Yes	
		Y	Yes	
		Y	Yes	
		Y	Yes	
		Y	Yes	
		Y	Yes	
		Y	Yes	Y
		Y	Yes	
		N	No	
		U	Unknown	
		Y	Yes	
		Y	Yes	Y
Y	COLOSTOMY BAG	N	No	
		N	No	
Y	RENAL LINE	Y	Yes	
		N	No	
		Y	Yes	
Y	CHEMO PORT	Y	Yes	
		Y	Yes	
Y	ENDOTRACHEAL TUBE	Y	Yes	Y
		Y	Yes	Y
		U	Unknown	
		N	No	
		N	No	
		U	Unknown	
		Y	Yes	Y
		N	No	
		Y	Yes	
Y	CHEMO PORT	Y	Yes	Y
		Y	Yes	Y
		N	No	
		N	No	
		N	No	
		N	No	
		U	Unknown	
		N	No	
Y	NASOGASTRIC TUBE	N	No	

		Y	Yes	
		N	No	
		Y	Yes	Y
		N	No	
		Y	Yes	Y
Y	AMBULICAL VENOUS LINE	N	No	
Y	INTERCOASTAL DRAIN	Y	Yes	
		Y	Yes	Y
Y	NASOGASTRIC TUBE	N	No	
		N	No	
Y	ET TUBE	N	No	
Y	GASTRIC TUBE	N	No	
		N	No	
		N	No	
		N	No	
		Y	Yes	
		N	No	
		Y	Yes	
		U	Unknown	
		U	Unknown	
		U	Unknown	
		N	No	
		N	No	
		N	No	
		N	No	
		Y	Yes	
		U	Unknown	

		Y	Yes	
		Y	Yes	
		U	Unknown	
		N	No	
		U	Unknown	
		Y	Yes	
		Y	Yes	
		U	Unknown	
		N	No	
		Y	Yes	Y
		Y	Yes	
		U	Unknown	
		Y	Yes	
		Y	Yes	
		Y	Yes	
		Y	Yes	
		Y	Yes	
Y	ILEOSTOMY BAG	N	No	
		Y	Yes	
		Y	Yes	
		N	No	
		N	No	
		U	Unknown	
		U	Unknown	
		Y	Yes	
Y	NASOGASTRIC TUBE	N	No	
		Y	Yes	Y
		N	No	
		N	No	
		Y	Yes	
		N	No	
		U	Unknown	
		Y	Yes	

		Y	Yes	
		N	No	
		U	Unknown	
Y	RENAL LINE	Y	Yes	
Y	CHEMO PORT	Y	Yes	Y
		Y	Yes	
		U	Unknown	
Y	NASO-GASTRIC TUBE	Y	Yes	
		Y	Yes	
Y	FOLEY CATHETER	Y	Yes	
Y	NASO-GASTRIC TUBE	N	No	
		Y	Yes	
		Y	Yes	
Y	DRAINAGE BAGS	Y	Yes	Y
Y	NASOGASTRIC TUBE	N	No	
		N	No	
Y	ILEOSTOMY	N	No	
		Y	Yes	Y
Y	STOMA BAG	N	No	
		U	Unknown	
Y		N	No	
		U	Unknown	
		U	Unknown	
		Y	Yes	
		Y	Yes	Y
		N	No	
		N	No	
		U	Unknown	
		Y	Yes	
		N	No	
		N	No	
		U	Unknown	
Y	NASOGASTRIC TUBE	Y	Yes	
		Y	Yes	
		Y	Yes	
Y	ILLOSTOMY BAG	N	No	
		N	No	
		Y	Yes	
		N	No	
Y	NASOGASTRIC TUBE	Y	Yes	
		Y	Yes	
		Y	Yes	
		Y	Yes	
		N	No	
Y	NG TUBE	N	No	
		Y	Yes	Y
		Y	Yes	

Y	NASOGASTRIC TUBE	Y	Yes	Y
		Y	Yes	
		N	No	
		N	No	
Y	TRACHIOSTOMY TUBE	Y	Yes	
Y	CHEMO PORT	Y	Yes	
		Y	Yes	
		Y	Yes	
		Y	Yes	
		Y	Yes	
		Y	Yes	
		U	Unknown	
		U	Unknown	
		N	No	
Y	STOMA	N	No	
		Y	Yes	
		N	No	
		N	No	
Y	NASOGASTRIC TUBE	Y	Yes	
		N	No	
Y	FEMORAL VEIN PERM. CATH.	Y	Yes	
Y	VENTILATED	N	No	
Y	ETT	Y	Yes	
		U	Unknown	
		N	No	
		N	No	
		N	No	
		N	No	
Y	NASOGASTRIC TUBE	Y	Yes	
		U	Unknown	
		N	No	
		U	Unknown	
		Y	Yes	
Y	NASOGASTRIC TUBE	Y	Yes	
		N	No	
		U	Unknown	
		N	No	
		N	No	
Y	NASOGASTRIC TUBE	N	No	
		Y	Yes	Y
		N	No	
		N	No	
		N	No	
		N	No	
		Y	Yes	
		N	No	
		N	No	
		U	Unknown	
		U	Unknown	
		N	No	
		N	No	
		U	Unknown	
		U	Unknown	

		N	No	
		U	Unknown	
		N	No	
		Y	Yes	Y
		N	No	
		Y	Yes	Y
Y	TRANCHEA TUBE	N	No	
		U	Unknown	
		N	No	
Y	NGTUBE	N	No	
		N	No	
		Y	Yes	
Y	NASOGASTRIC TUBE	N	No	
		Y	Yes	
		N	No	
		N	No	
		N	No	
		N	No	
		N	No	
		N	No	
		N	No	
		U	Unknown	
		Y	Yes	
		N	No	
Y	OROGASTRIC TUBE	Y	Yes	
Y	NASOGASTRIC TUBE	Y	Yes	
Y	NASOGASTRIC TUBE	N	No	
Y	COLOSTOMY BAG	N	No	
		N	No	
		Y	Yes	Y
		N	No	
		N	No	
Y	NGTUBE	Y	Yes	
Y	NG TUBE	Y	Yes	Y
Y	UMBILICAL LINE	N	No	
Y	NASOGASTRIC TUBE	N	No	
		N	No	
		N	No	
Y	COLOSTOMY BAG	N	No	
		Y	Yes	
		Y	Yes	
Y	NG TUBE	N	No	
Y	VASCULAR CATHETER	Y	Yes	
Y	NASOGASTRIC TUBE	N	No	

Y	NASOGASTRIC TUBE	Y	Yes	
		Y	Yes	Y
		N	No	
		Y	Yes	
Y	NASOGASTRIC TUBE	N	No	
		N	No	
Y	HICKMAN LINE	Y	Yes	
Y	NASOGASTRIC TUBE	N	No	
		N	No	
Y	ET	Y	Yes	
		N	No	
		N	No	
Y	UMBILICAL LINE	N	No	
		U	Unknown	
		U	Unknown	
		N	No	
		N	No	
		U	Unknown	
		N	No	
		N	No	
		N	No	
Y	NASOGASTRIC TUBE	Y	Yes	
		N	No	
		Y	Yes	
Y	NASGASTRIC TUBE	Y	Yes	Y
		Y	Yes	Y
		Y	Yes	
Y	UMBILICAL LINE	N	No	
		N	No	
		N	No	
		N	No	
		N	No	
		Y	Yes	
		Y	Yes	
Y	NG TUBE	Y	Yes	Y
Y	OG TUBE	Y	Yes	
		Y	Yes	
		Y	Yes	
		Y	Yes	
		Y	Yes	
Y	TRANCHE	Y	Yes	
Y	STERICATH	Y	Yes	
		N	No	
		N	No	
		Y	Yes	

Y	NASOGATRIC TUBE	N	No	
		N	No	
		N	No	
		N	No	
		N	No	
		N	No	
Y	NASO GASTRIC TUBE	N	No	
		Y	Yes	
Y	NASOGASTRIC TUBE	N	No	
		N	No	
Y	NASOGASTRIC TUBE	Y	Yes	
		Y	Yes	
		Y	Yes	
		Y	Yes	
		N	No	
		Y	Yes	
Y	NASOGASTRIC TUBE	N	No	
		N	No	
		Y	Yes	Y
Y	TRACHEOSTOMY	Y	Yes	
Y	ENDOTRACHEAL TUBE	N	No	
		Y	Yes	
		Y	Yes	
Y	NASO GASTRIC TUBE	N	No	
Y	NASOGASTRIC TUBE	Y	Yes	
Y	NASOGASTRIC TUBEINTERCOASTAL D	Y	Yes	
		N	No	
		N	No	
Y	TRANCHE	N	No	
		N	No	
		N	No	
		N	No	
		Y	Yes	
Y	STOMA BAG	N	No	
		Y	Yes	
		Y	Yes	
Y	DIALYSIS LINE	Y	Yes	
		Y	Yes	
		Y	Yes	
Y	UV AND UA LINE	Y	Yes	
Y	STOMA BAG	N	No	
		Y	Yes	Y
Y	NASOGASTRIC TUBE	N	No	
Y	NASOGASTRIC TUBE	N	No	
Y	TRACHEA LINE	N	No	
		N	No	
		Y	Yes	
		N	No	
Y	NG TUBE	Y	Yes	

		Y	Yes	Y
		Y	Yes	
Y	NASOGASTRIC TUBE	N	No	
		N	No	
Y	NASOGASTRIC TUBEINTER-COSTAL D	Y	Yes	
		N	No	
Y	NASOGASTRIC TUBE	Y	Yes	Y
Y	STOMA AND NGT	Y	Yes	
		Y	Yes	
		Y	Yes	
		Y	Yes	
		N	No	
		U	Unknown	
		Y	Yes	Y
		Y	Yes	
		N	No	
Y	NASOGASTRIC TUBEINTERCOASTAL D	Y	Yes	
		Y	Yes	
Y	NASO-GASTRIC TUBE	Y	Yes	Y
		N	No	
Y	NASOGASTRIC TUBE	Y	Yes	
Y	4 X PENCIL DRAINS	Y	Yes	Y
		U	Unknown	
		N	No	
Y	ET TUBE	Y	Yes	
		N	No	
Y	COLOSTOMY	Y	Yes	
		Y	Yes	
Y	NASOGASTRIC TUBE	Y	Yes	
		Y	Yes	Y
		N	No	
		N	No	
Y	NASOGASTRIC TUBE	Y	Yes	
		N	No	
Y	HICKMAN LINEJ-LINE NASOGASTRIC	Y	Yes	
		N	No	
		Y	Yes	
Y	COLOSTOMY BAG	N	No	
		N	No	
		Y	Yes	
Y	ET TUBE	N	No	
Y	NG TUBE	N	No	
		Y	Yes	
		N	No	
Y	NASO-GASTRIC TUBE	N	No	
Y	PICC LINE	Y	Yes	
Y	OGTUBE	N	No	
Y	NG TUBE	N	No	
		N	No	
Y	NASOGASTRIC TUBE	N	No	
Y	NG TUBE	Y	Yes	

		U	Unknown	
		Y	Yes	Y
		N	No	
		N	No	
		Y	Yes	Y
		Y	Yes	
		N	No	
		Y	Yes	Y
		N	No	
		Y	Yes	Y
		U	Unknown	
Y	NASOGASTRIC TUBE	N	No	
Y	NASOGASTRIC TUBE	Y	Yes	
		Y	Yes	
		Y	Yes	
		N	No	
		N	No	
Y	NASO-GASTRIC TUBE	N	No	
		N	No	
		N	No	
Y	ET TUBE	Y	Yes	
Y	ETUBE	N	No	
		Y	Yes	Y
		Y	Yes	Y
Y	NASO-GASTRIC TUBE	N	No	
		N	No	
		Y	Yes	Y
		U	Unknown	
		N	No	
Y	NASOGASTRIC TUBE	N	No	
		Y	Yes	
		U	Unknown	
Y	TRACHEOSTOMY	N	No	
		Y	Yes	
		N	No	
		Y	Yes	
		N	No	
		Y	Yes	Y
		U	Unknown	
		U	Unknown	
		N	No	
		N	No	
		N	No	
		N	No	
		Y	Yes	Y
		Y	Yes	

		U	Unknown	
		N	No	
		N	No	
		N	No	
		Y	Yes	
		Y	Yes	
		U	Unknown	
		Y	Yes	
		U	Unknown	
		Y	Yes	
Y	INTERCOSTAL DRAIN	N	No	
		Y	Yes	
		Y	Yes	
		Y	Yes	Y
		N	No	
		Y	Yes	
Y	NASOGASTRIC TUBE	N	No	
		Y	Yes	
Y	VENTILATOR	Y	Yes	Y
		N	No	
		N	No	
		Y	Yes	Y
Y	TRACHEOSTOMY	N	No	
		Y	Yes	Y
		Y	Yes	
		U	Unknown	
		N	No	
		U	Unknown	
		N	No	
		U	Unknown	
		U	Unknown	
		N	No	
		N	No	
		N	No	
		Y	Yes	
Y	NASOGASTRIC TUBE	N	No	
		Y	Yes	
		Y	Yes	
		Y	Yes	
		Y	Yes	
		N	No	
		U	Unknown	
		Y	Yes	
		Y	Yes	

Y	VENTILATOR	Y	Yes	
		Y	Yes	
		U	Unknown	
		Y	Yes	
Y	NASOGASTRIC TUBE	N	No	
		N	No	
		Y	Yes	
		Y	Yes	Y
		Y	Yes	Y
		Y	Yes	
		Y	Yes	
		U	Unknown	
		Y	Yes	Y
Y	UMBILICAL VEIN CATHETER	Y	Yes	
		Y	Yes	
		Y	Yes	
Y	UMBILICAL LINE	N	No	
Y	COLOSTOMY BAG	N	No	
Y	PIC LINE	N	No	
		N	No	
		N	No	
		U	Unknown	
Y	NG TUBE	N	No	
		Y	Yes	Y
Y	NG TUBE	Y	Yes	
		N	No	
		N	No	
Y	NASOGASTRIC TUBE	N	No	
		N	No	
		Y	Yes	
		N	No	
		U	Unknown	
Y	PERM CATHETER	Y	Yes	
		Y	Yes	
Y	NASOGASTRIC TUBE	N	No	
		U	Unknown	
		U	Unknown	
		N	No	
		N	No	
		U	Unknown	
		N	No	
		Y	Yes	
		N	No	
		N	No	
Y	NASO GASTRIC	Y	Yes	
		Y	Yes	
Y	TRACHEA TUBE	N	No	
		N	No	
		Y	Yes	
Y	NASOGASTRIC TUBE	Y	Yes	
		N	No	

Y	HDU CATHETER	Y	Yes	
		U	Unknown	
		U	Unknown	
		N	No	
		N	No	
Y	PERIFERAL LINE	Y	Yes	
Y	DIALYSIS CATHETER	N	No	
		Y	Yes	
		N	No	
		Y	Yes	Y
		Y	Yes	
		N	No	
		N	No	
		Y	Yes	
		Y	Yes	
		N	No	
		N	No	
		Y	Yes	
Y	HICKMAN LINE	Y	Yes	
		Y	Yes	
		Y	Yes	Y
		Y	Yes	
		Y	Yes	
Y	NG TUBE	Y	Yes	
		N	No	
		Y	Yes	
		N	No	
		U	Unknown	
		N	No	
		N	No	
Y	TRACHEOSTOMY	Y	Yes	Y
		Y	Yes	
Y	PICC LINE	N	No	
		Y	Yes	Y
		Y	Yes	
		Y	Yes	
Y	NASOGASTRIC TUBE	N	No	
Y	PIGTAIL	Y	Yes	
		Y	Yes	
Y	NASOGASTRIC TUBE	N	No	
		N	No	
		U	Unknown	
Y	NASOGASTRIC TUBE	U	Unknown	
		N	No	
		N	No	
		Y	Yes	
Y		U	Unknown	
Y	NASOGASTRIC TUBE	N	No	
		U	Unknown	
		Y	Yes	
		N	No	

		N	No	
		N	No	
Y	OROGASTRIC TUBE	N	No	
Y	NG TUBE	N	No	
Y	NGTUBE	Y	Yes	
		Y	Yes	Y
		N	No	
		N	No	
		N	No	
		Y	Yes	
		Y	Yes	
Y	IC DRAIN	N	No	
		N	No	
		Y	Yes	
Y	NGTUBE	N	No	
		N	No	
		N	No	
Y	NASOGASTRIC TUBE	Y	Yes	
Y	PIC LINE	N	No	
		Y	Yes	
Y	NG TUBE	N	No	
Y	LEFT FEMORAL CATHETER	Y	Yes	
		Y	Yes	
		Y	Yes	Y
Y	NASOGASTRIC TUBE	N	No	
		N	No	
		Y	Yes	
		N	No	
Y	NASO GASTRIC TUBE AND ENDO TRA	N	No	
Y	NASOGASTRIC TUBE	Y	Yes	
Y	GASTRIC TUBE	N	No	
Y	VASCATH	N	No	
		N	No	
		Y	Yes	
		Y	Yes	
Y	VASCULAR CATHETER	Y	Yes	
		N	No	
		Y	Yes	
		Y	Yes	
		Y	Yes	
Y	NASOGASTIC TUBE	N	No	
		Y	Yes	
		Y	Yes	
		N	No	
Y	NASOGASTRIC TUBE	Y	Yes	
Y	NG TUBE	N	No	
		Y	Yes	
		N	No	
		N	No	
		N	No	
		N	No	
		U	Unknown	

		U	Unknown	
		U	Unknown	
		U	Unknown	
		U	Unknown	
		U	Unknown	
		Y	Yes	
		Y	Yes	Y
		Y	Yes	Y
		Y	Yes	
		U	Unknown	
		U	Unknown	
		N	No	
		N	No	
		Y	Yes	
		Y	Yes	
		U	Unknown	
Y	NASOGASTRIC TUBE	Y	Yes	
		U	Unknown	
		Y	Yes	Y
		Y	Yes	
		U	Unknown	
		N	No	
Y	NG TUBE	N	No	
		U	Unknown	
		N	No	
		Y	Yes	
		U	Unknown	
Y	UMBILICAL VEIN CATHETER	Y	Yes	
		N	No	
		U	Unknown	
Y	NG TUBE	Y	Yes	
Y	STOMA	Y	Yes	
		U	Unknown	
		N	No	
		U	Unknown	
		Y	Yes	
		N	No	
Y	NG TUBE	Y	Yes	
		U	Unknown	
Y	N G TUBE	N	No	
		Y	Yes	Y
		Y	Yes	Y
		Y	Yes	
		U	Unknown	
		U	Unknown	
		Y	Yes	
		N	No	
		Y	Yes	
		U	Unknown	
		N	No	
Y	NASOGASTRIC TUBE	N	No	
		N	No	

		Y	Yes	
		U	Unknown	
		N	No	
Y	NASOGASTRIC TUBE	N	No	
		Y	Yes	
		N	No	
		N	No	
		Y	Yes	Y
		Y	Yes	Y
		U	Unknown	
		N	No	
Y	NG TUBE	N	No	
		Y	Yes	Y
		Y	Yes	
		U	Unknown	
		N	No	
		Y	Yes	
Y	HD CATHETER	Y	Yes	
		Y	Yes	
		N	No	
		U	Unknown	
		Y	Yes	
Y	NASOGASTRIC TUBE	N	No	
		N	No	
		Y	Yes	
		U	Unknown	
		U	Unknown	
		N	No	
		N	No	
		N	No	
		Y	Yes	
		Y	Yes	Y
Y	NG TUBE	N	No	
		N	No	
Y	NASOGASTRIC TUBE	Y	Yes	Y
Y	STENT	Y	Yes	
		Y	Yes	
		N	No	
		Y	Yes	
		U	Unknown	
		Y	Yes	
Y	NASOGASTRIC TUBE	Y	Yes	
		Y	Yes	
Y	STENT	Y	Yes	
		U	Unknown	
Y	NG TUBE	Y	Yes	Y

		U	Unknown	
		N	No	
Y	DIALYSIS CATHETER	Y	Yes	
		U	Unknown	
Y	OGT	Y	Yes	
Y	NASOGASTRIC TUBE	N	No	
Y	TRACHEOTOMY	N	No	
		Y	Yes	
		U	Unknown	
		N	No	
Y	NG TUBE	Y	Yes	
		Y	Yes	
		Y	Yes	
		Y	Yes	
		N	No	
Y	NGTUBE	Y	Yes	
		Y	Yes	
Y	STOMA	N	No	
		Y	Yes	Y
Y	NASOJEJUNAL TUBE	Y	Yes	
		N	No	
		N	No	
Y	NASOGASTRIC TUBE	U	Unknown	
		Y	Yes	
Y	NASO GASTRIC TUBE	Y	Yes	
Y	NGT	N	No	
Y	NASOGASTRIC TUBE	Y	Yes	
Y	NASO GASTRIC TUBE	Y	Yes	
		N	No	
		N	No	
Y	PIC LINE	N	No	
Y	NG TUBE	Y	Yes	
Y	TRACHEOSTOMY	Y	Yes	Y
		N	No	
		N	No	
		Y	Yes	
		Y	Yes	
		N	No	
		Y	Yes	Y
		Y	Yes	
		Y	Yes	
Y	COLOSTOMY BAG	Y	Yes	
		Y	Yes	
		U	Unknown	
Y	NG TUBE	Y	Yes	
Y	OG TUBE	Y	Yes	

		N	No	
Y	NG TUBE	N	No	
Y	DIALYSIS CATHETER	Y	Yes	
Y	NASOGASTRIC TUBE	N	No	
Y	NASOGASTRIC TUBE	Y	Yes	
Y	COLOSTOMY BAG	Y	Yes	
Y	NASOGASTRIC TUBE	Y	Yes	
Y	NASOGASTRIC TUBE	Y	Yes	
		Y	Yes	
		Y	Yes	
Y	NASOGASTRIC TUBE	N	No	
		N	No	
		Y	Yes	Y
Y	NASOGASTRIC	Y	Yes	
		N	No	
		N	No	
		U	Unknown	
		Y	Yes	
		N	No	
		Y	Yes	
		N	No	
		Y	Yes	
		Y	Yes	
Y	VENTILATIOR	N	No	
Y	NG TUBE	Y	Yes	
		Y	Yes	
		N	No	
		Y	Yes	
		U	Unknown	
		Y	Yes	
Y	ENDO TRACHEAL TUBE	N	No	
		Y	Yes	
		N	No	

		Y	Yes	Y
		Y	Yes	Y
		U	Unknown	
		N	No	
		U	Unknown	
		N	No	
		N	No	
		N	No	
		Y	Yes	
		Y	Yes	
		Y	Yes	
		N	No	
		N	No	
Y	NASOGASTRIC TUBE	Y	Yes	
Y	NASOGASTRC TUBE	Y	Yes	
		N	No	
		U	Unknown	
		N	No	
		Y	Yes	
Y	NG TUBE	Y	Yes	
		Y	Yes	
		Y	Yes	
		N	No	
		Y	Yes	Y
		Y	Yes	
		Y	Yes	Y
Y	COLOSTOMY BAG	Y	Yes	
		Y	Yes	
		Y	Yes	
		N	No	
		Y	Yes	
Y	NASOGASTRIC TUBE	N	No	
Y	COLOSTOMY BAG	Y	Yes	
Y	NASOGASTRIC TUBE	Y	Yes	
		Y	Yes	
		Y	Yes	
Y	ILEOSTOMY	Y	Yes	
		N	No	
		Y	Yes	

		N	No	
		Y	Yes	
Y	TRACHEOSTOMY	Y	Yes	
		N	No	
		Y	Yes	
		Y	Yes	
Y	NG TUBE, ET TUBE	N	No	
		Y	Yes	
Y	UMBILICAL VEIN CATHETER	N	No	
		N	No	
		N	No	
		N	No	
		N	No	
		U	Unknown	
		Y	Yes	
		N	No	
		Y	Yes	
		U	Unknown	
		N	No	
		Y	Yes	
		N	No	
Y	NGT	N	No	
		Y	Yes	
		N	No	
Y	NASOGASTRIC TUBE	N	No	
Y	NASOGASTRIC TUBE	N	No	
Y	NASOGASTRIC TUBE	N	No	
Y	NASOGASTRIC TUBE	Y	Yes	
Y	VASCULAR CATHETER	Y	Yes	
		N	No	
		Y	Yes	
		Y	Yes	Y
		Y	Yes	
		U	Unknown	
		Y	Yes	
Y	NG TUBE	Y	Yes	
		N	No	
		Y	Yes	
Y	ETT	Y	Yes	
		N	No	
Y	NG TUBE	N	No	

		Y	Yes	
		U	Unknown	
		U	Unknown	
		Y	Yes	
		U	Unknown	
		Y	Yes	
Y	NASOGASTRIC TUBE	N	No	
Y	COLOSTOMY	N	No	
Y	COLOSTOMY BAG	N	No	
Y	NASOGASTRIC TUBE	N	No	
Y	NASOGASTRIC TUBE	N	No	
Y	NASOGASTRIC TUBE	N	No	
Y	NASOGASTRIC TUBE	Y	Yes	
		Y	Yes	
Y	NASOGASTRIC TUBE	N	No	
		N	No	
		N	No	
Y	NG TUBE	N	No	
Y	NG TUBE	Y	Yes	
		Y	Yes	Y
Y	NG TUBE	Y	Yes	
Y	NASOGASTRIC TUBE	Y	Yes	
		N	No	
		N	No	
		N	No	
Y	NG TUBE	Y	Yes	
		N	No	
		N	No	
		N	No	
		Y	Yes	
Y	JEJENOSTOMY FEEDING TUBE	N	No	
		N	No	
		Y	Yes	
Y	ET TUBE	Y	Yes	
		Y	Yes	
		U	Unknown	
		U	Unknown	
		U	Unknown	
		U	Unknown	
		U	Unknown	
		N	No	
		Y	Yes	Y
		N	No	
		N	No	
		U	Unknown	
		N	No	
Y	PENCIL DRAIN	Y	Yes	

Y	NASOGASTRIC TUBE AND ENDOTRACH	Y	Yes	
		Y	Yes	
		N	No	
Y	TRACHEOSTOMY	N	No	
		U	Unknown	
		Y	Yes	
		U	Unknown	
		U	Unknown	
		N	No	
		N	No	
		N	No	
		U	Unknown	
		U	Unknown	
		N	No	
Y	NG TUBE	N	No	
		U	Unknown	
		Y	Yes	
		Y	Yes	
		N	No	
		Y	Yes	
Y	NG TUBE	Y	Yes	
		U	Unknown	
		N	No	
Y	NGTUBE	N	No	
		Y	Yes	Y
		Y	Yes	Y
		N	No	
		Y	Yes	
		Y	Yes	Y
Y	NASOGASTRIC TUBE	Y	Yes	Y
		N	No	
		N	No	
		U	Unknown	
		Y	Yes	
		N	No	
		Y	Yes	
Y	COLOSTOMY BAG	U	Unknown	
		Y	Yes	
Y	INTUBATION	Y	Yes	
Y	HEMODIALYSIS CATHETER	Y	Yes	
		Y	Yes	
		N	No	
		Y	Yes	
		N	No	
Y	NASOGASTRIC TUBE	N	No	
		Y	Yes	
		Y	Yes	
		Y	Yes	

Y	PEG FEEDING	Y	Yes	
		Y	Yes	
		N	No	
		Y	Yes	Y
		Y	Yes	
		Y	Yes	
		N	No	
Y	UMBILICAL VEIN LINE	N	No	
Y	NASOGASTRIC TUBE	U	Unknown	
		Y	Yes	
		Y	Yes	
Y	NASOGASTRIC TUBE	N	No	
		U	Unknown	
Y	NASOGASTIRC TUBE	N	No	
		Y	Yes	Y
		Y	Yes	
Y	NG TUBE	N	No	
Y	NG TUBE	Y	Yes	

		Y	Yes
Y	TRACHEOSTOMY	Y	Yes
Y	ILEOSTOMY BAG	N	No
Y	NASO-GASTRIC TUBE	Y	Yes

		Y	Yes	
		N	No	
		Y	Yes	
		N	No	
Y	ENDOTRACHEAL TUBE	Y	Yes	
		N	No	
		N	No	
		Y	Yes	
		Y	Yes	
Y	HEMODIALYSIS CATHETER	Y	Yes	
Y	NASOGASTRIC TUBE	N	No	
Y	NASOGASTRIC TUBE	N	No	
Y	RENAL LINE AND THE TRACEOSTOMY	N	No	
Y	NASOGASTRIC TUBE	N	No	
		Y	Yes	Y
		N	No	
		Y	Yes	
		N	No	
		N	No	
		Y	Yes	Y
Y	CHEMO PORT	Y	Yes	
Y	NG TUBE	Y	Yes	Y
		Y	Yes	
		Y	Yes	
		Y	Yes	
		N	No	
Y	NASOGASTRIC CUBE	N	No	
Y	NGT	Y	Yes	
Y	NG TUBE	Y	Yes	
Y	NG TUBE	Y	Yes	
Y	HEMODIALYSIS RENAL LINE	Y	Yes	
Y	NASOGASTRIC TUBE	N	No	
		Y	Yes	
		N	No	
Y	UMBILICAL LINE	N	No	
		N	No	
		Y	Yes	
		Y	Yes	Y
Y	NGT	N	No	
		Y	Yes	
		Y	Yes	
		Y	Yes	
		N	No	
		Y	Yes	Y
		Y	Yes	
		N	No	
		N	No	
Y	NASOGASTRIC TUBE	N	No	
		N	No	

		Y	Yes	
		Y	Yes	
		Y	Yes	
Y	OGTUBE	Y	Yes	
Y	OGTUBE	Y	Yes	
		N	No	
		U	Unknown	
Y	NASOGASTRIC TUBE	N	No	
		N	No	
		U	Unknown	
Y	NASOGASTRIC TUBE	Y	Yes	
		N	No	
		N	No	
		Y	Yes	
		N	No	
		N	No	
Y	NASOGASTRIC TUBE	Y	Yes	
		U	Unknown	
		U	Unknown	
Y	PORT	Y	Yes	
		N	No	
		U	Unknown	
		N	No	
		N	No	
		Y	Yes	Y
		Y	Yes	
		U	Unknown	
		Y	Yes	
		Y	Yes	
		Y	Yes	
		N	No	
		Y	Yes	
		N	No	
		N	No	
		U	Unknown	
		U	Unknown	
		Y	Yes	
		N	No	
		U	Unknown	
		Y	Yes	

Y	NGT	Y	Yes
		U	Unknown
Y	NG TUBE	N	No
		Y	Yes
		N	No
		N	No
		Y	Yes
Y	NASOGASTRIC TUBE	Y	Yes
		N	No
Y	NASOGASTRIC TUBE	N	No
		U	Unknown
		Y	Yes
Y	TRACHEOSTOMY TUBE	N	No
Y	NGT	N	No
		U	Unknown
Y	NASOGASTRIC TUBE	Y	Yes
		N	No
Y	NASOGASTRIC TUBE	Y	Yes
Y	NASOGASTRIC TUBE	N	No
Y	NASOGASTRIC TUBE,TRACHEOSTOMY	Y	Yes
		Y	Yes
Y	NASOGASTRIC TUBE	Y	Yes
		N	No
Y	NASOGASRIC TUBE	N	No
		N	No
Y	NASOGASTRIC TUBE	Y	Yes
		Y	Yes
Y	NASOGASTRIC TUBE	Y	Yes
		U	Unknown
		Y	Yes
		U	Unknown
		N	No
Y	NASOGASTRIC TUBE	Y	Yes
		N	No
		N	No
		Y	Yes
		U	Unknown
		N	No
Y	NASOGASTRIC TUBE	Y	Yes
		Y	Yes
Y	NASOGASTIC TUBE	N	No
Y	TRACHEOSTOMY AND NGT - SETUP 8	N	No
Y	NASOGASTRIC TUBE	N	No
		Y	Yes
		Y	Yes

		Y	Yes	
		U	Unknown	
		N	No	
		Y	Yes	
		U	Unknown	
		N	No	
		Y	Yes	
		Y	Yes	Y
Y	NASOGASTRIC TUBE	Y	Yes	
		Y	Yes	
		Y	Yes	
		U	Unknown	
		N	No	
		Y	Yes	
		Y	Yes	
Y	OGTUBE	Y	Yes	
Y	NG TUBE	Y	Yes	
Y	COLOSTOMY	Y	Yes	
		Y	Yes	
		Y	Yes	
		Y	Yes	
		Y	Yes	
		U	Unknown	
		N	No	
Y	NG TUBE	Y	Yes	
		N	No	
		N	No	
		Y	Yes	Y
		Y	Yes	
Y	PD CATHETER	Y	Yes	
		U	Unknown	
		Y	Yes	Y
		U	Unknown	
		N	No	
		U	Unknown	
		N	No	
		Y	Yes	
Y	COLOSTOMY	Y	Yes	
		Y	Yes	
		N	No	
		Y	Yes	
Y	NASOGASTRIC TUBE	Y	Yes	
		Y	Yes	
Y	ENDOTRACHEAL TUBE	Y	Yes	
		N	No	
		N	No	
Y	NASOGASTRIC TUBE	N	No	
Y	NASOGASTRIC TUBE	N	No	
Y	NASOGASTRIC TUBE	N	No	

		Y	Yes	Y
Y	NASOGASTRIC TUBE	N	No	
		Y	Yes	
		Y	Yes	Y
		U	Unknown	
		Y	Yes	
		N	No	
		Y	Yes	Y
Y	NASOGASTRIC TUBE	Y	Yes	
		Y	Yes	
		N	No	
		U	Unknown	
		Y	Yes	
		Y	Yes	
Y	NASOGASTRIC TUBE	Y	Yes	
		N	No	
		Y	Yes	
		Y	Yes	Y
		N	No	
Y	NG TUBE	U	Unknown	
Y	PIGTAIL	Y	Yes	
		Y	Yes	
		N	No	
		N	No	
Y	RENAL LINE	Y	Yes	
		N	No	
Y	NASOGASTRIC TUBE	N	No	
Y	NASOGASTRIC TUBE	N	No	
Y	NASOGASTRIC TUBE	N	No	
Y	NASOGASTRIC TUBE	N	No	
Y	ENDOTRACHEAL TUBE	Y	Yes	
		Y	Yes	
Y	ENDOTRACHEAL TUBE,NASOGASTRIC	Y	Yes	
		Y	Yes	
		N	No	
		Y	Yes	
Y	NASOGASTRIC TUBE	N	No	
		Y	Yes	Y
Y	NASOGASRIC	Y	Yes	
		N	No	
		U	Unknown	
		Y	Yes	Y
Y	NASOGASTRIC TUBE	N	No	
Y	NASOGASTRIC TUBE	Y	Yes	
		N	No	
		N	No	
Y	NASOGASTRIC TUBE	Y	Yes	

Y	STENT	Y	Yes	
		N	No	
Y	ENDOTRACHEAL TUBE AND NASOGAS	Y	Yes	
Y	QUINTON LINE	Y	Yes	
		U	Unknown	
		Y	Yes	Y
Y	COOKS CATHETER	Y	Yes	
Y	NG TUBE	Y	Yes	
Y	COLOSTOMY BAG	Y	Yes	
		Y	Yes	
Y	NG TUBE	N	No	
Y	HD CATHETER	Y	Yes	
		N	No	
		Y	Yes	Y
Y	TRACHEOSTOMY	Y	Yes	Y
		N	No	
		Y	Yes	
		Y	Yes	
Y	NASOGASTRIC TUBE	N	No	
		N	No	
		Y	Yes	Y
Y	NASOGASTRIC TUBE	N	No	
		N	No	
Y	OROGASTRIC TUBE	Y	Yes	
		Y	Yes	
		Y	Yes	
		Y	Yes	Y
		Y	Yes	
Y	TRACHEOSTOMY	N	No	
		N	No	
		Y	Yes	
Y	NASOGASTRIC TUBE	Y	Yes	
		Y	Yes	
		Y	Yes	Y
		Y	Yes	
		N	No	
Y	UMBILICAL VEIN CORD	N	No	
		U	Unknown	
		N	No	

Y	NASOGASTRIC TUBE	U	Unknown	
		U	Unknown	
		N	No	
		N	No	
		N	No	
		N	No	
Y	NASOGASTRIC TUBE	Y	Yes	
Y	NASOGASTRIC TUBE	N	No	
		Y	Yes	
		Y	Yes	Y
		N	No	
Y	IC DRAIN	Y	Yes	
Y	NASOGASTRIC TUBE	Y	Yes	
		Y	Yes	
		Y	Yes	Y
		N	No	
		Y	Yes	
		Y	Yes	
		N	No	
		Y	Yes	Y
		Y	Yes	
Y	HD LINE	Y	Yes	Y
Y	NASOGASTRIC TUBE	N	No	
		N	No	
		Y	Yes	
Y	OROGASTRIC TUBE	Y	Yes	
		Y	Yes	Y
Y	ILEOSTOMY	N	No	
Y	NASOGASTRIC TUBE	N	No	
Y	NASOGASTRIC TUBE	N	No	
		Y	Yes	Y
Y	OROGASRIC TUBE	Y	Yes	
Y	NASOGASTRIC TUBE	Y	Yes	
		N	No	
Y	NASOGASTRIC TUBE	N	No	

		Y	Yes	
Y	NASOGASTRIC TUBE	N	No	
		N	No	
		Y	Yes	
		Y	Yes	
Y	OROGASTRIC TUBE	Y	Yes	
Y	NASOGASTRIC TUBE	N	No	
Y	NASOGASTRIC TUBE	N	No	
Y	NASOGASTRIC TUBE	Y	Yes	
		N	No	
		Y	Yes	
		N	No	
		U	Unknown	
		N	No	
Y	OROGASTRIC TUBE	Y	Yes	
Y	NASOGASTRIC TUBE	N	No	
		Y	Yes	
Y	UMBILICAL LINE 22/08/20 NGT ON	N	No	
		U	Unknown	
		N	No	
Y	PEG	N	No	
		N	No	
		N	No	
Y	NASOGASTRIC TUBE	N	No	
		Y	Yes	
		Y	Yes	
		Y	Yes	
		N	No	
		N	No	
		Y	Yes	Y
		N	No	
Y	NG TUBE	N	No	
		N	No	
		N	No	
Y	NASOGASTRIC TUBE	N	No	
Y	NG TUBE	N	No	
		U	Unknown	
		Y	Yes	
Y	NASOGASTRIC TUBE	Y	Yes	

		N	No	
		U	Unknown	
		Y	Yes	Y
		U	Unknown	
		Y	Yes	
		N	No	
Y	NASOGASTRIC TUBE	U	Unknown	
		U	Unknown	
		Y	Yes	
Y	NASOGASTRIC TUBE	Y	Yes	
		U	Unknown	
		N	No	
Y	NASOGASTRIC TUBE	Y	Yes	
Y	PACEMAKER	Y	Yes	
		Y	Yes	
Y	ET TUBE	Y	Yes	
		N	No	
Y	CENTRAL AND ARTERIAL LINE AND	Y	Yes	Y
		Y	Yes	
		N	No	
		N	No	
		Y	Yes	
Y	NG TUBE	N	No	
Y	TRACHEOSTOMY AND NGT INSERTED	N	No	
		N	No	
		Y	Yes	Y
		N	No	
		U	Unknown	
		Y	Yes	Y
		U	Unknown	
		N	No	
		Y	Yes	
Y	NASOGASTRIC TUBE	N	No	
		N	No	
Y	TENKOFF FOR DIALYSIS	Y	Yes	Y
		N	No	
		N	No	
		Y	Yes	
		Y	Yes	
		U	Unknown	
Y	E T TUBE	Y	Yes	

		Y	Yes	
		Y	Yes	
		U	Unknown	
Y	NASOGASTRIC TUBE	N	No	
Y	NASOGASTRIC TUBE	N	No	
Y	NCPAP	Y	Yes	
Y	BROVIAC	N	No	
Y	E T TUBE, TRACHEOSTOMY	Y	Yes	
		N	No	
		N	No	
		U	Unknown	
		U	Unknown	
		N	No	
Y	OGT	Y	Yes	
		N	No	
		N	No	
		U	Unknown	
		Y	Yes	Y
		N	No	
		N	No	
		N	No	
		N	No	
		N	No	
		Y	Yes	
		Y	Yes	
Y	ET TUBE	N	No	
		Y	Yes	Y
Y	NASOGASTRIC TUBE	Y	Yes	
		N	No	
		N	No	
Y	BROVIAC	N	No	
		N	No	
		Y	Yes	
Y	NASOGASRIC TUBE	N	No	
		U	Unknown	
		U	Unknown	
Y	NCPAP	Y	Yes	
Y	NG TUBE	Y	Yes	
		Y	Yes	
		Y	Yes	
Y	NASOGASTRIC TUBE	N	No	

		Y	Yes	Y
Y	BROVIAC	Y	Yes	
Y	NASOGASRIC TUBE	Y	Yes	
Y	NG TUBE	N	No	
		Y	Yes	
		Y	Yes	
		Y	Yes	Y
		N	No	
Y	OROGASTRIC TUBE	Y	Yes	
		Y	Yes	Y
		N	No	
Y	NASOGASTRIC TUBE	N	No	
		N	No	
		U	Unknown	
		Y	Yes	
		U	Unknown	
		Y	Yes	Y
		Y	Yes	
		U	Unknown	
		U	Unknown	
		N	No	
Y	TANCOFF	U	Unknown	
		Y	Yes	Y
		Y	Yes	Y
Y	NG TUBE	N	No	
		Y	Yes	
		N	No	
		U	Unknown	
		Y	Yes	
Y	OROGASTRIC TUBE	Y	Yes	
Y	FOLEY CATHETER	Y	Yes	
		N	No	

		N	No	
		Y	Yes	
		N	No	
		N	No	
		N	No	
		N	No	
		Y	Yes	
Y	IC DRAIN	Y	Yes	
Y	NASOGASTRIC TUBE	Y	Yes	
		Y	Yes	
		N	No	
Y		N	No	
Y	NGT	N	No	
		Y	Yes	Y
Y	RENAL LINE	Y	Yes	
		N	No	
Y	NGT	Y	Yes	
		N	No	
		N	No	
		Y	Yes	
Y	NASOGASTRIC TUBE	Y	Yes	
		U	Unknown	
		N	No	
		N	No	
Y	NASOGASTRIC TUBE	Y	Yes	
		Y	Yes	
Y	TRACHEOSTOMY TUBE	Y	Yes	Y
		N	No	
		U	Unknown	
		Y	Yes	
Y	OROGASTRIC TUBE	Y	Yes	
		Y	Yes	
		U	Unknown	
		U	Unknown	
		N	No	
		Y	Yes	
Y	VASCULAR CATHETER	Y	Yes	
		N	No	
		U	Unknown	
Y	COLOSTOMY BAG	N	No	
		N	No	
		N	No	

		Y	Yes	
Y	NG TUBE	U	Unknown	
Y	TRACHEOSTOMY	Y	Yes	Y
Y	NASOGASTRIC TUBE	N	No	
Y	NASOGASTRIC TUBE	N	No	
		N	No	
		Y	Yes	
		Y	Yes	
		Y	Yes	
Y	VASCULAR CATHETER	Y	Yes	
Y	NASOGASTRIC TUBE	Y	Yes	
		Y	Yes	
		Y	Yes	
		N	No	
		Y	Yes	Y
		N	No	
		Y	Yes	
		N	No	
		Y	Yes	
		N	No	
		N	No	
		N	No	
Y	TRACHEOSTOMY TUBE	Y	Yes	
		N	No	
		N	No	
		N	No	
		N	No	
Y	NGT	N	No	
Y	NASOGASTRIC TUBE	N	No	
		Y	Yes	Y
		N	No	
		Y	Yes	
		Y	Yes	
		Y	Yes	Y
Y	NASOGASTRIC TUBE	Y	Yes	
		N	No	
		U	Unknown	
		N	No	

		Y	Yes	
		N	No	
		Y	Yes	
Y	E T TUBE , N G TUBE	Y	Yes	
		U	Unknown	
		U	Unknown	
		Y	Yes	
		Y	Yes	
		Y	Yes	Y
Y	TRACHEOSTOMY 24/10/20 OUT 1/1/	N	No	
Y	TRACHEOSTOMY	N	No	
Y	RENAL LINE	Y	Yes	
		N	No	
		N	No	
Y	NASOGASTRIC TUBE	Y	Yes	
Y	NG TUBE	N	No	
Y	NASOGASTRIC TUBE	N	No	
		N	No	
		Y	Yes	Y
Y	NASOGASTRIC TUBE	Y	Yes	
Y	NASOGASTRIC TUBE	Y	Yes	
Y	COLOSTOMY BAG	N	No	
		N	No	
		N	No	
Y	NASOGASTRIC TUBE	N	No	
		N	No	
Y	NASOGASTRIC TUBE	Y	Yes	
		N	No	
		N	No	
Y	NASOGASTRIC TUBE	Y	Yes	Y
		Y	Yes	
		Y	Yes	
		N	No	
		N	No	
Y	NASOGASTRIC TUBE	N	No	
Y	NASOGASTRIC TUBE	N	No	
		N	No	
Y	NASOGASTRIC TUBE	Y	Yes	

Y	PICC LINE	N	No	
Y	ET TUBE , NG TUBE	N	No	
Y	STENT	Y	Yes	
		N	No	
		N	No	
Y	NASOGASTRIC TUBE	N	No	
Y	NASOGASTRIC TUBE	N	No	
		N	No	
		N	No	
		N	No	
		Y	Yes	
		N	No	
		Y	Yes	
		N	No	
Y	NASOGASTRIC TUBE	N	No	
Y	NASOGASTRIC TUBE	N	No	
		Y	Yes	
Y	ENDO TRACHEAL TUBE	N	No	
Y	E T TUBE	N	No	
		U	Unknown	
		N	No	
		N	No	
		N	No	
		N	No	
		N	No	
		N	No	
		N	No	
		Y	Yes	
		N	No	
		U	Unknown	
Y	ENDOTRACHEAL TUBE	Y	Yes	Y
		N	No	
Y	NASOGASTRIC TUBE	Y	Yes	

		Y	Yes	
		Y	Yes	Y
Y	ET TUBE AND NG TUBE	N	No	
		Y	Yes	
		U	Unknown	
		Y	Yes	Y
Y	NG TUBE	N	No	
		N	No	
Y	NASOGASTRIC TUBE	N	No	
		N	No	
Y	NASOGASTRIC TUBE	N	No	
Y	NASOGASTRIC TUBE	U	Unknown	
		Y	Yes	
		Y	Yes	
		U	Unknown	
		Y	Yes	
		U	Unknown	
		N	No	
Y	NASOGASTRIC TUBE	N	No	
		Y	Yes	
		N	No	
		Y	Yes	Y
		Y	Yes	
		N	No	
		N	No	
Y	NASOGASTRIC TUBE	Y	Yes	Y
Y	STOMA	N	No	
		N	No	
		U	Unknown	
Y	NG TUBE	N	No	
		Y	Yes	Y
		N	No	
		U	Unknown	

		N	No	
		Y	Yes	
Y	VENTILATOR	Y	Yes	
		N	No	
		N	No	
		Y	Yes	
		N	No	
Y	NGT TUBE	Y	Yes	Y
		U	Unknown	
		N	No	
		U	Unknown	
Y	OROGASTRIC TUBE	Y	Yes	
		Y	Yes	Y
		Y	Yes	
		Y	Yes	
		Y	Yes	
Y	NG TUBE	N	No	
Y	NASOGASTRIC TUBE	N	No	
Y	NASOGASTRIC TUBE,INTRAVENOUS T	N	No	
		U	Unknown	
		Y	Yes	Y
		N	No	
		Y	Yes	Y
Y	OROGASTRIC TUBE	Y	Yes	
		N	No	
Y	CHEMO PORT	Y	Yes	
		Y	Yes	
		Y	Yes	

		Y	Yes	Y
		N	No	
		Y	Yes	
		U	Unknown	
		Y	Yes	Y
		Y	Yes	
Y	NG TUBE	N	No	
		N	No	
Y	NASOGASTRIC TUBE	N	No	
Y	OROGASTRIC TUBE	N	No	
Y	NG TUBE	N	No	
		Y	Yes	
		Y	Yes	
		Y	Yes	
		N	No	
		N	No	
		Y	Yes	
		Y	Yes	Y
Y	NG TUBE	Y	Yes	Y
		Y	Yes	
		Y	Yes	
Y	NASOGASTRIC TUBE	Y	Yes	
		Y	Yes	
Y	QUINTINE LINE	N	No	
		Y	Yes	Y
		Y	Yes	
		N	No	
		N	No	
		Y	Yes	Y

		Y	Yes	
		N	No	
Y	OROGASTRIC TUBE	Y	Yes	
		Y	Yes	
		N	No	
		N	No	
Y	OROGASTRIC TUBE	N	No	
		Y	Yes	
Y	NASOGASTRIC TUBE	Y	Yes	Y
		U	Unknown	
		Y	Yes	
Y	NCPAP	Y	Yes	
		U	Unknown	
		Y	Yes	
		N	No	
		Y	Yes	Y
		Y	Yes	Y
		Y	Yes	
		Y	Yes	Y
		U	Unknown	
		U	Unknown	
		N	No	

		Y	Yes	Y
Y	NG TUBE	N	No	
		N	No	
		U	Unknown	
		U	Unknown	
Y	TRACHEOSTOMY	Y	Yes	
		Y	Yes	
		Y	Yes	
Y	NASOGASRIC	N	No	
		U	Unknown	
		U	Unknown	
		N	No	
		Y	Yes	
Y	TRACHEOSTOMY AND NASOGASTRIC T	Y	Yes	Y
		N	No	

respiratory_diseases	renal	cvs	malignancy	other_risk
				Y
		Y		
				Y
				Y
		Y		
			Y	
			Y	Y
	Y			
				Y
			Y	
				Y
	Y			Y
				Y
			Y	
				Y
	Y			
				Y
			Y	
			Y	
		Y		
			Y	
			Y	
			Y	

Y			
		Y	
Y			
			Y
			Y
		Y	
		Y	
			Y
			Y
	Y		
Y			Y
		Y	
		Y	
	Y		
		Y	
		Y	
		Y	
		Y	
		Y	
		Y	
			Y
			Y
Y		Y	
			Y
		Y	
Y			

			Y
		Y	
		Y	
		Y	
		Y	
		Y	
Y			
			Y
			Y
		Y	
			Y
		Y	
Y		Y	
	Y		Y
			Y
		Y	
			Y
		Y	
		Y	
			Y
			Y
			Y
			Y

	Y	
	Y	
		Y
Y		
Y		Y
	Y	
		Y
	Y	
		Y
	Y	
	Y	
	Y	
		Y
		Y
		Y
		Y
		Y
	Y	
		Y
		Y
Y		
		Y
	Y	
	Y	
		Y
		Y

		Y
Y		
		Y
		Y
		Y
Y		
	Y	
	Y	
	Y	
	Y	
		Y
	Y	
		Y
	Y	
	Y	
	Y	
		Y
		Y
	Y	
	Y	Y
	Y	
	Y	
		Y

			Y
		Y	
Y			
			Y
		Y	
		Y	
			Y
			Y
		Y	
Y	Y		Y
		Y	
			Y
		Y	Y
		Y	
		Y	
Y			Y
		Y	
Y			
		Y	
		Y	
		Y	
Y			

Y

Y

Y
Y
Y
Y

Y Y

Y

Y

Y
Y
Y

Y

Y

Y

Y

			Y
			Y
		Y	Y
Y			Y
Y			Y
			Y
	Y		Y
			Y
			Y
			Y
			Y
Y			Y
			Y
		Y	
		Y	Y
		Y	
			Y
		Y	
Y			
			Y
		Y	
Y			
	Y		Y
			Y
		Y	
			Y
		Y	

			Y
			Y
		Y	
	Y		
		Y	
		Y	
Y			
Y			
			Y
		Y	
			Y
			Y
			Y
Y			Y
			Y
			Y
Y			
Y		Y	
			Y
			Y
			Y
		Y	

Y
Y

Y
Y
Y
Y
Y
Y
Y
Y
Y
Y
Y
Y
Y
Y
Y
Y
Y
Y
Y
Y

			Y
			Y
			Y
		Y	
			Y
			Y
	Y		
		Y	
		Y	
Y			
		Y	
			Y
		Y	
			Y
		Y	
			Y
			Y
		Y	
			Y
		Y	
	Y		
	Y		
			Y
			Y
	Y		
Y			
			Y
			Y

	Y	
		Y
		Y
	Y	
		Y
		Y
		Y
		Y
		Y
Y		Y
Y		
	Y	
		Y
Y		
		Y
	Y	
		Y
		Y
	Y	

	Y	
	Y	
Y		
		Y
Y		
		Y
		Y
		Y
		Y
	Y	
		Y
Y		
		Y
		Y
		Y
	Y	
	Y	
Y		
	Y	
	Y	Y
		Y
	Y	

Y	Y		
		Y	
	Y	Y	
			Y
Y			Y
Y			
			Y
			Y
Y			Y
			Y
		Y	
Y			
		Y	
			Y
			Y
		Y	
		Y	
Y			
			Y

Y

Y

Y

Y

Y Y

Y

Y

Y

Y
Y
Y Y
Y
Y
Y
Y
Y
Y
Y
Y
Y
Y

	Y	
		Y
	Y	
		Y
	Y	Y
		Y
		Y
	Y	
	Y	
		Y
Y		
		Y
	Y	
		Y
	Y	
	Y	
		Y
		Y

		Y
	Y	
		Y
Y		
		Y
	Y	
	Y	Y
Y		Y
	Y	
		Y
	Y	
	Y	
	Y	
		Y
		Y
	Y	

			Y
		Y	
		Y	
		Y	
Y			
		Y	
	Y		
		Y	
			Y
Y			
		Y	
		Y	
			Y
Y			
			Y
	Y		
		Y	
			Y
Y			
		Y	
			Y
Y			
			Y
Y			

			Y
	Y		
			Y
		Y	
Y			
			Y
Y			Y
			Y
		Y	
Y		Y	
			Y
			Y
	Y		
Y			
Y			Y
		Y	
	Y	Y	
Y		Y	
Y			

	Y	
Y		
	Y	
Y		
	Y	
	Y	
Y		
	Y	
		Y
Y		Y
Y		
		Y
	Y	
Y		
Y		
	Y	
	Y	
		Y
		Y
		Y
		Y
Y		
	Y	

	Y	
Y	Y	
	Y	
Y		
Y		
Y	Y	
		Y
		Y
	Y	
	Y	Y
	Y	
Y		
	Y	
		Y

			Y
			Y
			Y
Y			
Y			Y
Y			
			Y
		Y	
Y			
			Y
		Y	
Y			
		Y	
			Y
Y			
Y			
			Y
		Y	
Y			Y
Y	Y		
		Y	
		Y	
			Y
		Y	

Y

Y

Y Y

Y Y

Y

Y

Y

Y

		Y	
		Y	
Y			Y
			Y
		Y	
Y			
			Y
			Y
			Y
			Y
			Y
			Y
			Y
Y			Y
	Y		Y
	Y		
Y			

		Y
Y		
		Y
	Y	
		Y
		Y
		Y
	Y	
	Y	
		Y
		Y
		Y
Y		Y
		Y
		Y
		Y
		Y
		Y
		Y
Y		

		Y	
	Y		
		Y	
			Y
			Y
			Y
		Y	
		Y	
		Y	
			Y
Y			
			Y
			Y
		Y	
	Y		Y
		Y	

Y Y

Y

Y

Y

Y

Y

Y

Y

Y

Y Y

Y Y

	Y	
Y		
Y		Y
	Y	
		Y
		Y
		Y
	Y	Y
Y		
	Y	
	Y	
		Y
Y		
		Y
Y		
		Y
		Y
Y		Y
		Y
		Y
		Y

		Y	Y
			Y
Y		Y	
Y			
		Y	Y
			Y
	Y		Y
			Y
		Y	
			Y
		Y	
Y			
			Y
			Y
		Y	
Y		Y	
Y			
			Y
			Y
Y			
		Y	
			Y

			Y
			Y
		Y	Y
		Y	
	Y	Y	
		Y	
Y			
			Y
			Y
			Y
		Y	
			Y
			Y
			Y
			Y
		Y	
		Y	Y
Y			Y
		Y	
			Y
		Y	
			Y
			Y
			Y

Y
Y

Y

Y

Y

Y

Y

Y

Y

Y

Y

Y		Y
		Y
	Y	
	Y	
		Y
		Y
		Y
		Y
		Y
	Y	
Y		
	Y	
Y		
		Y
	Y	
	Y	
	Y	

		Y	
		Y	
			Y
			Y
			Y
		Y	
		Y	
	Y		
			Y
Y		Y	Y
Y			
			Y
Y			
Y			
		Y	
		Y	
			Y
		Y	
			Y
Y			

Y			
			Y
		Y	
			Y
Y			
		Y	Y
			Y
		Y	
		Y	
Y			
Y			
			Y
			Y
	Y		
		Y	
		Y	
		Y	
			Y
Y			
			Y
		Y	

		Y
	Y	
	Y	
Y		
		Y
		Y
Y		
	Y	
	Y	
		Y
		Y
		Y
Y		
	Y	
Y		
		Y
	Y	
Y		Y
		Y
	Y	

	Y		
			Y
		Y	
		Y	
Y			
Y			Y
Y			Y
			Y
			Y
			Y
		Y	
			Y
		Y	
Y			
Y			

	Y	
		Y
		Y
		Y
Y		
		Y
	Y	
	Y	
	Y	
Y		
	Y	
Y		Y
	Y	
Y		
	Y	
	Y	
	Y	
Y		

Y			
			Y
		Y	
Y			
			Y
		Y	
			Y
			Y
Y		Y	
Y			
Y			
		Y	Y
		Y	
			Y
			Y
		Y	
		Y	
		Y	
	Y		
		Y	
Y			
			Y
			Y
			Y

Y		
		Y
	Y	Y
		Y
		Y
Y		
		Y
	Y	
		Y
		Y
Y		Y
		Y
		Y
		Y
		Y
	Y	
	Y	

	Y		
Y			
Y			
			Y
			Y
		Y	
			Y
			Y
Y			
	Y		Y
			Y
Y			Y
			Y
Y			
		Y	
			Y
		Y	
			Y
		Y	
			Y
	Y		Y

		Y
		Y
	Y	
		Y
Y		
Y		Y
Y		
Y		Y
	Y	
		Y
Y		
		Y
Y		
Y		Y
	Y	
	Y	
		Y
		Y

Y

Y

Y

Y

Y

Y

Y

Y Y

Y

Y

Y

Y

Y

Y

			Y
			Y
	Y		
Y			
		Y	Y
		Y	
			Y
		Y	
Y			
			Y
		Y	
			Y
		Y	
Y			Y
Y			
			Y
		Y	
		Y	
			Y

Y
Y

Y
Y

Y
Y

Y
Y

Y

Y

Y

Y

	Y	
	Y	
		Y
Y		Y
	Y	
Y		
	Y	
	Y	
	Y	
	Y	
		Y
	Y	
	Y	
		Y
		Y
Y		
		Y
	Y	
	Y	
	Y	
Y		
		Y

		Y
Y		Y
Y		
		Y
		Y
	Y	
	Y	
	Y	
	Y	
		Y
	Y	
	Y	
		Y
	Y	
Y		
	Y	

Y

Y

Y

Y

Y

Y

Y

Y

Y

Y

Y

Y

Y

Y

Y

Y

Y

Y

Y

Y

			Y
		Y	
		Y	
			Y
			Y
			Y
Y			Y
			Y
			Y
			Y
Y			
Y			
Y		Y	
		Y	
		Y	
		Y	
		Y	
			Y
Y			
			Y
	Y		
			Y
			Y
		Y	
Y			
			Y
			Y

		Y	
		Y	
Y	Y		
Y			
		Y	
			Y
			Y
		Y	
			Y
		Y	
Y			
Y			
			Y
			Y
			Y
Y			Y
Y			
Y		Y	
		Y	
			Y
			Y

	Y	Y
		Y
Y		Y
	Y	
Y		
	Y	
	Y	
Y		
Y		
Y		Y
		Y
		Y
		Y
		Y
		Y
		Y
		Y
Y		
	Y	
		Y
Y		Y
		Y
		Y
	Y	

			Y
		Y	
			Y
			Y
			Y
			Y
	Y		Y
		Y	
		Y	
		Y	
Y			Y
			Y
			Y
			Y
			Y
			Y
			Y

		Y
		Y
Y	Y	Y
		Y
		Y
	Y	
		Y
		Y
Y		
		Y
	Y	
	Y	
		Y

			Y
Y			
Y			Y
			Y
		Y	
			Y
Y			
	Y		Y
Y			
Y			
Y			
Y		Y	
		Y	
Y			
		Y	
			Y
	Y		Y

		Y
		Y
		Y
Y		Y
		Y
	Y	
	Y	
		Y
Y		
		Y
	Y	
	Y	Y
Y		

Y

Y
Y

Y
Y

Y

Y
Y

Y

Y

Y

Y

Y

Y

Y
Y
Y

Y			
			Y
			Y
			Y
		Y	
	Y	Y	
Y			
			Y
		Y	
			Y
			Y
		Y	
			Y
Y			
			Y
			Y
Y			
Y			Y

Y

Y

Y
Y

Y

Y Y
Y
Y Y
Y

Y

Y

Y

Y

Y
Y
Y
Y

Y

Y

Y

Y

Y

Y

Y Y

Y

Y

Y

Y

Y

Y

Y

Y

Y

Y

Y

Y

Y

Y

Y

Y

Y

Y

Y

Y
Y

Y

Y

Y
Y Y

Y
Y

Y

Y

Y
Y
Y

Y
Y

Y
Y
Y

Y

Y
Y

Y

Y
Y

Y

Y

Y

Y
Y

Y

Y

Y

	Y	
		Y
	Y	
		Y
		Y
Y	Y	
		Y
		Y
		Y
		Y

Y
Y
Y
Y
Y

other_risk_spec	previous_hospitalization
HYPERTANSION	N
	Y
PREMATURITY	N
	N
	N
	U
	N
	N
LEUKEMIA	N
	U
	Y
	N
TB SURGERY	Y
	Y
	N
	N
	N
	N
	N
	Y
	N
	N
INBORN ERROR OF METABOLISM, FAILURE TO T	Y
	N
	N
	Y
CONENITAL SYPHILIS	N
	N
	N
KCI	N
	N
	N
NEPHROBLASTOMA	Y
	Y
MALNUTRITION; HYPOALBUMINAEMIA	Y
	N
	N
	U
	N
	N
SLE (DEE LUPUS), HSV	N
	Y
	Y
	Y
	N
	Y
	N
	Y
	N
	N

	N
	N
	N
LEUKEMIA	N
HEART DISEASE	N
	U
	N
	Y
	Y
	Y
	N
	Y
	Y
	N
	N
	N
	N
ARTHRITIS	N
CCF	Y
	N
DECUBITUS ULCER BOTH ELBOWS AND BOTH BUT	Y
	N
	U
	Y
	Y
	Y
	Y
	Y
	Y
	N
	Y
	N
	Y
	Y
	U
	N
CHRONIC ARTHRITIS AND GOUT	N
	Y
	N
	N
	N
	N
PERFORATED BOWEL / PREMATURITY 30/40	N
	N
GASTRIC ALCERS	Y
	N
	N
	N
	N
	Y
	Y

EPILEPSY	Y
	U
	U
	N
CHOLELITHIASIS	Y
	N
	N
	N
	N
	Y
	N
	N
	N
	Y
	N
	U
	N
	N
	Y
	U
	U
	Y
TRACHEOSTOMY - NECROSIS OF PHARNYX / EXP	N
NEONATAL SEPSIS	N
	N
	U
	N
	N
	N
	N
	N
MALNUTRITION, ANAEMIA	N
	N
	N
	N
PARTIAL DEAFNESS AT 2 YEARS - GROMMETS I	N
	N
CRYPTOCCOCAL MENINGITIS	U
	N
EXPLORATORY LAPAROTOMY - PANCREATICODUOD	Y
	N
	Y
	N
	N
EXTREME PREMATURITY	N
	Y
HEPATO BILIARY GASTROINTESTINAL TRACT B	Y
	N
	N
HYPERTENSION	N
	Y
ALCOHOL HEPATITIS	N
	U
	N

	Y
	Y
	N
	N
SURGERY. INSERTION OF TRACHEAL TUBE	Y
	N
	U
	Y
	N
	U
ASHMATIC	Y
	Y
	N
	N
	U
	U
	Y
SLE	N
	Y
	U
RENAL FAILURE	N
	Y
	Y
	Y
SYSTEMIC LUPUS ERYTHEMATOSUS, NEPHRITIC	Y
ANGINA	Y
	U
	Y
	Y
	Y
LEFT BELOW KNEE AMPUTATION NECROTISING F	N
NECROTIC BOWEL	N
OESOPHAGEAL ATRESIA/ PREMATURITY 32/40	N
	N
	Y
PREMATURE	N
	Y
	N
	Y
	N
	N
	N
ACUTE GASTROENTERITIS, MALROTATION & VOL	Y
	U
	Y
PROSTHETIC VALVE INEFFECTIVE ENDOCARDITI	Y
	Y
	U
	N
	U
OVARIAN CYST	Y
	N
	N
HIV POSITIVE & TB	N
	N

	N
	N
	N
	Y
PERIPHERAL VASCULAR DISEASE	N
	N
MULTIPLE FUSIFORM ANEURYSM INTRACRANIALY	Y
	U
HYPERTENSION	N
	N
RENAL FAILURE	N
	N
	N
	U
	N
	U
	N
	N
	Y
	Y
	Y
	N
	N
PREMATURITY	N
	U
	N
	Y
	N
	Y
	N
	N
PREMATURITY	N
	Y
	N
	U
	N
SURGERY	N
PREMATURITY	N
	U
	N
	N
	N
	N
	N
	N
	N
	N
SEVERE ANEAMIA, ?OBSTRUCTIVE JAUNDICE	Y
	N
	N
	Y
	U
	N
LIVER TRANSPLANT	Y

MILD RENAL FAILUREPOSITIVE SOCIAL SUBSTA	Y
	U
	N
	U
	N
	N
	U
CHRONIC LUNG DISEASE	N
	N
	Y
	N
	N
HOLLOW VISCERAL MYOPATHY, DEGENERATIVE M	Y
ERBS PALSY / SHOULDER DYSTOCIA	N
	N
	U
	U
	U
	N
	U
	U
	U
	U
	U
	U
	N
	U
	N
	N
DECUBITUS ULCER	Y
	N
EPILEPSY AND CEREBRAL PALSY	Y
	N
CHRONIC LIVER DISEASE	Y
	Y
	Y
	Y
	N
EXPLORATORY LAPAROTOMY-FEEDING JEJUNOSTO	N
	N
	N
	Y
	N
	Y
	U
	U
	N
	N
	N
	N
	N
	N
	N
	Y
	N

	N
	Y
	U
	U
	U
	N
	N
	U
	N
	U
	N
	N
	N
	N
MALNUTRITION	N
	N
	Y
	N
HIV POS	Y
PULMONARY TB	N
MULTIPLE BED SORES	N
	N
	U
HYPERTENSIVE ON TREATMENT	N
	N
	U
	U
	U
	U
CO - PULMONALE	U
	N
	Y
	U
	U
	U
	U
HEART DISEASE (CYNOTIC HEART)	Y
	U
ENCEPHALITIS	N
	U
	U
PREMATURITY	N
	Y
PREMATURITY	N
	N
	U
	N
	N
CHOLANGITIS WITH CHOLANGITIC ABSCESS	N

POST ST SEGMENT ELEVATION MYOCARDIAL INF	U
	N
ABDOMINAL MISCHIEF	N
AKUTE KIDNEY INJURY	N
CAUNDA ENQUINA CONUS SYNDROMEINCISION AN	N
HYPERTENTION	Y
GUN SHOT ABDOMEN INJURED ORGANS - DEUDEN	N
ATRIAL FIBRILLATION, ISCHEMIA OF THE LEF	N
MULTIPLE RELOOK LAPAROTOMIES WITH SPLENE	N
PREMATURITY	N
BILATERAL CRITICAL LIMB ISCHAEMIA	Y
	N
	U
EPILIM SENSITIVITY	N
UPPER GASTROINTESTINAL BLEED	N
PREVIOUS ASSAULTED	U
	N
	U
	U
CANCER OF THE CERVIX WITH BILATERAL HYDR	Y
	N
	U
	U
	U
	U
SYSTEMIC LUPUS ERYTHROMYTOSIS WITH MULTI	Y
	Y
	N
	N
	U
CEREBAL PALSY	Y
	U
	U
	Y
	N
	U
	U
DEMENTIA, MULTIPLE BEDSORES	N
TUBERCULOSIS	N
	N
	Y
	U
PRETERM	N
	U
	U
	N
	N
	U
	N
	Y
	N
	N
	U
	N

ENHANCING MYOMETRIUM, LARGE FIBROID	N
CYTOMEGALOVIRUS COLITIS	N
	Y
	Y
	N
	Y
	N
	N
	N
	Y
	U
	N
	N
	Y
	Y
	Y
	Y
	N
	U
	N
	U
	N
GASTROCHISIS (OOMPHOCELE) BIRTH DEFECT	N
	N
	U
	N
	N
HYPERTENSION	N
TB	N
PREMATURITY	N
	Y
	Y
	U
HYPERTENSION	N
NECROTIZING ENTEROCOLITIS	N
SEPSIS, STOMA AND ABDOMINAL FISTULA	Y
	N
	U
	U
	U
	N
	U
	U
	N
	Y
	N
	Y
COPD	N
PATENT DUCTUS ARTERIOSUS	N
	U
ACUTE ABDOMINAL INTESTINAL OBSTRUCTION	Y
	N
	U
	Y
	Y

	Y
GASTROCHISIS (BIRTH DEFECT)	N
	N
	U
	U
	N
	Y
	U
	U
	N
	N
	N
	U
	Y
	N
	Y
STEMI	N
	N
	N
	Y
PREMATURITY, DRIPSITE SEPSIS	N
	U
PREMATURITY,TREPONEMA PALLIDUM HAEMOGGLU	N
	Y
HYALINE MEMBRANE DISEASE	N
	Y
	Y
	N
	Y
	N
	N
	N
EPILEPSY, COAD, HPT	Y
CARDIAC	N
	N
	Y
	N
	N
	Y
	N
	N
	U
	U
EPILEPSY AND HYPERTESION	N
	U
	U
	N
	N
	U
TRAUMATIC BRAIN INJURY	N
	U
	N
CONNECTIVE TISSUE DISEASE	N
KIDNEY FAILURE	N

PREMATURITY	N
TB MENINGITIS, GLIOMATOSIS CEREBRI	N
	U
HYPERTENSION	N
	Y
PREMATURITY	N
GASTROESOPHAGEAL REFLUX	N
	N
	N
	N
	Y
	U
	N
	N
	U
	U
	Y
	N
	N
DISSEMINATED TB	Y
	Y
	N
	N
	N
RENAL FAILURE	N
	Y
	N
OSTEOPOROSIS/OESTEO ARTHRITIS	N
PREMATURITY	N
	Y
	N
	N
PREMATURITY, NECROTIZING ENTEROCOLITIS	N
	Y
	N
	N
PREMATURITY	N
PREMATURITY	N
	N
	N
	N
	U
	N
	N
JEJUNAL ATRESIA	N
	N
CHRONIC KIDNEY DISEASE STAGE 3	Y
	U
	N
	N
	U
	N
	U
	U

	U
	Y
	U
	U
	U
	N
RIGHT HYDROCELE	N
PREMATURITY	N
	Y
CONGENITAL TB	N
HYPERTENSION	N
	N
	U
ACUTE KIDNEY INJURIES, GASTRITIS	Y
	N
	U
	Y
	U
	N
ASPIRATION PNEUMONIA AND INTRA CRANIAL B	N
COPD ON STEROIDS	N
HIV POSITIVE	N
	N
	N
	N
	U
	N
	N
	Y
	U
GANGRENE BOTH LEGS	N
	N
	N
HEAD OF PANCREAS MASS	N
	Y
	N
	N
	Y
	U
PERITONITIS	N
	N
	N
	N
	N
	N
	N
PREMATURITY	N
	N
	U
	N
	N
	U
	U
	U

	U
	Y
	U
	N
	U
	Y
	N
	N
	N
NECROTISING ENTEROCOLITIS	N
	N
	N
	N
	N
	U
NECROTIZING ENTEROCOLITIS	N
PREMATURITY	N
SUSPECTED NECROTIZING ENTEROCOLITIS	N
ILEUS	N
	N
	N
	N
PERFORATED PEPTIC ULCER	U
	N
	N
	N
APPENDICITIS,INTESTINAL OBSTRUCTION	Y
	N
	N
	N
	N
SEPTIC ARTHRITIS,EPISTAXIS(NOSE BLEEDS,	U
	U
	Y
	N
	N
NECROTIZING APPENDIX AND CAECUM,FASCITIS	N
	N
	N
	Y
	U
	N
	U
	N
	N
	N
	U
	Y
	N
	N
	N
MULTIPLE SCHLEROSIS	Y
HYPERTENSION	N
	N
	N

	N
	N
	N
	N
	N
	N
	N
	N
	Y
	N
	N
	N
	N
	U
	N
	N
CRYPTO	Y
	U
COPD	Y
	N
	N
	U
	N
	N
	N
	N
	N
ORGANOPHOSPHATE POISINING	N
PREMATURITY	N
DYSPLASIA CARDIAC DYSFUNTION HYPERTENSIO	Y
	N
PREMATURITY	N
	N
	N
	N
	U
	N
	N
	Y
	N
SEPSIS	N
	N
CARDIAC CONDITION INCLUDING VALVULAR DIS	Y
	Y
	N
	N
	N
	N
	N
	N
	N
	N
	N
	N
KNEE SURGERY	U
	N

	N
	N
	N
	N
	N
SLE	N
	N
	N
	U
	Y
	U
	N
	N
	U
	N
	N
	U
	N
	N
VASCULAR DEMEMTIA	U
	U
	U
	U
	N
	U
	U
	U
	U
	N
AFFECTED LEFT KIDNEY (MALIGNANCY)	Y
	U
	U
	U
	Y
	N
EPILEPSY, ANAEMIA	Y
	N
	U
	U
	N
	U
	N
PEFORATED CEACUM	N
	U
	N
	U
	U

	N
FORNIERS GANGRENE	N
PERICARDIAL EFFUSION/EPILEPSY	Y
	N
COPD, EPILEPSY, GORD	N
	U
	Y
	U
	U
	N
	N
	Y
PREMATURITY	N
	U
	N
	U
	N
	U
	N
	N
	U
	N
	U
SLE, HEPATOMEGALY	Y
	N
	N
	N
	Y
	N
	N
	N
	U
	U
	N
	U
	N
	N
	U
	U
	U
	U
	N
	U
	N
	U
	N
	U

	U
CARDIAC	Y
	N
	U
	U
	N
	Y
	U
	U
PYELONEPHRITS	Y
ACUTE MEGALOBLASTIC LEUKEAMIA, CA. PROST	Y
	U
SEPTIC ARTHRITIS, PREMATURITY, NOSOCOMIA	N
	U
	U
CONGENITAL HEART DISEASES	Y
	N
	U
	N
	U
	U
	N
	N
	N
	N
SEPSIS	N
	N
	N
	N
	N
	N
	U
CONGENITAL BOWEL PERFORATIONS	Y
	N
	N
	N
	N
	U
	N
	N
CHRONIC GASTRITIS	U
	N
	N
	N
	N
	N
	N
	N
	N
	U
	U
	U
HEMOPHILIA	N
NASOCOMIAL SEPSIS	N

	N
	U
	U
CHICKENPOX	Y
	N
	N
PREMATURITY, FAILURE TO THRIVE	N
	N
	N
	U
	U
	N
	U
	U
	N
	N
	N
	U
PREGNANCY - C/SECTION 28/12/2017	Y
	Y
	U
CCF	N
	N
	N
	N
	N
	N
	Y
	U
CARDIAC CONDITION	Y
	Y
	U
PEPTIC ULCER	U
	N
	N
	N
	N
	N
	Y
	Y
	N
	Y
	N
	N
	U
	N
CHRONIC LEFT SUBDURAL HAEMORRHAGE ON CT	Y
	N
	N
	N
SUSPECTED CARDIAC FAILURE	U
	U
	U
	U
	Y
	N

	U
TRACHEAL STENOSIS, PREVIOUS PTB	Y
	N
	N
	N
	N
	N
	N
	N
	N
	N
	Y
	N
	Y
	N
	N
	N
	N
	N
OCCIPITAL PRESSURE SORE	U
	N
	Y
	Y
CHOLEDOCHOLI	U
	N
	N
	N
	N
HYDROCEPHALUS	N
	N
	N
	U
	N
	N
BOERHAAVE SYNDROME	N
	U
	U
	N
	N
	N
	U
	U
	N
	N
PREMATURITY	N
	N
	U
	U
	U
PREMATURITY	N
	U
	Y
	N
	N
	N
	N

CKD,SARCODOSIS	Y
	N
	N
NECROTIZING ENTEROCOLITIS	N
	Y
	N
	N
	U
	U
	N
	U
	U
	N
	U
	U
	U
	N
	U
	N
	N
	U
	Y
	N
	N
	U
	U
	N
NOENATAL JAUNDICE, ? SEPSIS	N
LIVER TRANSPLANT	Y
	U
	N
CHRONIC GASTROENTERITIS DISORDER	Y
	Y
	N
	N
	N
	N
HYPERTENSION	U
	N
	N
JEJUNAL PERFORATION	N
	N
	Y
	N
	N
SYSTEMIC LUPUS ERYTHROMATOSSUS	N
	N
	N
	Y
	N
	N
	Y

	U
	Y
	Y
	Y
	N
	U
	N
	N
	N
	N
	N
	N
	Y
	Y
	N
	N
CCF+COPD	Y
HEART BLOCK	N
	N
	Y
NOENATAL JAUNDICE, ? SEPSIS	N
	U
	N
	N
	N
	N
	N
	N
	N
	N
	N
	Y
	N
	N
	N
	N
TB AND RENAL FAILURE	N
	N
	U
	U
PREMATURITY	N
PREMATURITY	N
	U
	U
	N
TBM	N
	N
	N
	N
	N
	N
	N

	N
	U
	U
	N
	N
	U
	U
	N
	N
	N
	N
	U
	N
	U
	N
	U
	U
	N
	U
	N
	U
	U
	Y
	U
	N
	U
	U
	N
	U
	N
	N
PREMATURITY	N
	N
	N
	N
SEPTIC RIGHT LEG	U
	N
	N
	U
	Y
	N
	N
SEVERE MUCOSITISNEUTROPENIC FEVERACUTE L	Y
	U
	N
	Y
	Y
	N
	U
LEFT UPPER QUADRANT PREMATOSIS	N

	N
	N
PREMATURITY	N
	N
	U
	N
	N
PREMATURITY	N
	N
EPILEPSY	N
	Y
	N
	N
	N
ISCHAEMIC HEPATITISAKI /ACUTE KIDNEY INJ	N
	N
LIVER FAILURE	N
	N
	N
THROMBOCYTOPENIA	N
	N
	Y
	N
	N
	U
JAUNDICE AND RESPIRATORY DISTRESS	N
	N
	N
	N
	U
	N
LTR	N
	Y
UPPER GASTROINTESTINAL BLEED	U
	N
	Y
	U
	N
	N
	U
	Y
	U
MULTI DRUG RESISTANT TUBERCULOSIS, STEVE	N
	Y
	Y
	Y
	Y
	N
	N
	U
	N
	N

EXTREME LOW BIRTH WEIGHT	N
	Y
	Y
	N
RIGHT ARM AMPUTATION-CELLULITIS	Y
	N
CARDIAC CODITIONS	N
	N
	N
	U
	N
	N
	N
	Y
	Y
	N
	N
PRETERM	N
	U
	U
	N
	N
	N
	N
	N
PREMATURE	N
	U

	N
	U
	U
	N
	N
BOWEL NECROSIS/INTRA ABDOMINAL HYPERTENS	Y
PREMATURITY	N
	N
	N
	N
	N
	N
HEART DISEASES	N
HYPERTENSION	N
NEWLY DIAGNOSED WITH DISSEMINATED TB OF	Y
RENAL DYSFUNCTION	N
FANCONI ANAEMIA	Y
	N
PREMATURITY & SURGERY-CRICOID SPLIT & DI	Y
ASTHMA	N
	N
	N
	N
	U
PERICARDIAL EFFUSION, PERSISTENT ANAEMIA	Y
	N
PERIPHERAL VASCULAR DISEASE	Y
	N
	N
	Y
	N
	U
	Y

PREMATURITY 26/40, EXTREMELY LOW BIRTH W	N
	N
	U
	N
	N
PREMATURITY	N
	U
	N
PTB	N
PTB	N
	N
PREMATURITY	N
	N
	Y
	Y
	N
	N
	Y
	N
	U
	N
	N
	U
EPILEPSY	U
	N
	N
	N
	U
MVA(SEPSIS AND THROMBOCYTOPENIA	U
	N
	N
	N
PREMATURITY	N
HYPERTENSION	N
HYPERTENSIVE	N
	N
	N
HYPERTENSIVE	N
	N
TRACHEOMALACIA, PATENT DUCTUS ARTERIOSUS	Y
CHEMOTHERAPY	U
PREMATURITY	N
ASTHMA, COPD, RHEUMATOID ARTHRITIS	Y
	Y
	N
	N
	N

	N
	N
	N
	N
	N
	Y
CHRONIC LUNG DISEASE	N
PREMATURITY, IGG RUBELLA	N
TUBERCULOSIS	N
	N
	Y
	Y
	N
	N
	N
	Y
	N
	U
LIVER FAILURE	Y
	N
	Y
	N
ORGAN FAILURE	U
	U
	N
	N
	N
	Y
PREMATURITY, VERY LOW BIRTH WEIGHT	N
	N
	N
	N
	Y
BRAIN TUMOR	U
	N
	U
	N
	N
	Y
	N
	N
	N
	N
	N
	U
	U
	N
	U
	U
	N
	Y
	U
	U

	Y
	U
	N
CARDIAC CONDITION: AORTIC DISSECTION	N
	N
	N
	N
	N
	N
	N
	N
CARDIAC ILLNESS	Y
	N
	Y
	N
	N
	N
	N
	N
	N
	N
	U
VERY LOW BIRTH WEIGHT	N
	N
PREMATURITY	N
	Y
	N
	N
	N
	N
	N
	N
	N
	Y
	N
	N
	N
	N
	N
PNEUMONIA	N
HYPERTENSION	N
	N
	N
	N

	Y
	Y
	Y
HYPERTENSION,GOUT,PLASTIC ANAEMIA	N
	N
	N
	Y
	N
	N
PTB	N
	N
	N
	N
	N
	U
	U
	N
	U
	N
	N
	N
RENAL FAILURE AND LIVER FAILURE	N
	N
TB	N
HYPERTENSION	Y
	N
	N
	N
	Y
	N
	N
	N
	N
PNEUMONIA	N
	N
PREMATURITY	N
	N
PREMATURITY	N
SEVERE ACUTE MALNUTRITION	N
NECROTIZING PANCREATITIS	N
ARTHRITIS	N
LRT	N
	N
	N
GASTROCTOMY	N

	N
	N
	N
	N
	N
	N
	N
DEEP VEIN THROMBOSIS	Y
	Y
	N
ELEOTOMY SURGERY	Y
	U
	Y
SLE	Y
	U
LIVER FAILURE	N
	N
	N
RESPIRATORY DISEASES	Y
GUILLAIN-BARRE SYNDROME	N
	N
	Y
LRTI AND RENAL IMPAIREMENT	N
	N
	N
	N
	N
	N
	N
	N
	N
	N
SYSTEMIC LUPUS ERYTHEMATOSUSSEVERE MALNU	Y
	N
CCF	N
	U
	N
	N
CHRONIC OBSTRUCTIVE PULMONARY DISEASE	Y
PREMATURITY	N
	N
	N
	N
	N
	N
	N
	Y
	N
PREMATURITY	N

	N
CHRONIC OBSTRUCTIVE PULMONARY DISEASE /	N
	N
	N
GULLIAN BARRE SYNDROMEMILITARY TBEXTENSI	Y
	N
HEPATECTOMY	N
	N
	Y
	N
	N
	N
	U
HYPERTENSION	Y
HYPERTENSION AND COPD	N
	N
EXPLORATORY LAPROTOMYSLE SYSTEMIC LUPUS	Y
	N
	Y
	Y
CHRONIC GASTRO ENTERITISEMPERIC PCP	Y
SURGERY FOR RELOOK LAPAROTOMY X 4HEMICOL	Y
	U
	N
SURGERY	N
	N
LEFT ILEOPSOASTOXIC MEGA COLON- TOTAL C	N
	N
CANCER OF RECTUMDOUBLE BARREL ILEOSTOMYM	Y
	N
	N
	N
SURGERY DISSECTING THORACIC ABDOMINALANE	Y
	N
	Y
	N
PREMATURITY	N
	N
	N
	U
	N
	N
ASTHMAGORDRHEUMATOID ARTHRITISIRON DEFIC	N
	N
	N
PREMATURITY	N
	N
	U
	Y
	N
PREMATURITY	N

	N
	U
	N
	Y
COMPLICATED CROHN'S DISEASE	Y
MUTI INGESTION	N
	N
	N
	N
	N
	N
	N
COPD	Y
BENIGN OESOPHAGEAL STRICTURE	Y
DIABETES	N
	N
	N
	N
	Y
	Y
TRACHEASTOMY	U
	N
	N
	N
	Y
	N
	Y
	U
	N
	N
	N
	N
	N
	N
	N
RIGHT CHAMBERLAIN'S PROCEDURE & BRONCHOS	Y
	N
	N
	U
	U
	Y
	Y
	Y
	N
	N
	Y

	U
	N
	N
	N
LUPUS	Y
SLE	Y
	U
	N
	U
	Y
	U
JAUNDICE	N
PREMATURITY	N
	N
	N
LIVER DISEASE	N
	U
SCHIZOPHRENIA	N
	N
	N
	N
	N
	N
CHRONIC KIDNEY DISEASE	N
	Y
	N
	N
	N
	U
	U
	U
	N
	Y
	N
	N
	N
	Y
	Y
DISSEMINATED TUBERCULOSIS	N
	N
	U
	N
	N
	Y

	Y
	U
	U
RENAL DYSFUNCTION	N
	N
	N
CARDIAC DISEASE	N
PREVIOUS ALCOHOLIC/JAUNDICE & WAISTING	Y
	U
	N
	Y
	U
	N
PREMATURE	N
ASTHMATIC	Y
	N
	N
	N
	N
	N
	N
	U
	N
	U
PREMATURITY	N
	N
	N
	N
	N
	N
	N
	N
	N
	N
	N
	U
	U
	N
	N
	U
	N
	Y
	N
	N
CONGENITAL ABNORMALITIES	N
	U
	U
	N
CRYPTOCOCCAL MENINGITIS	N
	U
	N

	U
	N
	N
	N
	Y
PREMATURITYLOW BIRTH WEIGHTPULMONARY HAE	N
	N
	Y
	N
	Y
MULTIPLE ABDOMINAL OPERATIONS FOR BOWEL	U
	U
	N
	Y
HYPERTENSIVE	N
	N
	N
PREVIOUS ABDOMINAL SURGERY	U
	N
	U
	N
	N
	U
PREMATURITY	N
	N
ACUTE KIDNEY INFECTION - HAEMODIALYSIS	N
	N
	N
	U
	N
	Y
	N
	N
	N
	Y
	N
	Y
EPILEPSY	N
	N
	N
	N
	U
	U
	N
	N
HEREDITARY TELANGIECTASIA ANEMIA	N
	U
	N
	U
	U
	N

	N
	N
	N
	N
PREMATURITY	N
	Y
	N
	Y
	N
	N
	N
	N
	N
	N
	N
	N
	N
PREMATURE_LOW BIRTH WEIGHT	N
	N
SMALL BOWEL OBSTRUCTION	Y
	N
	N
	N
	N
	N
	N
	N
	N
	N
CONGENITAL HELP SYNDROME	N
	N
	N
	N
RENAL FAILURE AND HYPERTENTION	N
SURGERY	N
	N
	N
	N
	N
BONE MARROW TRANSPLANT	N
	N
	N
SMALL BOWEL OBSTRUCTION, KIDNEY INFECTIO	N
	N
CONGENITAL BOWEL PERFORATION	N
	N
	N
	N
	N
	N
	N
	N

	U
	U
	U
	U
	U
	N
	U
	N
PARAPLEGIC	N
	U
	U
	Y
	N
	N
	N
	N
	N
	N
	N
LIVER FAILURE,PTB,HEART FAILURE,COPD	Y
	U
	N
	N
	U
	N
TB ABDOMEN	Y
	U
PREMATURE	N
	N
	N
WARDENBURG SYNDROME	N
PREMATURE	N
	U
	N
	U
	N
	N
PREMATURITY	N
	U
	N
	U
	U
	N
	U
	U
	N
	N
	N
	U
	N
	N
	N

	N
	U
	N
	N
PREMATURE	N
	N
	N
	U
	N
	U
	N
	N
	U
EXPLORATORY LAPAROTOMY FOR ? MALROTATION	N
	U
	N
PREMATURITY / EXTREMELY LOW BIRTH WEIGHT	N
	N
MILITARY TB	N
	N
	U
	N
	N
	N
	N
	N
	N
	N
	N
	N
	N
	U
	N
	N
	N
	U
LUPUS,NEPHRITIS	N
	N
	N
	U
	N
	N
	U
	Y
	N
	N

	U
	N
	U
	N
PREMATURITY	N
	N
	N
	Y
	N
	N
	N
URINARY INCONTINANCE	N
	U
PREMATURITY 28/40	N
	Y
PREMATURITY	N
	N
	N
	N
	N
	N
	N
	U
	N
COPD	N
	N
	Y
PREMATURITY	N
	Y
	N
	Y
PREMATURITY	N
	N
	N
	N
	N
	U
	N
	Y
	N
	N
	N
SLE,ANAEMIA	U
	U
PREMATURITY	N
PREMATURITY	N

	N
	N
	N
	N
LIVER NECROSIS	N
RENAL FAILURE	N
LIVER IMPAIRMENT	N
CONGENITAL ABNORMALITY	N
	N
RESPIRATORY DISTRESS, PREMATURE	N
	N
	U
	N
	Y
	N
	N
	N
PREMATURITY	N
	N
NEC,RDS,INTRAVENTRICULAR HAEMORRHAGE	N
	N
HYPERTENSION	N
NEC	N
	N
PREMATURITY	N
PREMATURITY	N
	Y
HEAD INJURY	N
	Y
	N
	N
	Y
	N

	N
	N
	U
	N
	N
	N
	N
	N
	N
	Y
	N
	N
	N
INTESTINAL FAILURE	Y
PREMATURE	N
	N
	N
	N
	N
PREMATURITY	N
PREMATURITY	N
	N
	N
HPT	Y
PERFORATED ULCER AND TB	N
	Y
NEC,ANAEMIA,GANGRENOUS FEET	N
ABDOMINAL SURGERY	N
	Y
	N
	Y
	N
RECTAL VAGINAL FISTULA	N
	Y
MITRAL VALVE REPLACEMENT	Y
	N
FISTULA	N
	N
EPILEPSY	N

	N
PREMATURITY	N
EMPYEMA	N
	N
	Y
MILLIARY TB	N
	N
	N
	N
	N
	N
	N
	N
	U
PULMONARY HEMORRHAGE, GRADE 4 IVH	N
	N
	N
	N
	N
CARDIAC LESION, RENAL DISEASE	N
	N
	N
	N
	N
	U
	N
	Y
PREMATURITY	N
	N
	N
LEUKEMIA	N
	N
LIVER FAILURE,PANCREATITIS, ENCEPHALITIS	N
	U
	N
	U
	N
CHRONIC MYELOID LEUKAEMIA	Y
PREMATURE AT 33/40	N
	U
	Y

	U
	U
	U
	N
	U
HYPERTENSIVE	N
	N
	Y
	N
	Y
	N
	N
	N
	N
	N
	U
	Y
	N
PREMATURITY	N
	Y
PREMATURITY	N
	Y
	N
	N
	N
CARDIAC DISEASES	Y
	N
	N
	N
	Y
	Y
	N
	N
SURGERY	N
	N
	U
	U
	U
	N
	N
	N
	N
	N
	N
	U
	N
ANAEMIA,NEC	N

BURNS TO BODY 27%	U
BURKITTS LEUKAEMIA	N
	N
	N
	Y
	N
	N
	U
	N
	Y
	N
	U
	U
	N
	N
	N
	N
HYPERTENSION	Y
	N
	N
SEVERE GERD	N
	N
	N
	N
	Y
	N
	N
	Y
	N
	Y
	N
	N
	U
SURGERY	N
	U
	N
	N
LIVER,JAUNDICE	N
	N
SLE	Y
	N
	N
CHRONIC KIDNEY DISEASE	N
	U
	N
	N
	N
ANTRECTOMY	N

CEREBRAL PALSY	Y
	N
	N
	N
	N
	U
	N
	N
	U
MALFORMATIONS/ BIRTH DEFECTS, TRACHEO-OE	N
COPD	Y
	N
	U
	N
CARDIAC	U
SYSTEMIC LUPUS ERYTHEMATOSUS	Y
	N
SURGERY	N

N
N

Y

Y

	N
	N
	N
	N
PERFORATED NEC	N
	N
	N
PULMONARY OEDEMA AND HYPERTENSION	N
	N
	N
	N
	N
	U
	U
	Y
	Y
	N
	N
	N
	Y
	N
	N
	N
LRTI	N
	U
	U
	Y
	N
PREMATURITY	N
PREMATURITY	N
	Y
	N
CHYLOTHORAX	Y
	N
	N
	U
	Y
	N
	Y
	Y
	Y
	N
	N
	N
OLIVER FAILURE	N
	N
	Y
	N
	N

PRE-TERM AND RENAL IMPAIREMENT	N
HYPERTENSIVE	N
	N
PREMATURITY	N
PREMATURITY	N
	U
	N
	Y
	N
	U
	Y
	N
	Y
	U
	N
	N
	Y
	N
	U
	N
	N
	U
	N
	Y
	N
PREMATURITY & EXPLORATORY LAPAROTOMY FOR	N
	U
	N
	N
HYPERTENSION	N
	N
	N
	Y
	U
	N
	N
	N
	N
	U
	Y

	Y
	N
	N
	N
	N
	N
SURGERY	U
	U
	N
	Y
	U
	N
	N
	Y
	N
	Y
	U
POLIOMYELITIS	U
	N
CHRON'S DISEASE AND HEART FAILURE	N
	U
RDS,AKI,PROM,NNJ	N
	N
	U
	U
JEJENAK ATRESIA,THROMBOCYTOPAENIA,	N
	Y
ASTHMATIC	Y
	N
	N
	U
	N
COPD,UTI,HYPOTHYROIDISM,GRAVES DISEASE	N
	N
	N
	Y
	U
	N
	Y
	N
	N
	N
	N
SURGERY	N
	N

PREMATURE	N
	U
	N
	N
	N
	N
	Y
	Y
BURNS	N
CONGENITAL HEART DISEASE, PREMATURITY 33	N
CARDIAC LESIONS	N
	N
	N
TOXIC EPIDURAL NECROLYSIS	N
PREMATURITY	N
PREMATURITY	N
PREMATURITY	N
	N
	N
	N
	Y
	N
	U
	N
	N
	N
	N
	N
PREMATURITY	N
	N
	N
CHOLELITHIASIS	N
	U
	N
	U
	N
	N
PARAPLEGIA	N
EPILEPSY	N
	N
	N
	U
LARGE PATENT DUCTUS ARTERIOSUS ON ECHO	N
RDS,PULMONARY HAEMORRHAGE,THROMBOCYTOPAE	N
	N
	N
	N
	N
	N

	U
	N
	N
	Y
	U
	N
	N
	N
	Y
	N
	N
	Y
SURGERY	N
SURGERY	N
	Y
	N
ACUTE MYLOID LEUKEMIA	N
	U
	N
	U
	U
	U
	N
	N
	Y
	U
	N
	N
	N
	N
RDS,NEC,THROMBOCYTOPAENIA	N
ANAEMIA	N
GASTROCHISIS,PLEURAL EFFUSION	N
COVID POSITIVE, SEVERE OSTEOARTHRITIS	N
	N
	N
	N
	N
	Y
	N
	N
	N
	N
INCREASE BODY MASS INDEX, COVID 19 POSI	N
	U
	N
GASTROCHISIS AND THROMBOSIS	N

OBSTRUCTIVE JAUNDICE	Y
	N
NEC 3B	N
CEREBELLAR MASS	U
	N
	Y
	Y
	N
	N
	N
	N
	N
	N
COVID 19 POSITIVE	N
EPILEPSY	N
	N
PAROCOLITIC GUTTER ABSCESS	N
COVID 19, HYPOTHYROIDISM	N
	N
	N
CCF	N
	N
	N
RDS NEC,ANAEMIA,NNJ	N
HEART FAILURE	N
ACUTE MYELOID LEUKEMIA	U
	U
	N
	N
	N
UROLITHROSIS	N
LUPUS,PERICARDIAL EFFUSION, NEPHRITIS	U
LIVER FAILURE	N
CHRONIC TOPHACIOUS GOUT	Y
	N
	N
	N
	U
	N

	U
	U
	N
	N
	N
	N
NEC,CONGENITAL SYPHILIS, PULMONARY HAEMO	N
	U
	N
HYPERTENSION	N
	N
ACUTE LYMPHOBLASTIC LEUKEMIA	Y
OMPHALOCELE	N
SURGERY	N
DUODENAL ULCER	N
	N
	N
	N
	N
	N
	Y
HYPERTENSION	N
	N
	Y
SURGERY	N
NEC 2,RDS,NNJ	N
COPD	Y
	N
	N
	N
HYPERTENSION	U
RDS,IDM,THROMBOCYTOPENIA	N
RDS,HYPOGLYCEMIA, AKI,NNJ	N
	N
	N

HYPERTENSION	N
	N
	N
LEUKEMIA	N
POST COVID	U
RDS	N
	N
	N
NEPHROTIC SYNDROME	N
	N
	N
	U
	U
	N
RDS,AKI	N
	N
RDS	N
	N
	U
	N
	N
	N
	N
	U
	N
ABDOMINAL SURGERY	Y
	Y
	N
	N
	N
	N
	Y
	N
	N
	N
	N
	U
	N
QUADRAPLEGIC	Y

	N
	U
LEUKEMIA	U
	U
	U
	N
	U
	N
CCF	N
COPD	Y
	N
	N
	N
EPILEPSY AND HEAD INJURY	N
	U
VALVULAR HEART DISEASE, MITRAL STENOSIS	N
	N
	N
	U
	N
	N
	U
	N
	N
	N
	N
	N
	N
	N
	U
	N
	N
	N
	N
	N
	N
	N
	N
PARAPLEGIA(MULTIPLE MYELOMA)	N
	U
VALVULAR HEART DISEASE, MITRAL STENOSIS	N

ASTHMA	U
EMPHYSEMA, COPD,PERFORATED DUODENAL ULCE	U
	U
	N
	N
CHRONIC LUNG DISEASE	N
	N
LUPUS NEPHRITIS, HYPERTENSION,ILD	N
	N
	U
	N
	N
	N
PREMATURITY	N
	N
	N
	U
	Y
	N
	N
	N
	N
	N
	Y
	Y
	N
	N
GASTROCHISIS	N
	N
	N
	N
	N
	N
	N
	U
	N
LOW BIRTH WEIGHT	N
	N
COVID-19 PNEUMONIA	N
	U
	N

	N
PREMATURE	N
	U
	N
PREMATURE	N
	N
COPD	N
	Y
PREMATURITY	N
CCF, HYPERTENSION	N
	N
	N
	N
	N
	N
	N
	N
	N
	U
	N
	N
	N
	N
HYPERTENSION	N
	N
NEC,RDS,APNEA	N
	N
	U
	N
RDS,AKI,ANAEMIA, ACIDOSIS	N
HYPERTENSION	N
	N

	N
	N
	N
	N
	N
	N
MULTIPLE GUNSHOT WOUNDS	U
RENAL FAILURE,RDS	N
NECROTIC BOWEL,RDS	N
	U
	N
	N
	Y
	U
	Y
	N
HYPERTENSION	N
	N
	N
	U
PARALYSIS	N
	N
	N
	N
JEJENAL ATRESIA	N
	N
HYPERTENSIVE	N
	N
	U
	U
RDS,ANAEMIA, OSTEOPENIA	N
TB	N
	U
	N
	N
	N
ANAEMIA	N
	N
	U
	N
	N
	N

	N
	N
	U
	N
	N
	N
HEMOPHILIA	Y
RDS,NNE	N
AKI,MSL GRADE 3	N
ANAEMIA, LIVER TRANSPLANT	Y
HYPERTENSION	U
END STAGE RENAL FAILURE	U
	N
	N
	N
	N
	N
	N
PULMONARY TB	N
	N
	N
	N
RIGHT ARTERY ANEURISM	N
	N
	N
	N
	N
	N
	N
HYPERTENSION	N
	N
	Y
SEVERE DEHYDRATION, TOXIN INGESTION	N
EPILEPSY, CRANIOTOMY	N
RDS	N
	U
	U
	N

	N
	N
	Y
BORN WITH TOF	N
	U
	U
	N
EPILEPSY	U
TRISOMY 21,HYPOTHYROIDISM, PDA,VSD,ANAEM	U
	U
	N
HYPERTENSION	N
	N
	N
	U
	U
	N
	N
HYPERTENSIVE, CARDIOMYOPATHY	N
RDS,AKI,HYPOTENSION	N
	N
	U
	N
	N
	N
	Y
CHRONIC GASTRIC OBSTRUCTION	N
	N
	N
HYPERTENSION	N
INGUINAL HERNIA	U
PREMATURITY	N
	N
	N
	U
	N
	N
LIVER FAILURE	N

	N
	N
	N
	U
	N
	N
	N
	Y
	Y
	N
	N
	N
	N
	N
	Y
	N
	U
	U
	U
	U
	N
	N
	N
	N
	N
	N
	N
	U
	N
	U
	U
	N
OESAPHAGEAL STRICTURE	Y

ACUTE MYELOID LEUKEMIA	N
	Y
	N
	U
	N
	N
	Y
	U
	N
	U
	N
	U
PREMATURITY	N
PREMATURITY	N
	U
	N
	U
	N
	U
THROMBOCYTOPENIA, INFECTIVE ENDOCARDITIS	N
	N
	Y
HYPERTENSION, GOUD,G0	N
	U
	N
	N
	U
	N
	U
	N
	N
	N
	N

	N
HYPERTENSION	Y
POLYMYOSITIS	N
	N
	N
	N
	N
	U
	U
	U
	N
RDS	N
	N
PREMATURITY	N
TB	N
PREMATURITY	N
	U
	N
	U
	U
	N
	N
RIGHT HEMICOLECTOMY,MESTENTERIC ISCHAEMI	N
RDS,CUS,NEC GRADE 1	N
	N
	U
DCMO,ADH	U
REPAIRED LIVER INJURY ,PELVIC FRACTURE,H	N

	Y
	N
	N
	U
COPD	Y
VASCULAR DEMENTIA	N
	N
	N
	U
	U
	U
ACUTE MYELOID LEUKEMIA	N
RENAL DYSFUNCTION, HYDROCEPHALUS	Y
	N
	N
	U
	N
	N
HYPERTENSION	Y
QUADRIPLEGIA	Y
	N
CRYPTOCCOCUS MENINGITIS	U
PARAPLEGIA	U
	N
	N
BICYTOPENIA AND LEUCOCYTOSIS	U
	N
	N
HYPERTENSION	N

	Y
	N
RDS	N
	N
	N
	N
	N
PREMATURITY	N
DYSLIPEDEMIA	N
	U
	N
NECROTIZING ENTEROCOLITIS, ANAEMIA, FU	N
	N
PREMATURITY	N
	N
HIV, HYPERTENSION	N
	Y
HYPERTENSION	N
	N
	U
	N
	N

	N
	N
	N
	U
	N
COVID 19 POSITIVE	N
	Y
CARDIAC	N
	N
	N
	U
	N
	U
HYP6	N
	N

antibiotics_6months	oxygen	mechanical	mental	group_outcome	amimic
			unknown		16
N		N	unknown	Alive	<=8
Y	Y	Y	alert	Alive	>32
Y	Y	Y	sedated	Died	>32
N	N	N	alert	Alive	<=8
N	Y	Y	sedated	Alive	>32
U	N	U	unknown	Died	<=8
N	N	N	alert	Alive	<=8
N	Y	Y	unknown	Died	<=8
N	Y	N	alert	Alive	<=8
			unknown		>32
			unknown		<=8
			unknown		>32
U	Y	Y	sedated	Died	>32
Y	N	N	alert	Alive	<=8
N	Y	N	unknown	Died	16
U	N	N	alert	Alive	<=8
N	N	N	alert	Died	>32
N	N	N	unknown	Alive	>32
N	Y	N	alert	Alive	>32
N	N	Y	sedated	Alive	>32
N	Y	N	unknown	Alive	>32
N	Y	Y	sedated	Alive	>32
Y	Y	N	alert	Died	>32
Y	N	N	alert	Alive	>32
N	Y	N	disorientated	Died	16
Y	Y	N	alert	Alive	16
N	N	N	alert	Alive	>32
	Y	Y	sedated	Alive	>32
Y	N	N	alert	Alive	<=8
N	Y	N	unknown	Alive	>32
Y	Y	N	alert	Alive	<=8
N	N	N	alert	Alive	<=8
			unknown		>32
U	N	N	alert	Died	
N			sedated	Alive	
N	Y	N	unknown	Died	
Y	Y	Y	sedated	Died	
Y	N	N	alert	Alive	<=8
U	Y	Y	sedated	Died	>32
N	N	N	alert	Alive	>32
U	N	N	alert	Alive	>32
U	Y	Y	sedated	Alive	>32
N	N	N	alert	Alive	>32
N	Y	N	alert	Alive	>32
N	N	N	alert	Alive	<=8
Y	N	N	unknown	Alive	<=8
Y	N	N	alert	Died	16
Y	Y	N	unknown	Died	<=8
N	N	N	alert	Died	>32
Y	Y	N	alert	Died	16
N	Y	N	unknown	Alive	>32
N	N	N	disorientated	Died	<=8
N	N	N	alert	Alive	>32
N	Y	Y	sedated	Alive	<=8

N	Y	N	alert	Alive	16
N	Y	Y	sedated	Alive	<=8
N	N	N	alert	Died	<=8
N	N	N	alert	Died	<=8
N	Y	Y	sedated	Alive	<=8
U	N	U	unknown	Died	<=8
N	N	N	alert	Alive	<=8
Y	N	N	alert	Alive	>32
N	N	N	alert	Alive	>32
N	Y	Y	sedated	Alive	>32
N	Y	N	sedated	Alive	<=8
N	Y	N	alert	Alive	32
Y	Y	N	alert	Alive	<=8
N	N	N	alert	Alive	
N	Y	Y	sedated	Alive	>32
N	Y	Y	sedated	Alive	<=8
N	Y	U	alert	Alive	<=8
N	N	N	alert	Died	<=8
N	Y	Y	sedated	Died	>32
Y	Y	Y	sedated	Died	<=8
U	N	N	unknown	Died	32
Y	Y	Y	unconscious	Died	<=8
N	N	Y	sedated	Alive	>32
U	Y	Y	sedated	Alive	<=8
N	N	N	alert	Alive	<=8
Y	Y	N	alert	Alive	<=8
Y	Y	N	disorientated	Died	>32
Y	N	N	alert	Alive	>32
			unknown		<=8
Y	Y	N	disorientated	Alive	>32
Y	N	N	alert	Alive	>32
			unknown		>32
N	N	N	alert	Alive	>32
N	Y	N	alert	Alive	
N	Y	N	unknown	Died	
Y	N	N	alert	Died	
N	Y	N	unknown	Died	
U	N	U	unknown	Died	
N	Y	N	alert	Died	
U	Y	U	unknown	Died	
Y	N	N	alert	Alive	
Y	N	N	alert	Alive	
Y	N	N	unknown	Died	
N	Y	Y	sedated	Alive	
N	Y	N	alert	Alive	
N	Y	Y	sedated	Died	
N	Y	N	alert	Alive	
Y	Y	N	alert	Died	
N	Y	N	disorientated	Alive	
N	Y	N	unconscious	Died	
N	Y	Y	unconscious	Alive	16
N	N	N	alert	Died	<=8
Y	N	N	unknown	Alive	
			unknown		
			unknown		
Y	Y	Y	sedated	Died	

N	Y	Y	sedated	Alive	
U	N	U	unknown	Died	
U	N	U	unknown	Died	
N	N	N	alert	Alive	
N	N	N	unknown	Died	
U	Y	N	alert	Alive	
N	Y	N	alert	Died	<=8
N	Y	N	sedated	Alive	>32
N	N	N	alert	Alive	>32
U	N	N	alert	Alive	>32
N	N	N	alert	Alive	>32
U	Y	N	alert	Alive	>32
N	Y	N	alert	Alive	>32
Y	Y	N	disorientated	Alive	>32
N	N	N	alert	Alive	<=8
U	N	U	unknown		<=8
			unknown		<=8
N	Y	N	disorientated	Alive	16
Y	Y	N	alert	Alive	<=8
Y	N	N	alert	Alive	<=8
U	N	U	unknown	Died	32
U	Y	Y	sedated	Died	<=8
Y	Y	N	sedated	Alive	<=8
N	Y	N	sedated	Alive	<=8
N	Y	N	disorientated	Died	32
N	N	N	alert	Alive	>32
U	U	U	unknown	Alive	>32
	N	N	alert		>32
N	N	N	alert	Alive	>32
N	Y	N	unknown	Alive	<=8
N	N	N	alert	Alive	>32
N	N	Y	sedated	Alive	<=8
Y	N	N	alert	Alive	<=8
Y	Y	N	alert	Alive	<=8
N	Y	Y	sedated	Alive	32
N	N	N	unknown	Alive	<=8
N	Y	N	disorientated	Died	<=8
N	Y	N	disorientated	Alive	<=8
N	Y	N	sedated	Alive	<=8
U	N	N	unknown	Alive	<=8
N	Y	Y	sedated	Alive	<=8
Y	Y	Y	sedated	Died	<=8
N	N	N	alert	Died	<=8
Y	Y	N	alert	Alive	<=8
N	N	N	unknown	Alive	<=8
N	N	N	unknown	Alive	<=8
N	Y	Y	sedated	Died	>32
N	Y	Y	sedated	Died	>32
U	Y	Y	sedated	Died	<=8
N	N	N	alert	Alive	<=8
N	Y	N	alert	Died	>32
N	Y	N	alert	Died	<=8
N	Y	N	alert	Alive	<=8
N	N	N	alert	Died	16
U	N	Y	disorientated	Died	32
N	Y	N	alert	Alive	<=8

Y	N	U	disorientated	Died	<=8
Y	N	N	unknown	Alive	16
U	N	N	alert	Alive	>32
N	Y	Y	sedated	Alive	16
N	Y	Y	sedated	Alive	<=8
N	N	N	alert	Alive	>32
U	U	U	unknown		<=8
N	Y	Y	sedated	Alive	>32
N	Y	Y	unknown	Died	<=8
U	N	U	unknown		>32
Y	N	N	unknown	Died	<=8
Y	N	N	alert	Alive	>32
N	N	N	unknown	Alive	>32
N	N	N	disorientated	Alive	<=8
U	U	U	unknown		<=8
U	U	U	unknown	Died	<=8
U	N	U	unknown	Alive	<=8
U	N	Y	sedated	Died	<=8
N	N	N	alert	Alive	>32
U	U	U	unknown	Alive	<=8
U	N	N	unknown	Died	>32
Y	Y	N	sedated	Alive	<=8
U	N	N	alert	Alive	>32
N	N	N	alert	Alive	<=8
U	Y	Y	sedated	Died	<=8
Y	N	Y	alert	Alive	<=8
U	N	N	alert	Alive	>32
N	Y	Y	sedated	Died	>32
			unknown		>32
N	Y	N	alert	Alive	
N	Y	Y	sedated	Alive	>32
N	Y	N	alert	Alive	<=8
N	Y	N	alert	Alive	<=8
N	Y	Y	sedated	Died	<=8
Y	Y	Y	sedated	Alive	<=8
U	Y	Y	sedated	Alive	
N	Y	Y	sedated	Alive	
Y	N	N	alert	Alive	
N	Y	N	sedated	Died	
Y	N	N	alert	Died	
N	N	N	disorientated	Alive	
N	Y	N	disorientated	Died	
N	N	N	unknown	Died	16
Y	Y	Y	sedated	Died	<=8
U	U	U	unknown		<=8
U	Y	N	alert	Died	<=8
Y	Y	Y	sedated	Died	<=8
U	N	N	disorientated	Died	<=8
Y	N	N	unconscious	Died	<=8
Y	N	N	alert	Alive	<=8
U	U	U	unknown	Alive	<=8
U	N	N	alert	Alive	<=8
N	Y	N	alert	Alive	<=8
N	Y	N	alert	Died	<=8
N	Y	N	alert	Died	<=8
N	N	N	alert	Alive	>32

N	Y	N	alert	Alive	>32
N	N	Y	sedated	Alive	<=8
N	N	N	unknown	Alive	<=8
N	N	N	alert	Alive	>32
Y	N	Y	sedated	Alive	<=8
U	Y	Y	sedated	Died	<=8
			unknown		
Y	N	U	unknown	Died	<=8
U	N	U	unknown	Alive	<=8
N	Y	N	alert	Alive	<=8
N	N	Y	sedated	Died	<=8
U	N	Y	sedated	Died	32
N	N	Y	sedated	Alive	<=8
U	N	N	alert	Alive	16
U	U	U	unknown	Alive	>32
N	Y	N	alert	Alive	<=8
U	U	U	unknown	Alive	<=8
N	Y	Y	sedated	Alive	<=8
Y	Y	N	disorientated	Alive	<=8
Y	N	N	unknown	Alive	>32
U	N	N	alert	Alive	>32
Y	N	N	unknown	Died	<=8
N	Y	Y	sedated	Alive	<=8
Y	Y	N	alert	Alive	<=8
N	Y	Y	sedated	Alive	>32
U	N	U	unknown		<=8
N	Y	Y	sedated	Alive	<=8
N	Y	N	alert	Died	16
N	Y	N	unknown	Died	<=8
			unknown		>32
U	Y	Y	sedated	Died	>32
Y	Y	Y	alert	Alive	<=8
N	Y	Y	unconscious	Died	<=8
Y	N	N	alert	Alive	>32
Y	Y	N	alert	Alive	>32
N	N	N	unknown	Alive	>32
U	Y	N	disorientated	Died	16
Y	Y	N	disorientated	Died	
Y	N	N	alert	Alive	>32
Y	N	N	alert	Alive	>32
U	U	U	unknown		
U	Y	N	unknown	Alive	<=8
U	Y	N	alert	Died	
N	Y	N	sedated	Died	<=8
U	Y	N	unknown	Died	
N	N	Y	sedated	Alive	>32
N	N	N	disorientated	Died	<=8
Y	Y	Y	sedated	Died	
Y	N	Y	alert	Died	
Y	N	N	unknown	Died	
N	N	N	alert	Alive	
N	N	N	alert	Alive	
U	Y	Y	sedated	Died	<=8
U	N	U	unknown	Died	<=8
N	Y	N	disorientated	Died	<=8
Y	N	N	alert	Alive	<=8

N	N	N	alert	Alive	
U	U	U	unknown	Died	<=8
N	N	N	alert	Died	<=8
U	U	U	unknown	Alive	<=8
U	Y	N	alert	Alive	>32
N	N	N	alert	Alive	<=8
U	N	Y	sedated	Died	>32
N	Y	N	alert	Alive	<=8
N	Y	N	unknown	Alive	>32
Y	Y	N	disorientated	Alive	>32
N	Y	N	alert	Died	<=8
Y	Y	N	alert	Alive	<=8
Y	Y	N	alert	Alive	<=8
N	Y	N	disorientated	Died	<=8
U	Y	N	unconscious	Died	<=8
U	U	U	unknown	Died	<=8
U	U	U	unknown	Died	<=8
			unknown		<=8
U	N	U	unknown	Died	<=8
Y	Y	N	unknown	Alive	<=8
U	N	U	unknown	Died	16
U	U	U	unknown		<=8
U	N	U	unknown	Died	<=8
U	Y	Y	sedated	Died	<=8
U	U	U	unknown	Died	<=8
U	N	U	unknown	Alive	<=8
N	N	N	disorientated	Alive	32
U	N	U	unknown	Died	<=8
N	Y	N	alert	Died	>32
N	Y	N	unknown	Alive	>32
U	Y	Y	sedated	Died	<=8
U	N	N	alert	Died	16
N	Y	N	unknown	Alive	<=8
N	N	N	disorientated	Died	>32
Y	Y	N	alert	Alive	<=8
Y	Y	N	sedated	Alive	>32
U	Y	N	disorientated	Alive	<=8
Y	N	N	unknown	Alive	<=8
U	N	Y	sedated	Alive	<=8
N	Y	Y	sedated	Died	16
N	N	N	alert	Alive	>32
U	N	N	alert	Died	
Y	N	N	disorientated	Alive	
U	Y	N	sedated	Died	
Y	Y	N	disorientated	Died	
U	N	U	disorientated	Died	>32
U	N	U	unknown	Died	
U	Y	N	alert	Died	
Y	N	N	unconscious	Died	
N	N	N	alert	Alive	
N	N	N	alert	Alive	
N	N	N	alert	Alive	<=8
N	Y	N	alert	Alive	
N	N	Y	alert	Alive	
Y	Y	Y	sedated	Died	16
N	N	N	alert	Alive	<=8

N	N	Y	sedated	Died	<=8
Y	N	N	alert	Died	<=8
U	N	U	unknown	Died	<=8
U	N	U	unknown	Died	>32
U	N	U	unknown	Died	>32
N	Y	N	unknown	Alive	>32
U	Y	U	alert	Alive	<=8
			unknown		<=8
U	N	U	unknown	Died	<=8
N	Y	N	unknown	Alive	<=8
U	N	U	unknown	Alive	<=8
N	Y	N	unknown	Alive	>32
N	N	Y	alert	Alive	<=8
N	Y	N	unconscious	Died	<=8
N	Y	Y	unknown	Died	<=8
N	N	N	alert	Alive	>32
U	N	N	disorientated	Died	<=8
Y	U	U	unknown		>32
U	N	N	unknown	Alive	>32
Y	N	N	alert	Alive	<=8
N	Y	N	alert	Alive	
U	Y	N	disorientated	Died	>32
			unknown		
N	N	Y	sedated	Died	
U	U	U	unknown		
			unknown		
N	N	N	alert	Alive	
N	N	N	alert	Alive	
N	N	U	unknown	Alive	
			unknown		
U	Y	N	alert	Alive	
U	N	U	unknown	Died	
U	N	N	alert	Died	
U	Y	Y	alert	Died	
			unknown		
Y	Y	N	unknown	Alive	
U	Y	N	alert	Alive	
U	U	N	unknown	Died	
			unknown		
			unknown		
U	U	U	unknown		
U	Y	N	alert	Alive	
U	U	U	unknown	Alive	
U	U	U	unknown	Died	
Y	N	N	alert	Alive	
N	N	N	alert	Alive	
U	U	U	unknown		
U	U	U	unknown		
N	N	N	alert	Alive	
N	Y	Y	sedated	Died	
N	Y	N	alert	Alive	
N	N	N	alert	Alive	
U	U	U	unknown	Died	
N	N	Y	sedated	Died	
N	N	N	alert	Alive	
Y	N	N	disorientated	Alive	

U	Y	N	disorientated	Alive	
N	N	Y	sedated	Died	
Y	Y	Y	unknown	Died	
N	N	N	alert	Alive	
N	N	N	alert	Alive	
Y	N	N	unknown	Alive	
U	Y	Y	sedated	Died	
U	N	N	unknown	Died	
Y	Y	Y	alert	Died	
N	N	N	alert	Alive	
N	N	N	alert	Alive	
U	N	U	unknown	Died	
U	Y	N	alert	Died	
Y	Y	Y	sedated	Died	
N	Y	Y	sedated	Alive	
U	Y	N	unknown	Alive	
N	N	Y	alert	Alive	
U	U	U	unknown		
U	U	U	unknown	Died	
Y	N	N	alert	Alive	
N	Y	N	alert	Died	
U	N	U	unknown	Died	
U	U	U	unknown		
U	Y	U	alert	Alive	
Y	N	N	unknown	Died	
U	N	N	alert	Alive	
Y	Y	N	disorientated	Died	
Y	N	Y	unconscious	Died	
N	N	Y	alert	Died	
U	U	U	unknown		
U	N	N	unknown	Alive	
U	N	Y	sedated	Died	
U	U	U	unknown		
Y	N	N	alert	Alive	
N	N	Y	unconscious	Died	
U	N	U	unknown		
U	U	U	unknown		
U	N	N	unconscious	Died	
Y	N	N	alert	Alive	
N	N	U	alert	Alive	
U	N	N	disorientated	Alive	
U	U	U	unknown	Alive	
N	N	N	alert	Alive	
U	U	U	unknown		
U	U	U	unknown		
			unknown		
			unknown		
N	Y	N	disorientated	Alive	<=8
N	Y	N	unknown	Alive	>32
U	N	U	unknown		>32
N	N	N	unknown	Alive	16
U	N	N	alert	Alive	>32
N	Y	Y	alert	Alive	
U	N	Y	sedated	Died	>32
U	N	U	unknown		16
N	N	N	alert	Alive	<=8

N	N	Y	sedated	Died	<=8
N	Y	N	unknown	Died	<=8
U	Y	N	disorientated	Died	<=8
Y	Y	N	disorientated	Alive	<=8
N	N	N	disorientated	Alive	<=8
N	Y	Y	sedated	Died	<=8
N	N	Y	sedated	Alive	>32
N	Y	Y	sedated	Died	<=8
N	Y	N	sedated	Died	<=8
Y	N	N	disorientated	Died	<=8
U	N	U	unknown		<=8
N	Y	N	alert	Alive	<=8
Y	Y	N	unknown	Alive	>32
Y	N	N	alert	Alive	<=8
			unknown		<=8
Y	N	N	alert	Alive	<=8
Y	Y	N	disorientated	Died	<=8
N	N	N	unknown	Alive	>32
N	Y	Y	alert	Died	
U	N	U	unknown	Died	<=8
U	Y	Y	sedated	Died	<=8
U	N	U	unknown		<=8
N	Y	N	unknown	Died	>32
Y	Y	Y	sedated	Alive	>32
Y	N	N	alert	Alive	>32
U	U	U	unknown	Died	<=8
Y	Y	Y	unknown	Alive	>32
Y	Y	Y	unknown	Alive	>32
N	N	N	alert	Alive	>32
N	N	N	alert	Alive	<=8
N	Y	N	unknown	Alive	>32
N	Y	Y	alert	Alive	
Y	N	N	alert	Died	
U	Y	Y	sedated	Died	
N	N	Y	alert	Died	
N	Y	N	unknown	Alive	
Y	N	N	alert	Alive	
N	Y	N	alert	Alive	<=8
U	Y	U	unknown	Died	
Y	N	N	alert	Alive	<=8
U	N	U	unknown		<=8
U	Y	N	sedated	Died	<=8
U	Y	Y	sedated	Died	<=8
U	U	U	unknown		>32
Y	Y	Y	disorientated	Died	<=8
U	Y	Y	disorientated	Died	<=8
N	Y	N	alert	Alive	<=8
U	Y	U	unknown	Died	<=8
Y	Y	Y	sedated	Died	<=8
N	Y	Y	sedated	Alive	<=8
U	Y	Y	unconscious	Died	
Y	Y	N	alert	Died	<=8
N	N	Y	alert	Died	<=8
U	N	N	alert	Died	<=8
N	Y	N	alert	Alive	>32
Y	N	N	alert	Alive	<=8

U	N	N	unknown	Died	>32
Y	Y	Y	sedated	Alive	
N	Y	N	disorientated	Alive	>32
U	U	U	unknown		>32
U	N	U	unknown		<=8
Y	Y	N	alert		<=8
U	U	U	unknown	Alive	16
U	U	U	unknown		<=8
U	N	U	unknown	Died	<=8
N	Y	U	alert		>32
Y	U	U	unknown		>32
N	Y	Y	sedated	Died	<=8
U	N	U	unknown	Alive	>32
U	N	N	alert	Died	<=8
N	N	N	alert	Alive	>32
N	Y	Y	sedated	Alive	>32
N	N	Y	unconscious	Died	>32
N	N	N	alert	Alive	<=8
U	Y	N	disorientated	Died	<=8
N	N	N	disorientated	Alive	<=8
Y	Y	Y	unknown	Alive	<=8
U	Y	N	disorientated	Died	>32
N	N	N	alert	Alive	<=8
N	Y	N	sedated	Alive	<=8
N	Y	N	alert	Died	<=8
Y	Y	N	disorientated	Died	<=8
Y	N	N	unknown	Died	<=8
N	N	N	alert	Alive	<=8
Y	N	Y	sedated	Died	
			unknown		
U	Y	N	sedated	Alive	
Y	N	N	disorientated	Died	
			unknown		
N	N	N	alert	Alive	
N	N	N	unknown	Alive	<=8
N	Y	Y	sedated	Died	
N	Y	N	alert	Died	
Y	N	N	alert	Alive	
N	Y	Y	disorientated	Alive	
N	N	N	alert	Alive	
N	N	N	alert	Alive	
N	Y	Y	sedated	Died	
N	N	N	unknown	Alive	
U	N	U	unknown	Alive	
U	U	U	unknown		
N	Y	Y	alert	Died	
U	N	U	unknown		
U	N	U	unknown	Died	
U	N	N	disorientated	Alive	
N	Y	N	alert	Alive	
U	N	U	unknown	Alive	
Y	Y	Y	unconscious	Alive	
U	U	U	unknown	Died	
U	Y	U	unknown	Died	
N	N	N	alert	Alive	
N	N	Y	sedated	Alive	<=8

N	Y	Y	sedated	Alive	<=8
U	Y	N	unknown	Died	<=8
U	N	N	unknown	Alive	<=8
N	Y	N	alert	Died	
Y	Y	N	alert	Alive	>32
N	Y	Y	sedated	Died	<=8
N	Y	N	alert	Alive	<=8
N	N	N	alert	Alive	
N	Y	N	sedated	Alive	
N	Y	N	alert	Died	
U	Y	Y	sedated	Died	
U	U	U	unknown	Died	
U	Y	N	alert	Alive	
Y	N	N	alert	Alive	
U	N	N	disorientated	Died	
U	N	U	unknown	Alive	
U	N	N	alert	Alive	<=8
Y	N	Y	disorientated	Alive	<=8
N	N	N	alert	Alive	<=8
N	N	N	unknown	Alive	>32
Y	N	N	alert	Alive	<=8
N	Y	N	alert	Alive	<=8
N	N	N	unknown	Alive	>32
N	Y	N	disorientated	Alive	<=8
N	Y	N	alert	Alive	
U	N	N	alert	Alive	
U	Y	N	disorientated	Died	
N	N	N	alert	Alive	<=8
N	Y	Y	sedated	Alive	<=8
N	Y	N	unknown	Alive	<=8
N	Y	Y	unknown	Died	<=8
Y	Y	N	alert	Alive	
N	Y	N	disorientated	Alive	
U	N	N	alert	Alive	<=8
N	Y	Y	sedated	Alive	<=8
N	N	N	alert	Alive	<=8
N	Y	N	disorientated	Alive	<=8
N	Y	Y	sedated	Alive	<=8
U	N	N	unknown	Alive	<=8
Y	Y	N	alert	Alive	<=8
N	Y	N	alert	Alive	<=8
U	U	U	unknown	Alive	<=8
N	N	Y	sedated	Alive	<=8
N	Y	Y	unconscious	Died	<=8
N	N	N	alert	Alive	<=8
			unknown		16
N	Y	N	unknown	Alive	>32
Y	Y	N	unknown	Died	
U	N	U	unknown	Died	>32
N	N	N	disorientated	Alive	<=8
			unknown		>32
N	N	N	alert	Alive	<=8
U	N	U	unknown		>32
N	Y	Y	unconscious	Alive	<=8
U	U	U	unknown		>32
U	N	U	unknown	Alive	<=8

Y	N	N	alert	Died	<=8
U	Y	N	disorientated	Died	<=8
U	Y	U	unknown	Died	<=8
U	U	U	unknown	Died	<=8
U	N	U	unknown	Died	<=8
U	N	Y	alert	Died	>32
N	Y	Y	sedated	Died	<=8
N	Y	N	unknown	Died	<=8
Y	N	N	alert	Alive	<=8
N	N	N	unknown	Alive	16
U	N	N	unknown	Died	<=8
Y	N	N	alert	Alive	>32
U	N	Y	sedated	Died	>32
U	N	N	unknown	Died	<=8
N	Y	Y	disorientated	Alive	>32
U	U	U	unknown	Died	>32
U	N	N	disorientated	Died	<=8
U	U	U	unknown		>32
U	N	U	unknown	Died	<=8
N	Y	N	alert	Alive	<=8
N	Y	N	alert	Alive	32
N	Y	N	alert	Alive	>32
N	N	N	unknown	Alive	>32
N	N	N	unknown	Alive	>32
N	N	N	alert	Alive	
U	N	U	unknown	Died	
Y	N	Y	sedated	Alive	
Y	N	N	unknown	Died	
Y	Y	N	sedated	Alive	<=8
U	N	U	unknown	Died	<=8
N	Y	Y	alert	Died	<=8
N	Y	N	alert	Alive	<=8
N	Y	N	alert	Alive	<=8
U	Y	N	alert	Died	<=8
Y	N	N	alert	Alive	
			unknown		
U	Y	N	unknown	Died	<=8
N	Y	N	sedated	Alive	<=8
Y	Y	N	unknown	Alive	>32
U	U	U	unknown		<=8
N	Y	Y	sedated	Alive	
N	Y	Y	alert	Alive	<=8
N	Y	N	alert	Alive	<=8
N	Y	N	alert	Alive	>32
			unknown		
N	Y	Y	sedated	Alive	>32
Y	Y	N	alert	Alive	>32
N	N	N	alert	Died	<=8
N	Y	Y	sedated	Died	<=8
N	Y	N	disorientated	Died	<=8
U	N	U	unknown		<=8
N	Y	U	unknown	Died	<=8
N	N	N	alert	Alive	<=8
U	N	U	unknown		<=8
U	U	U	unknown		>32
U	U	U	unknown		>32

U	U	U	unknown	Alive	>32
U	N	N	unknown	Alive	<=8
U	U	U	unknown	Alive	<=8
N	Y	N	unknown		>32
			unknown		>32
U	U	U	unknown	Died	<=8
U	U	U	unknown	Alive	>32
N	Y	N	alert	Alive	>32
N	N	N	alert	Alive	<=8
N	Y	N	alert	Alive	32
N	Y	Y	sedated	Died	<=8
N	N	N	unknown	Alive	<=8
Y	N	N	disorientated	Alive	>32
Y	N	Y	sedated	Alive	<=8
N	Y	Y	unconscious	Died	
U	Y	N	alert	Alive	
N	Y	N	disorientated	Alive	<=8
N	Y	N	unknown	Alive	<=8
N	Y	N	disorientated	Alive	<=8
N	N	N	alert	Alive	<=8
Y	N	N	alert	Alive	
Y	Y	N	alert	Alive	<=8
U	N	Y	unconscious	Alive	
U	Y	Y	sedated	Died	<=8
U	N	Y	sedated	Died	>32
N	N	N	unknown	Alive	>32
N	N	N	alert	Alive	<=8
Y	Y	N	alert	Died	<=8
U	Y	N	unknown	Died	
N	Y	N	alert	Alive	<=8
N	Y	Y	disorientated	Alive	<=8
Y	Y	Y	unknown	Alive	
Y	N	N	unknown	Died	
Y	Y	N	unconscious	Died	
N	N	N	alert	Alive	<=8
N	Y	N	alert	Alive	<=8
N	Y	N	unknown	Died	>32
N	Y	Y	alert	Alive	>32
N	N	Y	sedated	Alive	<=8
N	N	Y	sedated	Died	<=8
N	N	N	alert	Alive	>32
U	U	U	unknown	Died	>32
N	N	N	alert	Alive	
U	N	U	unknown		<=8
N	N	N	alert	Alive	
N	N	N	unknown	Alive	>32
N	Y	N	unknown	Alive	<=8
U	Y	Y	sedated	Died	<=8
N	N	N	alert	Alive	<=8
N	Y	N	alert	Alive	<=8
N	Y	N	disorientated	Alive	<=8
N	Y	N	disorientated	Died	<=8
N	N	N	unknown	Alive	
N	Y	N	sedated	Alive	
N	N	N	alert	Alive	<=8
Y	N	Y	sedated	Alive	<=8

N	N	N	unknown	Alive	>32
U	Y	Y	sedated	Alive	32
N	N	N	alert	Alive	>32
Y	N	Y	disorientated	Died	<=8
N	Y	N	alert	Alive	<=8
N	Y	N	alert	Alive	<=8
Y	N	Y	sedated	Alive	<=8
N	N	N	alert	Alive	16
Y	Y	N	disorientated	Alive	>32
N	N	N	alert	Alive	>32
N	Y	N	unknown	Alive	>32
U	N	Y	sedated	Alive	>32
N	N	N	unknown	Alive	>32
Y	N	Y	unconscious	Alive	<=8
Y	N	Y	disorientated	Died	<=8
N	N	N	disorientated	Alive	<=8
Y	N	N	alert	Died	>32
Y	U	N	alert	Alive	>32
Y	N	Y	unconscious	Died	<=8
N	N	N	alert	Alive	<=8
N	Y	N	unknown	Died	>32
U	Y	Y	sedated	Died	<=8
N	Y	Y	unknown	Alive	<=8
N	N	N	alert	Alive	>32
N	Y	N	unknown	Alive	>32
N	N	N	unknown	Alive	>32
N	N	N	unknown	Alive	>32
N	Y	Y	sedated	Alive	<=8
N	Y	N	disorientated	Alive	<=8
Y	Y	Y	sedated	Died	>32
N	Y	Y	sedated	Died	<=8
N	Y	N	unknown	Alive	<=8
N	Y	N	alert	Alive	<=8
N	Y	Y	sedated	Alive	<=8
N	N	N	alert	Alive	>32
Y	Y	N	alert	Alive	<=8
N	Y	N	disorientated	Alive	<=8
N	Y	Y	sedated	Alive	<=8
Y	N	N	alert	Alive	<=8
N	N	N	sedated	Alive	<=8
N	Y	N	alert	Alive	<=8
N	N	Y	unknown	Alive	>32
Y	Y	Y	sedated	Alive	<=8
N	Y	N	alert	Alive	>32
N	Y	N	alert	Alive	<=8
N	Y	Y	sedated	Alive	>32
N	N	N	alert	Died	<=8
N	N	N	unknown	Alive	>32
N	N	Y	sedated	Alive	>32
N	N	N	unknown	Alive	>32
N	N	N	unknown	Alive	>32
Y	Y	Y	sedated	Died	<=8
N	N	Y	sedated	Alive	<=8
N	N	Y	sedated	Died	>32
U	Y	N	unknown	Died	<=8
N	N	N	unknown	Alive	>32

N	U	U	unknown		>32
N	N	Y	sedated	Died	<=8
N	Y	N	alert	Alive	<=8
Y	N	N	alert	Alive	<=8
Y	N	Y	sedated	Alive	<=8
Y	Y	N	alert	Alive	<=8
N	Y	N	disorientated	Died	<=8
N	Y	N	disorientated	Alive	<=8
U	Y	Y	sedated	Died	<=8
U	Y	N	disorientated	Died	<=8
U	U	U	unknown		<=8
N	N	N	alert	Alive	<=8
U	Y	U	unknown	Died	<=8
U	U	U	unknown	Died	<=8
			unknown		
Y	N	Y	sedated	Died	
Y	Y	N	alert	Alive	
			unknown		
			unknown		
U	Y	U	unknown	Died	
N	N	Y	sedated	Died	
Y	N	Y	sedated	Alive	
U	Y	N	alert	Alive	
U	U	N	unknown	Died	
U	N	U	unknown	Alive	
U	N	N	unknown	Alive	
N	Y	U	unknown	Died	
U	Y	U	unknown	Died	
U	N	N	alert	Alive	
			unknown		
U	U	U	unknown	Died	
			unknown		
U	U	U	unknown	Died	
N	N	Y	sedated	Alive	
Y	Y	Y	sedated	Alive	
U	U	U	unknown	Alive	
N	Y	N	unknown	Died	
U	N	U	unknown	Died	
Y	N	N	unknown	Died	
Y	U	U	unknown	Died	
U	Y	Y	sedated	Alive	
N	Y	N	alert	Alive	
U	Y	U	unknown	Died	
Y	Y	Y	unconscious	Died	
U	U	U	unknown	Died	
U	U	U	unknown	Died	
U	Y	N	alert	Alive	
			unknown		
			unknown		
U	Y	Y	sedated		
			unknown		
U	U	U	unknown		
Y	Y	Y	unknown	Alive	
			unknown		
U	U	U	unknown	Died	
N	N	U	unknown		

U	N	Y	sedated	Died
			unknown	
U	Y	N	alert	Died
Y	N	N	disorientated	Alive
N	Y	N	alert	Alive
Y	N	N	alert	Died
U	Y	Y	sedated	Alive
N	Y	N	alert	Died
Y	N	N	disorientated	Died
Y	N	Y	sedated	Alive
Y	N	N	unknown	Alive
U	Y	N	unknown	Died
Y	Y	N	unknown	Died
N	Y	N	alert	Alive
U	U	N	disorientated	Died
N	Y	N	unknown	Alive
			unknown	
U	Y	Y	sedated	Alive
N	N	Y	sedated	Alive
			unknown	
U	N	N	disorientated	Alive
Y	N	N	alert	Alive
			unknown	
N	Y	N	alert	Alive
U	Y	U	unknown	Died
			unknown	
N	N	U	unknown	Died
U	N	N	alert	Alive
Y	Y	N	disorientated	Alive
N	N	N	disorientated	Alive
N	N	N	unknown	Alive
N	N	N	alert	Alive
U	Y	N	disorientated	Died
N	N	N	unknown	Alive
N	N	N	unknown	Alive
N	Y	Y	sedated	Died
U	N	N	unknown	Alive
U	Y	U	unknown	Died
N	N	N	unknown	Died
U	U	U	unknown	Died
			unknown	
Y	N	Y	unconscious	Died
U	N	N	disorientated	Alive
N	N	N	unknown	Alive
			unknown	
U	U	U	unknown	Died
			unknown	
			unknown	
U	U	U	unknown	
U	U	U	unknown	Alive
Y	Y	N	alert	Alive
N	Y	Y	sedated	Alive
U	Y	N	unknown	Died
U	N	U	unknown	
Y	N	N	unknown	Alive
U	N	N	unknown	Alive

Y	N	N	unknown	Died	
U	N	U	disorientated	Died	
Y	N	Y	sedated	Died	
U	U	U	unknown		
U	N	U	unknown	Died	
N	Y	Y	sedated	Alive	
Y	Y	N	alert	Alive	
			unknown		
U	U	U	unknown	Alive	
U	N	N	disorientated	Alive	
Y	Y	Y	sedated	Died	
Y	N	N	alert	Alive	
U	Y	U	unknown	Died	
N	Y	Y	sedated	Died	
U	Y	U	unknown	Alive	
U	U	U	unknown	Alive	
N	Y	Y	sedated	Died	
N	N	N	alert	Alive	
U	U	U	unknown	Died	
N	Y	N	alert	Alive	
U	U	U	unknown		
U	U	U	unknown		
Y	Y	N	unknown	Died	
Y	Y	N	alert	Died	
N	N	Y	unknown	Alive	<=8
N	Y	Y	sedated	Alive	<=8
N	Y	Y	sedated	Alive	<=8
Y	N	N	alert	Alive	<=8
Y	N	N	alert	Alive	<=8
N	N	N	alert	Alive	<=8
N	Y	Y	sedated	Alive	<=8
N	N	Y	unknown	Alive	>32
Y	N	N	unknown	Died	>32
Y	Y	N	unknown	Died	<=8
N	Y	Y	sedated	Died	<=8
N	Y	Y	sedated	Died	<=8
Y	N	N	alert	Alive	<=8
N	Y	N	alert	Alive	>32
U	U	U	unknown	Died	<=8
Y	N	N	alert	Alive	>32
N	Y	N	alert	Alive	<=8
Y	Y	Y	sedated	Alive	<=8
N	Y	Y	sedated	Alive	>32
Y	N	N	unknown	Died	<=8
N	N	N	unknown	Alive	>32
N	N	Y	sedated	Alive	<=8
Y	Y	Y	sedated	Alive	<=8
N	Y	Y	sedated	Alive	<=8
U	N	N	alert	Alive	<=8
N	Y	Y	sedated	Alive	<=8
U	N	U	unknown	Died	<=8
			unknown		<=8
U	Y	Y	sedated	Died	<=8
U	U	U	unknown	Alive	<=8
N	N	N	alert	Alive	<=8
N	N	N	alert	Alive	<=8

N	N	Y	sedated	Alive	<=8
U	U	U	unknown		<=8
U	U	U	unknown		>32
U	Y	N	alert	Alive	>32
N	N	Y	sedated	Alive	<=8
N	N	N	alert	Alive	16
N	N	N	disorientated	Alive	<=8
N	Y	N	alert	Alive	<=8
N	N	N	unknown	Alive	<=8
U	U	U	unknown	Died	<=8
U	U	U	unknown		<=8
U	Y	N	disorientated	Alive	<=8
U	U	U	unknown		<=8
U	U	U	unknown		<=8
N	Y	N	alert	Alive	<=8
N	N	N	sedated	Alive	<=8
N	Y	Y	sedated	Alive	<=8
U	U	U	unknown	Died	<=8
U	Y	N	sedated	Alive	<=8
U	Y	N	unknown	Died	>32
U	U	U	unknown		<=8
N	N	N	alert	Alive	<=8
N	N	N	alert	Alive	<=8
N	N	Y	sedated	Alive	<=8
Y	Y	N	alert	Alive	<=8
Y	Y	N	alert	Alive	<=8
N	Y	N	alert	Alive	<=8
N	N	N	alert	Alive	>32
U	N	U	unknown		<=8
N	N	N	alert	Alive	<=8
U	N	N	alert	Alive	<=8
U	U	U	unknown		<=8
U	N	Y	sedated	Died	<=8
N	Y	N	alert	Alive	<=8
N	Y	N	alert	Alive	<=8
U	N	N	alert	Died	<=8
Y	Y	N	unknown	Alive	>32
N	Y	Y	sedated	Alive	
U	Y	N	unknown	Died	
Y	N	N	unknown	Died	
N	Y	Y	sedated	Died	
N	N	N	alert	Alive	<=8
N	Y	N	alert	Died	
N	N	N	alert	Alive	>32
U	Y	N	unknown	Died	
U	N	N	disorientated	Died	<=8
N	N	N	disorientated	Alive	<=8
N	Y	Y	sedated	Alive	>32
N	U	U	unknown	Died	>32
U	Y	Y	sedated	Alive	<=8
Y	Y	U	unknown	Alive	>32
U	U	U	unknown		<=8
U	U	U	unknown		<=8
U	U	U	unknown	Alive	
Y	Y	N	alert	Died	<=8
N	Y	N	unknown	Alive	<=8

U	U	U	unknown	Alive	>32
U	Y	Y	unconscious	Died	<=8
N	N	N	alert	Alive	<=8
Y	Y	N	alert	Died	<=8
N	Y	N	alert	Died	<=8
N	N	Y	alert	Alive	<=8
N	N	N	alert	Alive	<=8
N	N	N	alert	Alive	>32
N	N	N	alert	Alive	<=8
N	Y	Y	sedated	Died	<=8
N	Y	N	alert	Died	<=8
N	N	N	alert	Died	<=8
N	Y	N	alert	Alive	<=8
N	Y	Y	sedated	Alive	>32
N	N	N	alert	Alive	<=8
N	N	Y	sedated	Alive	>32
N	Y	N	unknown	Died	>32
Y	N	Y	sedated	Alive	<=8
Y	N	Y	sedated	Died	<=8
U	Y	N	disorientated	Died	>32
N	Y	N	alert	Alive	<=8
N	Y	N	disorientated	Alive	<=8
Y	N	N	alert	Alive	<=8
U	Y	N	alert	Died	<=8
Y	Y	N	alert	Alive	<=8
Y	N	N	alert	Alive	<=8
N	N	N	alert	Alive	<=8
N	Y	Y	sedated	Died	
N	Y	N	alert	Alive	
U	N	N	unknown	Alive	
N	N	N	disorientated	Died	
U	Y	N	unknown	Died	
U	N	N	alert	Alive	
Y	N	Y	sedated	Died	
N	Y	Y	sedated	Alive	
U	N	N	alert	Alive	
U	U	U	unknown	Died	
N	Y	Y	sedated	Alive	
N	Y	N	unknown	Alive	
N	Y	N	unknown	Alive	
U	N	Y	sedated	Alive	
N	N	N	disorientated	Alive	
Y	N	N	unknown	Died	<=8
N	N	N	alert	Alive	<=8
Y	Y	N	alert	Alive	<=8
Y	N	Y	sedated	Died	<=8
U	U	U	unknown		<=8
U	U	N	alert	Alive	>32
Y	Y	N	alert	Died	<=8
N	Y	N	alert	Alive	<=8
U	Y	N	disorientated	Died	<=8
Y	N	N	alert	Alive	<=8
N	Y	N	unconscious	Died	<=8
N	Y	N	alert	Died	<=8
N	N	N	alert	Alive	<=8
N	N	N	alert	Alive	>32

Y	Y	Y	sedated	Died	<=8
N	Y	N	alert	Died	<=8
N	Y	Y	sedated	Died	<=8
			unknown		>32
N	Y	N	alert	Alive	<=8
U	Y	Y	sedated	Died	>32
N	N	N	alert	Alive	<=8
N	N	N	alert	Alive	
U	U	U	unknown	Alive	<=8
			unknown		<=8
N	U	U	unknown		<=8
N	N	U	unknown	Died	<=8
			unknown		<=8
U	N	U	unknown		<=8
U	U	U	unknown		<=8
N	Y	N	alert	Died	<=8
U	N	U	unknown	Died	<=8
U	N	U	unknown	Alive	<=8
U	U	U	unknown		<=8
N	N	N	alert	Alive	<=8
U	U	U	unknown		16
N	N	N	alert	Alive	16
Y	Y	N	alert	Alive	<=8
U	U	U	unknown		<=8
U	N	U	unknown		>32
U	Y	Y	unconscious	Died	<=8
N	Y	U	unknown	Died	<=8
U	U	U	unknown		<=8
N	U	U	unknown		
U	N	N	unknown	Died	<=8
Y	Y	Y	sedated	Alive	<=8
			unknown		<=8
N	N	Y	sedated	Alive	>32
U	U	U	unknown		<=8
N	N	N	alert	Alive	>32
U	N	N	alert	Alive	<=8
Y	Y	N	alert	Alive	<=8
N	Y	N	disorientated	Alive	<=8
N	Y	N	alert	Alive	<=8
N	N	N	alert	Alive	<=8
N	N	Y	unconscious	Alive	<=8
U	N	U	unknown		<=8
N	Y	N	alert	Alive	<=8
N	N	N	unknown	Alive	<=8
N	Y	N	sedated	Alive	<=8
N	N	N	alert	Alive	<=8
N	N	N	alert	Died	<=8
N	N	N	alert	Died	<=8
N	N	N	alert	Alive	<=8
U	Y	Y	sedated	Died	<=8
N	Y	Y	sedated	Died	>32
Y	N	N	alert	Alive	<=8
N	N	N	unknown	Alive	<=8
N	N	Y	unconscious	Alive	<=8
N	N	N	alert	Alive	<=8
Y	Y	N	alert	Alive	<=8

U	Y	Y	sedated	Died	>32
N	N	N	alert	Alive	>32
U	Y	N	unconscious	Died	<=8
Y	N	N	alert	Died	<=8
Y	Y	Y	sedated	Alive	>32
U	U	U	unknown	Died	>32
N	N	N	alert	Alive	<=8
N	N	N	alert	Died	<=8
N	Y	N	sedated	Died	>32
N	N	N	alert	Alive	>32
N	Y	Y	sedated	Died	<=8
Y	N	N	alert	Alive	>32
Y	N	N	alert	Alive	>32
Y	Y	N	alert	Alive	<=8
N	Y	N	alert	Alive	<=8
N	N	Y	sedated	Alive	>32
Y	Y	N	alert	Alive	<=8
N	Y	N	disorientated	Alive	<=8
N	Y	Y	sedated	Alive	16
Y	Y	Y	sedated	Died	
Y	Y	Y	sedated	Alive	
Y	U	U	unknown	Died	
N	Y	N	alert	Alive	
N	Y	N	alert	Alive	
N	Y	N	alert	Alive	
N	Y	Y	sedated	Alive	>32
N	N	N	alert	Alive	
N	N	N	alert	Alive	
U	N	N	unknown	Alive	
N	Y	U	unknown	Died	
N	U	N	unknown	Died	
N	Y	N	alert	Alive	
U	N	N	unknown	Died	<=8
			unknown		16
N	N	N	unknown	Alive	>32
N	N	N	alert	Alive	<=8
N	Y	N	unknown	Alive	>32
Y	Y	N	unknown	Died	<=8
Y	N	N	disorientated	Died	<=8
U	U	U	unknown	Died	16
U	Y	N	unknown	Died	<=8
N	Y	N	alert	Alive	<=8
N	Y	N	alert	Alive	<=8
U	N	U	unknown	Alive	<=8
U	N	U	unknown	Died	<=8
N	U	N	sedated	Alive	<=8
			unknown		<=8
Y	Y	N	disorientated	Died	<=8
N	Y	N	sedated	Died	<=8
N	N	N	alert	Alive	>32
N	N	Y	sedated	Alive	>32
Y	Y	N	sedated	Died	<=8
Y	N	N	alert	Alive	>32
			unknown		
			unknown		
Y	N	N	alert	Alive	

Y	N	N	alert	Alive	
			unknown		
U	U	U	unknown		
U	N	U	unknown	Alive	
Y	N	Y	sedated	Died	
N	N	N	alert	Alive	
Y	U	U	unknown	Died	
U	U	U	unknown		
Y	N	Y	sedated	Died	
Y	Y	N	alert	Alive	
N	Y	N	alert	Alive	
N	Y	Y	alert	Died	
U	U	U	unknown	Died	
Y	N	N	alert	Alive	
U	U	U	unknown	Died	
N	Y	Y	sedated	Died	
U	U	U	unknown		
Y	N	N	alert	Alive	
Y	Y	Y	sedated	Alive	
U	N	U	unknown	Died	
N	N	N	alert	Alive	
N	U	U	unknown	Alive	
U	N	U	unknown	Died	
Y	N	N	alert	Died	
U	U	U	unknown		
N	N	Y	sedated	Alive	
			unknown		
U	U	U	unknown		
U	U	U	unknown	Died	
N	N	N	alert	Alive	
U	Y	N	unknown	Died	
N	Y	N	alert	Alive	
N	Y	N	alert	Alive	
N	N	N	alert	Alive	
N	N	N	alert	Alive	
N	Y	N	alert	Alive	
N	N	N	alert	Alive	<=8
			unknown		
U	Y	N	alert	Died	
Y	Y	N	alert	Alive	
Y	N	N	alert	Alive	
N	U	U	unknown		
U	Y	Y	sedated	Alive	
U	U	U	unknown	Alive	
			unknown		
N	Y	U	unknown	Alive	
Y	Y	N	alert	Died	
U	U	U	unknown		
N	Y	N	alert	Died	<=8
			unknown		
N	Y	N	disorientated	Died	
			unknown		
Y	Y	N	alert	Alive	
N	N	N	alert	Alive	<=8
N	Y	N	alert	Died	
N	Y	N	sedated	Died	

Y	N	N	alert	Alive	<=8
N	N	Y	sedated	Died	
N	Y	Y	alert	Alive	
N	N	N	alert	Alive	
U	U	U	unknown	Died	<=8
U	U	N	unknown	Died	
N	N	Y	sedated	Alive	
N	N	Y	sedated	Died	
Y	N	Y	sedated	Died	
			unknown		
N	N	N	alert	Alive	<=8
Y	N	N	alert	Alive	<=8
Y	N	Y	sedated	Died	<=8
N	Y	Y	alert	Died	>32
N	N	N	alert	Alive	<=8
Y	N	Y	sedated	Died	>32
N	Y	Y	unconscious	Alive	>32
Y	N	N	alert	Died	<=8
N	N	N	alert	Alive	<=8
N	Y	N	alert	Died	<=8
U	Y	N	unknown	Alive	<=8
Y	N	N	alert	Alive	<=8
			unknown		>32
Y	Y	N	alert	Alive	<=8
N	N	N	alert	Alive	32
Y	N	Y	sedated	Died	<=8
U	U	U	unknown		<=8
N	Y	Y	sedated	Alive	<=8
U	Y	Y	sedated	Died	<=8
U	N	Y	sedated	Died	<=8
Y	N	Y	sedated	Alive	<=8
U	Y	N	alert	Alive	<=8
N	N	N	unknown	Alive	>32
Y	N	N	alert	Alive	<=8
			unknown		<=8
U	Y	N	alert	Died	<=8
U	Y	Y	sedated	Alive	<=8
N	Y	Y	sedated	Died	>32
N	N	N	unknown	Alive	<=8
U	N	U	unknown		<=8
N	N	Y	unconscious	Alive	>32
			unknown		<=8
N	N	N	alert	Alive	<=8
U	N	N	alert	Alive	<=8
Y	N	N	disorientated	Alive	<=8
U	Y	Y	sedated	Died	<=8
U	Y	Y	sedated	Died	>32
Y	N	N	disorientated	Alive	<=8
Y	Y	N	alert	Alive	<=8
Y	Y	N	disorientated	Died	<=8
Y	Y	N	disorientated	Died	<=8
Y	N	Y	unconscious	Alive	<=8
Y	Y	N	alert	Alive	<=8
U	U	U	unknown		<=8
N	Y	N	alert	Alive	<=8
Y	N	N	alert	Alive	<=8

N	Y	Y	sedated	Died	>32
Y	Y	N	disorientated	Died	>32
N	Y	N	unknown	Died	<=8
N	N	Y	unconscious	Alive	<=8
Y	Y	Y	sedated	Alive	<=8
			unknown		<=8
N	N	N	alert	Died	<=8
N	N	Y	sedated	Alive	<=8
N	N	N	alert	Alive	<=8
			unknown		>32
Y	Y	Y	sedated	Alive	>32
U	N	Y	sedated	Died	<=8
N	Y	Y	sedated	Died	<=8
N	Y	N	alert	Alive	
N	Y	N	alert	Alive	>32
N	N	N	alert	Alive	<=8
Y	N	N	alert	Died	<=8
U	Y	N	unknown	Died	<=8
Y	Y	N	alert	Died	<=8
N	Y	N	alert	Alive	>32
			unknown		
			unknown		
			unknown		
			unknown		
U	U	U	unknown	Died	
			unknown		
			unknown		
			unknown		
			unknown		
			unknown		
			unknown		
U	N	U	unknown		
U	U	U	unknown	Died	
			unknown		
			unknown		
			unknown		
			unknown		
			unknown		
			unknown		
			unknown		
			unknown		
			unknown		
			unknown		
			unknown		
			unknown		
			unknown		
			unknown		
			unknown		
N	Y	Y	sedated	Alive	
			unknown		
N	N	N	alert	Alive	
			unknown		
N	Y	Y	sedated	Alive	
N	N	N	alert	Alive	
N	Y	N	alert	Alive	
U	U	U	unknown		

			unknown	
Y	N	N	unknown	Alive
			unknown	
U	Y	U	sedated	Alive
U	U	U	unknown	Died
			unknown	
N	N	N	alert	Alive
			unknown	
N	Y	N	sedated	Died
			unknown	
			unknown	
Y	Y	N	disorientated	Died
N	Y	N	alert	Alive
			unknown	
N	U	N	unknown	Died
			unknown	
N	Y	N	sedated	Alive
N	Y	N	alert	Alive
Y	Y	N	sedated	Died
			unknown	
U	U	U	unknown	Died
Y	N	N	alert	Alive
			unknown	
N	N	N	alert	Alive
			unknown	
Y	Y	N	alert	Alive
N	Y	N	alert	Alive
			unknown	
N	N	N	alert	Alive
			unknown	
			unknown	
			unknown	
			unknown	
			unknown	
			unknown	
			unknown	
N	Y	Y	sedated	Died
Y	Y	N	disorientated	Alive
Y	Y	Y	sedated	Alive
N	Y	Y	sedated	Alive
Y	N	N	unknown	Alive
U	Y	N	unknown	Died
U	N	U	unknown	
U	Y	Y	sedated	Alive
			unknown	
Y	Y	Y	sedated	Died
Y	N	N	unconscious	Died
			unknown	
			unknown	
N	N	N	alert	Alive
N	Y	Y	sedated	Alive
Y	N	N	disorientated	Alive
Y	Y	Y	sedated	Alive
			unknown	
U	U	U	unknown	
N	Y	N	disorientated	Died

			unknown		
Y	Y	Y	sedated	Died	
N	Y	Y	sedated	Alive	
			unknown		
			unknown		>32
U	U	U	unknown		>32
U	N	N	disorientated	Alive	<=8
N	Y	N	disorientated	Alive	<=8
N	N	N	alert	Alive	<=8
U	N	U	unknown		<=8
Y	N	N	alert	Alive	>32
N	Y	N	alert	Died	<=8
Y	Y	Y	alert	Alive	<=8
Y	Y	Y	sedated	Alive	>32
Y	Y	Y	sedated	Died	>32
Y	N	N	alert	Alive	>32
Y	Y	N	alert	Died	>32
Y	Y	N	alert	Alive	>32
Y	Y	N	alert	Died	<=8
N	Y	N	alert	Alive	
N	Y	N	alert	Alive	
N	Y	N	alert	Alive	
U	U	N	unknown	Alive	
N	Y	Y	sedated	Alive	
U	Y	N	alert	Alive	
N	U	U	unknown	Died	
U	Y	Y	sedated	Alive	
U	Y	N	unknown	Alive	
Y	N	N	unknown	Alive	
N	Y	N	alert	Alive	
U	N	U	unknown		
U	N	N	disorientated	Alive	
N	Y	N	disorientated	Died	
N	N	N	alert	Alive	
N	Y	N	alert	Alive	
N	N	Y	sedated	Alive	>32
N	Y	N	alert	Died	<=8
N	N	Y	sedated	Alive	
N	N	N	alert	Alive	<=8
N	N	N	alert	Alive	<=8
			unknown		<=8
N	Y	N	sedated	Died	<=8
N	Y	N	alert	Alive	>32
Y	Y	Y	sedated	Alive	<=8
Y	N	N	alert	Alive	>32
N	N	Y	sedated	Alive	
			unknown		<=8
			unknown		
			unknown		
			unknown		
U	N	N	unknown	Died	
Y	N	N	alert	Alive	
N	N	Y	sedated	Alive	
U	Y	N	alert	Alive	
Y	N	N	alert	Died	
			unknown		

			unknown		
N	Y	N	disorientated	Alive	
N	Y	N	alert	Died	
N	Y	N	alert	Alive	
N	Y	Y	sedated	Alive	
			unknown		
N	Y	Y	sedated	Alive	
N	N	N	disorientated	Alive	
N	Y	N	disorientated	Died	<=8
N	Y	N	alert	Alive	
N	Y	N	unconscious	Died	
N	N	N	alert	Alive	
U	Y	Y	sedated	Alive	
Y	N	N	alert	Alive	
N	Y	N	unconscious	Alive	
N	N	N	disorientated	Died	
Y	N	N	alert	Alive	
N	Y	N	unknown	Died	
N	Y	Y	sedated	Alive	
Y	N	N	unknown	Died	
Y	Y	Y	sedated	Died	
N	Y	N	alert	Alive	
N	Y	N	disorientated	Alive	
N	Y	Y	sedated	Alive	
U	Y	Y	sedated	Died	
U	U	U	unknown		
N	N	N	alert	Alive	
N	Y	N	alert	Alive	
N	Y	N	alert	Alive	
N	Y	Y	sedated	Alive	
N	Y	Y	disorientated	Alive	
N	Y	N	alert	Alive	
N	N	N	alert	Alive	
N	Y	Y	sedated	Died	
Y	N	N	alert	Alive	
N	Y	N	unknown	Died	
Y	Y	Y	sedated	Died	
U	Y	U	sedated	Died	
Y	Y	Y	sedated	Died	
N	Y	Y	sedated	Alive	
Y	Y	Y	sedated	Alive	
U	Y	N	alert	Alive	
N	Y	Y	sedated	Alive	
N	Y	Y	sedated	Alive	
N	Y	Y	sedated	Alive	
			unknown		
N	Y	Y	sedated	Died	
U	Y	Y	sedated	Died	
U	N	N	alert	Alive	
N	Y	N	alert	Alive	
U	Y	U	sedated	Died	
U	Y	U	sedated	Died	
N	Y	N	alert	Alive	
U	N	N	alert	Alive	
U	U	U	unknown	Died	
Y	N	N	alert	Died	

N	N	N	alert	Alive	
U	N	U	unknown	Died	
N	Y	N	alert	Alive	
			unknown		
			unknown		
U	Y	Y	sedated	Died	
			unknown		
U	Y	Y	unconscious	Alive	
			unknown		
N	N	N	alert	Alive	
N	Y	Y	sedated	Alive	>32
			unknown		<=8
			unknown		<=8
N	Y	N	alert	Alive	>32
Y	N	N	alert	Alive	<=8
			unknown		>32
			unknown		16
Y	Y	Y	sedated	Alive	>32
Y	Y	Y	sedated	Alive	<=8
U	Y	Y	sedated	Alive	>32
Y	N	N	alert	Alive	>32
U	N	N	alert	Alive	<=8
			unknown		>32
N	Y	Y	sedated	Alive	>32
Y	Y	Y	sedated	Alive	>32
N	N	N	alert	Alive	<=8
			unknown		<=8
Y	N	N	alert	Died	>32
Y	Y	Y	sedated	Died	<=8
N	N	N	alert	Alive	<=8
Y	Y	Y	sedated	Alive	>32
U	Y	U	disorientated	Died	16
Y	Y	Y	sedated	Died	
Y	N	N	alert	Alive	>32
N	Y	Y	alert	Alive	>32
Y	Y	N	alert	Alive	>32
U	Y	Y	sedated	Died	<=8
			unknown		>32
Y	N	N	alert	Alive	32
N	Y	Y	unconscious	Died	>32
N	N	N	alert	Alive	<=8
Y	N	Y	sedated	Died	<=8
N	Y	N	alert	Alive	>32
Y	Y	N	alert	Alive	>32
U	Y	Y	sedated	Died	
Y	N	N	alert	Alive	>32
Y	Y	N	alert	Died	>32
N	Y	N	alert	Alive	<=8
N	Y	Y	sedated	Alive	<=8
			unknown		<=8
Y	N	N	alert	Alive	<=8
Y	Y	Y	sedated	Alive	>32
Y	Y	N	alert	Died	<=8
N	N	Y	sedated	Died	<=8
Y	N	Y	sedated	Died	16
N	Y	Y	sedated	Alive	>32

N	Y	N	alert	Died	32
Y	Y	N	sedated	Died	>32
Y	Y	Y	unconscious	Died	>32
Y	N	N	alert	Alive	16
N	Y	Y	sedated	Alive	<=8
Y	N	Y	sedated	Alive	<=8
Y	N	N	alert	Died	>32
Y	Y	Y	unconscious	Alive	>32
N	N	Y	sedated	Died	>32
			unknown		<=8
N	Y	Y	sedated	Died	16
N	N	N	alert	Alive	>32
N	N	N	alert	Alive	>32
Y	Y	Y	sedated	Died	>32
U	Y	U	unknown		<=8
U	Y	N	unknown	Died	<=8
Y	Y	Y	sedated	Alive	16
			unknown		<=8
			unknown		<=8
N	Y	Y	sedated	Died	<=8
U	U	U	unknown	Died	<=8
N	Y	Y	sedated	Alive	>32
			unknown		
			unknown		<=8
Y	N	N	alert	Alive	<=8
			unknown		>32
			unknown		>32
N	N	N	alert	Alive	>32
U	N	N	alert	Alive	>32
N	Y	Y	sedated	Alive	>32
N	Y	Y	sedated	Died	<=8
Y	Y	Y	sedated	Died	<=8
			unknown		>32
Y	N	N	alert	Alive	<=8
Y	N	Y	sedated	Died	>32
Y	Y	N	alert	Alive	>32
Y	N	N	disorientated	Died	<=8
			unknown		16
Y	Y	Y	sedated	Died	<=8
N	Y	Y	sedated	Alive	<=8
N	Y	Y	sedated	Died	<=8
N	Y	N	alert	Alive	<=8
			unknown		>32
N	N	N	alert	Alive	>32
Y	N	N	alert	Alive	>32
Y	Y	Y	sedated	Died	>32
N	Y	Y	sedated	Died	<=8
N	Y	N	alert	Alive	>32
N	N	N	disorientated	Died	>32
Y	Y	Y	sedated	Died	>32
N	Y	Y	sedated	Alive	>32
			unknown		>32
N	Y	Y	sedated	Alive	<=8
N	N	N	alert	Alive	<=8
N	Y	Y	sedated	Died	<=8
Y	Y	Y	sedated	Died	16

U	Y	N	alert	Alive	<=8
Y	N	N	alert	Alive	<=8
N	N	N	alert	Alive	<=8
Y	N	Y	sedated	Died	
N	N	N	alert	Alive	
Y	N	N	alert	Alive	>32
Y	Y	Y	unknown	Alive	<=8
Y	N	N	alert	Alive	<=8
Y	Y	N	alert	Alive	>32
			unknown		>32
Y	Y	N	unconscious	Died	<=8
Y	Y	Y	sedated	Died	>32
U	N	N	alert	Alive	<=8
			unknown		>32
			unknown		<=8
Y	Y	N	alert	Died	>32
Y	N	N	alert	Alive	>32
U	Y	Y	sedated	Alive	>32
Y	N	N	alert	Alive	>32
			unknown		>32
Y	N	N	unknown	Alive	>32
Y	N	N	alert	Died	<=8
U	N	N	alert	Alive	>32
N	Y	Y	sedated	Alive	<=8
Y	Y	Y	sedated	Alive	<=8
N	N	N	alert	Alive	>32
Y	Y	N	sedated	Died	<=8
Y	Y	N	alert	Died	>32
Y	Y	N	alert	Died	>32
Y	Y	Y	unconscious	Died	>32
			unknown		>32
Y	N	N	alert	Alive	>32
Y	N	N	alert	Alive	<=8
Y	Y	Y	sedated	Alive	>32
N	N	N	alert	Died	>32
Y	N	N	alert	Alive	<=8
Y	Y	Y	sedated	Died	<=8
N	Y	N	alert	Alive	>32
N	N	N	alert	Alive	>32
Y	N	N	alert	Alive	32
Y	N	N	disorientated	Died	>32
N	N	N	alert	Died	>32
N	Y	Y	sedated	Alive	>32
U	Y	U	unknown	Died	>32
Y	Y	Y	sedated	Died	>32
Y	N	N	alert	Alive	>32
			unknown		>32
N	N	Y	sedated	Died	<=8
N	Y	Y	sedated	Alive	
Y	Y	Y	sedated	Alive	>32
N	Y	Y	sedated	Alive	>32
Y	N	N	sedated	Alive	<=8
			unknown		<=8
Y	Y	Y	sedated	Died	>32
Y	N	N	disorientated	Died	<=8
Y	Y	Y	sedated	Died	>32

U	Y	Y	sedated	Died	<=8
N	Y	N	alert	Died	>32
Y	Y	Y	sedated	Alive	>32
N	N	N	alert	Alive	>32
Y	N	Y	disorientated	Alive	<=8
Y	N	N	alert		<=8
Y	N	N	alert	Alive	>32
Y	N	N	alert	Alive	>32
N	N	N	alert	Died	
Y	N	Y	sedated	Alive	<=8
N	N	N	alert	Alive	<=8
Y	N	Y	sedated	Alive	32
Y	N	N	alert	Died	<=8
U	N	N	disorientated	Alive	<=8
Y	Y	N	disorientated	Died	<=8
Y	Y	N	alert	Alive	<=8
Y	N	N	alert	Alive	<=8
Y	N	N	alert	Alive	<=8
N	N	N	alert	Died	<=8
Y	Y	N	alert	Died	<=8
Y	Y	N	disorientated	Died	16
N	N	Y	unconscious	Died	16
U	U	U	unknown		>32
N	N	Y	sedated	Died	16
N	N	N	alert	Died	<=8
N	Y	Y	sedated	Alive	<=8
Y	Y	N	alert	Died	>32
Y	N	N	alert	Alive	16
Y	Y	N	alert	Alive	>32
N	N	N	alert	Alive	<=8
Y	N	Y	sedated	Died	<=8
			unknown		<=8
			unknown		>32
Y	Y	N	alert	Died	>32
Y	N	Y	sedated	Died	>32
Y	Y	N	alert	Alive	>32
Y	N	N	alert	Alive	>32
			unknown		>32
N	Y	N	alert	Alive	<=8
Y	N	Y	alert	Alive	>32
Y	N	N	alert	Died	<=8
Y	N	N	alert	Alive	<=8
Y	Y	N	unknown	Died	>32
N	Y	Y	alert	Alive	<=8
N	Y	Y	sedated	Alive	>32
Y	Y	N	disorientated	Died	>32
			unknown		<=8
Y	Y	Y	sedated	Died	
Y	Y	N	alert	Alive	
Y	Y	Y	sedated	Alive	>32
Y	Y	Y	sedated	Alive	>32
Y	Y	Y	sedated	Died	>32
Y	Y	Y	sedated	Died	>32
N	Y	Y	sedated	Alive	>32
Y	Y	Y	sedated	Alive	<=8
			unknown		

			unknown	
			unknown	
N	Y	N	unknown	Died
N	Y	Y	alert	Alive
N	Y	Y	sedated	Alive
N	N	N	alert	Alive
Y	N	Y	sedated	Alive
N	N	N	alert	Alive
N	Y	N	alert	Alive
N	N	N	alert	Died
N	N	N	alert	Alive
Y	N	N	alert	Alive
N	Y	U	unknown	
			unknown	
N	Y	N	alert	Alive
Y	Y	Y	sedated	Died
			unknown	
Y	N	N	alert	Alive
			unknown	
N	N	N	alert	Alive
U	U	U	unknown	Died
N	N	N	disorientated	Alive
			unknown	
Y	Y	Y	sedated	Alive
N	N	N	alert	Alive
Y	Y	N	alert	Died
Y	Y	Y	sedated	Died
Y	Y	Y	sedated	Alive
			unknown	
N	N	Y	sedated	Alive
Y	N	N	alert	Alive
N	N	N	unknown	Died
Y	Y	N	alert	Alive
U	Y	Y	sedated	Died
U	U	N	sedated	Died
N	Y	Y	unconscious	Died
			unknown	
Y	N	N	unknown	Alive
N	Y	N	alert	Died
			unknown	
U	Y	N	alert	Alive
Y	Y	N	alert	Alive
N	Y	N	disorientated	Alive
N	Y	Y	sedated	Alive
Y	Y	N	disorientated	Died
N	Y	Y	sedated	Alive
Y	Y	N	disorientated	Died
U	U	U	unknown	Died
Y	N	N	alert	Died
U	N	N	alert	Alive
Y	Y	N	alert	Died
Y	N	N	alert	Alive
Y	Y	Y	sedated	Died
N	N	Y	sedated	Died
			unknown	
Y	Y	N	disorientated	Died

U	U	U	unknown		
N	N	N	alert	Alive	
N	U	N	alert	Alive	
N	N	N	alert	Alive	
Y	N	N	alert	Alive	
Y	N	N	alert	Alive	
U	Y	N	unknown	Died	
Y	U	U	unknown	Died	
U	U	U	unknown		
			unknown		
Y	N	N	alert	Alive	
Y	N	Y	sedated		
Y	Y	Y	alert	Alive	
Y	Y	N	alert	Alive	
U	Y	N	sedated	Died	
N	Y	N	alert	Alive	
			unknown		
Y	N	N	alert	Alive	
			unknown		
Y	Y	Y	sedated	Alive	
N	Y	Y	unconscious	Died	
N	Y	Y	sedated	Died	
			unknown		
			unknown		
N	Y	Y	unconscious	Alive	
Y	N	Y	sedated	Died	
			unknown		
U	N	N	unknown	Alive	
			unknown		
N	Y	Y	sedated	Alive	
N	Y	N	alert	Alive	
			unknown		
Y	N	N	alert	Alive	
N	N	U	unknown		
			unknown		
N	N	Y	sedated	Alive	
N	N	U	unknown	Alive	
Y	Y	N	disorientated	Died	
U	N	U	alert	Alive	
			unknown		
			unknown		
U	U	U	unknown		
N	N	N	alert	Died	
			unknown		
Y	N	N	alert	Died	
N	N	N	alert	Alive	
Y	N	N	alert	Alive	
Y	N	N	alert	Alive	
Y	Y	Y	sedated	Died	
Y	N	Y	sedated	Alive	>32
Y	Y	N	alert	Died	
Y	N	N	alert	Alive	
Y	U	U	disorientated	Died	
N	N	N	alert	Alive	
Y	N	N	alert	Alive	
Y	Y	N	alert	Alive	

N	Y	Y	sedated	Died	
U	U	U	unknown	Alive	
U	Y	U	unknown	Alive	
N	N	N	alert	Alive	
N	Y	Y	sedated	Died	
			unknown		
			unknown		
Y	Y	Y	sedated	Alive	
Y	Y	Y	sedated	Died	
N	N	N	alert	Alive	
U	N	N	alert	Alive	
Y	Y	N	alert	Alive	
Y	Y	N	alert	Alive	
			unknown		<=8
U	U	U	unknown	Died	32
Y	Y	N	disorientated	Died	<=8
Y	Y	Y	unknown	Died	<=8
Y	N	N	alert	Died	<=8
Y	N	N	alert	Died	<=8
Y	N	N	alert	Alive	<=8
Y	Y	Y	sedated	Alive	<=8
Y	Y	N	alert	Alive	<=8
Y	Y	N	unconscious	Died	<=8
Y	N	Y	sedated	Alive	<=8
U	U	U	unknown		>32
Y	Y	Y	sedated	Died	>32
			unknown		<=8
Y	Y	Y	sedated	Died	
Y	Y	Y	sedated	Alive	
N	N	N	unknown	Alive	
			unknown		
N	Y	N	sedated	Died	
N	N	N	alert	Died	
N	N	N	alert	Alive	
Y	Y	Y	sedated	Alive	
Y	Y	Y	sedated	Alive	
N	Y	N	alert	Alive	
N	N	N	alert	Alive	
N	N	N	alert	Alive	
N	N	N	alert	Alive	
U	U	U	unknown	Died	
U	Y	N	alert	Alive	
N	Y	Y	sedated	Died	
N	Y	Y	sedated	Died	
Y	U	N	unknown	Died	
Y	Y	Y	sedated	Alive	
N	N	N	alert	Alive	
Y	Y	Y	sedated	Alive	
N	N	N	alert	Alive	
Y	N	N	unknown	Died	
Y	N	N	alert	Died	
Y	Y	N	alert	Died	
N	Y	Y	sedated	Alive	
Y	N	N	alert	Alive	
Y	Y	Y	sedated	Died	
N	Y	N	alert	Alive	

U	N	N	alert	Alive	
N	Y	N	alert	Alive	
N	Y	N	alert	Alive	
N	N	N	alert	Alive	
N	N	N	alert	Alive	
Y	N	Y	sedated	Died	
Y	N	N	alert	Alive	
Y	N	N	alert	Alive	
Y	Y	Y	sedated	Alive	
Y	N	N	alert	Died	
			unknown		
Y	Y	Y	unconscious	Died	
Y	Y	Y	unconscious	Alive	
N	Y	N	alert	Alive	
U	N	N	alert	Alive	<=8
Y	N	N	alert	Alive	
N	N	N	alert	Alive	
Y	Y	Y	sedated	Alive	
Y	Y	Y	sedated	Died	
Y	N	N	alert	Alive	
Y	N	N	alert	Alive	
N	Y	Y	sedated	Died	
Y	Y	Y	sedated	Died	>32
U	Y	N	unknown	Alive	
Y	Y	Y	sedated	Alive	
N	Y	U	unknown	Died	
Y	Y	Y	sedated	Alive	
Y	N	N	alert	Died	
Y	Y	N	alert	Alive	
			unknown		
Y	Y	Y	sedated	Died	
Y	Y	Y	unknown	Died	
Y	Y	Y	sedated	Died	
Y	Y	N	alert	Alive	
Y	Y	Y	sedated	Alive	>32
			unknown		
Y	Y	N	disorientated	Alive	
Y	N	N	alert	Alive	
			unknown		>32
Y	Y	N	alert	Died	16
Y	Y	N	alert	Died	>32
Y	Y	Y	sedated	Died	<=8
N	N	N	alert	Alive	>32
Y	Y	N	alert	Alive	>32
			unknown		>32
Y	Y	Y	sedated	Alive	>32
U	N	U	unknown	Died	<=8
N	Y	Y	sedated	Died	>32
Y	Y	Y	sedated	Alive	>32
Y	N	N	alert	Alive	>32
Y	N	N	alert	Alive	>32
Y	Y	Y	sedated	Alive	<=8
Y	N	N	alert	Alive	>32
U	U	U	unknown	Died	32
Y	N	N	alert	Died	<=8
N	Y	N	alert	Alive	<=8

Y	N	N	alert	Alive	>32
Y	N	Y	sedated	Alive	<=8
Y	Y	Y	sedated	Alive	<=8
Y	Y	Y	sedated	Alive	>32
Y	Y	Y	sedated	Alive	>32
			unknown		>32
Y	N	N	alert	Alive	>32
N	N	N	alert	Alive	16
N	N	N	alert	Alive	>32
Y	Y	Y	sedated	Died	<=8
N	N	N	alert	Alive	>32
Y	N	N	alert	Alive	16
Y	N	Y	sedated	Died	<=8
Y	Y	N	alert	Alive	<=8
N	Y	N	disorientated	Alive	>32
Y	N	N	alert	Alive	>32
Y	N	N	alert	Alive	>32
Y	Y	N	alert	Alive	16
N	Y	Y	sedated	Alive	>32
Y	Y	N	alert	Alive	>32
Y	N	N	alert	Died	>32
Y	Y	Y	unconscious	Died	<=8
N	N	N	alert	Alive	>32
Y	N	N	alert	Alive	>32
Y	Y	N	disorientated	Died	>32
Y	Y	Y	sedated	Died	>32
Y	Y	N	alert	Alive	<=8
Y	N	N	alert	Alive	>32
			unknown		>32
N	Y	Y	sedated	Alive	<=8
			unknown		<=8
Y	Y	Y	alert	Alive	<=8
Y	Y	Y	sedated	Alive	>32
N	Y	Y	alert	Alive	>32
Y	N	N	alert	Alive	<=8
Y	Y	Y	sedated	Alive	>32
Y	N	Y	alert	Alive	<=8
Y	N	N	alert	Alive	<=8
Y	N	Y	sedated	Died	<=8
N	N	Y	sedated	Died	<=8
Y	N	N	alert	Alive	<=8
N	N	N	alert	Alive	>32
N	N	N	alert	Alive	<=8
N	Y	Y	sedated	Alive	<=8
N	N	N	alert	Alive	>32
Y	N	Y	sedated	Alive	<=8
N	N	Y	sedated	Alive	<=8
N	Y	Y	sedated	Alive	>32
N	N	Y	sedated	Died	<=8
Y	Y	Y	sedated	Died	<=8
Y	N	N	unknown	Alive	<=8
			unknown		<=8
Y	N	N	alert	Alive	<=8
Y	Y	N	alert	Alive	>32
N	Y	N	alert	Alive	<=8
Y	Y	N	unknown	Alive	

			unknown		
U	Y	N	sedated	Died	32
U	U	U	unknown		
U	N	U	unknown	Alive	
U	U	U	unknown		
U	N	U	unknown		
N	N	N	unconscious	Died	
Y	Y	Y	sedated	Died	
Y	N	Y	sedated	Died	
N	N	N	alert	Alive	
U	U	U	unknown	Died	
U	U	U	unknown	Died	
Y	Y	Y	sedated	Died	
N	Y	N	alert	Alive	
Y	Y	Y	sedated	Died	
Y	N	N	unknown	Alive	
Y	Y	N	alert	Died	
Y	Y	Y	sedated	Alive	
N	Y	U	unknown	Died	
N	Y	N	disorientated	Died	
U	Y	N	unknown	Died	
U	U	U	unknown		
N	Y	U	unknown		
Y	Y	N	alert	Died	
U	N	U	unknown		
N	N	Y	sedated	Died	
U	N	U	unknown	Died	
U	U	U	unknown		
N	Y	N	alert	Died	
Y	N	N	alert	Alive	
			unknown		
N	N	N	alert	Alive	
Y	N	Y	unknown	Died	
Y	Y	N	alert	Died	
U	U	U	unknown		
Y	Y	Y	sedated	Alive	
U	U	U	unknown		<=8
N	Y	U	alert	Alive	
N	Y	N	disorientated	Alive	
Y	Y	Y	sedated	Died	
			unknown		
U	U	U	unknown		
Y	N	Y	sedated	Died	
U	N	N	alert	Alive	
U	N	U	alert	Alive	
N	Y	U	unknown	Died	
U	U	U	unknown		
U	N	U	alert	Alive	
Y	Y	Y	sedated	Alive	
Y	N	N	alert	Alive	
Y	Y	N	alert	Died	
U	U	U	unknown		
N	N	N	alert	Died	
			unknown		
N	Y	Y	sedated	Alive	
Y	N	N	alert	Alive	

N	N	N	alert	Died	
U	N	U	unknown		
N	Y	N	alert	Alive	
N	Y	Y	sedated	Alive	
Y	Y	Y	alert	Died	
Y	Y	Y	sedated	Alive	
N	N	Y	sedated	Died	
U	Y	N	sedated	Died	<=8
N	N	N	alert	Died	
U	N	U	unknown		16
Y	N	Y	sedated	Alive	
N	Y	N	alert	Alive	
U	Y	Y	sedated	Died	
Y	N	N	disorientated	Alive	
U	N	N	unknown	Alive	
Y	Y	Y	sedated	Alive	
Y	Y	Y	disorientated	Died	
N	N	N	alert	Alive	
Y	Y	N	disorientated	Died	
Y	N	Y	sedated	Died	
			unknown		
U	U	U	unknown	Died	
N	N	N	unknown	Alive	
Y	Y	Y	sedated	Alive	
			unknown		
Y	N	N	alert	Alive	
			unknown		
Y	Y	N	disorientated	Died	
N	N	U	unknown	Alive	
N	Y	N	alert	Alive	
Y	Y	Y	sedated	Alive	
N	Y	Y	sedated	Alive	
Y	Y	N	unconscious	Died	
Y	Y	Y	sedated	Died	
Y	Y	N	disorientated	Died	
Y	Y	Y	sedated	Alive	
Y	N	N	alert	Alive	
N	Y	N	disorientated	Died	
U	Y	N	alert	Died	
U	N	Y	sedated	Died	
Y	Y	Y	sedated	Died	
N	Y	N	unconscious	Died	<=8
U	U	U	unknown		<=8
			unknown		
N	N	N	alert	Alive	>32
			unknown		<=8
			unknown		
N	Y	Y	sedated	Died	
Y	N	N	alert	Died	
			unknown		
			unknown		
U	N	N	alert	Died	
N	Y	N	alert	Alive	
Y	N	Y	sedated	Died	
			unknown		
			unknown		

			unknown		
U	Y	N	alert	Died	
N	N	N	alert	Alive	
Y	N	N	alert	Died	<=8
U	Y	N	alert	Alive	
Y	Y	N	alert	Alive	>32
Y	Y	Y	sedated	Died	<=8
Y	Y	Y	sedated	Alive	<=8
Y	N	N	alert	Alive	
N	N	N	unknown	Died	<=8
N	Y	N	unknown	Alive	>32
Y	Y	Y	sedated	Alive	>32
Y	N	N	alert	Alive	<=8
Y	N	Y	sedated	Alive	<=8
N	Y	Y	sedated	Alive	<=8
Y	Y	N	unconscious	Died	<=8
Y	Y	Y	sedated	Alive	>32
Y	Y	N	disorientated	Died	<=8
			unknown		<=8
Y	N	N	alert	Alive	<=8
			unknown		<=8
Y	N	N	alert	Died	32
Y	Y	N	alert	Alive	<=8
N	Y	Y	disorientated	Alive	>32
N	Y	Y	sedated	Alive	>32
Y	N	N	alert	Alive	<=8
			unknown		<=8
Y	N	N	alert	Alive	<=8
			unknown		<=8
Y	Y	N	alert	Alive	<=8
N	Y	Y	sedated	Died	>32
			unknown		<=8
Y	N	N	alert	Alive	<=8
Y	Y	Y	sedated	Alive	<=8
Y	Y	N	alert	Died	<=8
N	Y	N	sedated	Alive	<=8
Y	Y	Y	sedated	Died	>32
Y	N	N	alert	Alive	>32
			unknown		<=8
			unknown		>32
N	Y	Y	alert	Alive	>32
Y	N	Y	sedated	Died	>32
Y	N	N	alert	Alive	<=8
			unknown		>32
Y	N	N	alert	Alive	<=8
			unknown		<=8
U	N	N	unknown	Alive	<=8
N	Y	N	alert	Alive	<=8
Y	N	N	disorientated	Alive	<=8
Y	N	N	disorientated	Alive	<=8
Y	Y	N	alert	Alive	<=8
Y	N	N	alert	Alive	<=8
U	N	Y	unconscious	Died	<=8
Y	N	N	alert	Died	<=8
Y	Y	N	alert	Alive	>32
Y	N	N	alert	Alive	>32

Y	N	N	disorientated	Alive	>32
Y	Y	Y	sedated	Alive	>32
Y	N	N	alert	Alive	
N	N	N	alert	Alive	<=8
Y	N	N	alert	Died	<=8
Y	N	N	alert	Died	<=8
Y	Y	Y	unconscious	Died	
			unknown		
			unknown		
N	Y	Y	sedated	Died	
N	N	N	alert	Died	
			unknown		
			unknown		
			unknown		
			unknown		
			unknown		
			unknown		
			unknown		
Y	Y	Y	sedated	Alive	
			unknown		
			unknown		
Y	Y	N	unknown	Died	
			unknown		
Y	N	N	alert	Died	
			unknown		
			unknown		
			unknown		
			unknown		
N	N	Y	sedated	Died	
			unknown		
			unknown		
Y	N	N	alert	Alive	
Y	Y	N	disorientated	Died	
N	N	N	unknown	Alive	
N	Y	Y	sedated	Died	
			unknown		
Y	Y	Y	disorientated	Alive	
N	N	N	alert	Alive	
			unknown		
N	N	Y	sedated	Died	
N	Y	N	disorientated	Alive	
N	N	N	alert	Alive	
Y	Y	N	unknown	Alive	
Y	Y	Y	unconscious	Died	
N	Y	N	alert	Alive	
			unknown		
N	Y	N	disorientated	Alive	
Y	N	N	alert	Alive	
N	Y	Y	sedated	Alive	
N	N	N	disorientated	Died	
U	N	N	alert	Alive	
			unknown		
N	Y	Y	unconscious	Alive	
			unknown		
Y	Y	N	disorientated	Died	
Y	Y	N	unknown	Alive	

			unknown	
			unknown	
Y	Y	N	sedated	Died
			unknown	
N	N	N	disorientated	Alive
Y	N	N	unknown	Died
N	Y	N	alert	Alive
			unknown	
Y	Y	N	unknown	Alive
N	Y	Y	alert	Alive
N	Y	Y	alert	Died
Y	Y	N	unknown	Alive
N	N	N	alert	Alive
			unknown	
			unknown	
Y	Y	N	disorientated	Alive
			unknown	
			unknown	
Y	Y	Y	sedated	Alive
N	Y	Y	sedated	Alive
N	Y	N	disorientated	Died
Y	Y	Y	sedated	Died
N	Y	Y	sedated	Died
N	Y	N	disorientated	Alive
N	Y	N	alert	Alive
N	N	N	alert	Alive
			unknown	
Y	N	N	alert	Alive
Y	Y	Y	sedated	Alive
			unknown	
			unknown	
			unknown	
Y	Y	Y	sedated	Died
N	Y	Y	sedated	Alive
			unknown	
Y	Y	N	disorientated	Alive
U	N	N	alert	Alive
N	N	Y	alert	Alive
			unknown	
Y	Y	N	unconscious	Died
Y	N	Y	unknown	Alive
N	Y	Y	sedated	Alive
Y	N	N	alert	Alive
Y	Y	Y	sedated	Died
Y	N	Y	unknown	Died
			unknown	
N	Y	Y	sedated	Died
Y	N	N	unknown	Died
			unknown	
U	Y	N	unknown	Died
			unknown	
Y	Y	Y	sedated	Died
N	Y	N	disorientated	Died
Y	N	U	unknown	Alive
N	Y	Y	sedated	Died
N	N	N	disorientated	Alive

			unknown		
Y	Y	N	alert	Alive	
Y	Y	Y	sedated	Alive	
N	N	Y	sedated	Died	
N	N	N	alert	Alive	
Y	N	N	alert	Died	
			unknown		
			unknown		
			unknown		
Y	N	N	alert	Alive	
			unknown		
N	Y	Y	sedated	Alive	
			unknown		
Y	Y	Y	disorientated	Died	
			unknown		
			unknown		
Y	Y	N	alert	Alive	
N	Y	N	alert	Alive	
N	N	Y	sedated	Alive	
Y	Y	Y	sedated	Died	
N	N	N	alert	Alive	
			unknown		
U	U	U	unknown		
			unknown		
N	N	N	unknown	Died	
N	Y	U	unknown	Died	
Y	Y	N	alert	Alive	
N	Y	U	unknown	Died	
Y	Y	N	disorientated	Alive	
Y	Y	N	disorientated	Alive	
N	Y	Y	sedated	Died	<=8
N	Y	Y	alert	Died	<=8
Y	N	N	alert	Alive	<=8
N	Y	Y	sedated	Alive	<=8
			unknown		<=8
			unknown		<=8
Y	Y	Y	sedated	Alive	<=8
Y	Y	Y	sedated	Alive	<=8
Y	N	N	alert	Alive	
Y	Y	N	alert	Alive	
Y	N	Y	sedated	Died	
N	Y	N	alert	Alive	
N	N	N	disorientated	Died	<=8
Y	N	N	alert	Alive	
N	N	N	alert	Alive	
U	U	U	unknown		
Y	Y	N	disorientated	Died	
			unknown		<=8
			unknown		>32
Y	Y	Y	sedated	Died	>32
N	Y	Y	sedated	Died	>32
N	N	N	alert	Alive	<=8
			unknown		16
Y	Y	Y	alert	Alive	<=8
U	Y	N	alert	Alive	>32
U	Y	Y	sedated	Died	<=8

Y	Y	N	alert	Died	32
U	U	U	unknown		<=8
			unknown		<=8
U	U	U	unknown		<=8
N	Y	Y	sedated	Alive	<=8
U	U	U	unknown		<=8
Y	Y	Y	sedated	Alive	<=8
Y	Y	Y	sedated	Alive	>32
Y	Y	Y	sedated	Alive	<=8
U	N	N	alert	Alive	<=8
N	Y	Y	sedated	Alive	<=8
N	Y	Y	sedated	Alive	<=8
N	Y	Y	sedated	Died	>32
N	N	N	alert	Alive	<=8
N	N	N	alert	Alive	>32
N	Y	N	alert	Alive	>32
U	Y	N	disorientated	Alive	>32
N	N	N	alert	Alive	>32
Y	N	N	alert	Alive	>32
Y	Y	Y	sedated	Alive	>32
Y	N	N	alert	Alive	<=8
U	Y	Y	sedated	Alive	<=8
Y	Y	N	alert	Alive	
N	Y	N	alert	Alive	<=8
N	Y	Y	sedated	Died	<=8
N	Y	Y	sedated	Died	<=8
Y	Y	N	disorientated	Alive	<=8
			unknown		<=8
Y	Y	Y	sedated	Died	<=8
			unknown		<=8
			unknown		<=8
N	Y	N	alert	Alive	>32
N	N	N	disorientated	Died	<=8
Y	N	N	alert	Alive	<=8
Y	Y	N	unknown	Died	<=8
Y	Y	N	alert	Alive	<=8
Y	N	N	unknown	Died	<=8
N	Y	Y	sedated	Alive	<=8
			unknown		<=8
			unknown		>32
			unknown		>32
N	Y	N	alert	Alive	16
U	U	U	unknown		<=8
U	N	U	unknown	Died	<=8
			unknown		<=8
U	N	U	unknown	Alive	<=8
			unknown		<=8
N	N	U	unknown	Died	<=8
Y	Y	N	sedated	Alive	<=8
Y	Y	Y	sedated	Died	<=8
N	Y	N	unconscious	Alive	<=8
N	Y	N	alert	Alive	32
N	Y	N	alert	Alive	<=8
U	U	U	unknown		<=8
N	Y	Y	unknown	Alive	<=8
Y	N	Y	sedated	Alive	<=8

			unknown		<=8
Y	N	Y	sedated	Died	<=8
N	N	N	unknown	Alive	>32
N	N	Y	sedated	Alive	<=8
			unknown		
			unknown		
N	N	Y	sedated	Alive	
U	N	U	unknown	Alive	
N	Y	Y	alert	Alive	
U	Y	Y	sedated	Alive	
U	N	U	unknown	Alive	
N	Y	N	sedated	Alive	
N	N	N	alert	Alive	
Y	N	Y	sedated	Died	
U	N	U	unknown		
			unknown		
			unknown		
			unknown		
			unknown		
			unknown		
U	U	U	unknown	Alive	
N	N	N	alert	Alive	
N	Y	Y	sedated	Died	
N	N	N	alert	Alive	
Y	Y	N	alert	Alive	
Y	N	N	alert	Alive	
N	Y	N	alert	Died	
Y	N	N	alert	Alive	
N	N	N	unknown	Alive	
Y	Y	N	alert	Alive	
Y	Y	N	alert	Alive	
Y	N	N	alert	Alive	
U	N	N	unconscious	Alive	
N	Y	N	disorientated	Died	
N	N	N	disorientated	Alive	
Y	Y	N	alert	Died	
N	N	N	alert	Alive	
Y	Y	N	disorientated	Died	
Y	Y	Y	sedated	Alive	
N	Y	Y	sedated	Alive	
Y	Y	N	sedated	Alive	
N	Y	N	disorientated	Died	
U	Y	Y	sedated	Died	
N	Y	N	unconscious	Died	
Y	Y	Y	sedated	Alive	
Y	N	N	alert	Alive	
Y	Y	N	sedated	Alive	
Y	Y	N	alert	Alive	
N	N	N	alert	Alive	
N	N	Y	alert	Alive	
N	N	N	alert	Alive	
Y	Y	Y	sedated	Died	
Y	Y	N	disorientated	Alive	
N	N	N	alert	Alive	
Y	N	N	disorientated	Died	
N	Y	Y	sedated	Alive	

Y	Y	N	disorientated	Alive
N	Y	N	alert	Alive
N	Y	N	alert	Alive
N	Y	N	alert	Alive
Y	N	N	alert	Alive
Y	N	Y	sedated	Alive
N	Y	Y	sedated	Died
Y	Y	Y	sedated	Alive
Y	N	N	alert	Died
Y	Y	Y	sedated	Died
Y	Y	N	disorientated	Died
N	Y	Y	sedated	Alive
U	Y	Y	sedated	Died
Y	Y	Y	sedated	Alive
N	N	N	unknown	Died
Y	Y	Y	sedated	Alive
N	Y	Y	sedated	Died
N	Y	N	disorientated	Died
			unknown	
			unknown	
			unknown	
			unknown	
			unknown	
			unknown	
			unknown	
			unknown	
			unknown	
			unknown	
			unknown	
			unknown	
			unknown	
			unknown	
			unknown	
			unknown	
			unknown	
			unknown	
			unknown	
			unknown	
			unknown	
			unknown	
			unknown	
			unknown	
			unknown	
			unknown	
			unknown	
			unknown	
			unknown	
			unknown	
			unknown	
			unknown	
			unknown	
			unknown	
			unknown	
			unknown	
			unknown	
			unknown	

			unknown		
			unknown		
			unknown		
			unknown		
			unknown		
			unknown		
			unknown		
			unknown		
			unknown		
			unknown		
			unknown		
			unknown		
			unknown		
			unknown		
			unknown		
			unknown		
			unknown		
			unknown		
			unknown		
			unknown		
			unknown		
			unknown		
			unknown		
			unknown		
			unknown		
			unknown		
			unknown		
			unknown		
			unknown		
			unknown		
			unknown		
			unknown		
			unknown		
			unknown		
			unknown		
			unknown		
			unknown		
			unknown		
			unknown		
			unknown		
			unknown		
			unknown		
			unknown		
			unknown		
			unknown		
			unknown		
			unknown		
			unknown		
			unknown		
			unknown		<=8
Y	N	Y	alert	Died	<=8
Y	Y	N	unconscious	Died	<=8
			unknown		<=8
Y	Y	N	alert	Alive	>32
			unknown		<=8
Y	Y	Y	sedated	Alive	>32

			unknown		<=8
Y	Y	N	unconscious	Died	<=8
Y	N	Y	sedated	Died	>32
Y	Y	N	alert	Died	<=8
			unknown		<=8
N	N	N	disorientated	Alive	<=8
Y	N	Y	unknown	Alive	<=8
			unknown		<=8
Y	N	N	alert	Alive	<=8
N	Y	N	sedated	Died	<=8
Y	Y	Y	sedated	Alive	<=8
Y	N	N	alert	Alive	>32
Y	N	N	alert	Alive	<=8
Y	Y	N	alert	Alive	>32
Y	Y	N	alert	Alive	>32
Y	Y	Y	sedated	Alive	<=8
Y	Y	Y	sedated	Died	>32
N	N	N	alert	Alive	<=8
Y	N	N	disorientated	Alive	16
			unknown		<=8
Y	N	N	alert	Alive	<=8
N	N	N	alert	Alive	<=8
Y	N	Y	sedated	Alive	<=8
U	Y	N	alert	Alive	<=8
Y	N	N	alert	Alive	>32
N	N	Y	sedated	Died	<=8
			unknown		<=8
N	Y	Y	sedated	Alive	<=8
Y	Y	N	unconscious	Died	<=8
			unknown		<=8
Y	N	U	unknown	Died	<=8
Y	Y	N	unknown	Died	<=8
Y	Y	N	alert	Died	<=8
Y	Y	Y	sedated	Died	>32
Y	Y	Y	sedated	Died	<=8
Y	N	N	alert	Alive	>32
Y	N	N	alert	Alive	<=8
Y	Y	N	alert	Alive	<=8
Y	N	U	unknown	Died	16
Y	Y	Y	sedated	Died	<=8
Y	Y	Y	sedated	Alive	>32
Y	Y	N	alert	Died	>32
N	Y	N	alert	Alive	16
Y	N	N	alert	Alive	16
Y	Y	Y	sedated	Died	>32
			unknown		<=8
Y	N	N	alert	Alive	>32
U	N	N	disorientated	Died	32
Y	Y	N	sedated	Alive	<=8
Y	Y	N	alert	Died	<=8
N	N	N	alert	Alive	<=8
Y	N	N	alert	Died	>32
N	Y	N	alert	Alive	<=8
U	N	N	alert	Alive	32
Y	N	N	alert	Alive	>32
N	Y	N	alert	Alive	32

N	Y	Y	sedated	Died	16
Y	Y	Y	disorientated	Alive	<=8
Y	Y	N	disorientated	Died	<=8
			unknown		<=8
Y	Y	Y	sedated	Died	>32
			unknown		<=8
Y	Y	Y	sedated	Alive	>32
U	Y	Y	sedated	Alive	<=8
			unknown		<=8
N	Y	N	alert	Alive	<=8
			unknown		16
N	Y	Y	sedated	Alive	<=8
			unknown		<=8
N	Y	N	alert	Alive	<=8
U	U	U	unknown		<=8
Y	Y	N	alert	Alive	>32
Y	Y	N	alert	Died	<=8
Y	N	N	disorientated	Alive	<=8
Y	N	N	alert	Died	16
N	Y	U	unknown	Alive	<=8
Y	N	N	alert	Alive	<=8
			unknown		<=8
			unknown		<=8
Y	Y	N	unknown	Alive	>32
N	N	U	unknown	Died	<=8
U	U	U	unknown	Alive	>32
U	N	N	alert	Alive	>32
Y	Y	Y	sedated	Alive	<=8
			unknown		<=8
U	U	U	unknown	Died	<=8
Y	Y	Y	sedated	Alive	>32
Y	Y	Y	sedated	Died	<=8
			unknown		<=8
U	N	N	unknown	Died	>32
			unknown		<=8
			unknown		<=8
Y	Y	Y	sedated	Died	<=8
U	U	U	unknown	Died	<=8
N	N	N	alert	Alive	>32
Y	N	N	alert	Alive	<=8
N	Y	Y	sedated	Died	<=8
Y	N	N	alert	Died	<=8
Y	Y	N	alert	Alive	<=8
Y	N	N	alert	Died	>32
U	Y	Y	sedated	Died	>32
N	Y	N	alert	Alive	>32
N	Y	N	alert	Alive	>32
N	N	N	alert	Alive	>32
Y	Y	N	alert	Alive	<=8
U	U	U	unknown		
			unknown		<=8
			unknown		>32
			unknown		<=8
			unknown		<=8
Y	Y	N	alert	Died	>32
			unknown		16

Y	N	N	alert	Alive	>32
			unknown		16
			unknown		<=8
			unknown		>32
U	N	N	alert	Alive	<=8
			unknown		<=8
Y	Y	N	alert	Alive	<=8
N	N	Y	sedated	Died	<=8
N	N	Y	sedated	Died	<=8
Y	N	N	alert	Alive	<=8
N	N	N	disorientated	Died	<=8
Y	N	N	alert	Died	>32
Y	N	N	alert	Alive	<=8
U	N	N	alert	Alive	<=8
Y	Y	N	sedated	Died	<=8
			unknown		<=8
Y	N	N	alert	Died	<=8
N	Y	N	disorientated	Alive	16
N	Y	Y	sedated	Alive	<=8
U	Y	N	sedated	Alive	<=8
N	N	N	alert	Alive	32
U	Y	Y	sedated	Died	>32
			unknown		<=8
Y	Y	Y	sedated	Died	<=8
			unknown		<=8
Y	N	Y	alert	Died	<=8
			unknown		<=8
U	Y	N	unknown	Died	<=8
U	Y	N	disorientated	Died	<=8
Y	Y	N	unknown	Alive	<=8
Y	N	N	alert	Alive	<=8
Y	N	N	unknown	Died	<=8
Y	Y	Y	sedated	Died	<=8
Y	N	N	unknown	Alive	<=8
Y	N	N	alert	Alive	>32
Y	Y	Y	sedated	Alive	<=8
U	U	U	unknown	Died	<=8
Y	N	N	alert	Died	<=8
			unknown		<=8
U	Y	N	alert	Died	<=8
Y	Y	N	alert	Alive	<=8
Y	N	Y	unknown	Died	<=8
N	Y	Y	sedated	Alive	<=8
			unknown		<=8
N	Y	N	alert	Alive	>32
Y	Y	N	alert	Alive	<=8
Y	N	N	alert	Alive	<=8
Y	N	N	alert	Died	<=8
N	N	U	alert	Alive	<=8
Y	Y	Y	sedated	Died	<=8
			unknown		<=8
Y	Y	Y	sedated	Alive	<=8
Y	Y	Y	sedated	Alive	<=8
N	N	N	alert	Alive	<=8
N	Y	N	disorientated	Alive	<=8
Y	Y	Y	sedated	Died	>32

Y	Y	N	alert	Alive	<=8
U	Y	N	disorientated	Died	<=8
Y	Y	N	alert	Died	<=8
N	Y	N	alert	Died	<=8
N	Y	N	unknown	Alive	<=8
N	Y	Y	sedated	Alive	<=8
Y	Y	N	disorientated	Died	<=8
Y	N	N	alert	Alive	<=8
Y	Y	Y	sedated	Alive	>32
Y	Y	Y	sedated	Died	>32
N	Y	Y	sedated	Died	<=8
Y	N	N	alert	Alive	<=8
Y	Y	Y	disorientated	Died	<=8
N	N	N	alert	Alive	>32
			unknown		<=8
N	N	N	alert	Alive	>32
Y	Y	Y	sedated	Alive	>32
Y	Y	Y	sedated	Died	>32
Y	Y	N	sedated	Died	<=8
Y	Y	N	alert	Alive	>32
N	N	N	alert	Alive	>32
N	N	N	alert	Died	>32
Y	Y	N	unconscious	Died	16
U	U	U	unknown	Died	<=8
Y	Y	N	alert	Died	<=8
			unknown		<=8
N	N	N	alert	Alive	<=8
Y	Y	N	alert	Alive	<=8
N	N	N	alert	Alive	<=8
N	Y	Y	alert	Died	<=8
N	Y	Y	sedated	Alive	<=8
Y	N	N	alert	Alive	>32
Y	N	N	alert	Alive	<=8
N	N	N	alert	Alive	<=8
U	Y	N	unknown	Alive	<=8
N	Y	N	sedated	Alive	<=8
U	N	U	unknown	Died	<=8
N	Y	Y	sedated	Alive	<=8
			unknown		<=8
			unknown		<=8
N	Y	Y	sedated	Died	16
Y	Y	N	alert	Alive	<=8
N	Y	N	alert	Alive	<=8
Y	Y	Y	sedated	Died	<=8
N	N	N	disorientated	Died	>32
Y	N	U	unknown		<=8
Y	Y	Y	sedated	Died	<=8
			unknown		<=8
N	N	Y	unknown	Died	<=8
			unknown		<=8
N	Y	Y	sedated	Died	<=8
			unknown		16
N	N	N	alert	Alive	<=8
N	N	N	disorientated	Alive	<=8
Y	Y	N	alert	Alive	<=8
Y	N	N	alert	Alive	>32

Y	N	N	unknown	Died	<=8
Y	N	N	alert	Alive	>32
			unknown		<=8
Y	Y	N	unconscious	Died	16
Y	Y	N	alert	Died	<=8
Y	Y	N	sedated	Died	<=8
Y	N	N	alert	Alive	>32
N	N	N	alert	Alive	>32
N	N	N	alert	Alive	
Y	Y	N	sedated	Died	<=8
Y	N	Y	sedated	Alive	<=8
N	N	Y	sedated	Died	<=8
			unknown		<=8
U	N	U	unknown		<=8
N	Y	Y	sedated	Died	<=8
N	Y	Y	sedated	Alive	<=8
U	N	N	disorientated	Died	<=8
Y	Y	N	alert	Alive	>32
			unknown		>32
Y	Y	N	alert	Alive	16
			unknown		>32
U	Y	N	alert	Alive	<=8
Y	Y	N	alert	Alive	<=8
U	Y	Y	sedated	Died	>32
Y	N	N	unknown	Died	<=8
U	U	U	unknown	Alive	<=8
Y	Y	Y	sedated	Died	<=8
Y	N	N	alert	Died	<=8
Y	Y	Y	sedated	Died	<=8
Y	Y	N	unconscious	Died	<=8
Y	N	N	disorientated	Alive	<=8
Y	Y	N	alert	Alive	<=8
Y	Y	Y	sedated	Died	<=8
Y	N	N	alert	Alive	<=8
N	N	Y	unknown	Alive	<=8
Y	N	N	unknown	Alive	<=8
Y	Y	N	unknown	Alive	<=8
			unknown		<=8
Y	Y	Y	sedated	Died	<=8
			unknown		16
Y	N	Y	sedated	Alive	<=8
			unknown		<=8
Y	Y	Y	sedated	Died	16
N	Y	Y	sedated	Alive	<=8
Y	Y	N	alert	Died	>32
Y	N	N	unknown	Alive	<=8
Y	Y	N	alert	Alive	>32
Y	Y	N	alert	Alive	<=8
N	N	N	alert	Alive	>32
Y	N	N	alert	Alive	<=8
Y	Y	Y	sedated	Alive	>32
U	Y	Y	sedated	Died	16
N	N	Y	sedated	Alive	<=8
Y	N	N	unknown	Alive	16
			unknown		<=8
			unknown		<=8

U	N	Y	unknown	Died	<=8
N	N	N	alert	Alive	<=8
			unknown		<=8
N	N	Y	unknown	Died	<=8
Y	N	N	unknown	Died	>32
N	Y	U	unknown	Died	<=8
Y	N	N	alert	Alive	<=8
Y	N	N	alert	Died	<=8
Y	Y	Y	sedated	Alive	<=8
Y	N	N	alert	Died	>32
N	N	N	alert	Alive	>32
Y	N	N	alert	Alive	>32
N	N	N	alert	Alive	>32
Y	Y	Y	sedated	Alive	>32
Y	N	N	alert	Alive	<=8
Y	Y	Y	sedated	Alive	<=8
			unknown		<=8
N	N	N	disorientated	Died	>32
			unknown		>32
			unknown		<=8
N	N	Y	unconscious	Died	>32
Y	Y	Y	sedated	Died	<=8
N	N	N	alert	Alive	>32
N	Y	Y	sedated	Alive	>32
N	Y	N	alert	Died	>32
Y	N	N	alert	Alive	>32
N	Y	N	unknown	Died	>32
N	Y	N	alert	Alive	>32
N	Y	Y	sedated	Died	<=8
			unknown		<=8
			unknown		<=8
			unknown		<=8
Y	N	U	unknown	Died	16
U	N	Y	sedated	Died	<=8
Y	N	N	alert	Died	<=8
			unknown		<=8
			unknown		<=8
Y	Y	N	alert	Alive	<=8
			unknown		<=8
			unknown		<=8
Y	Y	N	alert	Died	<=8
N	N	N	sedated	Alive	<=8
U	N	Y	unknown	Died	<=8
Y	N	N	alert	Alive	<=8
N	N	N	alert	Alive	<=8
			unknown		<=8
			unknown		<=8
			unknown		<=8
			unknown		<=8
			unknown		<=8
Y	N	N	alert	Alive	<=8
N	Y	Y	sedated	Alive	<=8
N	Y	Y	sedated	Alive	<=8
U	N	U	unknown		<=8
			unknown		<=8
Y	Y	N	alert	Alive	<=8

			unknown		<=8
Y	Y	N	unconscious	Died	<=8
			unknown		>32
U	Y	N	alert	Died	<=8
Y	Y	Y	sedated	Alive	<=8
			unknown		<=8
			unknown		16
N	N	N	unknown	Alive	>32
Y	N	N	alert	Alive	<=8
N	Y	Y	sedated	Died	>32
Y	N	Y	sedated	Died	<=8
Y	N	N	unknown	Alive	<=8
Y	N	N	alert	Alive	<=8
Y	Y	N	disorientated	Alive	<=8
Y	N	Y	sedated	Died	<=8
			unknown		<=8
Y	N	N	unknown	Died	<=8
			unknown		<=8
Y	N	Y	sedated	Alive	<=8
Y	Y	Y	sedated	Died	<=8
Y	Y	Y	sedated	Alive	<=8
N	Y	N	alert	Alive	<=8
N	Y	N	alert	Died	<=8
			unknown		<=8
N	Y	N	unconscious	Died	16
N	Y	N	alert	Alive	>32
N	Y	N	alert	Alive	>32
N	Y	N	alert	Alive	<=8
			unknown		<=8
			unknown		<=8
			unknown		<=8
			unknown		<=8
			unknown		<=8
N	N	N	alert	Alive	<=8
Y	Y	N	alert	Alive	<=8
			unknown		<=8
Y	N	N	alert	Alive	<=8
N	Y	Y	alert	Alive	16
			unknown		<=8
N	Y	N	unknown	Alive	<=8
U	N	N	alert	Alive	<=8
Y	N	N	alert	Alive	>32
N	Y	N	alert	Alive	<=8
Y	Y	N	sedated	Alive	<=8
U	Y	N	disorientated	Died	<=8
Y	Y	N	alert	Alive	<=8
			unknown		<=8
			unknown		<=8
			unknown		<=8
Y	N	N	alert	Alive	<=8
			unknown		<=8
			unknown		<=8
			unknown		<=8
N	Y	N	alert	Alive	<=8
U	Y	N	alert	Alive	<=8
			unknown		<=8

Y	Y	Y	unconscious	Died	32
Y	N	N	alert	Alive	>32
N	N	N	alert	Alive	>32
U	N	N	unknown	Alive	<=8
Y	N	U	unknown	Died	>32
			unknown		16
Y	Y	N	alert	Alive	<=8
Y	Y	Y	sedated	Alive	>32
			unknown		
N	N	N	alert	Alive	
Y	Y	N	alert	Died	<=8
N	Y	Y	sedated	Alive	
N	N	N	disorientated	Alive	
U	N	N	alert	Alive	>32
U	U	U	unknown	Alive	
			unknown		<=8
N	Y	Y	sedated	Alive	<=8
			unknown		<=8
Y	Y	N	unknown	Alive	<=8
			unknown		16
Y	Y	Y	sedated	Alive	<=8
Y	Y	N	unknown	Alive	<=8
Y	Y	N	alert	Alive	>32
U	U	U	unknown	Alive	>32
			unknown		>32
			unknown		<=8
Y	N	Y	sedated	Died	<=8
Y	Y	N	alert	Alive	<=8
			unknown		
Y	N	Y	unconscious	Died	
			unknown		
			unknown		
Y	Y	Y	sedated	Alive	
Y	Y	Y	sedated	Alive	
			unknown		
U	N	N	alert	Alive	
U	N	N	alert	Alive	
U	N	N	alert	Alive	
			unknown		
N	Y	Y	sedated	Died	
			unknown		
			unknown		
N	N	N	alert	Alive	
Y	Y	N	disorientated	Alive	
			unknown		
N	Y	Y	sedated	Died	
			unknown		
Y	N	N	disorientated	Died	
N	N	N	disorientated	Alive	
N	Y	N	sedated	Alive	
N	Y	Y	sedated	Alive	
Y	N	Y	sedated	Died	
U	U	U	unknown	Alive	
Y	Y	N	alert	Died	
			unknown		
N	Y	Y	sedated	Died	

N	N	N	alert	Alive
N	N	N	unknown	Died
U	N	N	alert	Died
U	N	N	alert	Died
			unknown	
U	N	N	alert	Died
Y	Y	Y	sedated	Alive
Y	Y	N	unknown	
Y	Y	N	alert	Alive
			unknown	
Y	N	Y	sedated	Died
			unknown	
Y	Y	N	unknown	Died
			unknown	
N	Y	N	alert	Alive
N	N	N	alert	Alive
			unknown	
			unknown	
N	N	N	disorientated	Alive
Y	Y	N	alert	Died
U	N	N	alert	Alive
Y	Y	N	alert	Died
Y	N	N	unknown	Alive
Y	Y	Y	sedated	Died
U	N	N	alert	Alive
N	N	N	alert	Alive
N	Y	Y	sedated	Died
			unknown	
U	N	N	alert	Alive
			unknown	
N	Y	Y	sedated	Died
			unknown	
Y	Y	Y	sedated	Alive
N	Y	N	alert	Alive
			unknown	
N	N	N	alert	Alive
Y	N	N	disorientated	Died
N	N	N	sedated	Alive
N	N	N	alert	Alive
			unknown	
			unknown	
U	U	U	unknown	
			unknown	
N	Y	N	alert	Alive
Y	N	N	alert	Alive
			unknown	
N	Y	Y	unconscious	Died
N	N	Y	sedated	Died
Y	N	N	alert	Died
N	N	N	alert	Alive
Y	N	N	alert	Died
U	N	N	alert	Alive
Y	N	N	unknown	Alive
			unknown	
N	U	U	unknown	Died
Y	Y	N	alert	Died

Y	N	N	unknown	Died	
U	N	U	unknown	Alive	<=8
Y	Y	Y	sedated	Alive	
Y	N	N	unknown	Alive	
			unknown		
N	N	N	unknown	Alive	
Y	Y	Y	alert	Alive	
Y	Y	N	alert	Alive	
			unknown		
			unknown		
Y	Y	Y	sedated	Died	
U	Y	Y	sedated	Died	
Y	N	N	alert		
N	N	N	alert	Alive	
U	Y	Y	unknown	Alive	
			unknown		
			unknown		
U	N	N	alert	Died	
Y	Y	N	alert	Died	
Y	N	N	sedated	Died	
N	Y	Y	sedated	Alive	
U	U	U	unknown		
N	Y	N	alert	Alive	
Y	Y	Y	alert	Alive	
N	N	N	alert	Alive	
Y	N	N	unconscious	Alive	
N	N	N	alert	Alive	
N	Y	N	sedated	Alive	
Y	N	N	alert	Died	
Y	N	N	alert	Alive	
Y	Y	Y	sedated	Alive	
			unknown		
			unknown		
N	Y	N	alert	Alive	
Y	N	Y	unknown	Died	
Y	N	N	alert	Alive	
N	Y	Y	sedated	Died	
			unknown		
Y	N	N	alert	Died	
			unknown		
			unknown		
N	Y	N	alert	Alive	
			unknown		
Y	N	N	alert	Alive	
Y	N	N	unknown	Alive	
U	Y	N	alert	Died	
U	U	N	unknown	Died	
Y	Y	N	alert	Alive	
N	Y	N	alert	Alive	
			unknown		
Y	N	N	alert	Alive	<=8
			unknown		
			unknown		
U	N	N	alert	Died	
Y	N	N	alert	Alive	
			unknown		

N	N	N	alert	Alive	
			unknown		
			unknown		
Y	N	N	alert	Alive	
Y	N	N	unknown	Alive	
			unknown		
			unknown		
Y	Y	N	alert	Alive	
			unknown		
N	Y	N	alert	Alive	
N	Y	N	alert	Died	
			unknown		
			unknown		
			unknown		
N	Y	N	alert	Alive	
Y	Y	N	alert	Alive	
Y	Y	Y	sedated	Alive	
N	N	N	alert	Died	
			unknown		
N	N	N	alert	Alive	
			unknown		
Y	Y	Y	sedated	Alive	
Y	Y	Y	unconscious	Died	
			unknown		
			unknown		
			unknown		
			unknown		
			unknown		
			unknown		
N	N	N	alert	Alive	
Y	N	N	alert	Alive	
N	U	U	unknown	Alive	
N	N	N	alert	Alive	
			unknown		
U	Y	N	unknown	Died	
Y	N	N	alert	Alive	
U	U	U	unknown		
N	N	N	alert	Alive	
U	N	N	alert	Alive	
			unknown		
Y	Y	N	disorientated	Died	
			unknown		
			unknown		
Y	N	N	sedated	Died	
Y	Y	Y	sedated	Alive	
Y	U	Y	unknown	Died	
			unknown		
N	Y	Y	sedated	Died	
			unknown		
			unknown		
U	U	U	unknown		
			unknown		
U	N	N	alert	Alive	<=8
N	Y	N	alert	Died	<=8
N	N	N	disorientated	Died	<=8
N	N	N	alert	Alive	<=8

			unknown		<=8
N	N	Y	sedated	Died	<=8
Y	N	Y	sedated	Alive	<=8
N	N	N	alert	Alive	<=8
			unknown		16
			unknown		<=8
			unknown		<=8
			unknown		<=8
N	Y	N	alert	Alive	16
Y	Y	Y	sedated	Alive	16
N	N	N	alert	Alive	<=8
Y	N	U	unknown	Alive	>32
Y	N	Y	unknown	Died	>32
N	N	Y	sedated	Died	<=8
Y	U	N	unknown	Alive	<=8
Y	Y	N	alert	Alive	<=8
Y	Y	Y	sedated	Alive	<=8
			unknown		>32
Y	Y	Y	sedated	Alive	
Y	N	N	alert	Alive	
U	N	N	alert	Alive	
Y	N	N	disorientated	Alive	
N	U	N	alert	Alive	
Y	Y	Y	sedated	Alive	
Y	N	N	disorientated	Alive	
Y	N	N	unknown	Died	
U	N	U	unknown	Died	
N	Y	N	unknown	Alive	
Y	N	N	alert	Alive	
Y	N	N	alert	Died	
Y	N	N	unknown	Alive	
Y	Y	N	alert	Alive	<=8
N	Y	Y	sedated	Alive	
N	Y	Y	disorientated	Died	
U	N	N	unknown	Died	
			unknown		<=8
			unknown		<=8
U	N	N	unknown	Alive	<=8
			unknown		<=8
Y	Y	N	unknown	Alive	<=8
N	Y	N	alert	Alive	<=8
U	Y	Y	sedated	Alive	<=8
Y	N	N	alert	Alive	<=8
N	Y	N	unknown	Alive	<=8
Y	Y	N	alert	Alive	<=8
Y	Y	Y	sedated	Alive	<=8
N	N	N	alert	Alive	<=8
Y	N	N	alert	Alive	<=8
Y	Y	Y	sedated	Died	<=8
			unknown		<=8
N	Y	N	alert	Alive	<=8
			unknown		<=8
Y	Y	N	alert	Died	<=8
			unknown		<=8
			unknown		<=8
			unknown		<=8

N		Y	N	alert	Alive	<=8
				unknown		<=8
N		N	Y	sedated	Died	<=8
Y		Y	Y	sedated	Alive	>32
U		Y	Y	sedated	Alive	<=8
Y		Y	Y	sedated	Died	<=8
N		N	N	disorientated	Died	<=8
U		Y	N	unknown	Alive	<=8
N		N	Y	unknown	Alive	<=8
				unknown		<=8
				unknown		<=8
				unknown		<=8
				unknown		<=8
				unknown		<=8
N		Y	N	unknown	Alive	<=8
				unknown		<=8
				unknown		<=8
				unknown		<=8
				unknown		<=8
				unknown		<=8
Y		Y	Y	disorientated	Alive	<=8
Y		Y	Y	sedated	Died	<=8
Y		Y	N	disorientated	Died	<=8
Y		Y	N	disorientated	Alive	<=8
				unknown		<=8
N		N	N	disorientated	Died	<=8
N		N	Y	unconscious	Died	16
Y		N	N	unknown	Alive	<=8
				unknown		<=8
N		Y	N	alert	Alive	>32
N		Y	Y	sedated	Died	<=8
Y		Y	Y	alert	Died	<=8
N		Y	N	alert	Alive	<=8
N		Y	N	alert	Alive	<=8
Y		N	Y	sedated	Died	<=8
Y		Y	Y	sedated	Died	<=8
				unknown		<=8
N		N	N	disorientated	Died	<=8
N		Y	N	alert	Died	<=8
N		Y	N	alert	Alive	<=8
N		Y	N	alert	Died	<=8
Y		Y	Y	sedated	Alive	>32
Y		N	N	alert	Died	>32
Y		Y	N	alert	Alive	16
Y		Y	N	alert	Alive	>32
				unknown		16
				unknown		16
Y		Y	N	disorientated	Alive	>32
N		N	N	alert	Alive	>32
N		Y	Y	sedated	Alive	<=8
N		Y	N	unknown	Alive	<=8
				unknown		<=8
Y		Y	Y	sedated	Died	<=8
U		U	U	unknown		<=8
				unknown		<=8
N		N	N	alert	Alive	<=8

Y	Y	N	alert	Died	<=8
N	N	Y	sedated	Died	<=8
			unknown		<=8
Y	N	N	alert	Alive	<=8
			unknown		16
Y	Y	Y	sedated	Died	>32
U	N	N	unknown	Died	<=8
U	U	U	unknown	Died	>32
			unknown		<=8
Y	N	U	unknown	Died	<=8
N	Y	N	disorientated	Died	>32
Y	N	N	unknown	Alive	<=8
			unknown		<=8
			unknown		<=8
Y	Y	Y	sedated	Alive	<=8
Y	Y	Y	sedated	Alive	<=8
Y	N	N	alert	Alive	>32
Y	Y	Y	sedated	Died	<=8
Y	N	N	alert	Alive	<=8
			unknown		<=8
U	N	N	alert	Alive	<=8
Y	N	N	alert	Alive	<=8
Y	Y	N	alert	Alive	<=8
Y	N	N	alert	Alive	<=8
N	Y	Y	disorientated	Died	>32
			unknown		<=8
Y	N	Y	unknown	Alive	<=8
Y	Y	N	alert	Alive	<=8
U	Y	N	alert	Alive	<=8
Y	U	U	unknown	Alive	<=8
			unknown		<=8
Y	N	N	alert	Alive	>32
Y	Y	N	alert	Alive	16
Y	Y	Y	sedated	Alive	>32
			unknown		<=8
N	N	N	alert	Alive	>32
			unknown		>32
Y	Y	N	alert	Alive	>32
N	N	N	alert	Alive	<=8
			unknown		>32
			unknown		>32
			unknown		<=8
			unknown		16
N	Y	Y	sedated	Died	32
Y	N	Y	unknown	Died	<=8
			unknown		16
N	Y	Y	sedated	Alive	<=8
Y	N	N	alert	Died	<=8
Y	Y	N	alert	Alive	16
			unknown		>32
Y	N	N	disorientated	Died	<=8
Y	Y	Y	sedated	Died	<=8
			unknown		16
N	Y	U	alert	Alive	>32
			unknown		
Y	N	U	unknown	Alive	32

			unknown		<=8
			unknown		>32
Y	N	N	alert	Alive	>32
			unknown		32
			unknown		<=8
Y	Y	Y	sedated	Died	>32
Y	N	N	alert	Alive	<=8
			unknown		<=8
N	Y	Y	sedated	Alive	>32
N	Y	N	alert	Alive	<=8
			unknown		<=8
Y	Y	Y	sedated	Died	<=8
N	N	N	alert	Alive	<=8
Y	Y	N	alert	Alive	>32
Y	Y	N	alert	Died	>32
Y	N	N	sedated	Died	<=8
N	Y	N	alert	Alive	>32
N	Y	N	alert	Alive	<=8
			unknown		<=8
N	Y	N	alert	Alive	<=8
			unknown		16
Y	Y	Y	disorientated	Died	<=8
			unknown		<=8
			unknown		<=8
Y	N	N	alert	Alive	<=8
N	N	N	alert	Alive	<=8
			unknown		<=8
			unknown		>32
			unknown		<=8
Y	N	N	unknown	Died	<=8
			unknown		>32
Y	N	N	disorientated	Died	>32
			unknown		<=8
			unknown		<=8
			unknown		16
Y	Y	Y	sedated	Died	<=8
			unknown		>32
			unknown		>32
U	Y	U	unknown	Died	<=8
N	Y	N	alert	Alive	<=8
N	N	Y	sedated	Alive	16
N	N	N	sedated	Died	16
N	U	N	sedated	Died	<=8
N	Y	Y	unconscious	Alive	<=8
N	Y	Y	unconscious	Died	<=8
N	Y	Y	sedated	Alive	16
Y	N	N	unknown	Alive	>32
U	Y	Y	sedated	Alive	<=8
Y	N	N	alert		>32
Y	Y	Y	sedated	Alive	<=8
			unknown		<=8
Y	Y	N	alert	Alive	16
Y	N	N	unknown	Alive	<=8
			unknown		<=8
			unknown		16
			unknown		<=8

N	N	N	alert	Alive	>32
Y	Y	Y	sedated	Died	>32
			unknown		<=8
Y	Y	Y	sedated	Alive	<=8
			unknown		
			unknown		
U	Y	Y	sedated	Died	
			unknown		
N	N	Y	sedated	Died	
			unknown		
Y	Y	N	alert	Alive	
			unknown		
			unknown		
			unknown		
			unknown		
N	N	N	alert	Alive	
Y	N	N	alert	Died	
Y	Y	Y	sedated	Alive	
			unknown		
			unknown		
Y	Y	Y	sedated	Alive	
			unknown		
			unknown		
Y	N	N	alert	Alive	
Y	U	U	unknown	Alive	
Y	N	N	sedated	Alive	
Y	N	N	alert	Alive	
U	U	U	unknown	Died	
			unknown		
Y	N	N	alert	Alive	
U	U	U	unknown	Alive	
			unknown		
N	U	Y	disorientated	Alive	
U	Y	Y	sedated	Alive	
			unknown		
Y	Y	N	unknown	Alive	
N	Y	N	alert	Died	
U	Y	N	disorientated	Died	
U	N	Y	sedated	Died	
			unknown		
Y	Y	Y	sedated	Died	
N	N	Y	alert	Died	
			unknown		
			unknown		
			unknown		
Y	Y	Y	sedated	Died	
U	N	N	alert	Alive	
Y	N	N	alert	Died	
U	U	U	unknown	Died	
			unknown		
			unknown		
Y	Y	Y	sedated	Died	
Y	Y	U	alert	Died	
N	Y	N	alert	Alive	
			unknown		
N	Y	N	sedated	Died	

			unknown	
N	N	N	alert	Alive
N	Y	N	alert	Alive
N	Y	Y	sedated	Alive
			unknown	
N	N	Y	alert	Died
N	Y	N	unknown	Alive
N	N	N	alert	Alive
N	Y	N	disorientated	Alive
			unknown	
			unknown	
Y	Y	N	alert	Died
			unknown	
U	U	U	unknown	
			unknown	
			unknown	
Y	Y	N	sedated	Alive
			unknown	
			unknown	
Y	Y	Y	sedated	Alive
Y	Y	N	unknown	Alive
			unknown	
			unknown	
Y	Y	Y	sedated	Alive
			unknown	
N	Y	Y	sedated	Died
N	Y	N	alert	Died
Y	Y	Y	alert	Alive
			unknown	
			unknown	
Y	N	N	alert	Died
			unknown	
			unknown	
			unknown	
			unknown	
			unknown	
Y	N	N	disorientated	Alive
			unknown	
			unknown	
U	N	Y	sedated	Died
			unknown	
U	U	U	unknown	
N	Y	Y	unconscious	Died
			unknown	
N	Y	N	unconscious	Alive
N	Y	N	alert	Alive
N	Y	N	unknown	Alive
			unknown	
N	Y	Y	sedated	Alive
			unknown	
			unknown	
			unknown	
			unknown	
U	N	N	alert	Alive
U	U	N	unknown	Alive
N	Y	Y	sedated	Died

Y	Y	Y	sedated	Died
Y	Y	Y	sedated	Died
N	Y	Y	sedated	Died
			unknown	
			unknown	
U	N	N	unknown	Alive
N	N	N	alert	Alive
N	N	N	alert	Alive
Y	N	Y	unconscious	Died
			unknown	
			unknown	
N	N	N	alert	Alive
			unknown	
Y	Y	Y	sedated	Died
U	Y	Y	sedated	Died
			unknown	
			unknown	
			unknown	
Y	Y	N	alert	Died
Y	N	N	unknown	Died
			unknown	
			unknown	
			unknown	
			unknown	
			unknown	
Y	N	N	disorientated	Died
Y	N	N	alert	Alive
N	Y	Y	sedated	Died
Y	N	N	alert	Died
			unknown	
N	N	N	alert	Alive
			unknown	
			unknown	
N	Y	N	alert	Alive
Y	Y	N	alert	Died
			unknown	
			unknown	
Y	N	N	alert	Alive
			unknown	
N	N	N	alert	Died
Y	N	Y	sedated	Died
			unknown	
Y	Y	N	unknown	Died
			unknown	
N	Y	Y	sedated	Alive
			unknown	
Y	Y	Y	sedated	Died
Y	Y	N	alert	Alive
N	Y	N	alert	Alive
			unknown	
N	Y	U	alert	Alive
			unknown	
			unknown	
			unknown	
			unknown	
Y	N	N	disorientated	Died

			unknown	
U	N	N	alert	Alive
			unknown	
			unknown	
			unknown	
			unknown	
			unknown	
			unknown	
			unknown	
			unknown	
			unknown	
			unknown	
N	N	N	alert	Alive
Y	Y	N	alert	Alive
N	Y	N	alert	Died
			unknown	
			unknown	
Y	Y	N	unknown	Alive
			unknown	
			unknown	
N	Y	N	alert	Alive
			unknown	
			unknown	
			unknown	
			unknown	
			unknown	
Y	Y	Y	unconscious	Died
N	Y	N	unknown	Alive
Y	N	N	alert	Alive
			unknown	
			unknown	
			unknown	
			unknown	
U	N	N	alert	Alive
N	N	N	alert	Alive
			unknown	
			unknown	
N	Y	N	alert	Alive
N	Y	N	alert	Alive
N	Y	Y	sedated	Died
			unknown	
Y	Y	N	alert	Alive
			unknown	
			unknown	
N	Y	Y	sedated	Alive
			unknown	
Y	Y	N	disorientated	Alive
N	Y	Y	disorientated	Died
			unknown	
N	Y	Y	sedated	Died
U	Y	N	disorientated	Alive
			unknown	
N	N	N	alert	Alive
			unknown	
			unknown	
N	N	N	alert	Alive

Y	Y	N	alert	Alive	
U	U	N	alert	Alive	
			unknown		
N	Y	N	alert	Alive	
U	U	U	unknown		
			unknown		
			unknown		
			unknown		
			unknown		
			unknown		
Y	Y	N	alert	Alive	
N	Y	Y	sedated	Died	
N	Y	N	alert	Alive	
U	Y	Y	unconscious	Died	
Y	N	N	alert	Alive	
Y	Y	N	sedated	Alive	
Y	N	N	alert	Alive	
N	N	N	alert	Alive	
Y	N	N	unknown	Died	
			unknown		
			unknown		
			unknown		<=8
U	Y	N	unconscious	Died	
N	N	N	alert	Alive	
			unknown		<=8
			unknown		<=8
			unknown		<=8
			unknown		<=8
			unknown		<=8
			unknown		>32
			unknown		<=8
			unknown		<=8
			unknown		<=8
			unknown		<=8
			unknown		<=8
			unknown		16
			unknown		<=8
			unknown		<=8
			unknown		<=8
			unknown		<=8
			unknown		<=8
			unknown		<=8
			unknown		<=8
			unknown		<=8
			unknown		32
			unknown		<=8
			unknown		<=8
			unknown		<=8
			unknown		16
			unknown		<=8
			unknown		<=8
			unknown		<=8
			unknown		<=8
			unknown		<=8
			unknown		16
			unknown		<=8

unknown	<=8
unknown	>32
unknown	<=8
unknown	>32
unknown	<=8
unknown	<=8
unknown	<=8
unknown	>32
unknown	<=8
unknown	<=8
unknown	32
unknown	<=8
unknown	<=8
unknown	<=8
unknown	32
unknown	<=8
unknown	<=8
unknown	<=8
unknown	<=8
unknown	>32
unknown	>32
unknown	<=8
unknown	<=8
unknown	>32
unknown	<=8
unknown	<=8
unknown	<=8
unknown	>32
unknown	>32
unknown	<=8
unknown	<=8
unknown	<=8
unknown	<=8
unknown	<=8
unknown	<=8
unknown	<=8
unknown	<=8
unknown	<=8
unknown	<=8
unknown	<=8
unknown	16
unknown	<=8
unknown	<=8
unknown	<=8
unknown	<=8
unknown	<=8
unknown	16
unknown	>32
unknown	16
unknown	<=8
unknown	<=8
unknown	>32
unknown	>32
unknown	<=8
unknown	<=8
unknown	<=8

unknown	<=8
unknown	<=8
unknown	<=8
unknown	>32
unknown	<=8
unknown	<=8
unknown	<=8
unknown	<=8
unknown	<=8
unknown	<=8
unknown	<=8
unknown	<=8
unknown	<=8
unknown	<=8
unknown	<=8
unknown	<=8
unknown	<=8
unknown	16
unknown	<=8
unknown	<=8
unknown	<=8
unknown	<=8
unknown	<=8
unknown	>32
unknown	<=8
unknown	<=8
unknown	>32
unknown	<=8
unknown	<=8
unknown	<=8
unknown	<=8
unknown	<=8
unknown	<=8
unknown	<=8
unknown	<=8
unknown	<=8
unknown	<=8
unknown	>32
unknown	>32
unknown	<=8
unknown	<=8
unknown	<=8
unknown	<=8
unknown	>32
unknown	>32
unknown	<=8
unknown	<=8
unknown	<=8
unknown	>32
unknown	>32
unknown	16
unknown	>32
unknown	<=8
unknown	>32
unknown	16
unknown	32

unknown	<=8
unknown	<=8
unknown	<=8
unknown	<=8
unknown	<=8
unknown	<=8
unknown	<=8
unknown	<=8
unknown	<=8
unknown	<=8
unknown	<=8
unknown	<=8
unknown	<=8
unknown	>32
unknown	<=8
unknown	<=8
unknown	<=8
unknown	<=8
unknown	>32
unknown	>32
unknown	<=8
unknown	<=8
unknown	>32
unknown	<=8
unknown	16
unknown	<=8
unknown	<=8
unknown	<=8
unknown	<=8
unknown	<=8
unknown	<=8
unknown	>32
unknown	<=8
unknown	<=8
unknown	<=8
unknown	<=8
unknown	<=8
unknown	>32
unknown	<=8
unknown	<=8
unknown	<=8
unknown	<=8
unknown	<=8
unknown	<=8
unknown	<=8
unknown	>32
unknown	<=8
unknown	>32
unknown	>32
unknown	<=8
unknown	<=8
unknown	<=8
unknown	<=8
unknown	>32
unknown	>32
unknown	>32

unknown	>32
unknown	>32
unknown	<=8
unknown	<=8
unknown	<=8
unknown	<=8
unknown	<=8
unknown	<=8
unknown	<=8
unknown	<=8
unknown	>32
unknown	<=8
unknown	<=8
unknown	>32
unknown	>32
unknown	<=8
unknown	<=8
unknown	<=8
unknown	>32
unknown	<=8
unknown	<=8
unknown	<=8
unknown	<=8
unknown	<=8
unknown	<=8
unknown	<=8
unknown	<=8
unknown	>32
unknown	<=8
unknown	>32
unknown	<=8
unknown	<=8
unknown	<=8
unknown	<=8
unknown	<=8
unknown	>32
unknown	>32
unknown	
unknown	<=8
unknown	<=8
unknown	<=8
unknown	<=8
unknown	<=8
unknown	<=8
unknown	<=8
unknown	<=8
unknown	<=8
unknown	<=8
unknown	>32
unknown	<=8
unknown	>32
unknown	<=8
unknown	<=8
unknown	<=8
unknown	>32
unknown	<=8

unknown	16
unknown	<=8
unknown	>32
unknown	>32
unknown	>32
unknown	>32
unknown	>32
unknown	>32
unknown	>32
unknown	<=8
unknown	<=8
unknown	<=8
unknown	<=8
unknown	<=8
unknown	<=8
unknown	<=8
unknown	<=8
unknown	<=8
unknown	<=8
unknown	<=8
unknown	<=8
unknown	<=8
unknown	<=8
unknown	<=8
unknown	<=8
unknown	16
unknown	<=8
unknown	
unknown	
unknown	
unknown	
unknown	
unknown	
unknown	
unknown	
unknown	
unknown	
unknown	
unknown	
unknown	
unknown	
unknown	
unknown	
unknown	
unknown	
unknown	
unknown	
unknown	
unknown	
unknown	
unknown	
unknown	
unknown	
unknown	
unknown	
unknown	

unknown	
unknown	
unknown	
unknown	
unknown	
unknown	
unknown	
unknown	
unknown	
unknown	
unknown	
unknown	
unknown	
unknown	
unknown	
unknown	
unknown	
unknown	
unknown	
unknown	
unknown	
unknown	
unknown	
unknown	
unknown	
unknown	
unknown	
unknown	
unknown	>32
unknown	
unknown	
unknown	
unknown	
unknown	
unknown	
unknown	
unknown	
unknown	
unknown	
unknown	
unknown	
unknown	
unknown	
unknown	
unknown	
unknown	
unknown	
unknown	
unknown	
unknown	
unknown	
unknown	
unknown	
unknown	
unknown	
unknown	

unknown	
unknown	
unknown	
unknown	
unknown	
unknown	
unknown	
unknown	
unknown	
unknown	
unknown	
unknown	
unknown	
unknown	
unknown	
unknown	
unknown	
unknown	>32
unknown	
unknown	
unknown	
unknown	
unknown	
unknown	
unknown	
unknown	
unknown	
unknown	
unknown	
unknown	
unknown	
unknown	
unknown	
unknown	
unknown	
unknown	
unknown	
unknown	
unknown	
unknown	
unknown	
unknown	
unknown	>32
unknown	
unknown	
unknown	
unknown	
unknown	
unknown	
unknown	
unknown	
unknown	
unknown	
unknown	
unknown	
unknown	
unknown	
unknown	

unknown	
unknown	
unknown	
unknown	
unknown	
unknown	
unknown	
unknown	
unknown	
unknown	
unknown	
unknown	
unknown	
unknown	
unknown	
unknown	
unknown	
unknown	
unknown	
unknown	
unknown	>32
unknown	
unknown	
unknown	
unknown	
unknown	
unknown	
unknown	
unknown	
unknown	
unknown	
unknown	
unknown	
unknown	
unknown	
unknown	
unknown	
unknown	
unknown	>32
unknown	>32
unknown	>32
unknown	>32
unknown	<=8
unknown	<=8
unknown	<=8
unknown	<=8
unknown	>32
unknown	<=8
unknown	>32
unknown	<=8
unknown	<=8
unknown	<=8
unknown	<=8
unknown	<=8
unknown	<=8
unknown	<=8

unknown	<=8
unknown	>32
unknown	>32
unknown	>32
unknown	<=8
unknown	<=8
unknown	>32
unknown	<=8
unknown	<=8
unknown	<=8
unknown	>32
unknown	>32
unknown	<=8
unknown	32
unknown	16
unknown	<=8
unknown	<=8
unknown	<=8
unknown	<=8
unknown	<=8
unknown	>32
unknown	<=8
unknown	<=8
unknown	<=8
unknown	<=8
unknown	<=8
unknown	32
unknown	32
unknown	<=8
unknown	<=8
unknown	>32
unknown	32
unknown	>32
unknown	>32
unknown	>32
unknown	<=8
unknown	<=8
unknown	<=8
unknown	>32
unknown	>32
unknown	16
unknown	<=8
unknown	32
unknown	>32
unknown	<=8
unknown	<=8
unknown	<=8
unknown	<=8
unknown	>32
unknown	>32
unknown	>32
unknown	>32
unknown	>32
unknown	<=8
unknown	<=8
unknown	>32

unknown	>32
unknown	>32
unknown	>32
unknown	32
unknown	
unknown	>32
unknown	<=8
unknown	<=8
unknown	<=8
unknown	16
unknown	<=8
unknown	<=8
unknown	<=8
unknown	<=8
unknown	<=8
unknown	<=8
unknown	<=8
unknown	<=8
unknown	>32
unknown	<=8
unknown	<=8
unknown	32
unknown	<=8
unknown	>32
unknown	32
unknown	32
unknown	32
unknown	16
unknown	32
unknown	16
unknown	32
unknown	16
unknown	<=8
unknown	<=8
unknown	16
unknown	>32
unknown	>32
unknown	>32
unknown	16
unknown	<=8
unknown	>32
unknown	>32
unknown	<=8
unknown	<=8
unknown	>32
unknown	<=8
unknown	<=8
unknown	16
unknown	>32
unknown	<=8
unknown	<=8
unknown	<=8
unknown	<=8
unknown	>32
unknown	16
unknown	<=8

unknown	<=8
unknown	<=8
unknown	<=8
unknown	<=8
unknown	<=8
unknown	>32
unknown	>32
unknown	<=8
unknown	>32
unknown	<=8
unknown	<=8
unknown	<=8
unknown	<=8
unknown	>32
unknown	<=8
unknown	<=8
unknown	<=8
unknown	<=8
unknown	>32
unknown	<=8
unknown	>32
unknown	>32
unknown	<=8
unknown	<=8
unknown	<=8
unknown	<=8
unknown	<=8
unknown	<=8
unknown	<=8
unknown	<=8
unknown	<=8
unknown	16
unknown	<=8
unknown	<=8
unknown	<=8
unknown	16
unknown	<=8
unknown	>32
unknown	>32
unknown	<=8
unknown	<=8
unknown	<=8
unknown	>32
unknown	<=8
unknown	<=8
unknown	<=8
unknown	<=8
unknown	<=8
unknown	<=8
unknown	16
unknown	<=8
unknown	<=8
unknown	
unknown	<=8
unknown	>32
unknown	>32

unknown	>32
unknown	>32
unknown	<=8
unknown	<=8
unknown	<=8
unknown	>32
unknown	<=8
unknown	<=8
unknown	<=8
unknown	<=8
unknown	<=8
unknown	>32
unknown	<=8
unknown	16
unknown	<=8
unknown	>32
unknown	<=8
unknown	<=8
unknown	<=8
unknown	<=8
unknown	32
unknown	<=8
unknown	<=8
unknown	<=8
unknown	<=8
unknown	<=8
unknown	<=8
unknown	<=8
unknown	<=8
unknown	<=8
unknown	<=8
unknown	>32
unknown	<=8
unknown	16
unknown	<=8
unknown	<=8
unknown	16
unknown	<=8
unknown	<=8
unknown	>32
unknown	<=8
unknown	<=8
unknown	<=8
unknown	>32
unknown	<=8
unknown	<=8
unknown	>32
unknown	<=8
unknown	32
unknown	<=8
unknown	<=8
unknown	<=8
unknown	<=8
unknown	<=8
unknown	>32
unknown	<=8

unknown	<=8
unknown	<=8
unknown	<=8
unknown	<=8
unknown	<=8
unknown	16
unknown	>32
unknown	<=8
unknown	<=8
unknown	<=8
unknown	<=8
unknown	32
unknown	<=8
unknown	<=8
unknown	<=8
unknown	>32
unknown	<=8
unknown	<=8
unknown	<=8
unknown	16
unknown	<=8
unknown	>32
unknown	<=8
unknown	<=8
unknown	<=8
unknown	<=8
unknown	>32
unknown	<=8
unknown	<=8
unknown	<=8
unknown	<=8
unknown	>32
unknown	>32
unknown	<=8
unknown	>32
unknown	>32
unknown	16
unknown	<=8
unknown	<=8
unknown	16
unknown	
unknown	<=8
unknown	<=8
unknown	
unknown	
unknown	
unknown	
unknown	
unknown	
unknown	
unknown	
unknown	
unknown	
unknown	
unknown	
unknown	

unknown	
unknown	
unknown	
unknown	
unknown	
unknown	
unknown	
unknown	
unknown	
unknown	
unknown	
unknown	
unknown	
unknown	
unknown	
unknown	
unknown	
unknown	
unknown	
unknown	
unknown	
unknown	
unknown	
unknown	
unknown	
unknown	
unknown	
unknown	
unknown	
unknown	
unknown	
unknown	
unknown	
unknown	
unknown	
unknown	
unknown	
unknown	
unknown	<=8
unknown	
unknown	
unknown	
unknown	
unknown	
unknown	
unknown	
unknown	
unknown	
unknown	
unknown	
unknown	
unknown	
unknown	
unknown	
unknown	
unknown	

unknown
unknown
unknown
unknown
unknown
unknown
unknown
unknown
unknown
unknown
unknown
unknown
unknown
unknown
unknown
unknown
unknown
unknown
unknown
unknown
unknown
unknown
unknown
unknown
unknown
unknown
unknown
unknown
unknown
unknown
unknown
unknown
unknown
unknown
unknown
unknown
unknown
unknown
unknown
unknown
unknown
unknown
unknown
unknown
unknown
unknown
unknown
unknown
unknown
unknown
unknown
unknown
unknown
unknown
unknown
unknown

unknown
unknown
unknown
unknown
unknown
unknown
unknown
unknown
unknown
unknown
unknown
unknown
unknown
unknown
unknown
unknown
unknown
unknown
unknown
unknown
unknown
unknown
unknown
unknown
unknown
unknown
unknown
unknown
unknown
unknown
unknown
unknown
unknown
unknown
unknown
unknown
unknown
unknown
unknown
unknown
unknown
unknown
unknown
unknown
unknown
unknown
unknown
unknown
unknown
unknown
unknown
unknown
unknown
unknown
unknown
unknown

unknown
unknown
unknown
unknown
unknown
unknown
unknown
unknown
unknown
unknown
unknown
unknown
unknown
unknown
unknown
unknown
unknown
unknown
unknown
unknown
unknown
unknown
unknown
unknown
unknown
unknown
unknown
unknown
unknown
unknown
unknown
unknown
unknown
unknown
unknown
unknown
unknown
unknown
unknown
unknown
unknown
unknown
unknown
unknown
unknown
unknown
unknown
unknown
unknown
unknown
unknown
unknown
unknown
unknown
unknown
unknown

unknown	
unknown	
unknown	
unknown	
unknown	
unknown	
unknown	
unknown	
unknown	
unknown	
unknown	
unknown	
unknown	
unknown	
unknown	
unknown	
unknown	
unknown	
unknown	
unknown	
unknown	
unknown	
unknown	
unknown	
unknown	
unknown	
unknown	
unknown	
unknown	<=8
unknown	
unknown	
unknown	<=8
unknown	
unknown	<=8
unknown	
unknown	<=8
unknown	<=8
unknown	>32
unknown	<=8
unknown	16
unknown	>32
unknown	
unknown	32
unknown	>32
unknown	>32
unknown	16
unknown	<=8
unknown	<=8
unknown	<=8
unknown	<=8
unknown	<=8
unknown	<=8
unknown	>32
unknown	<=8
unknown	<=8
unknown	<=8

unknown	>32
unknown	>32
unknown	>32
unknown	>32
unknown	<=8
unknown	>32
unknown	>32
unknown	>32
unknown	>32
unknown	>32
unknown	>32
unknown	>32
unknown	>32
unknown	<=8
unknown	>32
unknown	>32
unknown	>32
unknown	<=8
unknown	32
unknown	<=8
unknown	>32
unknown	<=8
unknown	<=8
unknown	<=8
unknown	<=8
unknown	>32
unknown	>32
unknown	<=8
unknown	<=8
unknown	<=8
unknown	16
unknown	<=8
unknown	>32
unknown	<=8
unknown	<=8
unknown	<=8
unknown	<=8
unknown	>32
unknown	<=8
unknown	<=8
unknown	>32
unknown	<=8
unknown	<=8
unknown	<=8
unknown	<=8
unknown	<=8
unknown	<=8
unknown	<=8
unknown	>32
unknown	>32
unknown	16
unknown	<=8
unknown	<=8
unknown	<=8
unknown	<=8
unknown	<=8

unknown	>32
unknown	32
unknown	<=8
unknown	<=8
unknown	<=8
unknown	>32
unknown	>32
unknown	>32
unknown	<=8
unknown	>32
unknown	<=8
unknown	16
unknown	<=8
unknown	<=8
unknown	<=8
unknown	<=8
unknown	<=8
unknown	<=8
unknown	<=8
unknown	<=8
unknown	<=8
unknown	>32
unknown	>32
unknown	16
unknown	<=8
unknown	16
unknown	<=8
unknown	<=8
unknown	<=8
unknown	>32
unknown	<=8
unknown	<=8
unknown	>32
unknown	<=8
unknown	<=8
unknown	<=8
unknown	16
unknown	<=8
unknown	<=8
unknown	>32
unknown	>32
unknown	16
unknown	<=8
unknown	<=8
unknown	<=8
unknown	16
unknown	<=8
unknown	<=8
unknown	<=8
unknown	>32
unknown	<=8
unknown	>32
unknown	<=8
unknown	<=8
unknown	<=8
unknown	>32

unknown	<=8
unknown	>32
unknown	16
unknown	>32
unknown	<=8
unknown	>32
unknown	>32
unknown	<=8
unknown	>32
unknown	<=8
unknown	<=8
unknown	<=8
unknown	>32
unknown	>32
unknown	>32
unknown	>32
unknown	<=8
unknown	<=8
unknown	<=8
unknown	>32
unknown	<=8
unknown	<=8
unknown	<=8
unknown	>32
unknown	<=8
unknown	<=8
unknown	>32
unknown	>32
unknown	
unknown	
unknown	
unknown	
unknown	
unknown	
unknown	
unknown	
unknown	
unknown	
unknown	
unknown	
unknown	
unknown	
unknown	
unknown	
unknown	
unknown	
unknown	
unknown	
unknown	
unknown	
unknown	
unknown	
unknown	
unknown	
unknown	
unknown	

unknown
unknown
unknown
unknown
unknown
unknown
unknown
unknown
unknown
unknown
unknown
unknown
unknown
unknown
unknown
unknown
unknown
unknown
unknown
unknown
unknown
unknown
unknown
unknown
unknown
unknown
unknown
unknown
unknown
unknown
unknown
unknown
unknown
unknown
unknown
unknown
unknown
unknown
unknown
unknown
unknown
unknown
unknown
unknown
unknown
unknown
unknown
unknown
unknown
unknown
unknown
unknown
unknown
unknown
unknown
unknown

unknown
unknown
unknown
unknown
unknown
unknown
unknown
unknown
unknown
unknown
unknown
unknown
unknown
unknown
unknown
unknown
unknown
unknown
unknown
unknown
unknown
unknown
unknown
unknown
unknown
unknown
unknown
unknown
unknown
unknown
unknown
unknown
unknown
unknown
unknown
unknown
unknown
unknown
unknown
unknown
unknown
unknown
unknown
unknown
unknown
unknown
unknown
unknown
unknown
unknown
unknown
unknown
unknown
unknown
unknown
unknown

unknown
unknown
unknown
unknown
unknown
unknown
unknown
unknown
unknown
unknown
unknown
unknown
unknown
unknown
unknown
unknown
unknown
unknown
unknown
unknown
unknown
unknown
unknown
unknown
unknown
unknown
unknown
unknown
unknown
unknown
unknown
unknown
unknown
unknown
unknown
unknown
unknown
unknown
unknown
unknown
unknown
unknown
unknown
unknown
unknown
unknown
unknown
unknown
unknown
unknown
unknown
unknown
unknown
unknown
unknown
unknown

unknown
unknown
unknown
unknown
unknown
unknown
unknown
unknown
unknown
unknown
unknown
unknown
unknown
unknown
unknown
unknown
unknown
unknown
unknown
unknown
unknown
unknown
unknown
unknown
unknown
unknown
unknown
unknown
unknown
unknown
unknown
unknown
unknown
unknown
unknown
unknown
unknown
unknown
unknown
unknown
unknown
unknown
unknown
unknown
unknown
unknown
unknown
unknown
unknown
unknown
unknown
unknown
unknown
unknown
unknown
unknown

unknown
unknown
unknown
unknown
unknown
unknown
unknown
unknown
unknown
unknown
unknown
unknown
unknown
unknown
unknown
unknown
unknown
unknown
unknown
unknown
unknown
unknown
unknown
unknown
unknown
unknown
unknown
unknown
unknown
unknown
unknown
unknown
unknown
unknown
unknown
unknown
unknown
unknown
unknown
unknown
unknown
unknown
unknown
unknown
unknown
unknown
unknown
unknown
unknown
unknown
unknown
unknown
unknown
unknown
unknown
unknown

unknown
unknown
unknown
unknown
unknown
unknown
unknown
unknown
unknown
unknown
unknown
unknown
unknown
unknown
unknown
unknown
unknown
unknown
unknown
unknown
unknown
unknown
unknown
unknown
unknown
unknown
unknown
unknown
unknown
unknown
unknown
unknown
unknown
unknown
unknown
unknown
unknown
unknown
unknown
unknown
unknown
unknown
unknown
unknown
unknown
unknown
unknown
unknown
unknown
unknown
unknown
unknown
unknown
unknown
unknown
unknown

unknown
unknown
unknown
unknown
unknown
unknown
unknown
unknown
unknown
unknown
unknown
unknown
unknown
unknown
unknown
unknown
unknown
unknown
unknown
unknown
unknown
unknown
unknown
unknown
unknown
unknown
unknown
unknown
unknown
unknown
unknown
unknown
unknown
unknown
unknown
unknown
unknown
unknown
unknown
unknown
unknown
unknown
unknown
unknown
unknown
unknown
unknown
unknown
unknown
unknown
unknown

ami	augmic	aug	asmic	as	ampmic	amp	aztmic	azt	cpemic	cpe
S	>16/8	R	>16/8	R	>16	R	>16	R	>16	R
S	>16/8	R	>16/8	R	>16	R	>16	R	>16	R
R	>16/8	R	>16/8	R	>16	R	>16	R	>16	R
R	>16/8	R	>16/8	R	>16	R	16	R	>16	R
S	>16/8	R	>16/8	R	>16	R	>16	R	>16	R
R	>16/8	R	>16/8	R	>16	R	8	I	>16	R
S	>16/8	R	>16/8	R	>16	R	>16	R	>16	R
S	>16/8	R	>16/8	R	>16	R	>16	R	>16	R
S	>16/8	R	>16/8	R	>16	R*	>16	R	>16	R*
S	>16/8	R	>16/8	R	>16	R	>16	R	>16	R
R	>16/8	R	>16/8	R	>16	R	>16	R	16	I
S	>16/8	R	>16/8	R	>16	R*	>16	R	>16	R*
R	>16/8	R	>16/8	R	>16	R	>16	R	>16	R
R	>16/8	R	>16/8	R	>16	R	>16	R	>16	R
S	>16/8	R	>16/8	R	>16	R	>16	R	>16	R
S	>16/8	R	>16/8	R	>16	R	>16	R	>16	R
S	>16/8	R	>16/8	R	>16	R	>16	R	4	S
R	>16/8	R	>16/8	R	>16	R	>16	R	>16	R
R	>16/8	R	>16/8	R	>16	R	>16	R	>16	R
R	>16/8	R	>16/8	R	>16	R	>16	R	>16	R
R	>16/8	R	>16/8	R	>16	R	>16	R	>16	R
R	>16/8	R	>16/8	R	>16	R	>16	R	>16	R
R	>16/8	R	>16/8	R	>16	R	>16	R	>16	R
R	>16/8	R	>16/8	R	>16	R	>16	R	>16	R
R	>16/8	R	>16/8	R	>16	R	>16	R	>16	R
S	>16/8	R	>16/8	R	>16	R	>16	R	>16	R
S	>16/8	R	>16/8	R	>16	R	>16	R	>16	R
R	>16/8	R	>16/8	R	>16	R	>16	R	>16	R
R	>16/8	R	>16/8	R	>16	R	>16	R	>16	R
S	>16/8	R	>16/8	R	>16	R	16	R	>16	R
R	>16/8	R	>16/8	R	>16	R	>16	R	>16	R
S	>16/8	R	>16/8	R	>16	R	<=1	S	<=1	S
S	>16/8	R	>16/8	R	>16	R*	>16	R	>16	R*
R	>16/8	R	>16/8	R	>16	R*	>16	R	>16	R*

S	>16/8	R	>16/8	R	>16	R*	>16	R	>16	R*
R	>16/8	R	>16/8	R	>16	R	>16	R	>16	R
R	>16/8	R	>16/8	R	>16	R	>16	R	>16	R
R	>16/8	R	>16/8	R	>16	R	16	R	>16	R
R	>16/8	R	>16/8	R	>16	R	>16	R	>16	R
R	>16/8	R	>16/8	R	>16	R	<=1	S	>16	R
R	>16/8	R	>16/8	R	>16	R	>16	R	>16	R
S	>16/8	R	>16/8	R	>16	R	>16	R	>16	R
S	>16/8	R	>16/8	R	>16	R	>16	R	>16	R
S	>16/8	R	>16/8	R	>16	R	16	R	16	I
S	>16/8	R	>16/8	R	>16	R	>16	R	>16	R
R	>16/8	R	>16/8	R	>16	R	<=1	S	>16	R
S	>16/8	R	>16/8	R	>16	R	<=1	S	<=1	S
R	>16/8	R	>16/8	R	>16	R	>16	R	>16	R
S	>16/8	R	>16/8	R	>16	R	>16	R	>16	R
R	>16/8	R	>16/8	R	>16	R	>16	R	>16	R
S	>16/8	R	>16/8	R	>16	R	>16	R	>16	R

S	>16/8	R	>16/8	R	>16	R*	>16	R	>16	R*
S	>16/8	R	>16/8	R	>16	R	<=1	S	<=1	S
S	>16/8	R	>16/8	R	>16	R*	>16	R	>16	R*
S	>16/8	R	>16/8	R	>16	R*	>16	R	>16	R*
S	>16/8	R	>16/8	R	>16	R*	>16	R	>16	R*
S	>16/8	R	>16/8	R	>16	R	<=1	S	<=1	S
S	>16/8	R	>16/8	R	>16	R	>16	R	>16	R
R	>16/8	R	>16/8	R	>16	R	>16	R	>16	R
R	>16/8	R	>16/8	R	>16	R	<=1	S	>16	R
R	>16/8	R	>16/8	R	>16	R	>16	R	>16	R
S	>16/8	R	>16/8	R	>16	R	>16	R	>16	R
I	>16/8	R	>16/8	R	>16	R	>16	R	>16	R
S	>16/8	R	>16/8	R	>16	R	>16	R	>16	R

R	>16/8	R	>16/8	R	>16	R	>16	R	>16	R
S	>16/8	R	>16/8	R	>16	R	4	S	4	S
S	>16/8	R	>16/8	R	>16	R*	>16	R	>16	R*
S	>16/8	R	>16/8	R	>16	R*	>16	R	>16	R*
R	>16/8	R	>16/8	R	>16	R	>16	R	>16	R
S	>16/8	R	>16/8	R	>16	R*	>16	R	>16	R*
I	>16/8	R	>16/8	R	>16	R*	>16	R	>16	R*
S	>16/8	R	>16/8	R	>16	R	<=1	S	<=1	S
R	>16/8	R	>16/8	R	>16	R	>16	R	>16	R
S	>16/8	R	>16/8	R	>16	R	>16	R	>16	R
S	>16/8	R	>16/8	R	>16	R*	>16	R*	>16	R*
S	>16/8	R	>16/8	R	>16	R	>16	R	>16	R
R	>16/8	R	>16/8	R	>16	R	4	S	>16	R
R	>16/8	R	>16/8	R	>16	R	>16	R	>16	R
S	>16/8	R	>16/8	R	>16	R*	>16	R*	>16	R*
R	>16/8	R	>16/8	R	>16	R	>16	R	>16	R
R	>16/8	R	>16/8	R	>16	R	>16	R	>16	R
R	>16/8	R	>16/8	R	>16	R	>16	R	>16	R
R	>16/8	R	>16/8	R	>16	R	>16	R	>16	R

S	>16/8	R	>16/8	R	>16	R	>16	R	>16	R
S	>16/8	R	16/8	I	>16	R	<=1	S	<=1	S

S	>16/8	R	>16/8	R	>16	R*	>16	R	>16	R*
R	>16/8	R	>16/8	R	>16	R	>16	R	>16	R
R	>16/8	R	>16/8	R	>16	R	>16	R	>16	R
R	>16/8	R	>16/8	R	>16	R	>16	R	>16	R
R	>16/8	R	>16/8	R	>16	R	>16	R	>16	R
R	>16/8	R	>16/8	R	>16	R	>16	R	>16	R
R	>16/8	R	>16/8	R	>16	R	>16	R	>16	R
R	>16/8	R	>16/8	R	>16	R	>16	R	>16	R
S	>16/8	R	>16/8	R	>16	R*	>16	R	>16	R*
S	>16/8	R	>16/8	R	>16	R	>16	R	>16	R
S	>16/8	R	>16/8	R	>16	R*	>16	R	>16	R*
S	>16/8	R	>16/8	R	>16	R	>16	R	>16	R
S	>16/8	R	>16/8	R	>16	R	>16	R	>16	R
S	>16/8	R	>16/8	R	>16	R	>16	R	>16	R
I	>16/8	R	>16/8	R	>16	R	>16	R	>16	R
S	>16/8	R	>16/8	R	>16	R	>16	R	>16	R
S	>16/8	R	<=8/4	S	<=8	S	<=1	S	<=1	S
S	>16/8	R	>16/8	R	>16	R*	>16	R	>16	R*
I	>16/8	R	>16/8	R	>16	R	>16	R	>16	R
R	>16/8	R	>16/8	R	>16	R	>16	R	>16	R
R	>16/8	R	>16/8	R	>16	R	>16	R	>16	R
R	>16/8	R	>16/8	R	>16	R	<=1	S	>16	R
R	>16/8	R	>16/8	R	>16	R	>16	R	>16	R
S	>16/8	R	>16/8	R	>16	R	>16	R	>16	R
R	>16/8	R	>16/8	R	>16	R	>16	R	>16	R
S	>16/8	R	>16/8	R	>16	R*	>16	R	>16	R*
S	>16/8	R	>16/8	R	>16	R*	>16	R	>16	R
S	>16/8	R	>16/8	R	>16	R	>16	R	>16	R
I	>16/8	R	>16/8	R	>16	R	>16	R	>16	R
S	>16/8	R	>16/8	R	>16	R	16	R	<=1	S
S	>16/8	R	>16/8	R	>16	R	>16	R	16	I
S	>16/8	R	>16/8	R	>16	R	>16	R	>16	R
S	>16/8	R	>16/8	R	>16	R	>16	R	>16	R
S	>16/8	R	>16/8	R	>16	R	>16	R	>16	R
S	>16/8	R	>16/8	R	>16	R*	>16	R	>16	R
S	>16/8	R	>16/8	R	>16	R*	>16	R	>16	R*
S	>16/8	R	>16/8	R	>16	R	>16	R	>16	R
S	>16/8	R	>16/8	R	>16	R	>16	R	>16	R
S	>16/8	R	>16/8	R	>16	R	>16	R	>16	R
S	>16/8	R	>16/8	R	>16	R	>16	R	>16	R
R	>16/8	R	>16/8	R	>16	R	>16	R	>16	R
R	>16/8	R	>16/8	R	>16	R	>16	R	>16	R
S	>16/8	R	>16/8	R	>16	R	<=1	S	<=1	S
S	>16/8	R	>16/8	R	>16	R*	>16	R	>16	R*
R	>16/8	R	>16/8	R	>16	R	>16	R	>16	R
S	>16/8	R	>16/8	R	>16	R*	>16	R	>16	R*
S	>16/8	R	>16/8	R	>16	R	<=1	S	<=1	S
S	>16/8	R	>16/8	R	>16	R	>16	R	4	S
I	>16/8	R	>16/8	R	>16	R	>16	R	>16	R
S	>16/8	R	>16/8	R	>16	R	>16	R	>16	R

S	>16/8	R	>16/8	R	>16	R	>16	R	>16	R
S	>16/8	R	>16/8	R	>16	R*	>16	R	>16	R*
R	>16/8	R	>16/8	R	>16	R	>16	R	>16	R
S	>16/8	R	>16/8	R	>16	R	>16	R	>16	R
S	>16/8	R	>16/8	R	>16	R	>16	R	>16	R
R	>16/8	R	>16/8	R	>16	R	>16	R	>16	R
S	>16/8	R	>16/8	R	>16	R	>16	R	>16	R
R	>16/8	R	>16/8	R	>16	R	>16	R	>16	R
S	>16/8	R	>16/8	R	>16	R*	>16	R	>16	R*
R	>16/8	R	>16/8	R	>16	R	>16	R	>16	R
S	>16/8	R	>16/8	R	>16	R*	>16	R	>16	R*
R	>16/8	R	>16/8	R	>16	R	>16	R	>16	R
R	>16/8	R	>16/8	R	>16	R	>16	R	>16	R
S	>16/8	R	>16/8	R	>16	R	<=1	S	4	S
S	>16/8	R	>16/8	R	>16	R	>16	R	>16	R
S	>16/8	R	>16/8	R	>16	R*	>16	R	16	R*
S	16/8	I	>16/8	R	>16	R*	>16	R	>16	R*
S	>16/8	R	>16/8	R	>16	R	>16	R	>16	R
R	>16/8	R	>16/8	R	>16	R	4	S	>16	R
S	>16/8	R	>16/8	R	>16	R	>16	R	>16	R
R	>16/8	R	>16/8	R	>16	R	>16	R	>16	R
S	>16/8	R	>16/8	R	>16	R	>16	R	>16	R
R	>16/8	R	>16/8	R	>16	R	>16	R	>16	R
S	>16/8	R	>16/8	R	>16	R	<=1	S	<=1	S
S	>16/8	R	>16/8	R	>16	R*	>16	R	>16	R*
S	>16/8	R	>16/8	R	>16	R*	>16	R	>16	R*
R	>16/8	R	>16/8	R	>16	R	>16	R	>16	R
R	>16/8	R	>16/8	R	>16	R	>16	R	>16	R
R	>16/8	R	>16/8	R	>16	R	<=1	S	>16	R

R	>16/8	R	>16/8	R	>16	R	>16	R	>16	R
S	>16/8	R	>16/8	R	>16	R	>16	R	>16	R
S	>16/8	R	>16/8	R	>16	R*	>16	R	>16	R*
S	>16/8	R	<=8/4	S	>16	R	<=1	S	<=1	S
S	16/8	I	>16/8	R	>16	R*	16	R	16	R*

S	>16/8	R	>16/8	R	>16	R	>16	R	>16	R
S	>16/8	R	>16/8	R	>16	R	>16	R	>16	R
S	>16/8	R	>16/8	R	>16	R	<=1	S	<=1	S
S	>16/8	R	>16/8	R	>16	R	>16	R	>16	R
S	>16/8	R	>16/8	R	>16	R*	>16	R	>16	R*
S	>16/8	R	>16/8	R	>16	R	>16	R	>16	R
S	>16/8	R	>16/8	R	>16	R*	>16	R	16	R*
S	>16/8	R	>16/8	R	>16	R*	>16	R	>16	R*
S	>16/8	R	>16/8	R	>16	R	<=1	S	>16	R
S	>16/8	R	>16/8	R	>16	R	>16	R	>16	R
S	>16/8	R	>16/8	R	>16	R	>16	R	>16	R
S	>16/8	R	>16/8	R	>16	R	>16	R	>16	R
S	>16/8	R	>16/8	R	>16	R*	>16	R	>16	R*
R	>16/8	R	>16/8	R	>16	R	>16	R	>16	R

R	>16/8	R	>16/8	R	>16	R	>16	R	>16	R
S	>16/8	R	>16/8	R	>16	R	>16	R	>16	R
S	>16/8	R	>16/8	R	>16	R	>16	R	>16	R
R	>16/8	R	>16/8	R	>16	R	>16	R	>16	R
S	>16/8	R	>16/8	R	>16	R	<=1	S	<=1	S
S	>16/8	R	>16/8	R	>16	R*	>16	R	>16	R*
S	>16/8	R	>16/8	R	>16	R*	>16	R	16	R*
S	>16/8	R	>16/8	R	>16	R*	>16	R	>16	R*
S	>16/8	R	>16/8	R	>16	R	>16	R	>16	R
S	>16/8	R	>16/8	R	>16	R	>16	R	>16	R
I	>16/8	R	>16/8	R	>16	R	>16	R	>16	R
S	>16/8	R	>16/8	R	>16	R	>16	R	>16	R
S	>16/8	R	>16/8	R	>16	R	>16	R	>16	R
R	>16/8	R	>16/8	R	>16	R	>16	R	>16	R
S	>16/8	R	>16/8	R	>16	R*	>16	R	>16	R*
S	>16/8	R	>16/8	R	>16	R*	>16	R	>16	R*
S	>16/8	R	>16/8	R	>16	R*	>16	R	>16	R*
S	>16/8	R	>16/8	R	>16	R	>16	R	>16	R
R	>16/8	R	>16/8	R	>16	R*	>16	R	>16	R*
R	>16/8	R	>16/8	R	>16	R	>16	R	>16	R
S	>16/8	R	>16/8	R	>16	R	>16	R	>16	R
S	>16/8	R	>16/8	R	>16	R	<=1	S	>16	R
S	>16/8	R	>16/8	R	>16	R*	>16	R	16	R*
R	>16/8	R	>16/8	R	>16	R	>16	R	>16	R
S	>16/8	R	>16/8	R	>16	R*	>16	R	>16	R*
S	>16/8	R	>16/8	R	>16	R	>16	R	>16	R
S	>16/8	R	>16/8	R	>16	R	>16	R	>16	R
S	>16/8	R	>16/8	R	>16	R*	>16	R	>16	R*
R	>16/8	R	>16/8	R	>16	R	>16	R	>16	R
R	>16/8	R	>16/8	R	>16	R	>16	R	>16	R
S	>16/8	R	>16/8	R	>16	R*	>16	R	>16	R*
S	>16/8	R	>16/8	R	>16	R	>16	R	>16	R
R	>16/8	R	>16/8	R	>16	R	>16	R	>16	R
R	>16/8	R	>16/8	R	>16	R	>16	R	>16	R
R	>16/8	R	>16/8	R	>16	R	<=1	S	>16	R
S	>16/8	R	>16/8	R	>16	R*	>16	R	>16	R*
R	>16/8	R	>16/8	R	>16	R	>16	R	>16	R
R	>16/8	R	>16/8	R	>16	R	>16	R	>16	R
S	>16/8	R	>16/8	R	>16	R*	>16	R	16	R*
S	>16/8	R	>16/8	R	>16	R*	>16	R	>16	R*
R	>16/8	R	>16/8	R	>16	R	>16	R	>16	R
S	>16/8	R	>16/8	R	>16	R	<=1	S	<=1	S
S	>16/8	R	>16/8	R	>16	R*	>16	R	>16	R*
S	>16/8	R	>16/8	R	>16	R*	>16	R	16	R*
S	>16/8	R	>16/8	R	>16	R	>16	R	16	I
S	>16/8	R	>16/8	R	>16	R	<=1	S	<=1	S

S	>16/8	R	>16/8	R	>16	R	>16	R	>16	R
S	>16/8	R	>16/8	R	>16	R	>16	R	>16	R
S	>16/8	R	>16/8	R	>16	R	<=1	S	<=1	S
R	>16/8	R	>16/8	R	>16	R	>16	R	>16	R
S	>16/8	R	>16/8	R	>16	R*	>16	R	>16	R*
R	>16/8	R	>16/8	R	>16	R	>16	R	>16	R
S	>16/8	R	>16/8	R	>16	R	>16	R	>16	R
R	>16/8	R	>16/8	R	>16	R	>16	R	>16	R
R	16/8	I	>16/8	R	>16	R*	>16	R	16	R*
S	>16/8	R	>16/8	R	>16	R*	16	R	16	R*
S	>16/8	R	>16/8	R	>16	R	>16	R	>16	R
S	>16/8	R	>16/8	R	>16	R	16	R	>16	R
S	>16/8	R	>16/8	R	>16	R	>16	R	>16	R
S	>16/8	R	>16/8	R	>16	R*	>16	R	>16	R*
S	>16/8	R	>16/8	R	>16	R*	>16	R	>16	R*
S	>16/8	R	>16/8	R	>16	R	>16	R	>16	R
S	>16/8	R	>16/8	R	>16	R	>16	R	>16	R
S	>16/8	R	>16/8	R	>16	R	>16	R	>16	R
S	>16/8	R	>16/8	R	>16	R*	>16	R	>16	R*
S	>16/8	R	>16/8	R	>16	R	>16	R	>16	R
S	>16/8	R	>16/8	R	>16	R*	>16	R	>16	R*
S	>16/8	R	>16/8	R	>16	R	8	I	<=1	S
S	>16/8	R	>16/8	R	>16	R*	>16	R	16	R*
S	>16/8	R	>16/8	R	>16	R*	>16	R	>16	R*
S	>16/8	R	>16/8	R	>16	R*	>16	R	>16	R*
I	>16/8	R	>16/8	R	>16	R	>16	R	>16	R
S	>16/8	R	>16/8	R	>16	R*	>16	R	>16	R*
R	>16/8	R	>16/8	R	>16	R	>16	R	>16	R
R	>16/8	R	>16/8	R	>16	R	<=1	S	>16	R
S	>16/8	R	>16/8	R	>16	R	>16	R	>16	R
S	>16/8	R	>16/8	R	>16	R	>16	R	>16	R
S	>16/8	R	>16/8	R	>16	R	>16	R	>16	R
R	>16/8	R	>16/8	R	>16	R	>16	R	>16	R
S	>16/8	R	>16/8	R	>16	R	<=1	S	<=1	S
R	>16/8	R	>16/8	R	>16	R	>16	R	>16	R
S	>16/8	R	>16/8	R	>16	R*	>16	R	>16	R*
S	>16/8	R	>16/8	R	>16	R*	>16	R	>16	R*
S	>16/8	R	16/8	I	>16	R	<=1	S	<=1	S
S	>16/8	R	>16/8	R	>16	R	>16	R	>16	R
R	>16/8	R	>16/8	R	>16	R	>16	R	>16	R
R	>16/8	R	>16/8	R	>16	R	>16	R	>16	R
S	>16/8	R	>16/8	R	>16	R*	>16	R	>16	R*
S	>16/8	R	>16/8	R	>16	R	<=1	S	4	S
S	>16/8	R	>16/8	R	>16	R	>16	R	>16	R

S	>16/8	R	>16/8	R	>16	R*	16	R	>16	R*		
S	>16/8	R	>16/8	R	>16	R	>16	R	>16	R		
S	<=8/4	S	16/8	I	>16	R*	16	R	8	R*		
R	>16/8	R	>16/8	R	>16	R	>16	R	>16	R		
R	>16/8	R	>16/8	R	>16	R	>16	R	>16	R		
R	>16/8	R	>16/8	R	>16	R	>16	R	>16	R		
S	>16/8	R	>16/8	R	>16	R	>16	R	>16	R		
S	>16/8	R	>16/8	R	>16	R*	>16	R	>16	R*		
S	>16/8	R	>16/8	R	>16	R*	>16	R	>16	R*		
S	>16/8	R	>16/8	R	>16	R*	>16	R	>16	R*		
S	>16/8	R	>16/8	R	>16	R*	>16	R	>16	R*		
R	>16/8	R	>16/8	R	>16	R	>16	R	>16	R		
S	>16/8	R	>16/8	R	>16	R*	>16	R	>16	R*		
S	>16/8	R	>16/8	R	>16	R*	>16	R	>16	R*		
S	>16/8	R	<=8/4	S	>16	R	<=1	S	<=1	S		
R	>16/8	R	>16/8	R	>16	R	>16	R	>16	R		
S	>16/8	R	>16/8	R	>16	R*	>16	R	>16	R*		
R	>16/8	R	>16/8	R	>16	R	16	R	>16	R		
R	>16/8	R	>16/8	R	>16	R	>16	R	>16	R		
S	>16/8	R	>16/8	R	>16	R	>16	R	>16	R		
R	>16/8	R	>16/8	R	>16	R	>16	R	>16	R		

S	>16/8	R	>16/8	R	>16	R	>16	R	>16	R
R	>16/8	R	>16/8	R	>16	R	>16	R	>16	R
R	>16/8	R	>16/8	R	>16	R	>16	R	>16	R
S	>16/8	R	>16/8	R	>16	R	>16	R	>16	R
R	>16/8	R	>16/8	R	>16	R	>16	R	>16	R
R	>16/8	R	>16/8	R	>16	R	>16	R	>16	R
S	>16/8	R	>16/8	R	>16	R	<=1	S	>16	R
S	>16/8	R	>16/8	R	>16	R	>16	R	>16	R

S	>16/8	R	16/8	I	>16	R	<=1	S	<=1	S
S	>16/8	R	>16/8	R	>16	R	16	R	>16	R
S	>16/8	R	>16/8	R	>16	R*	>16	R	>16	R*
S	>16/8	R	>16/8	R	>16	R*	>16	R	>16	R*
S	>16/8	R	>16/8	R	>16	R*	>16	R	>16	R*
S	>16/8	R	>16/8	R	>16	R*	>16	R	>16	R*
R	>16/8	R	>16/8	R	>16	R	>16	R	>16	R
S	>16/8	R	>16/8	R	>16	R*	>16	R	>16	R*
S	>16/8	R	>16/8	R	>16	R*	>16	R	>16	R*
S	>16/8	R	>16/8	R	>16	R*	>16	R	>16	R*
S	>16/8	R	>16/8	R	>16	R	>16	R	>16	R
S	>16/8	R	>16/8	R	>16	R*	>16	R	>16	R*
R	>16/8	R	>16/8	R	>16	R	>16	R	>16	R
S	>16/8	R	>16/8	R	>16	R	>16	R	>16	R
S	>16/8	R	>16/8	R	>16	R*	16	R	8	R*
S	>16/8	R	>16/8	R	>16	R	>16	R	>16	R
S	>16/8	R	>16/8	R	>16	R*	>16	R	>16	R*
R	>16/8	R	>16/8	R	>16	R	<=1	S	>16	R
S	<=8/4	S	<=8/4	S	<=8	S	<=1	S	<=1	S
S	>16/8	R	>16/8	R	>16	R	<=1	S	<=1	S
S	>16/8	R	>16/8	R	>16	R*	>16	R	>16	R*
R	>16/8	R	>16/8	R	>16	R	>16	R	>16	R
R	>16/8	R	>16/8	R	>16	R	>16	R	>16	R
R	>16/8	R	>16/8	R	>16	R	>16	R	>16	R
S	>16/8	R	>16/8	R	>16	R*	>16	R	>16	R*
R	>16/8	R	>16/8	R	>16	R	>16	R	>16	R
R	>16/8	R	>16/8	R	>16	R	>16	R	>16	R
R	>16/8	R	>16/8	R	>16	R	<=1	S	>16	R
S	16/8	I	16/8	I	>16	R*	>16	R	16	R*
R	>16/8	R	>16/8	R	>16	R	>16	R	>16	R
S	>16/8	R	>16/8	R	>16	R*	>16	R	>16	R*
S	>16/8	R	>16/8	R	>16	R	>16	R	>16	R
S	>16/8	R	>16/8	R	>16	R	>16	R	>16	R
S	>16/8	R	>16/8	R	>16	R*	>16	R	>16	R*
S	>16/8	R	>16/8	R	>16	R*	>16	R	>16	R*
R	>16/8	R	>16/8	R	>16	R	>16	R	>16	R
S	16/8	I	>16/8	R	>16	R*	>16	R	>16	R*
S	>16/8	R	>16/8	R	>16	R*	>16	R	>16	R*
S	>16/8	R	>16/8	R	>16	R*	>16	R	>16	R*
S	>16/8	R	>16/8	R	>16	R	>16	R	>16	R
S	>16/8	R	>16/8	R	>16	R*	>16	R	>16	R*
S	>16/8	R	>16/8	R	>16	R*	>16	R	>16	R*
S	16/8	I	>16/8	R	>16	R*	>16	R	>16	R*
S	>16/8	R	>16/8	R	>16	R*	>16	R	>16	R*
S	>16/8	R	>16/8	R	>16	R	>16	R	>16	R
R	>16/8	R	>16/8	R	>16	R	>16	R	>16	R
S	>16/8	R	>16/8	R	>16	R*	>16	R	>16	R*

R	>16/8	R	>16/8	R	>16	R	>16	R	>16	R
R	>16/8	R	>16/8	R	>16	R	>16	R	>16	R
R	>16/8	R	>16/8	R	>16	R	>16	R	>16	R
S	>16/8	R	>16/8	R	>16	R*	>16	R	>16	R*
S	>16/8	R	>16/8	R	>16	R	16	R	8	S
S	>16/8	R	>16/8	R	>16	R*	>16	R	>16	R*
S	>16/8	R	>16/8	R	>16	R*	>16	R	>16	R*
S	>16/8	R	>16/8	R	>16	R	>16	R	>16	R
R	>16/8	R	>16/8	R	>16	R	>16	R	>16	R
R	>16/8	R	>16/8	R	>16	R	<=1	S	>16	R
S	>16/8	R	>16/8	R	>16	R	>16	R	16	I
R	>16/8	R	>16/8	R	>16	R	>16	R	>16	R
S	>16/8	R	>16/8	R	>16	R	<=1	S	<=1	S
R	>16/8	R	>16/8	R	>16	R	>16	R	>16	R
R	>16/8	R	>16/8	R	>16	R	>16	R	>16	R
R	>16/8	R	>16/8	R	>16	R	>16	R	>16	R
S	16/8	I	>16/8	R	>16	R	>16	R	16	I
S	>16/8	R	>16/8	R	>16	R*	>16	R	16	R*
S	>16/8	R	>16/8	R	>16	R	>16	R	>16	R
S	>16/8	R	>16/8	R	>16	R	>16	R	>16	R
R	>16/8	R	>16/8	R	>16	R	>16	R	>16	R
S	>16/8	R	>16/8	R	>16	R*	>16	R	>16	R*
S	>16/8	R	>16/8	R	>16	R*	>16	R	>16	R*
S	>16/8	R	>16/8	R	>16	R*	>16	R	>16	R*
S	>16/8	R	>16/8	R	>16	R	>16	R	>16	R
S	>16/8	R	>16/8	R	>16	R*	>16	R	>16	R*
S	>16/8	R	>16/8	R	>16	R	<=1	S	>16	R
S	>16/8	R	>16/8	R	>16	R*	>16	R	>16	R*
S	>16/8	R	>16/8	R	>16	R	>16	R	>16	R

S	>16/8	R	>16/8	R	>16	R*	>16	R	>16	R*
S	>16/8	R	>16/8	R	>16	R	>16	R	>16	R
S	>16/8	R	>16/8	R	>16	R	>16	R	>16	R

R	>16/8	R	>16/8	R	>16	R	>16	R	>16	R
S	>16/8	R	>16/8	R	>16	R*	>16	R	>16	R*
S	>16/8	R	>16/8	R	>16	R	>16	R	>16	R

S	>16/8	R	>16/8	R	>16	R	16	R	>16	R
S	<=8/4	S	<=8/4	S	<=8	S	<=1	S	<=1	S
S	>16/8	R	>16/8	R	>16	R	>16	R	>16	R
R	>16/8	R	>16/8	R	>16	R	>16	R	>16	R
S	>16/8	R	>16/8	R	>16	R	>16	R	>16	R
S	>16/8	R	>16/8	R	>16	R	>16	R	>16	R
R	>16/8	R	>16/8	R	>16	R	>16	R	>16	R
S	>16/8	R	>16/8	R	>16	R*	>16	R	>16	R*

S	>16/8	R	>16/8	R	>16	R*	>16	R	>16	R*
S	>16/8	R	>16/8	R	>16	R*	>16	R	>16	R*
S	>16/8	R	>16/8	R	>16	R	>16	R	>16	R
S	>16/8	R	>16/8	R	>16	R	>16	R	>16	R

S	>16/8	R	>16/8	R	>16	R*	>16	R	>16	R*
S	>16/8	R	>16/8	R	>16	R*	>16	R	>16	R*
S	>16/8	R	>16/8	R	>16	R	<=1	S	<=1	S
S	>16/8	R	>16/8	R	>16	R*	>16	R	>16	R*
S	>16/8	R	>16/8	R	>16	R*	>16	R	>16	R*
S	>16/8	R	>16/8	R	>16	R*	>16	R	>16	R*
S	16/8	I	>16/8	R	>16	R	>16	R	>16	R
S	>16/8	R	>16/8	R	>16	R*	>16	R	>16	R*
S	>16/8	R	>16/8	R	>16	R*	>16	R	>16	R*
S	>16/8	R	>16/8	R	>16	R*	8	I	16	R*
S	>16/8	R	>16/8	R	>16	R*	>16	R	>16	R*
S	>16/8	R	>16/8	R	>16	R*	>16	R	>16	R*
S	<=8/4	S	<=8/4	S	>16	R*	>16	R	>16	R*
R	>16/8	R	>16/8	R	>16	R	>16	R	>16	R

R	>16/8	R	>16/8	R	>16	R	>16	R	>16	R
S	>16/8	R	>16/8	R	>16	R	>16	R	>16	R
R	>16/8	R	>16/8	R	>16	R	>16	R	>16	R
S	>16/8	R	>16/8	R	>16	R	>16	R	>16	R
R	>16/8	R	>16/8	R	>16	R	>16	R	>16	R
S	>16/8	R	>16/8	R	>16	R	>16	R	>16	R
R	>16/8	R	>16/8	R	>16	R	>16	R	>16	R
S	>16/8	R	>16/8	R	>16	R	>16	R	>16	R

S	>16/8	R	>16/8	R	>16	R	>16	R	>16	R
S	>16/8	R	>16/8	R	>16	R	>16	R	>16	R
S	>16/8	R	>16/8	R	>16	R	>16	R	>16	R
S	>16/8	R	>16/8	R	>16	R	>16	R	>16	R
S	>16/8	R	>16/8	R	>16	R	>16	R	>16	R
R	>16/8	R	>16/8	R	>16	R	>16	R	>16	R
S	>16/8	R	>16/8	R	>16	R	>16	R	>16	R
S	>16/8	R	>16/8	R	>16	R	>16	R	>16	R
S	>16/8	R	>16/8	R	>16	R	>16	R	>16	R
S	>16/8	R	>16/8	R	>16	R	>16	R	>16	R
S	>16/8	R	>16/8	R	>16	R	16	R	8	S
R	>16/8	R	>16/8	R	>16	R	>16	R	>16	R
R	>16/8	R	>16/8	R	>16	R	>16	R	>16	R
S	>16/8	R	>16/8	R	>16	R	>16	R	>16	R
R	>16/8	R	>16/8	R	>16	R	>16	R	>16	R
R	>16/8	R	>16/8	R	>16	R	<=1	S	>16	R
S	>16/8	R	>16/8	R	>16	R	>16	R	>16	R
R	>16/8	R	>16/8	R	>16	R	>16	R	>16	R
S	>16/8	R	>16/8	R	>16	R	>16	R	>16	R
S	>16/8	R	>16/8	R	>16	R	>16	R	>16	R
I	>16/8	R	>16/8	R	>16	R*	>16	R	>16	R*
R	>16/8	R	>16/8	R	>16	R	>16	R	>16	R
R	>16/8	R	>16/8	R	>16	R	>16	R	>16	R
R	>16/8	R	>16/8	R	>16	R	>16	R	>16	R

S	>16/8	R	>16/8	R	>16	R	<=1	S	<=1	S
S	16/8	I	>16/8	R	>16	R	>16	R	>16	R
S	>16/8	R	>16/8	R	>16	R	>16	R	>16	R
S	>16/8	R	>16/8	R	>16	R	>16	R	>16	R
S	>16/8	R	>16/8	R	>16	R	>16	R	>16	R
S	>16/8	R	>16/8	R	>16	R	>16	R	>16	R

S	>16/8	R	>16/8	R	>16	R	<=1	S	<=1	S
S	>16/8	R	>16/8	R	>16	R	>16	R	>16	R
R	>16/8	R	>16/8	R	>16	R	>16	R	>16	R
S	>16/8	R	>16/8	R	>16	R*	>16	R	>16	R*

S	>16/8	R	>16/8	R	>16	R	>16	R	16	I
S	>16/8	R	>16/8	R	>16	R	>16	R	>16	R
R	>16/8	R	>16/8	R	>16	R	>16	R	>16	R

R	>16/8	R	>16/8	R	>16	R	>16	R	>16	R
R	>16/8	R	>16/8	R	>16	R	>16	R	>16	R
S	>16/8	R	>16/8	R	>16	R	>16	R	>16	R
S	>16/8	R	>16/8	R	>16	R	>16	R	>16	R
S	>16/8	R	>16/8	R	>16	R	<=1	S	<=1	S
S	>16/8	R	>16/8	R	>16	R	>16	R	>16	R
S	>16/8	R	>16/8	R	>16	R	>16	R	>16	R
S	>16/8	R	>16/8	R	>16	R	>16	R	>16	R
S	>16/8	R	>16/8	R	>16	R	>16	R	>16	R
R	>16/8	R	>16/8	R	>16	R	>16	R	>16	R
R	>16/8	R	>16/8	R	>16	R	>16	R	>16	R

R	>16/8	R	>16/8	R	>16	R	>16	R	>16	R
S	>16/8	R	>16/8	R	>16	R	>16	R	>16	R
S	>16/8	R	>16/8	R	>16	R	>16	R	>16	R
R	>16/8	R	>16/8	R	>16	R	>16	R	>16	R
R	>16/8	R	>16/8	R	>16	R	>16	R	>16	R
S	>16/8	R	>16/8	R	>16	R	>16	R	>16	R
R	>16/8	R	>16/8	R	>16	R	>16	R	>16	R
R	>16/8	R	>16/8	R	>16	R	>16	R	>16	R
S	>16/8	R	>16/8	R	>16	R	>16	R	>16	R
I	>16/8	R	>16/8	R	>16	R	>16	R	>16	R
S	>16/8	R	>16/8	R	>16	R	>16	R	>16	R
S	>16/8	R	>16/8	R	>16	R	8	I	>16	R
R	>16/8	R	>16/8	R	>16	R	>16	R	>16	R
S	>16/8	R	>16/8	R	>16	R	>16	R	>16	R
S	>16/8	R	>16/8	R	>16	R	>16	R	>16	R
S	>16/8	R	>16/8	R	>16	R	>16	R	16	I
S	>16/8	R	>16/8	R	>16	R	>16	R	>16	R
S	>16/8	R	>16/8	R	>16	R*	>16	R	16	R*
S	>16/8	R	>16/8	R	>16	R	<=1	S	2	S
S	>16/8	R	>16/8	R	>16	R	>16	R	>16	R
R	>16/8	R	>16/8	R	>16	R	>16	R	>16	R
R	>16/8	R	>16/8	R	>16	R	>16	R	>16	R
S	>16/8	R	>16/8	R	>16	R	<=1	S	<=1	S
S	<=8/4	S	<=8/4	S	>16	R	<=1	S	<=1	S
S	>16/8	R	>16/8	R	>16	R	>16	R	>16	R
S	>16/8	R	>16/8	R	>16	R	>16	R	>16	R
S	>16/8	R	>16/8	R	>16	R	>16	R	>16	R
S	>16/8	R	>16/8	R	>16	R	16	R	>16	R
R	>16/8	R	>16/8	R	>16	R	>16	R	>16	R
R	>16/8	R	>16/8	R	>16	R	>16	R	>16	R
S	>16/8	R	>16/8	R	>16	R	>16	R	>16	R
S	>16/8	R	>16/8	R	>16	R	>16	R	>16	R
R	>16/8	R	>16/8	R	>16	R	<=1	S	>16	R
R	>16/8	R	>16/8	R	>16	R	16	R	>16	R
S	>16/8	R	>16/8	R	>16	R	>16	R	>16	R
R	>16/8	R	>16/8	R	>16	R	>16	R	>16	R
S	16/8	I	>16/8	R	>16	R	>16	R	>16	R
S	>16/8	R	>16/8	R	>16	R	>16	R	>16	R
S	>16/8	R	>16/8	R	>16	R	16	R	>16	R
S	>16/8	R	>16/8	R	>16	R	>16	R	>16	R
S	>16/8	R	>16/8	R	>16	R	>16	R	>16	R
S	>16/8	R	>16/8	R	>16	R	>16	R	>16	R
S	>16/8	R	>16/8	R	>16	R	<=1	S	<=1	S
S	>16/8	R	>16/8	R	>16	R	>16	R	>16	R

R	>16/8	R	>16/8	R	>16	R	>16	R	>16	R
I	>16/8	R	>16/8	R	>16	R	<=1	S	>16	R
R	>16/8	R	>16/8	R	>16	R	>16	R	>16	R
S	>16/8	R	>16/8	R	>16	R	>16	R	>16	R
S	>16/8	R	>16/8	R	>16	R	>16	R	>16	R
S	>16/8	R	>16/8	R	>16	R	>16	R	>16	R
S	>16/8	R	>16/8	R	>16	R	<=1	S	<=1	S
S	16/8	I	>16/8	R	>16	R*	4	S	16	R*
R	>16/8	R	>16/8	R	>16	R	8	I	>16	R
R	>16/8	R	>16/8	R	>16	R	>16	R	>16	R
R	>16/8	R	>16/8	R	>16	R	>16	R	>16	R
R	>16/8	R	>16/8	R	>16	R	>16	R	>16	R
R	>16/8	R	>16/8	R	>16	R	>16	R	>16	R
S	>16/8	R	>16/8	R	>16	R	>16	R	>16	R
S	>16/8	R	>16/8	R	>16	R	>16	R	>16	R
S	>16/8	R	>16/8	R	>16	R*	>16	R	>16	R*
R	>16/8	R	>16/8	R	>16	R	>16	R	>16	R
R	>16/8	R	>16/8	R	>16	R	>16	R	>16	R
S	>16/8	R	>16/8	R	>16	R	>16	R	>16	R
S	>16/8	R	>16/8	R	>16	R	>16	R	>16	R
R	>16/8	R	>16/8	R	>16	R	>16	R	>16	R
S	>16/8	R	>16/8	R	>16	R	16	R	>16	R
S	16/8	I	>16/8	R	>16	R	>16	R	>16	R
R	>16/8	R	>16/8	R	>16	R	>16	R	>16	R
R	>16/8	R	>16/8	R	>16	R	>16	R	>16	R
R	>16/8	R	>16/8	R	>16	R	>16	R	>16	R
R	>16/8	R	>16/8	R	>16	R	>16	R	>16	R
S	>16/8	R	>16/8	R	>16	R	>16	R	>16	R
S	>16/8	R	>16/8	R	>16	R	>16	R	>16	R
R	>16/8	R	>16/8	R	>16	R	>16	R	>16	R
S	>16/8	R	>16/8	R	>16	R	>16	R	>16	R
S	<=8/4	S	<=8/4	S	16	I	<=1	S	<=1	S
S	>16/8	R	>16/8	R	>16	R	<=1	S	>16	R
S	>16/8	R	>16/8	R	>16	R	>16	R	>16	R
R	>16/8	R	>16/8	R	>16	R	>16	R	>16	R
S	>16/8	R	>16/8	R	>16	R	<=1	S	4	S
S	>16/8	R	>16/8	R	>16	R	<=1	S	<=1	S
S	>16/8	R	>16/8	R	>16	R	>16	R	>16	R
S	>16/8	R	>16/8	R	>16	R	>16	R	>16	R
S	>16/8	R	>16/8	R	>16	R	>16	R	>16	R
S	>16/8	R	>16/8	R	>16	R	>16	R	>16	R
R	>16/8	R	>16/8	R	>16	R	>16	R	>16	R
S	>16/8	R	>16/8	R	>16	R	>16	R	>16	R
R	>16/8	R	>16/8	R	>16	R	>16	R	>16	R
S	>16/8	R	>16/8	R	>16	R	>16	R	>16	R
R	>16/8	R	>16/8	R	>16	R	>16	R	>16	R
S	>16/8	R	>16/8	R	>16	R	>16	R	>16	R
R	>16/8	R	>16/8	R	>16	R	>16	R	>16	R
R	>16/8	R	>16/8	R	>16	R	>16	R	>16	R
R	>16/8	R	>16/8	R	>16	R	>16	R	>16	R
R	>16/8	R	>16/8	R	>16	R	>16	R	>16	R
S	>16/8	R	16/8	I	>16	R	<=1	S	<=1	S
S	>16/8	R	>16/8	R	>16	R*	>16	R	>16	R*
R	>16/8	R	>16/8	R	>16	R	>16	R	>16	R
S	>16/8	R	>16/8	R	>16	R	>16	R	>16	R
R	>16/8	R	>16/8	R	>16	R	>16	R	>16	R

R	>16/8	R	>16/8	R	>16	R	>16	R	>16	R	
S	>16/8	R	>16/8	R	>16	R	>16	R	>16	R	
S	>16/8	R	>16/8	R	>16	R	>16	R	>16	R	
S	>16/8	R	>16/8	R	>16	R	>16	R	>16	R	
S	>16/8	R	>16/8	R	>16	R	>16	R	>16	R	
S	>16/8	R	>16/8	R	>16	R	>16	R	>16	R	
S	>16/8	R	>16/8	R	>16	R	>16	R	>16	R	
S	>16/8	R	>16/8	R	>16	R	>16	R	>16	R	
S	>16/8	R	>16/8	R	>16	R	>16	R	>16	R	
S	>16/8	R	>16/8	R	>16	R	>16	R	>16	R	
S	>16/8	R	>16/8	R	>16	R	<=1	S	<=1	S	
S	>16/8	R	>16/8	R	>16	R	>16	R	>16	R	
S	>16/8	R	>16/8	R	>16	R	>16	R	>16	R	
S	>16/8	R	>16/8	R	>16	R	>16	R	>16	R	

S	>16/8	R	>16/8	R	>16	R	>16	R	>16	R
S	>16/8	R	>16/8	R	>16	R	>16	R	>16	R
S	>16/8	R	>16/8	R	>16	R	<=1	S	2	S
S	>16/8	R	>16/8	R	>16	R	>16	R	>16	R
S	>16/8	R	>16/8	R	>16	R*	>16	R	>16	R*
S	>16/8	R	>16/8	R	>16	R	>16	R	>16	R
S	>16/8	R	>16/8	R	>16	R	<=1	S	<=1	S
R	>16/8	R	>16/8	R	>16	R	>16	R	>16	R
R	>16/8	R	>16/8	R	>16	R	>16	R	>16	R
S	>16/8	R	>16/8	R	>16	R	>16	R	>16	R
S	>16/8	R	>16/8	R	>16	R	>16	R	>16	R
S	>16/8	R	>16/8	R	>16	R	<=1	S	<=1	S
S	>16/8	R	<=8/4	S	>16	R	<=1	S	<=1	S
R	>16/8	R	>16/8	R	>16	R	<=1	S	>16	R
S	>16/8	R	>16/8	R	>16	R	>16	R	>16	R
R	>16/8	R	>16/8	R	>16	R	<=1	S	>16	R
S	>16/8	R	>16/8	R	>16	R	>16	R	>16	R
S	>16/8	R	>16/8	R	>16	R	>16	R	>16	R
R	>16/8	R	>16/8	R	>16	R	>16	R	>16	R
S	>16/8	R	>16/8	R	>16	R	>16	R	>16	R
R	>16/8	R	>16/8	R	>16	R	>16	R	>16	R
S	>16/8	R	>16/8	R	>16	R	>16	R	>16	R
S	<=8/4	S	<=8/4	S	16	I	<=1	S	<=1	S
S	>16/8	R	>16/8	R	>16	R	<=1	S	>16	R
S	>16/8	R	>16/8	R	>16	R	>16	R	>16	R
S	>16/8	R	>16/8	R	>16	R	>16	R	>16	R
S	>16/8	R	>16/8	R	>16	R	>16	R	>16	R
S	>16/8	R	>16/8	R	>16	R	>16	R	>16	R
S	>16/8	R	>16/8	R	>16	R	>16	R	>16	R
S	>16/8	R	>16/8	R	>16	R	>16	R	>16	R
S	>16/8	R	>16/8	R	>16	R	<=1	S	<=1	S
S	>16/8	R	>16/8	R	>16	R	<=1	S	<=1	S

S	>16/8	R	>16/8	R	>16	R	4	S	>16	R
S	>16/8	R	>16/8	R	>16	R	>16	R	>16	R
R	>16/8	R	>16/8	R	>16	R	>16	R	>16	R
R	>16/8	R	>16/8	R	>16	R	>16	R	>16	R
S	>16/8	R	>16/8	R	>16	R	>16	R	>16	R
S	>16/8	R	>16/8	R	>16	R	>16	R	>16	R
S	>16/8	R	>16/8	R	>16	R	>16	R	>16	R
S	>16/8	R	>16/8	R	>16	R	>16	R	>16	R
S	>16/8	R	>16/8	R	>16	R	>16	R	>16	R
S	>16/8	R	>16/8	R	>16	R	>16	R	>16	R
S	>16/8	R	>16/8	R	>16	R	>16	R	>16	R
S	>16/8	R	>16/8	R	>16	R	4	S	>16	R
S	>16/8	R	>16/8	R	>16	R	>16	R	>16	R
S	>16/8	R	>16/8	R	>16	R	>16	R	>16	R
S	>16/8	R	>16/8	R	>16	R	>16	R	>16	R
S	>16/8	R	>16/8	R	>16	R	>16	R	>16	R
S	>16/8	R	>16/8	R	>16	R	>16	R	>16	R
S	>16/8	R	>16/8	R	>16	R	>16	R	>16	R
S	>16/8	R	>16/8	R	>16	R	>16	R	>16	R
R	>16/8	R	>16/8	R	>16	R	>16	R	>16	R
S	>16/8	R	>16/8	R	>16	R	>16	R	>16	R
S	>16/8	R	>16/8	R	>16	R	>16	R	>16	R
S	>16/8	R	>16/8	R	>16	R	>16	R	>16	R
S	>16/8	R	>16/8	R	>16	R	>16	R	>16	R
S	>16/8	R	>16/8	R	>16	R	<=1	S	<=1	S
S	>16/8	R	>16/8	R	>16	R	<=1	S	<=1	S
S	>16/8	R	>16/8	R	>16	R*	>16	R	>16	R*
R	>16/8	R	>16/8	R	>16	R	<=1	S	>16	R
S	>16/8	R	>16/8	R	>16	R	<=1	S	4	S
S	>16/8	R	>16/8	R	>16	R*	>16	ESBL	>16	R*
S	>16/8	R	>16/8	R	>16	R	<=1	S	<=1	S
S	>16/8	R	>16/8	R	>16	R*	>16	ESBL	>16	R*
S	>16/8	R	>16/8	R	>16	R*	>16	ESBL	>16	R*
S	>16/8	R	>16/8	R	>16	R*	>16	ESBL	>16	R*
S	>16/8	R	>16/8	R	>16	R	>16	R	>16	R
S	>16/8	R	>16/8	R	>16	R*	8	ESBL	8	R*
R	>16/8	R	>16/8	R	>16	R	>16	R	>16	R
S	>16/8	R	>16/8	R	>16	R*	>16	ESBL	>16	R*
R	>16/8	R	>16/8	R	>16	R	>16	R	>16	R
S	>16/8	R	>16/8	R	>16	R*	>16	ESBL	>16	R*
S	>16/8	R	>16/8	R	>16	R	>16	R	>16	R
R	>16/8	R	>16/8	R	>16	R	>16	R	>16	R
R	>16/8	R	>16/8	R	>16	R	>16	R	>16	R
S	>16/8	R	>16/8	R	>16	R	>16	R	>16	R
R	>16/8	R	>16/8	R	>16	R	>16	R	>16	R
S	>16/8	R	>16/8	R	>16	R*	>16	ESBL	>16	R*
S	>16/8	R	>16/8	R	>16	R*	>16	ESBL	>16	R*
S	>16/8	R	>16/8	R	>16	R	<=1	S	<=1	S
S	>16/8	R	>16/8	R	>16	R	>16	R	>16	R

R	>16/8	R	>16/8	R	>16	R	>16	R	>16	R
S	>16/8	R	>16/8	R	>16	R*	>16	ESBL	>16	R*
S	>16/8	R	>16/8	R	>16	R	<=1	S	<=1	S
S	>16/8	R	>16/8	R	>16	R	>16	R	>16	R
S	>16/8	R	>16/8	R	>16	R	<=1	S	<=1	S
S	>16/8	R	>16/8	R	>16	R*	>16	ESBL	>16	R*
S	>16/8	R	>16/8	R	>16	R	>16	R	>16	R
R	>16/8	R	>16/8	R	>16	R	>16	R	>16	R
S	>16/8	R	>16/8	R	>16	R	>16	R	>16	R
S	>16/8	R	>16/8	R	>16	R	>16	R	>16	R
S	>16/8	R	>16/8	R	>16	R*	8	ESBL	2	R*
S	>16/8	R	>16/8	R	>16	R*	>16	ESBL	>16	R*
S	>16/8	R	>16/8	R	>16	R*	>16	ESBL	>16	R*
R	>16/8	R	>16/8	R	>16	R	>16	R	>16	R
S	>16/8	R	>16/8	R	>16	R*	>16	ESBL	>16	R*
R	>16/8	R	>16/8	R	>16	R	>16	R	>16	R
R	>16/8	R	>16/8	R	>16	R	<=1	S	>16	R
S	>16/8	R	>16/8	R	>16	R	<=1	S	<=1	S
S	>16/8	R	>16/8	R	>16	R*	>16	ESBL	>16	R*
R	>16/8	R	>16/8	R	>16	R	16	R	>16	R
S	>16/8	R	>16/8	R	>16	R	>16	R	>16	R
S	>16/8	R	>16/8	R	>16	R	<=1	S	<=1	S
S	>16/8	R	>16/8	R	>16	R*	>16	ESBL	>16	R*
S	>16/8	R	16/8	I	>16	R	<=1	S	<=1	S
S	>16/8	R	>16/8	R	>16	R	<=1	S	>16	R
S	>16/8	R	>16/8	R	>16	R	>16	R	>16	R
S	>16/8	R	>16/8	R	>16	R*	>16	ESBL	>16	R*

S	>16/8	R	>16/8	R	>16	R*	8	ESBL	4	R*
S	>16/8	R	>16/8	R	>16	R	<=1	S	<=1	S
S	>16/8	R	>16/8	R	>16	R*	>16	ESBL	>16	R*
S	>16/8	R	>16/8	R	>16	R*	>16	ESBL	>16	R*
S	>16/8	R	>16/8	R	>16	R*	>16	ESBL	>16	R*
R	>16/8	R	>16/8	R	>16	R	16	R	>16	R
S	>16/8	R	>16/8	R	>16	R*	>16	ESBL	>16	R*
S	>16/8	R	>16/8	R	>16	R	>16	R	>16	R
S	>16/8	R	>16/8	R	>16	R*	>16	ESBL	>16	R*
S	16/8	I	>16/8	R	>16	R*	>16	ESBL	>16	R*
S	>16/8	R	>16/8	R	>16	R	>16	R	>16	R
S	>16/8	R	16/8	I	>16	R	<=1	S	<=1	S
S	>16/8	R	>16/8	R	>16	R	>16	R	>16	R
R	>16/8	R	>16/8	R	>16	R	>16	R	>16	R

S	>16/8	R	>16/8	R	>16	R	>16	R	>16	R
S	>16/8	R	>16/8	R	>16	R	<=1	S	<=1	S
S	>16/8	R	>16/8	R	>16	R*	>16	ESBL	>16	R*
R	>16/8	R	>16/8	R	>16	R	>16	R	>16	R
S	>16/8	R	>16/8	R	>16	R*	>16	ESBL	>16	R*
R	>16/8	R	>16/8	R	>16	R	>16	R	>16	R
S	>16/8	R	>16/8	R	>16	R	<=1	S	>16	R
S	>16/8	R	>16/8	R	>16	R*	>16	ESBL	>16	R*
S	>16/8	R	>16/8	R	>16	R*	>16	ESBL	>16	R*
S	>16/8	R	>16/8	R	>16	R	<=1	S	<=1	S
S	>16/8	R	>16/8	R	>16	R*	>16	ESBL	>16	R*
S	>16/8	R	>16/8	R	>16	R*	>16	ESBL	>16	R*
S	>16/8	R	>16/8	R	>16	R*	>16	ESBL	>16	R*
S	>16/8	R	>16/8	R	>16	R*	>16	ESBL	>16	R*
S	>16/8	R	>16/8	R	>16	R*	>16	ESBL	>16	R*
S	>16/8	R	>16/8	R	>16	R*	>16	ESBL	>16	R*
S	>16/8	R	>16/8	R	>16	R*	>16	ESBL	>16	R*
S	>16/8	R	>16/8	R	>16	R*	>16	ESBL	>16	R*
S	>16/8	R	>16/8	R	>16	R*	>16	ESBL	>16	R*
S	>16/8	R	>16/8	R	>16	R	>16	R	>16	R
S	>16/8	R	>16/8	R	>16	R*	>16	ESBL	>16	R*
S	>16/8	R	>16/8	R	>16	R*	>16	ESBL	>16	R*
S	>16/8	R	>16/8	R	>16	R*	>16	ESBL	>16	R*
R	>16/8	R	>16/8	R	>16	R	>16	R	>16	R
S	>16/8	R	>16/8	R	>16	R*	>16	ESBL	>16	R*
S	>16/8	R	>16/8	R	>16	R*	>16	ESBL	>16	R*
S	>16/8	R	>16/8	R	>16	R*	>16	ESBL	>16	R*
S	>16/8	R	>16/8	R	>16	R*	>16	ESBL	>16	R*
S	>16/8	R	>16/8	R	>16	R*	>16	ESBL	>16	R*
S	>16/8	R	>16/8	R	>16	R*	>16	ESBL	>16	R*
R	>16/8	R	>16/8	R	>16	R	>16	R	>16	R
S	>16/8	R	>16/8	R	>16	R	>16	R	>16	R
R	>16/8	R	>16/8	R	>16	R	>16	R	>16	R
S	>16/8	R	>16/8	R	>16	R*	>16	ESBL	>16	R*
S	>16/8	R	>16/8	R	>16	R*	>16	ESBL	>16	R*
S	>16/8	R	>16/8	R	>16	R*	>16	ESBL	>16	R*
S	>16/8	R	>16/8	R	>16	R*	>16	ESBL	>16	R*
S	>16/8	R	16/8	I	>16	R	<=1	S	<=1	S
S	>16/8	R	>16/8	R	>16	R	16	R	<=1	S
S	>16/8	R	>16/8	R	>16	R*	>16	ESBL	>16	R*
S	>16/8	R	>16/8	R	>16	R	>16	R	8	S
S	>16/8	R	>16/8	R	>16	R*	>16	ESBL	>16	R*
S	>16/8	R	>16/8	R	>16	R*	>16	ESBL	16	R*
S	>16/8	R	>16/8	R	>16	R*	>16	ESBL	>16	R*
S	>16/8	R	>16/8	R	>16	R*	>16	ESBL	>16	R*
S	>16/8	R	>16/8	R	>16	R	<=1	S	>16	R
S	<=8/4	S	16/8	I	>16	R	<=1	S	<=1	S
S	>16/8	R	>16/8	R	>16	R	>16	R	>16	R
R	>16/8	R	>16/8	R	>16	R	>16	R	>16	R
S	>16/8	R	>16/8	R	>16	R*	>16	ESBL	>16	R*
S	>16/8	R	>16/8	R	>16	R*	>16	ESBL	>16	R*
S	>16/8	R	>16/8	R	>16	R	>16	R	8	S
S	>16/8	R	>16/8	R	>16	R	8	I	8	S
S	>16/8	R	>16/8	R	>16	R*	>16	ESBL	>16	R*

R	>16/8	R	>16/8	R	>16	R	>16	R	>16	R
R	>16/8	R	>16/8	R	>16	R	>16	R	>16	R
S	>16/8	R	>16/8	R	>16	R	>16	R	>16	R
S	>16/8	R	>16/8	R	>16	R	<=1	S	>16	R
R	>16/8	R	>16/8	R	>16	R	>16	R	>16	R
R	>16/8	R	>16/8	R	>16	R	>16	R	>16	R
S	>16/8	R	>16/8	R	>16	R*	>16	ESBL	>16	R*
S	>16/8	R	>16/8	R	>16	R*	>16	ESBL	>16	R*
R	>16/8	R	>16/8	R	>16	R	<=1	S	>16	R
R	>16/8	R	>16/8	R	>16	R	>16	R	>16	R
S	>16/8	R	>16/8	R	>16	R	>16	R	>16	R
R	>16/8	R	16/8	I	>16	R	<=1	S	<=1	S
R	>16/8	R	>16/8	R	>16	R	>16	R	>16	R
S	>16/8	R	>16/8	R	>16	R	>16	R	>16	R
S	>16/8	R	>16/8	R	>16	R	<=1	S	<=1	S
R	>16/8	R	>16/8	R	>16	R	<=1	S	>16	R
S	>16/8	R	>16/8	R	>16	R*	>16	ESBL	>16	R*
S	>16/8	R	>16/8	R	>16	R	>16	R	>16	R
S	>16/8	R	>16/8	R	>16	R*	>16	ESBL	>16	R*
R	>16/8	R	>16/8	R	>16	R	>16	R	>16	R
S	>16/8	R	>16/8	R	>16	R*	>16	ESBL	>16	R*
S	>16/8	R	>16/8	R	>16	R	>16	R	>16	R
R	>16/8	R	>16/8	R	>16	R	>16	R	>16	R
S	>16/8	R	>16/8	R	>16	R*	>16	ESBL	>16	R*
R	>16/8	R	>16/8	R	>16	R	>16	R	>16	R
S	>16/8	R	>16/8	R	>16	R	>16	R	>16	R
S	>16/8	R	>16/8	R	>16	R*	>16	ESBL	>16	R*
S	>16/8	R	>16/8	R	>16	R	>16	R	>16	R
S	>16/8	R	>16/8	R	>16	R	>16	R	>16	R
S	>16/8	R	>16/8	R	>16	R*	>16	ESBL	>16	R*
S	>16/8	R	>16/8	R	>16	R*	>16	ESBL	>16	R*
S	>16/8	R	>16/8	R	>16	R*	>16	ESBL	>16	R*
S	>16/8	R	>16/8	R	>16	R	16	R	>16	R
S	>16/8	R	>16/8	R	>16	R*	>16	ESBL	16	R*
S	>16/8	R	>16/8	R	>16	R*	>16	ESBL	>16	R*
S	>16/8	R	>16/8	R	>16	R*	>16	ESBL	>16	R*
S	>16/8	R	>16/8	R	>16	R*	>16	ESBL	>16	R*
R	>16/8	R	>16/8	R	>16	R	>16	R	>16	R
R	>16/8	R	>16/8	R	>16	R	>16	R	>16	R
S	>16/8	R	>16/8	R	>16	R	<=1	S	>16	R
R	>16/8	R	>16/8	R	>16	R	>16	R	>16	R

S	>16/8	R	>16/8	R	>16	R	16	R	>16	R
S	>16/8	R	>16/8	R	>16	R*	>16	ESBL	>16	R*
S	>16/8	R	>16/8	R	>16	R*	>16	ESBL	>16	R*

S	>16/8	R	>16/8	R	>16	R	<=1	S	4	S
S	>16/8	R	>16/8	R	>16	R*	>16	ESBL	16	R*
S	>16/8	R	>16/8	R	>16	R*	>16	ESBL	>16	R*
S	>16/8	R	>16/8	R	>16	R*	>16	ESBL	>16	R*
S	>16/8	R	>16/8	R	>16	R*	>16	ESBL	16	R*
R	>16/8	R	>16/8	R	>16	R	>16	R	>16	R
S	>16/8	R	>16/8	R	>16	R	>16	R	>16	R
R	>16/8	R	>16/8	R	>16	R	>16	R	>16	R
R	>16/8	R	>16/8	R	>16	R	4	S	<=1	S
S	>16/8	R	>16/8	R	>16	R*	>16	ESBL	>16	R*
S	>16/8	R	>16/8	R	>16	R*	>16	ESBL	>16	R*
S	>16/8	R	>16/8	R	>16	R*	>16	ESBL	>16	R*
S	>16/8	R	>16/8	R	>16	R*	>16	ESBL	>16	R*
S	>16/8	R	>16/8	R	>16	R	>16	R	>16	R
R	>16/8	R	>16/8	R	>16	R	>16	R	>16	R
S	>16/8	R	>16/8	R	>16	R*	>16	ESBL	>16	R*
I	>16/8	R	>16/8	R	>16	R	<=1	S	>16	R
S	>16/8	R	>16/8	R	>16	R	>16	R	>16	R
S	>16/8	R	>16/8	R	>16	R*	16	ESBL	8	R*
S	>16/8	R	>16/8	R	>16	R	8	I	16	I
S	>16/8	R	>16/8	R	>16	R	>16	R	>16	R
S	>16/8	R	>16/8	R	>16	R*	>16	ESBL	>16	R*
S	>16/8	R	>16/8	R	>16	R	8	I	16	I
S	>16/8	R	>16/8	R	>16	R*	>16	ESBL	>16	R*
R	>16/8	R	>16/8	R	>16	R	4	S	>16	R
S	>16/8	R	>16/8	R	>16	R	16	R	>16	R
S	16/8	I	>16/8	R	>16	R*	>16	ESBL	>16	R*
S	>16/8	R	>16/8	R	>16	R	>16	R	>16	R
S	>16/8	R	>16/8	R	>16	R*	>16	ESBL	>16	R*
R	>16/8	R	>16/8	R	>16	R	>16	R	>16	R
S	>16/8	R	>16/8	R	>16	R*	>16	ESBL	>16	R*
S	<=8/4	S	16/8	I	>16	R	<=1	S	<=1	S
R	>16/8	R	>16/8	R	>16	R	>16	R	>16	R
S	>16/8	R	>16/8	R	>16	R	<=1	S	<=1	S
S	>16/8	R	>16/8	R	>16	R	>16	R	>16	R
S	>16/8	R	>16/8	R	>16	R	>16	R	>16	R
S	>16/8	R	>16/8	R	>16	R*	>16	ESBL	>16	R*
S	>16/8	R	>16/8	R	>16	R	>16	R	>16	R
R	>16/8	R	>16/8	R	>16	R	>16	R	>16	R
S	>16/8	R	>16/8	R	>16	R*	>16	ESBL	>16	R*
S	>16/8	R	>16/8	R	>16	R*	>16	ESBL	>16	R*
S	>16/8	R	>16/8	R	>16	R*	>16	ESBL	>16	R*
S	>16/8	R	>16/8	R	>16	R	>16	R	>16	R
S	>16/8	R	>16/8	R	>16	R	<=1	S	>16	R
S	>16/8	R	>16/8	R	>16	R	<=1	S	4	S
S	>16/8	R	>16/8	R	>16	R*	>16	ESBL	>16	R*
S	>16/8	R	>16/8	R	>16	R*	>16	ESBL	>16	R*
S	<=8/4	S	<=8/4	S	<=8	R*	<=1	S	<=1	S

R	>16/8	R	>16/8	R	>16	R	>16	R	>16	R
R	>16/8	R	>16/8	R	>16	R	>16	R	>16	R
S	>16/8	R	>16/8	R	>16	R*	>16	ESBL	>16	R*
S	>16/8	R	>16/8	R	>16	R	>16	R	>16	R
S	>16/8	R	>16/8	R	>16	R*	>16	ESBL	>16	R*
S	<=8/4	S	16/8	I	>16	R*	>16	ESBL	>16	R*
S	>16/8	R	>16/8	R	>16	R	<=1	S	4	S
S	>16/8	R	>16/8	R	>16	R*	>16	ESBL	>16	R*
S	>16/8	R	>16/8	R	>16	R	>16	R	>16	R
R	>16/8	R	>16/8	R	>16	R	>16	R	16	I
R	>16/8	R	>16/8	R	>16	R	4	S	2	S
S	16/8	I	>16/8	R	>16	R	<=1	S	<=1	S
S	>16/8	R	>16/8	R	>16	R*	>16	ESBL	>16	R*
R	>16/8	R	>16/8	R	>16	R	>16	R	>16	R
S	>16/8	R	>16/8	R	>16	R*	>16	ESBL	>16	R*
S	>16/8	R	>16/8	R	>16	R*	>16	ESBL	>16	R*
S	>16/8	R	>16/8	R	>16	R*	>16	ESBL	>16	R*
S	16/8	I	>16/8	R	>16	R	>16	R	>16	R
R	>16/8	R	>16/8	R	>16	R	>16	R	>16	R

R	>16/8	R	>16/8	R	>16	R	>16	R	>16	R
R	>16/8	R	>16/8	R	>16	R	>16	R	16	I
S	>16/8	R	>16/8	R	>16	R	>16	R	>16	R
S	>16/8	R	>16/8	R	>16	R*	>16	ESBL	>16	R*
S	>16/8	R	>16/8	R	>16	R*	>16	ESBL	>16	R*
S	>16/8	R	>16/8	R	>16	R	<=1	S	16	I
R	>16/8	R	>16/8	R	>16	R	>16	R	>16	R
S	>16/8	R	>16/8	R	>16	R	>16	R	>16	R
S	>16/8	R	>16/8	R	>16	R*	>16	ESBL	16	R*
R	>16/8	R	>16/8	R	>16	R	>16	R	>16	R
R	>16/8	R	>16/8	R	>16	R	>16	R	>16	R
R	>16/8	R	>16/8	R	>16	R	>16	R	16	I
R	>16/8	R	16/8	I	>16	R	<=1	S	<=1	S
R	>16/8	R	>16/8	R	>16	R	>16	R	>16	R
S	<=8/4	S	<=8/4	S	>16	R*	<=1	R*	8	R*

R	>16/8	R	>16/8	R	>16	R	>16	R	>16	R
S	>16/8	R	>16/8	R	>16	R	>16	R	>16	R
S	>16/8	R	>16/8	R	>16	R	>16	R	>16	R
S	>16/8	R	>16/8	R	>16	R*	>16	ESBL	>16	R*
S	>16/8	R	>16/8	R	>16	R	<=1	S	<=1	S
S	>16/8	R	>16/8	R	>16	R	>16	R	>16	R
R	>16/8	R	>16/8	R	>16	R	>16	R	>16	R
S	>16/8	R	>16/8	R	>16	R*	>16	ESBL	>16	R*
R	>16/8	R	>16/8	R	>16	R	>16	R	>16	R
S	>16/8	R	>16/8	R	>16	R	>16	R	>16	R

S	>16/8	R	>16/8	R	>16	R	<=1	S	<=1	S

R	>16/8	R	>16/8	R	>16	R	>16	R	16	I
S	>16/8	R	>16/8	R	>16	R	>16	R	>16	R
S	>16/8	R	>16/8	R	>16	R	8	I	<=1	S
R	>16/8	R	>16/8	R	>16	R	>16	R	>16	R
S	>16/8	R	>16/8	R	>16	R	>16	R	>16	R
R	>16/8	R	>16/8	R	>16	R	>16	R	>16	R
S	>16/8	R	>16/8	R	>16	R*	>16	ESBL	8	R*
R	>16/8	R	>16/8	R	>16	R	>16	R	>16	R
S	>16/8	R	>16/8	R	>16	R*	>16	ESBL	>16	R*
R	>16/8	R	>16/8	R	>16	R	>16	R	>16	R
R	>16/8	R	>16/8	R	>16	R	>16	R	>16	R
S	>16/8	R	>16/8	R	>16	R	>16	R	>16	R
R	>16/8	R	>16/8	R	>16	R	8	I	>16	R
R	>16/8	R	>16/8	R	>16	R	>16	R	>16	R
R	>16/8	R	>16/8	R	>16	R	>16	R	>16	R
S	>16/8	R	>16/8	R	>16	R	>16	R	>16	R
S	>16/8	R	>16/8	R	>16	R*	>16	ESBL	>16	R*
R	>16/8	R	>16/8	R	>16	R	>16	R	>16	R
S	>16/8	R	16/8	I	>16	R	>16	R	16	I
S	>16/8	R	>16/8	R	>16	R	>16	R	>16	R
R	>16/8	R	>16/8	R	>16	R	>16	R	>16	R
S	>16/8	R	>16/8	R	>16	R	>16	R	>16	R

R	>16/8	R	>16/8	R	>16	R	<=1	S	<=1	S
R	>16/8	R	>16/8	R	>16	R	>16	R	>16	R
R	>16/8	R	>16/8	R	>16	R	>16	R	>16	R
S	>16/8	R	<=8/4	S	>16	R	<=1	S	<=1	S
R	>16/8	R	>16/8	R	>16	R	<=1	S	<=1	S
I	>16/8	R	>16/8	R	>16	R	>16	R	>16	R
R	>16/8	R	>16/8	R	>16	R	>16	R	>16	R
S	>16/8	R	>16/8	R	>16	R*	>16	ESBL	>16	R*
S	>16/8	R	>16/8	R	>16	R	<=1	S	<=1	S
R	>16/8	R	>16/8	R	>16	R	16	R	>16	R
R	>16/8	R	>16/8	R	>16	R	>16	R	>16	R

R	>16/8	R	>16/8	R	>16	R	>16	R	>16	R
R	>16/8	R	>16/8	R	>16	R	>16	R	>16	R
S	>16/8	R	>16/8	R	>16	R*	>16	ESBL	>16	R*
S	>16/8	R	>16/8	R	>16	R*	>16	ESBL	>16	R*
S	>16/8	R	>16/8	R	>16	R*	>16	ESBL	>16	R*
S	>16/8	R	>16/8	R	>16	R*	>16	ESBL	>16	R*
R	>16/8	R	>16/8	R	>16	R	>16	R	>16	R
S	>16/8	R	>16/8	R	>16	R*	>16	ESBL	>16	R*
S	>16/8	R	>16/8	R	>16	R	>16	R	>16	R
S	>16/8	R	>16/8	R	>16	R	>16	R	>16	R
R	>16/8	R	>16/8	R	>16	R	>16	R	>16	R

I	>16/8	R	>16/8	R	>16	R*	16	ESBL	>16	R*
R	>16/8	R	16/8	I	>16	R	16	R	<=1	S
R	16/8	I	16/8	I	>16	R*	>16	R*	>16	R*
S	>16/8	R	>16/8	R	>16	R	>16	R	>16	R
S	>16/8	R	>16/8	R	>16	R*	>16	ESBL	>16	R*
S	>16/8	R	>16/8	R	>16	R*	>16	ESBL	>16	R*
R	>16/8	R	>16/8	R	>16	R	>16	R	>16	R
R	>16/8	R	>16/8	R	>16	R	16	R	8	S
R	>16/8	R	>16/8	R	>16	R	>16	R	>16	R
S	>16/8	R	>16/8	R	>16	R	<=1	S	<=1	S
S	>16/8	R	>16/8	R	>16	R	>16	R	>16	R
R	>16/8	R	>16/8	R	>16	R	>16	R	>16	R
R	>16/8	R	>16/8	R	>16	R	>16	R	>16	R
R	>16/8	R	>16/8	R	>16	R	>16	R	>16	R
S	>16/8	R	>16/8	R	>16	R	<=1	S	<=1	S
S	>16/8	R	>16/8	R	>16	R*	>16	ESBL	>16	R*
S	>16/8	R	>16/8	R	>16	R	>16	R	>16	R
S	>16/8	R	>16/8	R	>16	R*	>16	ESBL	>16	R*
S	16/8	I	>16/8	R	>16	R*	>16	ESBL	>16	R*
S	>16/8	R	>16/8	R	>16	R*	>16	ESBL	>16	R*
S	>16/8	R	>16/8	R	>16	R*	>16	ESBL	>16	R*
R	>16/8	R	>16/8	R	>16	R	>16	R	>16	R
S	>16/8	R	>16/8	R	>16	R	>16	R	>16	R
S	>16/8	R	>16/8	R	>16	R	<=1	S	>16	R
R	>16/8	R	>16/8	R	>16	R	>16	R	>16	R
R	>16/8	R	>16/8	R	>16	R	>16	R	>16	R
R	>16/8	R	>16/8	R	>16	R	16	R	>16	R
R	>16/8	R	>16/8	R	>16	R	>16	R	>16	R
R	>16/8	R	>16/8	R	>16	R	>16	R	>16	R
S	>16/8	R	>16/8	R	>16	R*	>16	ESBL	>16	R*
S	>16/8	R	>16/8	R	>16	R	>16	R	16	I
R	>16/8	R	>16/8	R	>16	R	>16	R	4	S
S	>16/8	R	>16/8	R	>16	R	>16	R	>16	R
R	>16/8	R	>16/8	R	>16	R	<=1	S	<=1	S
R	>16/8	R	>16/8	R	>16	R	16	R	>16	R
S	>16/8	R	>16/8	R	>16	R	>16	R	>16	R
S	>16/8	R	>16/8	R	>16	R*	>16	ESBL	>16	R*
S	>16/8	R	>16/8	R	>16	R	>16	R	>16	R
S	>16/8	R	>16/8	R	>16	R*	>16	ESBL	>16	R*
S	>16/8	R	>16/8	R	>16	R	>16	R	>16	R
S	>16/8	R	>16/8	R	>16	R*	>16	ESBL	>16	R*
R	>16/8	R	>16/8	R	>16	R	>16	R	>16	R
R	>16/8	R	>16/8	R	>16	R	>16	R	>16	R
R	>16/8	R	>16/8	R	>16	R	>16	R	>16	R
R	>16/8	R	>16/8	R	>16	R	>16	R	>16	R
S	>16/8	R	>16/8	R	>16	R*	>16	ESBL	>16	R*
R	>16/8	R	>16/8	R	>16	R	>16	R	>16	R
R	>16/8	R	>16/8	R	>16	R	>16	R	>16	R
R	>16/8	R	>16/8	R	>16	R	>16	R	>16	R
R	>16/8	R	>16/8	R	>16	R	4	S	<=1	S
R	>16/8	R	>16/8	R	>16	R	>16	R	>16	R
S	>16/8	R	>16/8	R	>16	R*	>16	ESBL	>16	R*
S	>16/8	R	>16/8	R	>16	R	>16	R	>16	R
S	>16/8	R	>16/8	R	>16	R*	>16	ESBL	16	R*
S	>16/8	R	>16/8	R	>16	R	<=1	S	16	I

S	16/8	I	>16/8	R	>16	R*	>16	ESBL	>16	R*
S	>16/8	R	>16/8	R	>16	R*	>16	ESBL	>16	R*
S	>16/8	R	>16/8	R	>16	R*	>16	ESBL	>16	R*
R	>16/8	R	>16/8	R	>16	R	>16	R	>16	R
S	>16/8	R	>16/8	R	>16	R*	>16	ESBL	>16	R*
S	>16/8	R	>16/8	R	>16	R*	>16	ESBL	>16	R*
R	>16/8	R	>16/8	R	>16	R	>16	R	>16	R
R	>16/8	R	>16/8	R	>16	R	>16	R	>16	R
S	>16/8	R	>16/8	R	>16	R*	>16	ESBL	>16	R*
R	>16/8	R	>16/8	R	>16	R	>16	R	>16	R
S	>16/8	R	>16/8	R	>16	R	>16	R	>16	R
R	>16/8	R	>16/8	R	>16	R	>16	R	>16	R
S	>16/8	R	>16/8	R	>16	R*	>16	ESBL	>16	R*
R	>16/8	R	>16/8	R	>16	R	>16	R	>16	R
R	>16/8	R	>16/8	R	>16	R	>16	R	>16	R
R	>16/8	R	>16/8	R	>16	R	>16	R	>16	R
R	>16/8	R	>16/8	R	>16	R	>16	R	>16	R
R	>16/8	R	>16/8	R	>16	R	>16	R	8	S
R	>16/8	R	>16/8	R	>16	R	>16	R	>16	R
S	>16/8	R	>16/8	R	>16	R	>16	R	>16	R
R	>16/8	R	>16/8	R	>16	R	>16	R	>16	R
S	>16/8	R	>16/8	R	>16	R	<=1	S	<=1	S
S	>16/8	R	>16/8	R	>16	R	<=1	S	<=1	S
R	>16/8	R	>16/8	R	>16	R	>16	R	>16	R
S	>16/8	R	>16/8	R	>16	R	>16	R	>16	R
R	>16/8	R	>16/8	R	>16	R	>16	R	>16	R
R	>16/8	R	>16/8	R	>16	R	>16	R	16	I
R	>16/8	R	>16/8	R	>16	R	>16	R	>16	R
R	>16/8	R	>16/8	R	>16	R	8	I	<=1	S
R	>16/8	R	>16/8	R	>16	R	>16	R	>16	R
S	>16/8	R	>16/8	R	>16	R	>16	R	>16	R
R	>16/8	R	>16/8	R	>16	R	>16	R	>16	R
R	>16/8	R	>16/8	R	>16	R	>16	R	>16	R
S	>16/8	R	>16/8	R	>16	R*	>16	ESBL	>16	R*
S	>16/8	R	>16/8	R	>16	R*	>16	ESBL	>16	R*
R	>16/8	R	>16/8	R	>16	R	8	I	4	S
R	>16/8	R	>16/8	R	>16	R	>16	R	>16	R
I	>16/8	R	>16/8	R	>16	R	<=1	S	>16	R
R	>16/8	R	>16/8	R	>16	R	16	R	<=1	S
R	>16/8	R	>16/8	R	>16	R	4	S	4	S
R	>16/8	R	>16/8	R	>16	R	>16	R	>16	R
R	>16/8	R	>16/8	R	>16	R	>16	R	>16	R
R	>16/8	R	>16/8	R	>16	R	>16	R	>16	R
R	>16/8	R	>16/8	R	>16	R	>16	R	>16	R
R	>16/8	R	>16/8	R	>16	R	>16	R	>16	R
S	>16/8	R	>16/8	R	>16	R	<=1	S	<=1	S
R	>16/8	R	>16/8	R	>16	R	>16	R	>16	R
R	>16/8	R	>16/8	R	>16	R	4	S	2	S
S	>16/8	R	>16/8	R	>16	R	<=1	S	<=1	S
S	>16/8	R	>16/8	R	>16	R	>16	R	>16	R
R	>16/8	R	>16/8	R	>16	R	>16	R	>16	R
S	<=8/4	S	<=8/4	S	16	R*	<=1	S	<=1	S
R	>16/8	R	>16/8	R	>16	R	>16	R	>16	R

S	>16/8	R	>16/8	R	>16	R	>16	R	>16	R
R	>16/8	R	>16/8	R	>16	R	>16	R	>16	R
R	>16/8	R	>16/8	R	>16	R	16	R	8	S
R	>16/8	R	>16/8	R	>16	R	>16	R	>16	R
S	>16/8	R	>16/8	R	>16	R	4	S	<=1	S
S	>16/8	R	>16/8	R	>16	R*	>16	ESBL	>16	R*
R	>16/8	R	>16/8	R	>16	R	>16	R	>16	R
R	>16/8	R	>16/8	R	>16	R	16	R	<=1	S
S	>16/8	R	>16/8	R	>16	R	<=1	S	>16	R
S	>16/8	R	>16/8	R	>16	R*	>16	ESBL	>16	R*
I	>16/8	R	>16/8	R	>16	R*	>16	ESBL	16	R*
S	>16/8	R	>16/8	R	>16	R*	>16	ESBL	>16	R*
S	>16/8	R	>16/8	R	>16	R	>16	R	>16	R
S	>16/8	R	>16/8	R	>16	R*	4	ESBL	>16	R*
S	>16/8	R	>16/8	R	>16	R	>16	R	>16	R
S	>16/8	R	>16/8	R	>16	R	>16	R	8	S
S	>16/8	R	>16/8	R	>16	R	>16	R	>16	R
S	>16/8	R	>16/8	R	>16	R	>16	R	4	S
S	>16/8	R	>16/8	R	>16	R	<=1	S	4	S
S	>16/8	R	>16/8	R	>16	R	>16	R	>16	R
S	>16/8	R	>16/8	R	>16	R	>16	R	>16	R
R	>16/8	R	>16/8	R	>16	R	<=1	S	>16	R
S	>16/8	R	>16/8	R	>16	R	>16	R	>16	R
S	>16/8	R	>16/8	R	>16	R	>16	R	>16	R
S	>16/8	R	>16/8	R	>16	R*	>16	ESBL	>16	R*
R	>16/8	R	>16/8	R	>16	R	16	R	<=1	S
S	>16/8	R	>16/8	R	>16	R*	>16	ESBL	>16	R*
R	>16/8	R	>16/8	R	>16	R	>16	R	>16	R
S	>16/8	R	>16/8	R	>16	R*	>16	ESBL	>16	R*
S	>16/8	R	>16/8	R	>16	R	<=1	S	>16	R
S	>16/8	R	>16/8	R	>16	R*	>16	ESBL	>16	R*
R	>16/8	R	>16/8	R	>16	R	>16	R	>16	R
R	>16/8	R	>16/8	R	>16	R	<=1	S	>16	R
R	>16/8	R	>16/8	R	>16	R	4	S	4	S
R	>16/8	R	>16/8	R	>16	R	>16	R	>16	R
R	>16/8	R	>16/8	R	>16	R	8	I	<=1	S
R	>16/8	R	>16/8	R	>16	R	>16	R	>16	R
S	>16/8	R	>16/8	R	>16	R*	<=1	R*	>16	R*
R	>16/8	R	>16/8	R	>16	R	>16	R	>16	R
S	>16/8	R	>16/8	R	>16	R*	>16	ESBL	>16	R*
S	>16/8	R	>16/8	R	>16	R*	>16	ESBL	16	R*
R	>16/8	R	>16/8	R	>16	R	>16	R	>16	R
S	>16/8	R	>16/8	R	>16	R*	>16	ESBL	>16	R*
R	>16/8	R	>16/8	R	>16	R	>16	R	>16	R
R	>16/8	R	>16/8	R	>16	R	>16	R	>16	R
S	>16/8	R	>16/8	R	>16	R*	>16	ESBL	>16	R*
R	>16/8	R	>16/8	R	>16	R	>16	R	>16	R
R	>16/8	R	>16/8	R	>16	R	>16	R	>16	R
R	>16/8	R	>16/8	R	>16	R	>16	R	>16	R
R	>16/8	R	>16/8	R	>16	R	>16	R	>16	R
R	>16/8	R	>16/8	R	>16	R	>16	R	>16	R
S	>16/8	R	>16/8	R	>16	R	>16	R	>16	R

R	>16/8	R	16/8	I	>16	R	<=1	S	<=1	S

S	<=8/4	S	>16/8	R	>16	R*	>16	ESBL	>16	R*
I	>16/8	R	>16/8	R	>16	R*	>16	ESBL	>16	R*
S	>16/8	R	>16/8	R	>16	R*	>16	ESBL	>16	R*
S	16/8	I	>16/8	R	>16	R*	>16	ESBL	>16	R*
S	>16/8	R	>16/8	R	>16	R*	>16	ESBL	>16	R*
S	>16/8	R	>16/8	R	>16	R	>16	R	>16	R
S	>16/8	R	>16/8	R	>16	R	>16	ESBL	>16	R
S	>16/8	R	>16/8	R	>16	R*	>16	ESBL	>16	R*
S	>16/8	R	>16/8	R	>16	R*	>16	ESBL	>16	R*
S	>16/8	R	>16/8	R	>16	R	>16	R	>16	R
S	>16/8	R	>16/8	R	>16	R*	>16	ESBL	>16	R*
R	>16/8	R	>16/8	R	>16	R	>16	R	>16	R
R	>16/8	R	>16/8	R	>16	R	>16	R	>16	R
S	>16/8	R	>16/8	R	>16	R	>16	R	>16	R

S	>16/8	R	>16/8	R	>16	R*	>16	ESBL	>16	R*
R	>16/8	R	>16/8	R	>16	R	>16	R	>16	R
R	>16/8	R	>16/8	R	>16	R	>16	R	>16	R
R	>16/8	R	>16/8	R	>16	R	>16	R	>16	R
S	>16/8	R	>16/8	R	>16	R	>16	R	>16	R
R	>16/8	R	>16/8	R	>16	R	>16	R	>16	R
S	>16/8	R	>16/8	R	>16	R	<=1	S	<=1	S
R	>16/8	R	>16/8	R	>16	R	>16	R	>16	R
R	>16/8	R	>16/8	R	>16	R	>16	R	>16	R
R	>16/8	R	>16/8	R	>16	R	>16	R	>16	R
R	>16/8	R	>16/8	R	>16	R	>16	R	>16	R
S	>16/8	R	>16/8	R	>16	R*	>16	ESBL	>16	R*
R	>16/8	R	>16/8	R	>16	R	>16	R	>16	R
R	>16/8	R	>16/8	R	>16	R	>16	R	>16	R
R	>16/8	R	>16/8	R	>16	R	>16	R	>16	R
R	>16/8	R	>16/8	R	>16	R	>16	R	>16	R
S	>16/8	R	>16/8	R	>16	R*	>16	ESBL	>16	R*
R	>16/8	R	>16/8	R	>16	R	>16	R	>16	R
I	>16/8	R	>16/8	R	>16	R*	>16	ESBL	>16	R*
S	>16/8	R	>16/8	R	>16	R*	>16	ESBL	>16	R*
S	>16/8	R	>16/8	R	>16	R	<=1	S	4	S

R	>16/8	R	>16/8	R	>16	R	>16	R	>16	R
S	>16/8	R	>16/8	R	>16	R	>16	R	>16	R
S	>16/8	R	>16/8	R	>16	R*	>16	ESBL	>16	R*
R	>16/8	R	>16/8	R	>16	R	>16	R	>16	R
R	>16/8	R	>16/8	R	>16	R	>16	R	>16	R
R	>16/8	R	>16/8	R	>16	R	>16	R	>16	R
R	>16/8	R	>16/8	R	>16	R	>16	R	>16	R
S	>16/8	R	>16/8	R	>16	R	>16	R	>16	R
R	>16/8	R	>16/8	R	>16	R	>16	R	>16	R
S	>16/8	R	>16/8	R	>16	R	>16	R	>16	R
R	>16/8	R	>16/8	R	>16	R	>16	R	>16	R
S	>16/8	R	>16/8	R	>16	R*	>16	ESBL	>16	R*
S	>16/8	R	>16/8	R	>16	R	<=1	S	>16	R
S	>16/8	R	>16/8	R	>16	R*	>16	ESBL	>16	R*
R	>16/8	R	>16/8	R	>16	R	>16	R	>16	R
R	>16/8	R	>16/8	R	>16	R	>16	R	>16	R
R	>16/8	R	>16/8	R	>16	R	>16	R	>16	R
S	>16/8	R	>16/8	R	>16	R	>16	R	>16	R
R	>16/8	R	>16/8	R	>16	R	>16	R	>16	R
R	>16/8	R	>16/8	R	>16	R	16	R	>16	R
R	>16/8	R	>16/8	R	>16	R	>16	R	>16	R
S	>16/8	R	>16/8	R	>16	R	>16	R	>16	R
R	>16/8	R	>16/8	R	>16	R	>16	R	>16	R
R	>16/8	R	>16/8	R	>16	R	>16	R	>16	R
R	>16/8	R	>16/8	R	>16	R*	>16	ESBL	>16	R*
R	>16/8	R	>16/8	R	>16	R	>16	R	>16	R
S	>16/8	R	>16/8	R	>16	R*	>16	ESBL	>16	R*
R	>16/8	R	>16/8	R	>16	R	>16	R	>16	R
R	>16/8	R	>16/8	R	>16	R	>16	R	>16	R
S	>16/8	R	>16/8	R	>16	R	>16	R	>16	R
S	>16/8	R	>16/8	R	>16	R*	>16	ESBL	>16	R*
S	>16/8	R	>16/8	R	>16	R*	>16	ESBL	>16	R*
R	>16/8	R	>16/8	R	>16	R	>16	R	>16	R
R	>16/8	R	>16/8	R	>16	R	>16	R	>16	R
S	>16/8	R	>16/8	R	>16	R*	>16	ESBL	>16	R*
R	>16/8	R	>16/8	R	>16	R	>16	R	>16	R
S	>16/8	R	>16/8	R	>16	R*	>16	ESBL	>16	R*
S	>16/8	R	>16/8	R	>16	R*	>16	ESBL	>16	R*
S	>16/8	R	>16/8	R	>16	R	<=1	S	>16	R
S	>16/8	R	>16/8	R	>16	R	>16	R	>16	R
S	>16/8	R	>16/8	R	>16	R	<=1	S	<=1	S
R	>16/8	R	>16/8	R	>16	R	>16	R	>16	R
S	>16/8	R	>16/8	R	>16	R*	>16	ESBL	>16	R*
S	>16/8	R	>16/8	R	>16	R*	>16	ESBL	>16	R*
R	>16/8	R	>16/8	R	>16	R	>16	R	>16	R
S	>16/8	R	>16/8	R	>16	R*	>16	ESBL	>16	R*
S	>16/8	R	>16/8	R	>16	R*	>16	ESBL	>16	R*
R	>16/8	R	>16/8	R	>16	R	>16	R	>16	R
S	>16/8	R	>16/8	R	>16	R*	>16	ESBL	>16	R*
S	>16/8	R	>16/8	R	>16	R	>16	R	>16	R
S	>16/8	R	>16/8	R	>16	R*	>16	ESBL	>16	R*
S	>16/8	R	>16/8	R	>16	R	>16	R	>16	R
S	>16/8	R	>16/8	R	>16	R*	>16	ESBL	>16	R*
R	>16/8	R	>16/8	R	>16	R	>16	R	>16	R
S	>16/8	R	>16/8	R	>16	R	>16	R	>16	R

I	>16/8	R	>16/8	R	>16	R*	>16	ESBL	>16	R*
S	>16/8	R	>16/8	R	>16	R*	>16	ESBL	>16	R*

S	>16/8	R	>16/8	R	>16	R*	>16	ESBL	>16	R*
S	>16/8	R	>16/8	R	>16	R*	>16	ESBL	>16	R*

S	>16/8	R	>16/8	R	>16	R*	>16	ESBL	>16	R*
S	>16/8	R	>16/8	R	>16	R	>16	R	>16	R
R	>16/8	R	>16/8	R	>16	R	>16	R	>16	R
S	>16/8	R	>16/8	R	>16	R	>16	R	>16	R

S	>16/8	R	>16/8	R	>16	R	>16	R	>16	R
R	>16/8	R	>16/8	R	>16	R	>16	R	>16	R
S	>16/8	R	>16/8	R	>16	R	>16	R	>16	R
S	>16/8	R	>16/8	R	>16	R	>16	R	>16	R
S	>16/8	R	>16/8	R	>16	R*	>16	ESBL	>16	R*
R	>16/8	R	>16/8	R	>16	R	>16	R	>16	R
R	>16/8	R	>16/8	R	>16	R	>16	R	>16	R
S	>16/8	R	>16/8	R	>16	R	>16	R	>16	R
S	>16/8	R	>16/8	R	>16	R*	>16	ESBL	>16	R*
S	>16/8	R	>16/8	R	>16	R*	>16	ESBL	>16	R*
S	>16/8	R	>16/8	R	>16	R*	>16	ESBL	>16	R*
R	>16/8	R	>16/8	R	>16	R	>16	R	>16	R
S	>16/8	R	>16/8	R	>16	R*	>16	ESBL	>16	R*
S	>16/8	R	>16/8	R	>16	R*	>16	ESBL	>16	R*
S	>16/8	R	>16/8	R	>16	R*	>16	ESBL	>16	R*
S	16/8	I	>16/8	R	>16	R*	>16	ESBL	>16	R*
I	>16/8	R	>16/8	R	>16	R	>16	R	>16	R
S	>16/8	R	>16/8	R	>16	R*	>16	ESBL	16	R*
R	>16/8	R	>16/8	R	>16	R	>16	R	>16	R
R	>16/8	R	>16/8	R	>16	R	>16	R	8	S
S	>16/8	R	>16/8	R	>16	R	>16	R	>16	R
S	<=8/4	S	<=8/4	S	>16	R	<=1	S	<=1	S
S	>16/8	R	<=8/4	S	>16	R*	16	ESBL	<=1	R*
S	<=8/4	S	16/8	I	16	R*	<=1	R*	16	R*
S	>16/8	R	>16/8	R	>16	R*	>16	ESBL	>16	R*
R	>16/8	R	16/8	I	>16	R	8	I	<=1	S
S	>16/8	R	>16/8	R	>16	R	>16	R	>16	R
S	>16/8	R	>16/8	R	>16	R	<=1	S	<=1	S
S	>16/8	R	>16/8	R	>16	R*	>16	ESBL	>16	R*
S	>16/8	R	>16/8	R	>16	R	>16	R	>16	R
S	>16/8	R	>16/8	R	>16	R*	>16	ESBL	16	R*
R	>16/8	R	>16/8	R	>16	R	>16	R	>16	R
R	>16/8	R	>16/8	R	>16	R	>16	R	>16	R
S	>16/8	R	>16/8	R	>16	R*	>16	ESBL	>16	R*
R	>16/8	R	>16/8	R	>16	R	>16	R	>16	R
R	>16/8	R	>16/8	R	>16	R	>16	R	>16	R
R	>16/8	R	>16/8	R	>16	R	>16	R	>16	R
S	>16/8	R	>16/8	R	>16	R	>16	R	8	S
R	>16/8	R	>16/8	R	>16	R	>16	R	>16	R
S	>16/8	R	>16/8	R	>16	R*	>16	ESBL	>16	R*
S	>16/8	R	>16/8	R	>16	R*	>16	ESBL	>16	R*
S	>16/8	R	>16/8	R	>16	R	>16	R	>16	R
S	>16/8	R	>16/8	R	>16	R*	>16	ESBL	>16	R*
S	>16/8	R	>16/8	R	>16	R	>16	R	>16	R
S	>16/8	R	>16/8	R	>16	R	>16	R	8	S
S	>16/8	R	>16/8	R	>16	R	>16	R	>16	R
S	>16/8	R	>16/8	R	>16	R	<=1	S	<=1	S
S	>16/8	R	>16/8	R	>16	R	>16	R	>16	R
S	>16/8	R	>16/8	R	>16	R*	>16	ESBL	>16	R*
R	>16/8	R	>16/8	R	>16	R	>16	R	>16	R
R	>16/8	R	>16/8	R	>16	R	>16	R	>16	R

R	>16/8	R	>16/8	R	>16	R	>16	R	>16	R
R	>16/8	R	>16/8	R	>16	R	>16	R	>16	R
S	>16/8	R	>16/8	R	>16	R*	>16	ESBL	16	R*
S	>16/8	R	>16/8	R	>16	R*	>16	ESBL	>16	R*
S	16/8	I	>16/8	R	>16	R*	>16	ESBL	>16	R*

S	>16/8	R	>16/8	R	>16	R*	>16	ESBL	>16	R*
S	>16/8	R	>16/8	R	>16	R	>16	R	16	I
S	>16/8	R	>16/8	R	>16	R	>16	R	>16	R
S	>16/8	R	>16/8	R	>16	R*	>16	ESBL	16	R*
S	<=8/4	S	>16/8	R	>16	R	<=1	S	<=1	S
S	16/8	I	>16/8	R	>16	R	<=1	S	<=1	S
S	>16/8	R	>16/8	R	>16	R	>16	R	>16	R
S	>16/8	R	>16/8	R	>16	R*	>16	ESBL	>16	R*
S	>16/8	R	>16/8	R	>16	R*	>16	ESBL	>16	R*
S	>16/8	R	>16/8	R	>16	R*	>16	ESBL	>16	R*
R	>16/8	R	>16/8	R	>16	R	>16	R	>16	R
R	>16/8	R	>16/8	R	>16	R	>16	R	>16	R
R	>16/8	R	>16/8	R	>16	R	>16	R	>16	R
S	>16/8	R	>16/8	R	>16	R*	>16	ESBL	>16	R*
S	<=8/4	S	<=8/4	S	>16	R*	<=1	R*	16	R*
S	>16/8	R	>16/8	R	>16	R*	>16	ESBL	>16	R*
R	>16/8	R	>16/8	R	>16	R	>16	R	>16	R
S	>16/8	R	>16/8	R	>16	R*	>16	ESBL	>16	R*

I	>16/8	R	>16/8	R	>16	R	>16	R	>16	R
S	>16/8	R	>16/8	R	>16	R	<=1	S	>16	R
S	>16/8	R	>16/8	R	>16	R*	>16	ESBL	>16	R*
S	>16/8	R	>16/8	R	>16	R	>16	R	>16	R
S	>16/8	R	>16/8	R	>16	R	>16	R	>16	R
S	>16/8	R	>16/8	R	>16	R	>16	R	>16	R
S	>16/8	R	>16/8	R	>16	R*	>16	ESBL	>16	R*
R	>16/8	R	>16/8	R	>16	R	>16	R	>16	R
S	>16/8	R	>16/8	R	>16	R	>16	R	>16	R
S	>16/8	R	>16/8	R	>16	R	>16	R	>16	R
S	>16/8	R	>16/8	R	>16	R*	>16	ESBL	>16	R*
S	<=8/4	S	<=8/4	S	>16	R	<=1	S	<=1	S
R	>16/8	R	>16/8	R	>16	R	>16	R	>16	R
S	>16/8	R	>16/8	R	>16	R*	>16	ESBL	>16	R*
R	>16/8	R	>16/8	R	>16	R	>16	R	>16	R
R	>16/8	R	>16/8	R	>16	R	>16	R	>16	R
R	>16/8	R	>16/8	R	>16	R	>16	R	>16	R
R	>16/8	R	>16/8	R	>16	R	>16	R	>16	R
R	>16/8	R	>16/8	R	>16	R	>16	R	>16	R
R	>16/8	R	>16/8	R	>16	R	>16	R	>16	R
S	>16/8	R	>16/8	R	>16	R*	>16	ESBL	>16	R*
S	>16/8	R	>16/8	R	>16	R*	>16	ESBL	>16	R*

S	>16/8	R	>16/8	R	>16	R*	>16	ESBL	>16	R*
S	>16/8	R	>16/8	R	>16	R	>16	R	>16	R
S	>16/8	R	>16/8	R	>16	R*	>16	ESBL	8	R*
S	>16/8	R	>16/8	R	>16	R	<=1	S	<=1	S
S	>16/8	R	16/8	I	>16	R	>16	R	>16	R
S	>16/8	R	>16/8	R	>16	R	>16	R	>16	R
S	>16/8	R	>16/8	R	>16	R*	>16	ESBL	>16	R*
S	>16/8	R	>16/8	R	>16	R*	>16	ESBL	>16	R*
R	>16/8	R	>16/8	R	>16	R	>16	R	>16	R
S	>16/8	R	>16/8	R	>16	R*	>16	ESBL	>16	R*
S	>16/8	R	>16/8	R	>16	R	>16	R	>16	R
S	>16/8	R	>16/8	R	>16	R*	>16	ESBL	>16	R*
S	>16/8	R	>16/8	R	>16	R*	>16	ESBL	>16	R*
S	>16/8	R	>16/8	R	>16	R	>16	R	>16	R
S	>16/8	R	>16/8	R	>16	R*	>16	ESBL	>16	R*
S	>16/8	R	>16/8	R	>16	R*	>16	ESBL	>16	R*
R	>16/8	R	>16/8	R	>16	R	>16	R	>16	R
R	>16/8	R	>16/8	R	>16	R	>16	R	>16	R
S	>16/8	R	>16/8	R	>16	R	>16	R	>16	R
S	>16/8	R	>16/8	R	>16	R*	>16	ESBL	>16	R*
S	>16/8	R	>16/8	R	>16	R*	>16	ESBL	>16	R*
S	>16/8	R	>16/8	R	>16	R	>16	R	>16	R
S	>16/8	R	>16/8	R	>16	R	>16	R	>16	R
S	>16/8	R	>16/8	R	>16	R	>16	R	>16	R
S	>16/8	R	>16/8	R	>16	R	<=1	S	16	I
S	>16/8	R	>16/8	R	>16	R	>16	R	>16	R
S	>16/8	R	>16/8	R	>16	R	>16	R	>16	R
S	>16/8	R	>16/8	R	>16	R	>16	R	>16	R
I	>16/8	R	>16/8	R	>16	R	>16	R	>16	R
S	>16/8	R	>16/8	R	>16	R*	>16	ESBL	>16	R*
S	>16/8	R	>16/8	R	>16	R*	>16	ESBL	>16	R*
S	>16/8	R	>16/8	R	>16	R	>16	R	>16	R
S	>16/8	R	>16/8	R	>16	R	>16	R	>16	R

S	>16/8	R	>16/8	R	>16	R	<=1	S	8	S
S	>16/8	R	>16/8	R	>16	R	>16	R	>16	R
R	>16/8	R	>16/8	R	>16	R	<=1	S	>16	R
S	>16/8	R	>16/8	R	>16	R	8	I	>16	R

S	>16/8	R	>16/8	R	>16	R*	>16	ESBL	>16	R*
S	>16/8	R	>16/8	R	>16	R	>16	R	>16	R
S	>16/8	R	>16/8	R	>16	R*	>16	ESBL	>16	R*
S	>16/8	R	>16/8	R	>16	R*	>16	ESBL	>16	R*
R	>16/8	R	>16/8	R	>16	R	>16	R	>16	R
S	<=8/4	S	<=8/4	S	>16	R*	>16	ESBL	>16	R*
R	>16/8	R	>16/8	R	>16	R	16	R	<=1	S

S	>16/8	R	>16/8	R	>16	R*	>16	ESBL	>16	R*
S	>16/8	R	>16/8	R	>16	R	>16	R	>16	R
R	>16/8	R	>16/8	R	>16	R	>16	R	>16	R
S	>16/8	R	>16/8	R	>16	R*	>16	ESBL	>16	R*
S	>16/8	R	16/8	I	>16	R	<=1	S	<=1	S
S	>16/8	R	>16/8	R	>16	R*	>16	ESBL	>16	R*
S	>16/8	R	>16/8	R	>16	R*	>16	ESBL	>16	R*
S	>16/8	R	>16/8	R	>16	R*	>16	ESBL	>16	R*
S	>16/8	R	16/8	I	>16	R	<=1	S	<=1	S
S	>16/8	R	>16/8	R	>16	R*	>16	ESBL	>16	R*
S	>16/8	R	>16/8	R	>16	R	>16	R	>16	R
R	>16/8	R	>16/8	R	>16	R	4	S	4	S
S	>16/8	R	>16/8	R	>16	R*	>16	ESBL	>16	R*
R	>16/8	R	>16/8	R	>16	R	16	R	>16	R
R	>16/8	R	>16/8	R	>16	R	>16	R	>16	R
S	>16/8	R	>16/8	R	>16	R*	>16	ESBL	>16	R*
R	>16/8	R	>16/8	R	>16	R	>16	R	>16	R
S	>16/8	R	>16/8	R	>16	R*	>16	ESBL	>16	R*
S	>16/8	R	>16/8	R	>16	R	<=1	S	>16	R
S	>16/8	R	>16/8	R	>16	R	>16	R	>16	R
S	>16/8	R	>16/8	R	>16	R	>16	R	>16	R
S	>16/8	R	>16/8	R	>16	R*	>16	ESBL	>16	R*
S	>16/8	R	>16/8	R	>16	R*	>16	ESBL	>16	R*
S	>16/8	R	>16/8	R	>16	R*	>16	ESBL	>16	R*
R	>16/8	R	>16/8	R	>16	R*	<=1	R*	<=1	R*
S	>16/8	R	>16/8	R	>16	R*	>16	ESBL	>16	R*
S	>16/8	R	>16/8	R	>16	R*	>16	ESBL	>16	R*
S	>16/8	R	>16/8	R	>16	R	>16	R	>16	R
S	>16/8	R	>16/8	R	>16	R	>16	R	>16	R
S	>16/8	R	>16/8	R	>16	R	<=1	S	<=1	S
S	>16/8	R	>16/8	R	>16	R	>16	R	>16	R
S	>16/8	R	>16/8	R	>16	R	>16	R	>16	R
S	>16/8	R	>16/8	R	>16	R*	>16	ESBL	>16	R*
R	>16/8	R	>16/8	R	>16	R	8	I	<=1	S
S	>16/8	R	>16/8	R	>16	R*	>16	ESBL	>16	R*
R	>16/8	R	>16/8	R	>16	R	>16	R	>16	R
S	>16/8	R	>16/8	R	>16	R*	>16	ESBL	>16	R*
S	>16/8	R	>16/8	R	>16	R*	>16	ESBL	>16	R*
S	>16/8	R	>16/8	R	>16	R	<=1	S	>16	R
S	>16/8	R	>16/8	R	>16	R	>16	R	>16	R
R	>16/8	R	>16/8	R	>16	R	>16	R	>16	R
R	>16/8	R	>16/8	R	>16	R	>16	R	>16	R
S	>16/8	R	>16/8	R	>16	R*	>16	ESBL	>16	R*
S	16/8	I	>16/8	R	>16	R*	>16	ESBL	>16	R*
R	>16/8	R	>16/8	R	>16	R	<=1	S	<=1	S
S	<=8/4	S	>16/8	R	>16	R*	>16	ESBL	>16	R*
R	>16/8	R	>16/8	R	>16	R	16	R	<=1	S
I	>16/8	R	>16/8	R	>16	R	>16	R	>16	R
S	>16/8	R	>16/8	R	>16	R	>16	R	>16	R
S	>16/8	R	>16/8	R	>16	R*	>16	ESBL	>16	R*
S	>16/8	R	>16/8	R	>16	R*	>16	ESBL	>16	R*
R	>16/8	R	>16/8	R	>16	R	>16	R	>16	R
S	>16/8	R	>16/8	R	>16	R*	>16	ESBL	>16	R*
I	>16/8	R	16/8	I	>16	R	>16	R	16	I
R	>16/8	R	>16/8	R	>16	R	>16	R	>16	R
I	>16/8	R	>16/8	R	>16	R	>16	R	>16	R

S	16/8	I	<=8/4	S	<=8	S	4	S	<=1	S
S	>16/8	R	>16/8	R	>16	R	>16	R	>16	R
S	>16/8	R	>16/8	R	>16	R	>16	R	>16	R
S	>16/8	R	>16/8	R	>16	R	>16	R	>16	R
R	>16/8	R	>16/8	R	>16	R	>16	R	>16	R
S	<=8/4	S	16/8	I	>16	R*	>16	ESBL	>16	R*
R	>16/8	R	>16/8	R	>16	R	>16	R	>16	R
S	>16/8	R	>16/8	R	>16	R*	>16	ESBL	>16	R*
S	>16/8	R	>16/8	R	>16	R	>16	R	>16	R
S	>16/8	R	>16/8	R	>16	R	>16	R	>16	R
S	>16/8	R	>16/8	R	>16	R*	>16	ESBL	>16	R*
S	>16/8	R	>16/8	R	>16	R*	>16	ESBL	>16	R*
S	16/8	I	>16/8	R	>16	R	<=1	S	<=1	S
S	>16/8	R	>16/8	R	>16	R*	>16	ESBL	>16	R*
S	>16/8	R	>16/8	R	>16	R*	>16	ESBL	>16	R*
R	>16/8	R	>16/8	R	>16	R	8	I	<=1	S
S	>16/8	R	>16/8	R	>16	R	>16	R	>16	R
S	>16/8	R	>16/8	R	>16	R	>16	R	>16	R
S	>16/8	R	>16/8	R	>16	R	>16	R	>16	R
S	>16/8	R	>16/8	R	>16	R	>16	R	>16	R
S	>16/8	R	>16/8	R	>16	R*	>16	ESBL	>16	R*
S	>16/8	R	>16/8	R	>16	R	>16	R	>16	R
S	>16/8	R	>16/8	R	>16	R	16	R	>16	R
R	>16/8	R	>16/8	R	>16	R	>16	R	>16	R
S	>16/8	R	>16/8	R	>16	R	>16	R	>16	R
R	>16/8	R	>16/8	R	>16	R	16	R	>16	R
R	>16/8	R	>16/8	R	>16	R	>16	R	>16	R
S	>16/8	R	>16/8	R	>16	R*	>16	ESBL	>16	R*
S	>16/8	R	>16/8	R	>16	R*	>16	ESBL	>16	R*
S	>16/8	R	>16/8	R	>16	R*	>16	ESBL	>16	R*
R	>16/8	R	>16/8	R	>16	R	>16	R	>16	R
S	>16/8	R	>16/8	R	>16	R*	>16	ESBL	>16	R*
S	>16/8	R	>16/8	R	>16	R	>16	R	>16	R
R	>16/8	R	>16/8	R	>16	R	>16	R	>16	R
S	>16/8	R	>16/8	R	>16	R	>16	R	>16	R
S	>16/8	R	>16/8	R	>16	R*	>16	ESBL	>16	R*
S	>16/8	R	>16/8	R	>16	R	>16	R	>16	R
S	>16/8	R	>16/8	R	>16	R	>16	R	>16	R
R	>16/8	R	>16/8	R	>16	R	<=1	S	>16	R
S	>16/8	R	>16/8	R	>16	R*	>16	ESBL	>16	R*
S	>16/8	R	>16/8	R	>16	R	>16	R	>16	R
S	>16/8	R	>16/8	R	>16	R*	>16	ESBL	>16	R*
S	>16/8	R	>16/8	R	>16	R	>16	R	>16	R
R	>16/8	R	>16/8	R	>16	R	>16	R	>16	R
R	>16/8	R	>16/8	R	>16	R	>16	R	>16	R
R	>16/8	R	>16/8	R	>16	R	>16	R	>16	R
R	>16/8	R	>16/8	R	>16	R	>16	R	>16	R
R	>16/8	R	>16/8	R	>16	R	>16	R	>16	R
S	>16/8	R	>16/8	R	>16	R	>16	R	>16	R
S	>16/8	R	>16/8	R	>16	R*	>16	ESBL	>16	R*
R	>16/8	R	>16/8	R	>16	R	>16	R	>16	R
S	>16/8	R	>16/8	R	>16	R	>16	R	>16	R
S	>16/8	R	>16/8	R	>16	R*	>16	ESBL	>16	R*
R	>16/8	R	>16/8	R	>16	R	>16	R	>16	R
S	>16/8	R	>16/8	R	>16	R	>16	R	>16	R

R	>16/8	R	>16/8	R	>16	R	>16	R	>16	R
S	>16/8	R	>16/8	R	>16	R	>16	R	>16	R
S	>16/8	R	>16/8	R	>16	R*	>16	ESBL	>16	R*
R	>16/8	R	>16/8	R	>16	R	>16	R	>16	R
S	>16/8	R	>16/8	R	>16	R	>16	R	>16	R
S	>16/8	R	<=8/4	S	>16	R	<=1	S	<=1	S
S	>16/8	R	>16/8	R	>16	R*	>16	ESBL	>16	R*
S	>16/8	R	>16/8	R	>16	R	>16	R	>16	R
S	>16/8	R	>16/8	R	>16	R	<=1	S	<=1	S
S	>16/8	R	>16/8	R	>16	R	>16	R	>16	R
S	>16/8	R	>16/8	R	>16	R*	>16	ESBL	>16	R*
R	>16/8	R	>16/8	R	>16	R	>16	R	>16	R
S	16/8	I	>16/8	R	>16	R*	>16	ESBL	>16	R*
S	>16/8	R	>16/8	R	>16	R	>16	R	>16	R
S	>16/8	R	>16/8	R	>16	R*	>16	ESBL	>16	R*
S	>16/8	R	>16/8	R	>16	R*	>16	ESBL	>16	R*
S	>16/8	R	>16/8	R	>16	R	>16	R	>16	R
S	>16/8	R	>16/8	R	>16	R	>16	R	>16	R
S	>16/8	R	>16/8	R	>16	R*	>16	ESBL	>16	R*
S	>16/8	R	>16/8	R	>16	R*	>16	ESBL	>16	R*
I	>16/8	R	<=8/4	S	>16	R	4	S	<=1	S
R	>16/8	R	>16/8	R	>16	R	<=1	S	<=1	S
S	>16/8	R	>16/8	R	>16	R	>16	R	>16	R
S	>16/8	R	>16/8	R	>16	R	>16	R	>16	R
S	<=8/4	S	<=8/4	S	>16	R	<=1	S	<=1	S
S	>16/8	R	16/8	I	>16	R	<=1	S	<=1	S
S	<=8/4	S	<=8/4	S	>16	R*	>16	ESBL	>16	R*
S	>16/8	R	>16/8	R	>16	R*	>16	ESBL	>16	R*
S	>16/8	R	>16/8	R	>16	R*	>16	ESBL	>16	R*
S	>16/8	R	>16/8	R	>16	R*	>16	ESBL	>16	R*
S	>16/8	R	>16/8	R	>16	R*	>16	ESBL	>16	R*
S	>16/8	R	>16/8	R	>16	R*	>16	ESBL	>16	R*
S	>16/8	R	>16/8	R	>16	R*	>16	ESBL	>16	R*
S	>16/8	R	>16/8	R	>16	R	<=1	S	2	S
R	>16/8	R	>16/8	R	>16	R	>16	R	>16	R
S	>16/8	R	>16/8	R	>16	R	<=1	S	16	I
S	>16/8	R	>16/8	R	>16	R*	>16	ESBL	>16	R*
S	>16/8	R	>16/8	R	>16	R*	>16	ESBL	>16	R*
S	>16/8	R	>16/8	R	>16	R*	>16	ESBL	>16	R*
S	>16/8	R	>16/8	R	>16	R*	>16	ESBL	>16	R*
S	>16/8	R	>16/8	R	>16	R*	>16	ESBL	>16	R*
S	>16/8	R	>16/8	R	>16	R	>16	R	>16	R
S	>16/8	R	>16/8	R	>16	R	<=1	S	16	I
S	>16/8	R	>16/8	R	>16	R	<=1	S	<=1	S
R	>16/8	R	>16/8	R	>16	R	>16	R	>16	R
S	>16/8	R	>16/8	R	>16	R*	>16	ESBL	>16	R*
S	>16/8	R	>16/8	R	>16	R*	>16	ESBL	>16	R*
S	>16/8	R	>16/8	R	>16	R*	>16	ESBL	>16	R*
S	>16/8	R	>16/8	R	>16	R	<=1	S	<=1	S
S	>16/8	R	>16/8	R	>16	R	>16	R	>16	R
S	>16/8	R	>16/8	R	>16	R*	>16	ESBL	>16	R*
S	>16/8	R	>16/8	R	>16	R	>16	R	>16	R
S	>16/8	R	>16/8	R	>16	R*	>16	ESBL	>16	R*
S	>16/8	R	>16/8	R	>16	R*	>16	ESBL	>16	R*
S	>16/8	R	>16/8	R	>16	R	<=1	S	4	S
R	>16/8	R	>16/8	R	>16	R	<=1	S	16	I

S	>16/8	R	>16/8	R	>16	R*	>16	ESBL	>16	R*
S	>16/8	R	>16/8	R	>16	R*	>16	ESBL	>16	R*
S	>16/8	R	>16/8	R	>16	R	<=1	S	4	S
S	>16/8	R	>16/8	R	>16	R*	>16	ESBL	>16	R*
S	>16/8	R	>16/8	R	>16	R*	>16	ESBL	>16	R*
S	>16/8	R	>16/8	R	>16	R	>16	R	>16	R
S	>16/8	R	>16/8	R	>16	R	>16	R	>16	R
S	>16/8	R	>16/8	R	>16	R*	>16	ESBL	>16	R*
R	>16/8	R	>16/8	R	>16	R	8	I	<=1	S
R	>16/8	R	>16/8	R	>16	R*	>16	ESBL	8	R*
S	>16/8	R	>16/8	R	>16	R*	>16	ESBL	>16	R*
S	>16/8	R	>16/8	R	>16	R*	>16	ESBL	>16	R*
S	>16/8	R	>16/8	R	>16	R*	>16	ESBL	>16	R*
R	>16/8	R	>16/8	R	>16	R	>16	R	>16	R
S	>16/8	R	>16/8	R	>16	R*	>16	ESBL	>16	R*
R	>16/8	R	>16/8	R	>16	R	8	I	>16	R
R	>16/8	R	>16/8	R	>16	R	>16	R	>16	R
R	>16/8	R	>16/8	R	>16	R	>16	R	>16	R
S	>16/8	R	>16/8	R	>16	R	<=1	S	<=1	S
R	>16/8	R	>16/8	R	>16	R	>16	R	>16	R
R	>16/8	R	>16/8	R	>16	R	>16	R	>16	R
R	>16/8	R	>16/8	R	>16	R	>16	R	>16	R
S	>16/8	R	>16/8	R	>16	R	>16	R	>16	R
S	>16/8	R	>16/8	R	>16	R*	>16	ESBL	>16	R*
S	>16/8	R	>16/8	R	>16	R	>16	R	>16	R
S	16/8	I	>16/8	R	>16	R*	>16	ESBL	>16	R*
S	>16/8	R	>16/8	R	>16	R*	>16	ESBL	>16	R*
S	>16/8	R	>16/8	R	>16	R	>16	R	>16	R
S	>16/8	R	>16/8	R	>16	R*	>16	ESBL	>16	R*
S	>16/8	R	>16/8	R	>16	R	>16	R	>16	R
S	>16/8	R	>16/8	R	>16	R*	>16	ESBL	16	R*
R	>16/8	R	>16/8	R	>16	R	>16	R	>16	R
S	>16/8	R	>16/8	R	>16	R*	>16	ESBL	>16	R*
S	>16/8	R	>16/8	R	>16	R*	>16	ESBL	>16	R*
S	>16/8	R	>16/8	R	>16	R	<=1	S	>16	R
S	>16/8	R	>16/8	R	>16	R*	>16	ESBL	>16	R*
S	>16/8	R	>16/8	R	>16	R*	>16	ESBL	>16	R*
S	>16/8	R	>16/8	R	>16	R	>16	R	>16	R
S	>16/8	R	>16/8	R	>16	R	>16	R	>16	R
S	>16/8	R	>16/8	R	>16	R*	>16	ESBL	>16	R*
S	>16/8	R	>16/8	R	>16	R*	>16	ESBL	>16	R*
S	>16/8	R	>16/8	R	>16	R*	>16	ESBL	>16	R*
S	>16/8	R	>16/8	R	>16	R*	>16	ESBL	>16	R*
S	>16/8	R	>16/8	R	>16	R*	>16	ESBL	>16	R*
R	>16/8	R	>16/8	R	>16	R	>16	R	>16	R
S	>16/8	R	>16/8	R	>16	R*	>16	ESBL	>16	R*
S	>16/8	R	>16/8	R	>16	R	>16	R	>16	R
S	>16/8	R	>16/8	R	>16	R	>16	R	>16	R
S	>16/8	R	>16/8	R	>16	R	>16	R	>16	R
S	>16/8	R	>16/8	R	>16	R*	>16	ESBL	>16	R*
S	>16/8	R	>16/8	R	>16	R*	>16	ESBL	>16	R*
S	>16/8	R	>16/8	R	>16	R*	>16	ESBL	>16	R*
S	>16/8	R	>16/8	R	>16	R*	>16	ESBL	>16	R*
S	>16/8	R	>16/8	R	>16	R*	>16	ESBL	>16	R*
S	>16/8	R	>16/8	R	>16	R*	>16	ESBL	>16	R*
R	>16/8	R	>16/8	R	>16	R	16	R	>16	R

S	>16/8	R	>16/8	R	>16	R*	>16	ESBL	>16	R*
R	>16/8	R	>16/8	R	>16	R	>16	R	>16	R
S	>16/8	R	>16/8	R	>16	R*	>16	ESBL	>16	R*
S	>16/8	R	>16/8	R	>16	R*	>16	ESBL	>16	R*
S	>16/8	R	>16/8	R	>16	R*	>16	ESBL	<=1	R*
S	>16/8	R	>16/8	R	>16	R	<=1	S	<=1	S
R	>16/8	R	>16/8	R	>16	R	<=1	S	>16	R
R	>16/8	R	>16/8	R	>16	R	>16	R	>16	R
S	>16/8	R	>16/8	R	>16	R*	>16	ESBL	>16	R*
S	>16/8	R	>16/8	R	>16	R*	>16	ESBL	>16	R*
S	>16/8	R	>16/8	R	>16	R*	>16	ESBL	>16	R*
S	>16/8	R	>16/8	R	>16	R	>16	R	>16	R
S	>16/8	R	>16/8	R	>16	R	>16	R	>16	R
S	>16/8	R	>16/8	R	>16	R	>16	R	>16	R
S	>16/8	R	>16/8	R	>16	R	<=1	S	8	S
S	>16/8	R	>16/8	R	>16	R	>16	R	2	S
R	>16/8	R	>16/8	R	>16	R	<=1	S	8	S
R	>16/8	R	>16/8	R	>16	R	>16	R	16	I
S	>16/8	R	>16/8	R	>16	R*	>16	ESBL	>16	R*
R	>16/8	R	>16/8	R	>16	R	4	S	<=1	S
S	>16/8	R	>16/8	R	>16	R*	>16	ESBL	16	R*
S	>16/8	R	>16/8	R	>16	R*	>16	ESBL	16	R*
R	>16/8	R	>16/8	R	>16	R	>16	R	>16	R
S	>16/8	R	>16/8	R	>16	R*	>16	ESBL	16	R*
S	>16/8	R	>16/8	R	>16	R*	>16	ESBL	>16	R*
S	>16/8	R	>16/8	R	>16	R*	>16	ESBL	>16	R*
S	>16/8	R	>16/8	R	>16	R*	>16	ESBL	>16	R*
S	>16/8	R	>16/8	R	>16	R	>16	R	>16	R
S	>16/8	R	>16/8	R	>16	R	>16	R	>16	R
S	>16/8	R	>16/8	R	>16	R*	>16	ESBL	>16	R*
S	>16/8	R	>16/8	R	>16	R	<=1	S	<=1	S
S	>16/8	R	>16/8	R	>16	R*	>16	ESBL	>16	R*
S	>16/8	R	>16/8	R	>16	R*	>16	ESBL	>16	R*
S	>16/8	R	>16/8	R	>16	R*	>16	ESBL	>16	R*
S	>16/8	R	>16/8	R	>16	R	>16	R	>16	R
S	>16/8	R	>16/8	R	>16	R	<=1	S	4	S
S	<=8/4	S	<=8/4	S	16	I	<=1	S	<=1	S
S	>16/8	R	>16/8	R	>16	R	>16	R	>16	R
S	>16/8	R	>16/8	R	>16	R	16	R	16	I
S	>16/8	R	>16/8	R	>16	R*	>16	ESBL	>16	R*
S	>16/8	R	>16/8	R	>16	R*	>16	ESBL	>16	R*
S	>16/8	R	>16/8	R	>16	R*	>16	ESBL	>16	R*
S	>16/8	R	>16/8	R	>16	R*	>16	ESBL	>16	R*
R	>16/8	R	>16/8	R	>16	R	>16	R	>16	R
S	>16/8	R	>16/8	R	>16	R	>16	R	>16	R
R	>16/8	R	>16/8	R	>16	R	>16	R	>16	R
S	>16/8	R	>16/8	R	>16	R	>16	R	>16	R
R	>16/8	R	>16/8	R	>16	R	>16	R	>16	R
S	>16/8	R	>16/8	R	>16	R*	>16	ESBL	>16	R*
R	>16/8	R	>16/8	R	>16	R	4	S	<=1	S
S	>16/8	R	>16/8	R	>16	R	16	R	4	S
S	>16/8	R	>16/8	R	>16	R	>16	R	>16	R
S	>16/8	R	>16/8	R	>16	R*	8	ESBL	8	R*
S	16/8	I	>16/8	R	>16	R	<=1	S	<=1	S
S	<=8/4	S	>16/8	R	>16	R	<=1	S	<=1	S

S	<=8/4	S	>16/8	R	>16	R*	>16	ESBL	>16	R*
S	>16/8	R	>16/8	R	>16	R	<=1	S	2	S
S	<=8/4	S	<=8/4	S	>16	R	<=1	S	<=1	S
S	>16/8	R	>16/8	R	>16	R	<=1	S	4	S
R	>16/8	R	>16/8	R	>16	R	>16	R	>16	R
S	>16/8	R	>16/8	R	>16	R	>16	R	>16	R
S	>16/8	R	>16/8	R	>16	R*	>16	ESBL	>16	R*
S	>16/8	R	>16/8	R	>16	R*	>16	ESBL	>16	R*
S	>16/8	R	>16/8	R	>16	R	>16	R	>16	R
R	>16/8	R	>16/8	R	>16	R	>16	R	>16	R
R	>16/8	R	>16/8	R	>16	R	>16	R	>16	R
R	>16/8	R	>16/8	R	>16	R	>16	R	>16	R
R	>16/8	R	>16/8	R	>16	R	>16	R	>16	R
R	>16/8	R	>16/8	R	>16	R	>16	R	>16	R
S	>16/8	R	>16/8	R	>16	R*	>16	ESBL	>16	R*
S	>16/8	R	>16/8	R	>16	R*	>16	ESBL	>16	R*
S	>16/8	R	>16/8	R	>16	R	8	I	<=1	S
R	>16/8	R	>16/8	R	>16	R	>16	R	>16	R
R	>16/8	R	>16/8	R	>16	R	>16	R	>16	R
S	>16/8	R	>16/8	R	>16	R	>16	R	16	I
R	>16/8	R	>16/8	R	>16	R*	16	ESBL	>16	R*
S	>16/8	R	>16/8	R	>16	R*	>16	ESBL	>16	R*
R	>16/8	R	>16/8	R	>16	R	>16	R	>16	R
R	>16/8	R	>16/8	R	>16	R	>16	R	>16	R
R	>16/8	R	>16/8	R	>16	R	>16	R	>16	R
R	>16/8	R	>16/8	R	>16	R	>16	R	>16	R
R	>16/8	R	>16/8	R	>16	R*	>16	ESBL	>16	R*
R	>16/8	R	>16/8	R	>16	R	>16	R	>16	R
S	>16/8	R	>16/8	R	>16	R	>16	R	>16	R
S	>16/8	R	>16/8	R	>16	R	>16	R	>16	R
S	>16/8	R	>16/8	R	>16	R*	>16	ESBL	>16	R*
S	>16/8	R	>16/8	R	>16	R	>16	R	>16	R
S	>16/8	R	>16/8	R	>16	R	>16	R	>16	R
S	>16/8	R	>16/8	R	>16	R*	>16	ESBL	>16	R*
S	>16/8	R	>16/8	R	>16	R	<=1	S	2	S
S	>16/8	R	>16/8	R	>16	R	>16	R	8	S
S	>16/8	R	>16/8	R	>16	R*	>16	ESBL	>16	R*
S	>16/8	R	>16/8	R	>16	R	>16	R	>16	R
S	>16/8	R	>16/8	R	>16	R*	>16	ESBL	>16	R*
S	>16/8	R	>16/8	R	>16	R*	>16	ESBL	>16	R*
S	>16/8	R	>16/8	R	>16	R*	>16	ESBL	>16	R*
S	>16/8	R	>16/8	R	>16	R	>16	R	>16	R
S	>16/8	R	>16/8	R	>16	R*	>16	ESBL	>16	R*
S	>16/8	R	>16/8	R	>16	R*	>16	ESBL	>16	R*
S	>16/8	R	>16/8	R	>16	R*	>16	ESBL	>16	R*
S	>16/8	R	>16/8	R	>16	R*	4	ESBL	16	R*
S	>16/8	R	>16/8	R	>16	R*	>16	ESBL	>16	R*
S	<=8/4	S	>16/8	R	>16	R	<=1	S	<=1	S
S	>16/8	R	>16/8	R	>16	R*	>16	ESBL	>16	R*
S	>16/8	R	>16/8	R	>16	R*	8	ESBL	4	R*
S	>16/8	R	>16/8	R	>16	R*	>16	ESBL	>16	R*
S	>16/8	R	>16/8	R	>16	R*	>16	ESBL	>16	R*
S	>16/8	R	>16/8	R	>16	R	>16	R	>16	R
S	>16/8	R	>16/8	R	>16	R*	>16	ESBL	>16	R*
S	>16/8	R	>16/8	R	>16	R	<=1	S	8	S
S	>16/8	R	>16/8	R	>16	R*	>16	ESBL	>16	R*

S	>16/8	R	>16/8	R	>16	R	>16	R	>16	R
S	>16/8	R	>16/8	R	>16	R	>16	R	>16	R
R	>16/8	R	>16/8	R	>16	R	>16	R	>16	R
S	>16/8	R	>16/8	R	>16	R	>16	R	>16	R
S	>16/8	R	>16/8	R	>16	R	>16	R	>16	R
S	>16/8	R	>16/8	R	>16	R	<=1	S	<=1	S
S	16/8	I	>16/8	R	>16	R	4	S	>16	R
R	>16/8	R	>16/8	R	>16	R	16	R	>16	R
S	>16/8	R	>16/8	R	>16	R*	>16	ESBL	>16	R*
R	>16/8	R	>16/8	R	>16	R	>16	R	>16	R
S	>16/8	R	>16/8	R	>16	R	>16	R	>16	R
S	>16/8	R	>16/8	R	>16	R	>16	R	>16	R
S	>16/8	R	>16/8	R	>16	R	>16	R	>16	R
S	>16/8	R	>16/8	R	>16	R*	>16	ESBL	>16	R*
S	>16/8	R	>16/8	R	>16	R	<=1	S	<=1	S
S	>16/8	R	>16/8	R	>16	R	<=1	S	<=1	S
S	>16/8	R	>16/8	R	>16	R	>16	R	>16	R
S	<=8/4	S	<=8/4	S	>16	R	<=1	S	<=1	S
S	>16/8	R	>16/8	R	>16	R	>16	R	>16	R
S	>16/8	R	>16/8	R	>16	R*	>16	ESBL	>16	R*
S	>16/8	R	>16/8	R	>16	R*	>16	ESBL	>16	R*
S	>16/8	R	>16/8	R	>16	R*	>16	ESBL	>16	R*
S	>16/8	R	>16/8	R	>16	R	>16	R	>16	R
S	>16/8	R	>16/8	R	>16	R*	>16	ESBL	16	R*
S	>16/8	R	>16/8	R	>16	R*	>16	ESBL	>16	R*
R	>16/8	R	>16/8	R	>16	R	>16	R	>16	R
R	>16/8	R	>16/8	R	>16	R	>16	R	>16	R
S	>16/8	R	>16/8	R	>16	R*	>16	ESBL	>16	R*
S	16/8	I	>16/8	R	>16	R	<=1	S	<=1	S
S	16/8	I	>16/8	R	>16	R*	>16	ESBL	>16	R*
S	>16/8	R	>16/8	R	>16	R	>16	R	>16	R
S	16/8	I	>16/8	R	>16	R	<=1	S	<=1	S
S	>16/8	R	>16/8	R	>16	R	>16	R	>16	R
S	>16/8	R	>16/8	R	>16	R*	>16	ESBL	>16	R*
S	>16/8	R	>16/8	R	>16	R*	>16	ESBL	>16	R*
S	>16/8	R	>16/8	R	>16	R	>16	R	>16	R
S	>16/8	R	>16/8	R	>16	R	<=1	S	<=1	S
S	>16/8	R	>16/8	R	>16	R*	>16	ESBL	>16	R*
S	>16/8	R	>16/8	R	>16	R*	>16	ESBL	>16	R*
S	>16/8	R	>16/8	R	>16	R	>16	R	>16	R
S	>16/8	R	>16/8	R	>16	R*	>16	ESBL	>16	R*
R	>16/8	R	>16/8	R	>16	R	>16	R	>16	R
S	>16/8	R	>16/8	R	>16	R	>16	R	>16	R
S	>16/8	R	>16/8	R	>16	R*	>16	ESBL	>16	R*
S	>16/8	R	>16/8	R	>16	R*	>16	ESBL	16	R*
S	>16/8	R	>16/8	R	>16	R	>16	R	>16	R
S	>16/8	R	>16/8	R	>16	R	>16	R	4	S
S	>16/8	R	>16/8	R	>16	R*	>16	ESBL	>16	R*
S	>16/8	R	>16/8	R	>16	R	<=1	S	<=1	S
S	>16/8	R	>16/8	R	>16	R	>16	R	>16	R
S	>16/8	R	>16/8	R	>16	R	>16	R	>16	R
S	>16/8	R	>16/8	R	>16	R*	>16	ESBL	>16	R*
S	>16/8	R	>16/8	R	>16	R	>16	R	>16	R
S	>16/8	R	>16/8	R	>16	R*	>16	ESBL	>16	R*
S	>16/8	R	>16/8	R	>16	R*	>16	ESBL	>16	R*
S	16/8	I	>16/8	R	>16	R*	>16	ESBL	>16	R*

I	>16/8	R	>16/8	R	>16	R	>16	R	>16	R
R	>16/8	R	>16/8	R	>16	R	>16	R	>16	R
R	>16/8	R	>16/8	R	>16	R	>16	R	>16	R
S	>16/8	R	>16/8	R	>16	R	>16	R	>16	R
R	>16/8	R	>16/8	R	>16	R	>16	R	>16	R
S	>16/8	R	>16/8	R	>16	R	>16	R	>16	R
S	>16/8	R	>16/8	R	>16	R	>16	R	>16	R
R	>16/8	R	>16/8	R	>16	R	>16	R	>16	R
S	>16/8	R	>16/8	R	>16	R*	>16	ESBL	>16	R*
R	>16/8	R	>16/8	R	>16	R	>16	R	>16	R
S	>16/8	R	>16/8	R	>16	R*	>16	ESBL	>16	R*
S	>16/8	R	>16/8	R	>16	R*	>16	ESBL	>16	R*
S	>16/8	R	>16/8	R	>16	R*	>16	ESBL	>16	R*
S	>16/8	R	>16/8	R	>16	R	>16	R	>16	R
S	>16/8	R	>16/8	R	>16	R	>16	R	>16	R
S	>16/8	R	>16/8	R	>16	R	>16	R	>16	R
S	>16/8	R	>16/8	R	>16	R	>16	R	>16	R
R	>16/8	R	>16/8	R	>16	R	>16	R	>16	R
R	>16/8	R	>16/8	R	>16	R	>16	R	>16	R
R	>16/8	R	>16/8	R	>16	R	>16	R	>16	R
S	>16/8	R	>16/8	R	>16	R	<=1	S	<=1	S
S	>16/8	R	>16/8	R	>16	R*	>16	ESBL	>16	R*
S	>16/8	R	>16/8	R	>16	R*	>16	ESBL	>16	R*

S	>16/8	R	>16/8	R	>16	R	>16	R	>16	R

S	>16/8	R	>16/8	R	>16	R	>16	R	>16	R

S	>16/8	R	>16/8	R	>16	R*	>16	ESBL	>16	R*
S	>16/8	R	>16/8	R	>16	R	>16	R	>16	R
S	>16/8	R	>16/8	R	>16	R*	>16	ESBL	>16	R*
S	>16/8	R	>16/8	R	>16	R	<=1	S	4	S

S	>16/8	R	>16/8	R	>16	R*	>16	ESBL	>16	R*
S	>16/8	R	>16/8	R	>16	R*	>16	ESBL	>16	R*
S	>16/8	R	>16/8	R	>16	R	>16	R	>16	R
S	>16/8	R	>16/8	R	>16	R	>16	R	>16	R
S	>16/8	R	>16/8	R	>16	R	>16	R	>16	R
S	>16/8	R	>16/8	R	>16	R	<=1	S	4	S
S	>16/8	R	>16/8	R	>16	R	>16	R	>16	R
S	>16/8	R	>16/8	R	>16	R*	8	ESBL	>16	R*
S	>16/8	R	>16/8	R	>16	R	>16	R	>16	R
S	>16/8	R	>16/8	R	>16	R*	>16	ESBL	>16	R*
S	>16/8	R	>16/8	R	>16	R*	>16	ESBL	>16	R*
R	>16/8	R	>16/8	R	>16	R	>16	R	>16	R
R	>16/8	R	>16/8	R	>16	R	>16	R	>16	R
S	>16/8	R	>16/8	R	>16	R	>16	R	>16	R
S	>16/8	R	>16/8	R	>16	R*	>16	ESBL	>16	R*
S	>16/8	R	>16/8	R	>16	R	>16	R	>16	R
S	>16/8	R	>16/8	R	>16	R*	>16	ESBL	>16	R*
R	>16/8	R	>16/8	R	>16	R*	>16	ESBL	>16	R*
S	>16/8	R	>16/8	R	>16	R	>16	R	>16	R
S	>16/8	R	>16/8	R	>16	R*	>16	ESBL	>16	R*
S	>16/8	R	>16/8	R	>16	R*	16	ESBL	<=1	R*
S	>16/8	R	>16/8	R	>16	R	>16	R	>16	R
S	>16/8	R	>16/8	R	>16	R*	>16	ESBL	>16	R*
S	>16/8	R	>16/8	R	>16	R*	>16	ESBL	>16	R*
S	>16/8	R	>16/8	R	>16	R	>16	R	>16	R
S	>16/8	R	>16/8	R	>16	R	>16	R	>16	R
S	>16/8	R	>16/8	R	>16	R*	>16	ESBL	>16	R*
S	>16/8	R	>16/8	R	>16	R	>16	R	>16	R
S	>16/8	R	>16/8	R	>16	R*	>16	ESBL	>16	R*
S	>16/8	R	>16/8	R	>16	R*	>16	ESBL	>16	R*
S	>16/8	R	>16/8	R	>16	R*	>16	ESBL	>16	R*
S	>16/8	R	>16/8	R	>16	R	>16	R	>16	R
S	>16/8	R	>16/8	R	>16	R	>16	R	>16	R
S	>16/8	R	>16/8	R	>16	R*	>16	ESBL	>16	R*
S	>16/8	R	>16/8	R	>16	R*	>16	ESBL	>16	R*
S	>16/8	R	>16/8	R	>16	R	>16	R	>16	R
S	>16/8	R	>16/8	R	>16	R*	>16	ESBL	>16	R*
S	>16/8	R	16/8	I	>16	R*	4	ESBL	>16	R*
S	16/8	I	>16/8	R	>16	R	4	S	<=1	S
S	>16/8	R	>16/8	R	>16	R	>16	R	>16	R

S	>16/8	R	>16/8	R	>16	R	>16	R	>16	R
S	>16/8	R	>16/8	R	>16	R	>16	R	>16	R
S	>16/8	R	>16/8	R	>16	R*	8	ESBL	<=1	R*
R	>16/8	R	>16/8	R	>16	R	>16	R	>16	R
S	>16/8	R	>16/8	R	>16	R*	>16	ESBL	>16	R*
S	>16/8	R	>16/8	R	>16	R*	>16	ESBL	16	R*
S	>16/8	R	>16/8	R	>16	R*	>16	ESBL	>16	R*
S	>16/8	R	>16/8	R	>16	R	>16	R	>16	R
S	>16/8	R	>16/8	R	>16	R	>16	R	>16	R
S	>16/8	R	>16/8	R	>16	R	>16	R	>16	R
S	16/8	I	>16/8	R	>16	R	<=1	S	<=1	S
S	<=8/4	S	>16/8	R	>16	R	<=1	S	<=1	S
S	>16/8	R	<=8/4	S	>16	R	<=1	S	<=1	S
S	>16/8	R	>16/8	R	>16	R	<=1	S	<=1	S
S	>16/8	R	>16/8	R	>16	R	>16	R	>16	R
S	<=8/4	S	<=8/4	S	>16	R	<=1	S	<=1	S
S	<=8/4	S	<=8/4	S	>16	R	<=1	S	<=1	S
S	>16/8	R	>16/8	R	>16	R	<=1	S	<=1	S
S	<=8/4	S	<=8/4	S	>16	R	<=1	S	<=1	S
S	<=8/4	S	<=8/4	S	>16	R	<=1	S	<=1	S
S	>16/8	R	>16/8	R	>16	R*	>16	ESBL	>16	R*
S	>16/8	R	>16/8	R	>16	R	>16	R	>16	R
S	>16/8	R	>16/8	R	>16	R	>16	R	>16	R
S	>16/8	R	>16/8	R	>16	R*	>16	ESBL	>16	R*
S	>16/8	R	>16/8	R	>16	R*	>16	ESBL	>16	R*
S	>16/8	R	>16/8	R	>16	R*	>16	ESBL	>16	R*
S	>16/8	R	>16/8	R	>16	R	>16	R	>16	R
S	>16/8	R	>16/8	R	>16	R*	>16	ESBL	>16	R*
S	>16/8	R	>16/8	R	>16	R*	>16	ESBL	>16	R*
R	>16/8	R	>16/8	R	>16	R	>16	R	>16	R
S	>16/8	R	>16/8	R	>16	R*	>16	ESBL	>16	R*
S	>16/8	R	>16/8	R	>16	R	>16	R	>16	R
S	>16/8	R	>16/8	R	>16	R	>16	R	>16	R
S	>16/8	R	>16/8	R	>16	R*	>16	ESBL	>16	R*
S	>16/8	R	>16/8	R	>16	R*	>16	ESBL	>16	R*
S	>16/8	R	>16/8	R	>16	R	<=1	S	<=1	S
S	>16/8	R	16/8	I	>16	R*	8	ESBL	4	R*
S	>16/8	R	>16/8	R	>16	R*	>16	ESBL	>16	R*
S	>16/8	R	>16/8	R	>16	R*	>16	ESBL	16	R*
S	>16/8	R	>16/8	R	>16	R	<=1	S	<=1	S
S	>16/8	R	>16/8	R	>16	R*	>16	ESBL	>16	R*
R	>16/8	R	>16/8	R	>16	R	>16	R	>16	R
R	>16/8	R	>16/8	R	>16	R	8	I	<=1	S
S	>16/8	R	>16/8	R	>16	R	>16	R	>16	R
R	>16/8	R	>16/8	R	>16	R	>16	R	>16	R
S	>16/8	R	>16/8	R	>16	R	>16	R	>16	R
S	>16/8	R	>16/8	R	>16	R	>16	R	>16	R
R	>16/8	R	>16/8	R	>16	R	>16	R	>16	R
R	>16/8	R	>16/8	R	>16	R	>16	R	>16	R
S	>16/8	R	>16/8	R	>16	R	>16	R	>16	R
S	>16/8	R	>16/8	R	>16	R	<=1	S	8	S
S	>16/8	R	>16/8	R	>16	R	>16	R	>16	R
S	>16/8	R	>16/8	R	>16	R	>16	R	>16	R
S	>16/8	R	>16/8	R	>16	R*	>16	ESBL	>16	R*
S	>16/8	R	>16/8	R	>16	R*	>16	ESBL	>16	R*
S	16/8	I	>16/8	R	>16	R*	>16	ESBL	>16	R*

S	>16/8	R	>16/8	R	>16	R	>16	R	>16	R
S	>16/8	R	>16/8	R	>16	R	<=1	S	>16	R
S	>16/8	R	>16/8	R	>16	R*	>16	ESBL	>16	R*
S	>16/8	R	>16/8	R	>16	R*	>16	ESBL	>16	R*
S	>16/8	R	>16/8	R	>16	R*	>16	ESBL	>16	R*
R	>16/8	R	>16/8	R	>16	R	>16	R	>16	R
S	>16/8	R	>16/8	R	>16	R	>16	R	>16	R
R	>16/8	R	>16/8	R	>16	R	<=1	S	<=1	S
S	>16/8	R	>16/8	R	>16	R*	>16	ESBL	>16	R*
S	>16/8	R	>16/8	R	>16	R*	>16	ESBL	>16	R*
R	>16/8	R	>16/8	R	>16	R	>16	R	>16	R
S	>16/8	R	>16/8	R	>16	R	>16	R	>16	R
S	>16/8	R	>16/8	R	>16	R	>16	R	>16	R
S	>16/8	R	>16/8	R	>16	R*	>16	ESBL	>16	R*
S	>16/8	R	>16/8	R	>16	R*	>16	ESBL	>16	R*
S	>16/8	R	>16/8	R	>16	R*	>16	ESBL	>16	R*
R	>16/8	R	>16/8	R	>16	R	>16	R	>16	R
S	>16/8	R	>16/8	R	>16	R*	>16	ESBL	>16	R*
S	>16/8	R	>16/8	R	>16	R*	>16	ESBL	>16	R*
S	>16/8	R	>16/8	R	>16	R*	>16	ESBL	>16	R*
S	>16/8	R	>16/8	R	>16	R	>16	R	>16	R
S	>16/8	R	>16/8	R	>16	R*	8	ESBL	<=1	R*
S	>16/8	R	>16/8	R	>16	R*	>16	ESBL	>16	R*
S	>16/8	R	>16/8	R	>16	R	>16	R	>16	R
R	>16/8	R	>16/8	R	>16	R	>16	R	>16	R
S	>16/8	R	>16/8	R	>16	R	>16	R	>16	R
S	>16/8	R	>16/8	R	>16	R	<=1	S	8	S
S	>16/8	R	>16/8	R	>16	R	>16	R	>16	R
S	>16/8	R	>16/8	R	>16	R*	>16	ESBL	>16	R*
S	>16/8	R	>16/8	R	>16	R*	>16	ESBL	>16	R*
S	>16/8	R	>16/8	R	16	R*	>16	R	>16	R
R	>16/8	R	>16/8	R	>16	R	>16	R	>16	R
S	>16/8	R	>16/8	R	>16	R	>16	R	>16	R
R	>16/8	R	>16/8	R	>16	R	>16	R	8	S
S	>16/8	R	>16/8	R	>16	R	>16	R	>16	R
R	>16/8	R	>16/8	R	>16	R	>16	R	>16	R
R	>16/8	R	16/8	I	>16	R	>16	R	>16	R
R	>16/8	R	>16/8	R	>16	R	>16	R	>16	R
S	>16/8	R	>16/8	R	>16	R*	>16	ESBL	>16	R*
R	>16/8	R	>16/8	R	>16	R*	>16	ESBL	>16	R*
R	>16/8	R	16/8	I	>16	R	>16	R	>16	R
S	>16/8	R	>16/8	R	>16	R	>16	R	>16	R
S	>16/8	R	>16/8	R	>16	R	>16	R	>16	R
I	>16/8	R	>16/8	R	>16	R	>16	R	>16	R
S	>16/8	R	>16/8	R	>16	R	>16	R	>16	R
S	>16/8	R	>16/8	R	>16	R	>16	R	>16	R
S	>16/8	R	>16/8	R	>16	R	>16	R	>16	R
S	>16/8	R	>16/8	R	>16	R	<=1	S	4	S
S	>16/8	R	>16/8	R	>16	R	>16	R	>16	R
R	>16/8	R	>16/8	R	>16	R	<=1	S	>16	R
S	<=8/4	S	>16/8	R	>16	R*	>16	ESBL	>16	R*
S	>16/8	R	>16/8	R	>16	R*	>16	ESBL	>16	R*
S	>16/8	R	>16/8	R	>16	R	>16	R	>16	R
R	>16/8	R	>16/8	R	>16	R	>16	R	>16	R
I	>16/8	R	>16/8	R	>16	R*	>16	ESBL	>16	R*

S	>16/8	R	>16/8	R	>16	R*	>16	ESBL	>16	R*
R	>16/8	R	>16/8	R	>16	R	>16	R	>16	R
R	>16/8	R	>16/8	R	>16	R*	>16	ESBL	>16	R*
I	>16/8	R	>16/8	R	>16	R*	>16	ESBL	>16	R*
S	>16/8	R	>16/8	R	>16	R*	>16	ESBL	>16	R*
R	>16/8	R	>16/8	R	>16	R	>16	R	>16	R
S	>16/8	R	>16/8	R	>16	R	>16	R	>16	R
S	>16/8	R	>16/8	R	>16	R	>16	R	>16	R
R	>16/8	R	>16/8	R	>16	R	>16	R	>16	R
S	>16/8	R	>16/8	R	>16	R*	>16	ESBL	>16	R*
S	>16/8	R	>16/8	R	>16	R	<=1	S	<=1	S
S	>16/8	R	>16/8	R	>16	R*	>16	ESBL	>16	R*
S	>16/8	R	>16/8	R	>16	R*	>16	ESBL	>16	R*
R	>16/8	R	>16/8	R	>16	R	>16	R	>16	R
R	>16/8	R	>16/8	R	>16	R	>16	R	>16	R
S	>16/8	R	>16/8	R	>16	R	>16	R	>16	R
R	>16/8	R	>16/8	R	>16	R	>16	R	>16	R
S	>16/8	R	>16/8	R	>16	R	>16	R	>16	R
S	>16/8	R	>16/8	R	>16	R*	>16	ESBL	>16	R*
S	>16/8	R	>16/8	R	>16	R	>16	R	>16	R
S	>16/8	R	>16/8	R	>16	R	>16	R	>16	R
S	>16/8	R	>16/8	R	>16	R	<=1	S	4	S
S	>16/8	R	>16/8	R	>16	R	>16	R	>16	R
S	>16/8	R	>16/8	R	>16	R	>16	R	>16	R
S	>16/8	R	>16/8	R	>16	R*	>16	ESBL	>16	R*
S	>16/8	R	>16/8	R	>16	R*	>16	ESBL	>16	R*
S	>16/8	R	>16/8	R	>16	R*	>16	ESBL	>16	R*
R	>16/8	R	>16/8	R	>16	R	>16	R	>16	R
S	>16/8	R	>16/8	R	>16	R*	>16	ESBL	>16	R*
S	>16/8	R	>16/8	R	>16	R	>16	R	>16	R
R	>16/8	R	>16/8	R	>16	R	>16	R	>16	R
R	>16/8	R	>16/8	R	>16	R	>16	R	>16	R
S	>16/8	R	>16/8	R	>16	R	>16	R	>16	R
S	>16/8	R	>16/8	R	>16	R*	>16	ESBL	>16	R*
S	>16/8	R	>16/8	R	>16	R*	>16	ESBL	>16	R*
S	>16/8	R	>16/8	R	>16	R*	>16	ESBL	>16	R*
R	>16/8	R	>16/8	R	>16	R	>16	R	>16	R
R	>16/8	R	>16/8	R	>16	R	>16	R	>16	R
S	>16/8	R	>16/8	R	>16	R*	>16	ESBL	>16	R*
S	>16/8	R	>16/8	R	>16	R	>16	R	>16	R
S	>16/8	R	>16/8	R	>16	R	>16	R	>16	R
S	>16/8	R	>16/8	R	>16	R	>16	R	>16	R
S	>16/8	R	>16/8	R	>16	R	>16	R	>16	R
S	>16/8	R	>16/8	R	>16	R	>16	R	>16	R
S	>16/8	R	>16/8	R	>16	R*	>16	ESBL	>16	R*
S	>16/8	R	>16/8	R	>16	R	>16	R	>16	R
R	>16/8	R	>16/8	R	>16	R	>16	R	>16	R
S	>16/8	R	>16/8	R	>16	R*	>16	ESBL	>16	R*
R	>16/8	R	>16/8	R	>16	R	>16	R	>16	R
S	>16/8	R	>16/8	R	>16	R*	>16	ESBL	>16	R*
S	>16/8	R	>16/8	R	>16	R	>16	R	>16	R
S	>16/8	R	>16/8	R	>16	R	>16	R	>16	R
S	>16/8	R	>16/8	R	>16	R*	>16	ESBL	>16	R*
S	>16/8	R	>16/8	R	>16	R*	>16	ESBL	>16	R*
S	>16/8	R	>16/8	R	>16	R*	>16	ESBL	>16	R*
S	>16/8	R	>16/8	R	>16	R	>16	R	>16	R

R	>16/8	R	>16/8	R	>16	R	>16	R	>16	R
R	>16/8	R	>16/8	R	>16	R	8	I	<=1	S
S	>16/8	R	>16/8	R	>16	R*	>16	ESBL	>16	R*
S	>16/8	R	>16/8	R	>16	R*	>16	ESBL	>16	R*

S	>16/8	R	<=8/4	S	16	I	<=1	I	<=1	I
S	>16/8	R	>16/8	R	>16	R	>16	R	>16	R
S	>16/8	R	>16/8	R	>16	R*	>16	ESBL	>16	R*
S	>16/8	R	>16/8	R	>16	R*	>16	ESBL	>16	R*
S	>16/8	R	>16/8	R	>16	R	>16	R	>16	R
S	>16/8	R	>16/8	R	>16	R	>16	R	>16	R
R	>16/8	R	>16/8	R	>16	R	>16	R	>16	R
S	>16/8	R	>16/8	R	>16	R	<=1	S	16	I
S	>16/8	R	>16/8	R	>16	R	>16	R	>16	R
S	>16/8	R	>16/8	R	>16	R*	>16	ESBL	>16	R*
S	>16/8	R	>16/8	R	>16	R*	>16	ESBL	>16	R*
S	>16/8	R	>16/8	R	>16	R	<=1	S	4	S
S	>16/8	R	>16/8	R	>16	R*	>16	ESBL	8	R*
S	>16/8	R	>16/8	R	>16	R	>16	R	<=1	S
S	>16/8	R	>16/8	R	>16	R*	>16	ESBL	>16	R*
S	>16/8	R	>16/8	R	>16	R	>16	R	>16	R
S	>16/8	R	>16/8	R	>16	R	<=1	S	16	I
S	>16/8	R	>16/8	R	>16	R	>16	R	>16	R
S	>16/8	R	>16/8	R	>16	R	>16	R	>16	R
S	>16/8	R	>16/8	R	>16	R*	>16	ESBL	>16	R*
S	>16/8	R	>16/8	R	>16	R	<=1	S	<=1	S
I	>16/8	R	>16/8	R	>16	R	>16	R	>16	R
S	>16/8	R	>16/8	R	>16	R	>16	R	>16	R
S	>16/8	R	>16/8	R	>16	R	>16	R	>16	R
S	>16/8	R	>16/8	R	>16	R	>16	R	>16	R
S	>16/8	R	>16/8	R	>16	R*	>16	ESBL	>16	R*
S	>16/8	R	>16/8	R	>16	R	>16	R	>16	R
S	>16/8	R	>16/8	R	>16	R*	>16	ESBL	>16	R*
S	>16/8	R	>16/8	R	>16	R*	>16	ESBL	>16	R*
S	>16/8	R	>16/8	R	>16	R*	>16	ESBL	>16	R*
S	>16/8	R	>16/8	R	>16	R*	>16	ESBL	16	R*
S	>16/8	R	>16/8	R	>16	R	>16	R	>16	R
S	>16/8	R	>16/8	R	>16	R*	>16	ESBL	>16	R*

S	>16/8	R	>16/8	R	>16	R*	>16	ESBL	>16	R*
R	>16/8	R	>16/8	R	>16	R	>16	R	>16	R
S	>16/8	R	>16/8	R	>16	R*	>16	ESBL	>16	R*
R	>16/8	R	>16/8	R	>16	R	>16	R	>16	R
S	>16/8	R	>16/8	R	>16	R*	8	ESBL	16	R*
S	>16/8	R	>16/8	R	>16	R	>16	R	>16	R
S	>16/8	R	>16/8	R	>16	R*	>16	ESBL	>16	R*
R	>16/8	R	>16/8	R	>16	R	>16	R	>16	R
S	>16/8	R	>16/8	R	>16	R*	>16	ESBL	>16	R*
S	>16/8	R	>16/8	R	>16	R*	>16	ESBL	>16	R*
I	>16/8	R	>16/8	R	>16	R	>16	R	>16	R
S	>16/8	R	>16/8	R	>16	R	>16	R	>16	R
S	>16/8	R	>16/8	R	>16	R	>16	R	>16	R
S	>16/8	R	>16/8	R	>16	R*	>16	ESBL	>16	R*
I	>16/8	R	>16/8	R	>16	R*	>16	ESBL	>16	R*
S	>16/8	R	>16/8	R	>16	R	>16	R	>16	R
S	>16/8	R	>16/8	R	>16	R	16	R	>16	R
S	>16/8	R	>16/8	R	>16	R*	>16	ESBL	>16	R*
S	>16/8	R	>16/8	R	>16	R	>16	R	16	I
R	>16/8	R	>16/8	R	>16	R	>16	R	>16	R
R	>16/8	R	>16/8	R	>16	R	>16	R	>16	R
S	>16/8	R	>16/8	R	>16	R*	>16	ESBL	>16	R*
S	>16/8	R	>16/8	R	>16	R*	>16	ESBL	>16	R*
R	>16/8	R	>16/8	R	>16	R	>16	R	>16	R
S	>16/8	R	>16/8	R	>16	R*	<=1	R*	<=1	R*
S	>16/8	R	>16/8	R	>16	R	>16	R	>16	R
S	>16/8	R	>16/8	R	>16	R	>16	R	>16	R
R	>16/8	R	>16/8	R	>16	R	>16	R	>16	R
R	>16/8	R	>16/8	R	>16	R	>16	R	>16	R
S	>16/8	R	>16/8	R	>16	R*	>16	ESBL	>16	R*
S	>16/8	R	>16/8	R	>16	R*	>16	ESBL	>16	R*
S	>16/8	R	>16/8	R	>16	R*	>16	ESBL	>16	R*
S	>16/8	R	>16/8	R	>16	R	<=1	S	>16	R
S	>16/8	R	>16/8	R	>16	R*	>16	ESBL	>16	R*
S	>16/8	R	>16/8	R	>16	R*	>16	ESBL	>16	R*
S	>16/8	R	>16/8	R	>16	R	<=1	S	16	I
S	>16/8	R	>16/8	R	>16	R*	>16	ESBL	>16	R*
S	>16/8	R	>16/8	R	>16	R*	>16	ESBL	>16	R*
S	>16/8	R	>16/8	R	>16	R*	>16	ESBL	>16	R*
S	>16/8	R	>16/8	R	>16	R*	>16	ESBL	>16	R*
S	>16/8	R	>16/8	R	>16	R	>16	R	>16	R
S	>16/8	R	>16/8	R	>16	R	>16	R	>16	R
S	16/8	I	>16/8	R	>16	R*	>16	ESBL	>16	R*
S	>16/8	R	>16/8	R	>16	R*	>16	ESBL	>16	R*
S	>16/8	R	>16/8	R	>16	R*	>16	ESBL	>16	R*
S	>16/8	R	>16/8	R	>16	R*	>16	ESBL	>16	R*
S	>16/8	R	>16/8	R	>16	R	>16	R	>16	R
R	>16/8	R	>16/8	R	>16	R	>16	R	>16	R
S	>16/8	R	>16/8	R	>16	R*	>16	ESBL	>16	R*
S	>16/8	R	>16/8	R	>16	R*	>16	ESBL	>16	R*
S	>16/8	R	>16/8	R	>16	R*	>16	ESBL	>16	R*
R	>16/8	R	>16/8	R	>16	R*	>16	ESBL	>16	R*
R	>16/8	R	>16/8	R	>16	R	>16	R	>16	R
S	>16/8	R	>16/8	R	>16	R*	>16	ESBL	>16	R*
S	>16/8	R	>16/8	R	>16	R	8	R	>16	R
S	>16/8	R	>16/8	R	>16	R	>16	R	>16	R

S	>16/8	R	>16/8	R	>16	R	>16	R	>16	R
S	>16/8	R	>16/8	R	>16	R	>16	R	>16	R
S	>16/8	R	>16/8	R	>16	R	>16	R	>16	R
R	>16/8	R	>16/8	R	>16	R	>16	R	>16	R
S	>16/8	R	>16/8	R	>16	R	>16	R	>16	R
S	>16/8	R	>16/8	R	>16	R	<=1	S	<=1	S
S	>16/8	R	>16/8	R	>16	R	>16	R	>16	R
S	>16/8	R	>16/8	R	>16	R*	>16	ESBL	>16	R*
S	>16/8	R	>16/8	R	>16	R	<=1	S	4	S
S	>16/8	R	>16/8	R	>16	R	>16	R	>16	R
S	<=8/4	S	>16/8	R	>16	R*	>16	ESBL	2	R*
S	>16/8	R	>16/8	R	>16	R	<=1	S	>16	R
S	>16/8	R	>16/8	R	>16	R*	>16	ESBL	>16	R*
S	>16/8	R	>16/8	R	>16	R*	>16	ESBL	>16	R*
S	>16/8	R	>16/8	R	>16	R	>16	R	>16	R
S	>16/8	R	>16/8	R	>16	R	>16	R	>16	R
S	>16/8	R	>16/8	R	>16	R*	>16	ESBL	>16	R*
S	>16/8	R	>16/8	R	>16	R	>16	R	>16	R
S	<=8/4	S	<=8/4	S	>16	R	<=1	S	<=1	S
S	>16/8	R	>16/8	R	>16	R*	>16	ESBL	>16	R*
S	>16/8	R	>16/8	R	>16	R	>16	R	>16	R
S	>16/8	R	>16/8	R	>16	R*	>16	ESBL	>16	R*
S	>16/8	R	>16/8	R	>16	R	>16	R	>16	R
R	>16/8	R	>16/8	R	>16	R	>16	R	>16	R
S	>16/8	R	>16/8	R	>16	R*	>16	ESBL	>16	R*
S	>16/8	R	>16/8	R	>16	R	>16	R	>16	R
R	>16/8	R	>16/8	R	>16	R	>16	R	>16	R
S	>16/8	R	>16/8	R	>16	R*	>16	ESBL	>16	R*
S	>16/8	R	>16/8	R	>16	R*	>16	ESBL	>16	R*
S	>16/8	R	>16/8	R	>16	R*	>16	ESBL	>16	R*
S	>16/8	R	>16/8	R	>16	R	>16	R	>16	R
S	>16/8	R	>16/8	R	>16	R*	>16	ESBL	>16	R*
S	>16/8	R	>16/8	R	>16	R	>16	R	>16	R
S	>16/8	R	>16/8	R	>16	R	>16	R	>16	R
S	>16/8	R	>16/8	R	>16	R	>16	R	>16	R
S	>16/8	R	>16/8	R	>16	R	>16	R	>16	R
S	>16/8	R	>16/8	R	>16	R	<=1	S	8	S
R	>16/8	R	>16/8	R	>16	R	>16	R	>16	R
R	>16/8	R	>16/8	R	>16	R	>16	R	>16	R
S	>16/8	R	>16/8	R	>16	R	>16	R	>16	R
S	>16/8	R	>16/8	R	>16	R*	>16	ESBL	>16	R*
S	>16/8	R	>16/8	R	>16	R	<=1	S	<=1	S
S	>16/8	R	>16/8	R	>16	R*	>16	ESBL	>16	R*
R	>16/8	R	>16/8	R	>16	R	>16	R	>16	R
R	>16/8	R	>16/8	R	>16	R	>16	R	>16	R
S	>16/8	R	>16/8	R	>16	R*	>16	ESBL	>16	R*
S	>16/8	R	>16/8	R	>16	R	>16	R	>16	R
S	>16/8	R	>16/8	R	>16	R	>16	R	>16	R
R	>16/8	R	>16/8	R	>16	R	>16	R	>16	R
R	>16/8	R	>16/8	R	>16	R	>16	R	>16	R
S	>16/8	R	>16/8	R	>16	R*	>16	ESBL	>16	R*
R	>16/8	R	>16/8	R	>16	R	>16	R	>16	R
S	>16/8	R	16/8	I	>16	R	<=1	S	<=1	S
R	>16/8	R	>16/8	R	>16	R	>16	R	>16	R
S	>16/8	R	>16/8	R	>16	R	>16	R	>16	R
I	>16/8	R	>16/8	R	>16	R	>16	R	>16	R

S	>16/8	R	>16/8	R	>16	R	>16	R	>16	R
S	>16/8	R	>16/8	R	>16	R*	>16	ESBL	>16	R*
S	>16/8	R	>16/8	R	>16	R	<=1	S	4	S
S	>16/8	R	>16/8	R	>16	R*	>16	ESBL	>16	R*
S	>16/8	R	>16/8	R	>16	R	>16	R	>16	R
S	>16/8	R	>16/8	R	>16	R	>16	R	16	I
S	>16/8	R	>16/8	R	>16	R*	>16	ESBL	8	R*
S	>16/8	R	>16/8	R	>16	R	4	S	>16	R
S	>16/8	R	>16/8	R	>16	R	<=1	S	2	S
S	>16/8	R	>16/8	R	>16	R	>16	R	>16	R
S	>16/8	R	>16/8	R	>16	R	>16	R	>16	R
S	>16/8	R	>16/8	R	>16	R	>16	R	>16	R
S	>16/8	R	>16/8	R	>16	R*	>16	ESBL	>16	R*
R	>16/8	R	>16/8	R	>16	R	>16	R	>16	R
S	>16/8	R	>16/8	R	>16	R	>16	R	>16	R
S	>16/8	R	>16/8	R	>16	R	>16	R	>16	R
S	>16/8	R	>16/8	R	>16	R*	>16	ESBL	>16	R*
S	>16/8	R	>16/8	R	>16	R	>16	R	8	S
R	>16/8	R	>16/8	R	>16	R	>16	R	>16	R
R	>16/8	R	>16/8	R	>16	R	<=1	S	>16	R
S	>16/8	R	>16/8	R	>16	R	>16	R	>16	R
S	>16/8	R	>16/8	R	>16	R*	>16	ESBL	>16	R*
R	>16/8	R	>16/8	R	>16	R	>16	R	>16	R
S	>16/8	R	>16/8	R	>16	R*	>16	ESBL	>16	R*
S	>16/8	R	>16/8	R	>16	R*	>16	ESBL	>16	R*
S	>16/8	R	>16/8	R	>16	R	4	S	<=1	S
S	>16/8	R	>16/8	R	>16	R	>16	R	>16	R
S	>16/8	R	>16/8	R	>16	R*	>16	ESBL	>16	R*
S	>16/8	R	>16/8	R	>16	R	>16	R	>16	R
S	>16/8	R	>16/8	R	>16	R*	>16	ESBL	>16	R*
S	>16/8	R	>16/8	R	>16	R*	>16	ESBL	>16	R*
R	>16/8	R	>16/8	R	>16	R	>16	R	>16	R
S	>16/8	R	>16/8	R	>16	R	>16	R	>16	R
S	>16/8	R	>16/8	R	>16	R	4	S	8	S
S	<=8/4	S	<=8/4	S	>16	R	<=1	S	<=1	S
S	>16/8	R	>16/8	R	>16	R	>16	R	>16	R
S	>16/8	R	>16/8	R	>16	R*	>16	ESBL	>16	R*
R	>16/8	R	>16/8	R	>16	R	<=1	S	<=1	S
S	>16/8	R	>16/8	R	>16	R*	>16	ESBL	>16	R*
S	>16/8	R	16/8	I	>16	R	<=1	S	<=1	S
S	>16/8	R	>16/8	R	>16	R*	>16	ESBL	>16	R*
S	>16/8	R	>16/8	R	>16	R*	>16	ESBL	>16	R*
S	>16/8	R	>16/8	R	>16	R	>16	R	>16	R
S	>16/8	R	>16/8	R	>16	R*	>16	ESBL	>16	R*
S	>16/8	R	>16/8	R	>16	R	<=1	S	<=1	S
R	>16/8	R	>16/8	R	>16	R	>16	R	>16	R
S	>16/8	R	>16/8	R	>16	R*	>16	ESBL	>16	R*
R	>16/8	R	>16/8	R	>16	R	>16	R	>16	R
R	>16/8	R	>16/8	R	>16	R	>16	R	>16	R
S	>16/8	R	>16/8	R	>16	R*	>16	ESBL	>16	R*
S	>16/8	R	>16/8	R	>16	R	>16	R	>16	R
S	>16/8	R	>16/8	R	>16	R	<=1	S	4	S
S	>16/8	R	>16/8	R	>16	R*	>16	ESBL	>16	R*
R	>16/8	R	>16/8	R	>16	R	>16	R	>16	R
R	>16/8	R	>16/8	R	>16	R	>16	R	>16	R
R	>16/8	R	>16/8	R	>16	R	>16	R	>16	R

R	>16/8	R	>16/8	R	>16	R	>16	R	>16	R	
R	>16/8	R	>16/8	R	>16	R	>16	R	>16	R	
S	>16/8	R	>16/8	R	>16	R	<=1	S	<=1	S	
S	>16/8	R	>16/8	R	>16	R	>16	R	>16	R	
S	>16/8	R	>16/8	R	>16	R	>16	R	>16	R	
S	>16/8	R	>16/8	R	>16	R*	>16	ESBL	>16	R*	
S	>16/8	R	>16/8	R	>16	R*	>16	ESBL	>16	R*	
S	>16/8	R	16/8	I	>16	R	<=1	S	<=1	S	
S	>16/8	R	>16/8	R	>16	R	>16	R	>16	R	
S	>16/8	R	>16/8	R	>16	R	>16	R	>16	R	
R	>16/8	R	>16/8	R	>16	R	>16	R	>16	R	
S	<=8/4	S	>16/8	R	>16	R	4	S	<=1	S	
S	>16/8	R	>16/8	R	>16	R	>16	R	>16	R	
R	>16/8	R	>16/8	R	>16	R	>16	R	>16	R	
R	>16/8	R	>16/8	R	>16	R	>16	R	>16	R	
S	>16/8	R	>16/8	R	>16	R*	>16	ESBL	>16	R*	
S	>16/8	R	>16/8	R	>16	R	>16	R	>16	R	
S	>16/8	R	>16/8	R	>16	R*	>16	ESBL	>16	R*	
R	>16/8	R	>16/8	R	>16	R	>16	R	>16	R	
S	>16/8	R	>16/8	R	>16	R*	>16	ESBL	>16	R*	
S	>16/8	R	>16/8	R	>16	R*	>16	ESBL	>16	R*	
S	>16/8	R	>16/8	R	>16	R	>16	R	>16	R	
S	>16/8	R	>16/8	R	>16	R*	>16	ESBL	>16	R*	
S	>16/8	R	>16/8	R	>16	R*	>16	ESBL	>16	R*	
S	>16/8	R	>16/8	R	>16	R	>16	R	>16	R	
S	>16/8	R	>16/8	R	>16	R*	>16	ESBL	>16	R*	
S	>16/8	R	>16/8	R	>16	R	<=1	S	8	S	
R	>16/8	R	>16/8	R	>16	R	>16	R	>16	R	
S	>16/8	R	>16/8	R	>16	R*	>16	ESBL	>16	R*	
R	>16/8	R	>16/8	R	>16	R	>16	R	>16	R	
S	>16/8	R	>16/8	R	>16	R*	>16	ESBL	>16	R*	
S	>16/8	R	>16/8	R	>16	R*	>16	ESBL	>16	R*	
S	<=8/4	S	<=8/4	S	>16	R	<=1	S	<=1	S	
S	>16/8	R	>16/8	R	>16	R	>16	R	>16	R	
S	>16/8	R	>16/8	R	>16	R	<=1	S	2	S	
R	>16/8	R	>16/8	R	>16	R	>16	R	>16	R	
R	>16/8	R	16/8	I	>16	R*	>16	ESBL	2	R*	
S	>16/8	R	>16/8	R	>16	R*	>16	ESBL	>16	R*	
S	>16/8	R	>16/8	R	>16	R	>16	R	>16	R	
S	>16/8	R	>16/8	R	>16	R*	>16	ESBL	>16	R*	
S	>16/8	R	>16/8	R	>16	R	>16	R	>16	R	
S	>16/8	R	>16/8	R	>16	R	>16	R	>16	R	
S	>16/8	R	>16/8	R	>16	R*	>16	ESBL	>16	R*	
S	>16/8	R	>16/8	R	>16	R	>16	R	>16	R	
S	>16/8	R	>16/8	R	>16	R	>16	R	>16	R	
S	>16/8	R	>16/8	R	>16	R	>16	R	>16	R	
S	>16/8	R	>16/8	R	>16	R	>16	R	>16	R	
R	>16/8	R	>16/8	R	>16	R	>16	R	>16	R	
S	>16/8	R	>16/8	R	>16	R	>16	R	>16	R	
R	>16/8	R	>16/8	R	>16	R	>16	R	>16	R	
S	>16/8	R	>16/8	R	>16	R	>16	R	>16	R	
S	<=8/4	S	<=8/4	S	>16	R	<=1	S	<=1	S	
S	>16/8	R	>16/8	R	>16	R	>16	R	>16	R	
R	>16/8	R	>16/8	R	>16	R	>16	R	>16	R	
S	>16/8	R	>16/8	R	>16	R	>16	R	>16	R	

S	>16/8	R	>16/8	R	>16	R*	>16	ESBL	>16	R*
S	>16/8	R	>16/8	R	>16	R*	>16	ESBL	>16	R*
R	>16/8	R	>16/8	R	>16	R	>16	R	>16	R
R	>16/8	R	>16/8	R	>16	R	>16	R	>16	R
R	>16/8	R	>16/8	R	>16	R	>16	R	>16	R
R	>16/8	R	>16/8	R	>16	R	>16	R	>16	R
R	>16/8	R	>16/8	R	>16	R	>16	R	>16	R
R	>16/8	R	>16/8	R	>16	R	>16	R	>16	R
R	>16/8	R	>16/8	R	>16	R	>16	R	>16	R
S	>16/8	R	>16/8	R	>16	R	>16	R	>16	R
S	>16/8	R	>16/8	R	>16	R*	>16	ESBL	>16	R*
S	>16/8	R	>16/8	R	>16	R	>16	R	>16	R
S	>16/8	R	>16/8	R	>16	R*	>16	ESBL	>16	R*
S	>16/8	R	>16/8	R	>16	R*	>16	ESBL	>16	R*
S	>16/8	R	>16/8	R	>16	R	<=1	S	16	I
S	>16/8	R	>16/8	R	>16	R	>16	R	>16	R
S	>16/8	R	>16/8	R	>16	R	>16	R	>16	R
S	>16/8	R	>16/8	R	>16	R*	>16	ESBL	>16	R*
S	>16/8	R	>16/8	R	>16	R	<=1	S	2	S
S	>16/8	R	>16/8	R	>16	R*	>16	ESBL	>16	R*
S	>16/8	R	>16/8	R	>16	R	>16	R	>16	R
S	>16/8	R	16/8	I	>16	R	<=1	S	<=1	S
S	>16/8	R	>16/8	R	>16	R*	>16	ESBL	>16	R*
S	>16/8	R	>16/8	R	>16	R	>16	R	>16	R
S	>16/8	R	>16/8	R	>16	R*	>16	ESBL	>16	R*
S	>16/8	R	>16/8	R	>16	R	>16	R	>16	R
S	>16/8	R	>16/8	R	>16	R	>16	R	8	S

R	>16/8	R	>16/8	R	>16	R	>16	R	R

R	>16/8	R	>16/8	R	>16	R	>16	R	R
R	>16/8	R	>16/8	R	>16	R	<=1	S	R

R	>16/8	R	>16/8	R	>16	R	>16	R	R	

R	>16/8	R	>16/8	R	>16	R	4	S	>16	R
R	>16/8	R	>16/8	R	>16	R	>16	R	>16	R
R	>16/8	R	>16/8	R	>16	R	>16	R	>16	R
R	>16/8	R	>16/8	R	>16	R	4	S	16	I
S	>16/8	R	>16/8	R	>16	R	>16	R	>16	R
S	>16/8	R	>16/8	R	>16	R	>16	R	>16	R
S	>16/8	R	>16/8	R	>16	R	>16	R	>16	R
S	>16/8	R	>16/8	R	>16	R*	16	ESBL	>16	R*
R	>16/8	R	>16/8	R	>16	R	>16	R	>16	R
S	>16/8	R	>16/8	R	>16	R	>16	R	>16	R
R	>16/8	R	>16/8	R	>16	R	>16	R	>16	R
S	>16/8	R	>16/8	R	>16	R	<=1	S	<=1	S
S	>16/8	R	>16/8	R	>16	R*	>16	ESBL	>16	R*
S	>16/8	R	>16/8	R	>16	R	>16	R	>16	R
S	>16/8	R	>16/8	R	>16	R*	>16	ESBL	>16	R*
S	>16/8	R	>16/8	R	>16	R	>16	R	>16	R
S	>16/8	R	16/8	I	>16	R	<=1	S	<=1	S
S	>16/8	R	>16/8	R	>16	R	>16	R	>16	R

S	>16/8	R	>16/8	R	>16	R*	>16	ESBL	>16	R*
R	>16/8	R	>16/8	R	>16	R	<=1	S	16	I
R	>16/8	R	>16/8	R	>16	R	>16	R	>16	R
R	>16/8	R	>16/8	R	>16	R	>16	R	>16	R
S	>16/8	R	>16/8	R	>16	R	>16	R	>16	R
S	>16/8	R	>16/8	R	>16	R	>16	R	>16	R
R	>16/8	R	>16/8	R	>16	R	>16	R	>16	R
S	>16/8	R	>16/8	R	>16	R	<=1	S	<=1	S
S	>16/8	R	>16/8	R	>16	R	>16	R	>16	R
S	>16/8	R	>16/8	R	>16	R	>16	R	>16	R
R	>16/8	R	>16/8	R	>16	R	>16	R	>16	R
R	>16/8	R	>16/8	R	>16	R	>16	R	>16	R
S	>16/8	R	>16/8	R	>16	R	>16	R	R	
I	>16/8	R	>16/8	R	>16	R	>16	R	R	
S	>16/8	R	>16/8	R	>16	R	>16	R	>16	R
S	>16/8	R	>16/8	R	>16	R	<=1	S	>16	R
S	>16/8	R	>16/8	R	>16	R	>16	R	>16	R
S	>16/8	R	>16/8	R	>16	R	<=1	S	>16	R
S	>16/8	R	>16/8	R	>16	R	>16	R	>16	R
S	>16/8	R	>16/8	R	>16	R	>16	R	>16	R
R	>16/8	R	>16/8	R	>16	R	<=1	S	>16	R
S	>16/8	R	>16/8	R	>16	R	<=1	S	16	I
S	>16/8	R	>16/8	R	>16	R	>16	R	>16	R
S	>16/8	R	>16/8	R	>16	R	<=1	S	>16	R
S	>16/8	R	>16/8	R	>16	R*	>16	ESBL	>16	R*
S	>16/8	R	>16/8	R	>16	R*	>16	ESBL	>16	R*
I	>16/8	R	>16/8	R	>16	R	>16	R	R	
I	>16/8	R	>16/8	R	>16	R*	>16	ESBL	R*	
S	>16/8	R	>16/8	R	>16	R	>16	R	R	
S	>16/8	R	>16/8	R	>16	R	>16	R	R	
R	>16/8	R	>16/8	R	>16	R	>16	R	R	
I	>16/8	R	>16/8	R	>16	R	>16	R	R	
R	>16/8	R	>16/8	R	>16	R	>16	R	R	
R	>16/8	R	>16/8	R	>16	R	>16	R	R	
R	>16/8	R	>16/8	R	>16	R	>16	R	R	
S	>16/8	R	>16/8	R	>16	R*	>16	ESBL	4	R*
S	>16/8	R	>16/8	R	>16	R	8	I	R	
S	>16/8	R	>16/8	R	>16	R	<=1	S	R	
R	>16/8	R	>16/8	R	>16	R	>16	R	R	
R	>16/8	R	>16/8	R	>16	R	>16	R	R	
S	>16/8	R	>16/8	R	>16	R*	>16	ESBL	R*	
S	>16/8	R	>16/8	R	>16	R	>16	R	R	
I	>16/8	R	>16/8	R	>16	R	>16	R	R	
R	>16/8	R	>16/8	R	>16	R	>16	R	R	
S	>16/8	R	>16/8	R	>16	R	>16	R	R	
S	>16/8	R	>16/8	R	>16	R	>16	R	R	
S	>16/8	R	>16/8	R	>16	R	>16	R	R	
S	>16/8	R	>16/8	R	>16	R	>16	R	R	
R	>16/8	R	>16/8	R	>16	R	>16	R	R	
R	>16/8	R	>16/8	R	>16	R	>16	R	R	
R	>16/8	R	>16/8	R	>16	R	>16	R	R	
R	>16/8	R	>16/8	R	>16	R	>16	R	R	
R	>16/8	R	>16/8	R	>16	R	>16	R	R	
S	>16/8	R	>16/8	R	>16	R*	>16	ESBL	R*	
S	>16/8	R	>16/8	R	>16	R*	>16	ESBL	R*	
R	>16/8	R	>16/8	R	>16	R	4	S	S	

R	>16/8	R	>16/8	R	>16	R	>16	R	R
R	>16/8	R	>16/8	R	>16	R	>16	R	R
R	>16/8	R	>16/8	R	>16	R	>16	R	R
I	>16/8	R	>16/8	R	>16	R*	16	ESBL	R*
R	>16/8	R	>16/8	R	>16	R	>16	R	R
S	>16/8	R	>16/8	R	>16	R*	>16	ESBL	R*
S	>16/8	R	>16/8	R	>16	R	>16	R	R
S	>16/8	R	>16/8	R	>16	R	>16	R	R
S	>16/8	R	>16/8	R	>16	R	>16	R	R
S	>16/8	R	>16/8	R	>16	R	>16	R	R
S	>16/8	R	>16/8	R	>16	R	>16	R	R
S	>16/8	R	>16/8	R	>16	R	<=1	S	R
S	>16/8	R	>16/8	R	>16	R*	>16	ESBL	R*
S	>16/8	R	>16/8	R	>16	R	<=1	S	S
S	>16/8	R	>16/8	R	>16	R	16	R	R
S	>16/8	R	>16/8	R	>16	R	>16	R	R
S	>16/8	R	>16/8	R	>16	R	>16	R	R
R	>16/8	R	>16/8	R	>16	R	>16	R	R
S	>16/8	R	>16/8	R	>16	R*	>16	ESBL	R*
S	>16/8	R	>16/8	R	>16	R*	>16	ESBL	R*
I	>16/8	R	>16/8	R	>16	R	>16	R	R
S	>16/8	R	>16/8	R	>16	R*	>16	ESBL	R*
R	>16/8	R	>16/8	R	>16	R*	>16	ESBL	R*
I	>16/8	R	>16/8	R	>16	R	>16	R	R
I	>16/8	R	>16/8	R	>16	R	>16	R	R
I	>16/8	R	>16/8	R	>16	R	16	R	S
S	>16/8	R	>16/8	R	>16	R*	>16	ESBL	R*
I	>16/8	R	>16/8	R	>16	R	>16	R	R
S	>16/8	R	>16/8	R	>16	R	>16	R	R
I	>16/8	R	>16/8	R	>16	R	>16	R	R
S	>16/8	R	>16/8	R	>16	R	>16	R	R
S	>16/8	R	>16/8	R	>16	R	>16	R	R
S	>16/8	R	>16/8	R	>16	R*	>16	ESBL	R*
S	>16/8	R	>16/8	R	>16	R	>16	R	R
R	>16/8	R	>16/8	R	>16	R	>16	R	R
R	>16/8	R	>16/8	R	>16	R	>16	R	R
R	>16/8	R	>16/8	R	>16	R	>16	R	R
S	>16/8	R	>16/8	R	>16	R*	>16	ESBL	R*
S	>16/8	R	>16/8	R	>16	R	>16	R	R
R	>16/8	R	>16/8	R	>16	R	>16	R	R
R	>16/8	R	>16/8	R	>16	R	>16	R	R
S	>16/8	R	>16/8	R	>16	R*	>16	ESBL	R*
S	>16/8	R	>16/8	R	>16	R*	>16	ESBL	R*
R	>16/8	R	>16/8	R	>16	R	>16	R	R
S	>16/8	R	>16/8	R	>16	R	>16	R	R
S	>16/8	R	>16/8	R	>16	R	>16	R	R
S	>16/8	R	>16/8	R	>16	R*	>16	ESBL	R*
R	>16/8	R	>16/8	R	>16	R	16	R	S
S	>16/8	R	<=8/4	S	>16	R*	<=1	R*	R*
S	>16/8	R	>16/8	R	>16	R	>16	R	R
S	>16/8	R	>16/8	R	>16	R	>16	R	R
S	>16/8	R	>16/8	R	>16	R*	>16	ESBL	R*
R	>16/8	R	>16/8	R	>16	R	>16	R	R
S	>16/8	R	>16/8	R	>16	R	>16	R	R
S	>16/8	R	>16/8	R	>16	R*	>16	ESBL	R*

S	>16/8	R	>16/8	R	>16	R*	>16	ESBL	R*	
S	>16/8	R	>16/8	R	>16	R*	>16	ESBL	R*	
S	>16/8	R	>16/8	R	>16	R	>16	R	R	
S	>16/8	R	>16/8	R	>16	R*	>16	ESBL	R*	
S	>16/8	R	>16/8	R	>16	R	<=1	S	R	
R	>16/8	R	>16/8	R	>16	R	16	R	R	
R	>16/8	R	>16/8	R	>16	R	>16	R	R	
S	>16/8	R	>16/8	R	>16	R	>16	R	R	
R	>16/8	R	>16/8	R	>16	R	>16	R	R	
S	>16/8	R	>16/8	R	>16	R	>16	R	R	
S	>16/8	R	>16/8	R	>16	R*	>16	ESBL	R*	
S	>16/8	R	>16/8	R	>16	R*	>16	ESBL	R*	
S	>16/8	R	>16/8	R	>16	R*	>16	ESBL	R*	
R	>16/8	R	>16/8	R	>16	R	>16	R	R	
S	>16/8	R	>16/8	R	>16	R*	>16	ESBL	R*	
S	>16/8	R	>16/8	R	>16	R*	>16	ESBL	R*	
S	>16/8	R	>16/8	R	>16	R*	>16	ESBL	R*	
S	>16/8	R	>16/8	R	>16	R*	>16	ESBL	R*	
R	>16/8	R	>16/8	R	>16	R	8	I	S	
S	>16/8	R	>16/8	R	>16	R*	>16	ESBL	16	R*
R	>16/8	R	>16/8	R	>16	R	16	R	>16	R
R	>16/8	R	>16/8	R	>16	R	>16	R	>16	R
S	>16/8	R	>16/8	R	>16	R	>16	R	>16	R
S	>16/8	R	>16/8	R	>16	R*	>16	ESBL	>16	R*
S	>16/8	R	>16/8	R	>16	R*	>16	ESBL	>16	R*
S	>16/8	R	>16/8	R	>16	R*	>16	ESBL	>16	R*
S	>16/8	R	>16/8	R	>16	R	>16	R	>16	R
S	>16/8	R	>16/8	R	>16	R*	>16	ESBL	>16	R*
S	>16/8	R	>16/8	R	>16	R	>16	R	>16	R
S	>16/8	R	>16/8	R	>16	R*	>16	ESBL	>16	R*
S	>16/8	R	>16/8	R	>16	R*	>16	ESBL	>16	R*
S	>16/8	R	>16/8	R	>16	R	>16	R	>16	R
S	>16/8	R	>16/8	R	>16	R	>16	R	>16	R
S	>16/8	R	>16/8	R	>16	R*	>16	ESBL	R*	
S	>16/8	R	>16/8	R	>16	R*	>16	ESBL	R*	
S	>16/8	R	>16/8	R	>16	R*	>16	ESBL	R*	
S	>16/8	R	>16/8	R	>16	R	>16	R	R	
R	>16/8	R	>16/8	R	>16	R	>16	R	R	
R	>16/8	R	>16/8	R	>16	R	>16	R	R	
S	>16/8	R	>16/8	R	>16	R*	>16	ESBL	R*	
S	>16/8	R	>16/8	R	>16	R	8	I	R	
S	>16/8	R	>16/8	R	>16	R*	>16	ESBL	R*	
R	>16/8	R	>16/8	R	>16	R	>16	R	R	
S	>16/8	R	>16/8	R	>16	R	>16	R	R	
S	>16/8	R	>16/8	R	>16	R*	8	ESBL	R*	
S	>16/8	R	>16/8	R	>16	R	>16	R	>16	R
S	>16/8	R	>16/8	R	>16	R	>16	R	>16	R
S	>16/8	R	>16/8	R	>16	R*	>16	ESBL	>16	R*
S	>16/8	R	<=8/4	S	>16	R	<=1	S	<=1	S
S	>16/8	R	>16/8	R	>16	R	>16	R	>16	R
S	>16/8	R	>16/8	R	>16	R	>16	R	>16	R
S	>16/8	R	>16/8	R	>16	R	>16	R	>16	R
S	>16/8	R	>16/8	R	>16	R	>16	R	>16	R
R	>16/8	R	>16/8	R	>16	R	>16	R	>16	R
R	>16/8	R	>16/8	R	>16	R	>16	R	>16	R

R	>16/8	R	>16/8	R	>16	R	>16	R	>16	R
R	>16/8	R	>16/8	R	>16	R	>16	R	>16	R
S	>16/8	R	>16/8	R	>16	R*	>16	ESBL	>16	R*
S	>16/8	R	>16/8	R	>16	R*	>16	ESBL	>16	R*
S	>16/8	R	>16/8	R	>16	R*	>16	ESBL	>16	R*
R	>16/8	R	>16/8	R	>16	R	>16	R	>16	R
S	>16/8	R	>16/8	R	>16	R*	>16	ESBL	>16	R*
S	>16/8	R	>16/8	R	>16	R*	>16	ESBL	>16	R*
S	>16/8	R	>16/8	R	>16	R	>16	R	>16	R
S	>16/8	R	>16/8	R	>16	R	>16	R	>16	R
S	>16/8	R	>16/8	R	>16	R*	>16	ESBL	>16	R*
R	>16/8	R	>16/8	R	>16	R	>16	R	>16	R
S	>16/8	R	>16/8	R	>16	R*	>16	ESBL	>16	R*
S	>16/8	R	>16/8	R	>16	R	>16	R	>16	R
S	>16/8	R	>16/8	R	>16	R*	>16	ESBL	>16	R*
R	>16/8	R	>16/8	R	>16	R	>16	R	>16	R
S	>16/8	R	>16/8	R	>16	R	>16	R	>16	R
S	>16/8	R	>16/8	R	>16	R*	>16	ESBL	>16	R*
S	<=8/4	S	<=8/4	S	>16	R	<=1	S	<=1	S
S	>16/8	R	>16/8	R	>16	R*	>16	ESBL	>16	R*
I	>16/8	R	>16/8	R	>16	R	>16	R	>16	R
S	>16/8	R	>16/8	R	>16	R	>16	R	>16	R
S	>16/8	R	>16/8	R	>16	R	4	S	>16	R
S	>16/8	R	>16/8	R	>16	R*	>16	ESBL	>16	R*
S	>16/8	R	>16/8	R	>16	R*	>16	ESBL	>16	R*
S	>16/8	R	>16/8	R	>16	R	>16	R	>16	R
S	>16/8	R	>16/8	R	>16	R	>16	R	>16	R
S	>16/8	R	>16/8	R	>16	R*	>16	ESBL	>16	R*
S	>16/8	R	>16/8	R	>16	R*	>16	ESBL	>16	R*
S	>16/8	R	>16/8	R	>16	R	>16	R	>16	R
S	>16/8	R	>16/8	R	>16	R*	>16	ESBL	>16	R*
R	>16/8	R	>16/8	R	>16	R	>16	R	>16	R
S	>16/8	R	>16/8	R	>16	R	>16	R	>16	R
S	>16/8	R	>16/8	R	>16	R	>16	R	>16	R
S	>16/8	R	>16/8	R	>16	R*	>16	ESBL	>16	R*
S	>16/8	R	>16/8	R	>16	R*	>16	ESBL	>16	R*
S	>16/8	R	>16/8	R	>16	R*	>16	ESBL	>16	R*
S	>16/8	R	>16/8	R	>16	R	>16	R	>16	R
S	>16/8	R	>16/8	R	>16	R	>16	R	>16	R
R	>16/8	R	>16/8	R	>16	R	<=1	S	>16	R
S	>16/8	R	>16/8	R	>16	R	>16	R	>16	R
S	>16/8	R	>16/8	R	>16	R	>16	R	>16	R
S	>16/8	R	>16/8	R	>16	R	>16	R	>16	R
R	>16/8	R	>16/8	R	>16	R*	>16	ESBL	2	R*
S	>16/8	R	>16/8	R	>16	R*	>16	ESBL	>16	R*
S	>16/8	R	>16/8	R	>16	R	<=1	S	4	S
R	>16/8	R	>16/8	R	>16	R	>16	R	>16	R
S	>16/8	R	>16/8	R	>16	R	>16	R	>16	R
I	>16/8	R	>16/8	R	>16	R	>16	R	>16	R
S	>16/8	R	>16/8	R	>16	R*	>16	ESBL	>16	R*
S	>16/8	R	>16/8	R	>16	R	>16	R	>16	R
S	>16/8	R	>16/8	R	>16	R	16	R	>16	R
S	>16/8	R	>16/8	R	>16	R	>16	R	>16	R
S	>16/8	R	>16/8	R	>16	R	>16	R	>16	R
R	>16/8	R	>16/8	R	>16	R*	<=1	R*	<=1	R*
S	>16/8	R	>16/8	R	>16	R	>16	R	>16	R

S	>16/8	R	>16/8	R	>16	R	>16	R	>16	R
S	>16/8	R	>16/8	R	>16	R*	>16	ESBL	>16	R*
S	>16/8	R	>16/8	R	>16	R*	>16	ESBL	>16	R*
S	>16/8	R	>16/8	R	>16	R*	>16	ESBL	>16	R*
S	>16/8	R	>16/8	R	>16	R	<=1	S	>16	R
S	>16/8	R	>16/8	R	>16	R*	>16	ESBL	>16	R*
R	>16/8	R	>16/8	R	>16	R	>16	R	>16	R
S	>16/8	R	>16/8	R	>16	R*	>16	ESBL	>16	R*
S	>16/8	R	16/8	I	>16	R	<=1	S	<=1	S
S	>16/8	R	>16/8	R	>16	R	>16	R	>16	R
S	>16/8	R	>16/8	R	>16	R	>16	R	>16	R
I	>16/8	R	>16/8	R	>16	R	>16	R	>16	R
S	>16/8	R	>16/8	R	>16	R*	>16	ESBL	>16	R*
S	>16/8	R	>16/8	R	>16	R	>16	R	>16	R
S	>16/8	R	>16/8	R	>16	R	>16	R	>16	R
R	>16/8	R	>16/8	R	>16	R	>16	R	>16	R
S	>16/8	R	>16/8	R	>16	R*	>16	ESBL	>16	R*
S	>16/8	R	>16/8	R	>16	R*	>16	ESBL	>16	R*
S	>16/8	R	>16/8	R	>16	R*	>16	ESBL	>16	R*
S	>16/8	R	>16/8	R	>16	R	>16	R	>16	R
S	>16/8	R	>16/8	R	>16	R*	>16	ESBL	16	R*
R	>16/8	R	>16/8	R	>16	R	>16	R	>16	R
S	>16/8	R	>16/8	R	>16	R*	>16	ESBL	>16	R*
S	>16/8	R	>16/8	R	>16	R	>16	R	>16	R
S	>16/8	R	>16/8	R	>16	R	>16	R	>16	R
S	>16/8	R	>16/8	R	>16	R*	>16	ESBL	>16	R*
R	>16/8	R	>16/8	R	>16	R	<=1	S	>16	R
S	>16/8	R	>16/8	R	>16	R	>16	R	>16	R
S	>16/8	R	>16/8	R	>16	R	>16	R	>16	R
S	>16/8	R	>16/8	R	>16	R	>16	R	>16	R
S	>16/8	R	>16/8	R	>16	R	>16	R	>16	R
R	>16/8	R	>16/8	R	>16	R	16	R	>16	R
R	>16/8	R	>16/8	R	>16	R	>16	R	>16	R
S	>16/8	R	>16/8	R	>16	R	>16	R	>16	R
R	>16/8	R	>16/8	R	>16	R	>16	R	>16	R
R	>16/8	R	>16/8	R	>16	R	>16	R	>16	R
S	>16/8	R	>16/8	R	>16	R	>16	R	>16	R
S	>16/8	R	>16/8	R	>16	R	>16	R	>16	R
S	>16/8	R	>16/8	R	>16	R	>16	R	>16	R
S	>16/8	R	>16/8	R	>16	R	>16	R	>16	R
S	>16/8	R	>16/8	R	>16	R	>16	R	>16	R
S	>16/8	R	>16/8	R	>16	R*	>16	ESBL	>16	R*

S	>16/8	R	>16/8	R	>16	R*	>16	ESBL	>16	R*

S	>16/8	R	>16/8	R	>16	R	16	R	>16	R
S	>16/8	R	>16/8	R	>16	R	<=1	S	>16	R
S	>16/8	R	>16/8	R	>16	R*	>16	ESBL	>16	R*
S	>16/8	R	>16/8	R	>16	R*	>16	ESBL	>16	R*
S	>16/8	R	>16/8	R	>16	R*	>16	ESBL	>16	R*
R	>16/8	R	>16/8	R	>16	R	>16	R	>16	R
S	>16/8	R	>16/8	R	>16	R	>16	R	>16	R
S	>16/8	R	>16/8	R	>16	R	>16	R	>16	R
R	>16/8	R	>16/8	R	>16	R	<=1	S	>16	R
I	>16	R	>16	R	>16	R*	>16	ESBL	>16	R*
R	>16/8	R	>16/8	R	>16	R	4	S	>16	R
R	>16/8	R	>16/8	R	>16	R	>16	R	>16	R
S	>16/8	R	>16/8	R	>16	R*	>16	ESBL	>16	R*
S	>16/8	R	>16/8	R	>16	R*	>16	ESBL	>16	R*
S	>16/8	R	>16/8	R	>16	R*	>16	ESBL	>16	R*
S	>16/8	R	>16/8	R	>16	R*	>16	ESBL	>16	R*
S	>16/8	R	>16/8	R	>16	R	>16	R	>16	R
S	>16/8	R	>16/8	R	>16	R*	>16	ESBL	>16	R*
S	>16/8	R	>16/8	R	>16	R*	>16	ESBL	>16	R*
R	>16/8	R	>16/8	R	>16	R	>16	R	>16	R
S	>16/8	R	>16/8	R	>16	R*	>16	ESBL	>16	R*
S	>16/8	R	>16/8	R	>16	R	>16	R	>16	R
S	>16/8	R	>16/8	R	>16	R	>16	R	>16	R

R	>16/8	R	>16/8	R	>16	R	>16	R	>16	R
R	>16/8	R	>16/8	R	>16	R	>16	R	>16	R
R	>16/8	R	>16/8	R	>16	R	>16	R	>16	R
R	>16/8	R	>16/8	R	>16	R	>16	R	>16	R
S	>16/8	R	>16/8	R	>16	R	>16	R	>16	R
R	>16/8	R	>16/8	R	>16	R	>16	R	>16	R
R	>16/8	R	>16/8	R	>16	R	>16	R	>16	R
R	>16/8	R	>16/8	R	>16	R	>16	R	>16	R
R	>16/8	R	>16/8	R	>16	R	>16	R	>16	R
R	>16/8	R	>16/8	R	>16	R	>16	R	>16	R
R	>16/8	R	>16/8	R	>16	R	>16	R	>16	R
R	>16/8	R	>16/8	R	>16	R	>16	R	>16	R
R	>16/8	R	>16/8	R	>16	R	>16	R	>16	R
S	>16/8	R	>16/8	R	>16	R	>16	R	>16	R
R	>16/8	R	>16/8	R	>16	R	>16	R	>16	R
R	>16/8	R	>16/8	R	>16	R	>16	R	>16	R
R	>16/8	R	>16/8	R	>16	R	>16	R	>16	R
S	>16/8	R	>16/8	R	>16	R*	>16	ESBL	>16	R*
I	>16/8	R	>16/8	R	>16	R*	8	ESBL	>16	R*
S	>16/8	R	>16/8	R	>16	R	>16	R	>16	R
R	>16/8	R	>16/8	R	>16	R	>16	R	>16	R
S	>16/8	R	>16/8	R	>16	R*	>16	ESBL	>16	R*
S	>16/8	R	>16/8	R	>16	R*	>16	ESBL	>16	R*
S	>16/8	R	>16/8	R	>16	R*	>16	ESBL	>16	R*
S	>16/8	R	>16/8	R	>16	R*	>16	ESBL	>16	R*
R	>16/8	R	>16/8	R	>16	R	>16	R	>16	R
R	>16/8	R	>16/8	R	>16	R	>16	R	>16	R
S	>16/8	R	>16/8	R	>16	R*	>16	ESBL	>16	R*
S	>16/8	R	>16/8	R	>16	R	>16	R	>16	R
S	>16/8	R	>16/8	R	>16	R	>16	R	>16	R
S	>16/8	R	>16/8	R	>16	R	>16	R	>16	R
S	>16/8	R	>16/8	R	>16	R*	>16	ESBL	>16	R*
R	>16/8	R	>16/8	R	>16	R	4	S	16	I
S	>16/8	R	>16/8	R	>16	R*	>16	ESBL	>16	R*
S	>16/8	R	>16/8	R	>16	R	>16	R	>16	R
S	>16/8	R	>16/8	R	>16	R	>16	R	>16	R
S	>16/8	R	>16/8	R	>16	R	>16	R	>16	R
R	>16/8	R	>16/8	R	>16	R	>16	R	>16	R
S	>16/8	R	>16/8	R	>16	R	>16	R	>16	R
S	>16/8	R	>16/8	R	>16	R*	>16	ESBL	>16	R*
R	>16/8	R	>16/8	R	>16	R	>16	R	>16	R
S	>16/8	R	>16/8	R	>16	R*	>16	ESBL	>16	R*
S	>16/8	R	>16/8	R	>16	R	>16	R	>16	R
S	>16/8	R	>16/8	R	>16	R*	>16	ESBL	>16	R*
S	>16/8	R	>16/8	R	>16	R*	>16	ESBL	>16	R*
S	>16/8	R	>16/8	R	>16	R*	>16	ESBL	>16	R*
S	>16/8	R	>16/8	R	>16	R	>16	R	>16	R
S	>16/8	R	>16/8	R	>16	R*	>16	ESBL	>16	R*
R	>16/8	R	>16/8	R	>16	R	>16	R	>16	R
R	>16/8	R	>16/8	R	>16	R	>16	R	>16	R
S	>16/8	R	>16/8	R	>16	R	>16	R	>16	R
S	>16/8	R	>16/8	R	>16	R	<=1	S	>16	R
S	>16/8	R	>16/8	R	>16	R	8	I	4	S
S	>16/8	R	>16/8	R	>16	R	4	S	>16	R
S	>16/8	R	>16/8	R	>16	R*	>16	ESBL	>16	R*
S	>16/8	R	>16/8	R	>16	R*	8	ESBL	>16	R*

R	>16/8	R	>16/8	R	<=8	R*	8	I	16	I
I	>16/8	R	>16/8	R	>16	R*	<=1	R*	2	R*
S	>16/8	R	>16/8	R	>16	R	<=1	S	>16	R
S	>16/8	R	>16/8	R	>16	R*	>16	ESBL	>16	R*
S	>16/8	R	>16/8	R	>16	R*	>16	ESBL	>16	R*
R	>16/8	R	>16/8	R	>16	R	>16	R	>16	R
R	>16/8	R	>16/8	R	>16	R	>16	R	>16	R
R	>16/8	R	>16/8	R	>16	R	>16	R	>16	R
S	>16/8	R	>16/8	R	>16	R	>16	R	>16	R
R	>16/8	R	>16/8	R	>16	R	>16	R	>16	R
S	>16/8	R	>16/8	R	>16	R*	>16	ESBL	>16	R*
S	>16/8	R	>16/8	R	>16	R	>16	R	>16	R
S	>16/8	R	>16/8	R	>16	R*	>16	ESBL	>16	R*
S	>16/8	R	>16/8	R	>16	R	<=1	S	4	S
S	>16/8	R	>16/8	R	>16	R	<=1	S	<=1	S
S	>16/8	R	>16/8	R	>16	R*	<=1	R*	>16	R*
S	>16/8	R	>16/8	R	>16	R*	>16	ESBL	>16	R*
S	>16/8	R	>16/8	R	>16	R*	16	ESBL	16	R*
S	>16/8	R	>16/8	R	>16	R	>16	R	>16	R
S	>16/8	R	>16/8	R	>16	R*	>16	ESBL	>16	R*
S	>16/8	R	>16/8	R	>16	R	>16	R	>16	R
R	>16/8	R	>16/8	R	>16	R	>16	R	>16	R
R	>16/8	R	>16/8	R	>16	R	>16	R	>16	R
S	>16/8	R	>16/8	R	>16	R*	>16	ESBL	>16	R*
S	>16/8	R	>16/8	R	>16	R	>16	R	>16	R
S	>16/8	R	>16/8	R	>16	R	>16	R	>16	R
S	>16/8	R	>16/8	R	>16	R	>16	R	>16	R
S	>16/8	R	>16/8	R	>16	R*	>16	ESBL	>16	R*
S	>16/8	R	>16/8	R	>16	R*	>16	ESBL	>16	R*
R	>16/8	R	>16/8	R	>16	R	>16	R	>16	R
S	>16/8	R	>16/8	R	>16	R	<=1	S	<=1	S
S	>16/8	R	>16/8	R	>16	R*	>16	ESBL	>16	R*
R	>16/8	R	>16/8	R	>16	R	>16	R	>16	R
S	>16/8	R	>16/8	R	>16	R*	>16	ESBL	>16	R*
S	>16/8	R	>16/8	R	>16	R*	>16	ESBL	>16	R*
S	>16/8	R	>16/8	R	>16	R*	>16	ESBL	>16	R*
S	>16/8	R	>16/8	R	>16	R*	>16	ESBL	>16	R*
S	>16/8	R	>16/8	R	>16	R	>16	R	>16	R
S	>16/8	R	>16/8	R	>16	R	>16	R	>16	R
R	>16/8	R	>16/8	R	>16	R	>16	R	>16	R
R	>16/8	R	>16/8	R	>16	R	>16	R	>16	R
S	>16/8	R	>16/8	R	>16	R*	>16	ESBL	>16	R*
S	>16/8	R	>16/8	R	>16	R*	>16	ESBL	16	R*
S	>16/8	R	>16/8	R	>16	R	>16	R	>16	R
S	>16/8	R	>16/8	R	>16	R	>16	R	>16	R
S	>16/8	R	>16/8	R	>16	R	<=1	S	<=1	S
S	>16/8	R	>16/8	R	>16	R	>16	R	>16	R
S	>16/8	R	>16/8	R	>16	R*	>16	ESBL	>16	R*
S	>16/8	R	>16/8	R	>16	R	>16	R	>16	R
R	>16/8	R	>16/8	R	>16	R	>16	R	>16	R
S	>16/8	R	>16/8	R	>16	R	>16	R	>16	R
R	>16/8	R	>16/8	R	>16	R	>16	R	>16	R
S	>16/8	R	>16/8	R	>16	R*	>16	ESBL	>16	R*
S	>16/8	R	>16/8	R	>16	R	>16	R	>16	R
S	>16/8	R	>16/8	R	>16	R	>16	R	>16	R
R	>16/8	R	>16/8	R	>16	R	>16	R	>16	R

S	>16/8	R	>16/8	R	>16	R	>16	R	>16	R
R	>16/8	R	>16/8	R	>16	R	>16	R	>16	R
S	>16/8	R	>16/8	R	>16	R*	8	ESBL	16	R*
R	>16/8	R	>16/8	R	>16	R	8	I	2	S
S	>16/8	R	>16/8	R	>16	R*	>16	ESBL	>16	R*
R	>16/8	R	>16/8	R	>16	R	>16	R	>16	R
R	>16/8	R	>16/8	R	>16	R	>16	R	>16	R
S	>16/8	R	>16/8	R	>16	R*	>16	ESBL	>16	R*
R	>16/8	R	>16/8	R	>16	R	>16	R	>16	R
S	>16/8	R	>16/8	R	>16	R*	>16	ESBL	>16	R*
S	>16/8	R	>16/8	R	>16	R	>16	R	>16	R
S	>16/8	R	>16/8	R	>16	R	>16	R	>16	R
R	>16/8	R	>16/8	R	>16	R	>16	R	>16	R
R	>16/8	R	>16/8	R	>16	R	>16	R	>16	R
R	>16/8	R	>16/8	R	>16	R	>16	R	>16	R
R	>16/8	R	>16/8	R	>16	R	>16	R	>16	R
S	>16/8	R	>16/8	R	>16	R*	>16	ESBL	>16	R*
S	>16/8	R	>16/8	R	>16	R*	>16	ESBL	>16	R*
S	>16/8	R	>16/8	R	>16	R*	>16	ESBL	>16	R*
R	>16/8	R	>16/8	R	>16	R	>16	R	>16	R
S	>16/8	R	>16/8	R	>16	R*	>16	ESBL	>16	R*
S	>16/8	R	>16/8	R	>16	R	>16	R	>16	R
S	>16/8	R	>16/8	R	>16	R*	>16	ESBL	>16	R*
R	>16/8	R	>16/8	R	>16	R	>16	R	>16	R
S	>16/8	R	>16/8	R	>16	R	>16	R	>16	R
S	>16/8	R	>16/8	R	>16	R	>16	R	>16	R
R	>16/8	R	>16/8	R	>16	R	>16	R	>16	R
R	>16/8	R	>16/8	R	>16	R	>16	R	>16	R

cftmic	cft	crmmic	crm	cipmic	cip	colmic	col	dormic	dor	etpmic	etp
>32	R	>16	R	>2	R	>4	R	2	I	>1	R
>32	R	>16	R	>2	R	<=2	S	2	I	>1	R
>32	R	>16	R	>2	R	<=2	S	>4	R	>1	R
>32	R	>16	R	<=0.5	S	<=2	S	>4	R	>1	R
>32	R	>16	R	>2	R	<=2	S	4	R	>1	R
>32	R	>16	R	<=0.5	S	<=2	S	4	R	>1	R
>32	R	>16	R	>2	R	<=2	S	2	I	1	I
>32	R	>16	R	>2	R	<=2	S	>4	R	>1	R
>32	ESBL	>16	R*	<=0.5	S	32		2	I	>1	R
>32	R	>16	R	>2	R	<=2	S	2	I	>1	R
>32	R	>16	R	2	I	<=2	S	<=1	S	>1	R
>32	ESBL	>16	R*	>2	R	<=2	S	<=1	S	<=0.5	S
>32	R	>16	R	>2	R	16		>4	R	>1	R
>32	R	>16	R	>2	R	16		>4	R	>1	R
>32	R	>16	R	>2	R	<=2	S	<=1	S	>1	R
>32	R	>16	R	>2	R	1		2	I	>1	R
>32	R	>16	R	>2	R	<=2	S	<=1	S	>1	R
>32	R	>16	R	>2	R	<=2	S	>4	R	>1	R
>32	R	>16	R	>2	R	32		>4	R	>1	R
>32	R	>16	R	>2	R	16		>4	R	>1	R
>16	R	>16	R	>2	R	16		>4	R	>1	R
>32	R	>16	R	<=0.5	S	<=2	S	>4	R	>1	R
>32	R	>16	R	>2	R	<=2	S	>4	R	>1	R
>32	R	>16	R	>2	R	<=2	S	>4	R	>1	R
>32	R	>16	R	>2	R	<=2	S	>4	R	>1	R
>32	R	>16	R	>2	R	<=2	S	<=1	S	>1	R
>32	R	>16	R	>2	R	<=2	S	4	R	>1	R
>32	R	>16	R	>2	R	<=2	S	>4	R	>1	R
>32	R	>16	R	>2	R	16		>4	R	>1	R
>32	R	>16	R	2	I	<=2	S	<=1	S	<=0.5	S
>32	R	>16	R	>2	R	<=2	S	>4	R	>1	R
<=1	S	8	S	2	I	<=2	S	<=1	S	>1	R
>32	ESBL	>16	R*	>2	R	<=2	S	<=1	S	1	I
>32	ESBL	>16	R*	>2	R	<=2	S	<=1	S	>1	R
>32	ESBL	>16	R*	>2	R	<=2	S	<=1	S	1	I
>32	R	>16	R	>2	R	2		>4	R	>1	R
>32	R	>16	R	>2	R	<=2	S	>4	R	>1	R
>32	R	>16	R	>2	R	<=2	S	>4	R	>1	R
>32	R	>16	R	>2	R	16		>4	R	>1	R
>32	R	>16	R	1	S	<=2	S	>4	R	>1	R
>32	R	>16	R	>2	R	<=2	S	>4	R	>1	R
>32	R	>16	R	<=0.5	S	<=2	S	>4	R	>1	R
>32	R	>16	R	2	I	<=2	S	2	I	1	I
>32	R	>16	R	>2	R	<=2	S	2	I	<=0.5	S
>32	R	>16	R	2	I	<=2	S	<=0.1	S	<=0.5	S
>32	R	>16	R	<=0.5	S	<=2	S	>4	R	>1	R
<=1	S	8	S	>2	R	<=2	S	<=1	S	>1	R
>32	R	>16	R	>2	R	16		>4	R	>1	R
>32	R	>16	R	>2	R	<=2	S	<=1	S	>1	R
>32	R	>16	R	>2	R	8		>4	R	>1	R
>32	ESBL	>16	R*	>2	R	<=2	S	4	R	1	I

>32	ESBL	>16	R*	>2	R	1		<=1	S	>1	R
<=1	S	>16	R	>2	R	>4	R	<=1	S	>1	R
>32	ESBL	>16	R*	>2	R	1		<=1	S	<=0.5	S
>32	ESBL	>16	R*	>2	R	<=2	S	<=1	S	<=0.5	S
>32	ESBL	>16	R*	>2	R	1		<=1	S	<=0.5	S
<=1	S	8	S	1	S	<=2	S	<=1	S	1	I
>32	R	>16	R	>2	R	<=2	S	<=1	S	>1	R
>32	R	>16	R	>2	R	16		>4	R	>1	R
>32	R	>16	R	<=0.5	S	>4	R	>4	R	>1	R
>32	R	>16	R	>2	R	<=2	S	>4	R	>1	R
>32	R	>16	R	>2	R	<=2	S	<=1	S	>1	R
>32	R	>16	R	>2	R	0.5		>4	R	>1	R
>32	R	>16	R	>2	R	<=2	S	2	I	>1	R
>32	R	>16	R	>2	R	32		>4	R	>1	R
8	I	>16	R	<=0.5	S	16		<=1	S	>1	R
>32	ESBL	>16	R*	>2	R	1		<=1	S	>1	R
>32	ESBL	>16	R*	>2	R	<=2	S	<=1	S	1	I
>32	R	>16	R	>2	R	<=2	S	4	R	>1	R
>32	ESBL	>16	R	>2	R	<=2	S	<=1	S	1	I
>32	ESBL	>16	R*	>2	R	<=2	S	<=1	S	>1	R
2	I	>16	R	>2	R	<=2	S	<=1	S	>1	R
>32	R	>16	R	>2	R	128		>4	R	>1	R
>32	R	>16	R	>2	R	<=2	S	>4	R	>1	R
>32	ESBL	>16	R*	>2	R	<=2	S	<=1	S	<=0.5	S
>32	R	>16	R	2	I	<=2	S	4	R	>1	R
>32	R	>16	R	>2	R	<=2	S	>4	R	>1	R
>32	R	>16	R	>2	R	<=2	S	>4	R	>1	R
>32	ESBL	>16	R*	>2	R	<=2	S	<=1	S	>1	R
>32	R	>16	R	>2	R	0.5		4	R	>1	R
>32	R	>16	R	>2	R	32		2	I	>1	R
>32	R	>16	R	>2	R	1		>4	R	>1	R
>32	R	>16	R	>2	R	1		>4	R	>1	R
>32	R	>16	R	>2	R	<=2	S	4	R	>1	R
<=1	S	<=4	S	<=0.5	S	<=2	S	<=1	S	<=0.5	S

>32	ESBL	>16	R*	>2	R	<=2	S	<=1	S	<=0.5	S
>32	R	>16	R	>2	R	>4	R	>4	R	>1	R
>32	R	>16	R	>2	R	1		>4	R	>1	R
>32	R	>16	R	>2	R	1		>4	R	>1	R
>32	R	>16	R	>2	R	>128		>4	R	>1	R
>32	R	>16	R	>2	R	<=2	S	>4	R	>1	R
>32	R	>16	R	>2	R	1		>4	R	>1	R
>32	R	>16	R	>2	R	1		>4	R	>1	R
>32	ESBL	>16	R*	1	S	<=2	S	<=1	S	1	I
>32	R	>16	R	>2	R	<=2	S	2	I	>1	R
>32	ESBL	>16	R*	>2	R	<=2	S	<=1	S	>1	R
>32	R	>16	R	1	S	<=2	S	>4	R	>1	R
>32	R	>16	R	>2	R	0.5		<=1	S	1	I
>32	R	>16	R	>2	R	<=2	S	<=1	S	>1	R
>32	R	>16	R	>2	R	32		4	R	>1	R
>32	R	>16	R	<=0.5	S	<=2	S	<=1	S	<=0.5	S
<=1	S	<=4	S	<=0.5	S	<=2	S	<=1	S	<=0.5	S
>32	ESBL	>16	R*	>2	R	<=2	S	<=1	S	>1	R
>32	R	>16	R	>2	R	1		4	R	>1	R
>32	R	>16	R	>2	R	1		>4	R	>1	R
>32	R	>16	R	>2	R	1		>4	R	>1	R
>32	R	>16	R	>2	R	>4	R	>4	R	>1	R
>32	R	>16	R	>2	R	0.5		4	R	>1	R
>32	R	>16	R	>2	R	<=2	S	>4	R	>1	R
>32	R	>16	R	>2	R	<=2	S	>4	R	>1	R
>32	ESBL	>16	R*	>2	R	<=2	S	2	I	>1	R
>32	R	>16	R*	>2	R	<=2	S	2	I	>1	R
>32	R	>16	R	>2	R	<=2	S	4	R	>1	R
>32	R	>16	R	>2	R	1		<=1	S	>1	R
2	I	>16	R	<=0.5	S	0.5		<=1	S	>1	R
>32	R	>16	R	>2	R	0.5		2	I	>1	R
>32	R	>16	R	>2	R	>128		2	I	>1	R
>32	R	>16	R	>2	R	1		2	I	>1	R
>32	R	>16	R	>2	R	1		<=1	S	>1	R
>32	ESBL	>16	R*	>2	R	<=2	S	<=1	S	1	I
>32	ESBL	>16	R*	>2	R	<=2	S	<=1	S	>1	R
>32	R	>16	R	>2	R	<=2	S	<=1	S	>1	R
>32	R	>16	R	2	I	<=2	S	<=1	S	1	I
>32	R	>16	R	>2	R	<=2	S	>4	R	>1	R
>32	R	>16	R	>2	R	<=2	S	>4	R	>1	R
>32	R	>16	R	>2	R	<=2	S	>4	R	>1	R
>32	R	>16	R	>2	R	<=2	S	>4	R	>1	R
<=1	S	8	S	1	S	<=2	S	<=1	S	1	I
>32	ESBL	>16	R*	>2	R	<=2	S	<=1	S	<=0.5	S
>32	R	>16	R	>2	R	<=2	S	>4	R	>1	R
>32	ESBL	>16	R*	>2	R	<=2	S	<=1	S	<=0.5	S
<=1	S	8	S	>2	R	<=2	S	<=1	S	>1	R
>32	R	>16	R	1	S	<=2	S	2	I	1	I
>32	R	>16	R	>2	R	1		>4	R	>1	R
>32	R	>16	R	2	I	<=2	S	4	R	1	I

>32	R	>16	R	>2	R	<=2	S	<=1	S	>1	R
>32	ESBL	>16	R*	>2	R	<=2	S	<=1	S	>1	R
>32	R	>16	R	1	S	<=2	S	>4	R	>1	R
>32	R	>16	R	>2	R	<=2	S	<=1	S	>1	R
>32	R	>16	R	>2	R	<=2	S	>4	R	>1	R
>32	R	>16	R	2	I	1		2	I	>1	R
>32	R	>16	R	>2	R	<=2	S	<=1	S	>1	R
>32	R	>16	R	>2	R	<=2	S	>4	R	>1	R
>32	ESBL	>16	R*	>2	R	<=2	S	2	I	>1	R
>32	R	>16	R	>2	R	<=2	S	>4	R	>1	R
>32	ESBL	>16	R*	>2	R	<=2	S	<=1	S	>1	R
>32	R	>16	R	>2	R	<=2	S	>4	R	>1	R
>32	R	>16	R	>2	R	<=2	S	>4	R	>1	R
2	I	>16	R	2	I	<=2	S	4	R	>1	R
>32	R	>16	R	>2	R	<=2	S	>4	R	>1	R
>32	ESBL	>16	R*	>2	R	<=2	S	<=1	S	>1	R
>32	ESBL	>16	R*	<=0.5	S	<=2	S	<=1	S	<=0.5	S
>32	R	>16	R	>2	R	<=2	S	>4	R	>1	R
>32	R	>16	R	2	I	>4	R	>4	R	>1	R
>32	R	>16	R	2	I	<=2	S	<=1	S	>1	R
>32	R	>16	R	>2	R	<=2	S	>4	R	>1	R
>32	R	>16	R	>2	R	<=2	S	<=1	S	>1	R
>32	R	>16	R	>2	R	32		>4	R	>1	R
<=1	S	8	S	2	I	<=2	S	<=1	S	>1	R
>32	ESBL	>16	R*	<=0.5	S	<=2	S	<=1	S	>1	R
>32	ESBL	>16	R*	>2	R	<=2	S	<=1	S	<=0.5	S
>32	R	>16	R	>2	R	2		>4	R	>1	R
>32	R	>16	R	>2	R	<=2	S	>4	R	>1	R
>32	R	>16	R	>2	R	8		>4	R	>1	R

>32	R	>16	R	>2	R	<=2	S	>4	R	>1	R
>32	R	>16	R	1	S	<=2	S	>4	R	>1	R
>32	ESBL	>16	R*	>2	R	<=2	S	<=1	S	<=0.5	S
<=1	S	>16	R	<=0.5	S	16		<=1	S	<=0.5	S
>32	ESBL	>16	R*	2	I	<=2	S	<=1	S	<=0.5	S

>32	R	>16	R	>2	R	<=2	S	>4	R	>1	R
>32	R	>16	R	>2	R	<=2	S	2	I	1	I
<=1	S	<=4	S	>2	R	<=2	S	<=1	S	<=0.5	S
>32	R	>16	R	>2	R	<=2	S	4	R	>1	R
>32	ESBL	>16	R*	>2	R	<=2	S	<=1	S	>1	R
>32	R	>16	R	>2	R	<=2	S	<=1	S	>1	R
>32	ESBL	>16	R*	>2	R	<=2	S	<=1	S	1	I
>32	ESBL	>16	R*	>2	R	<=2	S	<=1	S	1	I
>32	R	>16	R	<=0.5	S	<=2	S	>4	R	>1	R
>32	R	>16	R	2	I	<=2	S	<=1	S	>1	R
>32	R	>16	R	>2	R	8		>4	R	>1	R
>32	R	>16	R	2	I	<=2	S	<=1	S	1	I
>32	ESBL	>16	R*	>2	R	<=2	S	<=1	S	<=0.5	S
>32	R	>16	R	>2	R	<=2	S	>4	R	>1	R

>32	R	>16	R	>2	R	<=2	S	>4	R	>1	R
>32	R	>16	R	>2	R	<=2	S	>4	R	>1	R
>32	R	>16	R	>2	R	<=2	S	>4	R	>1	R
>32	R	>16	R	>2	R	<=2	S	>4	R	>1	R
<=1	S	8	S	>2	R	<=2	S	<=1	S	>1	R
>32	ESBL	>16	R*	>2	R	<=2	S	<=1	S	1	I
>32	ESBL	>16	R*	>2	R	<=2	S	<=1	S	1	I
>32	ESBL	>16	R*	>2	R	<=2	S	<=1	S	<=0.5	S
>32	R	>16	R	>2	R	<=2	S	>4	R	>1	R
>32	R	>16	R	>2	R	<=2	S	>4	R	>1	R
>32	R	>16	R	>2	R	<=2	S	>4	R	>1	R
>32	R	>16	R	>2	R	<=2	S	>4	R	>1	R
>32	R	>16	R	>2	R	<=2	S	>4	R	>1	R
>32	R	>16	R	>2	R	<=2	S	>4	R	>1	R
>32	ESBL	>16	R*	>2	R	<=2	S	<=1	S	<=0.5	S
>32	ESBL	>16	R*	>2	R	<=2	S	<=1	S	1	I
>32	ESBL	>16	R*	>2	R	1		<=1	S	>1	R
>32	R	>16	R	>2	R	0.5		<=1	S	>1	R
>32	ESBL	>16	R*	>2	R	<=2	S	>4	R	>1	R
>32	R	>16	R	2	I	1		>4	R	>1	R
>32	R	>16	R	>2	R	<=2	S	<=1	S	>1	R
>32	R	>16	R	>2	R	>4	R	>4	R	>1	R
>32	ESBL	>16	R*	2	I	<=2	S	<=1	S	>1	R
>32	R	>16	R	>2	R	<=2	S	>4	R	>1	R
>32	ESBL	>16	R*	>2	R	<=2	S	<=1	S	<=0.5	S
>32	R	>16	R	>2	R	<=2	S	<=1	S	>1	R
>32	R	>16	R	>2	R	<=2	S	2	I	>1	R
>32	ESBL	>16	R*	>2	R	<=2	S	<=1	S	>1	R
>32	R	>16	R	>2	R	<=2	S	>4	R	>1	R
>32	R	>16	R	>2	R	<=2	S	>4	R	>1	R
>32	ESBL	>16	R*	>2	R	<=2	S	<=1	S	<=0.5	S
>32	R	>16	R	>2	R	8		>4	R	>1	R
>32	R	>16	R	>2	R	16		>4	R	>1	R
>32	R	>16	R	<=0.5	S	<=2	S	>4	R	>1	R
>32	R	>16	R	<=0.5	S	<=2	S	>4	R	>1	R
>32	ESBL	>16	R*	>2	R	<=2	S	4	R	>1	R
>32	R	>16	R	>2	R	<=2	S	>4	R	>1	R
>32	R	>16	R	>2	R	<=2	S	>4	R	>1	R
>32	ESBL	>16	R*	2	I	<=2	S	<=1	S	1	I
>32	ESBL	>16	R*	2	I	<=2	S	<=1	S	>1	R
>32	R	>16	R	>2	R	64		>4	R	>1	R
<=1	S	8	S	>2	R	<=2	S	<=1	S	<=0.5	S
>32	ESBL	>16	R*	>2	R	<=2	S	<=1	S	<=0.5	S
>32	ESBL	>16	R*	2	I	<=2	S	<=1	S	1	I
>32	R	>16	R	<=0.5	S	<=2	S	<=1	S	1	I
<=1	S	8	S	>2	R	<=2	S	<=1	S	>1	R

>32	R	>16	R	>2	R	<=2	S	>4	R	>1	R
>32	R	>16	R	2	I	<=2	S	>4	R	>1	R
<=1	S	8	S	2	I	<=2	S	<=1	S	1	I
>32	R	>16	R	>2	R	<=2	S	>4	R	>1	R
>32	ESBL	>16	R*	>2	R	1		<=1	S	<=0.5	S
>32	R	>16	R	>2	R	<=2	S	>4	R	>1	R
>32	R	>16	R	2	I	<=2	S	>4	R	>1	R
>32	R	>16	R	>2	R	<=2	S	>4	R	>1	R
>32	ESBL	>16	R*	2	I	<=2	S	<=1	S	<=0.5	S
>32	ESBL	>16	R*	>2	R	<=2	S	<=1	S	<=0.5	S
>32	R	>16	R	>2	R	<=2	S	>4	R	>1	R
>32	EBL?	>16	R	2	I	<=2	S	4	R	<=0.5	S
>32	R	>16	R	2	I	<=2	S	>4	R	>1	R
>32	ESBL	>16	R*	>2	R	8		<=1	S	>1	R
>32	ESBL	>16	R*	2	I	<=2	S	<=1	S	1	I
>32	R	>16	R	>2	R	1		>4	R	>1	R
>32	R	>16	R	>2	R	8		<=1	S	>1	R
>32	R	>16	R	>2	R	<=2	S	<=1	S	>1	R
>32	ESBL	>16	R*	2	I	<=2	S	4	R	1	I
>32	R	>16	R	>2	R	1		4	R	1	I
>32	ESBL	>16	R*	2	I	0.5		2	I	<=0.5	S
<=1	S	8	S	>2	R	<=2	S	<=1	S	<=0.5	S
>32	ESBL	>16	R*	>2	R	<=2	S	<=1	S	1	I
>32	ESBL	>16	R*	>2	R	<=2	S	<=1	S	1	I
>32	ESBL	>16	R*	>2	R	<=2	S	<=1	S	1	I
>32	R	>16	R	2	I	<=2	S	>4	R	>1	R
>32	ESBL	>16	R*	>2	R	<=2	S	<=1	S	1	I
>32	R	>16	R	>2	R	<=2	S	>4	R	>1	R
>32	R	>16	R	>2	R	<=2	S	>4	R	>1	R
>32	R	>16	R	>2	R	<=2	S	>4	R	>1	R
>32	R	>16	R	>2	R	<=2	S	<=1	S	>1	R
>32	R	>16	R	1	S	<=2	S	>4	R	>1	R
>32	R	>16	R	>2	R	<=2	S	>4	R	>1	R
<=1	S	8	S	1	S	<=2	S	<=1	S	>1	R
>32	R	>16	R	>2	R	<=2	S	>4	R	>1	R
>32	ESBL	>16	R*	>2	R	<=2	S	<=1	S	>1	R
>32	ESBL	>16	R*	>2	R	<=2	S	<=1	S	1	I
<=1	S	8	S	<=0.5	S	<=2	S	<=1	S	<=0.5	S
>32	R	>16	R	<=0.5	S	<=2	S	>4	R	>1	R
>32	R	>16	R	>2	R	<=2	S	>4	R	>1	R
>32	R	>16	R	>2	R	<=2	S	>4	R	>1	R
>32	ESBL	>16	R*	>2	R	1		<=1	S	>1	R
>32	R	16	I	2	I	<=2		4	R	>1	R
>32	R	>16	R	>2	R	<=2	S	>4	R	>1	R

>32	ESBL	>16	R*	<=0.5	S	<=2	S	<=1	S	>1	R
>32	R	>16	R	>2	R	<=2	S	>4	R	>1	R
32	ESBL	16	R*	2	I	<=2	S	<=1	S	<=0.5	S
>32	R	>16	R	>2	R	<=2	S	>4	R	>1	R
>32	R	>16	R	>2	R	32		>4	R	>1	R
>32	R	>16	R	>2	R	1		4	R	>1	R
>32	R	>16	R	>2	R	<=2	S	<=1	S	>1	R
>32	ESBL	>16	R*	>2	R	<=2	S	<=1	S	1	I
>32	ESBL	>16	R*	>2	R	32		>4	R	>1	R
>32	ESBL	>16	R*	>2	R	<=2	S	<=1	S	>1	R
>32	ESBL	>16	R*	>2	R	2		<=1	S	>1	R
>32	R	>16	R	>2	R	0.5		>4	R	>1	R
>32	ESBL	>16	R*	>2	R	<=2	S	<=1	S	<=0.5	S
>32	ESBL	>16	R*	>2	R	<=2	S	<=1	S	<=0.5	S
<=1	S	<=4	S	<=0.5	S	<=2		<=1	S	1	I
>32	R	>16	R	>2	R	<=2	S	>4	R	>1	R
>32	ESBL	>16	R*	>2	R	<=2	S	<=1	S	1	I
>32	R	>16	R	>2	R	<=2	S	>4	R	>1	R
>32	R	>16	R	>2	R	>4		<=1	S	>1	R
>32	R	>16	R	>2	R	<=2	S	<=1	S	>1	R
>32	R	>16	R	>2	R	<=2	S	>4	R	>1	R

>32	R	>16	R	2	I	<=2	S	<=1	S	<=0.5	S
>32	R	>16	R	>2	R	16		>4	R	>1	R
>32	R	>16	R	>2	R	16		>4	R	>1	R
>32	R	>16	R	>2	R	<=2	S	>4	R	>1	R
>32	R	>16	R	>2	R	<=2	S	>4	R	>1	R
>32	R	>16	R	>2	R	<=2	S	>4	R	>1	R
<=1	S	<=4	S	<=0.5	S	>4	R	>4	R	>1	R
>32	R	>16	R	>2	R	<=2	S	<=1	S	1	I

<=1	S	8	S	1	S	<=2	S	<=1	S	>1	R
>32	R	>16	R	>2	R	<=2	S	<=1	S	<=0.5	S
>32	ESBL	>16	R*	>2	R	<=2	S	<=1	S	1	I
>32	ESBL	>16	R*	>2	R	<=2	S	<=1	S	>1	R
>32	ESBL	>16	R*	>2	R	<=2	S	<=1	S	1	I
>32	ESBL	>16	R*	>2	R	<=2	S	<=1	S	1	I
>32	R	>16	R	>2	R	32		>4	R	>1	R
>32	ESBL	>16	R*	>2	R	<=2	S	<=1	S	<=0.5	S
>32	ESBL	>16	R*	2	I	<=2	S	<=1	S	>1	R
>32	ESBL	>16	R*	<=0.5	S	<=2	S	<=1	S	1	I
>32	R	>16	R	>2	R	<=2	S	<=1	S	>1	R
>32	ESBL	>16	R*	<=0.5	S	<=2	S	<=1	S	>1	R
>32	R	>16	R	>2	R	16		>4	R	>1	R
>32	R	>16	R	>2	R	<=2	S	>4	R	>1	R
>32	ESBL	>16	R*	1	S	<=2	S	<=1	S	<=0.5	S
>32	R	>16	R	>2	R	<=2	S	<=1	S	>1	R
>32	ESBL	>16	R*	>2	R	<=2	S	<=1	S	>1	R
>32	R	>16	R	<=0.5	S	<=2	S	>4	R	>1	R
<=1	S	<=4	S	1	S	>4	R	<=1	S	<=0.5	S
<=1	S	<=4	S	1	S	<=2	S	<=1	S	1	I
>32	ESBL	>16	R*	>2	R	<=2	S	<=1	S	1	I
>32	R	>16	R	>2	R	16		>4	R	>1	R
>32	R	>16	R	>2	R	16		>4	R	>1	R
>32	R	>16	R	>2	R	<=2	S	>4	R	>1	R
>32	ESBL	>16	R*	<=0.5	S	<=2	S	<=1	S	<=0.5	S
>32	R	>16	R	>2	R	16		>4	R	>1	R
>32	R	>16	R	>2	R	16		>4	R	>1	R
>32	R	>16	R	>2	R	1		>4	R	>1	R
>32	ESBL	>16	R*	>2	R	<=2	S	<=1	S	<=0.5	S
>32	R	>16	R	>2	R	32		>4	R	>1	R
>32	ESBL	>16	R*	>2	R	1		<=1	S	>1	R
>32	R	>16	R	>2	R	<=2	S	<=1	S	>1	R
>32	R	>16	R	>2	R	<=2	S	<=1	S	>1	R
>32	ESBL	>16	R*	>2	R	>4	R	<=1	S	<=0.5	S
>32	ESBL	>16	R*	>2	R	<=2	S	<=1	S	1	I
>32	R	>16	R	>2	R	<=2		>4	R	>1	R
>32	ESBL	>16	R*	>2	R	<=2	S	<=1	S	>1	R
>32	ESBL	>16	R*	>2	R	<=2	S	<=1	S	1	I
>32	ESBL	>16	R*	>2	R	<=2	S	<=1	S	<=0.5	S
>32	R	>16	R	2	I	<=2	S	>4	R	>1	R
>32	ESBL	>16	R*	>2	R	<=2	S	<=1	S	<=0.5	S
>32	ESBL	>16	R*	>2	R	<=2	S	<=1	S	1	I
>32	ESBL	>16	R*	>2	R	<=2	S	<=1	S	>1	R
>32	ESBL	>16	R*	>2	R	1		<=1	S	>1	R
>32	R	>16	R	>2	R	<=2	S	<=1	S	>1	R
>32	R	>16	R	>2	R	<=2	S	>4	R	>1	R
>32	ESBL	>16	R*	>2	R	<=2	S	2	I	>1	R

>32	R	>16	R	>2	R	<=2	S	>4	R	>1	R
>32	R	>16	R	>2	R	8		>4	R	>1	R
>32	R	>16	R	>2	R	8		>4	R	>1	R
>32	ESBL	>16	R*	>2	R	<=2	S	<=1	S	1	I
>32	R	>16	R	>2	R	<=2	S	<=1	S	<=0.5	S
>32	ESBL	>16	R*	>2	R	1		<=1	S	>1	R
>32	ESBL	>16	R*	>2	R	<=2	S	<=1	S	>1	R
>32	R	>16	R	>2	R	<=2	S	<=1	S	1	I
>32	R	>16	R	>2	R	8		>4	R	>1	R
>32	R	>16	R	<=0.5	S	<=2	S	>4	R	>1	R
>32	R	>16	R	>2	R	<=2	S	<=1	S	>1	R
>32	R	>16	R	>2	R	1		>4	R	>1	R
<=1	S	8	S	1	S	1		<=1	S	>1	R
>32	R	>16	R	>2	R	>4	R	>4	R	>1	R
>32	R	>16	R	>2	R	<=2	S	>4	R	>1	R
>32	R	>16	R	1	S	1		4	R	>1	R
>32	R	>16	R	<=0.5	S	<=2	S	<=1	S	1	I
>32	ESBL	>16	R*	<=0.5	S	<=2	S	<=1	S	>1	R
>32	R	>16	R	>2	R	<=2	S	>4	R	>1	R
>32	R	>16	R	2	I	<=2	S	<=1	S	1	I
>32	R	>16	R	>2	R	<=2	S	>4	R	>1	R
>32	ESBL	>16	R*	>2	R	<=2	S	<=1	S	1	I
>32	ESBL	>16	R*	>2	R	<=2	S	<=1	S	>1	R
>32	ESBL	>16	R*	>2	R	<=2	S	<=1	S	>1	R
>32	R	>16	R	>2	R	<=2		>4	R	>1	R
>32	ESBL	>16	R*	1	S	<=2	S	>4	R	>1	R
>32	R	>16	R	>2	R	<=2	S	>4	R	>1	R
>32	ESBL	>16	R*	>2	R	<=2	S	<=1	S	1	I
>32	R	>16	R	1	S	<=2	S	>4	R	>1	R

>32	ESBL	>16	R*	>2	R	<=2	S	<=1	S	1	I
>32	R	>16	R	>2	R	<=2	S	<=1	S	>1	R
>32	R	>16	R	>2	R	<=2	S	<=1	S	>1	R
>32	R	>16	R	>2	R	<=2	S	>4	R	>1	R
>32	ESBL	>16	R*	>2	R	<=2	S	<=1	S	>1	R
>32	R	>16	R	>2	R	<=2	S	4	R	>1	R
>32	EBL?	>16	R	2	I	<=2	S	<=1	S	<=0.5	S
<=1	S	16	I	1	S	<=2		<=1	S	<=0.5	S
>32	R	>16	R	>2	R	<=2	S	>4	R	>1	R
>32	R	>16	R	>2	R	<=2	S	>4	R	>1	R
>32	R	>16	R	>2	R	<=2	S	<=1	S	1	I
>32	R	>16	R	1	S	<=2	S	>4	R	>1	R
>32	R	>16	R	>2	R	<=2	S	4	R	>1	R
>32	ESBL	>16	R*	>2	R	<=2	S	<=1	S	1	I
>32	ESBL	>16	R*	>2	R	<=2	S	<=1	S	<=0.5	S
>32	ESBL	>16	R*	>2	R	<=2	S	<=1	S	>1	R
>32	R	>16	R	2	I	<=2	S	>4	R	>1	R
>32	EBL?	>16	R	>2	R	<=2	S	<=1	S	<=0.5	S
>32	ESBL	>16	R*	<=0.5	S	<=2	S	<=1	S	<=0.5	S
>32	ESBL	>16	R*	>2	R	<=2	S	<=1	S	>1	R
2	I	>16	R	<=0.5	S	<=2	S	<=1	S	>1	R
>32	ESBL	>16	R*	>2	R	<=2	S	<=1	S	1	I
>32	ESBL	>16	R*	>2	R	<=2	S	<=1	S	>1	R
>32	ESBL	>16	R*	2	I	<=2	S	<=1	S	1	I
>32	EBL?	>16	R	>2	R	<=2	S	<=1	S	>1	R
>32	ESBL	>16	R*	<=0.5	S	<=2	S	4	R	>1	R
>32	ESBL	>16	R*	>2	R	<=2	S	2	I	>1	R
>32	ESBL	>16	R*	1	S	<=2	S	<=1	S	1	I
>32	ESBL	>16	R*	2	I	<=2	S	<=1	S	>1	R
>32	ESBL	>16	R*	2	I	<=2	S	<=1	S	1	I
>32	ESBL	>16	R*	>2	R	>4	R	<=1	S	<=0.5	S
>32	EBL?	>16	R	>2	R	8		>4	R	>1	R
>32	EBL?	>16	R	>2	R	0.5		>4	R	>1	R
>32	EBL?	>16	R	>2	R	<=2	S	<=1	S	>1	R
>32	EBL?	>16	R	>2	R	0.5		>4	R	>1	R
>32	EBL?	>16	R	>2	R	<=2	S	<=1	S	>1	R
>32	EBL?	>16	R	>2	R	64		>4	R	>1	R
>32	EBL?	>16	R	>2	R	<=2	S	<=1	S	<=0.5	S
>32	EBL?	>16	R	2	I	<=2	S	>4	R	>1	R
>32	EBL?	>16	R	>2	R	<=2	S	4	R	>1	R

>32	EBL?	>16	R	2	I	<=2	S	<=1	S	>1	R
>32	EBL?	>16	R	2	I	<=2	S	<=1	S	>1	R
>32	EBL?	>16	R	>2	R	<=2	S	>4	R	>1	R
>32	EBL?	>16	R	>2	R	<=2	S	<=1	S	>1	R
>32	EBL?	>16	R	>2	R	<=2	S	<=1	S	1	I
>32	EBL?	>16	R	>2	R	<=2	S	>4	R	>1	R
>32	EBL?	>16	R	2	I	<=2	S	<=1	S	1	I
>32	EBL?	>16	R	>2	R	<=2	S	<=1	S	1	I
>32	EBL?	>16	R	>2	R	<=2	S	<=1	S	1	I
>32	R	>16	R	>2	R	<=2	S	>4	R	>1	R
>32	EBL?	>16	R	>2	R	<=2	S	<=1	S	<=0.5	S
>32	EBL?	>16	R	>2	R	<=2	S	>4	R	>1	R
>32	EBL?	>16	R	>2	R	<=2	S	>4	R	>1	R
>32	EBL?	>16	R	2	I	<=2	S	<=1	S	>1	R
>32	EBL?	>16	R	>2	R	<=2	S	>4	R	>1	R
>32	EBL?	>16	R	>2	R	1		>4	R	>1	R
>32	EBL?	>16	R	>2	R	<=2	S	<=1	S	1	I
>32	EBL?	>16	R	>2	R	8		>4	R	>1	R
>32	EBL?	>16	R	2	I	<=2	S	<=1	S	>1	R
>32	EBL?	>16	R	>2	R	8		>4	R	>1	R
>32	ESBL	>16	R*	>2	R	<=2	S	<=1	S	>1	R
>32	EBL?	>16	R	>2	R	<=2	S	>4	R	>1	R
>32	EBL?	>16	R	>2	R	<=2	S	>4	R	>1	R
>32	EBL?	>16	R	>2	R	<=2	S	>4	R	>1	R

<=1	S	16	I	1	S	<=2	S	<=1	S	>1	R
>32	EBL?	>16	R	2	I	<=2	S	<=1	S	<=0.5	S
>32	EBL?	>16	R	>2	R	<=2	S	>4	R	>1	R
>32	EBL?	>16	R	>2	R	<=2	S	<=1	S	>1	R
>32	EBL?	>16	R	>2	R	<=2	S	<=1	S	>1	R
>32	EBL?	>16	R	1	S	<=2	S	<=1	S	<=0.5	S

<=1	S	8	S	2	I	<=2	S	<=1	S	<=0.5	S
>32	EBL?	>16	R	>2	R	<=2	S	>4	R	>1	R
>32	EBL?	>16	R	>2	R	<=2	S	>4	R	>1	R
>32	ESBL	>16	R*	>2	R	<=2	S	>4	R	>1	R

>32	EBL?	>16	R	2	I	<=2	S	4	R	>1	R
>32	EBL?	>16	R	>2	R	<=2	S	<=1	S	<=0.5	S
>32	EBL?	>16	R	>2	R	1		>4	R	>1	R

>32	EBL?	>16	R	>2	R	64		>4	R	>1	R
>32	EBL?	>16	R	>2	R	<=2	S	>4	R	>1	R
>32	EBL?	>16	R	2	I	<=2	S	<=1	S	1	I
>32	EBL?	>16	R	2	I	<=2	S	<=1	S	1	I
<=1	S	8	S	>2	R	<=2	S	<=1	S	1	I
>32	EBL?	>16	R	>2	R	<=2	S	<=1	S	1	I
>32	EBL?	>16	R	>2	R	<=2	S	>4	R	>1	R
>32	EBL?	>16	R	>2	R	<=2	S	<=1	S	1	I
>32	EBL?	>16	R	2	I	<=2	S	4	R	>1	R
>32	R	>16	R	>2	R	<=2	S	>4	R	>1	R
>32	EBL?	>16	R	>2	R	<=2	S	>4	R	>1	R

>32	EBL?	>16	R	>2	R	<=2	S	>4	R	>1	R
>32	R	>16	R	>2	R	<=2	S	<=1	S	>1	R
>32	R	>16	R	>2	R	<=2	S	<=1	S	1	I
>32	EBL?	>16	R	>2	R	<=2	S	>4	R	>1	R
>32	EBL?	>16	R	>2	R	<=2	S	>4	R	>1	R
>32	R	>16	R	<=0.5	S	<=2	S	<=1	S	>1	R
>32	EBL?	>16	R	>2	R	<=2	S	>4	R	>1	R
>32	EBL?	>16	R	>2	R	<=2	S	>4	R	>1	R
>32	EBL?	>16	R	<=0.5	S	<=2	S	>4	R	>1	R
>32	R	>16	R	>2	R	<=2	S	<=1	S	>1	R
>32	EBL?	>16	R	>2	R	<=2	S	4	R	>1	R
>32	EBL?	>16	R	1	S	<=2	S	>4	R	>1	R
>32	R	>16	R	>2	R	1		>4	R	>1	R
>32	EBL?	>16	R	>2	R	<=2	S	<=1	S	<=0.5	S
>32	EBL?	>16	R	>2	R	<=2	S	<=1	S	1	I
>32	EBL?	>16	R	2	I	<=2	S	<=1	S	1	I
>32	EBL?	>16	R	2	I	<=2	S	<=1	S	1	I
>32	ESBL	>16	R*	1	S	<=2	S	<=1	S	<=0.5	S
4	EBL?	>16	R	1	S	<=2	S	4	R	>1	R
>32	EBL?	>16	R	>2	R	<=2	S	4	R	>1	R
>32	EBL?	>16	R	>2	R	<=2	S	>4	R	>1	R
>32	EBL?	>16	R	>2	R	<=2	S	>4	R	>1	R
<=1	S	8	S	>2	R	<=2	S	<=1	S	>1	R
<=1	S	<=4	S	<=0.5	S	>4	R	<=1	S	<=0.5	S
>32	EBL?	>16	R	>2	R	<=2	S	<=1	S	>1	R
>32	EBL?	>16	R	>2	R	<=2	S	<=1	S	<=0.5	S
>32	EBL?	>16	R	>2	R	<=2	S	<=1	S	<=0.5	S
>32	EBL?	>16	R	<=0.5	S	<=2	S	<=1	S	<=0.5	S
>32	EBL?	>16	R	>2	R	<=2		>4	R	>1	R
>32	R	>16	R	>2	R	<=2		<=1	S	>1	R
>32	EBL?	>16	R	>2	R	<=2		<=1	S	<=0.5	S
>32	EBL?	>16	R	1	S	<=2		4	R	>1	R
>32	R	>16	R	<=0.5	S	>4		>4	R	>1	R
>32	R	>16	R	>2	R	>4		>4	R	>1	R
>32	EBL?	>16	R	>2	R	<=2		<=1	S	1	I
>32	EBL?	>16	R	>2	R	<=2		>4	R	>1	R
>32	EBL?	>16	R	>2	R	<=2		<=1	S	<=0.5	S
>32	EBL?	>16	R	2	I	<=2		<=1	S	1	I
>32	EBL?	>16	R	1	S	<=2		2	I	>1	R
>32	EBL?	>16	R	>2	R	<=2		4	R	>1	R
>32	R	>16	R	>2	R	<=2		<=1	S	>1	R
>32	R	>16	R	>2	R	<=2	S	<=1	S	>1	R
<=1	S	8	S	1	S	<=2	S	<=1	S	1	I
>32	EBL?	>16	R	>2	R	<=2		<=1	S	<=0.5	S

>32	EBL?	>16	R	>2	R	<=2	>4	R	>1	R
>32	R	>16	R	>2	R	>4	>4	R	>1	R
>32	EBL?	>16	R	>2	R	<=2	>4	R	>1	R
>32	EBL?	>16	R	<=0.5	S	<=2	<=1	S	>1	R
>32	EBL?	>16	R	1	S	<=2	>4	R	>1	R
>32	EBL?	>16	R	>2	R	<=2	<=1	S	1	I
<=1	S	16	I	<=0.5	S	<=2	<=1	S	<=0.5	S
>32	ESBL	>16	R*	1	S	>4	<=1	S	<=0.5	S
>32	R	>16	R	2	I	>128	>4	R	>1	R
>32	R	>16	R	>2	R	<=2	>4	R	>1	R
>32	EBL?	>16	R	>2	R	<=2	>4	R	>1	R
>32	EBL?	>16	R	>2	R	<=2	>4	R	>1	R
>32	EBL?	>16	R	>2	R	<=2	>4	R	>1	R
>32	EBL?	>16	R	>2	R	<=2	<=1	S	1	I
>32	EBL?	>16	R	>2	R	<=2	<=1	S	1	I
>32	ESBL	>16	R*	>2	R	<=2	<=1	S	>1	R
>32	EBL?	>16	R	>2	R	<=2	>4	R	>1	R
>32	EBL?	>16	R	>2	R	<=2	>4	R	>1	R
>32	EBL?	>16	R	<=0.5	S	<=2	4	R	>1	R
>32	EBL?	>16	R	>2	R	<=2	<=1	S	1	I
>32	EBL?	>16	R	>2	R	<=2	>4	R	>1	R
>32	EBL?	>16	R	<=0.5	S	<=2	<=1	S	<=0.5	S
>32	EBL?	>16	R	<=0.5	S	<=2	<=1	S	<=0.5	S
>32	EBL?	>16	R	>2	R	<=2	>4	R	>1	R
>32	EBL?	>16	R	>2	R	<=2	>4	R	>1	R
>32	EBL?	>16	R	>2	R	<=2	>4	R	>1	R
>32	EBL?	>16	R	>2	R	<=2	>4	R	>1	R
>32	EBL?	>16	R	>2	R	<=2	2	I	>1	R
>32	EBL?	>16	R	2	I	<=2	<=1	S	1	I
>32	EBL?	>16	R	>2	R	1	<=1	S	>1	R
>32	EBL?	>16	R	>2	R	<=2	<=1	S	>1	R
<=1	S	<=4	S	<=0.5	S	2	<=1	S	<=0.5	S
>32	R	>16	R	>2	R	>4	>4	R	>1	R
>32	EBL?	>16	R	>2	R	<=2	<=1	S	<=0.5	S
>32	EBL?	>16	R	>2	R	<=2	>4	R	>1	R
32	R	>16	R	>2	R	>4	2	I	<=0.5	S
2	EBL?	>16	R	1	S	<=2	2	I	>1	R
>32	R	>16	R	>2	R	<=2	<=1	S	>1	R
>32	EBL?	>16	R	>2	R	<=2	<=1	S	>1	R
>32	EBL?	>16	R	>2	R	<=2	<=1	S	>1	R
>32	EBL?	>16	R	>2	R	<=2	<=1	S	<=0.5	S
>32	EBL?	>16	R	>2	R	<=2	>4	R	>1	R
>32	EBL?	>16	R	>2	R	<=2	<=1	S	>1	R
>32	EBL?	>16	R	<=0.5	S	<=2	>4	R	>1	R
>32	EBL?	>16	R	>2	R	<=2	<=1	S	>1	R
>32	EBL?	>16	R	>2	R	<=2	>4	R	>1	R
>32	R	>16	R	>2	R	<=2	<=1	S	>1	R
>32	EBL?	>16	R	>2	R	<=2	>4	R	>1	R
>32	EBL?	>16	R	>2	R	<=2	>4	R	>1	R
>32	EBL?	>16	R	>2	R	0.5	>4	R	>1	R
>32	EBL?	>16	R	>2	R	1	>4	R	>1	R
<=1	S	<=4	S	2	I	<=2	<=1	S	<=0.5	S
>32	ESBL	>16	R*	>2	R	<=2	<=1	S	>1	R
>32	R	>16	R	>2	R	1	>4	R	>1	R
>32	EBL?	>16	R	2	I	<=2	<=1	S	1	I
>32	EBL?	>16	R	>2	R	<=2	>4	R	>1	R

>32	EBL?	>16	R	>2	R	<=2	>4	R	>1	R
>32	EBL?	>16	R	>2	R	<=2	<=1	S	1	I
>32	EBL?	>16	R	>2	R	<=2	<=1	S	<=0.5	S
>32	EBL?	>16	R	<=0.5	S	<=2	<=1	S	<=0.5	S
>32	EBL?	>16	R	<=0.5	S	<=2	4	R	>1	R
>32	EBL?	>16	R	>2	R	16	>4	R	>1	R
>32	EBL?	>16	R	>2	R	<=2	<=1	S	1	I
>32	EBL?	>16	R	>2	R	<=2	<=1	S	<=0.5	S
>32	EBL?	>16	R	>2	R	<=2	>4	R	>1	R
>32	EBL?	>16	R	>2	R	<=2	>4	R	>1	R
<=1	S	>16	R	<=0.5	S	128	<=1	S	<=0.5	S
>32	EBL?	>16	R	>2	R	1	<=1	S	>1	R
>32	EBL?	>16	R	2	I	1	>4	R	>1	R
>32	EBL?	>16	R	>2	R	<=2	>4	R	>1	R

>32	R	>16	R	>2	R	<=2	<=1	S	>1	R
>32	EBL?	>16	R	>2	R	<=2	<=1	S	1	I
4	R	>16	R	<=0.5	S	>4	<=1	S	>1	R
>32	EBL?	>16	R	2	I	<=2	<=1	S	1	I
>32	ESBL	>16	R*	>2	R	<=2	<=1	S	>1	R
>32	EBL?	>16	R	2	I	<=2	<=1	S	>1	R
2	EBL?	>16	R	<=0.5	S	1	<=1	S	1	I
>32	EBL?	>16	R	>2	R	<=2	>4	R	>1	R
>32	EBL?	>16	R	>2	R	<=2	>4	R	>1	R
>32	R	>16	R	2	I	<=2	<=1	S	<=0.5	S
>32	EBL?	>16	R	>2	R	<=2	<=1	S	1	I
2	I	>16	R	<=0.5	S	>4	<=1	S	>1	R
<=1	S	<=4	S	<=0.5	S	<=2	<=1	S	<=0.5	S
>32	EBL?	>16	R	<=0.5	S	<=2	>4	R	>1	R
>32	EBL?	>16	R	>2	R	<=2	<=1	S	>1	R
>32	EBL?	>16	R	<=0.5	S	<=2	>4	R	>1	R
>32	EBL?	>16	R	2	I	<=2	<=1	S	>1	R
>32	EBL?	>16	R	2	I	<=2	<=1	S	>1	R
>32	R	>16	R	>2	R	<=2	>4	R	>1	R
>32	EBL?	>16	R	>2	R	<=2	<=1	S	1	I
>32	EBL?	>16	R	>2	R	<=2	>4	R	>1	R
>32	EBL?	>16	R	>2	R	<=2	<=1	S	1	I
<=1	S	<=4	S	<=0.5	S	<=2	<=1	S	<=0.5	S
>32	R	>16	R	2	I	>4	>4	R	>1	R
>32	EBL?	>16	R	>2	R	<=2	<=1	S	1	I
>32	EBL?	>16	R	>2	R	<=2	<=1	S	<=0.5	S
>32	R	>16	R	>2	R	1	<=1	S	>1	R
>32	EBL?	>16	R	>2	R	<=2	<=1	S	>1	R
>32	EBL?	>16	R	>2	R	<=2	4	R	>1	R
>32	EBL?	>16	R	>2	R	<=2	>4	R	>1	R
2	I	>16	R	<=0.5	S	>4	<=1	S	>1	R
2	I	>16	R	<=0.5	S	>4	<=1	S	>1	R

>32	R	>16	R	<=0.5	S	<=2	>4	R	>1	R
>32	EBL?	>16	R	>2	R	<=2	4	R	>1	R
>32	EBL?	>16	R	>2	R	16	>4	R	>1	R
>32	R	>16	R	>2	R	<=2	>4	R	>1	R
>32	EBL?	>16	R	>2	R	1	<=1	S	1	I
>32	EBL?	>16	R	>2	R	<=2	>4	R	>1	R
>32	EBL?	>16	R	<=0.5	S	<=2	>4	R	>1	R
>32	EBL?	>16	R	2	I	<=2	<=1	S	1	I
>32	EBL?	>16	R	2	I	1	<=1	S	<=0.5	S
>32	EBL?	>16	R	2	I	<=2	<=1	S	>1	R
>32	EBL?	>16	R	2	I	<=2	<=1	S	>1	R
>32	EBL?	>16	R	<=0.5	S	<=2	4	R	1	I
>32	EBL?	>16	R	2	I	<=2	<=1	S	1	I
>32	EBL?	>16	R	>2	R	<=2	<=1	S	>1	R
>32	EBL?	>16	R	>2	R	4	<=1	S	1	I
>32	EBL?	>16	R	>2	R	<=2	<=1	S	1	I
>32	EBL?	>16	R	>2	R	<=2	<=1	S	>1	R
>32	EBL?	>16	R	>2	R	<=2	<=1	S	>1	R
>32	EBL?	>16	R	2	I	<=2	>4	R	>1	R
>32	R	>16	R	>2	R	<=2	>4	R	>1	R
>32	EBL?	>16	R	>2	R	<=2	<=1	S	>1	R
>32	EBL?	>16	R	>2	R	<=2	<=1	S	1	I
>32	EBL?	>16	R	>2	R	<=2	>4	R	>1	R
>32	EBL?	>16	R	2	I	<=2	<=1	S	1	I
2	I	>16	R	<=0.5	S	>4	<=1	S	>1	R
16	R	>16	R	<=0.5	S	>4	2	I	>1	R
>32	ESBL	>16	R*	2	I	<=2	<=1	S	<=0.5	S
>32	R	>16	R	>2	R	<=2	>4	R	>1	R
		>16	R	<=0.5	S	>4	<=1	S	1	I
>32	ESBL	>16	R*	<=0.5	S	<=2	<=1	S	<=0.5	S
<=1	S	8	S	>2	R	<=2	<=1	S	1	I
>32	ESBL	>16	R*	2	I	<=2	<=1	S	1	I
>32	ESBL	>16	R*	>2	R	<=2	<=1	S	1	I
>32	ESBL	>16	R*	2	I	<=2	<=1	S	>1	R
>32	R	>16	R	>2	R	8	>4	R	>1	R
>32	ESBL	>16	R*	>2	R	<=2	<=1	S	1	I
>32	R	>16	R	>2	R	<=2	>4	R	>1	R
>32	ESBL	>16	R*	2	I	<=2	<=1	S	>1	R
>32	R	>16	R	>2	R	<=2	>4	R	>1	R
>32	ESBL	>16	R*	>2	R	<=2	<=1	S	<=0.5	S
>32	R	>16	R	>2	R	<=2	>4	R	>1	R
>32	R	>16	R	>2	R	<=2	>4	R	>1	R
>32	R	>16	R	>2	R	16	>4	R	>1	R
>32	R	>16	R	2	I	<=2	>4	R	>1	R
>32	R	>16	R	>2	R	<=2	>4	R	>1	R
>32	ESBL	>16	R*	2	I	<=2	>4	R	>1	R
>32	ESBL	>16	R*	2	I	<=2	<=1	S	>1	R
<=1	S	8	S	1	S	<=2	<=1	S	>1	R
>32	R	>16	R	<=0.5	S	<=2	>4	R	>1	R

>32	R	>16	R	<=0.5	S	1	4	R	>1	R
>32	ESBL	>16	R*	2	I	<=2	<=1	S	1	I
<=1	S	8	S	>2	R	<=2	<=1	S	1	I
>32	R	>16	R	>2	R	<=2	>4	R	>1	R
N/R		>16	R	<=0.5	S	>4	<=1	S	>1	R
>32	ESBL	>16	R*	2	I	<=2	4	R	>1	R
>32	R	>16	R	>2	R	<=2	>4	R	>1	R
>32	R	>16	R	>2	R	1	>4	R	>1	R
>32	R	>16	R	>2	R	1	2	I	>1	R
>32	R	>16	R	>2	R	16	>4	R	>1	R
<=1	R*	16	R*	2	I	4	>4	R	>1	R
>32	ESBL	>16	R*	2	I	<=2	>4	R	>1	R
>32	ESBL	>16	R*	>2	R	<=2	<=1	S	>1	R
>32	R	>16	R	>2	R	0.5	>4	R	>1	R
>32	ESBL	>16	R*	>2	R	<=2	<=1	S	1	I
>32	R	>16	R	>2	R	1	>4	R	>1	R
>32	R	>16	R	<=0.5	S	16	>4	R	>1	R
<=1	S	8	S	<=0.5	S	1	<=1	S	>1	R
>32	ESBL	>16	R*	<=0.5	S	<=2	<=1	S	>1	R
>32	R	>16	R	>2	R	<=2	>4	R	>1	R
>32	R	>16	R	>2	R	<=2	<=1	S	>1	R
<=1	S	8	S	1	S	<=2	<=1	S	1	I
>32	ESBL	>16	R*	>2	R	<=2	<=1	S	>1	R
<=1	S	8	S	1	S	<=2	<=1	S	<=0.5	S
>32	R	>16	R	<=0.5	S	1	>4	R	>1	R
>32	R	>16	R	>2	R	8	>4	R	>1	R
>32	ESBL	>16	R*	>2	R	<=2	<=1	S	1	I

>32	ESBL	>16	R*	<=0.5	S	<=2	<=1	S	<=0.5	S
N/R		>16	R	<=0.5	S	>4	4	R	>1	R
>32	ESBL	>16	R*	>2	R	<=2	<=1	S	1	I
>32	ESBL	>16	R*	>2	R	<=2	<=1	S	>1	R
>32	ESBL	>16	R*	2	I	<=2	<=1	S	>1	R
>32	R	>16	R	>2	R	<=2	>4	R	>1	R
>32	ESBL	>16	R*	>2	R	<=2	<=1	S	1	I
>32	R	>16	R	1	S	<=2	<=1	S	<=0.5	S
>32	ESBL	>16	R*	>2	R	<=2	<=1	S	<=0.5	S
>32	ESBL	>16	R*	>2	R	<=2	<=1	S	>1	R
>32	R	>16	R	>2	R	2	<=1	S	1	I
<=1	S	<=4	S	<=0.5	S	128	<=1	S	<=0.5	S
>32	R	>16	R	>2	R	16	>4	R	>1	R
>32	R	>16	R	>2	R	<=2	<=1	S	>1	R

>32	R	>16	R	>2	R	<=2	>4	R	>1	R
N/R		>16	R	<=0.5	S	>4	4	R	1	I
>32	ESBL	>16	R*	>2	R	<=2	<=1	S	>1	R
N/R		>16	R	>2	R	>4	>4	R	>1	R
>32	ESBL	>16	R*	<=0.5	S	<=2	<=1	S	1	I
>32	R	>16	R	>2	R	<=2	>4	R	>1	R
N/R		>16	R	>2	R	<=2	>4	R	>1	R
>32	ESBL	>16	R*	>2	R	<=2	<=1	S	>1	R
>32	ESBL	>16	R*	>2	R	<=2	<=1	S	>1	R
<=1	S	16	I	>2	R	<=2	<=1	S	>1	R
>32	ESBL	>16	R*	2	I	<=2	<=1	S	>1	R
>32	ESBL	>16	R*	2	I	<=2	<=1	S	>1	R
>32	ESBL	>16	R*	>2	R	<=2	<=1	S	>1	R
>32	ESBL	>16	R*	2	I	<=2	<=1	S	>1	R
>32	ESBL	>16	R*	2	I	<=2	<=1	S	>1	R
>32	ESBL	>16	R*	>2	R	<=2	<=1	S	1	I
>32	ESBL	>16	R*	2	I	<=2	<=1	S	>1	R
>32	ESBL	>16	R*	>2	R	<=2	<=1	S	<=0.5	S
>32	ESBL	>16	R*	<=0.5	S	<=2	<=1	S	>1	R
>32	R	>16	R	>2	R	<=2	<=1	S	>1	R
>32	ESBL	>16	R*	>2	R	<=2	<=1	S	<=0.5	S
>32	ESBL	>16	R*	2	I	<=2	2	I	>1	R
>32	ESBL	>16	R*	2	I	<=2	<=1	S	>1	R
>32	R	>16	R	>2	R	<=2	>4	R	>1	R
>32	ESBL	>16	R*	2	I	<=2	<=1	S	>1	R
>32	ESBL	>16	R*	2	I	<=2	<=1	S	>1	R
>32	ESBL	>16	R*	1	S	<=2	<=1	S	1	I
>32	ESBL	>16	R*	>2	R	<=2	<=1	S	>1	R
>32	ESBL	>16	R*	2	I	<=2	<=1	S	1	I
>32	ESBL	>16	R*	>2	R	<=2	<=1	S	<=0.5	S
>32	R	>16	R	<=0.5	S	<=2	<=1	S	>1	R
>32	R	>16	R	>2	R	>4	>4	R	>1	R
>32	R	>16	R	1	S	128	>4	R	>1	R
>32	ESBL	>16	R*	>2	R	<=2	4	R	>1	R
>32	ESBL	>16	R*	2	I	<=2	<=1	S	>1	R
>32	ESBL	>16	R*	>2	R	<=2	<=1	S	1	I
>32	ESBL	>16	R*	>2	R	<=2	<=1	S	>1	R
<=1	S	<=4	S	<=0.5	S	<=2	<=1	S	1	I
16	R	>16	R	<=0.5	S	<=2	<=1	S	<=0.5	S
>32	ESBL	>16	R*	>2	R	<=2	<=1	S	1	I
>32	R	>16	R	>2	R	<=2	<=1	S	>1	R
>32	ESBL	>16	R*	2	I	<=2	<=1	S	>1	R
>32	ESBL	>16	R*	2	I	>128	<=1	S	1	I
>32	ESBL	>16	R*	1	S	<=2	4	R	>1	R
>32	ESBL	>16	R*	2	I	<=2	<=1	S	1	I
N/R		>16	R	>2	R	>4	>4	R	>1	R
<=1	S	<=4	S	<=0.5	S	<=2	<=1	S	<=0.5	S
>32	R	>16	R	>2	R	<=2	4	R	>1	R
>32	R	>16	R	>2	R	>128	>4	R	>1	R
>32	ESBL	>16	R*	2	I	<=2	<=1	S	>1	R
>32	ESBL	>16	R*	>2	R	<=2	<=1	S	>1	R
>32	R	>16	R	<=0.5	S	<=2	4	R	>1	R
>32	R	>16	R	>2	R	>4	>4	R	>1	R
>32	ESBL	>16	R*	>2	R	<=2	2	I	1	I

>32	R	>16	R	>2	R	<=2	>4	R	>1	R
>32	R	>16	R	1	S	<=2	>4	R	>1	R
>32	R	>16	R	>2	R	<=2	>4	R	>1	R
N/R		>16	R	>2	R	>4	>4	R	>1	R
>32	R	>16	R	>2	R	<=2	>4	R	>1	R
>32	R	>16	R	>2	R	<=2	>4	R	>1	R
>32	ESBL	>16	R*	>2	R	<=2	<=1	S	>1	R
>32	ESBL	>16	R*	>2	R	<=2	<=1	S	1	I
>32	R	>16	R	>2	R	<=2	>4	R	>1	R
>32	R	>16	R	>2	R	<=2	>4	R	>1	R
>32	R	>16	R	>2	R	<=2	>4	R	>1	R
8	R	>16	R	<=0.5	S	>4	<=1	S	<=0.5	S
>32	R	>16	R	>2	R	<=2	<=1	S	>1	R
>32	R	>16	R	>2	R	<=2	<=1	S	>1	R
<=1	S	8	S	2	I	1	<=1	S	1	I
>32	R	>16	R	<=0.5	S	<=2	>4	R	>1	R
>32	ESBL	>16	R*	>2	R	<=2	<=1	S	>1	R
>32	R	>16	R	>2	R	<=2	>4	R	>1	R
>32	ESBL	>16	R*	2	I	<=2	<=1	S	>1	R
>32	R	>16	R	>2	R	<=2	>4	R	>1	R
>32	ESBL	>16	R*	>2	R	<=2	<=1	S	>1	R
>32	R	>16	R	>2	R	<=2	>4	R	>1	R
>32	R	>16	R	>2	R	<=2	>4	R	>1	R
>32	ESBL	>16	R*	>2	R	<=2	<=1	S	>1	R
>32	R	>16	R	>2	R	0.5	>4	R	>1	R
>32	R	>16	R	>2	R	<=2	>4	R	>1	R
>32	ESBL	>16	R*	>2	R	16	>4	R	>1	R
>32	R	>16	R	>2	R	<=2	>4	R	>1	R
>32	R	>16	R	>2	R	<=2	<=1	S	>1	R
>32	ESBL	>16	R*	2	I	<=2	<=1	S	>1	R
>32	ESBL	>16	R*	2	I	<=2	<=1	S	>1	R
>32	ESBL	>16	R*	2	I	<=2	<=1	S	>1	R
>32	R	>16	R	2	I	<=2	<=1	S	<=0.5	S
>32	ESBL	>16	R*	>2	R	<=2	<=1	S	>1	R
>32	ESBL	>16	R*	1	S	<=2	<=1	S	<=0.5	S
>32	ESBL	>16	R*	>2	R	<=2	<=1	S	>1	R
>32	ESBL	>16	R*	>2	R	<=2	<=1	S	1	I
>32	R	>16	R	>2	R	<=2	>4	R	>1	R
>32	R	>16	R	2	I	>128	>4	R	>1	R
N/R		>16	R	>2	R	>4	>4	R	>1	R
>32	R	>16	R	<=0.5	S	<=2	>4	R	>1	R

>32	R	>16	R	>2	R	<=2	>4	R	>1	R
>32	ESBL	>16	R*	2	I	<=2	<=1	S	1	I
>32	ESBL	>16	R*	>2	R	1	<=1	S	>1	R

N/R		>16	R	<=0.5	S	>4	>4	R	>1	R
>32	ESBL	>16	R*	>2	R	<=2	<=1	S	1	I
>32	ESBL	>16	R*	>2	R	<=2	<=1	S	1	I
>32	ESBL	>16	R*	>2	R	<=2	<=1	S	<=0.5	S
>32	ESBL	>16	R*	>2	R	<=2	<=1	S	>1	R
>32	R	>16	R	>2	R	<=2	>4	R	>1	R
>32	R	>16	R	>2	R	<=2	4	R	>1	R
>32	R	>16	R	>2	R	<=2	>4	R	>1	R
8	R	>16	R	<=0.5	S	<=2	<=1	S	>1	R
>32	ESBL	>16	R*	>2	R	<=2	<=1	S	>1	R
>32	ESBL	>16	R*	>2	R	<=2	<=1	S	>1	R
>32	ESBL	>16	R*	>2	R	<=2	<=1	S	1	I
>32	ESBL	>16	R*	>2	R	<=2	<=1	S	>1	R
>32	R	>16	R	>2	R	<=2	<=1	S	>1	R
>32	R	>16	R	>2	R	<=2	>4	R	>1	R
>32	ESBL	>16	R*	2	I	<=2	<=1	S	<=0.5	S
>32	R	>16	R	>2	R	<=2	>4	R	>1	R
>32	R	>16	R	1	S	<=2	2	I	>1	R
>32	ESBL	>16	R*	2	I	<=2	<=1	S	<=0.5	S
>32	R	>16	R	1	S	<=2	<=1	S	<=0.5	S
>32	R	>16	R	<=0.5	S	<=2	>4	R	>1	R
>32	ESBL	>16	R*	>2	R	<=2	<=1	S	>1	R
>32	R	>16	R	>2	R	>4	>4	R	>1	R
>32	ESBL	>16	R*	>2	R	<=2	<=1	S	>1	R
>32	R	>16	R	1	S	<=2	>4	R	>1	R
>32	R	>16	R	>2	R	<=2	>4	R	>1	R
>32	ESBL	>16	R*	>2	R	<=2	<=1	S	>1	R
>32	R	>16	R	>2	R	<=2	4	R	>1	R
>32	ESBL	>16	R*	>2	R	<=2	4	R	>1	R
>32	R	>16	R	>2	R	<=2	>4	R	>1	R
>32	ESBL	>16	R*	>2	R	<=2	<=1	S	>1	R
<=1	S	<=4	S	<=0.5	S	<=2	<=1	S	<=0.5	S
>32	R	>16	R	<=0.5	S	<=2	>4	R	>1	R
<=1	S	>16	R	>2	R	<=2	4	R	>1	R
N/R		>16	R	>2	R	<=2	>4	R	>1	R
N/R		>16	R	>2	R	<=2	>4	R	>1	R
>32	ESBL	>16	R*	>2	R	<=2	<=1	S	1	I
>32	R	>16	R	>2	R	<=2	4	R	>1	R
>32	R	>16	R	>2	R	<=2	>4	R	>1	R
>32	ESBL	>16	R*	>2	R	<=2	<=1	S	>1	R
>32	ESBL	>16	R*	>2	R	<=2	<=1	S	1	I
>32	ESBL	>16	R*	>2	R	<=2	<=1	S	1	I
>32	R	>16	R	>2	R	<=2	>4	R	>1	R
N/R		>16	R	>2	R	>4	>4	R	>1	R
N/R		>16	R	<=0.5	S	>4	>4	R	>1	R
>32	ESBL	>16	R*	>2	R	<=2	<=1	S	<=0.5	S
>32	ESBL	>16	R*	2	I	<=2	<=1	S	>1	R
<=1	S	<=4	S	<=0.5	S	<=2	<=1	S	<=0.5	S

>32	R	>16	R	>2	R	<=2		>4	R	>1	R
>32	R	>16	R	>2	R	<=2		>4	R	>1	R
>32	ESBL	>16	R*	>2	R	<=2		<=1	S	>1	R
>32	R	>16	R	>2	R	<=2		>4	R	>1	R
>32	ESBL	>16	R*	>2	R	<=2		<=1	S	>1	R
>32	ESBL	>16	R*	>2	R	<=2		<=1	S	<=0.5	S
N/R		>16	R	<=0.5	S	>4		>4	R	>1	R
>32	ESBL	>16	R*	1	S	<=2		<=1	S	1	I
>32	R	>16	R	>2	R	<=2		4	R	>1	R
>32	R	>16	R	>2	R	<=2		>4	R	>1	R
N/R		>16	R	>2	R	>4		4	R	>1	R
<=1	S	<=4	S	<=0.5	S	<=2		<=1	S	<=0.5	S
>32	ESBL	>16	R*	<=0.5	S	<=2		<=1	S	<=0.5	S
>32	R	16	I	1	S	<=2		>4	R	>1	R
>32	ESBL	>16	R*	>2	R	<=2		<=1	S	>1	R
>32	ESBL	>16	R*	2	I	<=2		<=1	S	<=0.5	S
>32	ESBL	>16	R*	>2	R	<=2		<=1	S	1	I
>32	R	>16	R	1	S	<=2		<=1	S	<=0.5	S
>32	R	>16	R	>2	R	<=2		>4	R	>1	R

>32	R	>16	R	>2	R	<=2	>4	R	>1	R
>32	R	>16	R	>2	R	<=2	>4	R	>1	R
>32	R	>16	R	>2	R	<=2	<=1	S	>1	R
>32	ESBL	>16	R*	>2	R	<=2	<=1	S	1	I
>32	ESBL	>16	R*	>2	R	<=2	<=1	S	<=0.5	S
>32	R	>16	R	>2	R	<=2	>4	R	>1	R
>32	R	>16	R	1	S	<=2	>4	R	>1	R
>32	R	>16	R	2	I	<=2	2	I	>1	R
>32	ESBL	>16	R*	1	S	<=2	<=1	S	<=0.5	S
>32	R	>16	R	1	S	<=2	4	R	>1	R
>32	R	>16	R	1	S	<=2	4	R	>1	R
>32	R	>16	R	>2	R	<=2	<=1	S	>1	R
8	R	>16	R	<=0.5	S	>4	<=1	S	<=0.5	S
>32	R	>16	R	>2	R	<=2	>4	R	>1	R
>32	ESBL	>16	R*	<=0.5	S	>4	<=1	S	<=0.5	S

>32	R	>16	R	>2	R	<=2	>4	R	>1	R
>32	R	>16	R	>2	R	<=2	>4	R	>1	R
>32	R	>16	R	>2	R	<=2	>4	R	>1	R
>32	ESBL	>16	R*	>2	R	<=2	<=1	S	<=0.5	S
<=1	S	<=4	S	<=0.5	S	>4	>4	R	>1	R
>32	R	>16	R	>2	R	<=2	4	R	>1	R
>32	R	>16	R	>2	R	<=2	>4	R	>1	R
>32	ESBL	>16	R*	>2	R	<=2	<=1	S	<=0.5	S
>32	R	>16	R	>2	R	<=2	>4	R	>1	R
>32	R	>16	R	>2	R	64	>4	R	>1	R

<=1	S	8	S	>2	R	<=2	<=1	S	>1	R

>32	R	>16	R	>2	R	<=2		<=1	S	>1	R
>32	R	>16	R	>2	R	<=2		>4	R	>1	R
16	R	>16	R	>2	R	<=2		<=1	S	>1	R
>32	R	>16	R	>2	R	<=2		>4	R	>1	R
>32	R	>16	R	1	S	<=2		4	R	>1	R
>32	R	>16	R	>2	R	1	I	>4	R	>1	R
>32	ESBL	>16	R*	2	I	32	R	<=1	S	1	I
>32	R	>16	R	>2	R	<=2		>4	R	>1	R
>32	ESBL	>16	R*	>2	R	<=2		<=1	S	>1	R
>32	R	>16	R	1	S	<=2		>4	R	>1	R
>32	R	>16	R	>2	R	<=2		>4	R	>1	R
>32	R	>16	R	>2	R	<=2		>4	R	>1	R
>32	R	>16	R	>2	R	<=2		>4	R	>1	R
>32	R	>16	R	>2	R	<=2		>4	R	>1	R
>32	R	>16	R	1	S	<=2		4	R	>1	R
>32	R	>16	R	>2	R	<=2		>4	R	>1	R
>32	ESBL	>16	R*	<=0.5	S	<=2		4	R	>1	R
>32	R	>16	R	>2	R	<=2		<=1	S	>1	R
16	R	>16	R	<=0.5	S	2	R	>4	R	>1	R
>32	R	>16	R	>2	R	<=2		4	R	>1	R
>32	R	>16	R	1	S	<=2		>4	R	>1	R
>32	R	>16	R	>2	R	1	I	>4	R	>1	R
32	R	>16	R	>2	R	>4		4	R	>1	R
>32	R	>16	R	1	S	<=2		>4	R	>1	R
>32	R	>16	R	<=0.5	S	<=2		4	R	>1	R
<=1	S	<=4	S	<=0.5	S	<=2		<=1	S	<=0.5	S
8	R	>16	R	>2	R	>4		2	I	1	I
>32	R	>16	R	1	S	>128	R	>4	R	>1	R
>32	R	>16	R	2	I	1	I	>4	R	>1	R
>32	ESBL	>16	R*	2	I	<=2		<=1	S	<=0.5	S
<=1	S	8	S	>2	R	<=2		<=1	S	>1	R
>32	R	>16	R	2	I	<=2		>4	R	>1	R
>32	R	>16	R	>2	R	<=2		>4	R	>1	R
>32	R	>16	R	>2	R	<=2		>4	R	>1	R
>32	R	>16	R	>2	R	<=2		>4	R	>1	R
>32	ESBL	>16	R*	>2	R	<=2		<=1	S	1	I
>32	ESBL	>16	R*	>2	R	<=2		4	R	>1	R
>32	ESBL	>16	R*	>2	R	<=2		2	I	>1	R
>32	ESBL	>16	R*	>2	R	<=2		2	I	>1	R
>32	R	>16	R	>2	R	1	I	>4	R	>1	R
>32	ESBL	>16	R*	>2	R	<=2		4	R	>1	R
>32	R	>16	R	>2	R	<=2		4	R	>1	R
>32	R	>16	R	>2	R	4	R	>4	R	>1	R
>32	R	>16	R	2	I	<=2		>4	R	>1	R

>32	ESBL	>16	R*	>2	R	<=2		<=1	S	>1	R
32	R	>16	R	<=0.5	S	<=2		2	I	>1	R
>32	ESBL	>16	R*	<=0.5	S	>4		<=1	S	<=0.5	S
>32	R	>16	R	>2	R	<=2		>4	R	>1	R
>32	ESBL	>16	R*	>2	R	<=2		>4	R	>1	R
>32	ESBL	>16	R*	>2	R	32	R	4	R	>1	R
>32	R	>16	R	>2	R	<=2		>4	R	>1	R
>32	R	>16	R	2	I	<=2		2	I	>1	R
>32	R	>16	R	>2	R	<=2		2	I	>1	R
N/R		>16	R	<=0.5	S	>4		<=1	S	<=0.5	S
>32	R	>16	R	>2	R	1	I	>4	R	>1	R
>32	R	>16	R	>2	R	1	I	4	R	>1	R
>32	R	>16	R	>2	R	<=2		>4	R	>1	R
>32	R	>16	R	>2	R	<=2		>4	R	>1	R
2	I	>16	R	<=0.5	S	1	I	2	I	1	I
>32	ESBL	>16	R*	>2	R	<=2		<=1	S	1	I
>32	R	>16	R	>2	R	>128	R	4	R	>1	R
>32	ESBL	>16	R*	>2	R	<=2		<=1	S	1	I
>32	ESBL	>16	R*	>2	R	<=2		<=1	S	<=0.5	S
>32	ESBL	>16	R*	>2	R	<=2		<=1	S	>1	R
>32	ESBL	>16	R*	<=0.5	S	<=2		4	R	>1	R
>32	R	>16	R	>2	R	2	I	>4	R	>1	R
>32	R	>16	R	1	S	<=2		4	R	>1	R
N/R		>16	R	>2	R	>4		>4	R	>1	R
>32	R	>16	R	>2	R	1	I	>4	R	>1	R
>32	R	>16	R	>2	R	<=2		>4	R	>1	R
>32	R	>16	R	2	I	<=2		>4	R	>1	R
>32	R	>16	R	>2	R	<=2		4	R	>1	R
>32	R	>16	R	>2	R	4	R	>4	R	>1	R
>32	ESBL	>16	R*	>2	R	<=2		4	R	>1	R
>32	R	>16	R	>2	R	<=2		>4	R	>1	R
>32	R	>16	R	>2	R	>4		>4	R	>1	R
>32	R	>16	R	>2	R	<=2		>4	R	>1	R
		>16	R	>2	R	>4		<=1	S	<=0.5	S
>32	R	>16	R	>2	R	<=2		>4	R	>1	R
>32	R	>16	R	>2	R	<=2		4	R	>1	R
>32	ESBL	>16	R*	2	I	<=2		4	R	>1	R
>32	R	>16	R	>2	R	<=2		>4	R	>1	R
>32	ESBL	>16	R*	>2	R	<=2		4	R	>1	R
>32	R	>16	R	>2	R	<=2		4	R	>1	R
>32	ESBL	>16	R*	2	I	<=2		<=1	S	>1	R
N/R		>16	R	>2	R	>4		4	R	>1	R
>32	R	>16	R	>2	R	<=2		>4	R	>1	R
>32	R	>16	R	1	S	<=2		>4	R	>1	R
>32	R	>16	R	>2	R	2	I	>4	R	>1	R
>32	ESBL	>16	R*	2	I	<=2		<=1	S	>1	R
>32	R	>16	R	>2	R	<=2		>4	R	>1	R
>32	R	>16	R	>2	R	1	I	>4	R	>1	R
>32	R	>16	R	>2	R	1	I	>4	R	>1	R
N/R		>16	R	2	I	>4		<=1	S	<=0.5	S
>32	R	>16	R	>2	R	<=2		>4	R	>1	R
>32	ESBL	>16	R*	>2	R	<=2		<=1	S	1	I
>32	R	>16	R	>2	R	<=2		4	R	>1	R
>32	ESBL	>16	R*	2	I	<=2		<=1	S	<=0.5	S
>32	R	>16	R	>2	R	>4		>4	R	>1	R

>32	ESBL	>16	R*	<=0.5	S	<=2		<=1	S	<=0.5	S
>32	ESBL	>16	R*	2	I	<=2		<=1	S	<=0.5	S
>32	ESBL	>16	R*	>2	R	<=2		<=1	S	>1	R
>32	R	>16	R	>2	R	1	I	>4	R	>1	R
>32	ESBL	>16	R*	>2	R	<=2		<=1	S	1	I
>32	ESBL	>16	R*	>2	R	<=2		2	I	>1	R
>32	R	>16	R	>2	R	<=2		>4	R	>1	R
>32	R	>16	R	>2	R	32	R	>4	R	>1	R
>32	ESBL	>16	R*	>2	R	<=2		<=1	S	>1	R
>32	R	>16	R	>2	R	<=2		<=1	S	>1	R
>32	R	>16	R	>2	R	<=2		<=1	S	>1	R
>32	R	>16	R	>2	R	<=2		>4	R	>1	R
>32	ESBL	>16	R*	>2	R	<=2		<=1	S	1	I
>32	R	>16	R	>2	R	<=2		>4	R	>1	R
>32	R	>16	R	1	S	<=2		4	R	>1	R
>32	R	>16	R	>2	R	<=2		>4	R	>1	R
>32	R	>16	R	>2	R	<=2		<=1	S	>1	R
>32	R	>16	R	>2	R	<=2		>4	R	>1	R
>32	R	>16	R	>2	R	<=2		>4	R	>1	R
>32	R	>16	R	2	I	<=2		>4	R	>1	R
>32	R	>16	R	1	S	<=2		>4	R	>1	R
N/R		>16	R	2	I	>4		<=1	S	<=0.5	S
N/R		>16	R	2	I	>4		<=1	S	<=0.5	S
>32	R	>16	R	1	S	<=2		>4	R	>1	R
>32	R	>16	R	1	S	<=2		>4	R	>1	R
>32	R	>16	R	2	I	<=2		>4	R	>1	R
>32	R	>16	R	>2	R	<=2		<=1	S	>1	R
>32	R	>16	R	<=0.5	S	<=2		<=1	S	>1	R
16	R	>16	R	<=0.5	S	<=2		<=1	S	>1	R
>32	R	>16	R	>2	R	<=2		<=1	S	>1	R
>32	R	>16	R	>2	R	<=2		>4	R	>1	R
>32	R	>16	R	>2	R	<=2		>4	R	>1	R
>32	R	>16	R	>2	R	<=2		4	R	>1	R
>32	ESBL	>16	R*	2	I	<=2		<=1	S	>1	R
>32	ESBL	>16	R*	>2	R	<=2		<=1	S	1	I
N/R		>16	R	>2	R	>4		>4	R	>1	R
>32	R	>16	R	>2	R	<=2		>4	R	>1	R
>32	R	>16	R	>2	R	>4		>4	R	>1	R
32	R	>16	R	1	S	<=2		<=1	S	>1	R
N/R		>16	R	>2	R	>4		<=1	S	1	I
>32	R	>16	R	2	I	<=2		>4	R	>1	R
>32	R	>16	R	>2	R	<=2		<=1	S	>1	R
>32	R	>16	R	>2	R	<=2		>4	R	>1	R
>32	R	>16	R	>2	R	<=2		<=1	S	>1	R
>32	R	>16	R	>2	R	<=2		<=1	S	>1	R
<=1	S	<=4	S	1	S	1	I	<=1	S	1	I
>32	R	>16	R	>2	R	<=2		>4	R	>1	R
N/R		>16	R	>2	R	>4		4	R	>1	R
<=1	S	<=4	S	2	I	<=2		<=1	S	>1	R
>32	R	>16	R	>2	R	<=2		4	R	>1	R
>32	R	>16	R	>2	R	8	R	>4	R	>1	R
<=1	S	<=4	S	<=0.5	S	<=2		<=1	S	<=0.5	S
>32	R	>16	R	>2	R	<=2		>4	R	>1	R

>32	R	>16	R	>2	R	<=2		>4	R	>1	R
>32	R	>16	R	>2	R	<=2		>4	R	>1	R
N/R		>16	R	>2	R	>4		>4	R	>1	R
>32	R	>16	R	>2	R	<=2		<=1	S	>1	R
N/R		>16	R	1	S	>4		<=1	S	<=0.5	S
>32	ESBL	>16	R*	>2	R	<=2		<=1	S	>1	R
>32	R	>16	R	>2	R	<=2		>4	R	>1	R
16	R	>16	R	>2	R	<=2		<=1	S	>1	R
N/R		>16	R	<=0.5	S	>4		>4	R	>1	R
>32	ESBL	>16	R*	>2	R	<=2		>4	R	>1	R
>32	ESBL	>16	R*	1	S	16	R	<=1	S	>1	R
>32	ESBL	>16	R*	>2	R	<=2		<=1	S	>1	R
>32	R	>16	R	>2	R	1	I	<=1	S	>1	R
>32	ESBL	>16	R*	1	S	<=2		<=1	S	1	I
>32	R	>16	R	1	S	<=2		>4	R	>1	R
N/R		>16	R	2	I	>4		2	I	1	I
>32	R	>16	R	1	S	<=2		4	R	>1	R
N/R		>16	R	>2	R	>4		<=1	S	<=0.5	S
2	I	>16	R	>2	R	<=2		>4	R	>1	R
>32	R	>16	R	>2	R	<=2		>4	R	>1	R
N/R		>16	R	>2	R	>4		4	R	>1	R
>32	R	>16	R	>2	R	32	R	>4	R	>1	R
>32	R	>16	R	>2	R	32	R	>4	R	>1	R
>32	R	>16	R	>2	R	<=2		>4	R	>1	R
>32	ESBL	>16	R*	>2	R	<=2		<=1	S	>1	R
>32	R	>16	R	<=0.5	S	<=2		<=1	S	>1	R
>32	ESBL	>16	R*	>2	R	<=2		<=1	S	1	I
>32	R	>16	R	>2	R	<=2		2	I	>1	R
>32	ESBL	>16	R*	>2	R	<=2		4	R	>1	R
N/R		>16	R	>2	R	>4		>4	R	>1	R
>32	ESBL	>16	R*	>2	R	2	I	2	I	>1	R
>32	R	>16	R	<=0.5	S	<=2		>4	R	>1	R
>32	R	>16	R	>2	R	<=2		>4	R	>1	R
N/R		>16	R	<=0.5	S	>4		<=1	S	1	I
>32	R	>16	R	>2	R	1	I	>4	R	>1	R
N/R		>16	R	2	I	>4		<=1	S	<=0.5	S
N/R		>16	R	>2	R	>4		>4	R	>1	R
N/R		>16	R*	<=0.5	S	>4		>4	R	>1	R
>32	R	>16	R	2	I	<=2		>4	R	>1	R
>32	ESBL	>16	R*	>2	R	<=2		<=1	S	>1	R
>32	ESBL	>16	R*	>2	R	<=2		<=1	S	<=0.5	S
>32	R	>16	R	>2	R	<=2		<=1	S	>1	R
>32	ESBL	>16	R*	>2	R	<=2		<=1	S	<=0.5	S
>32	R	>16	R	>2	R	1	I	>4	R	>1	R
>32	R	>16	R	>2	R	2	I	4	R	>1	R
N/R		>16	R*	>2	R	4		2	I	1	I
>32	R	>16	R	>2	R	<=2		>4	R	>1	R
>32	R	>16	R	>2	R	<=2		>4	R	>1	R
>32	R	>16	R	>2	R	<=2		>4	R	>1	R
>32	R	>16	R	>2	R	<=2		>4	R	>1	R
>32	R	>16	R	>2	R	<=2		>4	R	>1	R
>32	R	>16	R	>2	R	64	R	>4	R	>1	R

<=1	S	<=4	S	>2	R	<=2		>4	R	>1	R

>32	ESBL	>16	R*	<=0.5	S	<=2		<=1	S	<=0.5	S
>32	ESBL	>16	R*	>2	R	1	I	<=1	S	>1	R
>32	ESBL	>16	R*	>2	R	<=2		<=1	S	>1	R
>32	ESBL	>16	R*	<=0.5	S	<=2		<=1	S	<=0.5	S
>32	ESBL	>16	R*	>2	R	<=2		<=1	S	1	I
>32	R	>16	R	>2	R	64	R	>4	R	>1	R
>32	ESBL	>16	R	>2	R	<=2		<=1	S	1	I
>32	ESBL	>16	R*	>2	R	<=2		4	R	>1	R
>32	ESBL	>16	R*	>2	R	<=2		<=1	S	>1	R
N/R		>16	R	>2	R	<=2		>4	R	>1	R
>32	ESBL	>16	R*	2	I	<=2		<=1	S	<=0.5	S
>32	R	>16	R	>2	R	<=2		>4	R	>1	R
>32	R	>16	R	>2	R	>128	R	>4	R	>1	R
>32	R	>16	R	>2	R	<=2		4	R	>1	R

>32	ESBL	>16	R*	>2	R	<=2		<=1	S	1	I
>32	R	>16	R	>2	R	1	I	>4	R	>1	R
>32	R	>16	R	>2	R	<=2		>4	R	>1	R
>32	R	>16	R	>2	R	<=2		>4	R	>1	R
>32	R	>16	R	>2	R	<=2		>4	R	>1	R
>32	R	>16	R	1	S	<=2		>4	R	>1	R
N/R		>16	R	<=0.5	S	>4		<=1	S	<=0.5	S
>32	R	>16	R	>2	R	32	R	>4	R	>1	R
>32	R	>16	R	2	I	<=2		>4	R	>1	R
>32	R	>16	R	>2	R	<=2		>4	R	>1	R
>32	R	>16	R	>2	R	>4		<=1	S	1	I
>32	ESBL	>16	R*	2	I	<=2		<=1	S	1	I
>32	R	>16	R	>2	R	1	I	>4	R	>1	R
>32	R	>16	R	>2	R	>128	R	>4	R	>1	R
>32	R	>16	R	>2	R	1	I	4	R	>1	R
>32	R	>16	R	>2	R	1	I	>4	R	>1	R
>32	ESBL	>16	R*	1	S	<=2		<=1	S	>1	R
>32	R	>16	R	>2	R	1	I	>4	R	>1	R
>32	ESBL	>16	R*	>2	R	<=2		4	R	>1	R
>32	ESBL	>16	R*	>2	R	<=2		<=1	S	1	I
N/R		>16	R	<=0.5	S	>4		>4	R	>1	R

>32	R	>16	R	2	I	<=2		>4	R	>1	R
>32	R	>16	R	>2	R	<=2		4	R	>1	R
>32	ESBL	>16	R*	>2	R	<=2		<=1	S	1	I
>32	R	>16	R	>2	R	<=2		>4	R	>1	R
>32	R	>16	R	>2	R	<=2		>4	R	>1	R
>32	R	>16	R	>2	R	<=2		>4	R	>1	R
>32	R	>16	R	>2	R	<=2		>4	R	>1	R
N/R		>16	R	<=0.5	S	>4		>4	R	>1	R
>32	R	>16	R	>2	R	<=2		4	R	>1	R
>32	R	>16	R	>2	R	1	I	4	R	>1	R
>32	R	>16	R	>2	R	<=2		2	I	>1	R
>32	ESBL	>16	R*	>2	R	<=2		2	I	1	I
N/R		>16	R	>2	R	>4		>4	R	>1	R
>32	ESBL	>16	R*	>2	R	<=2		4	R	>1	R
>32	R	>16	R	>2	R	1	I	>4	R	>1	R
>32	R	>16	R	>2	R	<=2		>4	R	>1	R
>32	R	>16	R	>2	R	<=2		<=1	S	1	I
>32	R	>16	R	>2	R	<=2		4	R	>1	R
>32	R	>16	R	>2	R	<=2		>4	R	>1	R
>32	R	>16	R	2	I	<=2		>4	R	>1	R
>32	R	>16	R	>2	R	<=2		<=1	S	>1	R
>32	R	>16	R	>2	R	<=2		4	R	>1	R
>32	R	>16	R	>2	R	<=2		>4	R	>1	R
>32	R	>16	R	>2	R	<=2		<=1	S	>1	R
>32	ESBL	>16	R*	>2	R	<=2		<=1	S	>1	R
>32	R	>16	R	>2	R	<=2		>4	R	>1	R
>32	ESBL	>16	R*	2	I	<=2		<=1	S	>1	R
>32	R	>16	R	>2	R	<=2		>4	R	>1	R
>32	R	>16	R	>2	R	<=2		>4	R	>1	R
>32	R	>16	R	>2	R	4	R	2	I	>1	R
>32	ESBL	>16	R*	>2	R	<=2		<=1	S	1	I
>32	ESBL	>16	R*	>2	R	<=2		<=1	S	<=0.5	S
>32	R	>16	R	1	S	<=2		>4	R	>1	R
>32	R	>16	R	2	I	>128	R	>4	R	>1	R
>32	ESBL	>16	R*	>2	R	<=2		<=1	S	1	I
>32	R	>16	R	>2	R	<=2		>4	R	>1	R
>32	ESBL	>16	R*	>2	R	<=2		<=1	S	1	I
>32	ESBL	>16	R*	>2	R	1	I	<=1	S	>1	R
>32	R	>16	R	>2	R	<=2		>4	R	>1	R
>32	R	>16	R	>2	R	<=2		4	R	>1	R
<=1	S	8	S	>2	R	<=2		<=1	S	1	I
>32	R	>16	R	>2	R	<=2		>4	R	>1	R
>32	ESBL	>16	R*	>2	R	<=2		<=1	S	<=0.5	S
>32	ESBL	>16	R*	>2	R	<=2		4	R	>1	R
>32	R	>16	R	1	S	<=2		4	R	>1	R
>32	ESBL	>16	R*	>2	R	<=2		<=1	S	1	I
>32	ESBL	>16	R*	>2	R	<=2		<=1	S	<=0.5	S
>32	R	>16	R	1	S	<=2		>4	R	>1	R
>32	ESBL	>16	R*	>2	R	<=2		2	I	>1	R
>32	R	>16	R	>2	R	<=2		4	R	>1	R
>32	ESBL	>16	R*	>2	R	<=2		<=1	S	>1	R
>32	R	>16	R	>2	R	<=2		4	R	>1	R
>32	ESBL	>16	R*	>2	R	<=2		<=1	S	>1	R
>32	R	>16	R	>2	R	<=2		>4	R	>1	R
>32	R	>16	R	>2	R	<=2		>4	R	>1	R

>32	ESBL	>16	R*	>2	R	<=2	<=1	S	1	I

>32	ESBL	>16	R*	1	S	<=2	4	R	>1	R

>32	ESBL	>16	R*	>2	R	<=2		<=1	S	1	I
>32	ESBL	>16	R*	>2	R	<=2		4	R	>1	R
>32	ESBL	>16	R*	>2	R	<=2		<=1	S	>1	R
>32	R	>16	R	>2	R	<=2		4	R	>1	R
>32	R	>16	R	>2	R	1	I	>4	R	>1	R
>32	R	>16	R	<=0.5	S	<=2		>4	R	>1	R

>32	R	>16	R	>2	R	<=2		>4	R	>1	R
>32	R	>16	R	>2	R	<=2		>4	R	>1	R
>32	R	>16	R	>2	R	<=2		>4	R	>1	R
>32	R	>16	R	>2	R	<=2		4	R	>1	R
>32	ESBL	>16	R*	>2	R	<=2		<=1	S	>1	R
>32	R	>16	R	>2	R	<=2		>4	R	>1	R
>32	R	>16	R	>2	R	<=2		>4	R	>1	R
>32	R	>16	R	1	S	<=2		4	R	>1	R
>32	ESBL	>16	R*	>2	R	<=2		<=1	S	<=0.5	S
>32	ESBL	>16	R*	>2	R	<=2		<=1	S	>1	R
>32	ESBL	>16	R*	2	I	<=2		<=1	S	1	I
>32	R	>16	R	>2	R	<=2		>4	R	>1	R
>32	ESBL	>16	R*	>2	R	<=2		2	I	>1	R
>32	ESBL	>16	R*	2	I	1	I	2	I	>1	R
>32	ESBL	>16	R*	1	S	4	R	4	R	>1	R
>32	ESBL	>16	R*	>2	R	8	R	4	R	>1	R
>32	R	>16	R	>2	R	<=2		2	I	>1	R
N/R		>16	R*	>2	R	<=2		<=1	S	1	I
>32	R	>16	R	2	I	<=2		<=1	S	>1	R
>32	R	>16	R	<=0.5	S	<=2		<=1	S	>1	R
>32	R	>16	R	>2	R	<=2		>4	R	>1	R
<=1	S	<=4	S	<=0.5	S	<=2		<=1	S	<=0.5	S
8	ESBL	8	R*	<=0.5	S	<=2		<=1	S	<=0.5	S
16	ESBL	16	R*	<=0.5	S	<=2		<=1	S	<=0.5	S
>32	ESBL	>16	R*	2	I	<=2		<=1	S	<=0.5	S
16	R	>16	R	<=0.5	S	<=2		<=1	S	<=0.5	S
>32	R	>16	R	>2	R	<=2		<=1	S	>1	R
<=1	S	16	I	>2	R	<=2		<=1	S	1	I
>32	ESBL	>16	R*	>2	R	<=2		<=1	S	>1	R
>32	R	>16	R	>2	R	<=2		4	R	>1	R
32	ESBL	>16	R*	>2	R	<=2		<=1	S	<=0.5	S
>32	R	>16	R	>2	R	<=2		>4	R	>1	R
>32	R	>16	R	2	I	<=2		>4	R	>1	R
>32	ESBL	>16	R*	>2	R	<=2		<=1	S	>1	R
>32	R	>16	R	>2	R	<=2		4	R	>1	R
>32	R	>16	R	>2	R	<=2		4	R	>1	R
>32	R	>16	R	>2	R	<=2		>4	R	>1	R
2	I	>16	R	>2	R	<=2		4	R	>1	R
>32	R	>16	R	>2	R	<=2		>4	R	>1	R
>32	ESBL	>16	R*	>2	R	<=2		4	R	1	I
>32	ESBL	>16	R*	<=0.5	S	<=2		<=1	S	1	I
>32	R	>16	R	>2	R	1	I	4	R	>1	R
>32	ESBL	>16	R*	2	I	<=2		<=1	S	<=0.5	S
>32	R	>16	R	1	S	<=2		4	R	>1	R
>32	R	>16	R	<=0.5	S	<=2		<=1	S	1	I
>32	R	>16	R	>2	R	<=2		>4	R	>1	R
<=1	S	<=4	S	2	I	<=2		<=1	S	1	I
>32	R	>16	R	>2	R	<=2		4	R	>1	R
>32	ESBL	>16	R*	>2	R	<=2		<=1	S	<=0.5	S
>32	R	>16	R	>2	R	<=2		>4	R	>1	R
>32	R	>16	R	>2	R	<=2		>4	R	>1	R

>32	R	>16	R	>2	R	1	I	>4	R	>1	R
>32	R	>16	R	>2	R	16	R	>4	R	>1	R
>32	ESBL	>16	R*	<=0.5	S	<=2		<=1	S	1	I
>32	ESBL	>16	R*	>2	R	<=2		<=1	S	>1	R
>32	ESBL	>16	R*	>2	R	<=2		<=1	S	<=0.5	S

>32	ESBL	>16	R*	>2	R	16	R	<=1	S	>1	R
>32	R	>16	R	2	I	8	R	<=1	S	>1	R
>32	R	>16	R	2	I	<=2		4	R	>1	R
>32	ESBL	>16	R*	2	I	<=2		<=1	S	<=0.5	S
<=1	S	8	S	<=0.5	S	<=2		<=1	S	<=0.5	S
<=1	S	<=4	S	<=0.5	S	<=2		<=1	S	<=0.5	S
>32	R	>16	R	>2	R	<=2		4	R	>1	R
>32	ESBL	>16	R*	2	I	<=2		<=1	S	>1	R
>32	ESBL	>16	R*	>2	R	<=2		<=1	S	1	I
>32	ESBL	>16	R*	1	S	<=2		<=1	S	1	I
>32	R	>16	R	>2	R	<=2		>4	R	>1	R
>32	R	>16	R	>2	R	1	I	>4	R	>1	R
>32	R	>16	R	>2	R	<=2		>4	R	>1	R
>32	ESBL	>16	R*	>2	R	<=2		4	R	>1	R
8	ESBL	<=4	R*	<=0.5	S	<=2		4	R	<=0.5	S
>32	ESBL	>16	R*	>2	R	<=2		<=1	S	>1	R
>32	R	>16	R	>2	R	<=2		>4	R	>1	R
>32	ESBL	>16	R*	>2	R	<=2		<=1	S	<=0.5	S

>32	R	>16	R	>2	R	<=2		>4	R	>1	R
>32	R	>16	R	<=0.5	S	<=2		>4	R	>1	R
>32	ESBL	>16	R*	2	I	<=2		<=1	S	<=0.5	S
>32	R	>16	R	>2	R	<=2		4	R	>1	R
>32	R	>16	R	>2	R	<=2		>4	R	>1	R
>32	R	>16	R	<=0.5	S	<=2		>4	R	>1	R
>32	ESBL	>16	R*	<=0.5	S	<=2		<=1	S	1	I
>32	R	>16	R	>2	R	<=2		>4	R	>1	R
>32	R	>16	R	>2	R	1	I	4	R	>1	R
>32	R	>16	R	1	I	1	S	>4	R	>1	R
>32	ESBL	>16	R*	>2	R	<=2		<=1	S	>1	R
<=1	S	<=4	S	<=0.5	S	<=2		<=1	S	<=0.5	S
>32	R	>16	R	1	S	<=2		>4	R	>1	R
>32	ESBL	>16	R*	2	I	1	I	<=1	S	1	I
>32	R	>16	R	>2	R	<=2		>4	R	>1	R
>32	R	>16	R	>2	R	<=2		>4	R	>1	R
>32	R	>16	R	>2	R	<=2		>4	R	>1	R
>32	R	>16	R	>2	R	<=2		>4	R	>1	R
>32	R	>16	R	>2	R	16	R	>4	R	>1	R
>32	R	>16	R	>2	R	<=2		>4	R	>1	R
>32	ESBL	>16	R*	2	I	<=2		<=1	S	1	I
>32	ESBL	>16	R*	>2	R	<=2		<=1	S	1	I
>32	ESBL	>16	R*	>2	R	<=2		4	R	>1	R
>32	R	>16	R	>2	R	<=2		4	R	>1	R
N/R		>16	R*	>2	R	>4		2	I	>1	R
<=1	S	8	S	<=0.5	S	<=2		<=1	S	>1	R
>32	R	>16	R	<=0.5	S	<=2		>4	R	>1	R
>32	R	>16	R	>2	R	<=2		>4	R	>1	R
>32	ESBL	>16	R*	>2	R	<=2		4	R	>1	R
>32	ESBL	>16	R*	1	S	<=2		<=1	S	>1	R
>32	R	>16	R	>2	R	<=2		>4	R	>1	R
>32	ESBL	>16	R*	2	I	<=2		<=1	S	>1	R
>32	R	>16	R	>2	R	<=2		4	R	>1	R
>32	ESBL	>16	R*	>2	R	<=2		<=1	S	>1	R
>32	ESBL	>16	R*	>2	R	<=2		4	R	>1	R
>32	R	>16	R	>2	R	<=2		4	R	>1	R
>32	ESBL	>16	R*	>2	R	1	I	<=1	S	<=0.5	S
>32	ESBL	>16	R*	>2	R	<=2		<=1	S	1	I
>32	R	>16	R	>2	R	1	I	>4	R	>1	R
>32	R	>16	R	>2	R	<=2		>4	R	>1	R
>32	R	>16	R	<=0.5	S	<=2		4	R	>1	R
>32	ESBL	>16	R*	>2	R	<=2		<=1	S	1	I
>32	ESBL	>16	R*	2	I	<=2		<=1	S	1	I
>32	R	>16	R	>2	R	<=2		2	I	>1	R
>32	R	>16	R	>2	R	<=2		>4	R	>1	R
>32	R	>16	R	<=0.5	S	<=2		2	I	1	I
>32	R	>16	R	<=0.5	S	<=2		2	I	<=0.5	S
>32	R	>16	R	>2	R	1	I	4	R	>1	R
>32	R	>16	R	>2	R	<=2		>4	R	>1	R
>32	R	>16	R	1	S	1	I	2	I	>1	R
>32	R	>16	R	>2	R	<=2		>4	R	>1	R
>32	ESBL	>16	R*	>2	R	<=2		2	I	>1	R
>32	ESBL	>16	R*	1	S	<=2		<=1	S	1	I
>32	R	>16	R	>2	R	16	R	>4	R	>1	R
>32	R	>16	R	>2	R	<=2		4	R	>1	R

>32	R	>16	R	<=0.5	S	1	I	<=1	S	>1	R
>32	R	>16	R	>2	R	<=2		2	I	>1	R
>32	R	>16	R	<=0.5	S	<=2		>4	R	>1	R
>32	R	>16	R	<=0.5	S	<=2		>4	R	>1	R

>32	ESBL	>16	R*	2	I	1	I	<=1	S	>1	R
>32	R	>16	R	>2	R	0.5	I	4	R	>1	R
>32	ESBL	>16	R*	>2	R	>4		<=1	S	>1	R
>32	ESBL	>16	R*	>2	R	>4		<=1	S	>1	R
>32	R	>16	R	>2	R	<=2		>4	R	>1	R
>32	ESBL	>16	R*	>2	R	<=2		<=1	S	<=0.5	S
16	R	>16	R	<=0.5	S	<=2		<=1	S	>1	R

>32	ESBL	>16	R*	>2	R	<=2	<=1	S	>1	R
>32	R	>16	R	>2	R	<=2	4	R	>1	R
>32	R	>16	R	>2	R	4	>4	R	>1	R
>32	ESBL	>16	R*	>2	R	<=2	<=1	S	>1	R
<=1	S	8	S	2	I	<=2	<=1	S	1	I
>32	ESBL	>16	R*	2	I	<=2	<=1	S	>1	R
>32	ESBL	>16	R*	>2	R	<=2	<=1	S	1	I
>32	ESBL	>16	R*	>2	R	<=2	<=1	S	>1	R
<=1	S	<=4	S	2	I	<=2	<=1	S	<=0.5	S
>32	ESBL	>16	R*	>2	R	<=2	4	R	>1	R
>32	R	>16	R	>2	R	<=2	4	R	>1	R
N/R		>16	R	>2	R	>4	2	I	>1	R
>32	ESBL	>16	R*	>2	R	<=2	<=1	S	1	I
>32	R	>16	R	>2	R	<=2	>4	R	>1	R
>32	R	>16	R	>2	R	4	>4	R	>1	R
>32	ESBL	>16	R*	>2	R	<=2	<=1	S	1	I
>32	R	>16	R	>2	R	<=2	>4	R	>1	R
>32	ESBL	>16	R*	2	I	<=2	<=1	S	1	I
N/R		>16	R	>2	R	>4	>4	R	>1	R
>32	R	>16	R	<=0.5	S	<=2	<=1	S	>1	R
>32	R	>16	R	>2	R	<=2	4	R	>1	R
>32	ESBL	>16	R*	>2	R	<=2	<=1	S	<=0.5	S
>32	ESBL	>16	R*	>2	R	<=2	2	I	>1	R
>32	ESBL	>16	R*	2	I	<=2	<=1	S	1	I
<=1	R*	<=4	R*	<=0.5	S	<=2	<=1	S	<=0.5	S
>32	ESBL	>16	R*	>2	R	<=2	2	I	>1	R
>32	ESBL	>16	R*	>2	R	<=2	<=1	S	<=0.5	S
>32	R	>16	R	>2	R	<=2	<=1	S	>1	R
>32	R	>16	R	>2	R	<=2	<=1	S	>1	R
2	I	16	I	>2	R	<=2	4	R	>1	R
>32	R	>16	R	>2	R	<=2	2	I	>1	R
>32	R	>16	R	>2	R	<=2	4	R	>1	R
N/R		>16	R*	2	I	>4	<=1	S	1	I
16	R	>16	R	<=0.5	S	<=2	<=1	S	>1	R
>32	ESBL	>16	R*	>2	R	<=2	<=1	S	1	I
>32	R	>16	R	>2	R	<=2	>4	R	>1	R
>32	ESBL	>16	R*	>2	R	<=2	<=1	S	>1	R
>32	ESBL	>16	R*	>2	R	<=2	<=1	S	1	I
N/R		>16	R	>2	R	>4	>4	R	>1	R
>32	R	>16	R	>2	R	<=2	4	R	>1	R
>32	R	>16	R	2	I	<=2	>4	R	>1	R
>32	R	>16	R	>2	R	<=2	>4	R	>1	R
>32	ESBL	>16	R*	>2	R	<=2	<=1	S	>1	R
>32	ESBL	>16	R*	>2	R	<=2	<=1	S	>1	R
4	R	>16	R	>2	R	>4	<=1	S	<=0.5	S
>32	ESBL	>16	R*	<=0.5	S	<=2	<=1	S	<=0.5	S
32	R	>16	R	>2	R	<=2	<=1	S	1	I
>32	R	>16	R	>2	R	<=2	<=1	S	>1	R
>32	R	>16	R	>2	R	<=2	4	R	>1	R
>32	ESBL	>16	R*	>2	R	<=2	<=1	S	>1	R
>32	ESBL	>16	R*	>2	R	<=2	<=1	S	>1	R
>32	R	>16	R	<=0.5	S	<=2	>4	R	>1	R
>32	ESBL	>16	R*	2	I	<=2	<=1	S	>1	R
2	I	16	I	1	S	>4	2	I	>1	R
>32	R	>16	R	>2	R	4	>4	R	>1	R
>32	R	>16	R	>2	R	<=2	4	R	>1	R

N/R		>16	R	<=0.5	S	>4	<=1	S	<=0.5	S
>32	R	>16	R	>2	R	>4	<=1	S	>1	R
>32	R	>16	R	>2	R	<=2	<=1	S	>1	R
>32	R	>16	R	>2	R	4	<=1	S	>1	R
>32	R	>16	R	>2	R	>4	>4	R	>1	R
>32	ESBL	>16	R*	>2	R	<=2	<=1	S	<=0.5	S
>32	R	>16	R	>2	R	>4	>4	R	>1	R
>32	ESBL	>16	R*	2	I	>4	<=1	S	>1	R
>32	R	>16	R	>2	R	<=2	>4	R	>1	R
>32	R	>16	R	>2	R	<=2	4	R	>1	R
>32	ESBL	>16	R*	>2	R	<=2	2	I	>1	R
>32	ESBL	>16	R*	2	I	<=2	<=1	S	1	I
<=1	S	<=4	S	<=0.5	S	<=2	<=1	S	<=0.5	S
>32	ESBL	>16	R*	2	I	<=2	<=1	S	1	I
>32	ESBL	>16	R*	>2	R	<=2	<=1	S	1	I
16	R	>16	R	<=0.5	S	<=2	<=1	S	>1	R
>32	R	>16	R	>2	R	>4	>4	R	>1	R
>32	R	>16	R	>2	R	<=2	4	R	>1	R
>32	R	>16	R	>2	R	4	>4	R	>1	R
>32	R	>16	R	>2	R	<=2	4	R	>1	R
>32	ESBL	>16	R*	>2	R	<=2	<=1	S	1	I
>32	R	>16	R	2	I	<=2	<=1	S	1	I
N/R		>16	R	>2	R	>4	>4	R	>1	R
>32	R	>16	R	>2	R	<=2	>4	R	>1	R
>32	R	>16	R	>2	R	<=2	>4	R	>1	R
>32	R	>16	R	2	I	<=2	>4	R	>1	R
>32	R	>16	R	>2	R	<=2	>4	R	>1	R
>32	ESBL	>16	R*	>2	R	<=2	<=1	S	1	I
>32	ESBL	>16	R*	>2	R	<=2	<=1	S	>1	R
>32	ESBL	>16	R*	>2	R	<=2	<=1	S	>1	R
>32	R	>16	R	>2	R	<=2	4	R	>1	R
>32	ESBL	>16	R*	>2	R	<=2	<=1	S	>1	R
>32	R	>16	R	>2	R	>4	>4	R	>1	R
>32	R	>16	R	>2	R	>4	>4	R	>1	R
>32	R	>16	R	>2	R	<=2	>4	R	>1	R
>32	ESBL	>16	R*	>2	R	<=2	<=1	S	<=0.5	S
>32	R	>16	R	>2	R	>4	>4	R	>1	R
>32	R	>16	R	>2	R	<=2	>4	R	>1	R
>32	R	>16	R	2	I	<=2	>4	R	>1	R
>32	ESBL	>16	R*	>2	R	<=2	<=1	S	>1	R
>32	R	>16	R	>2	R	<=2	<=1	S	>1	R
>32	ESBL	>16	R*	>2	R	<=2	<=1	S	>1	R
>32	R	>16	R	>2	R	<=2	>4	R	>1	R
>32	R	>16	R	>2	R	<=2	4	R	>1	R
>32	R	>16	R	>2	R	<=2	>4	R	>1	R
>32	R	>16	R	>2	R	>4	>4	R	>1	R
>32	R	>16	R	<=0.5	S	<=2	4	R	>1	R
>32	R	>16	R	>2	R	<=2	>4	R	>1	R
>32	R	>16	R	>2	R	<=2	>4	R	>1	R
>32	ESBL	>16	R*	>2	R	<=2	<=1	S	>1	R
>32	R	>16	R	>2	R	<=2	>4	R	>1	R
>32	R	>16	R	>2	R	<=2	>4	R	>1	R
>32	ESBL	>16	R*	2	I	<=2	<=1	S	>1	R
>32	R	>16	R	>2	R	<=2	>4	R	>1	R
>32	R	>16	R	>2	R	>4	4	R	>1	R

>32	R	>16	R	>2	R	<=2	>4	R	>1	R
>32	R	>16	R	>2	R	<=2	>4	R	>1	R
>32	ESBL	>16	R*	>2	R	<=2	2	I	>1	R
>32	R	>16	R	>2	R	<=2	>4	R	>1	R
>32	R	>16	R	>2	R	<=2	4	R	>1	R
<=1	S	8	S	<=0.5	S	>4	<=1	S	<=0.5	S
>32	ESBL	>16	R*	>2	R	<=2	<=1	S	1	I
>32	R	>16	R	>2	R	<=2	<=1	S	>1	R
<=1	S	8	S	<=0.5	S	>4	<=1	S	1	I
>32	R	>16	R	>2	R	<=2	2	I	>1	R
>32	ESBL	>16	R*	2	I	<=2	<=1	S	>1	R
>32	R	>16	R	>2	R	<=2	>4	R	>1	R
>32	ESBL	>16	R*	<=0.5	S	<=2	<=1	S	<=0.5	S
>32	R	>16	R	>2	R	<=2	4	R	>1	R
>32	ESBL	>16	R*	2	I	<=2	<=1	S	<=0.5	S
>32	ESBL	>16	R*	>2	R	<=2	<=1	S	>1	R
>32	R	>16	R	>2	R	<=2	4	R	>1	R
>32	R	>16	R	>2	R	<=2	4	R	>1	R
>32	ESBL	>16	R*	>2	R	4	2	I	>1	R
>32	ESBL	>16	R*	>2	R	<=2	<=1	S	>1	R
N/R		16	I	<=0.5	S	>4	<=1	S	>1	R
4	R	>16	R	>2	R	>4	<=1	S	1	I
>32	R	>16	R	>2	R	<=2	<=1	S	>1	R
>32	R	>16	R	>2	R	<=2	4	R	>1	R
<=1	S	<=4	S	<=0.5	S	<=2	<=1	S	<=0.5	S
<=1	S	16	I	<=0.5	S	>4	<=1	S	<=0.5	S
>32	ESBL	>16	R*	<=0.5	S	<=2	<=1	S	<=0.5	S
>32	ESBL	>16	R*	>2	R	>4	<=1	S	>1	R
>32	ESBL	>16	R*	2	I	<=2	4	R	>1	R
>32	ESBL	>16	R*	>2	R	4	>4	R	>1	R
>32	ESBL	>16	R*	>2	R	<=2	<=1	S	1	I
>32	ESBL	>16	R*	2	I	<=2	<=1	S	1	I
>32	ESBL	>16	R*	>2	R	<=2	<=1	S	1	I
2	I	>16	R	>2	R	>4	2	I	>1	R
>32	R	>16	R	>2	R	<=2	<=1	S	>1	R
>32	R	>16	R	<=0.5	S	<=2	>4	R	>1	R
>32	ESBL	>16	R*	2	I	<=2	<=1	S	1	I
>32	ESBL	>16	R*	>2	R	<=2	<=1	S	>1	R
>32	ESBL	>16	R*	>2	R	<=2	<=1	S	1	I
>32	ESBL	>16	R*	1	S	<=2	<=1	S	1	I
>32	ESBL	>16	R*	>2	R	<=2	2	I	>1	R
>32	R	>16	R	>2	R	<=2	4	R	>1	R
N/R		>16	R	2	I	<=2	>4	R	>1	R
<=1	S	8	S	1	S	<=2	<=1	S	1	I
>32	R	>16	R	>2	R	<=2	>4	R	>1	R
>32	ESBL	>16	R*	<=0.5	S	<=2	<=1	S	<=0.5	S
>32	ESBL	>16	R*	>2	R	<=2	<=1	S	>1	R
>32	ESBL	>16	R*	1	S	<=2	4	R	>1	R
<=1	S	16	I	>2	R	<=2	<=1	S	>1	R
>32	R	>16	R	2	I	<=2	2	I	1	I
>32	ESBL	>16	R*	2	I	<=2	<=1	S	<=0.5	S
>32	R	>16	R	<=0.5	S	<=2	>4	R	>1	R
>32	ESBL	>16	R*	>2	R	<=2	<=1	S	>1	R
>32	ESBL	>16	R*	>2	R	<=2	<=1	S	>1	R
4	R	>16	R	>2	R	<=2	2	I	>1	R
>32	R	>16	R	<=0.5	S	4	>4	R	>1	R

>32	ESBL	>16	R*	>2	R	<=2		<=1	S	<=0.5	S
>32	ESBL	>16	R*	>2	R	<=2		<=1	S	<=0.5	S
N/R		>16	R	<=0.5	S	>4		>4	R	>1	R
>32	ESBL	>16	R*	>2	R	<=2		<=1	S	>1	R
>32	ESBL	>16	R*	>2	R	<=2		<=1	S	<=0.5	S
>32	R	>16	R	>2	R	<=2		4	R	>1	R
>32	R	>16	R	>2	R	4		4	R	>1	R
>32	ESBL	>16	R*	>2	R	<=2		<=1	S	>1	R
8	R	>16	R	>2	R	>4		<=1	S	<=0.5	S
>32	ESBL	>16	R*	>2	R	>4		4	R	>1	R
>32	ESBL	>16	R*	2	I	>4		<=1	S	>1	R
>32	ESBL	>16	R*	>2	R	<=2		<=1	S	>1	R
>32	ESBL	>16	R*	>2	R	4		2	I	>1	R
>32	R	>16	R	>2	R	<=2		4	R	>1	R
>32	ESBL	>16	R*	>2	R	<=2		<=1	S	<=0.5	S
>32	R	>16	R	>2	R	>4		>4	R	>1	R
>32	R	>16	R	>2	R	<=2		>4	R	>1	R
>32	R	>16	R	>2	R	>4		2	I	>1	R
<=1	S	16	I	2	I	<=2		<=1	S	1	I
>32	R	>16	R	>2	R	<=2		4	R	>1	R
>32	R	>16	R	>2	R	<=2		>4	R	>1	R
>32	R	>16	R	>2	R	<=2		>4	R	>1	R
>32	R	>16	R	>2	R	<=2		>4	R	>1	R
>32	ESBL	>16	R*	>2	R	4		<=1	S	1	I
>32	R	>16	R	>2	R	<=2		4	R	>1	R
>32	ESBL	>16	R*	>2	R	4		<=1	S	<=0.5	S
>32	ESBL	>16	R*	>2	R	<=2		4	R	>1	R
>32	R	>16	R	>2	R	<=2		4	R	>1	R
>32	ESBL	>16	R*	>2	R	<=2		<=1	S	>1	R
>32	R	>16	R	>2	R	<=2		>4	R	>1	R
>32	ESBL	>16	R*	>2	R	<=2		<=1	S	1	I
>32	R	>16	R	>2	R	<=2		>4	R	>1	R
>32	ESBL	>16	R*	2	I	<=2		<=1	S	1	I
>32	ESBL	>16	R*	1	S	<=2		<=1	S	>1	R
>32	R	>16	R	<=0.5	S	<=2		>4	R	>1	R
>32	ESBL	>16	R*	1	S	<=2		4	R	>1	R
>32	ESBL	>16	R*	2	I	<=2		<=1	S	>1	R
>32	R	>16	R	2	I	<=2		2	I	1	I
>32	R	>16	R	>2	R	<=2		4	R	>1	R
>32	ESBL	>16	R*	2	I	<=2		<=1	S	>1	R
>32	ESBL	>16	R*	>2	R	4		2	I	>1	R
>32	ESBL	>16	R*	1	S	0.5	I	<=1	S	1	I
>32	ESBL	>16	R*	>2	R	<=2		4	R	>1	R
>32	ESBL	>16	R*	>2	R	<=2		<=1	S	>1	R
>32	R	>16	R	>2	R	>4		>4	R	>1	R
>32	ESBL	>16	R*	<=0.5	S	<=2		4	R	>1	R
>32	R	>16	R	2	I	<=2		>4	R	>1	R
>32	R	>16	R	>2	R	<=2		>4	R	>1	R
>32	R	>16	R	2	I	<=2		>4	R	>1	R
>32	ESBL	>16	R*	2	I	<=2		<=1	S	1	I
>32	ESBL	>16	R*	>2	R	<=2		<=1	S	>1	R
>32	ESBL	>16	R*	>2	R	<=2		<=1	S	>1	R
>32	ESBL	>16	R*	>2	R	<=2		<=1	S	>1	R
>32	ESBL	>16	R*	>2	R	<=2		<=1	S	<=0.5	S
>32	ESBL	>16	R*	2	I	<=2		<=1	S	1	I
>32	R	>16	R	2	I	<=2		>4	R	>1	R

>32	ESBL	>16	R*	>2	R	<=2	4	R	>1	R
>32	R	>16	R	2	I	<=2	>4	R	>1	R
>32	ESBL	>16	R*	>2	R	<=2	<=1	S	>1	R
>32	ESBL	>16	R*	>2	R	<=2	2	I	>1	R
>32	ESBL	16	R*	<=0.5	S	<=2	<=1	S	1	I
<=1	S	16	I	>2	R	<=2	<=1	S	>1	R
>32	R	>16	R	>2	R	<=2	>4	R	>1	R
>32	R	>16	R	>2	R	<=2	>4	R	>1	R
>32	ESBL	>16	R*	1	S	<=2	<=1	S	1	I
>32	ESBL	>16	R*	>2	R	<=2	<=1	S	<=0.5	S
>32	ESBL	>16	R*	>2	R	<=2	<=1	S	>1	R
>32	R	>16	R	>2	R	<=2	>4	R	>1	R
>32	R	>16	R	>2	R	<=2	>4	R	>1	R
>32	R	>16	R	>2	R	<=2	>4	R	>1	R
N/R		>16	R	<=0.5	S	>4	>4	R	>1	R
>32	R	>16	R	2	I	<=2	<=1	S	<=0.5	S
N/R		>16	R	>2	R	>4	>4	R	>1	R
>32	R	>16	R	>2	R	<=2	<=1	S	>1	R
>32	ESBL	>16	R*	>2	R	<=2	<=1	S	1	I
2	I	>16	R	<=0.5	S	<=2	<=1	S	1	I
>32	ESBL	>16	R*	>2	R	<=2	<=1	S	>1	R
>32	ESBL	>16	R*	2	I	<=2	<=1	S	1	I
>32	R	>16	R	>2	R	<=2	>4	R	>1	R
>32	ESBL	>16	R*	2	I	<=2	<=1	S	>1	R
>32	ESBL	>16	R*	>2	R	<=2	2	I	1	I
>32	ESBL	>16	R*	>2	R	<=2	<=1	S	>1	R
>32	ESBL	>16	R*	>2	R	<=2	4	R	>1	R
>32	R	>16	R	>2	R	<=2	4	R	>1	R
>32	R	>16	R	>2	R	<=2	<=1	S	>1	R
>32	ESBL	>16	R*	2	I	<=2	<=1	S	1	I
<=1	S	<=4	S	>2	R	<=2	<=1	S	<=0.5	S
>32	ESBL	>16	R*	2	I	<=2	<=1	S	1	I
>32	ESBL	>16	R*	2	I	<=2	<=1	S	1	I
>32	ESBL	>16	R*	>2	R	<=2	>4	R	>1	R
>32	R	>16	R	>2	R	<=2	>4	R	>1	R
2	I	>16	R	2	I	<=2	2	I	>1	R
<=1	S	<=4	S	<=0.5	S	<=2	<=1	S	<=0.5	S
>32	R	>16	R	2	I	<=2	4	R	>1	R
>32	R	>16	R	>2	R	<=2	<=1	S	>1	R
>32	ESBL	>16	R*	2	I	<=2	<=1	S	>1	R
>32	ESBL	>16	R*	2	I	<=2	<=1	S	<=0.5	S
>32	ESBL	>16	R*	>2	R	<=2	<=1	S	>1	R
>32	ESBL	>16	R*	2	I	<=2	<=1	S	>1	R
>32	R	>16	R	>2	R	<=2	<=1	S	>1	R
>32	R	>16	R	2	I	<=2	>4	R	>1	R
>32	R	>16	R	1	S	>4	>4	R	>1	R
>32	R	>16	R	>2	R	<=2	>4	R	>1	R
>32	R	>16	R	2	I	>4	4	R	>1	R
>32	ESBL	>16	R*	2	I	>4	<=1	S	<=0.5	S
N/R		>16	R	2	I	>4	<=1	S	<=0.5	S
16	R	>16	R	1	S	>4	<=1	S	<=0.5	S
>32	R	>16	R	>2	R	4	>4	R	>1	R
N/R		>16	R*	<=0.5	S	>4	<=1	S	1	I
<=1	S	<=4	S	>2	R	<=2	<=1	S	<=0.5	S
<=1	S	<=4	S	<=0.5	S	<=2	<=1	S	<=0.5	S

>32	ESBL	>16	R*	>2	R	<=2	<=1	S	<=0.5	S
2	I	>16	R	>2	R	>4	>4	R	>1	R
<=1	S	<=4	S	<=0.5	S	<=2	<=1	S	<=0.5	S
8	R	>16	R	1	S	<=2	>4	R	>1	R
>32	R	>16	R	>2	R	>4	>4	R	>1	R
>32	R	>16	R	>2	R	<=2	>4	R	>1	R
>32	ESBL	>16	R*	1	S	<=2	<=1	S	1	I
>32	ESBL	>16	R*	2	I	<=2	<=1	S	>1	R
>32	R	>16	R	>2	R	<=2	>4	R	>1	R
>32	R	>16	R	>2	R	<=2	>4	R	>1	R
>32	R	>16	R	>2	R	<=2	>4	R	>1	R
>32	R	>16	R	>2	R	<=2	>4	R	>1	R
>32	R	>16	R	>2	R	<=2	>4	R	>1	R
>32	R	>16	R	>2	R	<=2	>4	R	>1	R
>32	ESBL	>16	R*	<=0.5	S	<=2	<=1	S	>1	R
>32	ESBL	>16	R*	>2	R	>4	4	R	>1	R
N/R		8	S	<=0.5	S	<=2	<=1	S	<=0.5	S
>32	R	>16	R	>2	R	<=2	>4	R	>1	R
>32	R	>16	R	>2	R	>4	4	R	>1	R
>32	R	>16	R	>2	R	<=2	4	R	>1	R
>32	ESBL	>16	R*	>2	R	<=2	>4	R	>1	R
>32	ESBL	>16	R*	>2	R	<=2	<=1	S	>1	R
>32	R	>16	R	>2	R	>4	>4	R	>1	R
>32	R	>16	R	>2	R	>4	>4	R	>1	R
>32	R	>16	R	>2	R	>4	>4	R	>1	R
>32	R	>16	R	>2	R	>4	>4	R	>1	R
>32	ESBL	>16	R*	>2	R	<=2	4	R	>1	R
>32	R	>16	R	>2	R	>4	>4	R	>1	R
>32	R	>16	R	>2	R	<=2	>4	R	>1	R
>32	R	>16	R	>2	R	<=2	4	R	>1	R
>32	ESBL	>16	R*	2	I	<=2	<=1	S	>1	R
>32	R	>16	R	>2	R	<=2	<=1	S	>1	R
>32	R	>16	R	>2	R	<=2	4	R	>1	R
>32	ESBL	>16	R*	>2	R	<=2	<=1	S	>1	R
2	I	>16	R	>2	R	<=2	4	R	>1	R
>32	R	>16	R	>2	R	<=2	<=1	S	>1	R
>32	ESBL	>16	R*	>2	R	<=2	<=1	S	<=0.5	S
>32	R	>16	R	>2	R	>4	<=1	S	>1	R
>32	ESBL	>16	R*	>2	R	<=2	<=1	S	<=0.5	S
>32	ESBL	>16	R*	>2	R	<=2	<=1	S	>1	R
>32	ESBL	>16	R*	>2	R	<=2	4	R	>1	R
>32	R	>16	R	>2	R	>4	2	I	>1	R
>32	ESBL	>16	R*	>2	R	>4	4	R	>1	R
>32	ESBL	>16	R*	>2	R	4	2	I	>1	R
>32	ESBL	>16	R*	>2	R	>4	<=1	S	>1	R
>32	ESBL	>16	R*	>2	R	<=2	<=1	S	<=0.5	S
>32	ESBL	>16	R*	>2	R	<=2	>4	R	>1	R
<=1	S	<=4	S	<=0.5	S	<=2	<=1	S	<=0.5	S
>32	ESBL	>16	R*	>2	R	<=2	<=1	S	1	I
4	ESBL	>16	R*	>2	R	<=2	<=1	S	>1	R
>32	ESBL	>16	R*	>2	R	<=2	<=1	S	>1	R
>32	ESBL	>16	R*	>2	R	<=2	<=1	S	>1	R
>32	R	>16	R	>2	R	<=2	>4	R	>1	R
>32	ESBL	>16	R*	>2	R	<=2	<=1	S	>1	R
32	R	>16	R	1	S	<=2	<=1	S	<=0.5	S
>32	ESBL	>16	R*	>2	R	<=2	<=1	S	>1	R

>32	R	>16	R	2	I	<=2	2	I	>1	R
>32	R	>16	R	>2	R	<=2	4	R	>1	R
>32	R	>16	R	>2	R	>4	4	R	>1	R
>32	R	>16	R	>2	R	<=2	4	R	>1	R
>32	R	>16	R	>2	R	<=2	>4	R	>1	R
2	I	>16	R	<=0.5	S	<=2	<=1	S	<=0.5	S
2	I	16	I	2	I	<=2	<=1	S	>1	R
>32	R	>16	R	2	I	<=2	>4	R	>1	R
>32	ESBL	>16	R*	>2	R	<=2	4	R	>1	R
>32	R	>16	R	>2	R	<=2	>4	R	>1	R
>32	R	>16	R	>2	R	<=2	2	I	>1	R
>32	R	>16	R	2	I	<=2	>4	R	>1	R
>32	R	>16	R	>2	R	<=2	>4	R	>1	R
>32	ESBL	>16	R*	>2	R	<=2	4	R	>1	R
2	I	16	I	<=0.5	S	<=2	2	I	>1	R
<=1	S	16	I	<=0.5	S	>4	<=1	S	<=0.5	S
>32	R	>16	R	>2	R	<=2	>4	R	>1	R
<=1	S	<=4	S	>2	R	<=2	<=1	S	<=0.5	S
>32	R	>16	R	>2	R	<=2	>4	R	>1	R
>32	ESBL	>16	R*	>2	R	<=2	2	I	>1	R
>32	ESBL	>16	R*	2	I	<=2	<=1	S	>1	R
>32	ESBL	>16	R*	>2	R	<=2	2	I	>1	R
>32	R	>16	R	>2	R	<=2	4	R	>1	R
>32	ESBL	>16	R*	<=0.5	S	<=2	<=1	S	1	I
>32	ESBL	>16	R*	>2	R	4	2	I	>1	R
>32	R	>16	R	2	I	<=2	>4	R	>1	R
>32	R	>16	R	1	S	<=2	>4	R	>1	R
>32	ESBL	>16	R*	>2	R	<=2	4	R	>1	R
<=1	S	<=4	S	<=0.5	S	<=2	<=1	S	<=0.5	S
>32	ESBL	>16	R*	>2	R	>4	<=1	S	<=0.5	S
>32	R	>16	R	>2	R	<=2	>4	R	>1	R
<=1	S	<=4	S	<=0.5	S	<=2	<=1	S	<=0.5	S
>32	R	>16	R	2	I	<=2	>4	R	>1	R
>32	ESBL	>16	R*	2	I	<=2	<=1	S	>1	R
>32	ESBL	>16	R*	2	I	<=2	<=1	S	<=0.5	S
>32	R	>16	R	>2	R	>4	4	R	>1	R
<=1	S	<=4	S	<=0.5	S	<=2	<=1	S	<=0.5	S
>32	ESBL	>16	R*	>2	R	<=2	<=1	S	>1	R
>32	ESBL	>16	R*	>2	R	<=2	<=1	S	>1	R
>32	R	>16	R	>2	R	<=2	4	R	>1	R
>32	ESBL	>16	R*	>2	R	<=2	2	I	>1	R
>32	R	>16	R	>2	R	<=2	4	R	>1	R
>32	R	>16	R	>2	R	>4	4	R	>1	R
>32	ESBL	>16	R*	>2	R	<=2	<=1	S	<=0.5	S
>32	ESBL	>16	R*	2	I	<=2	<=1	S	1	I
>32	R	>16	R	>2	R	<=2	>4	R	>1	R
>32	R	>16	R	<=0.5	S	<=2	<=1	S	<=0.5	S
>32	ESBL	>16	R*	>2	R	<=2	<=1	S	>1	R
<=1	S	8	S	<=0.5	S	<=2	<=1	S	<=0.5	S
>32	R	>16	R	>2	R	<=2	>4	R	>1	R
>32	R	>16	R	2	I	<=2	>4	R	>1	R
>32	ESBL	>16	R*	>2	R	<=2	<=1	S	>1	R
>32	R	>16	R	>2	R	<=2	4	R	>1	R
>32	ESBL	>16	R*	>2	R	<=2	2	I	>1	R
>32	ESBL	>16	R*	>2	R	<=2	<=1	S	<=0.5	S
>32	ESBL	>16	R*	2	I	<=2	<=1	S	<=0.5	S

>32	R	>16	R	>2	R	<=2		4	R	>1	R
>32	R	>16	R	>2	R	<=2		2	I	>1	R
>32	R	>16	R	>2	R	<=2		>4	R	>1	R
>32	R	>16	R	>2	R	<=2		>4	R	>1	R
>32	R	>16	R	>2	R	<=2		>4	R	>1	R
>32	R	>16	R	>2	R	<=2		>4	R	>1	R
>32	R	>16	R	>2	R	<=2		>4	R	>1	R
>32	R	>16	R	2	I	<=2		>4	R	>1	R
>32	ESBL	>16	R*	>2	R	1	I	<=1	S	1	I
>32	R	>16	R	>2	R	0.5	I	>4	R	>1	R
>32	ESBL	>16	R*	2	I	<=2		<=1	S	>1	R
>32	ESBL	>16	R*	2	I	<=2		<=1	S	>1	R
>32	ESBL	>16	R*	>2	R	<=2		<=1	S	<=0.5	S
>32	R	>16	R	>2	R	<=2		>4	R	>1	R
>32	R	>16	R	>2	R	<=2		4	R	>1	R
>32	R	>16	R	2	I	<=2		<=1	S	>1	R
>32	R	>16	R	>2	R	<=2		>4	R	>1	R
>32	R	>16	R	>2	R	<=2		>4	R	>1	R
>32	R	>16	R	>2	R	<=2		>4	R	>1	R
>32	R	>16	R	>2	R	<=2		>4	R	>1	R
<=1	S	<=4	S	2	I	<=2		<=1	S	1	I
>32	ESBL	>16	R*	>2	R	>4		<=1	S	>1	R
>32	ESBL	>16	R*	>2	R	0.5	I	<=1	S	1	I

>32	R	>16	R	>2	R			>4	R	>1	R
>32	R	>16	R	>2	R	0.5	I	4	R	>1	R

>32	ESBL	>16	R*	2	I	<=2	<=1	S	1	I
>32	R	>16	R	>2	R	<=2	>4	R	>1	R
>32	ESBL	>16	R*	>2	R	<=2	4	R	>1	R
2	I	>16	R	>2	R	<=2	4	R	>1	R

>32	ESBL	>16	R*	>2	R	>4		<=1	S	1	I
>32	ESBL	>16	R*	>2	R	<=2		<=1	S	>1	R
>32	R	>16	R	>2	R	>4		>4	R	>1	R
>32	R	>16	R	>2	R	>4		>4	R	>1	R
>32	R	>16	R	>2	R	<=2		>4	R	>1	R
4	R	>16	R	>2	R	<=2		>4	R	>1	R
>32	R	>16	R	>2	R	<=2		>4	R	>1	R
>32	ESBL	>16	R*	<=0.5	S	<=2		<=1	S	<=0.5	S
>32	R	>16	R	>2	R	<=2		>4	R	>1	R
>32	ESBL	>16	R*	>2	R	<=2		>4	R	>1	R
>32	ESBL	>16	R*	>2	R	<=2		>4	R	>1	R
>32	R	>16	R	>2	R	<=2		4	R	>1	R
>32	R	>16	R	>2	R	<=2		>4	R	>1	R
>32	R	>16	R	>2	R	<=2		>4	R	>1	R
>32	ESBL	>16	R*	>2	R	<=2		4	R	>1	R
>32	R	>16	R	>2	R	<=2		4	R	>1	R
>32	ESBL	>16	R*	>2	R	>4		2	I	>1	R
>32	ESBL	>16	R*	>2	R	>4		>4	R	>1	R
>32	R	>16	R	<=0.5	S	<=2		>4	R	>1	R
>32	ESBL	>16	R*	<=0.5	S	<=2		<=1	S	>1	R
4	ESBL	8	R*	<=0.5	S	<=2		<=1	S	<=0.5	S
>32	R	>16	R	2	I	<=2		>4	R	>1	R
>32	ESBL	>16	R*	>2	R	<=2		<=1	S	>1	R
>32	ESBL	>16	R*	>2	R	<=2		<=1	S	>1	R
>32	R	>16	R	>2	R	<=2		2	I	1	I
>32	R	>16	R	>2	R	<=2		4	R	>1	R
>32	ESBL	>16	R*	1	S	<=2		<=1	S	1	I
>32	R	>16	R	>2	R	<=2		>4	R	>1	R
>32	ESBL	>16	R*	>2	R	<=2		<=1	S	>1	R
>32	ESBL	>16	R*	>2	R	<=2		<=1	S	>1	R
N/R		>16	R*	2	I	>4		>4	R	>1	R
>32	R	>16	R	>2	R	<=2		4	R	>1	R
>32	R	>16	R	<=0.5	S	<=2		>4	R	>1	R
>32	ESBL	>16	R*	>2	R	<=2		<=1	S	>1	R
>32	ESBL	>16	R*	<=0.5	S	<=2		<=1	S	1	I
>32	R	>16	R	>2	R	<=2		>4	R	>1	R
>32	ESBL	>16	R*	>2	R	<=2		<=1	S	<=0.5	S
>32	ESBL	>16	R*	1	S	<=2		<=1	S	>1	R
<=1	S	8	S	<=0.5	S	<=2		<=1	S	<=0.5	S
>32	R	>16	R	2	I	>4		>4	R	>1	R

>32	R	>16	R	>2	R	<=2		>4	R	>1	R
>32	R	>16	R	1	S	<=2		>4	R	>1	R
>32	ESBL	>16	R*	1	S	<=2		<=1	S	1	I
>32	R	>16	R	>2	R	<=2		>4	R	>1	R
>32	ESBL	>16	R*	2	I	4		<=1	S	1	I
>32	ESBL	>16	R*	>2	R	4		<=1	S	1	I
>32	ESBL	>16	R*	1	S	<=2		<=1	S	1	I
>32	R	>16	R	<=0.5	S	<=2		4	R	>1	R
>32	R	>16	R	>2	R	<=2		>4	R	>1	R
>32	R	>16	R	>2	R	<=2		>4	R	>1	R
<=1	S	<=4	S	<=0.5	S	1	I	<=1	S	<=0.5	S
<=1	S	<=4	S	>2	R	0.5	I	<=1	S	<=0.5	S
2	I	8	S	<=0.5	S	1	I	<=1	S	<=0.5	S
N/R		>16	R	<=0.5	S	>128	R	<=1	S	<=0.5	S
>32	R	>16	R	>2	R	1	I	>4	R	>1	R
<=1	S	<=4	S	<=0.5	S	1	I	<=1	S	<=0.5	S
<=1	S	<=4	S	2	I	1	I	<=1	S	<=0.5	S
<=1	S	>16	R	<=0.5	S	1	I	<=1	S	<=0.5	S
<=1	S	<=4	S	<=0.5	S	0.5	I	<=1	S	<=0.5	S
<=1	S	<=4	S	<=0.5	S	1	I	<=1	S	<=0.5	S
>32	ESBL	>16	R*	2	I	8	R	<=1	S	>1	R
>32	R	>16	R	>2	R	0.5	I	2	I	>1	R
>32	R	>16	R	>2	R	1	S	<=1	S	>1	R
>32	ESBL	>16	R*	>2	R	0.5	I	<=1	S	1	I
>32	ESBL	>16	R*	1	S	0.5	I	<=1	S	>1	R
>32	ESBL	>16	R*	>2	R	0.5	I	4	R	>1	R
>32	R	>16	R	>2	R	0.5	I	4	R	>1	R
>32	ESBL	>16	R*	2	I	0.5	I	<=1	S	>1	R
>32	ESBL	>16	R*	>2	R	0.5	I	<=1	S	1	I
>32	R	>16	R	>2	R	0.5	I	>4	R	>1	R
>32	ESBL	>16	R*	1	S	0.5	I	<=1	S	1	I
>32	R	>16	R	>2	R	0.5	I	4	R	>1	R
>32	R	>16	R	>2	R	0.5	I	4	R	>1	R
>32	ESBL	>16	R*	2	I	0.5	I	<=1	S	1	I
>32	ESBL	>16	R*	>2	R	0.5	I	<=1	S	1	I
<=1	S	16	I	1	S	0.5	I	<=1	S	>1	R
32	ESBL	>16	R*	2	I	1	I	<=1	S	<=0.5	S
>32	ESBL	>16	R*	2	I	1	I	<=1	S	1	I
N/R		>16	R*	2	I	>128	R	2	I	>1	R
N/R		>16	R	<=0.5	S	>128	R	<=1	S	>1	R
>32	ESBL	>16	R*	1	S	>128	R	<=1	S	1	I
>32	R	>16	R	>2	R	4	R	<=1	S	>1	R
32	R	>16	R	>2	R	0.5	I	<=1	S	>1	R
>32	R	>16	R	>2	R	0.5	I	>4	R	>1	R
>32	R	>16	R	>2	R	2	I	<=1	S	>1	R
>32	R	>16	R	>2	R	64	R	>4	R	>1	R
>32	R	>16	R	>2	R	32	R	<=1	S	>1	R
>32	R	>16	R	>2	R	1	I	>4	R	>1	R
>32	R	>16	R	>2	R	1	I	>4	R	>1	R
>32	R	>16	R	>2	R	1	I	>4	R	>1	R
8	R	>16	R	>2	R	32	R	>4	R	>1	R
>32	R	>16	R	2	I	16	R	<=1	S	1	I
>32	R	>16	R	>2	R	64	R	>4	R	>1	R
>32	ESBL	>16	R*	>2	R	16	R	4	R	1	I
>32	ESBL	>16	R*	>2	R	1	I	4	R	>1	R
>32	ESBL	>16	R*	>2	R	>128	R	<=1	S	1	I

>32	R	>16	R	>2	R	2	I	<=1	S	>1	R
>32	R	>16	R	<=0.5	S	>128	R	>4	R	>1	R
>32	ESBL	>16	R*	>2	R	1	I	<=1	S	<=0.5	S
>32	ESBL	>16	R*	2	I	0.5	I	<=1	S	1	I
>32	ESBL	>16	R*	>2	R	1	I	<=1	S	1	I
>32	R	>16	R	>2	R	1	I	>4	R	>1	R
>32	R	>16	R	>2	R	64	R	>4	R	>1	R
16	R	>16	R	>2	R	>128	R	>4	R	>1	R
>32	ESBL	>16	R*	>2	R	1	I	<=1	S	>1	R
>32	ESBL	>16	R*	>2	R	>128	R	2	I	>1	R
>32	R	>16	R	>2	R	>128	R	>4	R	>1	R
>32	R	>16	R	>2	R			2	I	>1	R
>32	R	>16	R	>2	R	2	I	<=1	S	>1	R
>32	ESBL	>16	R*	>2	R	2	I	<=1	S	>1	R
>32	ESBL	>16	R*	2	I	16	R	<=1	S	<=0.5	S
>32	ESBL	>16	R*	2	I	8	R	<=1	S	1	I
>32	R	>16	R	2	I	>128	R	2	I	>1	R
>32	ESBL	>16	R*	>2	R	1	I	<=1	S	<=0.5	S
>32	ESBL	>16	R*	1	S	1	I	<=1	S	>1	R
>32	ESBL	>16	R*	>2	R	2	I	<=1	S	>1	R
>32	R	>16	R	2	I	1	I	2	I	>1	R
>32	ESBL	>16	R*	2	I	1	I	<=1	S	1	I
>32	ESBL	>16	R*	>2	R	2	I	<=1	S	>1	R
>32	R	>16	R	>2	R	1	I	>4	R	>1	R
>32	R	>16	R	>2	R	1	I	>4	R	>1	R
>32	R	>16	R	>2	R	1	I	2	I	>1	R
4	R	>16	R	>2	R	2	I	>4	R	>1	R
>32	R	>16	R	>2	R	2	I	2	I	>1	R
>32	ESBL	>16	R*	>2	R	1	I	<=1	S	>1	R
>32	ESBL	>16	R*	2	I	1	I	<=1	S	<=0.5	S
>32	R	>16	R	>2	R	1	I	>4	R	>1	R
>32	R	>16	R	>2	R	4	R	>4	R	>1	R
>32	R	>16	R	>2	R	4	R	4	R	>1	R
>32	R	>16	R	>2	R	4	R	4	R	>1	R
N/R		>16	R	1	S	>128	R	4	R	>1	R
>32	R	>16	R	>2	R	4	R	>4	R	>1	R
>32	R	>16	R	>2	R	2	R	>4	R	>1	R
>32	R	>16	R	>2	R	4	R	>4	R	>1	R
>32	ESBL	>16	R*	>2	R	2	R	>4	R	>1	R
>32	ESBL	>16	R*	>2	R	2	R	>4	R	>1	R
>32	R	>16	R	>2	R	2	R	>4	R	>1	R
>32	R	>16	R	>2	R	2	R	4	R	>1	R
>32	R	>16	R	>2	R	2	I	2	I	>1	R
N/R		>16	R	>2	R	128	R	>4	R	>1	R
>32	R	>16	R	>2	R	128	R	>4	R	>1	R
>32	R	>16	R	>2	R	2	I	4	R	>1	R
>32	R	>16	R	>2	R	1	I	>4	R	>1	R
N/R		>16	R	<=0.5	S	>128	R	>4	R	>1	R
>32	R	>16	R	2	I	4	R	4	R	>1	R
N/R		>16	R	>2	R	>128	R	2	I	>1	R
>32	ESBL	>16	R*	>2	R	2	I	2	I	>1	R
>32	ESBL	>16	R*	>2	R	2	I	<=1	S	>1	R
>32	R	>16	R	>2	R	1	I	<=1	S	>1	R
>32	R	>16	R	>2	R	1	I	>4	R	>1	R
>32	ESBL	>16	R*	>2	R	1	I	<=1	S	>1	R

>32	ESBL	>16	R*	>2	R	1	I	<=1	S	1	I
>32	R	>16	R	>2	R	4	R	<=1	S	>1	R
>32	ESBL	>16	R*	>2	R	2	I	<=1	S	>1	R
>32	ESBL	>16	R*	>2	R	1	I	<=1	S	1	I
>32	ESBL	>16	R*	>2	R	1	I	<=1	S	1	I
>32	R	>16	R	>2	R	1	I	>4	R	>1	R
>32	R	>16	R	>2	R	1	I	4	R	>1	R
N/R		>16	R	>2	R	>128	R	>4	R	>1	R
>32	R	>16	R	>2	R	0.5	I	>4	R	>1	R
>32	ESBL	>16	R*	<=0.5	S	4	R	<=1	S	>1	R
N/R		>16	R	<=0.5	S	>128	R	<=1	S	1	I
>32	ESBL	>16	R*	>2	R	4	R	2	I	>1	R
>32	ESBL	>16	R*	>2	R	1	I	<=1	S	1	I
>32	R	>16	R	>2	R	>128	R	>4	R	>1	R
>32	R	>16	R	>2	R	1	I	>4	R	>1	R
>32	R	>16	R	>2	R	1	I	>4	R	>1	R
>32	R	>16	R	2	I	1	I	2	I	>1	R
>32	R	>16	R	>2	R	2	I	>4	R	>1	R
N/R		>16	R*	>2	R	>128	R	>4	R	>1	R
>32	R	>16	R	<=0.5	S	0.5	I	>4	R	>1	R
>32	R	>16	R	>2	R	0.5	I	4	R	>1	R
N/R		>16	R	<=0.5	S	128	R	>4	R	>1	R
>32	R	>16	R	>2	R	0.5	I	>4	R	>1	R
>32	R	>16	R	>2	R	1	I	4	R	>1	R
>32	ESBL	>16	R*	2	I	0.5	I	<=1	S	<=0.5	S
>32	ESBL	>16	R*	>2	R	1	I	<=1	S	1	I
>32	ESBL	>16	R*	2	I	1	I	<=1	S	>1	R
>32	R	>16	R	2	I	1	I	>4	R	>1	R
>32	ESBL	>16	R*	1	S	1	I	<=1	S	>1	R
>32	R	>16	R	<=0.5	S	1	I	4	R	>1	R
>32	R	>16	R	>2	R	1	I	4	R	>1	R
>32	R	>16	R	>2	R	0.5	I	>4	R	>1	R
>32	R	>16	R	<=0.5	S	0.5	I	4	R	>1	R
>32	ESBL	>16	R*	>2	R	2	I	2	I	>1	R
>32	ESBL	>16	R*	>2	R	2	I	<=1	S	1	I
>32	ESBL	>16	R*	1	S	1	I	<=1	S	>1	R
>32	R	>16	R	>2	R	2	I	>4	R	>1	R
>32	R	>16	R	>2	R	1	I	>4	R	>1	R
>32	ESBL	>16	R*	>2	R	1	I	4	R	>1	R
>32	R	>16	R	>2	R	4	R	<=1	S	>1	R
>32	R	>16	R	>2	R	64	R	>4	R	>1	R
>32	R	>16	R	>2	R	8	R	>4	R	>1	R
>32	R	>16	R	>2	R	4	R	4	R	>1	R
>32	R	>16	R	>2	R	1	I	4	R	>1	R
>32	ESBL	>16	R*	2	I	>128	R	<=1	S	1	I
>32	R	>16	R	>2	R	1	I	4	R	>1	R
>32	R	>16	R	2	I	1	I	>4	R	>1	R
>32	ESBL	>16	R*	2	I	1	I	<=1	S	1	I
>32	R	>16	R	>2	R	1	I	<=1	S	1	I
>32	ESBL	>16	R*	2	I	1	I	2	I	>1	R
>32	R	>16	R	>2	R	1	I	>4	R	>1	R
>32	R	>16	R	>2	R	1	I	>4	R	>1	R
>32	ESBL	>16	R*	>2	R	1	I	<=1	S	<=0.5	S
>32	ESBL	>16	R*	2	I	>128	R	<=1	S	1	I
>32	ESBL	>16	R*	>2	R	4	R	<=1	S	>1	R
>32	R	>16	R	>2	R	0.5	I	<=1	S	>1	R

>32	R	>16	R	>2	R	32	R	>4	R	>1	R
N/R		>16	R	>2	R	>128	R	>4	R	>1	R
>32	ESBL	>16	R*	>2	R	1	I	<=1	S	>1	R
>32	ESBL	>16	R*	>2	R	1	I	<=1	S	<=0.5	S

		>16	R	<=0.5	S	>128	R	<=1	S	<=0.5	S
>32	R	>16	R	>2	R	1	I	4	R	>1	R
>32	ESBL	>16	R*	>2	R	1	I	<=1	S	1	I
>32	ESBL	>16	R*	>2	R	1	I	2	I	>1	R
>32	R	>16	R	>2	R	1	I	4	R	>1	R
>32	R	>16	R	>2	R	1	I	>4	R	>1	R
>32	R	>16	R	>2	R	1	I	<=1	S	>1	R
>32	R	>16	R	2	I	1	I	2	I	>1	R
>32	R	>16	R	<=0.5	S	1	I	>4	R	>1	R
>32	ESBL	>16	R*	1	S	1	I	2	I	>1	R
>32	ESBL	>16	R*	>2	R			<=1	S	>1	R
4	R	>16	R	<=0.5	S	0.5	I	>4	R	>1	R
>32	ESBL	>16	R*	>2	R	>128	R	>4	R	1	I
N/R		>16	R	>2	R	>128	R	2	I	>1	R
>32	ESBL	>16	R*	>2	R	>128	R	>4	R	>1	R
>32	R	>16	R	>2	R			4	R	>1	R
32	R	>16	R	<=0.5	S	>128	R	>4	R	>1	R
>32	R	>16	R	>2	R	>128	R	>4	R	>1	R
>32	R	>16	R	>2	R	1	I	4	R	>1	R
>32	ESBL	>16	R*	>2	R	1	R	<=1	S	1	I
4	R	>16	R	<=0.5	S	1	R	<=1	S	<=0.5	S
>32	R	>16	R	2	I	>128	R	>4	R	>1	R
>32	R	>16	R	>2	R	0.5	I	>4	R	>1	R
>32	R	>16	R	>2	R	0.5	I	>4	R	>1	R
>32	R	>16	R	>2	R	0.5	I	4	R	>1	R
>32	ESBL	>16	R*	>2	R	0.5	I	<=1	S	>1	R
>32	R	>16	R	<=0.5	S	>128	R	>4	R	>1	R
>32	ESBL	>16	R*	>2	R	0.5	I	<=1	S	>1	R
>32	ESBL	>16	R*	>2	R	1	I	<=1	S	>1	R
>32	ESBL	>16	R*	2	I	1	I	<=1	S	1	I
>32	ESBL	>16	R*	2	I	1	I	<=1	S	1	I
>32	R	>16	R	>2	R	1	I	>4	R	>1	R
>32	ESBL	>16	R*	<=0.5	S	1	I	4	R	>1	R

>32	ESBL	>16	R*	>2	R	1	I	2	I	>1	R
>32	R	>16	R	>2	R	1	I	>4	R	>1	R
>32	ESBL	>16	R*	>2	R	1	I	>4	R	>1	R
>32	R	>16	R	>2	R	1	I	>4	R	>1	R
32	ESBL	>16	R*	>2	R	1	I	4	R	<=0.5	S
>32	R	>16	R	>2	R	0.5	I	4	R	>1	R
>32	ESBL	>16	R*	>2	R	0.5	I	2	I	>1	R
>32	R	>16	R	>2	R	0.5	I	>4	R	>1	R
>32	ESBL	>16	R*	>2	R	0.5	I	<=1	S	>1	R
>32	ESBL	>16	R*	>2	R	0.5	I	2	I	>1	R
>32	R	>16	R	>2	R	1	I	>4	R	>1	R
>32	R	>16	R	>2	R	1	I	>4	R	>1	R
>32	R	>16	R	>2	R	1	I	>4	R	>1	R
>32	ESBL	>16	R*	2	I	>128	R	<=1	S	>1	R
>32	ESBL	>16	R*	1	S	0.5	I	<=1	S	1	I
>32	R	>16	R	2	I	1	I	2	I	>1	R
>32	R	>16	R	2	I	0.5	I	<=1	S	<=0.5	S
>32	ESBL	>16	R*	>2	R	1	I	<=1	S	1	I
>32	R	>16	R	2	I	1	I	2	I	>1	R
>32	R	>16	R	>2	R	0.5	I	>4	R	>1	R
>32	R	>16	R	>2	R	1	I	<=1	S	>1	R
>32	ESBL	>16	R*	>2	R	0.5	I	<=1	S	>1	R
>32	ESBL	>16	R*	>2	R	0.5	I	4	R	>1	R
>32	R	>16	R	>2	R	1	I	>4	R	>1	R
<=1	R*	8	R*	2	I	0.5	I	<=1	S	1	I
>32	R	>16	R	>2	R	1	I	>4	R	>1	R
>32	R	>16	R	>2	R	0.5	I	>4	R	>1	R
>32	R	>16	R	>2	R	>128	R	>4	R	>1	R
>32	R	>16	R	>2	R	0.5	I	>4	R	>1	R
>32	ESBL	>16	R*	2	I	0.5	I	<=1	S	1	I
>32	ESBL	>16	R*	<=0.5	S	0.5	I	<=1	S	<=0.5	S
>32	ESBL	>16	R*	>2	R	0.5	I	<=1	S	>1	R
N/R		>16	R	<=0.5	S	>128	R	>4	R	>1	R
>32	ESBL	>16	R*	>2	R			2	I	>1	R
>32	ESBL	>16	R*	2	I	0.5	I	4	R	>1	R
32	R	>16	R	>2	R	>128	R	>4	R	>1	R
>32	ESBL	>16	R*	2	I	0.5	I	<=1	S	1	I
>32	ESBL	>16	R*	2	I	0.5	I	2	I	>1	R
>32	ESBL	>16	R*	>2	R	0.5	I	2	I	>1	R
>32	ESBL	>16	R*	2	I	0.5	I	2	I	<=0.5	S
>32	R	>16	R	>2	R	0.5	I	4	R	>1	R
>32	R	>16	R	>2	R	0.5	I	2	I	>1	R
>32	ESBL	>16	R*	>2	R	4	R	<=1	S	1	I
>32	ESBL	>16	R*	>2	R	0.5	I	<=1	S	>1	R
>32	ESBL	>16	R*	<=0.5	S	0.5	I	4	R	>1	R
>32	ESBL	>16	R*	2	I	0.5	I	<=1	S	1	I
>32	R	>16	R	>2	R	0.5	I	>4	R	>1	R
>32	R	>16	R	2	I	0.5	I	>4	R	>1	R
>32	ESBL	>16	R*	>2	R	0.5	I	<=1	S	>1	R
>32	ESBL	>16	R*	>2	R	0.5	I	<=1	S	>1	R
>32	ESBL	>16	R*	>2	R	0.5	I	<=1	S	>1	R
>32	ESBL	>16	R*	>2	R	0.5	I	<=1	S	>1	R
>32	R	>16	R	>2	R	0.5	I	>4	R	>1	R
>32	ESBL	>16	R*	>2	R	0.5	I	<=1	S	1	I
>32	R	>16	R	<=0.5	S	0.5	I	<=1	S	<=0.5	S
>32	R	>16	R	1	S	0.5	I	>4	R	>1	R

>32	R	>16	R	>2	R	0.5	I	4	R	>1	R
>32	R	>16	R	<=0.5	S	0.5	I	>4	R	>1	R
>32	R	>16	R	<=0.5	S	1	I	>4	R	>1	R
>32	R	>16	R	>2	R	0.5	R	>4	R	>1	R
>32	R	>16	R	>2	R	0.5	I	4	R	>1	R
<=1	S	8	S	1	S	0.5	I	<=1	S	1	I
>32	R	>16	R	>2	R	0.5	I	4	R	>1	R
>32	ESBL	>16	R*	<=0.5	S	1	I	2	I	>1	R
N/R		>16	R	<=0.5	S	>128	R	>4	R	>1	R
>32	R	>16	R	>2	R	32	R	>4	R	>1	R
32	ESBL	>16	R*	<=0.5	S	1	I	<=1	S	<=0.5	S
>32	R	>16	R	<=0.5	S	2	I	>4	R	>1	R
>32	ESBL	>16	R*	>2	R	16	R	4	R	>1	R
>32	ESBL	>16	R*	>2	R	0.5	I	<=1	S	>1	R
>32	R	>16	R	<=0.5	S	0.5	I	4	R	>1	R
>32	R	>16	R	>2	R	0.5	I	>4	R	>1	R
>32	ESBL	>16	R*	2	I	0.5	I	<=1	S	<=0.5	S
>32	R	>16	R	>2	R	0.5	I	4	R	>1	R
<=1	S	<=4	S	<=0.5	S	1	I	<=1	S	<=0.5	S
>32	ESBL	>16	R*	>2	R	1	I	<=1	S	>1	R
>32	R	>16	R	<=0.5	S	4	R	4	R	>1	R
>32	ESBL	>16	R*	2	I	1	I	<=1	S	1	I
>32	R	>16	R	>2	R	1	I	>4	R	>1	R
>32	R	>16	R	2	I	1	I	>4	R	>1	R
>32	ESBL	>16	R*	>2	R	0.5	I	>4	R	>1	R
>32	R	>16	R	>2	R	2	I	>4	R	>1	R
>32	R	>16	R	>2	R	0.5	I	>4	R	>1	R
>32	ESBL	>16	R*	>2	R	0.5	I	<=1	S	>1	R
>32	ESBL	>16	R*	1	S	32	R	<=1	S	>1	R
>32	ESBL	>16	R*	2	I	0.5	I	<=1	S	>1	R
>32	R	>16	R	>2	R	1	I	>4	R	>1	R
>32	ESBL	>16	R*	2	I	1	I	<=1	S	>1	R
>32	R	>16	R	>2	R	0.5	I	>4	R	>1	R
>32	R	>16	R	>2	R	0.5	I	<=1	S	>1	R
>32	R	>16	R	>2	R	0.5	I	>4	R	>1	R
>32	R	>16	R	>2	R	0.5	I	>4	R	>1	R
N/R		>16	R	<=0.5	S	128	R	>4	R	>1	R
>32	R	>16	R	>2	R	0.5	I	>4	R	>1	R
>32	R	>16	R	2	I	0.5	I	>4	R	>1	R
>32	R	>16	R	<=0.5	S	0.5	I	4	R	>1	R
>32	ESBL	>16	R*	2	I	0.5	I	>4	R	>1	R
N/R		>16	R	<=0.5	S	>128	R	<=1	S	<=0.5	S
>32	ESBL	>16	R*	1	S	>128	R	<=1	S	1	I
>32	R	>16	R	>2	R	>128	R	>4	R	>1	R
>32	R	>16	R	>2	R	4	I	>4	R	>1	R
>32	ESBL	>16	R*	>2	R	0.5	I	<=1	S	1	I
>32	R	>16	R	>2	R	>128	R	>4	R	>1	R
>32	R	>16	R	>2	R	2	I	>4	R	>1	R
>32	R	>16	R	>2	R	>128	R	>4	R	>1	R
>32	R	>16	R	>2	R	0.5	I	>4	R	>1	R
>32	ESBL	>16	R*	>2	R	0.5	I	2	I	>1	R
>32	R	>16	R	>2	R	1	I	>4	R	>1	R
<=1	S	16	I	<=0.5	S	0.5	I	<=1	S	<=0.5	S
>32	R	>16	R	>2	R	>128	R	>4	R	>1	R
>32	R	>16	R	>2	R	1	I	>4	R	>1	R
>32	R	>16	R	>2	R	>128	R	>4	R	>1	R

>32	R	>16	R	>2	R	0.5	I	>4	R	>1	R
>32	ESBL	>16	R*	2	I	0.5	I	<=1	S	>1	R
N/R		>16	R	<=0.5	S	>128	R	>4	R	>1	R
>32	ESBL	>16	R*	<=0.5	S	0.5	I	4	R	>1	R
>32	R	>16	R	>2	R	>128	R	>4	R	>1	R
N/R		>16	R	2	I	>128	R	>4	R	>1	R
N/R		>16	R*	1	S	>128	R	>4	R	>1	R
>32	R	>16	R	>2	R	>128	R	>4	R	>1	R
2	I	>16	R	<=0.5	S	2	I	>4	R	>1	R
>32	R	>16	R	>2	R	>128	R	>4	R	>1	R
>32	R	>16	R	>2	R	0.5	I	>4	R	>1	R
>32	R	>16	R	>2	R	0.5	I	>4	R	>1	R
>32	ESBL	>16	R*	2	I	0.5	I	<=1	S	1	I
>32	R	>16	R	>2	R	0.5	I	>4	R	>1	R
>32	R	>16	R	>2	R	0.5	I	<=1	S	>1	R
>32	R	>16	R	>2	R	0.5	I	<=1	S	>1	R
>32	ESBL	>16	R*	>2	R	1	I	>4	R	>1	R
>32	R	>16	R	<=0.5	S	0.5	I	<=1	S	1	I
>32	R	>16	R	>2	R	0.5	I	>4	R	>1	R
>32	R	>16	R	<=0.5	S	32	R	>4	R	>1	R
>32	R	>16	R	1	S	0.5	I	4	R	>1	R
>32	ESBL	>16	R*	>2	R	0.5	I	4	R	>1	R
>32	R	>16	R	>2	R	0.5	I	>4	R	>1	R
>32	ESBL	>16	R*	>2	R	0.5	I	<=1	S	1	I
>32	ESBL	>16	R*	>2	R	0.5	I	2	I	>1	R
N/R		>16	R	<=0.5	S	>128	R	<=1	S	1	I
>32	R	>16	R	>2	R	0.5	I	4	R	>1	R
>32	ESBL	>16	R*	>2	R	1	I	>4	R	>1	R
>32	R	>16	R	<=0.5	S	0.5	I	>4	R	>1	R
>32	ESBL	>16	R*	2	I	>128	R	2	I	>1	R
>32	ESBL	>16	R*	1	S	1	I	<=1	S	>1	R
>32	R	>16	R	>2	R	64	R	>4	R	>1	R
>32	R	>16	R	>2	R	>128	R	>4	R	>1	R
>32	R	>16	R	<=0.5	S	0.5	I	<=1	S	<=0.5	S
<=1	S	<=4	S	<=0.5	S	2	I	<=1	S	<=0.5	S
>32	R	>16	R	>2	R	32	R	4	R	>1	R
>32	ESBL	>16	R*	>2	R	0.5	I	<=1	S	1	I
8	R	>16	R	>2	R	>128	R	4	R	>1	R
>32	ESBL	>16	R*	<=0.5	S	>128	R	<=1	S	1	I
<=1	S	>16	R	<=0.5	S	>128	R	<=1	S	<=0.5	S
>32	ESBL	>16	R*	1	S	0.5	I	<=1	S	1	I
>32	ESBL	>16	R*	2	I	>128	R	<=1	S	>1	R
>32	R	>16	R	>2	R	>128	R	>4	R	>1	R
>32	ESBL	>16	R*	>2	R	1	I	<=1	S	>1	R
N/R		>16	R	<=0.5	S	>128	R	<=1	S	1	I
>32	R	>16	R	>2	R	>128	R	>4	R	>1	R
>32	ESBL	>16	R*	2	I	>128	R	<=1	S	1	I
>32	R	>16	R	>2	R	0.5	I	>4	R	>1	R
>32	R	>16	R	>2	R	0.5	I	>4	R	>1	R
>32	ESBL	>16	R*	>2	R	0.5	I	<=1	S	1	I
>32	R	>16	R	>2	R	0.5	I	4	R	>1	R
32	R	>16	R	2	I	0.5	I	<=1	S	1	I
>32	ESBL	>16	R*	2	I	2	I	<=1	S	>1	R
N/R		>16	R	2	I	128	R	>4	R	>1	R
>32	R	>16	R	>2	R	>128	R	>4	R	>1	R
>32	R	>16	R	>2	R	>128	R	>4	R	>1	R

>32	R	>16	R	>2	R	0.5	I	>4	R	>1	R
>32	R	>16	R	>2	R	>128	R	>4	R	>1	R
N/R		>16	R	<=0.5	S	>128	R	<=1	S	<=0.5	S
>32	R	>16	R	>2	R	0.5	I	>4	R	>1	R
>32	R	>16	R	>2	R	>128	R	>4	R	>1	R
>32	ESBL	>16	R*	2	I	>128	R	<=1	S	1	I
>32	ESBL	>16	R*	2	I	>128	R	<=1	S	<=0.5	S
<=1	S	16	I	<=0.5	S	>128	R	<=1	S	<=0.5	S
>32	R	>16	R	2	I	0.5	I	>4	R	>1	R
>32	R	>16	R	>2	R	0.5	I	>4	R	>1	R
>32	R	>16	R	>2	R	>128	R	>4	R	>1	R
16	R	>16	R	<=0.5	S	>128	R	<=1	S	<=0.5	S
>32	R	>16	R	>2	R	2	I	4	R	>1	R
>32	R	>16	R	>2	R	>128	R	>4	R	>1	R
>32	R	>16	R	>2	R	>128	R	>4	R	>1	R
>32	ESBL	>16	R*	>2	R	0.5	I	<=1	S	1	I
>32	R	>16	R	>2	R	0.5	I	>4	R	>1	R
>32	ESBL	>16	R*	>2	R	64	R	>4	R	>1	R
>32	R	>16	R	>2	R	0.5	I	>4	R	>1	R
>32	ESBL	>16	R*	1	S	0.5	I	<=1	S	1	I
>32	ESBL	>16	R*	1	S	0.5	I	<=1	S	1	I
>32	R	>16	R	>2	R	>128	R	>4	R	>1	R
>32	ESBL	>16	R*	>2	R	0.5	I	2	I	>1	R
>32	ESBL	>16	R*	>2	R	0.5	I	<=1	S	>1	R
>32	R	>16	R	>2	R	32	R	>4	R	>1	R
>32	ESBL	>16	R*	1	S	0.5	I	<=1	S	1	I
N/R		>16	R	1	S	>128	R	>4	R	>1	R
>32	R	>16	R	>2	R	4	R	>4	R	>1	R
>32	ESBL	>16	R*	>2	R	0.5	I	4	R	>1	R
>32	R	>16	R	>2	R	0.5	I	>4	R	>1	R
>32	ESBL	>16	R*	>2	R	8	R	<=1	S	>1	R
>32	ESBL	>16	R*	>2	R	>128	R	<=1	S	>1	R
<=1	S	<=4	S	<=0.5	S	0.5	I	<=1	S	<=0.5	S
>32	R	>16	R	>2	R	1	I	>4	R	>1	R
4	R	>16	R	>2	R	>128	R	2	I	>1	R
>32	R	>16	R	>2	R	0.5	I	>4	R	>1	R
N/R		>16	R*	<=0.5	S	>128	R	2	I	>1	R
>32	ESBL	>16	R*	2	I	0.5	R	<=1	S	<=0.5	S
>32	R	>16	R	>2	R	0.5	I	>4	R	>1	R
>32	ESBL	>16	R*	2	I	0.5	I	<=1	S	1	I
>32	R	>16	R	>2	R	0.5	I	4	R	>1	R
>32	R	>16	R	2	I	>128	R	>4	R	>1	R
>32	ESBL	>16	R*	1	S	0.5	I	<=1	S	1	I
N/R		>16	R	>2	R	>128	R	>4	R	>1	R
>32	R	>16	R	>2	R	0.5	I	>4	R	>1	R
>32	R	>16	R	>2	R	0.5	I	4	R	>1	R
>32	R	>16	R	>2	R	0.5	I	>4	R	>1	R
>32	R	>16	R	>2	R	0.5	I	>4	R	>1	R
>32	R	>16	R	>2	R	0.5	I	>4	R	>1	R
>32	R	>16	R	<=0.5	S	0.5	I	>4	R	>1	R
>32	R	>16	R	>2	R	0.5	I	>4	R	>1	R
<=1	S	<=4	S	<=0.5	S	1	I	<=1	S	<=0.5	S
>32	R	>16	R	<=0.5	S	05	I	4	R	>1	R
>32	R	>16	R	<=0.5	S	1	I	>4	R	>1	R
>32	R	>16	R	>2	R	0.5	I	>4	R	>1	R

>32	ESBL	>16	R*	>2	R	8	R	<=1	S	>1	R
>32	ESBL	>16	R*	1	S	0.5	I	<=1	S	1	I
>32	R	>16	R	>2	R	0.5	I	>4	R	>1	R
>32	R	>16	R	>2	R	0.5	I	>4	R	>1	R
>32	R	>16	R	>2	R	2	I	>4	R	>1	R
>32	R	>16	R	>2	R	0.5	I	>4	R	>1	R
>32	R	>16	R	>2	R	>128	R	4	R	>1	R
>32	R	>16	R	>2	R	0.5	I	>4	R	>1	R
>32	R	>16	R	2	I	0.5	I	>4	R	>1	R
>32	R	>16	R	>2	R	0.5	I	>4	R	>1	R
>32	ESBL	>16	R*	2	I	0.5	I	<=1	S	<=0.5	S
>32	R	>16	R	>2	R	0.5	I	>4	R	>1	R
>32	ESBL	>16	R*	2	I	1	I	<=1	S	1	I
>32	ESBL	>16	R*	2	I	0.5	I	<=1	S	1	I
>32	R	>16	R	1	S	0.5	I	>4	R	>1	R
>32	R	>16	R	<=0.5	S	>128	R	>4	R	>1	R
>32	R	>16	R	2	I	0.5	I	>4	R	>1	R
>32	ESBL	>16	R*	>2	R	1	I	4	R	>1	R
N/R		>16	R	<=0.5	S	>128	R	>4	R	>1	R
>32	ESBL	>16	R*	2	I	0.5	I	<=1	S	>1	R
>32	R	>16	R	>2	R	0.5	I	>4	R	>1	R
<=1	S	8	S	1	S	0.5	I	<=1	S	1	I
>32	ESBL	>16	R*	2	I	1	I	<=1	S	>1	R
>32	R	>16	R	>2	R	0.5	I	<=1	S	<=0.5	S
>32	ESBL	>16	R*	>2	R	0.5	I	4	R	>1	R
>32	R	>16	R	>2	R	1	I	>4	R	>1	R
>32	R	>16	R	<=0.5	S	0.5	I	<=1	S	1	I

>16	>16	R	>2	R	>128	R	>4	R	>1	R

R	>16	>16	R	>2	R	0.5	I	>4	R	>1	R
R	>16	>16	R	<=0.5	S	0.5	I	>4	R	>1	R

R	>16	>16	R	>2	R	>128	R	>4	R	>1	R

>32	R	>16	R	1	S	0.5	I	4	R	>1	R
>32	R	>16	R	>2	R	2	I	>4	R	>1	R
>32	R	>16	R	>2	R	0.5	I	>4	R	>1	R
>32	R	>16	R	1	S	>128	R	>4	R	>1	R
>32	R	>16	R	>2	R	0.25	I	4	R	>1	R
>32	R	>16	R	>2	R	1	I	>4	R	>1	R
>32	R	>16	R	>2	R	1	I	>4	R	>1	R
>32	ESBL	>16	R*	1	S	1	I	<=1	S	>1	R
>32	R	>16	R	>2	R	1	I	>4	R	>1	R
>32	R	>16	R	1	S	0.5	I	>4	R	>1	R
>32	R	>16	R	>2	R	16	R	>4	R	>1	R
<=1	S	>16	R	>2	R	1	I	<=1	S	>1	R
>32	ESBL	>16	R*	>2	R	0.5	I	<=1	S	>1	R
>32	R	>16	R	>2	R	0.5	I	4	R	>1	R
>32	ESBL	>16	R*	<=0.5	S	0.5	I	<=1	S	1	I
>32	R	>16	R	>2	R	0.5	I	>4	R	>1	R
<=1	S	16	I	<=0.5	S	4	R	<=1	S	<=0.5	S
>32	R	>16	R	>2	R	0.5	I	>4	R	>1	R

>32	ESBL	>16	R*	2	I	0.5	I	<=1	S	>1	R
N/R		>16	R	2	I	>128	R	4	R	>1	R
>32	R	>16	R	>2	R	0.5	I	>4	R	>1	R
>32	R	>16	R	>2	R	0.5	I	>4	R	>1	R
>32	R	>16	R	1	S	1	I	4	R	>1	R
>32	R	>16	R	1	S	0.5	I	4	R	>1	R
>32	R	>16	R	2	I	16	R	4	R	>1	R
<=1	S	>16	R	>2	R	0.5	I	<=1	S	>1	R
>32	R	>16	R	>2	R	0.5	I	>4	R	>1	R
>32	R	>16	R	>2	R	0.5	I	4	R	>1	R
>32	R	>16	R	>2	R	0.5	I	>4	R	>1	R
>32	R	>16	R	>2	R	0.25	I	>4	R	>1	R
R	>16	>16	R	>2	R	2	I	>4	R	>1	R
R	>16	>16	R	>2	R	2	I	>4	R	>1	R
>32	R	>16	R	>2	R	0.25	I	>4	R	>1	R
>32	R	>16	R	1	S	0.5	I	>4	R	>1	R
>32	R	>16	R	>2	R	0.5	I	4	R	>1	R
>32	R	>16	R	1	S	0.5	I	>4	R	>1	R
>32	R	>16	R	>2	R	1	R	>4	R	>1	R
>32	R	>16	R	<=0.5	S	>128	R	>4	R	>1	R
>32	R	>16	R	>2	R	>128	R	>4	R	>1	R
>32	R	>16	R	1	S	0.5	I	>4	R	>1	R
>32	R	>16	R	<=0.5	S	>128	R	>4	R	>1	R
>32	R	>16	R	1	S	0.5	I	>4	R	>1	R
>32	ESBL	>16	R*	>2	R	2	I	<=1	S	>1	R
>32	ESBL	>16	R*	>2	R	0.5	I	<=1	S	<=0.5	S
R	>16	>16	R	>2	R	4	R	>4	R	>1	R
ESBL	>16	>16	R*	>2	R	0.5	I	4	R	>1	R
R	>16	>16	R	>2	R	1	I	<=1	S	>1	R
R	>16	>16	R	>2	R	1	I	4	R	>1	R
R	>16	>16	R	>2	R	1	I	>4	R	>1	R
R	>16	>16	R	>2	R	1	I	>4	R	>1	R
R	>16	>16	R	>2	R	8	R	>4	R	>1	R
R	>16	>16	R	>2	R	2	I	>4	R	>1	R
R	>16	>16	R	2	I	2	I	>4	R	>1	R
>32	ESBL	>16	R*	1	S	8	R	<=1	S	1	I
R	>16	>16	R	>2	R	1	I	>4	R	>1	R
R	>16	>16	R	1	S			>4	R	>1	R
R	>16	>16	R	>2	R	0.5	I	>4	R	>1	R
R	>16	>16	R	>2	R	>128	R	4	R	>1	R
ESBL	>16	>16	R*	>2	R	0.5	I	2	I	>1	R
R	>16	>16	R	>2	R	0.5	I	2	I	>1	R
R	>16	>16	R	>2	R	1	I	4	R	>1	R
R	>16	>16	R	>2	R	32	R	4	R	>1	R
R	>16	>16	R	>2	R	0.5	I	>4	R	>1	R
R	>16	>16	R	>2	R	0.5	I	>4	R	>1	R
R	>16	>16	R	>2	R	0.5	I	>4	R	>1	R
R	>16	>16	R	>2	R	>128	R	>4	R	>1	R
R	>16	>16	R	>2	R	>128	R	>4	R	>1	R
R	>16	>16	R	2	I	128	R	>4	R	>1	R
R	>16	>16	R	>2	R	0.5	I	>4	R	>1	R
R	>16	>16	R	>2	R	>128	R	>4	R	>1	R
R	>16	>16	R	>2	R	128	R	>4	R	>1	R
ESBL	>16	>16	R*	<=0.5	S	0.5	I	<=1	S	>1	R
ESBL	>16	>16	R*	>2	R	0.5	I	4	R	>1	R
	2	>16	R	>2	R	>128	R	>4	R	>1	R

R	>16	>16	R	>2	R	16	R	4	R	>1	R
R	>16	>16	R	>2	R	0.5	I	4	R	>1	R
R	>16	>16	R	>2	R	>128	R	4	R	>1	R
ESBL	2	>16	R*	>2	R	>128	R	>4	R	>1	R
R	>16	>16	R	>2	R	>128	R	>4	R	>1	R
	>16	>16	R*	>2	R	>128	R	2	I	>1	R
R	>16	>16	R	>2	R	128	R	>4	R	>1	R
R	>16	>16	R	>2	R	>128	R	>4	R	>1	R
R	>16	>16	R	>2	R	0.5	I	>4	R	>1	R
R	>16	>16	R	>2	R	0.5	I	4	R	>1	R
R	>16	>16	R	>2	R	>128	R	>4	R	>1	R
R	>16	>16	R	<=0.5	S	>128	R	>4	R	>1	R
ESBL	>16	>16	R*	>2	R	0.5	I	<=1	S	1	I
S	<=1	8	S	1	S	0.5	I	<=1	S	<=0.5	S
R	>16	>16	R	<=0.5	S	>128	R	>4	R	>1	R
R	>16	>16	R	>2	R	128	R	>4	R	>1	R
R	>16	>16	R	<=0.5	S	0.5	I	4	R	>1	R
R	>16	>16	R	>2	R	>128	R	<=1	S	>1	R
ESBL	>16	>16	R*	1	S	0.5	I	<=1	S	1	I
ESBL	>16	>16	R*	>2	R	0.5	I	4	R	>1	R
R	>16	>16	R	>2	R	>128	R	2	I	>1	R
ESBL	>16	>16	R*	2	I	0.5	I	2	I	>1	R
ESBL	>16	>16	R*	>2	R	0.5	I	<=1	S	>1	R
R	>16	>16	R	>2	R	1	I	>4	R	>1	R
R	>16	>16	R	>2	R	0.5	I	>4	R	>1	R
	8	>16	R	2	I	>128	R	2	I	>1	R
ESBL	>16	>16	R*	>2	R	128	R	2	I	>1	R
R	>16	>16	R	>2	R	>128	R	>4	R	>1	R
R	>16	>16	R	>2	R	>128	R	<=1	S	>1	R
R	>16	>16	R	>2	R	>128	R	2	I	>1	R
R	>16	>16	R	>2	R	0.5	I	>4	R	>1	R
R	>16	>16	R	<=0.5	S	>128	R	>4	R	>1	R
ESBL	>16	>16	R*	1	S	>128	R	2	I	>1	R
R	>16	>16	R	>2	R	>128	R	>4	R	>1	R
R	>16	>16	R	>2	R	>128	R	>4	R	>1	R
R	>16	>16	R	>2	R	0.5	I	>4	R	>1	R
R	>16	>16	R	>2	R	>128	R	>4	R	>1	R
ESBL	>16	>16	R*	>2	R	>128	R	<=1	S	>1	R
R	>16	>16	R	>2	R	2	I	>4	R	>1	R
R	>16	>16	R	>2	R	>128	R	>4	R	>1	R
	>16	>16	R	>2	R	>128	R	>4	R	>1	R
ESBL	16	>16	R*	1	S	0.5	I	<=1	S	1	I
ESBL	>16	>16	R*	>2	R	0.5	I	<=1	S	<=0.5	S
R	>16	>16	R	>2	R	0.5	I	>4	R	>1	R
R	>16	>16	R	<=0.5	S	0.5	I	>4	R	>1	R
R	>16	>16	R	>2	R	8	R	>4	R	>1	R
ESBL	>16	>16	R*	>2	R	0.5	I	<=1	S	>1	R
R	<=1	>16	R	<=0.5	S	0.5	I	<=1	S	>1	R
ESBL	<=1	8	R*	<=0.5	S	0.5	I	<=1	S	<=0.5	S
R	>16	>16	R	<=0.5	S	0.5	I	>4	R	>1	R
R	>16	>16	R	>2	R	0.5	I	>4	R	>1	R
ESBL	>16	>16	R*	>2	R	0.5	I	4	R	>1	R
R	>16	>16	R	<=0.5	S	>128	R	>4	R	>1	R
R	>16	>16	R	<=0.5	S	16	R	4	R	>1	R
ESBL	>16	>16	R*	>2	R	128	R	4	R	>1	R

ESBL	>16	>16	R*	1	S	0.5	I	<=1	S	1	I
ESBL	>16	>16	R*	1	S	0.5	I	<=1	S	<=0.5	S
R	>16	>16	R	>2	R	0.5	I	2	I	>1	R
ESBL	>16	>16	R*	1	S	0.5	I	<=1	S	1	I
R	>16	>16	R	1	S	0.5	I	>4	R	>1	R
R	>16	>16	R	1	S	0.5	I	>4	R	>1	R
R	>16	>16	R	2	I	0.5	I	>4	R	>1	R
R	>16	>16	R	>2	R	1	I	4	R	>1	R
R	>16	>16	R	<=0.5	S	0.5	I	>4	R	>1	R
R	>16	>16	R	>2	R	1	I	>4	R	>1	R
ESBL	16	>16	R*	2	I	1	R	<=1	S	1	I
ESBL	>16	>16	R*	>2	R	0.5	I	>4	R	>1	R
ESBL	>16	>16	R*	2	I	0.5	I	<=1	S	1	I
R	>16	>16	R	>2	R	0.5	I	>4	R	>1	R
ESBL	>16	>16	R*	>2	R	1	I	2	I	>1	R
ESBL	>16	>16	R*	>2	R	0.5	I	4	R	>1	R
ESBL	>16	>16	R*	>2	R	0.5	I	4	R	>1	R
ESBL	>16	>16	R*	>2	R	0.5	I	<=1	S	>1	R
R	<=1	>16	R	<=0.5	S	0.5	I	<=1	S	<=0.5	S
>32	ESBL	>16	R*	2	I	0.5	I	<=1	S	1	I
N/R		>16	R	>2	R	>128	R	4	R	>1	R
>32	R	>16	R	>2	R	0.5	I	2	I	>1	R
>32	R	>16	R	1	S	1	I	>4	R	>1	R
>32	ESBL	>16	R*	>2	R	16	R	4	R	>1	R
>32	ESBL	>16	R*	>2	R	0.5	I	4	R	>1	R
>32	ESBL	>16	R*	2	I	0.5	I	<=1	S	>1	R
>32	R	>16	R	>2	R	1	I	>4	R	>1	R
>32	ESBL	>16	R*	2	I	0.5	I	<=1	S	<=0.5	S
>32	R	>16	R	>2	R	0.5	I	>4	R	>1	R
>32	ESBL	>16	R*	1	S	1	I	<=1	S	1	I
>32	ESBL	>16	R*	>2	R	1	I	2	I	>1	R
>32	R	>16	R	>2	R	1	I	4	R	>1	R
>32	R	>16	R	1	S	0.5	I	2	I	>1	R
ESBL	>16	>16	R*	>2	R	0.5	I	>4	R	>1	R
ESBL	>16	>16	R*	>2	R	0.5	I	4	R	>1	R
ESBL	>16	>16	R*	>2	R	0.5	I	4	R	>1	R
R	>16	>16	R	>2	R	1	I	>4	R	>1	R
R	>16	>16	R	>2	R	0.5	I	>4	R	>1	R
R	>16	>16	R	>2	R	0.5	I	>4	R	>1	R
ESBL	>16	>16	R*	>2	R	0.5	I	4	R	>1	R
R	>16	>16	R	1	S	>128	R	>4	R	>1	R
	>16	>16	R*	>2	R	>128	R	<=1	S	>1	R
	>16	>16	R	>2	R	>128	R	>4	R	>1	R
R	>16	>16	R	>2	R	32	R	>4	R	>1	R
ESBL	4	>16	R*	2	I	0.5	I	2	I	>1	R
>32	R	>16	R	>2	R	0.5	I	>4	R	>1	R
>32	R	>16	R	<=0.5	S	1	I	4	R	>1	R
>32	ESBL	>16	R*	2	I	8	R	<=1	S	1	I
<=1	S	<=4	S	<=0.5	S	0.5	I	<=1	S	<=0.5	S
>32	R	>16	R	>2	R	0.5	I	<=1	S	>1	R
>32	R	>16	R	<=0.5	S	0.5	I	4	R	>1	R
>32	R	>16	R	<=0.5	S	1	I	4	R	>1	R
>32	R	>16	R	>2	R	0.5	I	4	R	>1	R
N/R		>16	R	>2	R	>128	R	>4	R	>1	R
>32	R	>16	R	>2	R	16	R	>4	R	>1	R

>32	R	>16	R	>2	R	0.5	I	>4	R	>1	R
>32	R	>16	R	2	I	0.5	I	>4	R	>1	R
>32	ESBL	>16	R*	1	S	0.5	I	<=1	S	1	I
>32	ESBL	>16	R*	1	S	0.5	I	<=1	S	>1	R
>32	ESBL	>16	R*	>2	R	0.5	I	<=1	S	>1	R
>32	R	>16	R	>2	R	8	R	>4	R	>1	R
>32	ESBL	>16	R*	>2	R	0.5	I	4	R	>1	R
>32	ESBL	>16	R*	>2	R	0.5	I	4	R	>1	R
>32	R	>16	R	>2	R	0.5	I	4	R	>1	R
>32	R	>16	R	>2	R	0.5	I	4	R	>1	R
>32	ESBL	>16	R*	>2	R	0.5	I	4	R	>1	R
>32	R	>16	R	>2	R	8	R	>4	R	>1	R
>32	ESBL	>16	R*	>2	R	0.5	I	<=1	S	>1	R
>32	R	>16	R	>2	R	0.5	I	2	I	>1	R
>32	ESBL	>16	R*	1	S	0.5	I	<=1	S	1	I
>32	R	>16	R	>2	R	0.5	I	>4	R	>1	R
>32	R	>16	R	>2	R	0.5	I	<=1	S	>1	R
>32	ESBL	>16	R*	2	I			<=1	S	>1	R
<=1	S	<=4	S	<=0.5	S	0.5	I	<=1	S	1	I
>32	ESBL	>16	R*	2	I	1	I	<=1	S	1	I
N/R		>16	R	>2	R	>128	R	>4	R	>1	R
>32	R	>16	R	>2	R	0.5	I	4	R	>1	R
>32	R	>16	R	<=0.5	S	>128	R	>4	R	>1	R
>32	ESBL	>16	R*	1	S	0.5	I	<=1	S	<=0.5	S
>32	ESBL	>16	R*	<=0.5	S	0.5	I	<=1	S	>1	R
>32	R	>16	R	>2	R	0.5	I	>4	R	>1	R
>32	R	>16	R	1	S	64	R	2	I	<=0.5	S
>32	ESBL	>16	R*	>2	R	0.5	I	<=1	S	<=0.5	S
>32	ESBL	>16	R*	>2	R	0.5	I	<=1	S	1	I
>32	R	>16	R	>2	R	0.5	I	4	R	>1	R
>32	ESBL	>16	R*	>2	R	0.5	I	<=1	S	1	I
>32	R	>16	R	>2	R	1	I	>4	R	>1	R
>32	R	>16	R	>2	R	1	I	>4	R	>1	R
>32	R	>16	R	>2	R	0.5	I	<=1	S	>1	R
>32	ESBL	>16	R*	2	I	0.5	I	<=1	S	1	I
N/R		>16	R*	1	S	>128	R	<=1	S	1	I
>32	ESBL	>16	R*	>2	R	>128	R	>4	R	>1	R
>32	R	>16	R	>2	R	>128	R	4	R	>1	R
>32	R	>16	R	>2	R	128	R	4	R	>1	R
>32	R	>16	R	>2	R	128	R	>4	R	>1	R
>32	R	>16	R	>2	R	0.5	I	>4	R	>1	R
>32	R	>16	R	2	I	0.5	I	>4	R	>1	R
>32	R	>16	R	>2	R	1	I	>4	R	>1	R
>32	ESBL	>16	R*	<=0.5	S	4	R	<=1	S	>1	R
>32	ESBL	>16	R*	1	S	0.5	I	2	I	>1	R
4	R	>16	R	>2	R	1	I	>4	R	>1	R
>32	R	>16	R	>2	R	64	R	4	R	>1	R
>32	R	>16	R	<=0.5	S	>128	R	>4	R	>1	R
>32	R	>16	R	>2	R	0.5	S	4	R	>1	R
>32	ESBL	>16	R*	>2	R	0.5	I	4	R	>1	R
>32	R	>16	R	>2	R	16	R	4	R	>1	R
>32	R	>16	R	<=0.5	S	0.5	I	>4	R	>1	R
>32	R	>16	R	>2	R	0.5	I	4	R	>1	R
>32	R	>16	R	>2	R	0.5	S	4	R	>1	R
N/R		>16	R*	<=0.5	S	>128	R	<=1	S	>1	R
>32	R	>16	R	<=0.5	S	>128	R	>4	R	>1	R

>32	R	>16	R	>2	R	0.5		>4	R	>1	R
>32	ESBL	>16	R*	>2	R	16	R	>4	R	>1	R
>32	ESBL	>16	R*	>2	R	1	I	<=1	S	1	I
>32	ESBL	>16	R*	2	I	2	I	<=1	S	1	I
>32	R	>16	R	<=0.5	S	0.5	S	>4	R	>1	R
>32	ESBL	>16	R*	>2	R	0.5	I	<=1	S	1	I
>32	R	>16	R	>2	R	1	I	>4	R	>1	R
>32	ESBL	>16	R*	>2	R	0.5	I	4	R	>1	R
<=1	S	8	S	2	I	0.5	I	<=1	S	<=0.5	S
>32	R	>16	R	>2	R	16	R	>4	R	>1	R
>32	R	>16	R	>2	R	0.5	I	2	I	>1	R
>32	R	>16	R	>2	R	0.5	I	>4	R	>1	R
>32	ESBL	>16	R*	2	I	0.5	I	<=1	S	<=0.5	S
>32	R	>16	R	<=0.5	S	>128	R	>4	R	>1	R
>32	R	>16	R	>2	R	0.5	I	4	R	>1	R
>32	R	>16	R	>2	R	0.25	I	>4	R	>1	R
>32	ESBL	>16	R*	2	I	1	I	<=1	S	1	I
>32	ESBL	>16	R*	2	I	1	I	<=1	S	1	I
>32	ESBL	>16	R*	2	I	0.5	I	<=1	S	1	I
>32	R	>16	R	>2	R	0.5	I	<=1	S	>1	R
>32	ESBL	>16	R*	1	S	1	I	<=1	S	<=0.5	S
>32	R	>16	R	>2	R	0.5	I	>4	R	>1	R
>32	ESBL	>16	R*	1	S	1	I	4	R	>1	R
>32	R	>16	R	>2	R	8	R	4	R	>1	R
>32	R	>16	R	>2	R	>128	R	4	R	>1	R
>32	ESBL	>16	R*	>2	R	16	R	<=1	S	>1	R
>32	R	>16	R	>2	R	1	I	>4	R	>1	R
>32	R	>16	R	>2	R	8	R	2	I	>1	R
>32	R	>16	R	>2	R	0.5	I	<=1	S	>1	R
>32	R	>16	R	<=0.5	S	32	R	4	R	>1	R
>32	R	>16	R	>2	R	16	R	2	I	>1	R
N/R		>16	R	>2	R	>128	R	>4	R	>1	R
>32	R	>16	R	>2	R	8	R	>4	R	>1	R
>32	R	>16	R	>2	R	1	I	4	R	>1	R
>32	R	>16	R	>2	R	8	R	<=1	S	>1	R
>32	R	>16	R	>2	R	0.5	I	>4	R	>1	R
>32	R	>16	R	2	I	0.5	I	>4	R	>1	R
>32	R	>16	R	>2	R	0.5	I	>4	R	>1	R
>32	R	>16	R	>2	R	0.5	I	>4	R	>1	R
>32	R	>16	R	<=0.5	S	4	R	2	I	1	I
>32	R	>16	R	>2	R	0.5	R	4	R	>1	R
>32	ESBL	>16	R*	>2	R	32	R	4	R	>1	R

>32	ESBL	>16	R*	2	I	1	I	4	R	1	I

>32	R	>16	R	<=0.5	S	0.5	I	2	I	>1	R
>32	R	>16	R	1	S	0.5	I	4	R	>1	R
>32	ESBL	>16	R*	1	S	0.5	I	<=1	S	1	I
>32	ESBL	>16	R*	>2	R	2	I	>4	R	>1	R
>32	ESBL	>16	R*	>2	R	64	R	<=1	S	1	I
>32	R	>16	R	>2	R	0.5	I	>4	R	>1	R
>32	R	>16	R	>2	R	0.5	I	4	R	>1	R
>32	R	>16	R	>2	R	1	I	>4	R	>1	R
>32	R	>16	R	<=0.5	S	32	R	>4	R	>1	R
>32	ESBL	>16	R*	>2	R	0.5	S	4	R	>1	R
>32	R	>16	R	>2	R	32	R	>4	R	>1	R
>32	R	>16	R	>2	R	32	R	>4	R	>1	R
>32	ESBL	>16	R*	>2	R	1	I	4	R	1	I
>32	ESBL	>16	R*	>2	R	2	I	<=1	S	<=0.5	S
>32	ESBL	>16	R*	>2	R	0.5	I	<=1	S	>1	R
>32	ESBL	>16	R*	1	S	0.5	I	<=1	S	<=0.5	S
>32	R	>16	R	>2	R	1	I	4	R	>1	R
>32	ESBL	>16	R*	>2	R	0.5	I	<=1	S	1	I
>32	ESBL	>16	R*	<=0.5	S	0.5	I	<=1	S	1	I
N/R		>16	R	2	I	128	R	>4	R	>1	R
>32	ESBL	>16	R*	2	I	128	R	<=1	S	<=0.5	S
>32	R	>16	R	>2	R	1	I	4	R	>1	R
>32	R	>16	R	>2	R	0.5	I	>4	R	>1	R

>32	R	>16	R	>2	R	16	R	>4	R	>1	R
>32	R	>16	R	>2	R	64	R	>4	R	>1	R
>32	R	>16	R	>2	R	128	R	>4	R	>1	R
>32	R	>16	R	>2	R	8	R	>4	R	>1	R
>32	R	>16	R	>2	R	16	R	4	R	>1	R
>32	R	>16	R	>2	R	16	R	2	I	>1	R
>32	R	>16	R	>2	R	2	I	>4	R	>1	R
>32	R	>16	R	>2	R	0.5	I	<=1	S	1	I
>32	R	>16	R	>2	R	1	I	4	R	>1	R
>32	R	>16	R	>2	R	1	I	>4	R	>1	R
>32	R	>16	R	>2	R	4	R	>4	R	>1	R
>32	R	>16	R	>2	R	1	I	>4	R	>1	R
>32	R	>16	R	>2	R	128	R	>4	R	>1	R
>32	R	>16	R	>2	R	0.5	I	4	R	>1	R
>32	R	>16	R	>2	R	0.5	I	>4	R	>1	R
>32	R	>16	R	>2	R	16	R	>4	R	>1	R
>32	R	>16	R	>2	R	128	R	>4	R	>1	R
>32	ESBL	>16	R*	>2	R	128	R	4	R	>1	R
>32	ESBL	>16	R*	>2	R	64	R	>4	R	>1	R
>32	R	>16	R	>2	R	64	R	>4	R	>1	R
>32	R	>16	R	>2	R	64	R	>4	R	>1	R
>32	ESBL	>16	R*	2	I	0.5	I	<=1	S	<=0.5	S
>32	ESBL	>16	R*	>2	R	1	I	<=1	S	1	I
>32	ESBL	>16	R*	1	S	1	I	<=1	S	>1	R
>32	ESBL	>16	R*	>2	R			<=1	S	>1	R
>32	R	>16	R	>2	R	64	R	>4	R	>1	R
>32	R	>16	R	1	S	0.5	I	4	R	>1	R
>32	ESBL	>16	R*	>2	R	64	R	<=1	S	>1	R
>32	R	>16	R	>2	R	16	R	>4	R	>1	R
>32	R	>16	R	>2	R	8	R	>4	R	>1	R
>32	R	>16	R	>2	R	1	I	>4	R	>1	R
>32	ESBL	>16	R*	>2	R	2	I	2	I	>1	R
N/R		>16	R	<=0.5	S	>128	R	2	I	>1	R
>32	ESBL	>16	R*	>2	R	0.5	I	4	R	>1	R
>32	R	>16	R	>2	R	0.5	I	>4	R	>1	R
>32	R	>16	R	>2	R	64	R	>4	R	>1	R
>32	R	>16	R	>2	R	2	I	>4	R	>1	R
>32	R	>16	R	>2	R	16	R	>4	R	>1	R
>32	R	>16	R	>2	R	0.5	I	4	R	>1	R
>32	ESBL	>16	R*	>2	R	1	I	2	I	>1	R
>32	R	>16	R	>2	R	0.5	I	>4	R	>1	R
>32	ESBL	>16	R*	>2	R	0.5	I	4	R	1	I
>32	R	>16	R	>2	R	2	I	2	I	>1	R
>32	ESBL	>16	R*	1	S	0.5	I	<=1	S	1	I
>32	ESBL	>16	R*	1	S	1	I	2	I	>1	R
>32	ESBL	>16	R*	>2	R	1	I	<=1	S	>1	R
>32	R	>16	R	>2	R	0.5	I	2	I	>1	R
>32	ESBL	>16	R*	>2	R	1	I	<=1	S	1	I
>32	R	>16	R	>2	R	64	R	>4	R	>1	R
>32	R	>16	R	>2	R	0.5	I	>4	R	>1	R
>32	R	>16	R	>2	R	0.5	I	>4	R	>1	R
>32	R	>16	R	>2	R	0.5	I	>4	R	>1	R
4	R	>16	R	>2	R	2	I	>4	R	>1	R
>32	R	>16	R	<=0.5	S	>128	R	>4	R	>1	R
>32	ESBL	>16	R*	>2	R	0.5	I	<=1	S	>1	R
>32	ESBL	>16	R*	>2	R	2	I	<=1	S	1	I

16	R	>16	R	>2	R	0.5	I	>4	R	>1	R
>32	ESBL	>16	R*	>2	R	1	I	>4	R	>1	R
>32	R	>16	R	<=0.5	S	128	R	>4	R	>1	R
>32	ESBL	>16	R*	>2	R	0.5	I	<=1	S	>1	R
>32	ESBL	>16	R*	1	S	1	I	<=1	S	<=0.5	S
>32	R	>16	R	>2	R	128	R	>4	R	>1	R
>32	R	>16	R	>2	R	32	R	>4	R	>1	R
>32	R	>16	R	>2	R	0.5	I	>4	R	>1	R
>32	R	>16	R	>2	R	1	I	>4	R	>1	R
>32	R	>16	R	>2	R	0.5	I	<=1	S	1	I
>32	ESBL	>16	R*	<=0.5	S	0.5	I	<=1	S	>1	R
>32	R	>16	R	1	S	0.5	I	2	I	>1	R
>32	ESBL	>16	R*	<=0.5	S	0.5	I	4	R	>1	R
2	I	>16	R	>2	R	0.5	I	>4	R	>1	R
N/R		>16	R	<=0.5	S	128	R	<=1	S	<=0.5	S
N/R		>16	R*	2	I	>128	R	4	R	>1	R
>32	ESBL	>16	R*	<=0.5	S	0.5	I	<=1	S	1	I
>32	ESBL	>16	R*	1	S	0.5	I	<=1	S	<=0.5	S
>32	R	>16	R	<=0.5	S	0.5	I	2	I	>1	R
>32	ESBL	>16	R*	1	S	0.5	I	<=1	S	<=0.5	S
>32	R	>16	R	>2	R	0.5	I	<=1	S	>1	R
>32	R	>16	R	>2	R	1	I	>4	R	>1	R
>32	R	>16	R	>2	R	0.5	I	>4	R	>1	R
>32	ESBL	>16	R*	>2	R	0.5	I	2	I	1	I
>32	R	>16	R	>2	R	0.5	I	2	I	>1	R
>32	R	>16	R	1	S	0.5	I	2	I	>1	R
>32	R	>16	R	>2	R	1	I	>4	R	>1	R
>32	ESBL	>16	R*	>2	R	0.5	I	<=1	S	>1	R
>32	ESBL	>16	R*	2	I	0.5	I	<=1	S	1	I
>32	R	>16	R	>2	R	8	R	>4	R	>1	R
N/R		>16	R	<=0.5	S	>128	R	<=1	S	>1	R
>32	ESBL	>16	R*	2	I	0.5	I	<=1	S	1	I
>32	R	>16	R	>2	R	16	R	>4	R	>1	R
>32	ESBL	>16	R*	2	I	0.5	I	<=1	S	>1	R
>32	ESBL	>16	R*	>2	R	4	I	2	I	>1	R
>32	ESBL	>16	R*	2	I	1	I	<=1	S	1	I
>32	ESBL	>16	R*	>2	R	0.5	I	<=1	S	>1	R
>32	R	>16	R	>2	R			4	R	>1	R
>32	R	>16	R	>2	R	8	R	2	I	>1	R
>32	R	>16	R	>2	R	0.5	I	>4	R	>1	R
>32	R	>16	R	>2	R	0.5	I	>4	R	>1	R
>32	ESBL	>16	R*	>2	R	0.5	I	<=1	S	>1	R
>32	ESBL	>16	R*	>2	R	0.5	I	<=1	S	<=0.5	S
>32	R	>16	R	>2	R	2	I	>4	R	>1	R
>32	R	>16	R	<=0.5	S	0.5	I	>4	R	>1	R
2	I	16	I	>2	R	2	I	<=1	S	>1	R
>32	R	>16	R	>2	R	0.5	I	<=1	S	>1	R
>32	ESBL	>16	R*	1	S	0.5	I	<=1	S	<=0.5	S
>32	R	>16	R	1	S	128	R	>4	R	>1	R
>32	R	>16	R	>2	R	8	R	>4	R	>1	R
>32	R	>16	R	<=0.5	S	8	R	4	R	>1	R
>32	R	>16	R	>2	R	4	R	>4	R	>1	R
>32	ESBL	>16	R*	1	S	0.5	I	<=1	S	>1	R
>32	R	>16	R	<=0.5	S	1	I	2	I	>1	R
>32	R	>16	R	>2	R	0.5	I	<=1	S	>1	R
>32	R	>16	R	>2	R	0.5	I	>4	R	>1	R

>32	R	>16	R	<=0.5	S	0.5	I	4	R	>1	R
>32	R	>16	R	>2	R	>128	R	>4	R	>1	R
>32	ESBL	>16	R*	1	S	16	R	<=1	S	>1	R
N/R		>16	R	>2	R	128	R	>4	R	>1	R
>32	ESBL	>16	R*	<=0.5	S	128	R	<=1	S	>1	R
>32	R	>16	R	>2	R	1	I	<=1	S	>1	R
>32	R	>16	R	>2	R	2	I	2	I	>1	R
>32	ESBL	>16	R*	1	S			<=1	S	1	I
>32	R	>16	R	>2	R	0.5	I	>4	R	>1	R
>32	ESBL	>16	R*	2	I	1	I	<=1	S	1	I
>32	R	>16	R	1	S	>128	R	>4	R	>1	R
>32	R	>16	R	1	S	>128	R	>4	R	>1	R
>32	R	>16	R	>2	R	64	R	>4	R	>1	R
>32	R	>16	R	>2	R	0.5	I	4	R	>1	R
>32	R	>16	R	2	I	0.5	I	>4	R	>1	R
>32	R	>16	R	>2	R	0.5	I	>4	R	>1	R
>32	ESBL	>16	R*	>2	R	0.5	I	<=1	S	<=0.5	S
>32	ESBL	>16	R*	>2	R	1	I	<=1	S	>1	R
>32	ESBL	>16	R*	1	S	0.5	I	2	I	>1	R
>32	R	>16	R	>2	R	8	R	>4	R	>1	R
>32	ESBL	>16	R*	>2	R	4	R	<=1	S	>1	R
>32	R	>16	R	1	S	2	I	4	R	>1	R
>32	ESBL	>16	R*	1	S	2	I	2	I	>1	R
>32	R	>16	R	>2	R	128	R	>4	R	>1	R
>32	R	>16	R	2	I	0.5	I	2	I	>1	R
>32	R	>16	R	1	S	>128	R	>4	R	>1	R
>32	R	>16	R	>2	R	>128	R	>4	R	>1	R
>32	R	>16	R	>2	R	0.5	I	>4	R	>1	R

fosmic	fos	genmic	gen	impmic	imp	lvxmic	lvx	mermic	mer	minmic
<=32	S	>8	R	4	R	>4	R	<=1	S	8
<=32	S	<=2	S	2	I	>4	R	2	I	>8
<=32	S	>8	R	>8	R	>4	R	>8	R	>8
<=32	S	>8	R	>8	R	<=1	S	>8	R	<=4
<=32	S	>8	R	4	R	>4	R	2	I	>8
<=32	S	>8	R	8	R	<=1	S	4	R	<=4
<=32	S	>8	R	8	R	<=1	S	2	I	<=4
64	R	>8	R	8	R	<=1	S	8	R	<=4
<=32	S	>8	R	<=1	S	<=1	S	2	I	>8
<=32	S	>8	R	2	I	>4	R	4	R	<=4
>64	R	>8	R	<=1	S	4	I	<=1	S	>8
>64	R	<=2	S	<=1	S	>4	R	2	I	>8
<=32	S	>8	R	>8	R	>4	R	>8	R	<=4
<=32	S	>8	R	>8	R	>4	R	>8	R	<=4
<=32	S	<=2	S	<=1	S	>4	R	<=1	S	>8
<=32	S	>8	R	<=1	S	>4	R	4	R	>8
<=32	S	<=2	S	<=1	S	2	S	<=1	S	8
<=32	S	>8	R	>8	R	>4	R	>8	R	<=4
<=32	S	>8	R	>8	R	>4	R	>8	R	<=4
<=32	S	>8	R	8	R	>4	R	>8	R	<=4
<=32	S	>8	R	>8	R	>4	R	>8	R	8
<=32	S	>8	R	8	R	<=1	S	>8	R	<=4
<=32	S	>8	R	8	R	>4	R	>8	R	>8
<=32	S	>8	R	>8	R	>4	R	>8	R	<=4
<=32	S	>8	R	>8	R	>4	R	>8	R	<=4
<=32	S	>8	R	<=1	S	>4	R	<=1	S	<=4
<=32	S	>8	R	4	R	>4	R	2	I	<=4
64	R	>8	R	>8	R	>4	R	>8	R	8
<=32	S	>8	R	>8	R	>4	R	>8	R	8
<=32	S	>8	R	4	R	<=1	S	<=1	S	<=4
<=32	S	>8	R	8	R	>4	R	>8	R	<=4
64	R	>8	R	2	I	<=1	S	<=1	S	8
<=32	S	>8	R	<=1	S	>4	R	<=1	S	<=4
>64	R	>8	R	<=1	S	>4	R	<=1	S	>8
<=32	S	>8	R	<=1	S	>4	R	<=1	S	>8
<=32	S	>8	R	>8	R	>4	R	>8	R	<=4
<=32	S	>8	R	>8	R	>4	R	>8	R	<=4
<=32	S	>8	R	>8	R	>4	R	>8	R	<=4
<=32	S	>8	R	8	R	>4	R	>8	R	<=4
<=32	S	>8	R	8	R	<=1	S	8	R	<=4
<=32	S	>8	R	>8	R	4	I	>8	R	>8
<=32	S	>8	R	>8	R	<=1	S	>8	R	<=4
64	R	>8	R	4	R	<=1	S	2	I	<=4
<=32	S	>8	R	4	R	<=1	S	2	I	<=4
>64	R	>8	R	2	I	<=1	S	<=1	S	<=4
<=32	S	>8	R	>8	R	<=1	S	>8	R	>8
<=32	S	>8	R	<=1	S	>4	R	<=1	S	>8
<=32	S	>8	R	8	R	>4	R	>8	R	<=4
>64	R	<=2	S	<=1	S	>4	R	<=1	S	<=4
<=32	S	>8	R	8	R	>4	R	>8	R	<=4
<=32	S	>8	R	<=1	S	>4	R	<=1	S	8

<=32	S	>8	R	<=1	S	>4	R	<=1	S	8
64	R	>8	R	2	I	>4	R	<=1	S	8
<=32	S	>8	R	<=1	S	>4	R	<=1	S	8
<=32	S	>8	R	<=1	S	>4	R	<=1	S	8
<=32	S	>8	R	<=1	S	>4	R	<=1	S	8
<=32	S	>8	R	<=1	S	<=1	S	<=1	S	<=4
<=32	S	<=2	S	<=1	S	>4	R	<=1	S	>8
<=32	S	>8	R	8	R	>4	R	>8	R	<=4
<=32	S	>8	R	>8	R	<=1	S	2	I	8
<=32	S	>8	R	>8	R	>4	R	>8	R	<=4
<=32	S	>8	R	<=1	S	>4	R	<=1	S	>8
<=32	S	>8	R	>8	R	>4	R	>8	R	>8
<=32	S	>8	R	4	R	>4	R	4	R	8

<=32	S	>8	R	8	R	>4	R	>8	R	<=4
64	R	<=2	S	<=1	S	<=1	S	<=1	S	<=4
<=32	S	>8	R	<=1	S	>4	R	<=1	S	>8
<=32	S	>8	R	<=1	S	>4	R	<=1	S	>8
<=32	S	>8	R	4	R	>4	R	8	R	<=4
<=32	S	>8	R	<=1	S	>4	R	<=1	S	8
<=32	S	>8	R	<=1	S	>4	R	<=1	S	>8
<=32	S	<=2	S	<=1	S	>4	R	2	I	8
<=32	S	>8	R	8	R	>4	R	>8	R	8
<=32	S	>8	R	8	R	4	I	8	R	8
<=32	S	>8	R	<=1	S	>4	R	<=1	S	<=4
<=32	S	>8	R	4	R	<=1	S	8	R	<=4
<=32	S	>8	R	8	R	>4	R	>8	R	<=4
<=32	S	>8	R	>8	R	>4	R	>8	R	8
<=32	S	>8	R	<=1	S	>4	R	<=1	S	>8
64	R	>8	R	2	I	>4	R	8	R	<=4
<=32	S	>8	R	<=1	S	4	I	2	I	8
<=32	S	>8	R	>8	R	>4	R	>8	R	>8
<=32	S	>8	R	>8	R	>4	R	>8	R	<=4

<=32	S	<=2	S	4	R	>4	R	2	I	<=4
<=32	S	<=2	S	<=1	S	<=1	S	<=1	S	<=4

<=32	S	>8	R	<=1	S	>4	R	<=1	S	<=4
>64	R	>8	R	>8	R	>4	R	>8	R	>8
64	R	>8	R	>8	R	>4	R	>8	R	<=4
<=32	S	>8	R	8	R	>4	R	>8	R	<=4
<=32	S	>8	R	4	R	>4	R	>8	R	>8
>64	R	>8	R	>8	R	>4	R	>8	R	8
64	R	>8	R	>8	R	>4	R	>8	R	>8
64	R	>8	R	>8	R	>4	R	>8	R	>8
<=32	S	>8	R	<=1	S	<=1	S	<=1	S	<=4
<=32	S	>8	R	4	R	>4	R	2	I	<=4
<=32	S	>8	R	<=1	S	>4	R	<=1	S	8
<=32	S	>8	R	8	R	<=1	S	8	R	<=4
64	R	>8	R	2	I	<=1	S	<=1	S	<=4
<=32	S	<=2	S	<=1	S	>4	R	<=1	S	>8
<=32	S	>8	R	8	R	>4	R	4	R	>8
<=32	S	>8	R	2	I	<=1	S	<=1	S	<=4
<=32	S	<=2	S	<=1	S	<=1	S	<=1	S	<=4
<=32	S	>8	R	<=1	S	>4	R	2	I	>8
>64	R	>8	R	2	I	>4	R	>8	R	>8
64	R	>8	R	>8	R	>4	R	>8	R	>8
>64	R	>8	R	>8	R	>4	R	>8	R	>8
>64	R	>8	R	>8	R	>4	R	>8	R	8
>64	R	>8	R	<=1	S	>4	R	8	R	>8
<=32	S	>8	R	>8	R	2	S	>8	R	8
<=32	S	>8	R	8	R	4	I	>8	R	>8
<=32	S	>8	R	4	R	>4	R	8	R	<=4
<=32	S	>8	R	2	I	>4	R	8	R	<=4
<=32	S	>8	R	2	I	>4	R	4	R	8
<=32	S	>8	R	<=1	S	>4	R	2	I	8
<=32	S	<=2	S	<=1	S	<=1	S	<=1	S	<=4
>64	R	>8	R	2	I	>4	R	<=1	S	>8
>64	R	>8	R	4	R	4	I	4	R	>8
>64	R	>8	R	2	I	>4	R	<=1	S	>8
>64	R	>8	R	<=1	S	>4	R	<=1	S	>8
64	R	>8	R	<=1	S	>4	R	<=1	S	8
64	R	>8	R	<=1	S	2	S	2	I	>8
<=32	S	>8	R	<=1	S	4	I	2	I	>8
<=32	S	>8	R	<=1	S	<=1	S	<=1	S	8
<=32	S	>8	R	>8	R	2	S	>8	R	<=4
<=32	S	>8	R	>8	R	2	S	>8	R	<=4
<=32	S	>8	R	8	R	>4	R	>8	R	>8
<=32	S	>8	R	8	R	>4	R	>8	R	>8
<=32	S	<=2	S	<=1	S	<=1	S	<=1	S	<=4
<=32	S	>8	R	<=1	S	>4	R	<=1	S	8
<=32	S	>8	R	>8	R	4	I	>8	R	>8
<=32	S	>8	R	<=1	S	>4	R	<=1	S	8
>64	R	<=2	S	<=1	S	<=1	S	<=1	S	<=4
<=32	S	<=2	S	<=1	S	2	S	2	I	<=4
>64	R	>8	R	>8	R	4	I	>8	R	>8
>64	R	>8	R	2	I	<=1	S	2	I	<=4

<=32	S	<=2	S	<=1	S	>4	R	<=1	S	>8
<=32	S	>8	R	<=1	S	>4	R	2	I	<=4
<=32	S	>8	R	8	R	<=1	S	>8	R	8
<=32	S	>8	R	<=1	S	>4	R	<=1	S	>8
<=32	S	>8	R	>8	R	2	S	>8	R	>8
<=32	S	>8	R	2	I	<=1	S	4	R	<=4
<=32	S	>8	R	<=1	S	2	S	<=1	S	8
<=32	S	>8	R	8	R	>4	R	>8	R	>8
<=32	S	>8	R	2	I	>4	R	2	I	<=4
64	R	>8	R	>8	R	>4	R	>8	R	>8
<=32	S	<=2	S	<=1	S	2	S	4	R	<=4
<=32	S	>8	R	8	R	>4	R	>8	R	<=4
<=32	S	>8	R	>8	R	>4	R	>8	R	>8
<=32	S	>8	R	2	I	<=1	S	8	R	>8
>64	R	<=2	S	8	R	>4	R	>8	R	<=4
<=32	S	>8	R	<=1	S	2	S	<=1	S	>8
<=32	S	>8	R	<=1	S	<=1	S	<=1	S	<=4
<=32	S	>8	R	>8	R	2	S	>8	R	<=4
<=32	S	>8	R	>8	R	2	S	8	R	<=4
>64	R	>8	R	<=1	S	<=1	S	<=1	S	8
<=32	S	>8	R	8	R	>4	R	>8	R	<=4
<=32	S	>8	R	<=1	S	>4	R	<=1	S	>8
<=32	S	>8	R	4	R	>4	R	>8	R	<=4
<=32	S	<=2	S	<=1	S	<=1	S	<=1	S	<=4
<=32	S	>8	R	<=1	S	<=1	S	2	I	<=4
<=32	S	<=2	S	<=1	S	>4	R	<=1	S	>8
>64	R	>8	R	>8	R	2	S	>8	R	8
<=32	S	>8	R	>8	R	>4	R	>8	R	<=4
<=32	S	>8	R	8	R	>4	R	>8	R	<=4
<=32	S	>8	R	>8	R	>4	R	>8	R	<=4
<=32	S	>8	R	>8	R	<=1	S	>8	R	<=4
<=32	S	>8	R	<=1	S	2	S	<=1	S	>8
<=32	S	<=2	S	<=1	S	<=1	S	<=1	S	<=4
<=32	S	>8	R	<=1	S	<=1	S	<=1	S	<=4
<=32	S	>8	R	>8	R	2	S	>8	R	<=4
64	R	>8	R	4	R	<=1	S	<=1	S	<=4
<=32	S	<=2	S	<=1	S	<=1	S	<=1	S	<=4
64	R	>8	R	2	I	<=1	S	8	R	>8
<=32	S	>8	R	<=1	S	>4	R	<=1	S	<=4
<=32	S	<=2	S	<=1	S	>4	R	<=1	S	>8
<=32	S	>8	R	<=1	S	<=1	S	<=1	S	>8
<=32	S	>8	R	<=1	S	<=1	S	<=1	S	>8
<=32	S	<=2	S	8	R	<=1	S	>8	R	<=4
<=32	S	>8	R	2	I	<=1	S	2	I	<=4
<=32	S	>8	R	>8	R	>4	R	>8	R	>8
>64	R	>8	R	<=1	S	<=1	S	<=1	S	<=4
<=32	S	>8	R	<=1	S	>4	R	<=1	S	<=4
<=32	S	>8	R	8	R	>4	R	>8	R	>8

<=32	S	>8	R	>8	R	>4	R	>8	R	>8
<=32	S	>8	R	>8	R	2	S	>8	R	<=4
<=32	S	>8	R	>8	R	2	S	>8	R	<=4
<=32	S	>8	R	>8	R	>4	R	>8	R	<=4
<=32	S	<=2	S	<=1	S	<=1	S	<=1	S	<=4
<=32	S	<=2	S	<=1	S	2	S	<=1	S	>8
<=32	S	>8	R	<=1	S	<=1	S	<=1	S	<=4
<=32	S	<=2	S	<=1	S	<=1	S	<=1	S	8
>64	R	>8	R	8	R	2	S	>8	R	>8
<=32	S	>8	R	>8	R	2	S	>8	R	<=4
<=32	S	>8	R	>8	R	4	I	>8	R	<=4
<=32	S	>8	R	>8	R	2	S	>8	R	<=4
<=32	S	>8	R	>8	R	2	S	>8	R	<=4
<=32	S	>8	R	>8	R	>4	R	>8	R	8
<=32	S	>8	R	<=1	S	>4	R	<=1	S	<=4
>64	R	>8	R	<=1	S	<=1	S	<=1	S	>8
<=32	S	>8	R	<=1	S	<=1	S	<=1	S	<=4
<=32	S	<=2	S	<=1	S	>4	R	<=1	S	>8
>64	R	>8	R	>8	R	<=1	S	>8	R	<=4
<=32	S	>8	R	8	R	2	S	>8	R	8
<=32	S	>8	R	<=1	S	>4	R	<=1	S	>8
64	R	>8	R	>8	R	>4	R	>8	R	>8
<=32	S	>8	R	<=1	S	<=1	S	<=1	S	>8
<=32	S	>8	R	>8	R	>4	R	>8	R	<=4
<=32	S	>8	R	<=1	S	>4	R	<=1	S	<=4
<=32	S	>8	R	<=1	S	>4	R	<=1	S	>8
<=32	S	<=2	S	2	I	>4	R	<=1	S	<=4
<=32	S	<=2	S	<=1	S	>4	R	<=1	S	<=4
<=32	S	>8	R	>8	R	>4	R	>8	R	<=4
<=32	S	>8	R	>8	R	>4	R	>8	R	<=4
<=32	S	>8	R	<=1	S	>4	R	<=1	S	<=4
<=32	S	>8	R	>8	R	>4	R	>8	R	<=4
<=32	S	>8	R	>8	R	>4	R	>8	R	<=4
<=32	S	>8	R	8	R	<=1	S	8	R	<=4
<=32	S	>8	R	8	R	<=1	S	>8	R	<=4
<=32	S	>8	R	<=1	S	>4	R	4	R	>8
<=32	S	>8	R	8	R	2	S	>8	R	>8
<=32	S	>8	R	>8	R	>4	R	>8	R	<=4
<=32	S	>8	R	<=1	S	<=1	S	<=1	S	>8
<=32	S	>8	R	<=1	S	<=1	S	<=1	S	>8
<=32	S	>8	R	8	R	>4	R	>8	R	<=4
<=32	S	<=2	S	<=1	S	2	S	<=1	S	<=4
<=32	S	<=2	S	<=1	S	<=1	S	<=1	S	<=4
<=32	S	>8	R	<=1	S	<=1	S	<=1	S	<=4
64	R	<=2	S	<=1	S	<=1	S	<=1	S	<=4
64	R	<=2	S	<=1	S	<=1	S	<=1	S	<=4

<=32	S	>8	R	8	R	<=1	S	>8	R	<=4
<=32	S	>8	R	2	I	<=1	S	8	R	>8
<=32	S	>8	R	<=1	S	<=1	S	<=1	S	<=4
<=32	S	>8	R	8	R	>4	R	>8	R	<=4
<=32	S	>8	R	<=1	S	>4	R	<=1	S	8
<=32	S	>8	R	8	R	>4	R	>8	R	<=4
64	R	>8	R	>8	R	4	I	>8	R	>8
<=32	S	>8	R	8	R	>4	R	>8	R	<=4
<=32	S	>8	R	<=1	S	<=1	S	<=1	S	<=4
<=32	S	>8	R	<=1	S	2	S	<=1	S	<=4
>64	R	<=2	S	>8	R	<=1	S	>8	R	<=4
<=32	S	>8	R	4	R	<=1	S	2	I	<=4
<=32	S	>8	R	8	R	<=1	S	>8	R	<=4
<=32	S	>8	R	<=1	S	<=1	S	<=1	S	>8
<=32	S	>8	R	<=1	S	<=1	S	<=1	S	>8
>64	R	>8	R	>8	R	>4	R	>8	R	>8
<=32	S	<=2	S	2	I	>4	R	<=1	S	>8
<=32	S	<=2	S	<=1	S	>4	R	<=1	S	>8
<=32	S	>8	R	>8	R	<=1	S	<=1	S	>8
64	R	>8	R	4	R	4	I	8	R	>8
<=32	S	>8	R	<=1	S	2	S	<=1	S	>8
<=32	S	<=2	S	<=1	S	2	S	<=1	S	<=4
<=32	S	>8	R	<=1	S	<=1	S	<=1	S	<=4
<=32	S	>8	R	<=1	S	<=1	S	<=1	S	<=4
64	R	>8	R	<=1	S	<=1	S	<=1	S	>8
64	R	>8	R	4	R	<=1	S	>8	R	>8
<=32	S	>8	R	<=1	S	<=1	S	<=1	S	>8
<=32	S	>8	R	>8	R	>4	R	>8	R	<=4
<=32	S	>8	R	>8	R	>4	R	>8	R	>8
<=32	S	>8	R	8	R	<=1	S	8	R	<=4
<=32	S	<=2	S	<=1	S	>4	R	<=1	S	>8
<=32	S	>8	R	8	R	<=1	S	>8	R	8
<=32	S	>8	R	>8	R	>4	R	>8	R	>8
>64	R	<=2	S	<=1	S	<=1	S	<=1	S	<=4
<=32	S	>8	R	8	R	>4	R	>8	R	<=4
<=32	S	>8	R	<=1	S	>4	R	<=1	S	>8
<=32	S	>8	R	<=1	S	>4	R	<=1	S	<=4
64	R	<=2	S	<=1	S	<=1	S	<=1	S	<=4
<=32	S	>8	R	4	R	<=1	S	>8	R	>8
<=32	S	>8	R	8	R	>4	R	>8	R	>8
<=32	S	>8	R	>8	R	>4	R	>8	R	>8
64	R	>8	R	<=1	S	>4	R	<=1	S	<=4
64	R	<=2	S	<=1	S	4	I	<=1	S	<=4
64	R	<=2	S	>8	R	<=1	S	>8	R	<=4

<=32	S	>8	R	<=1	S	<=1	S	<=1	S	<=4
<=32	S	>8	R	>8	R	>4	R	>8	R	>8
<=32	S	<=2	S	<=1	S	<=1	S	<=1	S	<=4
64	R	>8	R	8	R	>4	R	>8	R	>8
>64	R	>8	R	8	R	>4	R	>8	R	>8
<=32	S	>8	R	<=1	S	>4	R	4	R	>8
<=32	S	>8	R	<=1	S	<=1	S	<=1	S	>8
64	R	<=2	S	<=1	S	<=1	S	<=1	S	>8
>64	R	>8	R	>8	R	<=1	S	>8	R	>8
64	R	>8	R	<=1	S	<=1	S	<=1	S	<=4
>64	R	>8	R	<=1	S	>4	R	4	R	>8
64	R	>8	R	>8	R	>4	R	>8	R	>8
<=32	S	>8	R	<=1	S	>4	R	<=1	S	8
<=32	S	>8	R	<=1	S	>4	R	<=1	S	8
<=32	S	<=2	S	<=1	S	<=1	S	<=1	S	<=4
<=32	S	>8	R	>8	R	>4	R	>8	R	>8
<=32	S	>8	R	<=1	S	<=1	S	<=1	S	>8
<=32	S	>8	R	8	R	>4	R	>8	R	<=4
<=32	S	>8	R	4	R	2	S	<=1	S	<=4
<=32	S	>8	R	<=1	S	<=1	S	<=1	S	<=4
<=32	S	>8	R	>8	R	>4	R	>8	R	>8

<=32	S	>8	R	2	I	<=1	S	<=1	S	<=4
64	R	>8	R	>8	R	>4	R	>8	R	>8
64	R	>8	R	>8	R	>4	R	>8	R	>8
<=32	S	>8	R	>8	R	2	S	>8	R	8
<=32	S	>8	R	>8	R	>4	R	>8	R	>8
<=32	S	>8	R	>8	R	>4	R	>8	R	<=4
<=32	S	<=2	S	>8	R	<=1	S	>8	R	>8
64	R	>8	R	<=1	S	4	I	<=1	S	>8

<=32	S	<=2	S	<=1	S	<=1	S	<=1	S	<=4
<=32	S	>8	R	4	R	<=1	S	<=1	S	<=4
<=32	S	>8	R	<=1	S	<=1	S	<=1	S	<=4
<=32	S	<=2	S	<=1	S	>4	R	<=1	S	>8
<=32	S	>8	R	<=1	S	<=1	S	<=1	S	8
<=32	S	>8	R	<=1	S	<=1	S	<=1	S	<=4
<=32	S	>8	R	>8	R	4	I	>8	R	8
<=32	S	>8	R	<=1	S	>4	R	<=1	S	8
64	R	>8	R	<=1	S	<=1	S	<=1	S	>8
<=32	S	>8	R	<=1	S	<=1	S	<=1	S	>8
<=32	S	>8	R	<=1	S	>4	R	<=1	S	>8
<=32	S	>8	R	<=1	S	<=1	S	<=1	S	<=4
<=32	S	>8	R	>8	R	>4	R	>8	R	>8
<=32	S	>8	R	4	R	<=1	S	>8	R	>8
<=32	S	<=2	S	<=1	S	<=1	S	<=1	S	<=4
>64	R	>8	R	<=1	S	<=1	S	<=1	S	<=4
<=32	S	>8	R	<=1	S	>4	R	<=1	S	>8
<=32	S	>8	R	8	R	<=1	S	8	R	8

<=32	S	<=2	S	2	I	<=1	S	<=1	S	>8
<=32	S	<=2	S	<=1	S	<=1	S	<=1	S	<=4
<=32	S	>8	R	<=1	S	<=1	S	<=1	S	<=4
<=32	S	>8	R	>8	R	>4	R	>8	R	>8
<=32	S	>8	R	>8	R	>4	R	>8	R	>8
<=32	S	>8	R	>8	R	>4	R	>8	R	>8
<=32	S	>8	R	<=1	S	<=1	S	<=1	S	<=4
64	R	>8	R	>8	R	>4	R	>8	R	>8
<=32	S	>8	R	>8	R	>4	R	>8	R	>8
<=32	S	>8	R	>8	R	>4	R	>8	R	>8
<=32	S	<=2	S	<=1	S	<=1	S	<=1	S	<=4
<=32	S	>8	R	>8	R	>4	R	>8	R	>8

<=32	S	>8	R	<=1	S	>4	R	<=1	S	>8

<=32	S	>8	R	<=1	S	>4	R	4	R	8
<=32	S	>8	R	<=1	S	>4	R	<=1	S	>8
<=32	S	>8	R	<=1	S	>4	R	<=1	S	<=4
<=32	S	>8	R	<=1	S	>4	R	<=1	S	>8
<=32	R	>8	R	>8	R	>4	R	>8	R	<=4
<=32	S	>8	R	<=1	S	>4	R	<=1	S	<=4
<=32	S	>8	R	<=1	S	>4	R	<=1	S	>8
<=32	S	>8	R	<=1	S	>4	R	<=1	S	8
<=32	S	>8	R	8	R	<=1	S	8	R	>8
<=32	S	>8	R	<=1	S	>4	R	<=1	S	>8
<=32	S	>8	R	<=1	S	<=1	S	<=1	S	<=4

<=32	S	>8	R	<=1	S	>4	R	<=1	S	<=4
<=32	S	>8	R	<=1	S	>4	R	<=1	S	>8
<=32	S	>8	R	2	I	>4	R	<=1	S	<=4
<=32	S	>8	R	>8	R	>4	R	>8	R	>8
64	R	<=2	S	2	I	4	I	2	I	<=4

64	R	>8	R	>8	R	>4	R	>8	R	>8
64	R	>8	R	>8	R	>4	R	>8	R	>8
64	R	>8	R	>8	R	>4	R	>8	R	>8
64	R	>8	R	<=1	S	<=1	S	<=1	S	<=4
<=32	S	>8	R	4	R	2	S	<=1	S	<=4
<=32	S	>8	R	<=1	S	<=1	S	<=1	S	>8
<=32	S	>8	R	<=1	S	>4	R	<=1	S	8
>64	R	>8	R	<=1	S	<=1	S	<=1	S	8
64	R	>8	R	>8	R	>4	R	>8	R	>8
<=32	S	>8	R	>8	R	<=1	S	>8	R	<=4
<=32	S	<=2	S	2	I	>4	R	<=1	S	8
<=32	S	>8	R	4	R	>4	R	4	R	>8
64	R	<=2	S	2	I	<=1	S	<=1	S	<=4
<=32	S	>8	R	>8	R	<=1	S	>8	R	<=4
<=32	S	>8	R	>8	R	>4	R	>8	R	<=4
>64	R	>8	R	4	R	<=1	S	4	R	<=4
<=32	S	<=2	S	<=1	S	<=1	S	<=1	S	>8
<=32	S	>8	R	<=1	S	<=1	S	2	I	<=4
<=32	S	>8	R	>8	R	<=1	S	>8	R	<=4
<=32	S	>8	R	<=1	S	<=1	S	<=1	S	<=4
<=32	S	>8	R	>8	R	>4	R	>8	R	>8
<=32	S	>8	R	<=1	S	<=1	S	<=1	S	<=4
>64	R	<=2	S	<=1	S	4	I	2	I	<=4
<=32	S	>8	R	<=1	S	<=1	S	<=1	S	<=4
>64	R	>8	R	4	R	<=1	S	8	R	<=4
<=32	S	>8	R	>8	R	<=1	S	>8	R	<=4
<=32	S	<=2	S	>8	R	<=1	S	>8	R	<=4
<=32	S	>8	R	<=1	S	<=1	S	<=1	S	>8
64	R	>8	R	8	R	<=1	S	>8	R	<=4

<=32	S	>8	R	<=1	S	<=1	S	<=1	S	<=4
64	R	>8	R	<=1	S	<=1	S	<=1	S	<=4
<=32	S	>8	R	<=1	S	<=1	S	<=1	S	<=4

<=32	S	>8	R	>8	R	<=1	S	>8	R	<=4
<=32	S	>8	R	<=1	S	<=1	S	<=1	S	<=4
<=32	S	>8	R	8	R	<=1	S	2	I	<=4

<=32	S	>8	R	2	I	<=1	S	<=1	S	<=4
>64	R	<=2	S	<=1	S	<=1	S	<=1	S	<=4
<=32	S	>8	R	>8	R	<=1	S	>8	R	<=4
<=32	S	>8	R	8	R	>4	R	>8	R	>8
<=32	S	>8	R	<=1	S	<=1	S	<=1	S	<=4
>64	R	>8	R	8	R	<=1	S	>8	R	<=4
<=32	S	>8	R	8	R	<=1	S	8	R	8
<=32	S	>8	R	<=1	S	<=1	S	<=1	S	<=4

<=32	S	>8	R	<=1	S	<=1	S	<=1	S	<=4
>64	R	>8	R	<=1	S	<=1	S	<=1	S	<=4
<=32	S	>8	R	8	R	<=1	S	>8	R	<=4
<=32	S	>8	R	<=1	S	>4	R	<=1	S	<=4

64	R	>8	R	<=1	S	<=1	S	<=1	S	<=4
<=32	S	>8	R	4	R	<=1	S	<=1	S	<=4
<=32	S	<=2	S	2	I	<=1	S	2	I	<=4
<=32	S	>8	R	<=1	S	<=1	S	<=1	S	<=4
<=32	S	>8	R	<=1	S	<=1	S	<=1	S	<=4
>64	R	>8	R	<=1	S	<=1	S	<=1	S	<=4
<=32	S	<=2	S	<=1	S	4	I	4	R	<=4
<=32	S	>8	R	4	R	<=1	S	4	R	<=4
<=32	S	>8	R	2	I	>4	R	4	R	<=4
<=32	S	<=2	S	<=1	S	<=1	S	<=1	S	<=4
<=32	S	>8	R	<=1	S	<=1	S	<=1	S	>8
>64	R	>8	R	<=1	S	<=1	S	<=1	S	<=4
<=32	S	4	S	2	I	>4	R	<=1	S	8
>64	R	>8	R	8	R	>4	R	>8	R	>8

<=32	S	>8	R	>8	R	>4	R	>8	R	>8
<=32	S	>8	R	<=1	S	<=1	S	<=1	S	<=4
64	R	>8	R	>8	R	>4	R	>8	R	>8
>64	R	>8	R	<=1	S	>4	R	<=1	S	<=4
>64	R	>8	R	>8	R	>4	R	>8	R	>8
<=32	S	>8	R	<=1	S	<=1	S	<=1	S	<=4
<=32	S	>8	R	>8	R	<=1	S	>8	R	<=4
<=32	S	>8	R	2	I	<=1	S	8	R	<=4

<=32	S	>8	R	<=1	S	<=1	S	<=1	S	>8
<=32	S	>8	R	<=1	S	<=1	S	<=1	S	>8
<=32	S	>8	R	8	R	>4	R	>8	R	<=4
<=32	S	>8	R	<=1	S	<=1	S	<=1	S	>8
<=32	S	>8	R	<=1	S	<=1	S	<=1	S	<=4
<=32	S	>8	R	>8	R	2	S	>8	R	8
<=32	S	>8	R	<=1	S	<=1	S	<=1	S	<=4
<=32	S	>8	R	<=1	S	<=1	S	<=1	S	<=4
<=32	S	<=2	S	<=1	S	>4	R	<=1	S	>8
<=32	S	>8	R	>8	R	2	S	>8	R	8
<=32	S	<=2	S	<=1	S	<=1	S	<=1	S	<=4
<=32	S	>8	R	>8	R	>4	R	>8	R	<=4
<=32	S	>8	R	>8	R	>4	R	>8	R	<=4
<=32	S	>8	R	<=1	S	2	S	<=1	S	<=4
<=32	S	>8	R	>8	R	>4	R	>8	R	>8
64	R	>8	R	>8	R	>4	R	>8	R	>8
<=32	S	<=2	S	<=1	S	<=1	S	<=1	S	>8
64	R	>8	R	8	R	>4	R	>8	R	>8
<=32	S	>8	R	<=1	S	2	S	<=1	S	<=4
<=32	S	>8	R	>8	R	>4	R	>8	R	>8
64	R	>8	R	<=1	S	<=1	S	<=1	S	>8
<=32	S	>8	R	8	R	>4	R	>8	R	>8
<=32	S	>8	R	>8	R	>4	R	>8	R	<=4
<=32	S	>8	R	8	R	>4	R	>8	R	<=4

<=32	S	<=2	S	<=1	S	<=1	S	<=1	S	<=4
<=32	S	>8	R	<=1	S	<=1	S	<=1	S	<=4
<=32	S	>8	R	>8	R	>4	R	>8	R	8
<=32	S	>8	R	<=1	S	>4	R	<=1	S	<=4
<=32	S	>8	R	<=1	S	2	S	<=1	S	8
<=32	S	>8	R	<=1	S	<=1	S	<=1	S	<=4

<=32	S	<=2	S	<=1	S	2	S	<=1	S	<=4
64	R	>8	R	8	R	<=1	S	8	R	<=4
<=32	S	>8	R	2	I	>4	R	>8	R	>8
<=32	S	>8	R	4	R	2	S	>8	R	>8

64	R	8	I	>8	R	>4	R	8	R	>8
<=32	S	>8	R	<=1	S	>4	R	<=1	S	8
<=32	S	>8	R	4	R	4	I	>8	R	>8

<=32	S	>8	R	8	R	4	I	>8	R	>8
<=32	S	>8	R	>8	R	>4	R	>8	R	>8
<=32	S	>8	R	<=1	S	<=1	S	<=1	S	<=4
64	R	>8	R	<=1	S	<=1	S	<=1	S	<=4
>64	R	<=2	S	<=1	S	2	S	<=1	S	<=4
<=32	S	>8	R	<=1	S	<=1	S	<=1	S	<=4
64	R	>8	R	4	R	<=1	S	>8	R	<=4
<=32	S	>8	R	<=1	S	<=1	S	<=1	S	<=4
64	R	>8	R	2	I	2	S	8	R	<=4
>64	R	>8	R	>8	R	4	I	>8	R	8
<=32	S	>8	R	8	R	>4	R	>8	R	>8

<=32	S	>8	R	8	R	>4	R	>8	R	>8
<=32	S	>8	R	<=1	S	<=1	S	<=1	S	<=4
<=32	S	>8	R	<=1	S	>4	R	<=1	S	<=4
<=32	S	>8	R	8	R	>4	R	>8	R	>8
<=32	S	>8	R	8	R	>4	R	>8	R	<=4
>64	R	>8	R	<=1	S	<=1	S	2	I	<=4
<=32	S	>8	R	8	R	>4	R	>8	R	>8
<=32	S	>8	R	>8	R	>4	R	>8	R	>8
64	R	>8	R	8	R	<=1	S	>8	R	<=4
64	R	>8	R	<=1	S	>4	R	<=1	S	<=4
>64	R	>8	R	4	R	<=1	S	4	R	8
<=32	S	<=2	S	>8	R	<=1	S	>8	R	<=4
<=32	S	>8	R	>8	R	>4	R	>8	R	8
<=32	S	>8	R	<=1	S	>4	R	<=1	S	>8

<=32	S	>8	R	<=1	S	<=1	S	<=1	S	<=4
<=32	S	>8	R	<=1	S	<=1	S	<=1	S	<=4
>64	R	>8	R	<=1	S	<=1	S	<=1	S	<=4
<=32	S	>8	R	<=1	S	<=1	S	<=1	S	<=4

>64	R	<=2	S	2	I	<=1	S	4	R	<=4

64	R	>8	R	4	R	<=1	S	>8	R	<=4
<=32	S	>8	R	8	R	>4	R	>8	R	>8
<=32	S	>8	R	8	R	>4	R	>8	R	<=4
<=32	S	<=2	S	<=1	S	<=1	S	<=1	S	<=4
<=32	S	<=2	S	2	I	<=1	S	<=1	S	>8

64	R	<=2	S	<=1	S	>4	R	<=1	S	<=4
<=32	S	>8	R	<=1	S	>4	R	<=1	S	8

>64	R	>8	R	<=1	S	<=1	S	<=1	S	<=4
<=32	S	>8	R	<=1	S	<=1	S	<=1	S	<=4
<=32	S	>8	R	8	R	>4	R	>8	R	<=4
64	R	>8	R	<=1	S	<=1	S	<=1	S	<=4
<=32	S	>8	R	<=1	S	>4	R	<=1	S	8
<=32	S	>8	R	2	I	<=1	S	4	R	<=4
<=32	S	>8	R	>8	R	<=1	S	8	R	<=4
<=32	S	>8	R	>8	R	>4	R	>8	R	>8

<=32	S	>8	R	<=1	S	<=1	S	<=1	S	<=4

<=32	S	>8	R	>8	R	>4	R	>8	R	<=4
<=32	S	>8	R	<=1	S	<=1	S	<=1	S	<=4
<=32	S	>8	R	<=1	S	<=1	S	<=1	S	<=4
<=32	S	>8	R	8	R	<=1	S	2	I	<=4
<=32	S	>8	R	4	R	>4	R	8	R	>8
>64	R	>8	R	<=1	S	>4	R	<=1	S	>8
64	R	>8	R	<=1	S	>4	R	<=1	S	>8

<=32	S	<=2	S	<=1	S	<=1	S	<=1	S	<=4
<=32	S	>8	R	<=1	S	>4	R	<=1	S	8

<=32	S	>8	R	>8	R	>4	R	>8	R	<=4
64	R	>8	R	8	R	>4	R	>8	R	>8
<=32	S	>8	R	>8	R	>4	R	>8	R	<=4
<=32	S	>8	R	<=1	S	<=1	S	<=1	S	<=4
64	R	>8	R	8	R	<=1	S	>8	R	<=4
<=32	S	<=2	S	<=1	S	>4	R	<=1	S	>8
>64	R	<=2	S	<=1	S	<=1	S	<=1	S	<=4
<=32	S	<=2	S	<=1	S	<=1	S	<=1	S	8
>64	R	>8	R	>8	R	4	I	>8	R	>8
<=32	S	>8	R	>8	R	>4	R	>8	R	<=4
<=32	S	>8	R	>8	R	>4	R	>8	R	<=4
<=32	S	>8	R	>8	R	>4	R	>8	R	>8
<=32	S	>8	R	>8	R	>4	R	>8	R	<=4
<=32	S	>8	R	<=1	S	>4	R	<=1	S	>8
<=32	S	>8	R	<=1	S	>4	R	<=1	S	8
<=32	S	>8	R	<=1	S	>4	R	<=1	S	>8
<=32	S	>8	R	>8	R	4	I	>8	R	8
<=32	S	>8	R	>8	R	>4	R	>8	R	<=4
<=32	S	>8	R	2	I	<=1	S	4	R	<=4
<=32	S	<=2	S	<=1	S	>4	R	<=1	S	8
<=32	S	>8	R	>8	R	>4	R	>8	R	<=4
<=32	S	>8	R	<=1	S	<=1	S	<=1	S	<=4
<=32	S	>8	R	<=1	S	<=1	S	<=1	S	<=4
<=32	S	>8	R	>8	R	>4	R	>8	R	>8
<=32	S	>8	R	>8	R	>4	R	>8	R	<=4
<=32	S	>8	R	8	R	>4	R	>8	R	<=4
<=32	S	>8	R	8	R	>4	R	>8	R	<=4
<=32	S	>8	R	4	R	>4	R	2	I	>8
<=32	S	>8	R	<=1	S	<=1	S	<=1	S	<=4
<=32	S	>8	R	<=1	S	>4	R	<=1	S	<=4
<=32	S	<=2	S	<=1	S	4	I	<=1	S	>8
<=32	S	<=2	S	<=1	S	<=1	S	<=1	S	<=4
<=32	S	>8	R	>8	R	4	I	>8	R	>8
<=32	S	>8	R	<=1	S	>4	R	<=1	S	8
<=32	S	>8	R	>8	R	>4	R	>8	R	<=4
>64	R	<=2	S	>8	R	>4	R	<=1	S	8
<=32	S	<=2	S	<=1	S	<=1	S	4	R	<=4
<=32	S	>8	R	<=1	S	>4	R	<=1	S	>8
<=32	S	>8	R	<=1	S	4	I	<=1	S	<=4
<=32	S	<=2	S	<=1	S	4	I	<=1	S	>8
<=32	S	>8	R	<=1	S	>4	R	<=1	S	8
<=32	S	>8	R	>8	R	>4	R	>8	R	<=4
<=32	S	>8	R	<=1	S	2	S	<=1	S	<=4
<=32	S	>8	R	8	R	<=1	S	8	R	<=4
<=32	S	<=2	S	<=1	S	>4	R	<=1	S	<=4
<=32	S	>8	R	>8	R	>4	R	>8	R	>8
<=32	S	>8	R	<=1	S	>4	R	<=1	S	>8
<=32	S	>8	R	>8	R	>4	R	>8	R	<=4
<=32	S	>8	R	>8	R	>4	R	>8	R	<=4
<=32	S	>8	R	>8	R	>4	R	>8	R	<=4
<=32	S	>8	R	8	R	>4	R	>8	R	<=4
64	R	<=2	S	<=1	S	<=1	S	<=1	S	<=4
<=32	S	<=2	S	<=1	S	<=1	S	<=1	S	<=4
<=32	S	>8	R	>8	R	>4	R	>8	R	<=4
<=32	S	>8	R	<=1	S	<=1	S	<=1	S	<=4
<=32	S	>8	R	8	R	>4	R	>8	R	<=4

<=32	S	>8	R	>8	R	>4	R	>8	R	<=4
<=32	S	>8	R	<=1	S	>4	R	<=1	S	8
<=32	S	>8	R	<=1	S	>4	R	<=1	S	8
>64	R	>8	R	<=1	S	<=1	S	<=1	S	<=4
<=32	S	>8	R	2	I	<=1	S	4	R	<=4
<=32	S	>8	R	>8	R	>4	R	>8	R	>8
<=32	S	>8	R	<=1	S	<=1	S	<=1	S	<=4
<=32	S	>8	R	<=1	S	>4	R	<=1	S	8
>64	R	>8	R	8	R	<=1	S	>8	R	<=4
>64	R	<=2	S	>8	R	<=1	S	>8	R	<=4
<=32	S	<=2	S	<=1	S	<=1	S	<=1	S	<=4
64	R	>8	R	>8	R	<=1	S	<=1	S	8
<=32	S	>8	R	>8	R	4	I	>8	R	8
<=32	S	>8	R	>8	R	<=1	S	>8	R	<=4

<=32	S	>8	R	<=1	S	2	S	<=1	S	8
64	R	>8	R	<=1	S	<=1	S	<=1	S	<=4
<=32	S	<=2	S	4	R	<=1	S	<=1	S	<=4
<=32	S	>8	R	<=1	S	<=1	S	<=1	S	<=4
<=32	S	>8	R	<=1	S	>4	R	<=1	S	<=4
<=32	S	>8	R	<=1	S	4	I	<=1	S	8
<=32	S	<=2	S	2	I	<=1	S	<=1	S	<=4
<=32	S	>8	R	>8	R	>4	R	>8	R	<=4
<=32	S	>8	R	>8	R	>4	R	>8	R	<=4
<=32	S	>8	R	2	I	<=1	S	<=1	S	<=4
64	R	>8	R	<=1	S	>4	R	<=1	S	8
<=32	S	<=2	S	2	I	<=1	S	<=1	S	<=4
<=32	S	<=2	S	2	I	<=1	S	<=1	S	<=4
64	R	>8	R	>8	R	<=1	S	>8	R	<=4
<=32	S	<=2	S	<=1	S	4	I	<=1	S	>8
<=32	S	>8	R	>8	R	<=1	S	>8	R	<=4
<=32	S	>8	R	<=1	S	4	I	<=1	S	<=4
64	R	>8	R	<=1	S	2	S	<=1	S	<=4
<=32	S	>8	R	8	R	>4	R	>8	R	<=4
<=32	S	<=2	S	<=1	S	<=1	S	<=1	S	<=4
<=32	S	>8	R	>8	R	>4	R	>8	R	<=4
<=32	S	>8	R	<=1	S	<=1	S	<=1	S	<=4
<=32	S	<=2	S	<=1	S	<=1	S	<=1	S	<=4
<=32	S	8	I	>8	R	2	S	>8	R	<=4
<=32	S	<=2	S	<=1	S	>4	R	<=1	S	8
<=32	S	>8	R	<=1	S	>4	R	<=1	S	<=4
<=32	S	>8	R	<=1	S	>4	R	<=1	S	>8
<=32	S	<=2	S	<=1	S	>4	R	<=1	S	<=4
64	R	>8	R	4	R	<=1	S	8	R	<=4
<=32	S	<=2	S	4	R	2	S	8	R	>8
<=32	S	<=2	S	4	R	<=1	S	<=1	S	<=4
<=32	S	4	S	4	R	<=1	S	<=1	S	<=4

>64	R	<=2	S	8	R	<=1	S	>8	R	<=4
<=32	S	>8	R	2	I	2	S	8	R	<=4
>64	R	>8	R	>8	R	>4	R	>8	R	>8
<=32	S	>8	R	8	R	>4	R	>8	R	>8
<=32	S	>8	R	<=1	S	<=1	S	<=1	S	8
<=32	S	8	I	8	R	4	I	>8	R	>8
<=32	S	<=2	S	4	R	<=1	S	4	R	<=4
>64	R	>8	R	<=1	S	<=1	S	<=1	S	<=4
<=32	S	>8	R	<=1	S	<=1	S	<=1	S	<=4
<=32	S	>8	R	<=1	S	2	S	<=1	S	<=4
<=32	S	>8	R	<=1	S	<=1	S	<=1	S	>8
<=32	S	<=2	S	4	R	<=1	S	4	R	<=4
<=32	S	>8	R	<=1	S	<=1	S	<=1	S	<=4
<=32	S	>8	R	<=1	S	<=1	S	<=1	S	<=4
<=32	S	>8	R	<=1	S	<=1	S	<=1	S	<=4
<=32	S	>8	R	<=1	S	<=1	S	<=1	S	<=4
64	R	>8	R	<=1	S	2	S	2	I	<=4
64	R	<=2	S	<=1	S	<=1	S	<=1	S	<=4
64	R	>8	R	>8	R	<=1	S	>8	R	<=4
64	R	>8	R	>8	R	>4	R	>8	R	>8
<=32	S	<=2	S	<=1	S	4	I	<=1	S	8
<=32	S	>8	R	<=1	S	>4	R	<=1	S	8
<=32	S	>8	R	2	I	>4	R	>8	R	>8
>64	R	>8	R	<=1	S	<=1	S	<=1	S	8
<=32	S	<=2	S	4	R	<=1	S	<=1	S	<=4
<=32	S	<=2	S	4	R	<=1	S	<=1	S	<=4
<=32	S	>8	R	<=1	S	<=1	S	<=1	S	<=4
<=32	S	>8	R	8	R	4	I	>8	R	<=4
<=32	S	<=2	S	8	R	<=1	S	<=1	S	<=4
<=32	S	>8	R	<=1	S	<=1	S	<=1	S	>8
<=32	S	>8	R	<=1	S	<=1	S	<=1	S	<=4
<=32	S	>8	R	<=1	S	<=1	S	<=1	S	>8
<=32	S	>8	R	<=1	S	<=1	S	<=1	S	<=4
<=32	S	>8	R	<=1	S	2	S	<=1	S	<=4
<=32	S	>8	R	>8	R	>4	R	>8	R	>8
>64	R	>8	R	<=1	S	>4	R	<=1	S	8
>64	R	>8	R	8	R	>4	R	>8	R	>8
<=32	S	>8	R	<=1	S	2	S	<=1	S	<=4
<=32	S	>8	R	>8	R	>4	R	>8	R	>8
<=32	S	>8	R	<=1	S	>4	R	<=1	S	>8
<=32	S	>8	R	4	R	>4	R	8	R	<=4
<=32	S	>8	R	>8	R	>4	R	>8	R	<=4
<=32	S	>8	R	>8	R	>4	R	>8	R	>8
<=32	S	>8	R	>8	R	2	S	>8	R	<=4
<=32	S	>8	R	8	R	>4	R	>8	R	>8
<=32	S	>8	R	8	R	2	S	>8	R	<=4
<=32	S	>8	R	<=1	S	2	S	<=1	S	<=4
<=32	S	<=2	S	<=1	S	<=1	S	<=1	S	<=4
<=32	S	>8	R	8	R	<=1	S	>8	R	<=4

<=32	S	>8	R	2	I	<=1	S	8	R	8
<=32	S	<=2	S	<=1	S	2	S	<=1	S	<=4
<=32	S	<=2	S	<=1	S	<=1	S	<=1	S	<=4
64	R	>8	R	8	R	<=1	S	>8	R	<=4
<=32	S	<=2	S	2	I	<=1	S	<=1	S	<=4
<=32	S	>8	R	<=1	S	2	S	4	R	<=4
<=32	S	>8	R	8	R	>4	R	8	R	<=4
<=32	S	>8	R	4	R	>4	R	>8	R	>8
<=32	S	>8	R	2	I	>4	R	4	R	>8
<=32	S	>8	R	>8	R	>4	R	>8	R	>8
64	R	<=2	S	>8	R	<=1	S	>8	R	<=4
<=32	S	<=2	S	4	R	>4	R	2	I	>8
<=32	S	<=2	S	<=1	S	4	I	<=1	S	>8
<=32	S	>8	R	>8	R	>4	R	>8	R	<=4
<=32	S	<=2	S	<=1	S	>4	R	<=1	S	8
<=32	S	>8	R	8	R	>4	R	>8	R	>8
<=32	S	>8	R	>8	R	<=1	S	>8	R	8
64	R	<=2	S	2	I	2	S	<=1	S	<=4
64	R	>8	R	<=1	S	<=1	S	<=1	S	>8
<=32	S	>8	R	8	R	4	I	>8	R	<=4
<=32	S	>8	R	<=1	S	>4	R	<=1	S	>8
<=32	S	<=2	S	<=1	S	<=1	S	<=1	S	<=4
<=32	S	>8	R	<=1	S	<=1	S	<=1	S	<=4
<=32	S	<=2	S	<=1	S	<=1	S	<=1	S	<=4
<=32	S	<=2	S	>8	R	<=1	S	>8	R	<=4
<=32	S	>8	R	>8	R	>4	R	>8	R	>8
64	R	<=2	S	<=1	S	<=1	S	<=1	S	<=4

<=32	S	<=2	S	<=1	S	<=1	S	<=1	S	>8
<=32	S	<=2	S	8	R	<=1	S	<=1	S	<=4
<=32	S	>8	R	<=1	S	>4	R	<=1	S	>8
<=32	S	>8	R	<=1	S	<=1	S	<=1	S	<=4
<=32	S	>8	R	<=1	S	<=1	S	<=1	S	<=4
<=32	S	>8	R	4	R	>4	R	8	R	<=4
<=32	S	>8	R	<=1	S	>4	R	<=1	S	<=4
<=32	S	>8	R	2	I	<=1	S	<=1	S	<=4
<=32	S	>8	R	<=1	S	<=1	S	<=1	S	<=4
<=32	S	>8	R	<=1	S	2	S	<=1	S	>8
<=32	S	>8	R	<=1	S	>4	R	<=1	S	<=4
<=32	S	<=2	S	<=1	S	<=1	S	<=1	S	<=4
<=32	S	>8	R	>8	R	>4	R	>8	R	>8
64	R	>8	R	<=1	S	>4	R	2	I	>8

>64	R	>8	R	>8	R	<=1	S	>8	R	<=4
<=32	S	<=2	S	4	R	<=1	S	<=1	S	<=4
<=32	S	>8	R	<=1	S	>4	R	<=1	S	>8
64	R	>8	R	>8	R	4	I	>8	R	>8
64	R	>8	R	<=1	S	<=1	S	<=1	S	<=4
<=32	S	>8	R	>8	R	>4	R	>8	R	<=4
<=32	S	>8	R	>8	R	2	S	>8	R	8

<=32	S	>8	R	<=1	S	4	I	<=1	S	8
<=32	S	<=2	S	<=1	S	4	I	<=1	S	8
<=32	S	>8	R	<=1	S	4	I	<=1	S	8
<=32	S	>8	R	<=1	S	2	S	<=1	S	<=4
<=32	S	>8	R	<=1	S	2	S	<=1	S	8
>64	R	<=2	S	2	I	<=1	S	<=1	S	<=4
<=32	S	>8	R	<=1	S	2	S	<=1	S	<=4
<=32	S	>8	R	<=1	S	2	S	<=1	S	8
<=32	S	>8	R	<=1	S	<=1	S	<=1	S	<=4
<=32	S	>8	R	<=1	S	2	S	<=1	S	<=4
<=32	S	>8	R	<=1	S	<=1	S	<=1	S	<=4
<=32	S	>8	R	<=1	S	<=1	S	<=1	S	<=4
<=32	S	<=2	S	<=1	S	>4	R	<=1	S	<=4
<=32	S	>8	R	<=1	S	<=1	S	<=1	S	<=4
<=32	S	>8	R	2	I	<=1	S	2	I	<=4
<=32	S	>8	R	<=1	S	2	S	<=1	S	<=4
<=32	S	>8	R	>8	R	>4	R	>8	R	>8
<=32	S	<=2	S	<=1	S	2	S	<=1	S	>8
<=32	S	>8	R	<=1	S	2	S	<=1	S	<=4
64	R	>8	R	<=1	S	<=1	S	<=1	S	<=4

<=32	S	>8	R	<=1	S	<=1	S	<=1	S	<=4
<=32	S	>8	R	<=1	S	2	S	<=1	S	<=4
<=32	S	<=2	S	<=1	S	<=1	S	<=1	S	8
<=32	S	>8	R	<=1	S	<=1	S	<=1	S	<=4
>64	R	<=2	S	>8	R	4	I	>8	R	8
>64	R	>8	R	>8	R	<=1	S	>8	R	<=4
<=32	S	<=2	S	<=1	S	>4	R	8	R	8
<=32	S	>8	R	<=1	S	2	S	<=1	S	8
<=32	S	<=2	S	<=1	S	<=1	S	<=1	S	<=4
<=32	S	>8	R	<=1	S	2	S	<=1	S	8
<=32	S	<=2	S	<=1	S	<=1	S	<=1	S	<=4
>64	R	<=2	S	<=1	S	<=1	S	<=1	S	<=4
<=32	S	>8	R	<=1	S	<=1	S	<=1	S	<=4
<=32	S	>8	R	<=1	S	2	S	<=1	S	>8
<=32	S	>8	R	<=1	S	2	S	<=1	S	8
<=32	S	<=2	S	<=1	S	<=1	S	<=1	S	<=4
<=32	S	>8	R	<=1	S	<=1	S	4	R	<=4
<=32	S	>8	R	<=1	S	<=1	S	<=1	S	<=4
64	R	>8	R	>8	R	>4	R	>8	R	>8
<=32	S	<=2	S	<=1	S	<=1	S	<=1	S	<=4
>64	R	<=2	S	8	R	<=1	S	8	R	<=4
<=32	S	>8	R	>8	R	2	S	>8	R	<=4
<=32	S	>8	R	<=1	S	2	S	<=1	S	8
<=32	S	<=2	S	<=1	S	<=1	S	<=1	S	<=4
64	R	<=2	S	>8	R	<=1	S	8	R	8
<=32	S	>8	R	>8	R	>4	R	>8	R	8
<=32	S	>8	R	<=1	S	>4	R	<=1	S	8

64	R	>8	R	>8	R	>4	R	>8	R	<=4
<=32	S	>8	R	8	R	<=1	S	>8	R	<=4
<=32	S	>8	R	>8	R	>4	R	>8	R	8
<=32	S	<=2	S	>8	R	>4	R	>8	R	>8
<=32	S	>8	R	>8	R	2	S	>8	R	8
<=32	S	>8	R	>8	R	>4	R	>8	R	<=4
>64	R	>8	R	<=1	S	<=1	S	<=1	S	<=4
<=32	S	>8	R	>8	R	<=1	S	8	R	<=4
<=32	S	>8	R	8	R	<=1	S	>8	R	8
<=32	S	>8	R	2	I	4	I	>8	R	>8
<=32	S	>8	R	>8	R	<=1	S	>8	R	<=4
<=32	S	>8	R	2	I	<=1	S	<=1	S	>8
<=32	S	>8	R	<=1	S	>4	R	<=1	S	<=4
<=32	S	>8	R	<=1	S	>4	R	2	I	>8
<=32	S	>8	R	<=1	S	2	S	<=1	S	<=4
<=32	S	>8	R	>8	R	<=1	S	>8	R	>8
<=32	S	>8	R	<=1	S	4	I	<=1	S	<=4
<=32	S	<=2	S	<=1	S	>4	R	>8	R	8
<=32	S	>8	R	<=1	S	<=1	S	4	R	<=4

<=32	S	>8	R	>8	R	>4	R	>8	R	>8

>64	R	>8	R	<=1	S	>4	R	<=1	S	<=4
<=32	S	>8	R	>8	R	<=1	S	8	R	8
<=32	S	>8	R	8	R	>4	R	>8	R	>8
<=32	S	>8	R	<=1	S	>4	R	<=1	S	8
<=32	S	>8	R	>8	R	>4	R	>8	R	>8
>64	R	>8	R	>8	R	<=1	S	>8	R	<=4
<=32	S	>8	R	>8	R	>4	R	>8	R	<=4
64	R	<=2	S	>8	R	2	S	>8	R	<=4
>64	R	<=2	S	<=1	S	<=1	S	<=1	S	8
<=32	S	>8	R	<=1	S	2	S	<=1	S	<=4
<=32	S	>8	R	<=1	S	2	S	<=1	S	<=4
<=32	S	>8	R	<=1	S	2	S	<=1	S	<=4
<=32	S	>8	R	2	I	<=1	S	<=1	S	<=4
<=32	S	>8	R	<=1	S	<=1	S	<=1	S	>8
<=32	S	>8	R	<=1	S	<=1	S	<=1	S	<=4
<=32	S	>8	R	2	I	<=1	S	<=1	S	<=4
<=32	S	>8	R	<=1	S	<=1	S	<=1	S	<=4
<=32	S	>8	R	>8	R	>4	R	>8	R	<=4
64	R	>8	R	>8	R	<=1	S	>8	R	<=4
64	R	>8	R	>8	R	>4	R	>8	R	>8
<=32	S	>8	R	8	R	<=1	S	>8	R	<=4

<=32	S	<=2	S	>8	R	>4	R	>8	R	>8
<=32	S	>8	R	<=1	S	<=1	S	<=1	S	<=4
<=32	S	<=2	S	<=1	S	4	I	<=1	S	>8

<=32	S	<=2	S	>8	R	<=1	S	>8	R	<=4
<=32	S	>8	R	<=1	S	<=1	S	<=1	S	<=4
<=32	S	>8	R	<=1	S	>4	R	<=1	S	8
<=32	S	>8	R	<=1	S	>4	R	<=1	S	8
<=32	S	>8	R	<=1	S	>4	R	<=1	S	>8
<=32	S	>8	R	>8	R	>4	R	>8	R	<=4
<=32	S	<=2	S	2	I	>4	R	8	R	8
<=32	S	>8	R	4	R	4	I	>8	R	>8
<=32	S	>8	R	2	I	<=1	S	<=1	S	<=4
>64	R	<=2	S	<=1	S	<=1	S	<=1	S	<=4
<=32	S	<=2	S	<=1	S	4	I	<=1	S	<=4
<=32	S	>8	R	<=1	S	>4	R	<=1	S	8
<=32	S	>8	R	<=1	S	<=1	S	<=1	S	<=4
<=32	S	>8	R	<=1	S	>4	R	2	I	>8
<=32	S	>8	R	>8	R	>4	R	>8	R	<=4
<=32	S	4	S	<=1	S	<=1	S	<=1	S	<=4
<=32	S	>8	R	>8	R	>4	R	>8	R	>8
<=32	S	>8	R	8	R	<=1	S	2	I	<=4
<=32	S	>8	R	<=1	S	<=1	S	<=1	S	>8
<=32	S	>8	R	4	R	<=1	S	<=1	S	<=4
<=32	S	<=2	S	8	R	<=1	S	>8	R	<=4
<=32	S	>8	R	2	I	>4	R	2	I	>8
>64	R	>8	R	>8	R	>4	R	>8	R	<=4
<=32	S	>8	R	<=1	S	>4	R	<=1	S	8
<=32	S	>8	R	8	R	<=1	S	>8	R	<=4
<=32	S	>8	R	>8	R	>4	R	>8	R	>8
<=32	S	>8	R	<=1	S	>4	R	2	I	8
<=32	S	<=2	S	2	I	>4	R	8	R	8
<=32	S	<=2	S	2	I	>4	R	8	R	8
<=32	S	>8	R	8	R	>4	R	>8	R	>8
<=32	S	<=2	S	<=1	S	<=1	S	<=1	S	<=4
<=32	S	<=2	S	<=1	S	<=1	S	<=1	S	<=4
<=32	S	>8	R	>8	R	<=1	S	>8	R	8
64	R	<=2	S	2	I	>4	R	>8	R	<=4
<=32	S	>8	R	>8	R	2	S	>8	R	8
<=32	S	>8	R	>8	R	2	S	>8	R	8
64	R	>8	R	<=1	S	<=1	S	<=1	S	<=4
<=32	S	<=2	S	2	I	>4	R	8	R	8
<=32	S	>8	R	8	R	>4	R	>8	R	<=4
<=32	S	>8	R	<=1	S	<=1	S	<=1	S	<=4
<=32	S	>8	R	<=1	S	<=1	S	<=1	S	<=4
<=32	S	>8	R	<=1	S	<=1	S	<=1	S	<=4
<=32	S	<=2	S	2	I	>4	R	8	R	8
>64	R	>8	R	>8	R	>4	R	>8	R	>8
<=32	S	<=2	S	>8	R	<=1	S	>8	R	<=4
<=32	S	>8	R	<=1	S	>4	R	<=1	S	<=4
<=32	S	<=2	S	<=1	S	2	S	<=1	S	<=4
64	R	<=2	S	2	I	<=1	S	<=1	S	<=4

<=32	S	>8	R	>8	R	>4	R	>8	R	<=4
64	R	>8	R	>8	R	>4	R	>8	R	8
>64	R	<=2	S	<=1	S	2	S	<=1	S	<=4
<=32	S	>8	R	>8	R	>4	R	>8	R	<=4
<=32	S	<=2	S	<=1	S	<=1	S	<=1	S	<=4
<=32	S	<=2	S	<=1	S	>4	R	<=1	S	<=4
<=32	S	<=2	S	>8	R	<=1	S	>8	R	<=4
<=32	S	<=2	S	<=1	S	<=1	S	<=1	S	<=4
<=32	S	<=2	S	2	I	>4	R	>8	R	8
<=32	S	>8	R	2	I	4	I	>8	R	>8
<=32	S	>8	R	8	R	>4	R	4	R	>8
<=32	S	<=2	S	<=1	S	<=1	S	<=1	S	<=4
64	R	4	S	<=1	S	<=1	S	<=1	S	>8
<=32	S	>8	R	8	R	<=1	S	4	R	>8
<=32	S	<=2	S	<=1	S	<=1	S	<=1	S	<=4
<=32	S	>8	R	<=1	S	<=1	S	<=1	S	<=4
<=32	S	<=2	S	<=1	S	<=1	S	<=1	S	<=4
<=32	S	<=2	S	<=1	S	<=1	S	<=1	S	<=4
<=32	S	>8	R	>8	R	>4	R	>8	R	<=4

<=32	S	>8	R	4	R	<=1	S	8	R	<=4
<=32	S	>8	R	2	I	4	I	>8	R	>8
<=32	S	<=2	S	<=1	S	<=1	S	<=1	S	<=4
<=32	S	>8	R	<=1	S	<=1	S	<=1	S	<=4
<=32	S	>8	R	<=1	S	<=1	S	<=1	S	<=4
<=32	S	>8	R	>8	R	<=1	S	>8	R	<=4
<=32	S	>8	R	8	R	<=1	S	>8	R	>8
64	R	>8	R	2	I	2	S	2	I	<=4
<=32	S	>8	R	<=1	S	<=1	S	<=1	S	<=4
<=32	S	>8	R	4	R	<=1	S	4	R	>8
<=32	S	>8	R	4	R	<=1	S	8	R	>8
<=32	S	>8	R	<=1	S	>4	R	<=1	S	>8
<=32	S	>8	R	2	I	<=1	S	<=1	S	>8
<=32	S	>8	R	>8	R	>4	R	>8	R	<=4
<=32	S	8	I	N/R		<=1	S	<=1	S	>8

<=32	S	>8	R	8	R	>4	R	>8	R	<=4
64	R	>8	R	4	R	>4	R	>8	R	<=4

<=32	S	>8	R	>8	R	>4	R	>8	R	<=4
<=32	S	>8	R	<=1	S	>4	R	<=1	S	<=4
<=32	S	<=2	S	N/R		<=1	S	>8	R	>8
64	R	>8	R	<=1	S	>4	R	8	R	<=4
<=32	S	>8	R	>8	R	>4	R	>8	R	<=4
<=32	S	>8	R	<=1	S	<=1	S	<=1	S	<=4
<=32	S	>8	R	8	R	>4	R	>8	R	<=4

<=32	S	>8	R	>8	R	2	S	>8	R	8

<=32	S	<=2	S	<=1	S	<=1	S	<=1	S	<=4

<=32	S	>8	R	<=1	S	>4	R	<=1	S	>8
64	R	>8	R	>8	R	>4	R	>8	R	<=4
<=32	S	<=2	S	<=1	S	4	I	<=1	S	8
<=32	S	>8	R	>8	R	>4	R	>8	R	<=4
<=32	S	>8	R	<=1	S	<=1	S	8	R	<=4
<=32	S	>8	R	>8	R	>4	R	>8	R	<=4
<=32	S	<=2	S	<=1	S	<=1	S	<=1	S	<=4
<=32	S	>8	R	8	R	>4	R	>8	R	<=4
<=32	S	<=2	S	<=1	S	>4	R	<=1	S	<=4
<=32	S	>8	R	4	R	<=1	S	8	R	>8
<=32	S	>8	R	8	R	>4	R	>8	R	<=4
<=32	S	>8	R	8	R	2	S	>8	R	<=4
<=32	S	>8	R	>8	R	>4	R	>8	R	<=4
<=32	S	>8	R	>8	R	>4	R	>8	R	<=4
<=32	S	>8	R	4	R	<=1	S	8	R	>8
<=32	S	>8	R	2	I	>4	R	>8	R	8
<=32	S	>8	R	<=1	S	<=1	S	4	R	<=4
<=32	S	>8	R	<=1	S	>4	R	<=1	S	>8
64	R	<=2	S	8	R	<=1	S	8	R	<=4
<=32	S	>8	R	2	I	>4	R	8	R	8
<=32	S	>8	R	8	R	<=1	S	>8	R	<=4
<=32	S	<=2	S	<=1	S	>4	R	>8	R	8

<=32	S	>8	R	>8	R	4	I	2	I	8
<=32	S	>8	R	8	R	<=1	S	>8	R	<=4
<=32	S	>8	R	2	I	<=1	S	8	R	>8
<=32	S	<=2	S	<=1	S	<=1	S	<=1	S	<=4
<=32	S	>8	R	8	R	4	I	<=1	S	8
<=32	S	4	S	>8	R	<=1	S	>8	R	8
<=32	S	>8	R	>8	R	>4	R	>8	R	8
<=32	S	>8	R	<=1	S	<=1	S	<=1	S	>8
<=32	S	<=2	S	<=1	S	>4	R	<=1	S	<=4
<=32	S	>8	R	>8	R	<=1	S	>8	R	<=4
<=32	S	>8	R	>8	R	<=1	S	>8	R	<=4

<=32	S	>8	R	>8	R	4	I	>8	R	<=4
<=32	S	>8	R	8	R	>4	R	>8	R	<=4
<=32	S	>8	R	<=1	S	>4	R	<=1	S	>8
<=32	S	>8	R	<=1	S	>4	R	8	R	<=4
<=32	S	>8	R	<=1	S	>4	R	4	R	>8
<=32	S	>8	R	<=1	S	>4	R	4	R	<=4
<=32	S	>8	R	>8	R	>4	R	>8	R	<=4
<=32	S	>8	R	<=1	S	>4	R	4	R	>8
<=32	S	>8	R	2	I	>4	R	8	R	>8
<=32	S	<=2	S	4	R	>4	R	>8	R	<=4
<=32	S	>8	R	>8	R	<=1	S	>8	R	<=4

<=32	S	8	I	<=1	S	2	S	2	I	<=4
<=32	S	>8	R	<=1	S	<=1	S	2	I	<=4
<=32	S	>8	R	N/R		<=1	S	<=1	S	>8
64	R	>8	R	>8	R	4	I	>8	R	>8
<=32	S	8	I	8	R	>4	R	<=1	S	<=4
<=32	S	>8	R	2	I	>4	R	8	R	>8
64	R	>8	R	>8	R	>4	R	>8	R	>8
<=32	S	>8	R	2	I	<=1	S	<=1	S	>8
<=32	S	>8	R	<=1	S	4	I	2	I	>8
<=32	S	4	S	<=1	S	<=1	S	<=1	S	<=4
<=32	S	<=2	S	4	R	>4	R	>8	R	<=4
<=32	S	>8	R	>8	R	>4	R	>8	R	>8
<=32	S	>8	R	>8	R	>4	R	>8	R	>8
<=32	S	>8	R	>8	R	>4	R	>8	R	>8
<=32	S	<=2	S	<=1	S	<=1	S	<=1	S	8
<=32	S	<=2	S	<=1	S	2	S	<=1	S	>8
<=32	S	>8	R	<=1	S	>4	R	4	R	8
<=32	S	>8	R	2	I	<=1	S	<=1	S	<=4
<=32	S	>8	R	<=1	S	>4	R	<=1	S	<=4
<=32	S	>8	R	<=1	S	2	S	<=1	S	<=4
<=32	S	>8	R	<=1	S	<=1	S	8	R	<=4
64	R	>8	R	>8	R	>4	R	>8	R	8
64	R	>8	R	2	I	<=1	S	8	R	<=4
>64	R	>8	R	>8	R	4	I	>8	R	>8
<=32	S	>8	R	>8	R	>4	R	>8	R	>8
<=32	S	>8	R	8	R	>4	R	8	R	>8
<=32	S	>8	R	>8	R	<=1	S	>8	R	<=4
<=32	S	>8	R	<=1	S	>4	R	8	R	<=4
64	R	>8	R	>8	R	>4	R	>8	R	>8
<=32	S	>8	R	2	I	>4	R	8	R	>8
<=32	S	<=2	S	2	I	4	I	>8	R	<=4
64	R	>8	R	8	R	2	S	>8	R	>8
64	R	>8	R	>8	R	4	I	>8	R	>8
64	R	>8	R	4	R	2	S	<=1	S	8
<=32	S	>8	R	>8	R	2	S	>8	R	8
<=32	S	>8	R	2	I	<=1	S	2	I	<=4
<=32	S	<=2	S	<=1	S	4	I	<=1	S	<=4
<=32	S	>8	R	4	R	>4	R	>8	R	>8
<=32	S	<=2	S	4	R	<=1	S	4	R	<=4
<=32	S	>8	R	2	I	>4	R	8	R	<=4
<=32	S	<=2	S	<=1	S	2	S	<=1	S	<=4
<=32	S	>8	R	8	R	>4	R	>8	R	<=4
64	R	>8	R	2	I	>4	R	>8	R	>8
<=32	S	>8	R	8	R	<=1	S	>8	R	<=4
64	R	>8	R	>8	R	>4	R	>8	R	8
<=32	S	<=2	S	<=1	S	2	S	<=1	S	<=4
<=32	S	>8	R	>8	R	>4	R	>8	R	<=4
<=32	S	>8	R	>8	R	>4	R	>8	R	>8
>64	R	>8	R	8	R	2	S	>8	R	<=4
64	R	>8	R	4	R	4	I	<=1	S	8
<=32	S	>8	R	>8	R	>4	R	>8	R	<=4
<=32	S	4	S	<=1	S	>4	R	<=1	S	<=4
<=32	S	<=2	S	<=1	S	>4	R	>8	R	>8
<=32	S	>8	R	<=1	S	<=1	S	<=1	S	<=4
<=32	S	>8	R	>8	R	4	I	>8	R	<=4

<=32	S	>8	R	<=1	S	<=1	S	<=1	S	8
<=32	S	>8	R	<=1	S	<=1	S	<=1	S	>8
<=32	S	4	S	<=1	S	>4	R	<=1	S	<=4

<=32	S	>8	R	>8	R	>4	R	>8	R	<=4
<=32	S	>8	R	<=1	S	<=1	S	<=1	S	<=4
>64	R	>8	R	<=1	S	>4	R	4	R	8
<=32	S	>8	R	>8	R	>4	R	>8	R	<=4
<=32	S	>8	R	>8	R	>4	R	>8	R	<=4
<=32	S	>8	R	<=1	S	>4	R	<=1	S	8
64	R	>8	R	2	I	4	I	<=1	S	<=4
<=32	S	<=2	S	<=1	S	4	I	2	I	8
<=32	S	>8	R	8	R	>4	R	>8	R	<=4
<=32	S	>8	R	<=1	S	<=1	S	<=1	S	<=4
<=32	S	>8	R	>8	R	>4	R	>8	R	>8
<=32	S	>8	R	4	R	<=1	S	8	R	>8
<=32	S	>8	R	>8	R	4	I	>8	R	<=4
<=32	S	>8	R	<=1	S	>4	R	<=1	S	<=4
<=32	S	>8	R	2	I	>4	R	>8	R	>8
<=32	S	>8	R	8	R	>4	R	>8	R	<=4
<=32	S	<=2	S	>8	R	2	S	>8	R	<=4
<=32	S	>8	R	8	R	<=1	S	8	R	<=4
<=32	S	<=2	S	2	I	4	I	<=1	S	8
<=32	S	<=2	S	2	I	4	I	<=1	S	8
<=32	S	>8	R	>8	R	4	I	>8	R	>8
<=32	S	>8	R	8	R	<=1	S	>8	R	<=4
64	R	>8	R	>8	R	<=1	S	>8	R	<=4
<=32	S	>8	R	2	I	>4	R	<=1	S	>8
<=32	S	>8	R	<=1	S	<=1	S	<=1	S	<=4
<=32	S	>8	R	<=1	S	<=1	S	<=1	S	<=4
<=32	S	>8	R	<=1	S	>4	R	<=1	S	<=4
<=32	S	>8	R	4	R	>4	R	>8	R	<=4
64	R	>8	R	8	R	2	S	8	R	<=4
<=32	S	>8	R	2	I	>4	R	8	R	8
<=32	S	<=2	S	<=1	S	4	I	<=1	S	<=4
64	R	>8	R	<=1	S	<=1	S	<=1	S	<=4
<=32	S	>8	R	>8	R	>4	R	>8	R	>8
<=32	S	>8	R	>8	R	>4	R	>8	R	>8
>64	R	>8	R	>8	R	>4	R	>8	R	8
<=32	S	>8	R	2	I	<=1	S	<=1	S	<=4
<=32	S	>8	R	4	R	4	I	<=1	S	>8
64	R	>8	R	>8	R	<=1	S	>8	R	8
<=32	S	>8	R	2	I	>4	R	<=1	S	>8
<=32	S	>8	R	>8	R	>4	R	>8	R	<=4
<=32	S	>8	R	<=1	S	4	I	<=1	S	<=4
<=32	S	>8	R	<=1	S	>4	R	<=1	S	>8
<=32	S	<=2	S	<=1	S	<=1	S	<=1	S	<=4

<=32	S	>8	R	>8	R	>4	R	>8	R	>8
<=32	S	>8	R	8	R	>4	R	8	R	>8
<=32	S	<=2	S	<=1	S	<=1	S	<=1	S	<=4
<=32	S	<=2	S	2	I	>4	R	>8	R	8
>64	R	>8	R	>8	R	>4	R	>8	R	8
<=32	S	<=2	S	<=1	S	<=1	S	<=1	S	<=4
<=32	S	>8	R	>8	R	>4	R	>8	R	<=4

<=32	S	>8	R	2	I	>4	R	>8	R	<=4
<=32	S	>8	R	>8	R	<=1	S	>8	R	<=4
<=32	S	>8	R	8	R	>4	R	>8	R	>8
<=32	S	>8	R	<=1	S	>4	R	2	I	>8
<=32	S	<=2	S	4	R	2	S	<=1	S	8
<=32	S	>8	R	<=1	S	<=1	S	<=1	S	>8
<=32	S	>8	R	2	I	4	I	>8	R	>8
<=32	S	>8	R	<=1	S	>4	R	<=1	S	>8

<=32	S	<=2	S	>8	R	<=1	S	>8	R	<=4
<=32	S	>8	R	2	I	>4	R	>8	R	8
<=32	S	<=2	S	2	I	<=1	S	<=1	S	<=4
<=32	S	>8	R	<=1	S	2	S	<=1	S	>8
<=32	S	>8	R	<=1	S	<=1	S	<=1	S	<=4
64	R	>8	R	<=1	S	<=1	S	<=1	S	>8
64	R	>8	R	<=1	S	<=1	S	8	R	8
64	R	<=2	S	8	R	>4	R	<=1	S	8
64	R	>8	R	2	I	<=1	S	8	R	<=4
64	R	8	I	4	R	>4	R	<=1	S	>8
>64	R	>8	R	>8	R	<=1	S	>8	R	>8
64	R	<=2	S	>8	R	>4	R	>8	R	<=4
<=32	S	<=2	S	8	R	2	S	4	R	<=4
<=32	S	>8	R	8	R	>4	R	>8	R	>8
64	R	>8	R	>8	R	>4	R	>8	R	>8
<=32	S	<=2	S	2	I	>4	R	8	R	8
64	R	<=2	S	<=1	S	4	I	<=1	S	8
<=32	S	>8	R	2	I	<=1	S	<=1	S	<=4
>64	R	<=2	S	<=1	S	>4	R	<=1	S	<=4
<=32	S	>8	R	<=1	S	>4	R	2	I	>8
<=32	S	<=2	S	<=1	S	>4	R	8	R	>8
<=32	S	>8	R	>8	R	4	I	>8	R	>8
<=32	S	>8	R	2	I	<=1	S	2	I	<=4
<=32	S	>8	R	8	R	<=1	S	>8	R	<=4
<=32	S	>8	R	>8	R	>4	R	>8	R	>8
<=32	S	>8	R	<=1	S	<=1	S	<=1	S	>8
<=32	S	>8	R	>8	R	>4	R	>8	R	<=4
64	R	>8	R	2	I	4	I	<=1	S	8
64	R	>8	R	>8	R	4	I	>8	R	>8
<=32	S	<=2	S	>8	R	<=1	S	>8	R	<=4
<=32	S	>8	R	4	R	<=1	S	8	R	>8
<=32	S	4	S	<=1	S	>4	R	<=1	S	<=4
<=32	S	>8	R	<=1	S	<=1	S	<=1	S	<=4
64	R	>8	R	<=1	S	4	I	<=1	S	>8
<=32	S	>8	R	<=1	S	>4	R	<=1	S	<=4
>64	R	>8	R	>8	R	<=1	S	>8	R	>8
<=32	S	>8	R	2	I	>4	R	8	R	<=4
<=32	S	>8	R	4	R	>4	R	<=1	S	<=4

<=32	S	>8	R	>8	R	>4	R	>8	R	8
<=32	S	>8	R	>8	R	>4	R	>8	R	<=4
<=32	S	>8	R	>8	R	>4	R	>8	R	<=4
<=32	S	>8	R	8	R	<=1	S	>8	R	<=4
<=32	S	>8	R	>8	R	>4	R	>8	R	<=4
64	R	>8	R	>8	R	>4	R	>8	R	<=4

>64	R	>8	R	>8	R	>4	R	>8	R	>8

<=32	S	>8	R	<=1	S	<=1	S	<=1	S	<=4
64	R	>8	R	<=1	S	2	S	8	R	<=4
<=32	S	<=2	S	<=1	S	<=1	S	<=1	S	<=4
<=32	S	>8	R	<=1	S	<=1	S	<=1	S	>8
<=32	S	<=2	S	<=1	S	<=1	S	<=1	S	<=4
<=32	S	<=2	S	>8	R	4	I	>8	R	8
<=32	S	<=1	S	<=1	S	>4	R	<=1	S	<=4
<=32	S	<=2	S	<=1	S	>4	R	8	R	<=4
<=32	S	>8	R	<=1	S	>4	R	<=1	S	<=4
<=32	S	>8	R	>8	R	>4	R	>8	R	<=4
<=32	S	<=2	S	<=1	S	<=1	S	<=1	S	>8
<=32	S	>8	R	>8	R	>4	R	>8	R	8
64	R	>8	R	>8	R	>4	R	>8	R	8
<=32	S	>8	R	<=1	S	>4	R	4	R	>8

<=32	S	>8	R	<=1	S	>4	R	<=1	S	<=4
<=32	S	>8	R	>8	R	<=1	S	>8	R	<=4
<=32	S	>8	R	8	R	>4	R	>8	R	<=4
<=32	S	>8	R	>8	R	>4	R	>8	R	>8
<=32	S	>8	R	8	R	2	S	8	R	>8
<=32	S	>8	R	8	R	<=1	S	>8	R	>8
64	R	<=2	S	4	R	<=1	S	<=1	S	<=4
<=32	S	>8	R	>8	R	>4	R	>8	R	<=4
<=32	S	>8	R	8	R	<=1	S	>8	R	<=4
<=32	S	>8	R	8	R	>4	R	>8	R	>8
64	R	>8	R	8	R	>4	R	<=1	S	8
<=32	S	<=2	S	<=1	S	2	S	<=1	S	<=4
<=32	S	>8	R	>8	R	>4	R	>8	R	>8
<=32	S	>8	R	>8	R	>4	R	>8	R	<=4
<=32	S	>8	R	2	I	>4	R	8	R	<=4
<=32	S	>8	R	>8	R	>4	R	>8	R	8
<=32	S	>8	R	<=1	S	<=1	S	<=1	S	<=4
<=32	S	>8	R	4	R	>4	R	8	R	<=4
<=32	S	<=2	S	<=1	S	>4	R	<=1	S	<=4
<=32	S	>8	R	<=1	S	<=1	S	<=1	S	>8
<=32	S	<=2	S	>8	R	<=1	S	>8	R	<=4

<=32	S	>8	R	>8	R	<=1	S	>8	R	<=4
<=32	S	>8	R	<=1	S	>4	R	8	R	>8
<=32	S	>8	R	<=1	S	>4	R	<=1	S	<=4
<=32	S	>8	R	>8	R	>4	R	>8	R	<=4
<=32	S	>8	R	>8	R	>4	R	>8	R	<=4
<=32	S	>8	R	>8	R	>4	R	>8	R	8
<=32	S	>8	R	2	I	>4	R	8	R	<=4
<=32	S	8	I	>8	R	<=1	S	>8	R	<=4
<=32	S	>8	R	2	I	>4	R	8	R	<=4
<=32	S	>8	R	4	R	>4	R	8	R	<=4
<=32	S	>8	R	<=1	S	>4	R	2	I	>8
<=32	S	8	I	4	R	>4	R	<=1	S	>8
<=32	S	>8	R	>8	R	>4	R	>8	R	>8
<=32	S	<=2	S	<=1	S	>4	R	8	R	>8
<=32	S	>8	R	>8	R	2	S	>8	R	<=4
<=32	S	>8	R	>8	R	>4	R	>8	R	<=4
<=32	S	>8	R	<=1	S	>4	R	<=1	S	<=4
<=32	S	>8	R	<=1	S	>4	R	>8	R	<=4
64	R	>8	R	8	R	4	I	8	R	<=4
<=32	S	>8	R	>8	R	<=1	S	>8	R	<=4
<=32	S	>8	R	2	I	>4	R	<=1	S	>8
<=32	S	>8	R	2	I	>4	R	8	R	8
<=32	S	>8	R	>8	R	>4	R	>8	R	<=4
<=32	S	>8	R	2	I	>4	R	<=1	S	>8
<=32	S	>8	R	2	I	2	S	2	I	<=4
<=32	S	>8	R	>8	R	4	I	>8	R	>8
64	R	>8	R	<=1	S	2	S	2	I	<=4
>64	R	>8	R	>8	R	>4	R	>8	R	<=4
<=32	S	>8	R	>8	R	<=1	S	>8	R	<=4
<=32	S	>8	R	<=1	S	>4	R	8	R	<=4
<=32	S	<=2	S	<=1	S	<=1	S	<=1	S	<=4
<=32	S	>8	R	<=1	S	<=1	S	<=1	S	<=4
<=32	S	>8	R	8	R	<=1	S	>8	R	<=4
<=32	S	>8	R	>8	R	<=1	S	>8	R	>8
<=32	S	<=2	S	<=1	S	>4	R	<=1	S	<=4
<=32	S	>8	R	>8	R	>4	R	>8	R	>8
<=32	S	4	S	<=1	S	>4	R	<=1	S	<=4
<=32	S	>8	R	2	I	2	S	<=1	S	<=4
<=32	S	<=2	S	>8	R	>4	R	>8	R	>8
<=32	S	<=2	S	2	I	>4	R	8	R	8
<=32	S	<=2	S	<=1	S	2	S	<=1	S	<=4
<=32	S	>8	R	8	R	2	S	>8	R	<=4
<=32	S	<=2	S	<=1	S	<=1	S	<=1	S	>8
<=32	S	>8	R	<=1	S	>4	R	8	R	<=4
<=32	S	>8	R	8	R	<=1	S	8	R	<=4
<=32	S	<=2	S	<=1	S	>4	R	<=1	S	<=4
<=32	S	>8	R	<=1	S	>4	R	<=1	S	<=4
<=32	S	>8	R	8	R	<=1	S	8	R	<=4
<=32	S	>8	R	2	I	>4	R	>8	R	8
<=32	S	>8	R	<=1	S	>4	R	8	R	8
<=32	S	<=2	S	<=1	S	2	S	<=1	S	<=4
64	R	<=2	S	2	I	>4	R	8	R	<=4
<=32	S	>8	R	<=1	S	>4	R	<=1	S	<=4
<=32	S	>8	R	>8	R	<=1	S	>8	R	<=4
<=32	S	>8	R	4	R	>4	R	>8	R	<=4

<=32	S	>8	R	<=1	S	4	I	2	I	>8
64	R	>8	R	<=1	S	<=1	S	8	R	<=4

<=32	S	<=2	S	<=1	S	>4	R	<=1	S	<=4
>64	R	>8	R	2	I	4	I	>8	R	>8

<=32	S	<=2	S	<=1	S	>4	R	<=1	S	>8
<=32	S	8	I	2	I	>4	R	8	R	<=4
64	R	>8	R	>8	R	>4	R	>8	R	>8
<=32	S	>8	R	>8	R	<=1	S	>8	R	<=4

<=32	S	>8	R	>8	R	<=1	S	>8	R	<=4
<=32	S	>8	R	8	R	>4	R	>8	R	<=4
<=32	S	>8	R	>8	R	4	I	>8	R	<=4
<=32	S	>8	R	<=1	S	>4	R	8	R	<=4
<=32	S	<=2	S	<=1	S	>4	R	<=1	S	>8
<=32	S	>8	R	>8	R	>4	R	>8	R	<=4
<=32	S	>8	R	>8	R	<=1	S	>8	R	<=4
<=32	S	>8	R	2	I	2	S	8	R	<=4
<=32	S	>8	R	<=1	S	>4	R	<=1	S	<=4
<=32	S	>8	R	<=1	S	<=1	S	<=1	S	<=4
<=32	S	>8	R	<=1	S	<=1	S	<=1	S	<=4
<=32	S	>8	R	8	R	>4	R	>8	R	<=4
<=32	S	>8	R	2	I	2	S	2	I	<=4
<=32	S	>8	R	<=1	S	2	S	4	R	<=4
<=32	S	>8	R	<=1	S	<=1	S	8	R	<=4
>64	R	8	I	<=1	S	4	I	4	R	8
<=32	S	>8	R	<=1	S	4	I	<=1	S	<=4
<=32	S	>8	R	4	R	2	S	<=1	S	<=4
<=32	S	>8	R	<=1	S	2	S	<=1	S	<=4
<=32	S	>8	R	<=1	S	<=1	S	<=1	S	<=4
<=32	S	>8	R	>8	R	>4	R	>8	R	<=4
<=32	S	<=2	S	<=1	S	<=1	S	<=1	S	<=4
<=32	S	<=2	S	2	I	<=1	S	<=1	S	<=4
<=32	S	4	S	<=1	S	<=1	S	<=1	S	<=4
<=32	S	>8	R	<=1	S	<=1	S	<=1	S	<=4
<=32	S	>8	R	2	I	<=1	S	<=1	S	8
<=32	S	>8	R	<=1	S	>4	R	<=1	S	8
<=32	S	<=2	S	<=1	S	<=1	S	<=1	S	<=4
<=32	S	>8	R	<=1	S	>4	R	<=1	S	8
<=32	S	>8	R	2	I	>4	R	8	R	<=4
<=32	S	>8	R	2	I	>4	R	<=1	S	>8
<=32	S	>8	R	>8	R	<=1	S	>8	R	8
<=32	S	>8	R	>8	R	<=1	S	>8	R	<=4
<=32	S	>8	R	<=1	S	<=1	S	<=1	S	<=4
<=32	S	>8	R	2	I	>4	R	8	R	>8
<=32	S	>8	R	2	I	>4	R	8	R	>8
<=32	S	>8	R	>8	R	>4	R	>8	R	>8
<=32	S	4	S	2	I	>4	R	8	R	8
<=32	S	>8	R	>8	R	>4	R	>8	R	>8
<=32	S	>8	R	<=1	S	2	S	4	R	<=4
<=32	S	<=2	S	2	I	<=1	S	<=1	S	<=4
<=32	S	>8	R	4	R	>4	R	8	R	<=4
<=32	S	>8	R	<=1	S	<=1	S	<=1	S	<=4
>64	R	>8	R	2	I	4	I	8	R	<=4
64	R	<=2	S	<=1	S	<=1	S	<=1	S	<=4
<=32	S	<=2	S	4	R	>4	R	>8	R	<=4
<=32	S	<=2	S	<=1	S	<=1	S	<=1	S	<=4
<=32	S	>8	R	2	I	>4	R	8	R	8
<=32	S	>8	R	<=1	S	>4	R	<=1	S	8
<=32	S	>8	R	>8	R	>4	R	>8	R	<=4
<=32	S	>8	R	>8	R	>4	R	>8	R	<=4

>64	R	>8	R	8	R	>4	R	>8	R	>8	
<=32	S	>8	R	>8	R	>4	R	>8	R	<=4	
<=32	S	<=2	S	2	I	<=1	S	<=1	S	<=4	
<=32	S	>8	R	2	I	<=1	S	<=1	S	<=4	
<=32	S	>8	R	<=1	S	>4	R	<=1	S	<=4	

<=32	S	<=2	S	<=1	S	>4	R	<=1	S	8
64	R	<=2	S	<=1	S	4	I	<=1	S	<=4
64	R	>8	R	4	R	<=1	S	8	R	>8
<=32	S	>8	R	<=1	S	<=1	S	<=1	S	8
<=32	S	>8	R	<=1	S	<=1	S	<=1	S	<=4
<=32	S	<=2	S	<=1	S	<=1	S	<=1	S	<=4
<=32	S	<=2	S	2	I	>4	R	8	R	8
<=32	S	<=2	S	<=1	S	2	S	<=1	S	<=4
<=32	S	>8	R	<=1	S	2	S	<=1	S	<=4
<=32	S	>8	R	<=1	S	<=1	S	<=1	S	<=4
<=32	S	>8	R	8	R	>4	R	>8	R	>8
<=32	S	>8	R	>8	R	>4	R	>8	R	>8
<=32	S	>8	R	>8	R	4	I	>8	R	<=4
<=32	S	<=2	S	2	I	>4	R	8	R	<=4
<=32	S	8	I	8	R	<=1	S	8	R	8
<=32	S	>8	R	<=1	S	>4	R	<=1	S	>8
<=32	S	>8	R	>8	R	>4	R	>8	R	>8
<=32	S	>8	R	<=1	S	<=1	S	<=1	S	<=4

<=32	S	<=2	S	>8	R	<=1	S	>8	R	8
<=32	S	<=2	S	>8	R	<=1	S	>8	R	<=4
<=32	S	>8	R	<=1	S	<=1	S	<=1	S	<=4
<=32	S	>8	R	2	I	>4	R	8	R	>8
<=32	S	>8	R	>8	R	>4	R	>8	R	<=4
<=32	S	>8	R	>8	R	<=1	S	>8	R	<=4
<=32	S	>8	R	2	I	<=1	S	<=1	S	<=4
>64	R	>8	R	>8	R	4	I	>8	R	>8
<=32	S	>8	R	4	R	>4	R	8	R	<=4
<=32	S	>8	R	8	R	<=1	S	>8	R	<=4
<=32	S	<=2	S	<=1	S	>4	R	<=1	S	8
<=32	S	<=2	S	<=1	S	<=1	S	<=1	S	<=4
<=32	S	>8	R	>8	R	<=1	S	>8	R	<=4
<=32	S	>8	R	2	I	<=1	S	<=1	S	<=4
64	R	>8	R	>8	R	>4	R	>8	R	>8
<=32	S	>8	R	>8	R	4	I	>8	R	>8
>64	R	>8	R	4	R	>4	R	>8	R	>8
<=32	S	>8	R	8	R	<=1	S	8	R	<=4
<=32	S	>8	R	>8	R	>4	R	>8	R	<=4
>64	R	>8	R	8	R	>4	R	>8	R	>8
<=32	S	>8	R	<=1	S	<=1	S	<=1	S	<=4
<=32	S	>8	R	<=1	S	<=1	S	<=1	S	<=4

<=32	S	>8	R	2	I	>4	R	8	R	<=4
<=32	S	8	I	2	I	>4	R	8	R	>8
<=32	S	<=2	S	4	R	>4	R	>8	R	<=4
<=32	S	<=2	S	<=1	S	<=1	S	<=1	S	<=4
64	R	>8	R	8	R	<=1	S	>8	R	>8
<=32	S	>8	R	2	I	>4	R	>8	R	8
<=32	S	<=2	S	<=1	S	>4	R	8	R	<=4
<=32	S	>8	R	<=1	S	2	S	<=1	S	<=4
<=32	S	>8	R	>8	R	>4	R	>8	R	8
<=32	S	<=2	S	<=1	S	4	I	<=1	S	<=4
<=32	S	>8	R	2	I	>4	R	>8	R	8
<=32	S	>8	R	<=1	S	2	S	2	I	<=4
<=32	S	>8	R	2	I	>4	R	8	R	<=4
<=32	S	>8	R	2	I	>4	R	8	R	8
<=32	S	>8	R	<=1	S	>4	R	<=1	S	<=4
<=32	S	>8	R	<=1	S	<=1	S	<=1	S	<=4
64	R	>8	R	>8	R	>4	R	>8	R	>8
<=32	S	>8	R	>8	R	>4	R	>8	R	>8
<=32	S	>8	R	4	R	<=1	S	<=1	S	<=4
<=32	S	<=2	S	2	I	2	S	<=1	S	<=4
<=32	S	<=2	S	<=1	S	<=1	S	<=1	S	<=4
<=32	S	>8	R	<=1	S	>4	R	8	R	>8
<=32	S	>8	R	>8	R	>4	R	>8	R	<=4
<=32	S	>8	R	8	R	<=1	S	<=1	S	<=4
<=32	S	<=2	S	4	R	<=1	S	2	I	<=4
<=32	S	<=2	S	2	I	>4	R	>8	R	<=4
<=32	S	<=2	S	2	I	>4	R	>8	R	<=4
<=32	S	>8	R	<=1	S	<=1	S	8	R	<=4
64	R	>8	R	2	I	>4	R	>8	R	<=4
<=32	S	>8	R	<=1	S	>4	R	4	R	<=4
<=32	S	>8	R	<=1	S	<=1	S	<=1	S	<=4
<=32	S	<=2	S	4	R	>4	R	>8	R	<=4
<=32	S	>8	R	4	R	>4	R	8	R	<=4

<=32	S	<=2	S	2	I	<=1	S	2	I	<=4
<=32	S	>8	R	4	R	4	I	4	R	<=4
<=32	S	>8	R	>8	R	<=1	S	>8	R	<=4
<=32	S	>8	R	8	R	<=1	S	>8	R	<=4

<=32	S	>8	R	<=1	S	<=1	S	2	I	<=4
<=32	S	>8	R	2	I	<=1	S	4	R	<=4
<=32	S	8	I	<=1	S	>4	R	<=1	S	>8
<=32	S	>8	R	<=1	S	>4	R	<=1	S	8
<=32	S	>8	R	>8	R	>4	R	>8	R	>8
<=32	S	<=2	S	<=1	S	>4	R	<=1	S	<=4
<=32	S	>8	R	<=1	S	<=1	S	<=1	S	8

<=32	S	<=2	S	<=1	S	<=1	S	<=1	S	<=4
<=32	S	<=2	S	2	I	>4	R	8	R	8
<=32	S	>8	R	8	R	>4	R	>8	R	>8
<=32	S	>8	R	<=1	S	>4	R	<=1	S	8
<=32	S	<=2	S	<=1	S	<=1	S	<=1	S	<=4
<=32	S	>8	R	<=1	S	2	S	<=1	S	<=4
<=32	S	<=2	S	<=1	S	<=1	S	<=1	S	<=4
<=32	S	>8	R	<=1	S	>4	R	<=1	S	8
<=32	S	<=2	S	<=1	S	<=1	S	<=1	S	<=4
<=32	S	>8	R	<=1	S	>4	R	8	R	8
<=32	S	>8	R	<=1	S	>4	R	8	R	<=4
<=32	S	>8	R	4	R	4	I	<=1	S	>8
<=32	S	<=2	S	<=1	S	<=1	S	<=1	S	<=4
<=32	S	>8	R	>8	R	<=1	S	>8	R	8
64	R	>8	R	>8	R	>4	R	>8	R	>8
<=32	S	>8	R	<=1	S	<=1	S	<=1	S	<=4
>64	R	>8	R	8	R	>4	R	>8	R	<=4
<=32	S	>8	R	<=1	S	<=1	S	<=1	S	<=4
<=32	S	>8	R	>8	R	>4	R	>8	R	>8
<=32	S	<=2	S	<=1	S	<=1	S	2	I	<=4
<=32	S	>8	R	<=1	S	>4	R	8	R	8
<=32	S	>8	R	<=1	S	>4	R	<=1	S	<=4
<=32	S	>8	R	<=1	S	>4	R	8	R	8
<=32	S	>8	R	2	I	<=1	S	<=1	S	<=4
<=32	S	>8	R	<=1	S	<=1	S	<=1	S	<=4
<=32	S	<=2	S	2	I	>4	R	8	R	<=4
<=32	S	<=2	S	<=1	S	<=1	S	<=1	S	<=4
<=32	S	>8	R	<=1	S	2	S	<=1	S	>8
64	R	8	I	4	R	>4	R	4	R	<=4
>64	R	<=2	S	<=1	S	>4	R	4	R	<=4
<=32	S	<=2	S	2	I	>4	R	8	R	8
<=32	S	<=2	S	2	I	>4	R	8	R	8
<=32	S	>8	R	<=1	S	<=1	S	<=1	S	<=4
<=32	S	>8	R	<=1	S	<=1	S	<=1	S	<=4
<=32	S	>8	R	<=1	S	<=1	S	<=1	S	<=4
<=32	S	>8	R	>8	R	>4	R	>8	R	<=4
<=32	S	>8	R	<=1	S	>4	R	<=1	S	<=4
<=32	S	>8	R	<=1	I	<=1	S	<=1	S	<=4
64	R	>8	R	>8	R	>4	R	>8	R	>8
<=32	S	<=2	S	2	I	>4	R	8	R	8
<=32	S	>8	R	>8	R	>4	R	>8	R	<=4
<=32	S	>8	R	>8	R	>4	R	>8	R	>8
<=32	S	>8	R	<=1	S	4	I	<=1	S	<=4
<=32	S	<=2	S	<=1	S	>4	R	<=1	S	8
<=32	S	>8	R	8	R	4	I	<=1	S	8
64	R	>8	R	<=1	S	<=1	S	<=1	S	<=4
<=32	S	>8	R	<=1	S	>4	R	<=1	S	<=4
<=32	S	>8	R	<=1	S	2	S	<=1	S	<=4
<=32	S	<=2	S	<=1	S	>4	R	>8	R	>8
<=32	S	<=2	S	<=1	S	>4	R	<=1	S	<=4
<=32	S	<=2	S	<=1	S	>4	R	<=1	S	8
<=32	S	>8	R	8	R	<=1	S	>8	R	<=4
<=32	S	>8	R	<=1	S	<=1	S	2	I	<=4
<=32	S	4	S	<=1	S	<=1	S	4	R	<=4
<=32	S	>8	R	>8	R	>4	R	>8	R	<=4
<=32	S	>8	R	2	I	>4	R	>8	R	<=4

<=32	S	<=2	S	<=1	S	<=1	S	<=1	S	<=4
<=32	S	>8	R	<=1	S	>4	R	<=1	S	8
<=32	S	>8	R	<=1	S	>4	R	<=1	S	>8
<=32	S	>8	R	<=1	S	>4	R	<=1	S	8
<=32	S	>8	R	>8	R	>4	R	>8	R	<=4
<=32	S	>8	R	<=1	S	>4	R	<=1	S	<=4
<=32	S	>8	R	>8	R	>4	R	>8	R	<=4
64	R	>8	R	<=1	S	<=1	S	<=1	S	>8
<=32	S	>8	R	8	R	<=1	S	>8	R	<=4
<=32	S	>8	R	2	I	>4	R	8	R	<=4
<=32	S	<=2	S	4	R	>4	R	<=1	S	<=4
<=32	S	>8	R	<=1	S	<=1	S	<=1	S	<=4
<=32	S	<=2	S	<=1	S	<=1	S	<=1	S	<=4
64	R	>8	R	<=1	S	<=1	S	<=1	S	>8
<=32	S	>8	R	<=1	S	>4	R	<=1	S	<=4
<=32	S	>8	R	2	I	<=1	S	<=1	S	<=4
64	R	>8	R	>8	R	>4	R	>8	R	8
<=32	S	<=2	S	<=1	S	>4	R	8	R	<=4
<=32	S	>8	R	2	I	>4	R	>8	R	<=4
<=32	S	>8	R	<=1	S	>4	R	>8	R	<=4
<=32	S	<=2	S	<=1	S	>4	R	<=1	S	8
<=32	S	>8	R	8	R	4	I	2	I	<=4
<=32	S	>8	R	>8	R	2	S	>8	R	<=4
64	R	>8	R	>8	R	>4	R	>8	R	8
<=32	S	>8	R	>8	R	2	S	>8	R	8
<=32	S	>8	R	8	R	<=1	S	>8	R	<=4
<=32	S	>8	R	>8	R	>4	R	>8	R	<=4
<=32	S	<=2	S	<=1	S	<=1	S	<=1	S	<=4
<=32	S	4	S	2	I	>4	R	<=1	S	<=4
<=32	S	<=2	S	<=1	S	>4	R	<=1	S	>8
<=32	S	>8	R	4	R	>4	R	8	R	8
<=32	S	>8	R	<=1	S	>4	R	<=1	S	<=4
<=32	S	>8	R	>8	R	2	S	>8	R	8
64	R	>8	R	>8	R	>4	R	>8	R	8
<=32	S	>8	R	>8	R	2	S	>8	R	8
<=32	S	>8	R	<=1	S	<=1	S	<=1	S	<=4
<=32	S	>8	R	>8	R	4	I	>8	R	<=4
>64	R	>8	R	4	R	>4	R	>8	R	8
<=32	S	>8	R	8	R	<=1	S	>8	R	8
<=32	S	4	S	<=1	S	>4	R	<=1	S	<=4
64	R	>8	R	<=1	S	>4	R	2	I	8
<=32	S	8	I	<=1	S	>4	R	<=1	S	<=4
<=32	S	>8	R	2	I	>4	R	>8	R	<=4
<=32	S	>8	R	2	I	>4	R	8	R	8
64	R	>8	R	>8	R	>4	R	>8	R	8
64	R	>8	R	>8	R	>4	R	>8	R	8
<=32	S	>8	R	4	R	<=1	S	8	R	<=4
<=32	S	>8	R	>8	R	>4	R	>8	R	>8
<=32	S	<=2	S	4	R	>4	R	>8	R	<=4
<=32	S	>8	R	2	I	<=1	S	<=1	S	8
<=32	S	>8	R	2	I	>4	R	>8	R	<=4
<=32	S	4	S	>8	R	4	I	>8	R	>8
<=32	S	<=2	S	<=1	S	<=1	S	<=1	S	<=4
<=32	S	>8	R	>8	R	>4	R	>8	R	>8
<=32	S	<=2	S	2	I	>4	R	8	R	>8

<=32	S	>8	R	>8	R	>4	R	>8	R	>8
<=32	S	8	I	4	R	>4	R	>8	R	8
<=32	S	<=2	S	2	I	>4	R	8	R	<=4
<=32	S	>8	R	>8	R	>4	R	>8	R	8
<=32	S	<=2	S	2	I	>4	R	8	R	<=4
64	R	<=2	S	<=1	S	<=1	S	<=1	S	<=4
<=32	S	>8	R	<=1	S	<=1	S	<=1	S	<=4
<=32	S	>8	R	<=1	S	2	S	<=1	S	<=4
<=32	S	<=2	S	2	I	<=1	S	<=1	S	<=4
<=32	S	>8	R	<=1	S	4	I	2	I	8
<=32	S	<=2	S	<=1	S	4	I	<=1	S	<=4
<=32	S	>8	R	>8	R	>4	R	>8	R	>8
64	R	>8	R	<=1	S	<=1	S	<=1	S	<=4
<=32	S	<=2	S	<=1	S	>4	R	8	R	<=4
<=32	S	>8	R	<=1	S	<=1	S	<=1	S	<=4
<=32	S	<=2	S	<=1	S	>4	R	<=1	S	<=4
<=32	S	<=2	S	2	I	>4	R	>8	R	>8
<=32	S	<=2	S	2	I	>4	R	8	R	>8
<=32	S	>8	R	2	I	<=1	S	2	I	<=4
<=32	S	<=2	S	<=1	S	>4	R	<=1	S	<=4
<=32	S	>8	R	<=1	S	<=1	S	2	I	<=4
<=32	S	>8	R	8	R	4	I	<=1	S	8
<=32	S	<=2	S	<=1	S	>4	R	<=1	S	>8
<=32	S	<=2	S	2	I	>4	R	>8	R	<=4
<=32	S	<=2	S	<=1	S	<=1	S	<=1	S	<=4
<=32	S	4	S	4	R	<=1	S	<=1	S	>8
<=32	S	<=2	S	<=1	S	<=1	S	<=1	S	<=4
<=32	S	>8	R	<=1	S	>4	R	<=1	S	<=4
<=32	S	>8	R	8	R	2	S	>8	R	<=4
64	R	>8	R	>8	R	4	I	>8	R	<=4
64	R	>8	R	<=1	S	<=1	S	<=1	S	<=4
<=32	S	>8	R	<=1	S	<=1	S	<=1	S	>8
<=32	S	<=2	S	<=1	S	<=1	S	<=1	S	<=4
>64	R	>8	R	2	I	4	I	4	R	<=4
<=32	S	>8	R	<=1	S	>4	R	<=1	S	>8
<=32	S	>8	R	>8	R	<=1	S	>8	R	<=4
<=32	S	>8	R	<=1	S	<=1	S	<=1	S	>8
<=32	S	4	S	<=1	S	>4	R	<=1	S	<=4
<=32	S	>8	R	<=1	S	<=1	S	<=1	S	<=4
>64	R	>8	R	<=1	S	<=1	S	<=1	S	<=4
<=32	S	<=2	S	<=1	S	>4	R	4	R	<=4
<=32	S	<=2	S	2	I	>4	R	8	R	8
<=32	S	<=2	S	>8	R	<=1	S	2	I	<=4
<=32	S	<=2	S	2	I	<=1	S	<=1	S	<=4
<=32	S	>8	R	>8	R	>4	R	>8	R	>8
64	R	<=2	S	2	I	<=1	S	<=1	S	<=4
<=32	S	>8	R	<=1	S	>4	R	<=1	S	<=4
<=32	S	>8	R	2	I	<=1	S	8	R	<=4
<=32	S	<=2	S	<=1	S	<=1	S	<=1	S	<=4
<=32	S	>8	R	8	R	<=1	S	<=1	S	<=4
<=32	S	>8	R	<=1	S	<=1	S	<=1	S	<=4
<=32	S	>8	R	8	R	<=1	S	8	R	<=4
<=32	S	<=2	S	2	I	>4	R	<=1	S	<=4
<=32	S	>8	R	2	I	>4	R	<=1	S	8
<=32	S	>8	R	4	R	4	I	4	R	8
<=32	S	>8	R	>8	R	<=1	S	>8	R	<=4

<=32	S	>8	R	<=1	S	<=1	S	<=1	S	<=4
<=32	S	>8	R	2	I	2	S	<=1	S	<=4
<=32	S	<=2	S	>8	R	<=1	S	>8	R	<=4
<=32	S	>8	R	2	I	>4	R	<=1	S	>8
<=32	S	>8	R	<=1	S	>4	R	<=1	S	<=4
<=32	S	<=2	S	4	R	>4	R	8	R	<=4
<=32	S	<=2	S	8	R	>4	R	8	R	<=4
<=32	S	>8	R	2	I	>4	R	<=1	S	8
<=32	S	>8	R	8	R	4	I	<=1	S	8
<=32	S	>8	R	2	I	>4	R	8	R	<=4
<=32	S	>8	R	<=1	S	<=1	S	<=1	S	<=4
<=32	S	>8	R	<=1	S	>4	R	<=1	S	<=4
<=32	S	>8	R	4	R	4	I	4	R	<=4
<=32	S	>8	R	<=1	S	>4	R	8	R	>8
<=32	S	>8	R	<=1	S	<=1	S	<=1	S	<=4
<=32	S	>8	R	>8	R	>4	R	>8	R	>8
<=32	S	>8	R	>8	R	>4	R	>8	R	8
<=32	S	>8	R	<=1	S	>4	R	4	R	<=4
<=32	S	<=2	S	<=1	S	<=1	S	<=1	S	<=4
<=32	S	>8	R	<=1	S	>4	R	8	R	8
<=32	S	>8	R	>8	R	>4	R	>8	R	>8
<=32	S	>8	R	>8	R	>4	R	>8	R	>8
<=32	S	>8	R	>8	R	>4	R	>8	R	<=4
<=32	S	>8	R	<=1	S	2	S	<=1	S	>8
<=32	S	>8	R	<=1	S	>4	R	8	R	<=4
<=32	S	<=2	S	<=1	S	<=1	S	<=1	S	<=4
<=32	S	<=2	S	<=1	S	>4	R	8	R	<=4
<=32	S	<=2	S	<=1	S	>4	R	>8	R	<=4
<=32	S	<=2	S	<=1	S	>4	R	<=1	S	<=4
<=32	S	>8	R	2	I	>4	R	>8	R	8
<=32	S	>8	R	<=1	S	2	S	<=1	S	<=4
<=32	S	>8	R	>8	R	>4	R	>8	R	<=4
<=32	S	>8	R	<=1	S	<=1	S	<=1	S	<=4
<=32	S	<=2	S	<=1	S	<=1	S	4	R	<=4
<=32	S	>8	R	>8	R	<=1	S	>8	R	<=4
<=32	S	>8	R	<=1	S	<=1	S	8	R	<=4
64	R	>8	R	<=1	S	<=1	S	<=1	S	<=4
<=32	S	>8	R	4	R	<=1	S	<=1	S	<=4
<=32	S	<=2	S	<=1	S	>4	R	8	R	8
<=32	S	>8	R	<=1	S	<=1	S	<=1	S	<=4
<=32	S	4	S	<=1	S	2	S	2	I	<=4
<=32	S	<=2	S	<=1	S	<=1	S	<=1	S	<=4
<=32	S	>8	R	<=1	S	>4	R	8	R	8
<=32	S	>8	R	<=1	S	>4	R	<=1	S	<=4
<=32	S	>8	R	>8	R	>4	R	>8	R	8
<=32	S	>8	R	<=1	S	<=1	S	8	R	<=4
<=32	S	>8	R	>8	R	2	S	>8	R	<=4
<=32	S	>8	R	>8	R	2	S	>8	R	<=4
<=32	S	>8	R	>8	R	2	S	>8	R	<=4
<=32	S	>8	R	<=1	S	<=1	S	<=1	S	<=4
<=32	S	<=2	S	<=1	S	>4	R	<=1	S	<=4
64	R	>8	R	<=1	S	>4	R	<=1	S	>8
<=32	S	<=2	S	<=1	S	4	I	<=1	S	<=4
<=32	S	>8	R	<=1	S	<=1	S	<=1	S	<=4
64	R	>8	R	<=1	S	<=1	S	<=1	S	<=4
<=32	S	>8	R	>8	R	<=1	S	>8	R	<=4

<=32	S	<=2	S	2	I	>4	R	8	R	<=4
<=32	S	>8	R	8	R	<=1	S	>8	R	<=4
<=32	S	<=2	S	<=1	S	<=1	S	<=1	S	<=4
<=32	S	>8	R	<=1	S	>4	R	2	I	<=4
<=32	S	<=2	S	<=1	S	<=1	S	<=1	S	<=4
<=32	S	<=2	S	<=1	S	<=1	S	2	I	<=4
<=32	S	>8	R	>8	R	<=1	S	>8	R	8
<=32	S	>8	R	>8	R	>4	R	>8	R	>8
<=32	S	<=2	S	<=1	S	<=1	S	<=1	S	<=4
<=32	S	>8	R	<=1	S	>4	R	<=1	S	<=4
<=32	S	>8	R	<=1	S	<=1	S	<=1	S	<=4
<=32	S	<=2	S	2	I	>4	R	>8	R	<=4
<=32	S	>8	R	8	R	4	I	>8	R	<=4
<=32	S	<=2	S	2	I	>4	R	>8	R	<=4
<=32	S	<=2	S	>8	R	<=1	S	>8	R	<=4
<=32	S	<=2	S	<=1	S	<=1	S	<=1	S	<=4
64	R	>8	R	>8	R	4	I	8	R	8
<=32	S	>8	R	<=1	S	>4	R	<=1	S	>8
<=32	S	<=2	S	<=1	S	>4	R	<=1	S	>8
<=32	S	>8	R	<=1	S	<=1	S	<=1	S	<=4
<=32	S	<=2	S	<=1	S	>4	R	<=1	S	<=4
<=32	S	>8	R	<=1	S	<=1	S	<=1	S	<=4
64	R	>8	R	>8	R	>4	R	>8	R	>8
<=32	S	>8	R	<=1	S	<=1	S	<=1	S	>8
<=32	S	>8	R	<=1	S	2	S	<=1	S	<=4
<=32	S	<=2	S	<=1	S	>4	R	<=1	S	<=4
<=32	S	<=2	S	<=1	S	>4	R	8	R	<=4
<=32	S	<=2	S	<=1	S	>4	R	>8	R	8
64	R	<=2	S	<=1	S	<=1	S	<=1	S	<=4
<=32	S	>8	R	<=1	S	<=1	S	<=1	S	<=4
<=32	S	<=2	S	<=1	S	<=1	S	<=1	S	<=4
<=32	S	>8	R	<=1	S	<=1	S	<=1	S	<=4
<=32	S	>8	R	<=1	S	<=1	S	<=1	S	<=4
<=32	S	>8	R	>8	R	4	I	>8	R	<=4
<=32	S	>8	R	>8	R	4	I	>8	R	<=4
<=32	S	>8	R	2	I	4	I	4	R	<=4
<=32	S	<=2	S	<=1	S	<=1	S	<=1	S	>8
<=32	S	>8	R	4	R	<=1	S	8	R	<=4
<=32	S	<=2	S	<=1	S	>4	R	<=1	S	>8
<=32	S	>8	R	<=1	S	<=1	S	<=1	S	<=4
<=32	S	>8	R	<=1	S	<=1	S	<=1	S	8
<=32	S	<=2	S	<=1	S	>4	R	<=1	S	<=4
<=32	S	>8	R	<=1	S	<=1	S	<=1	S	<=4
<=32	S	>8	R	<=1	S	>4	R	<=1	S	<=4
64	R	>8	R	>8	R	<=1	S	>8	R	<=4
>64	R	>8	R	>8	R	<=1	S	>8	R	8
64	R	>8	R	>8	R	4	I	>8	R	<=4
<=32	S	8	I	2	I	<=1	S	8	R	<=4
<=32	S	>8	R	<=1	S	<=1	S	<=1	S	>8
>64	R	>8	R	<=1	S	<=1	S	<=1	S	<=4
>64	R	<=2	S	<=1	S	<=1	S	<=1	S	<=4
64	R	>8	R	>8	R	>4	R	>8	R	8
<=32	S	8	I	<=1	S	<=1	S	<=1	S	8
<=32	S	<=2	S	<=1	S	>4	R	<=1	S	8
<=32	S	<=2	S	<=1	S	<=1	S	<=1	S	<=4

<=32	S	<=2	S	<=1	S	>4	R	<=1	S	<=4
<=32	S	<=2	S	2	I	>4	R	>8	R	<=4
<=32	S	<=2	S	<=1	S	<=1	S	<=1	S	<=4
64	R	<=2	S	4	R	<=1	S	>8	R	<=4
<=32	S	>8	R	>8	R	>4	R	>8	R	<=4
<=32	S	<=2	S	2	I	>4	R	>8	R	<=4
<=32	S	>8	R	<=1	S	<=1	S	<=1	S	<=4
<=32	S	>8	R	<=1	S	<=1	S	<=1	S	<=4
<=32	S	4	S	2	I	>4	R	>8	R	<=4
>64	R	>8	R	>8	R	>4	R	>8	R	8
>64	R	>8	R	8	R	>4	R	>8	R	>8
<=32	S	>8	R	>8	R	>4	R	>8	R	<=4
64	R	<=2	S	4	R	>4	R	>8	R	<=4
<=32	S	>8	R	>8	R	>4	R	>8	R	>8
<=32	S	>8	R	<=1	S	<=1	S	<=1	S	<=4
<=32	S	<=2	S	<=1	S	>4	R	<=1	S	8
<=32	S	<=2	S	<=1	S	<=1	S	<=1	S	<=4
>64	R	>8	R	2	I	>4	R	>8	R	>8
>64	R	>8	R	<=1	S	>4	R	>8	R	>8
64	R	4	S	<=1	S	>4	R	8	R	<=4
64	R	>8	R	>8	R	>4	R	>8	R	8
<=32	S	<=2	S	2	I	<=1	S	4	R	<=4
>64	R	>8	R	>8	R	>4	R	>8	R	>8
<=32	S	>8	R	>8	R	<=1	S	4	R	8
<=32	S	>8	R	>8	R	>4	R	>8	R	8
>64	R	>8	R	>8	R	>4	R	>8	R	>8
64	R	>8	R	<=1	S	>4	R	>8	R	>8
>64	R	>8	R	>8	R	>4	R	8	R	8
<=32	S	<=2	S	>8	R	<=1	S	>8	R	<=4
<=32	S	<=2	S	>8	R	<=1	S	8	R	<=4
<=32	S	<=2	S	<=1	S	<=1	S	2	I	>8
<=32	S	<=2	S	<=1	S	>4	R	4	R	>8
<=32	S	<=2	S	<=1	S	>4	R	4	R	>8
<=32	S	<=2	S	<=1	S	>4	R	<=1	S	<=4
64	R	<=2	S	8	R	>4	R	8	R	<=4
<=32	S	<=2	S	<=1	S	>4	R	<=1	S	>8
<=32	S	>8	R	<=1	S	<=1	S	<=1	S	>8
<=32	S	>8	R	<=1	S	4	I	2	I	8
<=32	S	>8	R	<=1	S	>4	R	<=1	S	<=4
<=32	S	>8	R	<=1	S	>4	R	<=1	S	<=4
<=32	S	>8	R	<=1	S	>4	R	8	R	<=4
<=32	S	>8	R	<=1	S	>4	R	4	R	<=4
<=32	S	8	I	<=1	S	>4	R	8	R	<=4
<=32	S	>8	R	<=1	S	>4	R	4	R	8
<=32	S	>8	R	<=1	S	>4	R	2	I	8
>64	R	>8	R	<=1	S	>4	R	<=1	S	>8
<=32	S	>8	R	>8	R	4	I	>8	R	<=4
<=32	S	<=2	S	<=1	S	<=1	S	<=1	S	<=4
<=32	S	>8	R	<=1	S	>4	R	<=1	S	8
<=32	S	>8	R	<=1	S	>4	R	2	I	<=4
<=32	S	<=2	S	<=1	S	>4	R	<=1	S	<=4
<=32	S	>8	R	<=1	S	<=1	S	<=1	S	<=4
<=32	S	>8	R	4	R	<=1	S	8	R	<=4
<=32	S	<=2	S	<=1	S	>4	R	<=1	S	<=4
<=32	S	<=2	S	2	I	<=1	S	<=1	S	<=4
<=32	S	<=2	S	<=1	S	<=1	S	<=1	S	<=4

<=32	S	>8	R	2	I	<=1	S	<=1	S	<=4
<=32	S	<=2	S	2	I	>4	R	>8	R	<=4
64	R	>8	R	2	I	>4	R	8	R	<=4
<=32	S	<=2	S	2	I	>4	R	8	R	<=4
>64	R	>8	R	>8	R	>4	R	>8	R	<=4
<=32	S	<=2	S	<=1	S	<=1	S	<=1	S	<=4
<=32	S	4	S	<=1	S	<=1	S	<=1	S	8
<=32	S	>8	R	8	R	<=1	S	8	R	<=4
<=32	S	>8	R	<=1	S	>4	R	8	R	<=4
<=32	S	>8	R	>8	R	4	I	>8	R	8
<=32	S	>8	R	2	I	2	S	4	R	<=4
<=32	S	>8	R	8	R	2	S	>8	R	<=4
>64	R	<=2	S	>8	R	<=1	S	>8	R	<=4
<=32	S	<=2	S	<=1	S	>4	R	8	R	<=4
<=32	S	<=2	S	4	R	<=1	S	4	R	<=4
64	R	<=2	S	<=1	S	<=1	S	<=1	S	<=4
<=32	S	<=2	S	>8	R	>4	R	>8	R	>8
<=32	S	<=2	S	<=1	S	2	S	<=1	S	<=4
<=32	S	>8	R	8	R	4	I	>8	R	<=4
<=32	S	>8	R	<=1	S	>4	R	8	R	8
<=32	S	>8	R	<=1	S	<=1	S	<=1	S	<=4
<=32	S	>8	R	<=1	S	>4	R	8	R	<=4
<=32	S	>8	R	2	I	>4	R	8	R	<=4
64	R	<=2	S	<=1	S	<=1	S	<=1	S	<=4
<=32	S	4	S	2	I	>4	R	2	I	8
<=32	S	>8	R	8	R	<=1	S	4	R	<=4
<=32	S	>8	R	8	R	<=1	S	>8	R	<=4
<=32	S	<=2	S	2	I	>4	R	8	R	<=4
<=32	S	<=2	S	<=1	S	<=1	S	<=1	S	>8
<=32	S	<=2	S	<=1	S	2	S	<=1	S	<=4
<=32	S	>8	R	8	R	2	S	>8	R	<=4
<=32	S	<=2	S	<=1	S	<=1	S	<=1	S	>8
>64	R	>8	R	8	R	2	S	>8	R	<=4
<=32	S	<=2	S	<=1	S	<=1	S	<=1	S	<=4
<=32	S	<=2	S	<=1	S	<=1	S	<=1	S	8
<=32	S	>8	R	2	I	>4	R	>8	R	8
<=32	S	<=2	S	<=1	S	<=1	S	<=1	S	<=4
<=32	S	<=2	S	<=1	S	>4	R	<=1	S	<=4
<=32	S	>8	R	<=1	S	>4	R	<=1	S	8
<=32	S	>8	R	4	R	2	S	8	R	<=4
<=32	S	>8	R	<=1	S	>4	R	8	R	<=4
<=32	S	>8	R	4	R	>4	R	4	R	>8
<=32	S	>8	R	2	I	>4	R	>8	R	8
<=32	S	>8	R	<=1	S	4	I	<=1	S	>8
<=32	S	>8	R	<=1	S	<=1	S	<=1	S	<=4
<=32	S	>8	R	8	R	4	I	>8	R	<=4
<=32	S	<=2	S	<=1	S	<=1	S	<=1	S	<=4
<=32	S	<=2	S	<=1	S	<=1	S	<=1	S	<=4
<=32	S	<=2	S	<=1	S	<=1	S	<=1	S	>8
<=32	S	>8	R	>8	R	4	I	>8	R	<=4
<=32	S	>8	R	8	R	2	S	>8	R	<=4
<=32	S	<=2	S	<=1	S	>4	R	<=1	S	<=4
<=32	S	<=2	S	2	I	>4	R	>8	R	<=4
<=32	S	>8	R	<=1	S	>4	R	8	R	<=4
<=32	S	>8	R	<=1	S	<=1	S	<=1	S	<=4
<=32	S	>8	R	<=1	S	<=1	S	<=1	S	<=4

<=32	S	>8	R	<=1	S	>4	R	8	R	8
<=32	S	>8	R	8	R	>4	R	4	R	<=4
<=32	S	>8	R	>8	R	>4	R	>8	R	>8
<=32	S	>8	R	8	R	2	S	>8	R	<=4
<=32	S	>8	R	4	R	>4	R	>8	R	>8
<=32	S	>8	R	2	I	>4	R	8	R	<=4
<=32	S	>8	R	8	R	2	S	>8	R	<=4
<=32	S	>8	R	>8	R	<=1	S	>8	R	<=4
<=32	S	>8	R	<=1	S	<=1	S	<=1	S	<=4
<=32	S	>8	R	>8	R	>4	R	>8	R	>8
64	R	<=2	S	<=1	S	4	I	<=1	S	8
<=32	S	>8	R	<=1	S	4	I	<=1	S	<=4
<=32	S	>8	R	<=1	S	<=1	S	<=1	S	<=4
<=32	S	>8	R	>8	R	4	I	>8	R	<=4
<=32	S	<=2	S	2	I	>4	R	8	R	<=4
<=32	S	>8	R	4	R	<=1	S	<=1	S	<=4
<=32	S	>8	R	8	R	2	S	>8	R	<=4
64	R	>8	R	>8	R	>4	R	>8	R	8
64	R	>8	R	>8	R	>4	R	>8	R	>8
64	R	>8	R	>8	R	>4	R	>8	R	>8
<=32	S	<=2	S	<=1	S	<=1	S	<=1	S	<=4
<=32	S	>8	R	2	I	>4	R	<=1	S	>8
<=32	S	>8	R	<=1	S	<=1	S	<=1	S	<=4

<=32	S	>8	R	>8	R	2	S	>8	R	8	
<=32	S	<=2	S	<=1	S	>4	R	8	R	<=4	

<=32	S	>8	R	<=1	S	<=1	S	<=1	S	<=4		
<=32	S	>8	R	8	R	4	I	>8	R	<=4		
<=32	S	>8	R	2	I	>4	R	>8	R	8		
<=32	S	<=2	S	4	R	>4	R	8	R	<=4		

<=32	S	>8	R	<=1	S	>4	R	<=1	S	>8
<=32	S	<=2	S	<=1	S	>4	R	2	I	<=4
<=32	S	>8	R	4	R	>4	R	>8	R	>8
<=32	S	>8	R	2	I	>4	R	>8	R	>8
64	R	<=2	S	>8	R	>4	R	>8	R	8
<=32	S	>8	R	8	R	4	I	>8	R	<=4
<=32	S	<=2	S	2	I	>4	R	>8	R	<=4
<=32	S	>8	R	<=1	S	4	I	<=1	S	<=4
<=32	S	<=2	S	>8	R	>4	R	>8	R	>8
64	R	>8	R	>8	R	4	I	>8	R	8
<=32	S	>8	R	8	R	2	S	<=1	S	<=4
<=32	S	>8	R	8	R	>4	R	8	R	>8
<=32	S	>8	R	8	R	>4	R	>8	R	>8
64	R	>8	R	>8	R	2	S	>8	R	<=4
<=32	S	8	I	2	I	>4	R	8	R	<=4
<=32	S	>8	R	<=1	S	>4	R	8	R	<=4
<=32	S	8	I	2	I	>4	R	2	I	<=4
64	R	>8	R	>8	R	>4	R	>8	R	<=4

>64	R	>8	R	>8	R	<=1	S	>8	R	<=4

<=32	S	>8	R	<=1	S	<=1	S	<=1	S	<=4
<=32	S	<=2	S	<=1	S	<=1	S	<=1	S	<=4
<=32	S	>8	R	8	R	2	S	>8	R	<=4
<=32	S	>8	R	2	I	<=1	S	<=1	S	8
<=32	S	>8	R	<=1	S	2	S	<=1	S	<=4
<=32	S	>8	R	4	R	2	S	<=1	S	<=4
64	R	>8	R	4	R	2	S	8	R	<=4
<=32	S	>8	R	<=1	S	<=1	S	<=1	S	<=4
<=32	S	>8	R	8	R	2	S	>8	R	<=4
<=32	S	8	I	<=1	S	>4	R	2	I	<=4
<=32	S	>8	R	<=1	S	>4	R	<=1	S	>8
<=32	S	>8	R	>8	R	2	S	>8	R	<=4
<=32	S	>8	R	2	I	>4	R	8	R	<=4
<=32	S	>8	R	2	I	4	I	8	R	<=4
>64	R	<=2	S	<=1	S	4	I	<=1	S	<=4
<=32	S	>8	R	<=1	S	<=1	S	<=1	S	<=4
<=32	S	>8	R	4	R	<=1	S	>8	R	<=4
<=32	S	>8	R	<=1	S	>4	R	<=1	S	<=4
>64	R	<=2	S	<=1	S	2	S	<=1	S	<=4
<=32	S	<=2	S	<=1	S	<=1	S	<=1	S	<=4
64	R	>8	R	>8	R	2	S	>8	R	<=4

<=32	S	>8	R	>8	R	>4	R	>8	R	<=4
<=32	S	>8	R	8	R	<=1	S	8	R	<=4
<=32	S	<=2	S	4	R	<=1	S	<=1	S	<=4
<=32	S	>8	R	>8	R	>4	R	>8	R	>8
<=32	S	>8	R	<=1	S	<=1	S	<=1	S	8
<=32	S	>8	R	<=1	S	<=1	S	<=1	S	<=4
<=32	S	>8	R	<=1	S	<=1	S	<=1	S	<=4
64	R	>8	R	<=1	S	<=1	S	8	R	<=4
<=32	S	>8	R	4	R	2	S	>8	R	<=4
<=32	S	>8	R	2	I	>4	R	>8	R	8
<=32	S	<=2	S	<=1	S	<=1	S	<=1	S	<=4
<=32	S	<=2	S	<=1	S	>4	R	<=1	S	<=4
<=32	S	<=2	S	<=1	S	<=1	S	<=1	S	<=4
<=32	S	<=2	S	<=1	S	<=1	S	<=1	S	<=4
<=32	S	>8	R	4	R	2	S	>8	R	<=4
<=32	S	<=2	S	<=1	S	<=1	S	<=1	S	<=4
64	R	<=2	S	<=1	S	4	I	<=1	S	<=4
<=32	S	<=2	S	<=1	S	<=1	S	<=1	S	<=4
<=32	S	<=2	S	<=1	S	<=1	S	<=1	S	<=4
<=32	S	<=2	S	<=1	S	<=1	S	<=1	S	<=4
<=32	S	>8	R	<=1	S	4	I	<=1	S	<=4
<=32	S	<=2	S	<=1	S	>4	R	8	R	8
<=32	S	>8	R	<=1	S	<=1	S	<=1	S	<=4
<=32	S	>8	R	<=1	S	<=1	S	<=1	S	<=4
<=32	S	>8	R	<=1	S	<=1	S	<=1	S	<=4
<=32	S	>8	R	4	R	>4	R	>8	R	<=4
<=32	S	8	I	4	R	>4	R	8	R	8
<=32	S	>8	R	<=1	S	<=1	S	<=1	S	<=4
<=32	S	>8	R	<=1	S	>4	R	<=1	S	>8
64	R	>8	R	>8	R	4	I	>8	R	8
<=32	S	>8	R	<=1	S	<=1	S	<=1	S	<=4
<=32	S	>8	R	<=1	S	>4	R	>8	R	>8
<=32	S	>8	R	<=1	S	>4	R	8	R	>8
<=32	S	>8	R	<=1	S	2	S	<=1	S	<=4
<=32	S	>8	R	<=1	S	<=1	S	<=1	S	<=4
<=32	S	<=2	S	<=1	S	<=1	S	<=1	S	<=4
<=32	S	>8	R	<=1	S	2	S	<=1	S	<=4
<=32	S	>8	R	<=1	S	<=1	S	<=1	S	<=4
<=32	S	>8	R	8	R	<=1	S	2	I	<=4
<=32	S	<=2	S	4	R	<=1	S	<=1	S	<=4
<=32	S	>8	R	<=1	S	<=1	S	<=1	S	<=4
<=32	S	>8	R	<=1	S	2	S	<=1	S	8
<=32	S	>8	R	<=1	S	>4	R	<=1	S	<=4
<=32	S	4	S	>8	R	>4	R	>8	R	<=4
<=32	S	>8	R	4	R	4	I	<=1	S	8
64	R	>8	R	>8	R	>4	R	>8	R	<=4
<=32	S	>8	R	2	I	>4	R	<=1	S	<=4
>64	R	>8	R	>8	R	4	I	>8	R	<=4
<=32	S	>8	R	>8	R	>4	R	>8	R	<=4
64	R	<=2	S	>8	R	>4	R	>8	R	>8
<=32	S	>8	R	8	R	4	I	>8	R	<=4
64	R	>8	R	<=1	S	<=1	S	<=1	S	<=4
<=32	S	<=2	S	4	R	>4	R	>8	R	<=4
<=32	S	>8	R	2	I	>4	R	<=1	S	<=4
<=32	S	>8	R	<=1	S	<=1	S	<=1	S	>8
<=32	S	<=2	S	<=1	S	>4	R	<=1	S	<=4

<=32	S	>8	R	<=1	S	>4	R	2	I	<=4
<=32	S	<=2	S	>8	R	<=1	S	>8	R	<=4
<=32	S	>8	R	<=1	S	<=1	S	<=1	S	<=4
<=32	S	>8	R	<=1	S	<=1	S	<=1	S	<=4
<=32	S	<=2	S	<=1	S	<=1	S	<=1	S	<=4
<=32	S	>8	R	8	R	>4	R	>8	R	<=4
<=32	S	>8	R	>8	R	>4	R	>8	R	8
<=32	S	>8	R	>8	R	4	I	>8	R	8
<=32	S	>8	R	<=1	S	4	I	2	I	>8
<=32	S	<=2	S	2	I	>4	R	8	R	<=4
<=32	S	>8	R	8	R	>4	R	>8	R	>8
<=32	S	<=2	S	2	I	4	I	4	R	<=4
<=32	S	<=2	S	<=1	S	2	S	<=1	S	<=4
<=32	S	<=2	S	2	I	4	I	2	I	8
<=32	S	>8	R	<=1	S	<=1	S	<=1	S	<=4
<=32	S	>8	R	<=1	S	<=1	S	<=1	S	<=4
<=32	S	>8	R	2	I	<=1	S	2	I	<=4
<=32	S	>8	R	<=1	S	<=1	S	<=1	S	<=4
<=32	S	>8	R	<=1	S	<=1	S	<=1	S	<=4
<=32	S	>8	R	<=1	S	4	I	<=1	S	8
<=32	S	8	I	<=1	S	2	S	2	I	>8
<=32	S	<=2	S	<=1	S	<=1	S	<=1	S	>8
<=32	S	>8	R	<=1	S	>4	R	<=1	S	8
<=32	S	>8	R	>8	R	2	S	>8	R	<=4
<=32	S	>8	R	>8	R	>4	R	>8	R	>8
<=32	S	>8	R	<=1	S	>4	R	8	R	<=4
<=32	S	>8	R	8	R	4	I	>8	R	<=4
<=32	S	>8	R	<=1	S	>4	R	4	R	>8
<=32	S	>8	R	<=1	S	>4	R	<=1	S	>8
<=32	S	>8	R	<=1	S	<=1	S	<=1	S	<=4
>64	R	>8	R	>8	R	>4	R	>8	R	>8
64	R	>8	R	8	R	>4	R	>8	R	<=4
64	R	>8	R	8	R	>4	R	>8	R	8
>64	R	>8	R	8	R	>4	R	8	R	>8
64	R	>8	R	2	I	<=1	S	8	R	<=4
64	R	>8	R	8	R	>4	R	>8	R	>8
64	R	>8	R	8	R	>4	R	>8	R	>8
64	R	>8	R	8	R	4	I	>8	R	>8
64	R	>8	R	4	R	4	I	>8	R	<=4
64	R	>8	R	4	R	2	S	>8	R	<=4
>64	R	>8	R	>8	R	>4	R	>8	R	>8
<=32	S	>8	R	4	R	<=1	S	4	R	<=4
64	R	>8	R	4	R	>4	R	<=1	S	<=4
<=32	S	4	S	>8	R	>4	R	>8	R	>8
<=32	S	>8	R	4	R	>4	R	8	R	>8
<=32	S	<=2	S	2	I	>4	R	8	R	>8
<=32	S	>8	R	>8	R	>4	R	>8	R	8
<=32	S	<=2	S	>8	R	<=1	S	>8	R	<=4
<=32	S	>8	R	4	R	<=1	S	8	R	<=4
<=32	S	>8	R	4	R	2	S	2	I	8
<=32	S	>8	R	<=1	S	>4	R	2	I	<=4
<=32	S	>8	R	<=1	S	>4	R	<=1	S	>8
<=32	S	<=2	S	<=1	S	>4	R	<=1	S	<=4
<=32	S	>8	R	>8	R	>4	R	>8	R	<=4
<=32	S	4	S	<=1	S	>4	R	<=1	S	<=4

<=32	S	>8	R	<=1	S	>4	R	<=1	S	<=4
<=32	S	>8	R	<=1	S	4	I	<=1	S	8
>64	R	>8	R	<=1	S	2	S	<=1	S	<=4
<=32	S	>8	R	<=1	S	>4	R	<=1	S	<=4
64	R	<=2	S	<=1	S	<=1	S	<=1	S	<=4
>64	R	>8	R	8	R	2	S	>8	R	<=4
64	R	>8	R	4	R	<=1	S	8	R	>8
<=32	S	<=2	S	>8	R	>4	R	8	R	<=4
<=32	S	>8	R	4	R	4	I	>8	R	<=4
>64	R	>8	R	<=1	S	<=1	S	<=1	S	<=4
<=32	S	<=2	S	2	I	<=1	S	<=1	S	<=4
>64	R	>8	R	4	R	<=1	S	4	R	<=4
<=32	S	>8	R	<=1	S	<=1	S	<=1	S	<=4
64	R	>8	R	>8	R	>4	R	>8	R	8
<=32	S	>8	R	>8	R	<=1	S	>8	R	<=4
64	R	<=2	S	>8	R	>4	R	>8	R	8
<=32	S	>8	R	>8	R	<=1	S	8	R	<=4
<=32	S	<=2	S	8	R	>4	R	>8	R	<=4
<=32	S	<=2	S	>8	R	>4	R	>8	R	<=4
64	R	<=2	S	>8	R	<=1	S	>8	R	<=4
<=32	S	<=2	S	2	I	>4	R	8	R	8
<=32	S	<=2	S	>8	R	2	S	>8	R	<=4
<=32	S	<=2	S	8	R	>4	R	>8	R	<=4
<=32	S	>8	R	<=1	S	>4	R	>8	R	8
<=32	S	>8	R	<=1	S	<=1	S	<=1	S	<=4
64	R	>8	R	<=1	S	<=1	S	<=1	S	<=4
<=32	S	>8	R	2	I	<=1	S	<=1	S	<=4
<=32	S	>8	R	8	R	<=1	S	>8	R	<=4
<=32	S	<=2	S	<=1	S	<=1	S	<=1	S	<=4
<=32	S	>8	R	4	R	<=1	S	>8	R	<=4
<=32	S	>8	R	2	I	>4	R	>8	R	<=4
<=32	S	>8	R	>8	R	>4	R	>8	R	<=4
<=32	S	>8	R	2	I	<=1	S	8	R	<=4
<=32	S	<=2	S	<=1	S	>4	R	4	R	<=4
64	R	>8	R	<=1	S	4	I	<=1	S	<=4
<=32	S	<=2	S	<=1	S	<=1	S	<=1	S	<=4
<=32	S	>8	R	>8	R	>4	R	>8	R	<=4
<=32	S	>8	R	>8	R	>4	R	>8	R	<=4
<=32	S	<=2	S	2	I	>4	R	>8	R	<=4
<=32	S	8	I	<=1	S	>4	R	<=1	S	>8
<=32	S	>8	R	4	R	>4	R	>8	R	<=4
<=32	S	>8	R	2	I	>4	R	>8	R	>8
<=32	S	8	I	2	I	>4	R	>8	R	<=4
64	R	>8	R	<=1	S	>4	R	8	R	8
<=32	S	>8	R	<=1	S	<=1	S	<=1	S	<=4
<=32	S	<=2	S	2	I	>4	R	8	R	8
<=32	S	>8	R	4	R	<=1	S	8	R	<=4
<=32	S	>8	R	<=1	S	<=1	S	<=1	S	<=4
<=32	S	>8	R	<=1	S	2	S	<=1	S	<=4
<=32	S	<=2	S	<=1	S	<=1	S	2	I	>8
>64	R	<=2	S	8	R	2	S	>8	R	<=4
<=32	S	>8	R	2	I	>4	R	>8	R	<=4
<=32	S	>8	R	<=1	S	<=1	S	<=1	S	<=4
<=32	S	>8	R	<=1	S	<=1	S	<=1	S	<=4
<=32	S	<=2	S	<=1	S	>4	R	<=1	S	<=4
<=32	S	>8	R	<=1	S	>4	R	<=1	S	<=4

<=32	S	>8	R	>8	R	>4	R	>8	R	<=4
>64	R	>8	R	8	R	4	I	>8	R	8
<=32	S	>8	R	<=1	S	>4	R	<=1	S	<=4
<=32	S	>8	R	<=1	S	<=1	S	<=1	S	<=4

<=32	S	<=2	S	<=1	S	<=1	S	<=1	S	<=4
>64	R	<=2	S	2	I	>4	R	8	R	>8
<=32	S	>8	R	<=1	S	<=1	S	<=1	S	<=4
<=32	S	>8	R	2	I	>4	R	8	R	<=4
<=32	S	>8	R	2	I	>4	R	8	R	>8
<=32	S	<=2	S	>8	R	<=1	S	>8	R	8
<=32	S	>8	R	<=1	S	4	I	<=1	S	8
<=32	S	>8	R	4	R	<=1	S	8	R	<=4
<=32	S	>8	R	2	I	<=1	S	>8	R	<=4
<=32	S	<=2	S	<=1	S	<=1	S	<=1	S	<=4
<=32	S	8	I	<=1	S	>4	R	4	R	>8
<=32	S	<=2	S	>8	R	<=1	S	>8	R	<=4
<=32	S	8	I	>8	R	>4	R	4	R	>8
<=32	S	>8	R	8	R	4	I	<=1	S	8
<=32	S	<=2	S	>8	R	>4	R	8	R	<=4
<=32	S	>8	R	>8	R	>4	R	>8	R	8
>64	R	<=2	S	>8	R	<=1	S	>8	R	<=4
<=32	S	>8	R	>8	R	>4	R	>8	R	8
<=32	S	>8	R	2	I	>4	R	8	R	<=4
<=32	S	>8	R	<=1	S	<=1	S	<=1	S	<=4
>64	R	<=2	S	<=1	S	<=1	S	<=1	S	<=4
<=32	S	>8	R	>8	R	2	S	>8	R	>8
<=32	S	>8	R	>8	R	>4	R	>8	R	>8
<=32	S	>8	R	2	I	>4	R	>8	R	<=4
<=32	S	>8	R	<=1	S	>4	R	8	R	8
<=32	S	<=2	S	<=1	S	>4	R	<=1	S	<=4
64	R	>8	R	>8	R	>4	R	8	R	<=4
<=32	S	4	S	<=1	S	>4	R	<=1	S	>8
>64	R	>8	R	>8	R	>4	R	<=1	S	>8
<=32	S	>8	R	2	I	<=1	S	2	I	>8
<=32	S	<=2	S	<=1	S	<=1	S	<=1	S	<=4
64	R	>8	R	8	R	4	I	>8	R	<=4
<=32	S	>8	R	<=1	S	<=1	S	4	R	<=4

<=32	S	<=2	S	2	I	<=1	S	2	I	<=4
<=32	S	>8	R	8	R	>4	R	>8	R	>8
<=32	S	>8	R	8	R	>4	R	>8	R	8
<=32	S	>8	R	>8	R	>4	R	>8	R	<=4
<=32	S	>8	R	2	I	<=1	S	8	R	<=4
<=32	S	<=2	S	<=1	S	>4	R	8	R	<=4
<=32	S	<=2	S	<=1	S	>4	R	4	R	<=4
64	R	>8	R	>8	R	>4	R	>8	R	>8
<=32	S	>8	R	<=1	S	>4	R	<=1	S	<=4
<=32	S	>8	R	<=1	S	>4	R	4	R	<=4
<=32	S	>8	R	8	R	>4	R	>8	R	8
<=32	S	>8	R	8	R	4	I	>8	R	>8
<=32	S	<=2	S	8	R	2	S	>8	R	<=4
>64	R	<=2	S	2	I	2	S	4	R	>8
<=32	S	>8	R	<=1	S	<=1	S	<=1	S	<=4
>64	R	>8	R	8	R	<=1	S	2	I	<=4
<=32	S	>8	R	4	R	<=1	S	<=1	S	<=4
<=32	S	>8	R	<=1	S	4	I	<=1	S	<=4
<=32	S	>8	R	<=1	S	<=1	S	8	R	<=4
<=32	S	>8	R	>8	R	>4	R	>8	R	>8
<=32	S	>8	R	<=1	S	4	I	<=1	S	8
<=32	S	>8	R	<=1	S	<=1	S	<=1	S	<=4
<=32	S	>8	R	<=1	S	>4	R	8	R	<=4
64	R	>8	R	>8	R	>4	R	>8	R	8
<=32	S	4	S	<=1	S	<=1	S	<=1	S	<=4
<=32	S	>8	R	8	R	2	S	>8	R	>8
>64	R	>8	R	>8	R	4	I	>8	R	<=4
>64	R	>8	R	>8	R	4	I	>8	R	>8
<=32	S	>8	R	>8	R	>4	R	>8	R	<=4
<=32	S	>8	R	<=1	S	<=1	S	<=1	S	<=4
<=32	S	>8	R	<=1	S	<=1	S	<=1	S	>8
<=32	S	>8	R	<=1	S	<=1	S	<=1	S	<=4
<=32	S	>8	R	>8	R	<=1	S	>8	R	<=4
<=32	S	>8	R	<=1	S	>4	R	<=1	S	>8
64	R	>8	R	4	R	2	S	8	R	<=4
>64	R	>8	R	>8	R	>4	R	8	R	8
<=32	S	>8	R	<=1	S	4	I	4	R	<=4
<=32	S	>8	R	2	I	4	I	2	I	<=4
<=32	S	>8	R	<=1	S	>4	R	<=1	S	8
<=32	S	>8	R	<=1	S	4	I	<=1	S	<=4
<=32	S	>8	R	<=1	S	>4	R	8	R	8
<=32	S	>8	R	4	R	4	I	2	I	8
<=32	S	<=2	S	<=1	S	>4	R	<=1	S	8
64	R	>8	R	<=1	S	>4	R	2	I	8
>64	R	>8	R	>8	R	4	I	4	R	<=4
<=32	S	>8	R	<=1	S	<=1	S	<=1	S	>8
<=32	S	<=2	S	4	R	>4	R	8	R	<=4
<=32	S	>8	R	4	R	<=1	S	>8	R	8
<=32	S	4	S	<=1	S	>4	R	<=1	S	<=4
<=32	S	<=2	S	<=1	S	>4	R	<=1	S	<=4
<=32	S	<=2	S	<=1	S	>4	R	<=1	S	<=4
64	R	8	I	<=1	S	>4	R	<=1	S	<=4
<=32	S	>8	R	>8	R	>4	R	>8	R	<=4
<=32	S	>8	R	<=1	S	2	S	<=1	S	<=4
64	R	<=2	S	4	R	<=1	S	<=1	S	<=4
<=32	S	>8	R	4	R	4	I	>8	R	<=4

<=32	S	>8	R	<=1	S	>4	R	8	R	<=4
<=32	S	>8	R	2	I	<=1	S	>8	R	<=4
<=32	S	>8	R	2	I	<=1	S	>8	R	<=4
<=32	S	>8	R	>8	R	>4	R	>8	R	8
<=32	S	>8	R	2	I	>4	R	8	R	<=4
<=32	S	>8	R	<=1	S	<=1	S	4	R	<=4
<=32	S	>8	R	4	R	>4	R	8	R	>8
>64	R	>8	R	<=1	S	<=1	S	2	I	<=4
<=32	S	<=2	S	>8	R	<=1	S	>8	R	<=4
>64	R	>8	R	>8	R	<=1	S	>8	R	<=4
<=32	S	>8	R	<=1	S	<=1	S	<=1	S	<=4
<=32	S	>8	R	8	R	<=1	S	>8	R	<=4
<=32	S	>8	R	2	I	>4	R	8	R	<=4
<=32	S	>8	R	<=1	S	<=1	S	<=1	S	<=4
64	R	>8	R	8	R	<=1	S	4	R	>8
64	R	>8	R	>8	R	<=1	S	>8	R	<=4
<=32	S	>8	R	<=1	S	<=1	S	<=1	S	<=4
<=32	S	>8	R	2	I	<=1	S	>8	R	<=4
<=32	S	<=2	S	<=1	S	<=1	S	<=1	S	<=4
<=32	S	<=2	S	<=1	S	<=1	S	<=1	S	<=4
<=32	S	>8	R	2	I	<=1	S	>8	R	<=4
<=32	S	>8	R	<=1	S	<=1	S	<=1	S	<=4
>64	R	>8	R	>8	R	<=1	S	>8	R	<=4
<=32	S	>8	R	8	R	<=1	S	>8	R	<=4
<=32	S	>8	R	8	R	>4	R	>8	R	>8
<=32	S	>8	R	2	I	>4	R	>8	R	<=4
<=32	S	>8	R	8	R	>4	R	>8	R	>8
<=32	S	<=2	S	<=1	S	>4	R	<=1	S	<=4
<=32	S	>8	R	<=1	S	<=1	S	<=1	S	<=4
<=32	S	>8	R	<=1	S	<=1	S	<=1	S	<=4
<=32	S	>8	R	4	R	4	I	>8	R	<=4
<=32	S	<=2	S	<=1	S	<=1	S	<=1	S	<=4
<=32	S	>8	R	2	I	>4	R	>8	R	<=4
<=32	S	>8	R	2	I	<=1	S	<=1	S	<=4
<=32	S	>8	R	2	I	>4	R	>8	R	<=4
<=32	S	>8	R	4	R	>4	R	>8	R	8
<=32	S	<=2	S	>8	R	<=1	S	>8	R	<=4
<=32	S	>8	R	>8	R	>4	R	>8	R	<=4
<=32	S	>8	R	>8	R	<=1	S	>8	R	>8
<=32	S	>8	R	<=1	S	<=1	S	8	R	<=4
<=32	S	>8	R	>8	R	2	S	>8	R	<=4
<=32	S	<=2	S	<=1	S	<=1	S	<=1	S	<=4
<=32	S	<=2	S	<=1	S	<=1	S	<=1	S	8
<=32	S	>8	R	>8	R	>4	R	>8	R	8
<=32	S	>8	R	>8	R	>4	R	>8	R	<=4
64	R	>8	R	<=1	S	<=1	S	<=1	S	<=4
64	R	>8	R	>8	R	<=1	S	>8	R	<=4
>64	R	>8	R	>8	R	>4	R	>8	R	8
<=32	S	>8	R	>8	R	>4	R	>8	R	<=4
<=32	S	>8	R	>8	R	>4	R	>8	R	<=4
<=32	S	>8	R	<=1	S	>4	R	4	R	<=4
<=32	S	>8	R	8	R	4	I	>8	R	8
<=32	S	<=2	S	<=1	S	<=1	S	<=1	S	<=4
<=32	S	>8	R	>8	R	>4	R	>8	R	>8
<=32	S	>8	R	8	R	>4	R	>8	R	>8
<=32	S	>8	R	>8	R	>4	R	>8	R	>8

<=32	S	>8	R	>8	R	>4	R	>8	R	>8
<=32	S	>8	R	<=1	S	4	I	<=1	S	<=4
<=32	S	<=2	S	>8	R	<=1	S	>8	R	<=4
<=32	S	>8	R	4	R	<=1	S	<=1	S	<=4
<=32	S	>8	R	4	R	>4	R	>8	R	>8
<=32	S	>8	R	>8	R	<=1	S	8	R	<=4
<=32	S	<=2	S	>8	R	<=1	S	>8	R	<=4
>64	R	>8	R	>8	R	>4	R	>8	R	>8
<=32	S	<=2	S	4	R	<=1	S	>8	R	<=4
<=32	S	>8	R	8	R	>4	R	>8	R	<=4
<=32	S	<=2	S	8	R	<=1	S	>8	R	<=4
<=32	S	>8	R	2	I	4	I	>8	R	<=4
<=32	S	>8	R	<=1	S	<=1	S	<=1	S	<=4
<=32	S	>8	R	>8	R	>4	R	>8	R	<=4
64	R	<=2	S	<=1	S	2	S	<=1	S	8
<=32	S	<=2	S	<=1	S	<=1	S	<=1	S	<=4
<=32	S	>8	R	8	R	>4	R	>8	R	>8
<=32	S	<=2	S	<=1	S	<=1	S	<=1	S	<=4
<=32	S	>8	R	>8	R	>4	R	>8	R	<=4
64	R	>8	R	8	R	<=1	S	>8	R	<=4
<=32	S	>8	R	2	I	<=1	S	8	R	<=4
<=32	S	<=2	S	<=1	S	>4	R	8	R	8
<=32	S	>8	R	>8	R	<=1	S	>8	R	<=4
>64	R	>8	R	<=1	S	>4	R	<=1	S	<=4
<=32	S	<=2	S	<=1	S	>4	R	2	I	<=4
<=32	S	<=2	S	8	R	<=1	S	<=1	S	<=4
<=32	S	>8	R	4	R	<=1	S	2	I	<=4
>64	R	>8	R	8	R	<=1	S	8	R	<=4
<=32	S	>8	R	>8	R	<=1	S	>8	R	<=4
<=32	S	<=2	S	<=1	S	<=1	S	2	I	>8
<=32	S	<=2	S	4	R	<=1	S	<=1	S	<=4
<=32	S	>8	R	>8	R	>4	R	>8	R	>8
<=32	S	>8	R	4	R	2	S	>8	R	<=4
<=32	S	<=2	S	<=1	S	<=1	S	<=1	S	>8
64	R	<=2	S	<=1	S	<=1	S	<=1	S	<=4
<=32	S	>8	R	2	I	>4	R	8	R	<=4
64	R	>8	R	<=1	S	<=1	S	<=1	S	<=4
>64	R	>8	R	>8	R	4	I	2	I	8
<=32	S	<=2	S	<=1	S	<=1	S	<=1	S	<=4
<=32	S	<=2	S	2	I	<=1	S	<=1	S	<=4
<=32	S	<=2	S	<=1	S	4	I	<=1	S	<=4
<=32	S	>8	R	<=1	S	<=1	S	<=1	S	<=4
<=32	S	>8	R	2	I	>4	R	>8	R	<=4
<=32	S	>8	R	<=1	S	>4	R	<=1	S	8
<=32	S	<=2	S	4	R	<=1	S	<=1	S	<=4
<=32	S	>8	R	>8	R	>4	R	>8	R	<=4
<=32	S	>8	R	<=1	S	<=1	S	<=1	S	<=4
<=32	S	>8	R	8	R	>4	R	>8	R	<=4
<=32	S	>8	R	>8	R	>4	R	>8	R	>8
<=32	S	>8	R	<=1	S	>4	R	<=1	S	<=4
<=32	S	>8	R	2	I	>4	R	8	R	<=4
<=32	S	>8	R	<=1	S	2	S	<=1	S	<=4
<=32	S	<=2	S	<=1	S	<=1	S	<=1	S	>8
<=32	S	>8	R	8	R	>4	R	>8	R	8
<=32	S	>8	R	8	R	>4	R	>8	R	<=4
<=32	S	>8	R	>8	R	>4	R	>8	R	>8

<=32	S	>8	R	>8	R	>4	R	>8	R	>8
<=32	S	>8	R	>8	R	>4	R	>8	R	>8
<=32	S	<=2	S	2	I	<=1	S	<=1	S	<=4
<=32	S	>8	R	4	R	2	S	>8	R	<=4
<=32	S	>8	R	2	I	>4	R	>8	R	8
<=32	S	<=2	S	<=1	S	<=1	S	<=1	S	<=4
<=32	S	<=2	S	<=1	S	<=1	S	<=1	S	<=4
<=32	S	8	I	<=1	S	<=1	S	<=1	S	<=4
<=32	S	4	S	8	R	>4	R	>8	R	<=4
<=32	S	>8	R	8	R	2	S	>8	R	<=4
<=32	S	>8	R	8	R	>4	R	2	I	<=4
<=32	S	<=2	S	<=1	S	<=1	S	<=1	S	<=4
<=32	S	<=2	S	2	I	>4	R	8	R	>8
<=32	S	>8	R	>8	R	>4	R	>8	R	>8
<=32	S	>8	R	>8	R	>4	R	>8	R	<=4
<=32	S	<=2	S	<=1	S	<=1	S	<=1	S	<=4
<=32	S	>8	R	>8	R	<=1	S	>8	R	<=4
<=32	S	>8	R	8	R	>4	R	>8	R	<=4
<=32	S	>8	R	>8	R	>4	R	>8	R	<=4
<=32	S	>8	R	<=1	S	<=1	S	<=1	S	
<=32	S	<=2	S	2	I	<=1	S	<=1	S	<=4
<=32	S	<=2	S	N/R		<=1	S	>8	R	8
<=32	S	>8	R	<=1	S	>4	R	8	R	<=4
<=32	S	>8	R	<=1	S	2	S	<=1	S	<=4
<=32	S	<=2	S	>8	R	>4	R	>8	R	>8
<=32	S	<=2	S	<=1	S	<=1	S	<=1	S	<=4
<=32	S	<=2	S	>8	R	<=1	S	>8	R	8
<=32	S	>8	R	>8	R	>4	R	>8	R	>8
<=32	S	>8	R	<=1	S	>4	R	8	R	<=4
64	R	>8	R	>8	R	>4	R	>8	R	<=4
<=32	S	<=2	S	<=1	S	>4	R	<=1	S	<=4
<=32	S	<=2	S	<=1	S	>4	R	<=1	S	<=4
<=32	S	<=2	S	<=1	S	<=1	S	<=1	S	<=4
<=32	S	>8	R	4	R	2	S	>8	R	<=4
<=32	S	<=2	S	2	I	2	S	2	I	<=4
<=32	S	>8	R	>8	R	>4	R	>8	R	>8
<=32	S	8	I	<=1	S	<=1	S	4	R	<=4
<=32	S	>8	R	<=1	S	<=1	S	<=1	S	<=4
<=32	S	>8	R	<=1	S	2	S	>8	R	<=4
<=32	S	>8	R	<=1	S	<=1	S	<=1	S	<=4
<=32	S	4	S	<=1	S	>4	R	8	R	8
<=32	S	<=2	S	N/R		<=1	S	>8	R	<=4
<=32	S	<=2	S	<=1	S	<=1	S	<=1	S	<=4
<=32	S	>8	R	8	R	>4	R	>8	R	<=4
<=32	S	>8	R	>8	R	2	S	>8	R	<=4
64	R	>8	R	<=1	S	>4	R	8	R	>8
<=32	S	>8	R	2	I	>4	R	>8	R	>8
<=32	S	>8	R	>8	R	>4	R	>8	R	<=4
<=32	S	>8	R	2	I	>4	R	>8	R	>8
<=32	S	>8	R	8	R	<=1	S	>8	R	<=4
<=32	S	>8	R	2	I	2	S	>8	R	<=4
<=32	S	<=2	S	<=1	S	<=1	S	<=1	S	<=4
<=32	S	>8	R	2	I	<=1	S	4	R	<=4
<=32	S	>8	R	8	R	<=1	S	>8	R	<=4
<=32	S	>8	R	>8	R	4	I	>8	R	<=4

<=32	S	4	S	4	R	>4	R	>8	R	<=4
<=32	S	>8	R	<=1	S	<=1	S	<=1	S	<=4
64	R	>8	R	>8	R	>4	R	>8	R	<=4
<=32	S	>8	R	>8	R	>4	R	>8	R	<=4
>64	R	>8	R	>8	R	>4	R	>8	R	<=4
<=32	S	>8	R	>8	R	>4	R	>8	R	>8
<=32	S	>8	R	4	R	>4	R	>8	R	>8
<=32	S	>8	R	>8	R	>4	R	>8	R	<=4
64	R	>8	R	>8	R	<=1	S	>8	R	<=4
<=32	S	>8	R	2	I	2	S	>8	R	<=4
<=32	S	>8	R	<=1	S	<=1	S	<=1	S	<=4
>64	R	>8	R	>8	R	2	S	>8	R	<=4
<=32	S	>8	R	<=1	S	<=1	S	<=1	S	<=4
<=32	S	>8	R	<=1	S	<=1	S	<=1	S	<=4
<=32	S	<=2	S	8	R	<=1	S	>8	R	<=4
<=32	S	<=2	S	N/R		<=1	S	>8	R	8
64	R	<=2	S	>8	R	<=1	S	>8	R	<=4
64	R	>8	R	<=1	S	>4	R	8	R	<=4
<=32	S	<=2	S	>8	R	<=1	S	>8	R	<=4
<=32	S	<=2	S	<=1	S	<=1	S	<=1	S	<=4
<=32	S	>8	R	2	I	4	I	>8	R	<=4
<=32	S	<=2	S	<=1	S	<=1	S	<=1	S	<=4
>64	R	4	S	<=1	S	4	I	<=1	S	<=4
<=32	S	<=2	S	<=1	S	>4	R	<=1	S	<=4
<=32	S	<=2	S	2	I	>4	R	8	R	<=4
<=32	S	>8	R	>8	R	>4	R	>8	R	>8
<=32	S	<=2	S	<=1	S	<=1	S	<=1	S	<=4

<=32	S	>8	R	>8	R	>4	R	>8	R	>8

<=32	S	8	I	>8	R	>4	R	>8	R	8	
<=32	S	>8	R	8	R	<=1	S	>8	R	<=4	

64	R	>8	R	>8	R	>4	R	>8	R	>8
<=32	S	>8	R	2	I	<=1	S	4	R	>8
<=32	S	>8	R	>8	R	>4	R	>8	R	8
<=32	S	>8	R	>8	R	4	I	>8	R	>8
>64	R	>8	R	>8	R	<=1	S	>8	R	>8
<=32	S	>8	R	2	I	>4	R	>8	R	>8
<=32	S	<=2	S	8	R	2	S	>8	R	<=4
64	R	>8	R	8	R	<=1	S	>8	R	<=4
>64	R	>8	R	<=1	S	<=1	S	2	I	<=4
>8	R	>8	R	>8	R	>4	R	>8	R	>8
<=32	S	>8	R	4	R	<=1	S	8	R	<=4
>64	R	>8	R	8	R	>4	R	>8	R	<=4
<=32	S	<=2	S	<=1	S	>4	R	<=1	S	>8
<=32	S	<=2	S	<=1	S	>4	R	<=1	S	<=4
>64	R	>8	R	2	I	>4	R	8	R	8
<=32	S	>8	R	<=1	S	<=1	S	<=1	S	<=4
<=32	S	<=2	S	>8	R	>4	R	>8	R	<=4
<=32	S	<=2	S	<=1	S	<=1	S	<=1	S	<=4
<=32	S	>8	R	8	R	4	I	>8	R	<=4

<=32	S	<=2	S	<=1	S	<=1	S	<=1	S	<=4
>64	R	>8	R	4	R	2	S	2	I	<=4
<=32	S	>8	R	>8	R	>4	R	>8	R	>8
<=32	S	>8	R	>8	R	>4	R	>8	R	>8
<=32	S	>8	R	2	I	<=1	S	8	R	<=4
<=32	S	>8	R	<=1	S	<=1	S	8	R	<=4
64	R	>8	R	2	I	2	S	8	R	8
<=32	S	<=2	S	<=1	S	2	S	<=1	S	<=4
<=32	S	>8	R	2	I	2	S	>8	R	<=4
>64	R	>8	R	<=1	S	>4	R	8	R	8
<=32	S	>8	R	>8	R	>4	R	>8	R	>8
<=32	S	>8	R	2	I	>4	R	8	R	<=4
<=32	S	>8	R	2	I	4	I	>8	R	<=4
<=32	S	>8	R	>8	R	>4	R	>8	R	8
<=32	S	<=2	S	8	R	>4	R	>8	R	<=4
<=32	S	<=2	S	>8	R	<=1	S	>8	R	<=4
<=32	S	<=2	S	2	I	>4	R	8	R	8
<=32	S	<=2	S	>8	R	<=1	S	>8	R	<=4
64	R	>8	R	>8	R	4	I	>8	R	>8
<=32	S	<=2	S	N/R		<=1	S	>8	R	8
<=32	S	>8	R	N/R		4	I	>8	R	>8
<=32	S	<=2	S	8	R	<=1	S	>8	R	<=4
<=32	S	<=2	S	N/R		<=1	S	8	R	8
<=32	S	<=2	S	8	R	<=1	S	>8	R	<=4
<=32	S	<=2	S	<=1	S	>4	R	<=1	S	<=4
<=32	S	>8	R	<=1	S	<=1	S	<=1	S	<=4
<=32	S	>8	R	>8	R	4	I	>8	R	8
<=32	S	>8	R	2	I	>4	R	4	R	<=4
64	R	>8	R	<=1	S	>4	R	<=1	S	>8
>64	R	>8	R	4	R	>4	R	8	R	8
64	R	>8	R	>8	R	>4	R	>8	R	8
<=32	S	>8	R	8	R	4	I	>8	R	8
<=32	S	>8	R	8	R	>4	R	>8	R	<=4
64	R	>8	R	>8	R	>4	R	>8	R	8
>64	R	>8	R	>8	R	>4	R	>8	R	<=4
<=32	S	>8	R	<=1	S	<=1	S	<=1	S	<=4
64	R	4	S	>8	R	4	I	>8	R	8
<=32	S	<=2	S	>8	R	<=1	S	>8	R	<=4
64	R	>8	R	>8	R	>4	R	>8	R	<=4
<=32	S	>8	R	<=1	S	>4	R	>8	R	8
<=32	S	>8	R	<=1	S	>4	R	8	R	8
<=32	S	>8	R	4	R	>4	R	4	R	>8
>64	R	>8	R	2	I	>4	R	8	R	8
<=32	S	>8	R	<=1	S	>4	R	8	R	8
<=32	S	>8	R	>8	R	>4	R	>8	R	8
64	R	>8	R	8	R	>4	R	>8	R	<=4
64	R	>8	R	8	R	>4	R	>8	R	<=4
<=32	S	>8	R	N/R		>4	R	>8	R	8
<=32	S	>8	R	8	R	>4	R	>8	R	>8
64	R	>8	R	>8	R	<=1	S	>8	R	<=4
64	R	>8	R	>8	R	>4	R	>8	R	<=4
<=32	S	>8	R	>8	R	>4	R	>8	R	<=4
<=32	S	>8	R	>8	R	>4	R	>8	R	8
>64	R	>8	R	<=1	S	<=1	S	<=1	S	<=4
>64	R	>8	R	<=1	S	>4	R	8	R	8
>64	R	>8	R	4	R	4	I	>8	R	<=4

<=32	S	>8	R	4	R	>4	R	8	R	<=4
64	R	>8	R	2	I	2	S	4	R	<=4
<=32	S	>8	R	2	I	>4	R	8	R	<=4
<=32	S	>8	R	2	I	>4	R	>8	R	<=4
<=32	S	>8	R	>8	R	>4	R	>8	R	<=4
<=32	S	>8	R	4	R	>4	R	<=1	S	<=4
<=32	S	>8	R	8	R	2	S	>8	R	<=4
<=32	S	>8	R	2	I	4	I	>8	R	<=4
<=32	S	>8	R	2	I	2	S	>8	R	<=4
<=32	S	>8	R	<=1	S	2	S	8	R	<=4
<=32	S	>8	R	>8	R	>4	R	>8	R	8
<=32	S	<=2	S	N/R		<=1	S	>8	R	<=4
<=32	S	<=2	S	<=1	S	2	S	<=1	S	<=4
<=32	S	<=2	S	<=1	S	<=1	S	<=1	S	<=4
<=32	S	<=2	S	N/R		<=1	S	>8	R	8
<=32	S	>8	R	8	R	>4	R	>8	R	<=4
<=32	S	>8	R	4	R	<=1	S	4	R	<=4
<=32	S	>8	R	2	I	>4	R	<=1	S	<=4
<=32	S	<=2	S	2	I	<=1	S	<=1	S	<=4
>64	R	>8	R	<=1	S	>4	R	8	R	8
<=32	S	>8	R	<=1	S	>4	R	4	R	8
<=32	S	<=2	S	2	I	<=1	S	<=1	S	<=4
64	R	>8	R	<=1	S	>4	R	<=1	S	<=4
64	R	>8	R	>8	R	>4	R	>8	R	<=4
<=32	S	>8	R	8	R	2	S	>8	R	<=4
<=32	S	<=2	S	>8	R	<=1	S	2	I	<=4
<=32	S	<=2	S	8	R	>4	R	2	I	<=4
<=32	S	>8	R	N/R		>4	R	>8	R	8
<=32	S	<=2	S	2	I	>4	R	2	I	<=4
<=32	S	>8	R	2	I	>4	R	4	R	<=4
64	R	>8	R	>8	R	>4	R	>8	R	8
<=32	S	<=2	S	N/R		<=1	S	>8	R	<=4
<=32	S	>8	R	<=1	S	4	I	<=1	S	<=4
<=32	S	>8	R	>8	R	>4	R	>8	R	8
<=32	S	>8	R	>8	R	>4	R	>8	R	<=4
<=32	S	>8	R	>8	R	>4	R	>8	R	>8
>64	R	>8	R	>8	R	>4	R	>8	R	8
<=32	S	4	S	2	I	>4	R	2	I	<=4
64	R	>8	R	4	R	>4	R	>8	R	<=4
<=32	S	>8	R	>8	R	>4	R	>8	R	<=4
<=32	S	>8	R	>8	R	>4	R	>8	R	>8
<=32	S	>8	R	<=1	S	<=1	S	<=1	S	<=4
<=32	S	>8	R	<=1	S	<=1	S	<=1	S	<=4
<=32	S	>8	R	8	R	>4	R	>8	R	>8
<=32	S	>8	R	4	R	<=1	S	>8	R	<=4
<=32	S	>8	R	2	I	>4	R	>8	R	<=4
<=32	S	<=2	S	<=1	S	>4	R	2	I	<=4
<=32	S	>8	R	<=1	S	<=1	S	<=1	S	<=4
<=32	S	<=2	S	<=1	S	<=1	S	<=1	S	<=4
<=32	S	<=2	S	8	R	<=1	S	>8	R	<=4
<=32	S	>8	R	>8	R	>4	R	>8	R	<=4
>64	R	>8	R	<=1	S	>4	R	8	R	8
<=32	S	>8	R	N/R		<=1	S	>8	R	8
<=32	S	>8	R	8	R	<=1	S	4	R	<=4
<=32	S	>8	R	2	I	>4	R	>8	R	<=4

<=32	S	>8	R	<=1	S	<=1	S	<=1	S	<=4
<=32	S	>8	R	<=1	S	<=1	S	<=1	S	<=4
<=32	S	>8	R	<=1	S	>4	R	4	R	8
<=32	S	>8	R	<=1	S	<=1	S	<=1	S	<=4
<=32	S	<=2	S	>8	R	<=1	S	>8	R	<=4
<=32	S	>8	R	8	R	<=1	S	>8	R	<=4
>64	R	>8	R	>8	R	<=1	S	>8	R	<=4
<=32	S	>8	R	<=1	S	4	I	8	R	<=4
<=32	S	>8	R	>8	R	<=1	S	>8	R	<=4
<=32	S	>8	R	>8	R	4	I	>8	R	8
<=32	S	<=2	S	<=1	S	<=1	S	<=1	S	<=4
<=32	S	>8	R	>8	R	>4	R	>8	R	<=4
<=32	S	>8	R	<=1	S	<=1	S	<=1	S	<=4
<=32	S	>8	R	>8	R	>4	R	>8	R	>8
>64	R	>8	R	<=1	S	>4	R	8	R	8
>64	R	>8	R	2	I	>4	R	8	R	8
>64	R	>8	R	8	R	>4	R	8	R	8
<=32	S	<=2	S	<=1	S	<=1	S	<=1	S	<=4
64	R	>8	R	<=1	S	<=1	S	<=1	S	<=4
64	R	>8	R	<=1	S	<=1	S	<=1	S	<=4
>64	R	>8	R	8	R	4	I	4	R	>8
>64	R	>8	R	2	I	2	S	2	I	>8
64	R	>8	R	4	R	<=1	S	8	R	<=4
<=32	S	>8	R	2	I	>4	R	8	R	<=4
<=32	S	>8	R	<=1	S	>4	R	>8	R	>8
<=32	S	<=2	S	<=1	S	2	S	<=1	S	<=4
>64	R	>8	R	>8	R	>4	R	>8	R	<=4
<=32	S	>8	R	<=1	S	<=1	S	<=1	S	<=4
<=32	S	>8	R	>8	R	>4	R	>8	R	<=4
<=32	S	>8	R	<=1	S	<=1	S	<=1	S	<=4
<=32	S	>8	R	<=1	S	>4	R	8	R	<=4
<=32	S	<=2	S	4	R	>4	R	8	R	>8
<=32	S	>8	R	2	I	<=1	S	2	I	<=4
<=32	S	>8	R	8	R	>4	R	>8	R	<=4
<=32	S	<=2	S	2	I	>4	R	>8	R	<=4
64	R	>8	R	2	I	>4	R	4	R	8
<=32	S	>8	R	>8	R	>4	R	>8	R	<=4
<=32	S	>8	R	>8	R	>4	R	>8	R	<=4
64	R	>8	R	>8	R	>4	R	>8	R	<=4
<=32	S	<=2	S	2	I	>4	R	8	R	<=4
>64	R	4	S	N/R		<=1	S	>8	R	8
<=32	S	>8	R	4	R	>4	R	<=1	S	<=4
<=32	S	>8	R	>8	R	>4	R	>8	R	>8
>64	R	>8	R	<=1	S	>4	R	8	R	<=4
<=32	S	<=2	S	<=1	S	2	S	<=1	S	8
<=32	S	>8	R	>8	R	<=1	S	>8	R	<=4
<=32	S	>8	R	4	R	<=1	S	8	R	<=4
<=32	S	>8	R	<=1	S	<=1	S	<=1	S	<=4
<=32	S	<=2	S	<=1	S	<=1	S	<=1	S	<=4
<=32	S	<=2	S	2	I	>4	R	2	I	<=4
64	R	>8	R	2	I	<=1	S	8	R	<=4
64	R	>8	R	2	I	<=1	S	8	R	<=4
<=32	S	>8	R	2	I	4	I	8	R	<=4
<=32	S	>8	R	8	R	>4	R	>8	R	>8
<=32	S	>8	R	>8	R	>4	R	>8	R	>8

64	R	>8	R	>8	R	>4	R	>8	R	<=4
<=32	S	>8	R	8	R	<=1	S	>8	R	<=4
<=32	S	<=2	S	<=1	S	<=1	S	<=1	S	<=4
64	R	>8	R	<=1	S	<=1	S	<=1	S	<=4
<=32	S	<=2	S	<=1	S	>4	R	<=1	S	>8
<=32	S	>8	R	>8	R	>4	R	>8	R	<=4
<=32	S	>8	R	2	I	2	S	2	I	<=4
<=32	S	>8	R	<=1	S	>4	R	8	R	8
<=32	S	>8	R	2	I	2	S	8	R	<=4
64	R	>8	R	2	I	2	S	4	R	<=4
<=32	S	>8	R	2	I	>4	R	8	R	<=4
<=32	S	>8	R	>8	R	>4	R	>8	R	>8
<=32	S	>8	R	2	I	2	S	2	I	<=4
<=32	S	>8	R	2	I	>4	R	4	R	>8
<=32	S	>8	R	<=1	S	4	I	<=1	S	<=4
<=32	S	>8	R	>8	R	>4	R	>8	R	<=4
64	R	>8	R	<=1	S	<=1	S	<=1	S	<=4
<=32	S	<=2	S	<=1	S	2	S	<=1	S	<=4
<=32	S	<=2	S	<=1	S	<=1	S	<=1	S	<=4
64	R	8	I	<=1	S	<=1	S	<=1	S	<=4
64	R	>8	R	>8	R	4	I	>8	R	8
<=32	S	>8	R	<=1	S	>4	R	8	R	<=4
<=32	S	<=2	S	N/R		<=1	S	>8	R	<=4
<=32	S	>8	R	<=1	S	<=1	S	<=1	S	<=4
<=32	S	>8	R	2	I	<=1	S	<=1	S	<=4
<=32	S	<=2	S	>8	R	>4	R	>8	R	>8
<=32	S	>8	R	4	R	2	S	<=1	S	<=4
>64	R	>8	R	<=1	S	<=1	S	<=1	S	<=4
<=32	S	>8	R	<=1	S	<=1	S	<=1	S	<=4
<=32	S	>8	R	<=1	S	>4	R	8	R	<=4
<=32	S	>8	R	<=1	S	<=1	S	<=1	S	<=4
<=32	S	>8	R	>8	R	>4	R	>8	R	>8
<=32	S	>8	R	>8	R	>4	R	>8	R	<=4
<=32	S	<=2	S	<=1	S	>4	R	<=1	S	<=4
64	R	>8	R	<=1	S	2	S	2	I	<=4
<=32	S	>8	R	<=1	S	<=1	S	<=1	S	<=4
>64	R	4	S	>8	R	>4	R	>8	R	8
64	R	<=2	S	4	R	<=1	S	8	R	<=4
64	R	>8	R	2	I	4	I	4	R	<=4
<=32	S	>8	R	>8	R	>4	R	>8	R	>8
<=32	S	>8	R	>8	R	<=1	S	8	R	<=4
<=32	S	>8	R	>8	R	<=1	S	>8	R	<=4
64	R	>8	R	8	R	<=1	S	8	R	<=4
<=32	S	>8	R	<=1	S	<=1	S	<=1	S	>8
<=32	S	>8	R	<=1	S	<=1	S	4	R	<=4
64	R	>8	R	>8	R	<=1	S	>8	R	<=4
<=32	S	>8	R	2	I	>4	R	>8	R	>8
<=32	S	<=2	S	N/R		<=1	S	>8	R	<=4
<=32	S	>8	R	8	R	2	S	8	R	8
<=32	S	>8	R	4	R	2	S	8	R	8
<=32	S	>8	R	2	I	>4	R	8	R	<=4
<=32	S	<=2	S	8	R	<=1	S	>8	R	<=4
<=32	S	>8	R	4	R	2	S	8	R	8
<=32	S	>8	R	2	I	>4	R	8	R	<=4
<=32	S	<=2	S	8	R	<=1	S	<=1	S	<=4
<=32	S	<=2	S	N/R		<=1	S	>8	R	<=4

<=32	S	>8	R	>8	R	>4	R	>8	R	>8
64	R	>8	R	>8	R	>4	R	>8	R	<=4
<=32	S	>8	R	<=1	S	<=1	S	<=1	S	<=4
<=32	S	>8	R	<=1	S	<=1	S	<=1	S	<=4
<=32	S	<=2	S	>8	R	<=1	S	>8	R	<=4
<=32	S	<=2	S	<=1	S	>4	R	<=1	S	<=4
<=32	S	>8	R	>8	R	>4	R	>8	R	>8
<=32	S	4	S	<=1	S	>4	R	8	R	<=4
64	R	<=2	S	<=1	S	<=1	S	<=1	S	<=4
<=32	S	8	I	>8	R	>4	R	>8	R	>8
<=32	S	<=2	S	2	I	>4	R	8	R	<=4
<=32	S	4	S	>8	R	>4	R	>8	R	>8
<=32	S	>8	R	<=1	S	<=1	S	<=1	S	<=4
<=32	S	<=2	S	N/R		<=1	S	>8	R	<=4
<=32	S	>8	R	<=1	S	>4	R	8	R	<=4
<=32	S	>8	R	>8	R	>4	R	>8	R	>8
<=32	S	>8	R	<=1	S	<=1	S	<=1	S	<=4
<=32	S	>8	R	<=1	S	<=1	S	<=1	S	<=4
64	R	>8	R	<=1	S	<=1	S	<=1	S	<=4
<=32	S	<=2	S	<=1	S	>4	R	<=1	S	<=4
<=32	S	>8	R	<=1	S	<=1	S	<=1	S	<=4
<=32	S	>8	R	>8	R	>4	R	>8	R	<=4
<=32	S	>8	R	<=1	S	<=1	S	8	R	<=4
<=32	S	>8	R	2	I	2	S	8	R	<=4
<=32	S	>8	R	2	I	4	I	8	R	<=4
<=32	S	>8	R	<=1	S	2	S	<=1	S	8
<=32	S	>8	R	8	R	>4	R	>8	R	<=4
<=32	S	>8	R	<=1	S	>4	R	4	R	<=4
<=32	S	4	S	<=1	S	>4	R	2	I	8
<=32	S	>8	R	<=1	S	<=1	S	4	R	<=4
<=32	S	>8	R	<=1	S	4	I	4	R	8
<=32	S	>8	R	>8	R	>4	R	8	R	>8
<=32	S	>8	R	>8	R	>4	R	>8	R	8
<=32	S	>8	R	2	I	>4	R	>8	R	<=4
<=32	S	>8	R	<=1	S	>4	R	<=1	S	>8
>64	R	>8	R	>8	R	>4	R	>8	R	<=4
64	R	<=2	S	>8	R	<=1	S	>8	R	<=4
<=32	S	>8	R	8	R	4	I	>8	R	<=4
>64	R	>8	R	>8	R	>4	R	>8	R	8
<=32	S	>8	R	4	R	<=1	S	2	I	<=4
<=32	S	>8	R	<=1	S	>4	R	8	R	>8
<=32	S	<=2	S	2	I	>4	R	8	R	<=4

<=32	S	<=2	S	2	I	4	I	2	I	<=4

<=32	S	>8	R	4	R	<=1	S	2	I	<=4
<=32	S	<=2	S	8	R	<=1	S	4	R	<=4
<=32	S	>8	R	<=1	S	<=1	S	<=1	S	<=4
64	R	>8	R	2	I	4	I	8	R	>8
<=32	S	<=2	S	<=1	S	4	I	<=1	S	<=4
64	R	>8	R	>8	R	>4	R	>8	R	8
<=32	S	>8	R	2	I	>4	R	8	R	>8
<=32	S	>8	R	8	R	>4	R	>8	R	8
<=32	S	>8	R	8	R	<=1	S	>8	R	<=4
<=32	S	>8	R	<=1	S	>4	R	4	R	<=4
<=32	S	>8	R	>8	R	>4	R	>8	R	>8
<=32	S	>8	R	>8	R	>4	R	<=1	S	<=4
<=32	S	>8	R	2	I	>4	R	8	R	<=4
<=32	S	>8	R	2	I	>4	R	<=1	S	<=4
<=32	S	>8	R	2	I	>4	R	<=1	S	<=4
<=32	S	>8	R	<=1	S	<=1	S	<=1	S	<=4
>64	R	>8	R	2	I	>4	R	4	R	<=4
<=32	S	>8	R	<=1	S	<=1	S	<=1	S	<=4
>64	R	>8	R	<=1	S	<=1	S	<=1	S	<=4
<=32	S	>8	R	>8	R	<=1	S	>8	R	<=4
<=32	S	>8	R	<=1	S	<=1	S	<=1	S	<=4
<=32	S	>8	R	4	R	>4	R	8	R	<=4
<=32	S	>8	R	>8	R	>4	R	>8	R	8

<=32	S	>8	R	>8	R	>4	R	>8	R	<=4
<=32	S	>8	R	>8	R	>4	R	>8	R	<=4
<=32	S	>8	R	>8	R	>4	R	>8	R	8
<=32	S	>8	R	8	R	>4	R	>8	R	>8
<=32	S	>8	R	2	I	4	I	8	R	<=4
<=32	S	>8	R	>8	R	>4	R	>8	R	>8
64	R	>8	R	8	R	>4	R	8	R	<=4
<=32	S	>8	R	<=1	S	<=1	S	<=1	S	<=4
<=32	S	>8	R	8	R	>4	R	4	R	8
64	R	>8	R	>8	R	>4	R	>8	R	8
<=32	S	>8	R	>8	R	>4	R	>8	R	<=4
64	R	>8	R	4	R	>4	R	>8	R	<=4
64	R	>8	R	>8	R	>4	R	>8	R	>8
<=32	S	>8	R	4	R	>4	R	>8	R	<=4
<=32	S	>8	R	2	I	>4	R	>8	R	<=4
64	R	>8	R	>8	R	>4	R	>8	R	8
<=32	S	>8	R	>8	R	>4	R	>8	R	<=4
<=32	S	>8	R	2	I	>4	R	8	R	8
<=32	S	4	S	2	I	>4	R	>8	R	<=4
>64	R	>8	R	>8	R	>4	R	>8	R	>8
>64	R	>8	R	>8	R	>4	R	>8	R	>8
64	R	>8	R	<=1	S	<=1	S	<=1	S	<=4
64	R	<=2	S	<=1	S	<=1	S	<=1	S	<=4
<=32	S	>8	R	<=1	S	<=1	S	<=1	S	<=4
64	R	>8	R	<=1	S	<=1	S	<=1	S	<=4
64	R	>8	R	>8	R	>4	R	>8	R	>8
<=32	S	>8	R	4	R	<=1	S	8	R	8
64	R	>8	R	<=1	S	>4	R	<=1	S	<=4
64	R	>8	R	>8	R	>4	R	>8	R	>8
>64	R	>8	R	4	R	>4	R	>8	R	<=4
<=32	S	>8	R	2	I	>4	R	>8	R	>8
>64	R	>8	R	<=1	S	>4	R	8	R	>8
<=32	S	>8	R	>8	R	<=1	S	4	R	<=4
<=32	S	>8	R	<=1	S	>4	R	8	R	>8
<=32	S	>8	R	8	R	2	S	4	R	>8
64	R	>8	R	4	R	>4	R	>8	R	<=4
64	R	>8	R	8	R	>4	R	>8	R	>8
<=32	S	>8	R	>8	R	>4	R	>8	R	<=4
64	R	>8	R	2	I	4	I	4	R	>8
64	R	>8	R	<=1	S	>4	R	8	R	>8
<=32	S	>8	R	>8	R	>4	R	>8	R	>8
<=32	S	>8	R	<=1	S	>4	R	8	R	>8
<=32	S	>8	R	<=1	S	4	I	4	R	<=4
<=32	S	<=2	S	<=1	S	<=1	S	<=1	S	<=4
<=32	S	>8	R	2	I	<=1	S	4	R	<=4
<=32	S	<=2	S	<=1	S	>4	R	<=1	S	<=4
<=32	S	>8	R	2	I	4	I	4	R	<=4
<=32	S	>8	R	<=1	S	<=1	S	<=1	S	<=4
64	R	>8	R	>8	R	<=1	S	>8	R	<=4
<=32	S	>8	R	>8	R	>4	R	>8	R	<=4
<=32	S	<=2	S	>8	R	>4	R	>8	R	8
<=32	S	<=2	S	>8	R	>4	R	>8	R	<=4
>64	R	8	I	>8	R	<=1	S	>8	R	<=4
<=32	S	<=2	S	N/R		<=1	S	>8	R	8
<=32	S	<=2	S	<=1	S	>4	R	<=1	S	<=4
>64	R	<=2	S	<=1	S	>4	R	<=1	S	8

<=32	S	>8	R	>8	R	>4	R	>8	R	>8
<=32	S	4	S	8	R	>4	R	4	R	<=4
<=32	S	<=2	S	N/R		<=1	S	4	R	8
<=32	S	>8	R	<=1	S	>4	R	<=1	S	<=4
<=32	S	>8	R	<=1	S	<=1	S	<=1	S	<=4
<=32	S	>8	R	>8	R	>4	R	>8	R	<=4
>64	R	>8	R	>8	R	2	S	>8	R	<=4
<=32	S	>8	R	8	R	>4	R	8	R	<=4
<=32	S	>8	R	>8	R	>4	R	>8	R	<=4
<=32	S	>8	R	<=1	S	<=1	S	<=1	S	8
<=32	S	>8	R	<=1	S	<=1	S	<=1	S	<=4
<=32	S	>8	R	<=1	S	<=1	S	4	R	<=4
<=32	S	>8	R	<=1	S	<=1	S	4	R	<=4
<=32	S	>8	R	>8	R	<=1	S	>8	R	<=4
<=32	S	<=2	S	<=1	S	<=1	S	<=1	S	<=4
<=32	S	<=2	S	4	R	<=1	S	8	R	<=4
<=32	S	>8	R	<=1	S	<=1	S	<=1	S	<=4
<=32	S	>8	R	<=1	S	<=1	S	<=1	S	<=4
<=32	S	<=2	S	4	R	<=1	S	2	I	<=4
<=32	S	<=2	S	<=1	S	<=1	S	<=1	S	<=4
<=32	S	>8	R	<=1	S	4	I	<=1	S	>8
64	R	>8	R	>8	R	>4	R	>8	R	<=4
<=32	S	>8	R	>8	R	>4	R	>8	R	<=4
<=32	S	>8	R	<=1	S	<=1	S	<=1	S	<=4
<=32	S	>8	R	2	I	4	I	4	R	<=4
<=32	S	>8	R	<=1	S	<=1	S	4	R	<=4
<=32	S	<=2	S	>8	R	>4	R	>8	R	<=4
<=32	S	>8	R	<=1	S	4	I	<=1	S	<=4
<=32	S	>8	R	<=1	S	<=1	S	<=1	S	<=4
<=32	S	>8	R	>8	R	>4	R	>8	R	>8
<=32	S	<=2	S	8	R	<=1	S	<=1	S	<=4
<=32	S	>8	R	<=1	S	<=1	S	<=1	S	<=4
64	R	>8	R	>8	R	>4	R	>8	R	8
<=32	S	>8	R	<=1	S	<=1	S	4	R	<=4
64	R	>8	R	4	R	4	I	<=1	S	<=4
<=32	S	>8	R	<=1	S	<=1	S	<=1	S	<=4
<=32	S	4	S	<=1	S	>4	R	<=1	S	<=4
<=32	S	>8	R	2	I	>4	R	8	R	<=4
<=32	S	<=2	S	<=1	S	>4	R	4	R	<=4
<=32	S	>8	R	>8	R	>4	R	>8	R	>8
<=32	S	>8	R	>8	R	>4	R	>8	R	>8
<=32	S	<=2	S	<=1	S	>4	R	<=1	S	<=4
<=32	S	<=2	S	<=1	S	4	I	<=1	S	<=4
64	R	>8	R	8	R	<=1	S	>8	R	<=4
<=32	S	>8	R	8	R	<=1	S	8	R	<=4
<=32	S	<=2	S	<=1	S	>4	R	<=1	S	<=4
<=32	S	>8	R	<=1	S	<=1	S	<=1	S	8
64	R	>8	R	<=1	S	<=1	S	<=1	S	<=4
<=32	S	>8	R	4	R	<=1	S	>8	R	<=4
<=32	S	>8	R	>8	R	>4	R	>8	R	<=4
<=32	S	>8	R	2	I	<=1	S	8	R	<=4
64	R	>8	R	>8	R	>4	R	>8	R	<=4
<=32	S	<=2	S	<=1	S	<=1	S	<=1	S	>8
<=32	S	>8	R	<=1	S	<=1	S	4	R	<=4
64	R	>8	R	<=1	S	<=1	S	<=1	S	<=4
<=32	S	>8	R	>8	R	>4	R	>8	R	<=4

<=32	S	>8	R	<=1	S	<=1	S	4	R	<=4
<=32	S	>8	R	>8	R	>4	R	>8	R	<=4
<=32	S	<=2	S	<=1	S	2	S	<=1	S	<=4
>64	R	>8	R	8	R	2	S	>8	R	<=4
<=32	S	>8	R	<=1	S	<=1	S	<=1	S	<=4
<=32	S	>8	R	<=1	S	<=1	S	<=1	S	8
<=32	S	>8	R	<=1	S	<=1	S	<=1	S	<=4
<=32	S	<=2	S	<=1	S	<=1	S	<=1	S	<=4
<=32	S	>8	R	>8	R	4	I	>8	R	<=4
64	R	>8	R	<=1	S	<=1	S	<=1	S	<=4
<=32	S	>8	R	4	R	<=1	S	>8	R	<=4
>64	R	>8	R	4	R	<=1	S	>8	R	<=4
<=32	S	>8	R	>8	R	>4	R	>8	R	<=4
<=32	S	>8	R	4	R	>4	R	>8	R	<=4
<=32	S	>8	R	>8	R	<=1	S	>8	R	<=4
<=32	S	>8	R	>8	R	<=1	S	>8	R	<=4
<=32	S	>8	R	<=1	S	>4	R	<=1	S	<=4
<=32	S	>8	R	<=1	S	>4	R	<=1	S	>8
<=32	S	>8	R	2	I	<=1	S	4	R	<=4
<=32	S	>8	R	>8	R	>4	R	>8	R	<=4
<=32	S	8	I	<=1	S	>4	R	<=1	S	>8
<=32	S	>8	R	<=1	S	2	S	8	R	<=4
<=32	S	>8	R	<=1	S	<=1	S	4	R	<=4
<=32	S	>8	R	>8	R	>4	R	>8	R	<=4
<=32	S	>8	R	<=1	S	<=1	S	8	R	<=4
<=32	S	>8	R	2	I	<=1	S	>8	R	<=4
<=32	S	>8	R	>8	R	>4	R	>8	R	<=4
<=32	S	>8	R	>8	R	>4	R	>8	R	>8

min	ptmic	pt	pipmic	pip	tetmic	tet	tgcmic	tgc	tsmic	ts	ndm
I	>64	R	>64	R	>8	R	2	I	4/76	R	Negative
R	>64	R	>64	R	>8	R	<=1	S	>4/76	R	Negative
R	>64	R	>64	R	>8	R	<=1	S	>4/76	R	Positive
S	>64	R	>64	R	<=4	S	<=1	S	>4/76	R	Positive
R	>64	R	>64	R	>8	R	<=1	S	>4/76	R	Negative
S	>64	R	>64	R	<=4	S	<=1	S	>4/76	R	Negative
S	>64	R	>64	R	<=4	S	<=1	S	>4/76	R	Negative
S	>64	R	>64	R	<=4	S	<=1	S	>4/76	R	Negative
R	>64	R	>64	R*	>8	R	<=1	S	>4/76	R	Negative
S	>64	R	>64	R	8	I	<=1	S	>4/76	R	Negative
R	>64	R	>64	R	>8	R	<=1	S	>4/76	R	Negative
R	64	I	>64	R*	8	I	<=1	S	>4/76	R	Negative
S	>64	R	>64	R	<=4	S	<=1	S	>4/76	R	Positive
S	>64	R	>64	R	<=4	S	<=1	S	>4/76	R	Positive
R	>64	R	>64	R	>8	R	<=1	S	>4/76	R	Negative
R	>64	R	>64	R	>8	R	2	I	>4/76	R	Negative
I	64	I	>64	R	8	I	2	I	>4/76	R	Negative
S	>64	R	>64	R	<=4	S	<=1	S	>4/76	R	Positive
S	>64	R	>64	R	<=4	S	<=1	S	>4/76	R	Positive
S	>64	R	>64	R	<=4	S	<=1	S	>4/76	R	Positive
I	>64	R	>64	R	<=4	S	<=1	S	>4/76	R	Positive
S	>64	R	>64	R	<=4	S	<=1	S	>4/76	R	Positive
R	>64	R	>64	R	>8	R	<=1	S	>4/76	R	Positive
S	>64	R	>64	R	<=4	S	<=1	S	>4/76	R	Positive
S	>64	R	>64	R	<=4	S	<=1	S	>4/76	R	Positive
S	>64	R	>64	R	<=4	S	<=1	S	>4/76	R	Positive
S	>64	R	>64	R	8	I	<=1	S	>4/76	R	Negative
I	>64	R	>64	R	>8	R	2	I	>4/76	R	Positive
I	>64	R	>64	R	8	I	<=1	S	>4/76	R	Positive
S	64	I	>64	R	<=4	S	<=1	S	>4/76	R	Negative
S	>64	R	>64	R	>8	R	2	I	>4/76	R	Positive
I	>64	R	>64	R	>8	R	<=1	S	>4/76	R	Negative
S	>64	R	>64	R*	>8	R	<=1	S	>4/76	R	Negative
R	>64	R	>64	R*	>8	R	2	I	>4/76	R	Negative
R	>64	R	>64	R*	>8	R	2	I	>4/76	R	Negative
S	>64	R	>64	R	<=4	S	<=1	S	>4/76	R	Positive
S	>64	R	>64	R	<=4	S	<=1	S	>4/76	R	Positive
S	>64	R	>64	R	<=4	S	<=1	S	>4/76	R	Positive
S	>64	R	>64	R	<=4	S	<=1	S	>4/76	R	Positive
S	>64	R	>64	R	<=4	S	<=1	S	>4/76	R	Positive
R	>64	R	>64	R	>8	R	<=1	S	>4/76	R	Positive
S	>64	R	>64	R	<=4	S	<=1	S	>4/76	R	Positive
S	>64	R	>64	R	<=4	S	<=1	S	>4/76	R	Negative
S	>64	R	>64	R	<=4	S	<=1	S	>4/76	R	Negative
S	64	I	>64	R	<=4	S	<=1	S	>4/76	R	Negative
R	>64	R	>64	R	>8	R	<=1	S	>4/76	R	Positive
R	>64	R	>64	R	>8	R	>2	R	<=2/38	S	Negative
S	>64	R	>64	R	<=4	S	<=1	S	>4/76	R	Positive
S	<=8	S	>64	R	>8	R	<=1	S	>4/76	R	Negative
S	>64	R	>64	R	>8	R	<=1	S	>4/76	R	Positive
I	>64	R	>64	R*	>8	R	<=1	S	>4/76	R	Negative

I	>64	R	>64	R*	>8	R	<=1	S	>4/79	R	Negative
I	<=8	S	<=8	S	>8	R	2	I	<=2/38	S	Negative
I	64	I	>64	R*	>8	R	<=1	S	>4/76	R	Negative
I	64	I	>64	R*	>8	R	<=1	S	>4/76	R	Negative
I	64	I	>64	R*	>8	R	<=1	S	>4/76	R	Negative
S	>64	R	>64	R	>8	R	<=1	S	>4/76	R	Negative
R	>64	R	>64	R	>8	R	<=1	S	>4/76	R	Negative
S	>64	R	>64	R	>8	R	<=1	S	>4/76	R	Positive
I	64	I	64	I	>8	R	2	I	<=2/38	S	Positive
S	>64	R	>64	R	<=4	S	<=1	S	>4/76	R	Positive
R	>64	R	>64	R	>8	R	<=1	S	>4/76	R	Negative
R	>64	R	>64	R	>8	R	<=1	S	>4/76	R	Negative
I	>64	R	>64	R	>8	R	2	I	>4/76	R	Negative
S	>64	R	>64	R	<=4	S	<=1	S	>4/76	R	Positive
S	64	I	64	I	8	I	<=1	S	<=2/38	S	Negative
R	>64	R	>64	R*	>8	R	2	I	>4/76	R	Negative
R	>64	R	>64	R*	>8	R	2	I	>4/76	R	Negative
S	>64	R	>64	R	<=4	S	>2	R	>4/76	R	Positive
I	>64	R	>64	R*	>8	R	2	I	>4/76	R	Negative
R	>64	R	>64	R*	>8	R	<=1	S	>4/76	R	Negative
I	>64	R	>64	R	>8	R	>2	R	>4/76	R	Negative
I	>64	R	>64	R	<=4	S	2	I	>4/76	R	Positive
I	>64	R	>64	R	>8	R	<=1	S	>4/76	R	Positive
S	64	I	>64	R*	<=4	S	<=1	S	>4/76	R	Negative
S	>64	R	>64	R	<=4	S	<=1	S	>4/76	R	Positive
S	>64	R	>64	R	<=4	S	<=1	S	>4/76	R	Positive
I	>64	R	>64	R	>6	R	<=1	S	>4/76	R	Positive
R	64	I	>64	R*	>8	R	>2	R	>4/76	R	Negative
S	>64	R	>64	R	>8	R	<=1	S	>4/76	R	Negative
I	>64	R	>64	R	>8	R	<=1	S	>4/76	R	Negative
R	64	I	64	I	>8	R	>2	R	>4/76	R	Negative
S	>64	R	>64	R	8	I	>2	R	>4/76	R	Positive
S	>64	R	>64	R	<=4	S	<=1	S	>4/76	R	Negative
S	<=8	S	64	I	<=4	S	<=1	S	<=2/38	S	Negative

S	64	I	>64	R*	<=4	S	<=1	S	>4/76	R	Negative
R	>64	R	>64	R	>8	R	2	I	>4/76	R	Positive
S	>64	R	>64	R	>8	R	<=1	S	>4/76	R	Positive
S	>64	R	>64	R	<=4	S	<=1	S	>4/76	R	Positive
R	>64	R	>64	R	>8	R	>2	R	>4/76	R	Negative
I	>64	R	>64	R	>8	R	<=1	S	>4/76	R	Positive
R	>64	R	>64	R	>6	R	2	I	>4/76	R	Positive
R	>64	R	>64	R	>8	R	2	I	>4/76	R	Positive
S	>64	R	>64	R*	<=4	S	<=1	S	>4/76	R	Negative
S	>64	R	>64	R	>8	R	<=1	S	>4/76	R	Negative
I	>64	R	>64	R*	>8	R	2	I	>4/76	R	Negative
S	>64	R	>64	R	<=4	S	<=1	S	>4/76	R	Positive
S	64	I	>64	R	<=4	S	<=1	S	>4/76	R	Negative
R	>64	R	>64	R	>8	R	<=1	S	>4/76	R	Negative
R	>64	R	>64	R	>8	R	>2	R	>4/76	R	Negative
S	64	I	>64	R	<=4	S	<=1	S	>4/76	R	Negative
S	<=8	S	<=8	S	<=4	S	<=1	S	<=2/38	S	Negative
R	>64	R	>64	R*	>8	R	<=1	S	>4/76	R	Negative
R	>64	R	>64	R	>8	R	2	I	>4/76	R	Negative
R	>64	R	>64	R	>8	R	<=1	S	>4/76	R	Positive
R	>64	R	>64	R	>8	R	<=1	S	>4/76	R	Negative
I	>64	R	>64	R	8	I	2	I	<=2/38	S	Positive
R	64	I	>64	R	>8	R	2	I	>4/76	R	Negative
I	>64	R	>64	R	>8	R	2	I	>4/76	R	Positive
R	>64	R	>64	R	>8	R	>2	R	>4/76	R	Positive
S	>64	R	>64	R*	8	I	<=1	S	>4/76	R	Negative
S	>64	R	>64	R*	>8	R	<=1	S	>4/76	R	Negative
I	>64	R	>64	R	>8	R	<=1	S	>4/76	R	Negative
I	64	I	>64	R	>8	R	2	I	>4/76	R	Negative
S	>64	R	>64	R	<=4	S	<=1	S	>4/76	R	Negative
R	>64	R	>64	R	>8	R	>2	R	>4/76	R	Negative
R	>64	R	>64	R	>8	R	>2	R	>4/76	R	Positive
R	>64	R	>64	R	>8	R	>2	R	>4/76	R	Negative
R	>64	R	>64	R	>8	R	2	I	>4/76	R	Negative
I	>64	R	>64	R*	>8	R	2	I	>4/76	R	Negative
R	>64	R	>64	R*	>8	R	2	I	>4/76	R	Negative
R	>64	R	>64	R	>8	R	<=1	S	>4/76	R	Negative
I	>64	R	>64	R	>8	R	<=1	S	>4/76	R	Negative
S	>64	R	>64	R	>8	R	2	I	>4/76	R	Positive
S	>64	R	>64	R	>8	R	<=1	S	>4/76	R	Positive
R	>64	R	>64	R	>8	R	2	I	>4/76	R	Positive
R	>64	R	>64	R	>8	R	>2	R	>4/76	R	Positive
S	64	I	>64	R	<=4	S	<=1	S	<=2/38	S	Negative
I	64	I	>64	R*	>8	R	<=1	S	>4/76	R	Negative
R	>64	R	>64	R	>8	R	<=1	S	>4/76	R	Positive
I	64	I	>64	R*	>8	R	<=1	S	>4/76	R	Negative
S	>64	R	>64	R	<=4	S	<=1	S	>4/76	R	Negative
S	>64	R	>64	R	<=4	S	<=1	S	4/76	R	Negative
R	>64	R	>64	R	>8	R	>2	R	>4/76	R	Negative
S	>64	R	>64	R	<=4	S	<=1	S	>4/76	R	Negative

R	>64	R	>64	R	>8	R	<=1	S	>4/76	R	Negative
S	>64	R	>64	R*	>8	R	<=1	S	>4/76	R	Negative
I	>64	R	>64	R	>8	R	<=1	S	>4/76	R	Positive
R	>64	R	>64	R	>8	R	>2	R	>4/76	R	Negative
R	>64	R	>64	R	>8	R	2	I	>4/76	R	Positive
S	>64	R	>64	R	<=4	S	<=1	S	>4/76	R	Positive
I	16	S	>64	R	>8	R	2	I	>4/76	R	Positive
R	>64	R	>64	R	>8	R	<=1	S	>4/76	R	Positive
S	>64	R	>64	R*	>8	R	<=1	S	>4/76	R	Negative
R	>64	R	>64	R	>8	R	>2	R	>4/76	R	Positive
S	<=8	S	>64	R*	<=4	S	<=1	S	>4/76	R	Negative
S	>64	R	>64	R	<=4	S	<=1	S	>4/76	R	Positive
R	>64	R	>64	R	>8	R	<=1	S	>4/76	R	Positive
R	>64	R	>64	R	>8	R	<=1	S	>4/76	R	Negative
S	>64	R	>64	R	<=4	S	<=1	S	>4/76	R	Negative
R	>64	R	>64	R*	>8	R	2	I	>4/76	R	Negative
S	>64	R	>64	R*	<=4	S	<=1	S	>4/76	R	Negative
S	>64	R	>64	R	>8	R	2	I	>4/76	R	Positive
S	>64	R	>64	R	>8	R	<=1	S	<=2/38	S	Positive
I	64	I	>64	R	>8	R	<=1	S	>4/76	R	Negative
S	>64	R	>64	R	>8	R	<=1	S	>4/76	R	Positive
R	>64	R	>64	R	>8	R	2	I	>4/76	R	Negative
S	>64	R	>64	R	<=4	S	<=1	S	>4/76	R	Positive
S	>64	R	>64	R	<=4	S	<=1	S	>4/76	R	Negative
S	>64	R	>64	R*	8	I	2	I	>4/76	R	Negative
R	>64	R	>64	R*	>8	R	2	I	>4/76	R	Negative
I	>64	R	>64	R	8	I	2	I	>4/76	R	Positive
S	>64	R	>64	R	<=4	S	<=1	S	>4/76	R	Positive
S	>64	R	>64	R	8	I	<=1	S	<=2/38	S	Positive

S	>64	R	>64	R	<=4	S	<=1	S	>4/76	R	Negative
S	>64	R	>64	R	<=4	S	<=1	S	>4/76	R	Positive
R	64	I	>64	R*	>8	R	<=1	S	>4/76	R	Negative
S	<=8	S	<=8	S	<=4	S	<=1	S	<=2/38	S	Positive
S	<=8	S	>64	R*	<=4	S	<=1	S	>4/76	R	Negative

S	>64	R	>64	R	>8	R	2	I	>4/76	R	Negative
S	>64	R	>64	R	<=4	S	<=1	S	>4/76	R	Negative
S	16	S	64	I	<=4	S	<=1	S	>4/76	R	Negative
R	>64	R	>64	R	>8	R	<=1	S	>4/76	R	Negative
S	>64	R	>64	R*	<=4	S	<=1	S	>4/76	R	Negative
R	>64	R	>64	R	>8	R	<=1	S	>4/76	R	Negative
R	>64	R	>64	R*	>8	R	<=1	S	>4/76	R	Negative
R	>64	R	>64	R*	>8	R	<=1	S	>4/76	R	Negative
S	>64	R	>64	R	<=4	S	<=1	S	>4/76	R	Positive
S	>64	R	>64	R	>8	R	<=1	S	>4/76	R	Negative
R	>64	R	>64	R	>8	R	<=1	S	>4/76	R	Positive
S	<=8	S	>64	R	<=4	S	<=1	S	>4/76	R	Negative
S	64	I	>64	R*	<=4	S	<=1	S	>4/76	R	Negative
R	>64	R	>64	R	>8	R	<=1	S	>4/76	R	Positive

R	>64	R	>64	R	>8	R	<=1	S	>4/76	R	Negative
S	>64	R	>64	R	>8	R	<=1	S	>4/76	R	Negative
S	>64	R	>64	R	>8	R	<=1	S	>4/76	R	Negative
S	>64	R	>64	R	<=4	S	<=1	S	>4/76	R	Negative
S	>64	R	>64	R	<=4	S	<=1	S	>4/76	R	Negative
R	>64	R	>64	R*	>8	R	2	I	>4/76	R	Negative
S	>64	R	>64	R*	<=4	S	<=1	S	>4/76	R	Negative
I	64	I	>64	R*	>8	R	<=1	S	>4/76	R	Negative
R	>64	R	>64	R	>8	R	2	I	>4/76	R	Negative
S	>64	R	>64	R	>8	R	2	I	>4/76	R	Positive
S	>64	R	>64	R	>8	R	2	I	>4/76	R	Positive
S	>64	R	>64	R	>8	R	2	I	>4/76	R	Positive
S	>64	R	>64	R	>8	R	2	I	>4/76	R	Positive
I	>64	R	>64	R	>8	R	<=1	S	>4/76	R	Positive
S	>64	R	>64	R*	>8	R	<=1	S	>4/76	R	Negative
R	>64	R	>64	R*	>8	R	<=1	S	>4/76	R	Negative
S	>64	R	>64	R*	<=4	S	<=1	S	>4/76	R	Negative
R	>64	R	>64	R	>8	R	<=1	S	>4/76	R	Negative
S	>64	R	>64	R*	<=4	S	<=1	S	>4/76	R	Negative
I	>64	R	>64	R	>8	R	<=1	S	>4/76	R	Positive
R	>64	R	>64	R	>8	R	>2	R	>4/76	R	Negative
R	64	I	64	I	>8	R	>2	R	>4/76	R	Positive
R	>64	R	>64	R*	>8	R	<=1	S	>4/76	R	Negative
S	>64	R	>64	R	<=4	S	<=1	S	>4/76	R	Positive
S	>64	R	>64	R*	<=4	S	<=1	S	>4/76	R	Negative
R	>64	R	>64	R	>8	R	2	I	>4/76	R	Negative
S	>64	R	>64	R	<=4	S	<=1	S	>4/76	R	Negative
S	>64	R	>64	R*	<=4	S	<=1	S	>4/76	R	Negative
S	>64	R	>64	R	<=4	S	<=1	S	>4/76	R	Positive
S	>64	R	>64	R	<=4	S	<=1	S	>4/76	R	Positive
S	64	I	>64	R*	<=4	S	<=1	S	>4/76	R	Negative
S	>64	R	>64	R	<=4	S	<=1	S	>4/76	R	Positive
S	>64	R	>64	R	<=4	S	<=1	S	>4/76	R	Positive
S	>64	R	>64	R	>8	R	<=1	S	>4/76	R	Positive
S	>64	R	>64	R	<=4	S	<=1	S	<=2/38	S	Positive
R	>64	R	>64	R*	>8	R	<=1	S	>4/76	R	Negative
R	>64	R	>64	R	>8	R	<=1	S	<=2/38	S	Positive
S	>64	R	>64	R	<=4	S	<=1	S	>4/76	R	Positive
R	>64	R	>64	R*	>8	R	<=1	S	>4/76	R	Negative
R	>64	R	>64	R*	>8	R	<=1	S	>4/76	R	Negative
S	>64	R	>64	R	<=4	S	<=1	S	>4/76	R	Positive
S	<=8	S	>64	R	>8	R	<=1	S	>4/76	R	Negative
S	64	I	>64	R*	>8	R	2	I	>4/76	R	Negative
S	>64	R	>64	R*	<=4	S	<=1	S	>4/76	R	Negative
S	>64	R	>64	R	<=4	S	<=1	S	<=2/38	S	Negative
S	>64	R	>64	R	<=4	S	<=1	S	>4/76	R	Negative

S	>64	R	>64	R	<=4	S	<=1	S	>4/76	R	Negative
R	>64	R	>64	R	>8	R	<=1	S	>4/76	R	Negative
S	>64	R	>64	R	<=4	S	<=1	S	<=2/38	S	Negative
S	>64	R	>64	R	<=4	S	<=1	S	>4/76	R	Positive
I	64	I	>64	R*	>8	R	<=1	S	>4/76	R	Negative
S	>64	R	>64	R	>8	R	<=1	S	>4/76	R	Positive
R	>64	R	>64	R	>8	R	2	I	>4/76	R	Negative
S	>64	R	>64	R	<=4	S	<=1	S	>4/76	R	Positive
S	<=8	S	>64	R*	>8	R	<=1	S	>4/76	R	Negative
S	64	I	>64	R*	>8	R	<=1	S	>4/76	R	Negative
S	>64	R	>64	R	<=4	S	<=1	S	>4/76	R	Positive
S	>64	R	>64	R	<=4	S	<=1	S	>4/76	R	Negative
S	>64	R	>64	R	<=4	S	<=1	S	>4/76	R	Positive
R	>64	R	>64	R*	>8	R	<=1	S	>4/76	R	Negative
R	>64	R	>64	R*	>8	R	<=1	S	>4/76	R	Negative
R	>64	R	>64	R	>8	R	<=1	S	>4/76	R	Negative
R	>64	R	>64	R	>8	R	<=1	S	>4/76	R	Negative
R	>64	R	>64	R	>8	R	<=1	S	>4/76	R	Negative
R	>64	R	>64	R*	>8	R	<=1	S	>4/76	R	Negative
R	>64	R	>64	R	>8	R	<=1	S	>4/76	R	Negative
R	>64	R	>64	R*	>8	R	<=1	S	>4/76	R	Negative
S	16	S	>64	R	<=4	S	<=1	S	>4/76	R	Negative
S	>64	R	>64	R*	<=4	S	<=1	S	>4/76	R	Negative
S	>64	R	>64	R*	<=4	S	<=1	S	>4/76	R	Negative
R	>64	R	>64	R*	>8	R	<=1	S	>4/76	R	Negative
R	>64	R	>64	R	>8	R	<=1	S	>4/76	R	Negative
R	>64	R	>64	R*	>8	R	<=1	S	>4/76	R	Negative
S	>64	R	>64	R	>8	R	<=1	S	>4/76	R	Positive
R	>64	R	>64	R	>8	R	<=1	S	>4/76	R	Positive
S	>64	R	>64	R	>8	R	<=1	S	>4/76	R	Negative
R	>64	R	>64	R	>8	R	2	I	>4/76	R	Negative
I	>64	R	>64	R	8	I	<=1	S	>4/76	R	Positive
R	>64	R	>64	R	>8	R	<=1	S	>4/76	R	Negative
S	>64	R	>64	R	<=4	S	<=1	S	<=2/38	S	Negative
S	>64	R	>64	R	>8	R	<=1	S	>4/76	R	Negative
R	>64	R	>64	R*	>8	R	>2	R	>4/76	R	Negative
S	>64	R	>64	R*	<=4	S	<=1	S	>4/76	R	Negative
S	<=8	S	<=8	S	<=4	S	<=1	S	<=2/38	S	Negative
R	>64	R	>64	R	>8	R	<=1	S	>4/76	R	Positive
R	>64	R	>64	R	>8	R	<=1	S	>4/76	R	Positive
R	>64	R	>64	R	>8	R	<=1	S	>4/76	R	Positive
S	>64	R	>64	R*	>8	R	<=1	S	>4/76	R	Negative
S	>64	R	>64	R	8	I	<=1	S	>4/76	R	Negative
S	>64	R	>64	R	<=4	S	<=1	S	>4/76	R	Positive

S	64	I	>64	R*	<=4	S	<=1	S	>4/76	R	Negative
R	>64	R	>64	R	>8	R	2	I	>4/76	R	Positive
S	<=8	S	64	R*	8	I	<=1	S	>4/76	R	Negative
R	>64	R	>64	R	>8	R	<=1	S	>4/76	R	Positive
R	>64	R	>64	R	>8	R	<=1	S	>4/76	R	Negative
R	>64	R	>64	R	>8	R	<=1	S	>4/76	R	Positive
R	>64	R	>64	R	>8	R	2	I	>4/76	R	Negative
R	>64	R	>64	R*	>8	R	<=1	S	>4/76	R	Negative
R	>64	R	>64	R*	>8	R	<=1	S	>4/76	R	Negative
S	>64	R	>64	R*	<=4	S	<=1	S	>4/76	R	Negative
R	>64	R	>64	R*	>8	R	<=1	S	>4/76	R	Negative
R	>64	R	>64	R	>8	R	<=1	S	>4/76	R	Positive
I	64	I	>64	R*	>8	R	2	I	>4/76	R	Negative
I	64	I	>64	R*	>8	R	2	I	>4/76	R	Negative
S	<=8	S	<=8	S	<=4	S	<=1	S	<=2/38	S	Negative
R	>64	R	>64	R	>8	R	<=1	S	>4/76	R	Positive
R	64	I	>64	R*	>8	R	<=1	S	>4/76	R	Negative
S	>64	R	>64	R	>8	R	<=1	S	>4/76	R	Positive
S	>64	R	>64	R	8	I	<=1	S	>4/76	R	Negative
S	>64	R	>64	R	>8	R	2	I	>4/76	R	Negative
R	>64	R	>64	R	>8	R	<=1	S	>4/76	R	Positive

S	64	I	>64	R	<=4	S	<=1	S	>4/76	R	Negative
R	>64	R	>64	R	>8	R	<=1	S	>4/76	R	Positive
R	>64	R	>64	R	>8	R	<=1	S	>4/76	R	Positive
I	>64	R	>64	R	>8	R	2	I	>4/76	R	Positive
R	>64	R	>64	R	>8	R	<=1	S	<=2/38	S	Positive
S	>64	R	>64	R	<=4	S	<=1	S	>4/76	R	Positive
R	>64	R	>64	R	>8	R	N/R		<=2/38	S	Negative
R	64	I	>64	R	>8	R	>2	R	>4/76	R	Negative

S	64	I	64	I	<=4	S	<=1	S	<=2/38	S	Negative
S	64	I	>64	R	<=4	S	<=1	S	>4/76	R	Negative
S	>64	R	>64	R*	<=4	S	<=1	S	>4/76	R	Negative
R	>64	R	>64	R*	>8	R	>2	R	>4/76	R	Negative
I	>64	R	>64	R*	>8	R	2	I	>4/76	R	Negative
S	>64	R	>64	R*	<=4	S	<=1	S	>4/76	R	Negative
I	>64	R	>64	R	8	I	>2	R	>4/76	R	Positive
I	64	I	>64	R*	>8	R	2	I	>4/76	R	Negative
R	>64	R	>64	R*	>8	R	<=1	S	>4/76	R	Negative
R	>64	R	>64	R*	>8	R	<=1	S	>4/76	R	Negative
R	>64	R	>64	R	>8	R	2	I	>4/76	R	Negative
S	>64	R	>64	R*	<=4	S	<=1	S	>4/76	R	Negative
R	>64	R	>64	R	>8	R	<=1	S	>4/76	R	Positive
R	>64	R	>64	R	>8	R	<=1	S	>4/76	R	Positive
S	16	S	>64	R*	>8	R	<=1	S	>4/76	R	Negative
S	>64	R	>64	R	>8	R	<=1	S	>4/76	R	Negative
R	>64	R	>64	R*	>8	R	>2	R	>4/76	R	Negative
I	>64	R	>64	R	>8	R	<=1	S	>4/76	R	Positive

R	<=8	S	<=8	S	>8	R	N/R		<=2/38	S	Negative
S	>64	R	>64	R	<=4	S	<=1	S	<=2/38	S	Negative
S	>64	R	>64	R*	<=4	S	<=1	S	>4/76	R	Negative
R	>64	R	>64	R	>8	R	<=1	S	>4/76	R	Positive
R	>64	R	>64	R	>8	R	<=1	S	>4/76	R	Positive
R	>64	R	>64	R	>8	R	<=1	S	>4/76	R	Positive
S	>64	R	>64	R*	<=4	S	<=1	S	>4/76	R	Positive
R	>64	R	>64	R	>8	R	<=1	S	>4/76	R	Positive
R	>64	R	>64	R	>8	R	<=1	S	>4/76	R	Positive
R	>64	R	>64	R	>8	R	<=1	S	<=2/38	S	Positive
S	<=8	S	>64	R*	<=4	S	<=1	S	>4/76	R	Positive
R	>64	R	>64	R	>8	R	<=1	S	>4/76	R	Positive

R	>64	R	>64	R*	>8	R	<=1	S	>4/76	R	Negative

I	>64	R	>64	R	8	I	2	I	>4/76	R	Negative
R	>64	R	>64	R	>8	R	2	I	>4/76	R	Negative
S	16	S	>64	R*	<=4	S	<=1	S	>4/76	R	Negative
R	>64	R	>64	R*	>8	R	2	I	>4/76	R	Negative
S	>64	R	>64	R	<=4	S	<=1	S	>4/76	R	Positive
S	>64	R	>64	R*	<=4	S	<=1	S	>4/76	R	Negative
R	>64	R	>64	R*	>8	R	<=1	S	>4/76	R	Negative
I	64	I	>64	R*	>8	R	2	I	>4/76	R	Negative
R	>64	R	>64	R	>8	R	<=1	S	>4/76	R	Positive
R	>64	R	>64	R*	>8	R	<=1	S	>4/76	R	Negative
S	>64	R	>64	R*	<=4	S	<=1	S	>4/76	R	Negative

S	>64	R	>64	R*	<=4	S	<=1	S	>4/76	R	Negative
R	>64	R	>64	R*	>8	R	<=1	S	>4/76	R	Negative
S	>64	R	>64	R	<=4	S	2	I	>4/76	R	Negative
R	>64	R	>64	R	>8	R	<=1	S	>4/76	R	Positive
S	>64	R	>64	R*	<=4	S	<=1	S	>4/76	R	Negative

R	>64	R	>64	R	>8	R	<=1	S	>4/76	R	Positive
R	>64	R	>64	R	>8	R	<=1	S	>4/76	R	Positive
R	>64	R	>64	R	>8	R	<=1	S	>4/76	R	Positive
S	>64	R	>64	R*	<=4	S	<=1	S	>4/76	R	Negative
S	64	I	>64	R	8	I	<=1	S	>4/76	R	Negative
R	>64	R	>64	R*	>8	R	<=1	S	>4/76	R	Negative
I	>64	R	>64	R*	>8	R	2	I	>4/76	R	Negative
I	>64	R	>64	R	>8	R	<=1	S	>4/76	R	Negative
R	>64	R	>64	R	>8	R	<=1	S	>4/76	R	Positive
S	>64	R	>64	R	<=4	S	<=1	S	<=2/38	S	Positive
I	>64	R	>64	R	>8	R	2	I	>4/76	R	Negative
R	>64	R	>64	R	>8	R	2	I	>4/76	R	Negative
S	>64	R	>64	R	<=4	S	<=1	S	<=2/38	S	Negative
S	>64	R	>64	R	8	I	<=1	S	<=2/38	S	Positive
S	>64	R	>64	R	<=4	S	<=1	S	>4/76	R	Positive
S	>64	R	>64	R	<=4	S	<=1	S	>4/76	R	Negative
R	64	I	>64	R	>8	R	<=1	S	>4/76	R	Positive
S	>64	R	>64	R*	8	I	2	I	>4/76	R	Negative
S	>64	R	>64	R	>8	R	<=1	S	>4/76	R	Positive
S	64	I	>64	R	>8	R	<=1	S	>4/76	R	Negative
R	>64	R	>64	R	>8	R	<=1	S	>4/76	R	Positive
S	>64	R	>64	R*	<=4	S	<=1	S	>4/76	R	Positive
S	64	I	>64	R*	<=4	S	<=1	S	>4/76	R	Negative
S	>64	R	>64	R*	<=4	S	<=1	S	>4/76	R	Negative
S	>64	R	>64		<=4	S	<=1	S	>4/76	R	Negative
S	>64	R	>64	R*	<=4	S	<=1	S	>4/76	R	Negative
S	>64	R	64	I	8	I	<=1	S	<=2/38	S	Positive
R	>64	R	>64	R*	>8	R	<=1	S	>4/76	R	Positive
S	>64	R	>64	R	<=4	S	<=1	S	>4/76	R	Negative

S	>64	R	>64	R*	<=4	S	<=1	S	>4/76	R	Negative
S	>64	R	>64	R	>8	R	<=1	S	>4/76	R	Negative
S	>64	R	>64	R	>8	R	<=1	S	>4/76	R	Negative
S	>64	R	>64	R	>8	R	<=1	S	>4/76	R	Positive
S	>64	R	>64	R*	<=4	S	<=1	S	>4/76	R	Negative
S	>64	R	>64	R	<=4	S	<=1	S	>4/76	R	Negative
S	>64	R	>64	R	<=4	S	<=1	S	>4/76	R	Negative
S	<=8	S	<=8	S	<=4	S	<=1	S	<=2/38	S	Negative
S	>64	R	>64	R	8	I	<=1	S	>4/76	R	Positive
R	>64	R	>64	R	>8	R	>2	R	>4/76	R	Negative
S	>64	R	>64	R	<=4	S	<=1	S	>4/76	R	Negative
S	>64	R	>64	R	<=4	S	<=1	S	>4/76	R	Negative
I	>64	R	>64	R	>8	R	2	I	>4/76	R	Positive
S	>64	R	>64	R*	<=4	S	<=1	S	>4/76	R	Negative
S	16	S	>64	R*	>8	R	<=1	S	>4/76	R	Negative
S	64	I	>64	R*	8	I	<=1	S	>4/76	R	Negative
S	>64	R	>64	R	<=4	S	<=1	S	>4/76	R	Positive
S	64	I	>64	R	<=4	S	<=1	S	>4/76	R	Negative
S	>64	R	>64	R*	<=4	S	<=1	S	>4/76	R	Negative
S	>64	R	>64	R*	<=4	S	<=1	S	>4/76	R	Negative
S	>64	R	>64	R	<=4	S	2	I	<=2/38	S	Negative
S	>64	R	>64	R*	<=4	S	<=1	S	>4/76	R	Negative
S	>64	R	>64	R*	<=4	S	<=1	S	>4/76	R	Negative
S	>64	R	>64	R*	<=4	S	<=1	S	>4/76	R	Negative
S	>64	R	>64	R	8	I	<=1	S	>4/76	R	Negative
S	>64	R	>64	R*	<=4	S	<=1	S	>4/76	R	Negative
S	>64	R	>64	R*	<=4	S	<=1	S	>4/76	R	Negative
S	64	I	>64	R*	<=4	S	<=1	S	>4/76	R	Negative
R	>64	R	>64	R*	>8	R	<=1	S	>4/76	R	Negative
S	>64	R	>64	R*	<=4	S	<=1	S	>4/76	R	Negative
I	<=8	S	>64	R*	>8	R	N/R		>4/76	R	Negative
R	>64	R	>64	R	>8	R	<=1	S	>4/76	R	Positive
R	>64	R	>64	R	>8	R	<=1	S	>4/76	R	Positive
S	>64	R	>64	R	<=4	S	<=1	S	>4/76	R	Negative
R	>64	R	>64	R	>8	R	<=1	S	>4/76	R	Positive
S	>64	R	>64	R	<=4	S	<=1	S	>4/76	R	Negative
R	>64	R	>64	R	>8	R	<=1	S	>4/76	R	Positive
S	64	I	>64	R	<=4	S	<=1	S	>4/76	R	Negative
S	>64	R	>64	R	<=4	S	<=1	S	>4/76	R	Positive
S	>64	R	>64	R	<=4	S	<=1	S	>4/76	R	Positive

R	>64	R	>64	R	>8	R	<=1	S	>4/76	R	Negative
R	>64	R	>64	R	>8	R	<=1	S	>4/76	R	Negative
S	>64	R	>64	R	<=4	S	<=1	S	>4/76	R	Negative
R	>64	R	>64	R	>8	R	<=1	S	>4/76	R	Negative
S	>64	R	>64	R	<=4	S	<=1	S	>4/76	R	Negative
I	>64	R	>64	R	8	I	<=1	S	>4/76	R	Positive
S	>64	R	>64	R	<=4	S	<=1	S	>4/76	R	Negative
S	64	I	>64	R	<=4	S	<=1	S	>4/76	R	Negative
R	>64	R	>64	R	>8	R	>2	R	>4/76	R	Negative
I	>64	R	>64	R	>8	R	<=1	S	>4/76	R	Positive
S	64	I	>64	R	<=4	S	<=1	S	>4/76	R	Negative
S	>64	R	>64	R	<=4	S	<=1	S	>4/76	R	Positive
S	>64	R	>64	R	<=4	S	<=1	S	>4/76	R	Positive
S	>64	R	>64	R	8	I	<=1	S	>4/76	R	Negative
R	>64	R	>64	R	>8	R	<=1	S	>4/76	R	Positive
R	>64	R	>64	R	>8	R	2	I	<=2/38	S	Positive
R	>64	R	>64	R	>8	R	<=1	S	>4/76	R	Negative
R	>64	R	>64	R	>8	R	<=1	S	>4/76	R	Positive
S	>64	R	>64	R	8	I	<=1	S	>4/76	R	Negative
R	>64	R	>64	R	>8	R	<=1	S	>4/76	R	Positive
R	64	I	>64	R*	>8	R	<=1	S	>4/76	R	Negative
R	>64	R	>64	R	>8	R	<=1	S	>4/76	R	Positive
S	>64	R	>64	R	<=4	S	<=1	S	>4/76	R	Positive
S	>64	R	>64	R	<=4	S	<=1	S	>4/76	R	Positive

S	64	I	64	I	<=4	S	<=1	S	>4/76	R	Negative
S	<=8	S	>64	R	<=4	S	<=1	S	>4/76	R	Negative
I	>64	R	>64	R	>8	R	<=1	S	>4/76	R	Negative
S	>64	R	>64	R	<=4	S	<=1	S	>4/76	R	Negative
I	>64	R	>64	R	8	I	2	I	>4/76	R	Negative
S	64	I	>64	R	<=4	S	<=1	S	>4/76	R	Negative

S	<=8	S	>64	R	<=4	S	<=1	S	>4/76	R	Negative
S	>64	R	>64	R	<=4	S	<=1	S	>4/76	R	Negative
R	>64	R	>64	R	>8	R	2	I	>4/76	R	Negative
R	64	I	>64	R*	>8	R	>2	R	>4/76	R	Negative

R	>64	R	>64	R	<=4	S	<=1	S	>4/76	R	Negative
I	64	I	>64	R	>8	R	<=1	S	>4/76	R	Negative
R	>64	R	>64	R	>8	R	<=1	S	>4/76	R	Positive

R	>64	R	>64	R	>8	R	>2	R	>4/76	R	Positive
R	>64	R	>64	R	>8	R	<=1	S	>4/76	R	Positive
S	>64	R	>64	R	<=4	S	<=1	S	>4/76	R	Negative
S	>64	R	>64	R	<=4	S	<=1	S	>4/76	R	Negative
S	>64	R	>64	R	<=4	S	<=1	S	>4/76	R	Negative
S	>64	R	>64	R	<=4	S	<=1	S	>4/76	R	Negative
S	>64	R	>64	R	<=4	S	<=1	S	>4/76	R	Negative
S	>64	R	>64	R	<=4	S	<=1	S	>4/76	R	Negative
S	>64	R	>64	R	<=4	S	<=1	S	>4/76	R	Negative
I	>64	R	>64	R	>8	R	<=1	S	>4/76	R	Negative
R	>64	R	>64	R	>8	R	<=1	S	>4/76	R	Positive

R	>64	R	>64	R	>8	R	<=1	S	>4/76	R	Positive
S	64	I	>64	R	>8	R	<=1	S	>4/76	R	Negative
S	>64	R	>64	R	>8	R	<=1	S	<=2/38	S	Negative
R	>64	R	>64	R	>8	R	<=1	S	>4/76	R	Positive
S	>64	R	>64	R	<=4	S	<=1	S	>4/76	R	Positive
S	>64	R	>64	R	<=4	S	<=1	S	>4/76	R	Negative
R	>64	R	>64	R	>8	R	<=1	S	>4/76	R	Positive
R	>64	R	>64	R	>8	R	<=1	S	>4/76	R	Positive
S	>64	R	>64	R	<=4	S	<=1	S	>4/76	R	Negative
S	>64	R	>64	R	<=4	S	<=1	S	>4/76	R	Negative
I	>64	R	>64	R	>8	R	<=1	S	>4/76	R	Negative
S	>64	R	>64	R	<=4	S	<=1	S	>4/76	R	Positive
I	>64	R	>64	R	8	I	2	I	>4/76	R	Positive
R	64	I	>64	R	>8	R	2	I	>4/76	R	Negative
S	>64	R	>64	R	<=4	S	<=1	S	>4/76	R	Negative
S	64	I	>64	R	<=4	S	<=1	S	>4/76	R	Negative
S	>64	R	>64	R	<=4	S	<=1	S	>4/76	R	Negative
S	<=8	S	>64	R*	>8	R	<=1	S	>4/76	R	Negative
S	>64	R	>64	R	<=4	S	<=1	S	>4/76	R	Negative
S	>64	R	>64	R	<=4	S	<=1	S	>4/76	R	Negative
R	>64	R	>64	R	>8	R	<=1	S	>4/76	R	Positive
S	>64	R	>64	R	<=4	S	<=1	S	>4/76	R	Positive
S	>64	R	>64	R	<=4	S	<=1	S	>4/76	R	Negative
R	<=8	S	>64	R	>8	R	N/R		<=2/38	S	Negative
S	>64	R	>64	R	<=4	S	<=1	S	>4/76	R	Negative
I	64	I	>64	R	>8	R	<=1	S	>4/76	R	Negative
S	64	I	>64	R	>8	R	<=1	S	>4/76	R	Negative
S	<=8	S	>64	R	<=4	S	<=1	S	>4/76	R	Negative
S	>64	R	>64	R	<=4	S	<=1	S	>4/76	R	Positive
S	>64	R	>64	R	>8	R	<=1	S	>4/76	R	Negative
I	64	I	>64	R	>8	R	<=1	S	>4/76	R	Negative
S	>64	R	>64	R	<=4	S	<=1	S	>4/76	R	Negative
S	>64	R	>64	R	>8	R	<=1	S	>4/76	R	Positive
R	>64	R	>64	R	>8	R	>2	R	>4/76	R	Negative
S	>64	R	>64	R	<=4	S	<=1	S	>4/76	R	Negative
S	>64	R	>64	R	<=4	S	<=1	S	>4/76	R	Positive
S	<=8	S	>64	R	<=4	S	<=1	S	>4/76	R	Negative
S	>64	R	>64	R	<=4	S	<=1	S	>4/76	R	Negative
S	64	I	>64	R	<=4	S	<=1	S	>4/76	R	Positive
R	>64	R	>64	R	>8	R	<=1	S	>4/76	R	Negative
R	>64	R	>64	R	>8	R	>2	R	>4/76	R	Negative
R	>64	R	>64	R	>8	R	2	I	>4/76	R	Negative
S	64	I	>64	R	<=4	S	<=1	S	<=2/38	S	Negative
I	64	I	>64	R	>8	R	2	I	>4/76	R	Negative

S	>64	R	>64	R	<=4	S	<=1	S	>4/76	R	Positive
R	64	I	16	S	8	I	>2	R	>4/76	R	Positive
S	>64	R	>64	R	<=4	S	<=1	S	>4/76	R	Positive
S	>64	R	>64	R	<=4	S	<=1	S	>4/76	R	Negative
S	>64	R	>64	R	<=4	S	<=1	S	>4/76	R	Negative
R	>64	R	>64	R	>8	R	>2	R	>4/76	R	Negative
S	<=8	S	<=8	S	<=4	S	<=1	S	<=2/38	S	Negative
I	<=8	S	>64	R*	>8	R	2	I	Apr-76	R	Negative
R	>64	R	>64	R	>8	R	2	I	>4/76	R	Positive
S	>64	R	>64	R	<=4	S	<=1	S	>4/76	R	Positive
S	>64	R	>64	R	<=4	S	<=1	S	>4/76	R	Positive
R	>64	R	>64	R	>8	R	<=1	S	>4/76	R	Positive
S	>64	R	>64	R	<=4	S	<=1	S	>4/76	R	Positive
R	>64	R	>64	R	>8	R	<=1	S	>4/76	R	Negative
I	>64	R	>64	R	>8	R	<=1	S	>4/76	R	Negative
R	>64	R	>64	R*	>8	R	>2	R	>4/76	R	Negative
I	>64	R	>64	R	>8	R	<=1	S	>4/76	R	Negative
S	>64	R	>64	R	<=4	S	<=1	S	>4/76	R	Positive
S	>64	R	>64	R	<=4	S	<=1	S	>4/76	R	Negative
I	64	I	>64	R	>8	R	2	I	>4/76	R	Negative
S	>64	R	>64	R	<=4	S	<=1	S	>4/76	R	Positive
S	<=8	S	>64	R	<=4	S	<=1	S	>4/76	R	Negative
S	<=8	S	>64	R	<=4	S	<=1	S	>4/76	R	Negative
R	>64	R	>64	R	>8	R	<=1	S	>4/76	R	Positive
S	>64	R	>64	R	<=4	S	<=1	S	>4/76	R	Positive
S	>64	R	>64	R	<=4	S	<=1	S	>4/76	R	Positive
S	>64	R	>64	R	<=4	S	<=1	S	>4/76	R	Positive
R	>64	R	>64	R	>8	R	<=1	S	>4/76	R	Negative
S	64	I	>64	R	<=4	S	<=1	S	>4/76	R	Negative
S	>64	R	>64	R	<=4	S	<=1	S	>4/76	R	Negative
R	>64	R	>64	R	>8	R	>2	R	>4/76	R	Positive
S	<=8	S	<=8	S	<=4	S	<=1	S	<=2/38	S	Negative
R	64	I	64	I	>8	R	>2	R	>4/76	R	Positive
I	64	I	>64	R	>8	R	2	I	>4/76	R	Negative
S	>64	R	>64	R	<=4	S	<=1	S	>4/76	R	Positive
I	64	I	64	I	>8	R	<=1	S	>4/76	R	Negative
S	64	I	>64	R	>8	R	<=1	S	>4/76	R	Negative
R	>64	R	>64	R	>8	R	2	I	>4/76	R	Positive
S	>64	R	>64	R	<=4	S	<=1	S	>4/76	R	Negative
R	64	I	>64	R	>8	R	>2	R	>4/76	R	Negative
I	64	I	>64	R	>8	R	2	I	>4/76	R	Negative
S	>64	R	>64	R	<=4	S	<=1	S	>4/76	R	Positive
S	64	I	>64	R	>8	R	<=1	S	>4/76	R	Negative
S	>64	R	>64	R	<=4	S	<=1	S	>4/76	R	Positive
S	64	I	>64	R	<=4	S	<=1	S	>4/76	R	Negative
R	>64	R	>64	R	>8	R	<=1	S	>4/76	R	Positive
R	>64	R	>64	R	>8	R	2	I	>4/76	R	Negative
S	>64	R	>64	R	<=4	S	<=1	S	>4/76	R	Positive
S	>64	R	>64	R	<=4	S	<=1	S	>4/76	R	Positive
S	>64	R	>64	R	<=4	S	<=1	S	>4/76	R	Positive
S	>64	R	>64	R	<=4	S	<=1	S	>4/76	R	Positive
S	16	S	64	I	<=4	S	<=1	S	>4/76	R	Negative
S	64	I	>64	R*	<=4	S	<=1	S	>4/76	R	Negative
S	>64	R	>64	R	<=4	S	<=1	S	>4/76	R	Positive
S	64	I	>64	R	<=4	S	<=1	S	>4/76	R	Negative
S	>64	R	>64	R	<=4	S	<=1	S	>4/76	R	Positive

S	>64	R	>64	R	<=4	S	<=1	S	>4/76	R	Positive
I	>64	R	>64	R	>8	R	<=1	S	>4/76	R	Negative
I	64	I	>64	R	>8	R	2	I	>4/76	R	Negative
S	>64	R	>64	R	<=4	S	<=1	S	>4/76	R	Negative
S	>64	R	>64	R	<=4	S	<=1	S	>4/76	R	Negative
R	>64	R	>64	R	>8	R	<=1	S	>4/76	R	Positive
S	>64	R	>64	R	<=4	S	<=1	S	>4/76	R	Negative
I	64	I	>64	R	>8	R	<=1	S	>4/76	R	Negative
S	>64	R	>64	R	<=4	S	<=1	S	>4/76	R	Negative
S	>64	R	>64	R	<=4	S	<=1	S	>4/76	R	Negative
S	<=8	S	16	S	8	I	<=1	S	<=2/38	S	Negative
I	64	I	>64	R	>8	R	<=1	S	>4/76	R	Positive
I	>64	R	>64	R	>8	R	<=1	S	>4/76	R	Negative
S	>64	R	>64	R	>8	R	<=1	S	>4/76	R	Positive

I	64	I	>64	R	>8	R	<=1	S	>4/76	R	Negative
S	>64	R	>64	R	<=4	S	<=1	S	>4/76	R	Negative
S	>64	R	>64	R	8	I	<=1	S	<=2/38	S	Negative
S	64	I	>64	R	<=4	S	<=1	S	>4/76	R	Negative
S	64	I	>64	R*	>8	R	<=1	S	>4/76	R	Negative
I	>64	R	>64	R	8	I	2	I	>4/76	R	Negative
S	64	I	64	I	<=4	S	<=1	S	<=2/38	S	Negative
S	>64	R	>64	R	<=4	S	<=1	S	>4/76	R	Positive
S	>64	R	>64	R	8	I	<=1	S	>4/76	R	Positive
S	64	I	>64	R	<=4	S	<=1	S	>4/76	R	Negative
I	64	I	>64	R	>8	R	>2	R	>4/76	R	Negative
S	>64	R	64	I	8	I	<=1	S	<=2/38	S	Negative
S	<=8	S	<=8	S	<=4	S	<=1	S	<=2/38	S	Negative
S	>64	R	>64	R	<=4	S	<=1	S	<=2/38	S	Positive
R	>64	R	>64	R	>8	R	>2	R	>4/76	R	Negative
S	>64	R	>64	R	<=4	S	<=1	S	<=2/38	S	Positive
S	>64	R	>64	R	8	I	2	I	>4/76	R	Negative
S	>64	R	>64	R	8	I	2	I	>4/76	R	Negative
S	>64	R	>64	R	<=4	S	<=1	S	>4/76	R	Positive
S	>64	R	>64	R	<=4	S	<=1	S	>4/76	R	Negative
S	>64	R	>64	R	<=4	S	<=1	S	>4/76	R	Positive
S	>64	R	>64	R	<=4	S	<=1	S	>4/76	R	Negative
S	<=8	S	<=8	S	<=4	S	<=1	S	<=2/38	S	Negative
S	>64	R	>64	R	>8	R	<=1	S	>4/76	R	Positive
I	>64	R	>64	R	>8	R	2	I	>4/76	R	Negative
S	64	I	>64	R	>8	R	<=1	S	>4/76	R	Negative
R	>64	R	>64	R	>8	R	2	I	>4/76	R	Negative
S	>64	R	>64	R	<=4	S	<=1	S	>4/76	R	Negative
S	>64	R	>64	R	<=4	S	<=1	S	>4/76	R	Negative
R	>64	R	>64	R	>8	R	>2	R	>4/76	R	Negative
S	>64	R	>64	R	8	I	<=1	S	<=2/38	S	Negative
S	>64	R	>64	R	8	I	<=1	S	<=2/38	S	Negative

S	64	I	>64	R	<=4	S	<=1	S	>4/76	R	Negative
S	>64	R	>64	R	8	I	<=1	S	>4/76	R	Negative
R	>64	R	>64	R	>8	R	<=1	S	>4/76	R	Positive
R	>64	R	>64	R	>8	R	<=1	S	>4/76	R	Positive
I	>64	R	>64	R	>8	R	<=1	S	>4/76	R	Negative
R	>64	R	>64	R	>8	R	>2	R	>4/76	R	Negative
S	>64	R	>64	R	<=4	S	2	I	>4/76	R	Negative
S	>64	R	>64	R	<=4	S	<=1	S	>4/76	R	Negative
S	64	I	>64	R	>8	R	<=1	S	>4/76	R	Negative
S	>64	R	>64	R	8	I	2	I	>4/76	R	Negative
R	>64	R	>64	R	>8	R	<=1	S	>4/76	R	Negative
S	>64	R	>64	R	>8	R	<=1	S	>4/76	R	Negative
S	64	I	>64	R	<=4	S	<=1	S	>4/76	R	Negative
S	>64	R	>64	R	<=4	S	<=1	S	>4/76	R	Negative
S	64	I	>64	R	<=4	S	<=1	S	>4/76	R	Negative
S	>64	R	>64	R	<=4	S	<=1	S	>4/76	R	Negative
S	>64	R	>64	R	8	I	<=1	S	>4/76	R	Negative
S	>64	R	>64	R	<=4	S	<=1	S	>4/76	R	Negative
S	>64	R	>64	R	<=4	S	<=1	S	>4/76	R	Negative
R	>64	R	>64	R	>8	R	2	I	>4/76	R	Positive
I	>64	R	>64	R	8	I	2	I	>4/76	R	Negative
I	>64	R	>64	R	>8	R	<=1	S	>4/76	R	Negative
R	>64	R	>64	R	>8	R	2	I	>4/76	R	Negative
I	>64	R	>64	R	>8	R	<=1	S	>4/76	R	Negative
S	64	I	64	I	8	I	<=1	S	<=2/38	S	Negative
S	>64	R	>64	R	8	I	<=1	S	<=2/38	S	Negative
S	64	I	>64	R*	<=4	S	<=1	S	>4/76	R	Negative
S	>64	R	>64	R	<=4	S	<=1	S	>4/76	R	Positive
S	>64	R	>64	R	8	I	<=1	S	<=2/38	S	Negative
R	>64	R	>64	R*	>8	R	<=1	S	>4/76	R	Negative
S	>64	R	>64	R	<=4	S	<=1	S	<=2/38	S	Negative
R	>64	R	>64	R*	>8	R	<=1	S	>4/76	R	Positive
S	>64	R	>64	R*	<=4	S	<=1	S	>4/76	R	Positive
S	>64	R	>64	R*	8	I	2	I	>4/76	R	Positive
R	>64	R	>64	R	>8	R	<=1	S	>4/76	R	Positive
I	<=8	S	>64	R*	>8	R	<=1	S	>4/76	R	Negative
R	>64	R	>64	R	>8	R	<=1	S	>4/76	R	Positive
S	>64	R	>64	R*	8	I	2	I	>4/76	R	Negative
R	>64	R	>64	R	>8	R	<=1	S	>4/76	R	Positive
R	64	I	>64	R*	>8	R	>2	R	>4/76	R	Negative
S	>64	R	>64	R	8	I	2	I	>4/76	R	Positive
S	>64	R	>64	R	<=4	S	<=1	S	>4/76	R	Positive
R	>64	R	>64	R	>8	R	<=1	S	>4/76	R	Positive
S	>64	R	>64	R	8	I	2	I	>4/76	R	Positive
R	>64	R	>64	R	>8	R	<=1	S	>4/76	R	Positive
S	>64	R	>64	R*	8	I	2	I	>4/76	R	Negative
S	>64	R	>64	R*	8	I	2	I	>4/76	R	Negative
S	>64	R	>64	R	<=4	S	<=1	S	>4/76	R	Negative
S	>64	R	>64	R	<=4	S	<=1	S	>4/76	R	Positive

I	>64	R	>64	R	>8	R	<=1	S	>4/76	R	Negative
S	>64	R	>64	R*	<=4	S	<=1	S	>4/76	R	Negative
S	>64	R	>64	R	<=4	S	<=1	S	<=2/38	S	Negative
S	>64	R	>64	R	<=4	S	<=1	S	>4/76	R	Negative
S	64	I	64	I	8	I	<=1	S	<=2/38	S	Negative
S	>64	R	>64	R*	<=4	S	<=1	S	>4/76	R	Negative
S	>64	R	>64	R	<=4	S	<=1	S	>4/76	R	Positive
R	>64	R	>64	R	>8	R	>2	R	>4/76	R	Positive
R	>64	R	>64	R	>8	R	<=1	S	>4/76	R	Positive
R	>64	R	>64	R	>8	R	<=1	S	>4/76	R	Positive
S	64	I	64	R*	<=4	S	<=1	S	>4/76	R	Negative
R	>64	R	>64	R*	8	I	2	I	>4/76	R	Negative
R	>64	R	>64	R*	>8	R	>2	R	>4/76	R	Negative
S	>64	R	>64	R	<=4	S	<=1	S	>4/76	R	Positive
I	>64	R	>64	R*	>8	R	2	I	>4/76	R	Negative
R	>64	R	>64	R	>8	R	<=1	S	>4/76	R	Positive
I	>64	R	>64	R	8	I	<=1	S	>4/76	R	Positive
S	>64	R	>64	R	<=4	S	<=1	S	<=2/38	S	Positive
R	>64	R	>64	R*	>8	R	<=1	S	>4/76	R	Positive
S	>64	R	>64	R	<=4	S	<=1	S	>4/76	R	Positive
R	>64	R	>64	R	>8	R	>2	R	>4/76	R	Negative
S	>64	R	>64	R	<=4	S	<=1	S	<=2/38	S	Negative
S	>64	R	>64	R*	>8	R	<=1	S	>4/76	R	Negative
S	16	S	64	I	<=4	S	<=1	S	<=2/38	S	Negative
S	>64	R	>64	R	<=4	S	<=1	S	>4/76	R	Positive
R	>64	R	>64	R	>8	R	<=1	S	>4/76	R	Positive
S	>64	R	>64	R*	<=4	S	<=1	S	>4/76	R	Positive

R	16	S	>64	R*	>8	R	<=1	S	>4/76	R	Negative
S	>64	R	>64	R	8	I	<=1	S	<=2/38	S	Positive
R	>64	R	>64	R*	>8	R	2	I	>4/76	R	Negative
S	>64	R	>64	R*	>8	R	<=1	S	>4/76	R	Positive
S	>64	R	>64	R*	>8	R	<=1	S	>4/76	R	Positive
S	>64	R	>64	R	>8	R	<=1	S	>4/76	R	Positive
S	>64	R	>64	R*	>8	R	<=1	S	>4/76	R	Negative
S	64	I	>64	R	<=4	S	<=1	S	>4/76	R	Positive
S	>64	R	>64	R*	<=4	S	<=1	S	>4/76	R	Positive
R	>64	R	>64	R*	>8	R	<=1	S	>4/76	R	Positive
S	64	I	>64	R	<=4	S	<=1	S	>4/76	R	Negative
S	64	I	64	I	<=4	S	<=1	S	<=2/38	S	Negative
R	>64	R	>64	R	>8	R	<=1	S	>4/76	R	Positive
R	>64	R	>64	R	>8	R	>2	R	>4/76	R	Negative

S	>64	R	>64	R	<=4	S	<=1	S	>4/76	R	Negative
S	>64	R	64	I	8	I	<=1	S	<=2/38	S	Negative
R	64	I	>64	R*	>8	R	>2	R	>4/76	R	Negative
R	64	I	>64	R	>8	R	>2	R	<=2/38	S	Positive
S	>64	R	>64	R*	<=4	S	2	I	>4/76	R	Negative
S	>64	R	>64	R	<=4	S	<=1	S	>4/76	R	Positive
I	>64	R	>64	R	>8	R	2	I	>4/76	R	Positive

I	>64	R	>64	R*	8	I	2	I	>4/76	R	Negative
I	64	I	>64	R*	>8	R	>2	R	>4/76	R	Negative
I	>64	R	>64	R	8	I	2	I	<=2/38	S	Negative
S	>64	R	>64	R*	8	I	2	I	>4/76	R	Negative
I	>64	R	>64	R*	>8	R	2	I	>4/76	R	Negative
S	>64	R	>64	R*	<=4	S	<=1	S	>4/76	R	Negative
S	>64	R	>64	R*	8	I	<=1	S	>4/76	R	Negative
I	>64	R	>64	R*	8	I	2	I	>4/76	R	Negative
S	>64	R	>64	R*	<=4	S	<=1	S	>4/76	R	Negative
S	>64	R	>64	R*	8	I	2	I	>4/76	R	Negative
S	64	I	>64	R*	<=4	S	<=1	S	>4/76	R	Negative
S	>64	R	>64	R*	8	I	<=1	S	>4/76	R	Negative
S	64	I	>64	R	<=4	S	<=1	S	>4/76	R	Negative
S	>64	R	>64	R*	>8	R	<=1	S	>4/76	R	Negative
S	>64	R	>64	R*	>8	R	<=1	S	>4/76	R	Negative
S	>64	R	>64	R*	8	I	2	I	>4/76	R	Negative
R	>64	R	>64	R	>8	R	<=1	S	>4/76	R	Positive
R	>64	R	>64	R*	>8	R	2	I	>4/76	R	Negative
S	>64	R	>64	R*	8	I	<=1	S	>4/76	R	Negative
S	>64	R	>64	R*	<=4	S	<=1	S	>4/76	R	Negative

S	>64	R	>64	R*	<=4	S	<=1	S	>4/76	R	Negative
S	>64	R	>64	R*	8	I	<=1	S	>4/76	R	Negative
I	>64	R	>64	R*	>8	R	2	I	>4/76	R	Negative
S	>64	R	>64	R	<=4	S	<=1	S	>4/76	R	Negative
I	>64	R	>64	R	>8	R	2	I	>4/76	R	Positive
S	>64	R	>64	R	>8	R	<=1	S	>4/76	R	Positive
I	>64	R	>64	R*	>8	R	2	I	>4/76	R	Negative
I	>64	R	>64	R*	8	I	2	I	>4/76	R	Negative
S	>64	R	>64	R*	<=4	S	<=1	S	>4/76	R	Negative
I	>64	R	>64	R*	>8	R	2	I	>4/76	R	Negative
S	>64	R	>64	R	<=4	S	<=1	S	<=2/38	S	Negative
S	64	I	64	I	<=4	S	<=1	S	<=2/38	S	Negative
S	>64	R	>64	R*	<=4	S	<=1	S	>4/76	R	Negative
R	>64	R	>64	R	>8	R	2	I	>4/76	R	Negative
I	>64	R	>64	R*	8	I	2	I	>4/76	R	Negative
S	>64	R	>64	R*	<=4	S	<=1	S	>4/76	R	Negative
S	>64	R	>64	R*	<=4	S	<=1	S	>4/76	R	Negative
S	>64	R	>64	R*	<=4	S	<=1	S	>4/76	R	Negative
R	64	I	>64	R	>8	R	>2	R	>4/76	R	Positive
S	<=8	S	64	I	<=4	S	<=1	S	<=2/38	S	Negative
S	>64	R	>64	R	<=4	S	<=1	S	>4/76	R	Negative
S	>64	R	>64	R	8	I	<=1	S	>4/76	R	Negative
I	>64	R	>64	R*	8	I	2	I	>4/76	R	Negative
S	>64	R	>64	R*	<=4	S	<=1	S	>4/76	R	Negative
I	64	I	>64	R	>8	R	2	I	<=2/38	S	Negative
I	64	I	64	I	>8	R	<=1	S	>4/76	R	Positive
I	>64	R	>64	R*	>8	R	<=1	S	>4/76	R	Negative

S	>64	R	>64	R	<=4	S	<=1	S	>4/76	R	Positive
S	>64	R	>64	R	<=4	S	<=1	S	>4/76	R	Positive
I	>64	R	>64	R	>8	R	<=1	S	>4/76	R	Positive
R	>64	R	>64	R	>8	R	>2	R	>4/76	R	Positive
I	>64	R	>64	R	>8	R	<=1	S	>4/76	R	Positive
S	>64	R	>64	R	<=4	S	<=1	S	>4/76	R	Positive
S	>64	R	>64	R*	<=4	S	<=1	S	>4/76	R	Negative
S	>64	R	>64	R*	<=4	S	2	I	>4/76	R	Negative
I	>64	R	>64	R	>8	R	<=1	S	>4/76	R	Positive
R	>64	R	>64	R	>8	R	<=1	S	>4/76	R	Negative
S	>64	R	>64	R	<=4	S	<=1	S	>4/76	R	Positive
R	16	S	64	I	>8	R	2	I	<=2/38	S	Negative
S	>64	R	>64	R	>8	R	<=1	S	>4/76	R	Negative
R	>64	R	>64	R	>8	R	2	I	>4/76	R	Negative
S	64	I	>64	R	<=4	S	<=1	S	>4/76	R	Negative
R	>64	R	>64	R	>8	R	<=1	S	>4/76	R	Positive
S	>64	R	>64	R*	8	I	2	I	>4/76	R	Negative
I	>64	R	>64	R	>8	R	2	I	>4/76	R	Negative
S	>64	R	>64	R*	<=4	S	<=1	S	>4/76	R	Negative
R	>64	R	>64	R	>8	R	<=1	S	>4/76	R	Positive
S	>64	R	>64	R*	>8	R	<=1	S	>4/76	R	Negative
I	>64	R	>64	R	>8	R	2	I	>4/76	R	Negative
R	>64	R	>64	R	>8	R	<=1	S	>4/76	R	Positive
I	>64	R	>64	R*	>8	R	<=1	S	>4/76	R	Negative
R	>64	R	>64	R	>8	R	<=1	S	>4/76	R	Positive
S	>64	R	>64	R	<=4	S	<=1	S	>4/76	R	Negative
S	64	I	>64	R*	>8	R	<=1	S	>4/76	R	Negative
S	>64	R	>64	R	8	I	<=1	S	>4/76	R	Negative
I	>64	R	>64	R	>8	R	<=1	S	>4/76	R	Negative
S	>64	R	>64	R*	8	I	2	I	>4/76	R	Negative
S	>64	R	>64	R*	8	I	2	I	>4/76	R	Negative
S	>64	R	>64	R*	8	I	2	I	>4/76	R	Negative
S	>64	R	>64	R	<=4	S	<=1	S	>4/76	R	Negative
R	>64	R	>64	R*	>8	R	<=1	S	>4/76	R	Negative
S	>64	R	>64	R*	<=4	S	<=1	S	>4/76	R	Negative
S	>64	R	>64	R*	<=4	S	<=1	S	>4/76	R	Negative
S	>64	R	>64	R*	<=4	S	<=1	S	>4/76	R	Negative
S	>64	R	>64	R	<=4	S	<=1	S	>4/76	R	Positive
S	>64	R	>64	R	>8	R	<=1	S	>4/76	R	Positive
R	64	I	>64	R	>8	R	>2	R	>4/76	R	Positive
S	>64	R	>64	R	>8	R	2	I	>4/76	R	Positive

R	>64	R	>64	R	>8	R	<=1	S	>4/76	R	Positive
S	>64	R	>64	R*	<=4	S	<=1	S	>4/76	R	Negative
R	>64	R	>64	R*	>8	R	>2	R	>4/76	R	Negative

S	>64	R	>64	R	>8	R	<=1	S	<=2/38	S	Negative
S	>64	R	>64	R*	<=4	S	<=1	S	>4/76	R	Negative
I	>64	R	>64	R*	>8	R	2	I	>4/76	R	Negative
I	64	I	>64	R*	>8	R	<=1	S	>4/76	R	Negative
R	>64	R	>64	R*	>8	R	>2	R	>4/76	R	Negative
S	>64	R	>64	R	<=4	S	<=1	S	>4/76	R	Positive
I	>64	R	>64	R	>8	R	<=1	S	>4/76	R	Negative
R	>64	R	>64	R	>8	R	<=1	S	>4/76	R	Negative
S	>64	R	>64	R	<=4	S	<=1	S	>4/76	R	Negative
S	>64	R	>64	R*	<=4	S	<=1	S	>4/76	R	Negative
S	>64	R	>64	R*	<=4	S	<=1	S	>4/76	R	Negative
I	64	I	>64	R*	>8	R	<=1	S	>4/76	R	Negative
S	>64	R	>64	R*	<=4	S	<=1	S	>4/76	R	Negative
R	>64	R	>64	R	>8	R	<=1	S	>4/76	R	Negative
S	>64	R	>64	R	<=4	S	<=1	S	>4/76	R	Positive
S	<=8	S	>64	R*	<=4	S	<=1	S	>4/76	R	Negative
R	>64	R	>64	R	>8	R	<=1	S	>4/76	R	Positive
S	>64	R	>64	R	<=4	S	<=1	S	>4/76	R	Negative
R	64	I	>64	R*	>8	R	<=1	S	>4/76	R	Negative
S	64	I	>64	R	<=4	S	<=1	S	>4/76	R	Negative
S	>64	R	>64	R	<=4	S	<=1	S	>4/76	R	Positive
R	>64	R	>64	R*	>8	R	2	I	>4/76	R	Negative
S	64	I	>64	R	<=4	S	2	I	>4/76	R	Positive
I	>64	R	>64	R*	>8	R	<=1	S	>4/76	R	Negative
S	>64	R	>64	R	>8	R	<=1	S	>4/76	R	Positive
R	>64	R	>64	R	>8	R	<=1	S	>4/76	R	Positive
I	>64	R	>64	R*	8	I	2	I	>4/76	R	Negative
I	>64	R	>64	R	>8	R	<=1	S	>4/76	R	Negative
I	>64	R	>64	R*	>8	R	<=1	S	>4/76	R	Negative
R	>64	R	>64	R	>8	R	<=1	S	>4/76	R	Positive
S	>64	R	>64	R*	<=4	S	<=1	S	>4/76	R	Negative
S	<=8	S	>64	R	>8	R	<=1	S	>4/76	R	Negative
I	>64	R	>64	R	>8	R	<=1	S	>4/76	R	Positive
S	64	I	>64	R	<=4	S	<=1	S	>4/76	R	Negative
I	>64	R	>64	R	>8	R	2	I	>4/76	R	Negative
I	>64	R	>64	R	>8	R	2	I	>4/76	R	Positive
S	>64	R	>64	R*	<=4	S	<=1	S	>4/76	R	Negative
I	>64	R	>64	R	>8	R	2	I	>4/76	R	Negative
S	>64	R	>64	R	<=4	S	<=1	S	>4/76	R	Positive
S	>64	R	>64	R*	<=4	S	<=1	S	>4/76	R	Negative
S	>64	R	>64	R*	<=4	S	<=1	S	>4/76	R	Negative
S	>64	R	>64	R*	<=4	S	<=1	S	>4/76	R	Negative
I	>64	R	>64	R	>8	R	<=1	S	>4/76	R	Negative
R	>64	R	>64	R	>8	R	>2	R	>4/76	R	Positive
S	>64	R	>64	R	>8	R	<=1	S	<=2/38	S	Negative
S	64	I	>64	R*	<=4	S	<=1	S	>4/76	R	Negative
S	>64	R	>64	R*	8	I	2	I	>4/76	R	Negative
S	<=8	S	<=8	S	<=4	S	<=1	S	<=2/38	S	Negative

S	>64	R	>64	R	<=4	S	<=1	S	>4/76	R	Positive
I	>64	R	>64	R	>8	R	<=1	S	>4/76	R	Negative
S	>64	R	>64	R*	<=4	S	<=1	S	>4/76	R	Negative
S	>64	R	>64	R	<=4	S	<=1	S	>4/76	R	Positive
S	>64	R	>64	R*	<=4	S	<=1	S	>4/76	R	Negative
S	<=8	S	>64	R*	<=4	S	<=1	S	>4/76	R	Negative
S	>64	R	>64	R	>8	R	<=1	S	<=2/38	S	Negative
S	>64	R	>64	R*	<=4	S	<=1	S	>4/76	R	Negative
I	>64	R	>64	R	>8	R	<=1	S	>4/76	R	Negative
R	>64	R	>64	R	>8	R	<=1	S	>4/76	R	Negative
R	64	I	>64	R	>8	R	>2	R	>4/76	R	Negative
S	<=8	S	>64	R	<=4	S	<=1	S	<=2/38	S	Negative
R	>64	R	>64	R*	>8	R	<=1	S	>4/76	R	Negative
R	>64	R	>64	R	>8	R	<=1	S	Apr-76	R	Positive
S	>64	R	>64	R*	<=4	S	<=1	S	>4/76	R	Negative
S	64	I	>64	R*	<=4	S	<=1	S	>4/76	R	Negative
S	>64	R	>64	R*	<=4	S	<=1	S	>4/76	R	Negative
S	<=8	S	>64	R	<=4	S	<=1	S	>4/76	R	Negative
S	>64	R	>64	R	<=4	S	<=1	S	>4/76	R	Positive

S	>64	R	>64	R	<=4	S	<=1	S	>4/76	R	Positive
R	>64	R	>64	R	>8	R	<=1	S	>4/76	R	Negative
S	>64	R	>64	R	>8	R	<=1	S	>4/76	R	Negative
S	>64	R	>64	R*	<=4	S	<=1	S	>4/76	R	Negative
S	>64	R	>64	R*	<=4	S	<=1	S	>4/76	R	Negative
S	>64	R	>64	R	>8	R	<=1	S	>4/76	R	Positive
R	>64	R	>64	R	>8	R	<=1	S	>4/76	R	Positive
S	64	I	>64	R	<=4	S	<=1	S	>4/76	R	Negative
S	64	I	>64	R*	<=4	S	<=1	S	>4/76	R	Negative
R	>64	R	>64	R	>8	R	<=1	S	>4/76	R	Positive
R	>64	R	>64	R	>8	R	<=1	S	>4/76	R	Positive
R	>64	R	>64	R	>8	R	<=1	S	>4/76	R	Negative
R	16	S	64	I	>8	R	2	I	<=2/38	S	Negative
S	>64	R	>64	R	<=4	S	<=1	S	>4/76	R	Positive
R	<=8	S	>64	R*	>8	R	N/R		>4/76	R	Negative

S	>64	R	>64	R	<=4	S	<=1	S	>4/76	R	Positive
S	>64	R	>64	R	<=4	S	<=1	S	>4/76	R	Negative
S	>64	R	>64	R	<=4	S	<=1	S	>4/76	R	Positive
S	64	I	>64	R*	>8	R	<=1	S	>4/76	R	Negative
R	16	S	>64	R	>8	R	N/R		<=2/38	S	Negative
S	>64	R	>64	R	<=4	S	<=1	S	>4/76	R	Negative
S	>64	R	>64	R	<=4	S	<=1	S	>4/76	R	Positive
S	>64	R	>64	R*	<=4	S	<=1	S	>4/76	R	Negative
S	>64	R	>64	R	<=4	S	<=1	S	>4/76	R	Positive
I	>64	R	>64	R	8	I	<=1	S	>4/76	R	Negative

S	>64	I	>64	R	<=4	S	<=1	S	<=2/38	S	Negative

R	>64	R	>64	R	>8	R	<=1	S	>4/76	R	Negative
S	>64	R	>64	R	8	I	<=1	S	>4/76	R	Positive
I	<=8	S	64	I	<=4	S	<=1	S	<=2/38	S	Negative
S	>64	R	>64	R	<=4	S	<=1	S	>4/76	R	Positive
S	>64	R	>64	R	<=4	S	<=1	S	>4/76	R	Negative
S	>64	R	>64	R	<=4	S	<=1	S	>4/76	R	Positive
S	64	I	>64	R*	<=4	S	<=1	S	<=2/38	S	Negative
S	>64	R	>64	R	<=4	S	<=1	S	>4/76	R	Positive
S	>64	R	>64	R*	8	I	2	I	>4/76	R	Negative
R	>64	R	>64	R	>8	R	<=1	S	>4/76	R	Positive
S	>64	R	>64	R	<=4	S	<=1	S	>4/76	R	Positive
S	>64	R	>64	R	<=4	S	<=1	S	<=2/38	S	Positive
S	>64	R	>64	R	<=4	S	<=1	S	>4/76	R	Positive
S	>64	R	>64	R	<=4	S	<=1	S	>4/76	R	Positive
R	>64	R	>64	R	>8	R	<=1	S	>4/76	R	Positive
I	>64	R	>64	R	>8	R	2	I	>4/76	R	Negative
S	>64	R	>64	R*	<=4	S	<=1	S	>4/76	R	Negative
R	>64	R	>64	R	>8	R	<=1	S	>4/76	R	Negative
S	>64	R	>64	R	>8	R	<=1	S	<=2/38	S	Negative
I	>64	R	>64	R	>8	R	2	I	>4/76	R	Negative
S	>64	R	>64	R	<=4	S	<=1	S	<=2/38	S	Positive
I	>64	R	>64	R	>8	R	2	I	>4/76	R	Negative
I	64	I	>64	R	>8	R	2	I	<=2/38	S	Positive
S	>64	R	>64	R	<=4	S	<=1	S	<=2/38	S	Positive
R	>64	R	>64	R	>8	R	<=1	S	>4/76	R	Positive
S	<=8	S	<=8	S	<=4	S	<=1	S	<=2/38	S	Negative
I	64	I	64	I	>8	R	2	I	<=2/38	S	Negative
I	>64	R	>64	R	>8	R	<=1	S	>4/76	R	Negative
I	>64	R	>64	R	>8	R	<=1	S	>4/76	R	Positive
R	64	I	>64	R*	>8	R	<=1	S	>4/76	R	Negative
S	64	I	>64	R	<=4	S	<=1	S	<=2/38	S	Negative
S	>64	R	>64	R	>8	R	<=1	S	>4/76	R	Positive
S	>64	R	>64	R	<=4	S	<=1	S	>4/76	R	Positive
S	>64	R	>64	R	<=4	S	<=1	S	>4/76	R	Positive
S	>64	R	>64	R	<=4	S	<=1	S	>4/76	R	Positive
R	>64	R	>64	R*	>8	R	>2	R	>4/76	R	Negative
S	>64	R	>64	R*	>8	R	>2	R	>4/76	R	Negative
R	>64	R	>64	R*	>8	R	>2	R	>4/76	R	Negative
S	>64	R	>64	R*	>8	R	<=1	S	>4/76	R	Negative
S	64	I	>64	R	<=4	S	<=1	S	<=2/38	S	Negative
R	>64	R	>64	R*	>8	R	>2	R	>4/76	R	Negative
R	>64	R	>64	R	>8	R	2	I	>4/76	R	Negative
S	>64	R	>64	R	<=4	S	<=1	S	>4/76	R	Negative
S	>64	R	>64	R	>8	R	<=1	S	>4/76	R	Positive

S	>64	R	>64	R*	>8	R	<=1	S	>4/76	R	Negative
S	64	I	64	I	<=4	S	<=1	S	>4/76	R	Negative
R	<=8	S	>64	R*	>8	R	N/R		>4/76	R	Negative
R	>64	R	>64	R	8	I	2	I	>4/76	R	Negative
S	>64	R	>64	R*	8	I	2	I	>4/76	R	Negative
R	>64	R	>64	R*	>8	R	2	I	>4/76	R	Negative
R	>64	R	>64	R	>8	R	2	I	>4/76	R	Negative
R	>64	R	>64	R	<=4	S	<=1	S	>4/76	R	Negative
R	>64	R	>64	R	>8	R	>2	R	>4/76	R	Negative
S	<=8	S	<=8	S	>8	R	<=1	S	<=2/38	S	Negative
S	>64	R	>64	R	<=4	S	<=1	S	>4/76	R	Negative
R	>64	R	>64	R	>8	R	<=1	S	>4/76	R	Negative
R	>64	R	>64	R	>8	R	>2	R	>4/76	R	Positive
R	>64	R	>64	R	>8	R	2	I	>4/76	R	Positive
I	<=8	S	<=8	S	>8	R	2	I	>4/76	R	Negative
R	>64	R	>64	R*	>8	R	2	I	>4/76	R	Negative
I	>64	R	>64	R	>8	R	2	I	>4/76	R	Negative
S	>64	R	>64	R*	>8	R	<=1	S	>4/76	R	Negative
S	<=8	S	>64	R*	>8	R	<=1	S	>4/76	R	Negative
S	64	I	>64	R*	>8	R	<=1	S	>4/76	R	Negative
S	>64	R	>64	R*	<=4	S	<=1	S	>4/76	R	Negative
I	>64	R	>64	R	>8	R	2	I	>4/76	R	Positive

S	>64	R	>64	R	<=4	S	<=1	S	>4/76	R	Negative
R	>64	R	>64	R	>8	R	>2	R	>4/76	R	Positive
R	>64	R	>64	R	>8	R	<=1	S	>4/76	R	Positive
R	>64	R	>64	R	>8	R	2	I	>4/76	R	Negative
S	>64	R	>64	R	>8	R	<=1	S	>4/76	R	Positive
S	>64	R	>64	R	<=4	S	<=1	S	>4/76	R	Negative
R	>64	R	>64	R	>8	R	2	I	>4/76	R	Negative
R	>64	R	>64	R*	>8	R	>2	R	>4/76	R	Negative
S	>64	R	>64	R	<=4	S	<=1	S	>4/76	R	Negative
R	>64	R	>64	R	>8	R	>2	R	>4/76	R	Negative
R	>64	R	>64	R	8	I	>2	R	>4/76	R	Negative
I	>64	R	>64	R	8	I	>2	R	>4/76	R	Negative
I	>64	R	>64	R	>8	R	<=1	S	>4/76	R	Positive
S	>64	R	>64	R	<=4	S	<=1	S	>4/76	R	Negative
S	>64	R	>64	R*	>8	R	2	I	>4/76	R	Negative
R	>64	R	>64	R	>8	R	2	I	>4/76	R	Negative
S	>64	R	>64	R*	<=4	S	<=1	S	>4/76	R	Negative
S	>64	R	>64	R	<=4	S	<=1	S	>4/76	R	Negative
S	>64	R	>64	R*	8	I	2	I	>4/76	R	Negative
S	>64	R	>64	R	>8	R	2	I	>4/76	R	Negative
R	>64	R	>64	R	>8	R	>2	R	>4/76	R	Negative
S	>64	R	>64	R	<=4	S	<=1	S	>4/76	R	Positive
I	>64	R	>64	R	8	I	2	I	>4/76	R	Positive
S	>64	R	>64	R*	8	I	<=1	S	>4/76	R	Negative
S	>64	R	>64	R	<=4	S	<=1	S	>4/76	R	Positive
R	>64	R	>64	R	>8	R	2	I	>4/76	R	Positive
S	>64	R	>64	R	<=4	S	<=1	S	<=2/38	S	Positive
I	>64	R	>64	R	8	I	>2	R	>4/76	R	Negative
S	>64	R	>64	R	<=4	S	<=1	S	>4/76	R	Positive
S	>64	R	>64	R*	<=4	S	<=1	S	>4/76	R	Negative
R	>64	R	>64	R	>8	R	>2	R	>4/76	R	Negative
S	64	I	>64	R*	<=4	S	<=1	S	>4/76	R	Negative
S	64	I	>64	R	<=4	S	2	I	>4/76	R	Positive

I	>64	R	>64	R*	>8	R	2	I	>4/76	R	Negative
R	>64	R	>64	R*	>8	R	<=1	S	>4/76	R	Negative
S	>64	R	>64	R*	<=4	S	<=1	S	>4/76	R	Negative

S	>64	R	>64	R	<=4	S	<=1	S	>4/76	R	Positive
S	>64	R	>64	R*	<=4	S	<=1	S	>4/76	R	Negative
I	>64	R	>64	R*	>8	R	2	I	>4/76	R	Negative
S	>64	R	>64	R	<=4	S	<=1	S	<=2/38	S	Positive
S	>64	R	>64	R	<=4	S	<=1	S	>4/76	R	Positive
I	>64	R	>64	R*	>8	R	<=1	S	>4/76	R	Negative
S	>64	R	>64	R	<=4	S	<=1	S	>4/76	R	Negative
I	64	I	>64	R	>8	R	2	I	>4/76	R	Negative
S	>64	R	>64	R	<=4	S	<=1	S	>4/76	R	Positive
S	>64	R	>64	R*	<=4	S	<=1	S	>4/76	R	Negative
R	>64	R	>64	R	>8	R	<=1	S	>4/76	R	Positive
R	>64	R	>64	R	>8	R	<=1	S	>4/76	R	Positive
S	>64	R	>64	R	<=4	S	<=1	S	>4/76	R	Positive
S	>64	R	>64	R	>8	R	<=1	S	>4/76	R	Negative
R	>64	R	>64	R	>8	R	<=1	S	>4/76	R	Negative
S	>64	R	>64	R	<=4	S	<=1	S	>4/76	R	Positive
S	>64	R	>64	R	8	I	<=1	S	>4/76	R	Negative
S	>64	R	>64	R	<=4	S	<=1	S	<=2/38	S	Positive
I	16	S	64	I	8	I	>2	R	>4/76	R	Negative
I	16	S	64	I	8	I	>2	R	>4/76	R	Negative
R	>64	R	>64	R	>8	R	<=1	S	>4/76	R	Positive
S	>64	R	>64	R	<=4	S	<=1	S	>4/76	R	Negative
S	>64	R	>64	R	>8	R	<=1	S	>4/76	R	Positive
R	>64	R	>64	R	>8	R	<=1	S	>4/76	R	Negative
S	>64	R	>64	R	<=4	S	<=1	S	>4/76	R	Negative
S	>64	R	>64	R	<=4	S	<=1	S	>4/76	R	Negative
S	>64	R	>64	R	>8	R	<=1	S	>4/76	R	Negative
S	>64	R	>64	R	<=4	S	<=1	S	<4/76	R	Negative
S	>64	R	>64	R	>8	R	<=1	S	>4/76	R	Negative
I	>64	R	>64	R	>8	R	<=1	S	>4/76	R	Negative
S	>64	R	>64	R*	8	I	<=1	S	<=2/38	S	Negative
S	>64	R	>64	R*	8	I	<=1	S	>4/76	R	Negative
R	64	I	>64	R	>8	R	>2	R	>4/76	R	Negative
R	>64	R	>64	R	>8	R	2	I	>4/76	R	Positive
I	>64	R	>64	R	>8	R	<=1	S	>4/76	R	Positive
S	>64	R	>64	R	<=4	S	<=1	S	>4/76	R	Negative
R	64	I	64	I	>8	R	>2	R	>4/76	R	Negative
I	>64	R	>64	R	8	I	<=1	S	>4/76	R	Negative
R	>64	R	>64	R	>8	R	<=1	S	>4/76	R	Negative
S	>64	R	>64	R	<=4	S	<=1	S	>4/76	R	Positive
S	>64	R	>64	R	<=4	S	<=1	S	>4/76	R	Negative
R	>64	R	>64	R	>8	R	<=1	S	>4/76	R	Negative
S	64	I	64	I	<=4	S	<=1	S	<=2/38	S	Negative

R	>64	R	>64	R	>8	R	<=1	S	>4/76	R	Positive
R	64	I	64	I	>8	R	>2	R	>4/76	R	Negative
S	>64	R	>64	R	<=4	S	<=1	S	<=2/38	S	Negative
I	>64	R	>64	R	>8	R	2	I	<=2/38	S	Negative
I	>64	R	>64	R	>8	R	2	I	>4/76	R	Positive
S	<=8	S	<=8	S	<=4	S	<=1	S	<=2/38	S	Negative
S	>64	R	>64	R	<=4	S	<=1	S	>4/76	R	Positive

S	>64	R	>64	R	>8	R	<=1	S	>4/76	R	Negative
S	>64	R	>64	R	<=4	S	<=1	S	>4/76	R	Positive
R	64	I	>64	R	>8	R	>2	R	>4/76	R	Negative
R	>64	R	>64	R	>8	R	<=1	S	>4/76	R	Negative
I	>64	R	>64	R	8	I	>2	R	>4/76	R	Negative
R	>64	R	>64	R*	>8	R	<=1	S	>4/76	R	Negative
R	>64	R	>64	R	>8	R	<=1	S	>4/76	R	Negative
R	>64	R	>64	R	>8	R	<=1	S	>4/76	R	Negative

S	>64	R	>64	R	>8	R	<=1	S	<=2/38	S	Positive
I	>64	R	>64	R*	>8	R	<=1	S	>4/76	R	Negative
S	>64	R	>64	R*	<=4	S	<=1	S	>4/76	R	Negative
R	>64	R	>64	R*	>8	R	<=1	S	>4/76	R	Negative
S	>64	R	>64	R	>8	R	<=1	S	>4/76	R	Negative
R	64	I	>64	R*	>8	R	<=1	S	>4/76	R	Negative
I	>64	R	>64	R	<=4	S	<=1	S	>4/76	R	Negative
I	>64	R	>64	R	8	I	>2	R	>4/76	R	Negative
S	>64	R	>64	R	<=4	S	<=1	S	>4/76	R	Negative
R	64	I	>64	R	>8	R	>2	R	>4/76	R	Negative
R	>64	R	>64	R	>8	R	<=1	S	>4/76	R	Negative
S	>64	R	>64	R	<=4	S	<=1	S	>4/76	R	Positive
S	>64	R	>64	R	8	I	<=1	S	>4/76	R	Negative
R	>64	R	>64	R	>8	R	<=1	S	>4/76	R	Positive
R	>64	R	>64	R	>8	R	>2	R	>4/76	R	Negative
I	>64	R	>64	R	>8	R	<=1	S	>4/76	R	Negative
I	>64	R	>64	R*	<=4	S	2	I	>4/76	R	Negative
S	>64	R	>64	R	<=4	S	<=1	S	>4/76	R	Negative
S	>64	R	>64	R*	<=4	S	<=1	S	>4/76	R	Negative
R	>64	R	>64	R	>8	R	<=1	S	>4/76	R	Negative
R	>64	R	>64	R*	>8	R	>2	R	>4/76	R	Negative
R	64	I	64	I	>8	R	>2	R	>4/76	R	Positive
S	>64	R	>64	R*	<=4	S	<=1	S	>4/76	R	Negative
S	>64	R	>64	R	<=4	S	<=1	S	>4/76	R	Positive
R	>64	R	>64	R	>8	R	<=1	S	>4/76	R	Positive
R	>64	R	>64	R	>8	R	>2	R	>4/76	R	Negative
S	>64	R	>64	R	<=4	S	<=1	S	>4/76	R	Negative
I	16	S	64	I	8	I	>2	R	>4/76	R	Negative
R	>64	R	>64	R	>8	R	>2	R	>4/76	R	Positive
S	>64	R	>64	R*	>8	R	2	I	<=2/38	S	Negative
R	>64	R	>64	R	>8	R	<=1	S	>4/76	R	Positive
S	>64	R	>64	R*	<=4	S	<=1	S	>4/76	R	Negative
S	>64	R	>64	R*	<=4	S	<=1	S	>4/76	R	Positive
R	>64	R	>64	R	>8	R	<=1	S	>4/76	R	Negative
S	64	I	>64	R*	<=4	S	<=1	S	>4/76	R	Negative
R	>64	R	>64	R	>8	R	<=1	S	>4/76	R	Positive
S	>64	R	>64	R	<=4	S	<=1	S	>4/76	R	Negative
S	64	I	>64	R*	8	I	<=1	S	>4/76	R	Negative

I	>64	R	>64	R	<=4	S	<=1	S	>4/76	R	Positive
S	>64	R	>64	R	8	I	<=1	S	>4/76	R	Positive
S	>64	R	>64	R	<=4	S	<=1	S	>4/76	R	Positive
S	>64	R	>64	R	<=4	S	<=1	S	>4/76	R	Positive
S	>64	R	>64	R	8	I	<=1	S	>4/76	R	Positive
S	>64	R	>64	R	<=4	S	<=1	S	>4/76	R	Negative

R	>64	R	<=8	S	>8	R	>2	R	>4/76	R	Negative

S	<=8	S	>64	R*	<=4	S	<=1	S	>4/76	R	Negative		
S	>64	R	>64	R*	>8	R	<=1	S	>4/76	R	Negative		
S	>64	R	>64	R*	<=4	S	<=1	S	>4/76	R	Negative		
R	<=8	S	>64	R*	>8	R	<=1	S	>4/76	R	Negative		
S	>64	R	>64	R*	<=4	S	<=1	S	>4/76	R	Negative		
I	>64	R	>64	R	8	I	<=1	S	>4/76	R	Negative		
S	>64	R	>64	R	<=4	S	<=1	S	>4/76	R	Negative		
S	>64	R	>64	R*	>8	R	<=1	S	>4/76	R	Negative		
S	>64	R	>64	R*	<=4	S	<=1	S	>4/76	R	Negative		
S	>64	R	>64	R	>8	R	2	I	>4/76	R	Positive		
R	64	I	>64	R*	>8	R	<=1	S	>4/76	R	Negative		
I	>64	R	>64	R	>8	R	2	I	>4/76	R	Negative		
I	>64	R	>64	R	>8	R	<=1	S	>4/76	R	Negative		
R	>64	R	>64	R	>8	R	2	I	>4/76	R	Negative		

S	64	I	>64	R*	<=4	S	<=1	S	>4/76	R	Negative
S	>64	R	>64	R	>8	R	<=1	S	>4/76	R	Positive
S	>64	R	>64	R	<=4	S	<=1	S	>4/76	R	Positive
R	>64	R	>64	R	>8	R	2	I	>4/76	R	Positive
R	>64	R	>64	R	>8	R	<=1	S	>4/76	R	Positive
R	>64	R	>64	R	>8	R	<=1	S	>4/76	R	Positive
S	<=8	S	<=8	S	<=4	S	<=1	S	<=2/38	S	Negative
S	>64	R	>64	R	<=4	S	<=1	S	>4/76	R	Positive
S	>64	R	>64	R	<=4	S	<=1	S	>4/76	R	Positive
R	>64	R	>64	R	>8	R	2	I	>4/76	R	Positive
I	64	I	>64	R	>8	R	<=1	S	>4/76	R	Negative
S	>64	R	>64	R*	8	I	<=1	S	>4/76	R	Negative
R	>64	R	>64	R	>8	R	<=1	S	>4/76	R	Negative
S	>64	R	>64	R	>8	R	<=1	S	>4/76	R	Negative
S	>64	R	>64	R	>8	R	<=1	S	>4/76	R	Negative
I	>64	R	>64	R	>8	R	<=1	S	>4/76	R	Positive
S	>64	R	>64	R*	>8	R	<=1	S	>4/76	R	Negative
S	>64	R	>64	R	8	I	<=1	S	>4/76	R	Negative
S	>64	R	>64	R*	<=4	S	<=1	S	>4/76	R	Negative
R	>64	R	>64	R*	>8	R	>2	R	>4/76	R	Negative
S	>64	R	>64	R	>8	R	<=1	S	<=2/38	S	Negative

S	>64	R	>64	R	>8	R	<=1	S	>4/76	R	Positive
R	>64	R	>64	R	>8	R	2	I	>4/76	R	Negative
S	>64	R	>64	R*	8	I	<=1	S	>4/76	R	Negative
S	>64	R	>64	R	>8	R	2	I	>4/76	R	Positive
S	>64	R	>64	R	>8	R	<=1	S	>4/76	R	Positive
I	>64	R	>64	R	>8	R	2	I	>4/76	R	Positive
S	>64	R	>64	R	8	I	<=1	S	>4/76	R	Negative
S	>64	R	>64	R	>8	R	<=1	S	>4/76	R	Negative
S	>64	R	>64	R	<=4	S	<=1	S	>4/76	R	Negative
S	>64	R	>64	R	>8	R	<=1	S	>4/76	R	Negative
R	>64	R	>64	R	>8	R	2	I	>4/76	R	Negative
R	>64	R	>64	R*	8	I	<=1	S	>4/76	R	Negative
R	64	I	64	I	>8	R	>2	R	>4/76	R	Positive
R	>64	R	>64	R*	>8	R	>2	R	>4/76	R	Negative
S	>64	R	>64	R	>8	R	<=1	S	>4/76	R	Negative
S	>64	R	>64	R	<=4	S	<=1	S	>4/76	R	Positive
S	64	I	>64	R	<=4	S	<=1	S	>4/76	R	Negative
S	>64	R	>64	R	8	I	<=1	S	>4/76	R	Negative
S	>64	R	>64	R	>8	R	<=1	S	>4/76	R	Positive
S	>64	R	>64	R	>8	R	<=1	S	>4/76	R	Positive
R	>64	R	>64	R	>8	R	<=1	S	>4/76	R	Negative
I	>64	R	>64	R	>8	R	2	I	>4/76	R	Negative
S	>64	R	>64	R	<=4	S	<=1	S	>4/76	R	Positive
R	>64	R	>64	R	>8	R	<=1	S	>4/76	R	Negative
S	>64	R	>64	R*	>8	R	<=1	S	>4/76	R	Negative
R	>64	R	>64	R	>8	R	2	I	>4/76	R	Positive
S	>64	R	>64	R*	8	I	<=1	S	>4/76	R	Negative
S	>64	R	>64	R	>8	R	2	I	>4/76	R	Negative
S	>64	R	>64	R	>8	R	<=1	S	>4/76	R	Positive
S	>64	R	>64	R	>8	R	<=1	S	>4/76	R	Negative
S	>64	R	>64	R*	<=4	S	<=1	S	>4/76	R	Negative
S	>64	R	>64	R*	<=4	S	<=1	S	>4/76	R	Negative
S	>64	R	>64	R	<=4	S	<=1	S	<=2/38	S	Positive
R	>64	R	>64	R	>8	R	2	I	>4/76	R	Positive
S	>64	R	>64	R*	<=4	S	<=1	S	>4/76	R	Negative
R	>64	R	>64	R	>8	R	2	I	>4/76	R	Positive
S	>64	R	>64	R*	<=4	S	<=1	S	>4/76	R	Negative
S	>64	R	>64	R*	<=4	S	<=1	S	>4/76	R	Negative
R	>64	R	>64	R	>8	R	<=1	S	>4/76	R	Positive
I	>64	R	>64	R	>8	R	2	I	>4/76	R	Negative
S	>64	R	>64	R	<=4	S	<=1	S	>4/76	R	Negative
S	>64	R	>64	R	<=4	S	<=1	S	>4/76	R	Positive
R	>64	R	>64	R*	>8	R	<=1	S	>4/76	R	Negative
S	>64	R	>64	R*	<=4	S	<=1	S	>4/76	R	Positive
S	>64	R	>64	R	<=4	S	<=1	S	<=2/38	S	Negative
S	>64	R	>64	R*	<=4	S	<=1	S	>4/76	R	Negative
S	64	I	>64	R*	<=4	S	<=1	S	>4/76	R	Negative
S	>64	R	>64	R	<=4	S	<=1	S	<=2/38	S	Positive
I	>64	R	>64	R*	>8	R	<=1	S	>4/76	R	Negative
I	>64	R	>64	R	>8	R	2	I	>4/76	R	Negative
S	>64	R	>64	R*	8	I	<=1	S	>4/76	R	Negative
S	>64	R	>64	R	<=4	S	<=1	S	>4/76	R	Negative
S	>64	R	>64	R*	<=4	S	<=1	S	>4/76	R	Negative
S	>64	R	>64	R	<=4	S	<=1	S	>4/76	R	Positive
S	>64	R	>64	R	<=4	S	<=1	S	>4/76	R	Negative

R	>64	R	>64	R*	>8	R	<=1	S	>4/76	R	Negative
S	>64	R	>64	R*	<=4	S	<=1	S	>4/76	R	Negative

S	64	I	>64	R*	<=4	S	<=1	S	>4/76	R	Negative
R	>64	R	>64	R*	>8	R	2	I	>4/76	R	Negative

R	>64	R	>64	R*	>8	R	>2	R	>4/76	R	Negative
S	>64	R	>64	R	<=4	S	<=1	S	>4/76	R	Negative
R	>64	R	>64	R	>8	R	2	I	>4/76	R	Positive
S	>64	R	>64	R	<=4	S	<=1	S	>4/76	R	Positive

S	>64	R	>64	R	<=4	S	<=1	S	>4/76	R	Negative
S	>64	R	>64	R	<=4	S	<=1	S	>4/76	R	Positive
S	>64	R	>64	R	<=4	S	<=1	S	>4/76	R	Negative
S	>64	R	>64	R	<=4	S	<=1	S	>4/76	R	Negative
R	>64	R	>64	R*	>8	R	>2	R	>4/76	R	Negative
S	>64	R	>64	R	<=4	S	<=1	S	>4/76	R	Positive
S	>64	R	>64	R	<=4	S	<=1	S	>4/76	R	Positive
S	>64	R	>64	R	<=4	S	<=1	S	>4/76	R	Negative
S	64	I	>64	R*	<=4	S	<=1	S	>4/76	R	Negative
S	>64	R	>64	R*	<=4	S	<=1	S	>4/76	R	Negative
S	>64	R	>64	R*	<=4	S	<=1	S	>4/76	R	Negative
S	>64	R	>64	R	<=4	S	<=1	S	>4/76	R	Positive
S	>64	R	>64	R*	<=4	S	<=1	S	>4/76	R	Negative
S	>64	R	>64	R*	<=4	S	2	I	>4/76	R	Negative
S	>64	R	>64	R*	<=4	S	<=1	S	>4/76	R	Negative
I	>64	R	>64	R*	>8	R	2	I	>4/76	R	Negative
S	>64	R	>64	R	8	I	<=1	S	>4/76	R	Negative
S	64	I	>64	R*	<=4	S	<=1	S	>4/76	R	Negative
S	>64	R	>64	R	8	I	2	I	>4/76	R	Negative
S	64	I	64	I	<=4	S	<=1	S	>4/76	R	Negative
S	>64	R	>64	R	<=4	S	<=1	S	>4/76	R	Positive
S	<=8	S	<=8	S	<=4	S	<=1	S	>4/76	R	Negative
S	<=8	S	>64	R*	<=4	S	<=1	S	>4/76	R	Negative
S	16	S	16	R*	<=4	S	<=1	S	>4/76	R	Negative
S	>64	R	>64	R*	<=4	S	<=1	S	>4/76	R	Negative
I	64	I	>64	R	<=4	S	<=1	S	>4/76	R	Negative
I	>64	R	>64	R	>8	R	2	I	>4/76	R	Negative
S	>64	R	>64	R	<=4	S	<=1	S	>4/76	R	Negative
I	>64	R	>64	R*	8	I	>2	R	>4/76	R	Negative
S	>64	R	>64	R	<=4	S	<=1	S	>4/76	R	Negative
R	>64	R	>64	R*	>8	R	<=1	S	>4/76	R	Negative
I	>64	R	>64	R	>8	R	<=1	S	>4/76	R	Positive
S	>64	R	>64	R	>8	R	<=1	S	>4/76	R	Positive
S	>64	R	>64	R*	<=4	S	<=1	S	>4/76	R	Negative
R	>64	R	>64	R	>8	R	<=1	S	>4/76	R	Negative
R	>64	R	>64	R	>8	R	<=1	S	>4/76	R	Negative
R	>64	R	>64	R	>8	R	<=1	S	>4/76	R	Positive
I	64	I	>64	R	>8	R	2	I	>4/76	R	Negative
R	>64	R	>64	R	>8	R	2	I	>4/76	R	Positive
S	>64	R	>64	R*	<=4	S	<=1	S	>4/76	R	Negative
S	64	I	>64	R*	<=4	S	<=1	S	>4/76	R	Negative
S	>64	R	>64	R	<=4	S	<=1	S	>4/76	R	Negative
S	64	I	>64	R*	8	I	<=1	S	>4/76	R	Negative
S	>64	R	>64	R	<=4	S	<=1	S	<=2/38	S	Negative
S	>64	R	>64	R	<=4	S	<=1	S	<=2/38	S	Negative
S	>64	R	>64	R	<=4	S	<=1	S	>4/76	R	Negative
S	64	I	>64	R	<=4	S	<=1	S	<=2/38	S	Negative
I	>64	R	>64	R	>8	R	2	I	>4/76	R	Negative
I	16	S	>64	R*	>8	R	2	I	>4/76	R	Negative
S	>64	R	>64	R	>8	R	<=1	S	>4/76	R	Positive
S	>64	R	>64	R	<=4	S	<=1	S	>4/76	R	Positive

R	>64	R	>64	R	>8	R	<=1	S	>4/76	R	Negative
S	>64	R	>64	R	<=4	S	<=1	S	>4/76	R	Positive
S	>64	R	>64	R*	<=4	S	<=1	S	>4/76	R	Negative
S	>64	R	>64	R*	<=4	S	<=1	S	>4/76	R	Negative
S	64	I	>64	R*	<=4	S	<=1	S	>4/76	R	Negative

I	>64	R	>64	R*	8	I	2	I	>4/76	R	Negative
S	>64	R	>64	R	>8	R	2	I	>4/76	R	Negative
R	>64	R	>64	R	>8	R	<=1	S	>4/76	R	Negative
I	64	I	>64	R*	<=4	S	<=1	S	>4/76	R	Negative
S	<=8	S	>64	R	>8	R	<=1	S	>4/76	R	Negative
S	<=8	S	>64	R	<=4	S	<=1	S	<=2/38	S	Negative
I	>64	R	>64	R	>8	R	<=1	S	>4/76	R	Negative
S	>64	R	>64	R*	8	I	<=1	S	<=2/38	S	Negative
S	64	I	>64	R*	8	I	<=1	S	>4/76	R	Negative
S	>64	R	>64	R*	>8	R	<=1	S	>4/76	R	Negative
R	>64	R	>64	R	>8	R	2	I	>4/76	R	Positive
R	>64	R	>64	R	8	I	<=1	S	>4/76	R	Negative
S	>64	R	>64	R	<=4	S	<=1	S	>4/76	R	Positive
S	>64	R	>64	R*	>8	R	<=1	S	>4/76	R	Negative
I	>64	R	16	R*	<=4	S	<=1	S	<=2/38	S	Negative
R	>64	R	>64	R*	>8	R	2	I	>4/76	R	Negative
R	>64	R	>64	R	>8	R	<=1	S	>4/76	R	Positive
S	>64	R	>64	R*	<=4	S	<=1	S	>4/76	R	Negative

I	>64	R	>64	R	>8	R	<=1	S	>4/76	R	Negative
S	>64	R	>64	R	>8	R	<=1	S	>4/76	R	Positive
S	>64	R	>64	R*	>8	R	<=1	S	>4/76	R	Negative
R	>64	R	>64	R	>8	R	2	I	>4/76	R	Negative
S	>64	R	>64	R	<=4	S	<=1	S	>4/76	R	Positive
S	>64	R	>64	R	>8	R	<=1	S	>4/76	R	Positive
S	>64	R	>64	R*	<=4	S	<=1	S	>4/76	R	Negative
R	>64	R	>64	R	<=4	S	<=1	S	>4/76	R	Positive
S	>64	R	>64	R	<=4	S	<=1	S	<=2/38	S	Negative
S	>64	R	>64	R	<=4	S	<=1	S	>4/76	R	Negative
I	>64	R	>64	R*	8	I	2	I	>4/76	R	Negative
S	<=8	S	16	S	<=4	S	<=1	S	<=2/38	S	Negative
S	>64	R	>64	R	<=4	S	<=1	S	<=2/38	S	Positive
S	>64	R	>64	R*	<=4	S	<=1	S	>4/76	R	Negative
R	>64	R	>64	R	>8	R	2	I	>4/76	R	Positive
R	>64	R	>64	R	>8	R	<=1	S	>4/76	R	Positive
R	>64	R	>64	R	>8	R	<=1	S	>4/76	R	Negative
S	>64	R	>64	R	<=4	S	<=1	S	>4/76	R	Positive
S	>64	R	>64	R	<=4	S	<=1	S	>4/76	R	Positive
R	>64	R	>64	R	>8	R	<=1	S	>4/76	R	Negative
S	>64	R	>64	R*	<=4	S	<=1	S	>4/76	R	Negative
S	>64	R	>64	R*	<=4	S	<=1	S	>4/76	R	Negative
S	>64	R	>64	R*	<=4	S	<=1	S	>4/76	R	Negative
R	>64	R	>64	R	>8	R	2	I	>4/76	R	Negative
S	>64	R	>64	R*	>8	R	<=1	S	<=2/38	S	Negative
S	64	I	>64	R	<=4	S	<=1	S	<=2/38	S	Negative
R	>64	R	>64	R	>8	R	<=1	S	>4/76	R	Negative
I	>64	R	>64	R	>8	R	<=1	S	>4/76	R	Negative
S	>64	R	>64	R*	<=4	S	<=1	S	>4/76	R	Negative
S	64	I	>64	R*	>8	R	<=1	S	>4/76	R	Negative
I	>64	R	>64	R	8	I	<=1	S	>4/76	R	Positive
S	>64	R	>64	R*	8	I	2	I	>4/76	R	Negative
I	>64	R	>64	R	>8	R	<=1	S	>4/76	R	Negative
S	>64	R	>64	R*	8	I	<=1	S	>4/76	R	Negative
S	>64	R	>64	R*	<=4	S	<=1	S	>4/76	R	Negative
I	>64	R	>64	R	>8	R	2	I	>4/76	R	Negative
S	>64	R	>64	R*	<=4	S	<=1	S	>4/76	R	Negative
S	>64	R	>64	R*	<=4	S	<=1	S	>4/76	R	Negative
R	>64	R	>64	R	>8	R	<=1	S	>4/76	R	Positive
R	>64	R	>64	R	>8	R	2	I	>4/76	R	Positive
S	>64	R	>64	R	<=4	S	<=1	S	<=2/38	S	Negative
S	>64	R	>64	R*	<=4	S	<=1	S	>4/76	R	Negative
S	>64	R	>64	R*	<=4	S	<=1	S	>4/76	R	Negative
R	>64	R	>64	R	>8	R	2	I	>4/76	R	Negative
S	>64	R	>64	R	>8	R	<=1	S	>4/76	R	Positive
S	>64	R	>64	R	<=4	S	<=1	S	>4/76	R	Negative
S	>64	R	>64	R	<=4	S	<=1	S	<=2/38	S	Negative
S	>64	R	>64	R	<=4	S	<=1	S	>4/76	R	Negative
S	>64	R	>64	R	<=4	S	<=1	S	>4/76	R	Negative
S	>64	R	>64	R	<=4	S	<=1	S	<=2/38	S	Negative
S	>64	R	>64	R	8	I	<=1	S	>4/76	R	Negative
S	>64	R	>64	R*	<=4	S	<=1	S	>4/76	R	Negative
S	>64	R	>64	R*	<=4	S	<=1	S	>4/76	R	Negative
S	>64	R	>64	R	<=4	S	<=1	S	>4/76	R	Negative
S	>64	R	>64	R	>8	R	<=1	S	>4/76	R	Negative

S	16	S	64	I	<=4	S	<=1	S	<=2/38	S	Positive
S	>64	R	>64	R	8	I	<=1	S	>4/76	R	Negative
S	>64	R	>64	R	<=4	S	<=1	S	>4/76	R	Positive
S	>64	R	>64	R	>8	R	<=1	S	>4/76	R	Positive

S	>64	R	>64	R*	<=4	S	<=1	S	>4/76	R	Negative
S	>64	R	>64	R	<=4	S	<=1	S	>4/76	R	Negative
R	>64	R	>64	R*	>8	R	<=1	S	>4/76	R	Negative
I	>64	R	>64	R*	>8	R	2	I	>4/76	R	Negative
R	>64	R	>64	R	>8	R	<=1	S	>4/76	R	Positive
S	<=8	S	>64	R*	<=4	S	<=1	S	>4/76	R	Negative
I	>64	R	>64	R	8	I	2	I	>4/76	R	Negative

S	>64	R	>64	R*	<=4	S	<=1	S	>4/76	R	Negative
I	>64	R	>64	R	>8	R	<=1	S	>4/76	R	Negative
R	>64	R	>64	R	>8	R	<=1	S	>4/76	R	Positive
I	>64	R	>64	R*	>8	R	<=1	S	>4/76	R	Negative
S	64	I	>64	R	<=4	S	<=1	S	>4/76	R	Negative
S	>64	R	>64	R*	8	I	<=1	S	>4/76	R	Negative
S	>64	R	>64	R*	<=4	S	<=1	S	>4/76	R	Negative
I	>64	R	>64	R*	>8	R	<=1	S	>4/76	R	Negative
S	64	I	64	I	<=4	S	<=1	S	>4/76	R	Negative
I	>64	R	>64	R*	>8	R	<=1	S	>4/76	R	Negative
S	>64	R	>64	R	<=4	S	<=1	S	>4/76	R	Negative
R	64	I	>64	R	>8	R	>2	R	>4/76	R	Negative
S	>64	R	>64	R*	<=4	S	<=1	S	>4/76	R	Negative
I	>64	R	>64	R	>8	R	<=1	S	>4/76	R	Positive
R	>64	R	>64	R	>8	R	<=1	S	>4/76	R	Positive
S	>64	R	>64	R*	<=4	S	<=1	S	>4/76	R	Negative
S	64	I	>64	R	>8	R	<=1	S	>4/76	R	Negative
S	64	I	>64	R*	<=4	S	<=1	S	>4/76	R	Negative
R	16	S	16	S	>8	R	>2	R	<=2/38	S	Positive
S	>64	R	>64	R	<=4	S	2	I	>4/76	R	Positive
I	>64	R	>64	R	>8	R	2	I	>4/76	R	Negative
S	64	I	>64	R*	<=4	S	<=1	S	>4/76	R	Negative
I	>64	R	>64	R*	>8	R	<=1	S	>4/76	R	Negative
S	>64	R	>64	R*	<=4	S	<=1	S	>4/76	R	Negative
S	>64	R	>64	R*	<=4	S	<=1	S	>4/76	R	Negative
S	>64	R	>64	R*	<=4	S	<=1	S	<=2/38	S	Negative
S	>64	R	>64	R*	<=4	S	<=1	S	<=2/38	S	Negative
R	>64	R	>64	R	>8	R	2	I	>4/76	R	Negative
S	>64	R	>64	R	<=4	S	<=1	S	>4/76	R	Negative
S	64	I	>64	R	<=4	S	<=1	S	<=2/38	S	Negative
I	>64	R	>64	R	>8	R	2	I	>4/76	R	Negative
I	>64	R	>64	R	>8	R	<=1	S	>4/76	R	Negative
S	>64	R	>64	R*	8	I	<=1	S	>4/76	R	Negative
S	>64	R	>64	R	<=4	S	<=1	S	>4/76	R	Negative
S	>64	R	>64	R*	<=4	S	<=1	S	>4/76	R	Negative
S	>64	R	>64	R	<=4	S	<=1	S	>4/76	R	Positive
S	>64	R	>64	R*	<=4	S	<=1	S	>4/76	R	Negative
S	>64	R	>64	R*	<=4	S	<=1	S	>4/76	R	Negative
R	16	S	64	I	>8	R	2	I	<=2/38	S	Positive
I	>64	R	>64	R	>8	R	<=1	S	>4/76	R	Negative
S	>64	R	>64	R	>8	R	<=1	S	>4/76	R	Positive
R	>64	R	>64	R	>8	R	2	I	>4/76	R	Positive
S	>64	R	>64	R*	<=4	S	<=1	S	>4/76	R	Negative
I	16	S	>64	R*	>8	R	<=1	S	Apr-76	R	Negative
I	64	I	64	I	>8	R	<=1	S	<=2/38	S	Negative
S	<=8	S	>64	R*	<=4	S	<=1	S	>4/76	R	Negative
S	>64	R	>64	R	<=4	S	<=1	S	>4/76	R	Negative
S	>64	R	>64	R	<=4	S	<=1	S	>4/76	R	Negative
R	>64	R	>64	R	>8	R	>2	R	>4/76	R	Negative
S	>64	R	>64	R*	8	I	<=1	S	>4/76	R	Negative
I	>64	R	>64	R*	8	I	2	I	>4/76	R	Negative
S	>64	R	>64	R	<=4	S	<=1	S	>4/76	R	Positive
S	>64	R	>64	R*	>8	R	<=1	S	>4/76	R	Negative
S	64	I	>64	R	>8	R	<=1	S	<=2/38	S	Negative
S	>64	R	>64	R	>8	R	<=1	S	>4/76	R	Positive
S	>64	R	>64	R	>8	R	<=1	S	>4/76	R	Negative

S	<=8	S	<=8	S	8	I	<=1	S	<=2/38	S	Negative
I	>64	R	>64	R	>8	R	<=1	S	>4/76	R	Negative
R	>64	R	>64	R	>8	R	2	I	>4/76	R	Negative
I	>64	R	>64	R	>8	R	<=1	S	>4/76	R	Negative
S	>64	R	>64	R	<=4	S	<=1	S	>4/76	R	Positive
S	<=8	S	>64	R*	>8	R	<=1	S	>4/76	R	Negative
S	>64	R	>64	R	<=4	S	<=1	S	>4/76	R	Positive
R	>64	R	>64	R*	>8	R	<=1	S	>4/76	R	Negative
S	>64	R	>64	R	<=4	S	<=1	S	>4/76	R	Positive
S	>64	R	>64	R	8	I	<=1	S	>4/76	R	Negative
S	>64	R	>64	R*	<=4	S	<=1	S	>4/76	R	Negative
S	64	I	>64	R*	<=4	S	<=1	S	>4/76	R	Negative
S	>64	R	>64	R	<=4	S	<=1	S	>4/76	R	Negative
R	>64	R	>64	R*	>8	R	<=1	S	>4/76	R	Negative
S	64	I	>64	R*	<=4	S	<=1	S	>4/76	R	Negative
S	>64	R	>64	R	<=4	S	<=1	S	>4/76	R	Negative
I	>64	R	>64	R	8	I	<=1	S	>4/76	R	Negative
S	>64	R	>64	R	<=4	S	<=1	S	>4/76	R	Negative
S	>64	R	>64	R	8	I	<=1	S	>4/76	R	Negative
S	>64	R	>64	R	8	I	<=1	S	>4/76	R	Negative
I	>64	R	>64	R*	>8	R	<=1	S	>4/76	R	Positive
S	>64	R	>64	R	<=4	S	<=1	S	>4/76	R	Negative
S	>64	R	>64	R	8	I	<=1	S	>4/76	R	Positive
I	>64	R	>64	R	>8	R	<=1	S	>4/76	R	Positive
I	>64	R	>64	R	>8	R	2	I	>4/76	R	Positive
S	>64	R	>64	R	<=4	S	<=1	S	>4/76	R	Positive
S	>64	R	>64	R	<=4	S	<=1	S	>4/76	R	Positive
S	>64	R	>64	R*	<=4	S	<=1	S	>4/76	R	Negative
S	>64	R	>64	R*	<=4	S	<=1	S	>4/76	R	Negative
R	>64	R	>64	R*	>8	R	>2	R	>4/76	R	Negative
I	>64	R	>64	R	>8	R	<=1	S	>4/76	R	Negative
S	>64	R	>64	R*	>8	R	<=1	S	>4/76	R	Negative
I	>64	R	>64	R	>8	R	<=1	S	>4/76	R	Negative
I	>64	R	>64	R	>8	R	<=1	S	>4/76	R	Negative
I	>64	R	>64	R	>8	R	<=1	S	>4/76	R	Positive
S	>64	R	>64	R*	<=4	S	<=1	S	>4/76	R	Negative
S	>64	R	>64	R	8	I	<=1	S	>4/76	R	Negative
I	>64	R	>64	R	>8	R	<=1	S	>4/76	R	Negative
I	>64	R	>64	R	>8	R	2	I	>4/76	R	Positive
S	>64	R	>64	R*	<=4	S	<=1	S	>4/76	R	Negative
I	>64	R	>64	R	>8	R	<=1	S	>4/76	R	Negative
S	>64	R	>64	R*	<=4	S	<=1	S	>4/76	R	Negative
S	>64	R	>64	R	<=4	S	<=1	S	>4/76	R	Negative
I	>64	R	>64	R	>8	R	<=1	S	>4/76	R	Negative
I	>64	R	>64	R	>8	R	<=1	S	>4/76	R	Negative
I	>64	R	>64	R	>8	R	<=1	S	>4/76	R	Negative
S	>64	R	>64	R	<=4	S	<=1	S	Apr-76	R	Positive
R	>64	R	>64	R	>8	R	2	I	>4/76	R	Positive
S	>64	R	>64	R	<=4	S	<=1	S	>4/76	R	Negative
I	>64	R	>64	R*	>8	R	<=1	S	>4/76	R	Negative
S	>64	R	>64	R	>8	R	<=1	S	>4/76	R	Negative
R	>64	R	>64	R	>8	R	<=1	S	>4/76	R	Negative
S	>64	R	>64	R*	>8	R	<=1	S	>4/76	R	Negative
R	>64	R	>64	R	>8	R	<=1	S	>4/76	R	Negative
R	>64	R	>64	R	>8	R	2	I	>4/76	R	Negative

R	>64	R	>64	R	>8	R	2	I	>4/76	R	Negative
I	>64	R	>64	R	>8	R	2	I	>4/76	R	Negative
S	>64	R	>64	R*	8	I	<=1	S	>4/76	R	Negative
I	>64	R	>64	R	>8	R	2	I	>4/76	R	Positive
S	>64	R	>64	R	8	I	<=1	S	>4/76	R	Negative
S	<=8	S	<=8	S	<=4	S	<=1	S	<=2/38	S	Negative
S	>64	R	>64	R*	<=4	S	<=1	S	>4/76	R	Negative
S	>64	R	>64	R	>8	R	<=1	S	>4/76	R	Negative
S	>64	R	>64	R	<=4	S	<=1	S	<=2/38	S	Negative
I	>64	R	>64	R	8	I	2	I	>4/76	R	Negative
S	64	I	>64	R*	8	I	2	I	<=2/38	S	Negative
R	>64	R	>64	R	>8	R	<=1	S	>4/76	R	Positive
S	64	I	>64	R*	<=4	S	<=1	S	>4/76	R	Negative
S	>64	R	>64	R	>8	R	<=1	S	>4/76	R	Negative
S	64	I	>64	R*	<=4	S	<=1	S	>4/76	R	Negative
S	>64	R	>64	R*	8	I	2	I	>4/76	R	Negative
R	>64	R	>64	R	>8	R	2	I	>4/76	R	Negative
R	>64	R	>64	R	>8	R	2	I	>4/76	R	Negative
S	>64	R	>64	R*	<=4	S	<=1	S	>4/76	R	Negative
S	>64	R	>64	R*	<=4	S	<=1	S	>4/76	R	Negative
S	<=8	S	<=8	S	8	I	2	I	<=2/38	S	Positive
I	16	S	64	I	>8	R	2	I	<=2/38	S	Positive
R	>64	R	>64	R	>8	R	<=1	S	>4/76	R	Negative
S	>64	R	>64	R	8	I	<=1	S	>4/76	R	Negative
S	<=8	S	16	S	<=4	S	<=1	S	<=2/38	S	Negative
R	<=8	S	<=8	S	>8	R	>2	R	<=2/38	S	Negative
S	<=8	S	>64	R*	>8	R	<=1	S	>4/76	R	Negative
S	>64	R	>64	R*	>8	R	<=1	S	>4/76	R	Negative
S	>64	R	>64	R*	>8	R	<=1	S	>4/76	R	Negative
S	>64	R	>64	R*	8	I	<=1	S	>4/76	R	Negative
S	>64	R	>64	R*	<=4	S	<=1	S	>4/76	R	Negative
R	>64	R	>64	R*	>8	R	<=1	S	>4/76	R	Negative
S	>64	R	>64	R*	<=4	S	<=1	S	>4/76	R	Negative
S	>64	R	>64	R	>8	R	<=1	S	<=2/38	S	Negative
R	>64	R	>64	R	>8	R	<=1	S	>4/76	R	Negative
S	>64	R	>64	R	<=4	S	<=1	S	<=2/38	S	Positive
R	>64	R	>64	R*	>8	R	<=1	S	>4/76	R	Negative
S	>64	R	>64	R*	<=4	S	<=1	S	>4/76	R	Negative
S	>64	R	>64	R*	<=4	S	<=1	S	>4/76	R	Negative
S	>64	R	>64	R*	<=4	S	<=1	S	>4/76	R	Negative
S	>64	R	>64	R*	<=4	S	<=1	S	>4/76	R	Negative
I	>64	R	>64	R	>8	R	<=1	S	>4/76	R	Negative
S	64	I	64	I	8	I	<=1	S	<=2/38	S	Positive
S	>64	R	>64	R	<=4	S	<=1	S	<=2/38	S	Negative
R	>64	R	>64	R	>8	R	2	I	>4/76	R	Positive
S	64	I	>64	R*	<=4	S	<=1	S	>4/76	R	Negative
S	>64	R	>64	R*	<=4	S	<=1	S	<=2/38	S	Negative
S	>64	R	>64	R*	<=4	S	<=1	S	>4/76	R	Negative
S	>64	R	>64	R	<=4	S	<=1	S	>4/76	R	Negative
S	>64	R	>64	R	<=4	S	<=1	S	>4/76	R	Negative
S	64	I	>64	R*	<=4	S	<=1	S	>4/76	R	Negative
S	>64	R	>64	R	<=4	S	<=1	S	>4/76	R	Positive
S	>64	R	>64	R*	<=4	S	<=1	S	>4/76	R	Negative
I	>64	R	>64	R*	>8	R	<=1	S	>4/76	R	Negative
I	>64	R	>64	R	8	I	<=1	S	<=2/38	S	Negative
S	>64	R	>64	R	<=4	S	<=1	S	<=2/38	S	Positive

S	>64	R	>64	R*	<=4	S	<=1	S	>4/76	R	Negative
S	64	I	>64	R*	<=4	S	<=1	S	>4/76	R	Negative
S	>64	R	>64	R	>8	R	<=1	S	<=2/38	S	Negative
R	>64	R	>64	R*	>8	R	<=1	S	>4/76	R	Negative
S	>64	R	>64	R*	<=4	S	<=1	S	>4/76	R	Negative
S	>64	R	>64	R	<=4	S	>2	R	>4/76	R	Negative
S	>64	R	>64	R	<=4	S	<=1	S	>4/76	R	Negative
I	>64	R	>64	R*	8	I	2	I	>4/76	R	Negative
I	64	I	>64	R	>8	R	<=1	S	>4/76	R	Negative
S	>64	R	>64	R*	>8	R	<=1	S	>4/76	R	Negative
S	64	I	>64	R*	<=4	S	<=1	S	>4/76	R	Negative
S	>64	R	>64	R*	>8	R	<=1	S	>4/76	R	Negative
S	>64	R	>64	R*	<=4	S	<=1	S	>4/76	R	Negative
R	>64	R	>64	R	>8	R	<=1	S	>4/76	R	Negative
S	>64	R	>64	R*	8	I	2	I	>4/76	R	Negative
R	>64	R	>64	R	>8	R	<=1	S	>4/76	R	Positive
I	>64	R	>64	R	>8	R	2	I	>4/76	R	Positive
S	>64	R	>64	R	>8	R	<=1	S	>4/76	R	Negative
S	>64	R	>64	R	>8	R	<=1	S	>4/76	R	Negative
I	>64	R	>64	R	>8	R	2	I	>4/76	R	Negative
R	>64	R	>64	R	>8	R	<=1	S	>4/76	R	Positive
R	>64	R	>64	R	>8	R	<=1	S	>4/76	R	Positive
S	>64	R	>64	R	>8	R	<=1	S	>4/76	R	Positive
R	>64	R	>64	R*	>8	R	2	I	>4/76	R	Negative
S	>64	R	>64	R	<=4	S	<=1	S	>4/76	R	Negative
S	<=8	S	>64	R*	>8	R	<=1	S	>4/76	R	Negative
S	>64	R	>64	R*	>8	R	<=1	S	>4/76	R	Negative
S	>64	R	>64	R	<=4	S	<=1	S	>4/76	R	Negative
S	>64	R	>64	R*	<=4	S	<=1	S	>4/76	R	Negative
I	>64	R	>64	R	>8	R	2	I	>4/76	R	Negative
S	>64	R	>64	R*	<=4	S	<=1	S	>4/76	R	Negative
S	>64	R	>64	R	8	I	>2	R	>4/76	R	Positive
S	64	I	>64	R*	<=4	S	<=1	S	>4/76	R	Negative
S	64	I	>64	R*	>8	R	<=1	S	>4/76	R	Negative
S	>64	R	>64	R	<=4	S	<=1	S	<=2/38	S	Positive
S	>64	R	>64	R*	<=4	S	<=1	S	>4/76	R	Negative
S	>64	R	>64	R*	>8	R	2	I	>4/76	R	Negative
S	>64	R	>64	R	<=4	S	<=1	S	>4/76	R	Negative
I	>64	R	>64	R	>8	R	<=1	S	>4/76	R	Negative
S	>64	R	>64	R*	<=4	S	<=1	S	>4/76	R	Negative
S	>64	R	>64	R*	<=4	S	<=1	S	>4/76	R	Negative
S	>64	R	>64	R*	>8	R	<=1	S	>4/76	R	Negative
I	>64	R	>64	R*	>8	R	<=1	S	>4/76	R	Negative
S	>64	R	>64	R*	>8	R	<=1	S	>4/76	R	Negative
I	>64	R	>64	R	>8	R	<=1	S	>4/76	R	Negative
S	>64	R	>64	R*	<=4	S	<=1	S	>4/76	R	Negative
S	>64	R	>64	R	8	I	<=1	S	>4/76	R	Negative
S	>64	R	>64	R	8	I	<=1	S	>4/76	R	Negative
S	>64	R	>64	R	8	I	<=1	S	>4/76	R	Negative
S	>64	R	>64	R*	<=4	S	<=1	S	>4/76	R	Negative
S	>64	R	>64	R*	<=4	S	<=1	S	>4/76	R	Negative
R	>64	R	>64	R*	>8	R	2	I	>4/76	R	Negative
S	>64	R	>64	R*	<=4	S	<=1	S	>4/76	R	Negative
S	>64	R	>64	R*	<=4	S	<=1	S	>4/76	R	Negative
S	64	I	>64	R*	>8	R	<=1	S	>4/76	R	Negative
S	>64	R	>64	R	>8	R	<=1	S	>4/76	R	Positive

S	>64	R	>64	R*	<=4	S	<=1	S	>4/76	R	Negative
S	>64	R	>64	R	>8	R	<=1	S	>4/76	R	Positive
S	>64	R	>64	R*	<=4	S	<=1	S	>4/76	R	Negative
S	>64	R	>64	R*	<=4	S	<=1	S	>4/76	R	Negative
S	>64	R	>64	R*	<=4	S	<=1	S	<=2/38	S	Negative
S	64	I	>64	R	<=4	S	<=1	S	>4/76	R	Negative
I	>64	R	>64	R	>8	R	<=1	S	>4/76	R	Positive
R	>64	R	>64	R	>8	R	<=1	S	>4/76	R	Positive
S	>64	R	>64	R*	>8	R	<=1	S	>4/76	R	Negative
S	64	I	>64	R*	>8	R	<=1	S	>4/76	R	Negative
S	>64	R	>64	R*	<=4	S	<=1	S	>4/76	R	Negative
S	>64	R	>64	R	<=4	S	<=1	S	>4/76	R	Negative
S	>64	R	>64	R	8	I	<=1	S	>4/76	R	Negative
S	>64	R	>64	R	<=4	S	<=1	S	>4/76	R	Negative
S	>64	R	>64	R	>8	R	<=1	S	<=2/38	S	Negative
S	64	I	>64	R	<=4	S	<=1	S	<=2/38	S	Negative
I	64	I	>64	R	>8	R	>2	R	<=2/38	S	Positive
R	>64	R	>64	R	>8	R	<=1	S	>4/76	R	Negative
R	>64	R	>64	R*	>8	R	<=1	S	>4/76	R	Negative
S	>64	R	>64	R	<=4	S	<=1	S	>4/76	R	Negative
S	>64	R	>64	R*	<=4	S	<=1	S	>4/76	R	Negative
S	64	I	>64	R*	<=4	S	<=1	S	>4/76	R	Negative
R	>64	R	>64	R	>8	R	<=1	S	>4/76	R	Positive
R	>64	R	>64	R*	>8	R	<=1	S	>4/76	R	Negative
S	>64	R	>64	R*	<=4	S	<=1	S	>4/76	R	Negative
S	>64	R	>64	R*	8	I	2	I	>4/76	R	Negative
S	>64	R	>64	R*	>8	R	<=1	S	>4/76	R	Negative
I	>64	R	>64	R	>8	R	2	I	>4/76	R	Negative
S	>64	R	>64	R	>8	R	<=1	S	>4/76	R	Negative
S	64	I	>64	R*	>8	R	<=1	S	>4/76	R	Negative
S	64	I	>64	R	<=4	S	<=1	S	<=2/38	S	Negative
S	>64	R	>64	R*	>8	R	<=1	S	>4/76	R	Negative
S	64	I	>64	R*	>8	R	<=1	S	>4/76	R	Negative
S	>64	R	>64	R*	8	I	<=1	S	>4/76	R	Negative
S	>64	R	>64	R	8	I	<=1	S	>4/76	R	Negative
S	>64	R	>64	R	8	I	<=1	S	<=2/38	S	Negative
R	<=8	S	<=8	S	>8	R	<=1	S	<=2/38	S	Negative
S	>64	R	>64	R	<=4	S	<=1	S	>4/76	R	Negative
R	64	I	>64	R	>8	R	>2	R	<=2/38	S	Negative
S	>64	R	>64	R*	<=4	S	<=1	S	>4/76	R	Negative
I	64	I	>64	R*	<=4	S	<=1	S	>4/76	R	Negative
S	>64	R	>64	R*	<=4	S	<=1	S	>4/76	R	Negative
S	>64	R	>64	R*	>8	R	<=1	S	>4/76	R	Negative
S	64	I	>64	R	>8	R	<=1	S	>4/76	R	Negative
S	>64	R	>64	R	<=4	S	<=1	S	>4/76	R	Negative
I	>64	R	>64	R	>8	R	<=1	S	>4/76	R	Negative
S	>64	R	>64	R	8	I	<=1	S	>4/76	R	Negative
S	>64	R	>64	R	<=4	S	<=1	S	>4/76	R	Negative
R	>64	R	>64	R*	>8	R	2	I	<=2/38	S	Negative
S	16	S	64	I	8	I	2	I	>4/76	R	Negative
S	16	S	64	I	>8	R	<=1	S	>4/76	R	Negative
I	>64	R	>64	R	8	I	2	I	>4/76	R	Negative
I	16	S	>64	R*	>8	R	2	I	>4/76	R	Negative
I	<=8	S	>64	R	>8	R	<=1	S	>4/76	R	Negative
S	<=8	S	64	I	>8	R	<=1	S	>4/76	R	Negative

S	<=8	S	>64	R*	>8	R	<=1	S	>4/76	R	Negative
S	64	I	>64	R	<=4	S	<=1	S	>4/76	R	Negative
S	<=8	S	<=8	S	<=4	S	<=1	S	<=2/38	S	Negative
S	>64	R	>64	R	<=4	S	<=1	S	>4/76	R	Positive
S	>64	R	>64	R	>8	R	<=1	S	>4/76	R	Negative
S	>64	R	>64	R	8	I	<=1	S	>4/76	R	Negative
S	64	I	>64	R*	<=4	S	<=1	S	>4/76	R	Negative
S	>64	R	>64	R*	>8	R	<=1	S	>4/76	R	Negative
S	>64	R	>64	R	>8	R	<=1	S	>4/76	R	Negative
I	>64	R	>64	R	>8	R	2	I	>4/76	R	Negative
R	>64	R	>64	R	>8	R	>2	R	>4/76	R	Negative
S	>64	R	>64	R	<=4	S	<=1	S	>4/76	R	Positive
S	>64	R	>64	R	>8	R	<=1	S	>4/76	R	Negative
R	>64	R	>64	R	>8	R	<=1	S	>4/76	R	Negative
S	>64	R	>64	R*	<=4	S	<=1	S	>4/76	R	Negative
I	>64	R	>64	R*	8	I	2	I	>4/76	R	Positive
S	<=8	S	<=8	S	<=4	S	<=1	S	<=2/38	S	Positive
R	>64	R	>64	R	>8	R	<=1	S	>4/76	R	Negative
R	>64	R	>64	R	>8	R	<=1	S	>4/76	R	Negative
S	>64	R	>64	R	<=4	S	<=1	S	>4/76	R	Negative
I	>64	R	>64	R*	>8	R	<=1	S	>4/76	R	Negative
S	>64	R	>64	R*	8	I	<=1	S	>4/76	R	Negative
R	>64	R	>64	R	>8	R	<=1	S	>4/76	R	Positive
I	>64	R	>64	R	>8	R	<=1	S	>4/76	R	Negative
I	>64	R	>64	R	>8	R	<=1	S	>4/76	R	Negative
R	>64	R	>64	R	>8	R	<=1	S	>4/76	R	Negative
R	>64	R	>64	R*	>8	R	<=1	S	>4/76	R	Negative
I	>64	R	>64	R	>8	R	2	I	>4/76	R	Negative
S	>64	R	>64	R	<=4	S	<=1	S	>4/76	R	Negative
S	>64	R	>64	R	<=4	S	<=1	S	>4/76	R	Negative
R	>64	R	>64	R*	>8	R	<=1	S	>4/76	R	Negative
R	>64	R	>64	R	>8	R	2	I	>4/76	R	Negative
R	>64	R	>64	R	>8	R	2	I	>4/76	R	Negative
S	>64	R	>64	R*	<=4	S	<=1	S	>4/76	R	Negative
S	64	I	>64	R	8	I	<=1	S	<=2/38	S	Negative
R	>64	R	>64	R	>8	R	2	I	>4/76	R	Negative
R	64	I	>64	R*	>8	R	<=1	S	>4/76	R	Negative
I	>64	R	>64	R	>8	R	2	I	>4/76	R	Negative
S	>64	R	>64	R*	>8	R	<=1	S	>4/76	R	Negative
S	>64	R	>64	R*	>8	R	2	I	>4/76	R	Negative
S	>64	R	>64	R*	<=4	S	<=1	S	>4/76	R	Negative
S	>64	R	>64	R	<=4	S	<=1	S	>4/76	R	Negative
S	>64	R	>64	R*	<=4	S	<=1	S	>4/76	R	Negative
I	>64	R	>64	R*	>8	R	<=1	S	>4/76	R	Negative
I	>64	R	>64	R*	>8	R	<=1	S	>4/76	R	Negative
R	64	I	>64	R*	>8	R	<=1	S	>4/76	R	Negative
S	>64	R	>64	R*	>8	R	<=1	S	>4/76	R	Negative
S	<=8	S	>64	R	<=4	S	<=1	S	<=2/38	S	Negative
I	>64	R	>64	R*	>8	R	2	I	>4/76	R	Negative
S	>64	R	>64	R*	>8	R	<=1	S	>4/76	R	Negative
S	>64	R	>64	R*	8	I	<=1	S	>4/76	R	Negative
S	>64	R	>64	R*	<=4	S	<=1	S	>4/76	R	Negative
S	>64	R	>64	R	<=4	S	<=1	S	>4/76	R	Positive
S	>64	R	>64	R*	<=4	S	<=1	S	>4/76	R	Negative
S	64	I	>64	R	<=4	S	<=1	S	>4/76	R	Negative
S	64	I	>64	R*	<=4	S	<=1	S	>4/76	R	Negative

S	64	I	>64	R	<=4	S	<=1	S	>4/76	R	Negative
S	>64	R	>64	R	<=4	S	<=1	S	<=2/38	S	Negative
S	>64	R	>64	R	>8	R	<=1	S	>4/76	R	Negative
S	>64	R	>64	R	<=4	S	<=1	S	>4/76	R	Negative
S	>64	R	>64	R	<=4	S	<=1	S	>4/76	R	Negative
S	<=8	S	<=8	S	<=4	S	<=1	S	<=2/38	S	Negative
I	64	I	<=8	S	>8	R	<=1	S	>4/76	R	Negative
S	>64	R	>64	R	<=4	S	<=1	S	>4/76	R	Positive
S	>64	R	>64	R*	<=4	S	<=1	S	>4/76	R	Negative
I	>64	R	>64	R	8	I	>2	R	>4/76	R	Negative
S	>64	R	>64	R	8	I	<=1	S	>4/76	R	Negative
S	>64	R	>64	R	8	I	<=1	S	>4/76	R	Negative
S	>64	R	>64	R	<=4	S	<=1	S	>4/76	R	Negative
S	>64	R	>64	R*	>8	R	<=1	S	>4/76	R	Negative
S	>64	R	>64	R	>8	R	<=1	S	>4/76	R	Negative
S	<=8	S	<=8	S	<=4	S	<=1	S	<=2/38	S	Negative
R	>64	R	>64	R	>8	R	<=1	S	>4/76	R	Negative
S	<=8	S	16	S	<=4	S	<=1	S	<=2/38	S	Negative
S	>64	R	>64	R	8	I	<=1	S	>4/76	R	Negative
I	>64	R	>64	R*	>8	R	>2	R	>4/76	R	Negative
S	>64	R	>64	R*	<=4	S	<=1	S	<=2/38	S	Negative
S	>64	R	>64	R*	<=4	S	<=1	S	>4/76	R	Negative
S	>64	R	>64	R	<=4	S	<=1	S	>4/76	R	Negative
S	>64	R	>64	R*	<=4	S	<=1	S	>4/76	R	Negative
I	>64	R	>64	R*	<=4	S	<=1	S	>4/76	R	Negative
S	>64	R	>64	R	<=4	S	<=1	S	>4/76	R	Positive
S	>64	R	>64	R	<=4	S	<=1	S	>4/76	R	Positive
S	>64	R	>64	R*	<=4	S	<=1	S	>4/76	R	Negative
R	<=8	S	>64	R	>8	R	<=1	S	>4/76	R	Negative
S	<=8	S	>64	R*	>8	R	<=1	S	>4/76	R	Negative
S	>64	R	>64	R	8	I	<=1	S	>4/76	R	Negative
R	64	I	>64	R	>8	R	<=1	S	<=2/38	S	Negative
S	>64	R	>64	R	8	I	<=1	S	>4/76	R	Negative
S	>64	R	>64	R*	<=4	S	<=1	S	>4/76	R	Negative
I	64	I	>64	R*	>8	R	<=1	S	>4/76	R	Negative
I	>64	R	>64	R	>8	R	<=1	S	>4/76	R	Negative
S	64	I	>64	R	<=4	S	<=1	S	>4/76	R	Negative
S	>64	R	>64	R*	<=4	S	<=1	S	>4/76	R	Negative
I	>64	R	>64	R*	>8	R	<=1	S	>4/76	R	Negative
S	>64	R	>64	R	8	I	<=1	S	>4/76	R	Negative
S	>64	R	>64	R*	8	I	<=1	S	>4/76	R	Negative
R	>64	R	>64	R	>8	R	<=1	S	>4/76	R	Positive
I	>64	R	>64	R	>8	R	<=1	S	>4/76	R	Negative
R	64	I	>64	R*	>8	R	<=1	S	>4/76	R	Negative
S	64	I	>64	R*	>8	R	<=1	S	>4/76	R	Negative
S	>64	R	>64	R	8	I	<=1	S	>4/76	R	Negative
S	64	I	>64	R	<=4	S	<=1	S	<=2/38	S	Negative
S	>64	R	>64	R*	<=4	S	<=1	S	>4/76	R	Negative
R	<=8	S	>64	R	>8	R	<=1	S	>4/76	R	Negative
S	>64	R	>64	R	8	I	<=1	S	>4/76	R	Negative
S	>64	R	>64	R	8	I	<=1	S	>4/76	R	Negative
S	>64	R	>64	R*	<=4	S	<=1	S	>4/76	R	Negative
S	>64	R	>64	R	<=4	S	<=1	S	>4/76	R	Negative
S	>64	R	>64	R*	<=4	S	<=1	S	>4/76	R	Negative
S	>64	R	>64	R*	<=4	S	<=1	S	>4/76	R	Negative
S	<=8	S	>64	R*	>8	R	2	I	>4/76	R	Negative

I	>64	R	>64	R	>8	R	<=1	S	>4/76	R	Negative
S	>64	R	>64	R	>8	R	<=1	S	>4/76	R	Negative
R	>64	R	>64	R	>8	R	<=1	S	>4/76	R	Positive
S	>64	R	>64	R	8	I	<=1	S	>4/76	R	Negative
R	>64	R	>64	R	>8	R	2	I	>4/76	R	Negative
S	>64	R	>64	R	>8	R	<=1	S	>4/76	R	Negative
S	>64	R	>64	R	8	I	<=1	S	>4/76	R	Negative
S	>64	R	>64	R	>8	R	<=1	S	>4/76	R	Positive
S	>64	R	>64	R*	<=4	S	<=1	S	>4/76	R	Negative
R	>64	R	>64	R	>8	R	<=1	S	>4/76	R	Positive
I	>64	R	>64	R*	8	I	2	I	<=2/38	S	Negative
S	>64	R	>64	R*	8	I	2	I	>4/76	R	Negative
S	<=8	S	>64	R*	>8	R	<=1	S	>4/76	R	Negative
S	>64	R	>64	R	8	I	<=1	S	>4/76	R	Negative
S	>64	R	>64	R	<=4	S	<=1	S	>4/76	R	Negative
S	>64	R	>64	R	<=4	S	<=1	S	>4/76	R	Negative
S	>64	R	>64	R	<=4	S	<=1	S	>4/76	R	Negative
I	>64	R	>64	R	>8	R	<=1	S	>4/76	R	Negative
R	>64	R	>64	R	>8	R	2	I	>4/76	R	Negative
R	>64	R	>64	R	>8	R	2	I	>4/76	R	Negative
S	64	I	>64	R	<=4	S	<=1	S	<=2/38	S	Negative
R	>64	R	>64	R*	>8	R	2	I	>4/76	R	Negative
S	>64	R	>64	R*	<=4	S	<=1	S	>4/76	R	Negative

I	>64	R	>64	R	>8	R	>2	R	>4/76	R	Positive
S	>64	R	>64	R	8	I	<=1	S	>4/76	R	Negative

S	64	I	>64	R*	>8	R	<=1	S	>4/76	R	Negative
S	>64	R	>64	R	8	I	<=1	S	>4/76	R	Negative
I	>64	R	>64	R*	>8	R	<=1	S	>4/76	R	Negative
S	>64	R	>64	R	<=4	S	<=1	S	>4/76	R	Negative

R	>64	R	>64	R*	>8	R	<=1	S	>4/76	R	Negative
S	>64	R	>64	R*	<=4	S	<=1	S	>4/76	R	Negative
R	>64	R	>64	R	>8	R	<=1	S	>4/76	R	Negative
R	>64	R	>64	R	>8	R	<=1	S	>4/76	R	Negative
I	>64	R	>64	R	>8	R	2	I	>4/76	R	Negative
S	>64	R	>64	R	>8	R	<=1	S	>4/76	R	Negative
S	>64	R	>64	R	<=4	S	<=1	S	>4/76	R	Negative
S	16	S	>64	R*	>8	R	<=1	S	>4/76	R	Negative
R	>64	R	>64	R	>8	R	>2	R	>4/76	R	Negative
I	>64	R	>64	R*	8	I	2	I	>4/76	R	Negative
S	>64	R	>64	R*	8	I	<=1	S	>4/76	R	Negative
R	>64	R	>64	R	>8	R	<=1	S	>4/76	R	Positive
R	>64	R	>64	R	>8	R	<=1	S	>4/76	R	Negative
S	>64	R	>64	R	<=4	S	<=1	S	>4/76	R	Negative
S	>64	R	>64	R*	8	I	<=1	S	>4/76	R	Negative
S	>64	R	>64	R	<=4	S	<=1	S	>4/76	R	Negative
S	>64	R	>64	R*	<=4	S	<=1	S	>4/76	R	Negative
S	64	I	>64	R*	<=4	S	<=1	S	<=2/38	S	Negative
S	>64	R	>64	R	<=4	S	<=1	S	>4/76	R	Positive
S	>64	R	>64	R*	<=4	S	<=1	S	>4/76	R	Negative
S	64	I	>64	R*	>8	R	<=1	S	<=2/38	S	Negative
S	>64	R	>64	R	<=4	S	<=1	S	>4/76	R	Negative
I	>64	R	>64	R*	>8	R	>2	R	>4/76	R	Negative
S	>64	R	>64	R*	>8	R	<=1	S	>4/76	R	Negative
S	>64	R	>64	R	8	I	<=1	S	>4/76	R	Negative
S	>64	R	>64	R	<=4	S	<=1	S	>4/76	R	Negative
S	>64	R	>64	R*	<=4	S	<=1	S	>4/76	R	Negative
S	>64	R	>64	R	<=4	S	<=1	S	>4/76	R	Negative
S	>64	R	>64	R*	<=4	S	<=1	S	>4/76	R	Negative
R	>64	R	>64	R*	>8	R	>2	R	>4/76	R	Negative
S	>64	R	>64	R*	>8	R	<=1	S	>4/76	R	Negative
S	>64	R	>64	R	<=4	S	<=1	S	>4/76	R	Negative
S	>64	R	>64	R	<=4	S	<=1	S	>4/76	R	Negative
S	>64	R	>64	R*	<=4	S	<=1	S	>4/76	R	Negative
S	>64	R	>64	R*	<=4	S	<=1	S	>4/76	R	Negative
S	>64	R	>64	R	<=4	S	<=1	S	>4/76	R	Negative
S	64	I	>64	R*	<=4	S	<=1	S	>4/76	R	Negative
S	16	S	>64	R*	<=4	S	<=1	S	>4/76	R	Negative
S	64	I	>64	R	<=4	S	<=1	S	>4/76	R	Negative
S	>64	R	>64	R	<=4	S	<=1	S	>4/76	R	Negative

S	>64	R	>64	R	<=4	S	<=1	S	>4/76	R	Negative
S	>64	R	>64	R	<=4	S	<=1	S	>4/76	R	Negative
S	>64	R	>64	R*	<=4	S	<=1	S	<=2/38	S	Negative
R	>64	R	>64	R	>8	R	2	I	>4/76	R	Positive
I	>64	R	>64	R*	>8	R	<=1	S	>4/76	R	Negative
S	>64	R	>64	R*	<=4	S	<=1	S	>4/76	R	Negative
S	>64	R	>64	R*	<=4	S	<=1	S	>4/76	R	Negative
S	>64	R	>64	R	<=4	S	<=1	S	>4/76	R	Negative
S	>64	R	>64	R	<=4	S	<=1	S	>4/76	R	Negative
I	>64	R	>64	R	>8	R	<=1	S	>4/76	R	Negative
S	<=8	S	>64	R	<=4	S	<=1	S	>4/76	R	Negative
S	<=8	S	>64	R	>8	R	<=1	S	>4/76	R	Negative
S	<=8	S	<=8	S	<=4	S	<=1	S	<=2/38	S	Negative
S	<=8	S	<=8	S	>8	R	<=1	S	<=2/38	S	Negative
S	>64	R	>64	R	<=4	S	<=1	S	>4/76	R	Negative
S	<=8	S	<=8	S	<=4	S	<=1	S	<=2/38	S	Negative
S	<=8	S	16	S	>8	R	<=1	S	<=2/38	S	Negative
S	<=8	S	<=8	S	<=4	S	<=1	S	<=2/38	S	Negative
S	<=8	S	16	S	<=4	S	<=1	S	<=2/38	S	Negative
S	<=8	S	16	S	<=4	S	<=1	S	<=2/38	S	Negative
S	>64	R	>64	R*	<=4	S	<=1	S	>4/76	R	Negative
I	>64	R	>64	R	>8	R	<=1	S	>4/76	R	Negative
S	>64	R	>64	R	>8	R	<=1	S	>4/76	R	Negative
S	>64	R	>64	R*	<=4	S	<=1	S	>4/76	R	Negative
S	>64	R	>64	R*	<=4	S	<=1	S	>4/76	R	Negative
S	>64	R	>64	R*	8	I	<=1	S	>4/76	R	Negative
I	>64	R	>64	R	>8	R	2	I	>4/76	R	Negative
S	>64	R	>64	R*	<=4	S	<=1	S	>4/76	R	Negative
R	>64	R	>64	R*	>8	R	<=1	S	>4/76	R	Negative
I	>64	R	>64	R	>8	R	<=1	S	>4/76	R	Negative
S	>64	R	>64	R*	<=4	S	<=1	S	>4/76	R	Negative
R	>64	R	>64	R	>8	R	>2	R	>4/76	R	Negative
R	>64	R	>64	R	>8	R	>2	R	>4/76	R	Negative
S	>64	R	>64	R*	<=4	S	<=1	S	>4/76	R	Negative
S	64	I	>64	R*	<=4	S	<=1	S	>4/76	R	Negative
S	64	I	>64	R	>8	R	<=1	S	<=2/38	S	Negative
S	64	I	>64	R*	>8	R	<=1	S	>4/76	R	Negative
S	64	I	>64	R*	>8	R	<=1	S	>4/76	R	Negative
S	>64	R	>64	R*	>8	R	<=1	S	>4/76	R	Negative
S	>64	R	>64	R	>8	R	<=1	S	<=2/38	S	Negative
S	>64	R	>64	R*	<=4	S	<=1	S	>4/76	R	Negative
I	>64	R	>64	R	8	I	2	I	>4/76	R	Negative
S	64	I	>64	R	<=4	S	<=1	S	>4/76	R	Negative
S	>64	R	>64	R	>8	R	<=1	S	<=2/38	S	Negative
I	>64	R	>64	R	8	I	2	I	>4/76	R	Negative
S	>64	R	>64	R	<=4	S	<=1	S	>4/76	R	Negative
S	>64	R	>64	R	<=4	S	<=1	S	>4/76	R	Negative
S	>64	R	>64	R	<=4	S	<=1	S	>4/76	R	Negative
S	>64	R	>64	R	<=4	S	<=1	S	>4/76	R	Negative
R	>64	R	>64	R	>8	R	<=1	S	>4/76	R	Negative
S	>64	R	>64	R	8	I	2	I	>4/76	R	Negative
S	>64	R	>64	R	>8	R	<=1	S	>4/76	R	Negative
S	>64	R	>64	R	<=4	S	<=1	S	>4/76	R	Negative
S	64	I	>64	R*	<=4	S	<=1	S	>4/76	R	Negative
R	>64	R	>64	R*	>8	R	<=1	S	>4/76	R	Negative
S	16	S	>64	R*	>8	R	<=1	S	>4/76	R	Negative

S	>64	R	>64	R	>8	R	<=1	S	>4/76	R	Negative
S	>64	R	>64	R	<=4	S	<=1	S	<=2/38	S	Positive
S	>64	R	>64	R*	<=4	S	<=1	S	>4/76	R	Negative
S	64	I	>64	R*	<=4	S	<=1	S	>4/76	R	Negative
S	>64	R	>64	R*	<=4	S	<=1	S	>4/76	R	Negative
S	>64	R	>64	R	<=4	S	<=1	S	>4/76	R	Positive
I	>64	R	>64	R	8	I	2	I	>4/76	R	Negative
I	64	I	64	I	>8	R	<=1	S	<=2/38	S	Negative
R	>64	R	>64	R*	>8	R	>2	R	>4/76	R	Negative
S	>64	R	>64	R*	<=4	S	<=1	S	>4/76	R	Negative
R	>64	R	>64	R	>8	R	2	I	>4/76	R	Negative
S	>64	R	>64	R	8	I	>2	R	>4/76	R	Negative
S	>64	R	>64	R	<=4	S	<=1	S	>4/76	R	Negative
I	>64	R	>64	R*	8	I	2	I	<=2/38	S	Negative
S	64	I	>64	R*	>8	R	<=1	S	>4/76	R	Negative
S	64	I	>64	R*	>8	R	<=1	S	>4/76	R	Negative
S	>64	R	>64	R	>8	R	<=1	S	>4/76	R	Negative
S	>64	R	>64	R*	<=4	S	<=1	S	>4/76	R	Negative
S	>64	R	>64	R*	<=4	S	<=1	S	>4/76	R	Negative
I	64	I	>64	R*	8	I	2	I	>4/76	R	Negative
R	>64	R	>64	R	>8	R	2	I	<=2/38	S	Negative
R	64	I	>64	R*	>8	R	<=1	S	<=2/38	S	Negative
I	>64	R	>64	R*	<=4	S	2	I	>4/76	R	Negative
S	>64	R	>64	R	<=4	S	<=1	S	>4/76	R	Negative
R	>64	R	>64	R	>8	R	2	I	>4/76	R	Negative
S	>64	R	>64	R	<=4	S	<=1	S	>4/76	R	Negative
S	>64	R	>64	R	8	I	2	I	>4/76	R	Negative
R	>64	R	>64	R	>8	R	<=1	S	>4/76	R	Negative
R	>64	R	>64	R*	>8	R	2	I	>4/76	R	Negative
S	64	I	>64	R*	<=4	S	<=1	S	>4/76	R	Negative
R	>64	R	>64	R	>8	R	2	I	>4/76	R	Negative
S	>64	R	>64	R	>8	R	<=1	S	>4/76	R	Negative
I	>64	R	>64	R	>8	R	2	I	>4/76	R	Negative
R	>64	R	>64	R	>8	R	<=1	S	>4/76	R	Negative
S	>64	R	>64	R	<=4	S	<=1	S	>4/76	R	Negative
R	>64	R	>64	R	>8	R	<=1	S	>4/76	R	Negative
R	>64	R	>64	R	>8	R	<=1	S	>4/76	R	Negative
R	>64	R	>64	R	8	I	<=1	S	>4/76	R	Negative
S	>64	R	>64	R*	>8	R	<=1	S	>4/76	R	Negative
S	64	I	>64	R*	>8	R	<=1	S	>4/76	R	Negative
R	>64	R	>64	R	>8	R	<=1	S	>4/76	R	Positive
S	>64	R	>64	R	<=4	S	<=1	S	>4/76	R	Negative
S	>64	R	>64	R	8	I	<=1	S	>4/76	R	Negative
R	>64	R	>64	R	>8	R	>2	R	<=2/38	S	Positive
R	64	I	>64	R	>8	R	>2	R	>4/76	R	Negative
R	>64	R	>64	R	>8	R	<=1	S	>4/76	R	Negative
I	>64	R	>64	R	8	I	2	I	>4/76	R	Negative
S	>64	R	>64	R	>8	R	<=1	S	<=2/38	S	Negative
S	>64	R	>64	R	<=4	S	<=1	S	>4/76	R	Negative
I	64	I	64	I	<=4	S	>2	R	>4/76	R	Positive
S	64	I	>64	R*	8	I	<=1	S	<=2/38	S	Negative
R	64	I	>64	R*	>8	R	2	I	>4/76	R	Negative
S	>64	R	>64	R	<=4	S	<=1	S	>4/76	R	Negative
S	>64	R	>64	R	<=4	S	<=1	S	>4/76	R	Positive
S	>64	R	>64	R*	<=4	S	<=1	S	>4/76	R	Negative

S	64	I	>64	R*	<=4	S	<=1	S	>4/76	R	Negative
I	>64	R	>64	R	8	I	<=1	S	>4/76	R	Negative
S	>64	R	>64	R*	<=4	S	<=1	S	>4/76	R	Negative
S	>64	R	>64	R*	<=4	S	<=1	S	>4/76	R	Negative
S	>64	R	>64	R*	<=4	S	<=1	S	>4/76	R	Negative
S	>64	R	>64	R	>8	R	<=1	S	>4/76	R	Negative
R	>64	R	>64	R	>8	R	<=1	S	>4/76	R	Negative
S	>64	R	>64	R	<=4	S	<=1	S	<=2/38	S	Positive
S	>64	R	>64	R	<=4	S	<=1	S	<=2/38	S	Positive
S	>64	R	>64	R*	<=4	S	<=1	S	>4/76	R	Negative
S	64	I	64	I	>8	R	<=1	S	<=2/38	S	Negative
S	>64	R	>64	R*	>8	R	2	I	>4/76	R	Positive
S	>64	R	>64	R*	<=4	S	<=1	S	>4/76	R	Negative
I	>64	R	>64	R	>8	R	<=1	S	>4/76	R	Negative
S	>64	R	>64	R	<=4	S	<=1	S	>4/76	R	Positive
I	>64	R	>64	R	8	I	2	I	>4/76	R	Positive
S	>64	R	>64	R	<=4	S	<=1	S	>4/76	R	Negative
S	>64	R	>64	R	<=4	S	<=1	S	>4/76	R	Negative
S	>64	R	>64	R*	>8	R	<=1	S	>4/76	R	Negative
S	>64	R	>64	R	<=4	S	<=1	S	>4/76	R	Negative
I	>64	R	>64	R	>8	R	2	I	>4/76	R	Negative
S	>64	R	>64	R	>8	R	<=1	S	<=2/38	S	Negative
S	>64	R	>64	R	<=4	S	<=1	S	>4/76	R	Negative
I	>64	R	>64	R	>8	R	2	I	>4/76	R	Negative
S	64	I	>64	R*	>8	R	<=1	S	>4/76	R	Negative
S	64	I	>64	R*	>8	R	<=1	S	>4/76	R	Negative
S	>64	R	>64	R*	<=4	S	<=1	S	>4/76	R	Negative
S	>64	R	>64	R	<=4	S	<=1	S	>4/76	R	Positive
S	64	I	>64	R*	<=4	S	<=1	S	>4/76	R	Negative
S	>64	R	>64	R	<=4	S	<=1	S	>4/76	R	Positive
S	>64	R	>64	R	<=4	S	<=1	S	>4/76	R	Negative
S	>64	R	>64	R	<=4	S	<=1	S	>4/76	R	Positive
S	>64	R	>64	R	<=4	S	<=1	S	>4/76	R	Negative
S	>64	R	>64	R*	<=4	S	<=1	S	>4/76	R	Negative
S	>64	R	>64	R*	<=4	S	<=1	S	>4/76	R	Negative
S	64	I	>64	R*	<=4	S	<=1	S	>4/76	R	Negative
S	>64	R	>64	R	<=4	S	<=1	S	>4/76	R	Positive
S	>64	R	>64	R	<=4	S	<=1	S	>4/76	R	Positive
S	>64	R	>64	R*	<=4	S	<=1	S	>4/76	R	Negative
R	>64	R	>64	R	>8	R	<=1	S	>4/76	R	Negative
S	>64	R	>64	R	>8	R	<=1	S	>4/76	R	Negative
R	>64	R	>64	R	>8	R	<=1	S	>4/76	R	Negative
S	>64	R	>64	R	8	I	<=1	S	>4/76	R	Negative
I	>64	R	>64	R	>8	R	<=1	S	>4/76	R	Negative
S	64	I	>64	R*	<=4	S	<=1	S	>4/76	R	Negative
I	>64	R	>64	R	>8	R	2	I	>4/76	R	Negative
S	>64	R	>64	R	>8	R	<=1	S	>4/76	R	Negative
S	64	I	>64	R*	<=4	S	<=1	S	>4/76	R	Negative
S	>64	R	>64	R	>8	R	<=1	S	>4/76	R	Negative
R	>64	R	>64	R*	>8	R	<=1	S	>4/76	R	Negative
S	>64	R	>64	R	<=4	S	<=1	S	>4/76	R	Negative
S	>64	R	>64	R	>8	R	<=1	S	>4/76	R	Negative
S	>64	R	>64	R*	>8	R	<=1	S	>4/76	R	Negative
S	64	I	>64	R*	<=4	S	<=1	S	>4/76	R	Negative
S	>64	R	>64	R*	<=4	S	<=1	S	>4/76	R	Negative
S	>64	R	>64	R	>8	R	<=1	S	>4/76	R	Negative

S	>64	R	>64	R	>8	R	<=1	S	>4/76	R	Negative
I	16	S	16	S	>8	R	2	I	>4/76	R	Negative
S	>64	R	>64	R	<=4	S	<=1	S	>4/76	R	Negative
S	>64	R	>64	R*	<=4	S	<=1	S	>4/76	R	Negative

S	<=8	S	<=8	S	>8	R	<=1	S	<=2/38	S	Negative
R	>64	R	>64	R	>8	R	>2	R	>4/76	R	Negative
S	>64	R	>64	R*	<=4	S	<=1	S	>4/76	R	Negative
S	>64	R	>64	R*	<=4	S	<=1	S	>4/76	R	Negative
R	>64	R	>64	R	>8	R	<=1	S	>4/76	R	Negative
I	>64	R	>64	R	<=4	S	<=1	S	>4/76	R	Negative
I	>64	R	>64	R	8	I	2	I	>4/76	R	Negative
S	>64	R	>64	R	<=4	S	<=1	S	<=2/38	S	Positive
S	>64	R	>64	R	<=4	S	<=1	S	>4/76	R	Negative
S	64	I	>64	R*	<=4	S	<=1	S	>4/76	R	Negative
R	>64	R	>64	R*	>8	R	<=1	S	>4/76	R	Negative
S	64	I	>64	R	<=4	S	<=1	S	<=2/38	S	Negative
R	>64	R	>64	R*	>8	R	2	I	>4/76	R	Positive
I	64	I	64	I	>8	R	2	I	<=2/38	S	Negative
S	>64	R	>64	R*	8	I	<=1	S	>4/76	R	Negative
I	>64	R	>64	R	8	I	<=1	S	>4/76	R	Negative
S	>64	R	64	I	<=4	S	2	I	>4/76	R	Positive
I	>64	R	>64	R	>8	R	<=1	S	>4/76	R	Negative
S	>64	R	>64	R	<=4	S	<=1	S	>4/76	R	Negative
S	>64	R	>64	R*	>8	R	<=1	S	>4/76	R	Negative
S	<=8	S	16	S	<=4	S	<=1	S	<=2/38	S	Negative
R	64	I	64	I	>8	R	2	I	>4/76	R	Positive
R	>64	R	>64	R	>8	R	>2	R	>4/76	R	Negative
S	>64	R	>64	R	<=4	S	<=1	S	>4/76	R	Negative
I	>64	R	>64	R	>8	R	<=1	S	>4/76	R	Negative
S	>64	R	>64	R*	>8	R	<=1	S	>4/76	R	Negative
S	>64	R	>64	R	>8	R	2	I	>4/76	R	Positive
R	>64	R	>64	R*	>8	R	<=1	S	>4/76	R	Negative
R	>64	R	>64	R*	>8	R	<=1	S	>4/76	R	Negative
R	>64	R	>64	R*	>8	R	>2	R	>4/76	R	Negative
S	64	I	>64	R*	<=4	S	<=1	S	>4/76	R	Negative
S	>64	R	>64	R	8	I	<=1	S	>4/76	R	Negative
S	>64	R	>64	R*	<=4	S	<=1	S	>4/76	R	Negative

S	>64	R	>64	R*	<=4	S	<=1	S	>4/76	R	Negative
R	>64	R	>64	R	>8	R	<=1	S	>4/76	R	Positive
I	>64	R	>64	R*	>8	R	2	I	>4/76	R	Negative
S	>64	R	>64	R	<=4	S	<=1	S	>4/76	R	Negative
S	64	I	>64	R*	<=4	S	<=1	S	>4/76	R	Negative
S	>64	R	>64	R	<=4	S	<=1	S	>4/76	R	Negative
S	>64	R	>64	R*	<=4	S	<=1	S	>4/76	R	Negative
R	>64	R	>64	R	>8	R	2	I	>4/76	R	Positive
S	>64	R	>64	R*	<=4	S	<=1	S	>4/76	R	Negative
S	>64	R	>64	R*	<=4	S	<=1	S	>4/76	R	Negative
I	>64	R	>64	R	>8	R	2	I	>4/76	R	Negative
R	>64	R	>64	R	>8	R	2	I	>4/76	R	Positive
S	>64	R	>64	R	8	I	<=1	S	>4/76	R	Negative
R	>64	R	>64	R*	>8	R	<=1	S	>4/76	R	Negative
S	>64	R	>64	R*	<=4	S	<=1	S	>4/76	R	Negative
S	>64	R	>64	R	<=4	S	<=1	S	>4/76	R	Negative
S	64	I	>64	R	<=4	S	<=1	S	>4/76	R	Negative
S	>64	R	>64	R*	<=4	S	<=1	S	>4/76	R	Negative
S	>64	R	>64	R	8	I	2	I	>4/76	R	Negative
R	>64	R	>64	R	>8	R	>2	R	>4/76	R	Positive
I	>64	R	>64	R	8	I	2	I	>4/76	R	Negative
S	>64	R	>64	R*	<=4	S	<=1	S	>4/76	R	Negative
S	>64	R	>64	R*	<=4	S	<=1	S	>4/76	R	Negative
I	>64	R	>64	R	>8	R	2	I	>4/76	R	Negative
S	16	S	>64	R*	<=4	S	<=1	S	>4/76	R	Negative
R	>64	R	>64	R	>8	R	2	I	>4/76	R	Positive
S	>64	R	>64	R	<=4	S	<=1	S	>4/76	R	Negative
R	>64	R	>64	R	>8	R	<=1	S	>4/76	R	Positive
S	>64	R	>64	R	8	I	<=1	S	>4/76	R	Positive
S	>64	R	>64	R*	<=4	S	<=1	S	>4/76	R	Negative
R	>64	R	>64	R*	>8	R	<=1	S	>4/76	R	Negative
S	>64	R	>64	R*	>8	R	<=1	S	>4/76	R	Negative
S	>64	R	>64	R	>8	R	<=1	S	<=2/38	S	Negative
R	>64	R	>64	R*	>8	R	<=1	S	>4/76	R	Negative
S	>64	R	>64	R*	8	I	<=1	S	>4/76	R	Negative
I	64	I	>64	R	>8	R	<=1	S	>4/76	R	Positive
S	>64	R	>64	R*	<=4	S	<=1	S	>4/76	R	Negative
S	64	I	>64	R*	>8	R	<=1	S	>4/76	R	Negative
I	>64	R	>64	R*	>8	R	<=1	S	>4/76	R	Negative
S	64	I	>64	R*	>8	R	<=1	S	>4/76	R	Negative
I	>64	R	>64	R	>8	R	<=1	S	>4/76	R	Negative
I	64	I	>64	R	>8	R	<=1	S	>4/76	R	Negative
I	64	I	>64	R*	>8	R	2	I	>4/76	R	Negative
I	>64	R	>64	R*	>8	R	<=1	S	>4/76	R	Negative
S	>64	R	>64	R*	<=4	S	<=1	S	>4/76	R	Negative
R	>64	R	>64	R*	>8	R	<=1	S	>4/76	R	Negative
S	>64	R	>64	R	<=4	S	<=1	S	>4/76	R	Negative
I	>64	R	>64	R	>8	R	<=1	S	>4/76	R	Positive
S	>64	R	>64	R*	<=4	S	<=1	S	>4/76	R	Negative
S	>64	R	>64	R*	<=4	S	<=1	S	>4/76	R	Negative
S	>64	R	>64	R*	<=4	S	<=1	S	>4/76	R	Negative
S	>64	R	>64	R*	<=4	S	<=1	S	>4/76	R	Negative
S	>64	R	>64	R	<=4	S	<=1	S	>4/76	R	Positive
S	64	I	>64	R*	<=4	S	<=1	S	>4/76	R	Negative
S	64	I	>64	I	>8	R	<=1	S	>4/76	R	Negative
S	>64	R	>64	R	>8	R	<=1	S	>4/76	R	Negative

S	>64	R	>64	R	>8	R	<=1	S	>4/76	R	Negative
S	>64	R	>64	R	<=4	S	<=1	S	>4/76	R	Negative
S	>64	R	>64	R	<=4	S	<=1	S	>4/76	R	Negative
I	>64	R	>64	R	>8	R	<=1	S	>4/76	R	Positive
S	>64	R	>64	R	<=4	S	<=1	S	>4/76	R	Negative
S	>64	R	>64	R	<=4	S	<=1	S	>4/76	R	Negative
R	>64	R	>64	R	>8	R	2	I	>4/76	R	Negative
S	>64	R	>64	R*	<=4	S	<=1	S	<=2/38	S	Negative
S	>64	R	>64	R	>8	R	<=1	S	<=2/38	S	Negative
S	>64	R	>64	R	<=4	S	<=1	S	>4/76	R	Negative
S	<=8	S	>64	R*	<=4	S	<=1	S	>4/76	R	Negative
S	>64	R	>64	R	>8	R	<=1	S	>4/76	R	Positive
S	>64	R	>64	R*	<=4	S	<=1	S	>4/76	R	Negative
S	>64	R	>64	R*	<=4	S	<=1	S	>4/76	R	Negative
R	>64	R	>64	R	>8	R	<=1	S	>4/76	R	Positive
S	>64	R	>64	R	<=4	S	<=1	S	>4/76	R	Negative
S	64	I	>64	R*	<=4	S	<=1	S	>4/76	R	Negative
S	>64	R	>64	R	>8	R	<=1	S	>4/76	R	Negative
S	<=8	S	64	I	<=4	S	<=1	S	<=2/38	S	Negative
S	>64	R	>64	R*	<=4	S	<=1	S	>4/76	R	Negative
S	>64	R	>64	R	<=4	S	<=1	S	>4/76	R	Negative
S	64	I	>64	R*	<=4	S	<=1	S	>4/76	R	Negative
S	>64	R	>64	R	<=4	S	<=1	S	>4/76	R	Negative
S	>64	R	>64	R	<=4	S	<=1	S	>4/76	R	Positive
R	>64	R	>64	R*	>8	R	<=1	S	>4/76	R	Negative
S	>64	R	>64	R	<=4	S	<=1	S	<=2/38	S	Negative
R	>64	R	>64	R	>8	R	<=1	S	>4/76	R	Positive
S	>64	R	>64	R*	<=4	S	<=1	S	>4/76	R	Negative
S	>64	R	>64	R*	<=4	S	<=1	S	>4/76	R	Negative
S	>64	R	>64	R*	>8	R	<=1	S	>4/76	R	Negative
S	>64	R	>64	R	<=4	S	<=1	S	>4/76	R	Negative
S	>64	R	>64	R*	>8	R	<=1	S	>4/76	R	Negative
S	>64	R	>64	R	<=4	S	<=1	S	>4/76	R	Positive
S	>64	R	>64	R	<=4	S	<=1	S	>4/76	R	Negative
S	>64	R	>64	R	<=4	S	<=1	S	>4/76	R	Negative
I	>64	R	>64	R	8	I	2	I	>4/76	R	Negative
S	>64	R	>64	R	>8	R	<=1	S	<=2/38	S	Negative
S	>64	R	>64	R	<=4	S	<=1	S	>4/76	R	Positive
R	>64	R	>64	R	>8	R	<=1	S	>4/76	R	Positive
S	>64	R	>64	R	<=4	S	<=1	S	>4/76	R	Negative
S	>64	R	>64	R*	8	I	<=1	S	>4/76	R	Negative
S	<=8	S	<=8	S	>8	R	<=1	S	<=2/38	S	Negative
I	64	I	>64	R*	>8	R	<=1	S	>4/76	R	Negative
I	>64	R	>64	R	>8	R	<=1	S	>4/76	R	Positive
S	>64	R	>64	R	<=4	S	<=1	S	>4/76	R	Positive
S	>64	R	>64	R*	<=4	S	<=1	S	>4/76	R	Negative
S	>64	R	>64	R	<=4	S	<=1	S	>4/76	R	Negative
I	>64	R	>64	R	8	I	<=1	S	>4/76	R	Negative
S	>64	R	>64	R	<=4	S	<=1	S	>4/76	R	Positive
S	>64	R	>64	R	<=4	S	<=1	S	>4/76	R	Positive
S	>64	R	>64	R*	<=4	S	<=1	S	>4/76	R	Negative
I	>64	R	>64	R	>8	R	<=1	S	>4/76	R	Positive
S	<=8	S	<=8	S	<=4	S	<=1	S	<=2/38	S	Negative
R	>64	R	>64	R	>8	R	<=1	S	>4/76	R	Positive
R	>64	R	>64	R	>8	R	2	I	>4/76	R	Negative
R	>64	R	>64	R	>8	R	2	I	>4/76	R	Negative

R	>64	R	>64	R	>8	R	<=1	S	>4/76	R	Negative
S	>64	R	>64	R*	<=4	S	<=1	S	>4/76	R	Negative
S	>64	R	>64	R	>8	R	<=1	S	<=2/38	S	Negative
S	>64	R	>64	R*	8	I	<=1	S	>4/76	R	Negative
R	>64	R	>64	R	>8	R	>2	R	>4/76	R	Negative
S	<=8	S	64	I	<=4	S	2	I	>4/76	R	Positive
S	>64	R	>64	R*	>8	R	<=1	S	4/76	R	Negative
R	>64	R	>64	R	>8	R	2	I	>4/76	R	Positive
S	64	I	>64	R	>8	R	<=1	S	>4/76	R	Negative
S	>64	R	>64	R	<=4	S	<=1	S	>4/76	R	Negative
S	>64	R	>64	R	<=4	S	<=1	S	>4/76	R	Negative
S	>64	R	>64	R	<=4	S	<=1	S	>4/76	R	Negative
S	>64	R	>64	R*	<=4	S	<=1	S	>4/76	R	Negative
S	>64	R	>64	R	<=4	S	<=1	S	>4/76	R	Positive
I	>64	R	>64	R	>8	R	<=1	S	>4/76	R	Negative
S	>64	R	>64	R	<=4	S	<=1	S	>4/76	R	Negative
R	>64	R	>64	R*	>8	R	>2	R	>4/76	R	Negative
S	>64	R	>64	R	<=4	S	<=1	S	<=2/38	S	Negative
S	>64	R	>64	R	<=4	S	<=1	S	>4/76	R	Positive
S	>64	R	>64	R	<=4	S	<=1	S	<=2/38	S	Positive
S	>64	R	>64	R	<=4	S	<=1	S	>4/76	R	Negative
I	>64	R	>64	R*	>8	R	2	I	4/76	R	Negative
S	>64	R	>64	R	<=4	S	<=1	S	>4/76	R	Positive
S	>64	R	>64	R*	<=4	S	<=1	S	>4/76	R	Negative
S	>64	R	>64	R*	<=4	S	<=1	S	>4/76	R	Negative
S	>64	R	>64	R	>8	R	<=1	S	>4/76	R	Negative
S	>64	R	>64	R	>8	R	<=1	S	>4/76	R	Negative
S	16	S	>64	R*	>8	R	<=1	S	>4/76	R	Negative
S	>64	R	>64	R	<=4	S	<=1	S	>4/76	R	Positive
R	>64	R	>64	R*	>8	R	<=1	S	>4/76	R	Negative
S	>64	R	>64	R*	>8	R	<=1	S	>4/76	R	Negative
R	>64	R	>64	R	>8	R	<=1	S	>4/76	R	Positive
S	>64	R	>64	R	>8	R	<=1	S	>4/76	R	Negative
R	<=8	S	>64	R	>8	R	<=1	S	>4/76	R	Negative
S	<=8	S	16	S	<=4	S	2	I	<=2/38	S	Negative
S	>64	R	>64	R	<=4	S	<=1	S	>4/76	R	Negative
S	>64	R	>64	R*	<=4	S	<=1	S	>4/76	R	Negative
I	64	I	>64	R	>8	R	<=1	S	<=2/38	S	Negative
S	>64	R	>64	R*	<=4	S	<=1	S	>4/76	R	Negative
S	<=8	S	64	I	<=4	S	<=1	S	<=2/38	S	Negative
S	64	I	>64	R*	<=4	S	<=1	S	>4/76	R	Negative
S	>64	R	>64	R*	<=4	S	<=1	S	>4/76	R	Negative
S	>64	R	>64	R	<=4	S	<=1	S	>4/76	R	Negative
I	>64	R	>64	R*	>8	R	<=1	S	>4/76	R	Negative
S	>64	R	>64	R	>8	R	<=1	S	<=2/38	S	Negative
S	>64	R	>64	R	>8	R	<=1	S	>4/76	R	Negative
S	64	I	>64	R*	>8	R	<=1	S	>4/76	R	Negative
S	>64	R	>64	R	<=4	S	<=1	S	>4/76	R	Positive
R	>64	R	>64	R	>8	R	2	I	>4/76	R	Positive
S	>64	R	>64	R*	>8	R	<=1	S	>4/76	R	Negative
S	>64	R	>64	R	8	I	<=1	S	>4/76	R	Negative
S	64	I	>64	R	<=4	S	<=1	S	<=2/38	S	Negative
R	>64	R	>64	R*	>8	R	<=1	S	>4/76	R	Negative
I	>64	R	64	I	>8	R	>2	R	4/76	R	Negative
S	>64	R	>64	R	<=4	S	2	I	>4/76	R	
R	>64	R	>64	R	>8	R	<=1	S	>4/76	R	Positive

R	>64	R	>64	R	>8	R	<=1	S	>4/76	R	Negative
R	>64	R	>64	R	>8	R	<=1	S	>4/76	R	Positive
S	16	S	64	I	>8	R	<=1	S	<=2/38	S	Negative
S	>64	R	>64	R	<=4	S	<=1	S	>4/76	R	Negative
I	>64	R	>64	R	>8	R	<=1	S	>4/76	R	Negative
S	>64	R	>64	R*	<=4	S	<=1	S	>4/76	R	Negative
S	>64	R	>64	R*	<=4	S	<=1	S	>4/76	R	Negative
S	64	I	64	I	<=4	S	<=1	S	<=2/38	S	Negative
S	>64	R	>64	R	>8	R	<=1	S	>4/76	R	Negative
S	>64	R	>64	R	<=4	S	<=1	S	>4/76	R	Negative
S	>64	R	>64	R	>8	R	<=1	S	>4/76	R	Negative
S	16	S	>64	R	>8	R	<=1	S	>4/76	R	Negative
R	>64	R	>64	R	>8	R	<=1	S	>4/76	R	Negative
R	>64	R	>64	R	>8	R	<=1	S	>4/76	R	Positive
S	>64	R	>64	R	<=4	S	<=1	S	>4/76	R	Negative
S	>64	R	>64	R*	<=4	S	<=1	S	>4/76	R	Negative
S	>64	R	>64	R	<=4	S	<=1	S	>4/76	R	Negative
S	>64	R	>64	R*	<=4	S	<=1	S	>4/76	R	Negative
S	>64	R	>64	R	<=4	S	<=1	S	>4/76	R	Positive
									>4/76	R	Negative
S	64	I	>64	R*	<=4	S	<=1	S	>4/76	R	Negative
I	>64	R	>64	R	>8	R	N/R		>4/76	R	Positive
S	>64	R	>64	R*	<=4	S	<1	S	>4/76	R	Negative
S	>64	R	>64	R*	8	I	2	I	>4/76	R	Negative
R	>64	R	>64	R	>8	R	<=1	S	>4/76	R	Negative
S	>64	R	>64	R*	<=4	S	<=1	S	>4/76	R	Negative
I	64	I	>64	R	>8	R	>2	R	>4/76	R	Negative
R	>64	R	>64	R	>8	R	<=1	S	>4/76	R	Positive
S	>64	R	>64	R*	<=4	S	<=1	S	>4/76	R	Negative
S	>64	R	>64	R	<=4	S	<=1	S	<=2/38	S	Positive
S	>64	R	>64	R*	<=4	S	<=1	S	>4/76	R	Negative
S	>64	R	>64	R*	8	I	<=1	S	4/76	R	Negative
S	<=8	S	<=8	S	<=4	S	<=1	S	<=2/38	S	Negative
S	>64	R	>64	R	8	I	<=1	S	>4/76	R	Positive
S	<=8	S	64	I	8	I	<=1	S	>4/76	R	Negative
R	>64	R	>64	R	>8	R	<=1	S	>4/76	R	Positive
S	<=8	S	<=8	R*	>8	R	<=1	S	<=2/38	S	Negative
S	64	I	>64	R*	<=4	S	<=1	S	>4/76	R	Negative
S	>64	R	>64	R	<=4	S	<=1	S	>4/76	R	Negative
S	64	I	>64	R*	8	I	<=1	S	>4/76	R	Negative
I	>64	R	>64	R	>8	R	<=1	S	>4/76	R	Negative
S	>64	R	>64	R	>8	R	N/R		>4/76	R	Positive
S	>64	R	>64	R*	<=4	S	<=1	S	>4/76	R	Negative
S	>64	R	>64	R	>8	R	<=1	S	>4/76	R	Negative
S	>64	R	>64	R	<=4	S	<=1	S	>4/76	R	Negative
R	>64	R	>64	R	>8	R	>2	R	>4/76	R	Negative
R	>64	R	>64	R	>8	R	2	I	>4/76	R	Negative
S	>64	R	>64	R	<=4	S	<=1	S	<=2/38	S	Positive
R	>64	R	>64	R	>8	R	>2	R	>4/76	R	Negative
S	>64	R	>64	R	<=4	S	<=1	S	>4/76	R	Positive
S	>64	R	>64	R	<=4	S	<=1	S	>4/76	R	Negative
S	<=8	S	<=8	S	<=4	S	<=1	S	<=2/38	S	Negative
S	>64	R	>64	R	<=4	S	<=1	S	>4/76	R	Negative
S	>64	R	>64	R	<=4	S	<=1	S	>4/76	R	Positive
S	>64	R	>64	R	<=4	S	<=1	S	>4/76	R	Negative

S	>64	R	>64	R*	<=4	S	<=1	S	>4/76	R	Negative
S	64	I	>64	R*	<=4	S	<=1	S	>4/76	R	Negative
S	>64	R	>64	R	<=4	S	<=1	S	>4/76	R	Positive
S	>64	R	>64	R	<=4	S	<=1	S	>4/76	R	Positive
S	>64	R	>64	R	<=4	S	<=1	S	<=2/38	S	Positive
R	>64	R	>64	R	>8	R	<=1	S	>4/76	R	Positive
R	>64	R	>64	R	>8	R	<=1	S	>4/76	R	Negative
S	>64	R	>64	R	<=4	S	<=1	S	>4/76	R	Positive
S	>64	R	>64	R	<=4	S	<=1	S	>4/76	R	Positive
S	>64	R	>64	R	<=4	S	<=1	S	>4/76	R	Negative
S	64	I	>64	R*	<=4	S	<=1	S	>4/76	R	Negative
S	>64	R	>64	R	<=4	S	2	I	>4/76	R	Negative
S	>64	R	>64	R*	<=4	S	<=1	S	>4/76	R	Negative
S	64	I	>64	R*	<=4	S	<=1	S	>4/76	R	Negative
S	>64	R	>64	R	<=4	S	<=1	S	>4/76	R	Positive
I	64	I	64	I	>8	R	N/R		>4/76	R	Positive
S	>64	R	>64	R	<=4	S	<=1	S	>4/76	R	Negative
S	>64	R	>64	R*	<=4	S	<=1	S	>4/76	R	Negative
S	64	I	64	I	>8	R	<=1	S	<=2/38	S	Negative
S	>64	R	>64	R*	<=4	S	<=1	S	>4/76	R	Negative
S	>64	R	>64	R	<=4	S	<=1	S	>4/76	R	Negative
S	64	I	>64	R	<=4	S	<=1	S	<=2/38	S	Positive
S	>64	R	>64	R*	8	I	<=1	S	<=2/38	S	Negative
S	>64	R	>64	R	<=4	S	<=1	S	>4/76	R	Negative
S	>64	R	>64	R*	<=4	S	<=1	S	>4/76	R	Negative
R	>64	R	>64	R	>8	R	<=1	S	>4/76	R	Positive
S	>64	R	>64	R	<=4	S	<=1	S	<=2/38	S	Negative

R	64	I	>64	R	>8	R	>2	R	>4/76	R	Positive

I	>64	R	>64	R	>8	R	<=1	S	>4/76	R	Positive
S	>64	R	>64	R	<=4	S	<=1	S	>4/76	R	Positive

R	>64	R	>64	R	>8	R	<=1	S	>4/76	R	Positive

R	>64	R	>64	R	>8	R	<=1	S	>4/76	R	Positive
I	>64	R	>64	R	8	I	<=1	S	>4/76	R	Positive
R	>64	R	>64	R	>8	R	<=1	S	>4/76	R	Negative
R	>64	R	>64	R	>8	R	>2	R	<=2/38	S	Positive
R	>64	R	>64	R	>8	R	>2	R	>4/76	R	Negative
S	>64	R	>64	R	<=4	S	<=1	S	<=2/38	S	Negative
S	>64	R	>64	R	>8	R	<=1	S	>4/76	R	Negative
S	64	I	>64	R*	<=4	S	<=1	S	>4/76	R	Negative
R	>64	R	>64	R	>8	R	<=1	S	>4/76	R	Positive
S	>64	R	>64	R	<=4	S	<=1	S	>4/76	R	Negative
S	>64	R	>64	R	>8	R	2	I	>4/76	R	Positive
R	64	I	>64	R	>8	R	>2	R	>4/76	R	Negative
S	>64	R	>64	R*	<=4	S	<=1	S	4/76	R	Negative
I	>64	R	>64	R	>8	R	2	I	>4/76	R	Negative
S	>64	R	>64	R*	<=4	S	<=1	S	>4/76	R	Negative
S	>64	R	>64	R	<=4	S	<=1	S	>4/76	R	Positive
S	<=8	S	<=8	S	<=4	S	<=1	S	<=2/38	S	Negative
S	>64	R	>64	R	8	I	<=1	S	>4/76	R	Positive

S	>64	R	>64	R*	<=4	S	<=1	S	>4/76	R	Negative
S	16	S	64	I	<=4	S	<=1	S	<=2/38	S	Negative
R	>64	R	>64	R	>8	R	<=1	S	>4/76	R	Positive
R	>64	R	>64	R	>8	R	<=1	S	>4/76	R	Positive
S	>64	R	>64	R	<=4	S	<=1	S	>4/76	R	Negative
S	>64	R	>64	R	<=4	S	<=1	S	>4/76	R	Positive
I	>64	R	>64	R	>8	R	<=1	S	>4/76	R	Negative
S	>64	R	>64	R	8	I	<=1	S	<=2/38	S	Negative
S	>64	R	>64	R	<=4	S	<=1	S	>4/76	R	Negative
I	>64	R	>64	R	>8	R	<=1	S	>4/76	R	Negative
R	>64	R	>64	R	>8	R	<=1	S	>4/76	R	Positive
S	>64	R	>64	R	<=4	S	<=1	S	<=2/38	S	Negative
S	>64	R	>64	R	<=4	S	<=1	S	>4/76	R	Negative
I	>64	R	>64	R	8	I	<=1	S	>4/76	R	Negative
S	>64	R	>64	R	<=4	S	<=1	S	>4/76	R	Positive
S	>64	R	>64	R	<=4	S	<=1	S	>4/76	R	Positive
I	>64	R	>64	R	>8	R	2	I	>4/76	R	Negative
S	>64	R	>64	R	<=4	S	<=1	S	>4/76	R	Positive
R	>64	R	>64	R	8	I	<=1	S	>4/76	R	Positive
I	>64	R	>64	R	>8	R	N/R		>4/76	R	Positive
R	64	I	64	I	>8	R	N/R		>4/76	R	Positive
S	>64	R	>64	R	<=4	S	<=1	S	>4/76	R	Positive
I	64	I	64	I	>8	R	N/R		>4/76	R	Positive
S	>64	R	>64	R	<=4	S	<=1	S	>4/76	R	Positive
S	>64	R	>64	R*	<=4	S	<=1	S	>4/76	R	Negative
S	64	I	>64	R*	>8	R	<=1	S	>4/76	R	Negative
I	>64	R	>64	R	>8	R	<=1	S	>4/76	R	Positive
S	>64	R	>64	R*	<=4	S	<=1	S	>4/76	R	Positive
R	>64	R	>64	R	>8	R	2	I	>4/76	R	Negative
I	>64	R	>64	R	>8	R	<=1	S	>4/76	R	Negative
I	>64	R	>64	R	>8	R	<=1	S	>4/76	R	Negative
I	>64	R	>64	R	>8	R	<=1	S	>4/76	R	Negative
S	>64	R	>64	R	>8	R	<=1	S	>4/76	R	Negative
I	>64	R	>64	R	>8	R	<=1	S	>4/76	R	Negative
S	>64	R	>64	R	8	I	<=1	S	>4/76	R	Positive
S	>64	R	>64	R*	8	I	<=1	S	>4/76	R	Negative
I	>64	R	>64	R	8	I	<=1	S	>4/76	R	Positive
S	>64	R	>64	R	<=4	S	<=1	S	>4/76	R	Positive
S	>64	R	>64	R	<=4	S	<=1	S	<=2/38	S	Positive
I	>64	R	>64	R	>8	R	<=1	S	>4/76	R	Negative
I	>64	R	>64	R*	>8	R	<=1	S	>4/76	R	Negative
R	>64	R	>64	R	>8	R	2	I	Apr-76	R	Negative
I	>64	R	>64	R	>8	R	2	I	>4/76	R	Negative
I	>64	R	>64	R	>8	R	<=1	S	>4/76	R	Negative
I	>64	R	>64	R	>8	R	<=1	S	>4/76	R	Positive
S	>64	R	>64	R	>8	R	<=1	S	>4/76	R	Negative
S	>64	R	>64	R	>8	R	<=1	S	>4/76	R	Negative
I	>64	R	>64	R	>8	R	N/R		>4/76	R	Negative
R	>64	R	>64	R	>8	R	<=1	S	>4/76	R	Positive
S	>64	R	>64	R	<=4	S	<=1	S	>4/76	R	Positive
S	>64	R	>64	R	<=4	S	<=1	S	<=2/38	S	Positive
S	>64	R	>64	R	<=4	S	<=1	S	>4/76	R	Positive
I	>64	R	>64	R	>8	R	<=1	S	>4/76	R	Positive
S	>64	R	>64	R*	<=4	S	<=1	S	>4/76	R	Negative
I	>64	R	>64	R*	>8	R	<=1	S	>4/76	R	Negative
S	16	S	64	I	8	I	2	I	>4/76	R	Negative

S	>64	R	>64	R	8	I	<=1	S	>4/76	R	Negative
S	>64	R	>64	R	<=4	S	<=1	S	<=2/38	S	Negative
S	>64	R	>64	R	<=4	S	<=1	S	>4/76	R	Negative
S	>64	R	>64	R*	<=4	S	<=1	S	>4/76	R	Negative
S	>64	R	>64	R	<=4	S	<=1	S	>4/76	R	Positive
S	64	I	>64	R*	8	I	<=1	S	Apr-76	R	Negative
S	>64	R	>64	R	<=4	S	<=1	S	>4/76	R	Negative
S	>64	R	>64	R	<=4	S	<=1	S	>4/76	R	Negative
S	>64	R	>64	R	<=4	S	<=1	S	>4/76	R	Negative
S	>64	R	>64	R	<=4	S	<=1	S	>4/76	R	Negative
I	>64	R	>64	R	>8	R	<=1	S	>4/76	R	Negative
S	64	I	64	I	>8	R	N/R		>4/76	R	Positive
S	>64	R	>64	R*	8	I	<=1	S	<=2/38	S	Negative
S	16	S	64	I	<=4	S	<=1	S	<=2/38	S	Negative
I	>64	R	>64	R	>8	R	N/R		>4/76	R	Positive
S	>64	R	>64	R	>8	R	<=1	S	>4/76	R	Positive
S	>64	R	>64	R	<=4	S	<=1	S	>4/76	R	Positive
S	>64	R	>64	R	<=4	S	<=1	S	>4/76	R	Negative
S	>64	R	>64	R*	<=4	S	<=1	S	>4/76	R	Negative
I	>64	R	>64	R*	>8	R	2	I	>4/76	R	Negative
I	>64	R	>64	R	>8	R	>2	R	>4/76	R	Negative
S	>64	R	>64	R*	<=4	S	<=1	S	>4/76	R	Negative
S	>64	R	>64	R*	<=4	S	<=1	S	>4/76	R	Negative
S	>64	R	>64	R	8	I	<=1	S	>4/76	R	Positive
S	>64	R	>64	R	<=4	S	<=1	S	>4/76	R	Positive
S	>64	R	>64	R	>8	R	2	I	>4/76	R	Negative
S	>64	R	>64	R*	<=4	S	<=1	S	>4/76	R	Negative
I	>64	R	>64	R	>8	R	N/R		>4/76	R	Positive
S	>64	R	>64	R	<=4	S	<=1	S	>4/76	R	Negative
S	>64	R	>64	R	>8	R	<=1	S	>4/76	R	Negative
I	>64	R	>64	R	>8	R	<=1	S	>4/76	R	Positive
S	>64	R	>64	R	>8	R	N/R		>4/76	R	Positive
S	>64	R	>64	R*	<=4	S	<=1	S	>4/76	R	Negative
I	>64	R	>64	R	>8	R	<=1	S	>4/76	R	Positive
S	>64	R	>64	R	>8	R	<=1	S	>4/76	R	Negative
R	>64	R	>64	R	>8	R	2	I	>4/76	R	Positive
I	>64	R	>64	R	>8	R	<=1	S	>4/76	R	Negative
S	>64	R	>64	R*	<=4	S	<=1	S	>4/76	R	Negative
S	>64	R	>64	R	>8	R	<=1	S	>4/76	R	Negative
S	>64	R	>64	R	>8	R	<=1	S	>4/76	R	Negative
R	>64	R	>64	R	>8	R	<=1	S	>4/76	R	Negative
S	>64	R	>64	R*	<=4	S	<=1	S	>4/76	R	Negative
S	>64	R	>64	R*	<=4	S	<=1	S	>4/76	R	Negative
R	>64	R	>64	R	>8	R	<=1	S	>4/76	R	Positive
S	>64	R	>64	R	<=4	S	<=1	S	>4/76	R	Positive
S	>64	R	>64	R	<=4	S	<=1	S	>4/76	R	Negative
S	>64	R	>64	R*	<=4	S	<=1	S	>4/76	R	Negative
S	>64	R	>64	R	<=4	S	<=1	S	>4/76	R	Negative
S	<=8	S	<=8	R*	<=4	S	<=1	S	<=2/38	S	Negative
S	>64	R	>64	R	<=4	S	<=1	S	>4/76	R	Positive
S	>64	R	>64	R	<=4	S	<=1	S	>4/76	R	Positive
I	>64	R	>64	R*	>8	R	<=1	S	>4/76	R	Negative
I	64	I	>64	R	>8	R	N/R		>4/76	R	Positive
S	64	I	>64	R	>8	R	<=1	S	>4/76	R	Positive
S	>64	R	>64	R*	<=4	S	<=1	S	>4/76	R	Negative

S	64	I	>64	R*	8	I	<=1	S	>4/76	R	Negative
S	64	I	>64	R*	<=4	S	<=1	S	>4/76	R	Negative
I	>64	R	>64	R	>8	R	<=1	S	>4/76	R	Negative
S	64	I	>64	R*	<=4	S	<=1	S	>4/76	R	Negative
S	>64	R	>64	R	<=4	S	<=1	S	>4/76	R	Positive
S	>64	R	>64	R	>8	R	<=1	S	>4/76	R	Positive
S	>64	R	>64	R	>8	R	<=1	S	>4/76	R	Positive
S	>64	R	>64	R	<=4	S	<=1	S	>4/76	R	Positive
S	>64	R	>64	R	<=4	S	<=1	S	>4/76	R	Positive
I	>64	R	>64	R	>8	R	2	I	>4/76	R	Negative
S	>64	R	>64	R*	<=4	S	<=1	S	<=2/38	S	Negative
S	>64	R	>64	R*	<=4	S	<=1	S	>4/76	R	Negative
S	>64	R	>64	R*	<=4	S	<=1	S	>4/76	R	Negative
R	>64	R	>64	R	>8	R	<=1	S	>4/76	R	Positive
I	>64	R	>64	R*	>8	R	<=1	S	>4/76	R	Negative
I	>64	R	>64	R*	>8	R	<=1	S	>4/76	R	Negative
I	>64	R	>64	R*	>8	R	<=1	S	>4/76	R	Negative
S	>64	R	>64	R*	<=4	S	<=1	S	>4/76	R	Negative
S	64	I	>64	R	>8	R	<=1	S	>4/76	R	Negative
S	>64	R	>64	R*	<=4	S	<=1	S	>4/76	R	Negative
R	64	I	>64	R	>8	R	>2	R	>4/76	R	Positive
R	>64	R	>64	R	>8	R	2	I	>4/76	R	Negative
S	>64	R	>64	R	<=4	S	<=1	S	>4/76	R	Positive
S	>64	R	>64	R*	<=4	S	<=1	S	>4/76	R	Negative
R	>64	R	>64	R*	>8	R	2	I	4/76	R	Negative
S	>64	R	>64	R*	8	I	<=1	S	>4/76	R	Negative
S	>64	R	>64	R	<=4	S	<=1	S	>4/76	R	Negative
S	16	S	>64	R*	>8	R	<=1	S	>4/76	R	Negative
S	>64	R	>64	R	<=4	S	<=1	S	>4/76	R	Positive
S	>64	R	>64	R*	<=4	S	<=1	S	>4/76	R	Negative
S	>64	R	>64	R*	<=4	S	<=1	S	>4/76	R	Negative
R	64	I	>64	R	>8	R	2	I	>4/76	R	Negative
S	>64	R	>64	R	>8	R	<=1	S	>4/76	R	Positive
S	>64	R	>64	R*	<=4	S	<=1	S	>4/76	R	Negative
S	>64	R	>64	R*	<=4	S	<=1	S	>4/76	R	Negative
I	>64	R	>64	R*	<=4	S	<=1	S	>4/76	R	Negative
S	>64	R	>64	R	<=4	S	<=1	S	>4/76	R	Positive
S	>64	R	>64	R	<=4	S	<=1	S	<=2/38	S	Positive
S	>64	R	>64	R	<=4	S	<=1	S	<=2/38	S	Positive
S	>64	R	>64	R*	<=4	S	<=1	S	<=2/38	S	Negative
I	>64	R	64	I	>8	R	N/R		>4/76	R	Positive
S	64	I	>64	R*	8	I	<=1	S	Apr-76	R	Negative
R	64	I	>64	R	>8	R	>2	R	>4/76	R	Positive
S	>64	R	>64	R	8	I	<=1	S	>4/76	R	Negative
I	>64	R	>64	R*	<=4	S	2	I	<=2/38	S	Negative
S	>64	R	>64	R	>8	R	<=1	S	>4/76	R	Negative
S	>64	R	>64	R	<=4	S	<=1	S	>4/76	R	Negative
S	>64	R	>64	R*	8	I	<=1	S	>4/76	R	Negative
S	<=8	S	<=8	S	<=4	S	<=1	S	<=2/38	S	Negative
S	>64	R	>64	R	<=4	S	<=1	S	>4/76	R	Negative
S	>64	R	>64	R	<=4	S	<=1	S	>4/76	R	Negative
S	>64	R	>64	R	<=4	S	<=1	S	>4/76	R	Negative
S	>64	R	>64	R	<=4	S	<=1	S	>4/76	R	Negative
R	>64	R	>64	R	>8	R	>2	R	>4/76	R	Negative
R	>64	R	>64	R	>8	R	2	I	>4/76	R	Positive

S	>64	R	>64	R	<=4	S	<=1	S	<=2/38	S	Positive
S	>64	R	>64	R	>8	R	<=1	S	>4/76	R	Positive
S	64	I	>64	R*	<=4	S	<=1	S	>4/76	R	Negative
S	>64	R	>64	R*	<=4	S	<=1	S	>4/76	R	Negative
R	>64	R	>64	R*	>8	R	2	I	>4/76	R	Negative
S	>64	R	>64	R	>8	R	2	I	>4/76	R	Positive
S	>64	R	>64	R*	<=4	S	<=1	S	>4/76	R	Negative
I	>64	R	>64	R*	>8	R	<=1	S	>4/76	R	Negative
S	>64	R	>64	R	<=4	S	<=1	S	>4/76	R	Negative
S	>64	R	>64	R	<=4	S	<=1	S	>4/76	R	Negative
S	>64	R	>64	R*	<=4	S	<=1	S	>4/76	R	Negative
R	>64	R	>64	R	>8	R	<=1	S	>4/76	R	Negative
S	>64	R	>64	R*	<=4	S	<=1	S	>4/76	R	Negative
R	>64	R	>64	R	>8	R	2	I	>4/76	R	Negative
S	64	I	>64	R*	<=4	S	<=1	S	>4/76	R	Negative
S	>64	R	>64	R	<=4	S	<=1	S	<=2/38	S	Positive
S	>64	R	>64	R	>8	R	<=1	S	>4/76	R	Negative
S	>64	R	>64	R*	8	I	2	I	>4/76	R	Negative
S	<=8	S	64	I	<=4	S	<=1	S	<=2/38	S	Negative
S	>64	R	>64	R*	<=4	S	<=1	S	>4/76	R	Negative
I	>64	R	>64	R	>8	R	2	I	>4/76	R	Positive
S	>64	R	>64	R	<=4	S	<=1	S	>4/76	R	Negative
S	64	I	64	I	>8	R	N/R		>4/76	R	Positive
S	64	I	>64	R*	<=4	S	<=1	S	>4/76	R	Negative
S	>64	R	>64	R*	<=4	S	<=1	S	>4/76	R	Negative
R	>64	R	>64	R	>8	R	<=1	S	>4/76	R	Positive
S	64	I	>64	R	>8	R	<=1	S	>4/76	R	Negative
S	>64	R	>64	R*	>8	R	<=1	S	>4/76	R	Negative
S	>64	R	>64	R*	<=4	S	<=1	S	<=2/38	S	Negative
S	>64	R	>64	R	<=4	S	<=1	S	>4/76	R	Negative
S	>64	R	>64	R*	<=4	S	<=1	S	>4/76	R	Negative
R	>64	R	>64	R	>8	R	2	I	>4/76	R	Positive
S	>64	R	>64	R	>8	R	<=1	S	>4/76	R	Positive
S	>64	R	>64	R	<=4	S	<=1	S	>4/76	R	Negative
S	>64	R	>64	R*	>8	R	2	I	>4/76	R	Negative
S	64	I	>64	R*	<=4	S	<=1	S	>4/76	R	Negative
I	>64	R	>64	R*	>8	R	<=1	S	>4/76	R	Negative
S	>64	R	>64	R	>8	R	<=1	S	>4/76	R	Negative
S	>64	R	>64	R	<=4	S	<=1	S	>4/76	R	Negative
R	>64	R	>64	R	>8	R	2	I	>4/76	R	Positive
S	>64	R	>64	R	>8	R	<=1	S	>4/76	R	Negative
S	>64	R	>64	R	>8	R	<=1	S	>4/76	R	Positive
S	>64	R	>64	R	>8	R	<=1	S	>4/76	R	Negative
R	64	I	>64	R*	>8	R	>2	R	>4/76	R	Negative
S	>64	R	>64	R*	<=4	S	<=1	S	>4/76	R	Negative
S	>64	R	>64	R	<=4	S	<=1	S	>4/76	R	Negative
R	>64	R	>64	R	>8	R	<=1	S	>4/76	R	Negative
S	>64	R	>64	R	>8	R	N/R		>4/76	R	Positive
I	>64	R	>64	R	>8	R	<=1	S	>4/76	R	Negative
I	>64	R	>64	R*	>8	R	<=1	S	>4/76	R	Negative
S	>64	R	>64	R	>8	R	<=1	S	>4/76	R	Negative
S	>64	R	>64	R	<=4	S	<=1	S	>4/76	R	Positive
I	>64	R	>64	R	>8	R	<=1	S	>4/76	R	Negative
S	>64	R	>64	R	<=4	S	2	I	>4/76	R	Negative
S	64	I	>64	R*	>8	R	<=1	S	<=2/38	S	Negative
S	>64	R	>64	R	>8	R	N/R		>4/76	R	Positive

R	>64	R	>64	R	>8	R	<=1	S	>4/76	R	Negative
S	>64	R	>64	R*	<=4	S	<=1	S	>4/76	R	Negative
S	>64	R	>64	R*	<=4	S	<=1	S	>4/76	R	Negative
S	>64	R	>64	R*	<=4	S	<=1	S	>4/76	R	Negative
S	>64	R	>64	R	<=4	S	<=1	S	<=2/38	S	Positive
S	>64	R	>64	R*	<=4	S	<=1	S	>4/76	R	Negative
R	>64	R	>64	R	>8	R	<=1	S	>4/76	R	Positive
S	>64	R	>64	R*	<=4	S	<=1	S	4/76	R	Negative
S	16	S	64	I	<=4	S	<=1	S	>4/76	R	Negative
R	>64	R	>64	R	>8	R	<=1	S	>4/76	R	Negative
S	>64	R	>64	R	<=4	S	<=1	S	>4/76	R	Negative
R	>64	R	>64	R	>8	R	<=1	S	>4/76	R	Positive
S	64	I	>64	R*	<=4	S	<=1	S	>4/76	R	Negative
S	>64	R	>64	R	>8	R	N/R		>4/76	R	Positive
S	>64	R	>64	R	<=4	S	<=1	S	>4/76	R	Negative
R	>64	R	>64	R	>8	R	<=1	S	>4/76	R	Positive
S	>64	R	>64	R*	<=4	S	<=1	S	>4/76	R	Negative
S	>64	R	>64	R*	<=4	S	<=1	S	>4/76	R	Negative
S	>64	R	>64	R*	<=4	S	<=1	S	>4/76	R	Negative
S	>64	R	>64	R	<=4	S	<=1	S	>4/76	R	Negative
S	64	I	>64	R*	<=4	S	<=1	S	>4/76	R	Negative
S	>64	R	>64	R	>8	R	<=1	S	>4/76	R	Positive
S	>64	R	>64	R*	<=4	S	<=1	S	>4/76	R	Negative
S	>64	R	>64	R	<=4	S	<=1	S	>4/76	R	Negative
S	>64	R	>64	R	<=4	S	<=1	S	>4/76	R	Negative
I	>64	R	>64	R*	8	I	2	I	>4/76	R	Negative
S	>64	R	>64	R	>8	R	<=1	S	>4/76	R	Positive
S	>64	R	>64	R	8	I	<=1	S	>4/76	R	Negative
I	>64	R	>64	R	8	I	<=1	S	>4/76	R	Negative
S	>64	R	>64	R	<=4	S	<=1	S	>4/76	R	Negative
I	>64	R	>64	R	>8	R	2	I	>4/76	R	Negative
R	>64	R	>64	R	>8	R	>2	R	>4/76	R	Positive
I	>64	R	>64	R	>8	R	<=1	S	>4/76	R	Positive
S	>64	R	>64	R	<=4	S	<=1	S	>4/76	R	Negative
R	>64	R	>64	R	>8	R	<=1	S	>4/76	R	Negative
S	>64	R	>64	R	<=4	S	<=1	S	>4/76	R	Positive
S	>64	R	>64	R	<=4	S	<=1	S	>4/76	R	Negative
S	>64	R	>64	R	8	I	2	I	>4/76	R	Positive
I	>64	R	>64	R	>8	R	<=1	S	>4/76	R	Negative
S	>64	R	>64	R	<=4	S	<=1	S	>4/76	R	Negative
R	>64	R	>64	R	>8	R	<=1	S	>4/76	R	Negative
S	>64	R	>64	R*	<=4	S	<=1	S	>4/76	R	Negative

S	>64	R	>64	R*	<=4	S	<=1	S	>4/76	R	Negative

S	64	I	>64	R	<=4	S	<=1	S	>4/76	R	Positive
S	>64	R	>64	R	>8	R	<=1	S	>4/76	R	Negative
S	64	I	>64	R*	<=4	S	<=1	S	>4/76	R	Negative
R	>64	R	>64	R*	>8	R	<=1	S	>4/76	R	Negative
S	>64	R	>64	R*	<=4	S	<=1	S	>4/76	R	Negative
I	>64	R	>64	R	>8	R	<=1	S	>4/76	R	Positive
R	>64	R	>64	R	>8	R	>2	R	>4/76	R	Negative
I	>64	R	>64	R	>8	R	<=1	S	>4/76	R	Negative
S	>64	R	>64	R	8	I	<=1	S	<=2/38	S	Positive
S	>64	R	>64	R*	>8	R	<=1	S	>4/76	R	Negative
R	>64	R	>64	R	>8	R	<=1	S	>4/76	R	Positive
S	>64	R	>64	R	<=4	S	<=1	S	>4/76	R	Negative
S	>64	R	>64	R*	>8	R	<=1	S	>4/76	R	Negative
S	64	I	>64	R*	>8	R	<=1	S	>4/76	R	Negative
S	>64	R	>64	R*	8	I	<=1	S	>4/76	R	Negative
S	64	I	>64	R*	<=4	S	<=1	S	>4/76	R	Negative
S	>64	R	>64	R	8	I	<=1	S	>4/76	R	Negative
S	>64	R	>64	R*	<=4	S	<=1	S	>4/76	R	Negative
S	>64	R	>64	R*	<=4	S	<=1	S	>4/76	R	Negative
S	64	I	>64	R	>8	R	<=1	S	4/76	R	Positive
S	>64	R	>64	R*	<=4	S	<=1	S	>4/76	R	Negative
S	>64	R	>64	R	<=4	S	>2	R	>4/76	R	Negative
I	>64	R	>64	R	>8	R	<=1	S	>4/76	R	Positive

S	>64	R	>64	R	<=4	S	<=1	S	>4/76	R	Positive
S	>64	R	>64	R	<=4	S	<=1	S	>4/76	R	Positive
I	>64	R	>64	R	>8	R	<=1	S	>4/76	R	Positive
R	>64	R	>64	R	>8	R	2	I	>4/76	R	Negative
S	>64	R	>64	R	<=4	S	<=1	S	>4/76	R	Negative
R	>64	R	>64	R	>8	R	<=1	S	>4/76	R	Negative
S	>64	R	>64	R	>8	R	>2	R	>4/76	R	Negative
S	>64	R	>64	R	>8	R	<=1	S	>4/76	R	Negative
I	>64	R	>64	R	>8	R	<=1	S	>4/76	R	Negative
I	>64	R	>64	R	>8	R	<=1	S	>4/76	R	Negative
S	>64	R	>64	R	>8	R	<=1	S	>4/76	R	Negative
S	>64	R	>64	R	>8	R	<=1	S	>4/76	R	Negative
R	>64	R	>64	R	>8	R	2	I	>4/76	R	Positive
S	>64	R	>64	R	>8	R	<=1	S	>4/76	R	Negative
S	>64	R	>64	R	>8	R	<=1	S	>4/76	R	Negative
I	>64	R	>64	R	>8	R	<=1	S	>4/76	R	Positive
S	>64	R	>64	R	>8	R	<=1	S	>4/76	R	Positive
I	>64	R	>64	R*	>8	R	<=1	S	>4/76	R	Negative
S	>64	R	>64	R*	>8	R	<=1	S	>4/76	R	Negative
R	>64	R	>64	R	>8	R	>2	R	>4/76	R	Negative
R	>64	R	>64	R	>8	R	<=1	S	>4/76	R	Positive
S	64	I	>64	R*	>8	R	<=1	S	>4/76	R	Negative
S	>64	R	>64	R*	<=4	S	<=1	S	>4/76	R	Negative
S	>64	R	>64	R*	<=4	S	<=1	S	>4/76	R	Negative
S	>64	R	>64	R*	<=4	S	<=1	S	>4/76	R	Negative
R	>64	R	>64	R	>8	R	<=1	S	>4/76	R	Positive
I	>64	R	>64	R	>8	R	<=1	S	>4/76	R	Negative
S	>64	R	>64	R*	<=4	S	<=1	S	>4/76	R	Negative
R	>64	R	>64	R	>8	R	2	I	>4/76	R	Negative
S	>64	R	>64	R	>8	R	<=1	S	>4/76	R	Negative
R	>64	R	>64	R	>8	R	>2	R	>4/76	R	Negative
R	64	I	>64	R*	>8	R	2	I	>4/76	R	Negative
S	>64	R	>64	R	>8	R	<=1	S	>4/76	R	Negative
R	>64	R	>64	R*	>8	R	2	I	>4/76	R	Negative
R	>64	R	>64	R	>8	R	<=1	S	4/76	R	Negative
S	>64	R	>64	R	<=4	S	<=1	S	>4/76	R	Negative
R	>64	R	>64	R	>8	R	2	I	>4/76	R	Negative
S	>64	R	>64	R	<=4	S	<=1	S	>4/76	R	Positive
R	>64	R	>64	R	>8	R	<=1	S	>4/76	R	Negative
R	>64	R	>64	R*	8	I	<=1	S	>4/76	R	Negative
R	>64	R	>64	R	>8	R	<=1	S	>4/76	R	Positive
R	>64	R	>64	R*	>8	R	2	I	>4/76	R	Negative
S	>64	R	>64	R	<=4	S	<=1	S	>4/76	R	Negative
S	64	I	>64	R*	>8	R	<=1	S	>4/76	R	Negative
S	>64	R	>64	R*	<=4	S	<=1	S	>4/76	R	Negative
S	>64	R	>64	R*	>8	R	<=1	S	>4/76	R	Negative
S	>64	R	>64	R	<=4	S	<=1	S	>4/76	R	Negative
S	64	I	>64	R*	>8	R	<=1	S	>4/76	R	Negative
S	>64	R	>64	R	>8	R	<=1	S	>4/76	R	Positive
S	>64	R	>64	R	8	I	<=1	S	>4/76	R	Negative
I	>64	R	>64	R	>8	R	2	I	>4/76	R	Negative
S	>64	R	>64	R	<=4	S	<=1	S	>4/76	R	Positive
S	>64	R	>64	R	<=4	S	<=1	S	>4/76	R	Negative
I	64	I	>64	R	>8	R	N/R		>4/76	R	Positive
S	>64	R	>64	R*	<=4	S	<=1	S	>4/76	R	Negative
I	64	I	>64	R*	>8	R	<=1	S	>4/76	R	Negative

R	16	S	>64	R	>8	R	2	I	>4/76	R	Negative
S	64	I	64	R*	<=4	S	2	I	<=2/38	S	Negative
I	>64	R	64	I	>8	R	N/R		>4/76	R	Positive
S	>64	R	>64	R*	<=4	S	<=1	S	>4/76	R	Negative
S	64	I	>64	R*	<=4	S	<=1	S	>4/76	R	Negative
S	>64	R	>64	R	8	I	<=1	S	>4/76	R	Positive
S	>64	R	>64	R	>8	R	<=1	S	>4/76	R	Negative
S	>64	R	>64	R	8	I	<=1	S	>4/76	R	Negative
S	>64	R	>64	R	>8	R	<=1	S	>4/76	R	Positive
I	>64	R	>64	R	>8	R	<=1	S	>4/76	R	Negative
S	>64	R	>64	R*	8	I	2	I	>4/76	R	Negative
S	>64	R	>64	R	>8	R	<=1	S	>4/76	R	Negative
S	>64	R	>64	R*	<=4	S	<=1	S	>4/76	R	Negative
S	64	I	>64	R	8	I	<=1	S	>4/76	R	Negative
S	16	S	64	I	>8	R	<=1	S	<=2/38	S	Negative
S	64	I	64	R*	>8	R	<=1	S	4/76	R	Negative
S	>64	R	>64	R*	8	I	2	I	>4/76	R	Negative
S	<=8	S	>64	R*	<=4	S	<=1	S	>4/76	R	Negative
S	64	I	>64	R	<=4	S	<=1	S	>4/76	R	Positive
S	64	I	>64	R*	<=4	S	<=1	S	<=2/38	S	Negative
R	16	S	>64	R	>8	R	>2	R	>4/76	R	Negative
S	>64	R	>64	R	<=4	S	<=1	S	4/76	R	Positive
S	>64	R	>64	R	<=4	S	<=1	S	<=2/38	S	Positive
S	>64	R	>64	R*	<=4	S	<=1	S	>4/76	R	Negative
S	>64	R	>64	R	<=4	S	<=1	S	>4/76	R	Negative
S	>64	R	>64	R	<=4	S	<=1	S	<=2/38	S	Negative
S	>64	R	>64	R	8	I	<=1	S	<=2/38	S	Positive
S	>64	R	>64	R*	<=4	S	<=1	S	>4/76	R	Negative
S	>64	R	>64	R*	>8	R	<=1	S	>4/76	R	Negative
R	>64	R	>64	R	>8	R	<=1	S	>4/76	R	Positive
S	64	I	>64	R	>8	R	<=1	S	<=2/38	S	Negative
S	>64	R	>64	R*	<=4	S	<=1	S	>4/76	R	Negative
I	>64	R	>64	R	>8	R	<=1	S	>4/76	R	Negative
S	>64	R	>64	R*	<=4	S	<=1	S	>4/76	R	Negative
S	>64	R	>64	R*	<=4	S	<=1	S	>4/76	R	Negative
S	>64	R	>64	R*	<=4	S	<=1	S	>4/76	R	Negative
S	>64	R	>64	R*	<=4	S	<=1	S	>4/76	R	Negative
S	>64	R	>64	R	<=4	S	<=1	S	>4/76	R	Negative
S	>64	R	>64	R	<=4	S	<=1	S	>4/76	R	Negative
R	>64	R	>64	R	>8	R	<=1	S	>4/76	R	Positive
R	>64	R	>64	R	>8	R	<=1	S	>4/76	R	Positive
S	>64	R	>64	R*	<=4	S	<=1	S	>4/76	R	Negative
S	>64	R	>64	R*	<=4	S	<=1	S	>4/76	R	Negative
S	>64	R	>64	R	<=4	S	<=1	S	>4/76	R	Positive
S	>64	R	>64	R	<=4	S	<=1	S	>4/76	R	Positive
S	>64	R	>64	R	<=4	S	<=1	S	4/76	R	Negative
I	>64	R	>64	R	>8	R	<=1	S	>4/76	R	Negative
S	64	I	>64	R*	>8	R	<=1	S	>4/76	R	Negative
S	>64	R	>64	R	<=4	S	<=1	S	>4/76	R	Negative
S	>64	R	>64	R	<=4	S	<=1	S	<=2/38	S	Positive
S	>64	R	>64	R	>8	R	<=1	S	>4/76	R	Negative
S	>64	R	>64	R	>8	R	<=1	S	>4/76	R	Positive
R	>64	R	>64	R*	>8	R	2	I	>4/76	R	Negative
S	>64	R	>64	R	<=4	S	<=1	S	>4/76	R	Negative
S	>64	R	>64	R	>8	R	<=1	S	>4/76	R	Negative
S	>64	R	>64	R	>8	R	<=1	S	>4/76	R	Negative

S	>64	R	>64	R	<=4	S	<=1	S	>4/76	R	Negative
S	>64	R	>64	R	>8	R	<=1	S	>4/76	R	Negative
S	>64	R	>64	R*	<=4	S	<=1	S	>4/76	R	Negative
S	64	I	>64	R	8	I	<=1	S	>4/76	R	Negative
S	>64	R	>64	R*	<=4	S	<=1	S	>4/76	R	Negative
I	>64	R	>64	R	>8	R	<=1	S	>4/76	R	Negative
S	>64	R	>64	R	>8	R	<=1	S	>4/76	R	Negative
S	64	I	>64	R*	>8	R	<=1	S	>4/76	R	Negative
S	>64	R	>64	R	>8	R	<=1	S	>4/76	R	Positive
S	>64	R	>64	R*	<=4	S	<=1	S	>4/76	R	Negative
S	>64	R	>64	R	<=4	S	<=1	S	>4/76	R	Negative
S	>64	R	>64	R	<=4	S	<=1	S	>4/76	R	Negative
S	>64	R	>64	R	>8	R	<=1	S	>4/76	R	Negative
S	16	S	>64	R	>8	R	<=1	S	>4/76	R	Negative
S	>64	R	64	I	>8	R	<=1	S	>4/76	R	Negative
S	>64	R	>64	R	>8	R	<=1	S	>4/76	R	Negative
S	>64	R	>64	R*	<=4	S	<=1	S	>4/76	R	Negative
R	64	I	>64	R*	>8	R	<=1	S	>4/76	R	Negative
S	>64	R	>64	R*	<=4	S	<=1	S	>4/76	R	Negative
S	>64	R	>64	R	<=4	S	<=1	S	>4/76	R	Positive
R	>64	R	>64	R*	>8	R	<=1	S	>4/76	R	Negative
S	>64	R	>64	R	<=4	S	<=1	S	>4/76	R	Negative
S	>64	R	>64	R*	<=4	S	<=1	S	>4/76	R	Negative
S	>64	R	>64	R	>8	R	<=1	S	4/76	R	Positive
S	>64	R	>64	R	<=4	S	<=1	S	>4/76	R	Negative
S	>64	R	>64	R	<=4	S	<=1	S	>4/76	R	Negative
S	>64	R	>64	R	>8	R	<=1	S	<=2/38	S	Positive
R	>64	R	>64	R	>8	R	<=1	S	>4/76	R	Negative

kpc	oxa48variants	vim	ges
Negative	Negative	Negative	Negative
Negative	Positive	Negative	Negative
Negative	Negative	Negative	Negative
Negative	Negative	Negative	Negative
Negative	Negative	Positive	Negative
Negative	Negative	Negative	Negative
Negative	Negative	Positive	Negative
Negative	Negative	Positive	Negative
Negative	Negative	Negative	Negative
Negative	Negative	Negative	Negative
Negative	Negative	Negative	Negative
Negative	Negative	Negative	Negative
Negative	Negative	Negative	Negative
Negative	Negative	Negative	Negative
Negative	Positive	Negative	Negative
Negative	Positive	Negative	Negative
Negative	Negative	Negative	Negative
Negative	Negative	Negative	Negative
Negative	Negative	Negative	Negative
Negative	Negative	Negative	Negative
Negative	Negative	Negative	Negative
Negative	Negative	Negative	Negative
Negative	Negative	Negative	Negative
Negative	Negative	Negative	Negative
Negative	Negative	Negative	Negative
Negative	Negative	Negative	Negative
Negative	Positive	Negative	Negative
Negative	Negative	Negative	Negative
Negative	Negative	Negative	Negative
Negative	Negative	Positive	Negative
Negative	Negative	Negative	Negative
Negative	Positive	Positive	Negative
Negative	Negative	Negative	Negative
Negative	Negative	Negative	Negative
Negative	Negative	Negative	Negative
Negative	Negative	Negative	Negative
Negative	Negative	Negative	Negative
Negative	Negative	Negative	Negative
Negative	Negative	Negative	Negative
Negative	Negative	Negative	Negative
Negative	Negative	Negative	Negative
Negative	Negative	Negative	Negative
Negative	Negative	Positive	Negative
Negative	Negative	Positive	Negative
Negative	Negative	Positive	Negative
Negative	Negative	Negative	Negative
Negative	Positive	Negative	Negative
Negative	Negative	Negative	Negative
Negative	Negative	Negative	Negative
Negative	Negative	Negative	Negative
Negative	Positive	Negative	Negative

Negative	Positive	Negative	Negative
Negative	Negative	Negative	Positive
Negative	Positive	Negative	Negative
Negative	Positive	Negative	Negative
Negative	Positive	Negative	Negative
Negative	Positive	Negative	Negative
Negative	Positive	Negative	Negative
Negative	Negative	Negative	Negative
Negative	Negative	Negative	Negative
Negative	Negative	Negative	Negative
Negative	Positive	Negative	Negative
Negative	Positive	Negative	Negative
Negative	Positive	Negative	Negative
Negative	Negative	Negative	Negative
Negative	Positive	Negative	Negative
Negative	Positive	Negative	Negative
Negative	Positive	Negative	Negative
Negative	Negative	Negative	Negative
Negative	Positive	Negative	Negative
Negative	Negative	Negative	Negative
Negative	Positive	Negative	Negative
Negative	Negative	Negative	Negative
Negative	Negative	Negative	Negative
Negative	Positive	Negative	Negative
Negative	Negative	Negative	Negative
Negative	Negative	Negative	Negative
Negative	Negative	Negative	Negative
Negative	Positive	Negative	Negative
Negative	Negative	Negative	Negative
Negative	Negative	Negative	Negative
Negative	Negative	Negative	Negative
Negative	Negative	Negative	Negative

Negative	Negative	Positive	Negative
Negative	Positive	Negative	Negative

Negative	Positive	Negative	Negative
Negative	Negative	Positive	Negative
Negative	Negative	Negative	Negative
Negative	Negative	Negative	Negative
Negative	Negative	Negative	Negative
Negative	Negative	Negative	Negative
Negative	Negative	Negative	Negative
Negative	Positive	Negative	Negative
Negative	Positive	Negative	Negative
Negative	Negative	Positive	Negative
Negative	Positive	Negative	Negative
Negative	Negative	Negative	Negative
Negative	Negative	Positive	Negative
Negative	Positive	Negative	Negative
Negative	Negative	Positive	Negative
Negative	Negative	Positive	Negative
Negative	Negative	Negative	Negative
Negative	Negative	Negative	Negative
Negative	Negative	Negative	Negative
Negative	Negative	Negative	Negative
Negative	Negative	Negative	Negative
Negative	Negative	Negative	Negative
Negative	Positive	Negative	Negative
Negative	Negative	Negative	Negative
Negative	Negative	Negative	Negative
Negative	Negative	Negative	Negative
Negative	Negative	Negative	Negative
Negative	Negative	Negative	Negative
Negative	Positive	Negative	Negative
Negative	Positive	Negative	Negative
Negative	Positive	Negative	Negative
Negative	Negative	Negative	Negative
Negative	Negative	Negative	Negative
Negative	Positive	Negative	Negative
Negative	Positive	Negative	Negative
Negative	Positive	Negative	Negative
Negative	Negative	Negative	Negative
Negative	Negative	Negative	Negative
Negative	Negative	Negative	Negative
Negative	Negative	Negative	Negative
Negative	Negative	Negative	Negative
Negative	Negative	Negative	Negative
Negative	Positive	Negative	Negative
Negative	Positive	Negative	Negative
Negative	Negative	Negative	Negative
Negative	Positive	Negative	Negative
Negative	Positive	Negative	Negative
Negative	Negative	Negative	Negative
Negative	Positive	Negative	Negative
Negative	Negative	Positive	Negative

Negative	Positive	Negative	Negative
Negative	Negative	Negative	Negative
Negative	Negative	Negative	Negative
Negative	Negative	Negative	Negative
Negative	Negative	Negative	Negative
Negative	Negative	Positive	Negative
Negative	Negative	Negative	Negative
Negative	Negative	Negative	Negative
Negative	Positive	Negative	Negative
Negative	Negative	Negative	Negative
Negative	Negative	Negative	Negative
Negative	Negative	Negative	Negative
Negative	Negative	Negative	Negative
Negative	Positive	Negative	Negative
Negative	Positive	Negative	Negative
Negative	Positive	Negative	Negative
Negative	Negative	Negative	Negative
Negative	Negative	Negative	Negative
Negative	Negative	Negative	Negative
Negative	Negative	Negative	Negative
Negative	Negative	Negative	Negative
Negative	Negative	Negative	Negative
Negative	Negative	Negative	Negative
Negative	Positive	Negative	Negative
Negative	Negative	Negative	Negative
Negative	Positive	Negative	Negative
Negative	Negative	Negative	Negative
Negative	Negative	Negative	Negative
Negative	Negative	Negative	Negative
Negative	Negative	Negative	Negative
Negative	Negative	Negative	Negative
Negative	Negative	Negative	Negative
Negative	Negative	Negative	Negative
Negative	Negative	Negative	Negative

Negative	Negative	Negative	Negative
Negative	Negative	Positive	Negative
Negative	Positive	Negative	Negative
Negative	Positive	Negative	Negative
Negative	Positive	Negative	Negative
Negative	Positive	Negative	Negative
Negative	Positive	Negative	Negative
Negative	Positive	Negative	Negative
Negative	Negative	Negative	Negative
Negative	Positive	Negative	Negative
Negative	Negative	Negative	Negative
Negative	Negative	Negative	Negative
Negative	Positive	Negative	Negative
Negative	Negative	Negative	Negative

Negative	Negative	Negative	Negative
Negative	Negative	Negative	Negative
Negative	Negative	Negative	Negative
Negative	Negative	Negative	Negative
Negative	Positive	Negative	Negative
Negative	Negative	Negative	Negative
Negative	Positive	Negative	Negative
Negative	Positive	Negative	Negative
Negative	Positive	Negative	Negative
Negative	Negative	Negative	Negative
Negative	Negative	Negative	Negative
Negative	Negative	Negative	Negative
Negative	Negative	Negative	Negative
Negative	Negative	Negative	Negative
Negative	Positive	Negative	Negative
Negative	Positive	Negative	Negative
Negative	Positive	Negative	Negative
Negative	Positive	Negative	Negative
Negative	Negative	Negative	Positive
Negative	Negative	Negative	Negative
Negative	Positive	Negative	Negative
Negative	Negative	Negative	Negative
Negative	Positive	Negative	Negative
Negative	Positive	Negative	Negative
Negative	Positive	Negative	Negative
Negative	Negative	Negative	Negative
Negative	Negative	Positive	Negative
Negative	Positive	Negative	Negative
Negative	Negative	Negative	Negative
Negative	Negative	Negative	Negative
Negative	Positive	Negative	Negative
Negative	Negative	Negative	Negative
Negative	Negative	Negative	Negative
Negative	Negative	Negative	Negative
Negative	Negative	Negative	Negative
Negative	Negative	Negative	Negative
Negative	Negative	Negative	Negative
Negative	Negative	Negative	Negative
Negative	Positive	Negative	Negative
Negative	Positive	Negative	Negative
Negative	Negative	Negative	Negative
Negative	Positive	Negative	Negative
Negative	Positive	Negative	Negative
Negative	Positive	Negative	Negative
Negative	Negative	Negative	Negative
Negative	Positive	Negative	Negative

Negative	Positive	Negative	Negative
Negative	Positive	Negative	Negative
Negative	Positive	Negative	Negative
Negative	Negative	Negative	Negative
Negative	Positive	Negative	Negative
Negative	Negative	Negative	Negative
Negative	Positive	Negative	Negative
Negative	Negative	Negative	Negative
Negative	Negative	Negative	Negative
Negative	Positive	Negative	Negative
Negative	Negative	Negative	Negative
Negative	Negative	Positive	Negative
Negative	Negative	Negative	Negative
Negative	Positive	Negative	Negative
Negative	Positive	Negative	Negative
Negative	Positive	Negative	Negative
Negative	Positive	Negative	Negative
Negative	Positive	Negative	Negative
Negative	Positive	Negative	Negative
Negative	Negative	Negative	Negative
Negative	Negative	Negative	Negative
Negative	Positive	Negative	Negative
Negative	Positive	Negative	Negative
Negative	Positive	Negative	Negative
Negative	Positive	Negative	Negative
Negative	Positive	Negative	Negative
Negative	Positive	Negative	Negative
Negative	Negative	Negative	Negative
Negative	Negative	Negative	Negative
Negative	Positive	Negative	Negative
Negative	Positive	Negative	Negative
Negative	Negative	Negative	Negative
Negative	Negative	Negative	Negative
Negative	Negative	Negative	Negative
Negative	Negative	Negative	Negative
Negative	Positive	Negative	Negative
Negative	Negative	Negative	Negative
Negative	Negative	Negative	Negative
Negative	Negative	Negative	Negative
Negative	Negative	Negative	Negative
Negative	Negative	Negative	Negative
Negative	Positive	Negative	Negative
Negative	Positive	Negative	Negative
Negative	Negative	Negative	Negative

Negative	Positive	Negative	Negative
Negative	Positive	Negative	Negative
Negative	Positive	Negative	Negative
Negative	Negative	Negative	Negative
Negative	Positive	Negative	Negative
Negative	Positive	Negative	Negative
Negative	Negative	Negative	Negative
Negative	Positive	Negative	Negative
Negative	Positive	Negative	Negative
Negative	Positive	Negative	Negative
Negative	Positive	Negative	Negative
Negative	Negative	Negative	Negative
Negative	Positive	Negative	Negative
Negative	Positive	Negative	Negative
Negative	Positive	Negative	Negative
Negative	Negative	Negative	Negative
Negative	Positive	Negative	Negative
Negative	Negative	Negative	Negative
Negative	Positive	Negative	Negative
Negative	Positive	Negative	Negative
Negative	Negative	Negative	Negative

Negative	Negative	Positive	Negative
Negative	Negative	Negative	Negative
Negative	Negative	Negative	Negative
Negative	Negative	Negative	Negative
Negative	Negative	Negative	Negative
Negative	Negative	Negative	Negative
Negative	Negative	Negative	Negative
Negative	Positive	Negative	Negative

Negative	Negative	Negative	Negative
Negative	Negative	Positive	Negative
Negative	Positive	Negative	Negative
Negative	Positive	Negative	Negative
Negative	Negative	Negative	Negative
Negative	Positive	Negative	Negative
Negative	Positive	Negative	Negative
Negative	Positive	Negative	Negative
Negative	Positive	Negative	Negative
Negative	Positive	Negative	Negative
Negative	Negative	Negative	Negative
Negative	Positive	Negative	Negative
Negative	Negative	Negative	Negative
Negative	Positive	Negative	Negative
Negative	Positive	Negative	Negative
Negative	Positive	Negative	Negative
Negative	Positive	Negative	Negative
Negative	Negative	Negative	Negative
Negative	Negative	Negative	Negative
Negative	Positive	Negative	Negative
Negative	Positive	Negative	Negative
Negative	Negative	Negative	Negative
Negative	Negative	Negative	Negative
Negative	Negative	Negative	Negative
Negative	Positive	Negative	Negative
Negative	Negative	Negative	Negative
Negative	Negative	Negative	Negative
Negative	Negative	Negative	Negative
Negative	Negative	Negative	Negative
Negative	Negative	Negative	Negative
Negative	Positive	Negative	Negative
Negative	Positive	Negative	Negative
Negative	Positive	Negative	Negative
Negative	Positive	Negative	Negative
Negative	Positive	Negative	Negative
Negative	Negative	Negative	Negative
Negative	Negative	Negative	Negative
Negative	Positive	Negative	Negative
Negative	Positive	Negative	Negative
Negative	Negative	Negative	Negative
Negative	Positive	Negative	Negative
Negative	Positive	Negative	Negative
Negative	Negative	Negative	Negative
Negative	Positive	Negative	Negative
Negative	Positive	Positive	Negative
Negative	Negative	Negative	Negative
Negative	Negative	Negative	Negative

Negative	Negative	Negative	Negative
Negative	Positive	Negative	Negative
Negative	Positive	Negative	Negative
Negative	Positive	Negative	Negative
Negative	Positive	Positive	Negative
Negative	Positive	Negative	Negative
Negative	Positive	Negative	Negative
Negative	Negative	Negative	Negative
Negative	Negative	Negative	Negative
Negative	Negative	Negative	Negative
Negative	Positive	Negative	Negative
Negative	Positive	Negative	Negative
Negative	Positive	Negative	Negative
Negative	Negative	Negative	Negative
Negative	Negative	Negative	Negative
Negative	Positive	Negative	Negative
Negative	Negative	Negative	Negative
Negative	Positive	Negative	Negative
Negative	Negative	Negative	Negative
Negative	Negative	Negative	Negative
Negative	Negative	Negative	Negative
Negative	Positive	Negative	Negative
Negative	Negative	Negative	Negative
Negative	Positive	Negative	Negative
Negative	Positive	Negative	Negative
Negative	Positive	Negative	Negative
Negative	Negative	Negative	Negative
Negative	Positive	Negative	Negative
Negative	Positive	Negative	Negative

Negative	Positive	Negative	Negative
Negative	Positive	Negative	Negative
Negative	Positive	Negative	Negative
Negative	Negative	Negative	Negative
Negative	Positive	Negative	Negative
Negative	Negative	Positive	Negative
Negative	Negative	Positive	Negative
Negative	Negative	Negative	Negative
Negative	Negative	Negative	Negative
Negative	Negative	Negative	Negative
Negative	Positive	Negative	Negative
Negative	Positive	Negative	Negative
Negative	Negative	Negative	Negative
Negative	Positive	Negative	Negative
Negative	Negative	Negative	Negative
Negative	Positive	Negative	Negative
Negative	Negative	Negative	Negative
Negative	Positive	Negative	Negative
Negative	Negative	Negative	Negative
Negative	Positive	Negative	Negative
Negative	Positive	Negative	Negative
Negative	Positive	Negative	Negative
Negative	Positive	Negative	Negative
Negative	Positive	Positive	Negative
Negative	Negative	Negative	Negative
Negative	Positive	Negative	Negative
Negative	Positive	Negative	Negative
Negative	Positive	Negative	Negative
Negative	Positive	Negative	Negative
Negative	Positive	Negative	Negative
Negative	Negative	Negative	Negative
Negative	Negative	Negative	Negative
Negative	Negative	Negative	Negative
Negative	Positive	Negative	Negative
Negative	Negative	Negative	Negative
Negative	Positive	Negative	Negative
Negative	Negative	Negative	Negative
Negative	Positive	Negative	Negative
Negative	Negative	Negative	Negative
Negative	Positive	Negative	Negative

Negative	Positive	Negative	Negative
Negative	Positive	Negative	Negative
Negative	Positive	Negative	Negative
Negative	Positive	Negative	Negative
Negative	Positive	Negative	Negative
Negative	Negative	Negative	Negative
Negative	Positive	Negative	Negative
Negative	Positive	Negative	Negative
Negative	Positive	Negative	Negative
Negative	Negative	Negative	Negative
Negative	Positive	Negative	Negative
Negative	Negative	Negative	Negative
Negative	Negative	Negative	Negative
Negative	Positive	Negative	Negative
Negative	Negative	Negative	Negative
Negative	Negative	Negative	Negative
Negative	Positive	Negative	Negative
Negative	Negative	Negative	Negative
Negative	Positive	Negative	Negative
Negative	Negative	Negative	Negative
Negative	Positive	Negative	Negative
Negative	Negative	Negative	Negative
Negative	Negative	Negative	Negative
Negative	Negative	Negative	Negative
Negative	Positive	Negative	Negative
Negative	Negative	Negative	Negative
Negative	Positive	Negative	Negative
Negative	Positive	Negative	Negative
Negative	Positive	Negative	Negative
Negative	Positive	Negative	Negative
Negative	Positive	Negative	Negative
Negative	Positive	Negative	Negative
Negative	Positive	Negative	Negative
Negative	Positive	Negative	Negative
Negative	Negative	Negative	Negative
Negative	Positive	Negative	Negative
Positive	Negative	Negative	Negative
Negative	Negative	Negative	Negative
Negative	Negative	Negative	Negative
Negative	Negative	Negative	Negative
Negative	Negative	Negative	Negative
Negative	Negative	Negative	Negative
Negative	Positive	Negative	Negative
Negative	Positive	Negative	Negative
Negative	Positive	Negative	Negative
Negative	Positive	Negative	Negative
Negative	Positive	Negative	Negative
Negative	Negative	Negative	Negative

Negative	Negative	Negative	Negative
Negative	Negative	Negative	Negative
Negative	Positive	Negative	Negative
Negative	Negative	Negative	Negative
Negative	Negative	Negative	Negative
Negative	Negative	Negative	Negative
Negative	Negative	Negative	Negative
Negative	Negative	Negative	Negative
Negative	Positive	Negative	Negative
Negative	Negative	Negative	Negative
Negative	Positive	Negative	Negative
Negative	Negative	Negative	Negative
Negative	Negative	Negative	Negative
Negative	Positive	Negative	Negative
Negative	Positive	Negative	Negative
Negative	Positive	Negative	Negative
Negative	Positive	Negative	Negative
Negative	Negative	Negative	Negative
Negative	Positive	Negative	Negative
Negative	Positive	Negative	Negative
Negative	Negative	Negative	Negative
Negative	Negative	Negative	Negative
Negative	Positive	Negative	Negative
Negative	Negative	Negative	Negative
Negative	Positive	Negative	Negative
Negative	Positive	Negative	Negative
Negative	Positive	Negative	Negative
Negative	Negative	Negative	Negative
Negative	Negative	Negative	Negative
Negative	Positive	Negative	Negative
Negative	Positive	Negative	Negative
Negative	Positive	Negative	Negative
Negative	Negative	Negative	Negative
Negative	Positive	Negative	Negative
Negative	Negative	Negative	Negative
Negative	Negative	Negative	Negative
Negative	Negative	Negative	Negative
Negative	Positive	Negative	Negative
Negative	Negative	Negative	Negative
Negative	Positive	Negative	Negative
Negative	Negative	Negative	Negative
Negative	Positive	Negative	Negative
Negative	Positive	Negative	Negative
Negative	Positive	Negative	Negative

Negative	Negative	Negative	Negative
Negative	Negative	Negative	Negative
Negative	Negative	Negative	Negative
Negative	Positive	Negative	Negative
Negative	Positive	Negative	Negative
Negative	Positive	Negative	Negative
Negative	Negative	Negative	Negative
Negative	Negative	Negative	Negative
Negative	Negative	Negative	Negative
Negative	Positive	Negative	Negative
Negative	Negative	Negative	Negative
Negative	Negative	Negative	Negative
Negative	Negative	Negative	Negative
Negative	Positive	Negative	Negative
Negative	Positive	Negative	Negative
Negative	Positive	Negative	Negative
Negative	Positive	Negative	Negative
Negative	Negative	Negative	Negative
Negative	Positive	Negative	Negative
Negative	Positive	Negative	Negative
Negative	Negative	Negative	Negative
Negative	Negative	Negative	Negative
Negative	Negative	Negative	Negative
Negative	Positive	Negative	Negative
Negative	Negative	Negative	Negative
Negative	Negative	Negative	Negative
Negative	Negative	Negative	Negative
Negative	Positive	Negative	Negative
Negative	Positive	Negative	Negative
Negative	Positive	Negative	Negative
Negative	Positive	Negative	Negative
Negative	Negative	Negative	Negative
Negative	Negative	Negative	Negative
Negative	Positive	Negative	Negative
Negative	Negative	Negative	Negative
Negative	Negative	Negative	Negative
Negative	Positive	Negative	Negative
Negative	Negative	Negative	Negative
Negative	Positive	Negative	Negative
Negative	Positive	Negative	Negative
Negative	Positive	Negative	Negative
Negative	Negative	Negative	Negative
Negative	Positive	Negative	Negative
Negative	Negative	Negative	Negative
Negative	Positive	Negative	Negative
Negative	Negative	Negative	Negative
Negative	Positive	Negative	Negative
Negative	Negative	Negative	Negative
Negative	Negative	Negative	Negative
Negative	Negative	Negative	Negative
Negative	Negative	Negative	Negative
Negative	Positive	Negative	Negative
Negative	Positive	Negative	Negative
Negative	Negative	Negative	Negative
Negative	Positive	Negative	Negative
Negative	Negative	Negative	Negative

Negative	Negative	Negative	Negative
Negative	Positive	Negative	Negative
Negative	Positive	Negative	Negative
Negative	Negative	Negative	Negative
Negative	Positive	Negative	Negative
Negative	Negative	Negative	Negative
Negative	Positive	Negative	Negative
Negative	Positive	Negative	Negative
Negative	Positive	Negative	Negative
Negative	Positive	Negative	Negative
Negative	Negative	Negative	Negative
Negative	Positive	Negative	Negative
Negative	Positive	Negative	Negative
Negative	Negative	Negative	Negative

Negative	Negative	Negative	Negative
Negative	Positive	Negative	Negative
Negative	Positive	Negative	Negative
Negative	Positive	Negative	Negative
Negative	Positive	Negative	Negative
Negative	Positive	Negative	Negative
Negative	Positive	Negative	Negative
Negative	Negative	Negative	Negative
Negative	Negative	Negative	Negative
Negative	Negative	Negative	Negative
Negative	Positive	Negative	Negative
Negative	Positive	Negative	Negative
Negative	Negative	Negative	Negative
Negative	Negative	Negative	Negative
Negative	Positive	Negative	Negative
Negative	Negative	Negative	Negative
Negative	Positive	Negative	Negative
Negative	Positive	Negative	Negative
Negative	Negative	Negative	Negative
Negative	Positive	Negative	Negative
Negative	Negative	Negative	Negative
Negative	Positive	Negative	Negative
Negative	Negative	Negative	Negative
Negative	Negative	Negative	Negative
Negative	Positive	Negative	Negative
Negative	Positive	Negative	Negative
Negative	Positive	Negative	Negative
Negative	Positive	Negative	Negative
Negative	Positive	Negative	Negative
Negative	Positive	Negative	Negative
Negative	Positive	Negative	Negative
Negative	Positive	Negative	Negative

Negative	Negative	Positive	Negative
Negative	Positive	Negative	Negative
Negative	Negative	Negative	Negative
Negative	Negative	Negative	Negative
Negative	Positive	Negative	Negative
Negative	Positive	Negative	Negative
Negative	Negative	Positive	Negative
Negative	Positive	Negative	Negative
Negative	Positive	Negative	Negative
Negative	Positive	Negative	Negative
Negative	Positive	Negative	Negative
Negative	Negative	Positive	Negative
Negative	Positive	Negative	Negative
Negative	Positive	Negative	Negative
Negative	Positive	Negative	Negative
Negative	Positive	Negative	Negative
Negative	Positive	Negative	Negative
Negative	Positive	Negative	Negative
Negative	Positive	Negative	Negative
Negative	Negative	Negative	Negative
Negative	Positive	Negative	Negative
Negative	Positive	Negative	Negative
Negative	Positive	Negative	Negative
Negative	Positive	Negative	Negative
Negative	Positive	Negative	Negative
Negative	Positive	Negative	Negative
Negative	Positive	Negative	Negative
Negative	Negative	Negative	Negative
Negative	Positive	Negative	Negative
Negative	Negative	Negative	Negative
Negative	Positive	Negative	Negative
Negative	Positive	Negative	Negative
Negative	Positive	Negative	Negative
Negative	Positive	Negative	Negative
Negative	Negative	Negative	Negative
Negative	Positive	Negative	Negative
Negative	Negative	Negative	Negative

Negative	Positive	Negative	Negative
Negative	Negative	Negative	Negative
Negative	Negative	Negative	Negative
Negative	Positive	Negative	Negative
Negative	Negative	Negative	Negative
Negative	Negative	Negative	Negative
Negative	Positive	Negative	Negative
Negative	Negative	Negative	Negative
Negative	Positive	Negative	Negative
Negative	Positive	Negative	Negative
Negative	Positive	Negative	Negative
Negative	Negative	Negative	Negative

Negative	Positive	Positive	Negative
Negative	Positive	Negative	Negative
Negative	Positive	Negative	Negative
Negative	Positive	Negative	Negative
Negative	Positive	Negative	Negative
Negative	Positive	Negative	Negative
Negative	Negative	Negative	Negative
Negative	Negative	Negative	Negative
Negative	Positive	Negative	Negative
Negative	Negative	Negative	Negative
Negative	Positive	Negative	Negative
Negative	Positive	Negative	Negative
Negative	Positive	Negative	Negative
Negative	Negative	Negative	Negative
Negative	Positive	Negative	Negative
Negative	Negative	Negative	Negative
Negative	Negative	Negative	Negative
Negative	Positive	Negative	Negative
Negative	Positive	Negative	Negative
Negative	Negative	Negative	Negative
Negative	Negative	Negative	Negative
Negative	Positive	Negative	Negative
Negative	Positive	Negative	
Negative	Positive	Negative	Negative
Negative	Negative	Negative	Negative
Negative	Negative	Negative	Negative
Negative	Positive	Negative	Negative

Negative	Positive	Negative	Negative
Negative	Positive	Negative	Negative
Negative	Positive	Negative	Negative
Negative	Positive	Negative	Negative
Negative	Positive	Negative	Negative
Negative	Positive	Negative	Negative
Negative	Positive	Negative	Negative
Negative	Negative	Positive	Negative
Negative	Positive	Negative	Negative
Negative	Negative	Positive	Negative
Negative	Positive	Negative	Negative
Negative	Positive	Negative	Negative
Negative	Negative	Negative	Negative
Negative	Positive	Negative	Negative

Negative	Positive	Negative	Negative
Negative	Positive	Negative	Negative
Negative	Positive	Negative	Negative
Negative	Negative	Negative	Negative
Negative	Positive	Negative	Negative
Negative	Negative	Negative	Negative
Negative	Negative	Negative	Negative

Negative	Positive	Negative	Negative
Negative	Positive	Negative	Negative
Negative	Positive	Negative	Negative
Negative	Positive	Negative	Negative
Negative	Positive	Negative	Negative
Negative	Positive	Negative	Negative
Negative	Positive	Negative	Negative
Negative	Positive	Negative	Negative
Negative	Positive	Negative	Negative
Negative	Positive	Negative	Negative
Negative	Positive	Negative	Negative
Negative	Positive	Negative	Negative
Negative	Negative	Negative	Negative
Negative	Positive	Negative	Negative
Negative	Positive	Negative	Negative
Negative	Positive	Negative	Negative
Negative	Negative	Negative	Negative
Negative	Positive	Negative	Negative
Negative	Positive	Negative	Negative
Negative	Positive	Negative	Negative

Negative	Positive	Negative	Negative
Negative	Positive	Negative	Negative
Negative	Positive	Negative	Negative
Negative	Positive	Negative	Negative
Negative	Negative	Negative	Negative
Negative	Negative	Negative	Negative
Negative	Positive	Negative	Negative
Negative	Positive	Negative	Negative
Negative	Positive	Negative	Negative
Negative	Positive	Negative	Negative
Negative	Positive	Negative	Negative
Negative	Negative	Negative	Negative
Negative	Positive	Negative	Negative
Negative	Negative	Negative	Negative
Negative	Positive	Negative	Negative
Negative	Positive	Negative	Negative
Negative	Positive	Negative	Negative
Negative	Positive	Negative	Negative
Negative	Negative	Negative	Negative
Negative	Negative	Negative	Negative
Negative	Positive	Negative	Negative
Negative	Positive	Negative	Negative
Negative	Positive	Negative	Negative
Negative	Positive	Negative	Negative
Negative	Negative	Negative	Negative
Negative	Negative	Negative	Negative
Negative	Positive	Negative	Negative

Negative	Negative	Negative	Negative
Negative	Negative	Negative	Negative
Negative	Positive	Negative	Negative
Negative	Negative	Negative	Negative
Negative	Negative	Negative	Negative
Negative	Negative	Negative	Negative
Negative	Positive	Negative	Negative
Negative	Positive	Negative	Negative
Negative	Negative	Negative	Negative
Negative	Positive	Negative	Negative
Negative	Negative	Negative	Negative
Negative	Negative	Negative	Negative
Negative	Positive	Negative	Negative
Negative	Positive	Negative	Negative
Negative	Positive	Negative	Negative
Negative	Negative	Negative	Negative
Negative	Positive	Negative	Negative
Negative	Positive	Negative	Negative
Negative	Negative	Negative	Negative
Negative	Negative	Negative	Negative
Negative	Positive	Negative	Negative
Negative	Positive	Negative	Negative
Negative	Negative	Negative	Negative
Negative	Positive	Negative	Negative
Negative	Positive	Negative	Negative
Negative	Positive	Negative	Negative
Negative	Positive	Negative	Negative
Negative	Positive	Negative	Negative
Negative	Positive	Negative	Negative
Negative	Positive	Negative	Negative
Negative	Positive	Negative	Negative
Negative	Positive	Negative	Negative
Negative	Negative	Positive	Negative
Negative	Positive	Negative	Negative
Negative	Negative	Negative	Negative
Negative	Positive	Negative	Negative
Negative	Positive	Negative	Negative
Negative	Negative	Negative	Negative
Negative	Negative	Negative	Negative
Negative	Negative	Negative	Negative
Negative	Negative	Negative	Negative

Negative Negative	Negative Negative
Negative Positive	Negative Negative
Negative Positive	Negative Negative

Negative	Positive	Negative	Negative
Negative	Positive	Negative	Negative
Negative	Positive	Negative	Negative
Negative	Positive	Negative	Negative
Negative	Positive	Negative	Negative
Negative	Negative	Negative	Negative
Negative	Positive	Negative	Negative
Negative	Positive	Negative	Negative
Negative	Positive	Negative	Negative
Negative	Positive	Negative	Negative
Negative	Positive	Negative	Negative
Negative	Positive	Negative	Negative
Negative	Positive	Negative	Negative
Negative	Positive	Negative	Negative
Negative	Negative	Negative	Negative
Negative	Negative	Negative	Positive
Negative	Positive	Negative	Negative
Negative	Negative	Negative	Negative
Negative	Positive	Negative	Negative
Negative	Negative	Positive	Negative
Negative	Negative	Negative	Negative
Negative	Positive	Negative	Negative
Negative	Negative	Negative	Negative
Negative	Positive	Negative	Negative
Negative	Negative	Negative	Negative
Negative	Negative	Negative	Negative
Negative	Negative	Negative	Negative
Negative	Positive	Negative	Negative
Negative	Positive	Negative	Negative
Negative	Negative	Negative	Negative
Negative	Positive	Negative	Negative
Negative	Negative	Negative	Negative
Negative	Negative	Negative	Negative
Negative	Positive	Negative	Negative
Negative	Negative	Negative	Negative
Negative	Negative	Negative	Negative
Negative	Positive	Negative	Negative
Negative	Positive	Negative	Negative
Negative	Negative	Negative	Negative
Negative	Positive	Negative	Negative
Negative	Positive	Negative	Negative
Negative	Positive	Negative	Negative
Negative	Positive	Negative	Negative
Negative	Negative	Negative	Negative
Negative	Positive	Negative	Negative
Negative	Positive	Negative	Negative
Negative	Positive	Negative	Negative
Negative	Negative	Negative	Negative

Negative	Negative	Negative	Negative
Negative	Positive	Negative	Negative
Negative	Positive	Negative	Negative
Negative	Negative	Negative	Negative
Negative	Positive	Negative	Negative
Negative	Negative	Negative	Negative
Negative	Positive	Negative	Negative
Negative	Positive	Negative	Negative
Negative	Positive	Negative	Negative
Negative	Positive	Negative	Negative
Negative	Positive	Negative	Negative
Negative	Negative	Negative	Negative
Negative	Positive	Negative	Negative
Negative	Negative	Negative	Negative
Negative	Positive	Negative	Negative
Negative	Positive	Negative	Negative
Negative	Positive	Negative	Negative
Negative	Negative	Negative	Negative
Negative	Negative	Negative	Negative

Negative	Negative	Negative	Negative
Negative	Positive	Negative	Negative
Negative	Positive	Negative	Negative
Negative	Positive	Negative	Negative
Negative	Positive	Negative	Negative
Negative	Negative	Negative	Negative
Negative	Negative	Negative	Negative
Negative	Negative	Negative	Negative
Negative	Positive	Negative	Negative
Negative	Negative	Negative	Negative
Negative	Negative	Negative	Negative
Negative	Positive	Negative	Negative
Negative	Positive	Negative	Negative
Negative	Negative	Negative	Negative
Negative	Negative	Negative	Negative

Negative	Negative	Negative	Negative
Negative	Positive	Negative	Negative
Negative	Negative	Negative	Negative
Negative	Positive	Negative	Negative
Negative	Positive	Negative	Negative
Negative	Positive	Negative	Negative
Negative	Negative	Negative	Negative
Negative	Negative	Negative	Negative
Negative	Negative	Negative	Negative
Negative	Positive	Negative	Negative

Negative Positive Negative Negative

Negative	Positive	Negative	Negative
Negative	Positive	Negative	Negative
Negative	Negative	Negative	Negative
Negative	Negative	Negative	Negative
Negative	Positive	Negative	Negative
Negative	Negative	Negative	Negative
Negative	Positive	Negative	Negative
Negative	Negative	Negative	Negative
Negative	Positive	Negative	Negative
Negative	Negative	Negative	Negative
Negative	Negative	Negative	Negative
Negative	Negative	Negative	Negative
Negative	Negative	Negative	Negative
Negative	Negative	Negative	Negative
Negative	Negative	Negative	Negative
Negative	Positive	Negative	Negative
Negative	Positive	Negative	Negative
Negative	Positive	Negative	Negative
Negative	Negative	Negative	Negative
Negative	Negative	Negative	Negative
Negative	Negative	Negative	Negative
Negative	Positive	Negative	Negative
Negative	Positive	Negative	Negative
Negative	Negative	Negative	Negative
Negative	Negative	Negative	Negative
Negative	Negative	Negative	Negative
Negative	Positive	Negative	Negative
Negative	Negative	Negative	Negative
Negative	Negative	Negative	Negative
Negative	Positive	Negative	Negative
Negative	Positive	Negative	Negative
Negative	Negative	Negative	Negative
Negative	Negative	Negative	Negative
Negative	Negative	Negative	Negative
Negative	Negative	Negative	Negative
Negative	Positive	Negative	Negative
Negative	Positive	Negative	Negative
Negative	Positive	Negative	Negative
Negative	Positive	Negative	Negative
Negative	Positive	Negative	Negative
Negative	Positive	Negative	Negative
Negative	Positive	Negative	Negative
Negative	Positive	Negative	Negative
Negative	Negative	Negative	Negative

Negative	Positive	Negative	Negative
Negative	Positive	Negative	Negative
Negative	Negative	Negative	Negative
Negative	Positive	Negative	Negative
Negative	Positive	Negative	Negative
Negative	Positive	Negative	Negative
Negative	Positive	Negative	Negative
Negative	Positive	Negative	Negative
Negative	Positive	Negative	Positive
Negative	Negative	Negative	Negative
Negative	Positive	Negative	Negative
Negative	Positive	Negative	Negative
Negative	Positive	Negative	Negative
Negative	Negative	Negative	Positive
Negative	Negative	Negative	Negative
Negative	Positive	Negative	Negative
Negative	Positive	Negative	Negative
Negative	Positive	Negative	Negative
Negative	Negative	Negative	Negative
Negative	Positive	Negative	Negative
Negative	Positive	Negative	Negative
Negative	Negative	Negative	Negative

Negative	Positive	Negative	Negative
Negative	Negative	Negative	Negative
Negative	Negative	Negative	Negative
Negative	Positive	Negative	Negative
Negative	Negative	Negative	Negative
Negative	Positive	Negative	Negative
Negative	Positive	Negative	Negative
Negative	Positive	Negative	Negative
Negative	Positive	Negative	Negative
Negative	Positive	Negative	Negative
Negative	Positive	Negative	Negative
Negative	Positive	Negative	Negative
Negative	Negative	Negative	Negative
Negative	Positive	Negative	Negative
Negative	Positive	Negative	Negative
Negative	Positive	Negative	Negative
Negative	Positive	Negative	Negative
Negative	Positive	Negative	Negative
Negative	Positive	Negative	Negative
Negative	Positive	Negative	Negative
Negative	Positive	Negative	Negative
Negative	Negative	Negative	Negative
Negative	Positive	Negative	Negative
Negative	Positive	Negative	Negative
Negative	Negative	Negative	Negative
Negative	Negative	Negative	Negative
Negative	Negative	Negative	Negative
Negative	Positive	Negative	Negative
Negative	Negative	Negative	Negative
Negative	Positive	Negative	Negative
Negative	Positive	Negative	Negative
Negative	Positive	Negative	Negative
Negative	Negative	Negative	Negative

Negative	Negative	Negative	Negative
Negative	Positive	Negative	Negative
Negative	Positive	Negative	Negative

Negative	Negative	Negative	Negative
Negative	Positive	Negative	Negative
Negative	Negative	Negative	Negative
Negative	Negative	Negative	Negative
Negative	Negative	Negative	Negative
Negative	Positive	Negative	Negative
Negative	Positive	Negative	Negative
Negative	Positive	Negative	Negative
Negative	Negative	Negative	Negative
Negative	Positive	Negative	Negative
Negative	Negative	Negative	Negative
Negative	Negative	Negative	Negative
Negative	Negative	Negative	Negative
Negative	Positive	Negative	Negative
Negative	Positive	Negative	Negative
Negative	Negative	Negative	Negative
Negative	Positive	Negative	Negative
Negative	Negative	Negative	Negative
Negative	Positive	Negative	Negative
Negative	Positive	Negative	Negative
Negative	Negative	Negative	Negative
Negative	Positive	Negative	Negative
Negative	Negative	Negative	Negative
Negative	Positive	Negative	Negative
Negative	Positive	Negative	Negative
Negative	Positive	Negative	Negative
Negative	Positive	Negative	Negative
Negative	Positive	Negative	Negative
Negative	Positive	Negative	Negative
Negative	Positive	Negative	Negative
Negative	Positive	Negative	Negative
Negative	Positive	Negative	Negative
Negative	Positive	Negative	Negative
Negative	Negative	Negative	Negative
Negative	Negative	Negative	Negative
Negative	Positive	Negative	Negative
Negative	Positive	Negative	Negative
Negative	Positive	Negative	Negative
Negative	Positive	Negative	Negative
Negative	Negative	Negative	Negative
Negative	Positive	Negative	Negative
Negative	Positive	Negative	Negative
Negative	Positive	Negative	Negative

Negative	Negative	Negative	Negative
Negative	Positive	Negative	Negative
Negative	Positive	Negative	Negative
Negative	Positive	Negative	Negative
Negative	Positive	Negative	Negative
Negative	Negative	Negative	Negative
Negative	Negative	Negative	Negative

Negative	Positive	Negative	Negative
Negative	Negative	Negative	Negative
Negative	Positive	Negative	Negative
Negative	Positive	Negative	Negative
Negative	Positive	Negative	Negative
Negative	Positive	Negative	Negative
Negative	Positive	Negative	Negative
Negative	Positive	Negative	Negative

Negative	Negative	Negative	Negative
Negative	Positive	Negative	Negative
Negative	Positive	Negative	Negative
Negative	Positive	Negative	Negative
Negative	Positive	Negative	Negative
Negative	Positive	Negative	Negative
Negative	Positive	Negative	Negative
Negative	Positive	Negative	Negative
Negative	Positive	Negative	Negative
Negative	Negative	Negative	Negative
Negative	Positive	Negative	Negative
Negative	Negative	Negative	Negative
Negative	Positive	Negative	Negative
Negative	Negative	Negative	Negative
Negative	Negative	Negative	Negative
Negative	Positive	Negative	Negative
Negative	Positive	Negative	Negative
Negative	Positive	Negative	Negative
Negative	Positive	Negative	Negative
Negative	Positive	Negative	Negative
Negative	Positive	Negative	Negative
Negative	Negative	Negative	Negative
Negative	Positive	Negative	Negative
Negative	Negative	Negative	Negative
Negative	Negative	Negative	Negative
Negative	Positive	Negative	Negative
Negative	Negative	Negative	Negative
Negative	Positive	Negative	Negative
Negative	Negative	Negative	Negative
Negative	Positive	Negative	Negative
Negative	Negative	Negative	Negative
Negative	Positive	Negative	Negative
Negative	Positive	Negative	Negative
Negative	Positive	Negative	Negative
Negative	Positive	Negative	Negative
Negative	Negative	Negative	Negative
Negative	Positive	Negative	Negative
Negative	Positive	Negative	Negative

Negative	Negative	Negative	Negative
Negative	Negative	Negative	Negative
Negative	Negative	Negative	Negative
Negative	Negative	Negative	Negative
Negative	Negative	Negative	Negative
Negative	Positive	Negative	Negative

Negative Positive Negative Negative

Negative	Negative	Negative	Negative
Negative	Positive	Negative	Negative
Negative	Positive	Negative	Negative
Negative	Negative	Negative	Negative
Negative	Positive	Negative	Negative
Negative	Positive	Negative	Negative
Negative	Positive	Negative	Negative
Negative	Positive	Negative	Negative
Negative	Positive	Negative	Negative
Negative	Positive	Negative	Negative
Negative	Positive	Negative	Negative
Negative	Negative	Negative	Negative
Negative	Positive	Negative	Negative
Negative	Positive	Negative	Negative

Negative	Positive	Negative	Negative
Negative	Negative	Negative	Negative
Negative	Negative	Negative	Negative
Negative	Positive	Negative	Negative
Negative	Negative	Negative	Positive
Negative	Negative	Negative	Negative
Negative	Negative	Negative	Negative
Negative	Negative	Negative	Negative
Negative	Negative	Negative	Negative
Negative	Negative	Negative	Negative
Negative	Positive	Negative	Negative
Negative	Positive	Negative	Negative
Negative	Negative	Negative	Negative
Negative	Positive	Negative	Negative
Negative	Positive	Negative	Negative
Negative	Negative	Negative	Negative
Negative	Positive	Negative	Negative
Negative	Negative	Negative	Negative
Negative	Positive	Negative	Negative
Negative	Positive	Negative	Negative
Negative	Positive	Negative	Negative

Negative	Negative	Negative	Negative
Negative	Positive	Negative	Negative
Negative	Positive	Negative	Negative
Negative	Negative	Negative	Negative
Negative	Negative	Negative	Negative
Negative	Negative	Negative	Negative
Negative	Positive	Negative	Negative
Negative	Positive	Negative	Negative
Negative	Positive	Negative	Negative
Negative	Positive	Negative	Negative
Negative	Positive	Negative	Negative
Negative	Positive	Negative	Negative
Negative	Negative	Negative	Negative
Negative	Positive	Negative	Negative
Negative	Positive	Negative	Negative
Negative	Negative	Negative	Negative
Negative	Positive	Negative	Negative
Negative	Positive	Negative	Negative
Negative	Negative	Negative	Negative
Negative	Negative	Negative	Negative
Negative	Positive	Negative	Negative
Negative	Positive	Negative	Negative
Negative	Negative	Negative	Negative
Negative	Positive	Negative	Negative
Negative	Positive	Negative	Negative
Negative	Negative	Negative	Negative
Negative	Positive	Negative	Negative
Negative	Positive	Negative	Negative
Negative	Negative	Negative	Negative
Negative	Positive	Negative	Negative
Negative	Positive	Negative	Negative
Negative	Positive	Negative	Negative
Negative	Negative	Negative	Negative
Negative	Positive	Negative	Negative
Negative	Positive	Negative	Negative
Negative	Positive	Negative	Negative
Negative	Positive	Negative	Negative
Negative	Positive	Negative	Negative
Negative	Negative	Negative	Negative
Negative	Positive	Negative	Negative
Negative	Positive	Negative	Negative
Negative	Negative	Negative	Negative
Negative	Negative	Negative	Negative
Negative	Negative	Negative	Negative
Negative	Negative	Negative	Negative
Negative	Negative	Negative	Negative
Negative	Negative	Negative	Negative
Negative	Negative	Negative	Negative
Negative	Negative	Negative	Negative
Negative	Negative	Negative	Negative
Negative	Positive	Negative	Negative
Negative	Negative	Negative	Negative
Negative	Negative	Negative	Negative
Negative	Negative	Negative	Negative
Negative	Positive	Negative	Negative

Negative Positive Negative Negative

Negative Positive Negative Negative

Negative Positive	Negative Negative
Negative Positive	Negative Negative

Negative Positive	Negative Negative
Negative Positive	Negative Negative
Negative Negative	Negative Negative
Negative Negative	Negative Negative

Negative	Positive	Negative	Negative
Negative	Negative	Negative	Negative
Negative	Positive	Negative	Negative
Negative	Positive	Negative	Negative
Negative	Negative	Negative	Negative
Negative	Positive	Negative	Negative
Negative	Negative	Negative	Negative
Negative	Negative	Negative	Negative
Negative	Positive	Negative	Negative
Negative	Positive	Negative	Negative
Negative	Positive	Negative	Negative
Negative	Negative	Negative	Negative
Negative	Positive	Negative	Negative
Negative	Positive	Negative	Negative
Negative	Positive	Negative	Negative
Negative	Negative	Negative	Negative
Negative	Positive	Negative	Negative
Negative	Positive	Negative	Negative
Negative	Positive	Negative	Negative
Negative	Positive	Negative	Negative
Negative	Positive	Negative	Negative
Negative	Negative	Negative	Negative
Negative	Negative	Negative	Negative
Negative	Negative	Negative	Negative
Negative	Positive	Negative	Negative
Negative	Positive	Negative	Negative
Negative	Negative	Negative	
Negative	Positive	Negative	Negative
Negative	Positive	Negative	Negative
Negative	Positive	Negative	Negative
Negative	Positive	Negative	Negative
Negative	Negative	Negative	Negative
Negative	Negative	Negative	Negative
Negative	Positive	Negative	Negative
Negative	Positive	Negative	Negative
Negative	Positive	Negative	Negative
Negative	Negative	Negative	Negative
Negative	Positive	Negative	Negative
Negative	Positive	Negative	Negative
Negative	Positive	Negative	Negative
Negative	Positive	Negative	Negative
Negative	Positive	Negative	Negative
Negative	Positive	Negative	Negative
Negative	Positive	Negative	Negative
Negative	Negative	Negative	Negative
Negative	Positive	Negative	Negative
Negative	Positive	Negative	Negative
Negative	Positive	Negative	Negative
Negative	Negative	Negative	Negative
Negative	Negative	Negative	Negative
Negative	Negative	Negative	Negative

Negative Positive	Negative Negative
Negative Negative	Negative Negative
Negative Positive	Negative Negative
Negative Positive	Negative Negative
Negative Negative	Negative Negative

Negative	Positive	Negative	Negative
Negative	Negative	Negative	Negative
Negative	Positive	Negative	Negative
Negative	Positive	Negative	Negative
Negative	Negative	Negative	Negative
Negative	Negative	Negative	Negative
Negative	Negative	Negative	Negative
Negative	Positive	Negative	Negative

Negative	Positive	Negative	Negative

Negative	Positive	Negative	Negative
Negative	Negative	Negative	Negative
Negative	Positive	Negative	Negative
Negative	Negative	Negative	Negative
Negative	Positive	Negative	Negative
Negative	Negative	Negative	Negative
Negative	Positive	Negative	Negative
Negative	Negative	Negative	Negative
Negative	Positive	Negative	Negative

Negative	Positive	Negative	Negative
Negative	Negative	Negative	Negative
Negative	Positive	Negative	Negative
Negative	Positive	Negative	Negative
Negative	Negative	Negative	Negative
Negative	Negative	Negative	Negative
Negative	Positive	Negative	Negative
Negative	Negative	Negative	Negative
Negative	Positive	Negative	Negative
Negative	Positive	Negative	Negative
Negative	Positive	Negative	Negative
Negative	Negative	Negative	Negative
Negative	Negative	Negative	Negative
Negative	Positive	Negative	Negative
Negative	Negative	Negative	Negative
Negative	Negative	Negative	Negative
Negative	Positive	Negative	Negative
Negative	Negative	Negative	Negative
Negative	Negative	Negative	Negative
Negative	Positive	Negative	Negative
Negative	Positive	Negative	Negative
Negative	Positive	Negative	Negative

Negative	Positive	Negative	Negative
Negative	Positive	Negative	Negative
Negative	Positive	Negative	Negative
Negative	Positive	Negative	Negative
Negative	Negative	Negative	Negative
Negative	Positive	Negative	Negative
Negative	Positive	Negative	Negative
Negative	Positive	Negative	Negative
Negative	Negative	Negative	Negative
Negative	Positive	Negative	Negative
Negative	Positive	Negative	Negative
Negative	Positive	Negative	Negative
Negative	Positive	Negative	Negative
Negative	Positive	Negative	Negative
Negative	Positive	Negative	Negative
Negative	Positive	Negative	Negative
Negative	Negative	Negative	Negative
Negative	Negative	Negative	Negative
Negative	Negative	Positive	Negative
Negative	Positive	Negative	Negative
Negative	Positive	Negative	Negative
Negative	Positive	Negative	Negative
Negative	Negative	Negative	Negative
Negative	Negative	Positive	Negative
Negative	Negative	Positive	Negative
Negative	Positive	Negative	Negative
Negative	Positive	Negative	Negative
Negative	Positive	Negative	Negative
Negative	Positive	Negative	Negative
Negative	Negative	Negative	Negative
Negative	Positive	Negative	Negative
Negative	Positive	Negative	Negative
Negative	Positive	Negative	Negative

Negative	Negative	Negative	Negative
Negative	Positive	Negative	Negative
Negative	Negative	Negative	Negative
Negative	Negative	Negative	Negative

Negative Positive	Negative Negative
Negative Positive	Negative Negative
Negative Positive	Negative Negative
Negative Negative	Negative Negative
Negative Negative	Negative Negative
Negative Negative	Negative Negative
Negative Positive	Negative Negative

Negative	Positive	Negative	Negative
Negative	Positive	Negative	Negative
Negative	Negative	Negative	Negative
Negative	Positive	Negative	Negative
Negative	Positive	Negative	Negative
Negative	Positive	Negative	Negative
Negative	Negative	Negative	Negative
Negative	Positive	Negative	Negative
Negative	Positive	Negative	Negative
Negative	Positive	Negative	Negative
Negative	Positive	Negative	Negative
Negative	Positive	Negative	Negative
Negative	Positive	Negative	Negative
Negative	Negative	Negative	Negative
Negative	Negative	Negative	Negative
Negative	Positive	Negative	Negative
Negative	Negative	Negative	Negative
Negative	Positive	Negative	Negative
Negative	Negative	Negative	Negative
Negative	Negative	Negative	Negative
Negative	Positive	Negative	Negative
Negative	Positive	Negative	Negative
Negative	Positive	Negative	Negative
Negative	Positive	Negative	Negative
Negative	Negative	Negative	Negative
Negative	Positive	Negative	Negative
Negative	Positive	Negative	Negative
Negative	Positive	Negative	Negative
Negative	Positive	Negative	Negative
Negative	Positive	Negative	Negative
Negative	Positive	Negative	Negative
Negative	Positive	Negative	Negative
Negative	Positive	Negative	Negative
Negative	Positive	Negative	Negative
Negative	Positive	Negative	Negative
Negative	Negative	Negative	Negative
Negative	Positive	Negative	Negative
Negative	Positive	Negative	Negative
Negative	Negative	Negative	Negative
Negative	Positive	Negative	Negative
Negative	Negative	Negative	Negative
Negative	Negative	Negative	Negative
Negative	Positive	Negative	Negative
Negative	Negative	Negative	Negative
Negative	Positive	Negative	Negative
Negative	Negative	Negative	Negative
Negative	Positive	Negative	Negative
Negative	Positive	Negative	Negative
Negative	Positive	Negative	Negative
Negative	Positive	Negative	Negative
Negative	Positive	Negative	Negative
Negative	Negative	Negative	Negative
Negative	Positive	Negative	Negative
Negative	Positive	Negative	Negative
Negative	Negative	Negative	Negative
Negative	Positive	Negative	Negative

Negative	Negative	Negative	Negative
Negative	Positive	Negative	Negative
Negative	Positive	Negative	Negative
Negative	Positive	Negative	Negative
Negative	Negative	Negative	Negative
Negative	Negative	Negative	Negative
Negative	Negative	Negative	Negative
Negative	Positive	Negative	Negative
Negative	Negative	Negative	Negative
Negative	Positive	Negative	Negative
Negative	Positive	Negative	Negative
Negative	Positive	Negative	Negative
Negative	Negative	Negative	Negative
Negative	Positive	Negative	Negative
Negative	Positive	Negative	Negative
Negative	Positive	Negative	Negative
Negative	Positive	Negative	Negative
Negative	Positive	Negative	Negative
Negative	Positive	Negative	Negative
Negative	Positive	Negative	Negative
Negative	Positive	Negative	Negative
Negative	Negative	Positive	Negative
Negative	Negative	Negative	Negative
Negative	Negative	Negative	Negative
Negative	Negative	Negative	Negative
Negative	Negative	Negative	Negative
Negative	Negative	Negative	Negative
Negative	Positive	Negative	Negative
Negative	Positive	Negative	Negative
Negative	Positive	Negative	Negative
Negative	Positive	Negative	Negative
Negative	Positive	Negative	Negative
Negative	Positive	Negative	Negative
Negative	Positive	Negative	Negative
Negative	Negative	Negative	Negative
Negative	Positive	Negative	Negative
Negative	Positive	Negative	Negative
Negative	Positive	Negative	Negative
Negative	Negative	Negative	Negative
Negative	Positive	Negative	Negative
Negative	Positive	Negative	Negative
Negative	Positive	Negative	Negative
Negative	Positive	Negative	Negative
Negative	Positive	Negative	Negative
Negative	Positive	Negative	Negative
Negative	Negative	Negative	Negative
Negative	Negative	Negative	Negative
Negative	Positive	Negative	Negative
Negative	Positive	Negative	Negative
Negative	Positive	Negative	Negative
Negative	Positive	Negative	Negative
Negative	Positive	Negative	Negative
Negative	Positive	Negative	Negative
Negative	Positive	Negative	Negative
Negative	Positive	Negative	Negative

Negative	Positive	Negative	Negative
Negative	Positive	Negative	Negative
Negative	Positive	Negative	Negative
Negative	Negative	Negative	Negative
Negative	Positive	Negative	Negative
Negative	Negative	Negative	Negative
Negative	Positive	Negative	Negative
Negative	Positive	Negative	Negative
Negative	Positive	Negative	Negative
Negative	Positive	Negative	Negative
Negative	Positive	Negative	Negative
Negative	Negative	Negative	Negative
Negative	Negative	Negative	Negative
Negative	Positive	Negative	Negative
Negative	Positive	Negative	Negative
Negative	Positive	Negative	Negative
Negative	Positive	Negative	Negative
Negative	Positive	Negative	Negative
Negative	Positive	Negative	Negative
Negative	Positive	Negative	Negative
Negative	Negative	Negative	Negative
Negative	Positive	Negative	Negative
Negative	Negative	Negative	Negative
Negative	Positive	Negative	Negative
Negative	Negative	Negative	Negative
Negative	Negative	Negative	Negative
Negative	Negative	Negative	Negative
Negative	Positive	Negative	Negative
Negative	Positive	Negative	Negative
Negative	Positive	Negative	Negative
Negative	Positive	Negative	Negative
Negative	Positive	Negative	Negative
Negative	Positive	Negative	Negative
Negative	Positive	Negative	Negative
Negative	Positive	Negative	Negative
Negative	Negative	Negative	Negative
Negative	Positive	Negative	Negative
Negative	Positive	Negative	Negative
Negative	Positive	Negative	Negative
Negative	Positive	Negative	Negative
Negative	Positive	Negative	Negative
Negative	Positive	Negative	Negative
Negative	Negative	Negative	Negative
Negative	Positive	Negative	Negative
Negative	Positive	Negative	Negative
Negative	Positive	Negative	Negative
Negative	Positive	Negative	Negative
Negative	Positive	Negative	Negative
Negative	Positive	Negative	Negative
Negative	Negative	Positive	Negative
Negative	Positive	Negative	Negative
Negative	Negative	Negative	Negative
Negative	Positive	Negative	Negative
Negative	Positive	Negative	Negative
Negative	Positive	Negative	Negative
Negative	Negative	Negative	Negative

Negative	Positive	Negative	Negative
Negative	Positive	Negative	Negative
Negative	Positive	Negative	Negative
Negative	Positive	Negative	Negative
Negative	Positive	Negative	Negative
Negative	Positive	Negative	Negative
Negative	Positive	Negative	Negative
Negative	Positive	Negative	Negative
Negative	Positive	Negative	Negative
Negative	Positive	Negative	Negative
Negative	Positive	Negative	Negative
Negative	Positive	Negative	Negative
Negative	Positive	Negative	Negative
Negative	Positive	Negative	Negative
Negative	Positive	Negative	Negative
Negative	Negative	Negative	Negative
Negative	Negative	Negative	Negative
Negative	Positive	Negative	Negative
Negative	Positive	Negative	Negative
Negative	Positive	Negative	Negative
Negative	Negative	Negative	Negative
Negative	Negative	Negative	Negative
Negative	Negative	Negative	Negative
Negative	Positive	Negative	Negative
Negative	Positive	Negative	Negative
Negative	Negative	Negative	Negative
Negative	Positive	Negative	Negative
Negative	Positive	Negative	Negative
Negative	Positive	Negative	Negative
Negative	Positive	Negative	Negative
Negative	Positive	Negative	Negative
Negative	Negative	Negative	Negative
Negative	Positive	Negative	Negative
Negative	Positive	Negative	Negative
Negative	Negative	Negative	Negative
Negative	Positive	Negative	Negative
Negative	Positive	Negative	Negative
Negative	Positive	Positive	Negative
Negative	Positive	Negative	Negative
Negative	Positive	Negative	Negative
Negative	Positive	Negative	Negative
Negative	Positive	Negative	Negative
Negative	Positive	Negative	Negative
Negative	Positive	Negative	Negative
Negative	Positive	Negative	Negative
Negative	Positive	Negative	Negative
Negative	Positive	Negative	Negative
Negative	Positive	Negative	Negative
Negative	Positive	Negative	Negative
Negative	Positive	Negative	Negative
Negative	Positive	Negative	Negative
Negative	Negative	Negative	Negative
Negative	Positive	Negative	Negative
Negative	Positive	Negative	Negative
Negative	Positive	Negative	Negative
Negative	Negative	Negative	Negative

Negative	Positive	Negative	Negative
Negative	Negative	Negative	Negative
Negative	Positive	Negative	Negative
Negative	Negative	Negative	Negative
Negative	Positive	Negative	Negative
Negative	Positive	Negative	Negative
Negative	Positive	Negative	Negative
Negative	Negative	Negative	Negative
Negative	Positive	Negative	Negative
Negative	Positive	Negative	Negative
Negative	Positive	Negative	Negative
Negative	Positive	Negative	Negative
Negative	Positive	Negative	Negative
Negative	Positive	Negative	Negative
Negative	Positive	Negative	Negative
Negative	Negative	Negative	Negative
Negative	Negative	Negative	Negative
Negative	Positive	Negative	Negative
Negative	Positive	Negative	Negative
Negative	Positive	Negative	Negative
Negative	Positive	Negative	Negative
Negative	Positive	Negative	Negative
Negative	Positive	Negative	Negative
Negative	Positive	Negative	Negative
Negative	Positive	Negative	Negative
Negative	Positive	Negative	Negative
Negative	Positive	Negative	Negative
Negative	Positive	Negative	Negative
Negative	Positive	Negative	Negative
Negative	Positive	Negative	Negative
Negative	Positive	Negative	Negative
Negative	Positive	Negative	Negative
Negative	Positive	Negative	Negative
Negative	Positive	Negative	Negative
Negative	Positive	Negative	Negative
Negative	Positive	Negative	Negative
Negative	Negative	Negative	Negative
Negative	Positive	Negative	Negative
Negative	Positive	Negative	Negative
Negative	Positive	Negative	Negative
Negative	Positive	Negative	Negative
Negative	Positive	Negative	Negative
Negative	Positive	Negative	Negative
Negative	Positive	Negative	Negative
Negative	Positive	Negative	Negative
Negative	Positive	Negative	Negative
Negative	Positive	Negative	Negative
Positive	Positive	Negative	Negative
Negative	Positive	Negative	Negative
Negative	Positive	Negative	Negative
Negative	Positive	Negative	Negative
Negative	Positive	Negative	Negative
Negative	Positive	Positive	Negative
Negative	Positive	Negative	Negative
Negative	Positive	Negative	Negative

Negative	Positive	Negative	Negative
Negative	Positive	Negative	Negative
Negative	Negative	Negative	Negative
Negative	Negative	Negative	Negative
Negative	Positive	Negative	Negative
Negative	Positive	Negative	Negative
Negative	Negative	Negative	Negative
Negative	Positive	Negative	Negative
Negative	Positive	Negative	Negative
Negative	Positive	Negative	Negative
Negative	Positive	Negative	Negative
Negative	Negative	Negative	Negative
Negative	Positive	Negative	Positive
Negative	Positive	Negative	Negative
Negative	Negative	Negative	Negative
Negative	Positive	Negative	Negative
Negative	Negative	Negative	Negative
Negative	Positive	Negative	Negative
Negative	Negative	Negative	Negative
Negative	Negative	Negative	Negative
Negative	Positive	Negative	Negative
Negative	Positive	Negative	Negative
Negative	Positive	Negative	Negative
Negative	Positive	Negative	Negative
Negative	Negative	Negative	Negative
Negative	Positive	Negative	Negative
Negative	Positive	Negative	Negative
Negative	Negative	Negative	Negative
Positive	Positive	Negative	Negative
Positive	Positive	Negative	Negative
Negative	Negative	Negative	Negative
Negative	Negative	Negative	Negative
Negative	Positive	Negative	Negative
Negative	Positive	Negative	Negative
Negative	Positive	Negative	Negative
Negative	Negative	Negative	Negative
Negative	Positive	Negative	Negative
Negative	Negative	Negative	Negative
Negative	Negative	Negative	Negative
Negative	Negative	Negative	Negative
Negative	Positive	Negative	Negative
Negative	Positive	Negative	Negative
Negative	Positive	Negative	Negative
Negative	Positive	Negative	Negative
Negative	Positive	Negative	Negative
Negative	Negative	Negative	Negative
Negative	Positive	Negative	Negative
Negative	Negative	Negative	Negative
Negative	Negative	Negative	Negative
Negative	Negative	Negative	Negative
Negative	Positive	Negative	Negative
Negative	Positive	Negative	Negative
Negative	Negative	Negative	Negative
Negative	Positive	Negative	Negative
Negative	Negative	Positive	Negative
Negative	Positive	Negative	Negative

Negative	Negative	Positive	Negative
Negative	Positive	Negative	Negative
Negative	Positive	Negative	Negative
Negative	Positive	Negative	Negative
Negative	Positive	Negative	Negative
Negative	Negative	Negative	Negative
Negative	Negative	Negative	Negative
Negative	Positive	Negative	Negative
Negative	Positive	Negative	Negative
Negative	Positive	Negative	Negative
Negative	Positive	Negative	Negative
Negative	Positive	Negative	Negative
Negative	Positive	Negative	Negative
Negative	Positive	Negative	Negative
Negative	Positive	Negative	Negative
Negative	Negative	Negative	Negative
Negative	Positive	Negative	Negative
Negative	Negative	Negative	Negative
Negative	Positive	Negative	Negative
Negative	Positive	Negative	Negative
Negative	Positive	Negative	Negative
Negative	Positive	Negative	Negative
Negative	Positive	Negative	Negative
Negative	Positive	Negative	Negative
Negative	Positive	Negative	Negative
Negative	Negative	Negative	Negative
Negative	Negative	Negative	Negative
Negative	Positive	Negative	Negative
Negative	Negative	Negative	Negative
Negative	Negative	Negative	Negative
Negative	Positive	Negative	Negative
Negative	Negative	Negative	Negative
Negative	Positive	Negative	Negative
Negative	Positive	Negative	Negative
Negative	Positive	Negative	Negative
Negative	Positive	Negative	Negative
Negative	Positive	Negative	Negative
Negative	Positive	Negative	Negative
Negative	Negative	Negative	Negative
Negative	Positive	Negative	Negative
Negative	Positive	Negative	Negative
Negative	Negative	Negative	Negative
Negative	Positive	Negative	Negative
Negative	Positive	Negative	Negative
Negative	Positive	Negative	Negative
Negative	Positive	Negative	Negative
Negative	Negative	Negative	Negative
Negative	Positive	Negative	Negative
Negative	Negative	Negative	Negative
Negative	Positive	Negative	Negative
Negative	Positive	Negative	Negative
Negative	Positive	Negative	Negative
Negative	Positive	Negative	Negative
Negative	Positive	Negative	Negative
Negative	Positive	Negative	Negative
Negative	Negative	Negative	Negative

Negative	Positive	Negative	Negative
Negative	Positive	Negative	Negative
Negative	Negative	Negative	Negative
Negative	Positive	Negative	Negative
Negative	Positive	Negative	Negative
Negative	Positive	Negative	Negative
Negative	Positive	Negative	Negative
Negative	Negative	Negative	Negative
Negative	Positive	Negative	Negative
Negative	Positive	Negative	Negative
Negative	Positive	Negative	Negative
Negative	Positive	Negative	Negative
Negative	Negative	Negative	Negative
Negative	Positive	Negative	Negative
Negative	Positive	Negative	Negative
Negative	Negative	Positive	Negative
Negative	Positive	Negative	Negative
Negative	Positive	Negative	Negative
Negative	Positive	Negative	Negative
Negative	Negative	Negative	Negative
Negative	Positive	Negative	Negative
Negative	Positive	Negative	Negative
Negative	Positive	Negative	Negative

Negative Negative Negative Negative

Negative Positive Negative Negative

Negative	Positive	Negative	Negative
Negative	Positive	Negative	Negative
Negative	Positive	Negative	Negative
Negative	Positive	Negative	Negative

Negative	Positive	Negative	Negative
Negative	Positive	Negative	Negative
Negative	Positive	Negative	Negative
Negative	Positive	Negative	Negative
Negative	Positive	Negative	Negative
Negative	Positive	Negative	Negative
Negative	Negative	Negative	Negative
Negative	Positive	Negative	Negative
Negative	Negative	Negative	Negative
Negative	Positive	Negative	Negative
Negative	Positive	Negative	Negative
Negative	Negative	Negative	Negative
Negative	Positive	Negative	Negative
Negative	Positive	Negative	Negative
Negative	Positive	Negative	Negative
Negative	Positive	Negative	Negative
Negative	Positive	Negative	Negative
Negative	Negative	Negative	Negative
Negative	Negative	Negative	Negative
Negative	Positive	Negative	Negative
Negative	Positive	Negative	Negative
Negative	Positive	Negative	Negative
Negative	Positive	Negative	Negative
Negative	Positive	Negative	Negative
Negative	Negative	Positive	Negative
Negative	Positive	Negative	Negative
Negative	Positive	Negative	Negative
Negative	Positive	Negative	Negative
Negative	Positive	Negative	Negative
Negative	Positive	Negative	Negative
Negative	Positive	Negative	Negative
Negative	Positive	Negative	Negative
Negative	Positive	Negative	Negative
Negative	Negative	Negative	Negative
Negative	Negative	Negative	Negative
Negative	Positive	Negative	Negative
Negative	Negative	Negative	Negative
Negative	Negative	Negative	Negative
Negative	Negative	Negative	Negative
Negative	Positive	Negative	Negative

Negative	Positive	Negative	Negative
Negative	Positive	Negative	Negative
Negative	Positive	Negative	Negative
Negative	Negative	Negative	Negative
Negative	Positive	Negative	Negative
Negative	Positive	Negative	Negative
Negative	Positive	Negative	Negative
Negative	Positive	Negative	Negative
Negative	Positive	Negative	Negative
Negative	Positive	Negative	Negative
Negative	Positive	Negative	Negative
Negative	Negative	Negative	Negative
Negative	Negative	Negative	Negative
Negative	Negative	Negative	Negative
Negative	Positive	Negative	Negative
Negative	Negative	Negative	Negative
Negative	Negative	Negative	Negative
Negative	Negative	Negative	Negative
Negative	Negative	Negative	Negative
Negative	Negative	Negative	Negative
Negative	Positive	Negative	Negative
Negative	Positive	Negative	Negative
Negative	Positive	Negative	Negative
Negative	Positive	Negative	Negative
Negative	Positive	Negative	Negative
Negative	Positive	Negative	Negative
Negative	Positive	Negative	Negative
Negative	Positive	Negative	Negative
Negative	Positive	Negative	Negative
Negative	Negative	Negative	Negative
Negative	Positive	Negative	Negative
Negative	Positive	Negative	Negative
Negative	Positive	Negative	Negative
Negative	Positive	Negative	Negative
Negative	Positive	Negative	Negative
Negative	Positive	Negative	Negative
Negative	Positive	Negative	Negative
Negative	Positive	Negative	Negative
Negative	Positive	Negative	Negative
Negative	Positive	Negative	Negative
Negative	Positive	Negative	Negative
Negative	Positive	Negative	Negative
Negative	Positive	Negative	Negative
Negative	Positive	Negative	Negative
Negative	Positive	Negative	Negative
Negative	Positive	Negative	Negative
Negative	Positive	Negative	Negative
Negative	Positive	Negative	Negative
Negative	Positive	Negative	Negative
Negative	Positive	Negative	Negative
Negative	Positive	Negative	Negative
Negative	Negative	Negative	Negative
Negative	Positive	Negative	Negative
Negative	Positive	Negative	Negative
Negative	Positive	Negative	Negative
Negative	Negative	Negative	Negative

Negative	Positive	Negative	Negative
Negative	Negative	Negative	Negative
Negative	Positive	Negative	Negative
Negative	Positive	Negative	Negative
Negative	Positive	Negative	Negative
Negative	Negative	Negative	Negative
Negative	Positive	Negative	Negative
Negative	Positive	Negative	Negative
Negative	Positive	Negative	Negative
Negative	Positive	Negative	Negative
Negative	Positive	Negative	Negative
Negative	Positive	Negative	Negative
Negative	Positive	Negative	Negative
Negative	Positive	Negative	Negative
Negative	Positive	Negative	Negative
Negative	Negative	Negative	Negative
Negative	Positive	Negative	Negative
Negative	Positive	Negative	Negative
Negative	Positive	Negative	Negative
Negative	Positive	Negative	Negative
Negative	Positive	Negative	Negative
Negative	Positive	Negative	Negative
Negative	Positive	Negative	Negative
Negative	Positive	Negative	Negative
Negative	Positive	Negative	Negative
Negative	Positive	Negative	Negative
Negative	Positive	Negative	Negative
Negative	Positive	Negative	Negative
Negative	Positive	Negative	Negative
Negative	Positive	Negative	Negative
Negative	Positive	Negative	Negative
Negative	Positive	Negative	Negative
Negative	Positive	Negative	Negative
Negative	Positive	Negative	Negative
Negative	Negative	Negative	Negative
Negative	Positive	Negative	Negative
Negative	Positive	Negative	Negative
Negative	Positive	Negative	Negative
Negative	Positive	Negative	Negative
Negative	Positive	Negative	Negative
Negative	Negative	Negative	Negative
Negative	Positive	Positive	Negative
Negative	Positive	Negative	Negative
Negative	Negative	Negative	Negative
Negative	Positive	Negative	Negative
Negative	Positive	Negative	Negative
Negative	Positive	Negative	Negative
Negative	Positive	Negative	Negative
Negative	Negative	Positive	Negative
Negative	Negative	Negative	Negative
Negative	Negative	Negative	Negative
Negative	Positive	Negative	Negative
Negative	Positive	Negative	Negative
Negative	Negative	Negative	Negative
Negative	Positive	Negative	Negative

Negative	Positive	Negative	Negative
Negative	Positive	Negative	Negative
Negative	Positive	Negative	Negative
Negative	Positive	Negative	Negative
Negative	Positive	Negative	Negative
Negative	Positive	Negative	Negative
Negative	Negative	Negative	Negative
Negative	Positive	Negative	Negative
Negative	Negative	Negative	Negative
Negative	Positive	Negative	Negative
Negative	Positive	Negative	Negative
Negative	Positive	Negative	Negative
Negative	Positive	Negative	Negative
Negative	Positive	Negative	Negative
Negative	Positive	Negative	Negative
Negative	Positive	Negative	Negative
Negative	Positive	Negative	Negative
Negative	Positive	Negative	Negative
Negative	Positive	Negative	Negative
Negative	Positive	Negative	Negative
Negative	Positive	Negative	Negative
Negative	Positive	Negative	Negative
Negative	Positive	Negative	Negative
Negative	Positive	Negative	Negative
Negative	Negative	Negative	Negative
Negative	Positive	Negative	Negative
Negative	Positive	Negative	Negative
Negative	Negative	Negative	Negative
Negative	Positive	Negative	Negative
Negative	Positive	Negative	Negative
Negative	Positive	Negative	Negative
Negative	Negative	Negative	Negative
Negative	Positive	Negative	Negative
Negative	Positive	Negative	Negative
Negative	Negative	Negative	Negative
Negative	Positive	Negative	Negative
Negative	Negative	Negative	Negative
Negative	Negative	Negative	Negative
Negative	Positive	Negative	Negative
Negative	Positive	Negative	Negative
Negative	Positive	Negative	Negative
Negative	Positive	Negative	Negative
Negative	Positive	Negative	Negative
Negative	Positive	Negative	Negative
Negative	Positive	Negative	Negative
Negative	Positive	Negative	Negative
Negative	Negative	Negative	Negative
Negative	Positive	Negative	Negative
Negative	Positive	Negative	Negative
Negative	Positive	Negative	Negative
Negative	Positive	Negative	Negative
Negative	Positive	Negative	Negative
Negative	Positive	Negative	Negative
Negative	Positive	Negative	Negative
Negative	Positive	Negative	Negative
Negative	Negative	Negative	Negative

Negative Positive	Negative Negative
Negative Negative	Negative Negative
Negative Positive	Negative Negative
Negative Positive	Negative Negative

Negative	Negative	Negative	Negative
Negative	Positive	Negative	Negative
Negative	Positive	Negative	Negative
Negative	Positive	Negative	Negative
Negative	Positive	Negative	Negative
Negative	Positive	Negative	Negative
Negative	Positive	Negative	Negative
Negative	Negative	Negative	Negative
Negative	Positive	Negative	Negative
Negative	Positive	Negative	Negative
Negative	Positive	Negative	Negative
Negative	Positive	Negative	Negative
Negative	Positive	Negative	Negative
Negative	Negative	Negative	Negative
Negative	Positive	Negative	Negative
Negative	Positive	Negative	Negative
Negative	Negative	Negative	Negative
Negative	Positive	Negative	Negative
Negative	Positive	Negative	Negative
Negative	Positive	Negative	Negative
Negative	Positive	Negative	Negative
Negative	Negative	Negative	Negative
Negative	Negative	Negative	Negative
Negative	Positive	Negative	Negative
Negative	Positive	Negative	Negative
Negative	Positive	Negative	Negative
Negative	Negative	Negative	Negative
Negative	Positive	Negative	Negative
Negative	Positive	Negative	Negative
Negative	Positive	Negative	Negative
Negative	Positive	Negative	Negative
Negative	Positive	Negative	Negative
Negative	Positive	Negative	Negative

Negative	Positive	Negative	Negative
Negative	Negative	Negative	Negative
Negative	Positive	Negative	Negative
Negative	Positive	Negative	Negative
Negative	Positive	Negative	Negative
Negative	Positive	Negative	Negative
Negative	Positive	Negative	Negative
Negative	Positive	Negative	Negative
Negative	Positive	Negative	Negative
Negative	Positive	Negative	Negative
Negative	Positive	Negative	Negative
Negative	Positive	Negative	Negative
Negative	Positive	Negative	Negative
Negative	Positive	Negative	Negative
Negative	Positive	Negative	Negative
Negative	Negative	Positive	Negative
Negative	Negative	Positive	Negative
Negative	Positive	Negative	Negative
Negative	Positive	Negative	Negative
Negative	Negative	Negative	Negative
Negative	Positive	Negative	Negative
Negative	Positive	Negative	Negative
Negative	Positive	Negative	Negative
Negative	Positive	Negative	Negative
Negative	Positive	Negative	Negative
Negative	Positive	Negative	Negative
Negative	Positive	Negative	Negative
Negative	Negative	Negative	Negative
Negative	Positive	Negative	Negative
Negative	Positive	Negative	Negative
Negative	Negative	Negative	Negative
Negative	Positive	Negative	Negative
Negative	Negative	Negative	Negative
Negative	Positive	Negative	Negative
Negative	Positive	Negative	Negative
Negative	Negative	Negative	Negative
Negative	Positive	Negative	Negative
Negative	Negative	Negative	Negative
Negative	Positive	Negative	Negative
Negative	Positive	Negative	Negative
Negative	Positive	Negative	Negative
Negative	Positive	Negative	Negative
Negative	Negative	Negative	Negative
Negative	Positive	Negative	Negative
Negative	Positive	Negative	Negative
Negative	Positive	Negative	Negative
Negative	Positive	Negative	Negative
Negative	Negative	Negative	Negative
Negative	Positive	Negative	Negative
Negative	Positive	Negative	Negative
Negative	Positive	Negative	Negative
Negative	Positive	Negative	Negative
Negative	Negative	Negative	Negative
Negative	Negative	Negative	Negative
Negative	Negative	Positive	Negative
Negative	Positive	Negative	Negative

Negative	Positive	Negative	Negative
Negative	Positive	Negative	Negative
Negative	Positive	Negative	Negative
Negative	Positive	Negative	Negative
Negative	Positive	Negative	Negative
Negative	Positive	Negative	Negative
Negative	Positive	Negative	Negative
Negative	Negative	Negative	Negative
Negative	Positive	Negative	Negative
Negative	Positive	Negative	Negative
Negative	Negative	Negative	Negative
Negative	Positive	Negative	Negative
Negative	Positive	Negative	Negative
Negative	Positive	Negative	Negative
Negative	Negative	Negative	Negative
Negative	Positive	Negative	Negative
Negative	Positive	Negative	Negative
Negative	Positive	Negative	Negative
Negative	Negative	Negative	Negative
Negative	Positive	Negative	Negative
Negative	Positive	Negative	Negative
Negative	Positive	Negative	Negative
Negative	Positive	Negative	Negative
Negative	Negative	Negative	Negative
Negative	Positive	Negative	Negative
Negative	Positive	Negative	Negative
Negative	Positive	Negative	Negative
Negative	Positive	Negative	Negative
Negative	Positive	Negative	Negative
Negative	Positive	Negative	Negative
Negative	Positive	Negative	Negative
Negative	Positive	Negative	Negative
Negative	Positive	Negative	Negative
Negative	Negative	Positive	Negative
Negative	Positive	Negative	Negative
Negative	Positive	Negative	Negative
Negative	Positive	Negative	Negative
Negative	Positive	Negative	Negative
Negative	Negative	Negative	Negative
Negative	Positive	Negative	Negative
Negative	Positive	Negative	Negative
Negative	Negative	Negative	Negative
Negative	Positive	Negative	Negative
Negative	Negative	Negative	Negative
Negative	Positive	Negative	Negative
Negative	Positive	Negative	Negative
Negative	Positive	Negative	Negative
Negative	Positive	Negative	Negative
Negative	Positive	Negative	Negative
Negative	Positive	Negative	Negative
Negative	Positive	Negative	Negative
Negative	Negative	Negative	Negative
Negative	Positive	Negative	Negative
Negative	Positive	Negative	Negative
Negative	Positive	Negative	Negative
Negative	Positive	Negative	Negative

Negative	Positive	Positive	Negative
Negative	Positive	Negative	Negative
Negative	Positive	Negative	Negative
Negative	Positive	Negative	Negative
Negative	Positive	Negative	Negative
Negative	Negative	Negative	Negative
Negative	Positive	Negative	Negative
Negative	Positive	Negative	Negative
Negative	Positive	Negative	Negative
Negative	Positive	Negative	Negative
Negative	Positive	Negative	Negative
Negative	Positive	Negative	Negative
Negative	Positive	Negative	Negative
Negative	Positive	Negative	Negative
Negative	Negative	Negative	Negative
Negative	Positive	Negative	Negative
Negative	Positive	Negative	Negative
Negative	Negative	Negative	Negative
Negative	Negative	Negative	Negative
Negative	Negative	Negative	Negative
Negative	Positive	Negative	Negative
Negative	Positive	Negative	Negative
Negative	Negative	Negative	Negative
Negative	Positive	Negative	Negative
Negative	Positive	Negative	Negative
Negative	Positive	Negative	Negative
Negative	Positive	Positive	Negative
Negative	Positive	Negative	Negative
Negative	Negative	Negative	Negative
Negative	Positive	Negative	Negative
Negative	Positive	Negative	Negative
Negative	Positive	Negative	Negative
Negative	Positive	Negative	Negative
Negative	Negative	Negative	Negative
Negative	Negative	Negative	Negative
Negative	Positive	Negative	Negative
Negative	Positive	Negative	Negative
Negative	Positive	Negative	Negative
Negative	Negative	Negative	Negative
Negative	Negative	Negative	Negative
Negative	Positive	Negative	Negative
Negative	Positive	Negative	Negative
Negative	Positive	Negative	Negative
Negative	Positive	Negative	Negative
Negative	Negative	Negative	Negative
Negative	Positive	Negative	Negative
Negative	Positive	Negative	Negative
Negative	Negative	Negative	Negative
Negative	Positive	Negative	Negative
Negative	Positive	Negative	Negative
Negative	Positive	Negative	Negative
Negative	Negative	Negative	Negative
Negative	Positive	Negative	Negative
Negative	Positive	Negative	Negative
Negative	Positive	Negative	Negative

Negative	Negative	Negative	Negative
Negative	Positive	Negative	Negative
Negative	Positive	Negative	Negative
Negative	Positive	Negative	Negative
Negative	Positive	Negative	Negative
Negative	Positive	Negative	Negative
Negative	Positive	Negative	Negative
Negative	Positive	Negative	Negative
Negative	Negative	Negative	Negative
Negative	Negative	Negative	Negative
Negative	Positive	Negative	Negative
Negative	Negative	Negative	Negative
Negative	Positive	Negative	Negative
Negative	Positive	Negative	Negative
Negative	Positive	Negative	Negative
Negative	Positive	Negative	Negative
Negative	Positive	Negative	Negative
Negative	Positive	Negative	Negative
Negative	Positive	Negative	Negative
Negative	Positive	Negative	Negative
Negative	Positive	Negative	Negative
Negative	Negative	Negative	Negative
Negative	Positive	Negative	Negative
Negative	Positive	Negative	Negative
Negative	Positive	Negative	Negative
Negative	Positive	Negative	Negative
Negative	Positive	Negative	Negative
Negative	Negative	Negative	Negative
Negative	Positive	Negative	Negative
Negative	Negative	Negative	Negative
Negative	Positive	Negative	Negative
Negative	Positive	Negative	Negative
Negative	Negative	Negative	Negative
Negative	Positive	Negative	Negative
Negative	Positive	Negative	Negative
Negative	Positive	Negative	Negative
Negative	Negative	Negative	Negative
Negative	Positive	Negative	Negative
Negative	Positive	Negative	Negative
Negative	Positive	Negative	Negative
Negative	Positive	Negative	Negative
Negative	Positive	Negative	Negative
Negative	Positive	Negative	Negative
Negative	Positive	Negative	Negative
Negative	Positive	Negative	Negative
Negative	Positive	Negative	Negative
Negative	Positive	Negative	Negative
Negative	Negative	Negative	Negative
Negative	Positive	Negative	Negative
Negative	Negative	Negative	Negative
Negative	Positive	Negative	Negative
Negative	Negative	Negative	Negative
Negative	Positive	Negative	Negative
Negative	Negative	Negative	Negative
Negative	Positive	Negative	Negative

Negative	Positive	Negative	Negative
Negative	Positive	Negative	Negative
Negative	Negative	Negative	Negative
Negative	Positive	Negative	Negative
Negative	Negative	Negative	Negative
Negative	Negative	Negative	Negative
Negative	Positive	Negative	Negative
Negative	Negative	Negative	Negative
Negative	Positive	Negative	Negative
Negative	Positive	Negative	Negative
Negative	Positive	Negative	Negative
Negative	Positive	Negative	Negative
Negative	Positive	Negative	Negative
Negative	Positive	Negative	Negative
Negative	Positive	Negative	Negative
Negative	Negative	Negative	Negative
Negative	Positive	Negative	Negative
Negative	Positive	Negative	Negative
Negative	Positive	Negative	Negative
Negative	Positive	Negative	Negative
Negative	Positive	Negative	Negative
Negative	Positive	Negative	Negative
Negative	Positive	Negative	Negative
Negative	Positive	Negative	Negative
Negative	Negative	Negative	Negative
Negative	Positive	Negative	Negative
Negative	Negative	Negative	Negative

Negative Negative Negative Negative

Positive Negative Negative

Negative Negative Negative Negative

Negative	Positive	Negative	Negative

Negative	Negative	Negative	Negative
Negative	Negative	Negative	Negative
Negative	Negative	Negative	Negative
Negative	Negative	Negative	Negative
Negative	Negative	Negative	Negative
Negative	Positive	Negative	Negative
Negative	Positive	Negative	Negative
Negative	Positive	Negative	Negative
Negative	Negative	Negative	Negative
Negative	Negative	Negative	Negative
Negative	Negative	Negative	Negative
Negative	Positive	Negative	Negative
Negative	Positive	Negative	Negative
Negative	Positive	Negative	Negative
Negative	Positive	Negative	Negative
Negative	Negative	Negative	Negative
Negative	Negative	Negative	Negative
Negative	Positive	Negative	Negative

Negative	Positive	Negative	Negative
Negative	Negative	Negative	Negative
Negative	Positive	Negative	Negative
Negative	Positive	Negative	Negative
Negative	Positive	Negative	Negative
Negative	Positive	Negative	Negative
Negative	Positive	Negative	Negative
Negative	Positive	Negative	Negative
Negative	Positive	Negative	Negative
Negative	Positive	Negative	Negative
Negative	Positive	Negative	Negative
Negative	Positive	Negative	Negative
Negative	Positive	Negative	Negative
Negative	Positive	Negative	Negative
Negative	Positive	Negative	Negative
Negative	Positive	Negative	Negative
Negative	Positive	Negative	Negative
Negative	Positive	Negative	Negative
Negative	Positive	Negative	Negative
Negative	Negative	Negative	Negative
Negative	Negative	Negative	Negative
Negative	Positive	Negative	Negative
Negative	Negative	Negative	Negative
Negative	Positive	Negative	Negative
Negative	Positive	Negative	Negative
Negative	Positive	Negative	Negative
Negative	Positive	Negative	Negative
Negative	Negative	Negative	Negative
Negative	Positive	Negative	Negative
Negative	Positive	Negative	Negative
Negative	Positive	Negative	Negative
Negative	Positive	Negative	Negative
Negative	Positive	Negative	Negative
Negative	Positive	Negative	Negative
Negative	Positive	Negative	Negative
Negative	Positive	Negative	Negative
Negative	Positive	Negative	Negative
Negative	Positive	Negative	Negative
Negative	Negative	Negative	Negative
Negative	Positive	Negative	Negative
Negative	Negative	Negative	Negative
Negative	Negative	Negative	Negative
Negative	Positive	Negative	Negative
Negative	Positive	Negative	Negative
Negative	Positive	Negative	Negative
Negative	Positive	Negative	Negative
Negative	Positive	Negative	Negative
Negative	Positive	Negative	Negative
Negative	Negative	Negative	Negative
Negative	Positive	Negative	Negative
Negative	Negative	Negative	Negative
Negative	Negative	Negative	Negative
Negative	Negative	Negative	Negative
Negative	Positive	Negative	Negative
Negative	Positive	Negative	Negative
Negative	Positive	Negative	Negative

Negative	Positive	Negative	Negative
Negative	Positive	Negative	Negative
Negative	Positive	Negative	Negative
Negative	Positive	Negative	Negative
Negative	Negative	Negative	Negative
Negative	Positive	Negative	Negative
Negative	Positive	Negative	Negative
Negative	Positive	Negative	Negative
Negative	Positive	Negative	Negative
Negative	Positive	Negative	Negative
Negative	Positive	Negative	Negative
Negative	Negative	Negative	Negative
Negative	Positive	Negative	Negative
Negative	Positive	Negative	Negative
Negative	Negative	Negative	Negative
	Negative	Negative	Negative
Negative	Negative	Negative	Negative
Negative	Positive	Negative	Negative
Negative	Positive	Negative	Negative
Negative	Positive	Negative	Negative
Negative	Positive	Negative	Negative
Negative	Positive	Negative	Negative
Negative	Positive	Negative	Negative
Negative	Positive	Negative	Negative
Negative	Negative	Negative	Negative
Negative	Positive	Negative	Negative
Negative	Positive	Negative	Negative
Negative	Negative	Negative	Negative
Negative	Positive	Negative	Negative
	Positive	Negative	Negative
Negative	Positive	Negative	Negative
Negative	Negative	Negative	Negative
Negative	Positive	Negative	Negative
Negative	Positive	Negative	Negative
Negative	Negative	Positive	Negative
Negative	Positive	Negative	Negative
Negative	Positive	Negative	Negative
Negative	Positive	Negative	Negative
Negative	Positive	Negative	Negative
Negative	Positive	Negative	Negative
Negative	Negative	Negative	Negative
Negative	Positive	Negative	Negative
Negative	Positive	Negative	Negative
Negative	Negative	Negative	Negative
Negative	Positive	Negative	Negative
Negative	Positive	Negative	Negative
Negative	Positive	Negative	Negative
Negative	Positive	Negative	Negative
Negative	Negative	Negative	Negative
Negative	Negative	Negative	Negative
Negative	Positive	Negative	Negative
Negative	Positive	Negative	Negative
Negative	Negative	Negative	Negative
Negative	Negative	Negative	Negative
Negative	Positive	Negative	Negative

Negative	Positive	Negative	Negative
Negative	Positive	Negative	Negative
Negative	Positive	Negative	Negative
Negative	Positive	Negative	Negative
Negative	Positive	Negative	Negative
Negative	Negative	Negative	Negative
Negative	Negative	Negative	Negative
Negative	Positive	Negative	Negative
Negative	Negative	Negative	Negative
Negative	Positive	Negative	Negative
Negative	Positive	Negative	Negative
Negative	Positive	Negative	Negative
Negative	Positive	Negative	Negative
Negative	Positive	Negative	Negative
Negative	Positive	Negative	Negative
Negative	Positive	Negative	Negative
Negative	Positive	Negative	Negative
Negative	Positive	Negative	Negative
Negative	Positive	Negative	Negative
Negative	Positive	Negative	Negative
Negative	Negative	Negative	Negative
Negative	Positive	Negative	Negative
Negative	Positive	Negative	Negative
Negative	Positive	Negative	Negative
Negative	Positive	Negative	Negative
Negative	Positive	Negative	Negative
Negative	Positive	Negative	Negative
Negative	Negative	Negative	Negative
Negative	Negative	Negative	Negative
Negative	Positive	Negative	Negative
Negative	Positive	Negative	Negative
Negative	Negative	Negative	Negative
Negative	Negative	Negative	Negative
Negative	Positive	Negative	Negative
Negative	Positive	Negative	Negative
Negative	Positive	Negative	Negative
Negative	Negative	Negative	Negative
Negative	Negative	Negative	Negative
Negative	Negative	Negative	Negative
Negative	Positive	Negative	Negative
Negative	Negative	Negative	Negative
Negative	Positive	Negative	Negative
Negative	Positive	Negative	Negative
Negative	Positive	Negative	Negative
Negative	Positive	Negative	Negative
Negative	Positive	Negative	Negative
Negative	Positive	Negative	Negative
Negative	Positive	Negative	Negative
Negative	Positive	Negative	Negative
Negative	Positive	Negative	Negative
Negative	Positive	Negative	Negative
Negative	Positive	Negative	Negative
Negative	Positive	Negative	Negative
Negative	Positive	Negative	Negative
Negative	Negative	Negative	Negative

Negative	Negative	Negative	Negative
Negative	Negative	Negative	Negative
Negative	Positive	Negative	Negative
Negative	Positive	Negative	Negative
Negative	Positive	Negative	Negative
Negative	Positive	Negative	Negative
Negative	Positive	Negative	Negative
Negative	Positive	Negative	Negative
Negative	Positive	Negative	Negative
Negative	Positive	Negative	Negative
Negative	Positive	Negative	Negative
Negative	Positive	Negative	Negative
Negative	Positive	Negative	Negative
Negative	Positive	Negative	Negative
Negative	Positive	Negative	Negative
Negative	Negative	Negative	Negative
Negative	Positive	Negative	Negative
Negative	Positive	Negative	Negative
Negative	Negative	Negative	Negative
Negative	Positive	Negative	Negative
Negative	Negative	Negative	Negative
Negative	Positive	Negative	Negative
Negative	Negative	Negative	Negative
Negative	Positive	Negative	Negative
Negative	Positive	Negative	Negative
Negative	Positive	Negative	Negative
Negative	Negative	Positive	Negative
Negative	Positive	Negative	Negative
Negative	Positive	Negative	Negative
Negative	Positive	Negative	Negative
Negative	Positive	Negative	Negative
Negative	Positive	Negative	Negative
Negative	Positive	Negative	Negative
Negative	Positive	Negative	Negative
Negative	Positive	Negative	Negative
Negative	Positive	Negative	Negative
Negative	Positive	Negative	Negative
Negative	Positive	Negative	Negative
Negative	Positive	Negative	Negative
Negative	Negative	Negative	Negative
Negative	Positive	Negative	Negative
Negative	Positive	Negative	Negative
Negative	Positive	Negative	Negative
Negative	Positive	Negative	Negative
Negative	Positive	Negative	Negative
Negative	Positive	Negative	Negative
Negative	Positive	Negative	Negative
Negative	Negative	Negative	Negative
Negative	Negative	Negative	Negative
Negative	Positive	Negative	Negative
Negative	Positive	Negative	Negative
Negative	Negative	Negative	Negative
Negative	Positive	Negative	Negative
Negative	Positive	Negative	Negative
Negative	Positive	Negative	Negative
Negative	Negative	Negative	Negative

Negative	Positive	Negative	Negative
Negative	Positive	Negative	Negative
Negative	Positive	Negative	Negative
Negative	Positive	Negative	Negative
Negative	Negative	Negative	Negative
Negative	Positive	Negative	Negative
Negative	Positive	Negative	Negative
Negative	Positive	Negative	Negative
Negative	Positive	Negative	Negative
Negative	Positive	Negative	Negative
Negative	Positive	Negative	Negative
Negative	Positive	Negative	Negative
Negative	Positive	Negative	Negative
Negative	Negative	Negative	Negative
Negative	Positive	Negative	Negative
Negative	Positive	Negative	Negative
Negative	Positive	Negative	Negative
Negative	Positive	Negative	Negative
Negative	Positive	Negative	Negative
Negative	Positive	Negative	Negative
Negative	Positive	Negative	Negative
Negative	Negative	Negative	Negative
Negative	Positive	Negative	Negative
Negative	Positive	Negative	Negative
Negative	Positive	Negative	Negative
Negative	Positive	Negative	Negative
Negative	Positive	Negative	Negative
Negative	Positive	Negative	Negative
Negative	Positive	Negative	Negative
Negative	Positive	Negative	Negative
Negative	Positive	Negative	Negative
Negative	Negative	Negative	Negative
Negative	Positive	Negative	Negative
Negative	Positive	Negative	Negative
Negative	Positive	Negative	Negative
Negative	Positive	Negative	Negative
Negative	Positive	Negative	Negative
Negative	Positive	Negative	Negative
Negative	Positive	Negative	Negative
Negative	Positive	Negative	Negative
Negative	Positive	Negative	Negative
Negative	Positive	Negative	Negative

Negative Positive Negative Negative

Negative	Negative	Negative	Negative
Negative	Negative	Positive	Negative
Negative	Positive	Negative	Negative
Negative	Positive	Negative	Negative
Negative	Positive	Negative	Negative
Negative	Positive	Negative	Negative
Negative	Positive	Negative	Negative
Negative	Positive	Negative	Negative
Negative	Negative	Negative	Negative
Negative	Positive	Negative	Negative
Negative	Positive	Negative	Negative
Negative	Positive	Negative	Negative
Negative	Positive	Negative	Negative
Negative	Positive	Negative	Negative
Negative	Positive	Negative	Negative
Negative	Positive	Negative	Negative
Negative	Positive	Negative	Negative
Negative	Positive	Negative	Negative
Negative	Positive	Negative	Negative
Negative	Positive	Negative	Negative
Negative	Positive	Negative	Negative
Negative	Positive	Negative	Negative
Negative	Positive	Negative	Negative

Negative	Positive	Negative	Negative
Negative	Negative	Negative	Negative
Negative	Positive	Negative	Negative
Negative	Negative	Positive	Negative
Negative	Positive	Negative	Negative
Negative	Positive	Negative	Negative
Negative	Positive	Negative	Negative
Negative	Positive	Negative	Negative
Negative	Positive	Negative	Negative
Negative	Positive	Negative	Negative
Negative	Positive	Negative	Negative
Negative	Positive	Negative	Negative
Negative	Positive	Positive	Negative
Negative	Negative	Positive	Negative
Negative	Positive	Negative	Negative
Negative	Positive	Negative	Negative
Negative	Negative	Negative	Negative
Negative	Positive	Negative	Negative
Negative	Positive	Negative	Negative
Negative	Positive	Negative	Negative
Negative	Positive	Negative	Negative
Negative	Positive	Negative	Negative
Negative	Positive	Negative	Negative
Negative	Positive	Negative	Negative
Negative	Positive	Negative	Negative
Negative	Positive	Negative	Negative
Negative	Positive	Negative	Negative
Negative	Positive	Negative	Negative
Negative	Positive	Negative	Negative
Negative	Positive	Negative	Negative
Negative	Positive	Negative	Negative
Negative	Positive	Negative	Negative
Negative	Positive	Negative	Negative
Negative	Positive	Negative	Negative
Negative	Negative	Positive	Negative
Negative	Positive	Negative	Negative
Negative	Positive	Negative	Negative
Negative	Negative	Negative	Negative
Negative	Positive	Negative	Negative
Negative	Positive	Negative	Negative
Negative	Positive	Negative	Negative
Negative	Positive	Negative	Negative
Negative	Positive	Negative	Negative
Negative	Positive	Negative	Negative
Negative	Positive	Negative	Negative
Negative	Positive	Negative	Negative
Negative	Positive	Negative	Negative
Negative	Positive	Negative	Negative
Negative	Negative	Negative	Negative
Negative	Positive	Negative	Negative
Negative	Positive	Negative	Negative
Negative	Positive	Negative	Negative
Negative	Positive	Negative	Negative
Negative	Negative	Negative	Negative
Negative	Positive	Negative	Negative
Negative	Positive	Negative	Negative

Negative	Positive	Negative	Negative
Negative	Positive	Negative	Negative
Negative	Negative	Negative	Negative
Negative	Positive	Negative	Negative
Negative	Positive	Negative	Negative
Negative	Positive	Negative	Negative
Negative	Positive	Negative	Negative
Negative	Positive	Negative	Negative
Negative	Positive	Negative	Negative
Negative	Positive	Negative	Negative
Negative	Positive	Negative	Negative
Negative	Positive	Negative	Negative
Negative	Positive	Negative	Negative
Negative	Positive	Negative	Negative
Negative	Positive	Negative	Negative
Negative	Positive	Negative	Negative
Negative	Positive	Negative	Negative
Negative	Positive	Negative	Negative
Negative	Negative	Negative	Negative
Negative	Positive	Negative	Negative
Negative	Negative	Negative	Negative
Negative	Negative	Negative	Negative
Negative	Negative	Negative	Negative
Negative	Positive	Negative	Negative
Negative	Positive	Negative	Negative
Negative	Positive	Negative	Negative
Negative	Negative	Negative	Negative
Negative	Positive	Negative	Negative
Negative	Positive	Negative	Negative
Negative	Positive	Negative	Negative
Negative	Positive	Negative	Negative
Negative	Positive	Negative	Negative
Negative	Positive	Negative	Negative
Negative	Positive	Negative	Negative
Negative	Positive	Negative	Negative
Negative	Positive	Negative	Negative
Negative	Positive	Negative	Negative
Negative	Positive	Negative	Negative
Negative	Positive	Negative	Negative
Negative	Positive	Negative	Negative
Negative	Positive	Negative	Negative
Negative	Positive	Negative	Negative
Negative	Positive	Negative	Negative
Negative	Positive	Negative	Negative
Negative	Positive	Negative	Negative
Negative	Positive	Negative	Negative
Negative	Positive	Negative	Negative
Negative	Positive	Negative	Negative
Negative	Positive	Negative	Negative
Negative	Negative	Negative	Negative
Negative	Positive	Negative	Negative
Negative	Negative	Negative	Negative
Negative	Positive	Negative	Negative
Negative	Positive	Negative	Negative
Negative	Positive	Negative	Negative
Negative	Positive	Negative	Negative

Negative	Positive	Negative	Negative
Negative	Positive	Negative	Negative
Negative	Positive	Negative	Negative
Negative	Positive	Negative	Negative
Negative	Negative	Negative	Negative
Negative	Positive	Negative	Negative
Negative	Positive	Negative	Negative
Negative	Positive	Negative	Negative
Negative	Positive	Negative	Negative
Negative	Positive	Negative	Negative
Negative	Positive	Negative	Negative
Negative	Positive	Negative	Negative
Negative	Positive	Negative	Negative
Negative	Positive	Negative	Negative
Negative	Positive	Negative	Negative
Negative	Positive	Negative	Negative
Negative	Positive	Negative	Negative
Negative	Positive	Negative	Negative
Negative	Positive	Negative	Negative
Negative	Negative	Negative	Negative
Negative	Positive	Negative	Negative
Negative	Positive	Negative	Negative
Negative	Positive	Negative	Negative
Negative	Positive	Negative	Negative
Negative	Positive	Negative	Negative
Negative	Positive	Negative	Negative
Negative	Positive	Negative	Negative
Negative	Positive	Negative	Negative

Variable
unique-id
year
province
month
ward_type
sex
age
ageunits
organism
specimen
viability
date_admission
antibiotic
medical_device
iv line
cvc
urinary catheter
drainage port
intraarterial line
risk
other risk
other_risk_spec
diabetes
respiratory diseases
renal
cvs
malignancy
other_risk
previous_hospitalization
antibiotics in the past 6 months
oxygen
mechanical
mental
grouped _outcome
outcome_date
amimic
ami
augmic
aug
aasmic
aas
ampemic
amp
aztmic
azt
cpemic
cpe
cftkmic
cft
crmmic

crm
cipmic
cip
colmic
col
dormic
dor
etpmic
etp
fosmic
fos
genmic
gen
impmic
imp
levmic
lev
mermic
mer
minmic
min
ptmic
pt
pipmic
pip
tetmic
tet
tgcmic
tgc
tsmic
ts
ndm
kpc
oxa48variants
vim
ges

description	response
Computer generated unique number	
year of specimen collection date	2015, 2016, 2017, 20
Provinces in South africa with surveillance sites	GA=GAUTENG, KZ=KV
Month specimen was collected	Jan, Feb, Mar, Apr, M
Whether patient’s ward was adult (age >15 years) or paediatric (<=15 years)	ADULT, PAED, UNKNO
Sex of the patient	FEMALE, MALE, UNKN
Age value (numeric)	
Age unit	Y=year, M=month, D=
Name of organism identified	Organism name
Type of specimen received	BLOOD CULTURE
Culture result of organism	A=audit, B/L=broken/
date the patient was admitted (current admission)	
Antibiotic prescrubed on current admission	Y=yes, N=no, U=unkn
Whether the patient had a medical device inserted during admission	Y=yes, N=no, U=unkn
List of medical devices: Intravenous line	Y=yes, N=no, U=unkn
List of medical devices: Central venous catheter	Y=yes, N=no, U=unkn
List of medical devices: Urinary catheter	Y=yes, N=no, U=unkn
List of medical devices: Drainage port	Y=yes, N=no, U=unkn
List of medical devices: Intra-arterial line	Y=yes, N=no, U=unkn
Whether the patient had an underlying illness	Y=yes, N=no, U=unkn
Underlying illness: Other illness	Y=yes, N=no, U=unkn
List of all other illness	
Underlying illness: Diabetes	Y=yes, N=no, U=unkn
Underlying illness: Respiratory	Y=yes, N=no, U=unkn
Underlying illness: Renal transplant/ dialysis	Y=yes, N=no, U=unkn
Underlying illness: CVS/ Stroke	Y=yes, N=no, U=unkn
Underlying illness: Malignancy	Y=yes, N=no, U=unkn
Underlying illness: Other illness	Y=yes, N=no, U=unkn
Whether patient was admitted prior to the current admission	Y=yes, N=no, U=unkn
Whether patient received antibiotics in the past 6 months	Y=yes, N=no, U=unkn
Whether petient was oxygenated in current admission	Y=yes, N=no, U=unkn
Whether patient was mechanically ventilated	Y=yes, N=no, U=unkn
Patient's mental status	Alert, sedated, disorie
Grouping of the patient outcome	A=alive, D=died
Date the outcome was recorderd	
MIC value for Amikacin	
MIC interpretation	S=susceptible, I=inter
MIC value for amoxicillin-clavulanate	
MiC interpretation	S=susceptible, I=inter
MIC value for Ampicillin/Sulbactam	
MIC interpretation	S=susceptible, I=inter
MIC value for Ampicillin	
MIC interpretation	S=susceptible, I=inter
MIC value for Azreonam	
MIC interpretation	S=susceptible, I=inter
MIC value for Cefepime	
MIC interpretation	S=susceptible, I=inter
MIC value for Cefotaxime	
MIC interpretation	S=susceptible, I=inter
MIC value for Cefuroxime	

MIC interpretation	S=susceptible, I=inter
MIC value for Ciprofloxacin	
MIC interpretation	S=susceptible, I=inter
MIC value for Colistin	
MIC interpretation	S=susceptible, I=inter
MIC value for Doripenem	
MIC interpretation	S=susceptible, I=inter
MIC value for Ertapenem	
MIC interpretation	S=susceptible, I=inter
MIC value for Fosfomycin	
MIC interpretation	S=susceptible, I=inter
MIC value for Gentamycin	
MIC interpretation	S=susceptible, I=inter
MIC value for Imipenem	
MIC interpretation	S=susceptible, I=inter
MIC value for Levofloxacin	
MIC interpretation	S=susceptible, I=inter
MIC value for Meropenem	
MIC interpretation	S=susceptible, I=inter
MIC value for Minocycline	
MIC interpretation	S=susceptible, I=inter
MIC value for Piperacillin/ Tazobactam	
MIC interpretation	S=susceptible, I=inter
MIC value for Piperacillin	
MIC interpretation	S=susceptible, I=inter
MIC value for Tetracycline	
MIC interpretation	S=susceptible, I=inter
MIC value for Tigecycline	
MIC interpretation	S=susceptible, I=inter
MIC value for Trimethoprim/Sulfa	
MIC interpretation	S=susceptible, I=inter
carbapenemase gene blaNDM	Negative, Positive
carbapenemase gene blaKPC	Negative, Positive
carbapenemse gene blaOXA48 & variants	Negative, Positive
carbapenemase gene blaVIM	Negative, Positive
carbapenemase gene blaGES	Negative, Positive
