# Supplementary material for: Carbapenem-resistant Enterobacterales among patients with bloodstream infections in South Africa: Consolidated surveillance data, 2015–2021
Source: PLoS One. 2025 Jul 2;20(7):e0324262. doi: 10.1371/journal.pone.0324262 (PMC12221022; doi:10.1371/journal.pone.0324262)
Supplement: Attachment — Submitted filename: Letter to address comments from editor.docx [file pone.0324262.s006.docx]

19 June 2024

Edwin Ularte

PLOS ONE

Dear Edwin Ularte,

**Re: PONE-D-24-22805-Carbapenem-resistant Enterobacterales among patients with bloodstream infections in South Africa: consolidated surveillance data, 2015-2021**

Please see responses to comments raised on 12 June 2024.

“(1) During our evaluation of the documents provided, we noted that your ethics approval letter did not cover the entire date when the data were accessed for research [i.e. 22 September 2022.] Before we can proceed further with the submission, please provide the original ethics document / ethics approval extension document(s) for the study. If the document is in another language, please also provide an English translation. Please note that if this document is not included when your manuscript is resubmitted, it may be rejected

**Authors ‘response:** Please find attached ethics approval documents (in English) that cover data collection during the surveillance period, when the data were accessed and time period that the data were analysed.

(2) As you are reporting a retrospective study of medical records or archived samples, please ensure that you have discussed whether all data were fully anonymized before you accessed them and/or whether the IRB or ethics committee waived the requirement for informed consent. If patients provided informed written consent to have data from their medical records used in research, please include this information.

**Authors ‘response:** Data anonymization is challenging in the South African setting. Firstly, we do not have a unique identifier for patients. Secondly, our laboratory information system is not linked to our hospital systems. Thirdly, private and public facilities utilize different systems. Informed consent is a requirement covered by ethics. We added in line 138: Data were accessed on 22 September 2022 and anonymised prior to analysis.

(3)We kindly ask that you address the discrepancy in the ethics documentation. The individual N Hoho, K Jensen, T Labaka, N Legare, M Manaka, S Meiring, C Miller, M Moremi, Ngubane, S Nkikho and V Quan named as investigator on the ethics documents is not an author on the present submission. As we would generally expect the investigator named on the ethical approval to be an author on the submission, can you please provide an explanation for this situation?"

**Authors ‘response:** The GERMS surveillance programme is centrally coordinated on behalf of a number of centres at the NICD. The NICD compromises of seven specialist centres, a division for public health surveillance and response, the national cancer registry and transversal functions. It is within these centres, such as the Centre for Healthcare-Associated Infections, Antimicrobial Resistance and Mycoses (CHARM) where the surveillance work actually occurs. The people named on the ethics document are from the coordination office of the GERMS surveillance programme and the heads of the respective centres and laboratories as principal investigators. Every individual involved in the surveillance activities could not be included in the ethics application. There have over the 21 year history of GERMS been numerous publications that reference GERMS as a co-author for example due to the input of the various unnamed individuals in the surveillance activities as “for the groups GERMS-South Africa”.

Sincerely,

Corresponding/ last author: Olga Perovic

Address: National Institute for Communicable Diseases, Private Bag X4, Sandringham,

2132, South Africa

Telephone: +27 11 386 6278

Email: [olgap@nicd.ac.za](mailto:olgap@nicd.ac.za)
