# Supplementary material for: Carbapenem-resistant Enterobacterales among patients with bloodstream infections in South Africa: Consolidated surveillance data, 2015–2021
Source: PLoS One. 2025 Jul 2;20(7):e0324262. doi: 10.1371/journal.pone.0324262 (PMC12221022; doi:10.1371/journal.pone.0324262)
Supplement: Attachment — Submitted filename: Response to reviewers.docx [file pone.0324262.s008.docx]

20 January 2025

The Editor-in-Chief

PLOS ONE

Dear Dr Emily Chenette,

**Re: Carbapenem-resistant Enterobacterales among patients with bloodstream infections in South Africa: consolidated surveillance data, 2015-2021 – response to reviewers’ comments**

**Reviewer number 3**

**Authors’ response:**

**Abstract**

Lines 35-38

Sentences modified as follows:

“We aimed to provide a holistic overview of the GERMS-SA CRE BSI surveillance data from 01 July 2015 to 31 December 2021.”

“We conducted a cross-sectional study.”

**Introduction**

Line 74:

Reference 8 on line 74 was updated to include the URL of the most recent WHO report on critical pathogens list

Line 83:

Reference 11 was kept for relevance and reference 12 changed to a study that demonstrated age as a risk factor

**Methods**

Lines 149-150

Sentence modified as follows:

“All CRE BSI isolates that met the case definition were transported to the NICD. Viable CRE BSI isolates were characterized at the Centre for Healthcare-Associated Infections, Antimicrobial Resistance and Mycoses (CHARM), NICD.”

**Results and figures**

Lines 199-200

Figure legend modified “Fig. 1: Captured specimens on the NHLS CDW from the participating hospitals and cases reported on the GERMS-SA surveillance with completed CRFs. NHLS- National Health Laboratory Service, CDW- corporate data warehouse, BC- blood culture, CRE- carbapenem-resistant Enterobacterales, CFR- case report forms. Other specimens included urine, respiratory, swabs and rectal specimens. *Case report forms with the clinical characteristics and outcome were collected during the enhanced surveillance, 01 July 2015 to 31 December 2021.”

Line 219

“*Known data shown only, % column percentage.”

Line 298

“*Known data shown only, % column percentage.”

Lines 244-245, 341

Uniformity of trimethoprim-sulfamethoxazole

**Discussion**

Lines 324-328

Sentence revised to “This analysis describes the CRE surveillance conducted in South Africa over the 7-year period and to augment previous reports with additional epidemiological data to investigate clinical correlations of disease severity and outcomes.”

Lines 334-336

Sentence deleted.

**Tables and supplementary materials**

Uniformity, spelling and figure alignment checked.
